# Anticancer Effects and Phytochemical Profile of *Lavandula stoechas*

**DOI:** 10.3390/ph18111706

**Published:** 2025-11-10

**Authors:** Hatice Sevim Nalkiran, Ihsan Nalkiran

**Affiliations:** Department of Medical Biology, Faculty of Medicine, Recep Tayyip Erdogan University, Rize 53020, Türkiye; hatice.sevim@erdogan.edu.tr

**Keywords:** *Lavandula stoechas*, cytotoxicity, anticancer effects, phytochemicals

## Abstract

**Background/Objectives:** *Lavandula stoechas* has reported bioactivities, but its selective anticancer potential in human models remains insufficiently defined. This study aimed to compare cytotoxicity and selectivity of ethanol and methanol extracts prepared from fresh and dried *L. stoechas* and to profile candidate bioactive metabolites. **Methods:** Aerial parts *Lavandula stoechas* L. subsp. *stoechas* (*L. stoechas* L.) were extracted with ethanol or methanol from fresh (LsFE, LsFM) and dried (LsDE, LsDM) material. Cytotoxicity was assessed in cancer (MDA-MB-231, T98G, RT4) and non-malignant (hGF, ARPE-19) cells using Hoechst 33342-stained nuclear counts and MTS viability at 24–48 h. Metabolite identification was performed using LC–QTOF–MS in both positive and negative ESI modes, supported by database search results. **Results:** All extracts reduced viability in a dose- and time-dependent manner. Among them, the ethanol extract from fresh material (LsFE) displayed the highest cytotoxic potency and the most favorable selectivity profile, markedly reducing viability in breast (MDA-MB-231) and glioblastoma (T98G) cells while exerting only mild effects on non-malignant fibroblast (hGF) and retinal epithelial (ARPE-19) cells. In contrast, extracts from dried material, particularly LsDE, showed broader cytotoxicity across both cancerous and non-cancerous lines. LC–MS highlighted sesquiterpenoids (Kikkanol A; 3(4→5)-Abeo-4,11:4,12-diepoxy-3-eudesmanol), phenolics (tyrosol; 3,4-dihydroxybenzoic acid), flavonoid/ionone derivatives (luteolin 5,3′-dimethyl ether; 3-hydroxy-β-ionone), oxidized fatty acids (9(S)-HpODE, α-EpODE, 5,12-dihydroxy-eicosatetraenoic acid), and jasmonates (12-hydroxyjasmonic acid; dihydrojasmonic acid methyl ester), especially enriched in LsFE. **Conclusions:** Ethanol extracts of *L. stoechas* L., especially LsFE, demonstrated selective cytotoxicity against cancer cells while exerting relatively mild effects on non-malignant cells. The metabolite profile of *L. stoechas* L. extracts revealed a diverse composition, including phenolics, terpenoids, flavonoids, and oxidized lipids, which are commonly associated with biological activity. These results suggest that LsFE is a promising candidate for further studies focusing on compound isolation and mechanistic analysis.

## 1. Introduction

Cancer continues to be a major contributor to global morbidity and mortality worldwide, with rising incidence driven by demographic changes and increasing life expectancy [1]. Despite advances in surgery, chemotherapy, and radiotherapy, the financial and health burden of cancer continues to escalate, underscoring the need for novel therapeutic agents that are both effective and affordable. Natural products have long served as a valuable source of anticancer compounds; for example, paclitaxel and vincristine are widely used plant-derived drugs [2]. To date, hundreds of plant metabolites are under investigation as potential anticancer candidates, with accumulating evidence highlighting their efficacy in preclinical models [3,4,5].

Lavandula species, part of the Lamiaceae family, are commonly found throughout Mediterranean regions, including Türkiye, France, Spain, and Italy [6]. Apart from their aromatic and ornamental attributes, these plants have exhibited a range of biological activities, including anticonvulsant, sedative, antispasmodic, nootropic, anti-inflammatory, and antifungal effects [7,8,9,10]. Among them, *Lavandula stoechas* (*L. stoechas*) has attracted attention due to its rich phytochemical composition. Previous studies have reported its antioxidant, antibacterial, and antiproliferative activities, as well as cytotoxic effects against fibroblast, hepatocellular carcinoma, colon, and murine leukemia cells [11,12].

Despite increasing interest in plant-derived compounds, systematic evaluations of Lavandula extracts against human cancer cell lines remain scarce, and comparative analyses of fresh versus dried material have not been extensively explored. Moreover, recent advances in phytochemical screening technologies underscore the importance of integrating metabolite profiling with variable assays to identify novel lead compounds [13,14,15,16]. Addressing these gaps, the present study investigates the cytotoxic potential and phytochemical profile of *Lavandula stoechas* L. subsp. *stoechas* (*L. stoechas* L.) extracts prepared from fresh and dried plant materials. This study investigates the cytotoxic properties and phytochemical composition of *L. stoechas* L. extracts, including dry ethanol (LsDE), dry methanol (LsDM), fresh ethanol (LsFE), and fresh methanol (LsFM). By comparing the responses of malignant and non-malignant cell lines, the study aims to determine extracts exhibiting anticancer activity and to identify potential bioactive constituents contributing to these effects.

## 2. Results

### 2.1. Cytotoxic Effects of L. stoechas L. Extracts on Non-Cancerous and Cancer Cell Lines: Nuclear Morphology and Cell Viability Analysis

**Figure 1:**
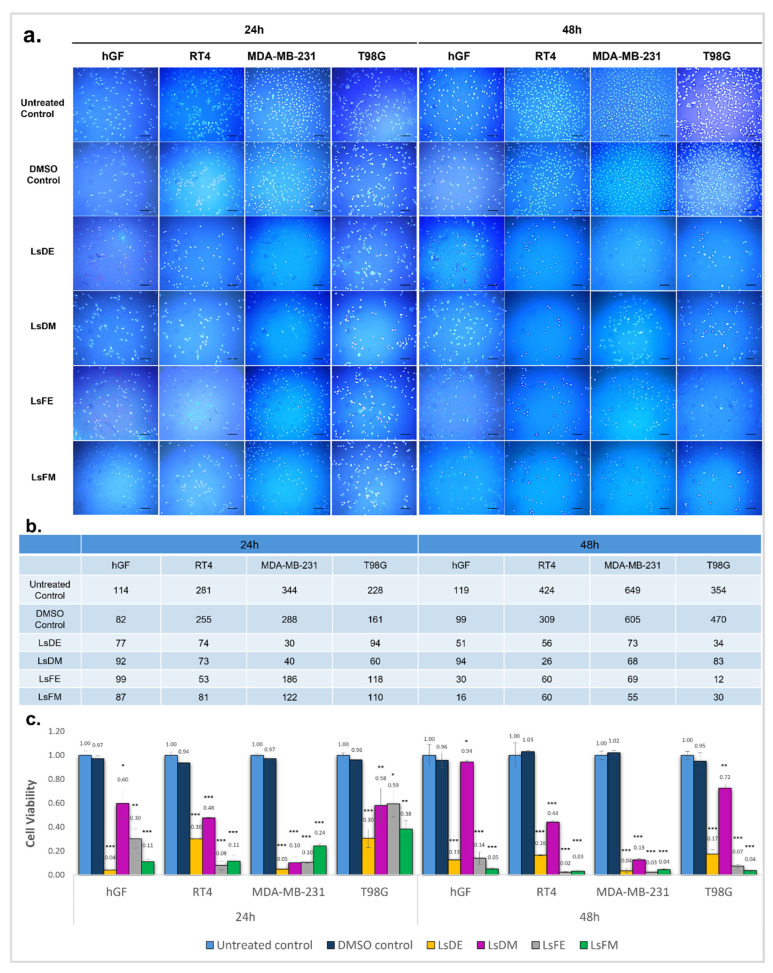
Hoechst staining and cell viability assessment following treatment with *L. stoechas* L. extracts at 2.5 µg/mL. (**a**) Representative fluorescence microscopy images of Hoechst 33342-stained nuclei in hGF, RT4, MDA-MB-231, and T98G cells after 24 h and 48 h of treatment with 2.5 µg/mL of *L. stoechas* L. extracts: LsDE, LsDM, LsFE and LsFM. Untreated control and DMSO control groups were included for comparison. Images were acquired at 10× magnification. Scale bar: 100 µm. (**b**) Quantification of nuclear counts from panel (**a**) using Hoechst staining, presented as the number of visible nuclei per image field. (**c**) Cell viability determined by MTS assay after 24 h and 48 h of treatment with the same extract groups. Viability is presented as relative absorbance values normalized to the untreated control. Data are presented as mean ± standard deviation (SD) from three independent experiments per-formed in triplicate. Statistical comparison was performed against the DMSO control using a two-tailed *t*-test. Significant differences are indicated as follows: *: *p* < 0.05, **: *p* < 0.01 and ***: *p* < 0.001.

The cytotoxic effects of *L. stoechas* L. extracts were assessed using fluorescence imaging and nuclear quantification after 24 and 48 h of treatment on hGF, RT4, MDA-MB-231, and T98G cells with 2.5 µg/mL of ethanol and methanol extracts from both dry and fresh plant sources. Hoechst 33342 staining showed a significant decrease in nuclear density in all extract-treated groups compared to untreated and DMSO controls, with more noticeable effects at 48 h (Figure 1a). Quantitative nuclear counts (Figure 1b) confirmed that LsDE and LsFE induced strong cytotoxic responses, especially in cancer cell lines. After 48 h, nuclear counts in MDA-MB-231 cells dropped from 649 (untreated control) to 73 with LsDE and 69 with LsFE. Similarly, T98G nuclear counts decreased from 354 to 34 (LsDE) and 12 (LsFE). In RT4 cells, which appeared more resistant, nuclear counts still decreased from 424 to 56 with LsDE and to 60 with LsFE. Conversely, hGF cells showed a less dramatic decline, from 119 to 51 (LsDE) and 94 (LsDM). Notably, while LsDM exhibited limited cytotoxicity in hGF cells at 48 h, its effect on cancer cells was also relatively moderate (e.g., 68 in MDA-MB-231 and 83 in T98G), indicating lower overall potency. These findings suggest that LsDE and LsFE extracts cause more significant nuclear loss in cancerous cells compared to non-cancerous fibroblasts, with effects becoming stronger over time and most apparent at 48 h post-treatment.

Cell viability was evaluated using an MTS assay to assess the cytotoxic effects *L. stoechas* L. extracts on four different cell lines, i.e., hGF, RT4, MDA-MB-231, and T98G, at a fixed concentration of 2.5 µg/mL for 24 and 48 h (Figure 1c). Statistical comparisons were performed using the DMSO-treated cells as the reference control. After 24 h, a significant reduction in cell viability was observed in all cancer cell lines treated with *L. stoechas* L. extracts. T98G and MDA-MB-231 cells showed pronounced reductions with LsDE (viability ratios: 0.30 and 0.05, respectively). RT4 cells exhibited significant sensitivity with a viability ratio of 0.08 (LsFE). In hGF cells, LsDE and LsFM also caused notable cytotoxicity (0.04 and 0.11, respectively), although the effects of LsDM (viability ratio, 0.60) and LsFE (viability ratios, 0.30) were less severe compared to LsDE and LsFM. At 48 h, cytotoxicity increased across all cell types and treatments. All cancer cells (RT4, MDA-MB-231, T98G) remained highly sensitive to LsDE, LsFE and LsFM (viability ratios: 0.16, 0.04 and 0.17 for LsDE; 0.02, 0.03 and 0.07 for LsFE; 0.03, 0.04 and 0.04 for LsFM, respectively). hGF viability remained relatively higher with LsDE and LsFE (viability ratios: 0.13 and 0.14, respectively), while LsFM led to more substantial inhibition (viability ratio: 0.05), indicating potential non-selective toxicity. Although LsDM showed relatively low cytotoxicity toward hGF cells, its ability to inhibit cancer cell lines was also limited. Throughout both time points, ethanol-based extracts (especially LsDE and LsFE) consistently exhibited strong anticancer effects, with somewhat lower but still significant impacts on the viability of normal fibroblasts. In contrast, methanol-based extracts, notably LsFM, displayed strong cytotoxicity across all cell types, including non-cancerous cells, indicating limited selectivity.

### 2.2. Dose-Dependent Cytotoxicity of L. stoechas L. Ethanol Extracts on Non-Cancerous and Cancer Cell Lines

**Figure 2:**
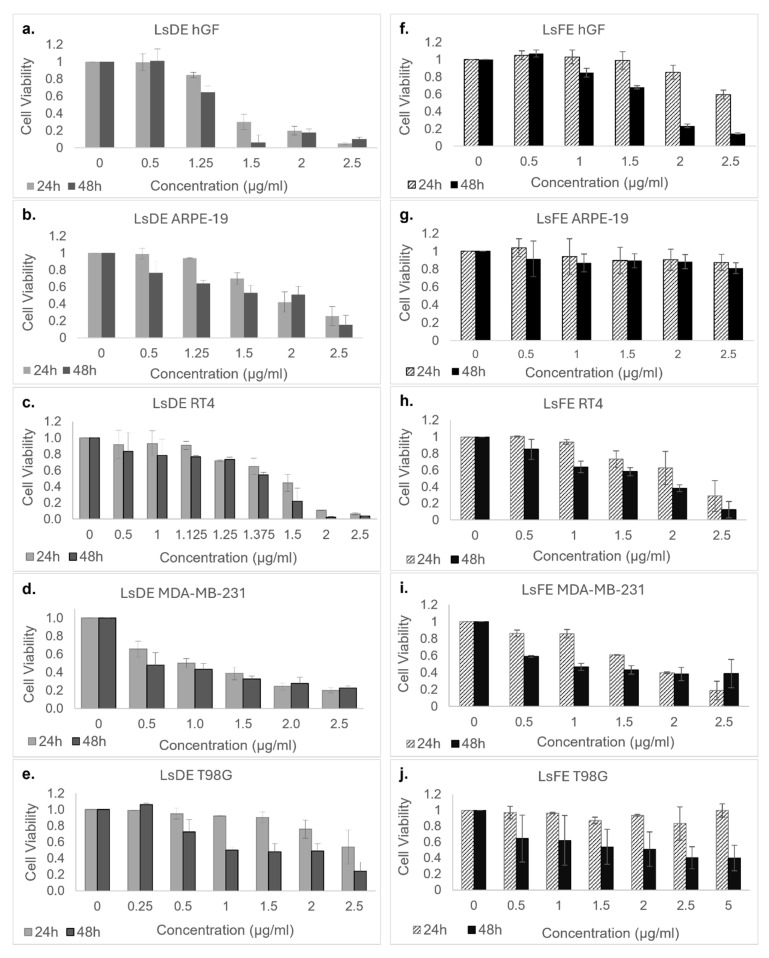
Dose- and Time-Dependent Cytotoxic Effects of LsDE and LsFE on Cancerous and Non-Cancerous Cell Lines. Cell viability was determined using the MTS assay following treatment with increasing concentrations of LsDE (**a**–**e**) and LsFE (**f**–**j**) ranging from 0 to 2.5 µg/mL (and up to 5 µg/mL in the T98G group) for 24 h and 48 h. The tested cell lines included non-cancerous hGF (**a**,**f**) and ARPE-19 (**b**,**g**), as well as cancerous RT4 (**c**,**h**), MDA-MB-231 (**d**,**i**), and T98G (**e**,**j**) cells. Cell viability was expressed relative to DMSO-treated control cells at each corresponding concentration. Concentration ‘0’ represents untreated control groups. Data are presented as mean ± standard deviation (SD) from three independent experiments performed in triplicate.

Based on the outcomes of the initial screening experiment, only the ethanol extracts (LsDE and LsFE) demonstrating both potent anticancer activity and improved selectivity were progressed to detailed dose–response analyses for IC_50_ determination. To further assess the cytotoxicity of *L. stoechas* L. LsDE and LsFE, dose–response analyses were performed on both non-cancerous cell lines (hGF and ARPE-19) and cancer cell lines (RT4, MDA-MB-231, and T98G) at increasing concentrations (0–2.5 µg/mL for LsDE; 0–5 µg/mL for LsFE) over 24 and 48 h (Figure 2).

LsDE treatment caused a dose- and time-dependent decrease in cell viability across all tested cell lines. In cancer cell lines, especially MDA-MB-231 and T98G, viability decreased significantly with increasing LsDE concentrations, dropping below 20% at 2.5 µg/mL after 48 h. RT4 cells also showed a gradual decline in viability, though to a lesser extent. Importantly, LsDE also induced a substantial cytotoxic response in both non-cancerous hGF and ARPE-19 cells, with viability falling below 40% at higher concentrations, particularly after 48 h, indicating limited selectivity.

In contrast, LsFE showed a more favorable cytotoxic profile. Notably, LsFE had stronger inhibitory effects on all three cancer cell lines in a dose- and time-dependent manner. At 2.5 µg/mL and 48 h, the viability of MDA-MB-231 and T98G cells dropped below 30%, while RT4 cells also consistently decreased. However, non-cancerous hGF and ARPE-19 cells maintained significantly higher viability across the same concentrations and durations—remaining above 60% at 48 h and even exceeding 80% viability at lower concentrations. This differential sensitivity indicates that LsFE may be more selective in targeting malignant cells while sparing non-cancerous cells. While LsDE showed broad efficacy, its toxicity affected non-cancerous cells, reducing its therapeutic selectivity. In contrast, LsFE proved to be a more selective inhibitor, showing strong anticancer effects in MDA-MB-231 and T98G cells, with a relatively lower impact on hGF and ARPE-19 cells, especially at 48 h. Collectively, the data support the potential of LsFE for subsequent mechanistic studies focused on selective anticancer activity.

To evaluate the dose-dependent cytotoxicity of LsDE and LsFE, IC_50_ values were determined for five different cell lines at 24 and 48 h after treatment (Table 1). The data show varying sensitivities between non-cancerous and cancerous cells, as well as between the two extracts tested. LsDE showed relatively strong cytotoxic effects across all tested cell lines. Notably, at 48 h, the IC_50_ values were significantly lower in cancer cells compared to non-cancerous cells, with the lowest IC_50_ observed in T98G (0.58 µg/mL) and MDA-MB-231 (0.63 µg/mL), indicating high sensitivity. In contrast, hGF and ARPE-19 cells exhibited higher IC_50_ values of 1.01 µg/mL and 1.90 µg/mL, respectively, suggesting a modest therapeutic window. The RT4 bladder cancer line also showed moderate sensitivity at 1.40 µg/mL. Compared to LsDE, LsFE was significantly less cytotoxic to non-cancerous cells. hGF cells exhibited IC_50_ values of 2.49 µg/mL at 24 h and 1.68 µg/mL at 48 h, whereas ARPE-19 cells did not reach the IC_50_ threshold even at the highest tested dose (>2.5 µg/mL). Notably, cancer cell lines were more sensitive, especially at 48 h. The IC_50_ decreased to 0.28 µg/mL in MDA-MB-231 and 0.43 µg/mL in T98G cells, demonstrating a more targeted cytotoxic effect. RT4 cells showed an intermediate IC_50_ of 1.72 µg/mL.

To further evaluate whether the extracts exerted preferential cytotoxicity toward cancer cells, Selectivity Index (SI) values were calculated using non-malignant hGF and ARPE-19 cells as reference models (Table 2). An SI value > 2 was considered to indicate selective anticancer activity.

LsDE exhibited limited selectivity at 24 h, with SI values generally below 2 across all cancer cell lines, suggesting a broad cytotoxic effect. At 48 h, moderate improvements were observed for LsDE, particularly against T98G cells, with SI values of 1.74 (hGF-based) and 3.28 (ARPE-19-based), although values remained < 2 against RT4.

In contrast, LsFE demonstrated enhanced selectivity, especially at 48 h. LsFE-48 h showed marked preference for malignant cells, with SI values of 6.00 (hGF) and 8.93 (ARPE-19) against MDA-MB-231, and 3.91 (hGF) and 5.81 (ARPE-19) against T98G cells. These results indicate substantial cancer-selective cytotoxicity. Selectivity toward RT4 cells remained lower (SI < 2), consistent with the IC_50_ data showing intermediate sensitivity of RT4 to LsFE.

Overall, selectivity analysis highlights LsFE at 48 h as the most promising formulation, demonstrating robust selectivity toward breast cancer and glioblastoma cells while exerting considerably milder effects on non-malignant cells.

The comparative evaluation of *L. stoechas* L. ethanol extracts showed that LsFE had a more favorable cytotoxicity profile LsDE. Although both extracts exhibited time- and dose-dependent effects, LsFE demonstrated greater selectivity by causing significant cytotoxicity in cancer cell lines, especially MDA-MB-231 and T98G, while having minimal effects on non-cancerous hGF and ARPE-19 cells. These findings emphasize LsFE as a promising candidate for further mechanistic studies, given its strong cytotoxic effects on malignant cells and relatively limited toxicity on non-cancerous cells.

### 2.3. Phytochemical Composition of LsDE and LsFE Extract Reveals Bioactive Compounds

LC–QTOF–MS profiling of the LSDE in negative ESI mode resulted in the annotation of six major metabolites belonging primarily to terpenoid and phenolic chemical classes (Table 3). Riesling acetal (*m/z* 225.149) was identified as the most abundant feature, while additional terpenoid-related compounds, including (5α,8β,9β)-5,9-epoxy-3,6-megastigmadien-8-ol (*m/z* 207.139), (S)-p-mentha-1,8-dien-10-yl acetate (*m/z* 239.129), and (S)-α-terpinyl glucoside (*m/z* 315.181), were detected with high database match scores, consistent with metabolic profiles characteristic of *Lavandula* species. The oxidized fatty acid derivative γ-9(10)-EpODE (*m/z* 293.212) was also identified, supporting the presence of lipid-derived bioactive metabolites within the extract. A phenolic compound with a coumarin-type structure (*m/z* 283.061; C_16_H_12_O_5_), annotated as 7,3′-dihydroxy-4′-methoxy-4-phenylcoumarin, was additionally detected in LSDE. This metabolite was also observed in the LsFE under positive ionization mode with matching retention behavior, indicating that it represents a shared phytochemical constituent across extraction conditions. The detection of both terpenoid and phenolic constituents in LSDE contributes to the interpretation of its observed cytotoxic properties.

The positive ion mode analysis of the LsDE identified four main compounds with distinct *m/z* values, abundances, and database matching scores (Table 4). The most abundant compound was 3-methyl-tetradecanedioic acid (*m/z* 295.1875), with an abundance of 158,475.45 and a high database score of 96.53, supported by four independent database hits. Another notable compound was 3(4→5)-Abeo-4,11:4,12-diepoxy-3-eudesmanol (*m/z* 275.1614), which showed a relatively high abundance (16,567.18) and the highest database hit count (10), with a strong confidence score of 94.1. Additionally, 3,4-dihydroxyphenyl ethanol (*m/z* 177.0522) was detected at an abundance of 12,840.17, with a strong score of 95.81 and eight supporting database hits, confirming its reliable identification. Finally, Kikkanol A (*m/z* 277.1770) was present at a similar abundance (12,793.35) but with a lower database score (77.72), though the identification was still supported by ten hits, indicating a possible presence of this compound in the extract. The results show that the LsDE extract contains a variety of compounds, including fatty acid derivatives, sesquiterpenoid epoxides, phenolic ethanol derivatives, and triterpenoid-related molecules. The high database scores (>90) for most detected compounds support their reliable identification, while Kikkanol A needs careful interpretation due to its lower confidence score despite multiple supporting hits.

The negative ionization mode LC–MS analysis of the LsFE revealed several compounds with high confidence scores (Table 5). The most prominent signal was 3-hydroxy-β-ionone (*m/z* 207.1387, abundance: 145,460.26), which showed the highest database match score (99.11, 10 hits). Similarly, riesling acetal (*m/z* 225.1492) and crispolide (*m/z* 279.124) were identified with high abundance values (141,806.06 and 79,414.87, respectively) and strong match confidence (scores 98.77 and 98.92, both with 10 hits). Other notable constituents included luteolin 5,3′-dimethyl ether (*m/z* 313.0713, score 98.61), 2,5-dimethoxycinnamic acid (*m/z* 207.0666, score 98.35), and vanilpyruvic acid (*m/z* 209.0448, score 95.24). Phenolic acids were also identified, such as 3,4-dihydroxybenzoic acid (*m/z* 153.0186, score 96.75, 7 hits). Flavonoid-related structures like 5,7-dihydroxychromone (*m/z* 177.0188) and 7,3′-dihydroxy-4′-methoxy-4-phenylcoumarin (*m/z* 283.0611) were present, the latter showing high confidence in identification (score 98.58, 10 hits). Finally, 12-hydroxyjasmonic acid (*m/z* 225.1125) was identified with a high confidence match (score 95.56, 10 hits), indicating the presence of jasmonate-derived compounds in the extract.

The LC-MS analysis of the LsFE in positive ionization mode (Table 6) identified several bioactive phytochemicals with high confidence based on accurate mass, database scores, and multiple database hits. The most abundant metabolite detected was 5,12-dihydroxy-6,8,10,14-eicosatetraenoic acid (*m/z* 359.2187, abundance 72,189, score 98.77, 10 hits), indicating a strong presence of hydroxylated fatty acid derivatives. Other major compounds included Kikkanol A (*m/z* 277.1774, abundance 33,388.4, score 86.44, 10 hits) and 3(4→5)-Abeo-4,11:4,12-diepoxy-3-eudesmanol (*m/z* 275.1614, abundance 31,302.5, score 85.37, 10 hits), both sesquiterpenoid-type metabolites, suggesting a diverse terpenoid profile in the extract. Notably, 9(S)-HpODE (*m/z* 335.2188, abundance 30,372.3, score 83.37, 10 hits) and α-9(10)-EpODE (*m/z* 317.2081, abundance 11,726, score 96.35, 10 hits) were identified, representing oxidized linoleic acid derivatives that are often associated with antioxidant and signaling functions. Dihydrojasmonic acid, methyl ester (*m/z* 249.1463, abundance 29,522.2, score 84.04, 3 hits) was also detected, emphasizing the jasmonate-related secondary metabolite profile of the extract. Among phenolic constituents, 3,4-dihydroxyphenyl ethanol (tyrosol, *m/z* 177.0522, abundance 16,426 score 99.35, 8 hits) was identified with the highest database score, highlighting the reliability of its identification. This metabolite is especially notable given its well-documented antioxidant and cytoprotective activities. In summary, the extract displayed a complex mixture of fatty acid derivatives, sesquiterpenoids, jasmonates, and phenolics, with several compounds (e.g., 5,12-dihydroxy-eicosatetraenoic acid, 9(S)-HpODE, tyrosol) standing out due to their high abundance or confidence scores, supporting the bioactive potential of *L. stoechas* L.

The comparative LC–MS analyses of *L. stoechas* L. extracts in positive and negative ionization modes revealed both common metabolites and group-specific signatures. Kikkanol A consistently appeared, being identified in both LsDE and LsFE positive ionization modes, highlighting it as a stable sesquiterpenoid marker of the species. Another shared sesquiterpenoid, 3(4→5)-Abeo-4,11:4,12-diepoxy-3-eudesmanol, was also found in multiple datasets, further confirming the role of eudesmane-type terpenoids. Besides these overlaps, different compounds were enriched in various extracts. Fatty acid derivatives were the most common in both modes, including 3-methyl-tetradecanedioic acid (LsDE positive) and hydroxylated or oxidized lipids such as 5,12-dihydroxy-6,8,10,14-eicosatetraenoic acid, 9(S)-HpODE, and α-9(10)-EpODE (LsFE positive), creating a diverse mix of lipid-derived metabolites. Jasmonate-related molecules appeared in both polarities, such as dihydrojasmonic acid methyl ester (positive mode) and 12-hydroxyjasmonic acid (negative mode), indicating a repeated jasmonate-like secondary metabolism. Phenolic compounds were also consistently detected: 3,4-dihydroxyphenyl ethanol (tyrosol) was confirmed in both positive-mode datasets with very high scores, while phenolic acids such as 3,4-dihydroxybenzoic acid and vanilpyruvic acid were unique to negative mode. Additionally, flavonoid- and ionone-derived structures including luteolin 5,3′-dimethyl ether, crispolide, and 3-hydroxy-β-ionone added further chemical diversity in the LsFE negative mode. Overall, the analyses show that *L. stoechas* L. extracts contain a mix of recurring markers including Kikkanol A and tyrosol, alongside group-specific compounds such as fatty acid derivatives, jasmonates, and flavonoid/ionone molecules. This balance of common and unique features indicates that the plant’s bioactive profile is influenced by both core metabolites found in all extracts and distinctive molecules identified under specific ionization conditions.

## 3. Discussion

The present study provides a comprehensive evaluation of the cytotoxic properties and phytochemical composition of *L. stoechas* L. ethanol and methanol extracts derived from fresh and dried plant material. By systematic comparison across malignant and non-malignant cell lines demonstrates that extract type and preparation strongly influence both cytotoxic potency and selectivity.

Ethanol-based extracts, particularly LsFE, emerged as the most promising candidates, showing strong dose- and time-dependent inhibitory effects on cancer cells while sparing non-cancerous fibroblasts and retinal pigment epithelial cells to a greater extent. The IC_50_ values highlight this selectivity: while LsFE reduced viability of MDA-MB-231 and T98G cells at sub-micromolar concentrations, non-cancerous hGF and ARPE-19 cells maintained comparatively high survival rates, even at the highest tested doses. In contrast, methanol-derived extracts exhibited broader cytotoxicity, affecting both Non-cancerous and malignant cells with limited discrimination. This difference shows the importance of extraction methods and solvent choice in shaping biological activity, possibly through differential enrichment of bioactive phytochemicals [17,18]. The LsDE extract demonstrated strong cytotoxic effects across all cell lines but lacked clear selectivity. While this broad activity may reflect a rich repertoire of secondary metabolites, its toxicity toward non-cancerous cells reduces translational potential. Conversely, LsFE demonstrated a more favorable therapeutic window. Its ability to induce potent cytotoxicity in cancer cell lines, particularly MDA-MB-231 and T98G, while minimizing damage to hGF and ARPE-19 cells, suggests the presence of compounds with tumor-preferential activity. Such selective effects are crucial for future drug development, as they reduce the possibility of off-target toxicity. In agreement with these findings, previous studies have also demonstrated cytotoxic properties of *L. stoechas* [19]. The ethanol extract was reported to exert significant antiproliferative effects on HepG2 hepatocellular carcinoma cells using the MTT assay [11]. A recent study demonstrated that the ethanol extract of *L. stoechas* exhibited selective cytotoxicity, being most effective against A549 lung cancer cells and least effective against MDA-MB-231 triple-negative breast cancer cells, while remaining non-toxic to normal fibroblasts, an effect attributed largely to its phenolic content [20]. Furthermore, the cytotoxicity of a chloroform extract derived from *L. stoechas* leaves was evaluated against several cancer cell lines, including human epidermoid carcinoma (KB), human breast cancer (BC1), human lung cancer (LU1), human colon cancer (COL-2), drug-resistant KB-V (+VLB), murine leukemia (P-388), hormone-dependent human prostate cancer (LNCaP), and rat glioma (ASK) cells. The extract exhibited strong activity against the P-388 cell line but showed no cytotoxic effect on ASK cells [12]. Siddiqui et al. evaluated fractions of *L. stoechas* aerial parts against HepG2 cells and found that the ethanolic fraction caused the strongest inhibition of cell survival in a dose-dependent manner using the MTT assay [21].

The LC–MS analyses provided valuable insights into the potential molecular basis of these differential effects (Table S2). Phytochemical profiling demonstrated that ethanol extracts, particularly those obtained from LsFE, contained a broader array of phenolics, coumarin derivatives, and oxidized fatty acids, whereas LsDE showed a comparatively terpenoid-enriched chemical profile. These metabolites are known for their antioxidant, pro-apoptotic, and signaling-modulatory properties, which may collectively contribute to the enhanced selectivity of LsFE [22,23]. Tyrosol, for example, is well-documented for its cytoprotective effects in normal cells and its ability to induce apoptosis in malignant contexts, potentially explaining the observed differential sensitivity [24]. A coumarin-type metabolite was also detected in both LSDE and LSFE at matched retention times, supporting its stability across processing methods. Coumarins are widely recognized for cytotoxic and chemosensitizing activities, suggesting a potential contribution to the anticancer actions of *L. stoechas* L. extracts. One report demonstrated that oleanolic acid, a triterpenoid obtained from the ethyl acetate fraction of *L. stoechas,* educed the viability of MCF-7 and MDA-MB-231 breast cancer cells (IC_50_: 13.09 µg/mL and 160.22 µg/mL, respectively) and triggered apoptosis in MCF-7 cells through Bcl-2 suppression [25]. In support of our findings, earlier phytochemical investigations of *L. stoechas* have linked its anticancer potential to specific metabolites. For example, GC–MS analysis of the active ethanolic fraction revealed lupeol, phytol, α-cadinol, lup-20(29)-en-3-one, and hydrocoumarin, indicating that triterpenoids and phytosterol-related compounds are likely contributors to the observed cytotoxic effects [21].

The detection of jasmonate-related molecules across both ionization modes is also noteworthy. Jasmonates are recognized for their role in stress signaling in plants and their pro-apoptotic effects in mammalian cancer models, suggesting a conserved mechanism that warrants further mechanistic studies [26,27].

Our findings align with earlier reports of *L. stoechas* L. extracts displaying antioxidant and cytotoxic activities, but they extend the evidence by highlighting selective anticancer potential in human-derived cancer cell models. The distinct advantage of LsFE over LsDE in sparing non-malignant cells suggests that fresh plant-derived ethanol extracts may harbor bioactive candidates with greater therapeutic promise. Importantly, these results underscore that plant processing (drying vs. fresh use) may alter the chemical profile and biological activity of extracts, an aspect often overlooked in phytomedicine research.

However, this study has limitations. The experiments were restricted to in vitro conditions, and the observed cytotoxicity does not directly translate to in vivo efficacy or safety. Although key metabolites were annotated in both ionization modes for LSFE and LSDE, the identification confidence for several features remains putative, as structural confirmation through reference standards or complementary techniques such as MS/MS fragmentation and NMR was not performed. Additionally, database-based annotation can be constrained by the availability of reference spectra, and some features may represent isomeric or previously unreported compounds. Further targeted phytochemical characterization and bioactivity-guided fractionation will be required to clarify the identity and mechanistic contribution of individual metabolites, particularly those associated with the selective cytotoxic profile of LsFE. Notably, several annotated metabolites detected in this study do not have well-established anticancer roles in the literature, indicating that they may represent previously unrecognized bioactive candidates within *L. stoechas* L. extracts and warrant further mechanistic exploration. In addition, given that the current phytochemical analyses are preliminary, subsequent studies may focus on isolating and characterizing representative bioactive compounds to clarify their contribution to the observed activity. Additionally, exploring apoptosis, oxidative stress modulation, and cell signaling pathways will provide mechanistic insights into the cytotoxic effects of LsFE on cancer cells.

## 4. Methods

### 4.1. Extraction of L. stoechas L. Plant Extract

The plant material used in this study was morphologically identified as *Lavandula stoechas* L. subsp. *stoechas* (Lamiaceae) by Dr. Hatice Sevim Nalkıran (Department of Medical Biology, RTEU) using diagnostic keys described in Flora of Turkey and the East Aegean Islands Davis, 1982 [28] and Kucuk, 2019 [29]. The specimen was collected in Ortaca, Mugla, Türkiye by an herbalist. Identification was cross-checked with records from the United Herbaria of the University and ETH Zurich (GBIF occurrence ID 3383283857; https://www.gbif.org/occurrence/3383283857) accessed on 12 November 2021 [30] and verified against published morphological descriptions. A digital voucher specimen containing images and complete metadata has been deposited in Zenodo under https://doi.org/10.5281/zenodo.17374963 for permanent public reference [31]. The dried specimen and its photographic documentation are also preserved in the authors’ laboratory for future verification. The extraction was performed separately for fresh and dried plant materials. Only the aerial flowering parts of *L. stoechas* L. (inflorescences with aromatic bracts) were used (see Figure S1). Fresh flowers were stored at 4 °C and processed within 24 h of collection to preserve volatile constituents. A separate portion of the same material was oven-dried at 35–40 °C until constant weight due to the high ambient humidity in the collection region. Both fresh and dried flowers were coarsely ground prior to extraction. The plant materials were extracted using ethanol (99.8%) and methanol (99.9%) at a solvent-to-material ratio of 1:20 (*w*/*v*) by agitation at 125 rpm for 48 h at ambient temperature. For dried samples (LsDE and LsDM), 1 g of plant powder was extracted with 20 mL of ethanol or methanol, respectively. For fresh samples (LsFE and LsFM), 5 g of plant powder was extracted with 50 mL of ethanol or methanol, respectively. After extraction, the mixtures were filtered through quantitative filter papers (110 mm, slow, Cat. No. S.105.01.110, Macherey–Nagel, Duren, Germany). The combined filtrates were diluted with distilled water (1:1, *v*/*v*), frozen at −80 °C, and lyophilized for 48 h to ensure complete solvent removal. The final lyophilized extract weights were approximately 50 mg (LsDE), 70 mg (LsDM), 130 mg (LsFE), and 160 mg (LsFM). The dried powders were stored in airtight tubes at −20 °C until use. The lyophilized extracts were subsequently weighed and dissolved in DMSO to obtain stock solutions at a concentration of 50 mg/mL. Prior to treatment, stock solutions were diluted into appropriate cell culture media to obtain a concentration range of 0.5–2.5 µg/mL for use in the cytotoxicity experiments. DMSO was used as the solvent, and its final concentration in all treated and control wells was maintained below 0.01% to avoid solvent-induced cytotoxicity.

### 4.2. Culturing of Cells

Human cell lines used in this study included hGF (human gingival fibroblasts, PCS-201-018) and T98G (glioblastoma), obtained from the American Type Culture Collection (ATCC). MDA-MB-231 breast cancer cells were kindly provided by Prof. Selcen Çelik Uzuner (Black Sea Technical University, Türkiye), and ARPE-19 retinal pigment epithelial cells were generously supplied by Prof. Saliha Ekşi (Recep Tayyip Erdoğan University, Rize, Türkiye). All cells were cultured under standard conditions at 37 °C in a humidified 5% CO_2_ atmosphere, using media supplemented with 10% fetal bovine serum (FBS; Sigma-Aldrich, St. Louis, MO, USA) and 1% penicillin–streptomycin. RT-4, MDA-MB-231, and ARPE-19 cells were maintained in RPMI-1640 medium, hGF cells in MEM, and T98G cells in high-glucose DMEM. Before use, all media and *L. stoechas* L. extract stock solutions were sterilized through 0.22 µm filters.

### 4.3. Treatment with L. stoechas L. Extracts, Hoechst Staining, and Cytotoxicity Assays

Cells were plated in 96-well flat-bottom plates at a density of 1 × 10^4^ cells per well and left overnight to ensure proper adherence before extract exposure. The cytotoxic effects of DMSO-dissolved forms of the LsDE, LsDM, LsFE, LsFM extracts were assessed. The samples were applied initially at 2.5 µg/mL dose and further in a series of serially diluted concentrations within a specified range for 24 h and 48 h. In this study, *L. stoechas* L.extracts were tested across a concentration range of 0.5 to 2.5 µg/mL in the ARPE-19, hGF, RT4, and MDA-MB-231 cell lines. For the T98G glioblastoma cell line, a slightly broader concentration range was used, reaching up to 5 µg/mL, to better assess dose-dependent effects in this cell type. Cytotoxicity evaluation was conducted in two stages: (i) an initial screening at a fixed concentration (2.5 µg/mL) using Hoechst nuclear staining and the MTS assay to identify extract- and time-dependent effects (Figure 1), followed by (ii) dose–response analyses across a concentration gradient for the calculation of IC_50_ values (Figure 2).

A standardized concentration of 2.5 µg/mL was applied across all experimental groups for the initial Hoechst and MTS screening assays. This concentration was selected in alignment with standard cytotoxicity screening protocols, which often utilize 2.5 µg/mL as an upper threshold to detect potential cytotoxic effects while minimizing non-specific cellular stress. To account for any solvent-related effects, DMSO-only control groups were included for each tested concentration, wherein cells received DMSO volumes equivalent to those present in the extract-treated samples. All cytotoxicity assays were conducted using triplicate wells for each condition and repeated in at least three independent experiments.

For each experimental condition, parallel cultures were treated identically to ensure data consistency across both assays. To prevent cross-interference between fluorescent nuclear labeling and metabolic measurements, Hoechst staining and MTS viability assessments were conducted on separate plates. After 24 or 48 h of treatment, viable cells were stained with Hoechst dye for nuclear visualization as previously described [32].

Cell proliferation was quantified using the MTS assay. A working MTS assay mixture was prepared as described in our previous study [33] and the cells were incubated with reagent mix at 37 °C. Absorbance values were recorded at 492 nm using a Multiskan GO microplate reader (Thermo Fisher Scientific, Waltham, MA, USA) following 24 and 48 h treatment periods with the extracts. Untreated controls and DMSO-treated controls were included to differentiate extract-specific effects from solvent-related influences. A blank control containing only MTS reagent without cells was used to correct for background absorbance. The final absorbance values for each well were obtained by subtracting the blank reading. Relative cell viability was calculated by normalizing the absorbance of treated cells to that of the DMSO control group. IC_50_ values were calculated using the IC50.org software platform. Selectivity Index (SI) values were calculated by dividing the IC_50_ obtained in non-malignant cell lines (hGF or ARPE-19) by the corresponding IC_50_ value in each cancer cell line (MDA-MB-231, T98G, RT4), where SI > 2 was considered indicative of preferential cytotoxicity toward cancer cells.

### 4.4. Q-TOF Analysis for Compounds

Plant material was extracted with ethanol or methanol, selected to maximize the solubility and recovery of phytochemicals. The crude extracts were concentrated under reduced pressure and passed through a filter to remove particulate matter, yielding a clear solution (prepared in methanol or acetonitrile containing 0.1% formic acid) suitable for LC–MS analysis. Detailed chromatographic and mass spectrometry parameters, including column type, gradient program, and run time, are provided in Table S1.

Chromatographic separation and mass analysis were carried out on an Agilent 6530B LC-QTOF mass spectrometer (Agilent Technologies, Santa Clara, CA, USA), part of the 6200/6500 series platform, equipped with an electrospray ionization (ESI) source. Data were acquired in positive ion mode as (M+H)+ and (M+Na)+, and negative ion mode as (M–H)−, (M+HCOO)- to ensure broad coverage of phenolic, terpenoid, and lipid constituents. Source conditions, including fragmentor voltage and collision energy, were tuned to promote efficient ionization. High-resolution mass detection enabled accurate *m/z* measurement, isotope pattern analysis, and database-assisted metabolite annotation.

Raw data were processed using Agilent MassHunter Qualitative Analysis software (v. B.08.00), which incorporates noise filtering, isotopic distribution checks, and database searches. Each detected compound was annotated with its accurate mass, molecular formula, ion type, abundance, database hit count, and identification confidence score (Score (DB)). Abundance values were used for relative comparisons across compounds, and internal reference mass (IRM) calibration ensured mass accuracy and reproducibility throughout acquisition. The resulting dataset includes retention times, chromatographic peak areas, and spectral features, which are summarized in tabular form.

To ensure reliable metabolite identification, strict exclusion criteria were followed. Signals present in blanks or with a sample-to-blank abundance ratio below three were removed. In positive mode, features observed only as (M+Na)+ for simple volatile hydrocarbons, aldehydes, or esters (e.g., decanal, hexenal) were excluded, as these likely represent ionization artifacts rather than true metabolites. In negative mode, ions appearing only as (M+HCOO)- without a corresponding (M–H)− were similarly discarded, since they typically originate from solvent-derived formate adducts. Additional exclusions included library matches with scores below 85, features lacking reproducibility within ±0.1–0.2 min across replicates, large lipid species (PI, PS, PE, PC), long-chain fatty acids, amides, sphingoid bases, and generic class-level annotations (e.g., triterpenoid) without specific structural evidence. Instrument- and solvent-derived ions (e.g., formate, acetate) were also removed. However, polar secondary metabolites such as phenolics and terpenoids that reproducibly appeared as (M+Na)+ adducts (e.g., Kikkanol A, 3,4-dihydroxyphenyl ethanol) were retained when consistent retention time, isotopic distribution, and biological plausibility supported their annotation.

### 4.5. Statistical Analysis

All experiments were performed in triplicate (n = 3), and the entire set of experiments was independently repeated at least three times. Results are expressed as mean ± standard deviation (SD). Comparisons between extract-treated groups and the DMSO control were conducted using a two-tailed unpaired Student’s *t*-test. Statistical significance was defined as *p* < 0.05, with significance levels indicated as follows: *p* < 0.05 (*), *p* < 0.01 (**), and *p* < 0.001 (***). Analyses were carried out using the GraphPad QuickCalcs *t*-test calculator, 2025 GraphPad Software, (https://www.graphpad.com/quickcalcs/ttest1, accessed on 26 August 2025).

## 5. Conclusions

In conclusion, our results demonstrate that ethanol extracts of *L. stoechas* L., particularly those derived from fresh plant material, exhibit selective cytotoxicity against cancer cells while exerting comparatively mild effects on non-cancerous cells. The phytochemical analyses revealed a wide variety of sesquiterpenoids, phenolics, and lipid derivatives that may contribute to this activity. Collectively, these findings support *L. stoechas* L. as a promising source of anticancer metabolites and justify further mechanistic and translational investigations aimed at harnessing its selective bioactivity.

## Figures and Tables

**Table 1:** IC_50_ Values (µg/mL) of *L. stoechas* L. Extracts (LsDE and LsFE) in Different Cell Lines at 24 h and 48 h Post-Treatment.

		hGF	ARPE-19	RT4	MDA-MB-231	T98G
LsDE	24 h	1.28	1.73	1.33	1.48	2.50
	48 h	1.01	1.90	1.40	0.63	0.58
LsFE	24 h	2.49	>2.50	2.05	1.75	>5.00
	48 h	1.68	>2.50	1.72	0.28	0.43

“>” indicates that the IC_50_ was not reached at the maximum tested dose.

**Table 2:** Selectivity Index (SI) values of *L. stoechas* L. extracts calculated using non-malignant hGF and ARPE-19 cells as reference models.

Extract	Time	hGF/RT4	hGF/MDA-MB-231	hGF/T98G	ARPE-19/RT4	ARPE-19/MDA-MB-231	ARPE-19/T98G
LsDE	24 h	0.96	0.86	0.51	1.30	1.17	0.69
	48 h	0.72	1.60	1.74	1.36	3.02	3.28
LsFE	24 h	1.21	1.42	0.50	1.22	1.43	0.50
	48 h	0.98	6.00	3.91	1.45	8.93	5.81

‘>’ indicates that 50% inhibition was not reached at the highest tested concentration in Table 1; therefore, the reported IC_50_ values represent minimum estimates and the corresponding SI values may be underestimated.

**Table 3:** Annotated metabolites detected in the ethanol extract of LsDE under negative ESI mode by LC-QTOF-MS.

No	*m/z*	z	Abundance	Name	Formula	Ion	Score (DB)	Hits (DB)
1	225.149	−1	128,791.64	Riesling acetal	C_13_ H_22_ O_3_	(M–H)−	95.83	10
2	207.13926	−1	79,761.27	(5alpha,8beta,9beta)-5,9-Epoxy-3,6-megastigmadien-8-ol	C_13_ H_20_ O_2_	(M–H)−	98.83	10
3	239.12915	−1	14,831.12	(S)-p-Mentha-1,8-dien-10-yl acetate	C_12_ H_18_ O_2_	(M+HCOO)-	93.91	10
4	315.18099	−1	10,437.16	(S)-alpha-Terpinyl glucoside	C_16_ H_28_ O_6_	(M–H)−	97.02	4
5	283.06114	−1	8447.77	7,3′-Dihydroxy-4′-methoxy-4-phenylcoumarin	C_16_ H_12_ O_5_	(M–H)−	85.35	10
6	293.21184	−1	7284.23	γ-9(10)-EpODE	C_18_ H_30_ O_3_	(M–H)−	95.03	10

**Table 4:** Annotated metabolites detected in the ethanol extract of LsDE under positive ESI mode by LC-QTOF-MS.

No	*m/z*	z	Abundance	Name	Formula	Ion	Score (DB)	Hits (DB)
1	295.18755	1	158,475.45	3-methyl-tetradecanedioic acid	C_15_ H_28_ O_4_	(M+Na)+	96.53	4
2	275.1614	1	16,567.18	3(4→5)-Abeo-4,11:4,12-diepoxy-3-eudesmanol	C_15_ H_24_ O_3_	(M+Na)+	94.1	10
3	177.0522	1	12,840.17	3,4-Dihydroxyphenyl ethanol	C_8_ H_10_ O_3_	(M+Na)+	95.81	8
4	277.17698	1	12,793.35	Kikkanol A	C_15_ H_26_ O_3_	(M+Na)+	77.72	10

**Table 5:** Annotated metabolites detected in the ethanol extract of LsFE under positive ESI mode by LC-QTOF-MS.

No	*m/z*	z	Abundance	Name	Formula	Ion	Score (DB)	Hits (DB)
1	207.1387	−1	145,460.26	3-Hydroxy-beta-ionone	C_13_ H_20_ O_2_	(M–H)−	99.11	10
2	225.1492	−1	141,806.06	Riesling acetal	C_13_ H_22_ O_3_	(M–H)−	98.77	10
3	279.124	−1	79,414.87	Crispolide	C_15_ H_20_ O_5_	(M–H)−	98.92	10
4	313.0713	−1	72,412.8	Luteolin 5,3′-dimethyl ether	C_17_ H_14_ O_6_	(M–H)−	98.61	10
5	207.0666	−1	71,270.55	2,5-Dimethoxycinnamic acid	C_11_ H_12_ O_4_	(M–H)−	98.35	10
6	209.0448	−1	28,402.86	Vanilpyruvic acid	C_10_ H_10_ O_5_	(M–H)−	95.24	10
7	153.0186	−1	26,201.32	3,4-Dihydroxybenzoic acid	C_7_ H_6_ O_4_	(M–H)−	96.75	7
8	177.0188	−1	15,438.27	5,7-Dihydroxychromone	C_9_ H_6_ O_4_	(M–H)−	85.62	6
9	283.0611	−1	11,233.25	7,3′-Dihydroxy-4′-methoxy-4-phenylcoumarin	C_16_ H_12_ O_5_	(M–H)−	98.58	10
10	225.1125	−1	10,204.81	12-hydroxyjasmonic acid	C_12_ H_18_ O_4_	(M–H)−	95.56	10

**Table 6:** Annotated metabolites detected in the ethanol extract of LsFE under negative ESI mode by LC-QTOF-MS.

No	*m/z*	z	Abundance	Name	Formula	Ion	Score (DB)	Hits (DB)
1	359.2187	1	72,189.0	5,12-dihydroxy-6,8,10,14-eicosatetraenoic acid	C_20_ H_32_ O_4_	(M+Na)+	98.77	10
2	277.1774	1	33,388.4	Kikkanol A	C_15_ H_26_ O_3_	(M+Na)+	86.44	10
3	275.1614	1	31,302.5	3(4->5)-Abeo-4,11:4,12-diepoxy-3-eudesmanol	C_15_ H_24_ O_3_	(M+Na)+	85.37	10
4	335.2188	1	30,372.3	9(S)-HpODE	C_18_ H_32_ O_4_	(M+Na)+	83.37	10
5	249.1463	1	29,522.2	Dihydrojasmonic Acid, Methyl Ester	C_13_ H_22_ O_3_	(M+Na)+	84.04	3
6	177.0522	1	16,426.0	3,4-Dihydroxyphenyl ethanol	C_8_ H_10_ O_3_	(M+Na)+	99.35	8
7	317.2081	1	11,726.0	α-9(10)-EpODE	C_18_ H_30_ O_3_	(M+Na)+	96.35	10

## Data Availability

The data presented in this study are available on request from the corresponding author.

## Supplementary Materials

The following supporting information can be downloaded at: https://www.mdpi.com/article/10.3390/ph18111706/s1, Table S1: Instrument and analytical parameters.; Table S2: LC–MS annotated metabolite classes in LsFE and LsDE extracts.; Figure S1: The parts of *L. stoechas* L. used for extraction. Qualitative analysis report. The data file named HSN_1.d corresponds to the LsDE sample, while HSN_2.d corresponds to the LsFE sample.
