# Supplementary material for: Anticancer Effects and Phytochemical Profile of Lavandula stoechas
Source: Pharmaceuticals (Basel). 2025 Nov 10;18(11):1706. doi: 10.3390/ph18111706 (PMC12655630; doi:10.3390/ph18111706)
Supplement: Supplementary file 1 [file pharmaceuticals-3934473-supplementary.pdf › pharmaceuticals-3934473-supplementary.pdf]

Table S1. Instrument and Analytical Parameters.

**Parameter**	**Details**
Instrument	Agilent 6530B LC-QTOF Mass Spectrometer (Agilent Technologies, Santa Clara, CA, USA)
Ionization Mode	Positive (M+H^+, M+Na^+) and Negative (M–H^−, M+HCOO^−) Electrospray Ionization (ESI)
Acquisition Mode	AutoMS^2
Column	Poroshell 120 EC-C18 (3 × 50 mm, 2.7 µm)
Column Temperature	30°C
Mobile Phase (Solvent A)	90% H_2O with 0.1% Formic Acid
Mobile Phase (Solvent B)	10% Acetonitrile/Methanol
Flow Rate	0.4 mL/min
Injection Volume	1.0 µL
Gradient Program	0.5 min: A 90% / B 10% → 4 min: A 50% / B 50% → 9 min: A 5% / B 95%
Nebulizer Pressure	35 psi
Gas Flow Rate	10 L/min
Gas Temperature	325°C
VCap Voltage	3000 V
Fragmentor Voltage	200 V
Skimmer Voltage	65 V
Octopole RF	750 V
MS Scan Range	m/z 50–1200
MS/MS Scan Rate	2 spectra/sec
Collision Energy (CE)	Stepped 10, 20, 40 eV
Isolation Width	Medium (~4 amu)
Retention Time (RT)	Variable, depending on compound elution
Calibration	Internal reference mass (IRM) calibration applied throughout acquisition

Table S2. LC–MS annotated metabolite classes in LsFE and LsDE extracts.

Chemical Class	Representative Compound(s) Mentioned	Ion Mode(s)	Detected in
**Sesquiterpenoids**	Kikkanol A<br>3(4→5)-Abeo-4,11:4,12-diepoxy-3-eudesmanol	±	LsFE, LsDE
**Sesquiterpenoids / Monoterpenoids**	(5α,8β,9β)-5,9-Epoxy-3,6-megastigmadien-8-ol<br>(S)-p-mentha-1,8-dien-10-yl acetate<br>(S)-α-terpinyl glucoside	–	LsDE
**Phenolics**	Tyrosol<br>Vanilpyruvic acid<br>3,4-Dihydroxybenzoic acid	±	LsFE, LsDE
**Oxidized fatty acids (Oxylipins)**	9(S)-HpODE<br>α-9(10)-EpODE<br>5,12-DiHETE	±	LsFE, LsDE
**Coumarins**	7,3'-Dihydroxy-4'-methoxy-4-phenylcoumarin	±	LsFE, LsDE
**Phytohormones (Jasmonates)**	12-Hydroxyjasmonic acid<br>Dihydrojasmonic acid methyl ester	±	LsFE

Figure S1. The parts of *L. stoechas* L. used for extraction.

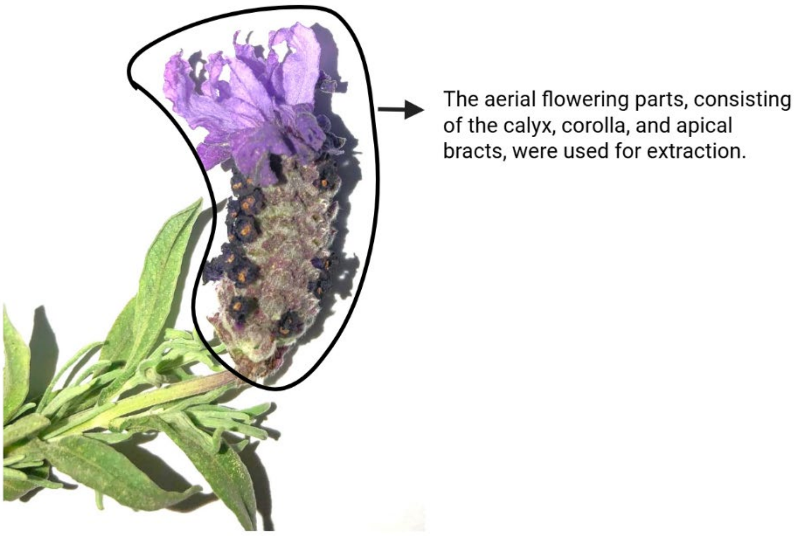

Qualitative Analysis Report

**Data Filename**	HSN_1.d	**Sample Name**	HSN_1
**Sample Type**	Sample	**Position**	P1-A4
**Instrument Name**	6530B LC Q-TOF	**User Name**	Hasan CAN (hcan)
**Acq Method**	Unknown_3-Expt_(neg).m	**Acquired Time**	3/24/2025 4:56:05 PM
**IRM Calibration Status**	Success	**DA Method**	Default.m
**Comment**			

**Sample Group**		**Info.**	
**Stream Name**	LC 1	**Method Version**	2025-0324-1207-54604
**Override DA Method Version**		**Data File Version**	2025-0324-1356-02117
**Acquisition Workstation**	DESKTOP-L73MD3C	**DA Workstation**	DESKTOP-L73MD3C
**Acquisition SW Version**	6200 series TOF/6500 series Q-TOF (11.0.203.0)		

User Chromatograms

Fragmentor Voltage 120 **Collision Energy** 0 **Ionization Mode** ESI

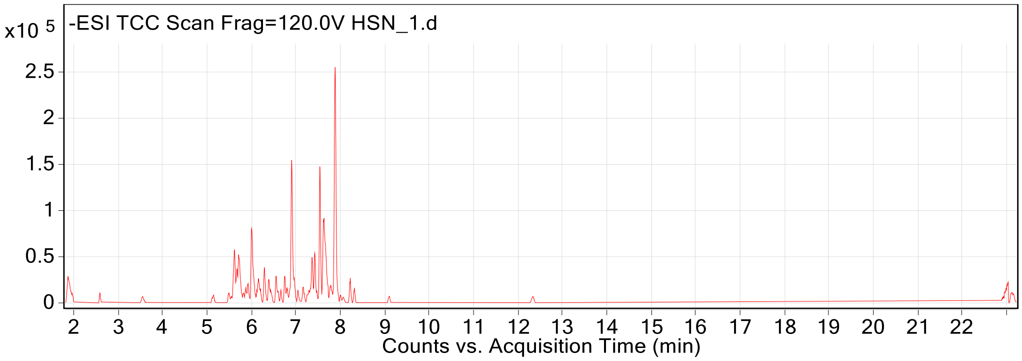

Compounds

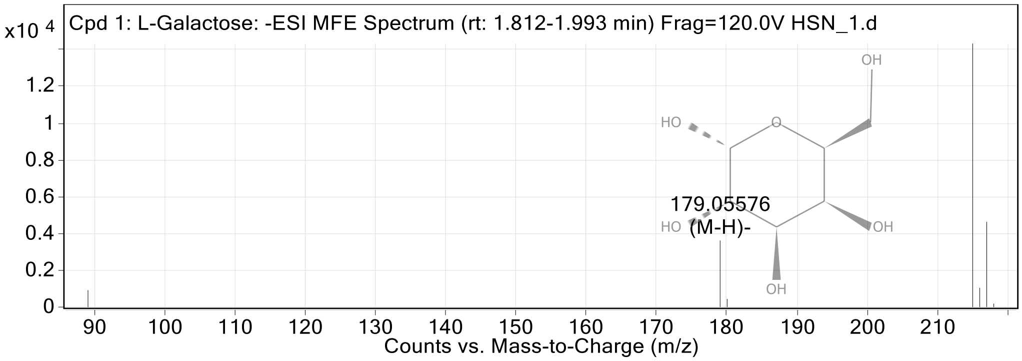

Peak List

m/z	z	Abund	Name	Formula	Ion	Score (DB)	Hits (DB)
89.02478	-2	919.65			(M-2H)-2		
179.05576	-1	3615.81			(M-H)-		
180.06092	-1	441.1			(M-H)-		
215.03298	-1	14317.53	L-Galactose	C6 H12 O6	(M+Cl)-	98.81	10
216.03616	-1	1045.82		C6 H12 O6	(M+Cl)-		
217.0309	-1	4631.3		C6 H12 O6	(M+Cl)-		
218.03202	-1	188.59		C6 H12 O6	(M+Cl)-		

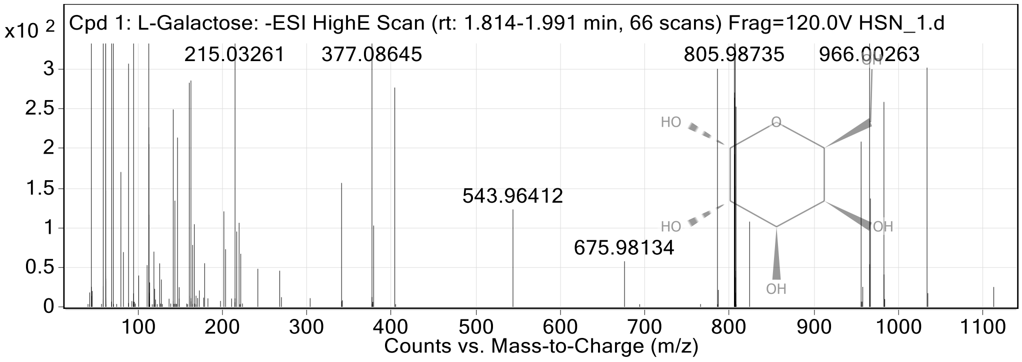

Peak List

m/z	z	Abund
44.99871	1	1038.09
59.01448		961.08
61.98895		7842.72
62.00496		727.12
68.99635		491.19
94.98117		642.26
112.98578	1	4649.87
805.98735	1	8326.31
806.99097	1	1491.4
966.00263	1	569.06

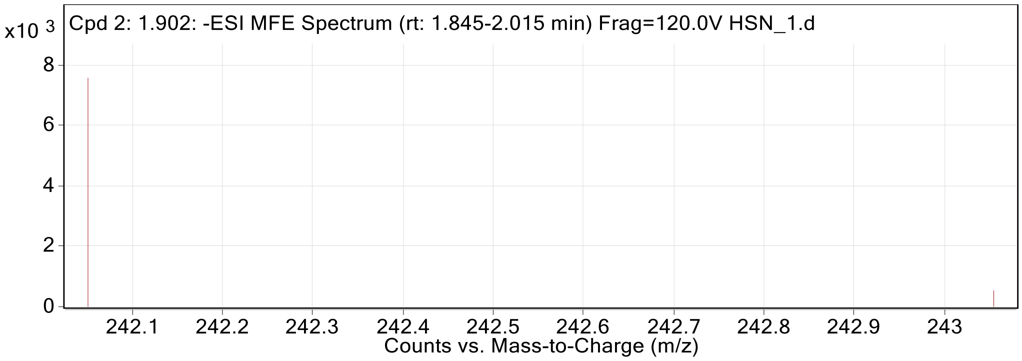

Peak List

m/z	z	Abund	Ion
242.05114	-1	7585.71	(M-H)-
243.05483	-1	538.57	(M-H)-

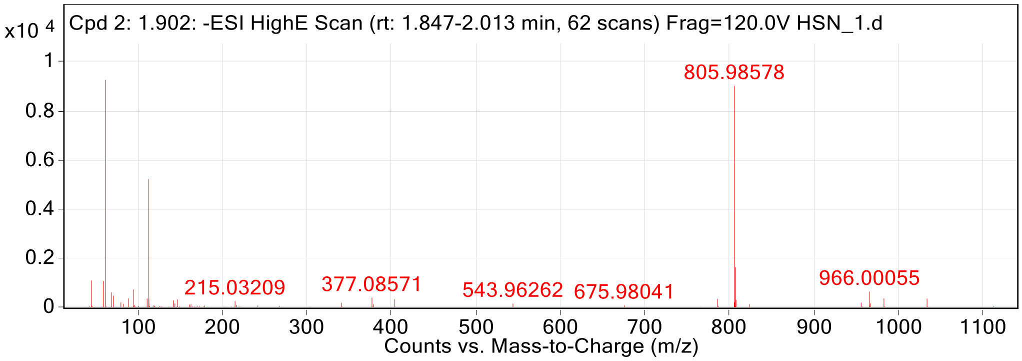

Peak List

m/z	z	Abund
44.99865	1	1075.9
59.01434		1060.72
61.98883		9260.71

62.0042		599.13
68.99619		589.45
94.98098		713.01
112.98554	1	5216.07
805.98578	1	9020.25
806.98932	1	1625.91
966.00055	1	626.61

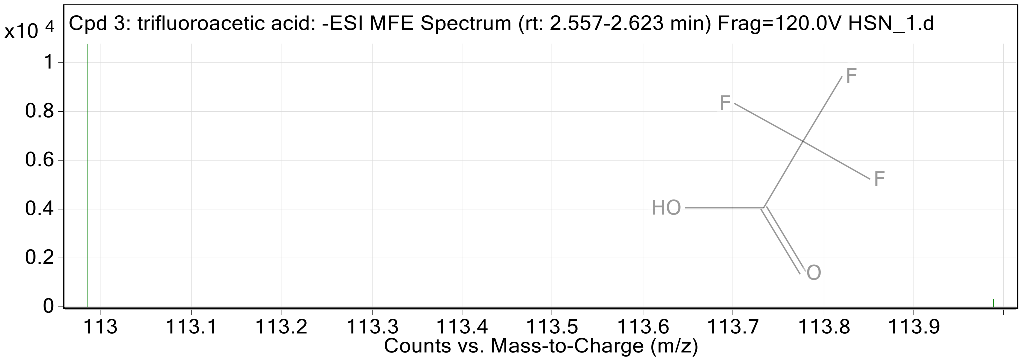

Peak List

m/z	z	Abund	Name	Formula	Ion	Score (DB)	Hits (DB)
112.98559	-1	10786.98	trifluoroacetic acid	C2 H F3 O2	(M-H)-	87.65	1
113.98827	-1	318.84		C2 H F3 O2	(M-H)-		

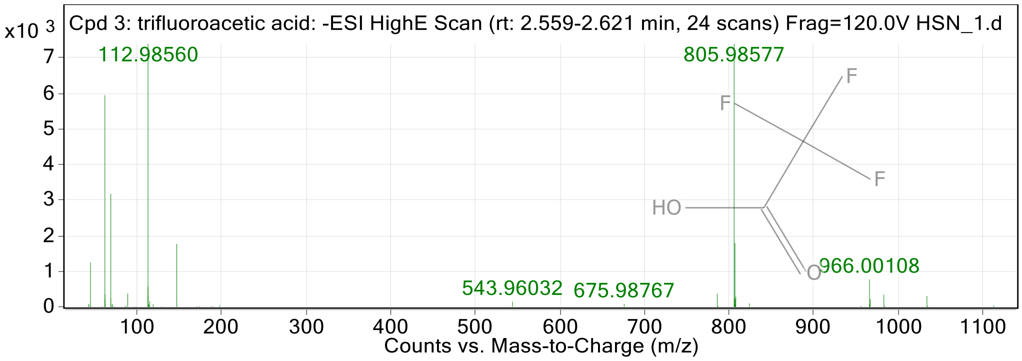

Peak List

m/z	z	Abund
44.99857		1244.13
61.98883		5946.33
68.99598		3176.85
89.02508		371.38
112.9856	1	7395.02
113.00626		575.8
146.96583		1766.38
805.98577	1	9879.12
806.98839	1	1794.2
966.00108	1	769.06

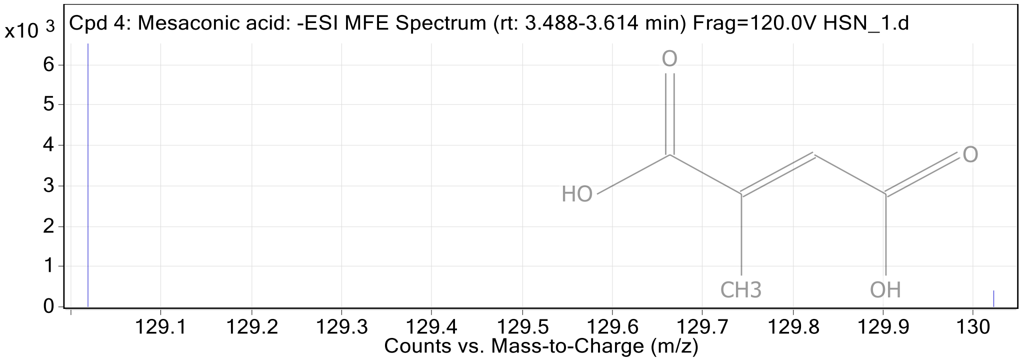

Peak List

m/z	z	Abund	Name	Formula	Ion	Score (DB)	Hits (DB)
129.019	-1	6523.15	Mesaconic acid	C5 H6 O4	(M-H)-	86.74	10
130.02285	-1	406.23		C5 H6 O4	(M-H)-		

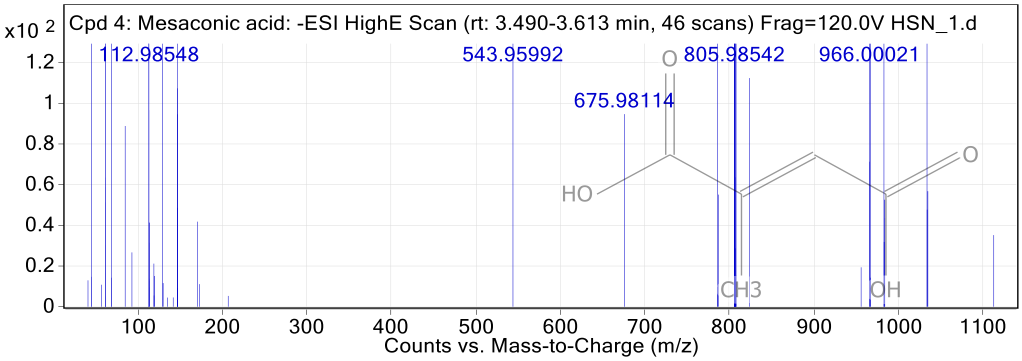

Peak List

m/z	z	Abund
44.99856		1018.6
61.98873		4861.11
68.99603		482.11
112.98548	1	5711.28
146.96587		2638.22
805.98542	1	9779.32
806.1376		463.36
806.98765	1	1738.96
966.00021	1	756.58
982.98996	1	424.14

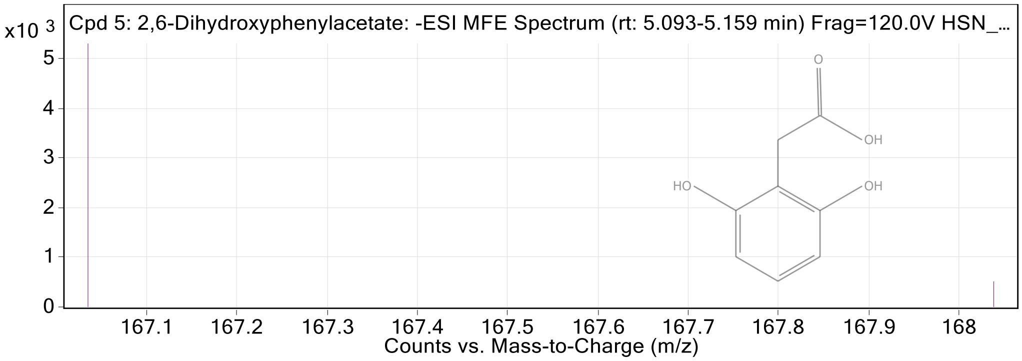

Peak List

m/z	z	Abund	Name	Formula	Ion	Score (DB)	Hits (DB)
167.03528	-1	5309.79	2,6-Dihydroxyphenylacetate	C8 H8 O4	(M-H)-	86.77	10
168.03861	-1	514.19		C8 H8 O4	(M-H)-		

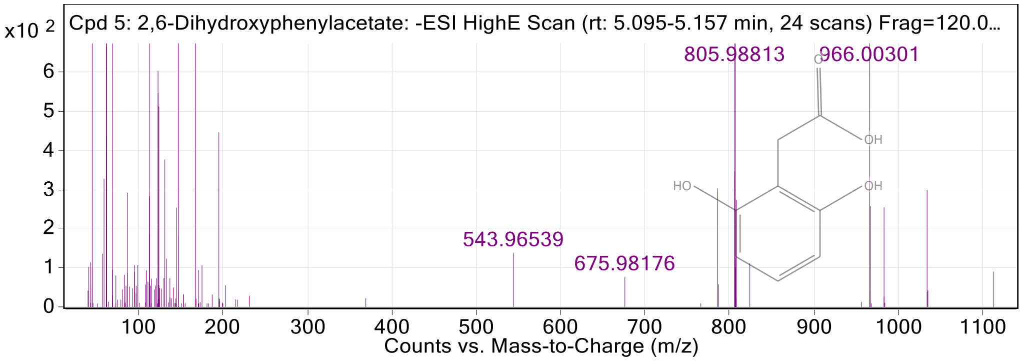

Peak List

m/z	z	Abund
44.99875		2416.35
61.98892		15880.49
62.0037		949.07
68.99629		1070.62
112.98578	1	5300.02
146.96619		2649.41
167.03542		673.12
805.98813	1	9911.31
806.99021	1	1741.59
966.00301	1	942.87

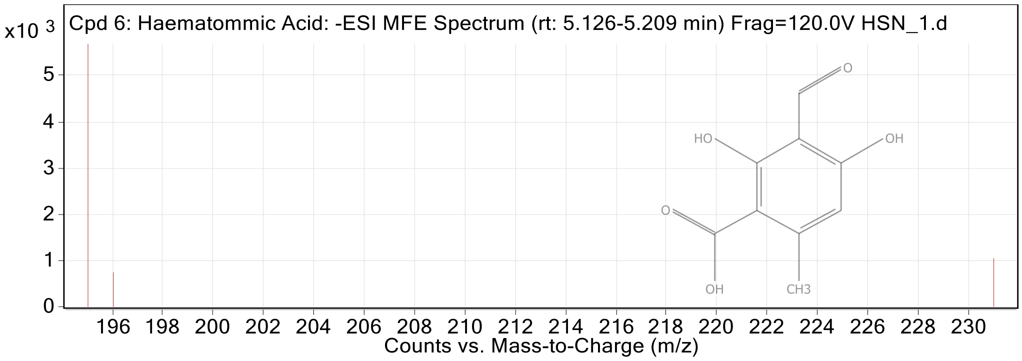

Peak List

m/z	z	Abund	Name	Formula	Ion	Score (DB)	Hits (DB)
195.0309	-1	5688.45	Haematommic Acid	C9 H8 O5	(M-H)-	73.45	3
196.0359	-1	746.18		C9 H8 O5	(M-H)-		
231.00703	-1	1051.23			(M+Cl)-		

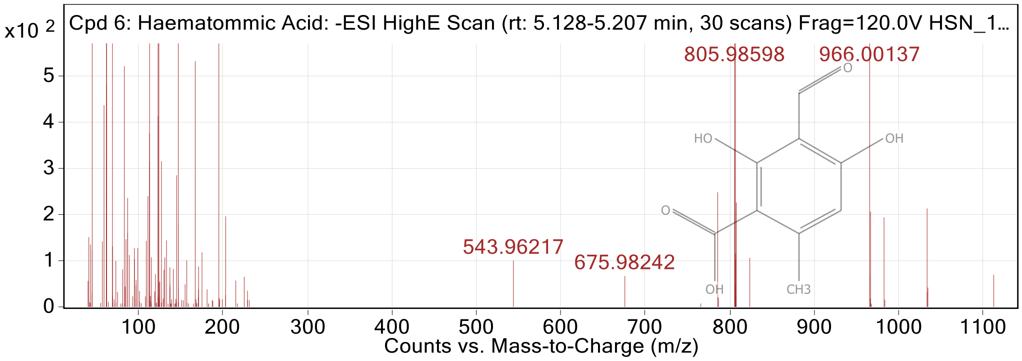

Peak List

m/z	z	Abund
44.99879		2616.18
61.98893		17235.12

62.00504		1739.84
68.99647		1050.2
112.98568		4779.42
123.00881		809.79
146.96598		2571.21
805.98598	1	8397.57
806.98746	1	1454.49
966.00137	1	824.74

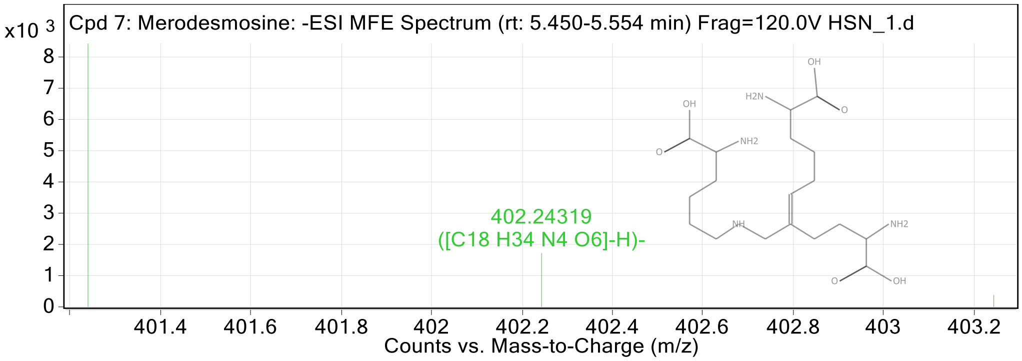

Peak List

m/z	z	Abund	Name	Formula	Ion	Score (DB)	Hits (DB)
401.23951	-1	8453.66	Merodesmosine	C18 H34 N4 O6	(M-H)-	96.17	10
402.24319	-1	1725.44		C18 H34 N4 O6	(M-H)-		
403.24377	-1	380.08		C18 H34 N4 O6	(M-H)-		

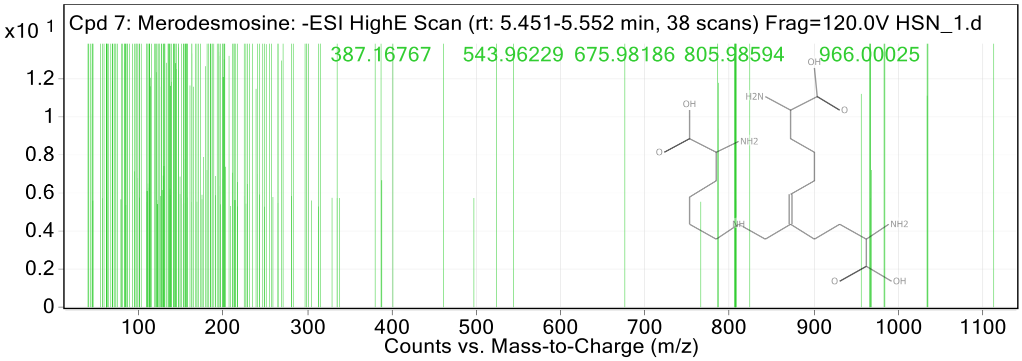

Peak List

m/z	z	Abund
44.99856		2711.19
61.98863		27790.4
62.00584		2207.15
68.99595		1039.88
112.98536		4827.63
130.99214		1062.53
146.96572		1402.85
805.98594	1	7989.61
806.98841	1	1474.33
966.00025	1	864.15

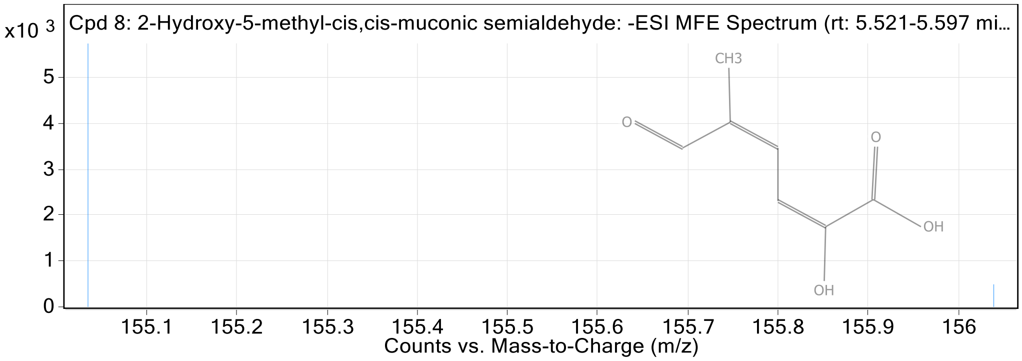

Peak List

m/z	z	Abund	Name	Formula	Ion	Score (DB)	Hits (DB)
155.03479	-1	5736.37	2-Hydroxy-5-methyl-cis,cis-muconic semialdehyde	C7 H8 O4	(M-H)-	85.51	10
156.03925	-1	490.62		C7 H8 O4	(M-H)-		

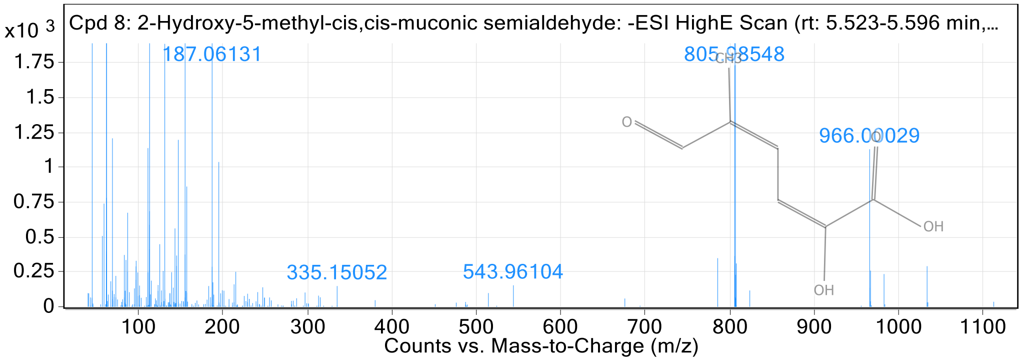

Peak List

m/z	z	Abund
44.99877		2975.69
61.98893		27585.86
62.00519		2873.62
68.99623		1206.51
112.98567	1	6311.6
130.99248		1883.12
155.03517	1	1887.24
187.06131	1	1942.98
805.98548	1	10642.04
806.98697	1	1887.36

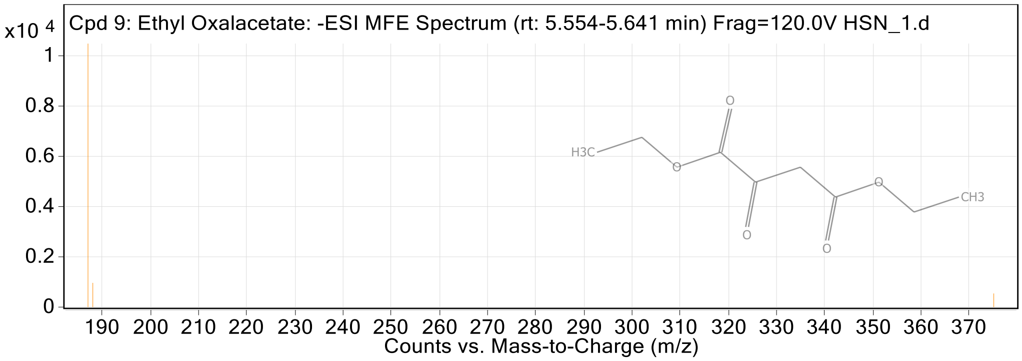

Peak List

m/z	z	Abund	Name	Formula	Ion	Score (DB)	Hits (DB)
187.06137	-1	10476.49	Ethyl Oxalacetate	C8 H12 O5	(M-H)-	87	10
188.0645	-1	963		C8 H12 O5	(M-H)-		

375.12741	-1	542.05			(2M-H)-		

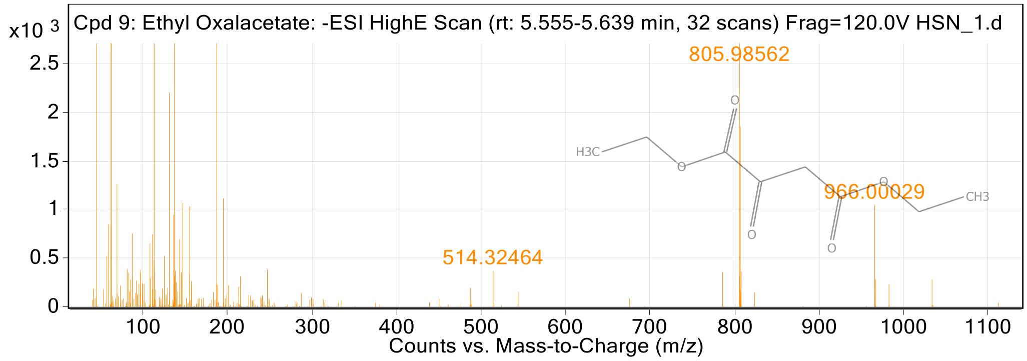

Peak List

m/z	z	Abund
44.99862		2973.45
61.98881		28886.52
62.00532		3034.84
68.99628		1262.17
112.9855		6238.37
130.9925		2201.7
137.02455	1	4379.3
187.06117	1	2711.73
805.98562	1	10806.53
806.98779	1	1856.15

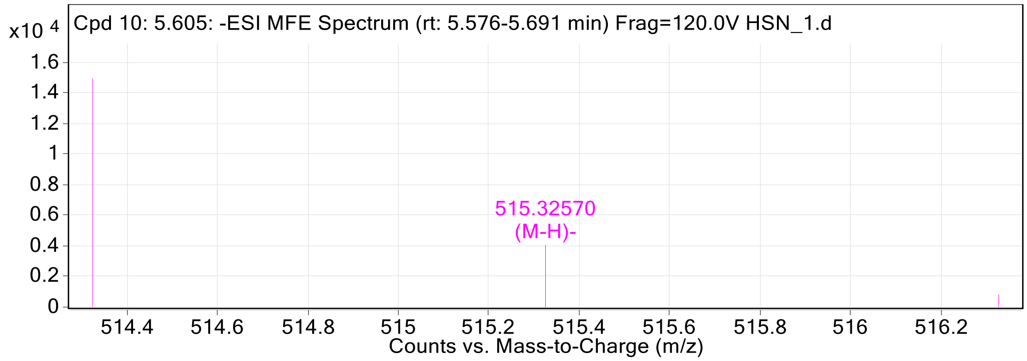

Peak List

m/z	z	Abund	Ion
514.32393	-1	14968.02	(M-H)-
515.3257	-1	4068.68	(M-H)-
516.32805	-1	816.54	(M-H)-

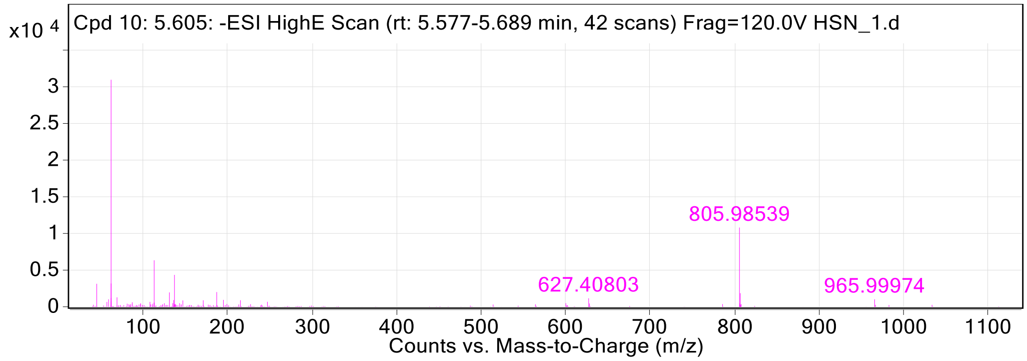

Peak List

m/z	z	Abund
44.99859		3144.09

61.9888		31029.7
62.00487		3182.04
68.9962		1294.03
112.98547		6355.61
130.99251		1965.81
137.02452	1	4361.14
187.06112		2012.14
805.98539	1	10830.3
806.98771	1	1859.37

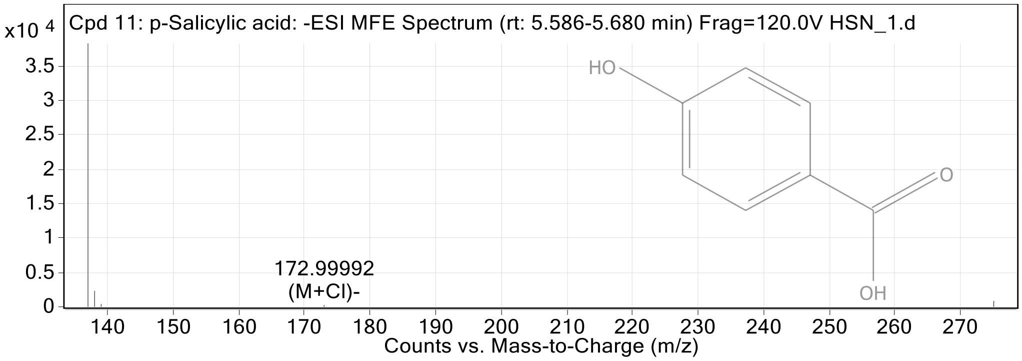

Peak List

m/z	z	Abund	Name	Formula	Ion	Score (DB)	Hits (DB)
137.02423	-1	38322.48	p-Salicylic acid	C7 H6 O3	(M-H)-	98.64	9
138.02774	-1	2326.06		C7 H6 O3	(M-H)-		
139.02772	-1	405.69		C7 H6 O3	(M-H)-		
172.99992	-1	279.6			(M+Cl)-		
275.05607	-1	859.87			(2M-H)-		

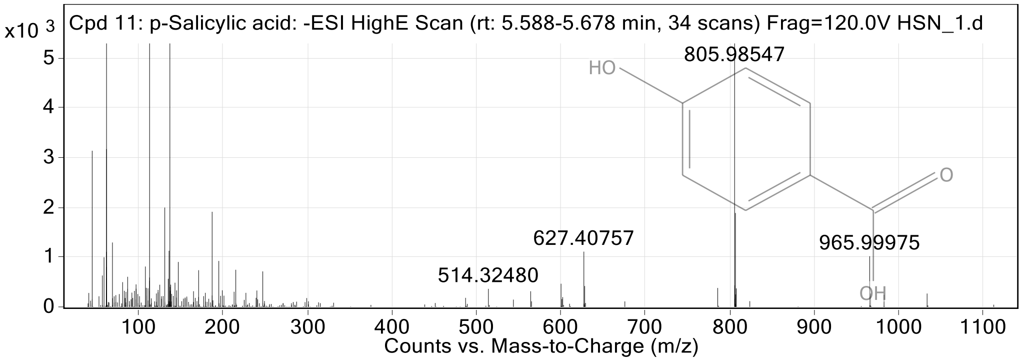

Peak List

m/z	z	Abund
44.99847		3133.43
61.98866		30942.25
62.00488		3166.66
68.99604		1292.39
112.98532		6358.64
130.99244		1993.44
137.02435	1	5286.73
187.06092		1908.53
805.98547	1	10911.36
806.98742	1	1884.42

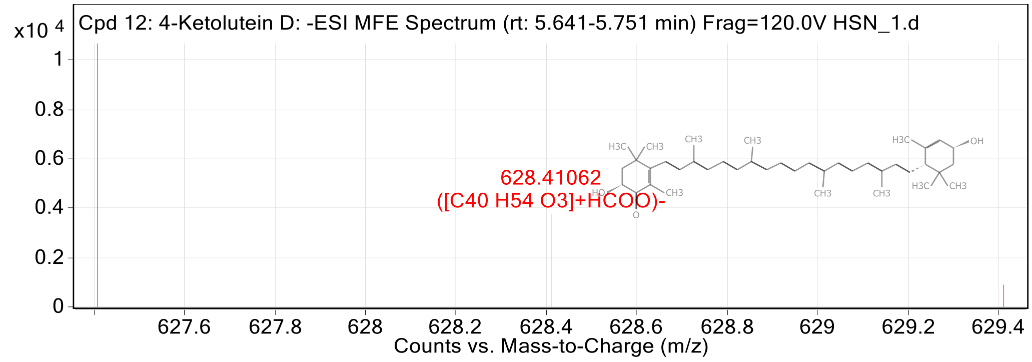

Peak List

m/z	z	Abund	Name	Formula	Ion	Score (DB)	Hits (DB)
627.40774	-1	10666.78	4-Ketolutein D	C40 H54 O3	(M+HCOO)-	84.21	10
628.41062	-1	3755.4		C40 H54 O3	(M+HCOO)-		
629.41159	-1	899.07		C40 H54 O3	(M+HCOO)-		

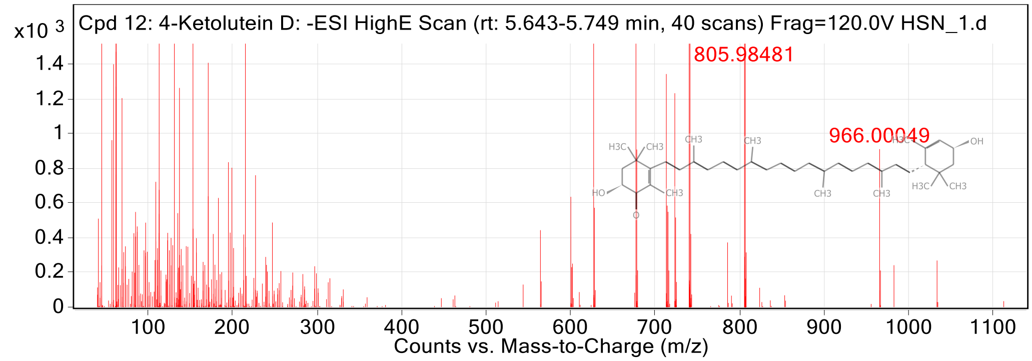

Peak List

m/z	z	Abund
44.99862		3673.65
61.98883		30803.32
112.98559		6163.23
153.0194		1960.33
215.0926		1902.74
677.49615	1	2163.27
740.49183	1	3987.46
741.49472	1	1623.64
805.98481	1	10171.1
806.98854	1	1823.35

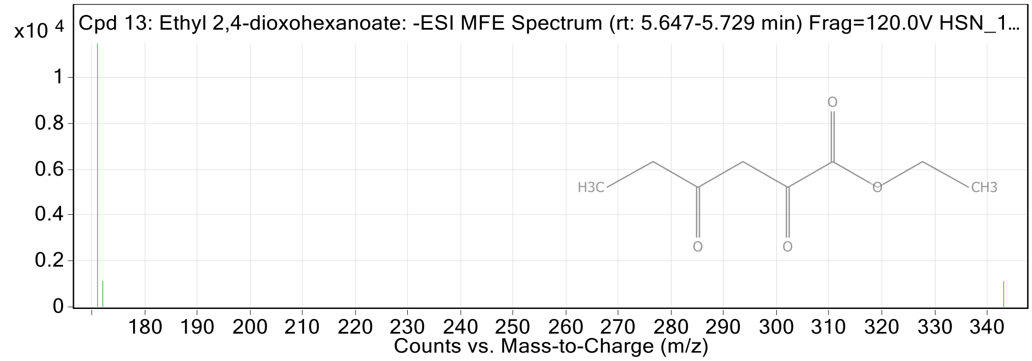

Peak List

m/z	z	Abund	Name	Formula	Ion	Score (DB)	Hits (DB)
171.06615	-1	11493.93	Ethyl 2,4-dioxohexanoate	C8 H12 O4	(M-H)-	86.94	10
172.06976	-1	1139.09		C8 H12 O4	(M-H)-		

343.13964	-1	1126.39			(2M-H)-		

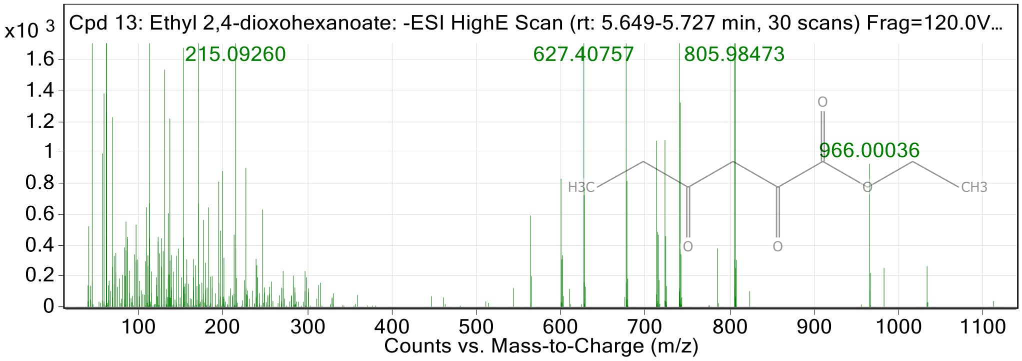

Peak List

m/z	z	Abund
44.99855		3522.81
61.98878		31356.69
62.00362		1769.95
112.98551		6163.47
215.0926		1925.15
627.40757	1	1951.77
677.49608	1	1904.96
740.49171	1	3232.23
805.98473	1	10251.09
806.98747	1	1780.92

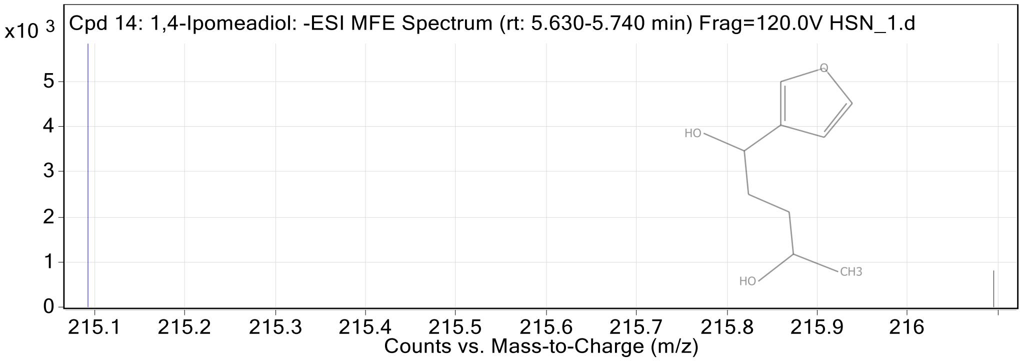

Peak List

m/z	z	Abund	Name	Formula	Ion	Score (DB)	Hits (DB)
215.09255	-1	5847.13	1,4-Ipomeadiol	C9 H14 O3	(M+HCOO)-	84.3	7
216.09554	-1	811.56		C9 H14 O3	(M+HCOO)-		

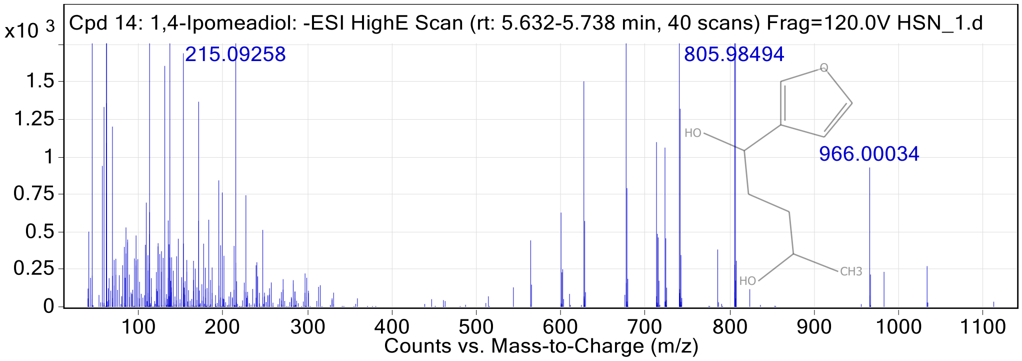

Peak List

m/z	z	Abund
44.99861		3527.73
61.98881		30496.8

62.00383		1934.51
112.98557		6110.15
137.02452		2193.02
215.09258		1757.34
677.49602	1	1861.63
740.49187	1	3261.27
805.98494	1	10294.14
806.98829	1	1817.37

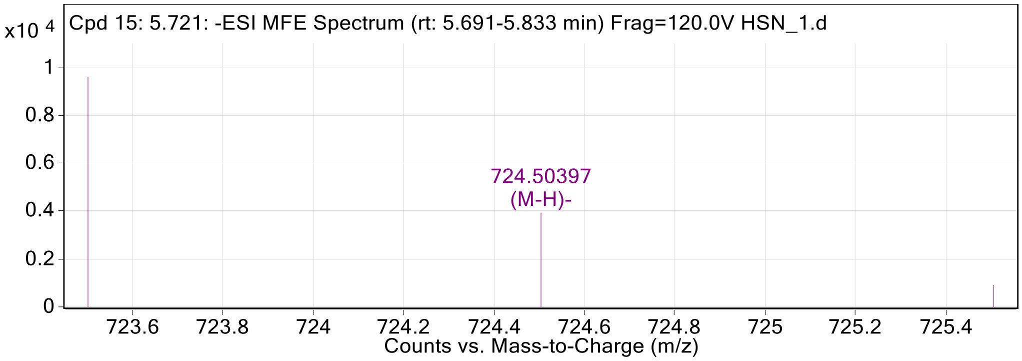

Peak List

m/z	z	Abund	Ion
723.5016	-1	9598.95	(M-H)-
724.50397	-1	3931.68	(M-H)-
725.50577	-1	914.42	(M-H)-

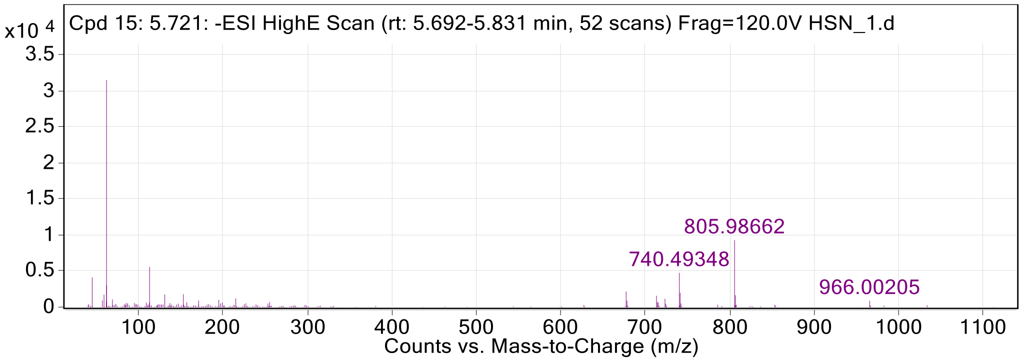

Peak List

m/z	z	Abund
44.99872		4112.49
59.01449		1739.28
61.9889		31533.02
62.00561		3048.78
112.98571		5568.33
153.01957		1796.12
677.49765	1	2158.47
740.49348	1	4756.09
741.49634	1	1991.17
805.98662	1	9297.1

Qualitative Analysis Report

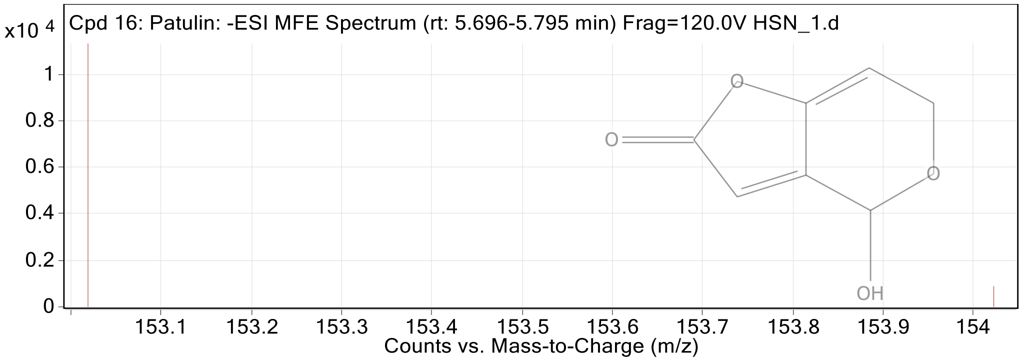

Peak List

m/z	z	Abund	Name	Formula	Ion	Score (DB)	Hits (DB)
153.01889	-1	11320.93	Patulin	C7 H6 O4	(M-H)-	86.34	9
154.0227	-1	894.29		C7 H6 O4	(M-H)-		

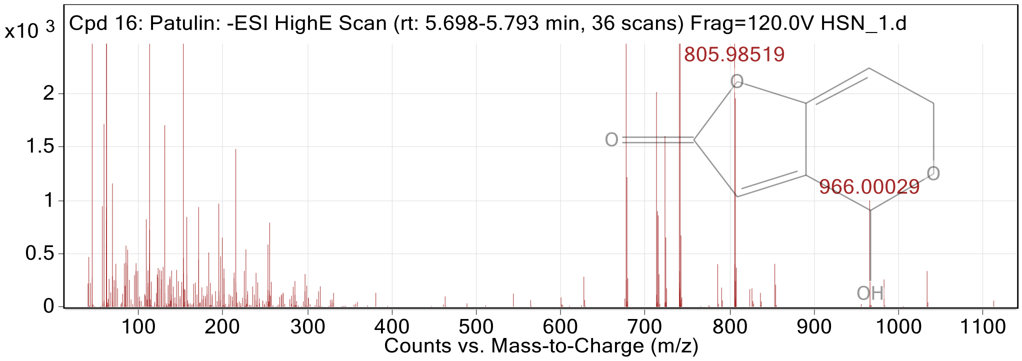

Peak List

m/z	z	Abund
44.9987		4069.01
61.98884		31344.49
62.00561		2996.65
112.98555		6639.07
153.01939		2472.87
677.49633	1	2916.94
713.4732	1	2018.65
740.4922	1	6074.17
741.49497	1	2530.1
805.98519	1	11133.97

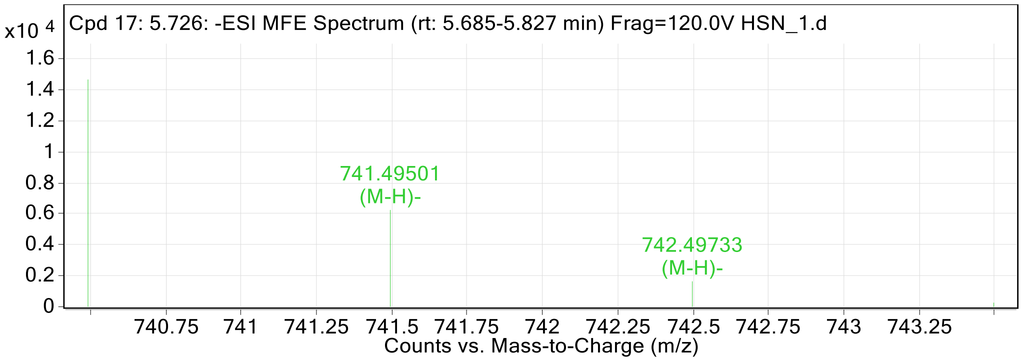

Peak List

m/z	z	Abund	Ion
740.49157	-1	14658.98	(M-H)-
741.49501	-1	6244.86	(M-H)-
742.49733	-1	1641.16	(M-H)-
743.49785	-1	262.78	(M-H)-

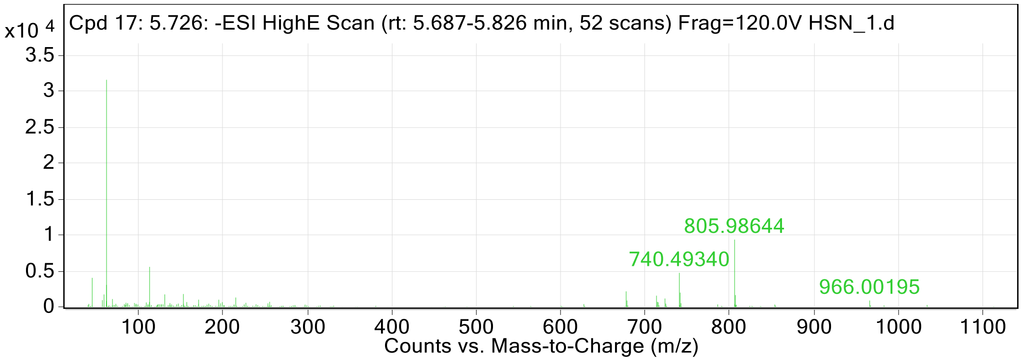

Peak List

m/z	z	Abund
44.99873		4031.48
61.98889		31558.52
62.00557		3051.61
112.98573		5557.18
130.99273		1728.84
153.01956		1781.48
677.49755	1	2152.96
740.4934	1	4724.21
741.49628	1	1977.95
805.98644	1	9343.42

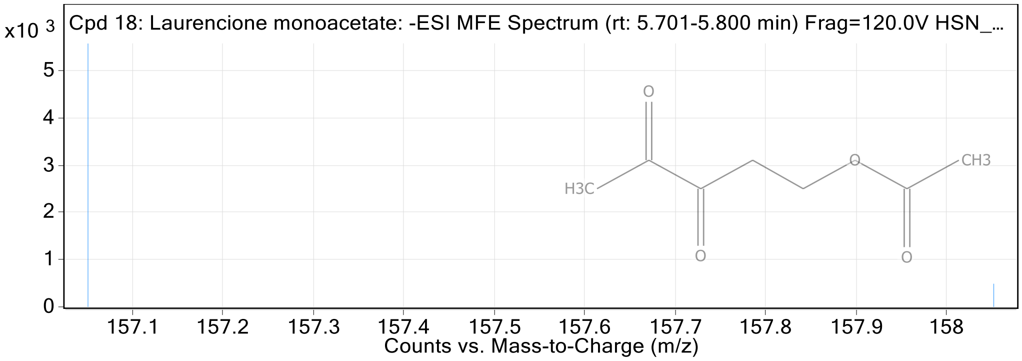

Peak List

m/z	z	Abund	Name	Formula	Ion	Score (DB)	Hits (DB)
157.05074	-1	5575.69	Laurencione monoacetate	C7 H10 O4	(M-H)-	82.94	10
158.05239	-1	490.12		C7 H10 O4	(M-H)-		

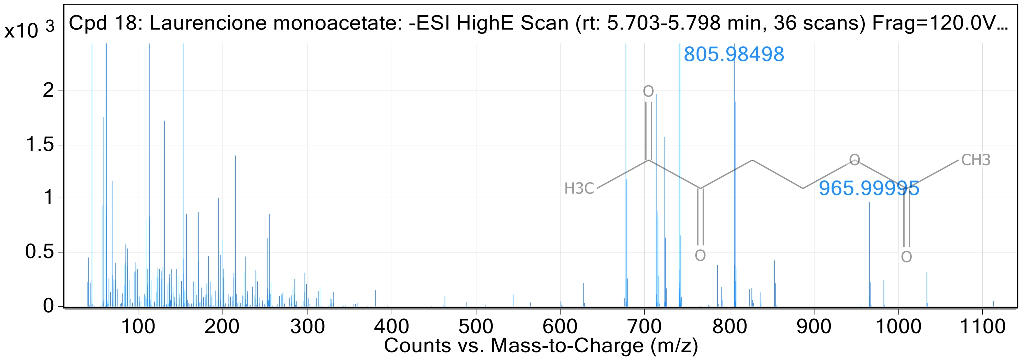

Peak List

m/z	z	Abund
44.99869		4105.8
61.98881		31286.39
62.00556		3017.35
112.98552		6343.2
153.01933		2435.21
677.49625	1	2832.7
713.47321	1	1966.21
740.49205	1	5877.24
741.49487	1	2459.34
805.98498	1	10710.51

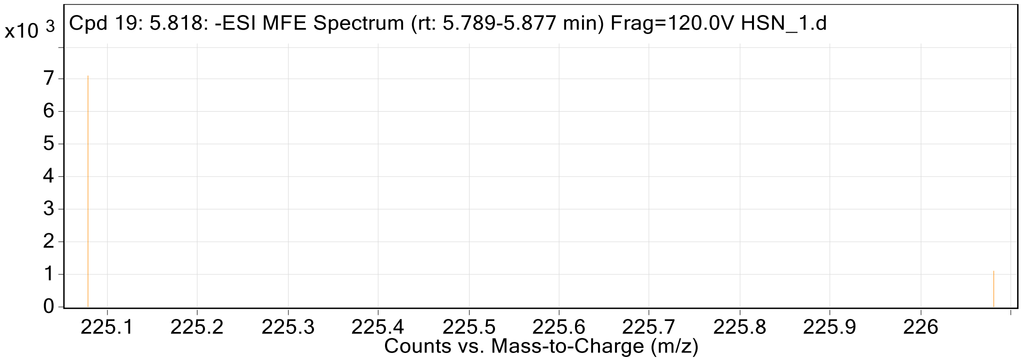

Peak List

m/z	z	Abund	Ion
225.07858	-1	7118.62	(M-H)-
226.08124	-1	1112.87	(M-H)-

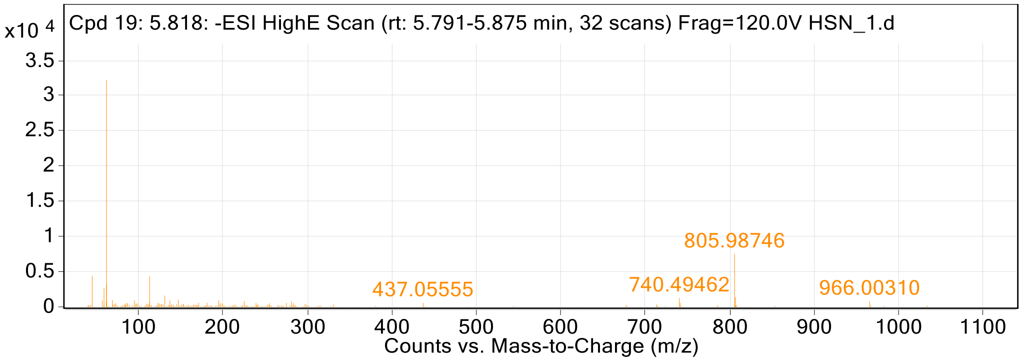

Peak List

m/z	z	Abund
44.99872		4407.62
59.0145		2690.07
61.98894		32248.15
62.0056		3241.52
112.98584		4346.7
130.99284		1594.99
146.96642		1014.44
740.49462	1	1231.96
805.98746	1	7481.82
806.99138	1	1363.12

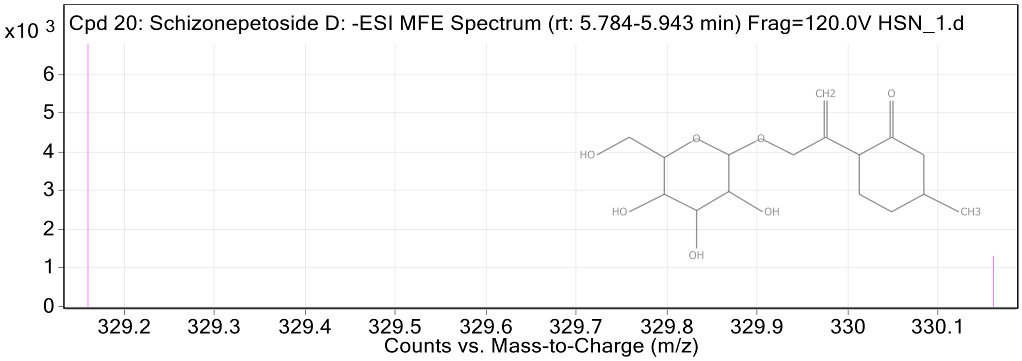

Peak List

m/z	z	Abund	Name	Formula	Ion	Score (DB)	Hits (DB)
329.16011	-1	6795.31	Schizonepetoside D	C16 H26 O7	(M-H)-	79.29	10
330.16181	-1	1316.26		C16 H26 O7	(M-H)-		

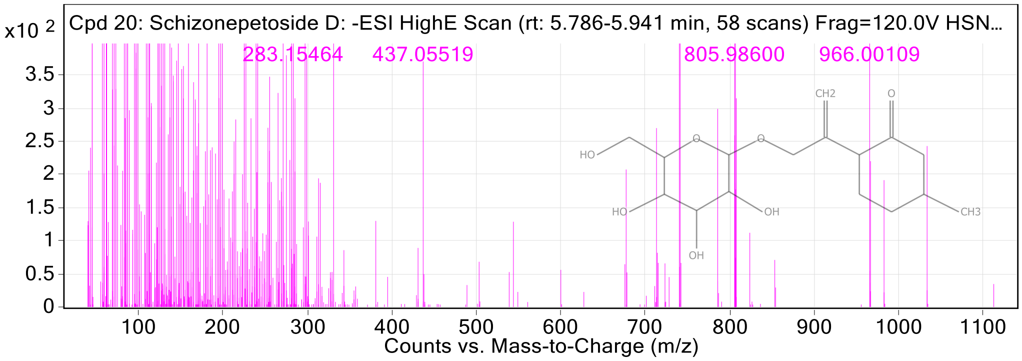

Peak List

m/z	z	Abund
44.99869		5274.67
57.03522		1047.21
59.01443		2868.34

61.9889		34401.45
62.00491		2601.21
112.9857		5379.11
130.99264		1340.84
146.96614		1020.87
805.986	1	9231.81
806.98922	1	1626.35

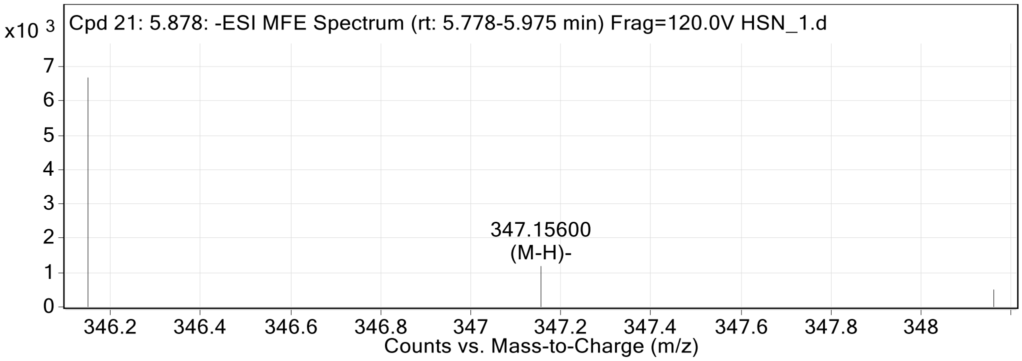

Peak List

m/z	z	Abund	Ion
346.15048	-1	6677.46	(M-H)-
347.156	-1	1188.76	(M-H)-
348.16113	-1	505.71	(M-H)-

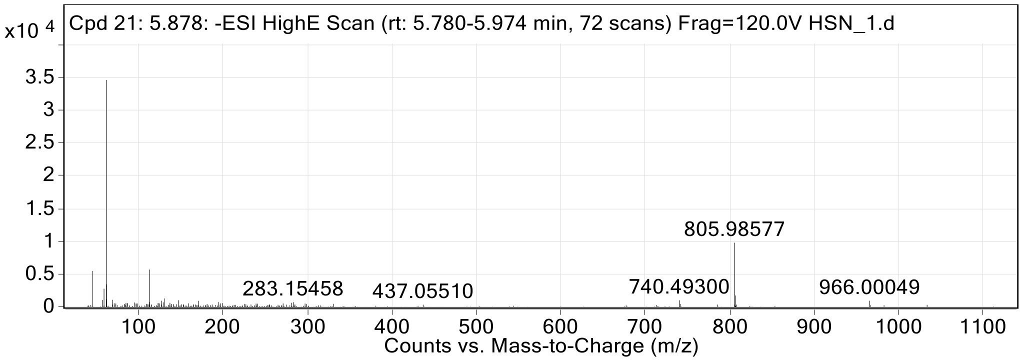

Peak List

m/z	z	Abund
44.99867		5442.26
59.01442		2739.14
61.98886		34591.42
62.0054		3414.72
62.01448		1133.38
68.99635		1056.87
112.98563		5669.11
130.99262		1249.68
805.98577	1	9771.65
806.98887	1	1714.27

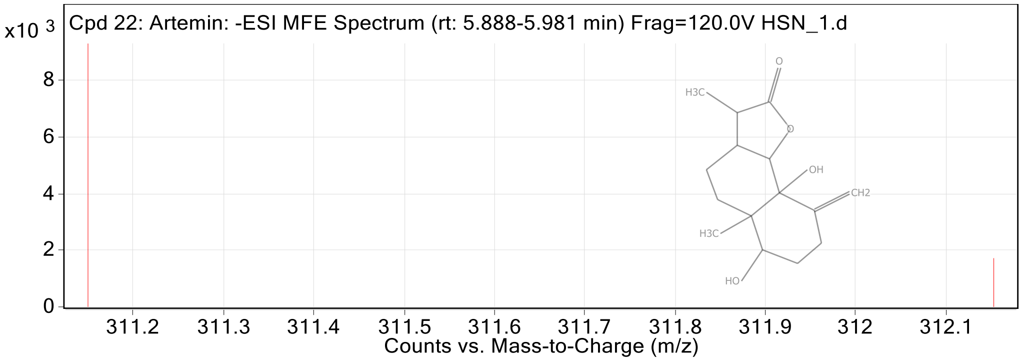

Peak List

m/z	z	Abund	Name	Formula	Ion	Score (DB)	Hits (DB)
311.14982	-1	9305.52	Artemin	C15 H22 O4	(M+HCOO)-	85.08	10
312.15315	-1	1720.81		C15 H22 O4	(M+HCOO)-		

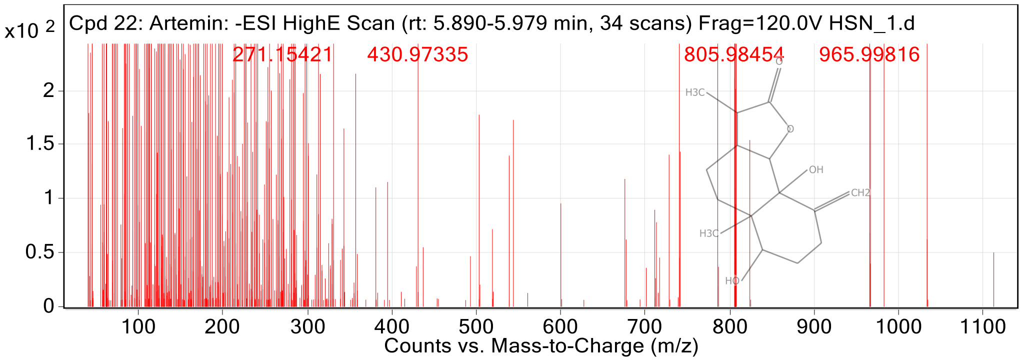

Peak List

m/z	z	Abund
44.99861		6744.34
59.01438		2621.44
61.9888		36475.87
62.00541		3541.23
62.0147		1255.06
112.9855		6366.04
127.07621		1516.66
171.06607		1231.61
805.98454	1	11067.38
806.98709	1	1927.32

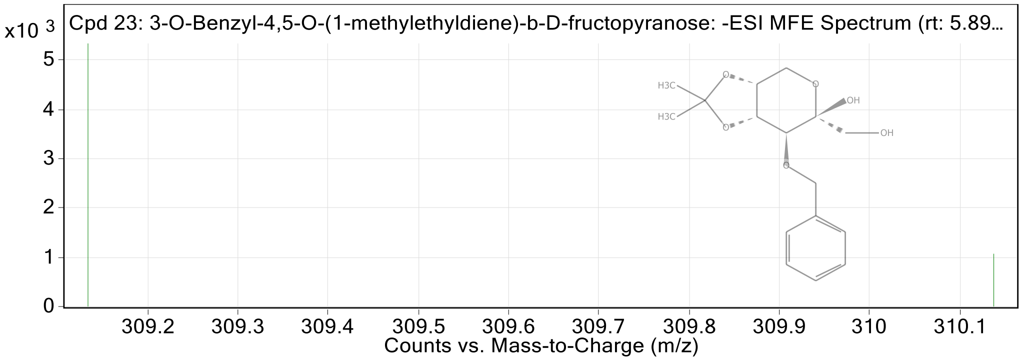

Peak List

m/z	z	Abund	Name	Formula	Ion	Score (DB)	Hits (DB)
309.13417	-1	5336.71	3-O-Benzyl-4,5-O-(1-methylethyldiene)-b-D-fructopyranose	C16 H22 O6	(M-H)-	83.65	10

310.13746	-1	1070.72		C16 H22 O6	(M-H)-		

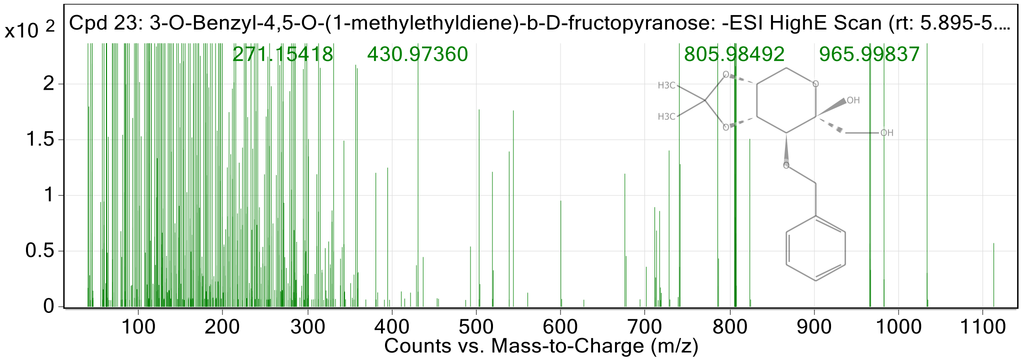

Peak List

m/z	z	Abund
44.99858		6733.92
59.01433		2493.57
61.98878		35747.38
62.00536		3516.97
62.01458		1239.19
112.98546		6280.54
127.07623		1521.04
171.06606		1245.3
805.98492	1	10972.81
806.98762	1	1906.66

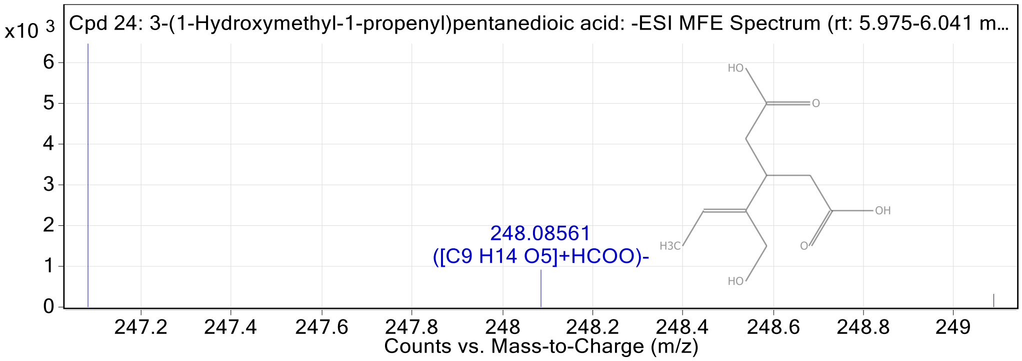

Peak List

m/z	z	Abund	Name	Formula	Ion	Score (DB)	Hits (DB)
247.08259	-1	6469.74	3-(1-Hydroxymethyl-1-propenyl)pentanedioic acid	C9 H14 O5	(M+HCOO)-	94.78	4
248.08561	-1	917.54		C9 H14 O5	(M+HCOO)-		
249.08822	-1	324.09		C9 H14 O5	(M+HCOO)-		

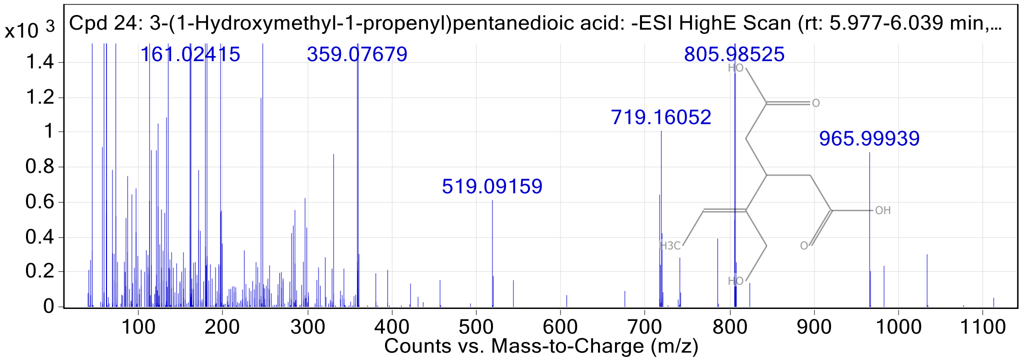

Peak List

m/z	z	Abund

44.99854		3533.92
61.98871		22242.1
62.00485		2352.42
72.99322		2458.64
112.98565		5834.19
161.02415	1	20669.08
179.03494		2793.88
197.04543	1	5458.61
359.07679	1	8398.38
805.98525	1	10113.34

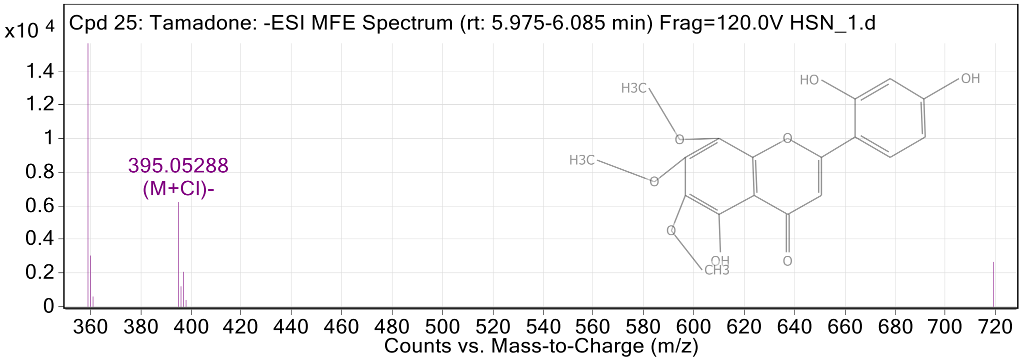

Peak List

m/z	z	Abund	Name	Formula	Ion	Score (DB)	Hits (DB)
359.07717	-1	15669.17	Tamadone	C18 H16 O8	(M-H)-	99.76	10
360.08081	-1	3044.12		C18 H16 O8	(M-H)-		
361.08272	-1	607.28		C18 H16 O8	(M-H)-		
395.05288	-1	6229.69			(M+Cl)-		
396.05739	-1	1210.03			(M+Cl)-		
397.05067	-1	2081.71			(M+Cl)-		
398.05441	-1	406.23			(M+Cl)-		
719.16048	-1	2674.29			(2M-H)-		

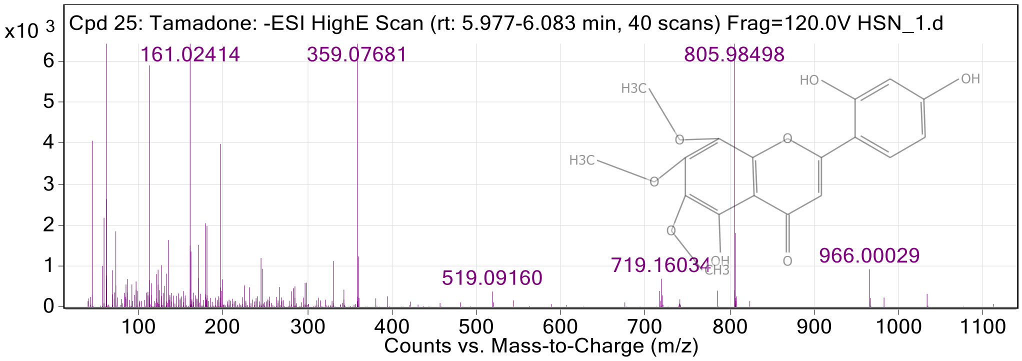

Peak List

m/z	z	Abund
44.99851		4055.89
59.01422		2174.98
61.98871		25455.99
62.00471		2630.29
112.98559		5900.43
161.02414	1	15113.39
179.03493		2041.78
197.04548	1	3981.02
359.07681	1	6432.9
805.98498	1	10430.5

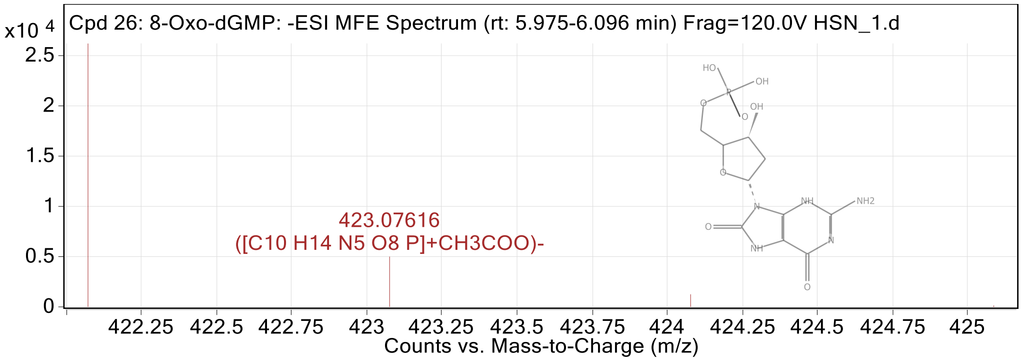

Peak List

m/z	z	Abund	Name	Formula	Ion	Score (DB)	Hits (DB)
422.07257	-1	26178.49	8-Oxo-dGMP	C10 H14 N5 O8 P	(M+CH3COO)-	93.31	4
423.07616	-1	4994.68		C10 H14 N5 O8 P	(M+CH3COO)-		
424.07774	-1	1250.4		C10 H14 N5 O8 P	(M+CH3COO)-		
425.08656	-1	132.33		C10 H14 N5 O8 P	(M+CH3COO)-		

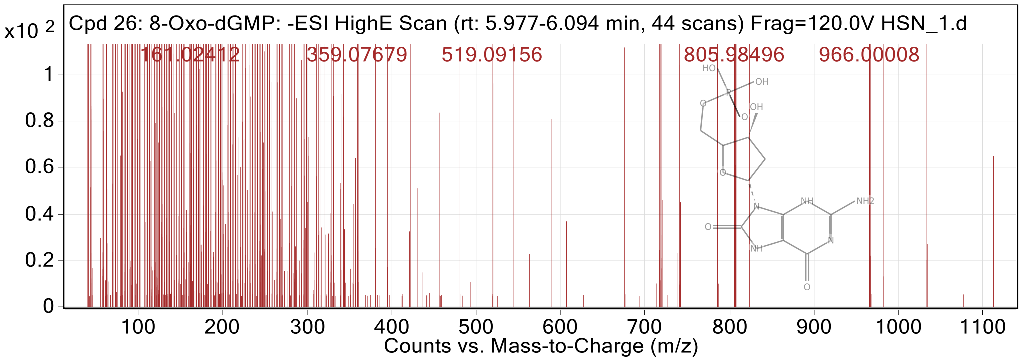

Peak List

m/z	z	Abund
44.99851		4182.94
59.01422		2188.42
61.98871		26458.4
62.00482		2629.33
112.9856		5912.58
161.02412	1	13839.92
180.98905		1898.02
197.04546	1	3638.94
359.07679	1	5898.57
805.98496	1	10508.6

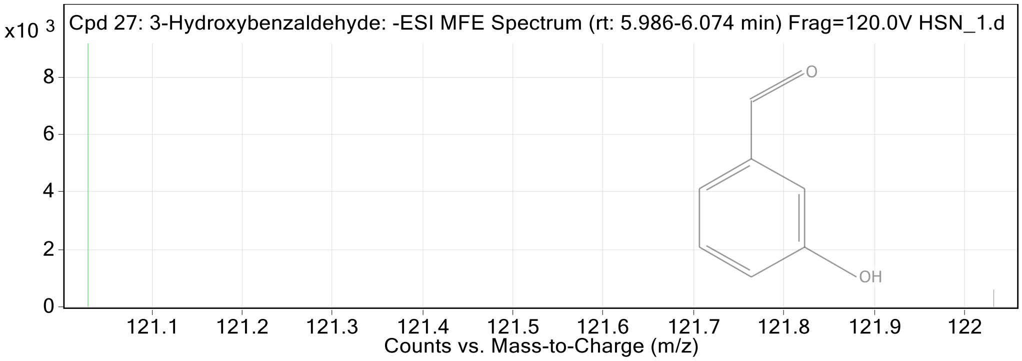

Peak List

m/z	z	Abund	Name	Formula	Ion	Score (DB)	Hits (DB)
121.02921	-1	9163.53	3-Hydroxybenzaldehyde	C7 H6 O2	(M-H)-	86.9	6

122.03264	-1	602.39		C7 H6 O2	(M-H)-		

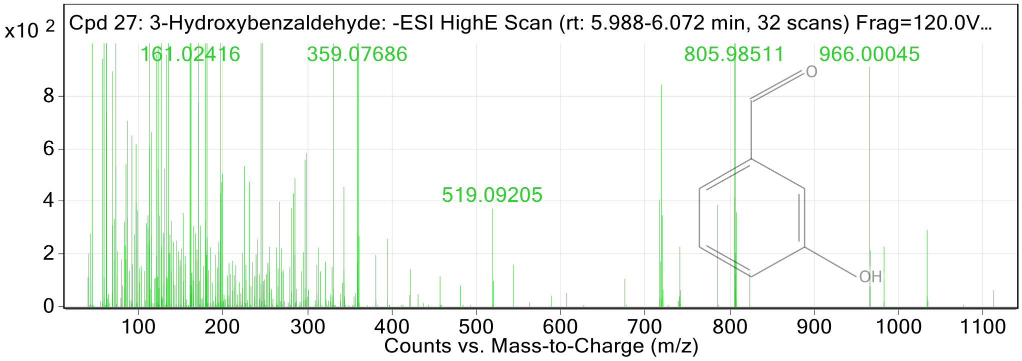

Peak List

m/z	z	Abund
44.9985		3817.81
61.98872		23986.41
62.00463		2469.85
112.98565		5889.87
161.02416	1	18076.33
179.03493		2369.45
180.98906		2221.47
197.0455	1	4721.67
359.07686	1	7707.86
805.98511	1	10302.27

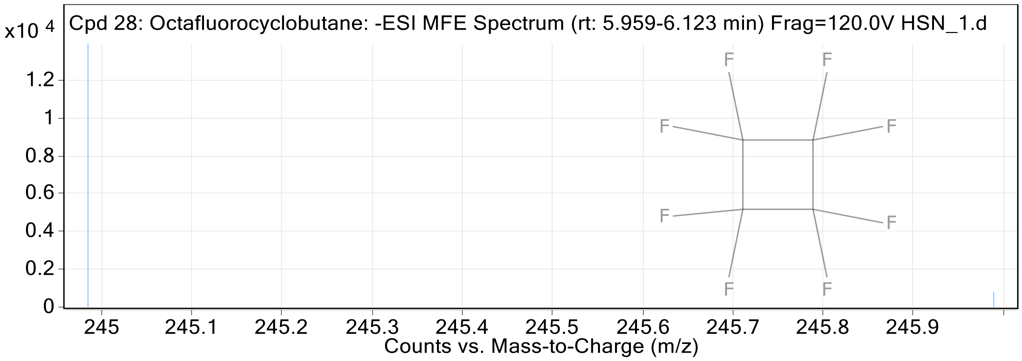

Peak List

m/z	z	Abund	Name	Formula	Ion	Score (DB)	Hits (DB)
244.98507	-1	13923.27	Octafluorocyclobutane	C4 F8	(M+HCOO)-	87.1	1
245.98922	-1	807.24		C4 F8	(M+HCOO)-		

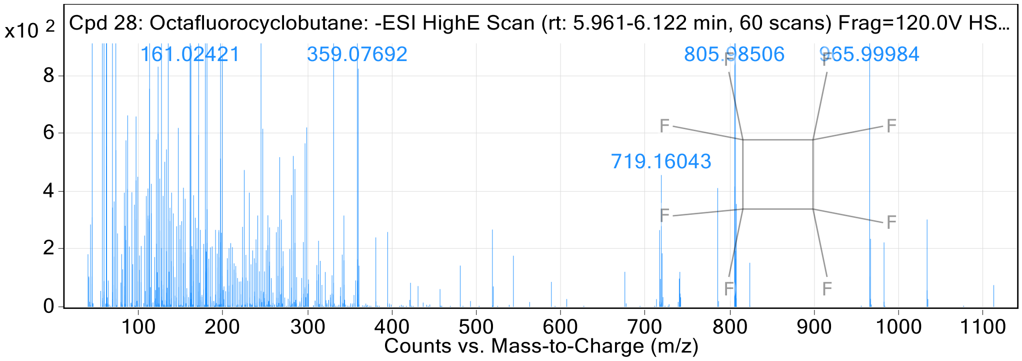

Peak List

m/z	z	Abund
44.99857		4732.57
59.01431		2390.45

61.98877		30102.65
62.0049		2694.97
112.98564		6311.47
161.02421	1	10268.94
197.04557		2727.22
359.07692	1	4356.86
805.98506	1	11127.21
806.98833	1	1901.2

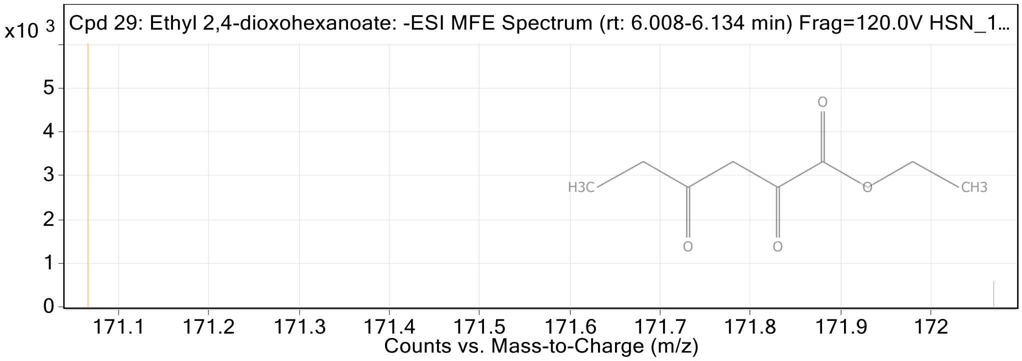

Peak List

m/z	z	Abund	Name	Formula	Ion	Score (DB)	Hits (DB)
171.06615	-1	6017.21	Ethyl 2,4-dioxohexanoate	C8 H12 O4	(M-H)-	87.09	10
172.06939	-1	582.5		C8 H12 O4	(M-H)-		

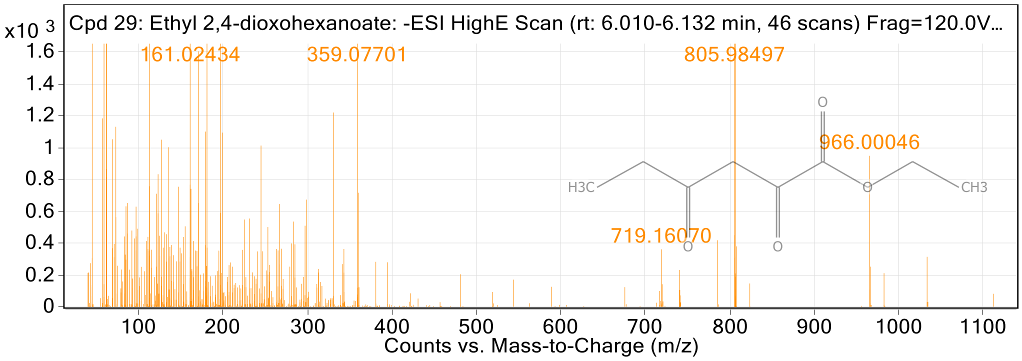

Peak List

m/z	z	Abund
44.99862		5104.07
59.01434		2625.07
61.98886		32857.43
62.00494		2677.42
112.98576		6239.14
161.02434	1	8492.49
197.04565		2267.05
359.07701	1	3818.23
805.98497	1	11090.96
806.98794	1	1905.17

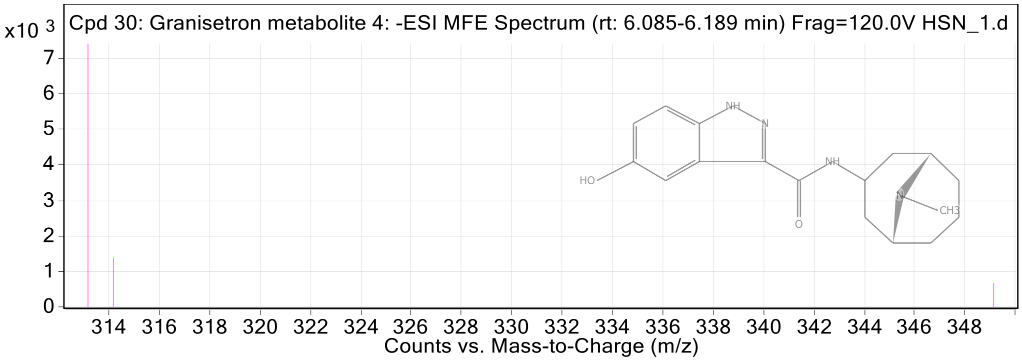

Peak List

m/z	z	Abund	Name	Formula	Ion	Score (DB)	Hits (DB)
313.16795	-1	7415.52	Granisetron metabolite 4	C17 H22 N4 O2	(M-H)-	82.92	1
314.17002	-1	1385.94		C17 H22 N4 O2	(M-H)-		
349.14158	-1	676.08			(M+Cl)-		

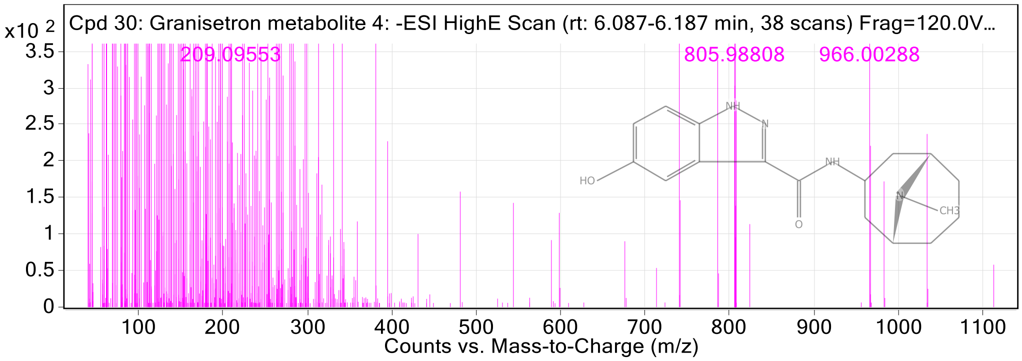

Peak List

m/z	z	Abund
44.99897		6618.3
57.03552		1495.06
59.01474		3042.25
61.98924		42379.86
62.00625		4004.62
78.95977		2123.24
112.98629		5267.58
209.09553	1	4196.25
805.98808	1	9375.32
806.99129	1	1618.85

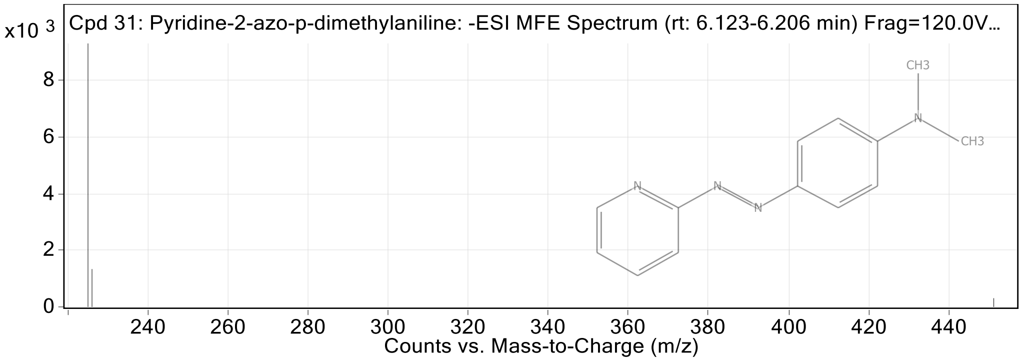

Peak List

m/z	z	Abund	Name	Formula	Ion	Score (DB)	Hits (DB)
225.11423	-1	9303.73	Pyridine-2-azo-p-dimethylaniline	C13 H14 N4	(M-H)-	86.21	7

226.11766	-1	1335.97		C13 H14 N4	(M-H)-		
451.23888	-1	307.25			(2M-H)-		

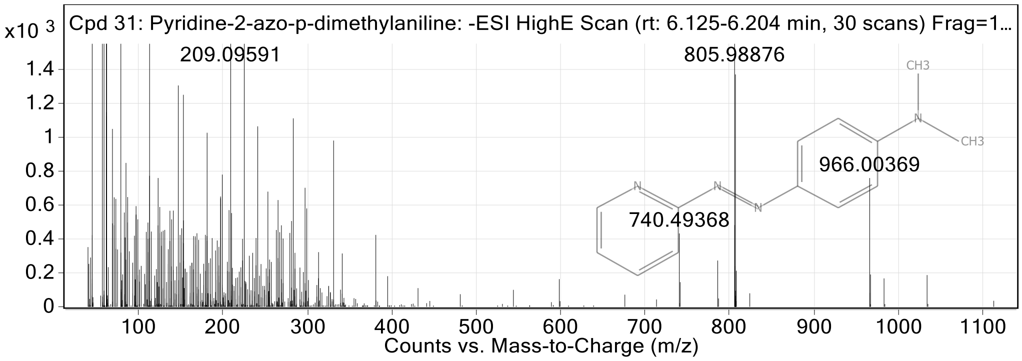

Peak List

m/z	z	Abund
44.99897		6858.94
57.03556		1653.28
59.01473		2878.78
61.98926		41725.55
62.00475		1972.86
62.00729		2001.21
78.95984		3024.29
112.9863		4414.9
209.09591	1	5956.23
805.98876	1	7945.7

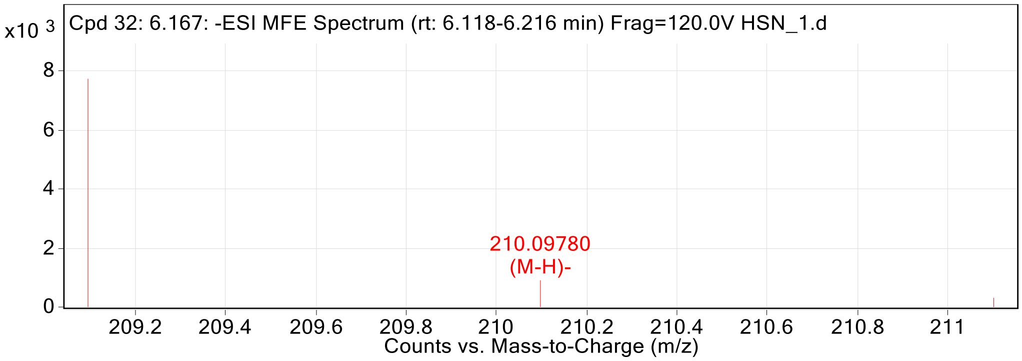

Peak List

m/z	z	Abund	Ion
209.09533	-1	7743.61	(M-H)-
210.0978	-1	908.42	(M-H)-
211.10243	-1	310.28	(M-H)-

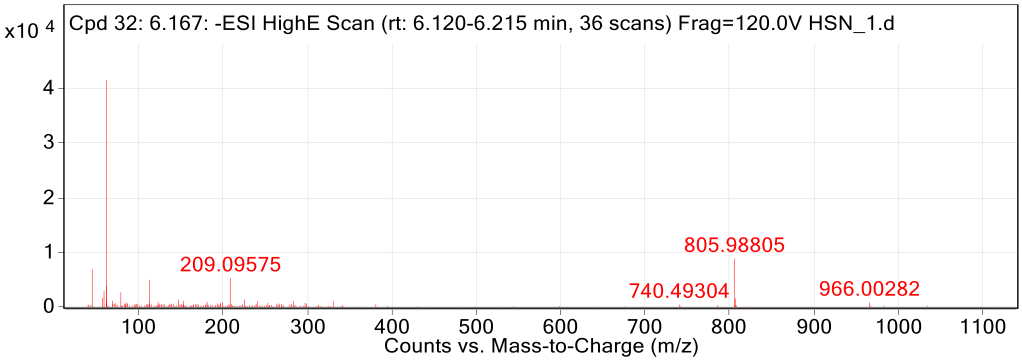

Peak List

m/z	z	Abund

44.99889		6859.73
57.03547		1642.43
59.01468		2889.63
61.98918		41598.22
62.0059		3900.41
78.95973		2642.07
112.98616		4974.38
209.09575	1	5271.46
805.98805	1	8839.79
806.99046	1	1546.39

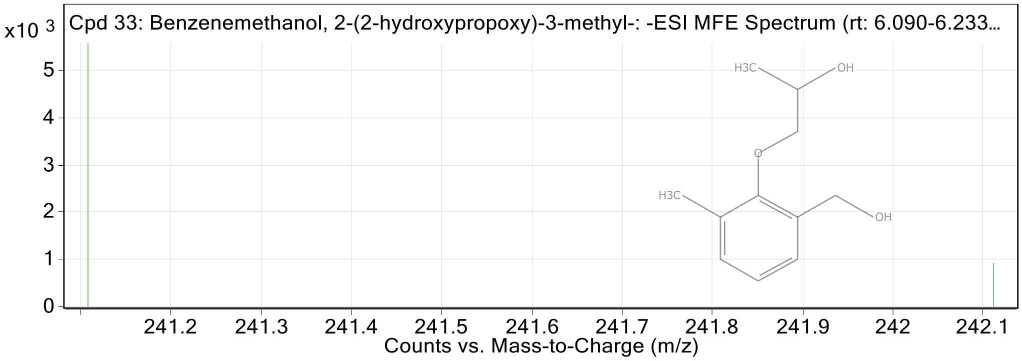

Peak List

m/z	z	Abund	Name	Formula	Ion	Score (DB)	Hits (DB)
241.10821	-1	5570.39	Benzenemethanol, 2-(2-hydroxypropoxy)-3-methyl-	C11 H16 O3	(M+HCOO)-	83.41	10
242.11166	-1	929.7		C11 H16 O3	(M+HCOO)-		

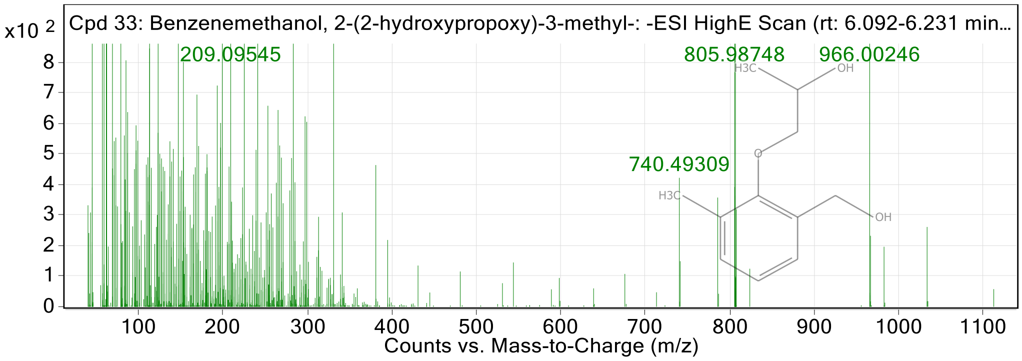

Peak List

m/z	z	Abund
44.9989		6694.87
57.03539		1610.32
59.01463		2926.92
61.98914		41260.7
62.0061		3890.62
78.95967		1921.53
112.98609		5635.15
209.09545	1	3941.43
805.98748	1	10000.23
806.99052	1	1727.99

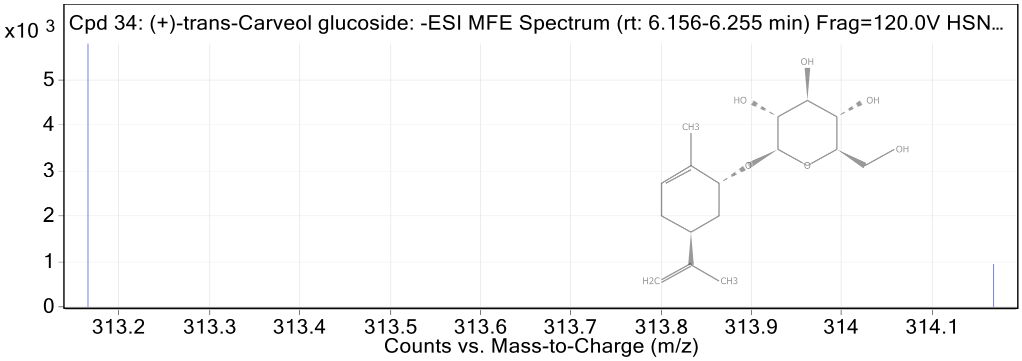

Peak List

m/z	z	Abund	Name	Formula	Ion	Score (DB)	Hits (DB)
313.16528	-1	5796.05	(+)-trans-Carveol glucoside	C16 H26 O6	(M-H)-	84.62	10
314.16841	-1	942.94		C16 H26 O6	(M-H)-		

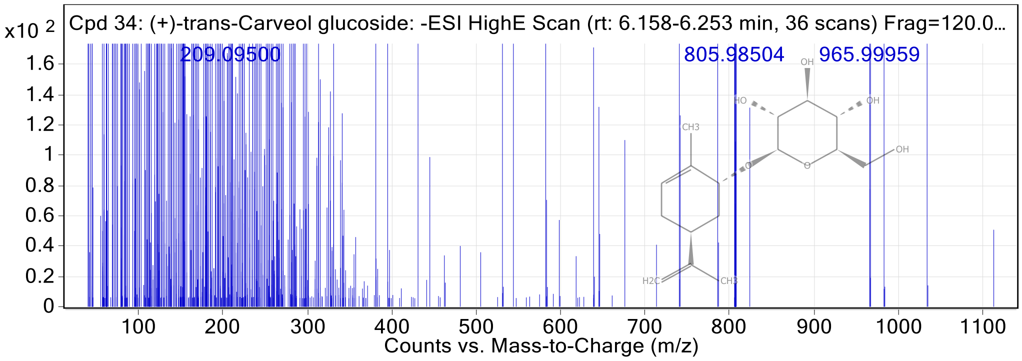

Peak List

m/z	z	Abund
44.99877		6630.1
57.03511		1644.74
59.01436		2661.75
61.98889		39373.3
62.00551		3749.22
78.95935		2122.43
112.98564		6169.89
209.095	1	4449.47
805.98504	1	10999.02
806.98809	1	1894.85

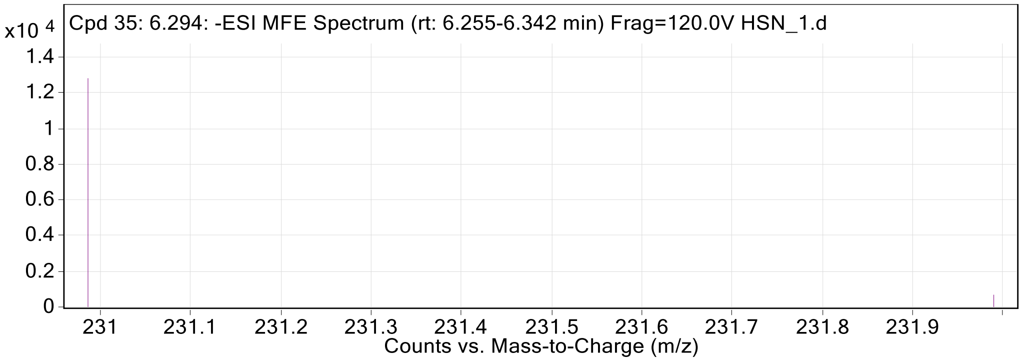

Peak List

m/z	z	Abund	Ion
230.98607	-1	12816.01	(M-H)-
231.98976	-1	684.2	(M-H)-

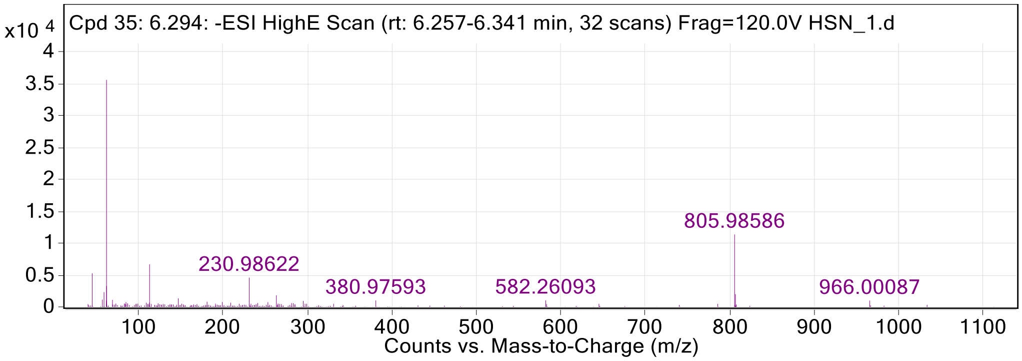

Peak List

m/z	z	Abund
44.99861		5273.63
59.01431		2313.74
61.98881		35605.02
62.00564		3265.13
112.98555		6684.46
146.96559		1350.79
230.98622		4591.74
263.12884		1819.93
805.98586	1	11361.79
806.98865	1	1993.21

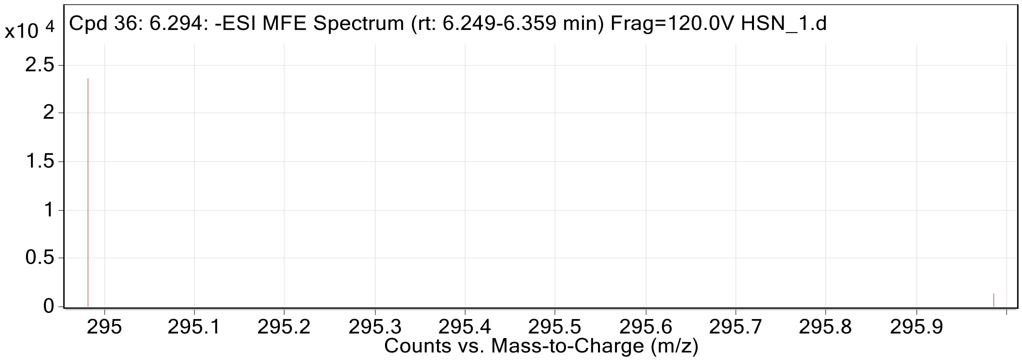

Peak List

m/z	z	Abund	Ion
294.98231	-1	23651	(M-H)-
295.98603	-1	1381.83	(M-H)-

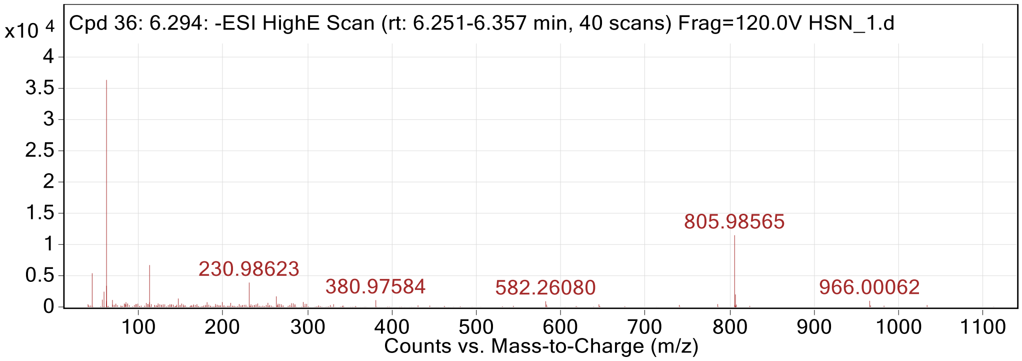

Peak List

m/z	z	Abund
44.99863		5389.68
59.0143		2444.4
61.98882		36276.93

62.00556		3378.42
112.98555		6694.74
146.96576		1347.28
230.98623		3899.2
263.1288		1692.85
805.98565	1	11464.08
806.98859	1	1998.16

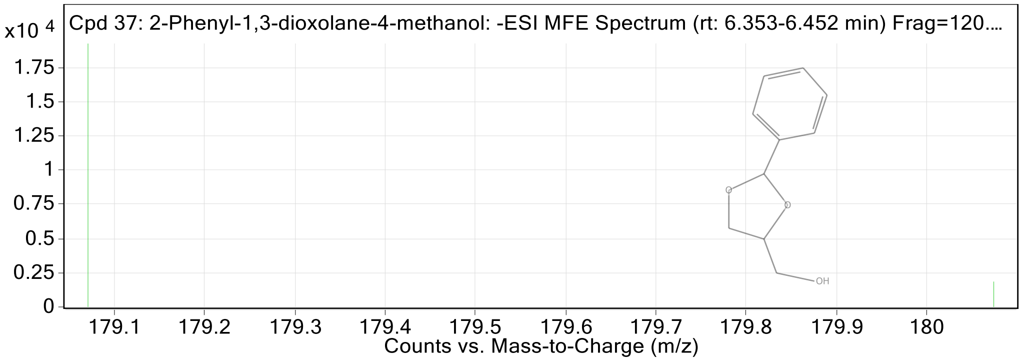

Peak List

m/z	z	Abund	Name	Formula	Ion	Score (DB)	Hits (DB)
179.07101	-1	19252.69	2-Phenyl-1,3-dioxolane-4-methanol	C10 H12 O3	(M-H)-	86.1	10
180.07414	-1	1851.99		C10 H12 O3	(M-H)-		

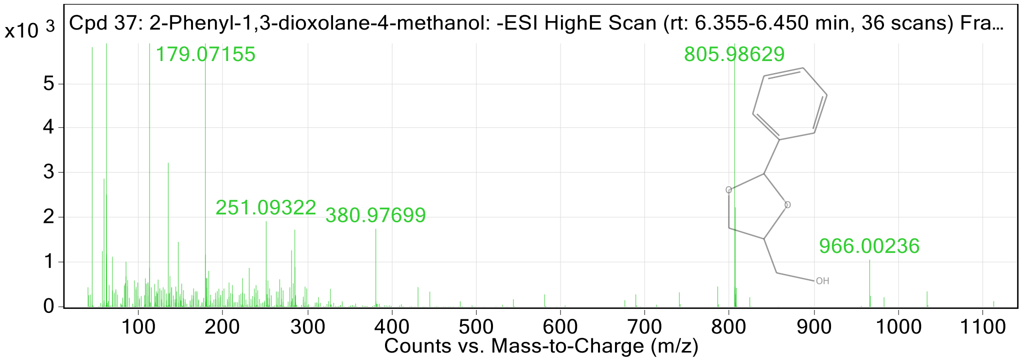

Peak List

m/z	z	Abund
44.99876		5816.15
59.01436		2871.59
61.98891		35827.73
62.00611		2515.95
112.98564		6953.53
135.08155		3226.58
179.07155	1	5900.06
251.09322		1917.95
805.98629	1	12462.21
806.98933	1	2228.22

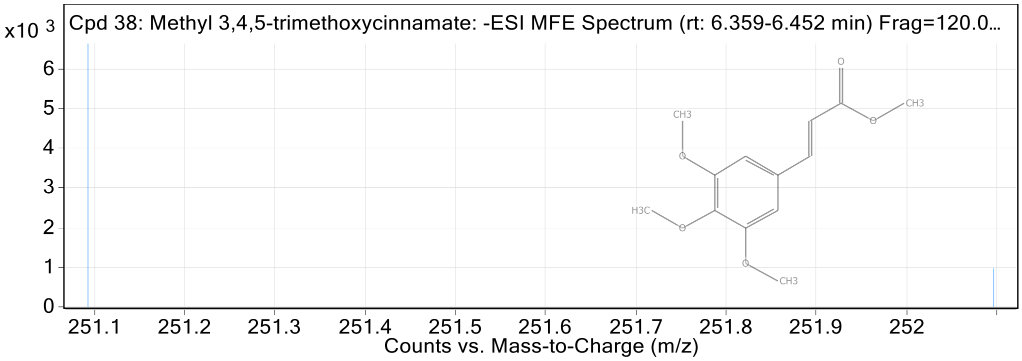

Peak List

m/z	z	Abund	Name	Formula	Ion	Score (DB)	Hits (DB)
251.09275	-1	6638.62	Methyl 3,4,5-trimethoxycinnamate	C13 H16 O5	(M-H)-	86.05	10
252.09647	-1	968.12		C13 H16 O5	(M-H)-		

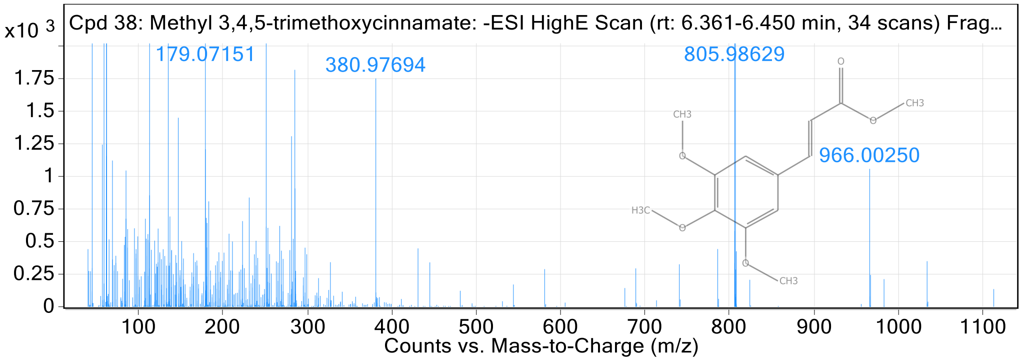

Peak List

m/z	z	Abund
44.99873		5823.66
59.01432		2849.92
61.98888		35655.07
62.00613		2431.15
112.98561		6941.55
135.08151		3381.06
179.07151	1	6220.42
251.09319		2023.48
805.98629	1	12499.38
806.9894	1	2228.51

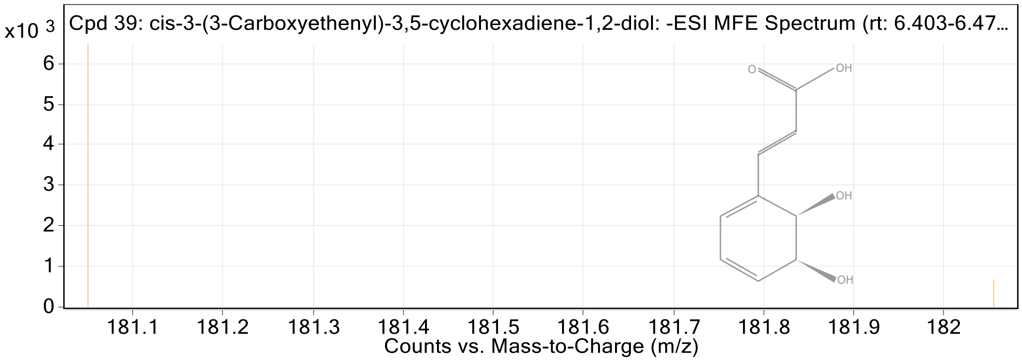

Peak List

m/z	z	Abund	Name	Formula	Ion	Score (DB)	Hits (DB)
181.05053	-1	6490.11	cis-3-(3-Carboxyethenyl)-3,5-cyclohexadiene-1,2-diol	C9 H10 O4	(M-H)-	85.13	10

182.05501	-1	682.03		C9 H10 O4	(M-H)-		

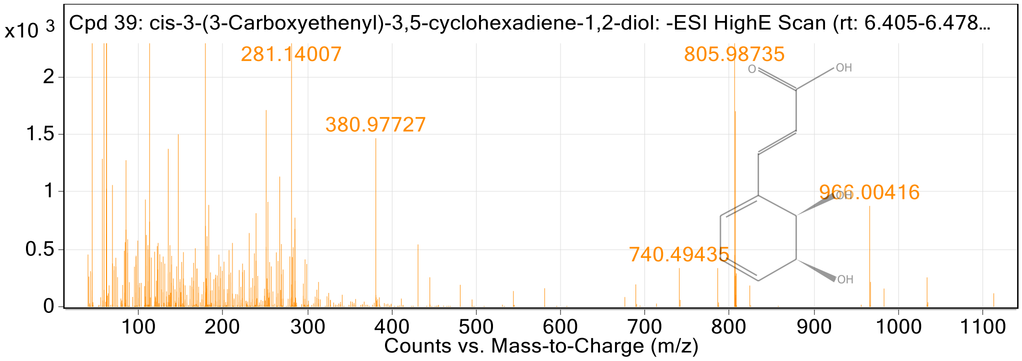

Peak List

m/z	z	Abund
44.99873		6214.05
59.01436		2777.45
61.98891		36032.8
62.00554		3644.7
112.98576		5658.79
179.07178		2290.12
251.09333		1709.72
281.14007	1	2520.82
805.98735	1	9946.1
806.9903	1	1699.46

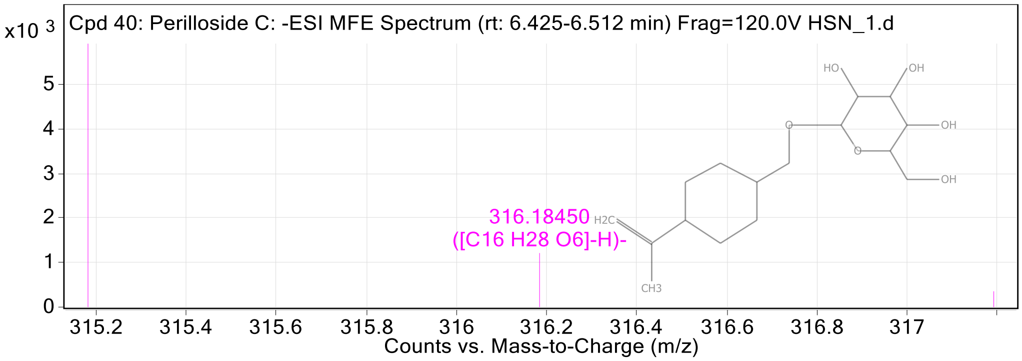

Peak List

m/z	z	Abund	Name	Formula	Ion	Score (DB)	Hits (DB)
315.18234	-1	5914.14	Perilloside C	C16 H28 O6	(M-H)-	87.25	8
316.1845	-1	1212.81		C16 H28 O6	(M-H)-		
317.19244	-1	354.5		C16 H28 O6	(M-H)-		

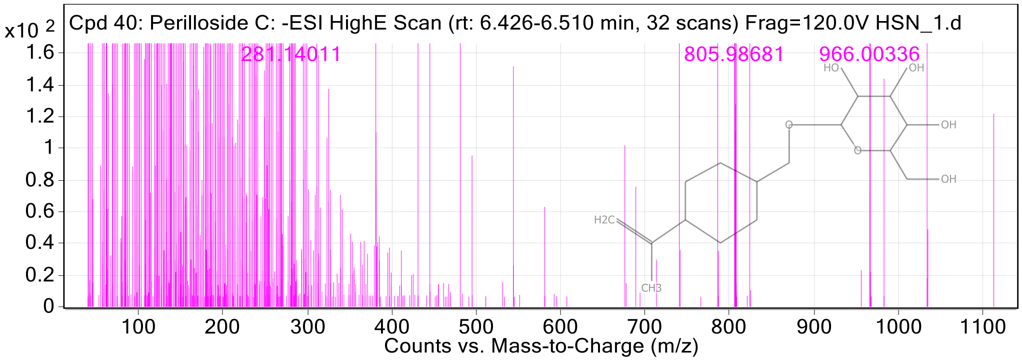

Peak List

m/z	z	Abund
44.99873		6272.48

59.01448		2779.09
61.98893		37782.84
62.00477		2116.94
62.00701		1409.89
112.98586		5902.17
146.96643		1479.13
281.14011	1	2368.58
805.98681	1	10231.3
806.99034	1	1762.45

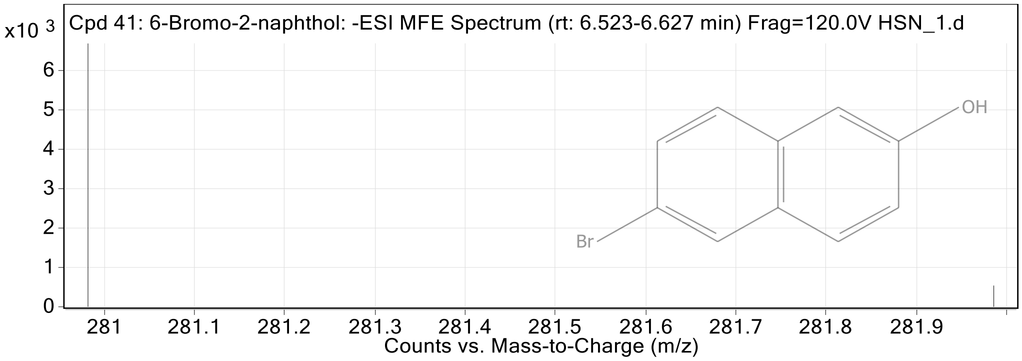

Peak List

m/z	z	Abund	Name	Formula	Ion	Score (DB)	Hits (DB)
280.98209	-1	6681.55	6-Bromo-2-naphthol	C10 H7 Br O	(M+CH3COO)-	58.91	1
281.98605	-1	539.46		C10 H7 Br O	(M+CH3COO)-		

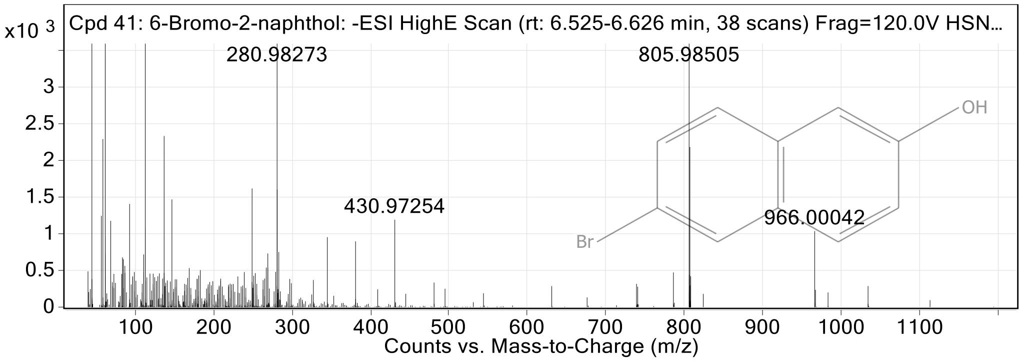

Peak List

m/z	z	Abund
44.99853		5365.58
59.01425		2289.76
61.98866		36496.84
62.00597		3391.51
112.9853		7069.98
137.02413		2330.97
249.11267		1615.57
280.98273		3589.53
805.98505	1	12277.68
806.9879	1	2181.32

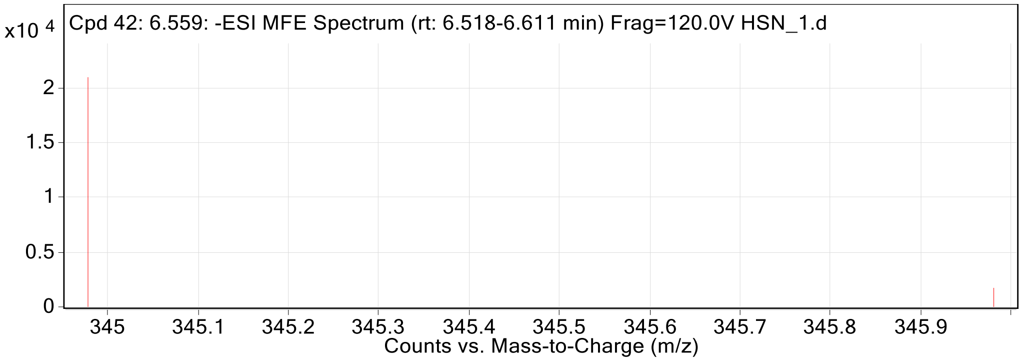

Peak List

m/z	z	Abund	Ion
344.97811	-1	20946.84	(M-H)-
345.98111	-1	1733.63	(M-H)-

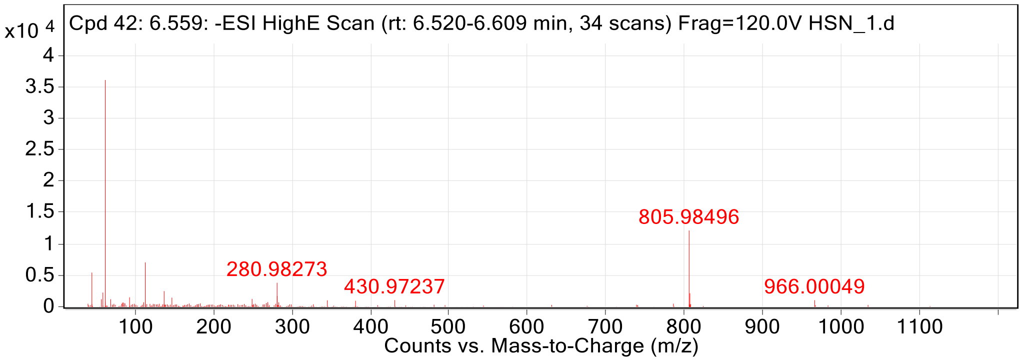

Peak List

m/z	z	Abund
44.99854		5427.94
59.01423		2253.13
61.98869		36067.57
62.00586		3474.85
112.98535		7049.89
137.0241		2482.4
280.98273		3807.01
281.13813		1701.26
805.98496	1	12123.48
806.98799	1	2140.92

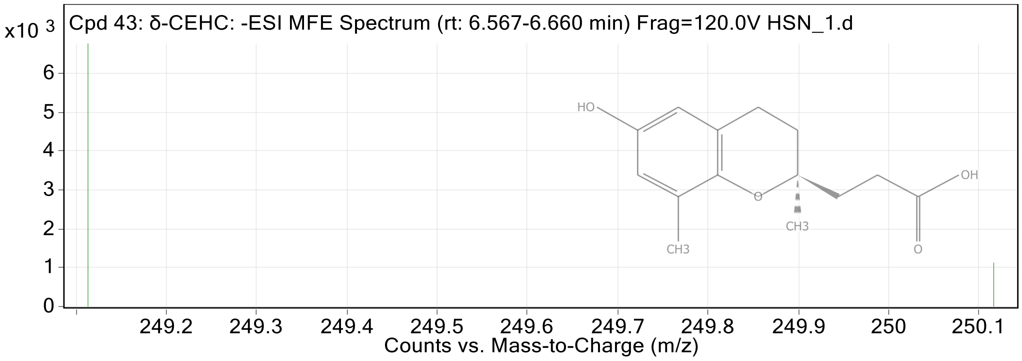

Peak List

m/z	z	Abund	Name	Formula	Ion	Score (DB)	Hits (DB)
249.11321	-1	6759.72	δ-CEHC	C14 H18 O4	(M-H)-	86.07	10
250.11649	-1	1129.81		C14 H18 O4	(M-H)-		

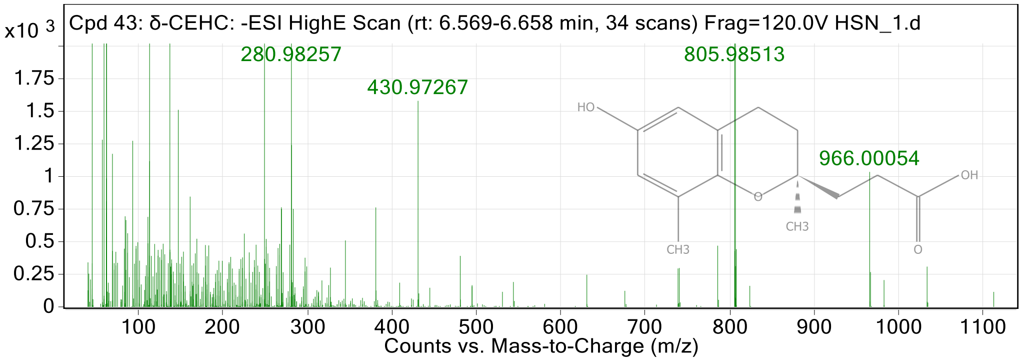

Peak List

m/z	z	Abund
44.99854		5097.07
59.0142		2404.53
61.98866		37379.74
62.0061		3059.13
112.98528		7173.74
137.0242		2096.37
249.11282	1	2020.96
280.98257		2063.67
805.98513	1	12701.18
806.98831	1	2218.81

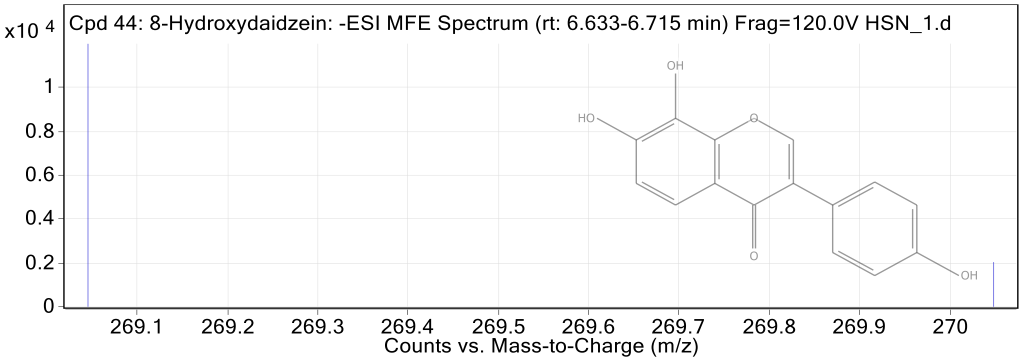

Peak List

m/z	z	Abund	Name	Formula	Ion	Score (DB)	Hits (DB)
269.04536	-1	11995.62	8-Hydroxydaidzein	C15 H10 O5	(M-H)-	85.85	10
270.04861	-1	2037.1		C15 H10 O5	(M-H)-		

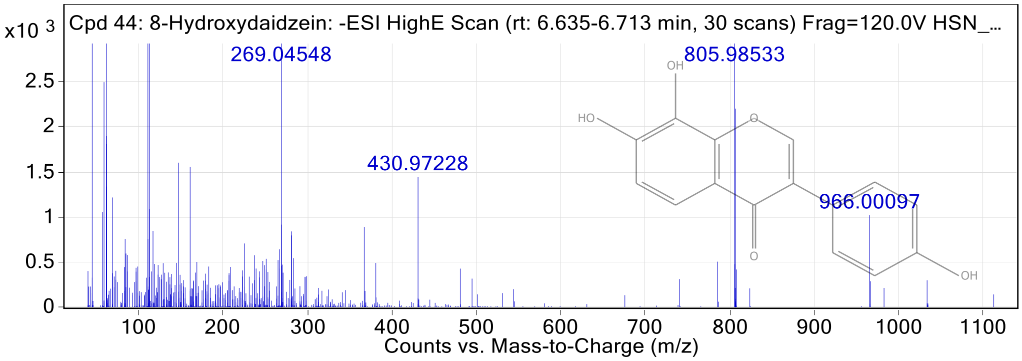

Peak List

m/z	z	Abund
44.99837		4835.97
59.01395		2495.88
61.98847		39503.69

62.00386		1896.54
62.00621		1809.21
111.00849		3540.18
112.98522		7191.97
269.04548	1	2925.51
805.98533	1	12655.67
806.98839	1	2202.47

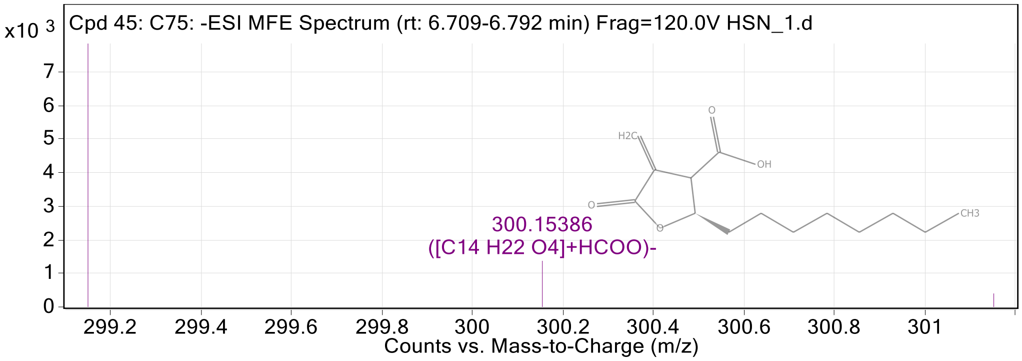

Peak List

m/z	z	Abund	Name	Formula	Ion	Score (DB)	Hits (DB)
299.14963	-1	7851.5	C75	C14 H22 O4	(M+HCOO)-	94.37	2
300.15386	-1	1374.11		C14 H22 O4	(M+HCOO)-		
301.15166	-1	402.2		C14 H22 O4	(M+HCOO)-		

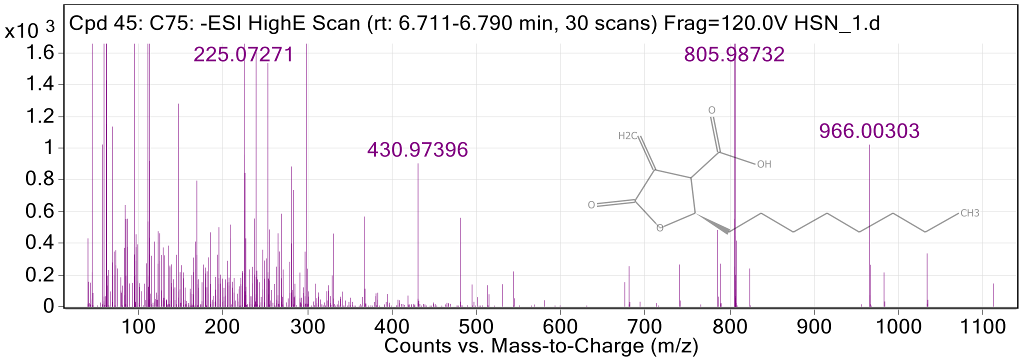

Peak List

m/z	z	Abund
44.99888		4262.66
59.01465		2223.21
61.98907		48642.02
62.00663		4303.74
94.93663		4849.73
111.0097		2020.66
112.98591		7172.16
225.07271	1	9407.15
805.98732	1	13236.57
806.99031	1	2240.72

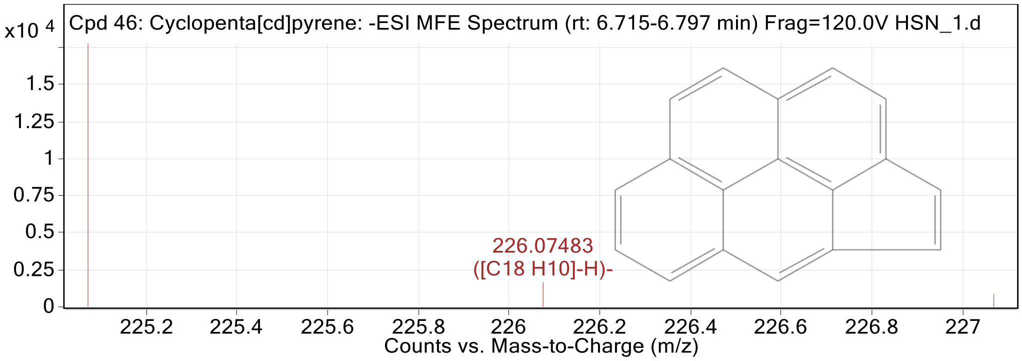

Peak List

m/z	z	Abund	Name	Formula	Ion	Score (DB)	Hits (DB)
225.07174	-1	17779.13	Cyclopenta[cd]pyrene	C18 H10	(M-H)-	70.74	2
226.07483	-1	1667.1		C18 H10	(M-H)-		
227.06825	-1	864.74		C18 H10	(M-H)-		

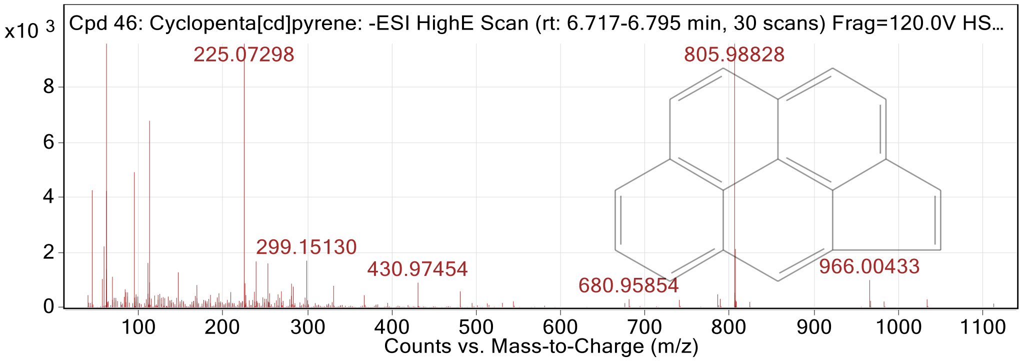

Peak List

m/z	z	Abund
44.99895		4260.08
59.01473		2212.31
61.98916		47310.51
62.00671		4237.26
94.93675		4915.88
112.98602		6794.18
225.07298	1	9607.68
299.1513	1	1691.9
805.98828	1	12498.75
806.99136	1	2118.47

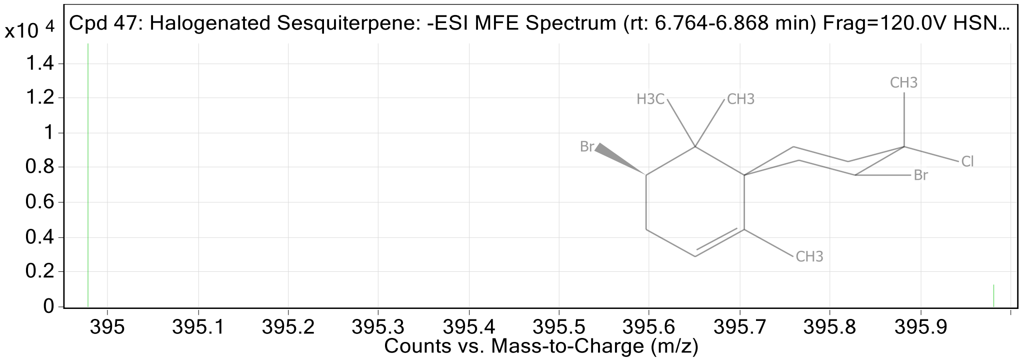

Peak List

m/z	z	Abund	Name	Formula	Ion	Score (DB)	Hits (DB)
394.97792	-1	15134.46	Halogenated Sesquiterpene	C15 H23 Br2 Cl	(M-H)-	59.28	2
395.98131	-1	1280.99		C15 H23 Br2 Cl	(M-H)-		

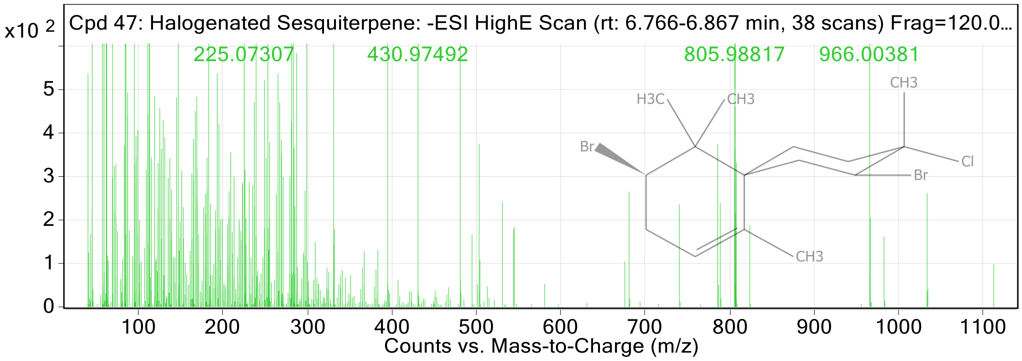

Peak List

m/z	z	Abund
44.99887		4800.39
59.01463		2485.73
61.98913		35676.92
62.00588		3591.01
112.98603		6084.81
225.07307	1	3542.94
283.15607	1	2454.42
330.98088		2835.9
805.98817	1	10869.87
806.99118	1	1890.11

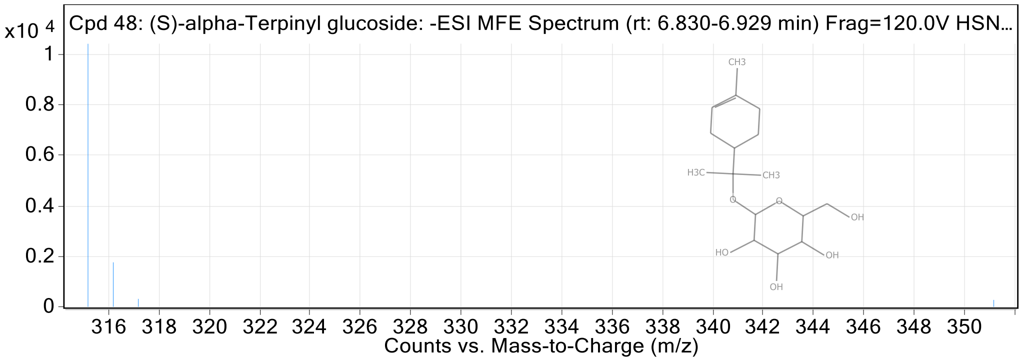

Peak List

m/z	z	Abund	Name	Formula	Ion	Score (DB)	Hits (DB)
315.18099	-1	10437.16	(S)-alpha-Terpinyl glucoside	C16 H28 O6	(M-H)-	97.02	4
316.18473	-1	1763		C16 H28 O6	(M-H)-		
317.18038	-1	313.21		C16 H28 O6	(M-H)-		
351.16149	-1	271.85			(M+Cl)-		

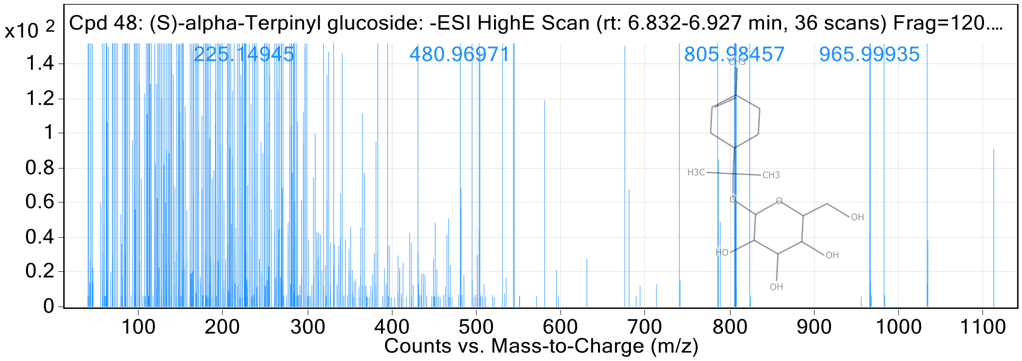

Peak List

m/z	z	Abund
44.99875		5169.37

59.01448		2099.42
61.98889		34731.15
62.00608		2149.51
112.98558		7385.84
225.14945	1	22182.79
226.15283	1	2768.05
283.15492	1	2638.61
805.98457	1	13235.95
806.98726	1	2291.85

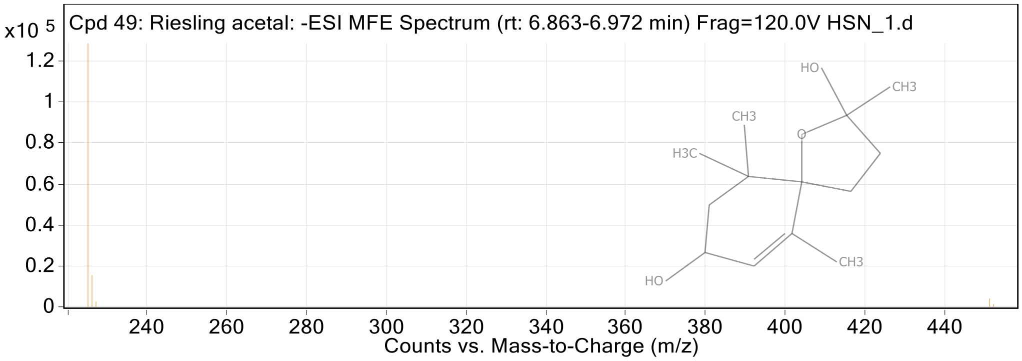

Peak List

m/z	z	Abund	Name	Formula	Ion	Score (DB)	Hits (DB)
225.149	-1	128791.64	Riesling acetal	C13 H22 O3	(M-H)-	95.83	10
226.15321	-1	15430.64		C13 H22 O3	(M-H)-		
227.15551	-1	2583.1		C13 H22 O3	(M-H)-		
451.30485	-1	4071.72			(2M-H)-		
452.30766	-1	1319.69			(2M-H)-		

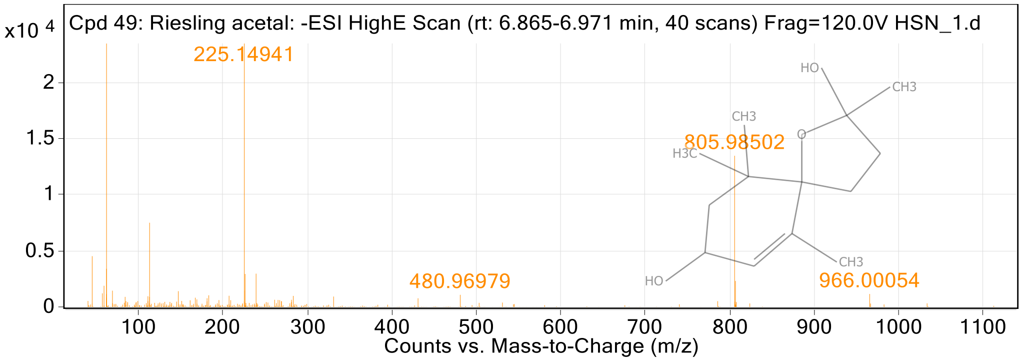

Peak List

m/z	z	Abund
44.99868		4528.97

m/z	z	Abund
59.01425		1886.68
61.98875		33836.52
62.00495		3400.95
112.98549		7513.53
225.14941	1	23514.24
226.1528	1	2941.54
239.12884	1	2967.3
805.98502	1	13489.76
806.98773	1	2314.65

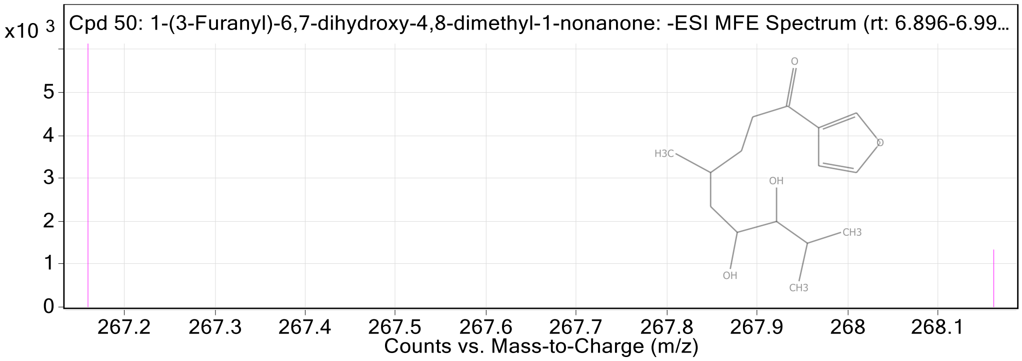

Peak List

m/z	z	Abund	Name	Formula	Ion	Score (DB)	Hits (DB)
267.15979	-1	6135.2	1-(3-Furanyl)-6,7-dihydroxy-4,8-dimethyl-1-nonanone	C15 H24 O4	(M-H)-	75.71	10
268.16177	-1	1338.72		C15 H24 O4	(M-H)-		

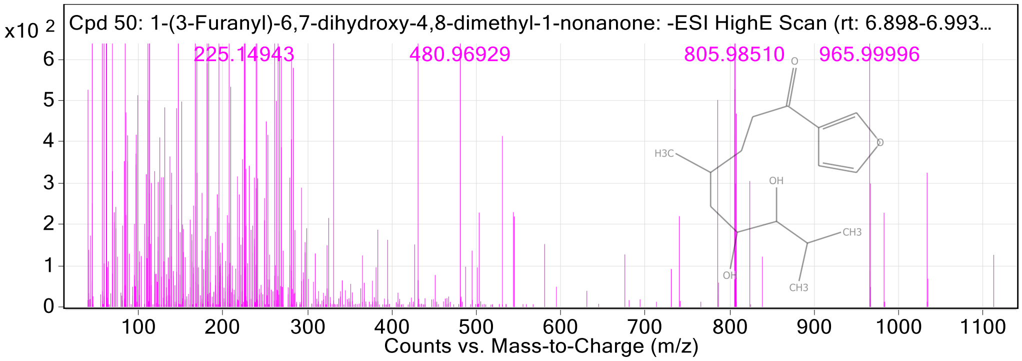

Peak List

m/z	z	Abund
44.99862		3996.48
59.01423		1767.26
61.98871		33606.88
62.00506		3072.3
112.98549		7541.95
225.14943	1	23092.84
226.1529	1	2855.84
239.12866	1	5369.2
805.9851	1	13796.18
806.98798	1	2352.12

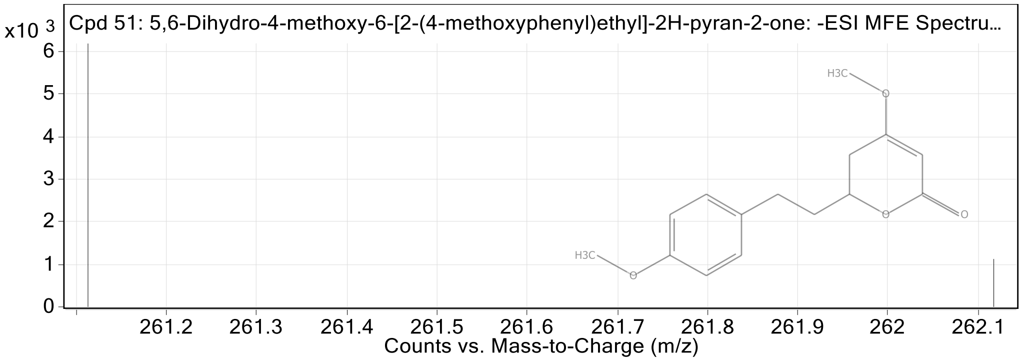

Peak List

m/z	z	Abund	Name	Formula	Ion	Score (DB)	Hits (DB)
261.113	-1	6188.89	5,6-Dihydro-4-methoxy-6-[2-(4-methoxyphenyl)ethyl]-2H-pyran-2-one	C15 H18 O4	(M-H)-	84.34	10
262.11728	-1	1124.93		C15 H18 O4	(M-H)-		

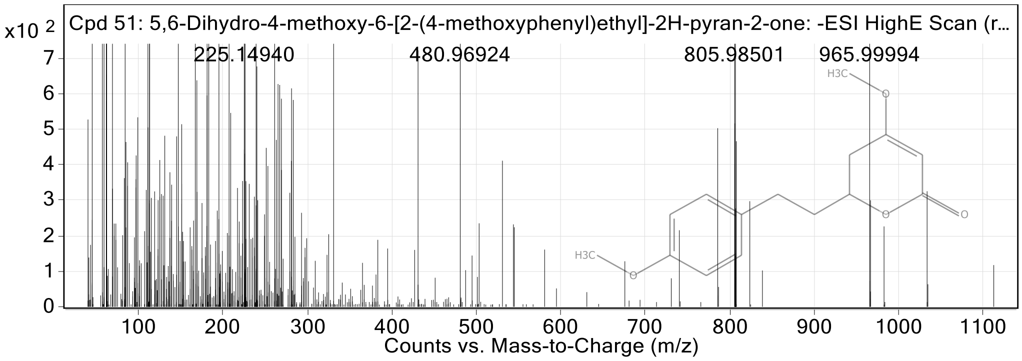

Peak List

m/z	z	Abund
44.99864		3988.17
59.0142		1765.72
61.9887		33397
62.00503		3150.28
112.98546		7512.22
225.1494	1	24324.64
226.15287	1	3007.13
239.12864	1	5291.83
805.98501	1	13722.74
806.98779	1	2338.99

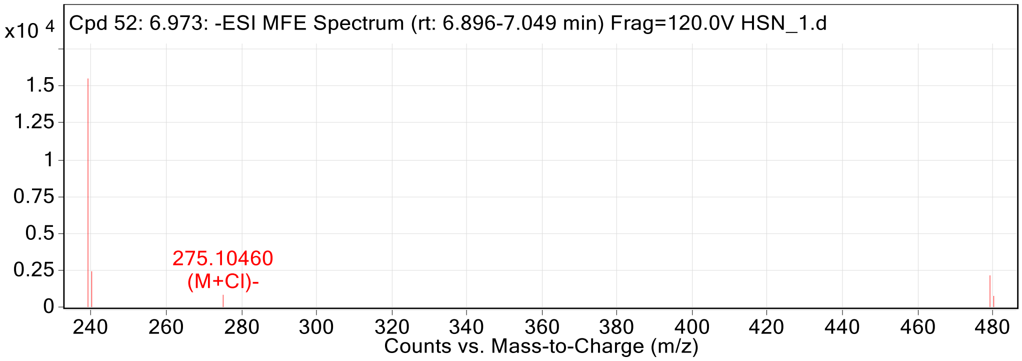

Peak List

m/z	z	Abund	Ion
239.1287	-1	15513.08	(M-H)-

240.13209	-1	2425.5	(M-H)-
275.1046	-1	830.54	(M+Cl)-
479.26351	-1	2149.44	(2M-H)-
480.26915	-1	753.52	(2M-H)-

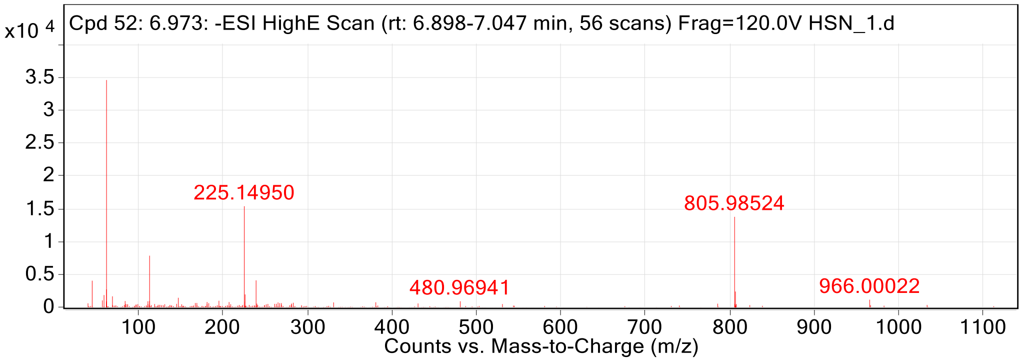

Peak List

m/z	z	Abund
44.9986		4023.45
59.01429		1833.52
61.9887		34608.46
62.00498		2700.42
112.98549		7836.98
225.1495	1	15377.22
226.15296	1	1929.74
239.12869	1	4074.06
805.98524	1	13758.37
806.98822	1	2370.84

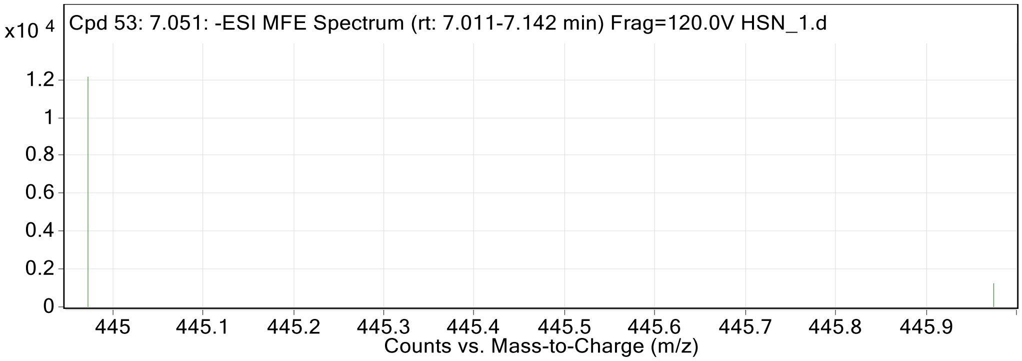

Peak List

m/z	z	Abund	Ion
444.97285	-1	12156.9	(M-H)-
445.97499	-1	1246.45	(M-H)-

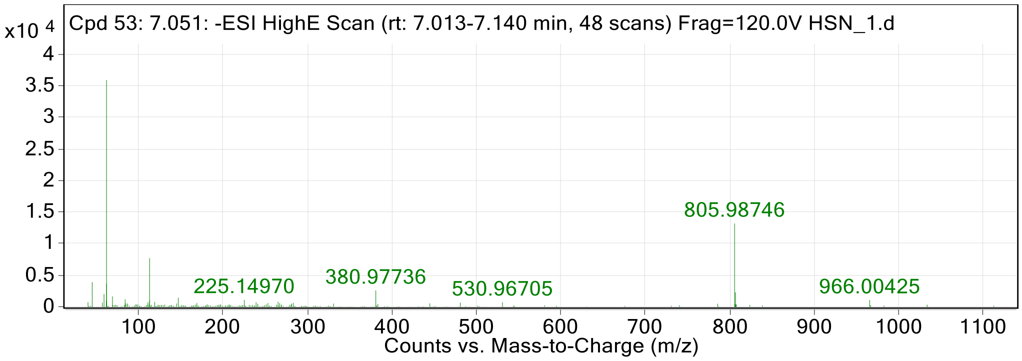

Qualitative Analysis Report

Peak List

m/z	z	Abund
44.99859		3893.05
59.01436		2001.13
61.98879		35928.9
62.00556		3655.58
68.99627		1634.77
112.98568		7698.97
146.9662		1425.37
380.97736		2586.48
805.98746	1	13203.36
806.99101	1	2276.17

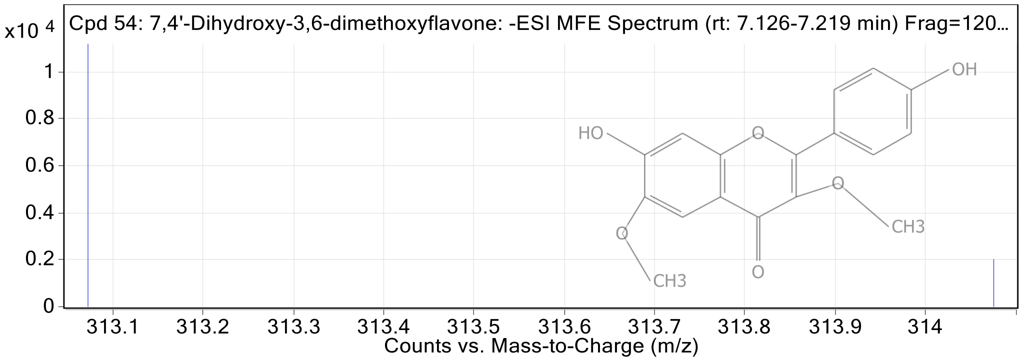

Peak List

m/z	z	Abund	Name	Formula	Ion	Score (DB)	Hits (DB)
313.07229	-1	11184.73	7,4'-Dihydroxy-3,6-dimethoxyflavone	C17 H14 O6	(M-H)-	84.46	10
314.07558	-1	2025.45		C17 H14 O6	(M-H)-		

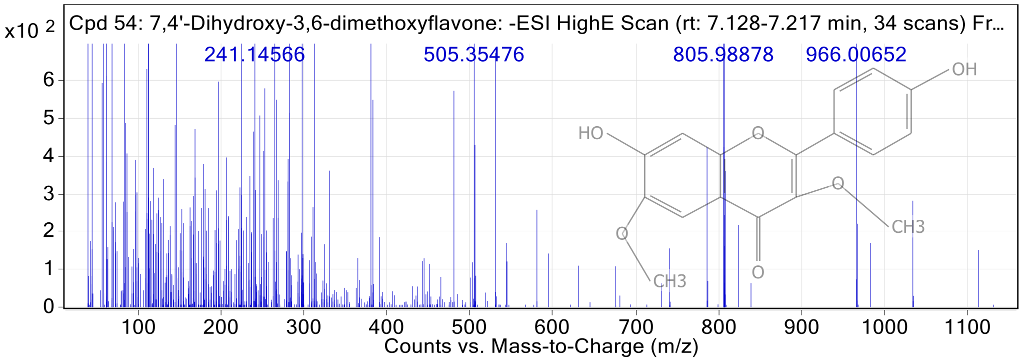

Peak List

m/z	z	Abund
44.99894		4001.81
59.01467		1783.54
61.98915		35025.69
62.00547		3605.05
62.01525		1300.99
112.98625		6678.47
146.9664		1294.08
241.14566	1	2213.83
805.98878	1	12159.25
806.99163	1	2079.67

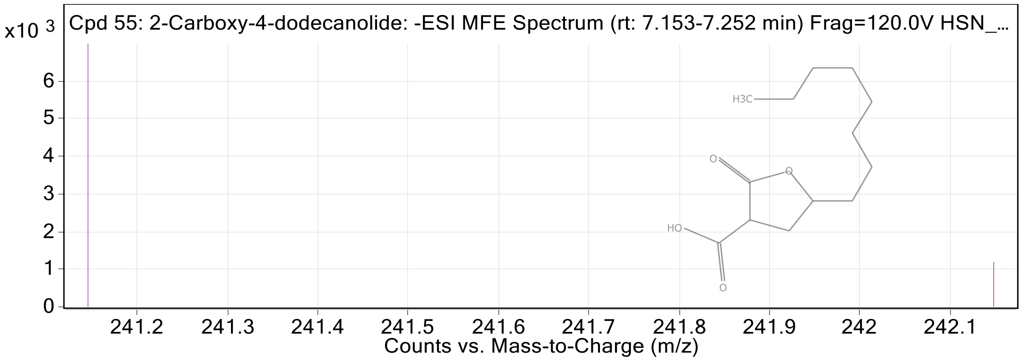

Peak List

m/z	z	Abund	Name	Formula	Ion	Score (DB)	Hits (DB)
241.14551	-1	6999.56	2-Carboxy-4-dodecanolide	C13 H22 O4	(M-H)-	80.46	2
242.14806	-1	1197.12		C13 H22 O4	(M-H)-		

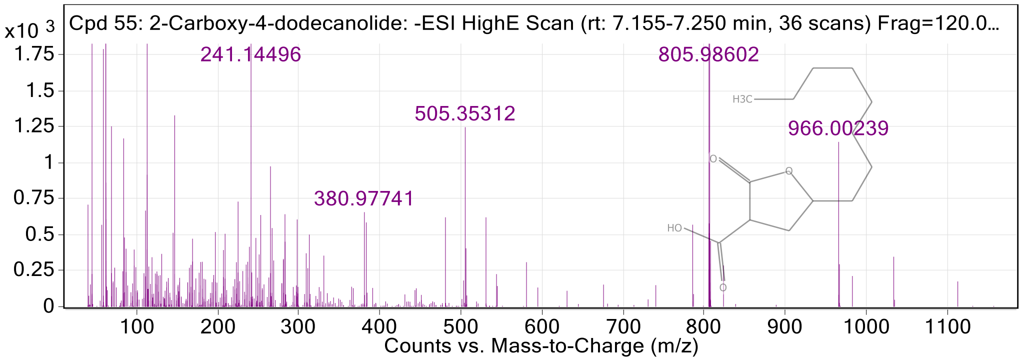

Peak List

m/z	z	Abund
44.99881		4062.33
59.01442		1790.53
61.98895		34131.75
62.0055		3521.18
68.99617		1253.71
112.98582		8195.12
146.96588		1329.86
241.14496	1	1828.37
805.98602	1	15008.64
806.98872	1	2582.61

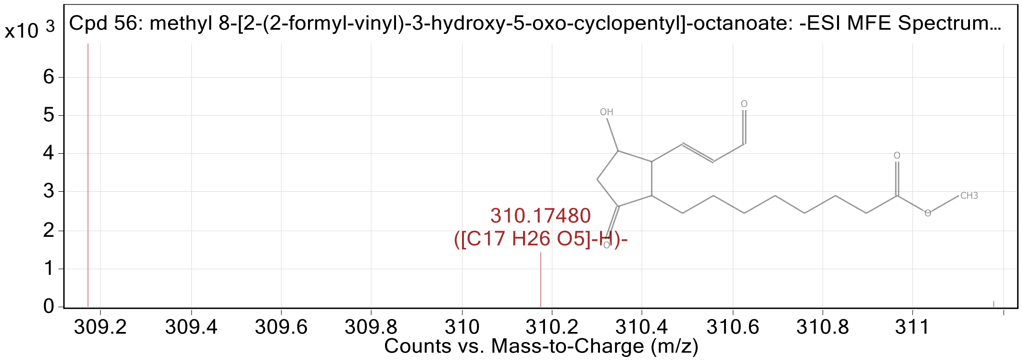

Peak List

m/z	z	Abund	Name	Formula	Ion	Score (DB)	Hits (DB)
309.17122	-1	6871.38	methyl 8-[2-(2-formyl-vinyl)-3-hydroxy-5-oxo-cyclopentyl]-octanoate	C17 H26 O5	(M-H)-	97.79	10

310.1748	-1	1429.69		C17 H26 O5	(M-H)-		
311.17876	-1	154.52		C17 H26 O5	(M-H)-		

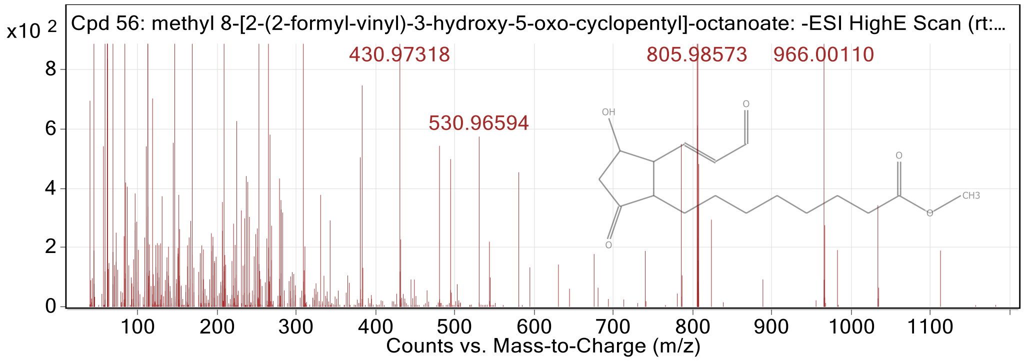

Peak List

m/z	z	Abund
44.9986		3874.51
59.0143		1852.86
61.98881		33418.28
62.00499		3418.61
112.98564		8116.95
146.96599		1560.72
209.15456		1488.99
430.97318	1	2451.92
805.98573	1	15157.46
806.98878	1	2691.89

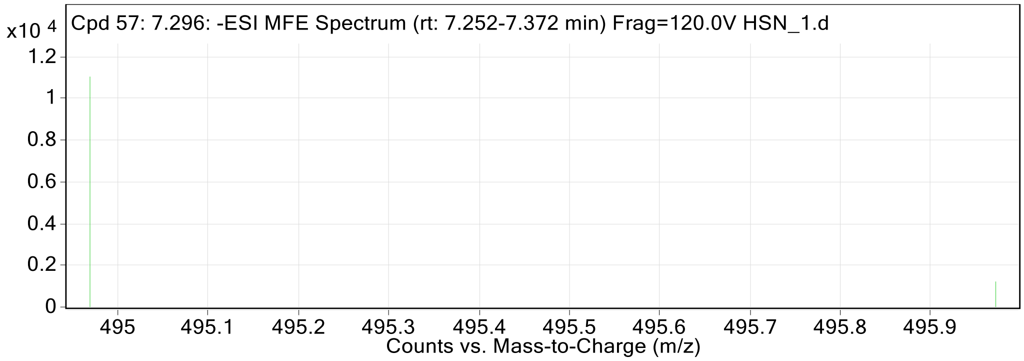

Peak List

m/z	z	Abund	Ion
494.96895	-1	11036.12	(M-H)-
495.97226	-1	1221.42	(M-H)-

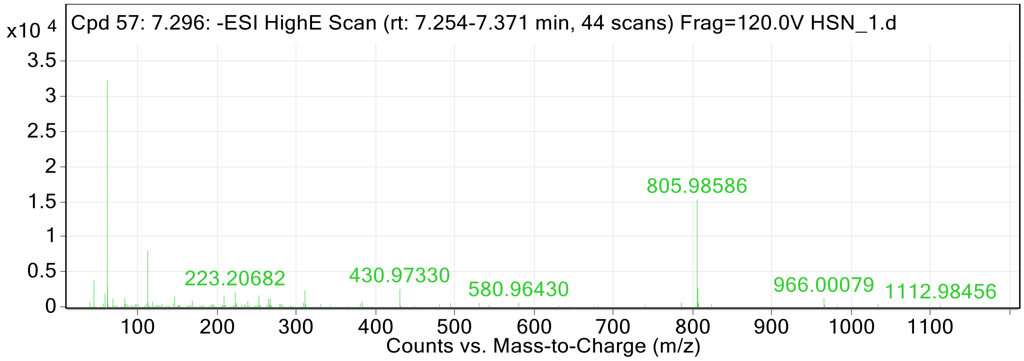

Peak List

m/z	z	Abund
44.99866		3790.73

59.01434		1852.64
61.98882		32410.35
62.00506		2951.48
112.98559		8085.96
223.20682	1	2098.82
311.18624	1	2375.63
430.9733	1	2545.05
805.98586	1	15311.88
806.98884	1	2679.86

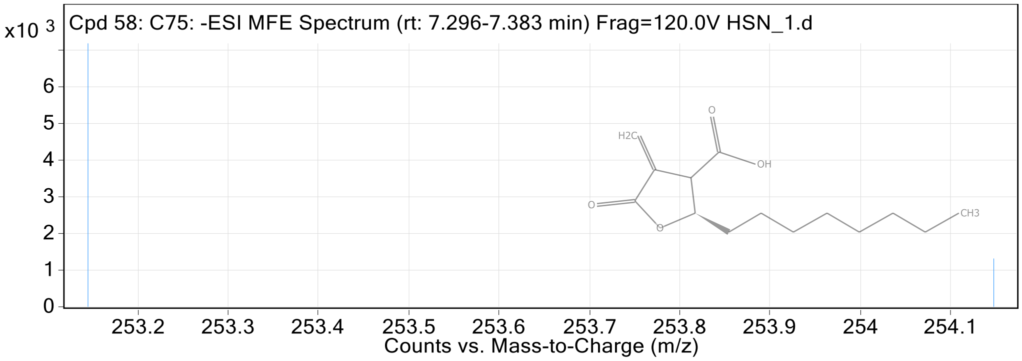

Peak List

m/z	z	Abund	Name	Formula	Ion	Score (DB)	Hits (DB)
253.14486	-1	7203.39	C75	C14 H22 O4	(M-H)-	83.8	10
254.14793	-1	1320.21		C14 H22 O4	(M-H)-		

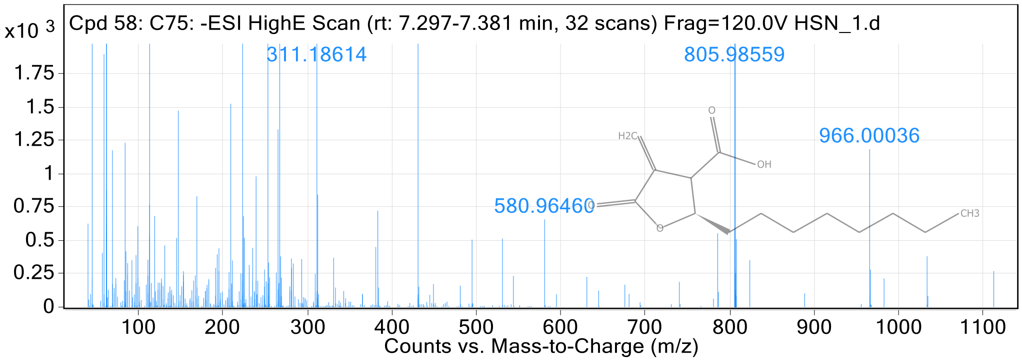

Peak List

m/z	z	Abund
44.99852		3620.88
61.98866		31895.42
62.00523		2541.98
112.98534		8210.82
223.20653	1	4181.41
267.19626	1	2129.25
311.18614	1	4357.77
430.97325		2273.37
805.98559	1	15301.71
806.98812	1	2692.24

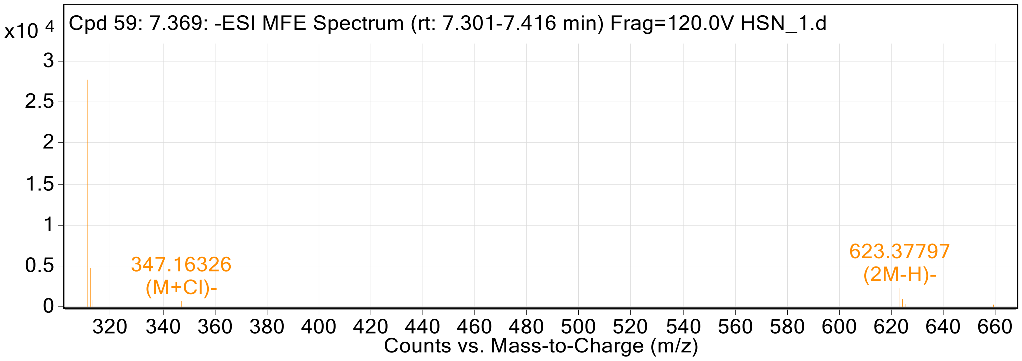

Peak List

m/z	z	Abund	Ion
311.18617	-1	27779.06	(M-H)-
312.18861	-1	4707.37	(M-H)-
313.1907	-1	828.14	(M-H)-
347.16326	-1	714.04	(M+Cl)-
623.37797	-1	2319.98	(2M-H)-
624.37997	-1	928.08	(2M-H)-
625.37235	-1	334.81	(2M-H)-
659.35382	-1	254.57	(2M+Cl)-

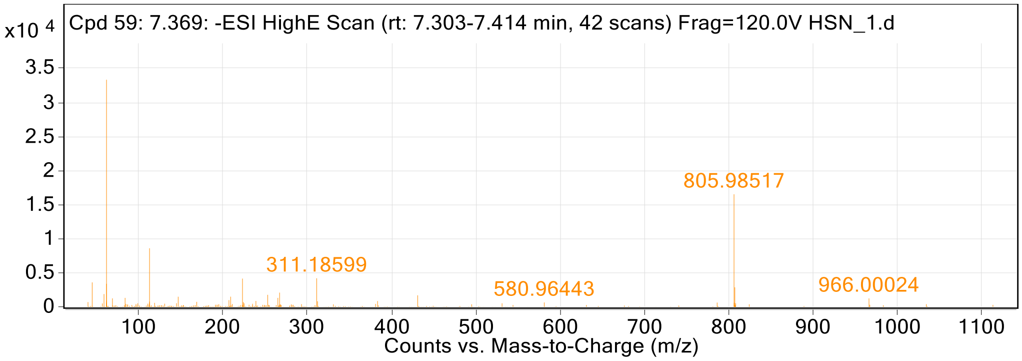

Peak List

m/z	z	Abund
44.99852		3581.58
59.01416		1869.38
61.98865		33334.38
62.0058		3372.4
112.98534		8590.84
223.20644	1	4138.5
267.19616	1	2080.73
311.18599	1	4183.61
805.98517	1	16518.21
806.98806	1	2858.91

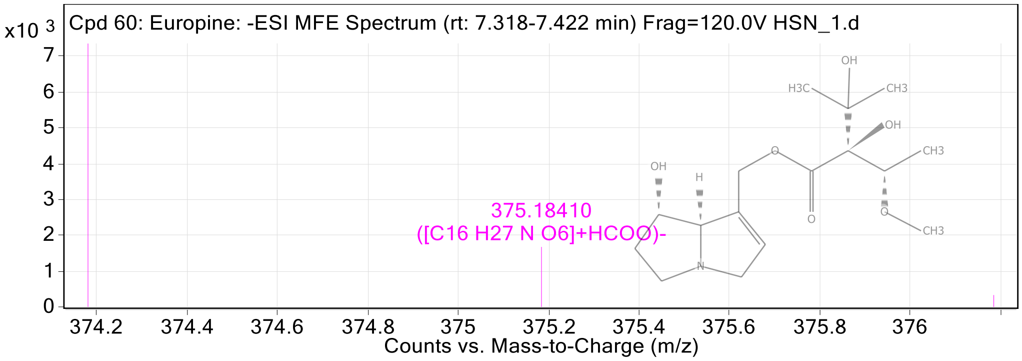

Peak List

m/z	z	Abund	Name	Formula	Ion	Score (DB)	Hits (DB)
374.1811	-1	7359.02	Europine	C16 H27 N O6	(M+HCOO)-	93.29	8
375.1841	-1	1674.5		C16 H27 N O6	(M+HCOO)-		
376.18447	-1	330.25		C16 H27 N O6	(M+HCOO)-		

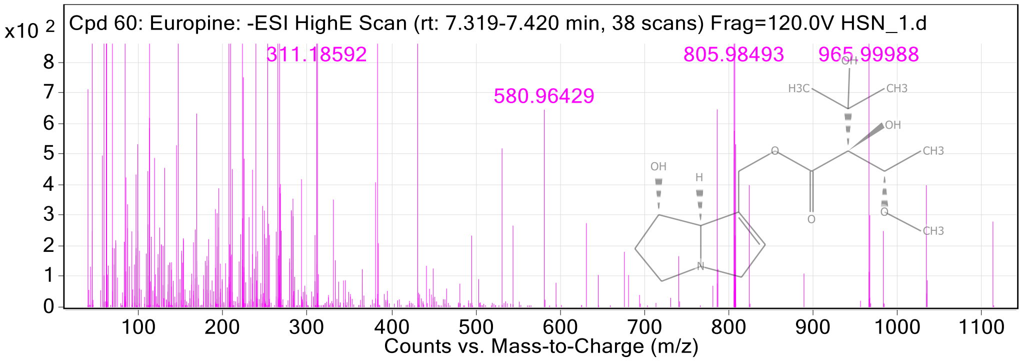

Peak List

m/z	z	Abund
44.99854		3550.6
59.01418		1824.3
61.98864		33448.76
62.00613		2823.43
112.98532		8908.07
223.20639	1	4617.46
267.19608	1	2310.77
311.18592	1	4634.38
805.98493	1	17212.61
806.98788	1	2954.74

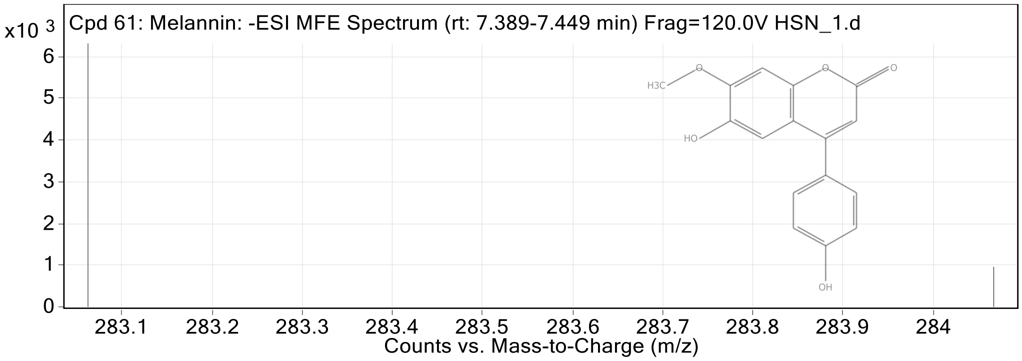

Peak List

m/z	z	Abund	Name	Formula	Ion	Score (DB)	Hits (DB)
283.06232	-1	6318.12	Melannin	C16 H12 O5	(M-H)-	76.68	10
284.06708	-1	960.61		C16 H12 O5	(M-H)-		

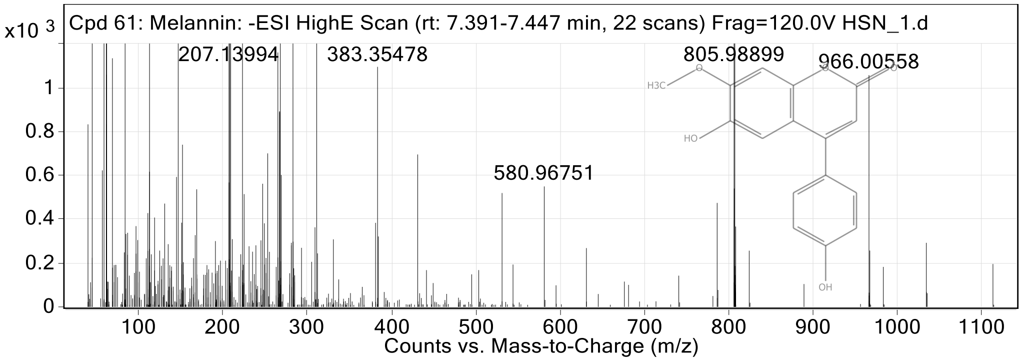

Peak List

m/z	z	Abund
44.99877		3259.44
59.01461		1590.2
61.98901		32814.04
62.00387		1550.35
62.00653		1856.57
112.98607		6953.69
207.13994	1	9608.63
223.20747		1560.37
805.98899	1	13604.62
806.99274	1	2282.59

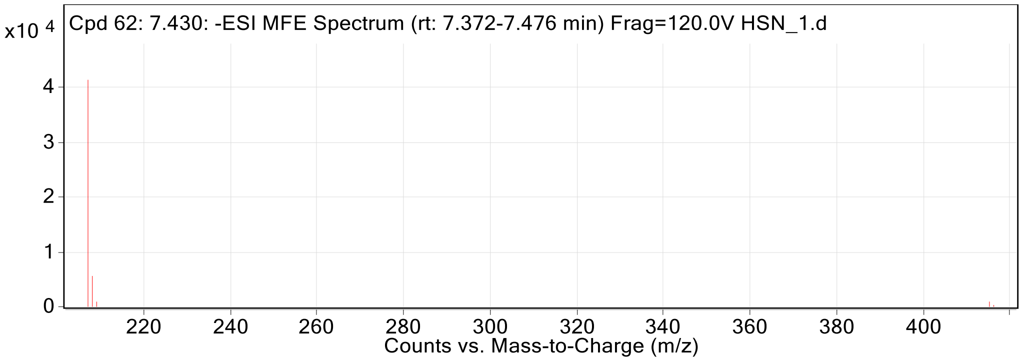

Peak List

m/z	z	Abund	Ion
207.14017	-1	41407.98	(M-H)-
208.14497	-1	5622.46	(M-H)-
209.15059	-1	934.71	(M-H)-
415.29076	-1	942.43	(2M-H)-
416.29202	-1	331.4	(2M-H)-

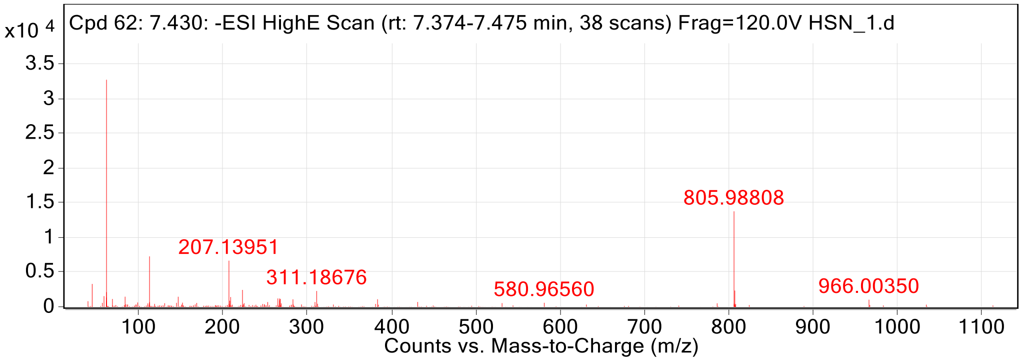

Peak List

m/z	z	Abund

44.99867		3296.66
59.01441		1537.35
61.98886		32744.81
62.00641		2112.6
112.9858		7272.29
207.13951	1	6650.48
223.207	1	2433.99
311.18676	1	2279.95
805.98808	1	13770.88
806.99134	1	2340.5

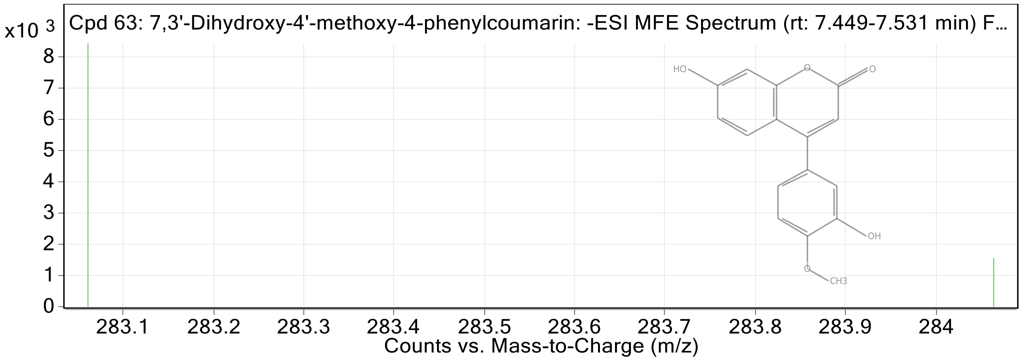

Peak List

m/z	z	Abund	Name	Formula	Ion	Score (DB)	Hits (DB)
283.06114	-1	8447.77	7,3'-Dihydroxy-4'-methoxy-4-phenylcoumarin	C16 H12 O5	(M-H)-	85.35	10
284.0642	-1	1561.33		C16 H12 O5	(M-H)-		

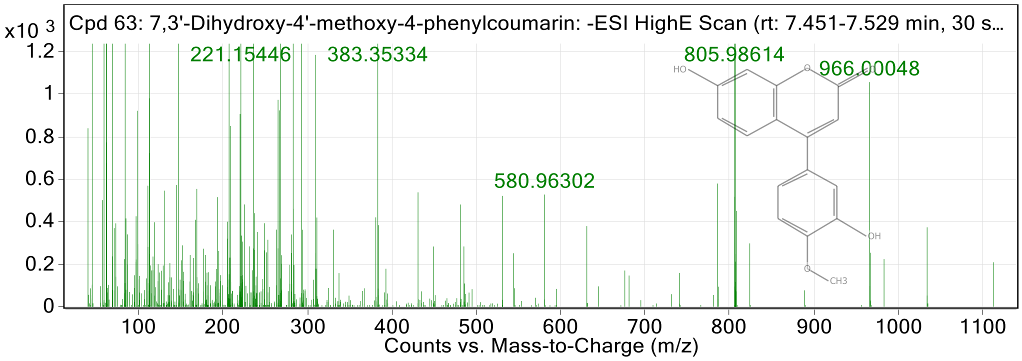

Peak List

m/z	z	Abund
44.99868		3417.8
61.98886		30286.63
62.00529		3064.01
68.99635		2117.64
112.98569		9191.03
221.15446	1	2606.07
236.10568	1	2127.4
293.17641	1	2178.2
805.98614	1	14938.36
806.98951	1	2562.25

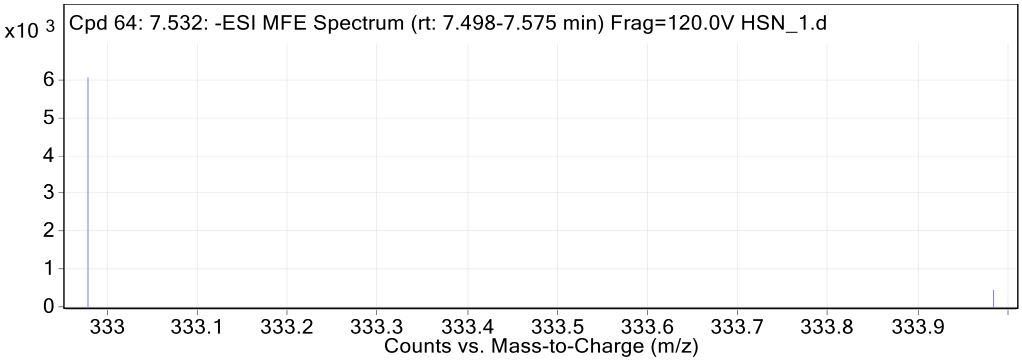

Peak List

m/z	z	Abund	Ion
332.97898	-1	6066.26	(M-H)-
333.9845	-1	449.79	(M-H)-

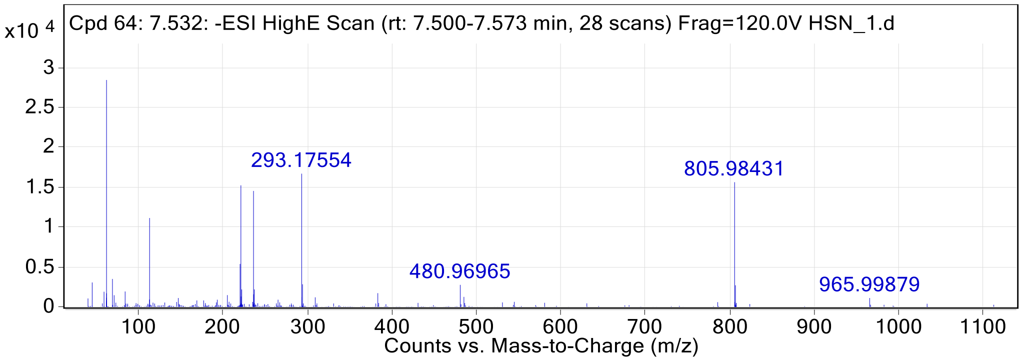

Peak List

m/z	z	Abund
44.99869		3043.98
61.98895		28453.5
68.99643		3485.15
112.98562		11131.94
220.14708		5371.61
221.15441	1	15234.48
236.1052	1	14529.76
293.17554	1	16694.89
294.17909	1	2817.32
805.98431	1	15627.41

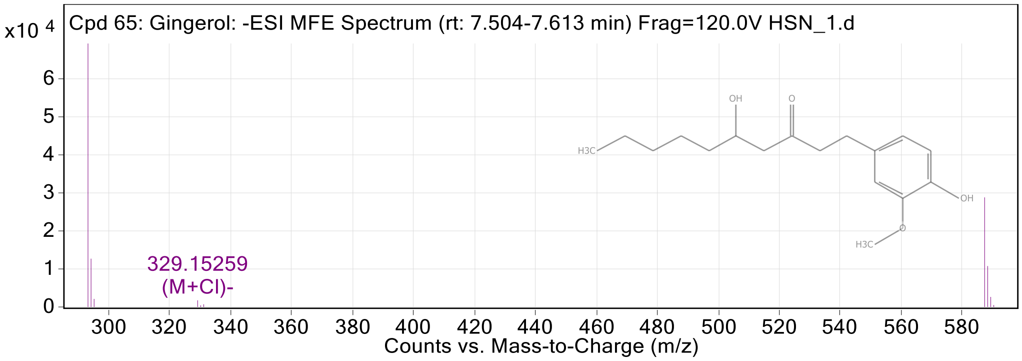

Peak List

m/z	z	Abund	Name	Formula	Ion	Score (DB)	Hits (DB)
293.17533	-1	69236.01	Gingerol	C17 H26 O4	(M-H)-	98.89	7
294.17915	-1	12690.39		C17 H26 O4	(M-H)-		
295.18184	-1	2118.8		C17 H26 O4	(M-H)-		

329.15259	-1	1745.68			(M+Cl)-		
330.15611	-1	424.87			(M+Cl)-		
331.15003	-1	698.1			(M+Cl)-		
587.35786	-1	28796.66			(2M-H)-		
588.36027	-1	10748.08			(2M-H)-		
589.36201	-1	2635.17			(2M-H)-		
590.35995	-1	505.88			(2M-H)-		

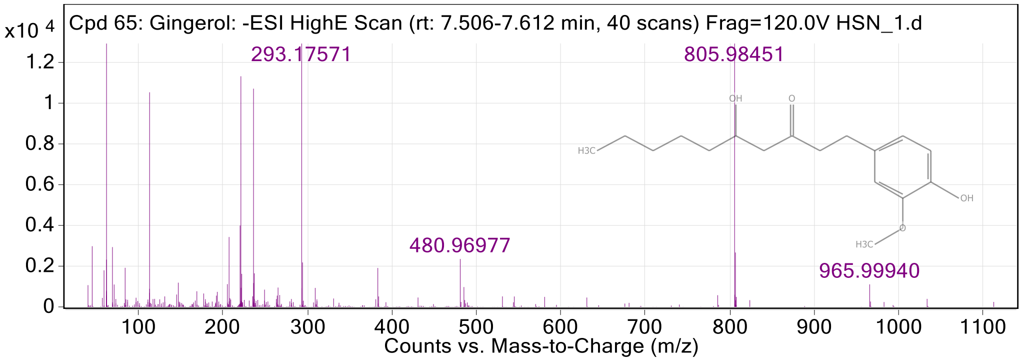

Peak List

m/z	z	Abund
44.99875		2972.98
61.98896		28395.56
68.99641		2928.68
112.98571		10508.15
207.13906	1	3422.44
220.14714		3985.21
221.15448	1	11293.17
236.1053	1	10686.03
293.17571	1	12900.15
805.98451	1	15787.22

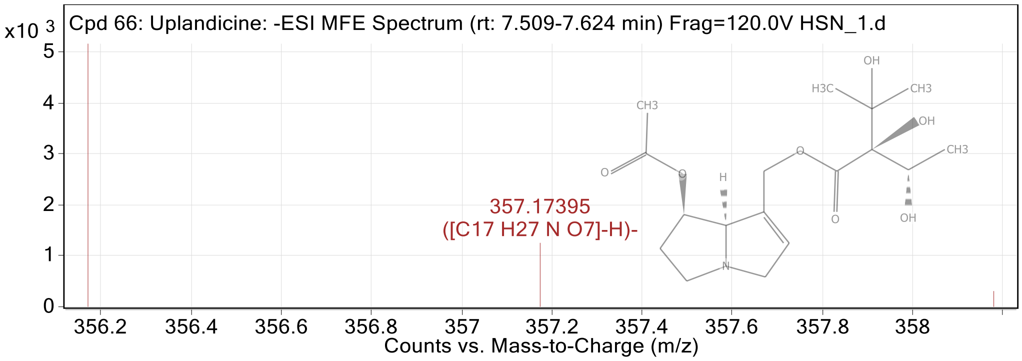

Peak List

m/z	z	Abund	Name	Formula	Ion	Score (DB)	Hits (DB)
356.17118	-1	5167.12	Uplandicine	C17 H27 N O7	(M-H)-	91.94	2
357.17395	-1	1255.8		C17 H27 N O7	(M-H)-		
358.17991	-1	308.94		C17 H27 N O7	(M-H)-		

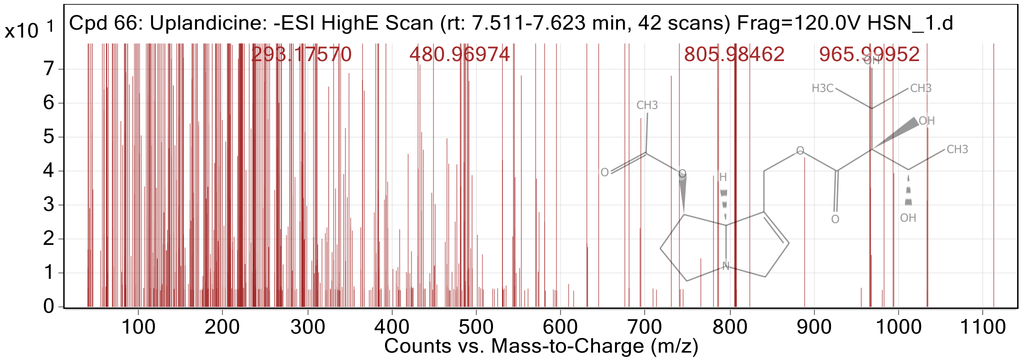

Peak List

m/z	z	Abund
44.99875		2920.71
61.98892		28078.09
68.99634		2844.58
112.98569		10371.42
207.139	1	6337.55
220.1471		3815.08
221.15446	1	10813.6
236.10529	1	10230.82
293.1757	1	12429.24
805.98462	1	15729.35

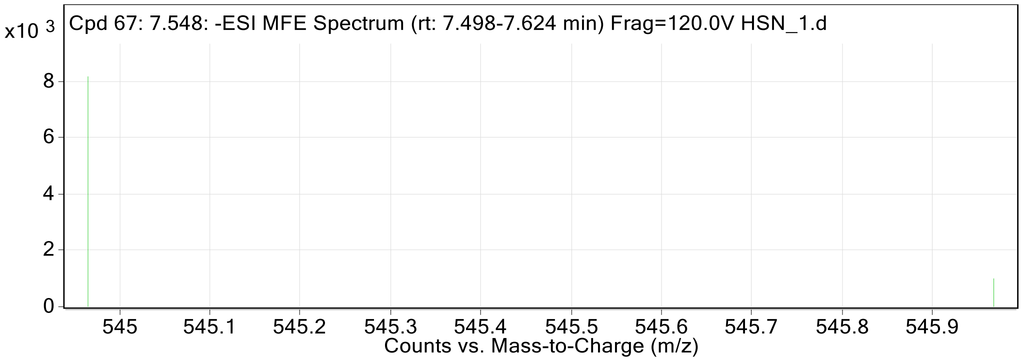

Peak List

m/z	z	Abund	Ion
544.96503	-1	8172.32	(M-H)-
545.96816	-1	1005.5	(M-H)-

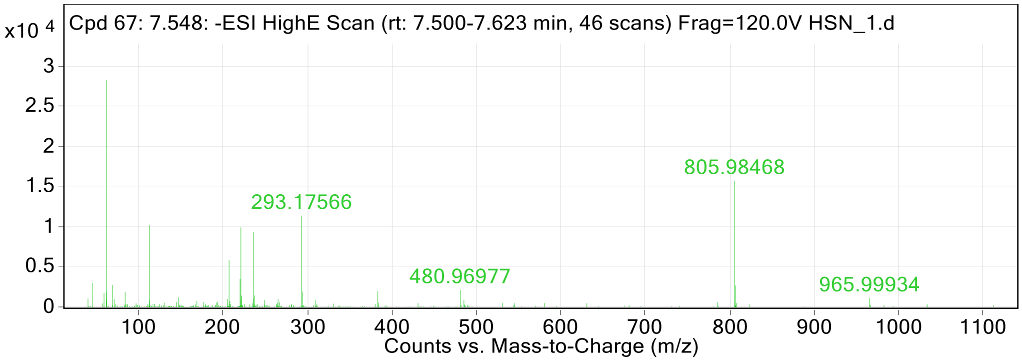

Peak List

m/z	z	Abund
44.99871		3000.13
61.9889		28317.54
68.99632		2737.24

112.98564		10238.71
207.13898	1	5845.35
220.14707		3501.44
221.15442	1	9913.69
236.10526	1	9367.86
293.17566	1	11386.83
805.98468	1	15746.83

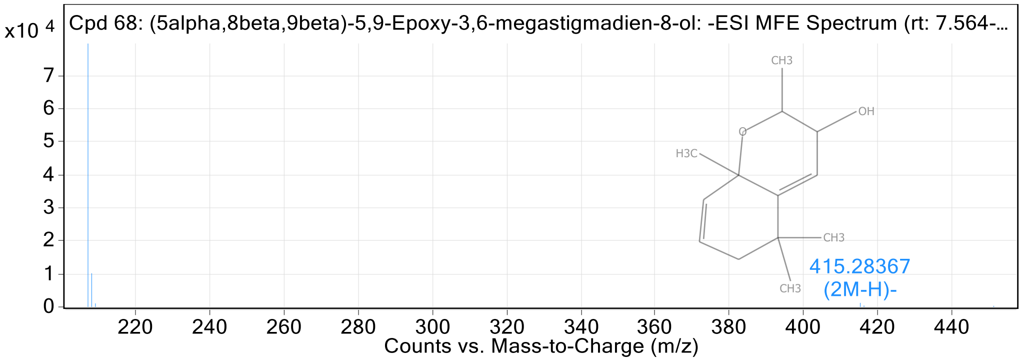

Peak List

m/z	z	Abund	Name	Formula	Ion	Score (DB)	Hits (DB)
207.13926	-1	79761.27	(5alpha,8beta,9beta)-5,9-Epoxy-3,6-megastigmadien-8-ol	C13 H20 O2	(M-H)-	98.83	10
208.14225	-1	10185.75		C13 H20 O2	(M-H)-		
209.14513	-1	1075.11		C13 H20 O2	(M-H)-		
415.28367	-1	1237.95			(2M-H)-		
416.28905	-1	423.41			(2M-H)-		
451.26607	-1	336.37			(2M+Cl)-		

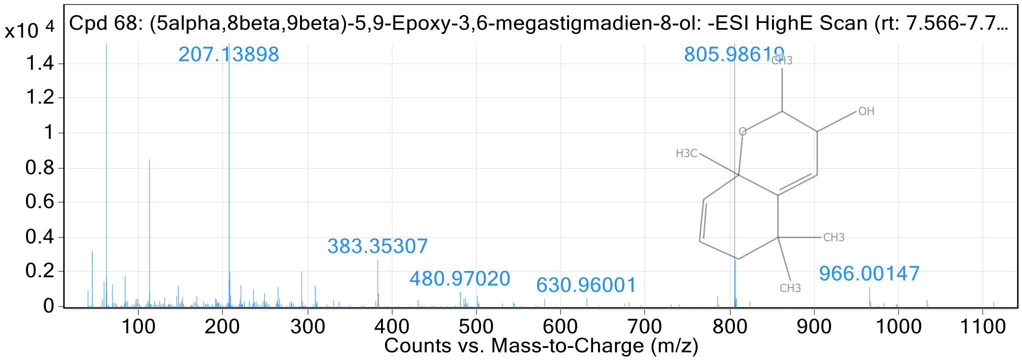

Peak List

m/z	z	Abund
44.99875		3191.14
61.98883		25522.24
84.00939		1758.66
112.98566		8478.54
207.13898	1	15152.63
208.14263	1	1968.19
293.17625	1	2023.84
383.35307	1	2699.44
805.98619	1	15893.5
806.98933	1	2708.09

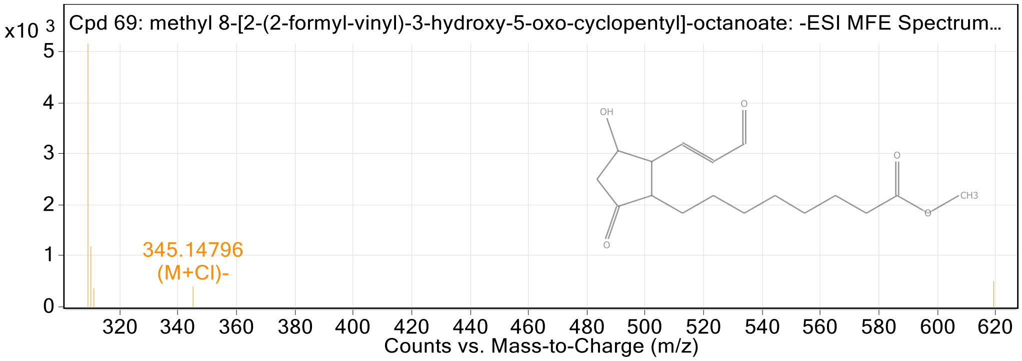

Peak List

m/z	z	Abund	Name	Formula	Ion	Score (DB)	Hits (DB)
309.17082	-1	5159.73	methyl 8-[2-(2-formyl-vinyl)-3-hydroxy-5-oxo-cyclopentyl]-octanoate	C17 H26 O5	(M-H)-	88.37	4
310.17404	-1	1189.15		C17 H26 O5	(M-H)-		
311.18108	-1	364.91		C17 H26 O5	(M-H)-		
345.14796	-1	404.04			(M+Cl)-		
619.34421	-1	505.94			(2M-H)-		

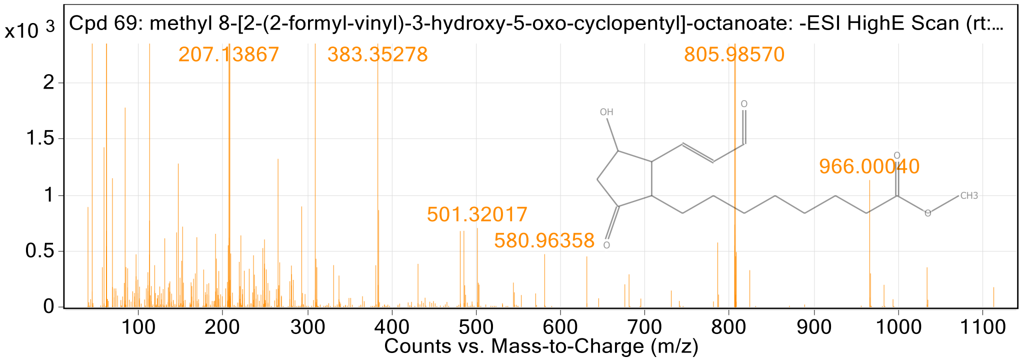

Peak List

m/z	z	Abund
44.99868		3249.39
61.98873		25045.19
62.00519		2649.46
112.98549		8404.91
207.13867	1	23430.59
207.17104	1	2484.65
208.14239	1	3035.5
383.35278	1	3053.56
805.9857	1	15698.83
806.98898	1	2636.26

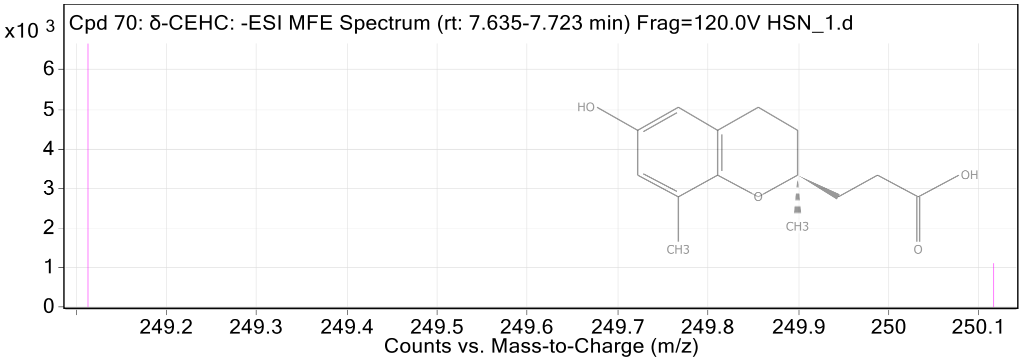

Peak List

m/z	z	Abund	Name	Formula	Ion	Score (DB)	Hits (DB)
249.11317	-1	6668.41	δ-CEHC	C14 H18 O4	(M-H)-	86.12	10
250.11636	-1	1103.56		C14 H18 O4	(M-H)-		

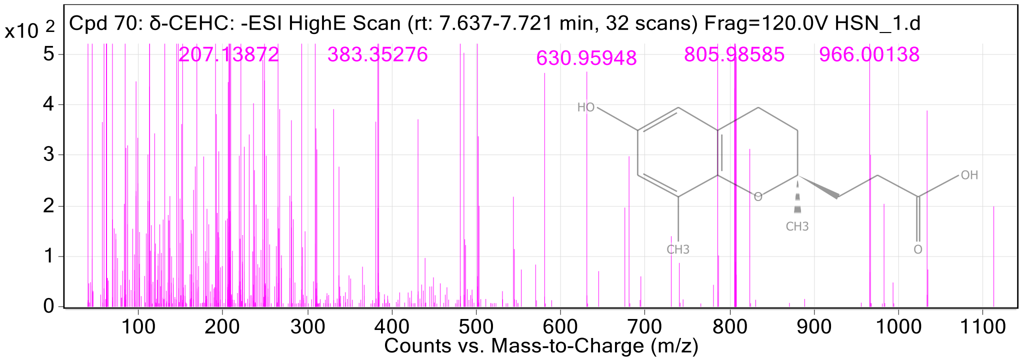

Peak List

m/z	z	Abund
44.99873		3138.81
61.98878		25464.32
62.005		2730.09
112.98556		8433.36
207.13872	1	19055.95
207.17074		2018.4
208.14246	1	2484.94
383.35276	1	2876.24
805.98585	1	15723.92
806.98886	1	2656.55

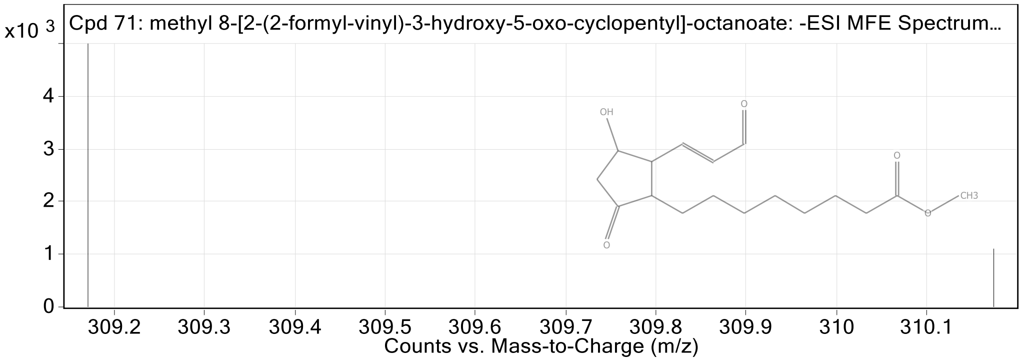

Peak List

m/z	z	Abund	Name	Formula	Ion	Score (DB)	Hits (DB)
309.17093	-1	5003.46	methyl 8-[2-(2-formyl-vinyl)-3-hydroxy-5-oxo-cyclopentyl]-octanoate	C17 H26 O5	(M-H)-	82.52	10

310.17423	-1	1106.95		C17 H26 O5	(M-H)-		

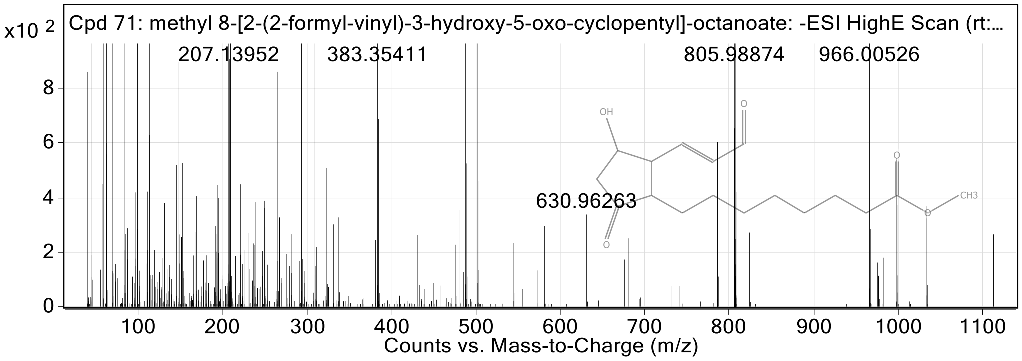

Peak List

m/z	z	Abund
44.99885		3322.64
61.98895		22017.94
62.00514		2113.82
84.00946		1545.5
112.98593		7651.65
207.13952	1	6664.88
383.35411	1	2565.05
487.34465	1	1546.13
805.98874	1	15060.61
806.99223	1	2566.57

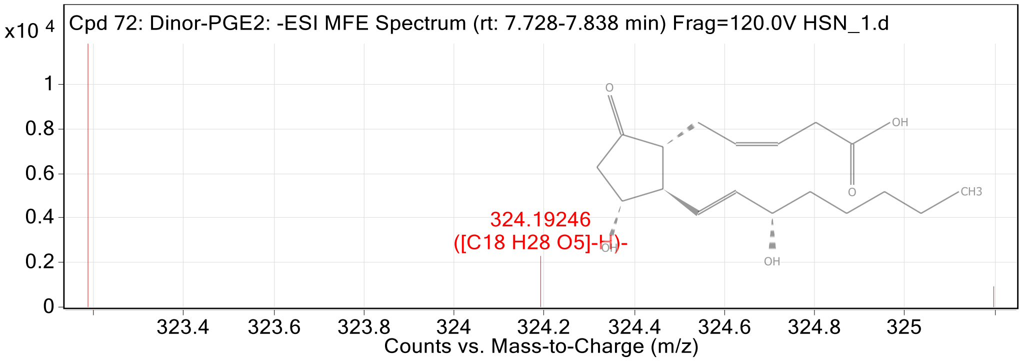

Peak List

m/z	z	Abund	Name	Formula	Ion	Score (DB)	Hits (DB)
323.18786	-1	11818.22	Dinor-PGE2	C18 H28 O5	(M-H)-	76.2	5
324.19246	-1	2292.94		C18 H28 O5	(M-H)-		
325.19798	-1	927.78		C18 H28 O5	(M-H)-		

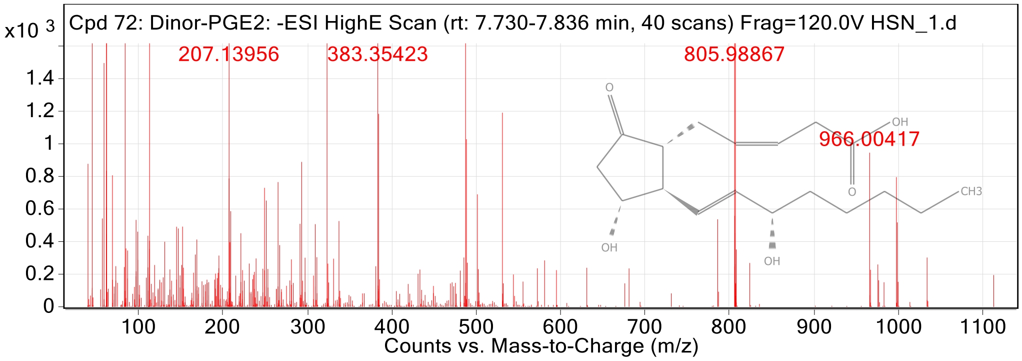

Peak List

m/z	z	Abund
44.9987		3510.56

61.98889		27097.07
62.00539		2475.31
84.00952		1761.74
112.98581		6484.98
207.13956	1	2825.56
383.35423	1	4812.31
487.34414	1	3000.43
805.98867	1	13007.3
806.99257	1	2221.52

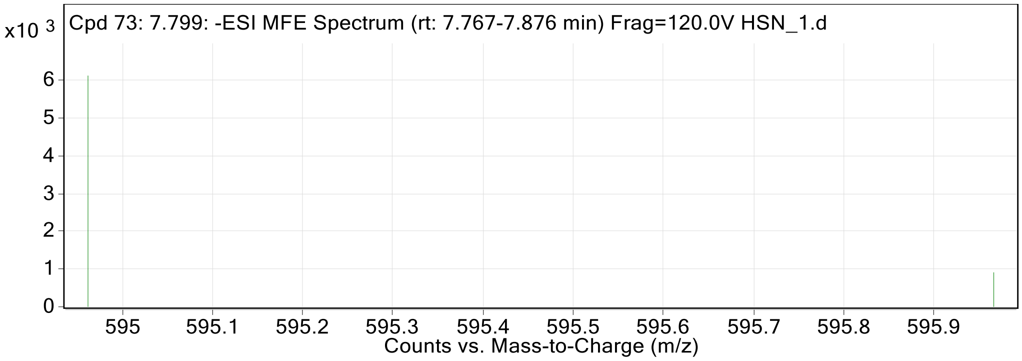

Peak List

m/z	z	Abund	Ion
594.96209	-1	6131.88	(M-H)-
595.96621	-1	913.34	(M-H)-

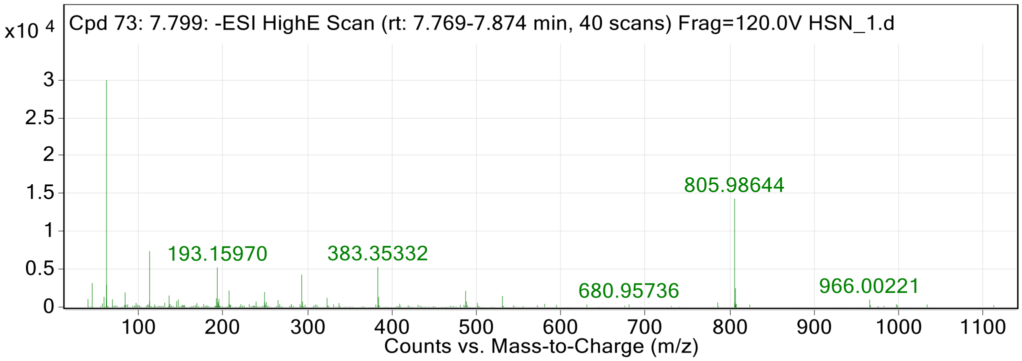

Peak List

m/z	z	Abund
44.99867		3186.42
61.98883		29971.57
62.0051		2945.85
112.98556		7385.5
193.1597	1	5232.39
207.1391	1	2178.92
293.17579	1	4285.37
383.35332	1	5280.14
805.98644	1	14306.72
806.98978	1	2489.01

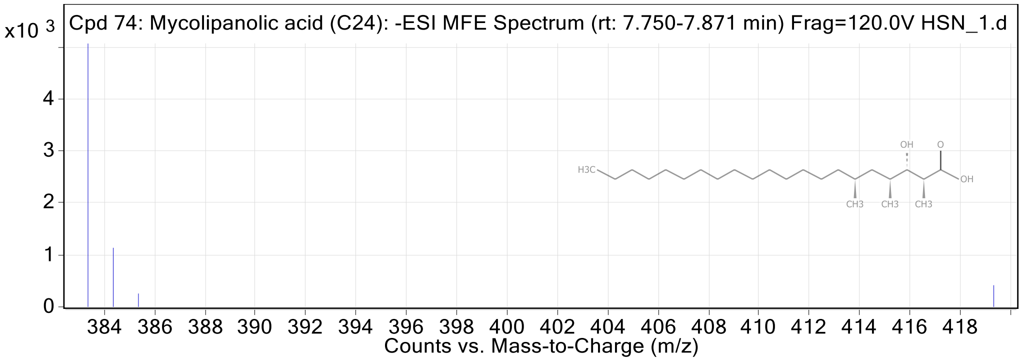

Peak List

m/z	z	Abund	Name	Formula	Ion	Score (DB)	Hits (DB)
383.3529	-1	5070.41	Mycolipanolic acid (C24)	C24 H48 O3	(M-H)-	94.9	7
384.35632	-1	1137.15		C24 H48 O3	(M-H)-		
385.35651	-1	256.85		C24 H48 O3	(M-H)-		
419.32932	-1	416.89			(M+Cl)-		

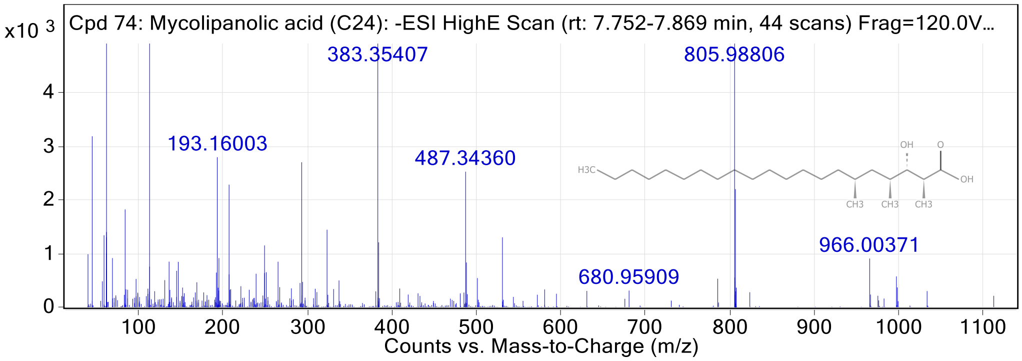

Peak List

m/z	z	Abund
44.99877		3190.39
61.98893		27681.28
112.98578		6459.99
193.16003	1	2799.37
207.13953	1	2286.47
293.17633	1	2704.17
383.35407	1	4922.49
487.3436	1	2528.63
805.98806	1	12613.4
806.99146	1	2202.5

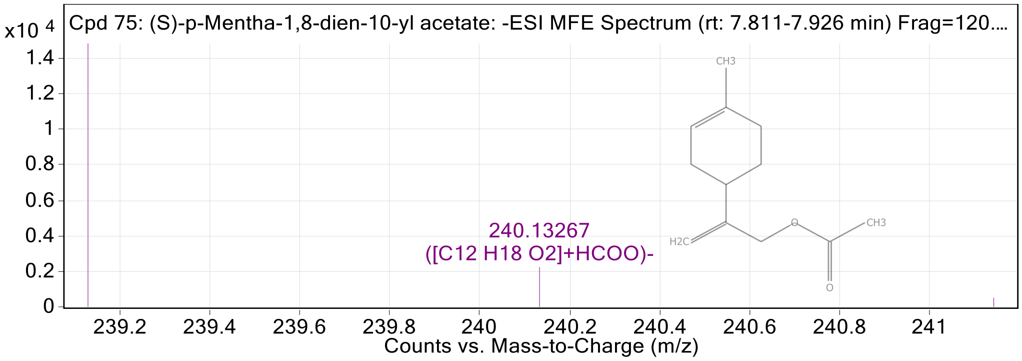

Peak List

m/z	z	Abund	Name	Formula	Ion	Score (DB)	Hits (DB)

239.12915	-1	14831.12	(S)-p-Mentha-1,8-dien-10-yl acetate	C12 H18 O2	(M+HCOO)-	93.91	10
240.13267	-1	2244.22		C12 H18 O2	(M+HCOO)-		
241.1422	-1	507.36		C12 H18 O2	(M+HCOO)-		

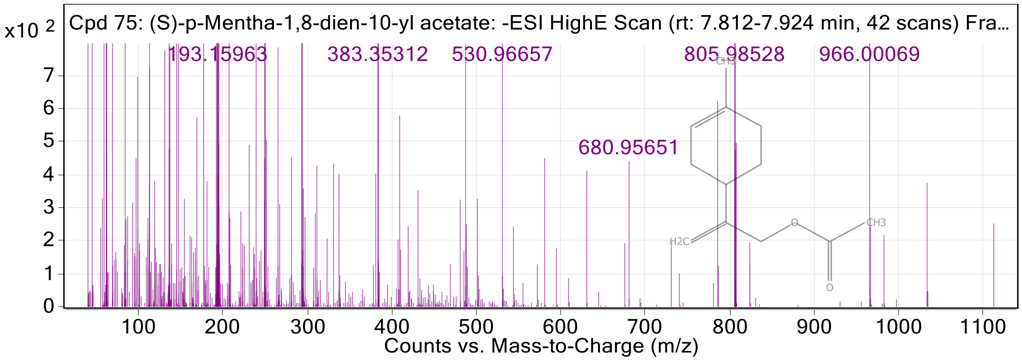

Peak List

m/z	z	Abund
61.98884		31161.87
62.00515		3117.03
112.98551		8246.34
136.0893	1	5214.16
192.11558	1	4234.72
193.15963	1	20332.51
249.18585	1	7227.59
293.17553	1	15491.01
383.35312	1	4400.58
805.98528	1	15560.28

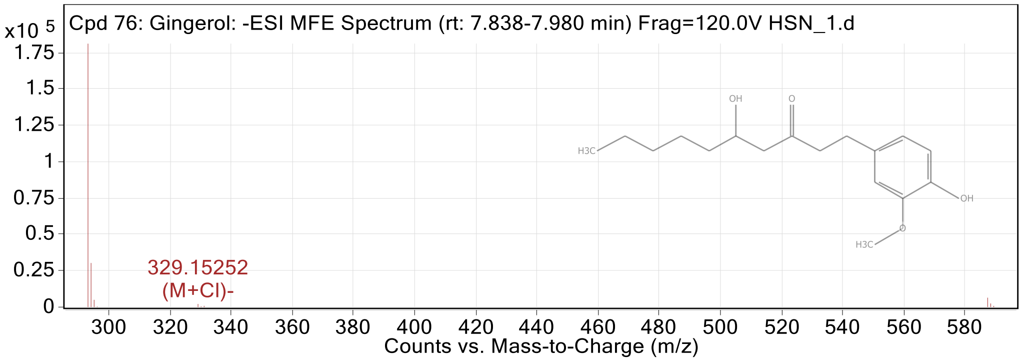

Peak List

m/z	z	Abund	Name	Formula	Ion	Score (DB)	Hits (DB)
293.17544	-1	181080.03	Gingerol	C17 H26 O4	(M-H)-	97.94	7
294.17941	-1	30305.04		C17 H26 O4	(M-H)-		
295.18038	-1	4986.81		C17 H26 O4	(M-H)-		
296.18366	-1	351.9		C17 H26 O4	(M-H)-		
329.15252	-1	2140.01			(M+Cl)-		
330.15731	-1	500.26			(M+Cl)-		
331.14897	-1	845.49			(M+Cl)-		
587.35763	-1	6456.03			(2M-H)-		
588.36016	-1	2489.27			(2M-H)-		
589.35332	-1	824.45			(2M-H)-		

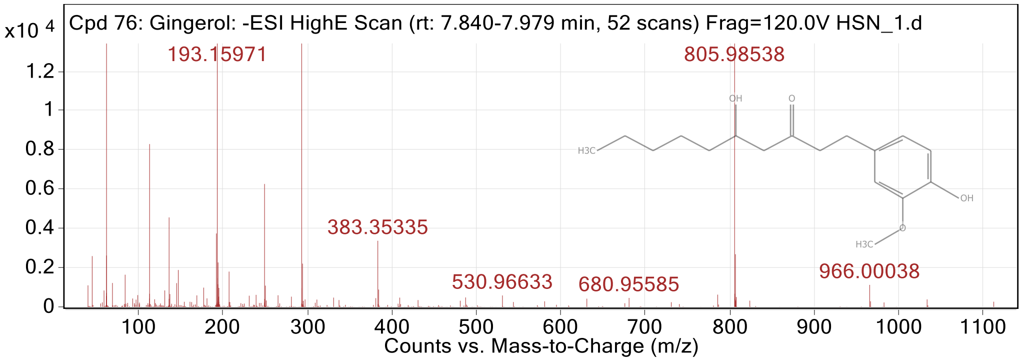

Peak List

m/z	z	Abund
61.98882		25209.02
112.98557		8295.52
136.08935	1	4558.38
192.11564	1	3741.25
193.15971	1	17607
249.18595	1	6262.59
293.17566	1	13424.47
383.35335	1	3368.79
805.98538	1	15833.91
806.98865	1	2681.84

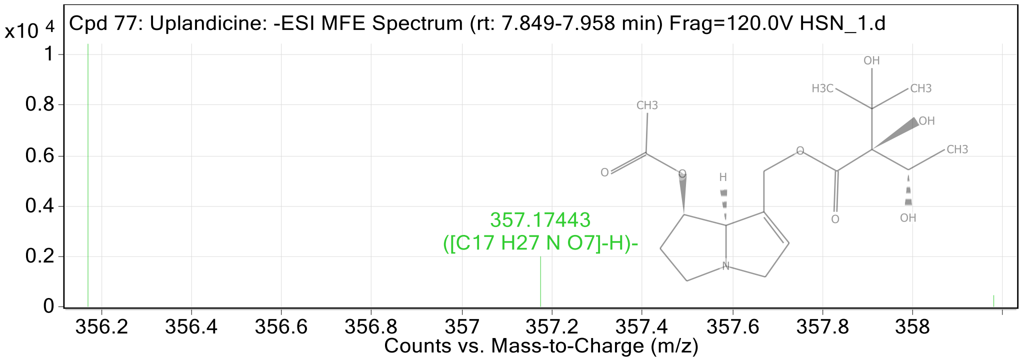

Peak List

m/z	z	Abund	Name	Formula	Ion	Score (DB)	Hits (DB)
356.17059	-1	10450.21	Uplandicine	C17 H27 N O7	(M-H)-	96.59	2
357.17443	-1	2002.39		C17 H27 N O7	(M-H)-		
358.17962	-1	460.29		C17 H27 N O7	(M-H)-		

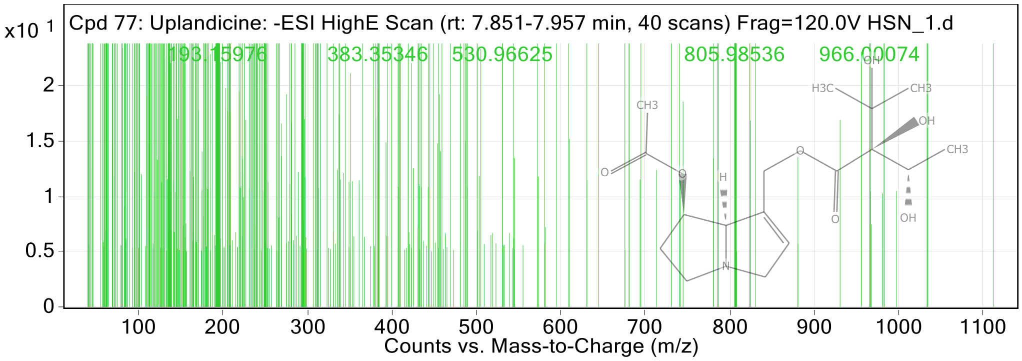

Peak List

m/z	z	Abund
61.98887		26152.61
112.98561		8180.33

136.0894	1	5779.92
192.11571	1	4718.79
193.15976	1	22393.56
194.16316	1	2878.92
249.18601	1	7926.91
293.17573	1	17003.93
383.35346	1	3266.19
805.98536	1	15752.38

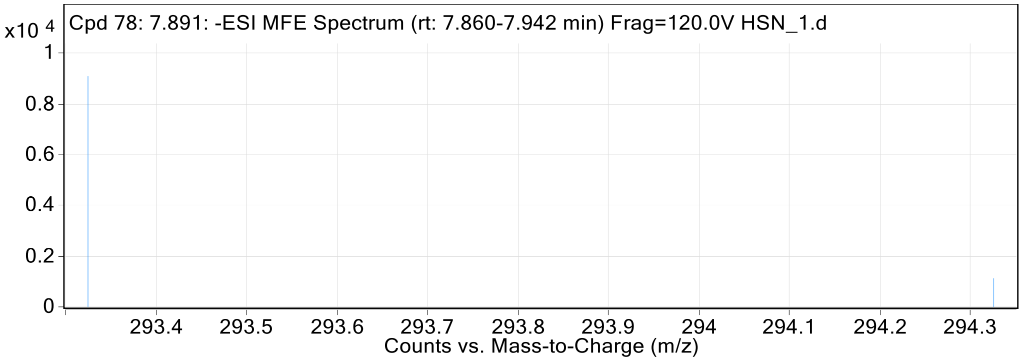

Peak List

m/z	z	Abund	Ion
293.32462	-1	9102.12	(M-H)-
294.32617	-1	1127.03	(M-H)-

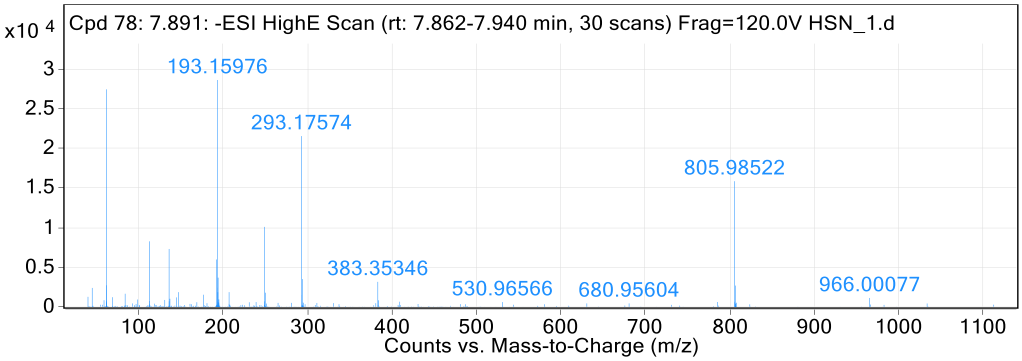

Peak List

m/z	z	Abund
61.98891		27398.05
112.98565		8242.21
136.08943	1	7278.27
192.11574	1	5953.96
193.15976	1	28577.04
194.16317	1	3662.67
249.18601	1	10060.66
293.17574	1	21509.6
294.17936	1	3482.83
805.98522	1	15820.27

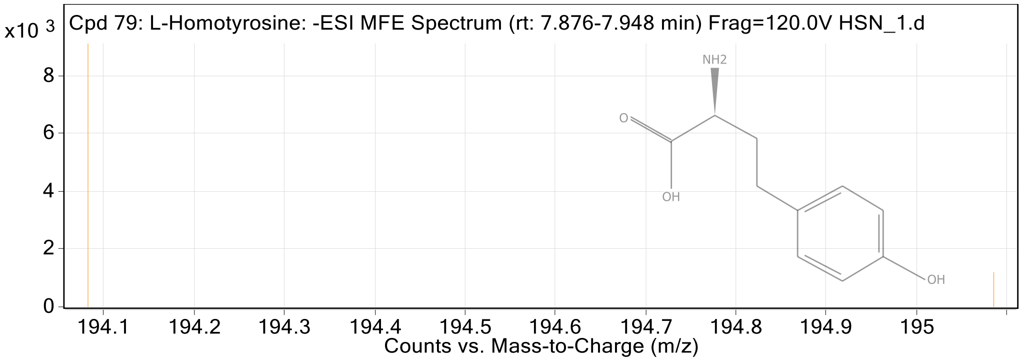

Peak List

m/z	z	Abund	Name	Formula	Ion	Score (DB)	Hits (DB)
194.08242	-1	9115.95	L-Homotyrosine	C10 H13 N O3	(M-H)-	86.29	10
195.08555	-1	1202.15		C10 H13 N O3	(M-H)-		

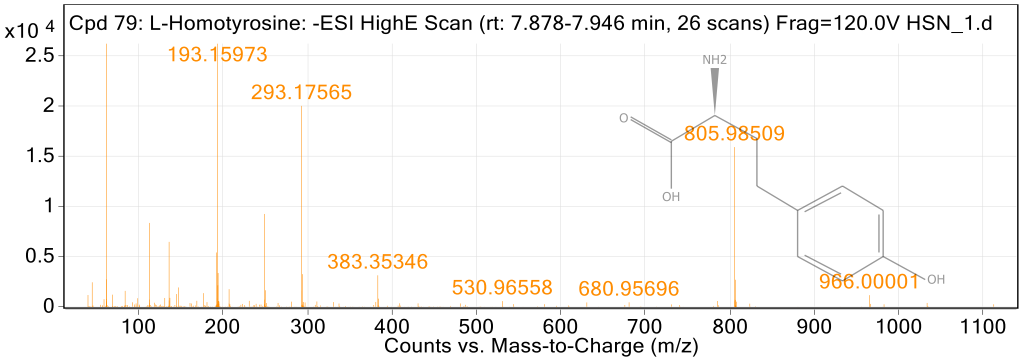

Peak List

m/z	z	Abund
61.98886		27416.66
112.98561		8347.21
136.08935	1	6459.51
192.1158	1	5401.28
193.15973	1	26212.46
194.16307	1	3355.27
249.18594	1	9240.95
293.17565	1	20012.33
294.17911	1	3245.64
805.98509	1	15903.7

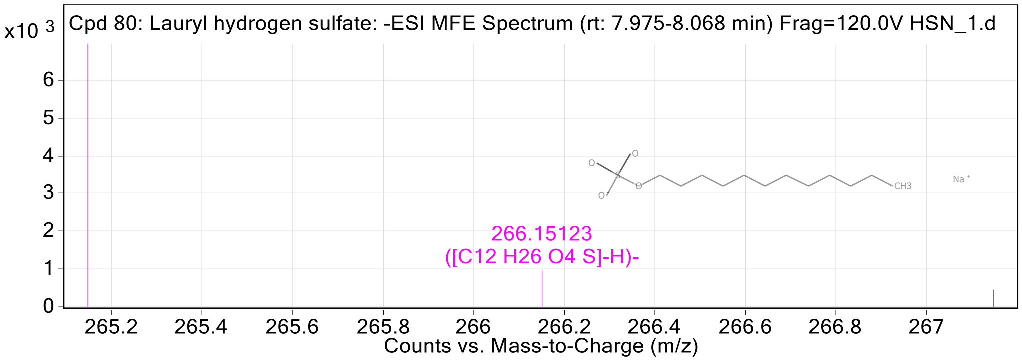

Peak List

m/z	z	Abund	Name	Formula	Ion	Score (DB)	Hits (DB)
265.14851	-1	6966.3	Lauryl hydrogen sulfate	C12 H26 O4 S	(M-H)-	97.76	1
266.15123	-1	974.39		C12 H26 O4 S	(M-H)-		
267.14793	-1	451.2		C12 H26 O4 S	(M-H)-		

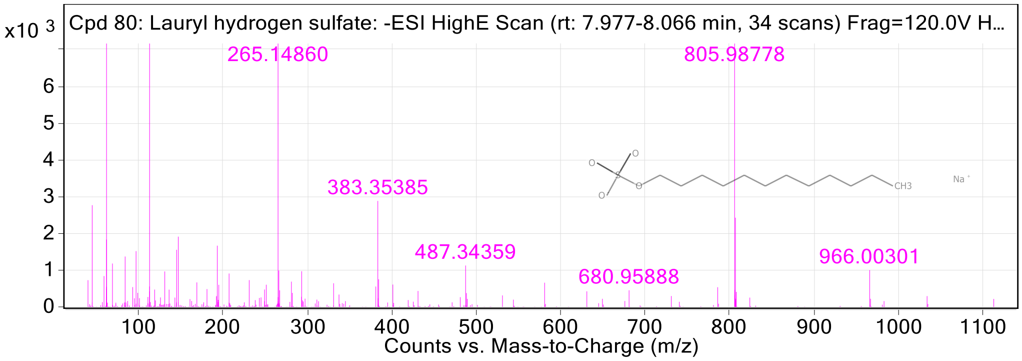

Peak List

m/z	z	Abund
44.99866		2765.82
61.98883		18395.98
62.00485		1831.35
112.98578		7365.39
146.96617		1909.96
193.16009	1	1664.19
265.1486	1	7165.47
383.35385	1	2881.1
805.98778	1	14020.72
806.99064	1	2425.1

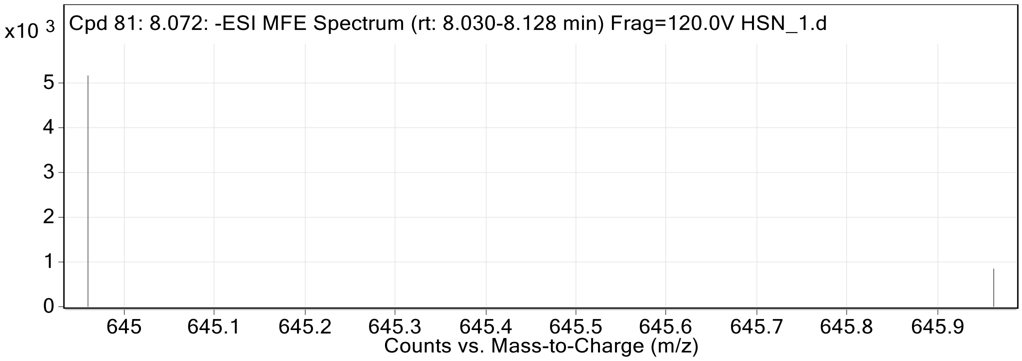

Peak List

m/z	z	Abund	Ion
644.96022	-1	5182.79	(M-H)-
645.96231	-1	854.23	(M-H)-

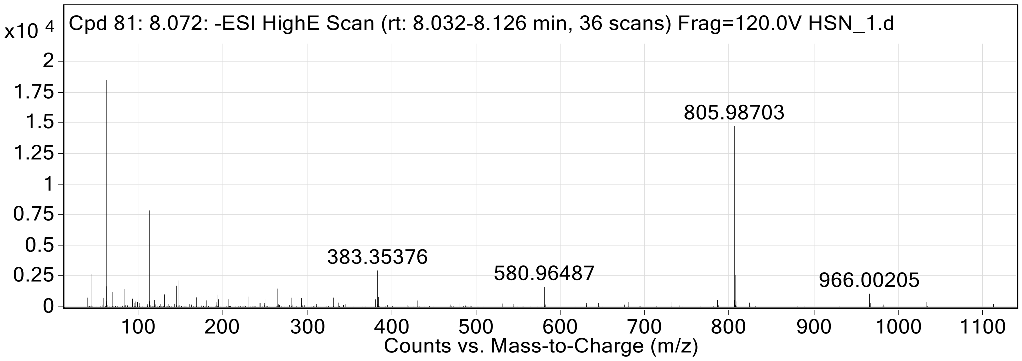

Peak List

m/z	z	Abund
44.99878		2685.6
61.98893		18504.52
62.00536		1665.88

112.98582		7863.13
144.99212		1720.38
146.96624		2148.08
383.35376	1	2963.19
580.96487	1	1630.46
805.98703	1	14747.57
806.99014	1	2590.74

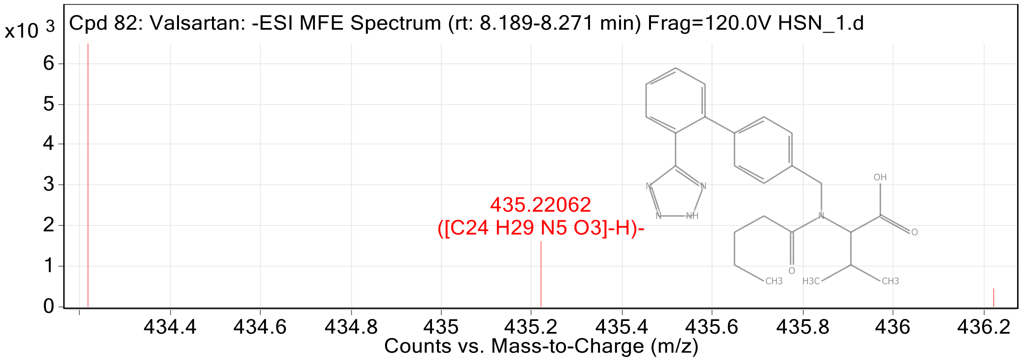

Peak List

m/z	z	Abund	Name	Formula	Ion	Score (DB)	Hits (DB)
434.21826	-1	6489.28	Valsartan	C24 H29 N5 O3	(M-H)-	88.34	1
435.22062	-1	1617.58		C24 H29 N5 O3	(M-H)-		
436.2225	-1	453.69		C24 H29 N5 O3	(M-H)-		

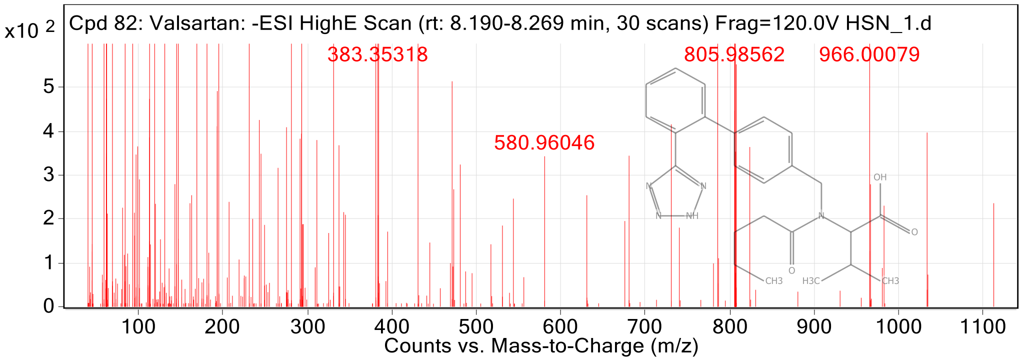

Peak List

m/z	z	Abund
44.99862		3687.06
61.98881	1	21595.26
62.00528		2048.99
68.99626		1265.65
112.98559	1	8295.56
144.99156		1420.72
146.96581		2411.38
383.35318	1	2846.28
805.98562	1	16635.44
806.98924	1	2813.83

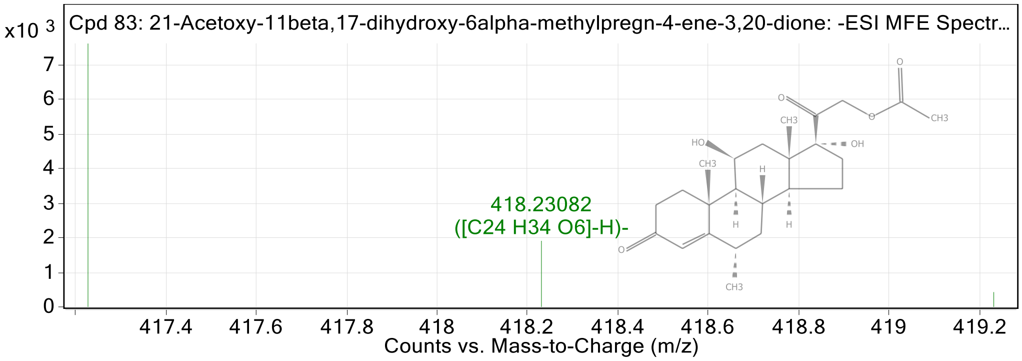

Peak List

m/z	z	Abund	Name	Formula	Ion	Score (DB)	Hits (DB)
417.22795	-1	7626.5	21-Acetoxy-11beta,17-dihydroxy-6alpha-methylpregn-4-ene-3,20-dione	C24 H34 O6	(M-H)-	97.02	10
418.23082	-1	1911.81		C24 H34 O6	(M-H)-		
419.23126	-1	428.1		C24 H34 O6	(M-H)-		

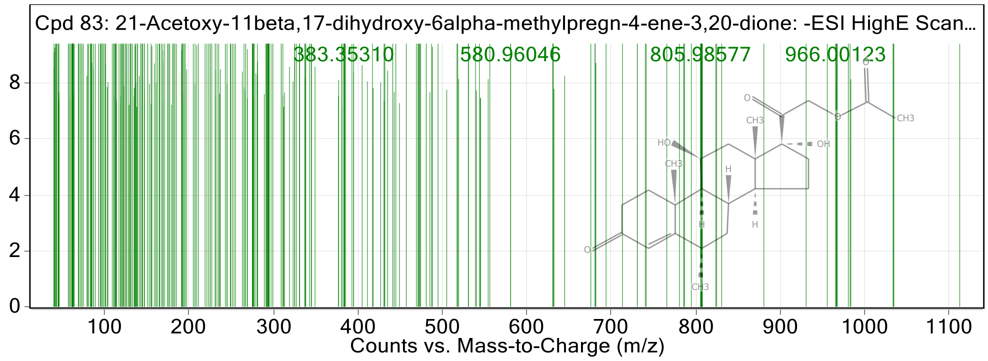

Peak List

m/z	z	Abund
44.9986		3754.37
61.98877	1	21873.36
62.00518		2061.03
84.00938		1272.22
112.98555	1	8249.74
144.99162		1417.03
146.96575		2413.08
383.3531	1	2867.79
805.98577	1	16502.29
806.98941	1	2801.87

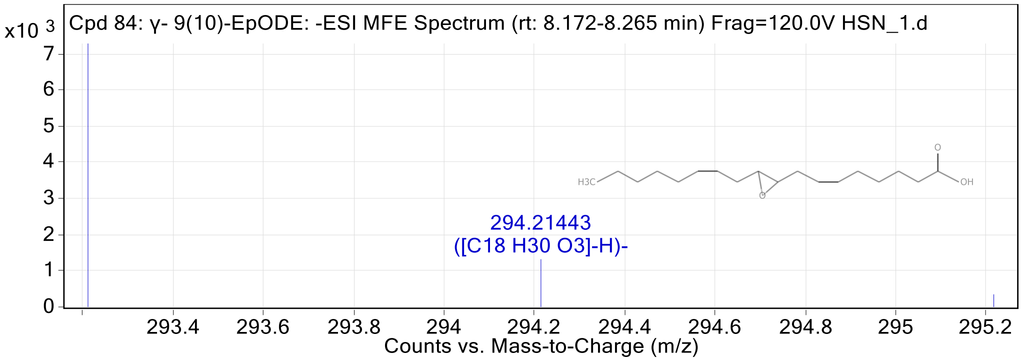

Peak List

m/z	z	Abund	Name	Formula	Ion	Score (DB)	Hits (DB)

293.21184	-1	7284.23	γ- 9(10)-EpODE	C18 H30 O3	(M-H)-	95.03	10
294.21443	-1	1321.07		C18 H30 O3	(M-H)-		
295.21678	-1	348.58		C18 H30 O3	(M-H)-		

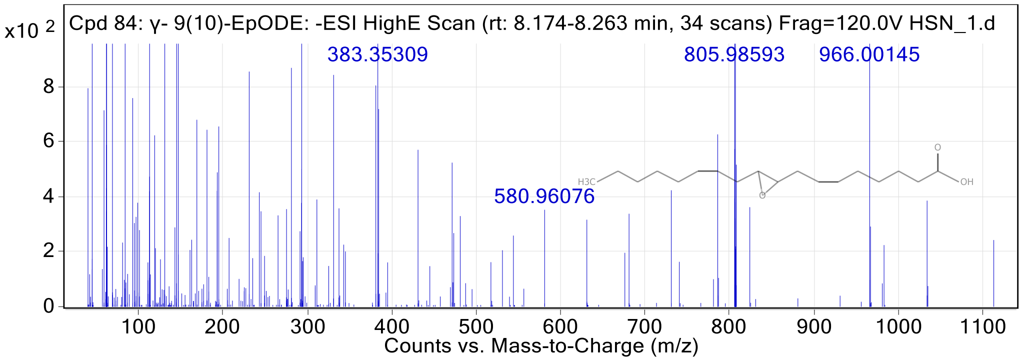

Peak List

m/z	z	Abund
44.99862		3499.31
61.9888	1	21245.27
62.00517		1999.87
68.99626		1262.33
112.9856		8222.69
144.99157		1439.42
146.96583		2373.15
383.35309	1	2815.33
805.98593	1	16270.83
806.98914	1	2803.71

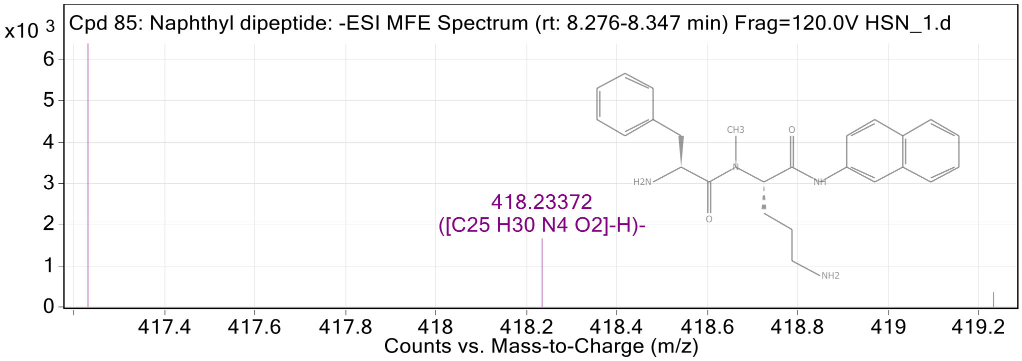

Peak List

m/z	z	Abund	Name	Formula	Ion	Score (DB)	Hits (DB)
417.23002	-1	6397.89	Naphthyl dipeptide	C25 H30 N4 O2	(M-H)-	95.2	10
418.23372	-1	1663.39		C25 H30 N4 O2	(M-H)-		
419.23216	-1	359		C25 H30 N4 O2	(M-H)-		

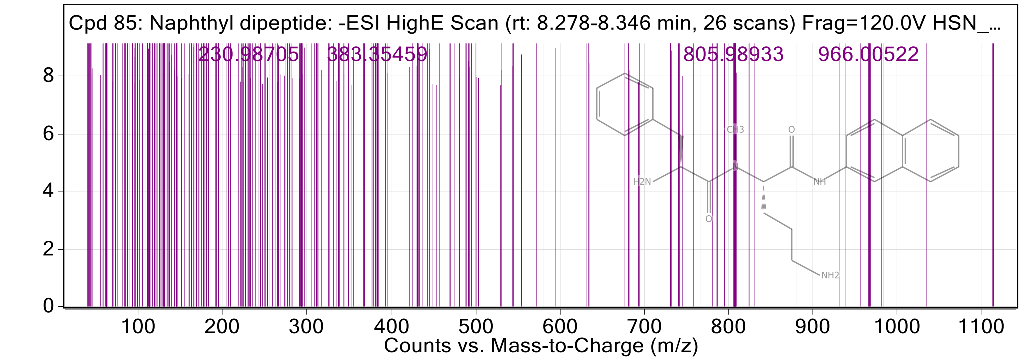

Peak List

m/z	z	Abund
44.99884		4071.65
61.98906	1	18422.21
62.00515		2068.61
84.00981		1500.62
112.98595	1	7931.8
144.99231		1623.55
146.96654		2256.37
383.35459	1	2946.69
805.98933	1	15391.23
806.99275	1	2560.23

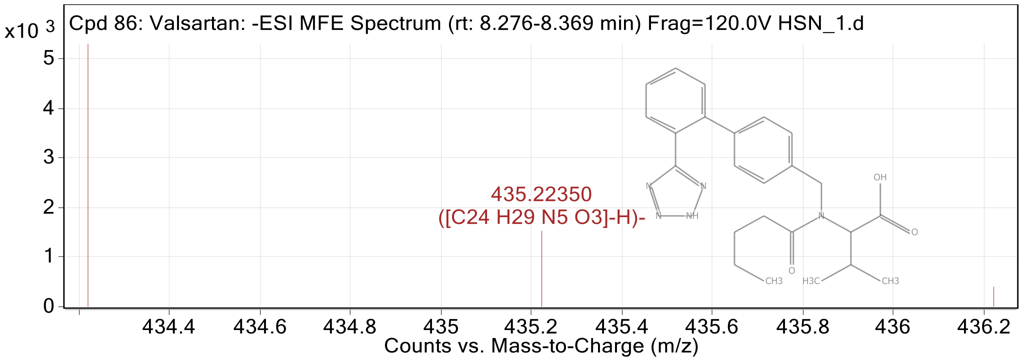

Peak List

m/z	z	Abund	Name	Formula	Ion	Score (DB)	Hits (DB)
434.22059	-1	5311.13	Valsartan	C24 H29 N5 O3	(M-H)-	93.41	1
435.2235	-1	1536.25		C24 H29 N5 O3	(M-H)-		
436.22234	-1	404.24		C24 H29 N5 O3	(M-H)-		

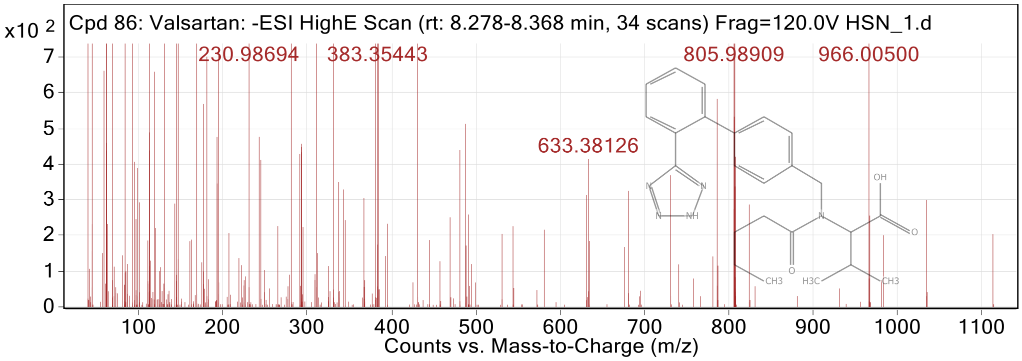

Peak List

m/z	z	Abund
44.99881		3803.53
61.98902	1	17697.06
62.00496		1855.4
84.00972		1530.91
112.98594	1	7468.13
144.99232		1606.84
146.9665		2213.59
383.35443	1	3248.97
805.98909	1	14384.3
806.99246	1	2410.39

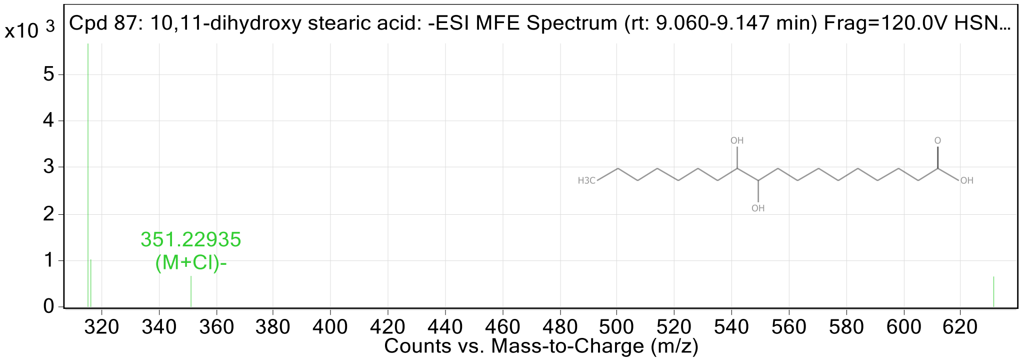

Peak List

m/z	z	Abund	Name	Formula	Ion	Score (DB)	Hits (DB)
315.25395	-1	5669.1	10,11-dihydroxy stearic acid	C18 H36 O4	(M-H)-	84.94	10
316.25783	-1	1021.88		C18 H36 O4	(M-H)-		
351.22935	-1	666			(M+Cl)-		
631.51179	-1	653.52			(2M-H)-		

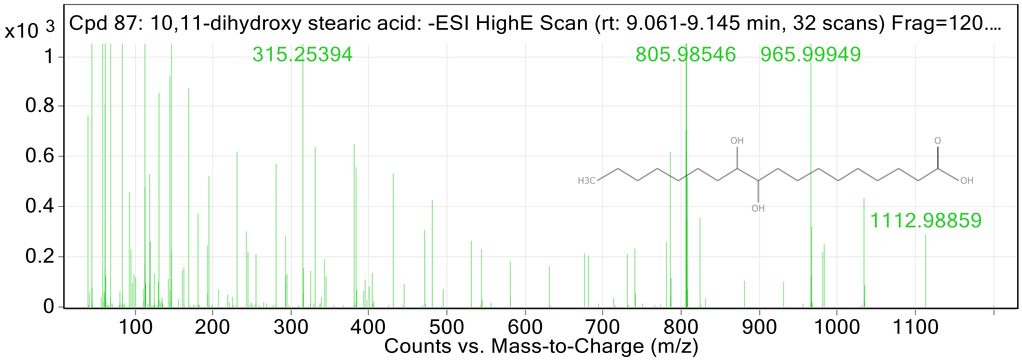

Peak List

m/z	z	Abund
44.99852		2417.51
59.01427		1601.33
61.98873	1	12438.41
68.99607		1159.38
84.00949		1283.96
112.98548	1	8616.13
146.96567		3699.15
805.98546	1	17298.22
806.98853	1	2933.12
965.99949	1	1292.82

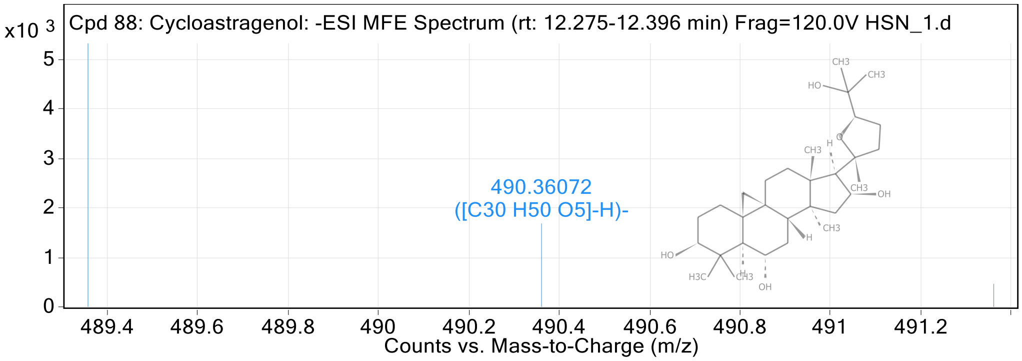

Peak List

m/z	z	Abund	Name	Formula	Ion	Score (DB)	Hits (DB)
489.35728	-1	5319.6	Cycloastragenol	C30 H50 O5	(M-H)-	92.08	10

490.36072	-1	1684.49		C30 H50 O5	(M-H)-		
491.36117	-1	464.79		C30 H50 O5	(M-H)-		

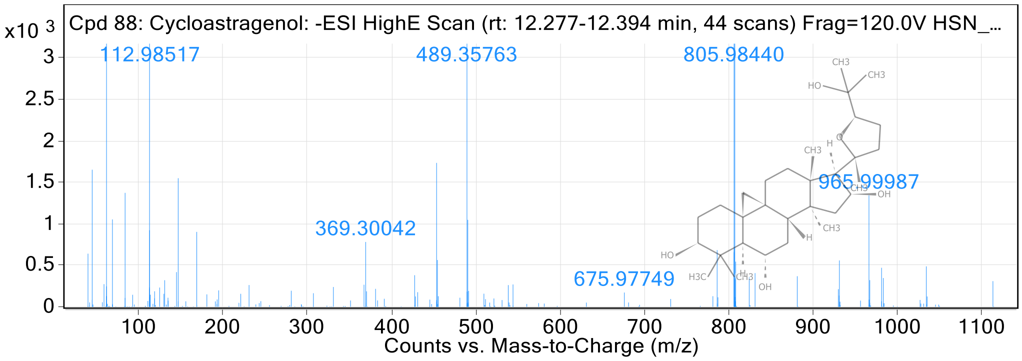

Peak List

m/z	z	Abund
44.99842		1651.42
61.98854		4618.94
84.00898		1372.39
112.98517	1	10437.78
146.9655		1549.1
453.33678	1	1733.45
489.35763	1	3169.9
805.9844	1	18839.27
806.98749	1	3216.56
965.99987	1	1341.85

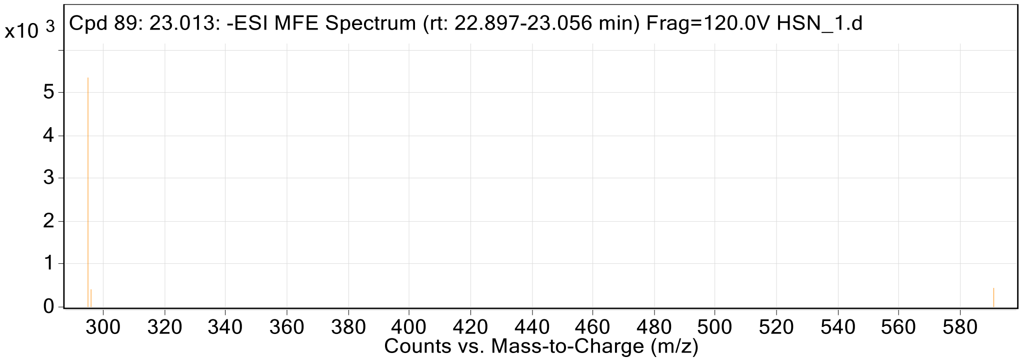

Peak List

m/z	z	Abund	Ion
294.98214	-1	5360.65	(M-H)-
295.98522	-1	413.67	(M-H)-
590.97204	-1	446.26	(2M-H)-

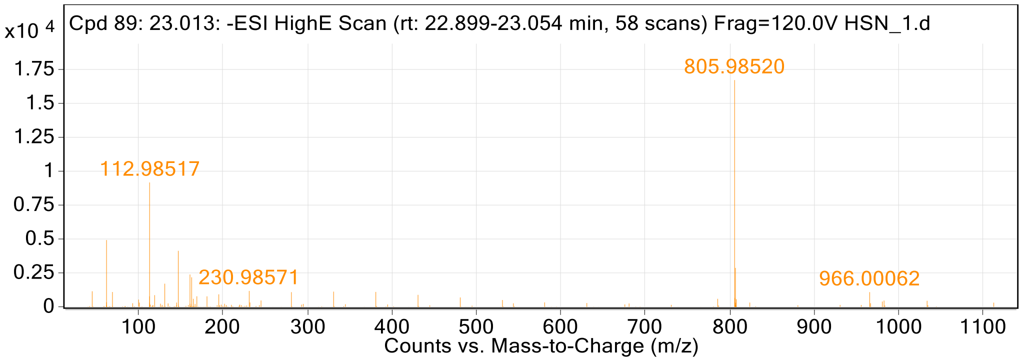

Peak List

m/z	z	Abund

44.99831		1143.73
61.98847		4918.1
112.98517	1	9161.82
130.99226		1702.68
146.96551		4123.73
160.84144		2380.96
162.8383		2173.13
230.98571		1168.61
805.9852	1	16703.92
806.98842	1	2864.95

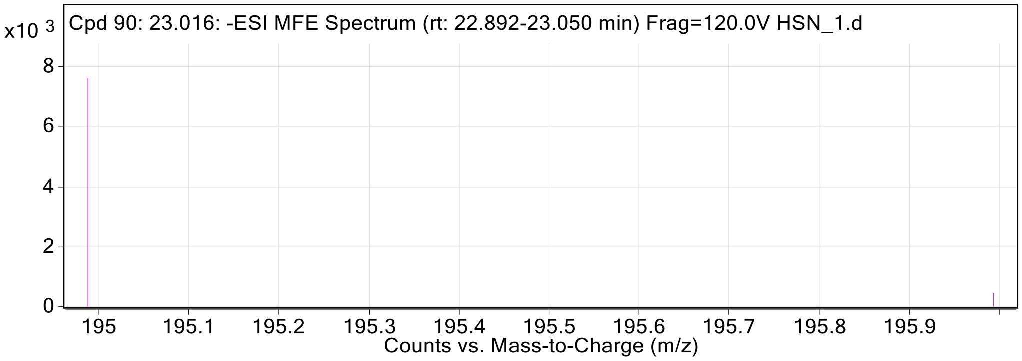

Peak List

m/z	z	Abund	Ion
194.98811	-1	7623.9	(M-H)-
195.99318	-1	457.71	(M-H)-

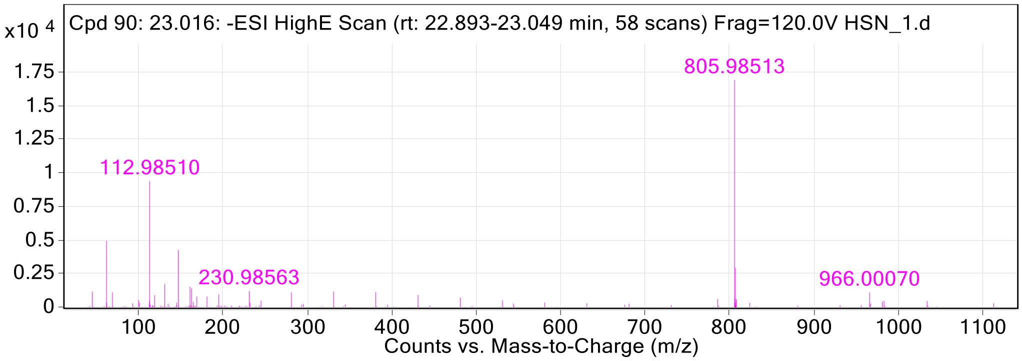

Peak List

m/z	z	Abund
61.98843		4936.01
112.9851	1	9384.5
130.99219		1718.3
146.96543		4252.01
160.84135		1514.94
162.83811		1389.42
230.98563		1192.83
330.97922		1146.2
805.98513	1	16917.19
806.98831	1	2914.78

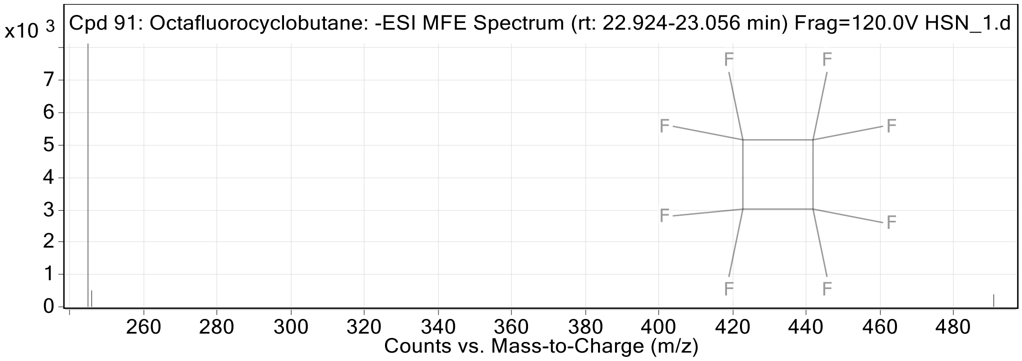

Peak List

m/z	z	Abund	Name	Formula	Ion	Score (DB)	Hits (DB)
244.98473	-1	8143.42	Octafluorocyclobutane	C4 F8	(M+HCOO)-	83.14	1
245.98647	-1	507.78		C4 F8	(M+HCOO)-		
490.97427	-1	390.98			(2M-H)-		

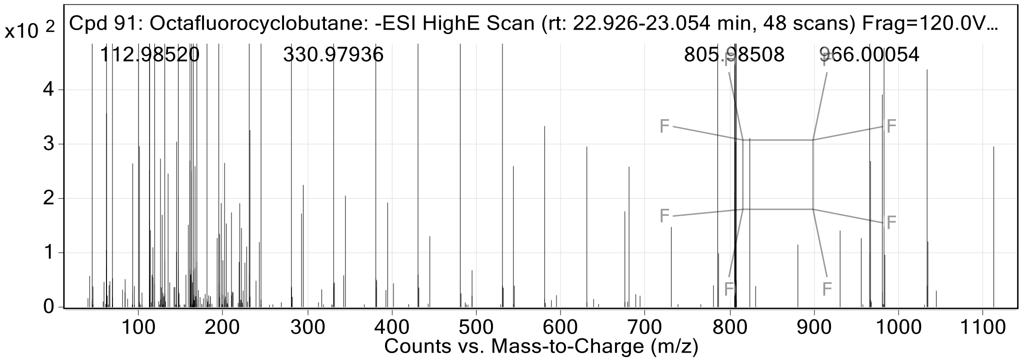

Peak List

m/z	z	Abund
61.98847		5020.05
112.9852	1	9132.49
130.99227		1786.63
146.96549		4207.75
160.84142		2876.99
162.83828		2625.86
230.98568		1211.43
330.97936		1158.47
805.98508	1	16629.33
806.98836	1	2835.71

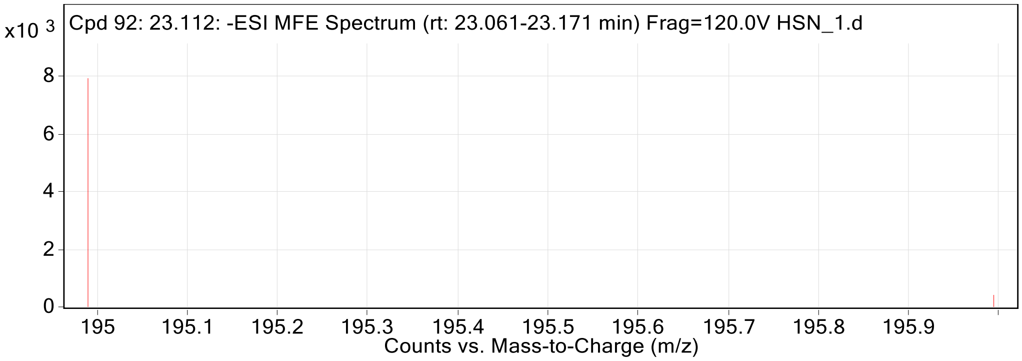

Peak List

m/z	z	Abund	Ion
194.98993	-1	7926.31	(M-H)-
195.99439	-1	415.12	(M-H)-

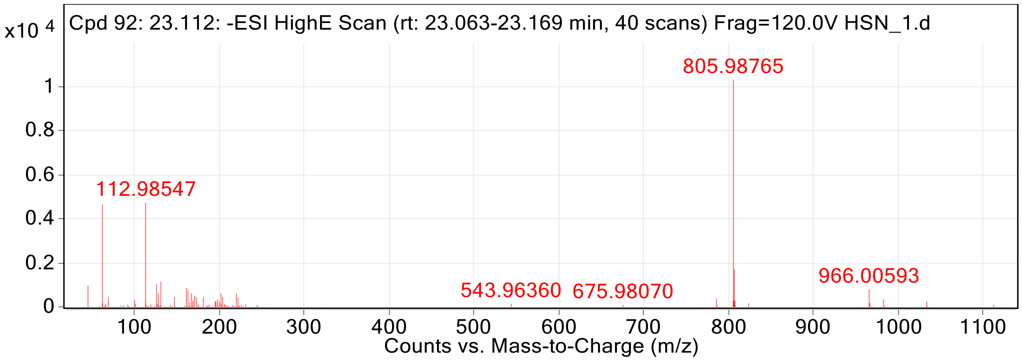

Peak List

m/z	z	Abund
44.99851		959.25
61.98858		4640.65
112.98547	1	4732.07
125.87307	1	1038.09
130.99241		1147.19
160.8419		861.73
162.83871		745.92
805.98765	1	10301.02
806.99069	1	1717.09
966.00593	1	793.45

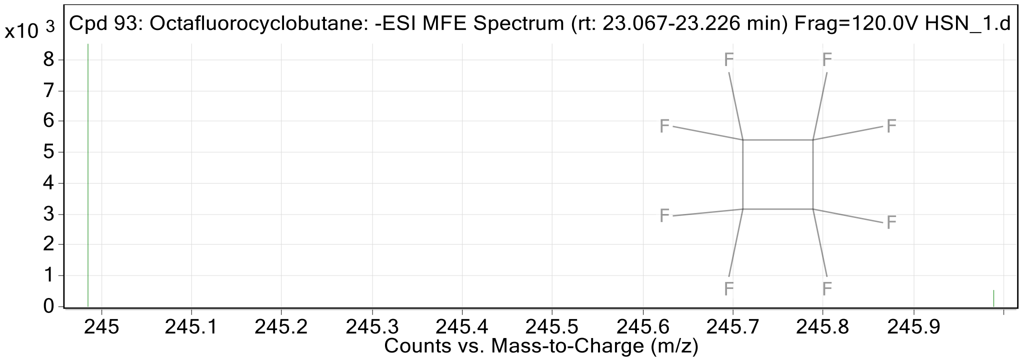

Peak List

m/z	z	Abund	Name	Formula	Ion	Score (DB)	Hits (DB)
244.98508	-1	8526.31	Octafluorocyclobutane	C4 F8	(M+HCOO)-	87.07	1
245.98872	-1	542.9		C4 F8	(M+HCOO)-		

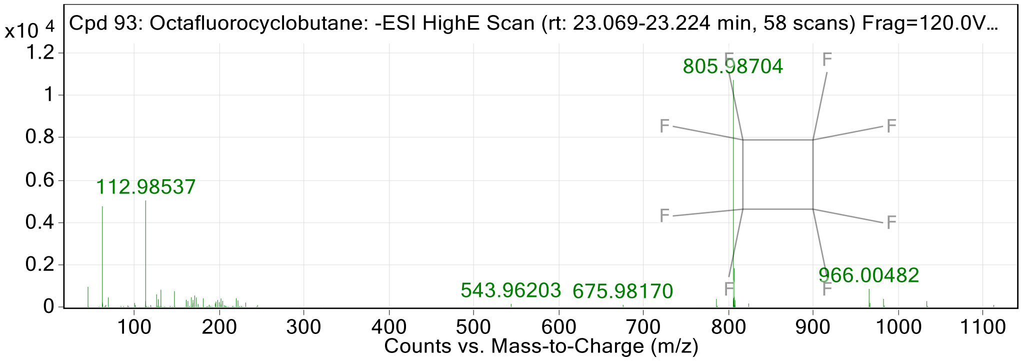

Peak List

m/z	z	Abund
44.9985		953.49
61.98852		4763.16
112.98537	1	5035.49

125.87303	1	595.22
130.99225		817.06
146.96548		745.82
170.83281		539.74
805.98704	1	10734.66
806.98977	1	1830.92
966.00482	1	854.1

--- End Of Report ---

Qualitative Analysis Report

**Data Filename**	HSN_1.d	**Sample Name**	HSN_1
**Sample Type**	Sample	**Position**	P1-A4
**Instrument Name**	6530B LC Q-TOF	**User Name**	Hasan CAN (hcan)
**Acq Method**	Unknown_3-Expt_(pos).m	**Acquired Time**	3/25/2025 11:40:50 AM
**IRM Calibration Status**	Success	**DA Method**	Default.m
**Comment**			

**Sample Group**		**Info.**	
**Stream Name**	LC 1	**Method Version**	2025-0325-0557-37078
**Override DA Method Version**		**Data File Version**	2025-0325-0840-46631
**Acquisition Workstation**	DESKTOP-L73MD3C	**DA Workstation**	DESKTOP-L73MD3C
**Acquisition SW Version**	6200 series TOF/6500 series Q-TOF (11.0.203.0)		

User Chromatograms

Fragmentor Voltage 120 **Collision Energy** 0 **Ionization Mode** ESI

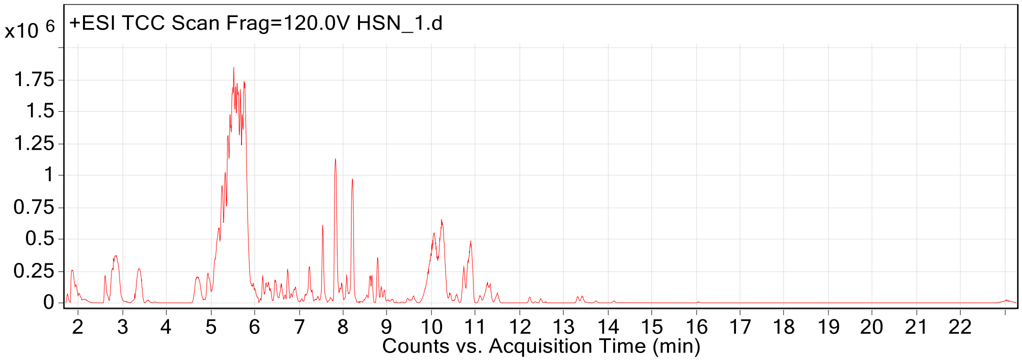

Compounds

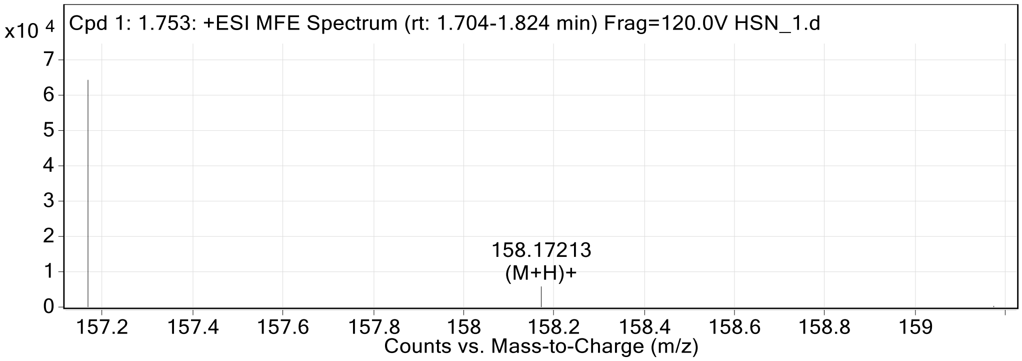

Peak List

m/z	z	Abund	Ion
157.16803	1	64217.82	(M+H)+
158.17213	1	5821.79	(M+H)+
159.17463	1	327.95	(M+H)+

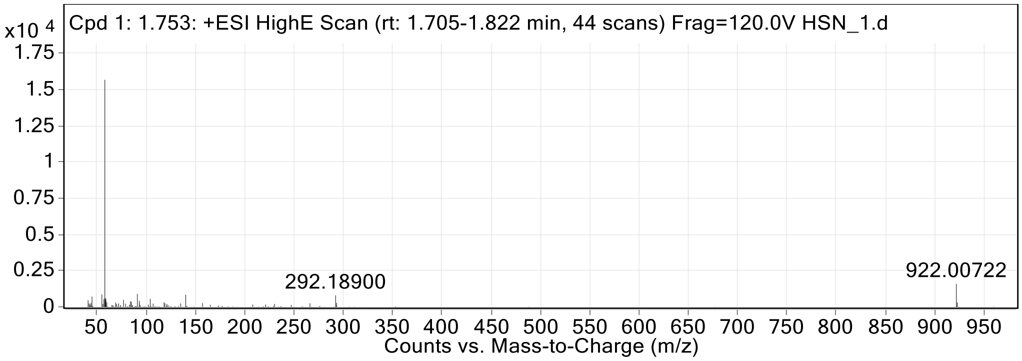

Peak List

m/z	z	Abund
45.03361		707.83
55.05427		857.84
58.06515	1	15655.97
58.08017		601.61
58.08317	1	598.25
59.0721		578.54
91.05369		893.9
140.14217	1	840.61
292.189	1	796.96
922.00722	1	1590.34

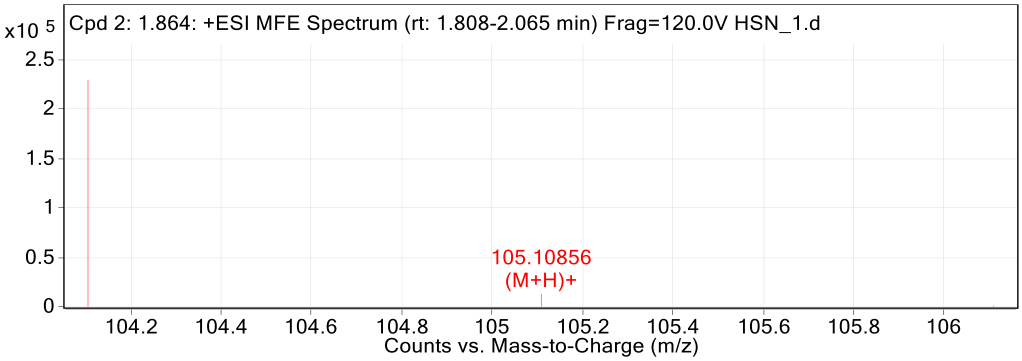

Peak List

m/z	z	Abund	Ion
104.10569	1	229734.19	(M+H)+
105.10856	1	13148.37	(M+H)+
106.11001	1	1169.9	(M+H)+

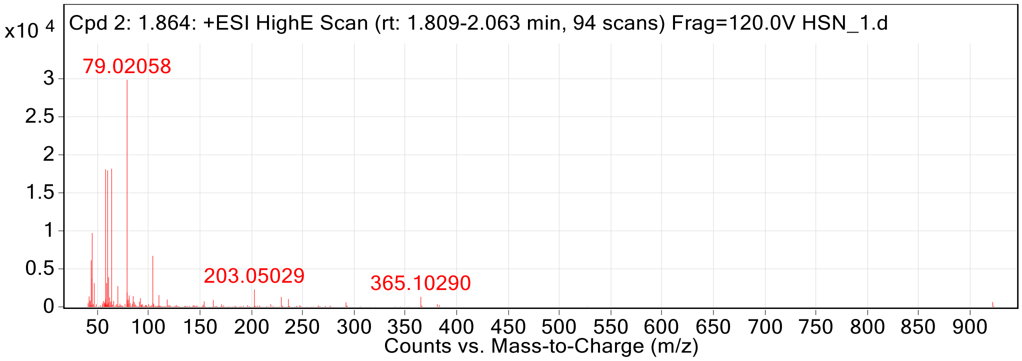

Peak List

m/z	z	Abund
44.04916		6122.34
45.03317	1	9703.88

45.05659		3760.43
58.0646	1	18071.47
59.07214		3135.51
60.08024	1	17940.91
61.01009		3883.04
63.99715	1	18166.74
79.02058	1	29830.06
104.1056	1	6710.28

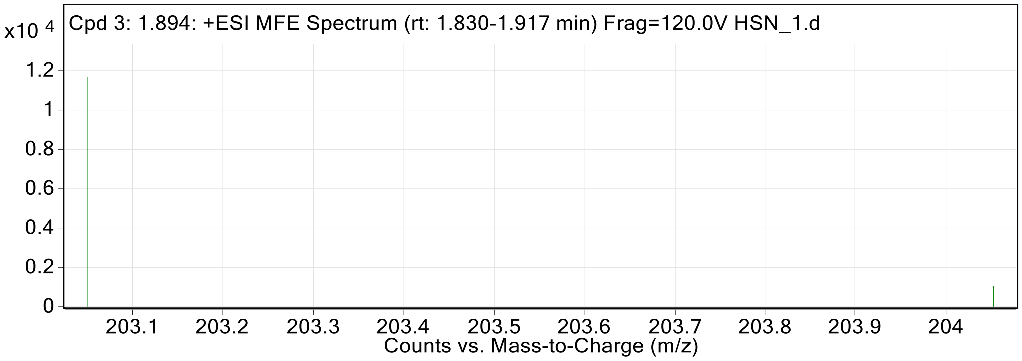

Peak List

m/z	z	Abund	Ion
203.05062	1	11666.18	(M+H)+
204.05278	1	1056.55	(M+H)+

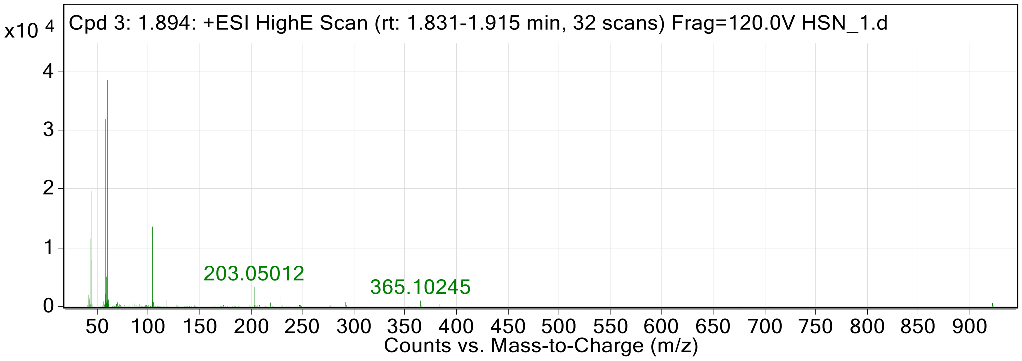

Peak List

m/z	z	Abund
42.03357		2007.18
44.04905		11561.95
45.03309	1	19672.76
45.05655	1	7951.93
58.0645	1	31880.87
58.08329		2136.24
59.07203		5075.7
60.08014	1	38566.52
104.10545	1	13580.66
203.05012	1	3288.16

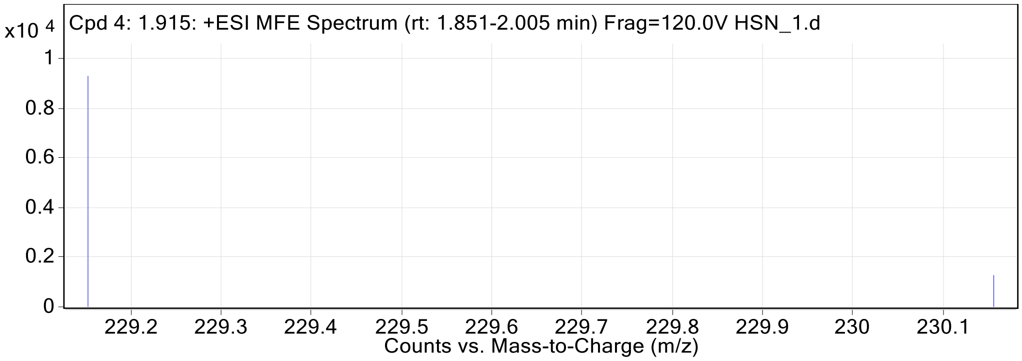

Peak List

m/z	z	Abund	Ion
229.15245	1	9298.37	(M+H)+
230.15606	1	1280.33	(M+H)+

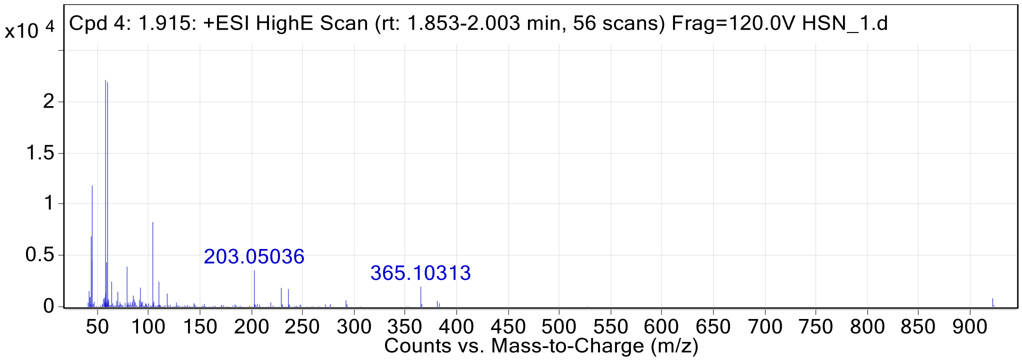

Peak List

m/z	z	Abund
44.04911		6841.2
45.03313	1	11800.8
45.05656		4548.55
58.06454	1	22099.85
59.07215		4318.31
60.0802	1	21884.72
63.99695		2453.24
79.02024		3910.38
104.10554	1	8253.77
203.05036	1	3563.85

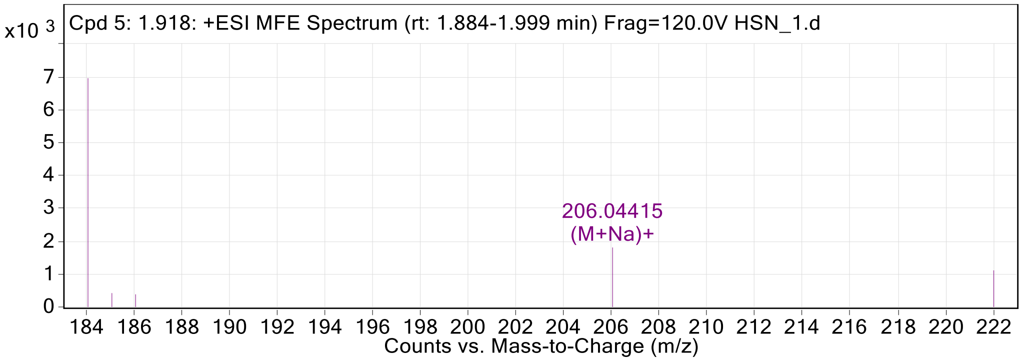

Peak List

m/z	z	Abund	Ion
184.06181	1	6958.27	(M+H)+
185.06386	1	421.89	(M+H)+
186.05682	1	383.93	(M+H)+

206.04415	1	1809.25	(M+Na)+
222.01804	1	1113.56	(M+K)+

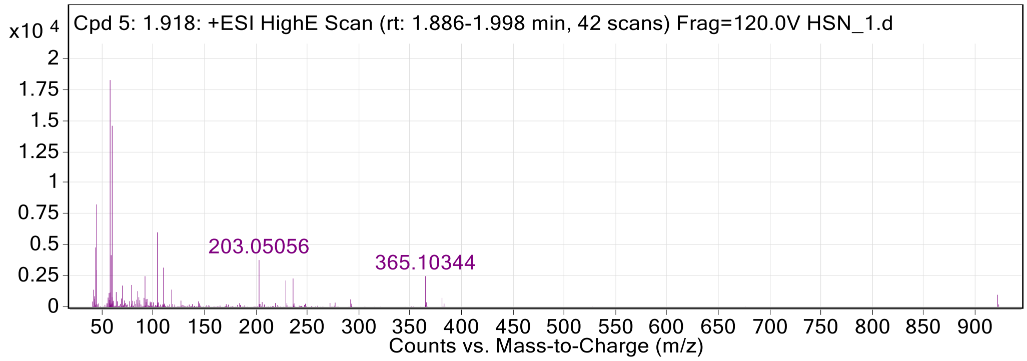

Peak List

m/z	z	Abund
44.04914	1	4782.42
45.03317	1	8250.23
45.05657		2960.49
58.06456	1	18270.26
59.07217		4157.51
60.0802	1	14583.21
104.10567	1	5998.7
110.05864	1	3153.64
203.05056	1	3765.5
365.10344	1	2467.49

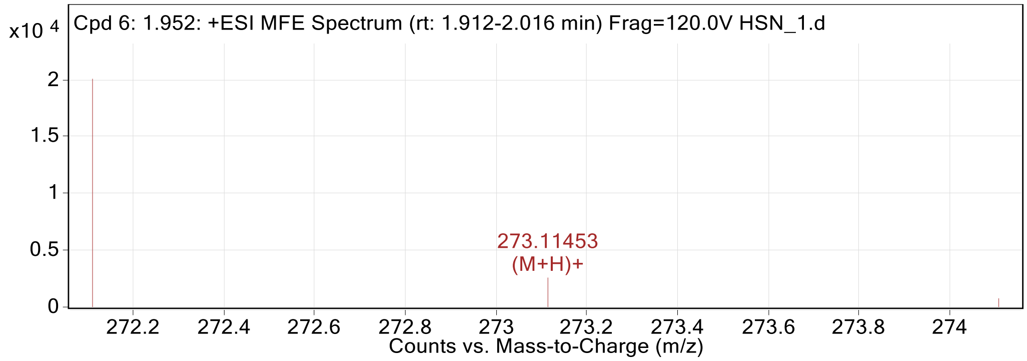

Peak List

m/z	z	Abund	Ion
272.11088	1	20079.55	(M+H)+
273.11453	1	2588.59	(M+H)+
274.10819	1	765.4	(M+H)+

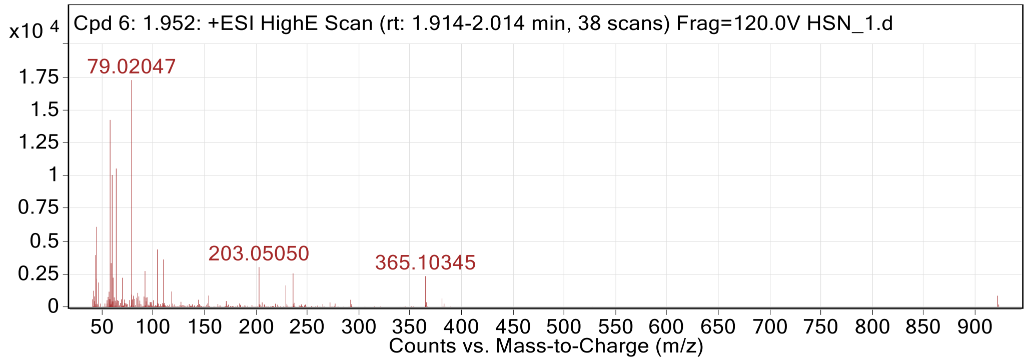

Peak List

m/z	z	Abund

44.04915		3938.75
45.03316		6087.94
58.06455		14229.27
59.07208		3325.53
60.08021	1	10036.27
63.9971		10529.36
79.02047	1	17264.87
104.10564	1	4369.16
110.05863	1	3605.65
203.0505		3022.48

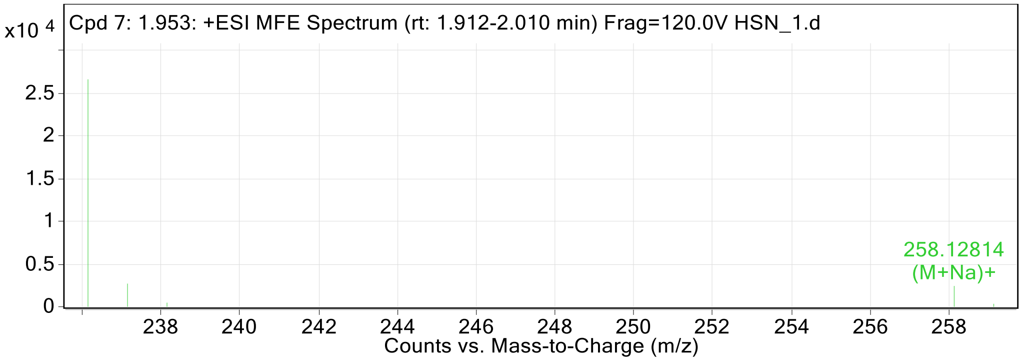

Peak List

m/z	z	Abund	Ion
236.14719	1	26606.83	(M+H)+
237.1501	1	2706.14	(M+H)+
238.15228	1	479.37	(M+H)+
258.12814	1	2432.31	(M+Na)+
259.13289	1	346.26	(M+Na)+

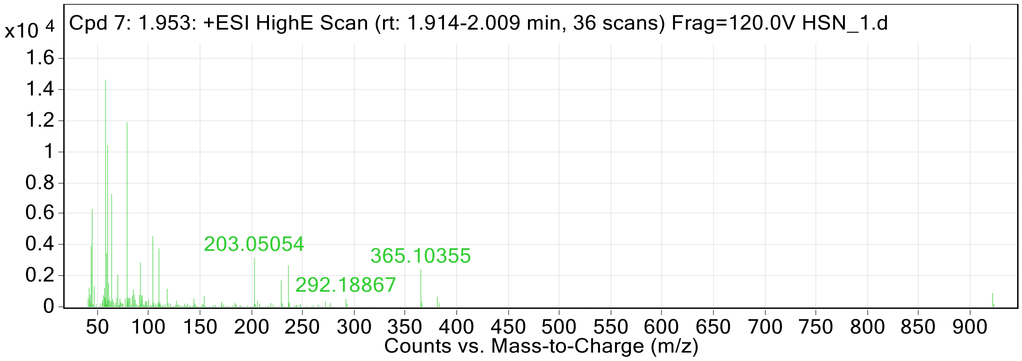

Peak List

m/z	z	Abund
44.04917		3925.21
45.03317		6293.71
58.06456		14636.34
59.07207		3482.05
60.08022	1	10391.23
63.99712		7277.37
79.0204	1	11912.96
104.10565	1	4548.94
110.05865	1	3761.29
203.05054		3171.71

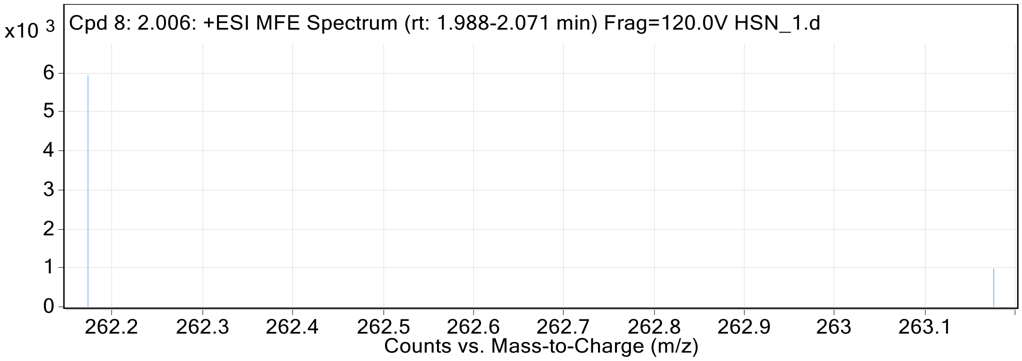

Peak List

m/z	z	Abund	Ion
262.17307	1	5937.93	(M+H)+
263.17647	1	985.9	(M+H)+

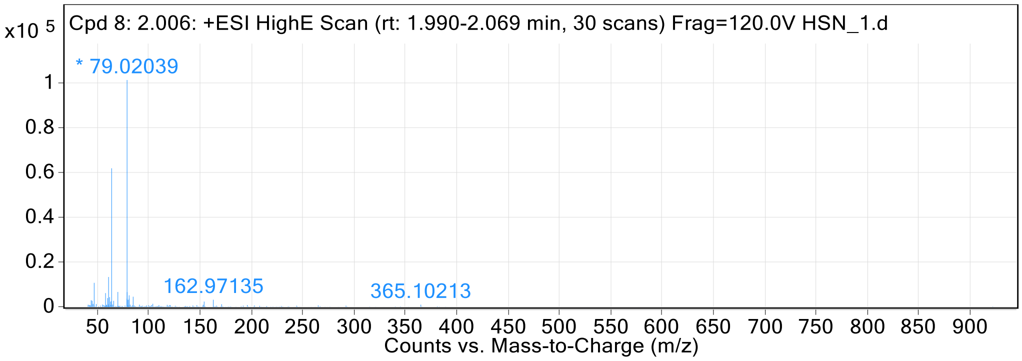

Peak List

m/z	z	Abund
46.99453		10690.29
58.06445		6088.48
61.00992		13305.38
62.01748		4292.62
63.99699	1	61962.25
70.0642	1	6603.9
79.02039	1	101477.59
79.09848		6524.98
81.01596	1	5107.67
84.95847		4469.98

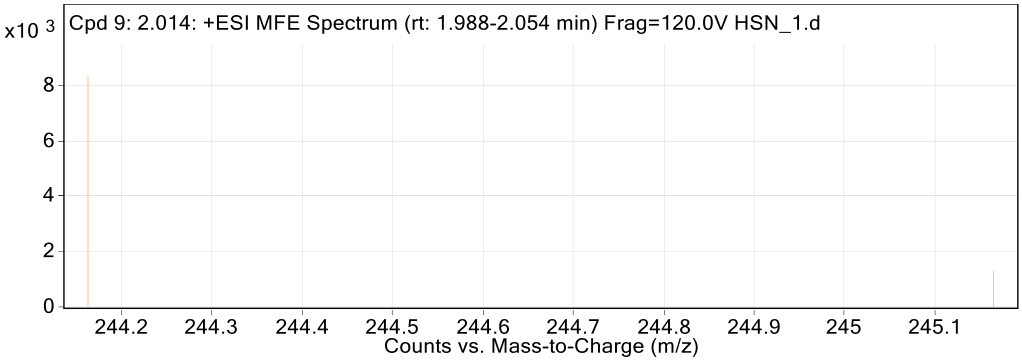

Peak List

m/z	z	Abund	Ion
244.16308	1	8357.05	(M+H)+
245.16507	1	1316.34	(M+H)+

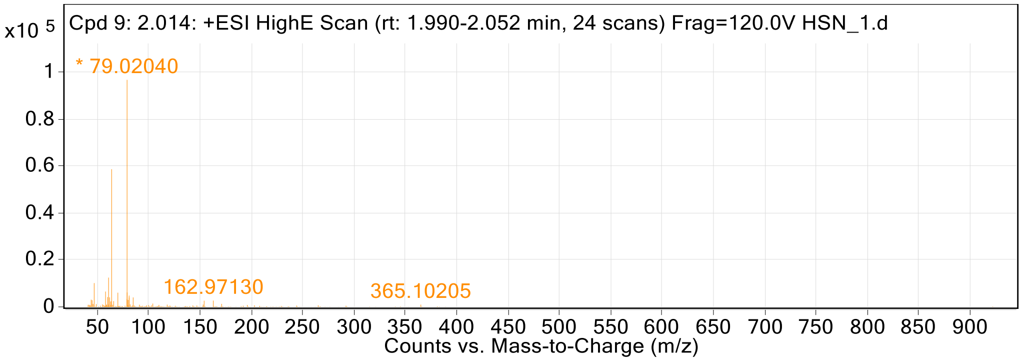

Peak List

m/z	z	Abund
46.99455		10147.97
58.06444		6536.52
60.08016		4179.64
61.00997		12482.51
62.01747		4062.35
63.99698	1	58702.51
70.06424	1	6045.57
79.0204	1	96799.78
79.09851		6145.73
81.01595	1	4828.46

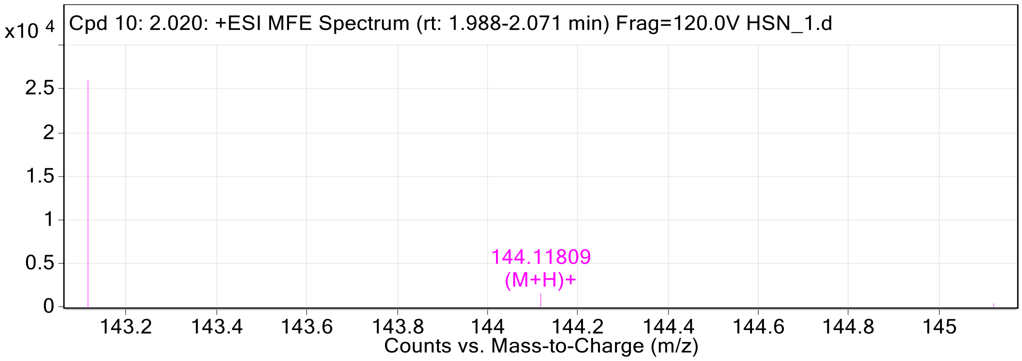

Peak List

m/z	z	Abund	Ion
143.11561	1	26082.06	(M+H)+
144.11809	1	1538.38	(M+H)+
145.12102	1	384.36	(M+H)+

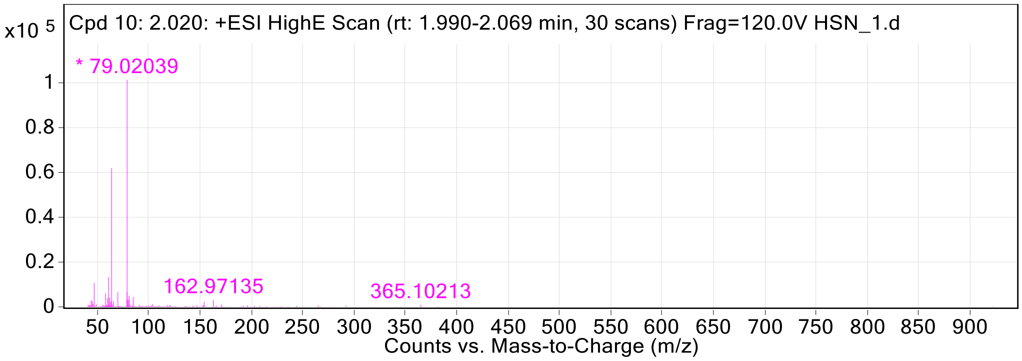

Peak List

m/z	z	Abund
46.99453		10690.29
58.06445		6088.48

61.00992		13305.38
62.01748		4292.62
63.99699	1	61962.25
70.0642	1	6603.9
79.02039	1	101477.59
79.09848		6524.98
81.01596	1	5107.67
84.95847		4469.98

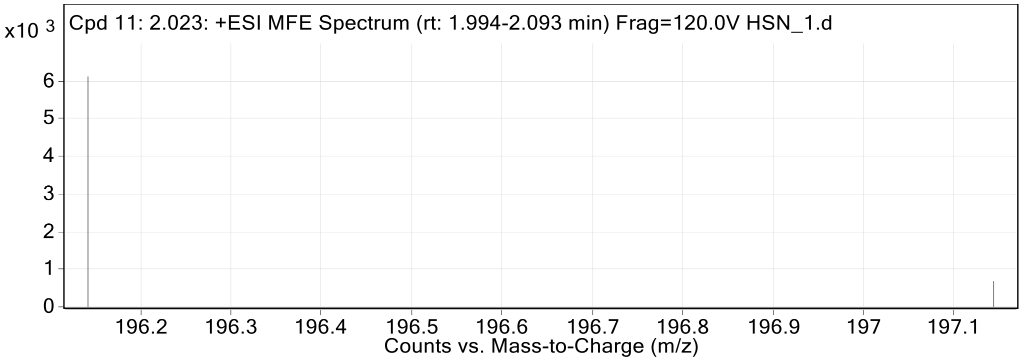

Peak List

m/z	z	Abund	Ion
196.14177	1	6117.94	(M+H)+
197.14425	1	687.98	(M+H)+

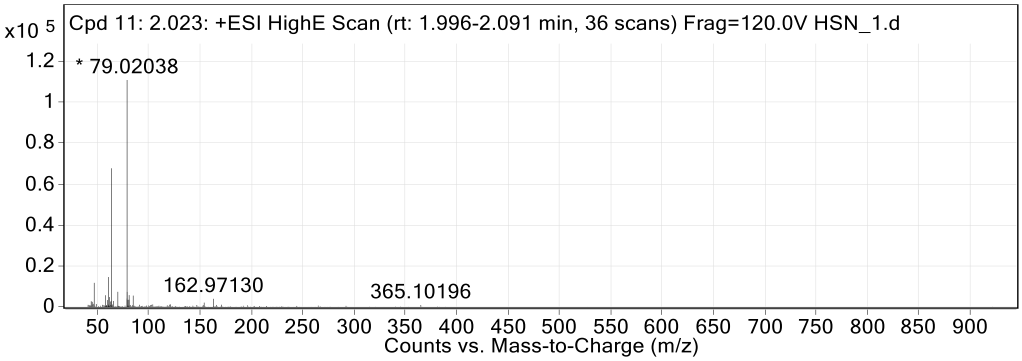

Peak List

m/z	z	Abund
46.99451		11691.45
58.06445		5612.99
61.00991		14528.83
62.01748		4730.18
63.99698	1	67770.86
70.06424	1	7298.27
79.02038	1	110976.68
79.09849		7243.03
81.01597	1	5669.05
84.95843		5440.54

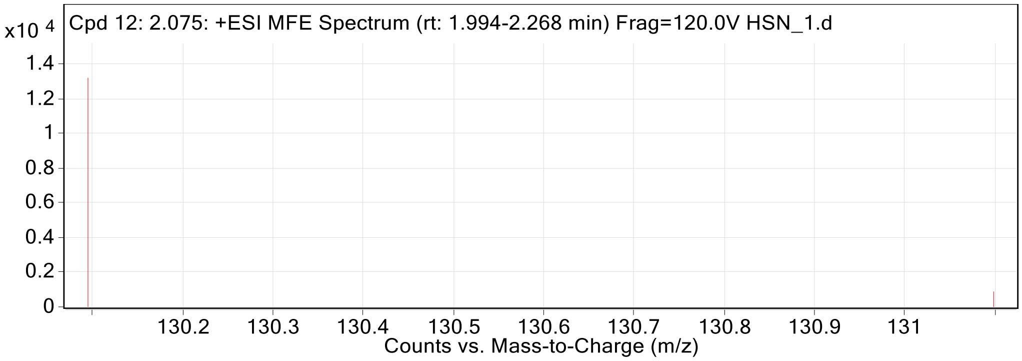

Peak List

m/z	z	Abund	Ion
130.09529	1	13216.28	(M+H)+
131.09846	1	885.55	(M+H)+

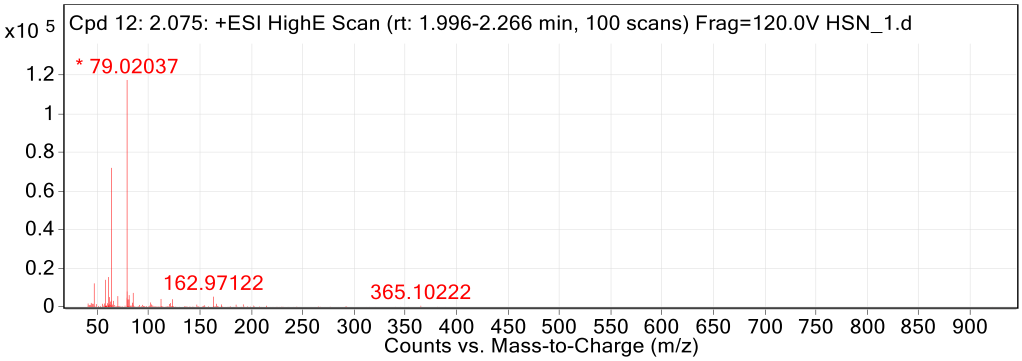

Peak List

m/z	z	Abund
46.99452		12085.91
58.06443	1	13912.98
61.00988		15356.24
63.99696	1	71861.35
70.06419		5487.7
79.02037	1	117339.31
79.09847		7890.5
81.01594	1	5982.2
84.95838		7148.53
162.97122		5205.56

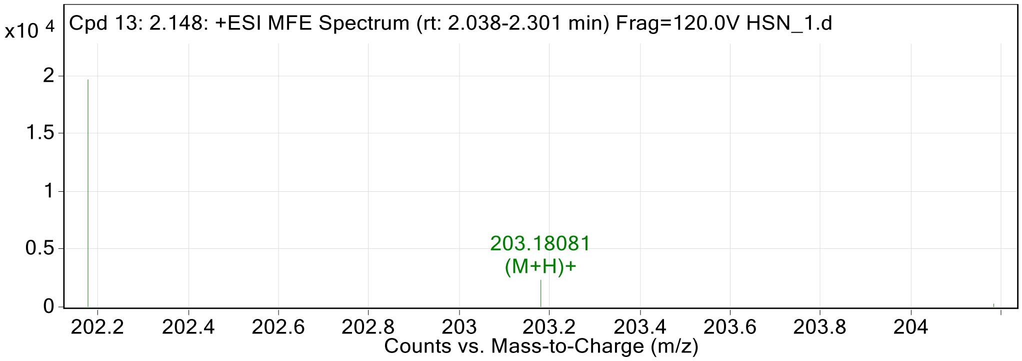

Peak List

m/z	z	Abund	Ion
202.17752	1	19675.31	(M+H)+
203.18081	1	2343.21	(M+H)+
204.18425	1	284.31	(M+H)+

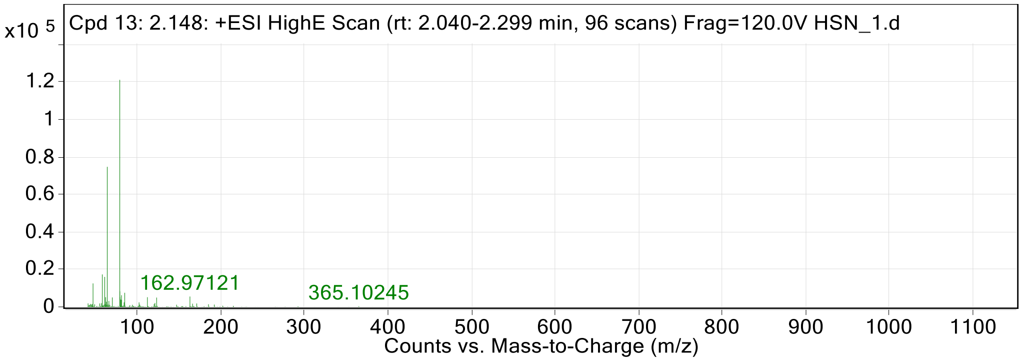

Peak List

m/z	z	Abund
46.99452		12531.2
58.06442	1	17325.01
61.00987		15994.66
63.99697	1	74751.13
79.02039	1	121186.69
79.09848		8293.2
79.11972		5218.48
81.01595	1	6229.28
84.95837		7529.3
162.97121		5528.85

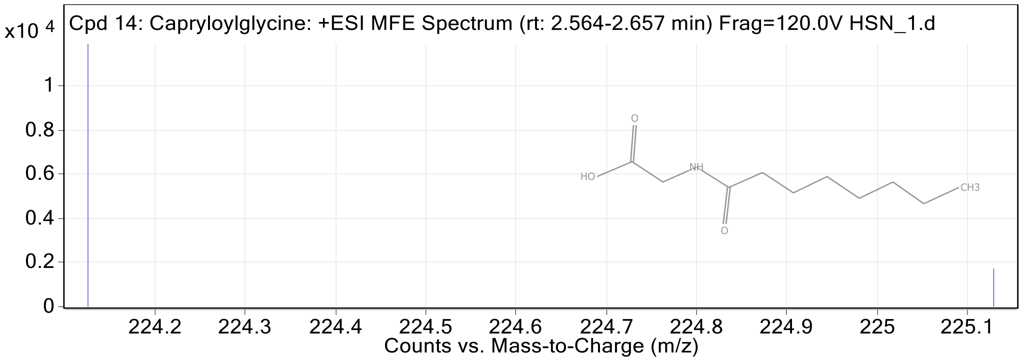

Peak List

m/z	z	Abund	Name	Formula	Ion	Score (DB)	Hits (DB)
224.12564	1	11918.53	Capryloylglycine	C10 H19 N O3	(M+Na)+	84.12	2
225.1293	1	1739.74		C10 H19 N O3	(M+Na)+		

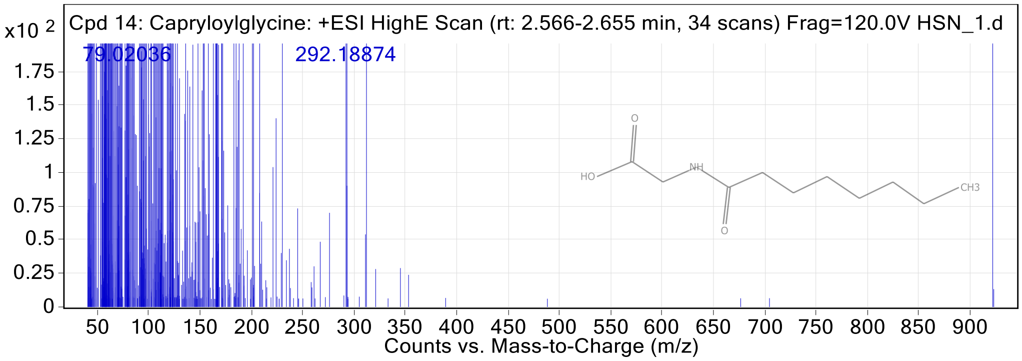

Peak List

m/z	z	Abund
46.99459		6667.82
58.0646	1	39416.38
61.01006		7645.99

63.9971	1	36697.92
79.02036	1	69037.34
79.0507		6506.93
120.07904		6510.3
121.08662		9117.93
166.08414		7978.8
171.14698	1	5602.08

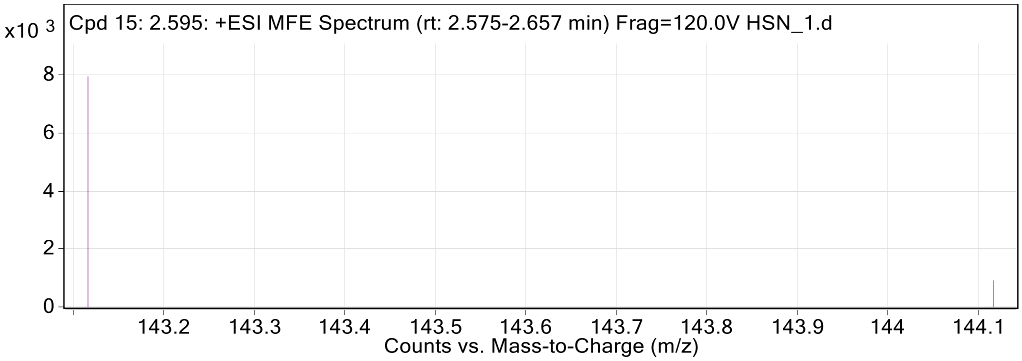

Peak List

m/z	z	Abund	Ion
143.11586	1	7954.33	(M+H)+
144.11691	1	913.47	(M+H)+

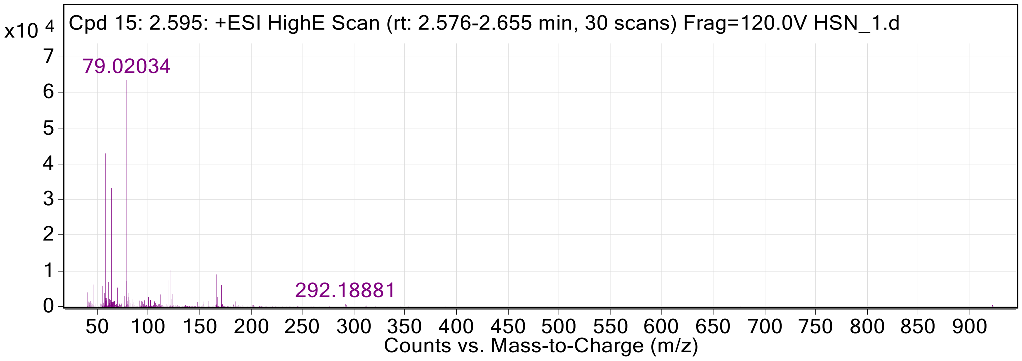

Peak List

m/z	z	Abund
46.9946		6168.87
58.06461	1	42984.9
61.01008		6933.73
63.99711	1	33212.01
79.02034	1	63645.43
79.0507	1	7200.39
120.07907		7336.58
121.08664		10283.87

m/z	z	Abund
166.08416		9012.8
171.14705	1	6053.75

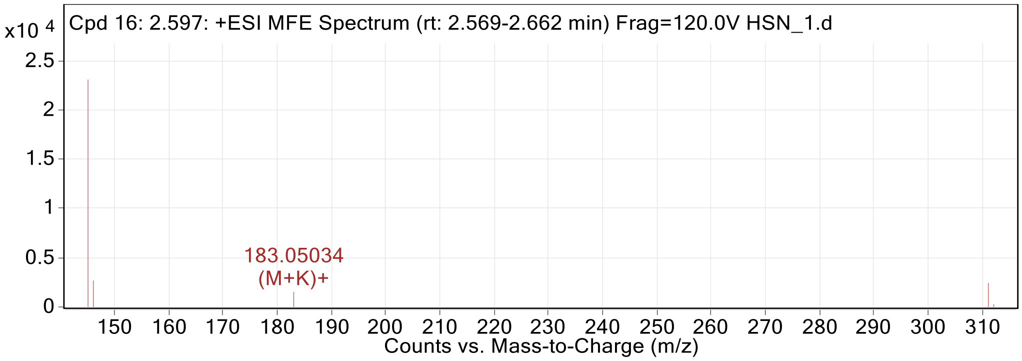

Peak List

m/z	z	Abund	Ion
145.0948	1	23086.9	(M+H)+
146.09374	1	2683.58	(M+H)+
183.05034	1	1531.54	(M+K)+
311.16627	1	2433.25	(2M+Na)+
312.16024	1	269.95	(2M+Na)+

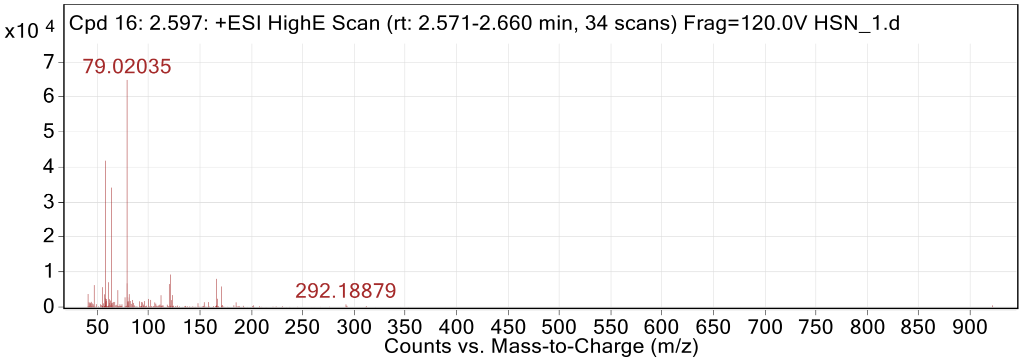

Peak List

m/z	z	Abund
46.99459		6277.06
58.06459	1	41866.91
61.01007		7071.18
63.99709	1	34153.19
79.02035	1	64897.18
79.05069		6750.06
120.07905		6600.08
121.08663		9282.24
166.08414		8078.3
171.14702	1	5864.83

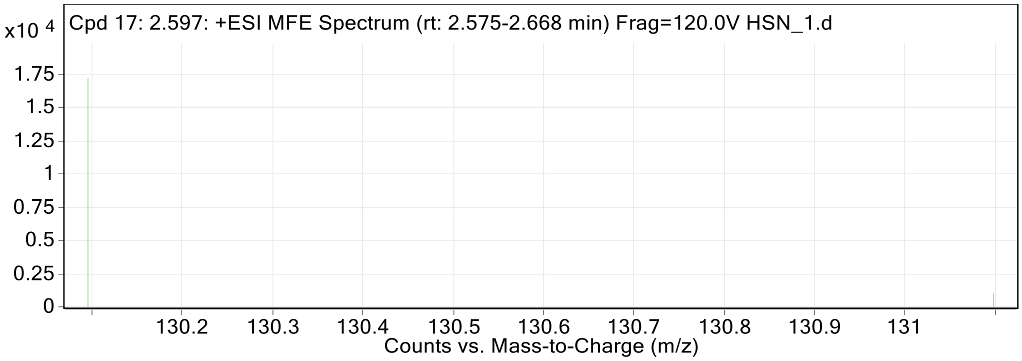

Peak List

m/z	z	Abund	Ion
130.09541	1	17250.66	(M+H)+
131.09842	1	1063.96	(M+H)+

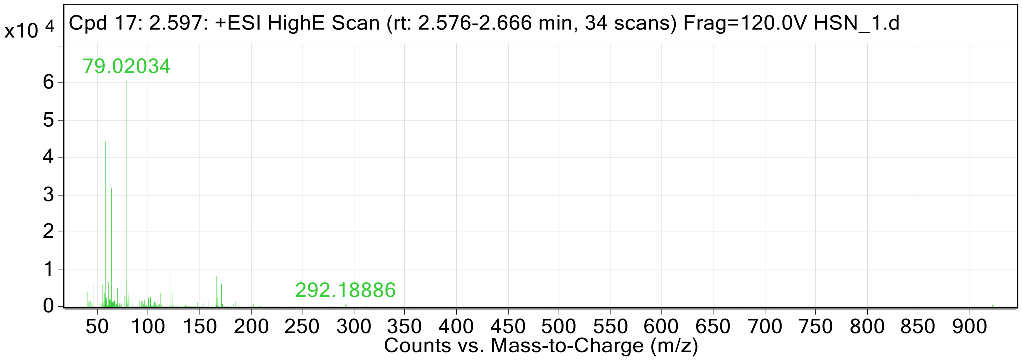

Peak List

m/z	z	Abund
55.05373		5968.53
58.0646	1	44250.53
61.01008		6599.01
63.9971	1	31708.82
79.02034	1	60893.47
79.05068		6975.91
120.07906		6694.5
121.08664		9394.13
166.08416		8153.3
171.14701	1	6150.26

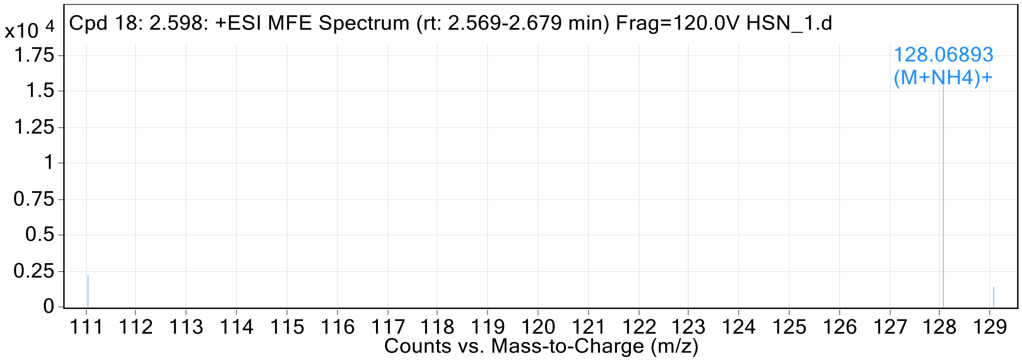

Peak List

m/z	z	Abund	Ion
111.04229	1	2217.69	(M+H)+
128.06893	1	15968.92	(M+NH4)+
129.07098	1	1404.33	(M+NH4)+

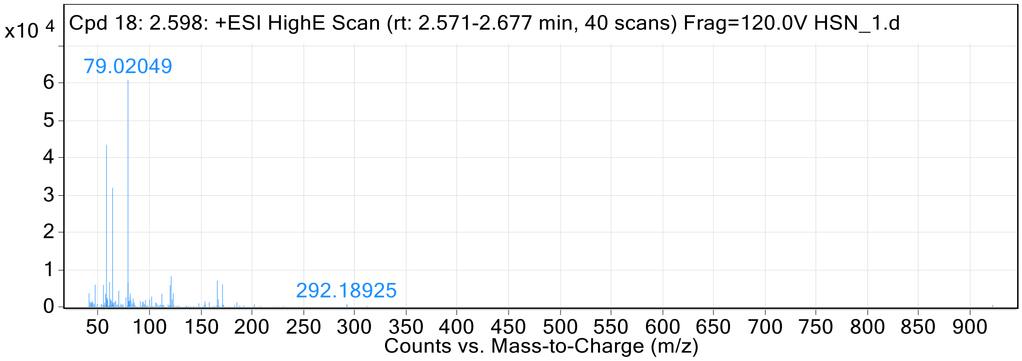

Peak List

m/z	z	Abund
46.9947		5957.05
55.05384		5893.53
58.06471	1	43489.3
61.0102		6676.45
63.99721	1	31972.15
79.02049	1	60914.71
79.05087		6486.15
121.08686		8209.14
166.08444		7081.9
171.1473	1	6021.42

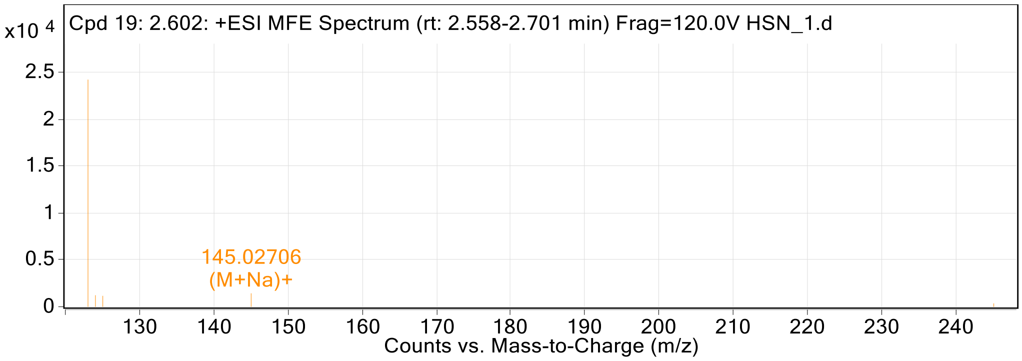

Peak List

m/z	z	Abund	Ion
123.04564	1	24205.26	(M+H)+
124.048	1	1230.54	(M+H)+
125.04126	1	1164.49	(M+H)+
145.02706	1	1433.13	(M+Na)+
245.08293	1	359.14	(2M+H)+

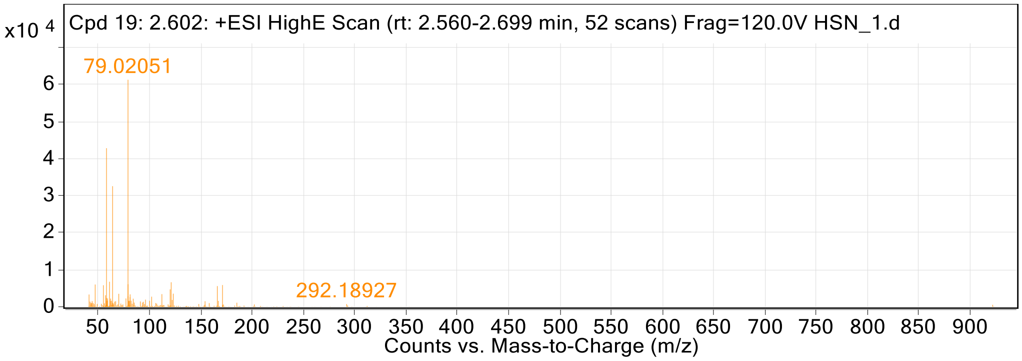

Peak List

m/z	z	Abund

46.99468		6014.03
55.05385		5822.38
58.06471	1	42831.55
61.01018		6762.07
63.99722	1	32577.65
79.02051	1	61317.75
79.0508		6077.7
121.08687		6629.03
166.08447		5589.04
171.14731	1	5870.02

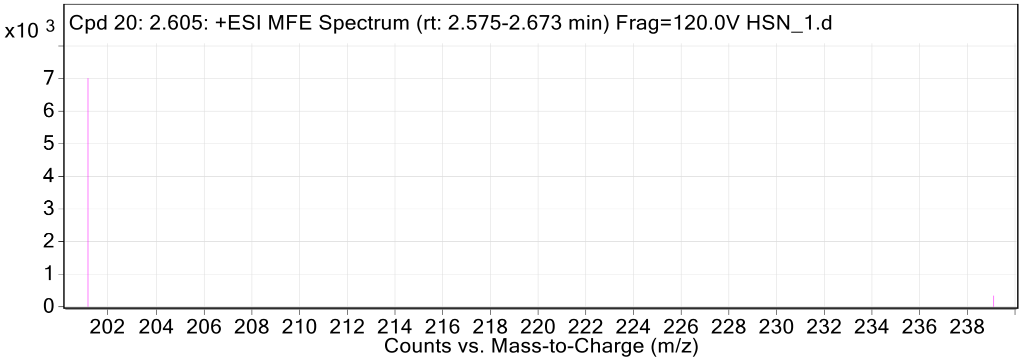

Peak List

m/z	z	Abund	Ion
201.15721	1	7030.61	(M+H)+
239.11117	1	342	(M+K)+

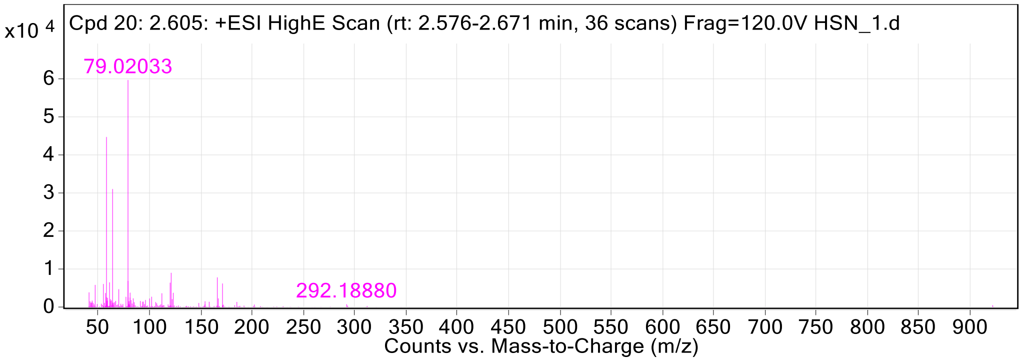

Peak List

m/z	z	Abund
55.05373		6054.58
58.06459	1	44697.6
61.01008		6479.6
63.99709	1	31019.39
79.02033	1	59672.56
79.0507		6854.94
120.07906		6407.18
121.08663		8994.95
166.08413		7787.69
171.14699	1	6186.77

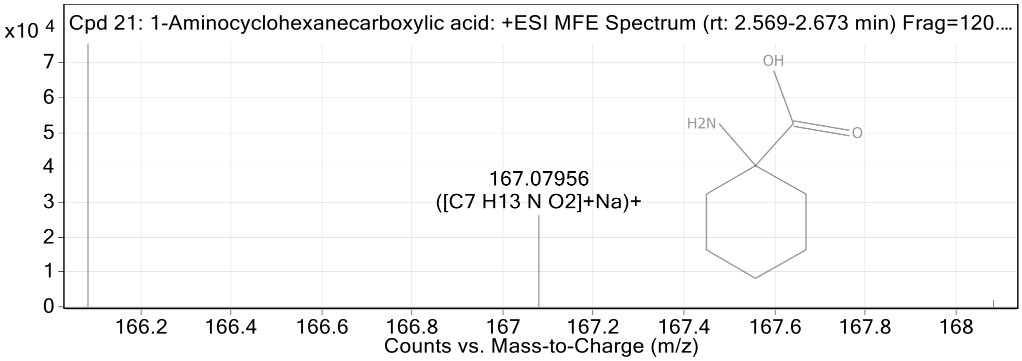

Peak List

m/z	z	Abund	Name	Formula	Ion	Score (DB)	Hits (DB)
166.08413	1	75582.04	Aminocyclohexanecarboxylic acid	C7 H13 N O2	(M+Na)+	44.3	3
167.07956	1	26335.37		C7 H13 N O2	(M+Na)+		
168.08363	1	1977.25		C7 H13 N O2	(M+Na)+		

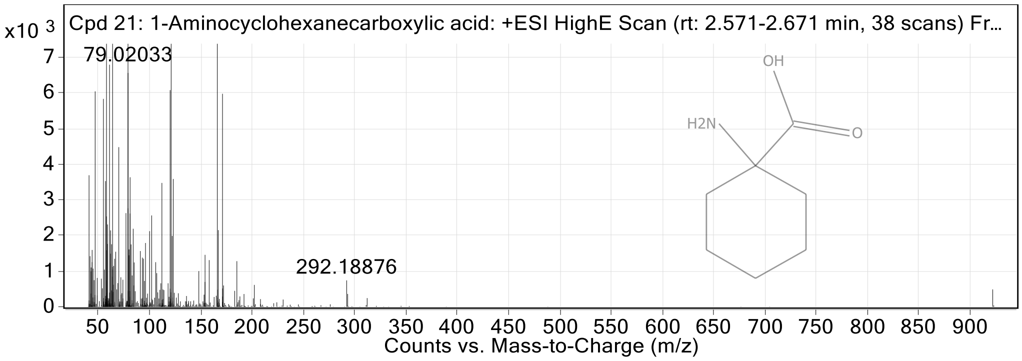

Peak List

m/z	z	Abund
46.9946		6046.54
58.06459	1	43019.28
61.01008		6795.26
63.99708	1	32617.24
79.02033	1	62172.41
79.0507		6563.31
120.07905		6081.27
121.08663		8540.42
166.08412		7385.15
171.14696	1	5978.66

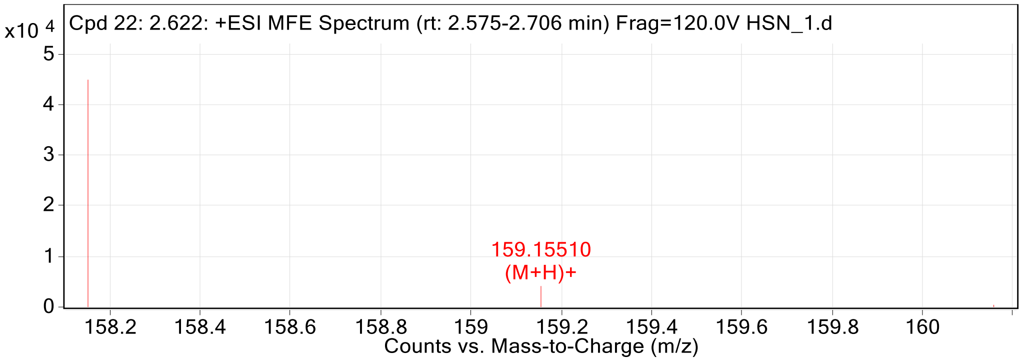

Peak List

m/z	z	Abund	Ion
158.15175	1	44888.37	(M+H)+

159.1551	1	4145.48	(M+H)+
160.15802	1	435.89	(M+H)+

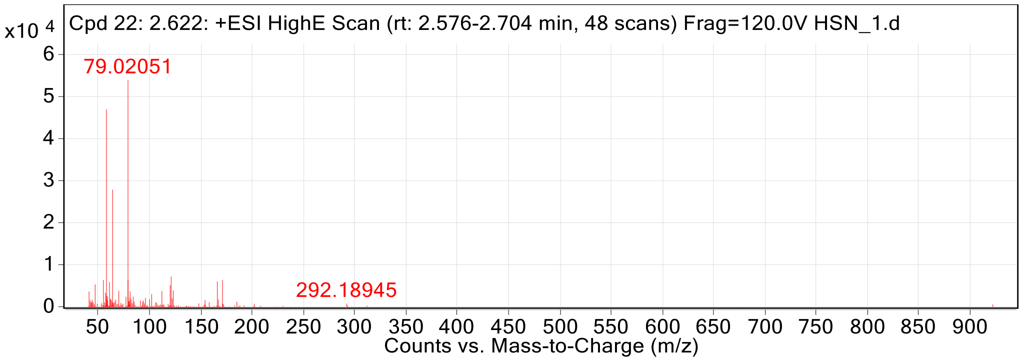

Peak List

m/z	z	Abund
46.9947		5347.88
55.05388		6375
58.06475	1	47050.61
61.01021		5887.13
63.99724	1	27903.52
79.02051	1	53988.09
79.05092		6318.27
121.08693		7192.98
166.08455		6051.62
171.1474	1	6352.39

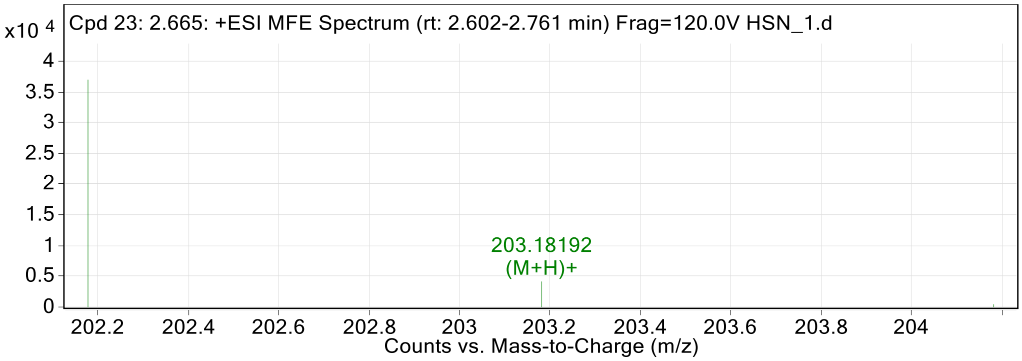

Peak List

m/z	z	Abund	Ion
202.17797	1	36983.91	(M+H)+
203.18192	1	4151.18	(M+H)+
204.18202	1	443.8	(M+H)+

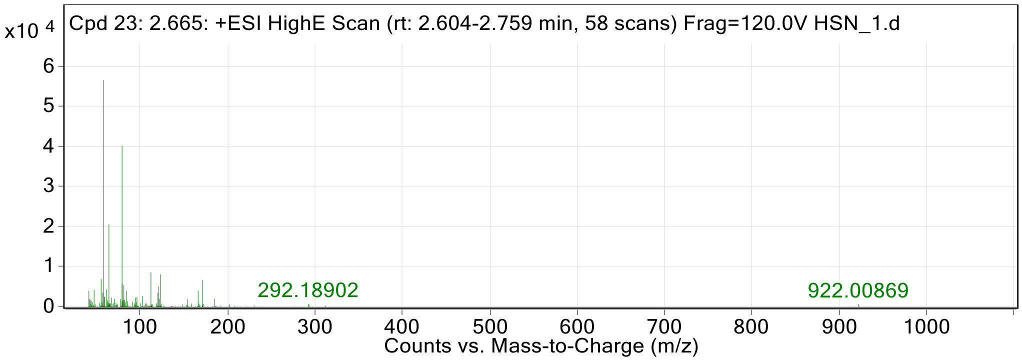

Peak List

m/z	z	Abund

55.05382		6981.12
58.06471	1	56622.37
63.99717	1	20611.57
79.02041	1	40244.29
79.05119		5783.63
81.06861		5387.74
112.07424		8617.21
121.08689		5066.12
123.11519	1	8124.49
171.14727	1	6721.15

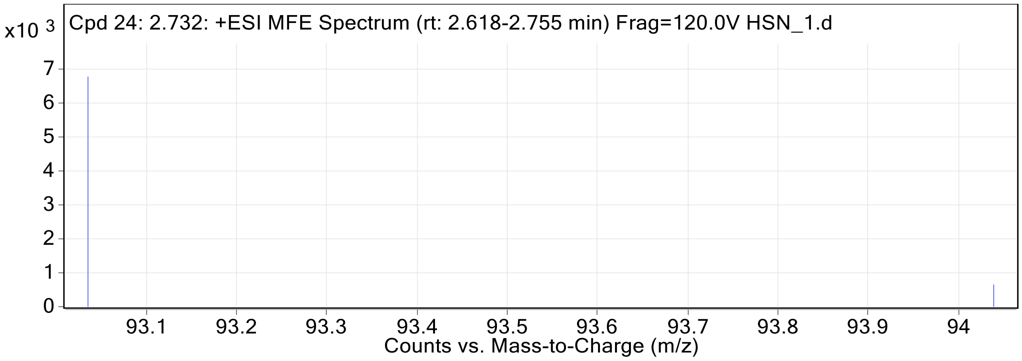

Peak List

m/z	z	Abund	Ion
93.03567	1	6777.13	(M+H)+
94.03902	1	653.97	(M+H)+

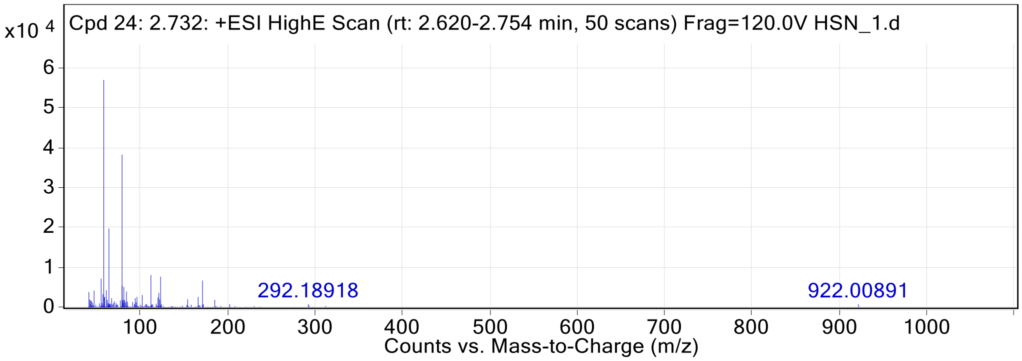

Peak List

m/z	z	Abund
55.05384		7102.9
58.06474	1	56970.41
61.01016		4211.5
63.99719	1	19692.61
79.02045	1	38274.52
79.05113		5357
81.06862		5028.7
112.07429	1	8037.09
123.1152	1	7615.4
171.14735	1	6654.18

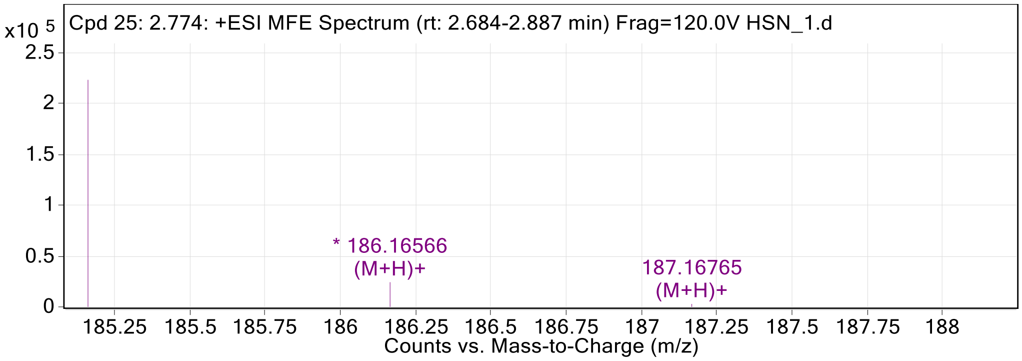

Peak List

m/z	z	Abund	Ion
185.16247	1	223467.22	(M+H)+
186.16566	1	24285.51	(M+H)+
187.16765	1	2892.96	(M+H)+
188.17055	1	26.06	(M+H)+

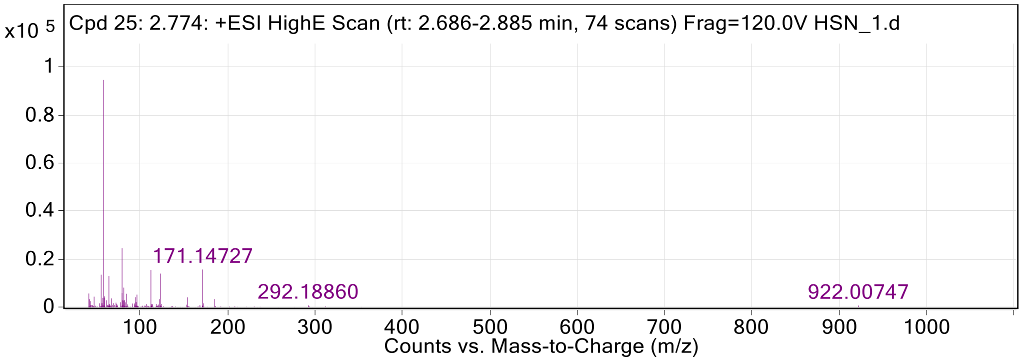

Peak List

m/z	z	Abund
41.03828		5621.1
55.0538		13464.3
58.06477	1	94572.35
63.9971		12937.93
79.02033	1	24505.99
79.05223		5847.42
81.06876		8072.89
112.07414	1	15448.76
123.11515	1	13921.95
171.14727	1	15613.49

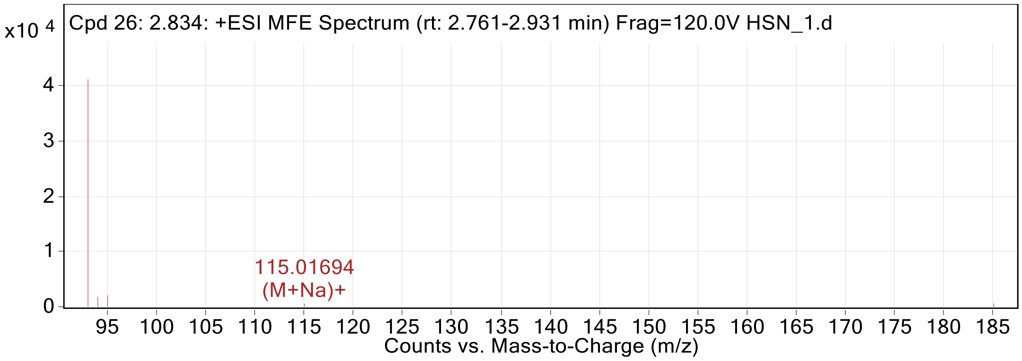

Peak List

m/z	z	Abund	Ion
93.03575	1	41199.48	(M+H)+

94.03866	1	1836.4	(M+H)+
95.03182	1	2082.48	(M+H)+
115.01694	1	578.01	(M+Na)+
185.06482	1	610.28	(2M+H)+

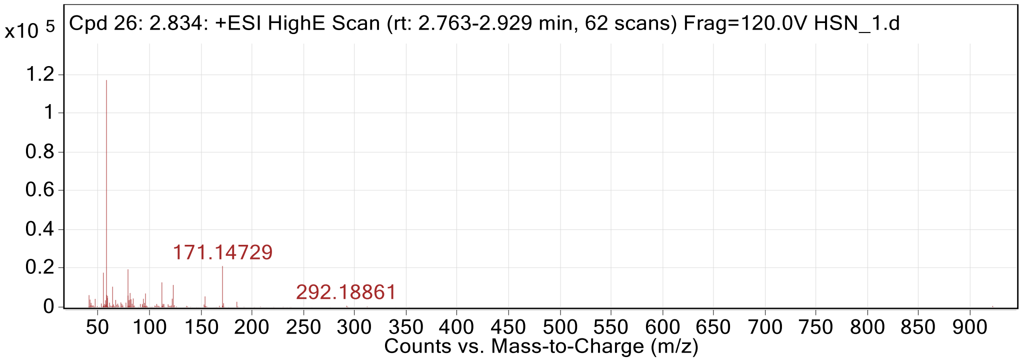

Peak List

m/z	z	Abund
55.05382		17736.01
58.0648	1	117055
58.13195		6271.77
63.99708		10511.93
79.02035	1	19463.38
81.06876		7264.85
96.07971	1	7005.59
112.07416	1	12745.73
123.11521	1	11405.31
171.14729	1	21171.39

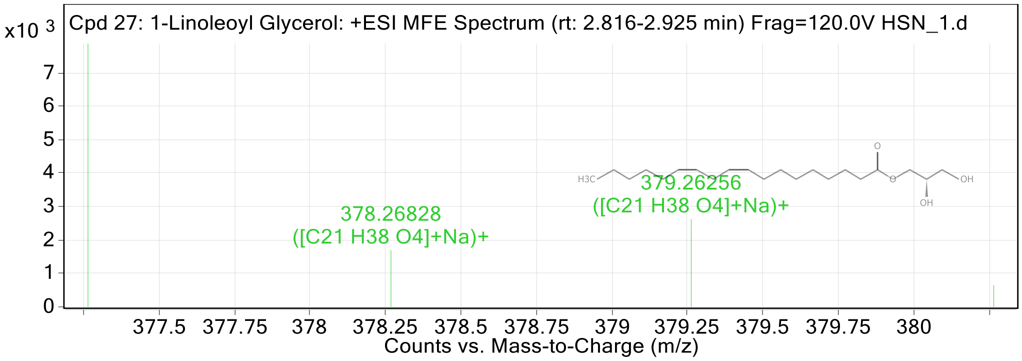

Peak List

m/z	z	Abund	Name	Formula	Ion	Score (DB)	Hits (DB)
377.26481	1	7874.65	1-Linoleoyl Glycerol	C21 H38 O4	(M+Na)+	35.62	8
378.26828	1	1691.82		C21 H38 O4	(M+Na)+		
379.26256	1	2623.71		C21 H38 O4	(M+Na)+		
380.26475	1	649.46		C21 H38 O4	(M+Na)+		

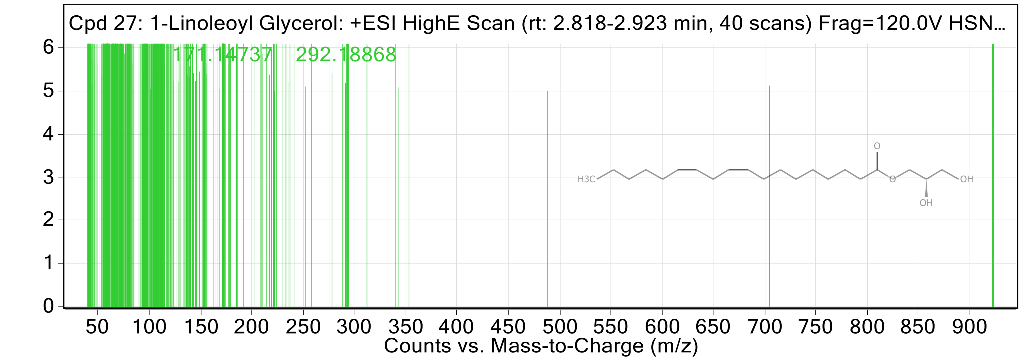

Peak List

m/z	z	Abund
55.05386		23536.96
58.06486	1	142345.28
58.13198		8127.18
59.04874		7937.52
63.99709		9509.74
79.02039	1	17334.74
79.05297		6350.51
96.07971		9631.33
154.12076	1	7604.49
171.14737	1	30054.93

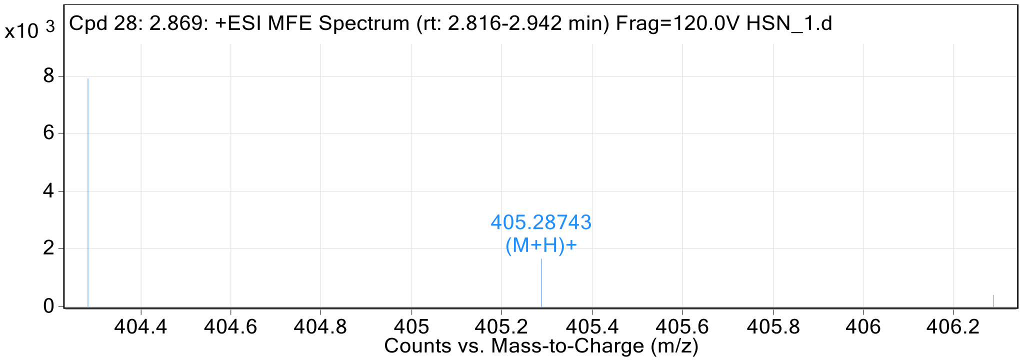

Peak List

m/z	z	Abund	Ion
404.28388	1	7905.27	(M+H)+
405.28743	1	1665.67	(M+H)+
406.28824	1	406.52	(M+H)+

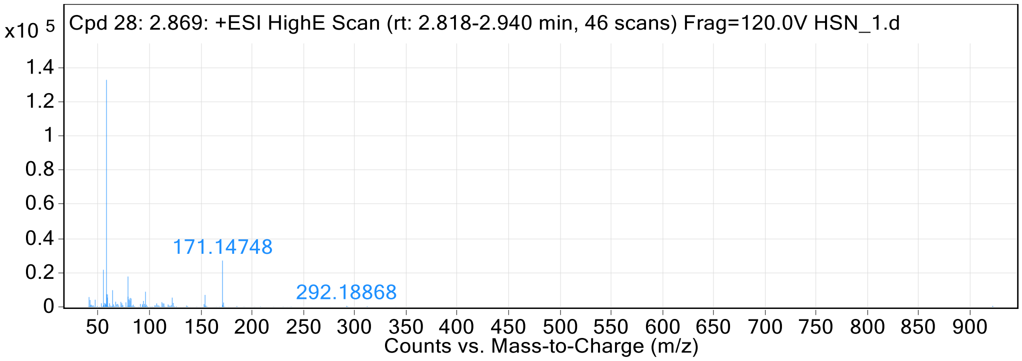

Peak List

m/z	z	Abund
55.0539		21708.96
58.06489	1	132690.8

58.13201		7428.71
59.04878		7314.09
63.99713		9772.05
79.02045	1	17750.28
79.05298		5972.4
96.07978		8863.79
154.12087	1	7009.29
171.14748	1	27082.46

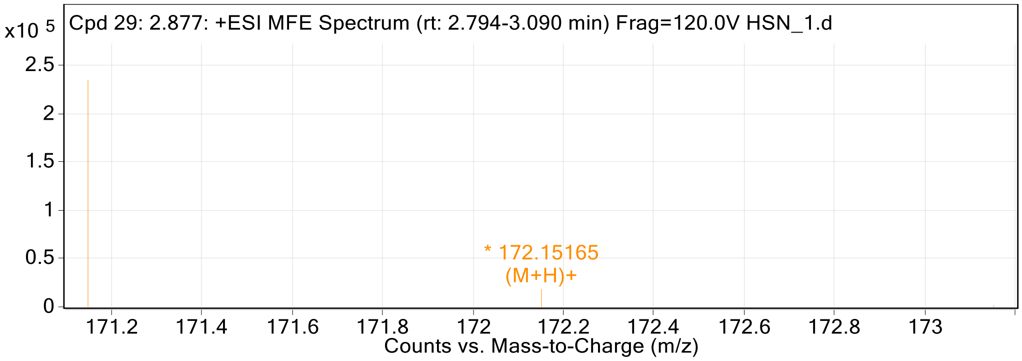

Peak List

m/z	z	Abund	Ion
171.1483	1	235226.31	(M+H)+
172.15165	1	18487.8	(M+H)+
173.15256	1	1464.57	(M+H)+

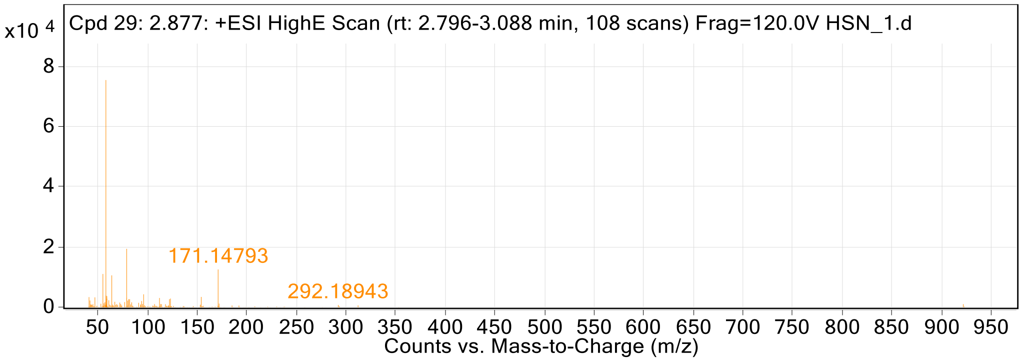

Peak List

m/z	z	Abund
41.03859		3312.1
55.0542		10992.51
58.06516	1	75392.74
58.13232		3793.85
59.04909		3651.39
63.99748		10529.98
79.02078	1	19346.2
96.08019		4243.21
154.12133	1	3372.15
171.14793	1	12492.37

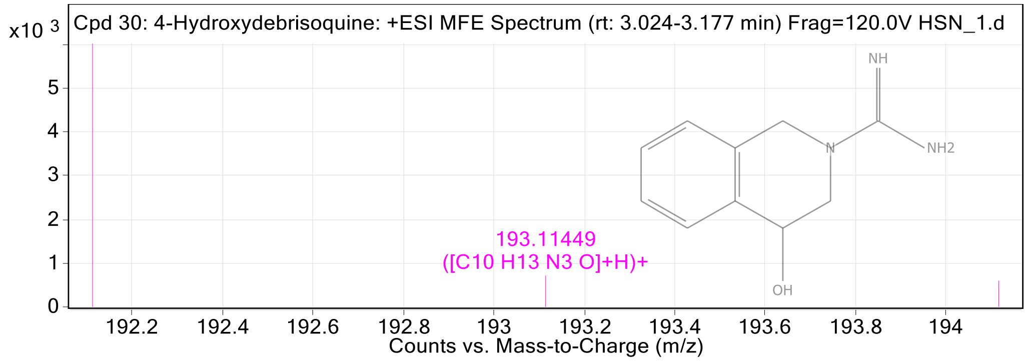

Peak List

m/z	z	Abund	Name	Formula	Ion	Score (DB)	Hits (DB)
192.11227	1	6027.6	4-Hydroxydebrisoquine	C10 H13 N3 O	(M+H)+	76.16	3
193.11449	1	716.43		C10 H13 N3 O	(M+H)+		
194.11677	1	598.35		C10 H13 N3 O	(M+H)+		

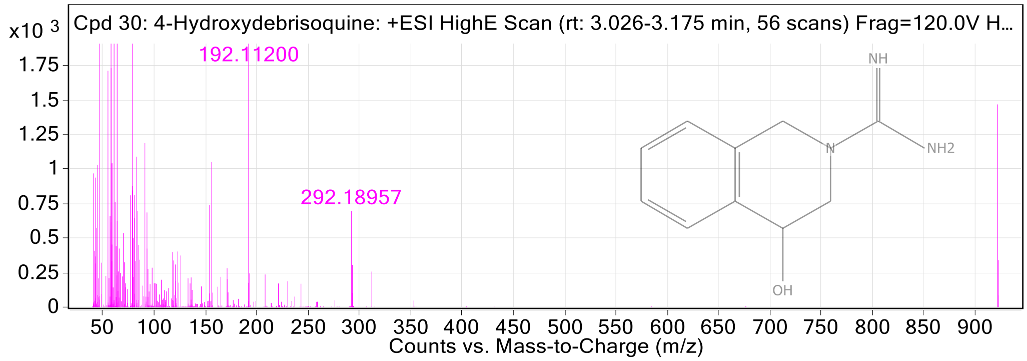

Peak List

m/z	z	Abund
46.9952		2040.61
55.0542		1712.45
58.0651	1	32009.48
58.08433		1731.91
61.01055		2133.63
63.99759		10374.89
79.0208	1	18511.88
91.05362		1187.73
192.112	1	1909.42
922.0089	1	1468.82

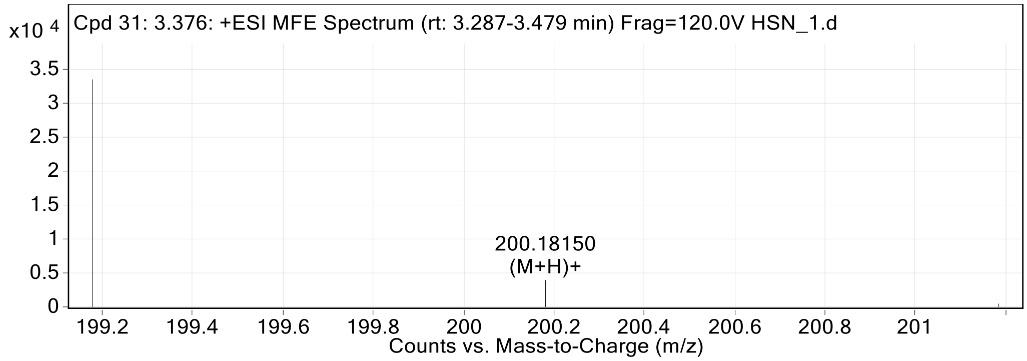

Peak List

m/z	z	Abund	Ion
199.1786	1	33567.16	(M+H)+
200.1815	1	3956	(M+H)+

201.18481	1	479.07	(M+H)+

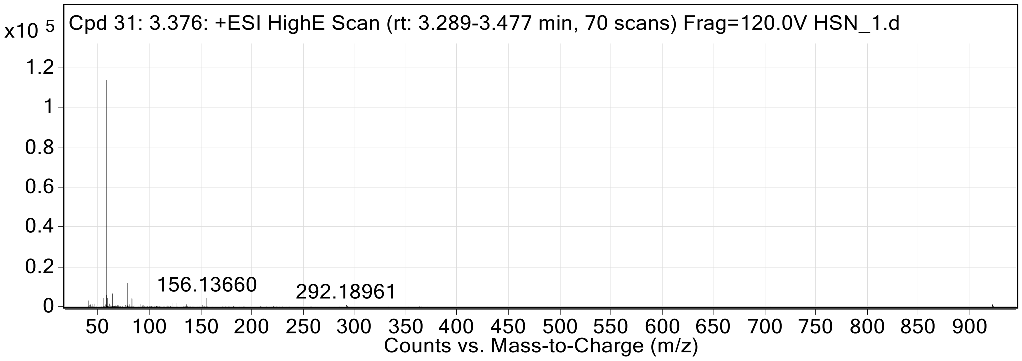

Peak List

m/z	z	Abund
41.03841		3042.87
55.0539	1	4233.01
58.06487	1	114071.8
58.13202		5958.47
59.06843	1	4239.01
63.99722		6568.71
79.02047	1	11923.71
83.04832	1	4113.55
84.07993	1	3981.51
156.1366	1	4201.16

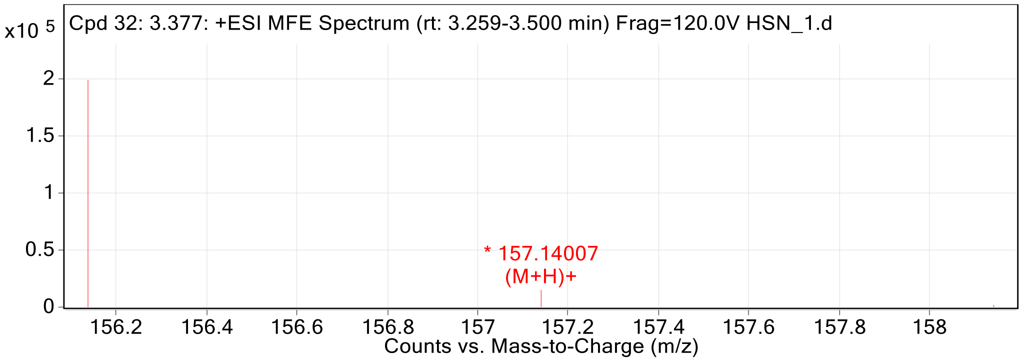

Peak List

m/z	z	Abund	Ion
156.13695	1	198811.34	(M+H)+
157.14007	1	15004.71	(M+H)+
158.14203	1	1760.92	(M+H)+

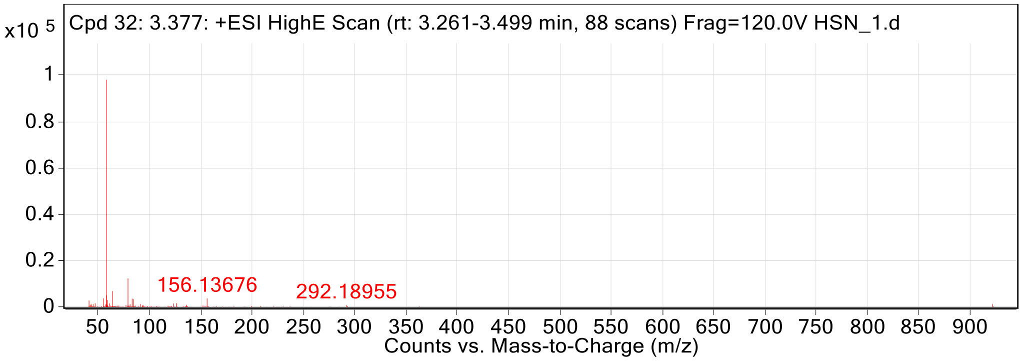

Peak List

m/z	z	Abund
41.03851		2698.88

55.05401	1	3674.5
58.06498	1	98044.98
58.13214		4997.69
59.06835	1	2945.52
63.99734		6786.18
79.02061	1	12274.34
83.04845	1	3512.09
84.08008	1	3312.22
156.13676	1	3583.93

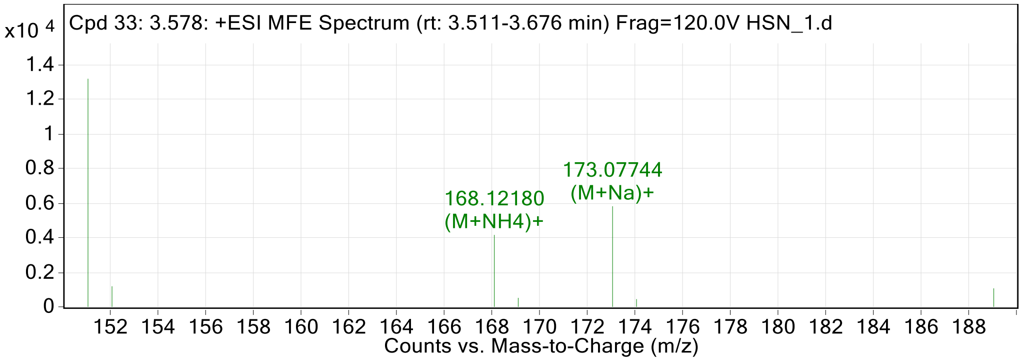

Peak List

m/z	z	Abund	Ion
151.09545	1	13192.04	(M+H)+
152.10194	1	1189.62	(M+H)+
168.1218	1	4159.22	(M+NH4)+
169.12337	1	517.67	(M+NH4)+
173.07744	1	5816.59	(M+Na)+
174.08041	1	445.35	(M+Na)+
189.05146	1	1069.54	(M+K)+

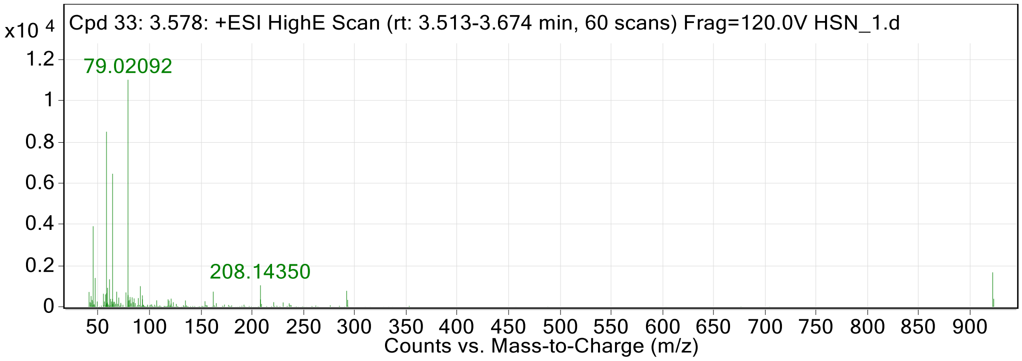

Peak List

m/z	z	Abund
45.03367		3909.61
46.99513		1398.89
58.0652	1	8497.01
59.07259		918.88
61.01073		1333.71
63.99765	1	6457.04
79.02092	1	11019.55
91.05362		999.96
208.1435	1	1042.92
922.00955	1	1670.61

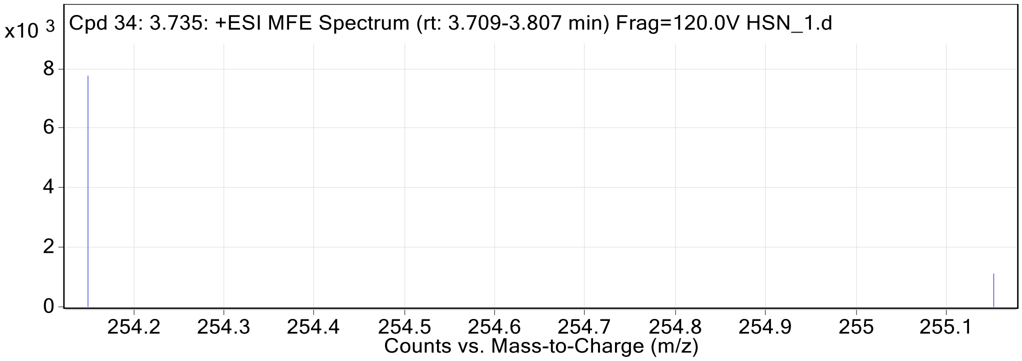

Peak List

m/z	z	Abund	Ion
254.1494	1	7761.16	(M+H)+
255.15276	1	1117.03	(M+H)+

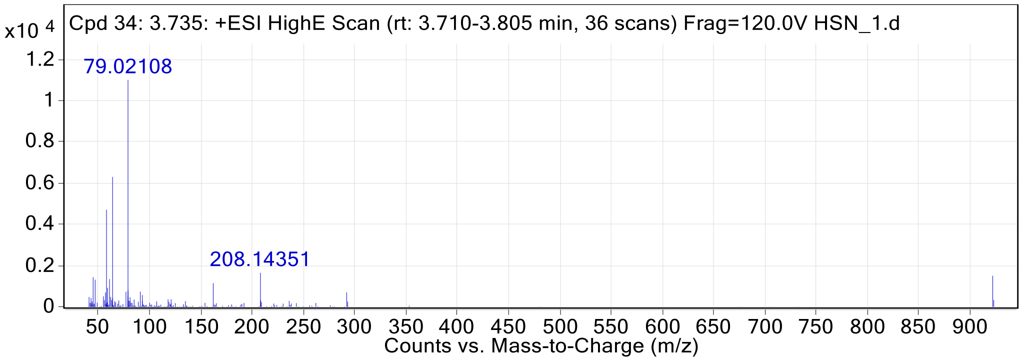

Peak List

m/z	z	Abund
45.03379		1430.83
46.99526		1311.94
58.06522		4708.68
59.07269		914.35
61.01063		1339.46
63.99777		6298.72
79.02108	1	11006.35
162.1016	1	1143.39
208.14351	1	1645.55
922.01125	1	1500.94

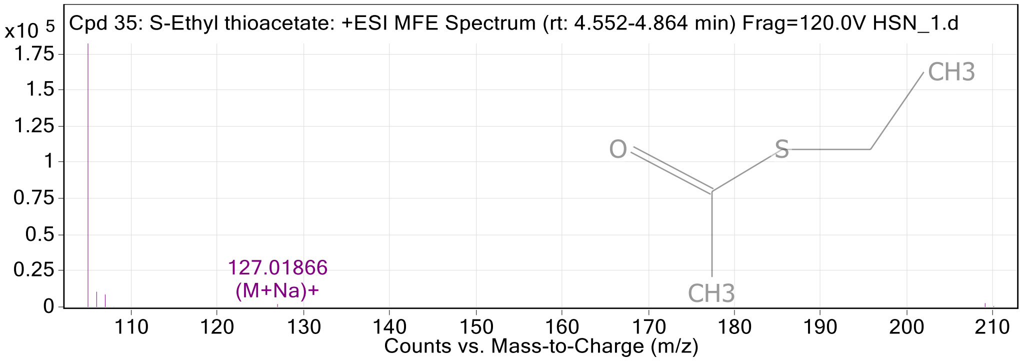

Peak List

m/z	z	Abund	Name	Formula	Ion	Score (DB)	Hits (DB)
105.03644	1	182069.22	S-Ethyl thioacetate	C4 H8 O S	(M+H)+	98.15	7
106.04014	1	10542.91		C4 H8 O S	(M+H)+		
107.03296	1	8619.58		C4 H8 O S	(M+H)+		

108.03599	1	79.71		C4 H8 O S	(M+H)+		
127.01866	1	1995.58			(M+Na)+		
209.06648	1	2653.77			(2M+H)+		
210.06833	1	581.14			(2M+H)+		

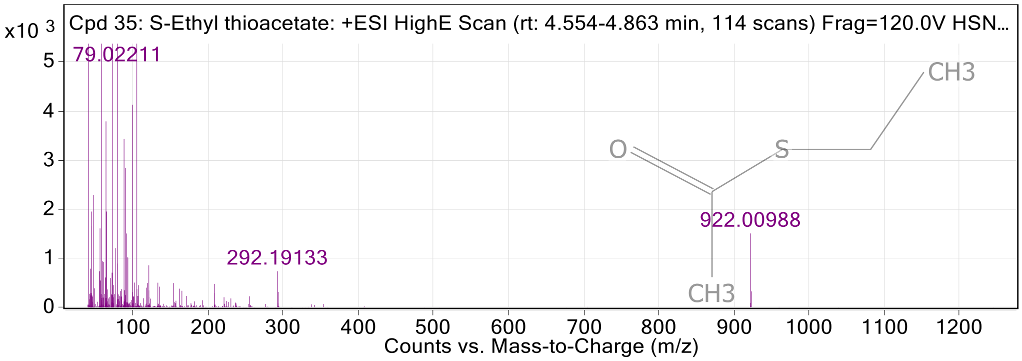

Peak List

m/z	z	Abund
41.03966	1	5544.4
46.99604		2285.58
58.06624		5878.05
63.99872		3787.38
73.01159	1	6022.7
79.02211	1	6316.47
88.03499	1	3427.37
90.01414		2836.13
99.09229	1	4127.29
105.03745	1	5372.37

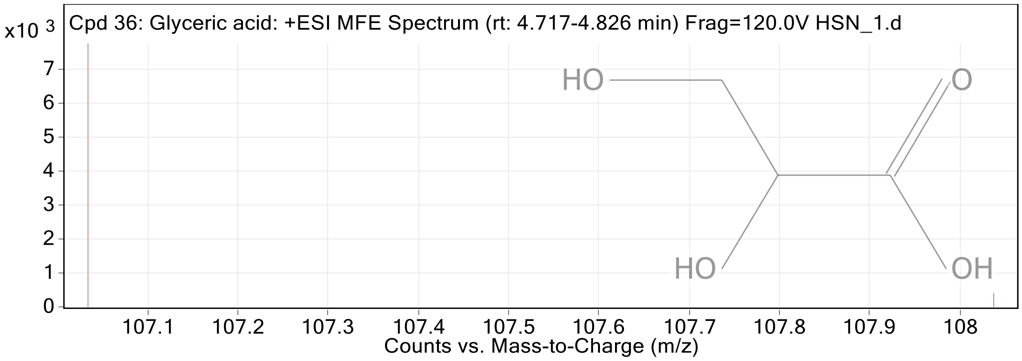

Peak List

m/z	z	Abund	Name	Formula	Ion	Score (DB)	Hits (DB)
107.03401	1	7774.27	Glyceric acid	C3 H6 O4	(M+H)+	86.21	2
108.03762	1	411.27		C3 H6 O4	(M+H)+		

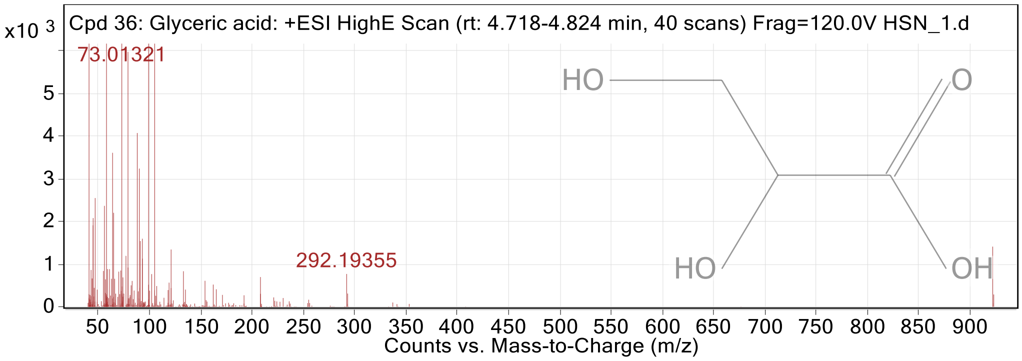

Peak List

m/z	z	Abund
41.04095	1	6450.98
46.99742		2550.26
58.06772		7049.04
64.00029		3611.96
73.01321	1	7139.14
79.02377	1	5979.37
88.03679	1	4076.43
90.01588		3242.15
99.09405	1	6610.47
105.03926	1	6173.69

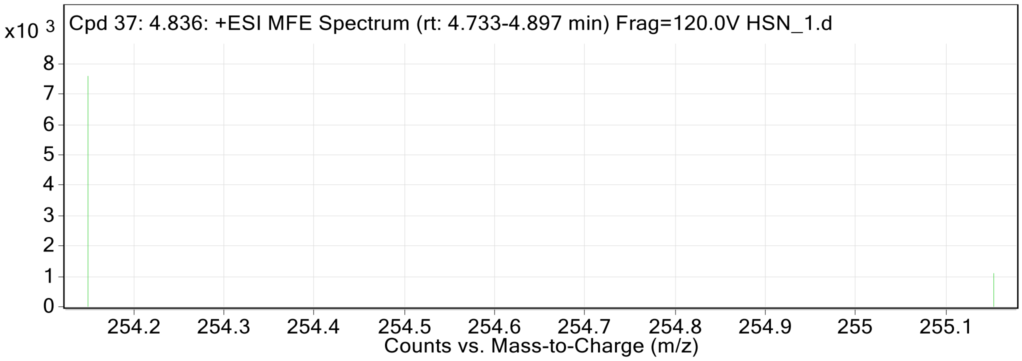

Peak List

m/z	z	Abund	Ion
254.14965	1	7604.7	(M+H)+
255.153	1	1106.27	(M+H)+

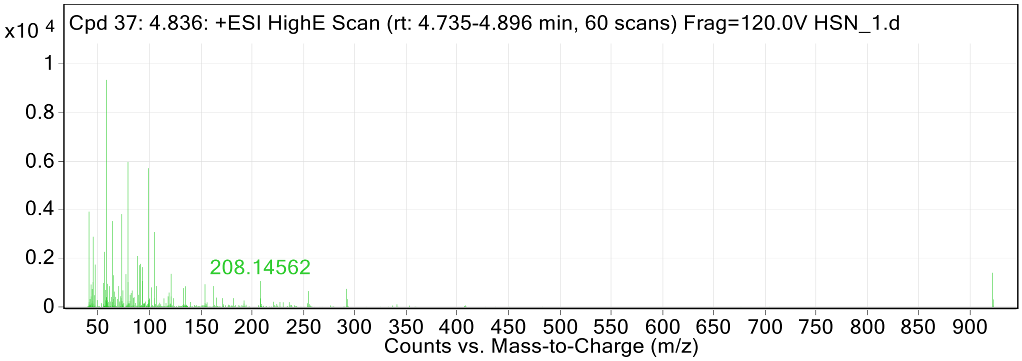

Peak List

m/z	z	Abund
41.04011		3920.8
45.03507		2890.09
56.05125		2270.16
58.06676		9335.46
63.99932		3533.82
73.01221		3811.94
79.02268		5968.42
88.03573		2101.48
99.09292	1	5700.34
105.03804		3089.7

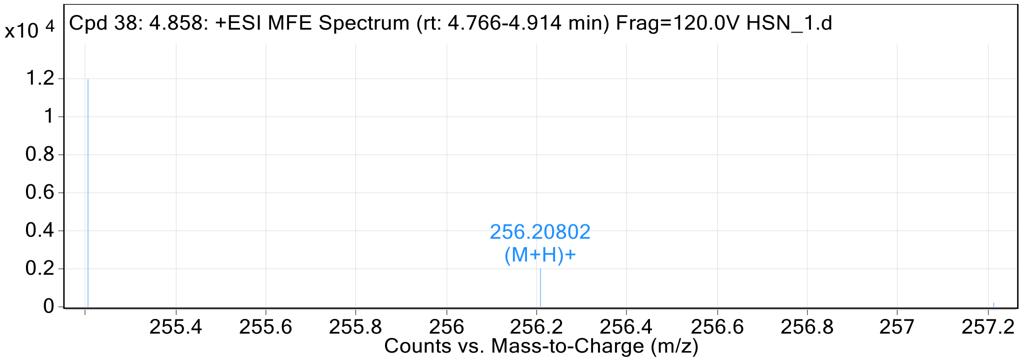

Peak List

m/z	z	Abund	Ion
255.20525	1	11985.6	(M+H)+
256.20802	1	2031	(M+H)+
257.21253	1	220.03	(M+H)+

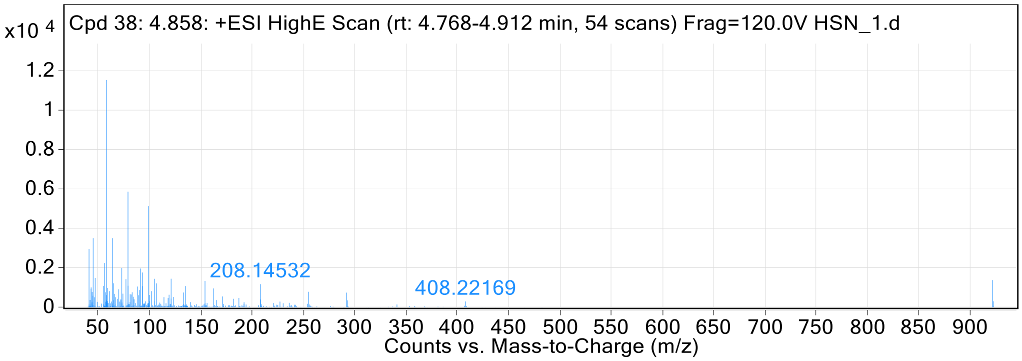

Peak List

m/z	z	Abund
41.0402		2951.31
45.03513		3495.5
56.05127		2242.02
58.06681	1	11523.83
63.99935		3491.44
73.01229		1991.77
79.02269		5858.44
91.05561		1955.33
93.0461		1753.87
99.09294	1	5120.13

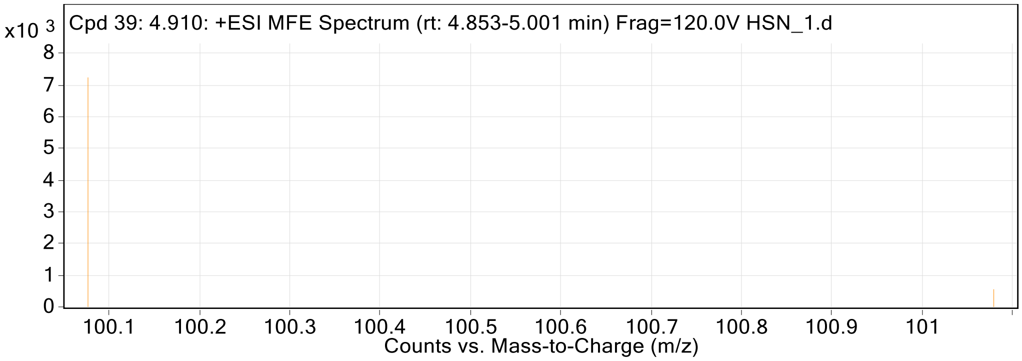

Peak List

m/z	z	Abund	Ion
100.07635	1	7246.19	(M+H)+
101.07938	1	557.37	(M+H)+

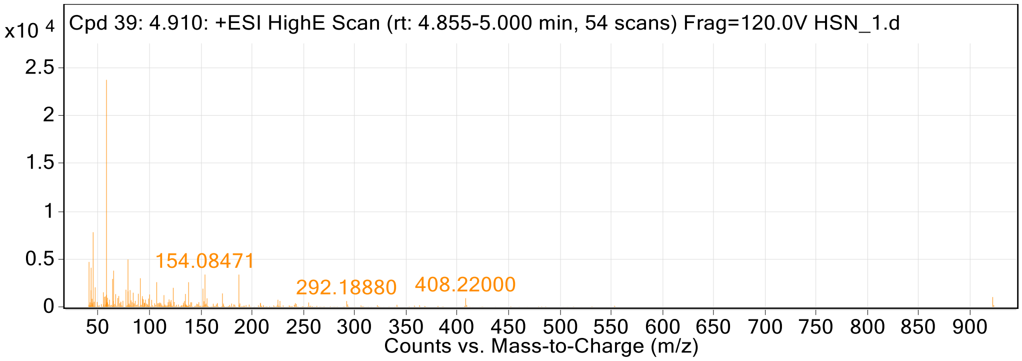

Peak List

m/z	z	Abund
41.03857		4701.4
43.05424		4098.75
45.03352		7813.15
58.06497	1	23752.27
63.99747		2922.82
65.00525		3790.51
79.02065		4962.21
91.05361		2998.1
154.08471	1	3381.96
187.14275	1	3377.51

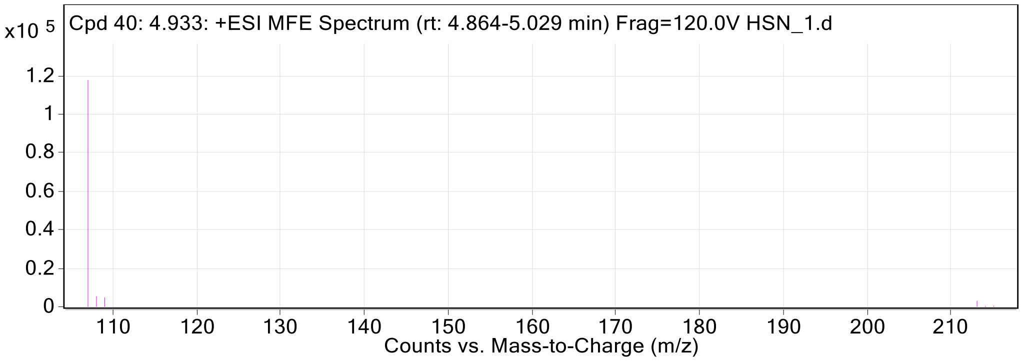

Peak List

m/z	z	Abund	Ion
107.05084	1	117679.05	(M+H)+
108.05454	1	5470.16	(M+H)+
109.04651	1	4854.28	(M+H)+
110.05001	1	94.54	(M+H)+
213.09679	1	3070.87	(2M+H)+
214.09982	1	442.61	(2M+H)+
215.09005	1	457.93	(2M+H)+

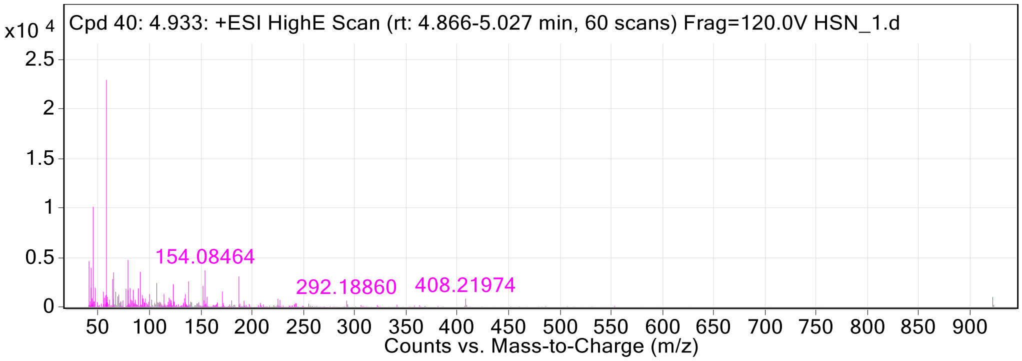

Peak List

m/z	z	Abund
41.03853		4612.14
43.05417		3956.57
45.03345		10101.92
58.06492	1	22945.48
63.99737		2823.76
65.00518		3499.57
79.02055		4755.06
91.05348		3541.29
154.08464	1	3696.3
187.14262		3089.67

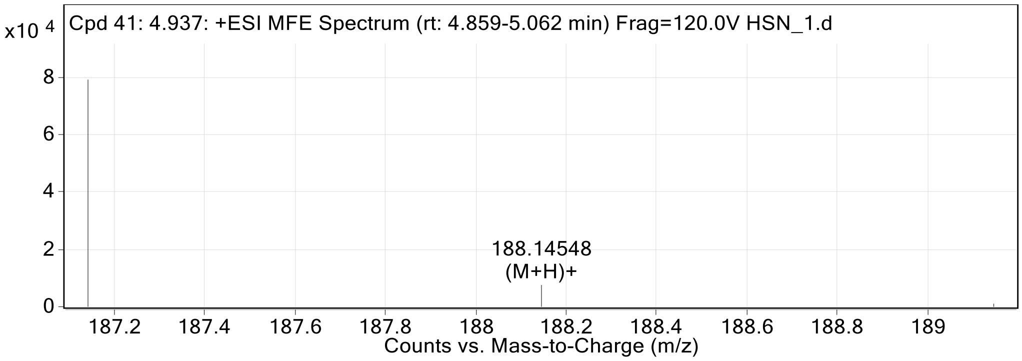

Peak List

m/z	z	Abund	Ion
187.14152	1	79141.5	(M+H)+
188.14548	1	7585.72	(M+H)+
189.14687	1	1064.14	(M+H)+

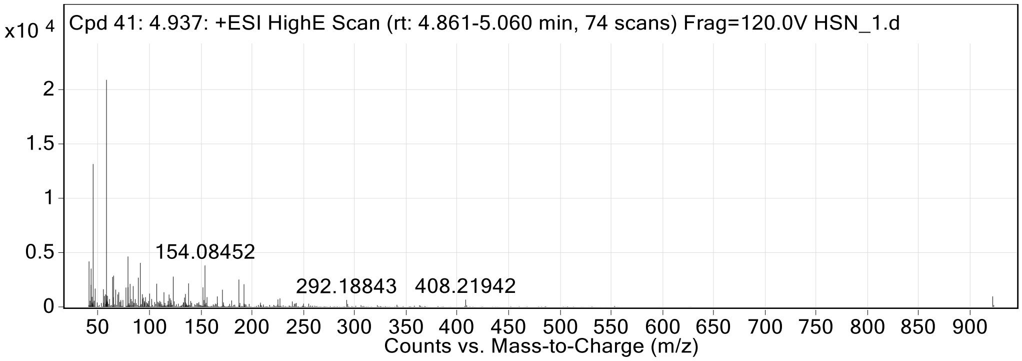

Peak List

m/z	z	Abund
41.03846		4194.78
43.05409		3533.11

45.03338		13175.91
58.06483	1	20934.39
63.99725		2763.06
65.00509		2884.12
79.02049		4646.23
91.05337		4058.85
123.11533		2793.5
154.08452	1	3822.58

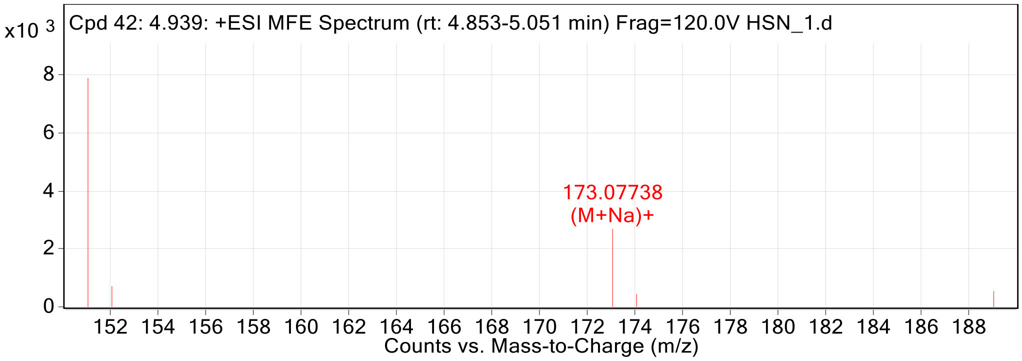

Peak List

m/z	z	Abund	Ion
151.09504	1	7884.86	(M+H)+
152.09828	1	714.32	(M+H)+
173.07738	1	2688.47	(M+Na)+
174.08111	1	441.63	(M+Na)+
189.05028	1	540.64	(M+K)+

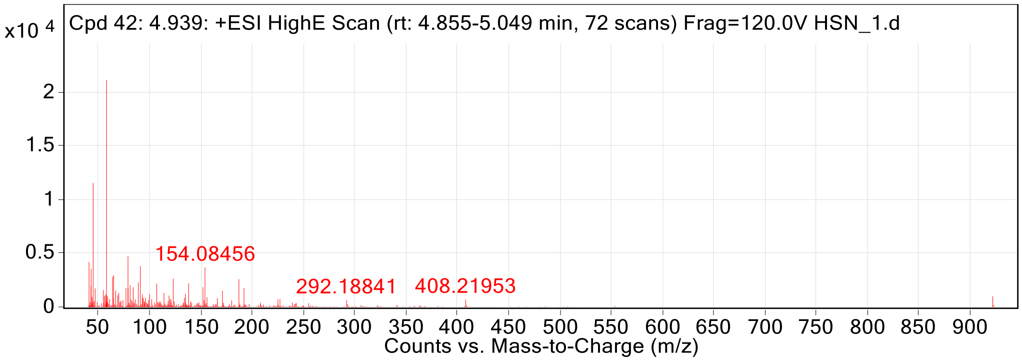

Peak List

m/z	z	Abund
41.03847		4194.26
43.05411		3539.33
45.0334		11536.8
58.06485	1	21124.71
63.99729		2800.13
65.00511		2957.33
79.0205		4727.79
91.0534		3798.47
123.11536		2633.71
154.08456	1	3676.58

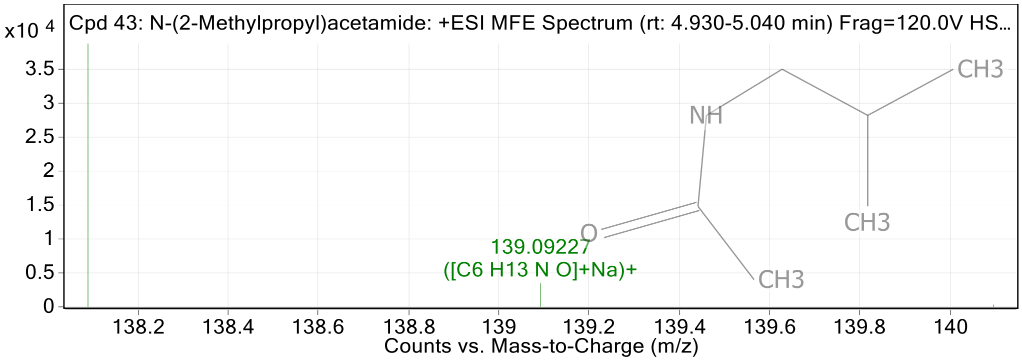

Peak List

m/z	z	Abund	Name	Formula	Ion	Score (DB)	Hits (DB)
138.0895	1	38891.73	N-(2-Methylpropyl)acetamide	C6 H13 N O	(M+Na)+	96.31	1
139.09227	1	3490.34		C6 H13 N O	(M+Na)+		
140.09663	1	335.28		C6 H13 N O	(M+Na)+		

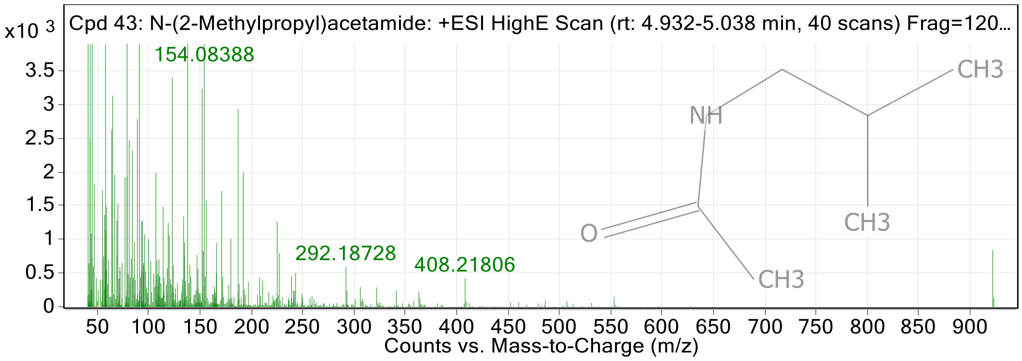

Peak List

m/z	z	Abund
41.03819		4790.1
43.05375		3893.04
45.03305		14250.94
58.06443	1	23526.73
79.01996		4409.1
91.05281	1	4554.94
123.11475		3398.95
138.089		3904.52
152.10465		3239.99
154.08388	1	4169.16

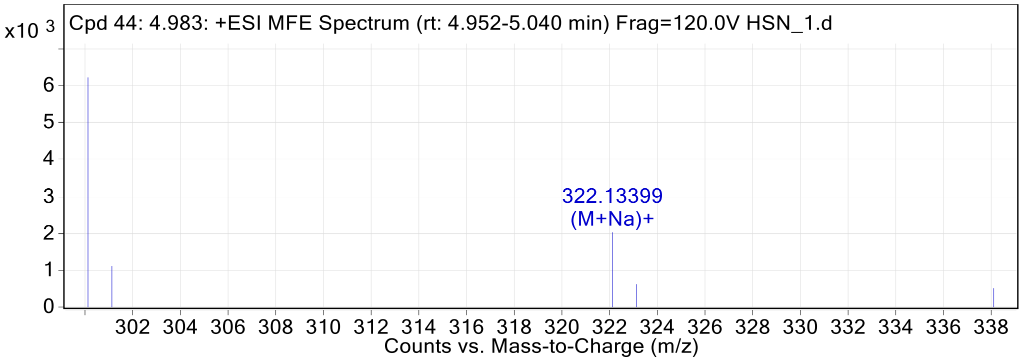

Peak List

m/z	z	Abund	Ion
300.15269	1	6231.1	(M+H)+

301.15477	1	1113.88	(M+H)+
322.13399	1	2025.22	(M+Na)+
323.1409	1	620.27	(M+Na)+
338.10751	1	511.76	(M+K)+

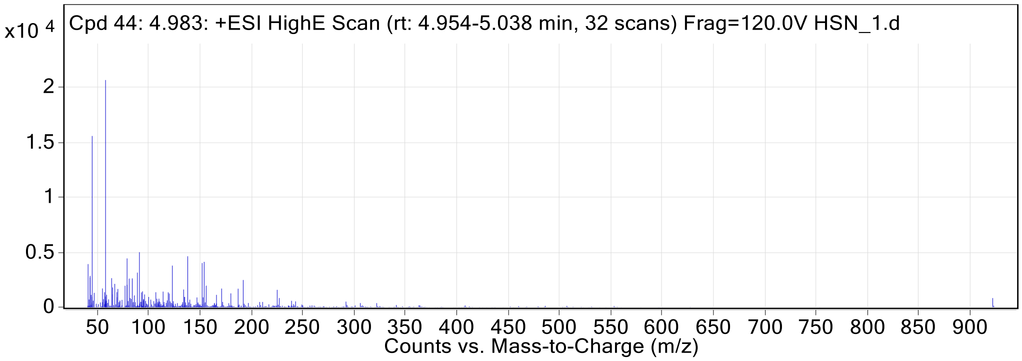

Peak List

m/z	z	Abund
41.03821		3907.64
45.03306		15554.48
58.06445	1	20639.54
79.01998		4422.85
89.05842		3132.69
91.05283	1	4991.52
123.1148	1	3770.15
138.08903		4616.31
152.10469		4004.5
154.08392	1	4112.01

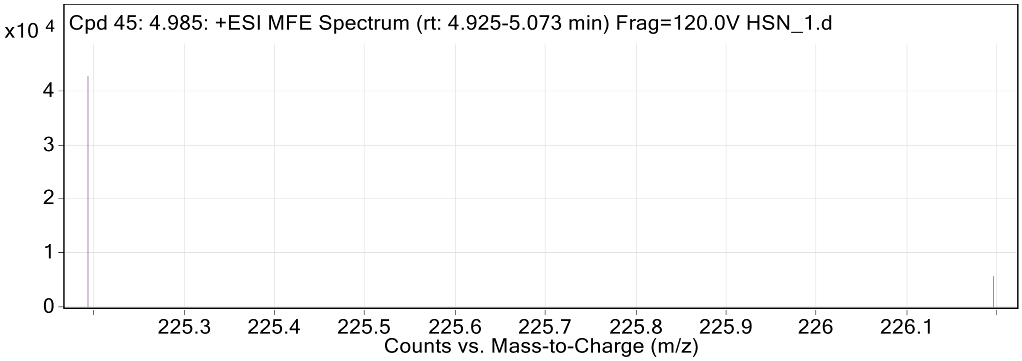

Peak List

m/z	z	Abund	Ion
225.19384	1	42743.68	(M+H)+
226.19628	1	5651.58	(M+H)+

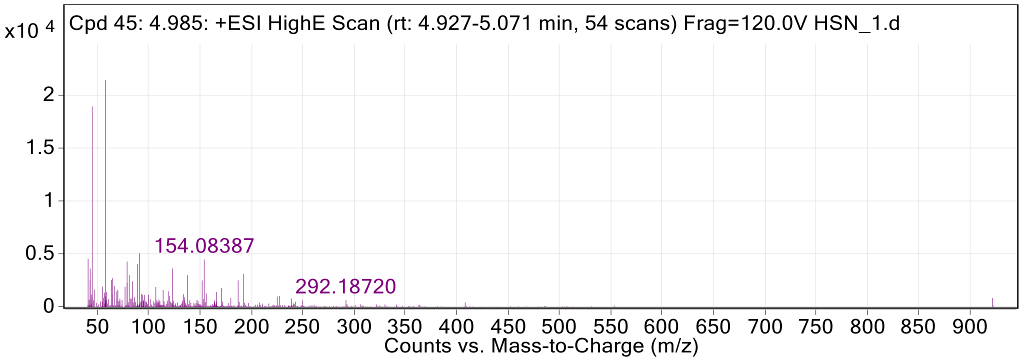

Peak List

m/z	z	Abund
41.03814		4531.47
43.05374		3626.49
45.03304		18970.98
58.06444	1	21473.53
79.01998		4276.58
89.05842		4055.68
91.05279	1	5062.36
123.11467	1	3622.16
154.08387		4482.87
192.11057		3113.59

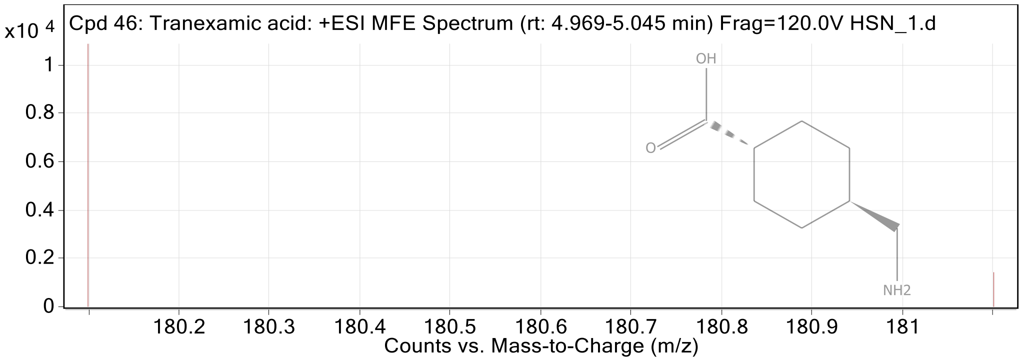

Peak List

m/z	z	Abund	Name	Formula	Ion	Score (DB)	Hits (DB)
180.09929	1	10881.59	Tranexamic acid	C8 H15 N O2	(M+Na)+	80.37	2
181.10139	1	1428.2		C8 H15 N O2	(M+Na)+		

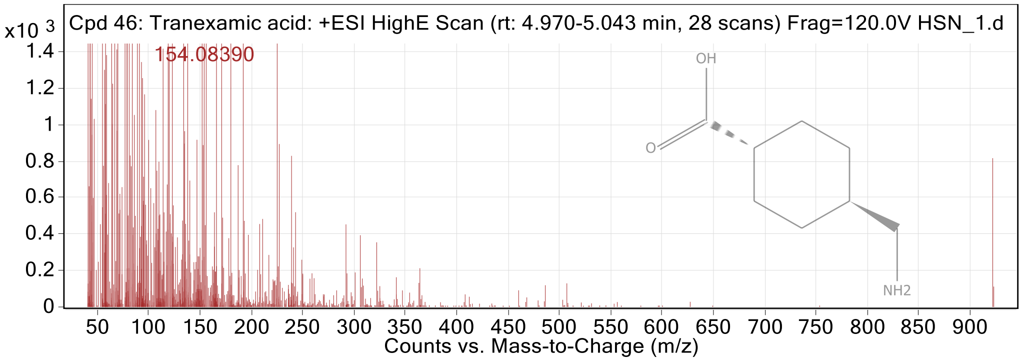

Peak List

m/z	z	Abund
45.03303		17522.05
58.06445		17799.55
79.01997		4314.15
89.0584		3659.47
91.05279		5420.83
123.11476	1	4268.59
138.08894		3890.59
152.10464		4122.19
154.0839	1	4277.58
192.11071	1	3646.34

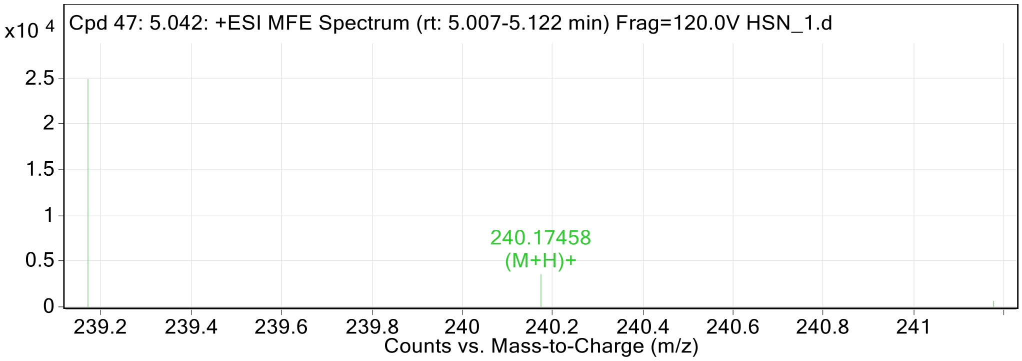

Peak List

m/z	z	Abund	Ion
239.17113	1	24919.39	(M+H)+
240.17458	1	3586.98	(M+H)+
241.17756	1	647.71	(M+H)+

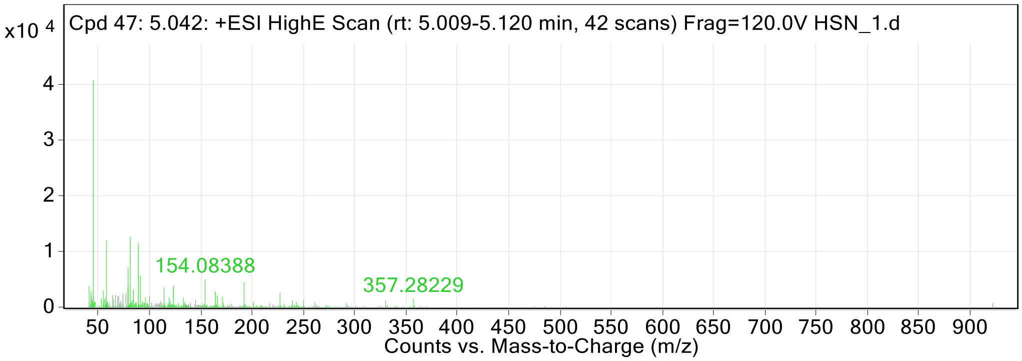

Peak List

m/z	z	Abund
41.03814		3759.32
45.03307	1	40746.34
58.06448		11949.69
79.05295		7128.01
81.06867	1	12633.95
89.05834		11405.49
91.05276		5677.75
123.11469		3755.38
154.08388		4990.63
192.11046		4484.79

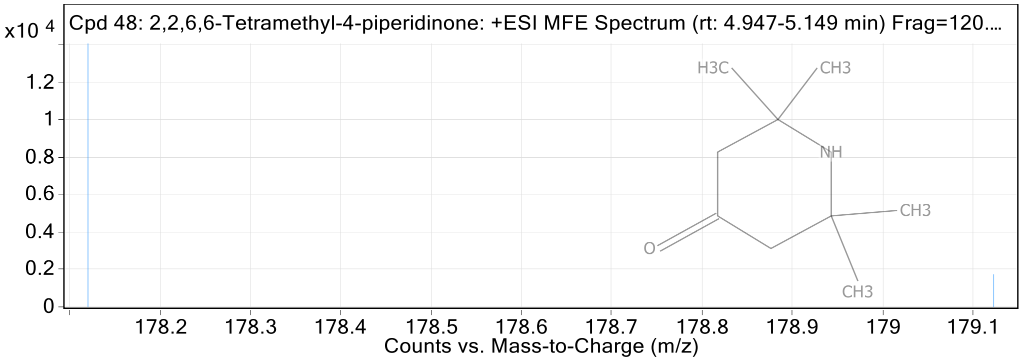

Peak List

m/z	z	Abund	Name	Formula	Ion	Score (DB)	Hits (DB)
178.12015	1	14080.71	2,2,6,6-Tetramethyl-4-piperidinone	C9 H17 N O	(M+Na)+	86.26	4

179.12307	1	1740.41		C9 H17 N O	(M+Na)+		

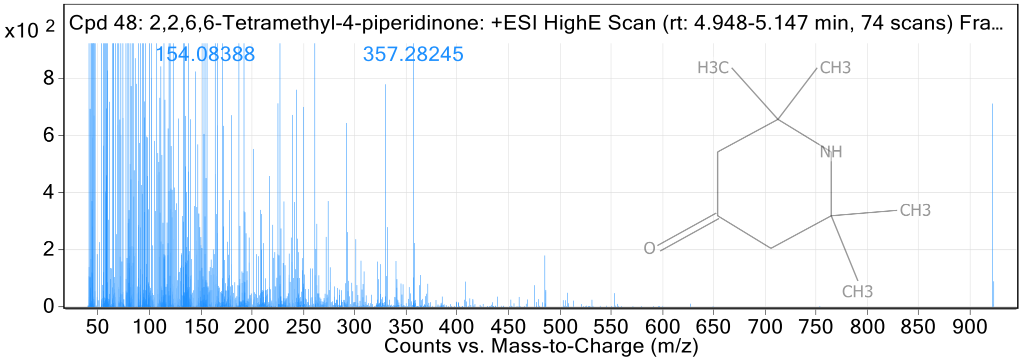

Peak List

m/z	z	Abund
41.03817		4001.13
45.0331	1	43004.3
58.06444	1	15691.42
79.02005		3846.34
79.05293		5314.08
81.06868	1	9031.82
89.05838	1	13649.1
91.05279	1	4827.7
123.11474		3405.23
154.08388		4472.94

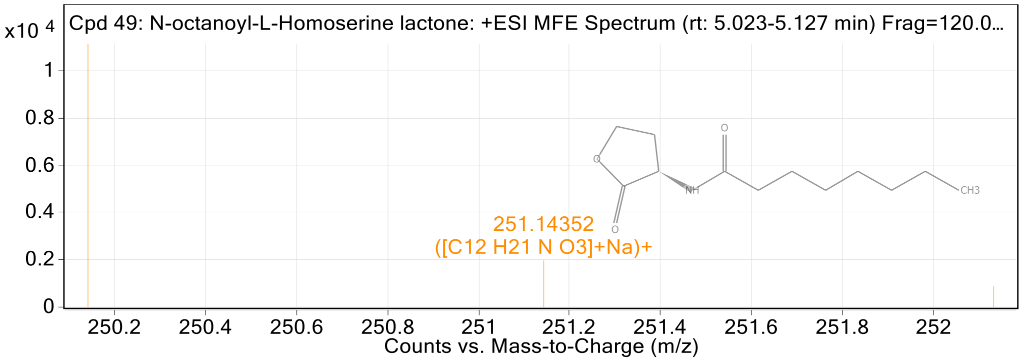

Peak List

m/z	z	Abund	Name	Formula	Ion	Score (DB)	Hits (DB)
250.14097	1	11155.8	N-octanoyl-L-Homoserine lactone	C12 H21 N O3	(M+Na)+	70.64	1
251.14352	1	1971.44		C12 H21 N O3	(M+Na)+		
252.13312	1	879.86		C12 H21 N O3	(M+Na)+		

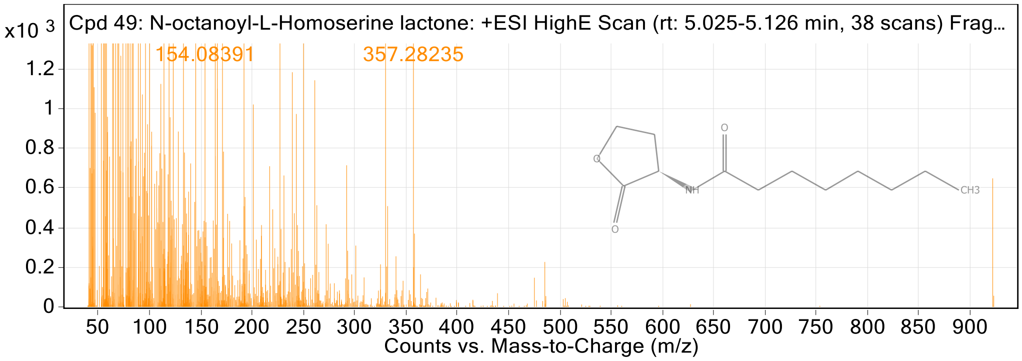

Peak List

m/z	z	Abund

41.03813		3909.62
45.03307	1	47055.76
58.06448		11484.87
79.05297		7975.93
81.06867	1	14266.05
89.05833	1	14061.38
91.05277	1	5528.83
114.08935		4050.18
154.08391		5039.06
192.11044	1	4598.72

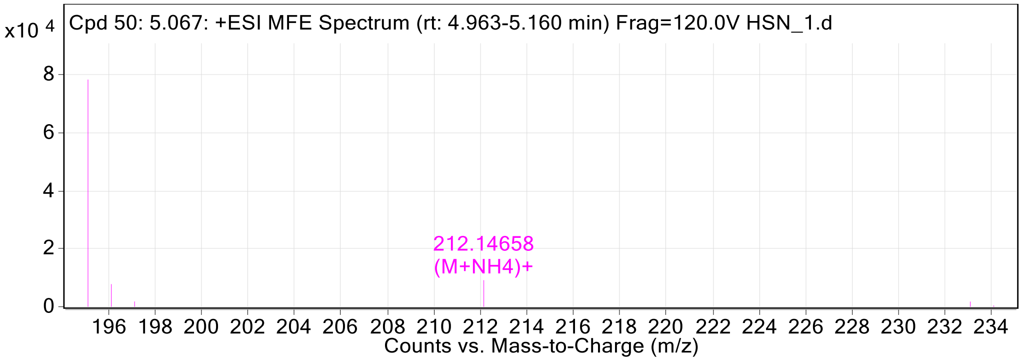

Peak List

m/z	z	Abund	Ion
195.11988	1	78319.02	(M+H)+
196.12389	1	7807.36	(M+H)+
197.12489	1	1823.61	(M+H)+
212.14658	1	9167.41	(M+NH4)+
233.07599	1	1783.21	(M+K)+
234.08502	1	437.59	(M+K)+

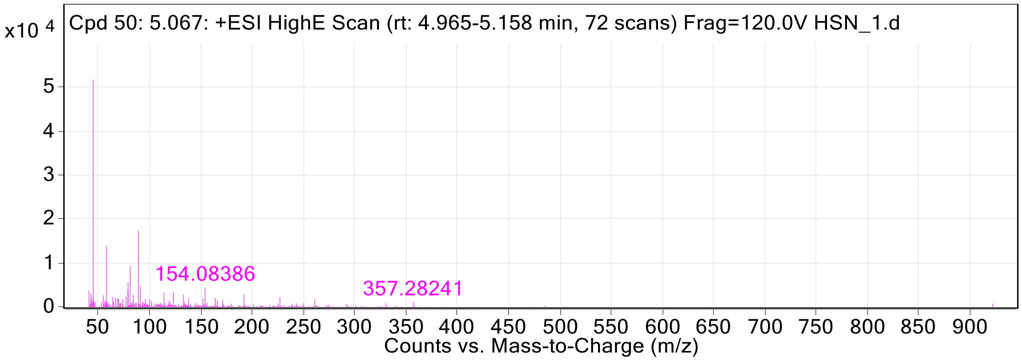

Peak List

m/z	z	Abund
41.03817		3655.43
45.03311	1	51687.63
58.06444	1	13847.5
79.02005		3774.48
79.05291		5511.15
81.06867	1	9352.58
89.05838	1	17325.64
91.05279	1	4818.58
123.11471		3402.9
154.08386		4397.06

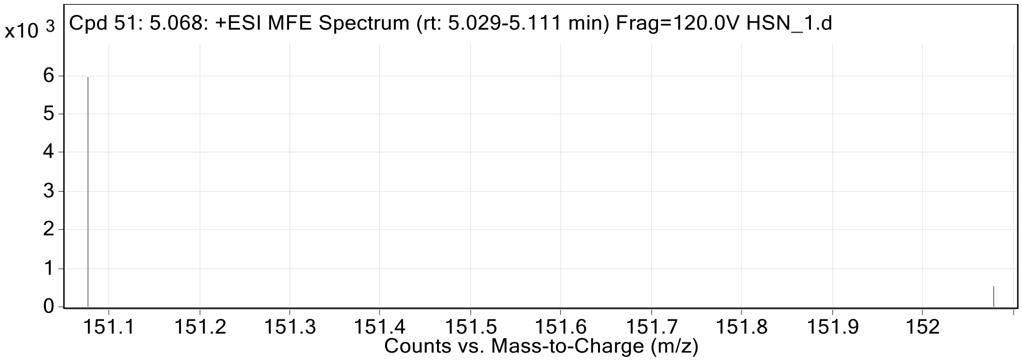

Peak List

m/z	z	Abund	Ion
151.0767	1	5960.68	(M+H)+
152.07868	1	541.03	(M+H)+

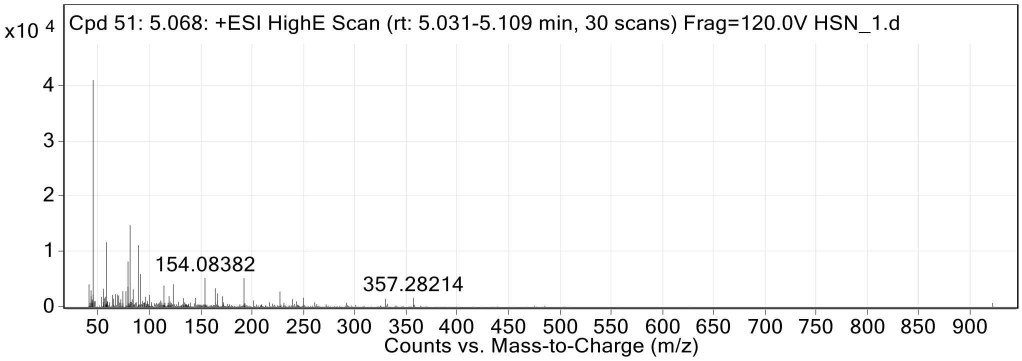

Peak List

m/z	z	Abund
41.03813		4042.01
45.03305	1	40968.52
58.06446		11664.08
79.05293	1	8154.7
81.06866	1	14741.86
89.05832		11101.58
91.05274		5897.34
123.11463		4046.16
154.08382		5187.17
192.11047	1	5163.14

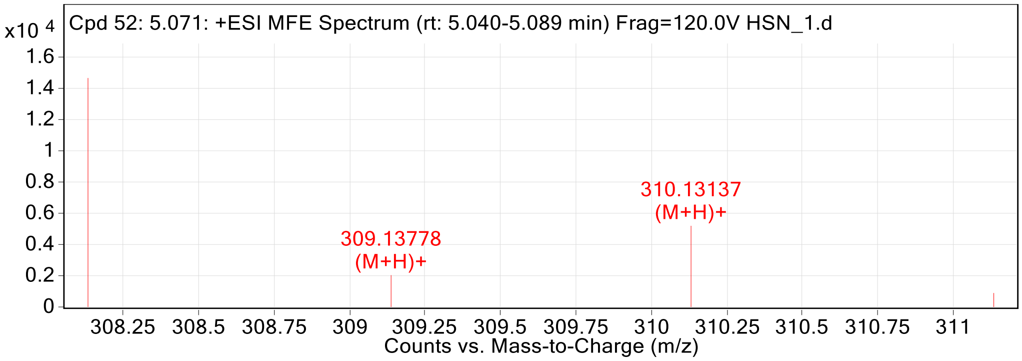

Peak List

m/z	z	Abund	Ion
308.13376	1	14648.15	(M+H)+
309.13778	1	2034.04	(M+H)+
310.13137	1	5192.27	(M+H)+

311.1342	1	892.85	(M+H)+

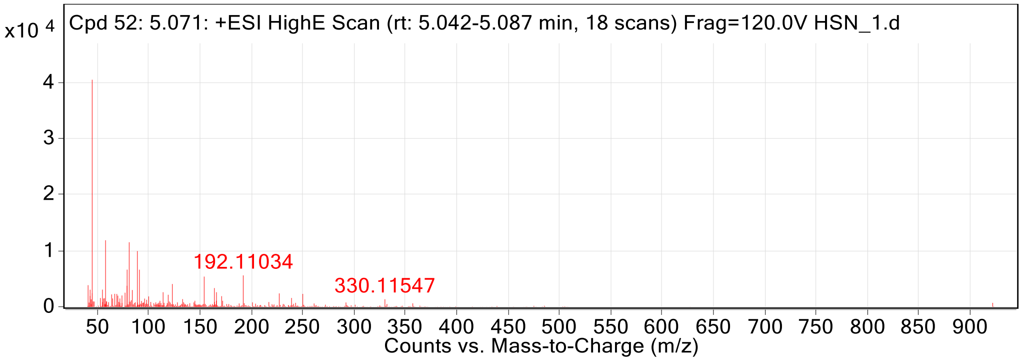

Peak List

m/z	z	Abund
41.03811		3854.26
45.03302	1	40519.41
58.06447		11869.68
79.05286		6629.07
81.06871	1	11515.5
89.05834		9939.92
91.05274	1	6616.63
123.11443		4062.11
154.08386		5384.1
192.11034	1	5599.34

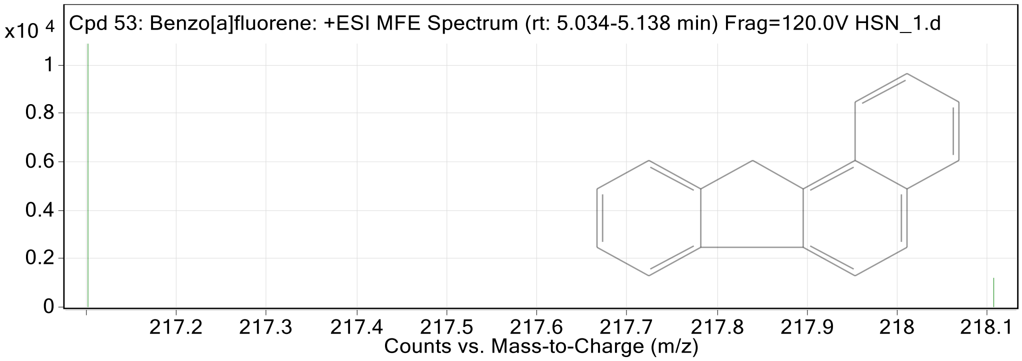

Peak List

m/z	z	Abund	Name	Formula	Ion	Score (DB)	Hits (DB)
217.10209	1	10871.1	Benzo[a]fluorene	C17 H12	(M+H)+	67.73	5
218.10694	1	1207.29		C17 H12	(M+H)+		

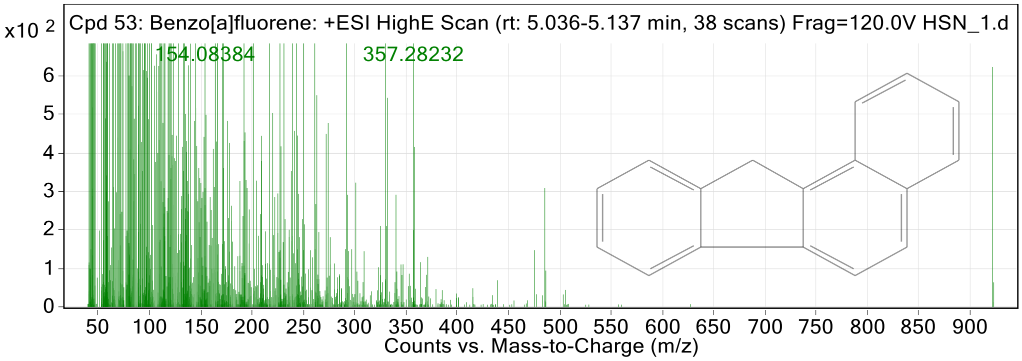

Peak List

m/z	z	Abund
41.03813		3913.46

m/z	z	Abund
45.03308	1	57230.11
58.06447		11082.5
79.05297	1	8294.21
81.06867	1	14835.87
89.05832	1	18379.63
91.05277	1	5139.53
114.08935		4384.51
154.08384		4982.74
192.1103		3727.53

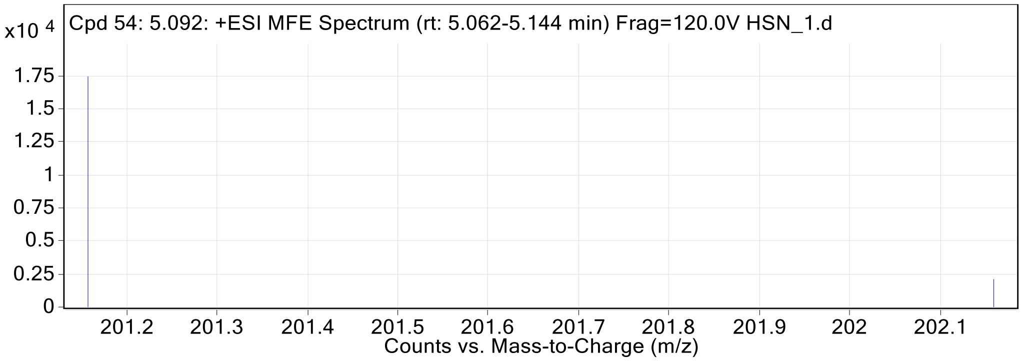

Peak List

m/z	z	Abund	Ion
201.15705	1	17466.01	(M+H)+
202.15941	1	2113.79	(M+H)+

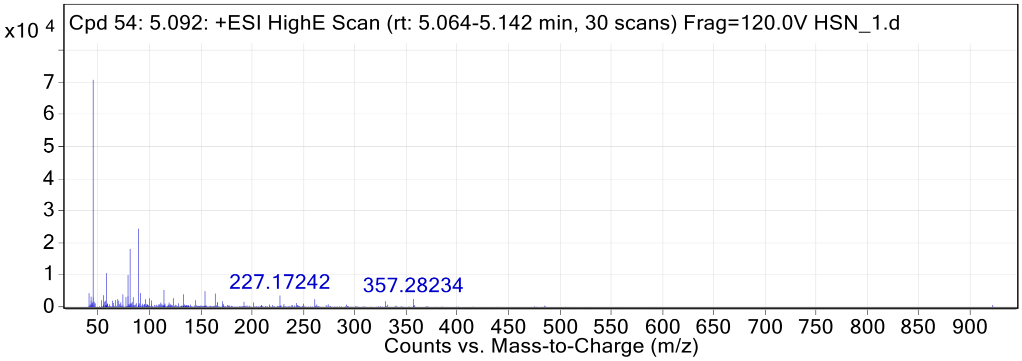

Peak List

m/z	z	Abund
41.03813		4268.71
45.0331	1	70793.36
58.06446		10512.29
79.05301	1	9957.48
81.06866	1	18102.78
89.05832	1	24375.58
91.05276		4316.66
114.08937		5287.53
154.08388		4848.88
164.04488		4121.11

Qualitative Analysis Report

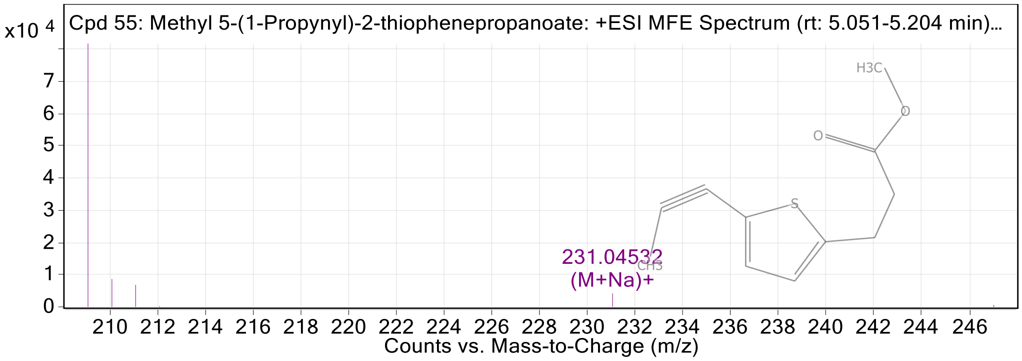

Peak List

m/z	z	Abund	Name	Formula	Ion	Score (DB)	Hits (DB)
209.06365	1	81855.39	Methyl 5-(1-Propynyl)-2-thiophenepropanoate	C11 H12 O2 S	(M+H)+	93.69	1
210.06682	1	8669.53		C11 H12 O2 S	(M+H)+		
211.06016	1	6845.47		C11 H12 O2 S	(M+H)+		
212.06365	1	260.14		C11 H12 O2 S	(M+H)+		
231.04532	1	4185.14			(M+Na)+		
247.0199	1	620.89			(M+K)+		

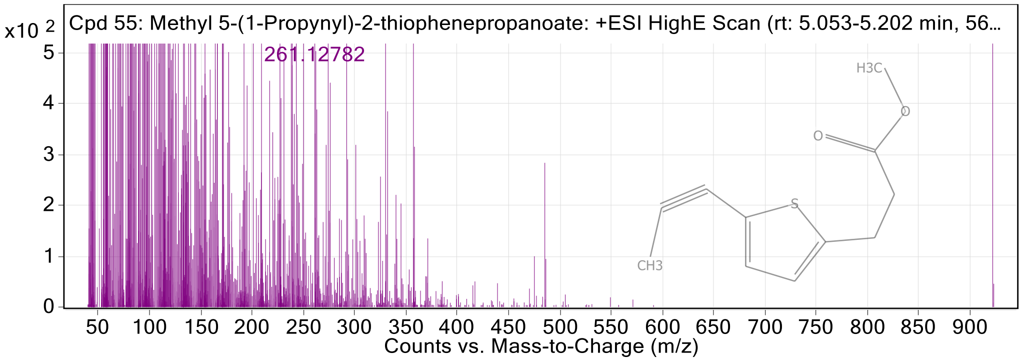

Peak List

m/z	z	Abund
45.03314	1	94111.17
45.09226		4717.28
58.06446	1	18745.42
79.053		6840.82
81.06864	1	11521.96
89.05838	1	36149.59
91.05284	1	3794.3
114.08935		4523.43
133.0838	1	6359.06
154.08382		3985.08

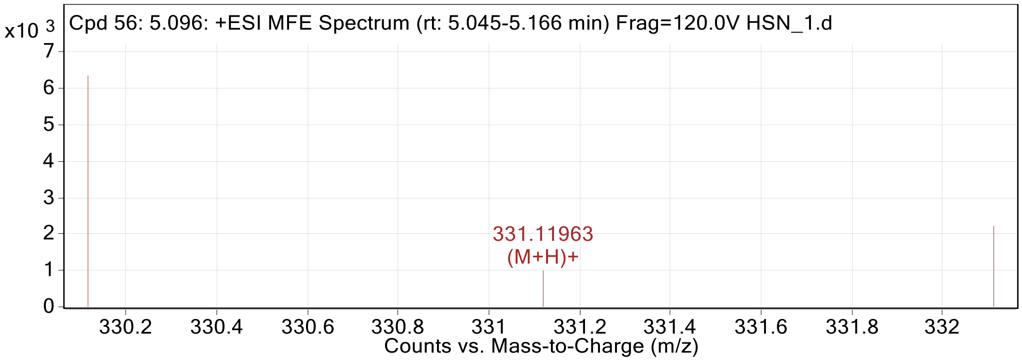

Peak List

m/z	z	Abund	Ion
330.1157	1	6360.48	(M+H)+
331.11963	1	1002.28	(M+H)+
332.11336	1	2230.46	(M+H)+

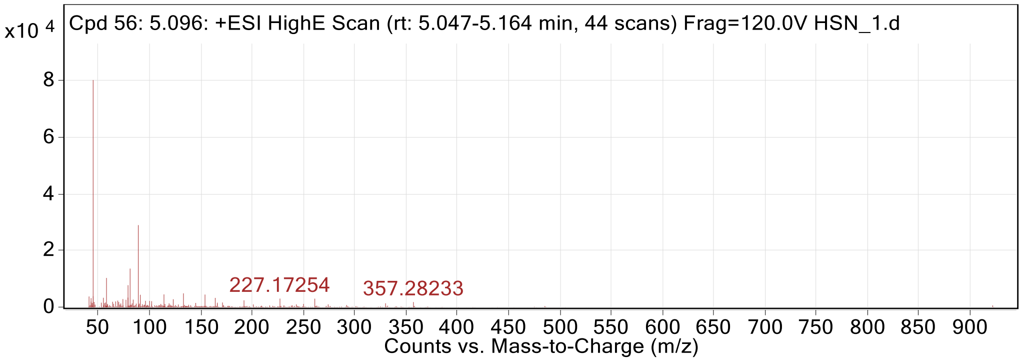

Peak List

m/z	z	Abund
45.03313	1	80147.98
45.09226		3812.12
58.06446		10284.53
79.05298		7732.34
81.06866	1	13588.79
89.05837	1	28935.51
91.0528		4340.83
114.08935		4476.92
133.08384	1	4848.82
154.08385		4420.66

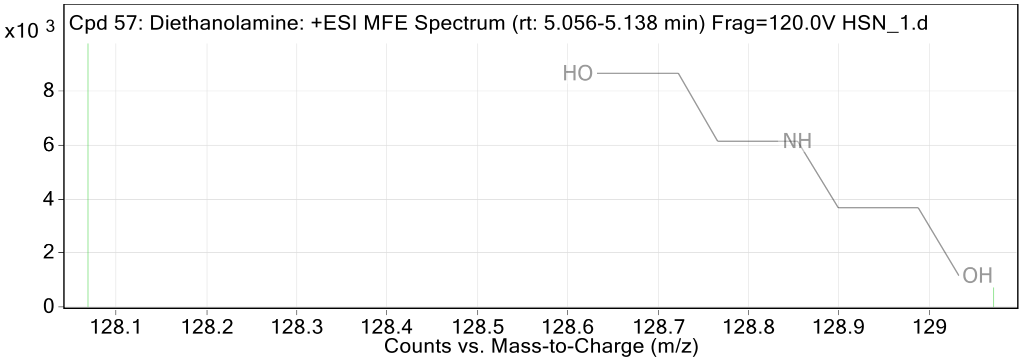

Peak List

m/z	z	Abund	Name	Formula	Ion	Score (DB)	Hits (DB)
128.06861	1	9761.66	Diethanolamine	C4 H11 N O2	(M+Na)+	84.42	4
129.07154	1	718.13		C4 H11 N O2	(M+Na)+		

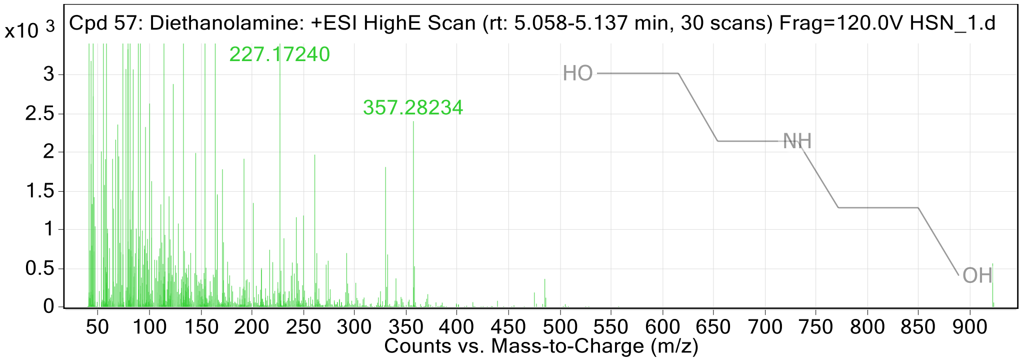

Peak List

m/z	z	Abund
41.03813		4262.88
45.03309	1	64781.88
58.06447		10724.15
79.05302	1	9857.51
81.06866	1	17831.53
89.05831	1	21576.69
91.05276		4679.99
114.08934		5119.63
154.08387		5015.68
164.04489		4240.61

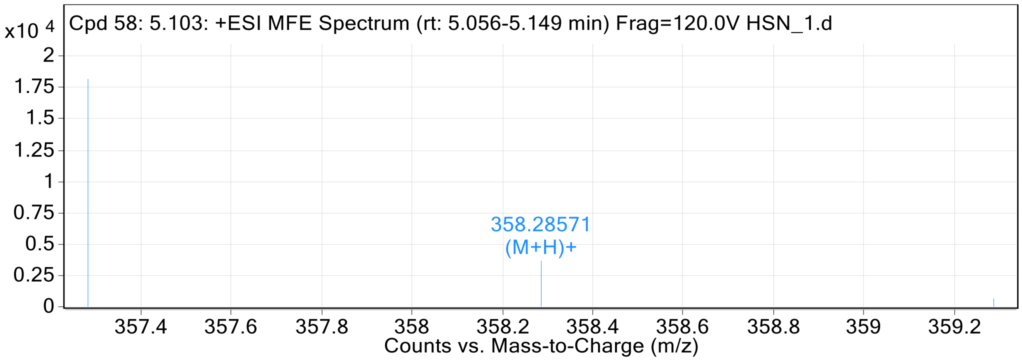

Peak List

m/z	z	Abund	Ion
357.28275	1	18183.93	(M+H)+
358.28571	1	3668.47	(M+H)+
359.28716	1	663.65	(M+H)+

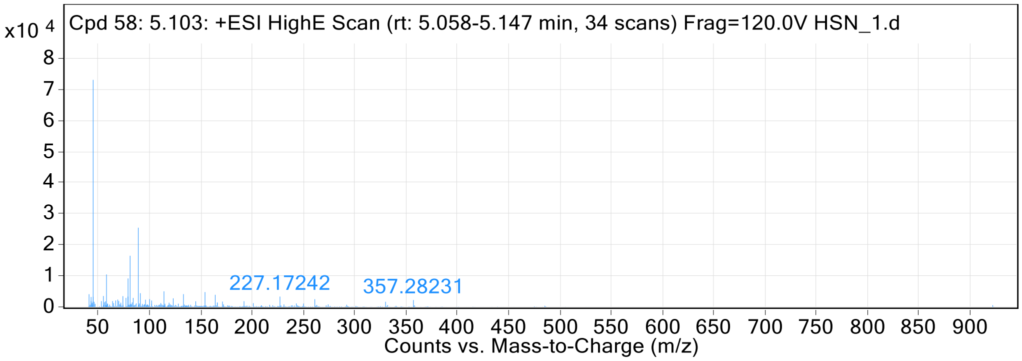

Peak List

m/z	z	Abund
41.03815		4086.17
45.0331	1	73038.82

58.06446		10434.57
79.05302	1	9147.01
81.06866	1	16455.25
89.05834	1	25481.34
91.05278		4410.95
114.08935		5009.95
133.08372		4111.15
154.0839		4766.56

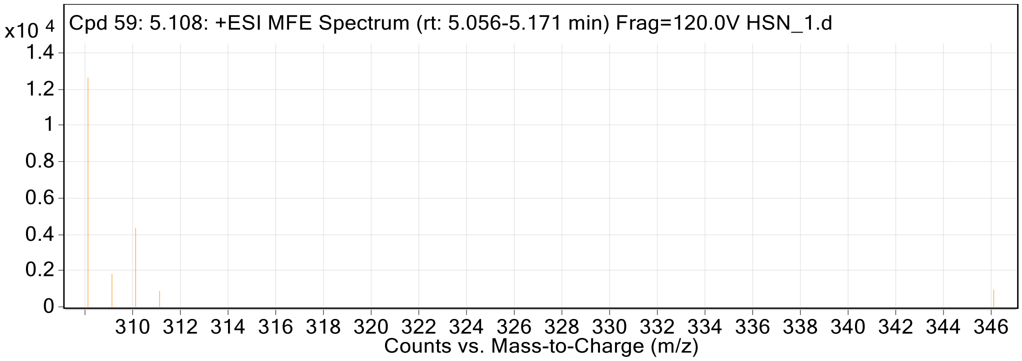

Peak List

m/z	z	Abund	Ion
308.1339	1	12631.37	(M+H)+
309.13879	1	1816.38	(M+H)+
310.1316	1	4354.51	(M+H)+
311.13477	1	883.64	(M+H)+
346.08952	1	955.27	(M+K)+

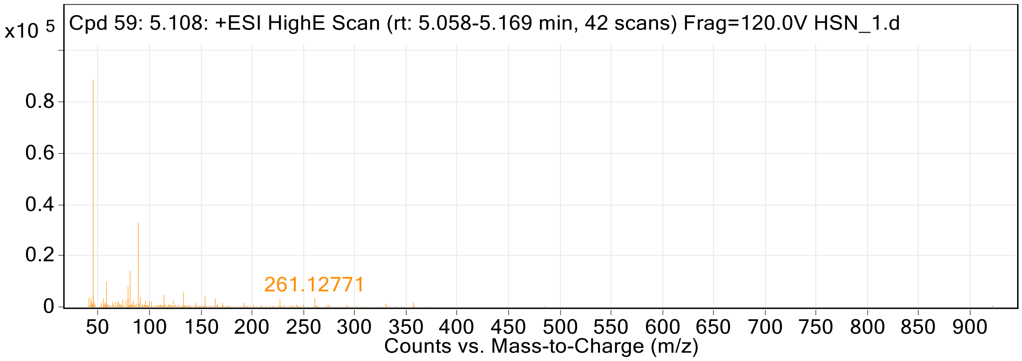

Peak List

m/z	z	Abund
45.03314	1	88650.89
45.09226		4365.68
58.06445		9974.99
79.053	1	8035.53
81.06866	1	14078.63
89.05837	1	32827.09
91.05281	1	3986.69
114.08936		4684.11
133.0838	1	5649.92
154.08388		4286.03

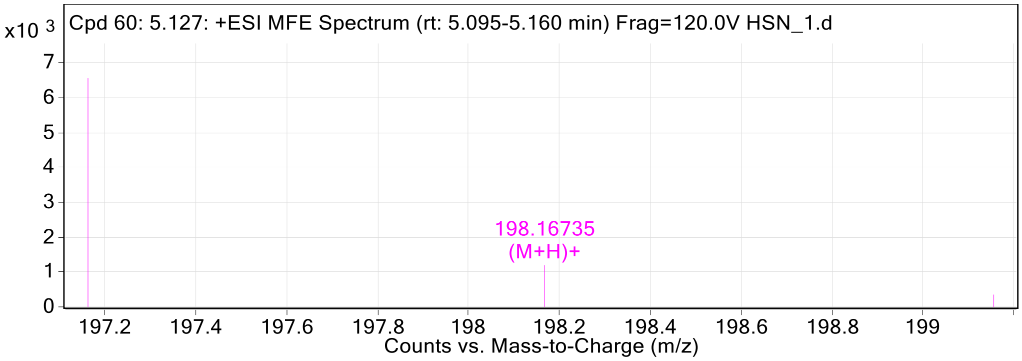

Peak List

m/z	z	Abund	Ion
197.16211	1	6556	(M+H)+
198.16735	1	1196.52	(M+H)+
199.15601	1	351.34	(M+H)+

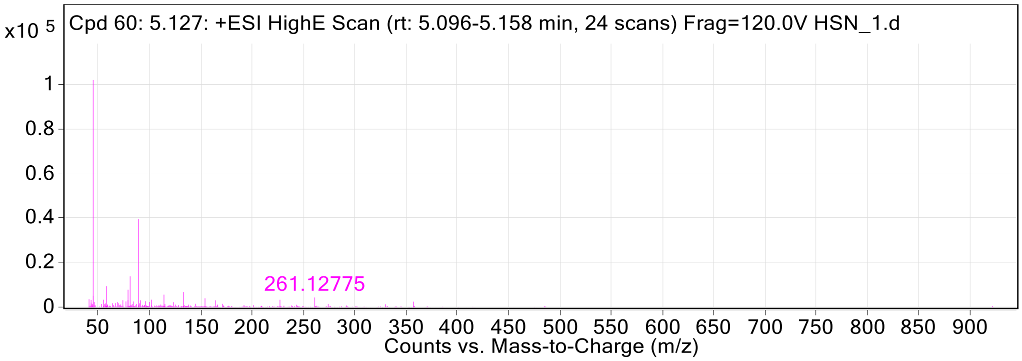

Peak List

m/z	z	Abund
45.03314	1	101869.63
45.09226		5064.3
58.06446		9412.78
79.05303		7786.12
81.06865	1	13789.11
89.05836	1	39447.83
114.08944		5568.44
133.0838	1	6773.53
154.08388		3924.14
261.12775		4295.3

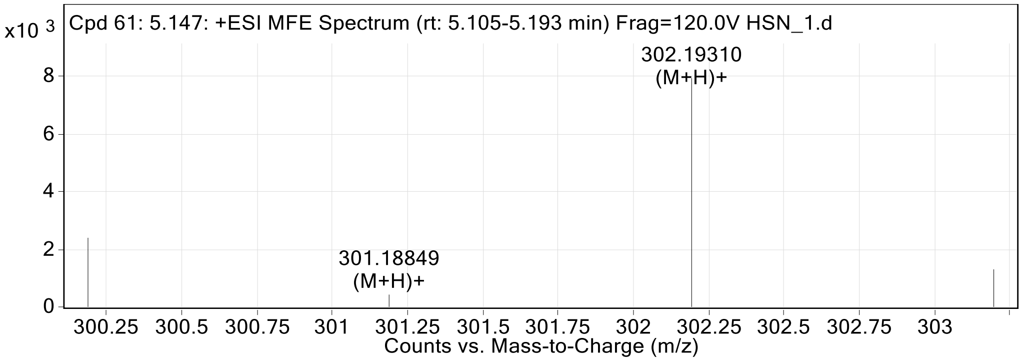

Peak List

m/z	z	Abund	Ion
300.18858	1	2397.57	(M+H)+
301.18849	1	426.77	(M+H)+

302.1931	1	7934.87	(M+H)+
303.19635	1	1303.22	(M+H)+

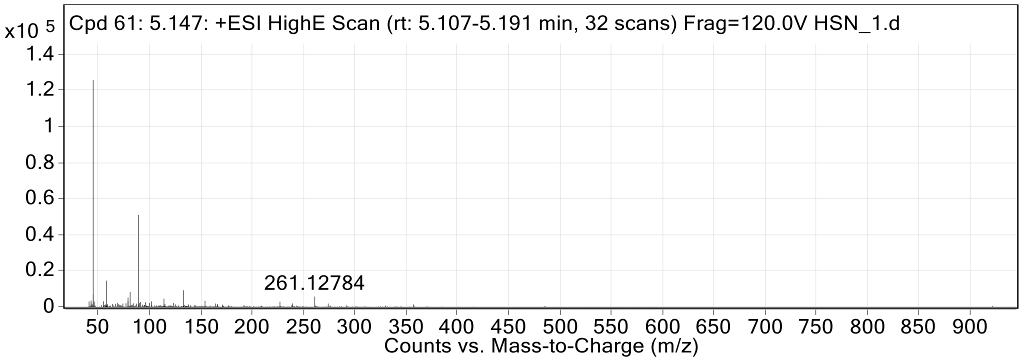

Peak List

m/z	z	Abund
43.01741		3440.22
45.03316	1	125578.02
45.09226		6761.17
58.06444	1	14477.5
79.05299		5099.11
81.06863		8236.32
89.05839	1	50930.29
114.0894		4476.03
133.0838	1	9158.85
261.12784	1	5653.45

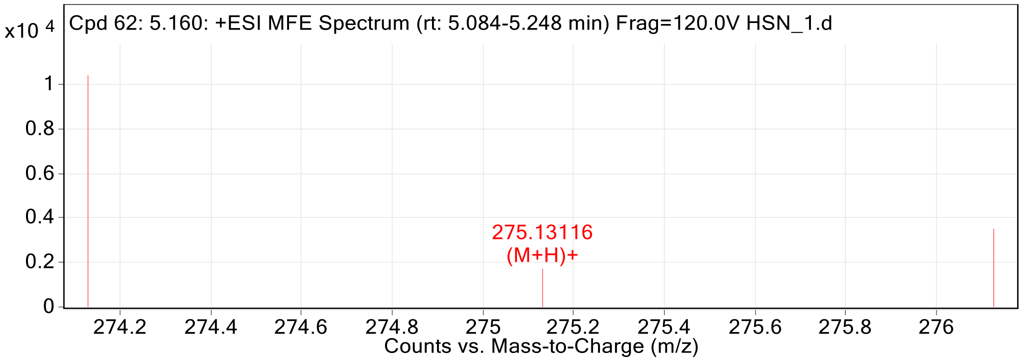

Peak List

m/z	z	Abund	Ion
274.1288	1	10407.14	(M+H)+
275.13116	1	1720.92	(M+H)+
276.12588	1	3510.4	(M+H)+

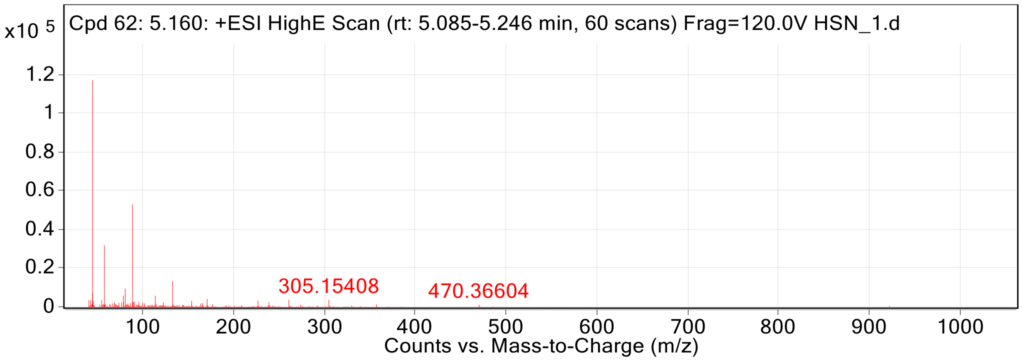

Peak List

m/z	z	Abund

45.03324	1	117073.33
45.09228		6907.87
58.06449	1	31795.86
79.05299		5843.21
81.06861		9364.78
89.05856	1	52885.02
114.08945		5760.79
133.08383	1	13355.09
171.14666		4020.57
305.15408		3674.46

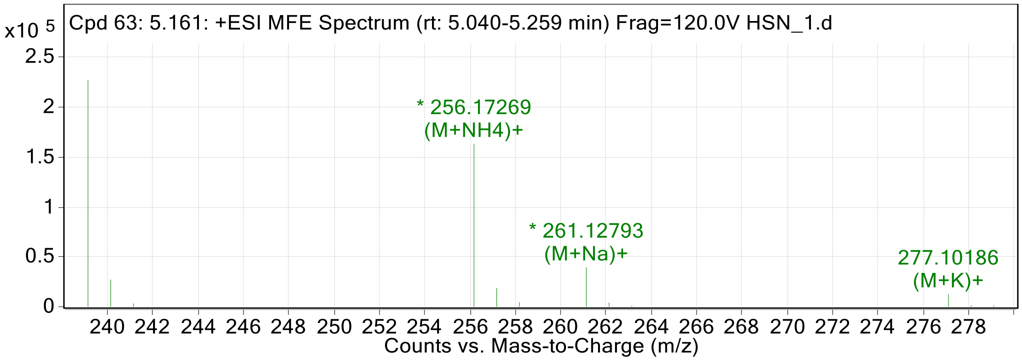

Peak List

m/z	z	Abund	Ion
239.14661	1	227473.53	(M+H)+
240.14958	1	27202.05	(M+H)+
241.15125	1	3257.95	(M+H)+
256.17269	1	163372.39	(M+NH4)+
257.17584	1	18647.06	(M+NH4)+
258.17691	1	4609.81	(M+NH4)+
261.12793	1	39623.46	(M+Na)+
262.13156	1	4180.59	(M+Na)+
277.10186	1	12678.16	(M+K)+
278.10623	1	1444.75	(M+K)+

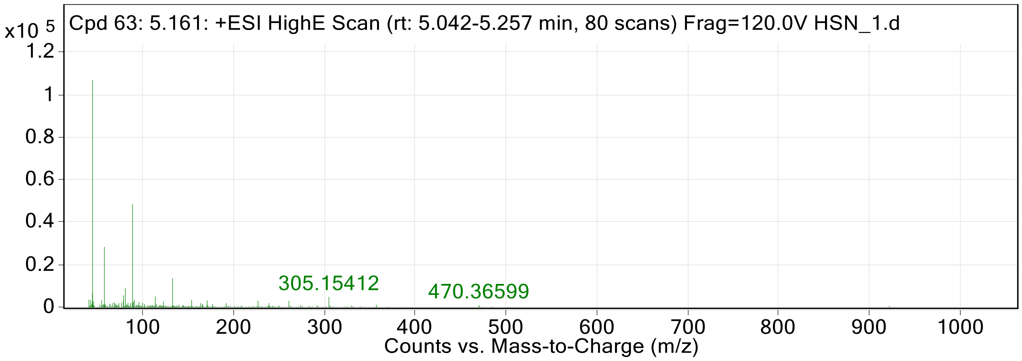

Peak List

m/z	z	Abund
41.03811		3491.88
45.0333	1	106869.55
45.09229		6722.07
58.06449	1	28354.74
79.05297		5544.53
81.06863		8906.39
89.05869	1	48439.7
114.08941		4924.48
133.08386	1	13585.02

305.15412		4783.75

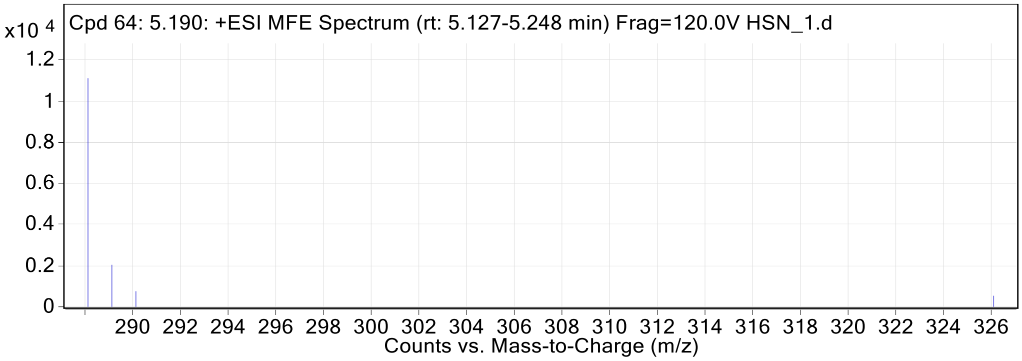

Peak List

m/z	z	Abund	Ion
288.15225	1	11116.83	(M+H)+
289.15491	1	2045.18	(M+H)+
290.16175	1	755.97	(M+H)+
326.1089	1	538.45	(M+K)+

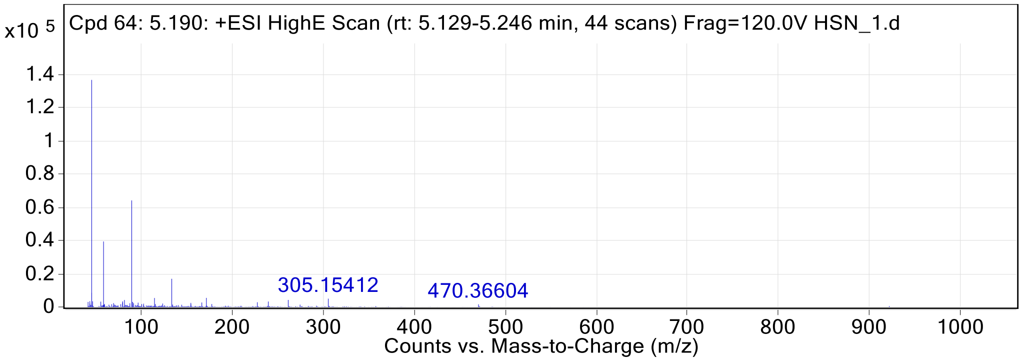

Peak List

m/z	z	Abund
45.03327	1	136740.03
45.09228		8493.4
58.06449	1	39423.98
81.06855		4191.46
89.05859	1	64154.86
114.08946		5395.54
133.08383	1	16965.29
171.14666		5456.89
261.12785		4229.41
305.15412	1	4981.41

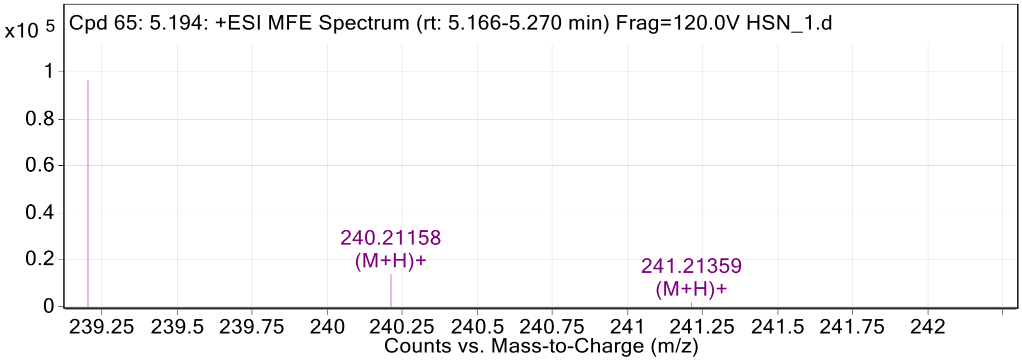

Peak List

m/z	z	Abund	Ion
239.20197	1	96753.36	(M+H)+
240.21158	1	13878.23	(M+H)+
241.21359	1	1848.35	(M+H)+
242.22014	1	101.9	(M+H)+

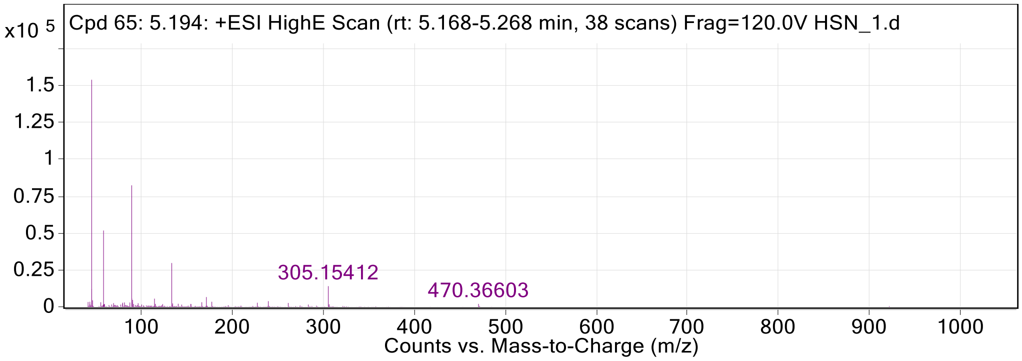

Peak List

m/z	z	Abund
45.03348	1	153983.2
45.0923		12126.55
45.10837		5282.77
58.06449	1	51743.84
89.05893	1	82376.52
89.14166		5779.11
114.08945		5531.31
133.08387	1	29688.35
171.14664	1	6609.22
305.15412	1	13999.58

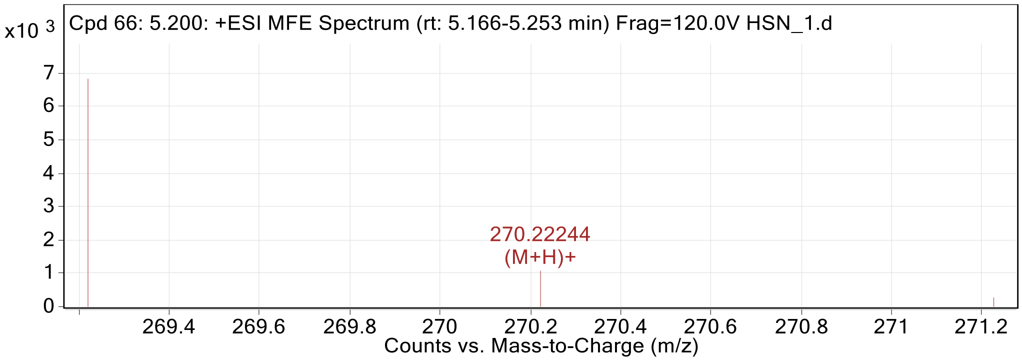

Peak List

m/z	z	Abund	Ion
269.21981	1	6831.01	(M+H)+
270.22244	1	1079.73	(M+H)+
271.22662	1	274.4	(M+H)+

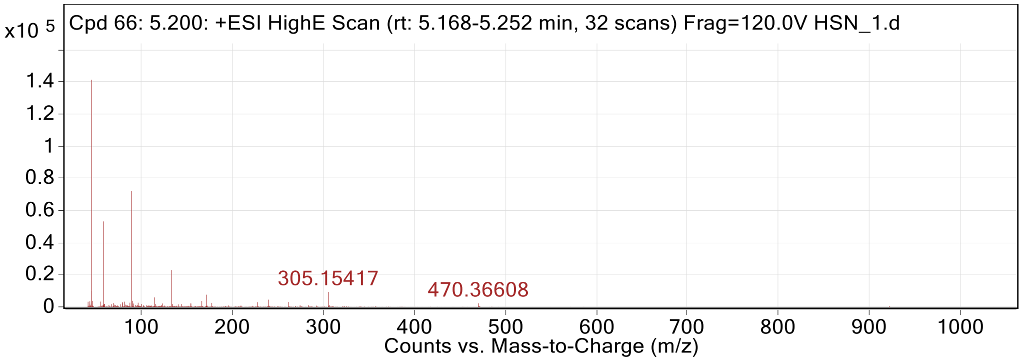

Peak List

m/z	z	Abund
45.03337	1	141342.44
45.0923		9889.98
58.0645	1	53196.75
89.05876	1	72168.06
89.14167		4298.93
114.08947		5881.23
133.08386	1	22967.41
171.14664	1	7584.12
239.20874	1	4494.53
305.15417	1	9293.08

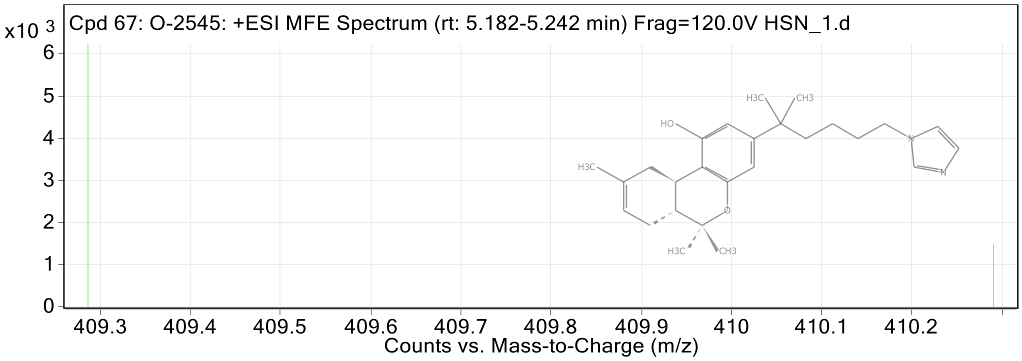

Peak List

m/z	z	Abund	Name	Formula	Ion	Score (DB)	Hits (DB)
409.28681	1	6238.17	O-2545	C26 H36 N2 O2	(M+H)+	70.7	1
410.29075	1	1501.22		C26 H36 N2 O2	(M+H)+		

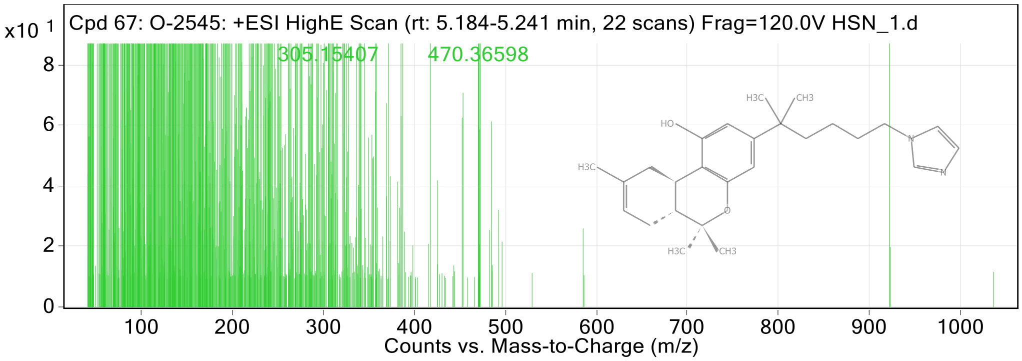

Peak List

m/z	z	Abund
45.03327	1	122415.05
45.09228		7502.95
58.06449	1	66326.88

89.0586	1	61929.11
114.08946		6971.29
133.08381	1	18275.29
166.12004		4500.41
171.14665	1	10566.57
239.2088	1	5656.1
305.15407	1	6712.28

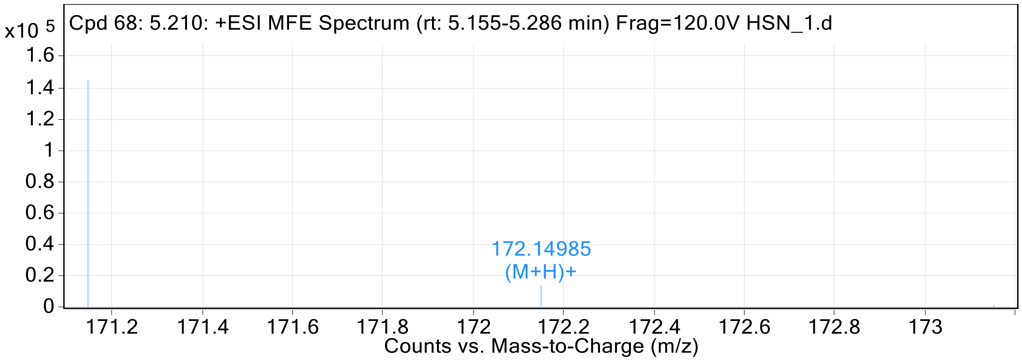

Peak List

m/z	z	Abund	Ion
171.14663	1	145113.77	(M+H)+
172.14985	1	13833.32	(M+H)+
173.15228	1	1356.65	(M+H)+

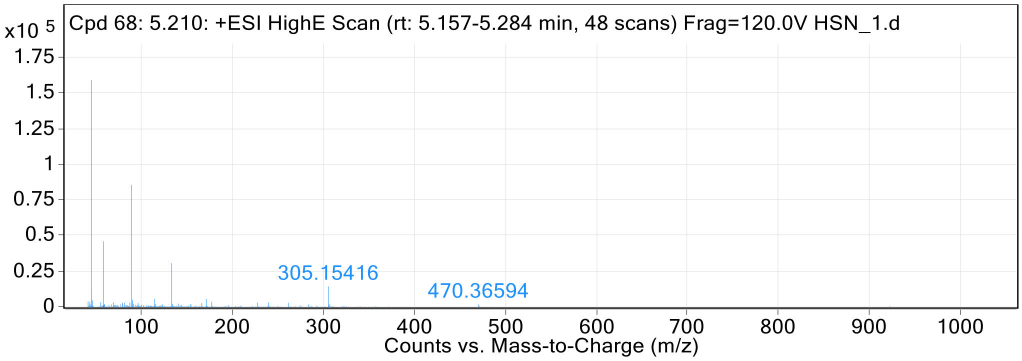

Peak List

m/z	z	Abund
45.03346	1	159122.52
45.09231		12270.84
45.10836		5109.15
58.06449	1	45947.86
89.05889	1	85501.47
89.14167		5947.72
114.08943		5382.78
133.08387	1	30532.05
171.14663		5332.2
305.15416	1	14181.17

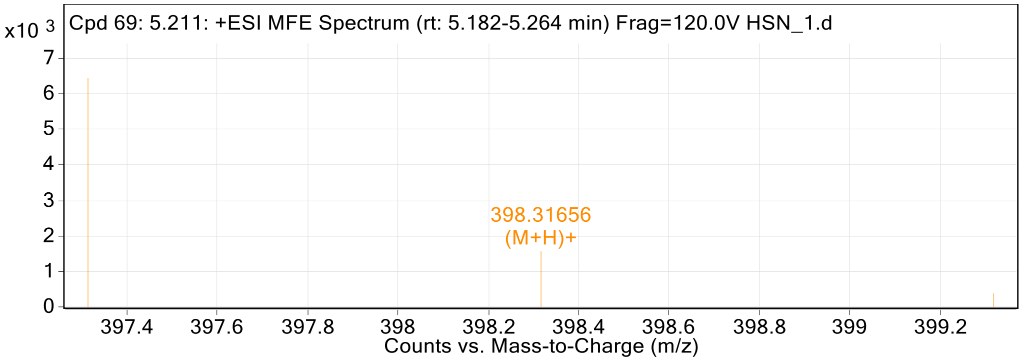

Peak List

m/z	z	Abund	Ion
397.31426	1	6450.36	(M+H)+
398.31656	1	1567.33	(M+H)+
399.3182	1	386.67	(M+H)+

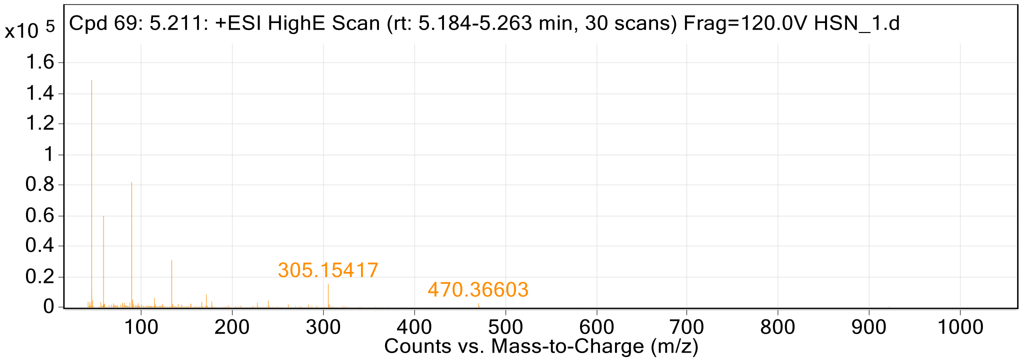

Peak List

m/z	z	Abund
45.03351	1	148810.31
45.09231		12005.48
45.10836		5750.86
58.0645	1	59843.27
89.05898	1	81937.88
89.14167		5850.62
114.08943		6108.86
133.08389	1	30790.88
171.14664	1	8264.27
305.15417	1	15244.92

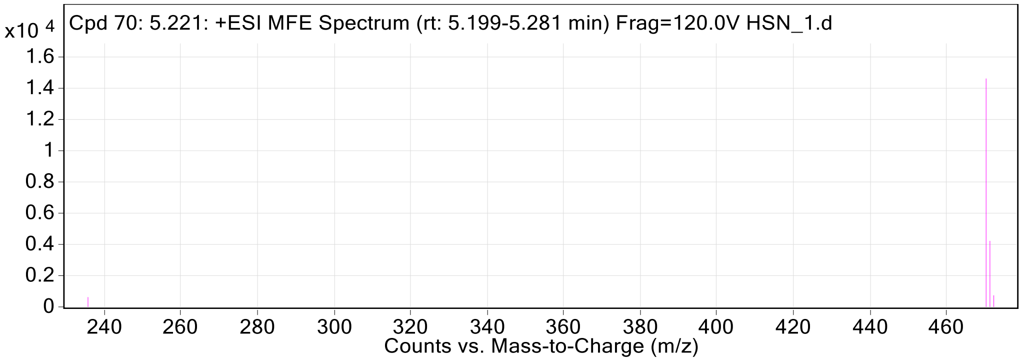

Peak List

m/z	z	Abund	Ion
235.68598	2	626.16	(M+2H)+2
470.36655	1	14629.78	(M+H)+

471.36907	1	4229.94	(M+H)+
472.36999	1	741.76	(M+H)+

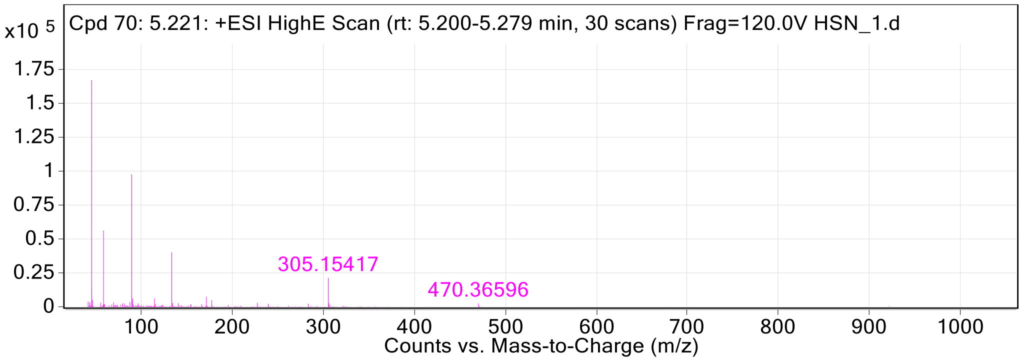

Peak List

m/z	z	Abund
45.03359	1	167516.83
45.05468		6739.24
45.09232		14580.21
45.10837		7877.69
58.0645	1	56395.58
89.05907	1	97684.45
89.14168		7761.49
133.08388	1	40233.33
171.14664	1	7360.49
305.15417	1	21378.86

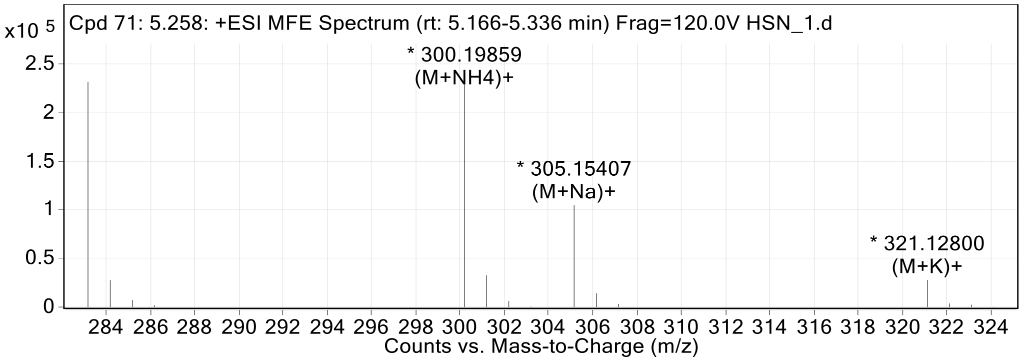

Peak List

m/z	z	Abund	Ion
283.17221	1	231872.59	(M+H)+
284.17584	1	27702.08	(M+H)+
285.1772	1	7130	(M+H)+
300.19859	1	233778.22	(M+NH4)+
301.20187	1	32752.22	(M+NH4)+
302.20407	1	6301.89	(M+NH4)+
305.15407	1	104932.74	(M+Na)+
306.15777	1	14188.64	(M+Na)+
321.128	1	27964.02	(M+K)+
322.13187	1	3685.53	(M+K)+

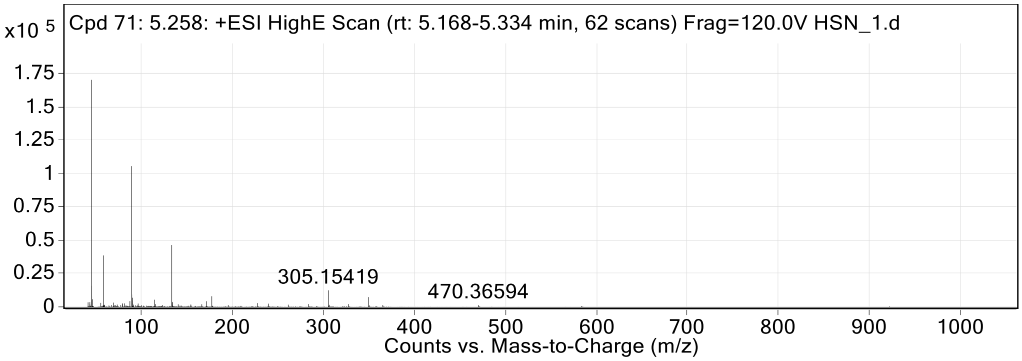

Peak List

m/z	z	Abund
45.03368	1	170238.58
45.09233		15845.57
45.10836		7941.09
58.06449	1	38544.62
89.05923	1	105414.94
89.14168		9249.11
133.08426	1	46427.58
177.10984	1	7953.66
305.15419	1	12382.26
349.18021	1	7348.09

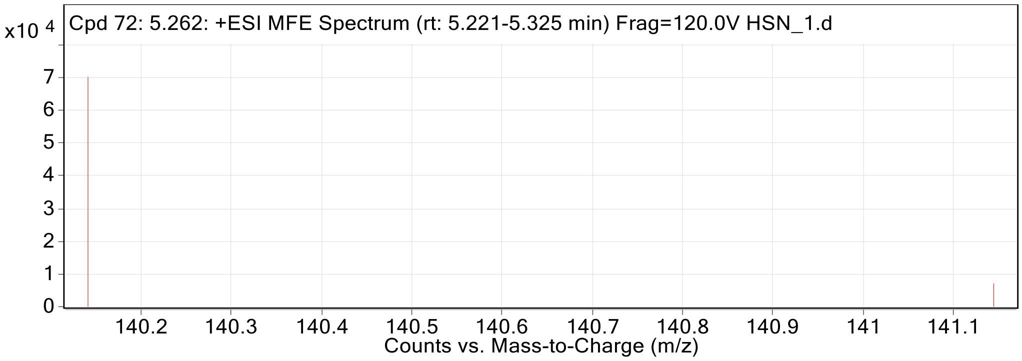

Peak List

m/z	z	Abund	Ion
140.14125	1	70252.63	(M+H)+
141.14494	1	7140.58	(M+H)+

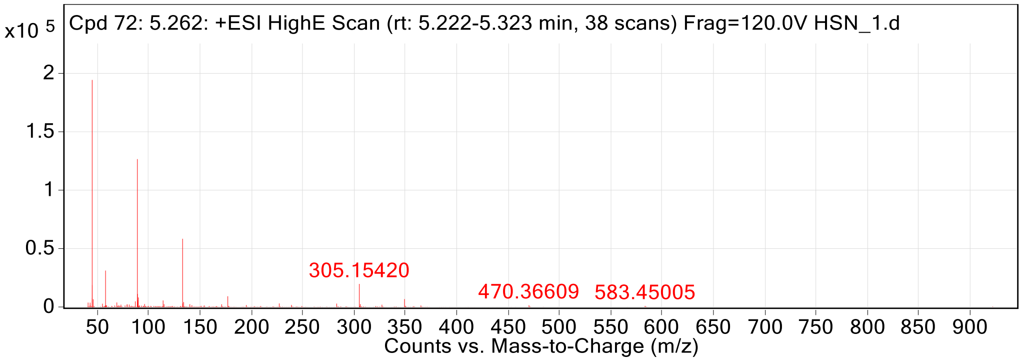

Peak List

m/z	z	Abund
45.03371	1	193936.13
45.09234		18697.87
45.10837		10335.13

58.06447	1	31088.53
89.0592	1	126309.15
89.14169		11124.4
90.0618	1	8131.08
133.08413	1	58265.93
177.10985	1	9150.39
305.1542	1	19663.23

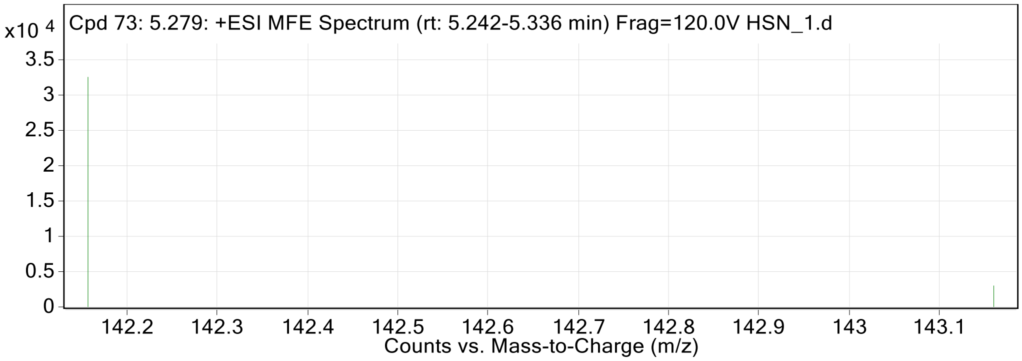

Peak List

m/z	z	Abund	Ion
142.15674	1	32500.43	(M+H)+
143.16048	1	3016.52	(M+H)+

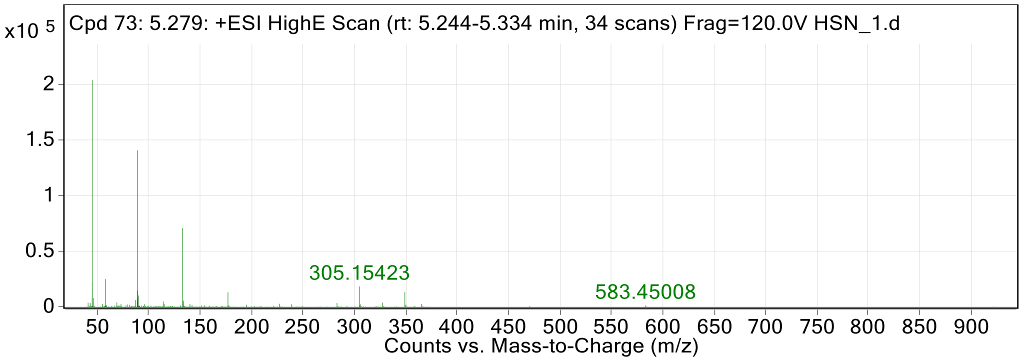

Peak List

m/z	z	Abund
45.03391	1	203269.42
45.09234		22357.87
45.10837		13106.53
58.06448	1	24789.19
89.05948	1	140299.53
89.14169		14393.49
133.08436	1	70518.3
177.10985	1	13046.42
305.15423	1	18202.04
349.18021	1	13386.41

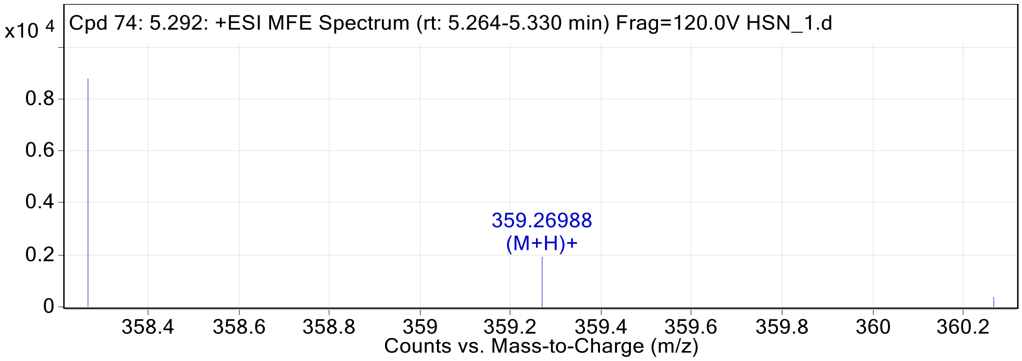

Peak List

m/z	z	Abund	Ion
358.26682	1	8784.77	(M+H)+
359.26988	1	1924.1	(M+H)+
360.2675	1	380.54	(M+H)+

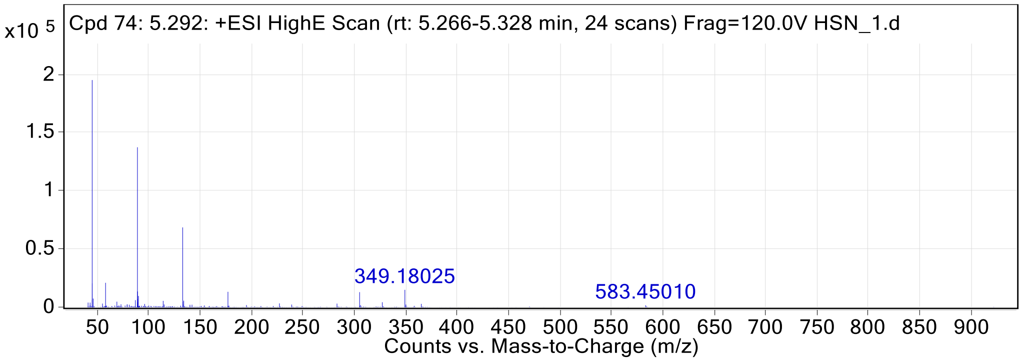

Peak List

m/z	z	Abund
45.03384	1	195028.94
45.09235		20362.66
45.10837		11235.66
58.06444	1	20869.03
89.05937	1	137284.2
89.1417		13410.51
133.08438	1	68436.65
177.10987	1	13117.21
305.15423	1	12840.76
349.18025	1	14808.42

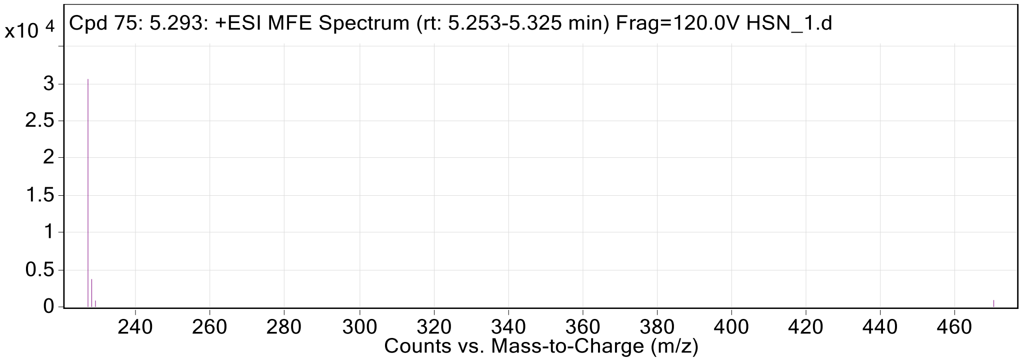

Peak List

m/z	z	Abund	Ion
227.17279	1	30612.55	(M+H)+
228.17578	1	3731.66	(M+H)+

229.17888	1	847.8	(M+H)+
470.36736	1	916.23	(2M+NH4)+

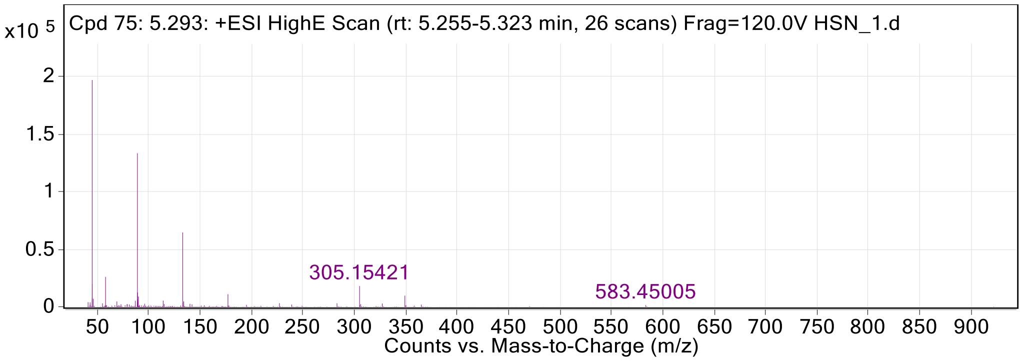

Peak List

m/z	z	Abund
45.03377	1	196749.67
45.09234		19728.96
45.10836		10994.06
58.06445	1	25949.61
89.05926	1	133239.38
89.1417		12379.5
133.0842	1	64509.75
177.10987	1	10927.18
305.15421	1	17992.21
349.18031	1	9662.64

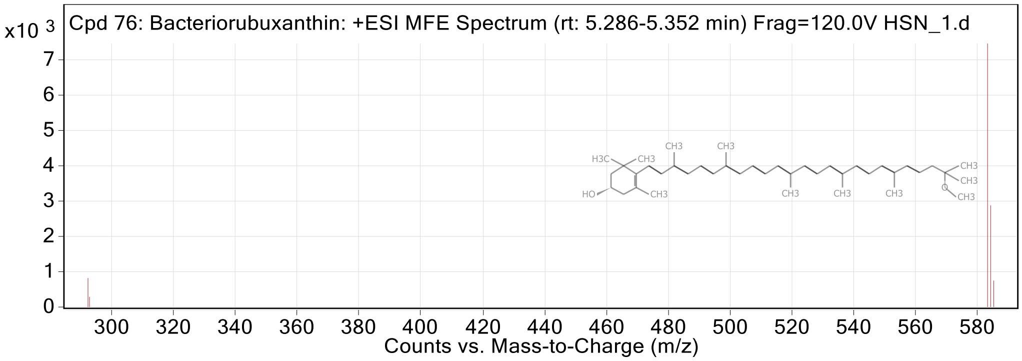

Peak List

m/z	z	Abund	Name	Formula	Ion	Score (DB)	Hits (DB)
292.22742	2	820.94			(M+2H)+2		
292.72541	2	288.56			(M+2H)+2		
583.44937	1	7454.46	Bacteriorubuxanthin	C41 H58 O2	(M+H)+	90.94	6
584.45403	1	2877.13		C41 H58 O2	(M+H)+		
585.45435	1	747.01		C41 H58 O2	(M+H)+		

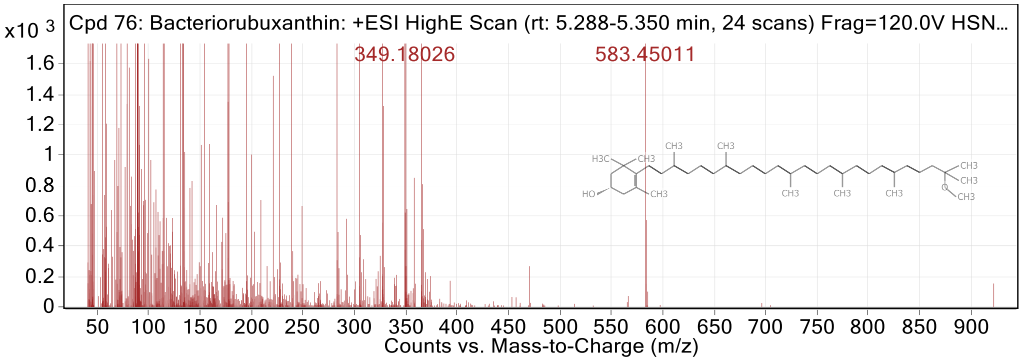

Peak List

m/z	z	Abund
45.03418	1	203542.92
45.07253		10474.92
45.09234		26193.88
45.10836		15674.46
89.05982	1	157794.89
89.14169		18791.69
90.06181	1	12748.33
133.08474	1	89639.47
177.10988	1	20952.86
349.18026	1	29087.67

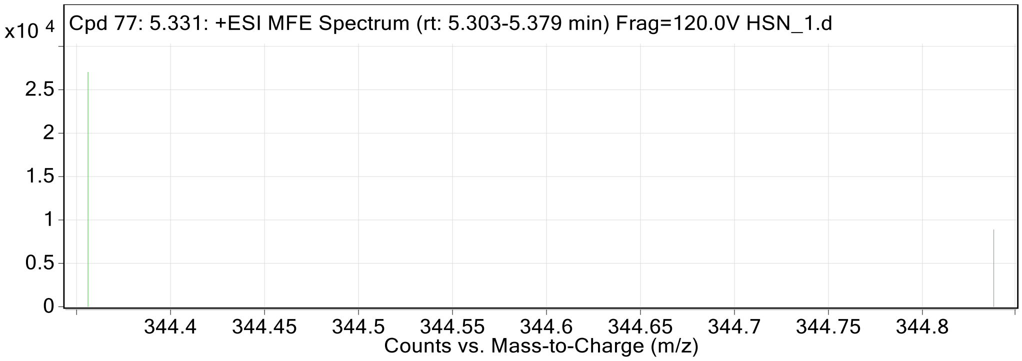

Peak List

m/z	z	Abund	Ion
344.35587	1	27069.6	(M+H)+
344.83836	1	8901.95	(M+H)+

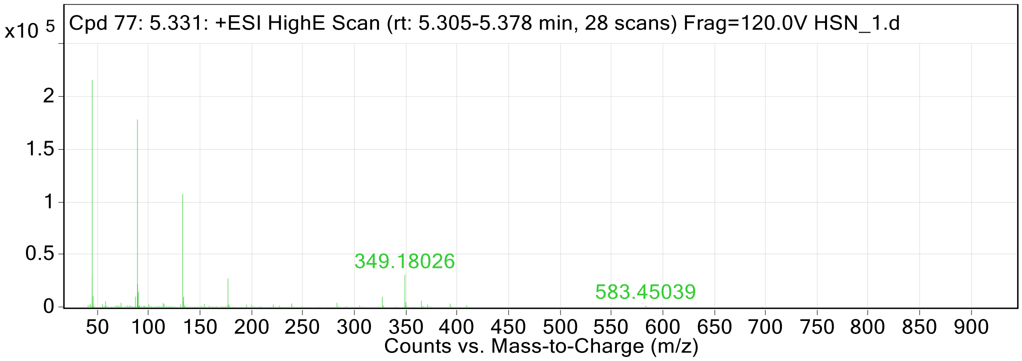

Peak List

m/z	z	Abund
45.03426	1	215900.52
45.07252		12847.61
45.09234		29097.67

45.10835		17874.05
89.05987	1	178384.95
89.14169		21461.46
90.06182	1	14609.86
133.08476	1	107558.29
177.10986	1	27085.92
349.18026	1	30504.14

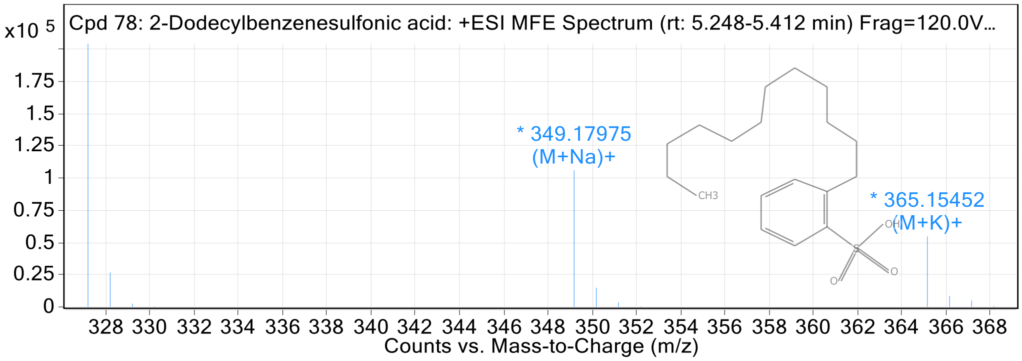

Peak List

m/z	z	Abund	Name	Formula	Ion	Score (DB)	Hits (DB)
327.19833	1	204410.59	2-Dodecylbenzenesulfonic acid	C18 H30 O3 S	(M+H)+	84.17	1
328.20173	1	26663.12		C18 H30 O3 S	(M+H)+		
329.20314	1	2587.62		C18 H30 O3 S	(M+H)+		
349.17975	1	106000.39			(M+Na)+		
350.1836	1	14856.83			(M+Na)+		
351.18591	1	3711.25			(M+Na)+		
365.15452	1	54757.6			(M+K)+		
366.15736	1	8592.32			(M+K)+		
367.1547	1	5056.98			(M+K)+		
368.15726	1	769.39			(M+K)+		

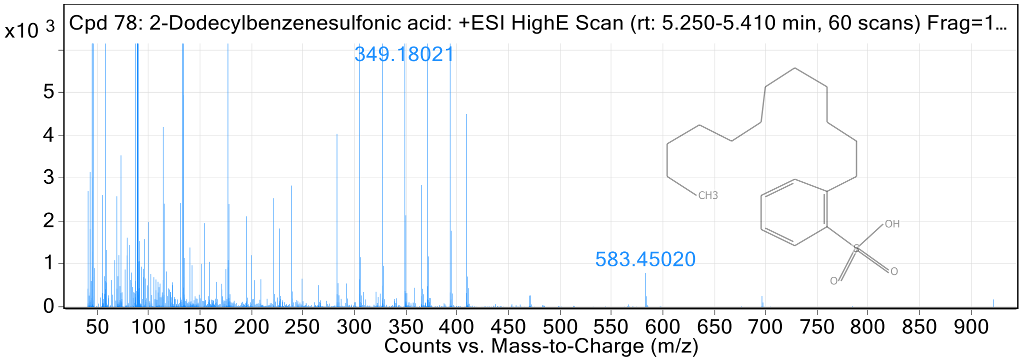

Peak List

m/z	z	Abund
45.03419	1	209314.78
45.09233		26874.86
45.10836		16298.24
58.06448		14531.84
89.05988	1	167392.23
89.14169		20224.75
90.06181	1	13867.11
133.08501	1	91909.87
177.10986	1	28335.19
349.18021	1	14810.39

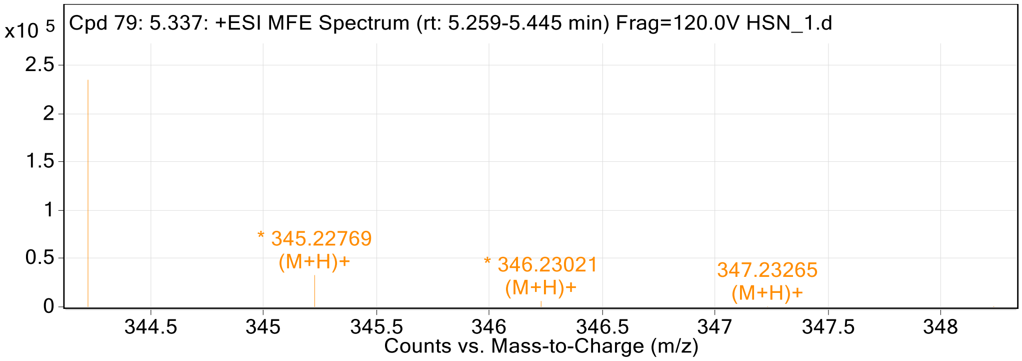

Peak List

m/z	z	Abund	Ion
344.22436	1	235174	(M+H)+
345.22769	1	32922.25	(M+H)+
346.23021	1	6048.32	(M+H)+
347.23265	1	457.99	(M+H)+
348.23408	1	692.35	(M+H)+

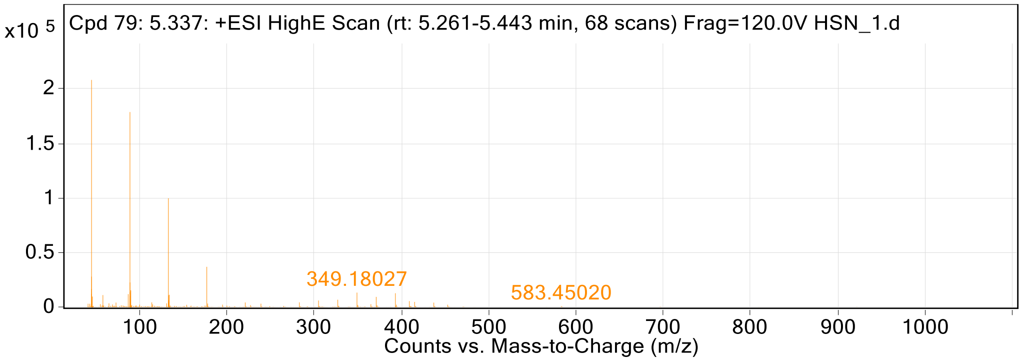

Peak List

m/z	z	Abund
45.03427	1	208163.5
45.09233		27823.32
45.10836		16542.77
89.05995	1	178731.72
89.14168		22490.44
89.16423		13467.09
90.06182	1	15306.63
133.08527	1	99655
177.10989	1	36814.34
349.18027	1	13166.85

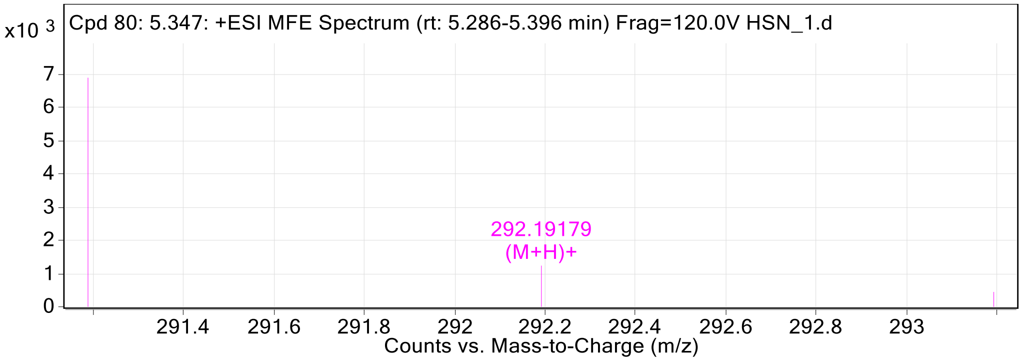

Peak List

m/z	z	Abund	Ion

291.18813	1	6887.43	(M+H)+
292.19179	1	1237.37	(M+H)+
293.19314	1	446.54	(M+H)+

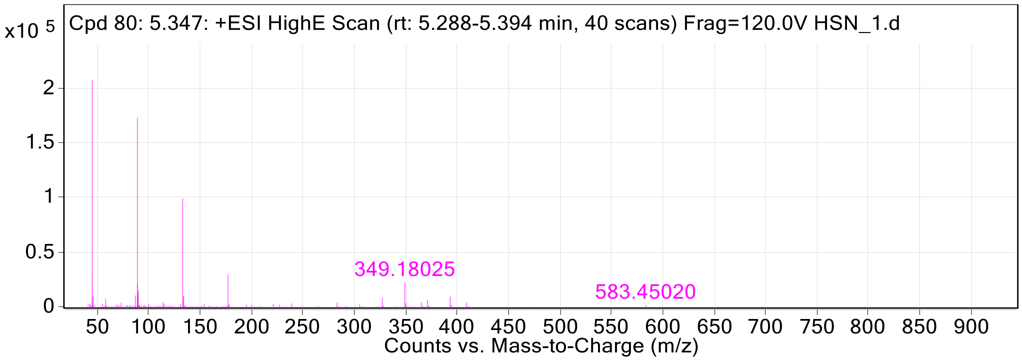

Peak List

m/z	z	Abund
45.03426	1	207250.91
45.09233		27614.71
45.10835		16552.72
89.0599	1	172741.63
89.14168		21063.55
89.16421		12076.59
90.06181	1	14413.33
133.08499	1	99066.77
177.10986	1	29822
349.18025	1	21926.34

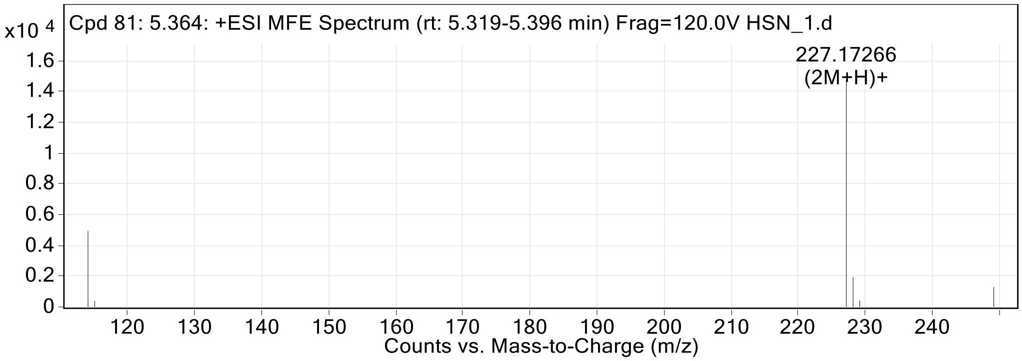

Peak List

m/z	z	Abund	Ion
114.08942	1	4939.99	(M+H)+
115.09154	1	391.34	(M+H)+
227.17266	1	14817.8	(2M+H)+
228.17533	1	1932.94	(2M+H)+
229.1781	1	414.92	(2M+H)+
249.15487	1	1295.28	(2M+Na)+

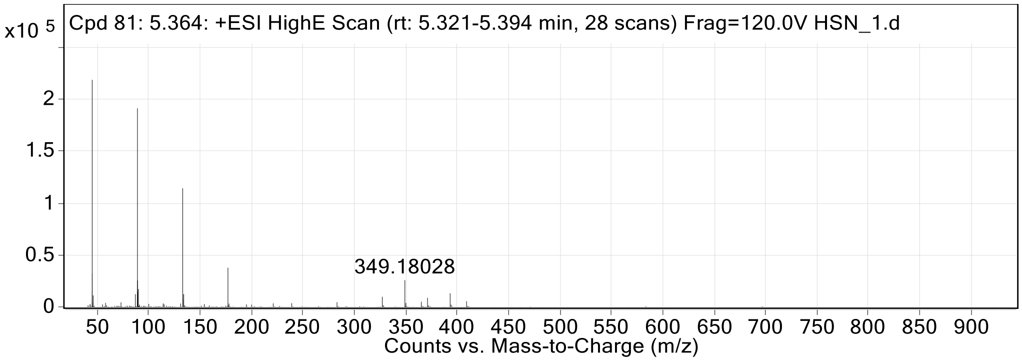

Peak List

m/z	z	Abund
45.03446	1	218839.73
45.09233		32228.85
45.10835		20123.94
89.06014	1	191415.03
89.14168		25418.47
89.16421		15206.93
90.06181	1	17234.91
133.08518	1	114359.66
177.10987	1	37712.26
349.18028	1	25739.54

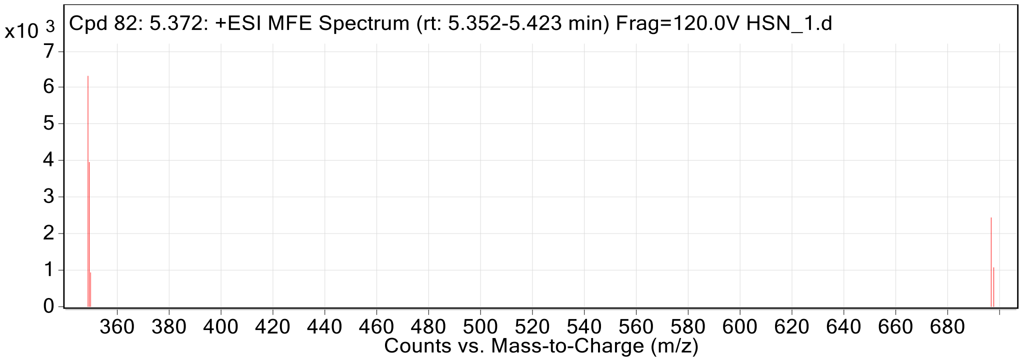

Peak List

m/z	z	Abund	Ion
348.76951	2	6324.17	(M+2H)+2
349.26985	2	3963.2	(M+2H)+2
349.76929	2	940.92	(M+2H)+2
696.53427	1	2445.87	(M+H)+
697.53626	1	1079.8	(M+H)+

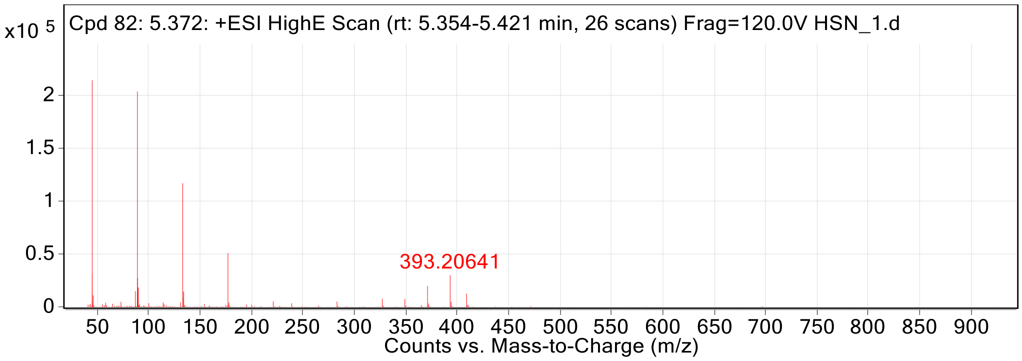

Peak List

m/z	z	Abund

45.03447	1	214338.83
45.09233		31413.27
45.10836		19272.9
89.06016	1	203644.36
89.14169		27217.77
90.06181	1	18479.02
133.08558	1	116923.73
177.10989	1	50866.87
371.22429	1	19881.08
393.20641	1	29909.51

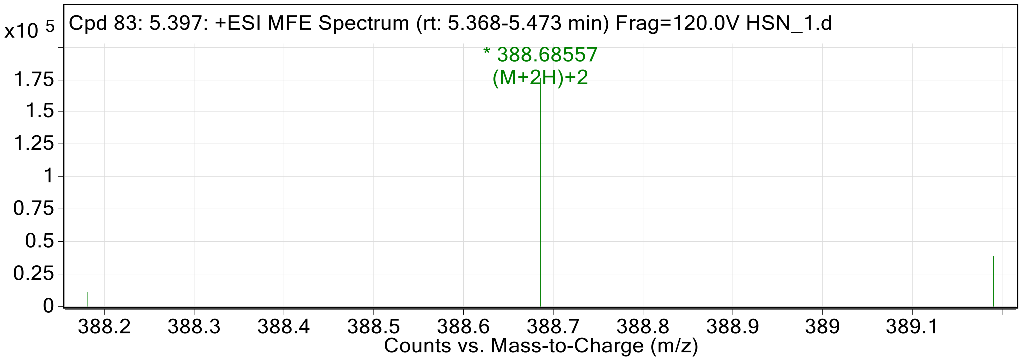

Peak List

m/z	z	Abund	Ion
388.18143	2	11344.18	(M+2H)+2
388.68557	2	176233.42	(M+2H)+2
389.19009	2	38989.2	(M+2H)+2

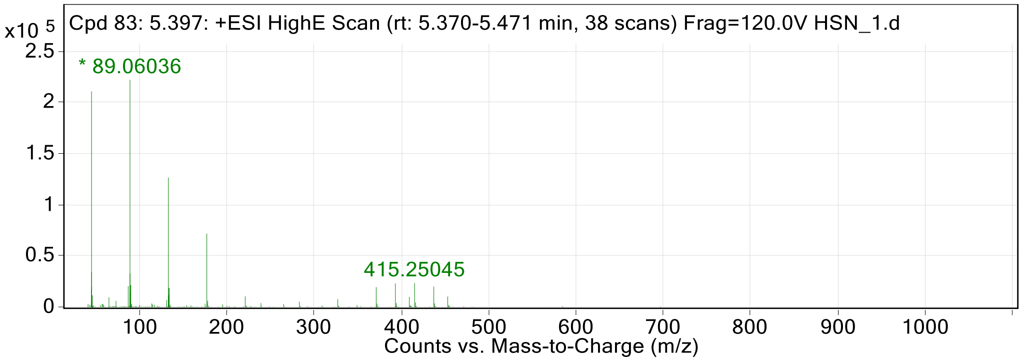

Peak List

m/z	z	Abund
45.03471		210675.61
45.09234		33900.69
89.06036	1	221902.03
89.14168	1	32666.34
89.16424		20915.02
90.06183	1	21433.58
133.08622	1	126449.98

m/z	z	Abund
177.10996	1	71638.02
393.20635	1	22937.8
415.25045	1	23346.31

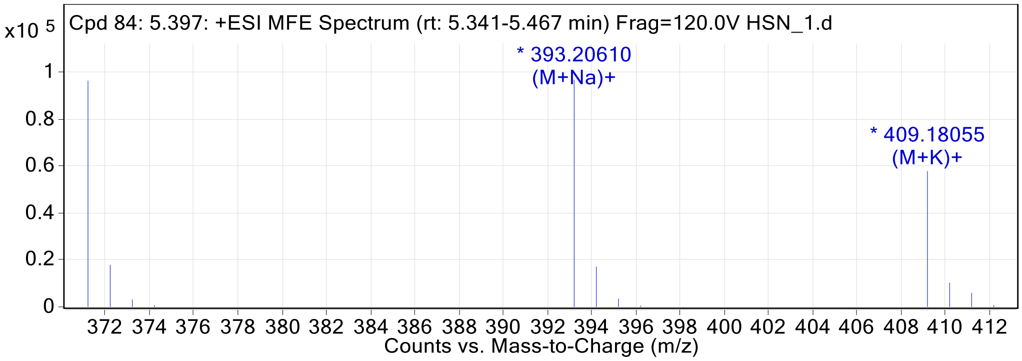

Peak List

m/z	z	Abund	Ion
371.22411	1	96245.38	(M+H)+
372.22801	1	17903.81	(M+H)+
373.22982	1	3160.53	(M+H)+
393.2061	1	96684.58	(M+Na)+
394.20995	1	17156.41	(M+Na)+
395.21203	1	3492.14	(M+Na)+
409.18055	1	57814.48	(M+K)+
410.18361	1	10300.68	(M+K)+
411.18	1	5990.55	(M+K)+
412.18065	1	792.6	(M+K)+

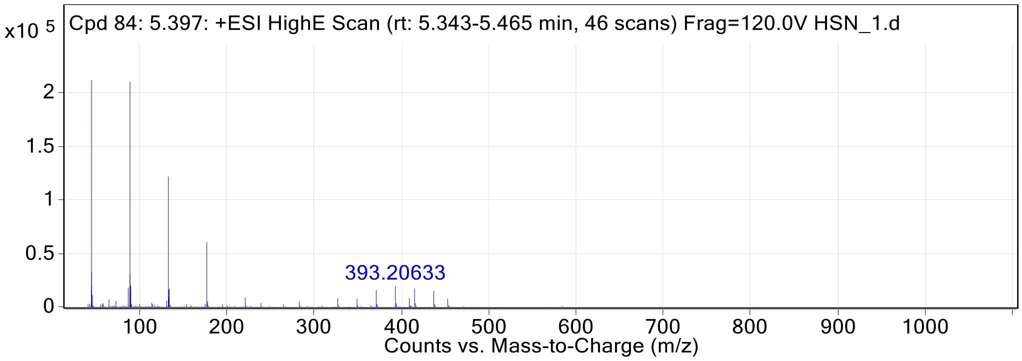

Peak List

m/z	z	Abund
45.03457	1	211974.91
45.09233		32246.46
45.10836		19157.94
89.06026	1	210439.77
89.14168		29690.67
89.16424		18825.8
90.06183	1	19780.87
133.08589	1	121817.73
177.10994	1	60594.96
393.20633	1	19087.65

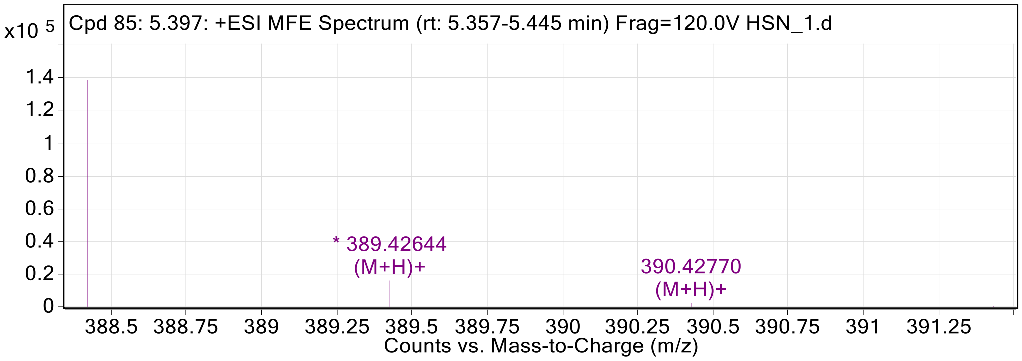

Peak List

m/z	z	Abund	Ion
388.42253	1	138813.42	(M+H)+
389.42644	1	16034.03	(M+H)+
390.4277	1	2356.46	(M+H)+
391.43227	1	151.81	(M+H)+

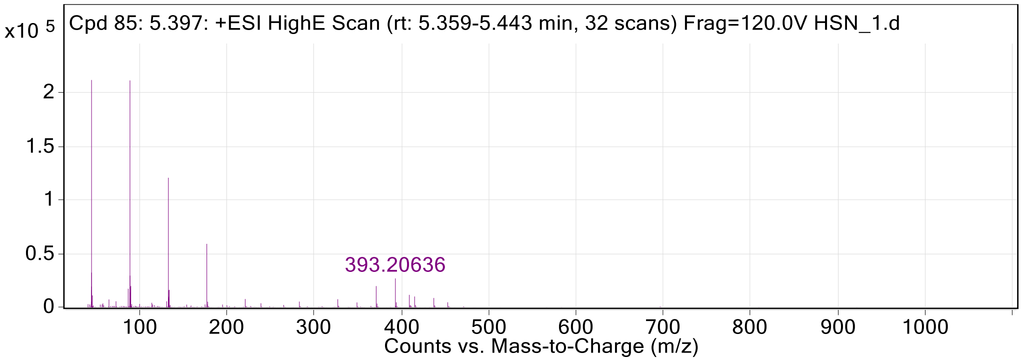

Peak List

m/z	z	Abund
45.03456	1	212010.72
45.09233		32154.7
45.10836		19136.59
89.0602	1	211609.94
89.14168		29308.16
90.06182	1	19631.44
133.08581	1	120714.19
177.1099	1	58996.81
371.22427	1	19572.59
393.20636	1	26714.85

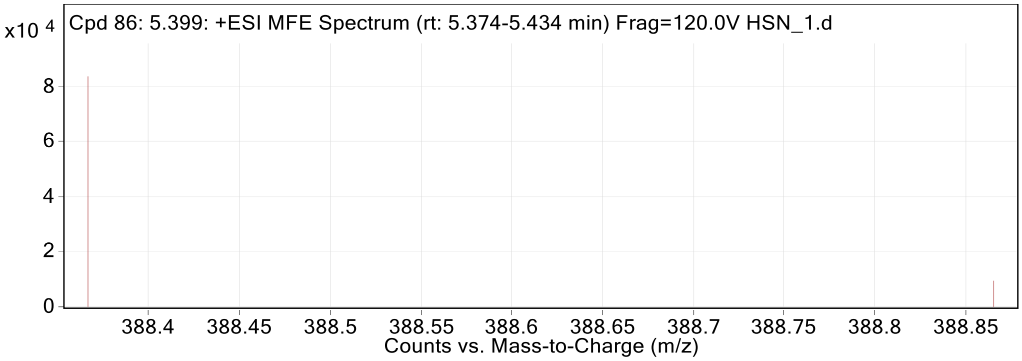

Peak List

m/z	z	Abund	Ion
388.36667	2	83757.98	(M+2H)+2

388.86539	2	9499.86	(M+2H)+2

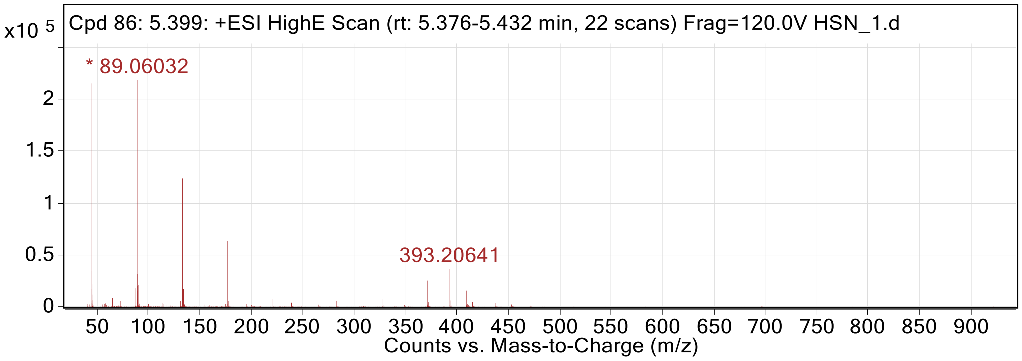

Peak List

m/z	z	Abund
45.03469	1	215452.2
45.09233		34431.76
45.10837		21513.93
89.06032	1	218851.89
89.14168	1	31517.01
90.06182	1	21014.4
133.08595	1	123713.83
177.10989	1	63473.26
371.2243	1	25098.46
393.20641	1	36469.22

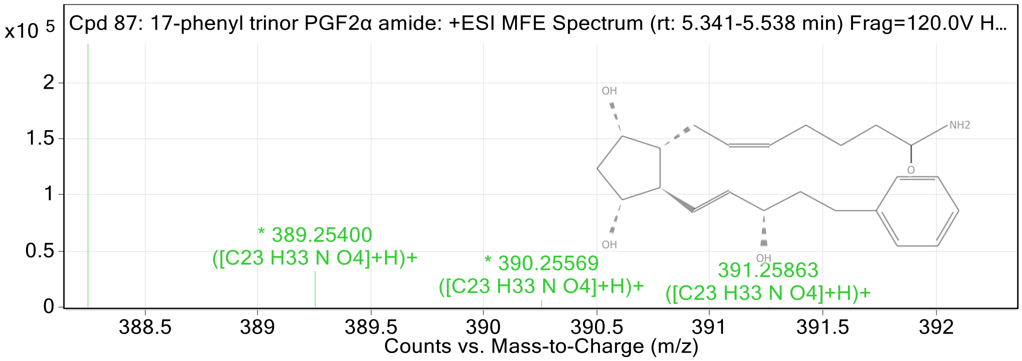

Peak List

m/z	z	Abund	Name	Formula	Ion	Score (DB)	Hits (DB)
388.24916	1	235119.25	17-phenyl trinor PGF2α amide	C23 H33 N O4	(M+H)+	74.55	1
389.254	1	31887.84		C23 H33 N O4	(M+H)+		
390.25569	1	6569.57		C23 H33 N O4	(M+H)+		
391.25863	1	440.45		C23 H33 N O4	(M+H)+		
392.25573	1	87.57		C23 H33 N O4	(M+H)+		

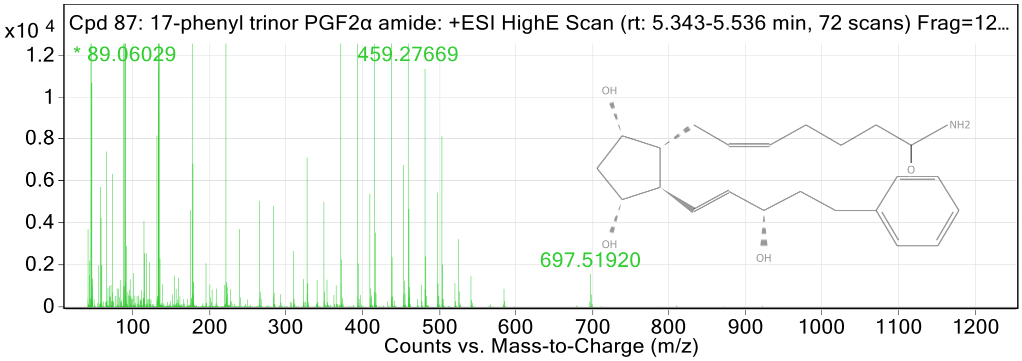

Peak List

m/z	z	Abund
45.03464	1	201566.42
45.09234		31353.32
87.04281		23097.31
89.06029	1	218612.39
89.14169		31563.49
89.16424		20289.15
90.06183	1	20892.52
133.08607	1	131428.94
177.11009	1	74992.58
459.27669	1	21827.55

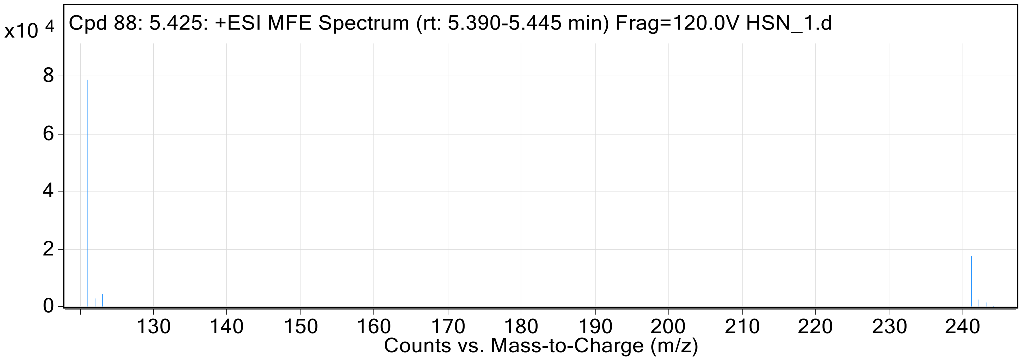

Peak List

m/z	z	Abund	Ion
121.0665	1	78721.11	(M+H)+
122.06907	1	2807.23	(M+H)+
123.06318	1	4318.69	(M+H)+
241.12625	1	17468.58	(2M+H)+
242.12979	1	2416.18	(2M+H)+
243.12244	1	1410.7	(2M+H)+
244.12353	1	217.25	(2M+H)+

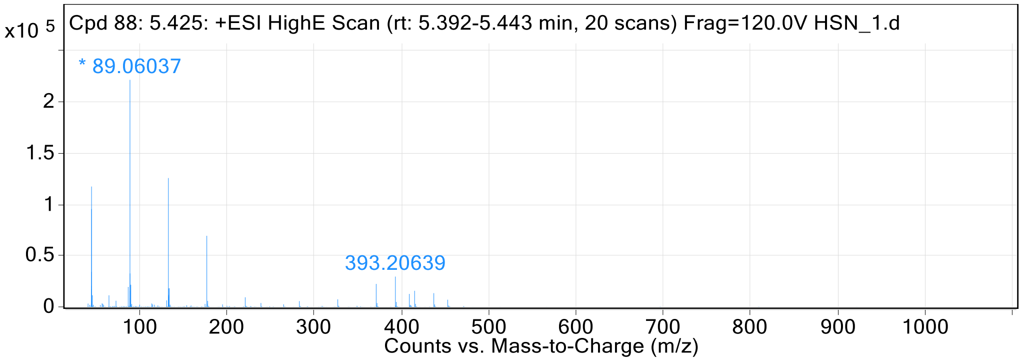

Peak List

m/z	z	Abund
45.03346		95506.83
45.03571		117504.73
45.09234		34149.36
89.06037	1	221698.3
89.14169	1	32650.31
90.06183	1	21665.72
133.08614	1	125819.66
177.10991	1	69407.67
371.22429	1	22418.59
393.20639	1	29584.56

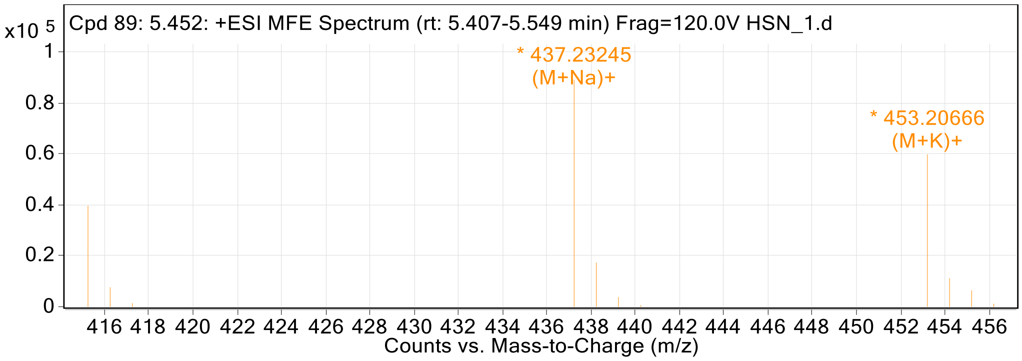

Peak List

m/z	z	Abund	Ion
415.25039	1	39641.27	(M+H)+
416.25349	1	7663.77	(M+H)+
417.25629	1	1439.47	(M+H)+
437.23245	1	88983.15	(M+Na)+
438.23551	1	17325.16	(M+Na)+
439.23694	1	3845.9	(M+Na)+
453.20666	1	59741.91	(M+K)+
454.20941	1	11239	(M+K)+
455.2073	1	6417.76	(M+K)+
456.20798	1	1199.88	(M+K)+

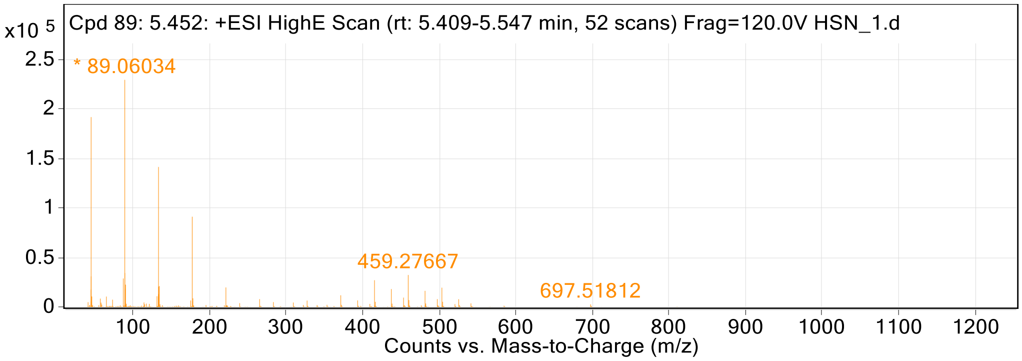

Peak List

m/z	z	Abund
45.03475		192077
45.09235		30852.03
87.04282	1	28755.92
89.06034	1	229754.91
89.14168		34275.16
90.06183	1	22486.05
133.0863	1	141577.59
177.11017	1	91269.55
415.25035	1	26987.6
459.27667	1	32279.83

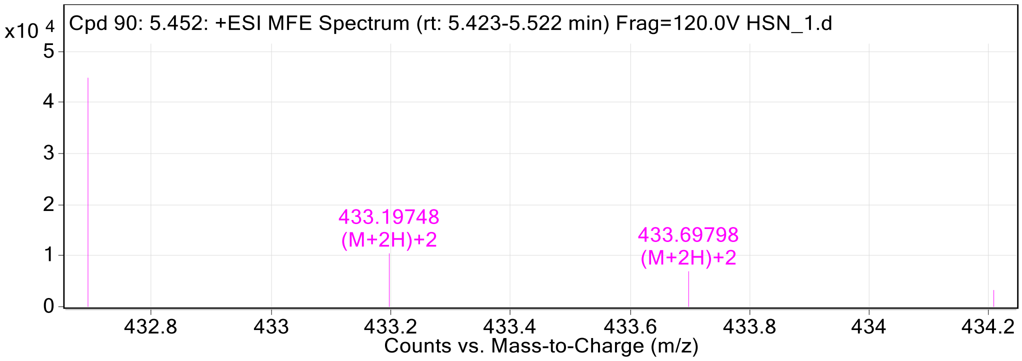

Peak List

m/z	z	Abund	Ion
432.69387	2	44851.73	(M+2H)+2
433.19748	2	10462.18	(M+2H)+2
433.69798	2	6951.67	(M+2H)+2
434.20804	2	3275.01	(M+2H)+2

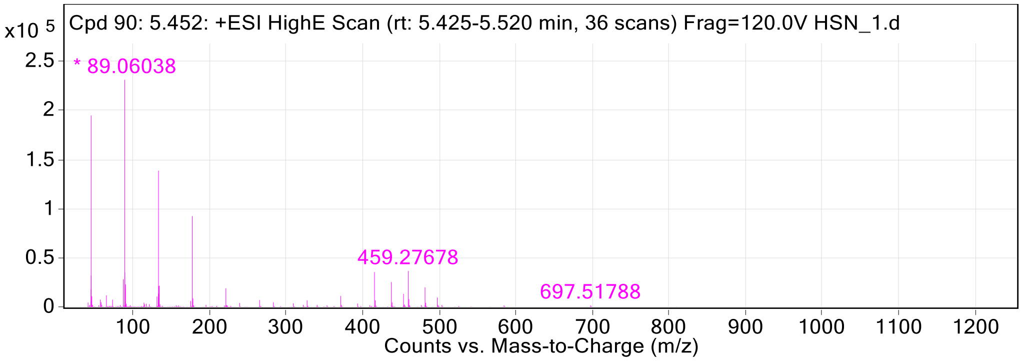

Peak List

m/z	z	Abund
45.03479		194975.44

45.09235		32007.21
87.04281	1	28069.54
89.06038	1	231323.22
89.14168		34942.71
133.0865	1	138770.94
177.11013	1	92502.13
415.25036	1	35454.09
437.23213	1	25309.3
459.27678	1	36571.95

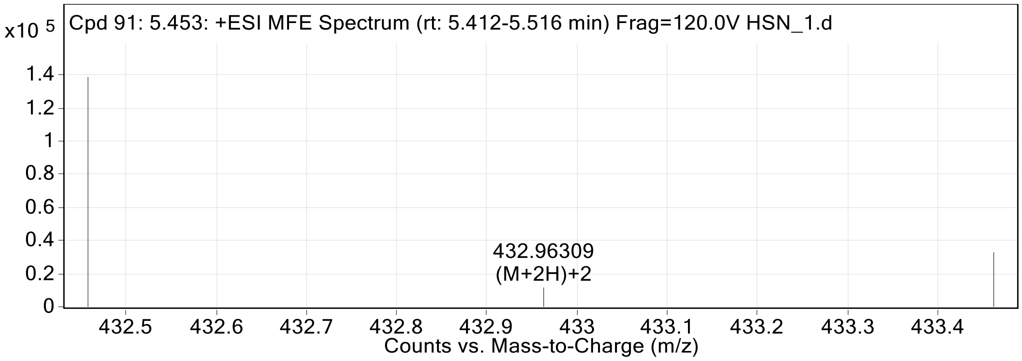

Peak List

m/z	z	Abund	Ion
432.4579	2	138671.17	(M+2H)+2
432.96309	2	11667.97	(M+2H)+2
433.46198	2	32919.83	(M+2H)+2

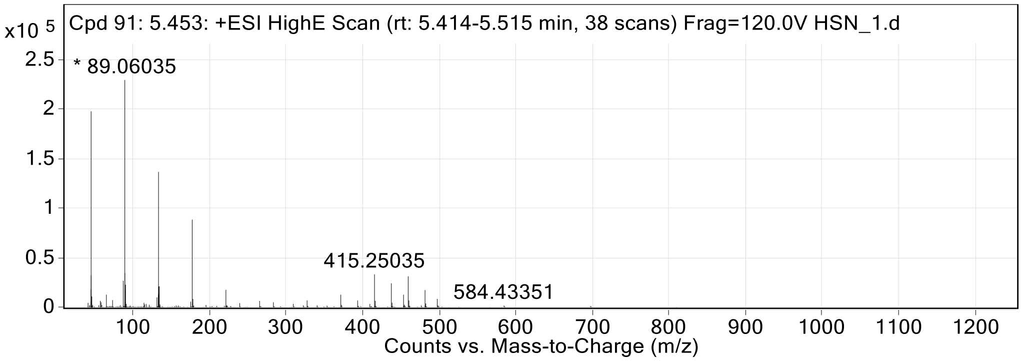

Peak List

m/z	z	Abund
45.03475		197578.7
45.09235		32022.61
87.04281	1	26529.17
89.06035	1	229201.89
89.14168		34298.38
133.08644	1	136411.47
177.11011	1	88317.71
415.25035	1	33064.06
437.23211	1	23958.11
459.27674	1	31008.2

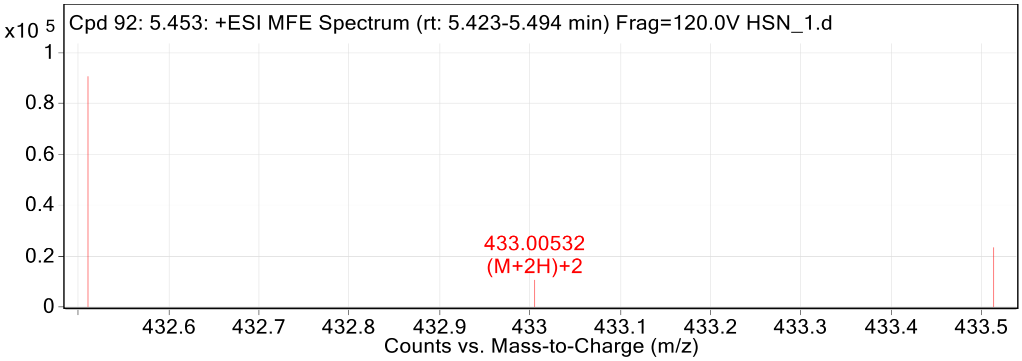

Peak List

m/z	z	Abund	Ion
432.51047	2	90552.83	(M+2H)+2
433.00532	2	10627.44	(M+2H)+2
433.51373	2	23338.26	(M+2H)+2

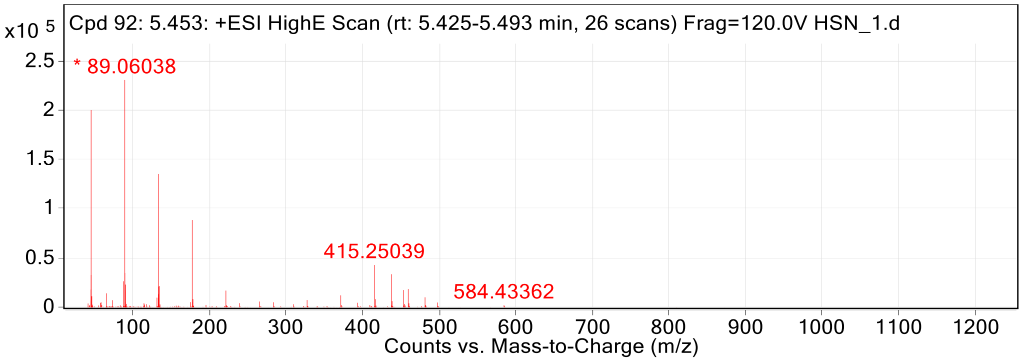

Peak List

m/z	z	Abund
45.03477		199821
45.09235		32630.71
87.04281	1	26116.01
89.06038	1	230359.48
89.14167		34712.21
90.06184	1	22791.62
133.08652	1	135239.56
177.11006	1	88445.3
415.25039	1	42789.14
437.23211	1	33300.88

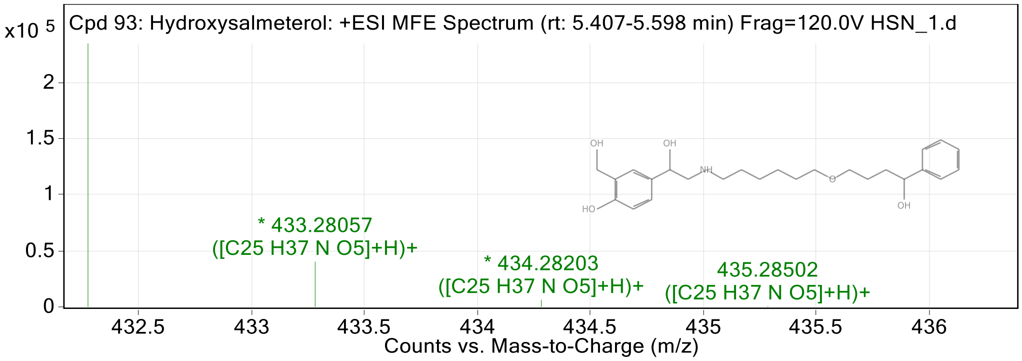

Peak List

m/z	z	Abund	Name	Formula	Ion	Score (DB)	Hits (DB)
432.2748	1	235119.88	Hydroxysalmeterol	C25 H37 N O5	(M+H)+	78.15	2
433.28057	1	40420.2		C25 H37 N O5	(M+H)+		

434.28203	1	6372.64		C25 H37 N O5	(M+H)+		
435.28502	1	661.02		C25 H37 N O5	(M+H)+		
436.28537	1	74.8		C25 H37 N O5	(M+H)+		

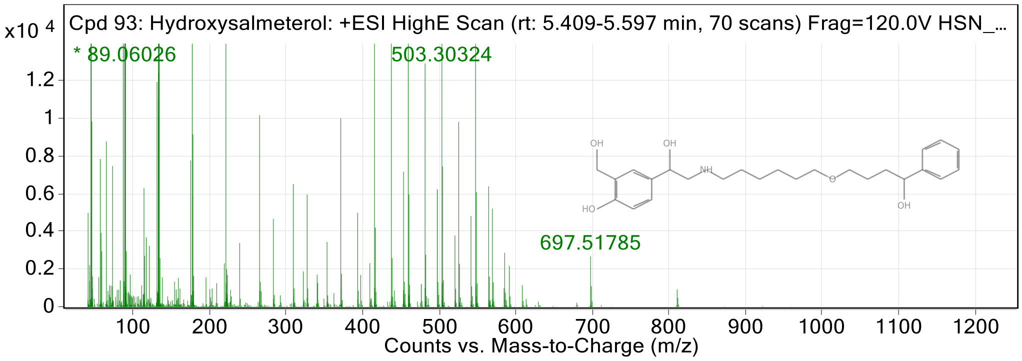

Peak List

m/z	z	Abund
45.03471	1	182419.28
45.09235		28878.3
87.04282	1	31771.71
89.06026	1	229969.34
89.14169		33425.05
133.08602	1	150199.52
177.1102	1	94653.2
221.13581	1	22467.86
459.27667	1	28070.69
503.30324	1	28871.29

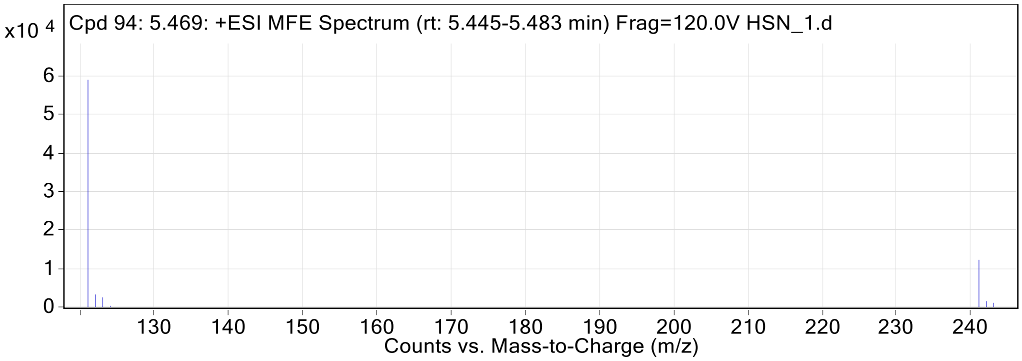

Peak List

m/z	z	Abund	Ion
121.06619	1	59002.54	(M+H)+
122.06963	1	3240.53	(M+H)+
123.06219	1	2480.75	(M+H)+
124.06433	1	294.5	(M+H)+
241.12663	1	12252.26	(2M+H)+
242.12928	1	1510.54	(2M+H)+
243.12328	1	1076.45	(2M+H)+

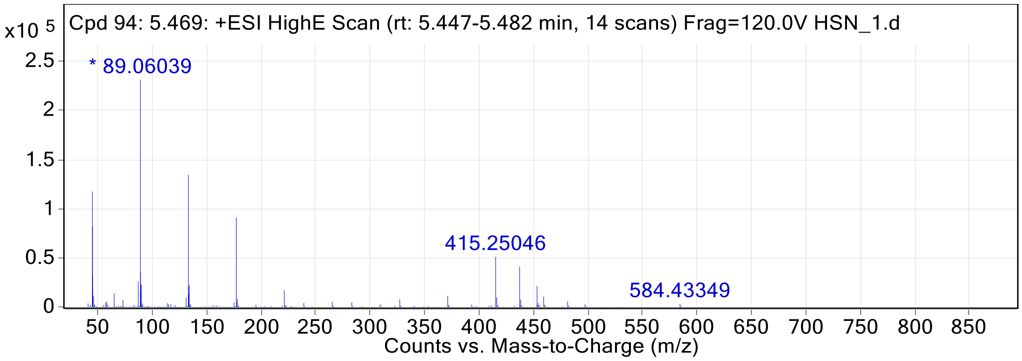

Peak List

m/z	z	Abund
45.03333		81640.38
45.03578		117525.23
45.09235		32614.01
87.04281	1	26134.69
89.06039	1	231256.98
89.14168		34858.39
133.08665		134521.73
177.11004	1	90911.55
415.25046	1	51031.59
437.23214	1	40964.24

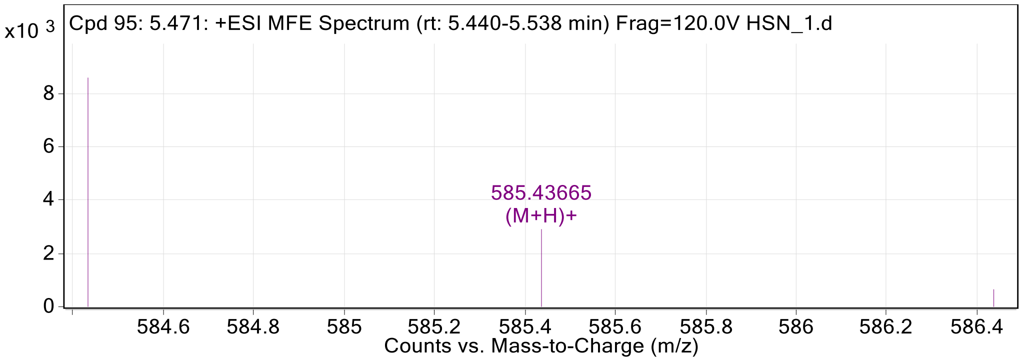

Peak List

m/z	z	Abund	Ion
584.43348	1	8592.84	(M+H)+
585.43665	1	2910.19	(M+H)+
586.43721	1	654.98	(M+H)+

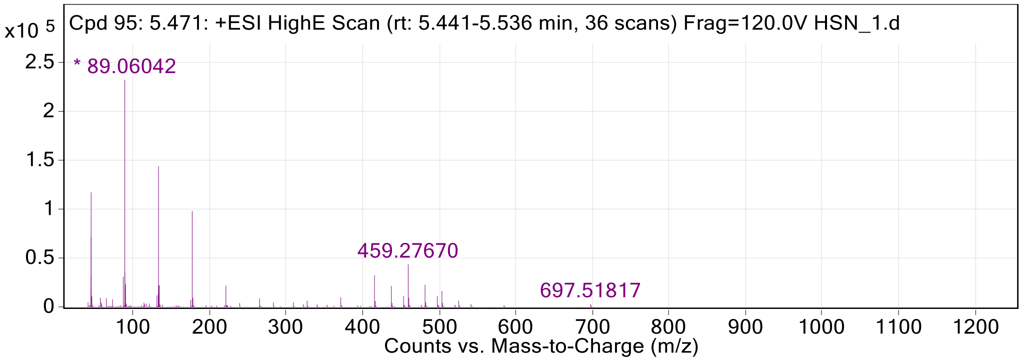

Peak List

m/z	z	Abund
45.03329		71772.31
45.03576		117521.27

45.09235		31303.33
87.04282		31017.79
89.06042	1	232791.61
89.14169		35613.22
133.08649	1	144254.45
177.1102	1	98353.4
415.25039	1	32301.68
459.2767	1	43648.67

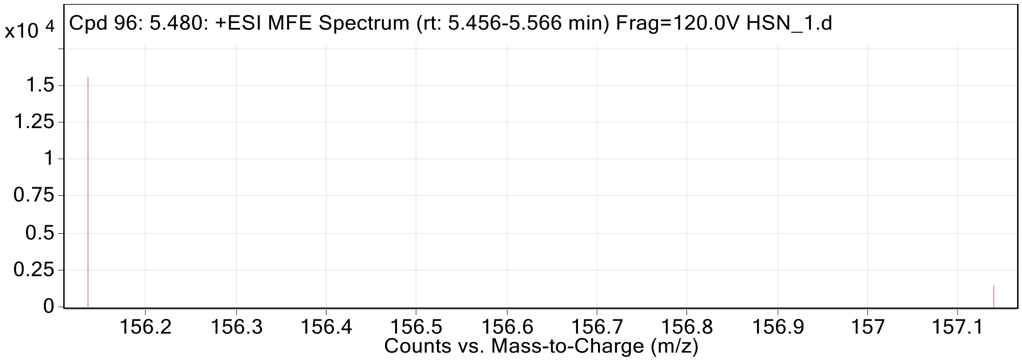

Peak List

m/z	z	Abund	Ion
156.13589	1	15560.83	(M+H)+
157.13942	1	1432.28	(M+H)+

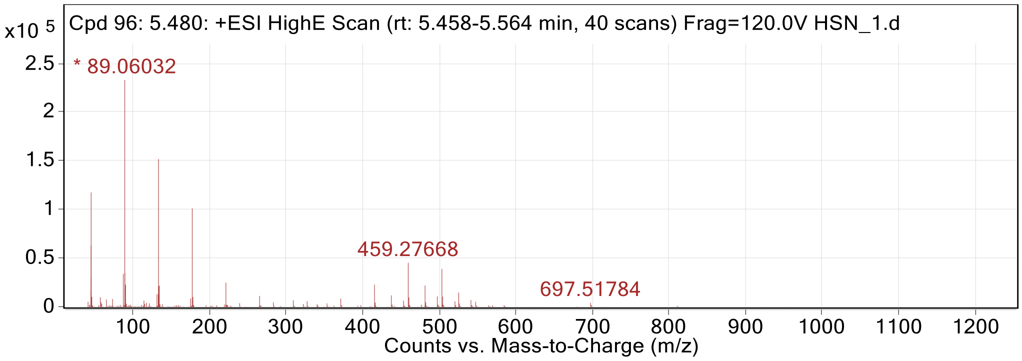

Peak List

m/z	z	Abund
45.03326		62812.74
45.03559		117499.5
45.09236		28931.23
87.04282	1	33746.45
89.06032	1	232967.66
89.14169		34686.15
133.08621	1	151721.45
177.11025	1	101018.23
459.27668	1	45119.31
503.3034	1	38920.62

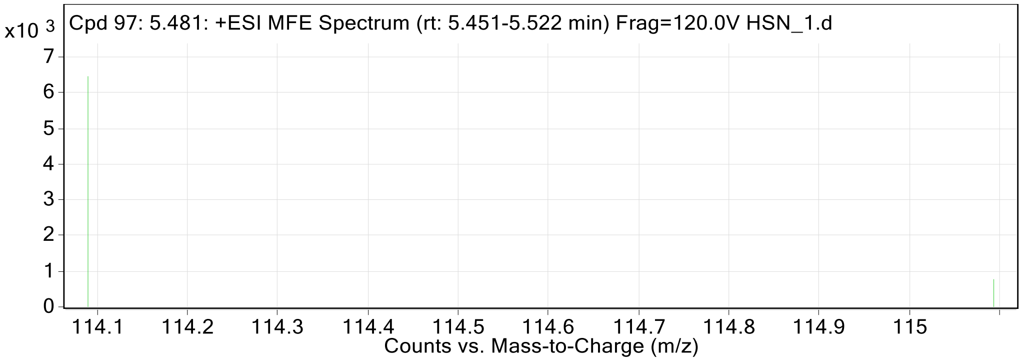

Peak List

m/z	z	Abund	Ion
114.08961	1	6463.29	(M+H)+
115.09351	1	775.41	(M+H)+

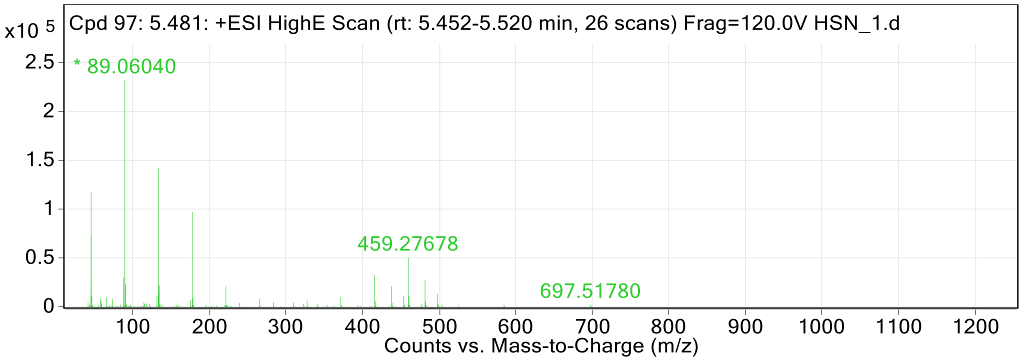

Peak List

m/z	z	Abund
45.0333		73275.39
45.03575		117533.64
45.09235		31442.54
87.04281	1	29812.8
89.0604	1	232638.58
89.14168		35298.32
133.08654	1	141880.72
177.11019	1	97265.29
415.25039	1	32781.64
459.27678	1	50638.08

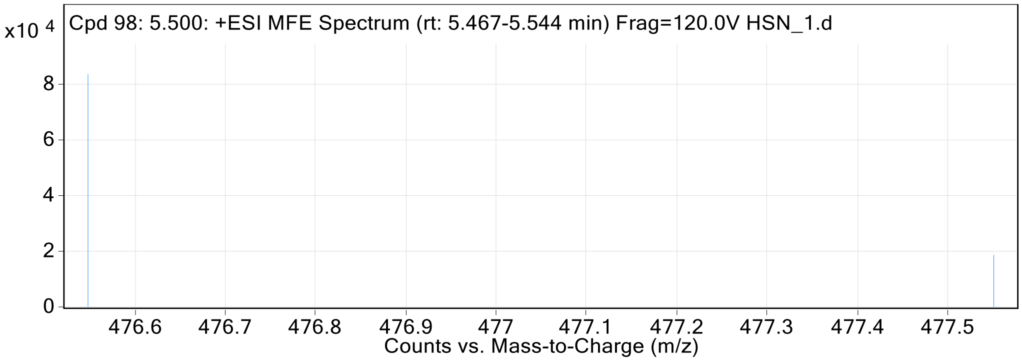

Peak List

m/z	z	Abund	Ion
476.54796	1	83755.84	(M+H)+
477.55119	1	18722.11	(M+H)+

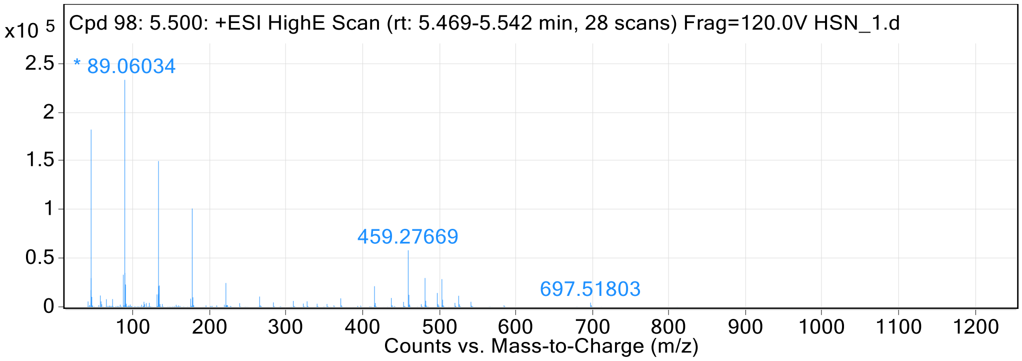
x10 5
Cpd 98: 5.500: +ESI HighE Scan (rt: 5.469-5.542 min, 28 scans) Frag=120.0V HSN_1.d
* 89.06034
459.27669
697.51803
2.5
2
1.5
1
0.5
0
100 200 300 400 500 600 700 800 900 1000 1100 1200
Counts vs. Mass-to-Charge (m/z)

Peak List

m/z	z	Abund
45.0348		182099.98
45.09236		29509.89
87.04282		33059
89.06034	1	233149.34
89.1417		34937.25
133.0863	1	149754.47
177.11026	1	100996.21
459.27669	1	58109.09
481.25815	1	29637.54
503.30336	1	28482.79

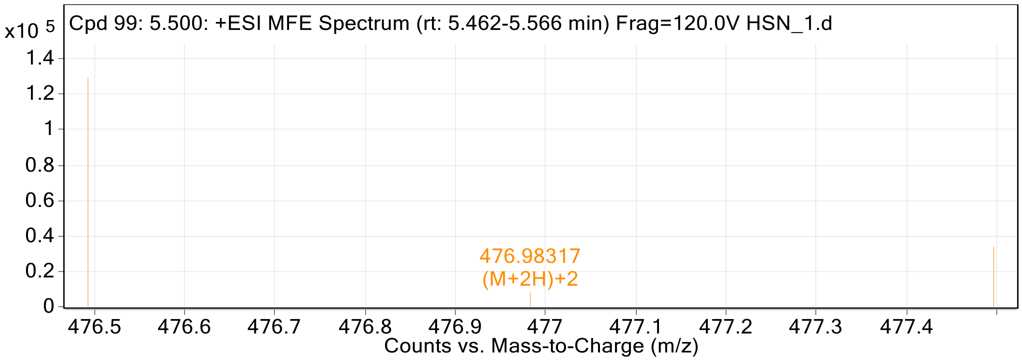

Peak List

m/z	z	Abund	Ion
476.49302	2	129127.23	(M+2H)+2
476.98317	2	8107.05	(M+2H)+2
477.49663	2	34005.85	(M+2H)+2

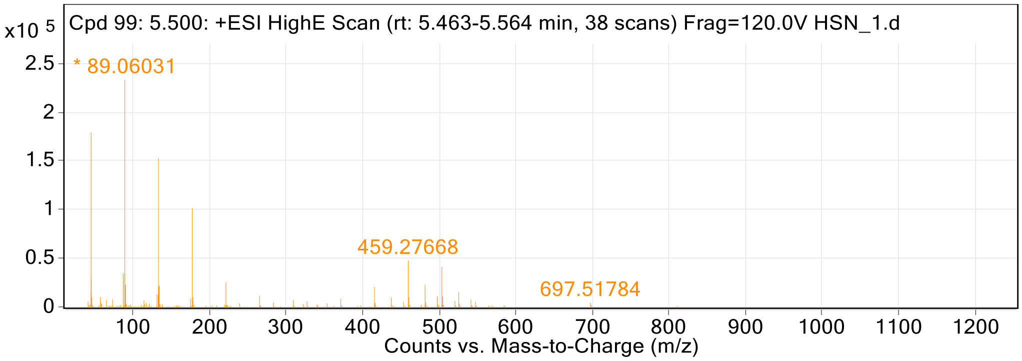

Peak List

m/z	z	Abund
45.03477		179048.88
45.09236		28605.53
87.04283	1	34116.8
89.06031	1	233010.11
89.14169		34554.53
133.08618	1	152686.22
177.11026	1	101450.98
221.13579	1	25158.41
459.27668	1	47481.81
503.3034	1	40969.07

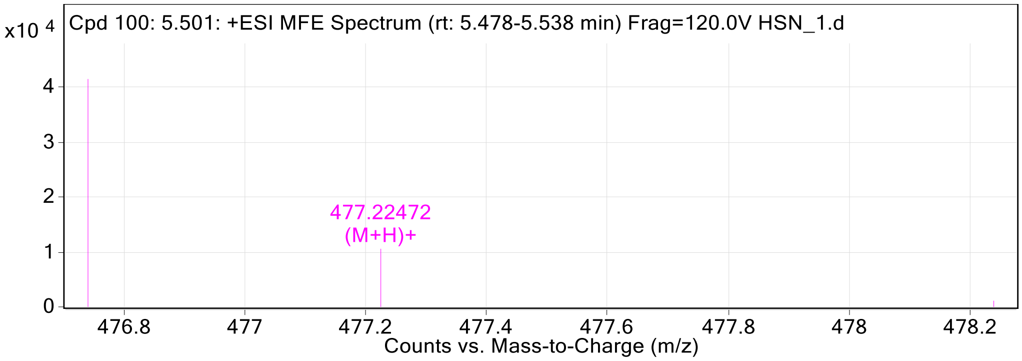

Peak List

m/z	z	Abund	Ion
476.73974	1	41549.64	(M+H)+
477.22472	1	10593.57	(M+H)+
478.23992	1	1121.77	(M+H)+

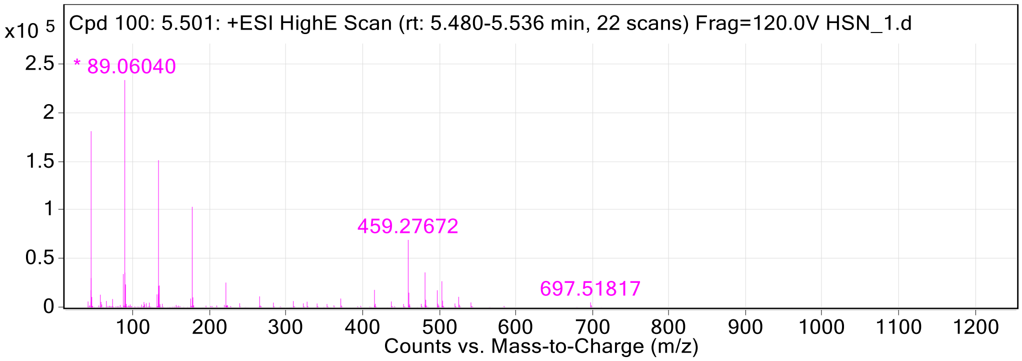

Peak List

m/z	z	Abund
45.03484		181095.44
45.09236		29864.11
87.04281		34057.84
89.0604	1	233717
89.1417		35578.22
133.08634	1	151194.23
177.1103	1	103218.05
459.27672	1	69169.49
481.25815	1	35685
503.30319	1	26609.35

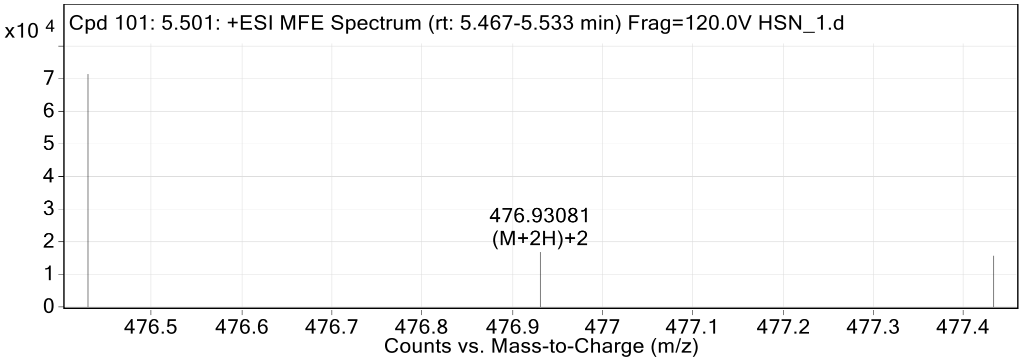

Peak List

m/z	z	Abund	Ion
476.42989	2	71486.06	(M+2H)+2
476.93081	2	16876.66	(M+2H)+2

477.4332	2	15724.6	(M+2H)+2

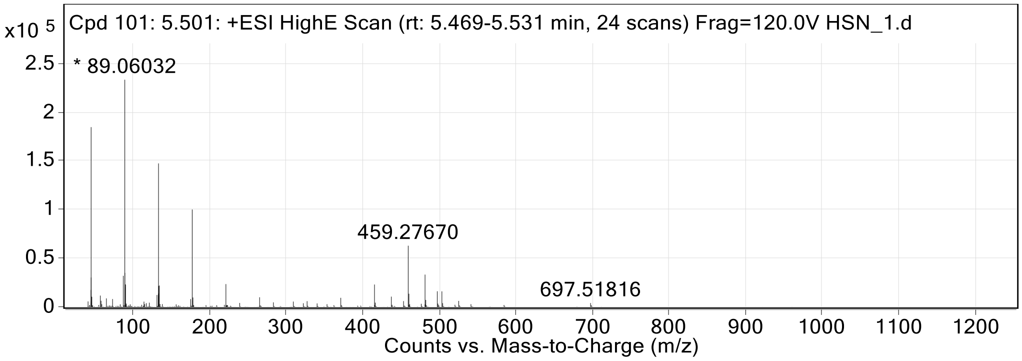

Peak List

m/z	z	Abund
45.03478		184382.06
45.09236		30038.65
87.04282		31822.91
89.06032	1	233022.13
89.14169		34783.46
133.08635	1	147122.97
177.11023	1	99803.37
221.13574	1	23279.59
459.2767	1	62668.86
481.25813	1	33018.73

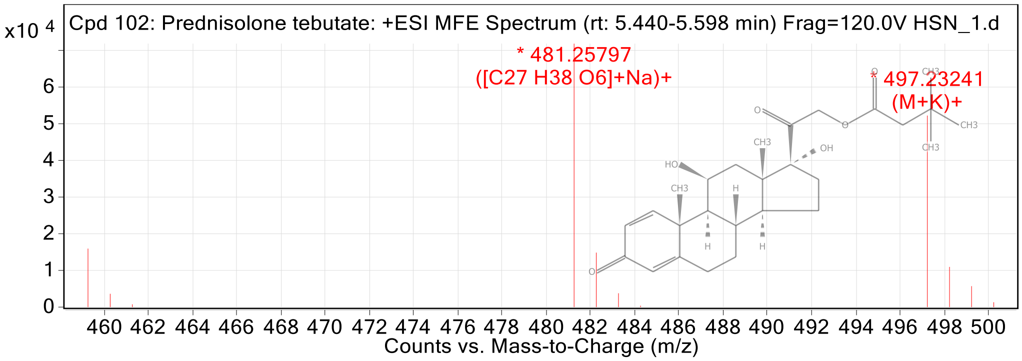

Peak List

m/z	z	Abund	Name	Formula	Ion	Score (DB)	Hits (DB)
459.27604	1	15907.36			(M+H)+		
460.28052	1	3619.27			(M+H)+		
461.28224	1	752.15			(M+H)+		
481.25797	1	71667.52	Prednisolone tebutate	C27 H38 O6	(M+Na)+	79.69	2
482.26152	1	14815.77		C27 H38 O6	(M+Na)+		
483.26528	1	3761.21		C27 H38 O6	(M+Na)+		
497.23241	1	52100.39			(M+K)+		
498.23533	1	10926.9			(M+K)+		
499.23283	1	5696.47			(M+K)+		
500.23689	1	1320.87			(M+K)+		

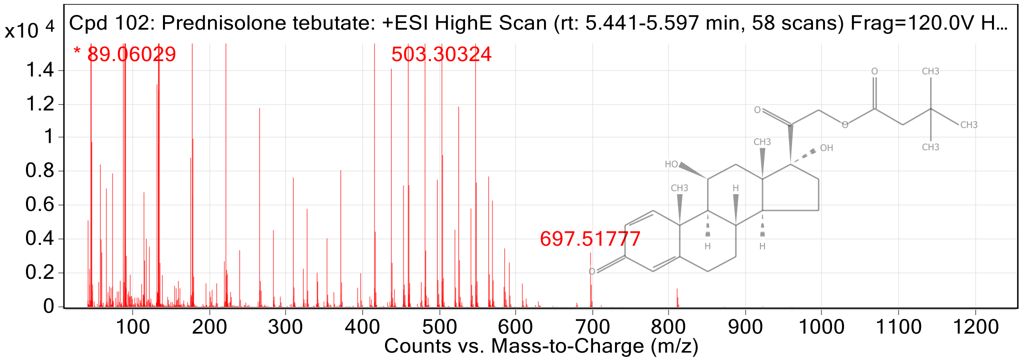

Peak List

m/z	z	Abund
45.03326		59494.23
45.03551		117438.43
45.09236		28377.58
87.04282	1	34609.33
89.06029	1	232189.89
89.14169		34150.26
133.08606	1	155302.59
177.11024	1	100882.18
459.27667	1	33878.42
503.30324	1	34844.66

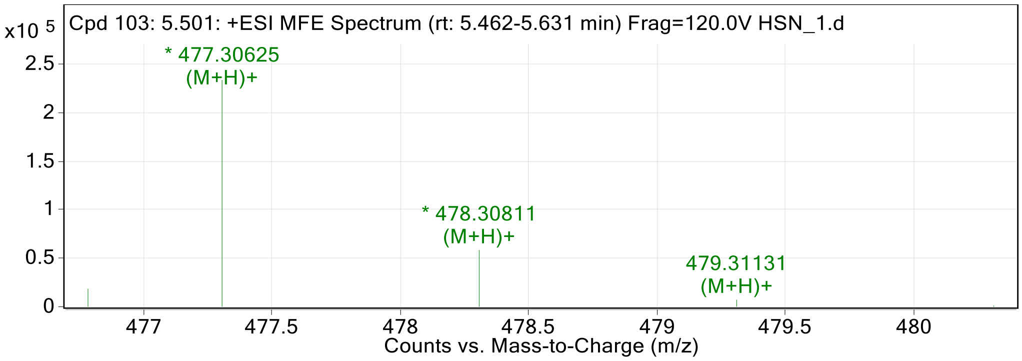

Peak List

m/z	z	Abund	Ion
476.78387	1	18564.83	(M+H)+
477.30625	1	233837.42	(M+H)+
478.30811	1	58495.17	(M+H)+
479.31131	1	7355.13	(M+H)+
480.3132	1	1053.29	(M+H)+

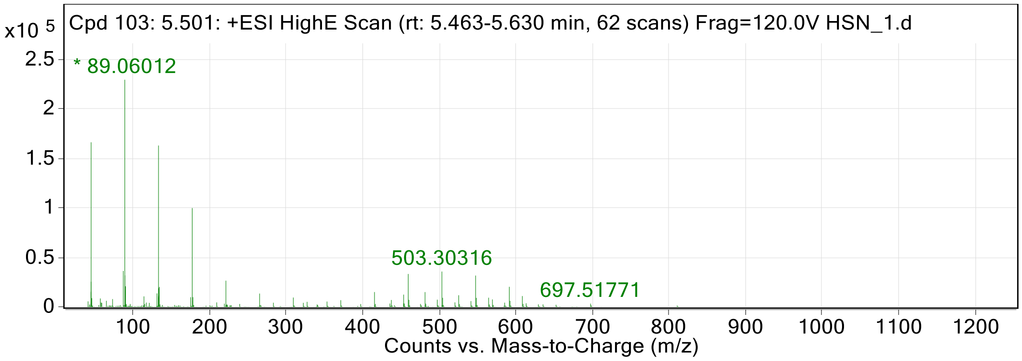

Peak List

m/z	z	Abund

45.03463	1	166069.44
87.04283		36216.41
89.06012	1	229211.36
89.1417		31722.39
133.08565	1	162827.11
177.11023	1	99674.3
221.13584	1	26373.21
459.27665	1	33126.09
503.30316	1	35578.57
547.32931	1	31525.99

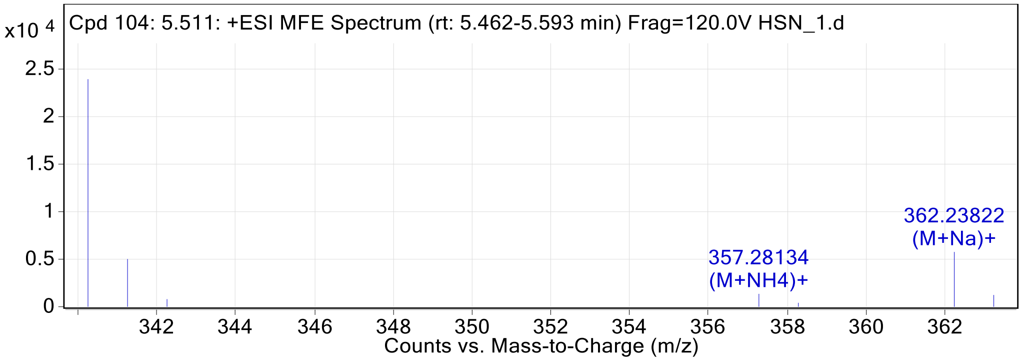

Peak List

m/z	z	Abund	Ion
340.25626	1	23906.04	(M+H)+
341.2596	1	5010.44	(M+H)+
342.26167	1	798.92	(M+H)+
357.28134	1	1355.51	(M+NH4)+
358.28172	1	414.38	(M+NH4)+
362.23822	1	5758.2	(M+Na)+
363.24093	1	1237.44	(M+Na)+

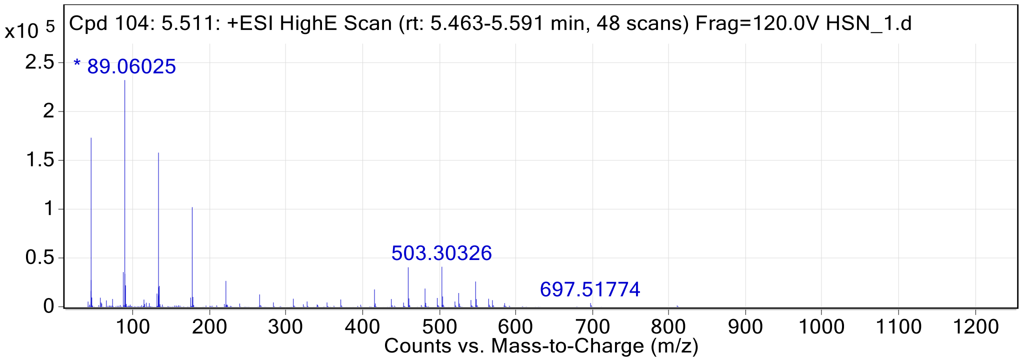

Peak List

m/z	z	Abund
45.03473		173317.13
45.09236		27423.44
87.04283	1	35582.86
89.06025	1	232392.73
89.1417		33758.23
133.08597	1	158012.41
177.11027	1	102126.98
221.13582	1	26538.6
459.27666	1	40513.3
503.30326	1	41037.18

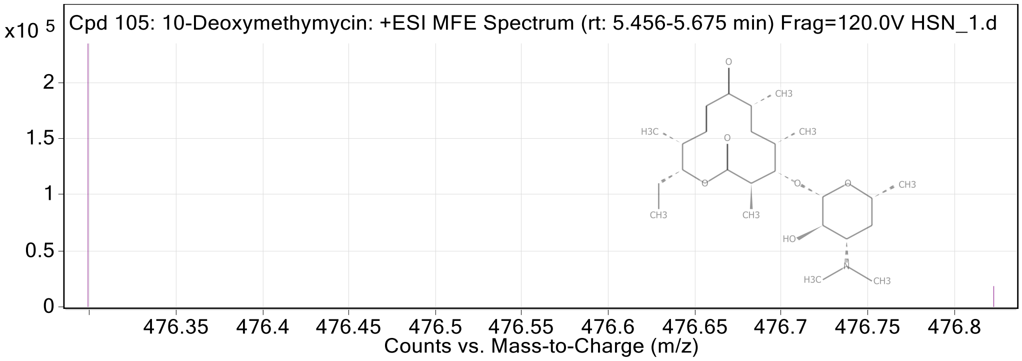

Peak List

m/z	z	Abund	Name	Formula	Ion	Score (DB)	Hits (DB)
476.29903	1	235125.38	10-Deoxymethymycin	C25 H43 N O6	(M+Na)+	0	3
476.82289	1	18543.33		C25 H43 N O6	(M+Na)+		

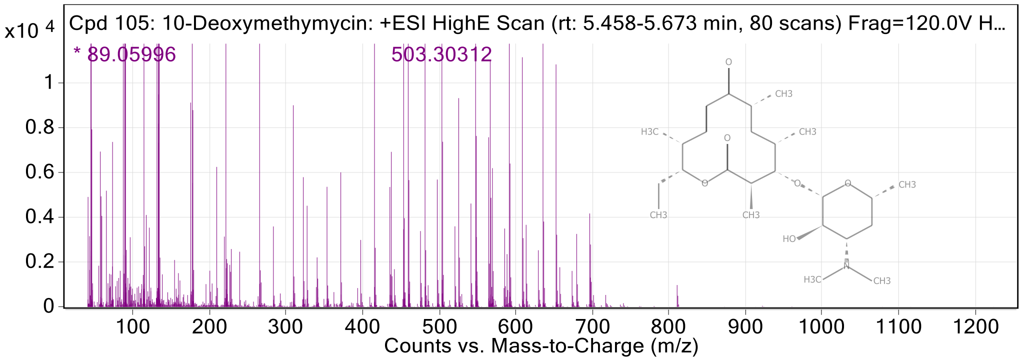

Peak List

m/z	z	Abund
45.03349		72658.26
45.03531		87975.83
87.04283		34849.6
89.05996	1	221756.45
89.1417		28884.94
133.08539	1	159920.95
177.11018	1	91840.88
459.27664	1	26589.35
503.30312	1	28918.64
547.32922	1	26942.34

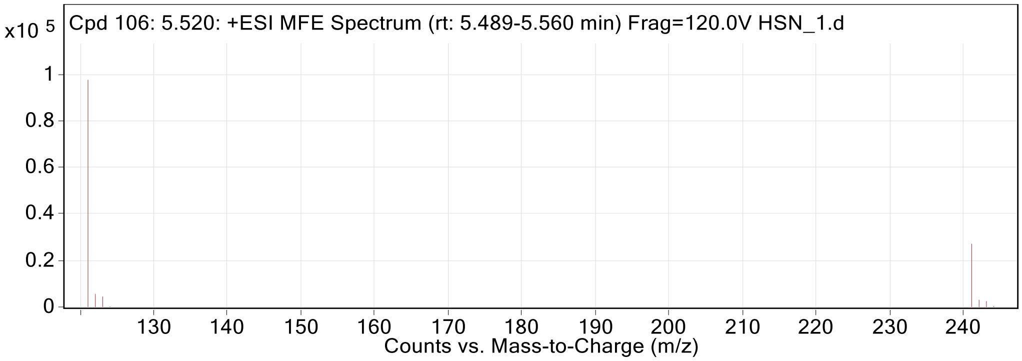

Peak List

m/z	z	Abund	Ion
121.0663	1	97517.98	(M+H)+
122.06949	1	5546.76	(M+H)+
123.06221	1	4473.44	(M+H)+

124.0661	1	206.77	(M+H)+
241.12655	1	27135.22	(2M+H)+
242.12924	1	3032.9	(2M+H)+
243.12245	1	2505.58	(2M+H)+
244.12457	1	362.98	(2M+H)+

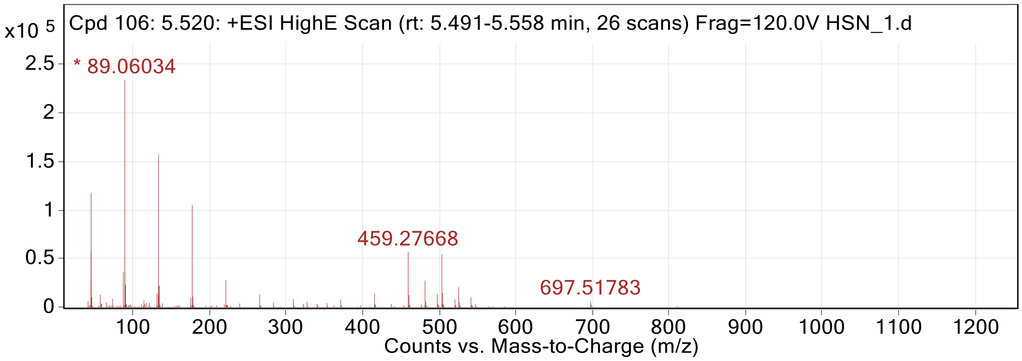

Peak List

m/z	z	Abund
45.03323		57588.21
45.03559		117502.95
45.09236		28203.55
87.04282	1	36446.74
89.06034	1	233813.11
89.1417		35183.43
133.08616	1	156775.81
177.11032	1	105128.07
459.27668	1	56474.71
503.30343	1	54102.06

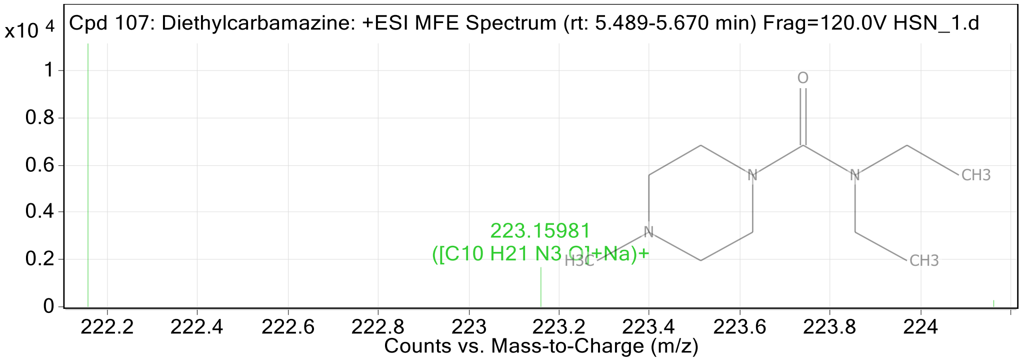

Peak List

m/z	z	Abund	Name	Formula	Ion	Score (DB)	Hits (DB)
222.15749	1	11161.97	Diethylcarbamazine	C10 H21 N3 O	(M+Na)+	96.18	1
223.15981	1	1681.04		C10 H21 N3 O	(M+Na)+		
224.16185	1	282.56		C10 H21 N3 O	(M+Na)+		

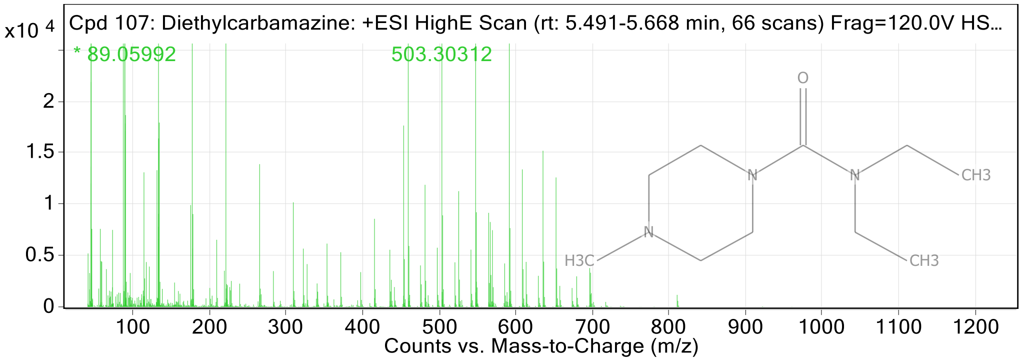

Peak List

m/z	z	Abund
45.03352		69997.33
45.03522		85269.86
87.04283		36760.26
89.05992	1	221487.53
89.1417		28458.53
133.08526	1	165206.47
177.1102	1	93363.76
459.27663	1	27585.55
503.30312	1	34906.8
547.32922	1	32475.27

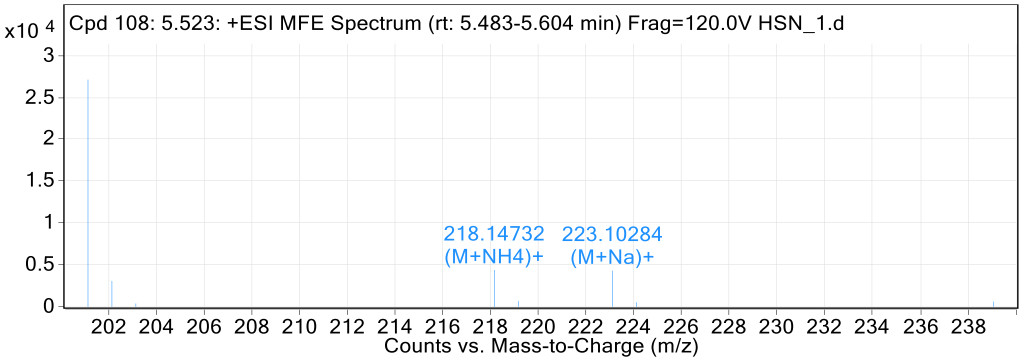

Peak List

m/z	z	Abund	Ion
201.1208	1	27116.45	(M+H)+
202.12349	1	3086.95	(M+H)+
203.12698	1	396.17	(M+H)+
218.14732	1	4384.35	(M+NH4)+
219.14973	1	693.28	(M+NH4)+
223.10284	1	4318.1	(M+Na)+
224.10511	1	541	(M+Na)+
239.0763	1	633.2	(M+K)+

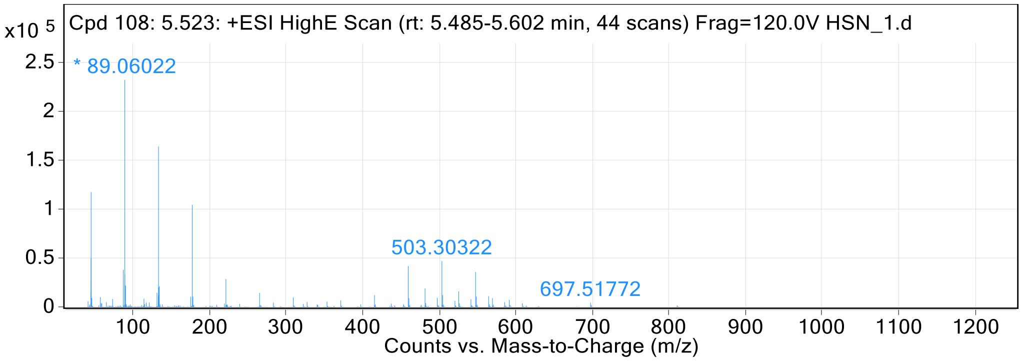

Peak List

m/z	z	Abund
45.03322		49603.46
45.03536		117378.33
87.04283		37839.56
89.06022	1	232079.34
89.1417		33375.84
133.08581	1	164033.64
177.11029	1	104222.68
459.27669	1	41713.39
503.30322	1	46844.04
547.32947	1	35704.02

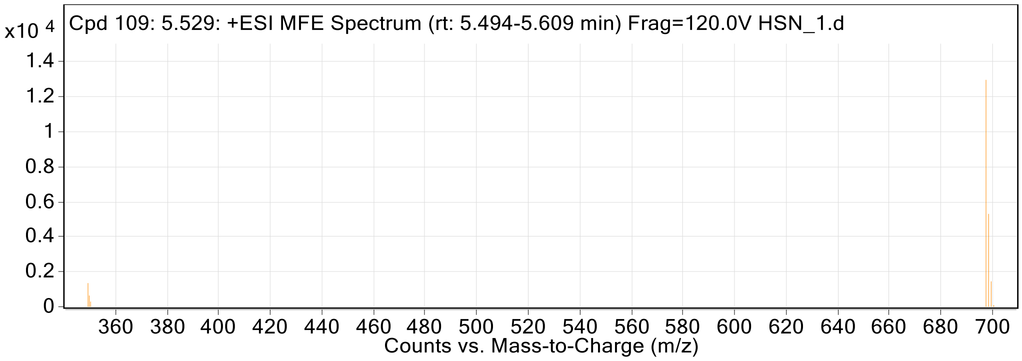

Peak List

m/z	z	Abund	Ion
349.26257	2	1354.03	(M+2H)+2
349.76236	2	645.69	(M+2H)+2
350.2634	2	299.08	(M+2H)+2
697.51812	1	12974.97	(M+H)+
698.52076	1	5310.31	(M+H)+
699.52149	1	1452.57	(M+H)+
700.52905	1	94.35	(M+H)+

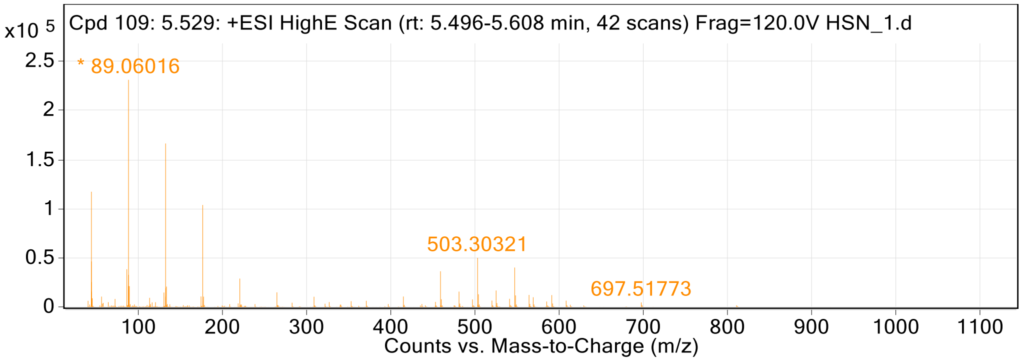

Peak List

m/z	z	Abund
45.0332		46240.54
45.03525		117326.36
87.04284		38327.43
89.06016	1	231272.27
89.1417		32618.34
133.08568	1	166450.45
177.11028	1	103799.82
459.27666	1	36266.47
503.30321	1	49949.21
547.32943	1	40073.18

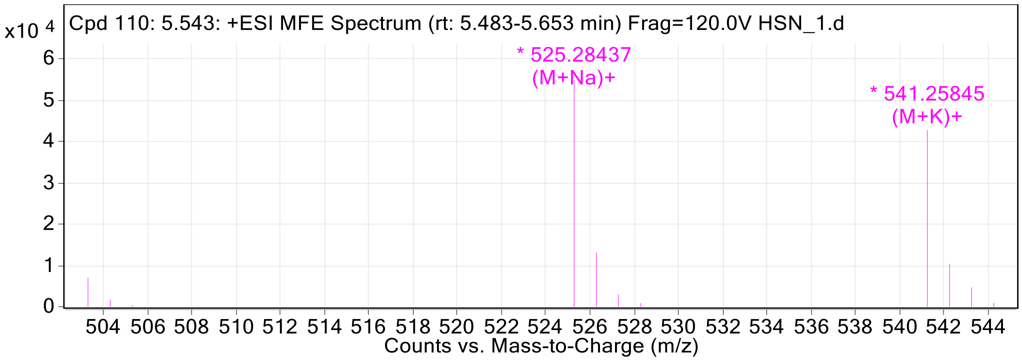

Peak List

m/z	z	Abund	Ion
503.30209	1	6999.43	(M+H)+
504.30698	1	1720.14	(M+H)+
525.28437	1	55011.1	(M+Na)+
526.28806	1	13176.72	(M+Na)+
527.28941	1	2971.63	(M+Na)+
528.29047	1	859.75	(M+Na)+
541.25845	1	42836.52	(M+K)+
542.26142	1	10373.41	(M+K)+
543.25908	1	4727	(M+K)+
544.26138	1	949.51	(M+K)+

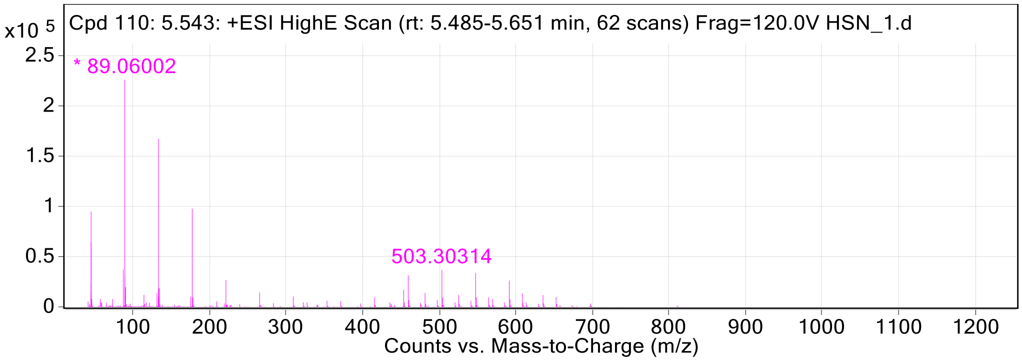

Peak List

m/z	z	Abund
45.0335		63989.43
45.03525		94562.52
87.04284		37328.18
89.06002	1	225347.42
89.1417		30016.4
133.0854	1	166859.88
177.11023	1	97520.98
459.27666	1	31420.71
503.30314	1	36622.76
547.32925	1	33714.6

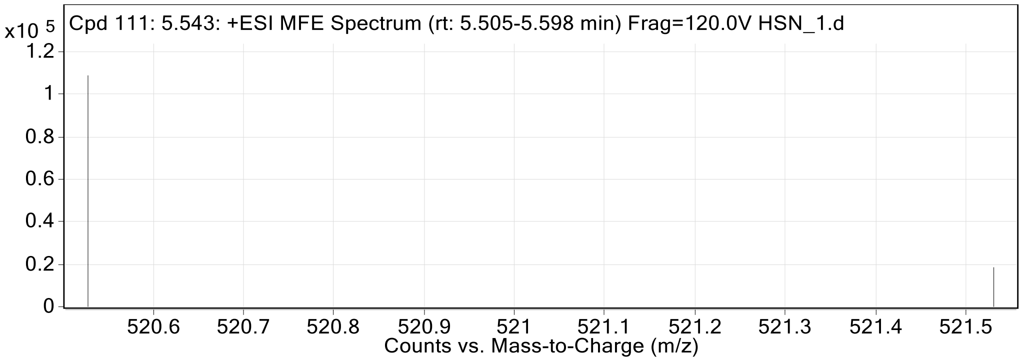

Peak List

m/z	z	Abund	Ion
520.52751	1	108849.51	(M+H)+
521.53111	1	18578.78	(M+H)+

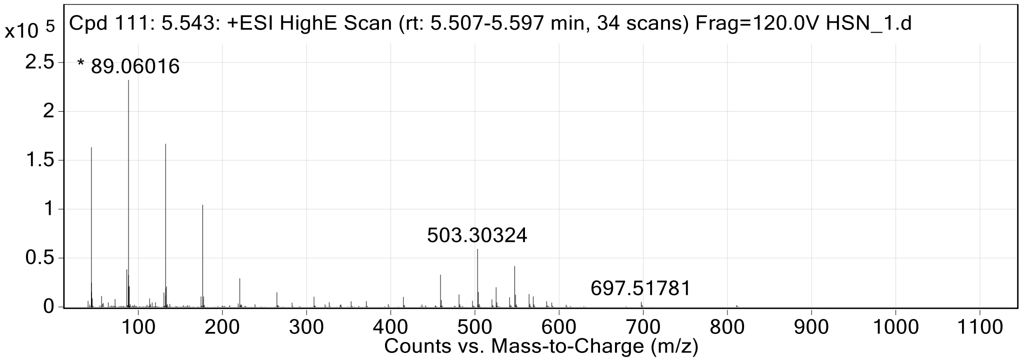

Peak List

m/z	z	Abund
45.03467	1	163237.39
87.04283	1	38392.39
89.06016	1	232044.88

89.14171		32644.4
133.08568	1	166765.94
177.11029	1	104474.5
221.13584	1	29226.1
459.27662	1	32981.39
503.30324	1	59403.94
547.32952	1	41931.17

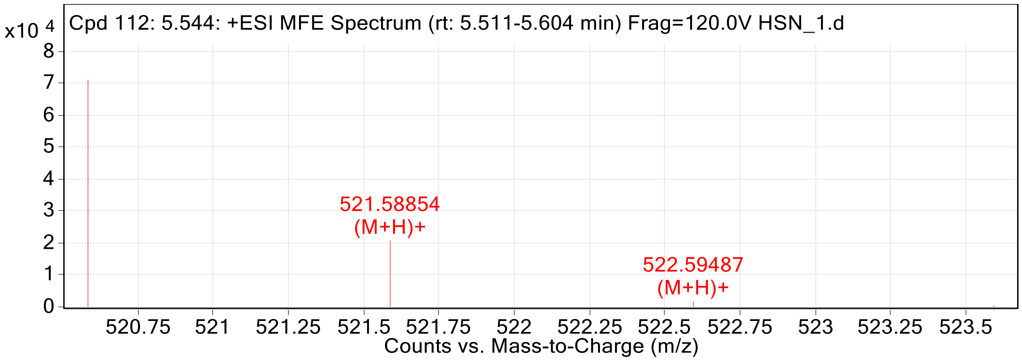

Peak List

m/z	z	Abund	Ion
520.58532	1	71170.55	(M+H)+
521.58854	1	20792.59	(M+H)+
522.59487	1	1808.05	(M+H)+
523.59242	1	411.03	(M+H)+

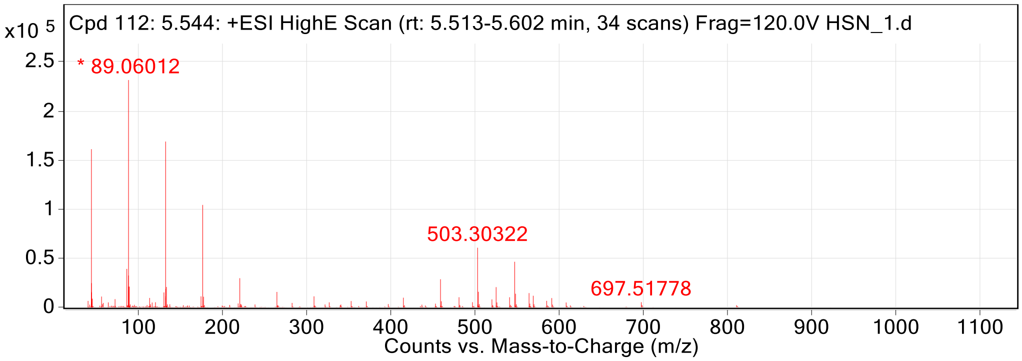

Peak List

m/z	z	Abund
45.03464	1	160982.86
87.04284		38960.11
89.06012	1	231451.78
89.14171		32152.66
133.08558	1	168758.13
177.11029	1	104133.83
221.13584	1	29451.81
459.27658	1	28384.04
503.30322	1	60531.69
547.32947	1	46205.2

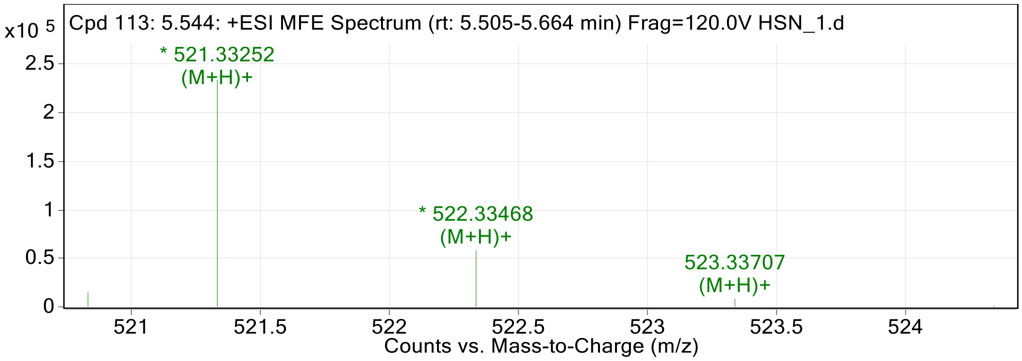
Cpd 113: 5.544: +ESI MFE Spectrum (rt: 5.505-5.664 min) Frag=120.0V HSN_1.d
x10 5
* 521.33252
(M+H)+
* 522.33468
(M+H)+
523.33707
(M+H)+
Counts vs. Mass-to-Charge (m/z)

Peak List

m/z	z	Abund	Ion
520.83198	1	15236.43	(M+H)+
521.33252	1	234121.03	(M+H)+
522.33468	1	58276.09	(M+H)+
523.33707	1	8298.77	(M+H)+
524.33921	1	1345.38	(M+H)+

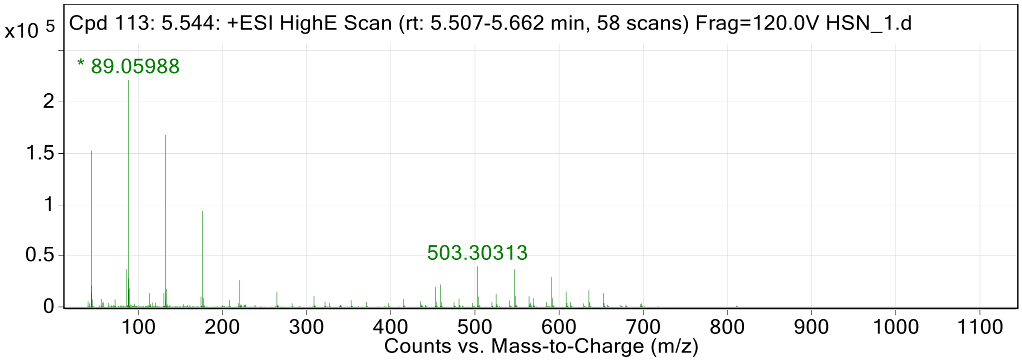

Peak List

m/z	z	Abund
45.03441	1	152777.72
87.04284		37191.94
89.05988	1	221676.95
89.14171		27929.16
133.08515	1	168049.86
177.11019	1	93639.2
221.13587	1	26062.56
503.30313	1	39548.87
547.32922	1	36727.45
591.35518	1	29404.45

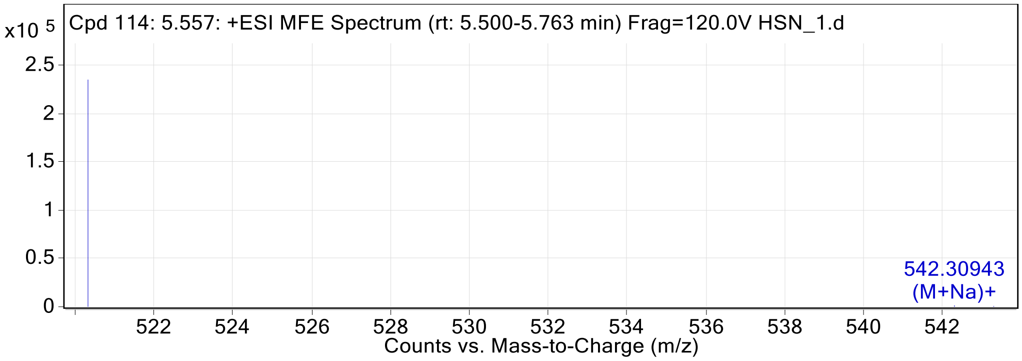

Peak List

m/z	z	Abund	Ion
520.32684	1	235119.38	(M+H)+
542.30943	1	1492.24	(M+Na)+
543.30716	1	814.14	(M+Na)+

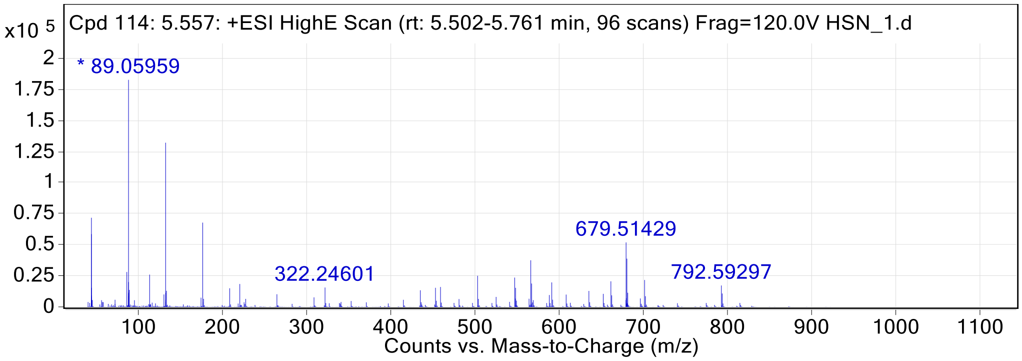

Peak List

m/z	z	Abund
45.0334		71700.94
45.03506		58526.72
87.04284		28064.2
89.05959	1	182612.08
114.08958		25949.44
133.08494	1	132074.89
177.11014	1	67780.59
566.42588	1	37501.11
679.51429	1	51843.08
680.51211	1	38794.64

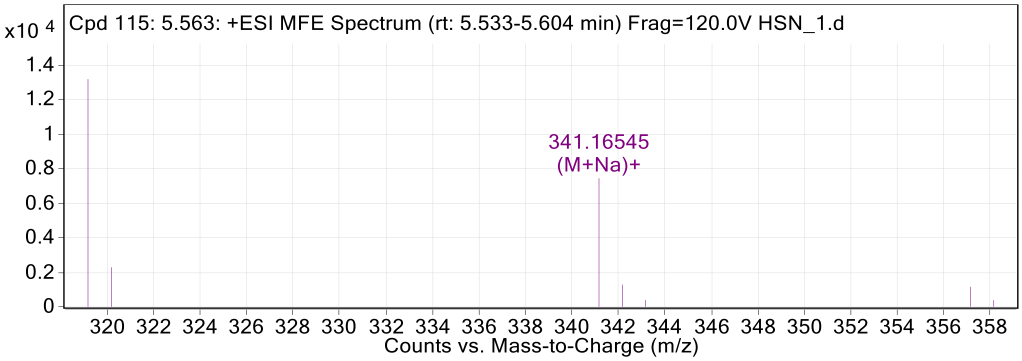

Peak List

m/z	z	Abund	Ion
319.1833	1	13175.65	(M+H)+
320.18637	1	2286.89	(M+H)+
341.16545	1	7439.66	(M+Na)+
342.16804	1	1279.9	(M+Na)+
343.17	1	385.84	(M+Na)+
357.13978	1	1159.89	(M+K)+
358.14851	1	382.36	(M+K)+

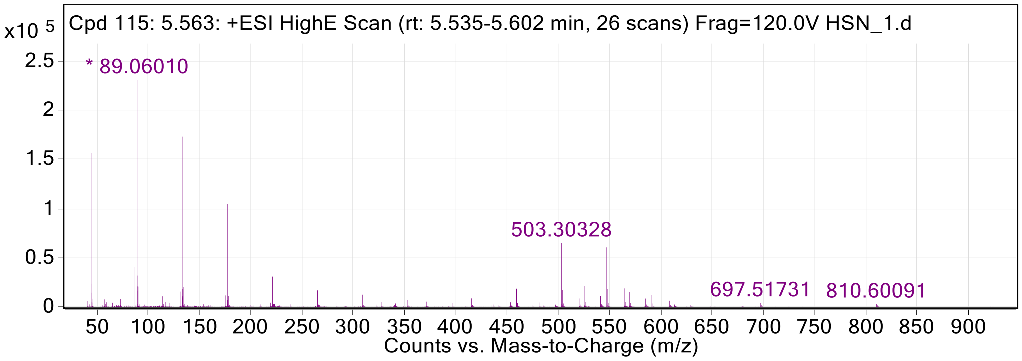

Peak List

m/z	z	Abund
45.03462	1	156687.22
45.09237		23482.17
87.04284	1	40513.15
89.0601	1	230823.28
89.14171		31709.58
133.08547	1	173186.73
177.1103	1	104692.59
221.13589	1	30667.46
503.30328	1	64680.75
547.32947	1	60422.18

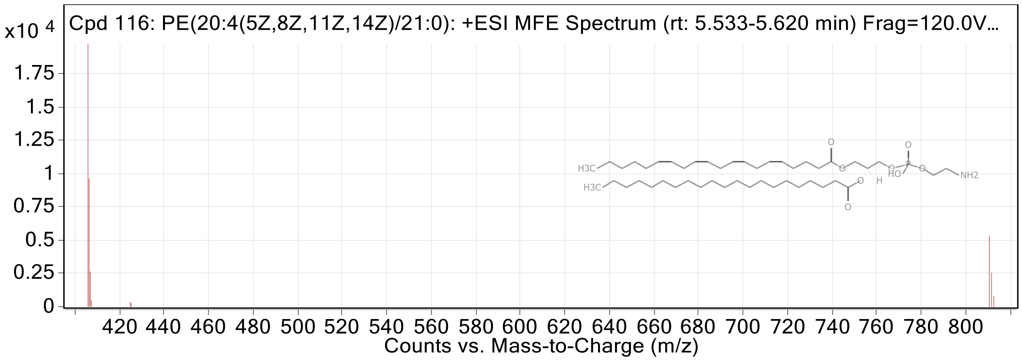

Peak List

m/z	z	Abund	Name	Formula	Ion	Score (DB)	Hits (DB)
405.80347	2	19738.65	PE(20:4(5Z,8Z,11Z,14Z)/21:0)	C46 H84 N O8 P	(M+2H)+2	97.86	4
406.30514	2	9637.04		C46 H84 N O8 P	(M+2H)+2		
406.80608	2	2652.16		C46 H84 N O8 P	(M+2H)+2		
407.30807	2	519.03		C46 H84 N O8 P	(M+2H)+2		
424.77799	2	364.5			(M+HK)+2		
425.28182	2	266.83			(M+HK)+2		
810.60105	1	5341.58			(M+H)+		
811.60432	1	2563.32			(M+H)+		
812.60604	1	780.45			(M+H)+		

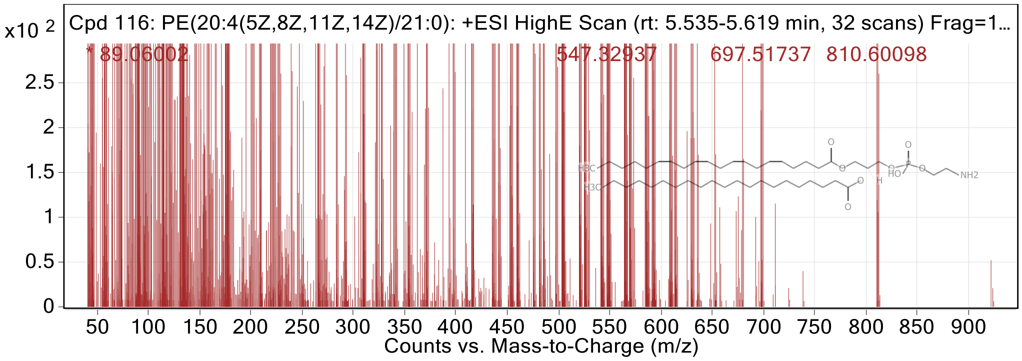

Peak List

m/z	z	Abund
45.03456	1	153660.02
87.04284		40244.7
89.06002	1	228523.05
89.14171		30489.01
133.08531	1	174952.41
177.11027	1	102395.73
221.1359	1	29847.8
503.30323	1	55592.06
547.32937	1	57727.71
591.35537	1	28262.38

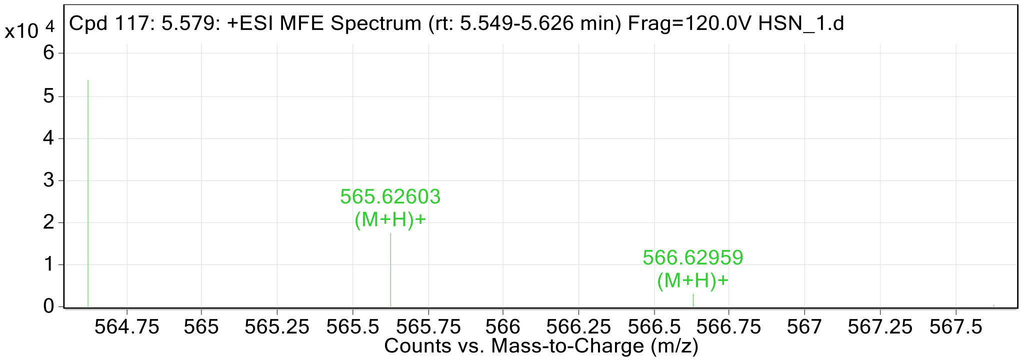

Peak List

m/z	z	Abund	Ion
564.62228	1	53840.98	(M+H)+
565.62603	1	17502.62	(M+H)+
566.62959	1	3035.76	(M+H)+
567.62657	1	445.25	(M+H)+

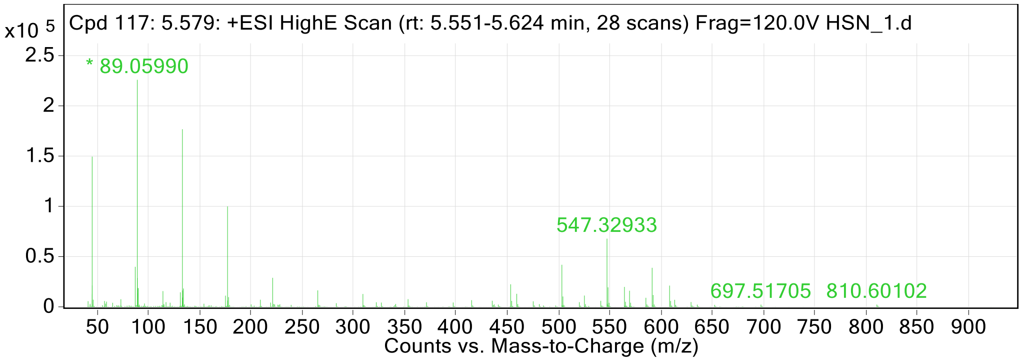

Peak List

m/z	z	Abund
45.03445	1	149682.22

87.04285		39909.82
89.0599	1	226223.2
89.14171		28810.15
133.08511	1	176992.14
177.11021	1	100055.64
221.13589	1	28934.76
503.30298	1	41912.54
547.32933	1	67965.84
591.35538	1	38914.18

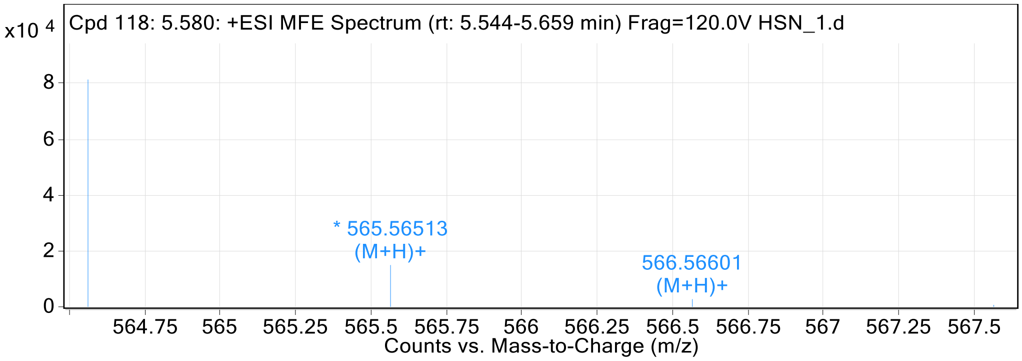

Peak List

m/z	z	Abund	Ion
564.56183	1	81336.55	(M+H)+
565.56513	1	14950.92	(M+H)+
566.56601	1	2743.8	(M+H)+
567.56568	1	695.75	(M+H)+

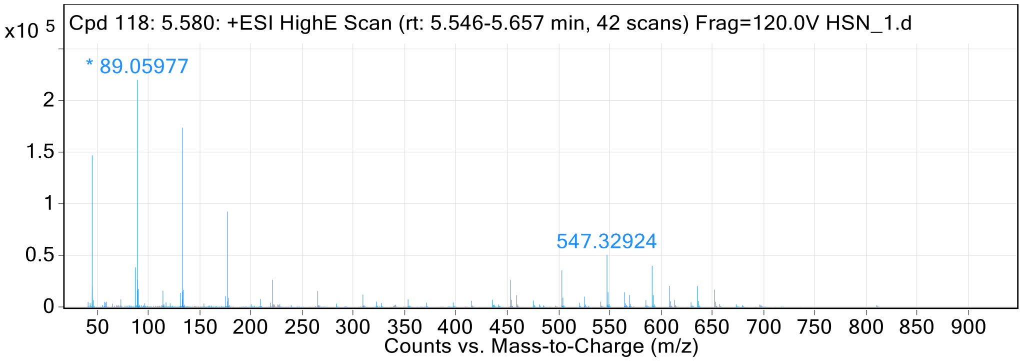

Peak List

m/z	z	Abund
45.0343	1	146585.41
87.04285		38225.97
89.05977	1	219405.19
89.14171		26362.21
133.08492	1	173164.81
177.11015	1	92212.98
221.1359	1	26246.14
503.30301	1	35394.22
547.32924	1	50261.35
591.35519	1	39735.96

Qualitative Analysis Report

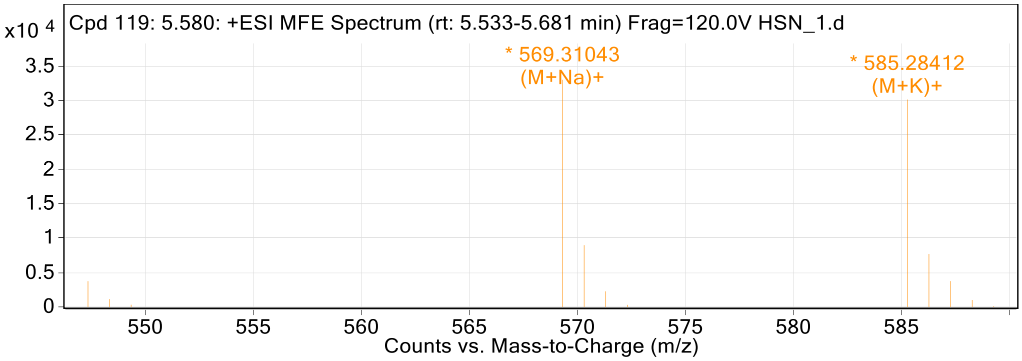

Peak List

m/z	z	Abund	Ion
547.32895	1	3735.42	(M+H)+
548.33209	1	1137.54	(M+H)+
549.32749	1	313.32	(M+H)+
569.31043	1	33015.73	(M+Na)+
570.31365	1	8956.23	(M+Na)+
571.31538	1	2251.21	(M+Na)+
585.28412	1	30144.63	(M+K)+
586.28775	1	7698.63	(M+K)+
587.28677	1	3750.68	(M+K)+
588.29008	1	1011.88	(M+K)+

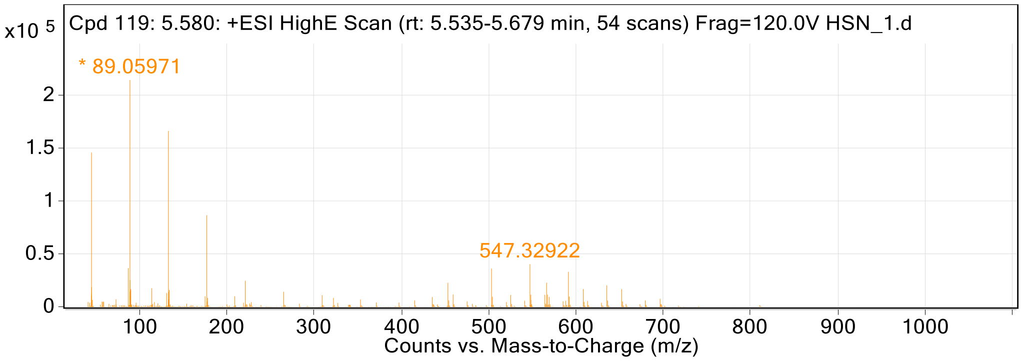

Peak List

m/z	z	Abund
45.03425	1	145546.66
87.04284		36386.54
89.05971	1	213981.77
89.14171		25078.8
133.0849	1	165898.67
177.11016	1	86371.75
221.13591	1	24511.49
503.30315	1	35984.18
547.32922	1	40154.65
591.35515	1	32987.88

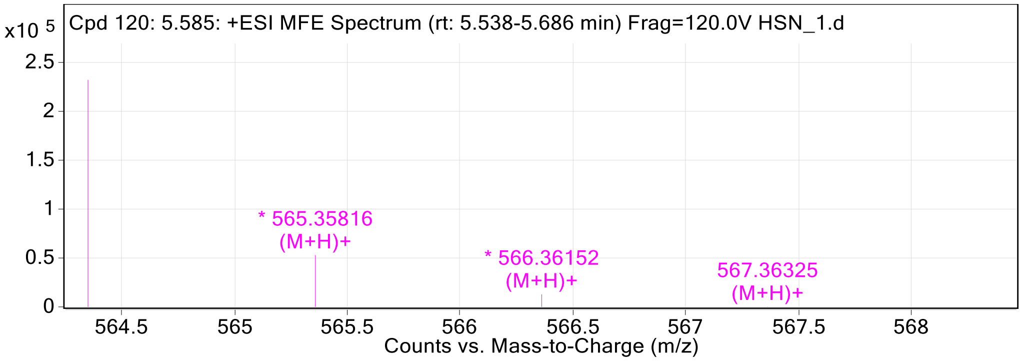

Peak List

m/z	z	Abund	Ion
564.34993	1	232215.94	(M+H)+
565.35816	1	52916.97	(M+H)+
566.36152	1	12837.97	(M+H)+
567.36325	1	189.47	(M+H)+
568.36574	1	93.57	(M+H)+

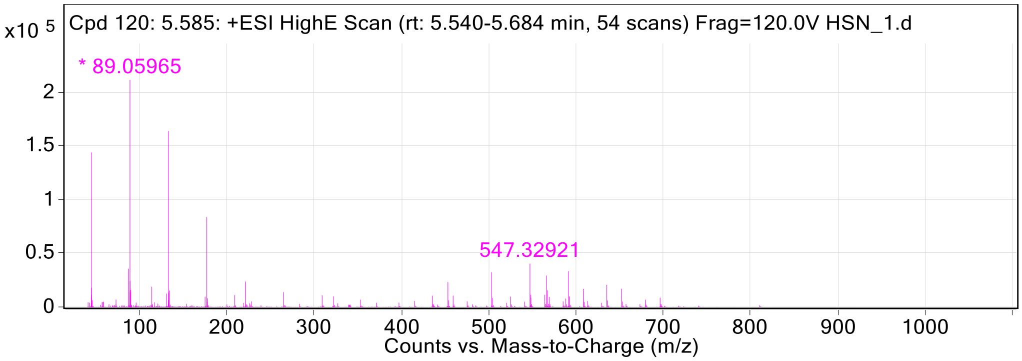

Peak List

m/z	z	Abund
45.0342	1	143797.03
87.04285		35580.71
89.05965	1	211231.52
89.14171		24005.47
133.08484	1	163684.39
177.11013	1	83632.36
503.30309	1	32321.47
547.32921	1	40344.57
566.42631	1	29216.95
591.35515	1	33280.19

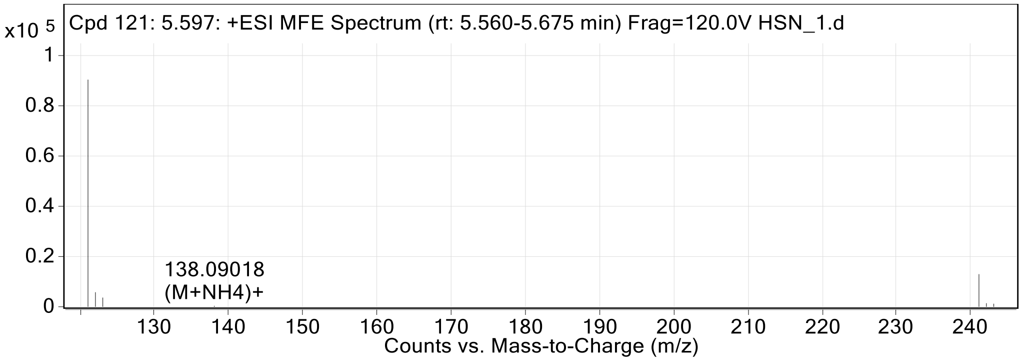

Peak List

m/z	z	Abund	Ion

121.06611	1	90192.31	(M+H)+
122.06951	1	5784.64	(M+H)+
123.06235	1	3651.49	(M+H)+
138.09018	1	377.5	(M+NH4)+
241.12654	1	12959.85	(2M+H)+
242.12995	1	1417.93	(2M+H)+
243.12352	1	1212.24	(2M+H)+

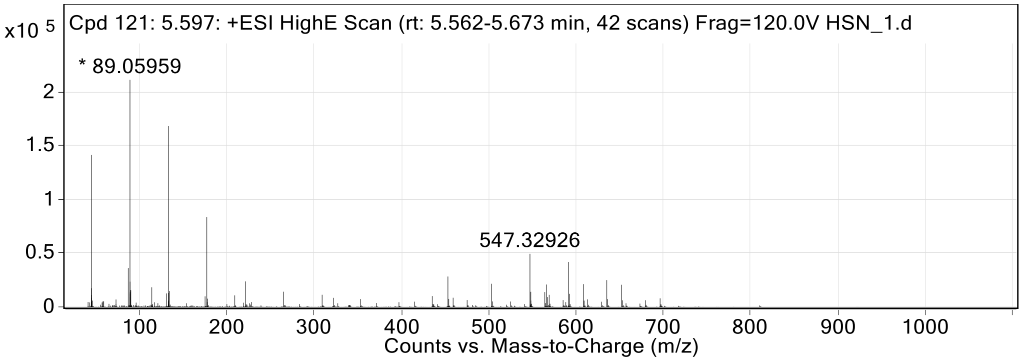

Peak List

m/z	z	Abund
45.03414	1	141672.66
87.04284		36241.79
89.05959	1	211548.77
133.0847	1	168392.19
177.1101	1	83721.43
221.13593	1	23726.67
453.34021	1	28289.96
547.32926	1	49501.52
591.35516	1	41924.62
635.38086	1	24992.34

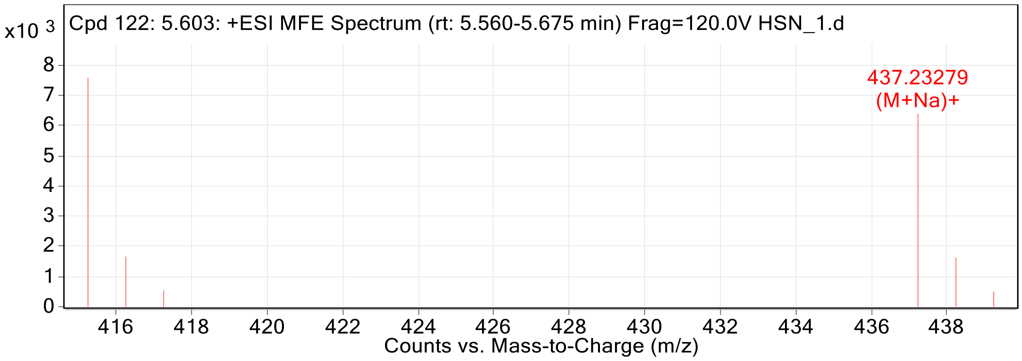

Peak List

m/z	z	Abund	Ion
415.25148	1	7576.98	(M+H)+
416.25484	1	1662.87	(M+H)+
417.25402	1	543.58	(M+H)+
437.23279	1	6388.94	(M+Na)+
438.23713	1	1628.05	(M+Na)+
439.23934	1	503.88	(M+Na)+

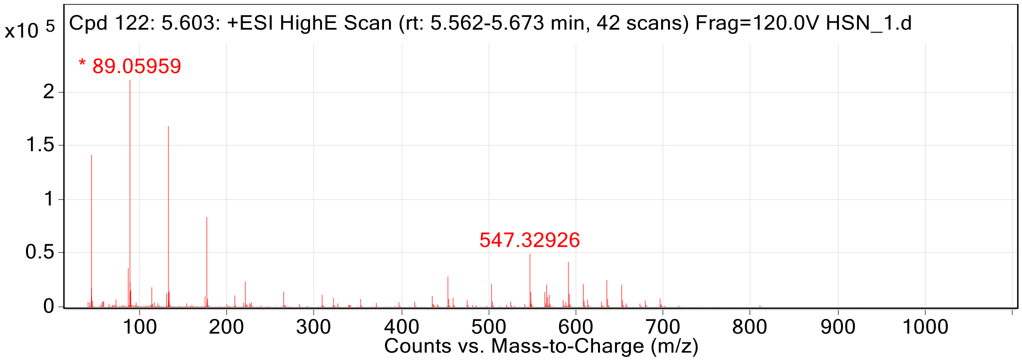

Peak List

m/z	z	Abund
45.03414	1	141672.66
87.04284		36241.79
89.05959	1	211548.77
133.0847	1	168392.19
177.1101	1	83721.43
221.13593	1	23726.67
453.34021	1	28289.96
547.32926	1	49501.52
591.35516	1	41924.62
635.38086	1	24992.34

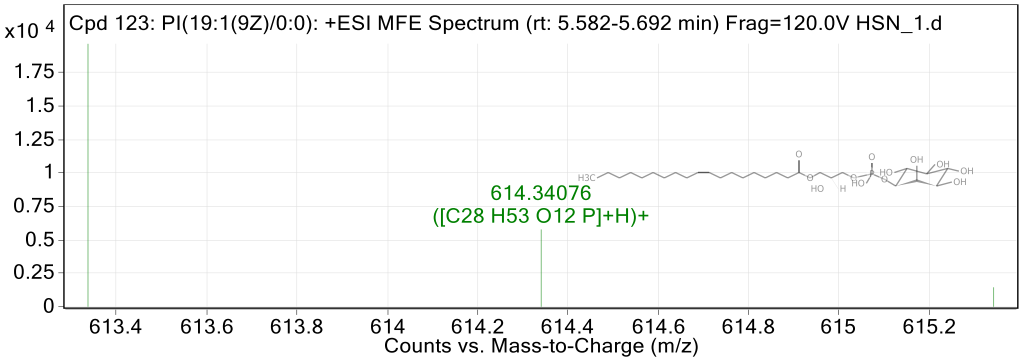

Peak List

m/z	z	Abund	Name	Formula	Ion	Score (DB)	Hits (DB)
613.337	1	19665.57	PI(19:1(9Z)/0:0)	C28 H53 O12 P	(M+H)+	90.84	1
614.34076	1	5780.57		C28 H53 O12 P	(M+H)+		
615.34347	1	1456.34		C28 H53 O12 P	(M+H)+		

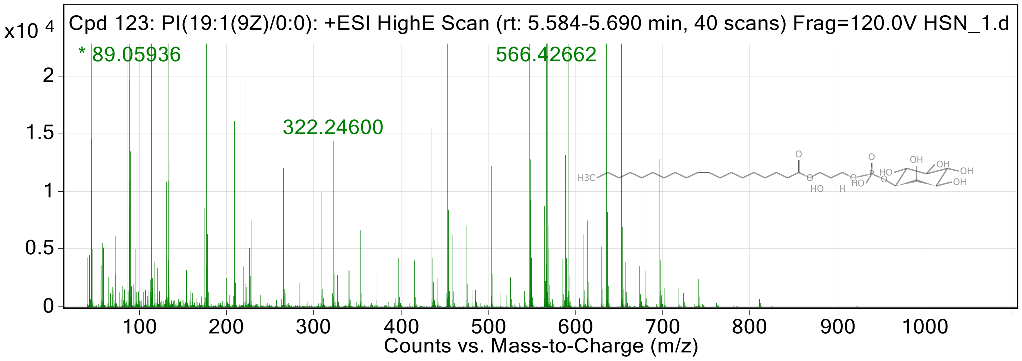

Peak List

m/z	z	Abund
45.03391	1	135747.31
87.04285		32428.57

89.05936	1	199525.53
133.08446	1	156923.72
177.11	1	71828.37
453.34028	1	31692.62
547.32906	1	34031.09
566.42662	1	47713.04
591.35515	1	45211.77
635.3808	1	28433.99

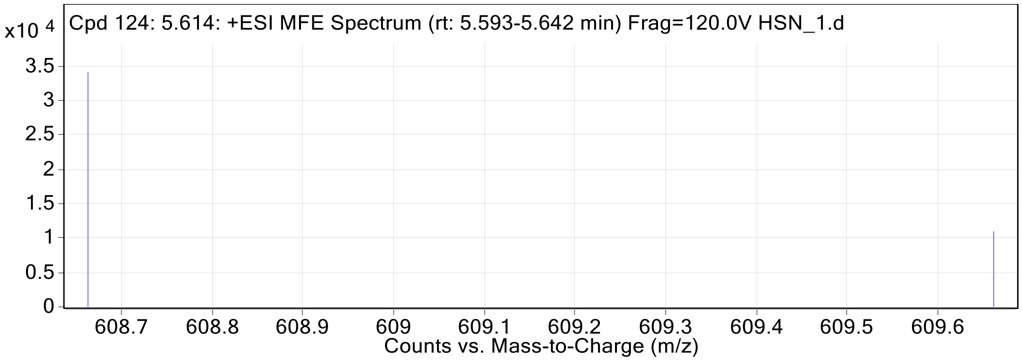

Peak List

m/z	z	Abund	Ion
608.66284	1	34182.58	(M+H)+
609.6619	1	11006.2	(M+H)+

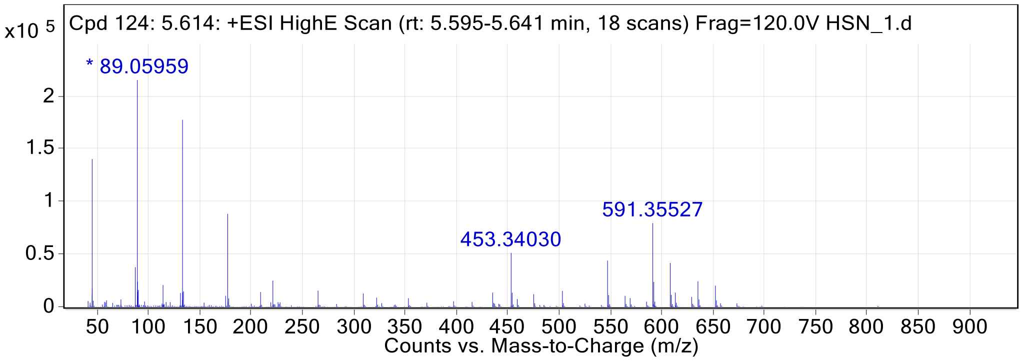

Peak List

m/z	z	Abund
45.03414	1	140103.2
87.04285		37541.48
89.05959	1	215208.25
133.08462	1	177408.08
177.11006	1	88183.07
221.13594	1	24735
453.3403	1	51051.04
547.32889	1	43785.39
591.35527	1	79373.84
608.38122	1	41505.53

Qualitative Analysis Report

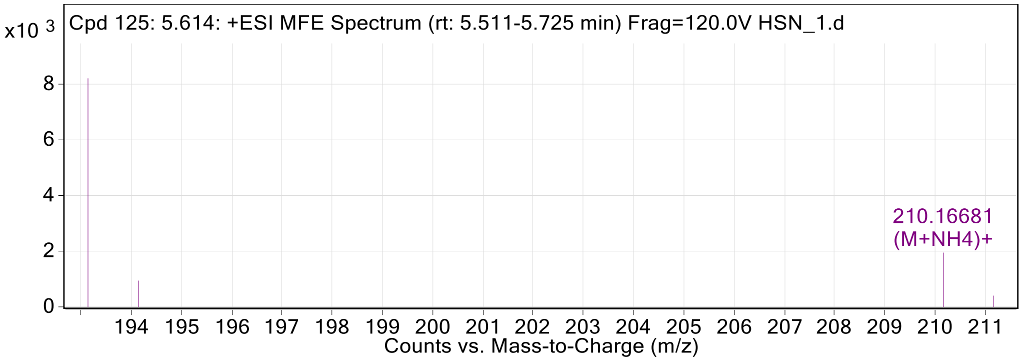

Peak List

m/z	z	Abund	Ion
193.14101	1	8209.32	(M+H)+
194.14459	1	943.28	(M+H)+
210.16681	1	1948.79	(M+NH4)+
211.16997	1	403.34	(M+NH4)+

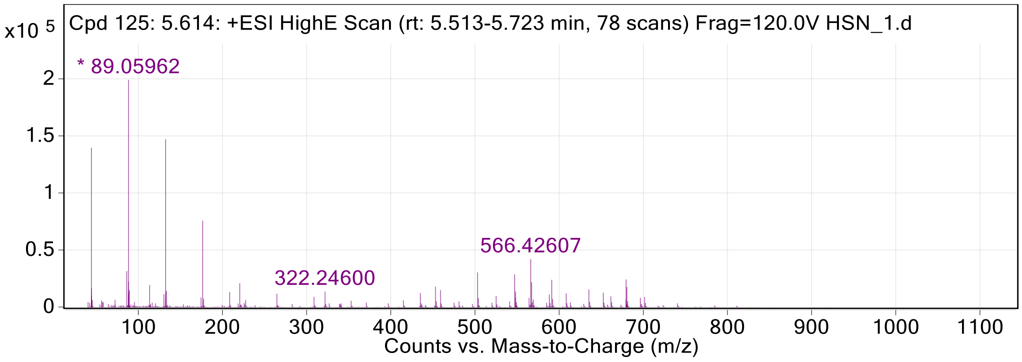

Peak List

m/z	z	Abund
45.03417	1	139705.78
87.04284		31553.96
89.05962	1	199113.23
133.08491	1	147135.7
177.11013	1	75692.23
503.3031	1	30407.75
547.32919	1	28628.01
566.42607	1	42013.28
591.35513	1	23880.93
679.51339	1	24172.51

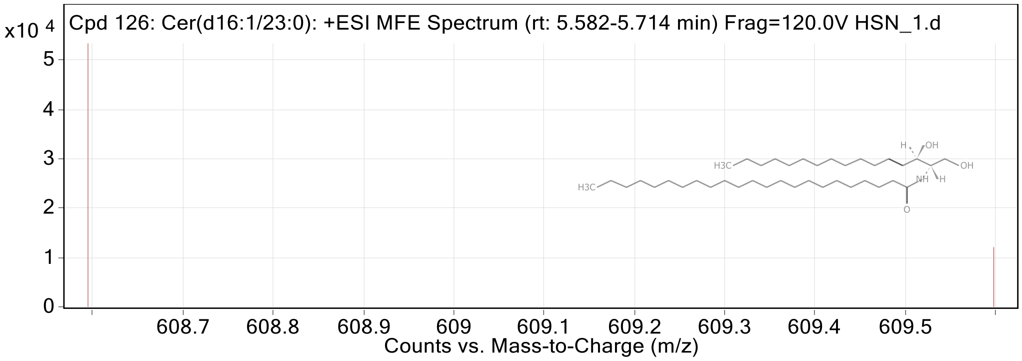

Peak List

m/z	z	Abund	Name	Formula	Ion	Score (DB)	Hits (DB)
608.59482	1	53412.41	Cer(d16:1/23:0)	C39 H77 N O3	(M+H)+	50.71	2

609.598	1	12102.5		C39 H77 N O3	(M+H)+		

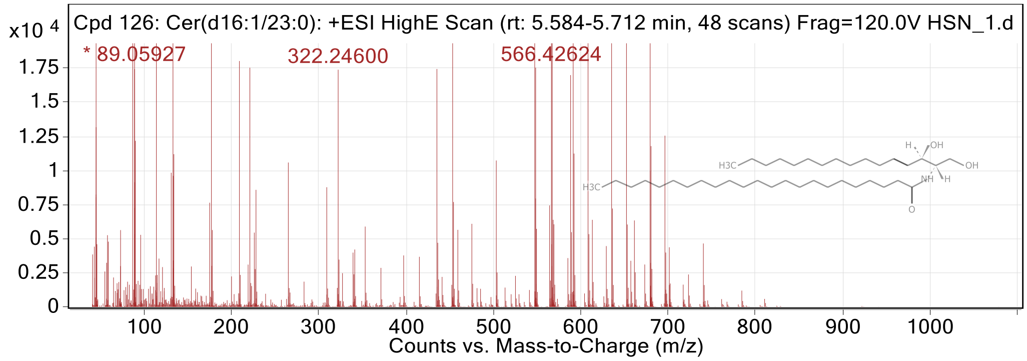

Peak List

m/z	z	Abund
45.03383	1	130785.99
87.04284		29410.97
89.05927	1	188690.86
133.08441	1	144437.64
177.10998	1	64307.56
453.34033	1	28028.81
547.32904	1	29035.04
566.42624	1	63661.07
567.4268	1	33777.31
591.35513	1	38517.76

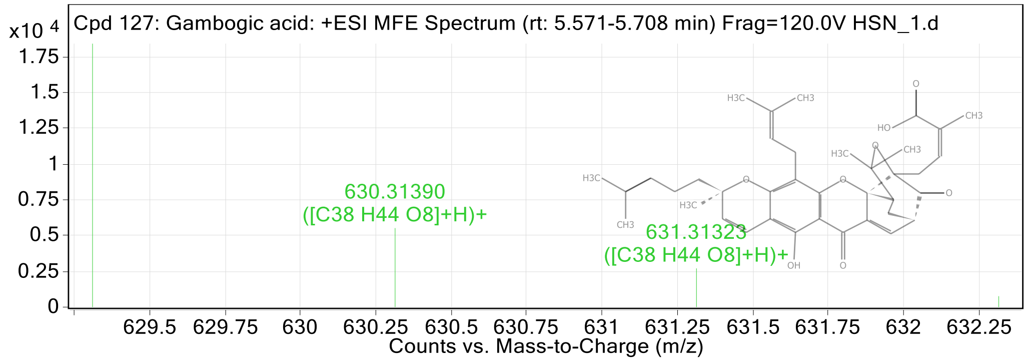

Peak List

m/z	z	Abund	Name	Formula	Ion	Score (DB)	Hits (DB)
629.31062	1	18438.12	Gambogic acid	C38 H44 O8	(M+H)+	76.57	1
630.3139	1	5528.91		C38 H44 O8	(M+H)+		
631.31323	1	2707.12		C38 H44 O8	(M+H)+		
632.31557	1	762.81		C38 H44 O8	(M+H)+		

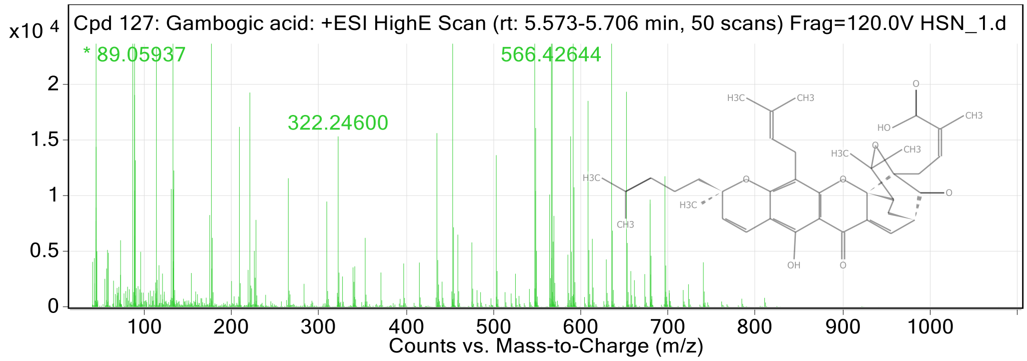

Peak List

m/z	z	Abund

45.03392	1	134094.59
87.04285		31196.53
89.05937	1	194797.55
133.08451	1	150413.13
177.11003	1	69448.41
453.34031	1	26693.44
547.32928	1	36377.36
566.42644	1	56950.52
567.42682	1	31034.74
591.35513	1	36870.24

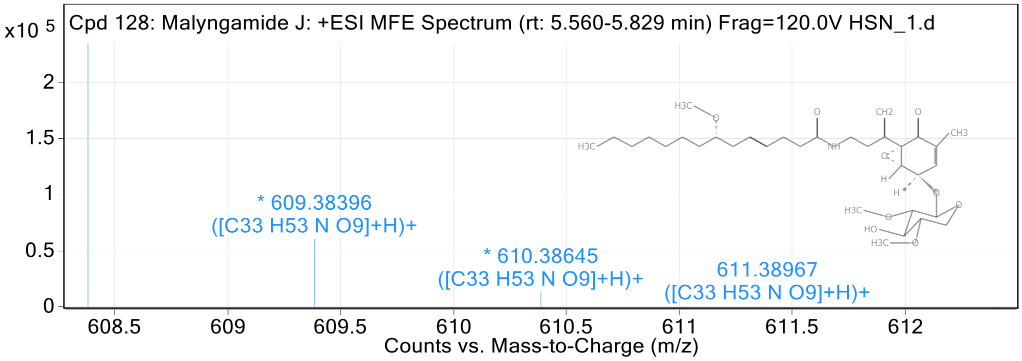

Peak List

m/z	z	Abund	Name	Formula	Ion	Score (DB)	Hits (DB)
608.38081	1	234461.94	Malyngamide J	C33 H53 N O9	(M+H)+	83.37	1
609.38396	1	60096.51		C33 H53 N O9	(M+H)+		
610.38645	1	12705.9		C33 H53 N O9	(M+H)+		
611.38967	1	601.35		C33 H53 N O9	(M+H)+		
612.39212	1	405.71		C33 H53 N O9	(M+H)+		

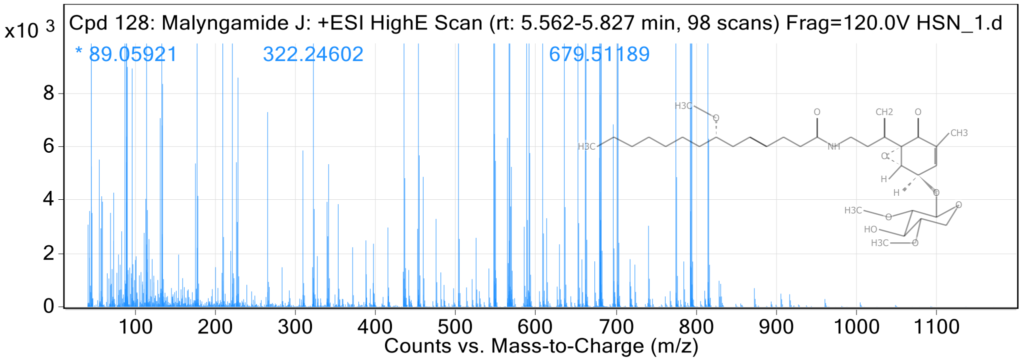

Peak List

m/z	z	Abund
45.03378	1	103494.79
89.05921	1	142543.34
114.08966	1	53273.6
133.08447	1	103124.31
177.11001	1	45825.45
566.42576	1	40086.43
679.51189	1	81828.67
680.51164	1	51698.97
792.59309	1	60462.89
793.59441	1	38148.14

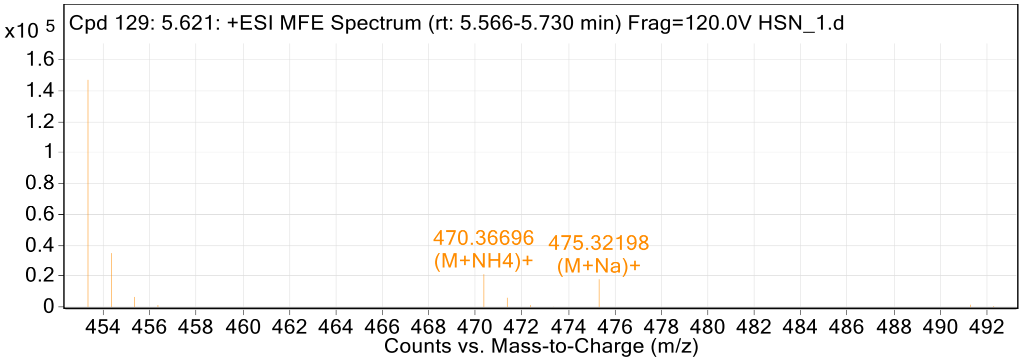

Peak List

m/z	z	Abund	Ion
453.33984	1	147182.56	(M+H)+
454.34313	1	34860.51	(M+H)+
455.3463	1	6486.17	(M+H)+
456.34834	1	1207.78	(M+H)+
470.36696	1	21117.11	(M+NH4)+
471.36997	1	5933.91	(M+NH4)+
472.37191	1	1188.84	(M+NH4)+
475.32198	1	17852.43	(M+Na)+
491.29617	1	1396.75	(M+K)+
492.29607	1	551.12	(M+K)+

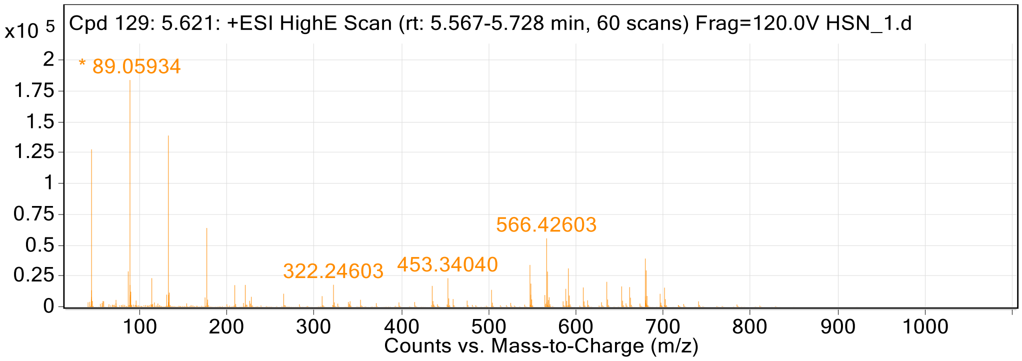

Peak List

m/z	z	Abund
45.0339	1	127709.42
87.04284		28706.18
89.05934	1	183969.23
133.08452	1	138931.02
177.11003	1	63892.64
547.32923	1	33915.72
566.42603	1	55477.13
591.35512	1	31113.78
679.51437	1	39098.27
680.51211	1	29477.04

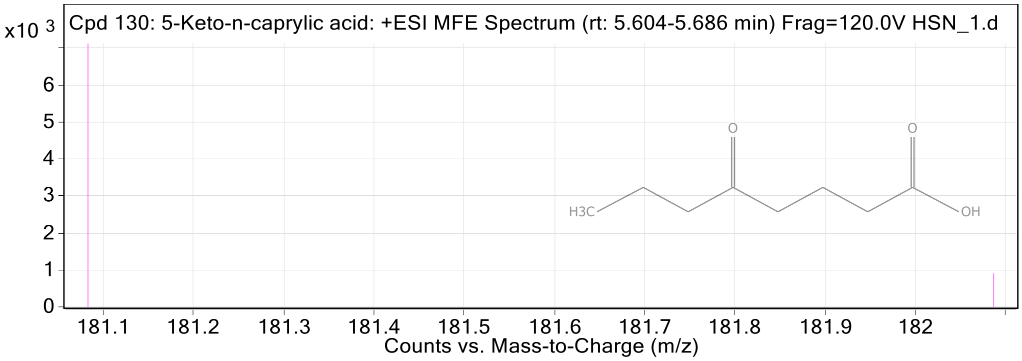

Peak List

m/z	z	Abund	Name	Formula	Ion	Score (DB)	Hits (DB)
181.08336	1	7126.37	5-Keto-n-caprylic acid	C8 H14 O3	(M+Na)+	82.77	10
182.08679	1	913.25		C8 H14 O3	(M+Na)+		

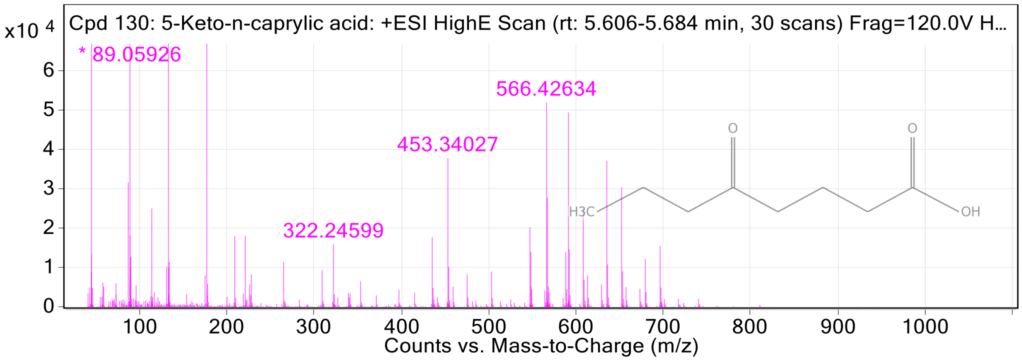

Peak List

m/z	z	Abund
45.03383	1	134264.59
87.04285		31677.38
89.05926	1	195747.61
133.08432	1	155480.64
177.10995	1	67031.9
453.34027	1	37900.83
566.42634	1	52057.63
591.35514	1	49517.54
635.38081	1	37141.52
652.40713	1	30470.7

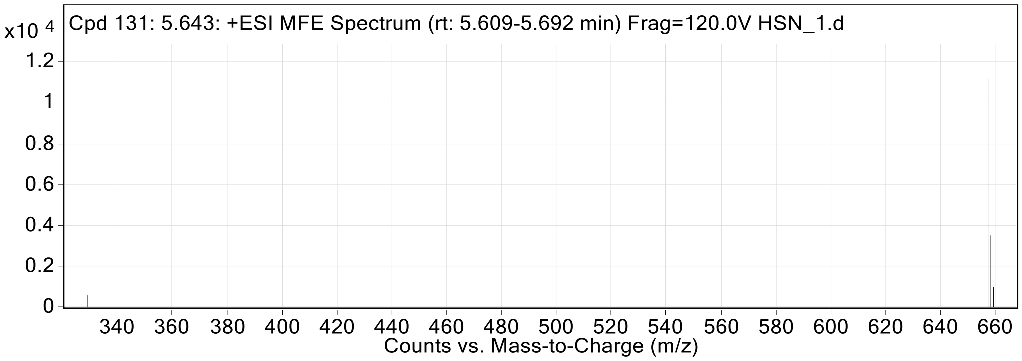

Peak List

m/z	z	Abund	Ion
329.18721	2	562.73	(M+2H)+2
657.36287	1	11180.74	(M+H)+
658.36468	1	3496.92	(M+H)+

659.37144	1	963.19	(M+H)+

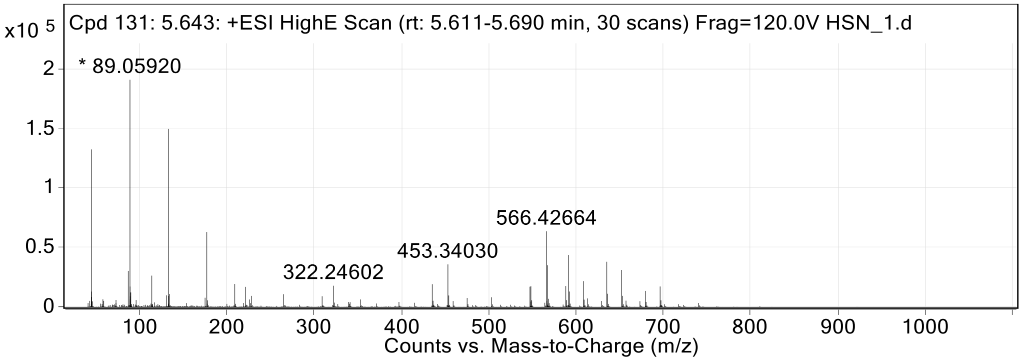

Peak List

m/z	z	Abund
45.03377	1	132387.73
89.0592	1	191006.16
133.08427	1	149602.84
177.10992	1	62935.43
453.3403	1	35625.54
566.42664	1	63437.01
567.42692	1	34925.49
591.35505	1	43601.82
635.3808	1	37881.98
652.40717	1	31030.72

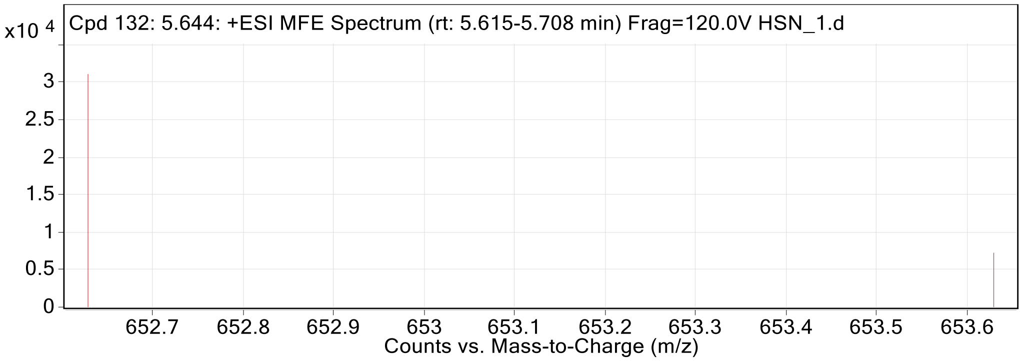

Peak List

m/z	z	Abund	Ion
652.62814	1	31077.36	(M+H)+
653.62946	1	7248.05	(M+H)+

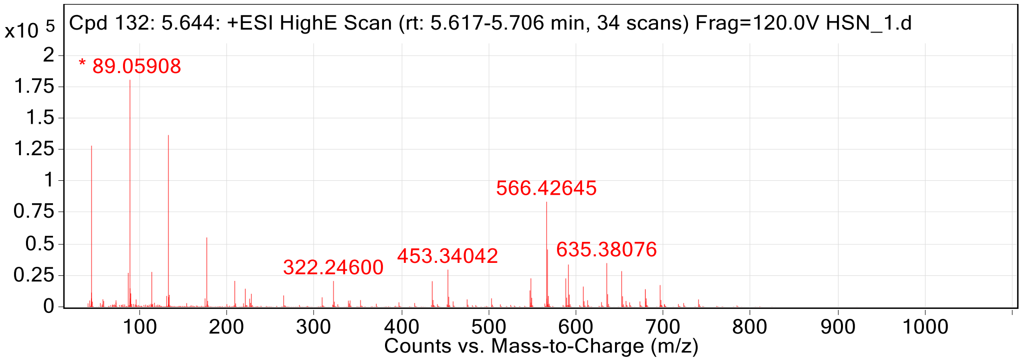

Peak List

m/z	z	Abund
45.03366	1	127974.77
89.05908	1	180270.39

133.0842	1	136420.73
177.10989	1	55084
453.34042	1	29550.79
566.42645	1	83471.83
567.42682	1	45548.21
591.35504	1	33586
635.38076	1	34731.48
652.40714	1	28351.84

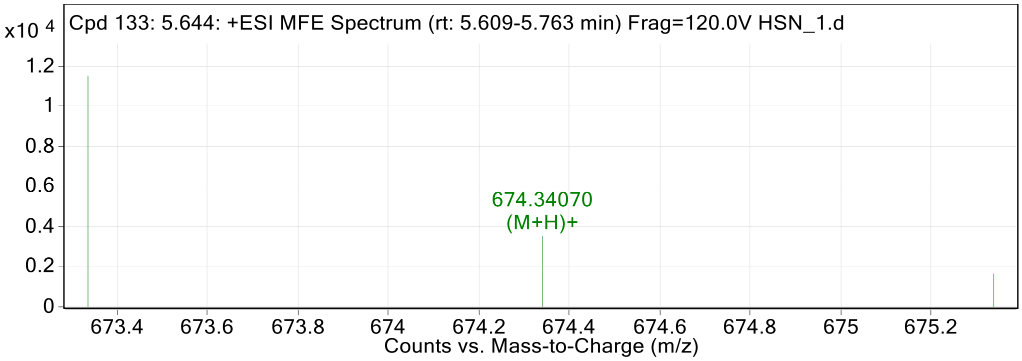

Peak List

m/z	z	Abund	Ion
673.3361	1	11525.25	(M+H)+
674.3407	1	3536.17	(M+H)+
675.33837	1	1654.66	(M+H)+

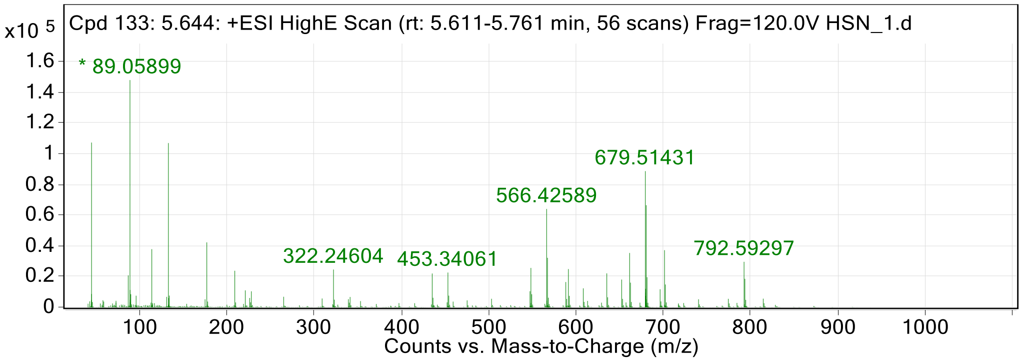

Peak List

m/z	z	Abund
45.0336	1	107236.45
89.05899	1	147965.61
114.08959		37753.08
133.08418	1	106902.86
177.10987	1	42172.57
566.42589	1	63943.88
661.4974	1	35256
679.51431	1	88612.79
680.51212	1	66383.55
701.48903	1	37082.48

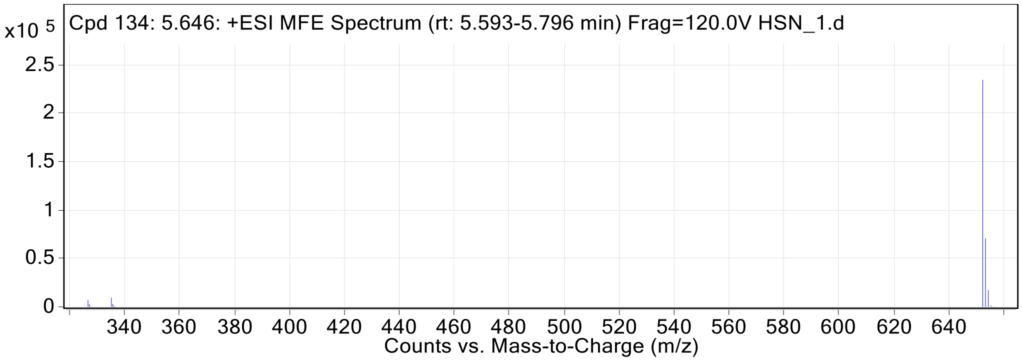

Peak List

m/z	z	Abund	Ion
326.70658	2	7256.27	(M+2H)+2
327.20247	2	2835.19	(M+2H)+2
327.70739	2	567.32	(M+2H)+2
335.22008	2	9497.16	(M+H5N)+2
335.72131	2	2944.27	(M+H5N)+2
336.22098	2	956.41	(M+H5N)+2
652.40683	1	234406.77	(M+H)+
653.40998	1	70886.27	(M+H)+
654.41297	1	17285.13	(M+H)+
655.41577	1	1290.49	(M+H)+

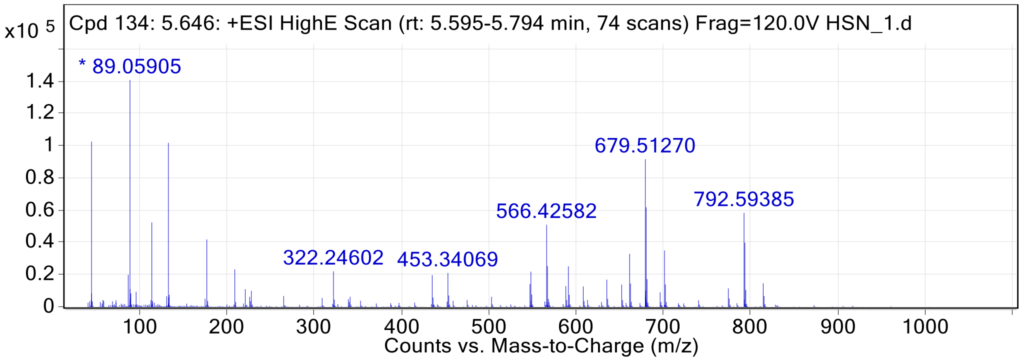

Peak List

m/z	z	Abund
45.03364	1	102497.27
89.05905	1	140706.34
114.08967	1	52349.1
133.08426	1	101731.21
177.10992	1	41778.18
566.42582	1	50913.71
679.5127	1	91582.26
680.51179	1	61713.43
792.59385	1	58271.04
793.5946	1	39706.5

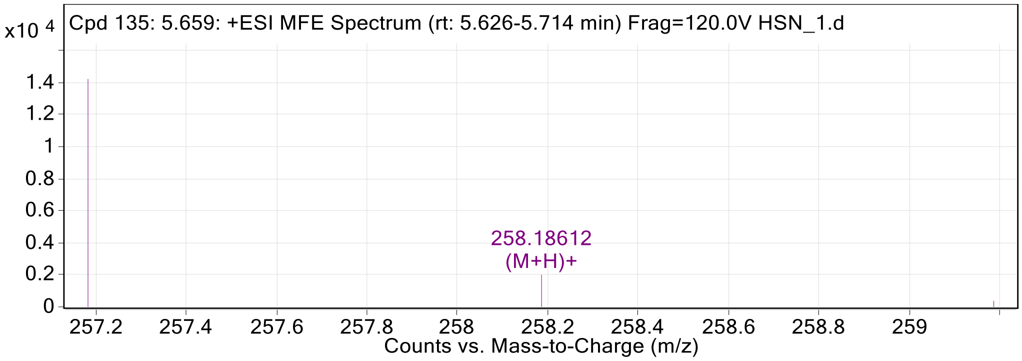

Peak List

m/z	z	Abund	Ion
257.18303	1	14225.75	(M+H)+
258.18612	1	2008.29	(M+H)+
259.18636	1	378.34	(M+H)+

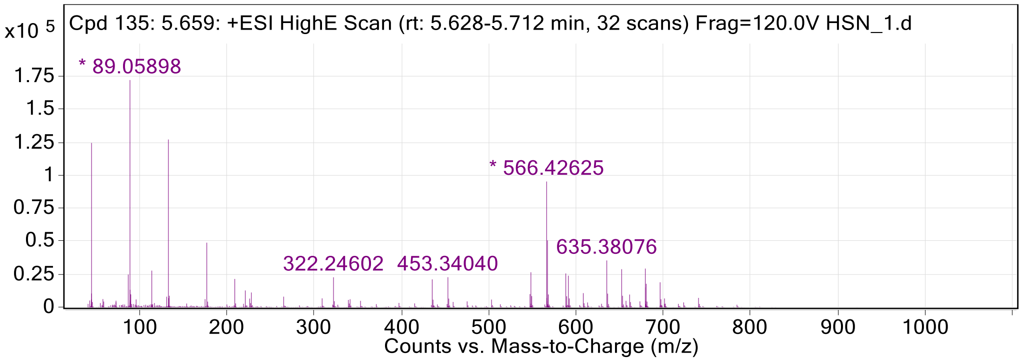

Peak List

m/z	z	Abund
45.03358	1	124500.89
89.05898	1	172183.78
114.08952		27652.17
133.08413	1	127094.45
177.10984	1	48849.6
566.42625	1	95234.97
567.4268	1	50588.67
635.38076	1	35372.27
652.40715	1	28736.27
679.50978	1	29189.13

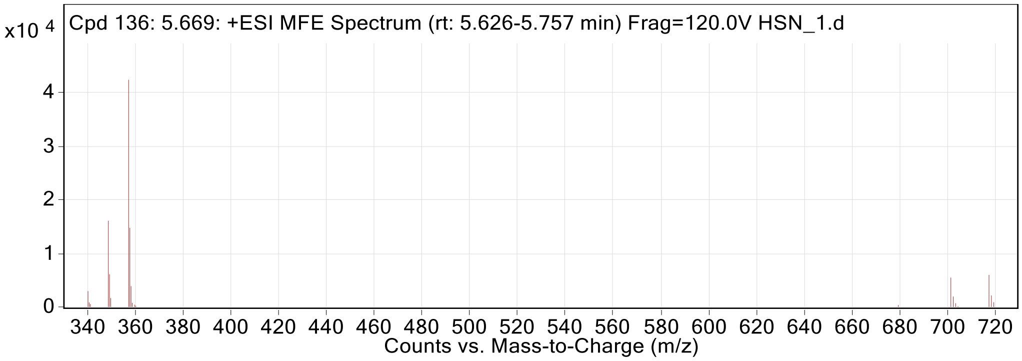

Peak List

m/z	z	Abund	Ion
340.20653	2	2939.6	(M+2H)+2
348.71977	2	16109.17	(M+H5N)+2

349.22084	2	6100.79	(M+H5N)+2
357.23285	2	42452.61	(M+2(NH4))+2
357.73452	2	14775.48	(M+2(NH4))+2
358.23597	2	3895.83	(M+2(NH4))+2
701.38888	1	5478.58	(M+Na)+
702.39295	1	1931.06	(M+Na)+
717.36201	1	5976.36	(M+K)+
718.3648	1	2132.02	(M+K)+

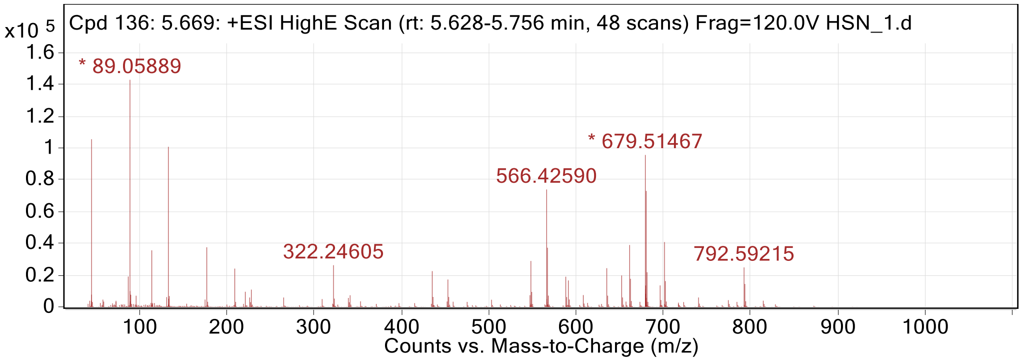

Peak List

m/z	z	Abund
45.03351	1	105436.37
89.05889	1	142811.3
133.0841	1	100672.13
177.10983	1	37474.43
566.4259	1	73830.38
567.42678	1	37185.24
661.49745	1	38891.91
679.51467	1	95544.2
680.51218	1	72908.45
701.48901	1	40753.14

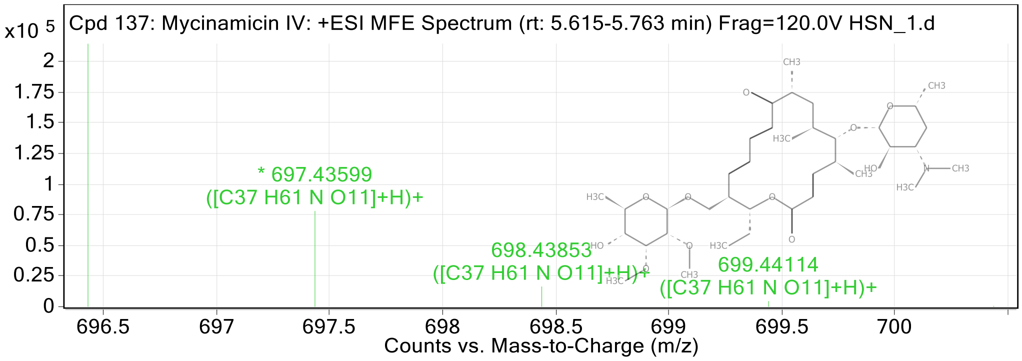

Peak List

m/z	z	Abund	Name	Formula	Ion	Score (DB)	Hits (DB)
696.43268	1	214442.58	Mycinamicin IV	C37 H61 N O11	(M+H)+	95.65	1
697.43599	1	78065.63		C37 H61 N O11	(M+H)+		
698.43853	1	16464.91		C37 H61 N O11	(M+H)+		
699.44114	1	4597.92		C37 H61 N O11	(M+H)+		
700.43663	1	712.24		C37 H61 N O11	(M+H)+		

Qualitative Analysis Report

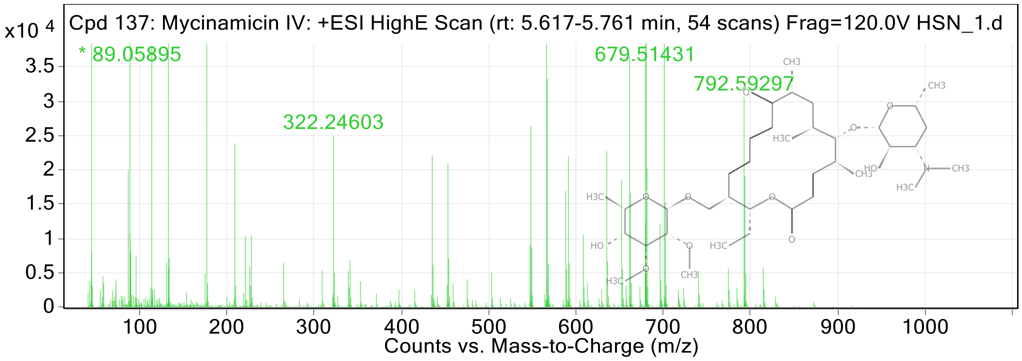

Peak List

m/z	z	Abund
45.03356	1	106002.09
89.05895	1	145384.52
114.08959	1	38173.37
133.08415	1	104069.02
177.10986	1	40304.88
566.42589	1	66293.67
661.4974	1	36561.78
679.51431	1	91888.06
680.51212	1	68842.21
701.48903	1	38455.91

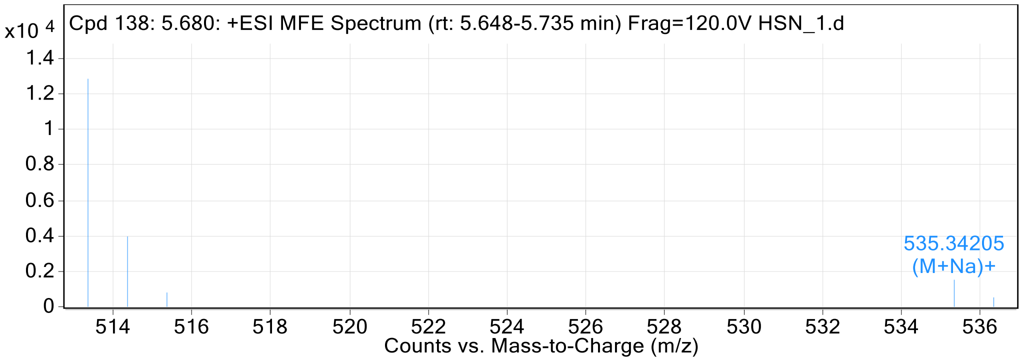

Peak List

m/z	z	Abund	Ion
513.36067	1	12869.33	(M+H)+
514.36419	1	3967.85	(M+H)+
515.36468	1	805.72	(M+H)+
535.34205	1	1524.95	(M+Na)+
536.34625	1	529.66	(M+Na)+

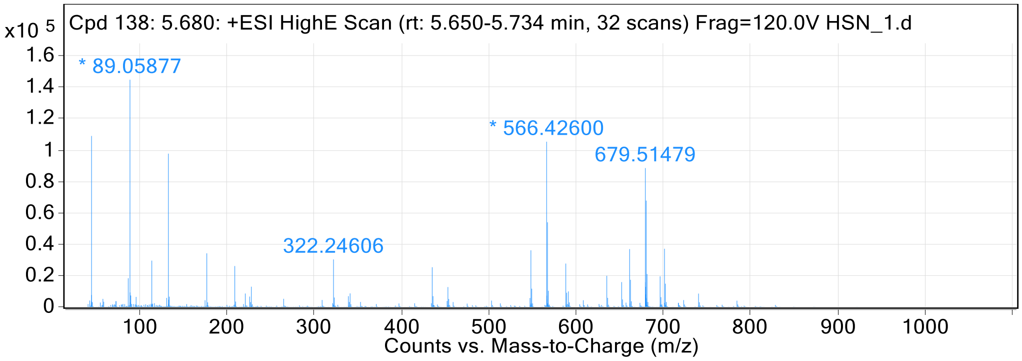

Peak List

m/z	z	Abund

45.03342	1	108907.25
89.05877	1	144738.19
133.08399	1	97625.67
548.41297	1	36014.02
566.426	1	105252.26
567.42681	1	53857.25
661.49761	1	36773.03
679.51479	1	88426.17
680.51235	1	67688.24
701.48896	1	36994.85

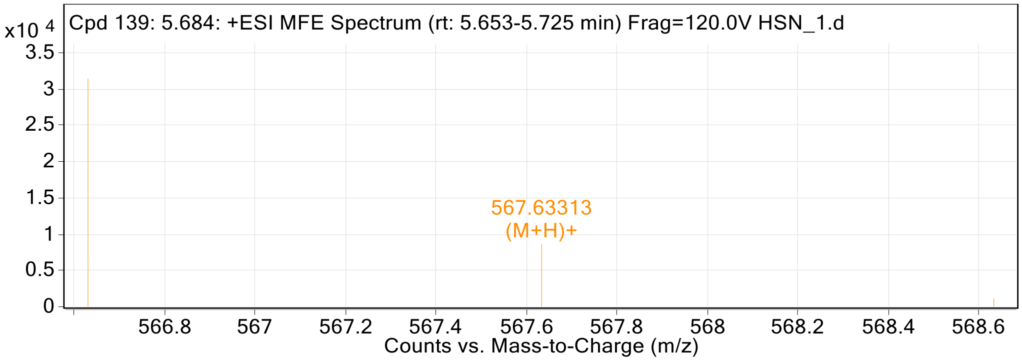

Peak List

m/z	z	Abund	Ion
566.63027	1	31438.13	(M+H)+
567.63313	1	8627.2	(M+H)+
568.63269	1	1155.84	(M+H)+

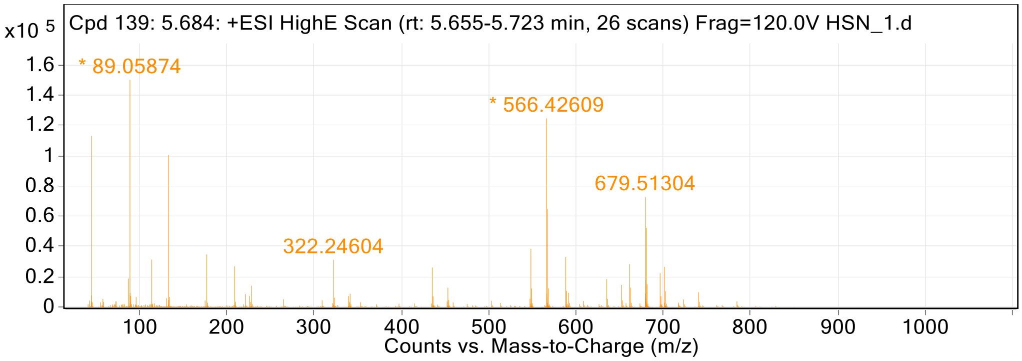

Peak List

m/z	z	Abund
45.03341	1	113100.51
89.05874	1	150054.73
133.08398	1	100422.04
177.10973	1	34728.5
548.41289	1	38409.02
566.42609	1	124621.2
567.4268	1	64676.72
588.40514	1	33026.83
679.51304	1	72684.81
680.51188	1	52325.59

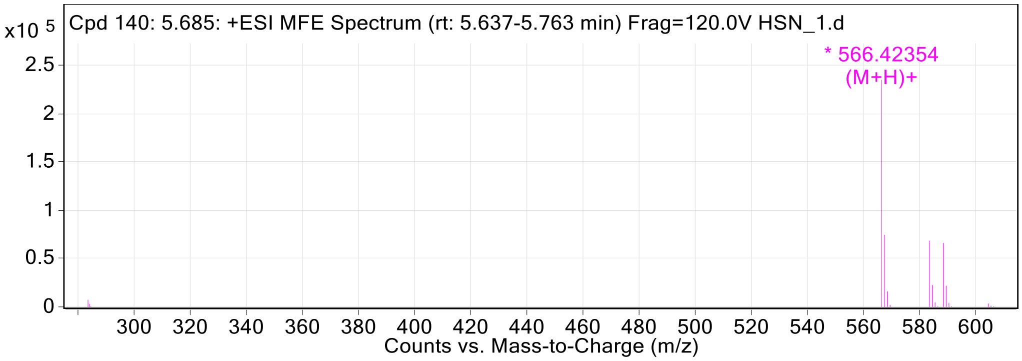

Peak List

m/z	z	Abund	Ion
283.71494	2	7305.69	(M+2H)+2
566.42354	1	235119	(M+H)+
567.42599	1	74615.54	(M+H)+
568.4295	1	16054.87	(M+H)+
583.45045	1	68441.59	(M+NH4)+
584.45306	1	22615.82	(M+NH4)+
585.45563	1	4704.64	(M+NH4)+
588.40502	1	66194.44	(M+Na)+
589.4088	1	21905.48	(M+Na)+
590.41102	1	4152.61	(M+Na)+

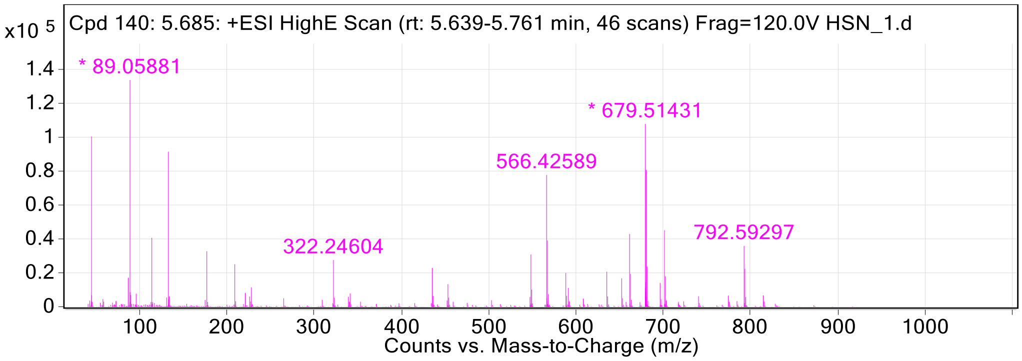

Peak List

m/z	z	Abund
45.03345	1	100495.95
89.05881	1	133790.55
114.08959	1	40706.18
133.08403	1	91495.12
566.42589	1	77755.49
567.42676	1	39062.31
661.49741	1	42914.31
679.51431	1	107815.59
680.51212	1	80797.4
701.48903	1	45143.89

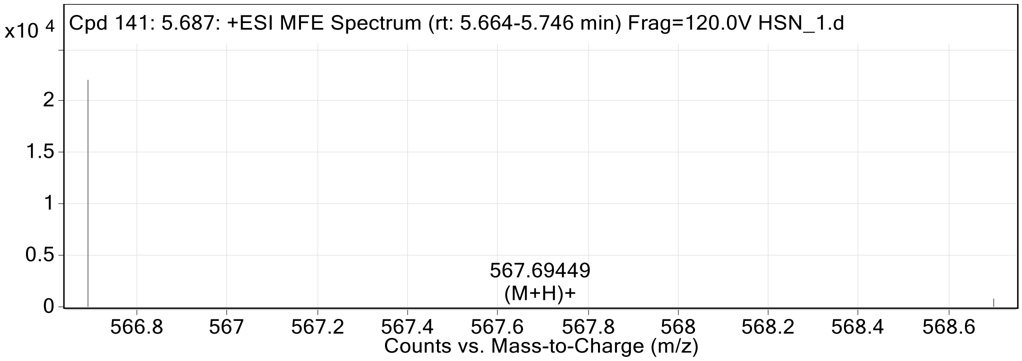

Peak List

m/z	z	Abund	Ion
566.69105	1	22061.03	(M+H)+
567.69449	1	0	(M+H)+
568.70037	1	804.91	(M+H)+

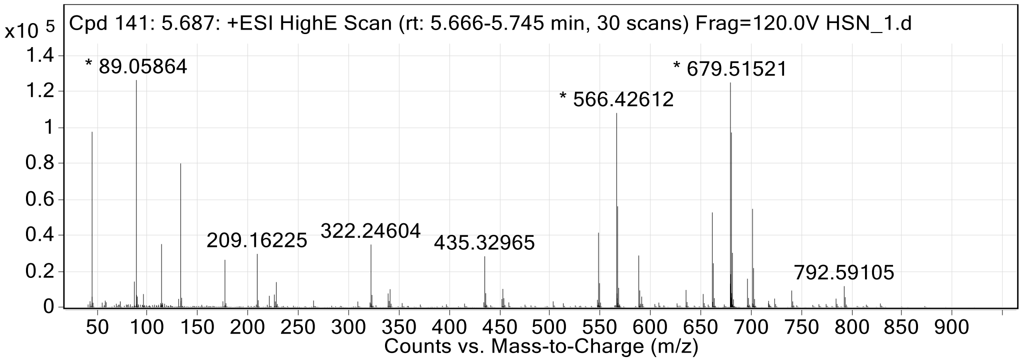

Peak List

m/z	z	Abund
45.03334	1	97499.32
89.05864	1	126266.98
133.08395	1	79880.75
548.41303	1	41381.07
566.42612	1	107946.78
567.42681	1	56038.24
661.49758	1	52633.94
679.51521	1	124916.02
680.51236	1	97107.32
701.48897	1	54628.46

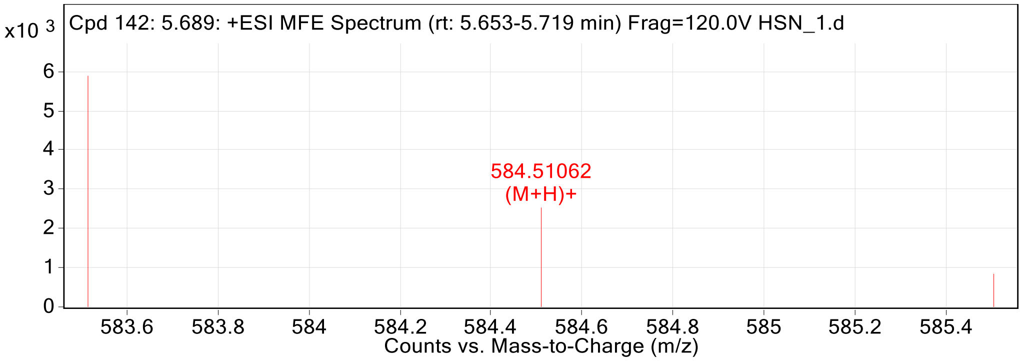

Peak List

m/z	z	Abund	Ion
583.51418	1	5890.94	(M+H)+
584.51062	1	2531.3	(M+H)+

585.50547	1	843.21	(M+H)+

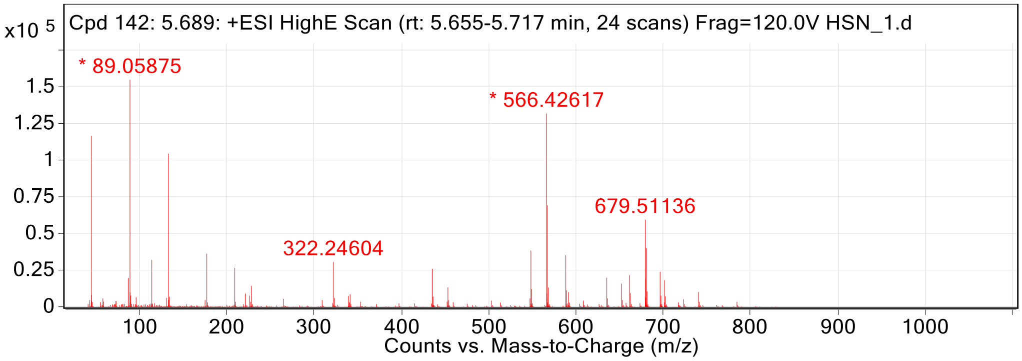

Peak List

m/z	z	Abund
45.03342	1	116198.77
89.05875	1	154468.38
133.08399	1	104188.16

m/z	z	Abund
177.10973	1	36163.71
548.41288	1	38263.94
566.42617	1	131490.23
567.4268	1	68947.86
588.40514	1	35154.81
679.51136	1	59212.83
680.5113	1	39820.57

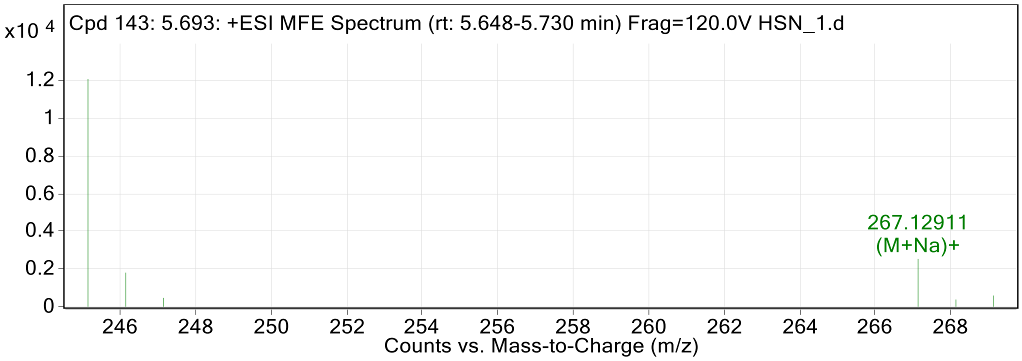

Peak List

m/z	z	Abund	Ion
245.14685	1	12076.9	(M+H)+
246.14936	1	1807.95	(M+H)+
247.15001	1	469.92	(M+H)+
267.12911	1	2537.36	(M+Na)+
268.13249	1	382.24	(M+Na)+
269.13429	1	593.77	(M+Na)+

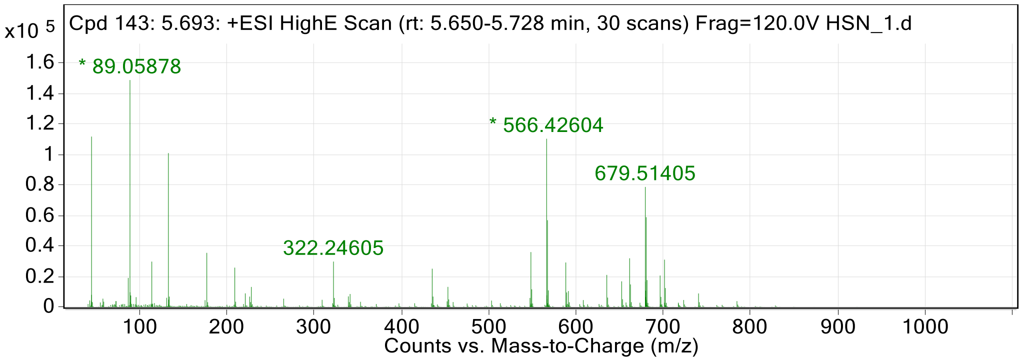

Peak List

m/z	z	Abund
45.03343	1	111770.77
89.05878	1	148912.33
133.08399	1	100914.84
177.10975	1	35405.86
548.41293	1	35912.85
566.42604	1	110350.9
567.4268	1	56853.45
661.49753	1	31844.04
679.51405	1	78706.09
680.51211	1	58817.83

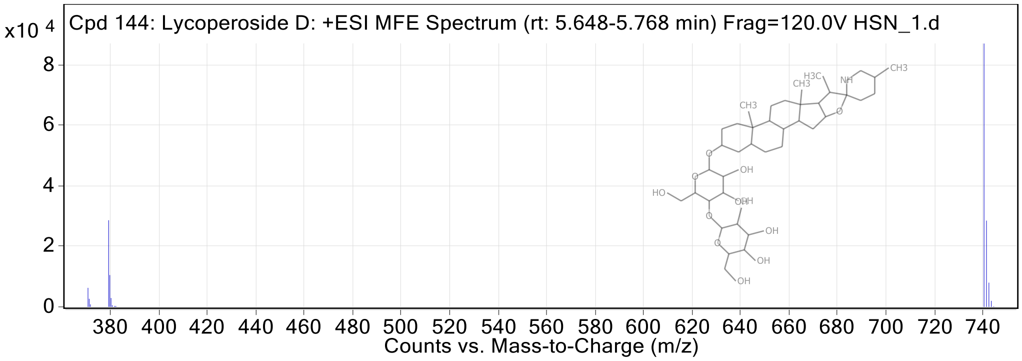

Peak List

m/z	z	Abund	Name	Formula	Ion	Score (DB)	Hits (DB)
370.73232	2	6298.34			(M+2H)+2		
371.22973	2	2681.34			(M+2H)+2		
371.73746	2	841.05			(M+2H)+2		
379.246	2	28654.94			(M+H5N)+2		
379.74783	2	10523.16			(M+H5N)+2		
380.2485	2	2936.4			(M+H5N)+2		
740.45859	1	87145.02	Lycoperoside D	C39 H65 N O12	(M+H)+	89.03	1
741.46182	1	28537.21		C39 H65 N O12	(M+H)+		
742.46402	1	8016.27		C39 H65 N O12	(M+H)+		
743.46564	1	1997.14		C39 H65 N O12	(M+H)+		

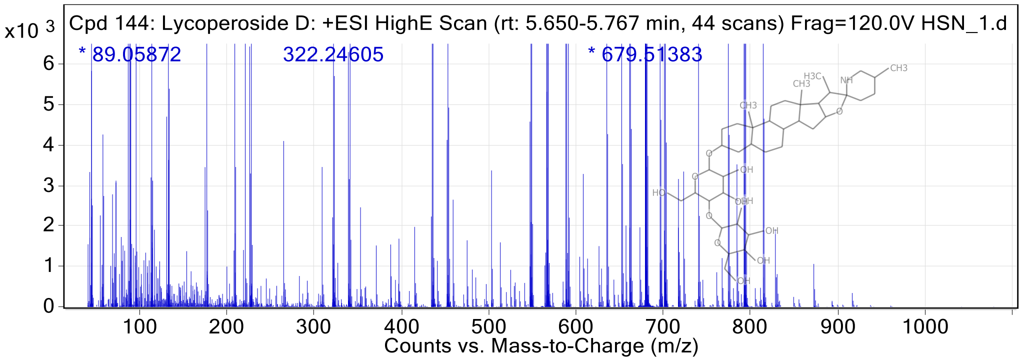

Peak List

m/z	z	Abund
45.03339	1	95413.6
89.05872	1	124838.24
114.08964	1	47351.4
133.08398	1	82267.38
566.42588	1	81951.8
661.4974	1	47130.99
679.51383	1	121188.67
680.51205	1	88665.77
701.48907	1	49494.48
792.59361	1	48085.45

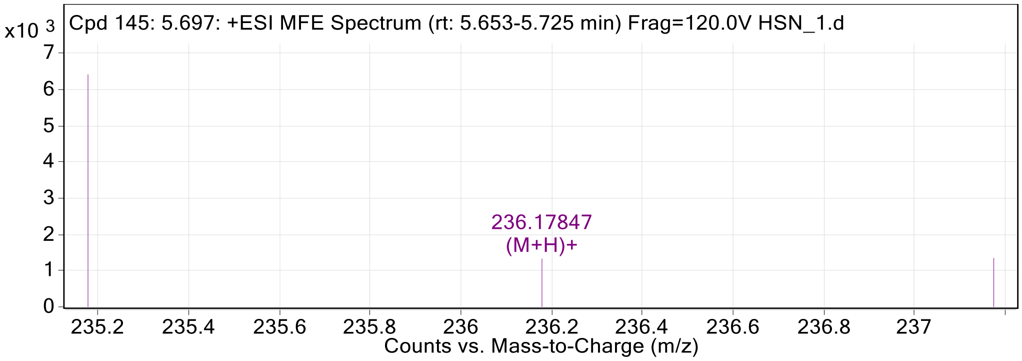

Peak List

m/z	z	Abund	Ion
235.1774	1	6414.85	(M+H)+
236.17847	1	1330.95	(M+H)+
237.17477	1	1349.62	(M+H)+

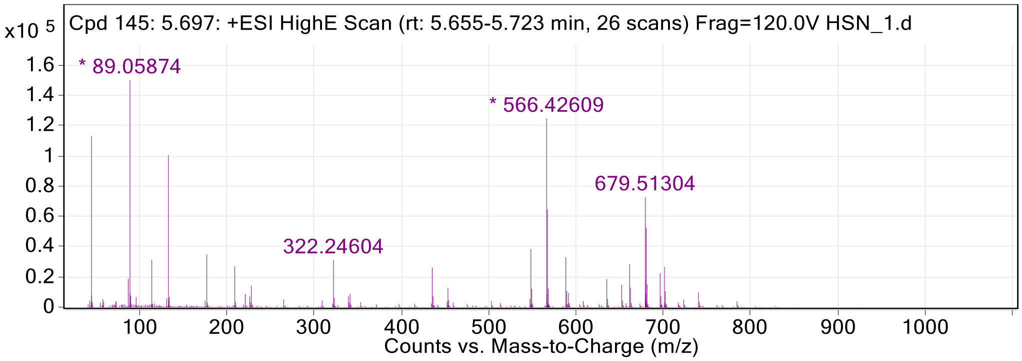

Peak List

m/z	z	Abund
45.03341	1	113100.51
89.05874	1	150054.73
133.08398	1	100422.04
177.10973	1	34728.5
548.41289	1	38409.02
566.42609	1	124621.2
567.4268	1	64676.72
588.40514	1	33026.83
679.51304	1	72684.81
680.51188	1	52325.59

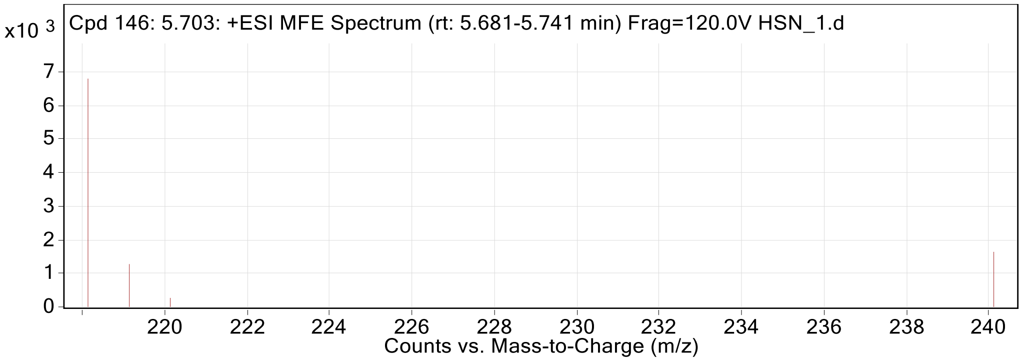

Peak List

m/z	z	Abund	Ion
218.13602	1	6802.96	(M+H)+
219.13892	1	1275.94	(M+H)+

220.13303	1	268.34	(M+H)+
240.11765	1	1641.09	(M+Na)+

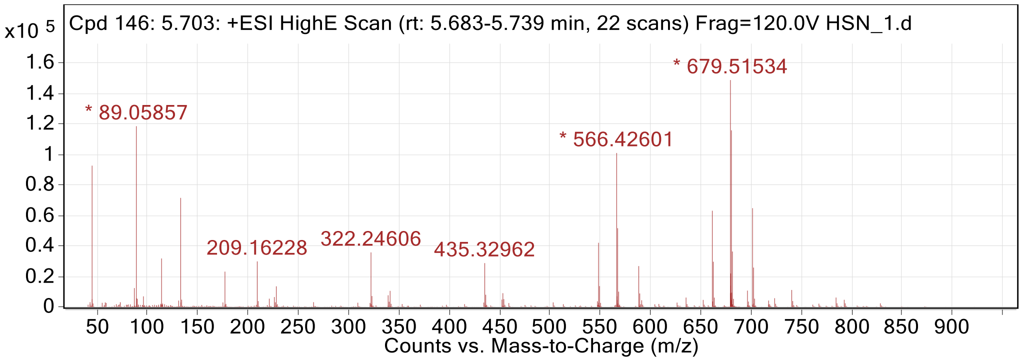

Peak List

m/z	z	Abund
45.03329	1	92523.69
89.05857	1	118392.42
133.08389	1	71381.57
548.41311	1	41948.44
566.42601	1	100818.03
567.42674	1	51507.61
661.49766	1	62965.73
679.51534	1	148623.31
680.51244	1	115666.43
701.48893	1	64636.16

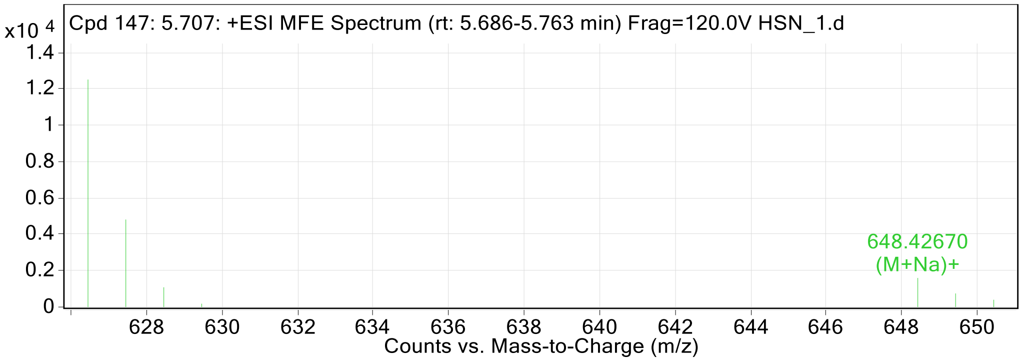

Peak List

m/z	z	Abund	Ion
626.44403	1	12516.07	(M+H)+
627.4466	1	4813.39	(M+H)+
628.45249	1	1085.27	(M+H)+
629.45671	1	182.31	(M+H)+
648.4267	1	1598.25	(M+Na)+
649.42821	1	745.82	(M+Na)+
650.43953	1	399.37	(M+Na)+

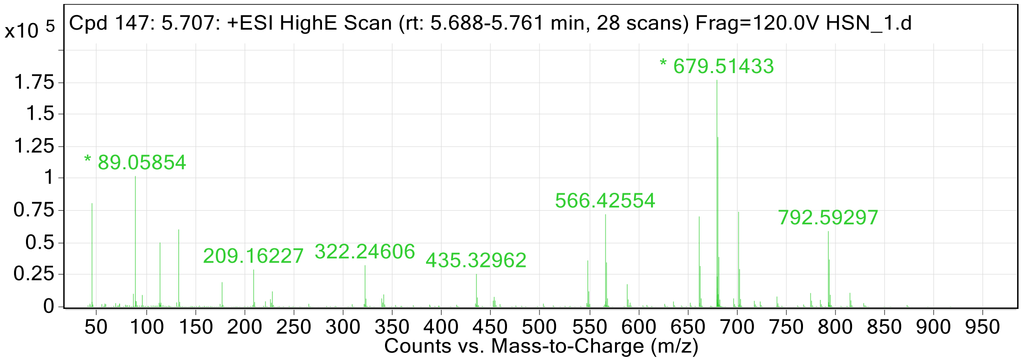

Peak List

m/z	z	Abund
45.03327		80787.29
89.05854	1	101903.42
114.08961	1	50114.02
133.0839	1	60311.48
566.42554	1	72133.82
661.49741	1	70414.04
679.51433	1	176811.83
680.51216	1	132282.72
701.48901	1	74066.06
792.59297	1	59016.79

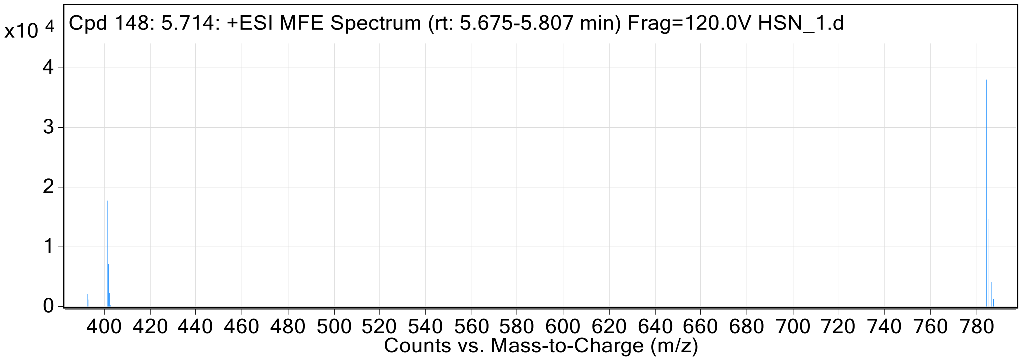

Peak List

m/z	z	Abund	Ion
392.74501	2	2118.13	(M+2H)+2
393.24585	2	1167.9	(M+2H)+2
401.25836	2	17787.46	(M+H5N)+2
401.76018	2	7115.03	(M+H5N)+2
402.26176	2	2294.1	(M+H5N)+2
402.76093	2	239.74	(M+H5N)+2
784.48482	1	38106.11	(M+H)+
785.48932	1	14657.75	(M+H)+
786.49115	1	4096.62	(M+H)+
787.49125	1	1226.93	(M+H)+

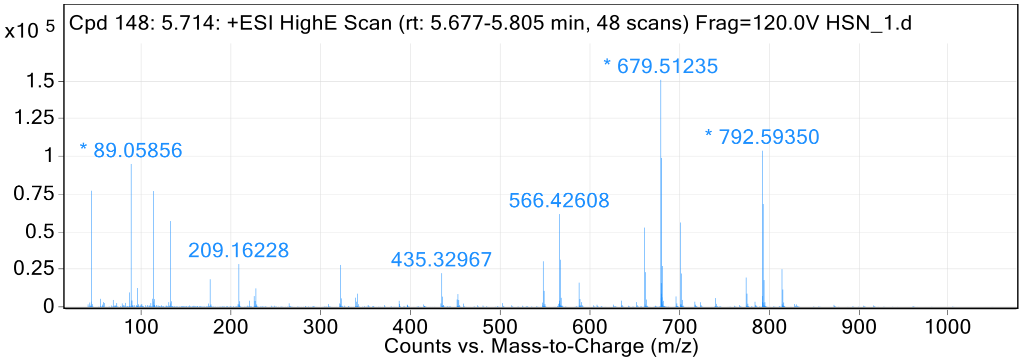

Peak List

m/z	z	Abund
45.03327		77206.05
89.05856	1	94874.34
114.08969	1	76771.34
133.08393	1	57036.32
566.42608	1	61601.16
679.51235	1	150769.17
680.51174	1	98982.93
701.48896	1	56021.41
792.5935	1	103788.4
793.59449	1	68429.23

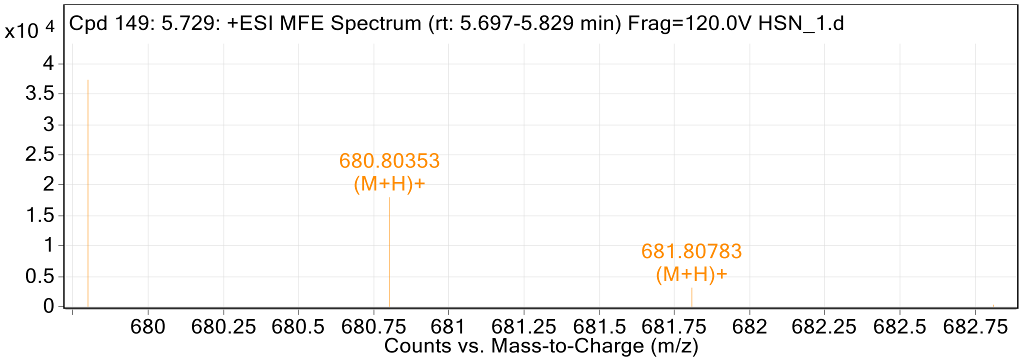

Peak List

m/z	z	Abund	Ion
679.80042	1	37341.36	(M+H)+
680.80353	1	18020.03	(M+H)+
681.80783	1	3142.45	(M+H)+
682.81123	1	339.65	(M+H)+

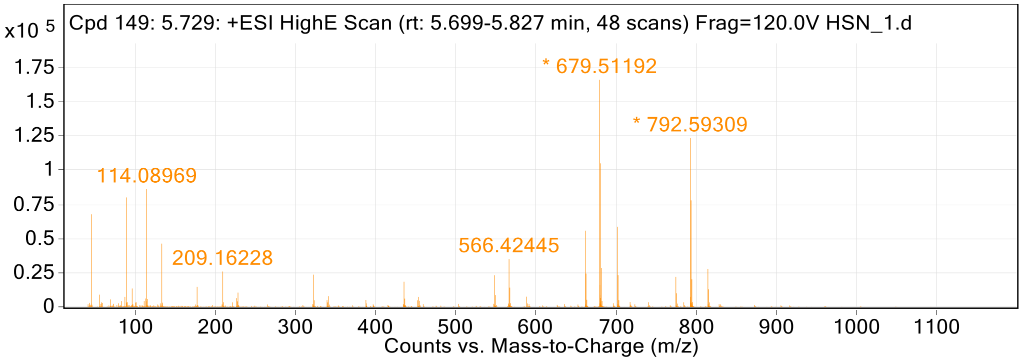

Peak List

m/z	z	Abund
45.03323		67676.51

89.05851	1	80048.31
114.08969	1	86073.75
133.08393	1	46245.53
661.49721	1	55731.17
679.51192	1	166140.83
680.51168	1	104987.74
701.48895	1	58619.02
792.59309	1	123445.06
793.59441	1	77881.41

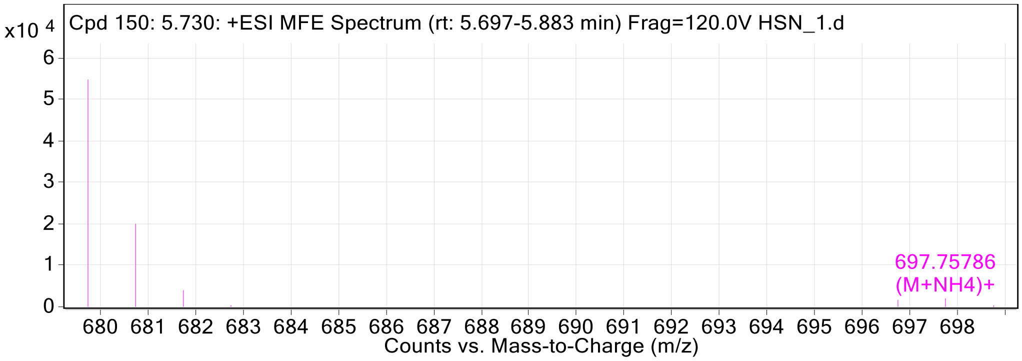

Peak List

m/z	z	Abund	Ion
679.73247	1	54803.53	(M+H)+
680.73437	1	20032.55	(M+H)+
681.73839	1	4020.41	(M+H)+
682.73742	1	399.97	(M+H)+
696.75881	1	1647.16	(M+NH4)+
697.75786	1	2043.63	(M+NH4)+
698.77307	1	342.64	(M+NH4)+

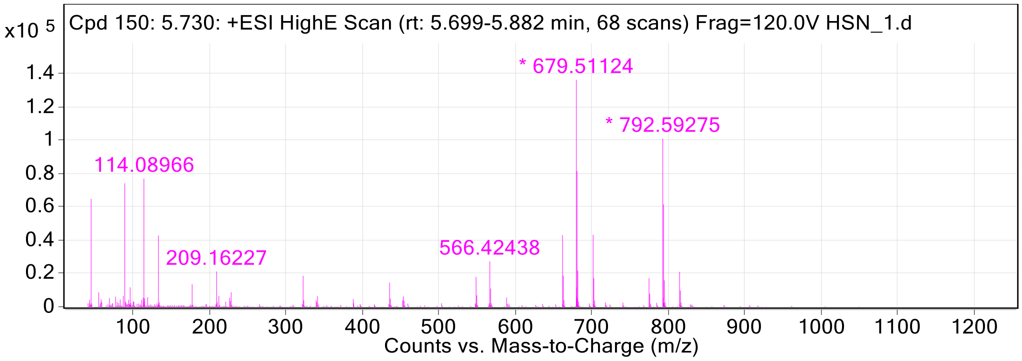

Peak List

m/z	z	Abund
45.03321		64767.16
89.05849	1	74130.61
114.08966	1	77000.77
133.08394	1	42794.66
661.49714	1	42970.55
679.51124	1	136275.63
680.5115	1	81474.66
701.48894	1	43225.96
792.59275	1	100982.71
793.59433	1	61433.92

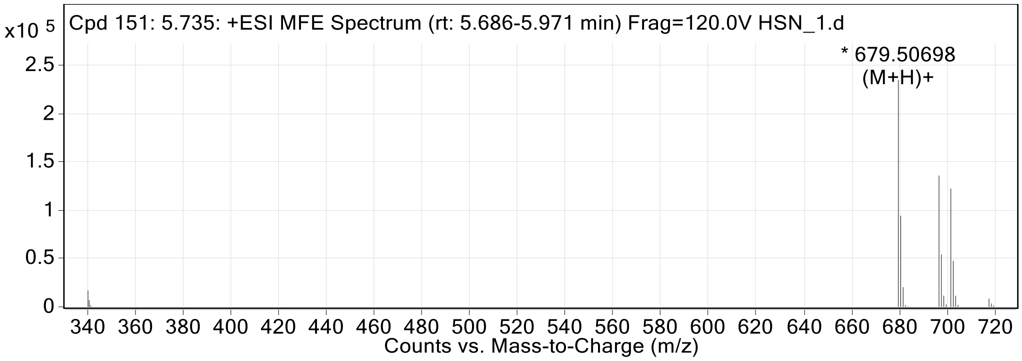

Peak List

m/z	z	Abund	Ion
340.25666	2	16970.69	(M+2H)+2
679.50698	1	235117.38	(M+H)+
680.51019	1	94532.83	(M+H)+
681.51268	1	20442.91	(M+H)+
696.53178	1	135970.45	(M+NH4)+
697.53622	1	54374.11	(M+NH4)+
698.53897	1	11657.67	(M+NH4)+
701.48844	1	122522.52	(M+Na)+
702.49211	1	47660.57	(M+Na)+
703.49501	1	11482.91	(M+Na)+

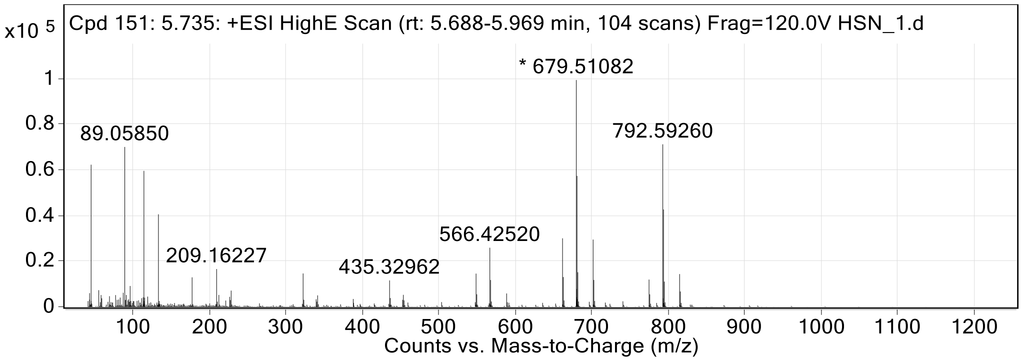

Peak List

m/z	z	Abund
45.03321		62236.85
89.0585	1	70011.16
114.08963	1	59524.63
133.08398	1	40506.85
661.4971	1	29983.12
679.51082	1	99339.14
680.5114	1	57340.92
701.48894	1	29429.39
792.5926	1	71174.51
793.59429	1	42610.76

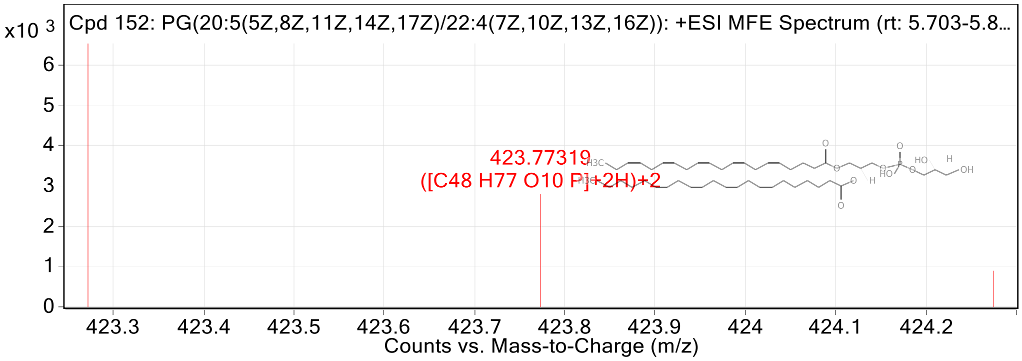

Peak List

m/z	z	Abund	Name	Formula	Ion	Score (DB)	Hits (DB)
423.2716	2	6538.7	PG(20:5(5Z,8Z,11Z,14Z,17Z)/ 22:4(7Z,10Z,13Z,16Z))	C48 H77 O10 P	(M+2H)+2	83.47	4
423.77319	2	2798.78		C48 H77 O10 P	(M+2H)+2		
424.27509	2	897.03		C48 H77 O10 P	(M+2H)+2		

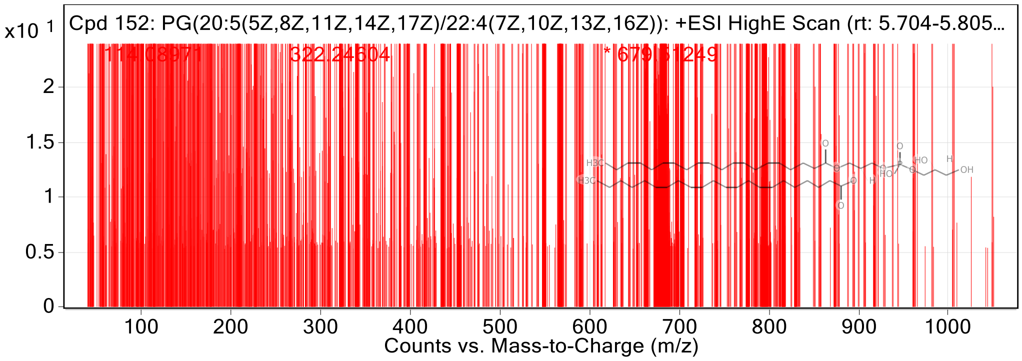

Peak List

m/z	z	Abund
45.03323		66758.8
89.05851	1	79855.32
114.08971	1	86683.55
133.08392	1	46152.53
661.49726	1	65826.65
679.51249	1	187238.05
680.51179	1	123714.73
701.48897	1	70414.1
792.5935	1	131091.84
793.59449	1	86431.4

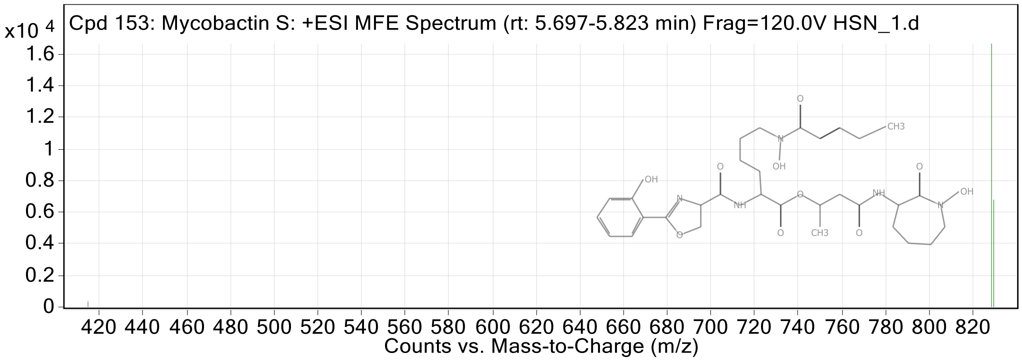

Peak List

m/z	z	Abund	Name	Formula	Ion	Score (DB)	Hits (DB)
414.76007	2	345.8			(M+2H)+2		

828.51084	1	16655.94	Mycobactin S	C44 H69 N5 O10	(M+H)+	65.81	1
829.51473	1	6785.28		C44 H69 N5 O10	(M+H)+		

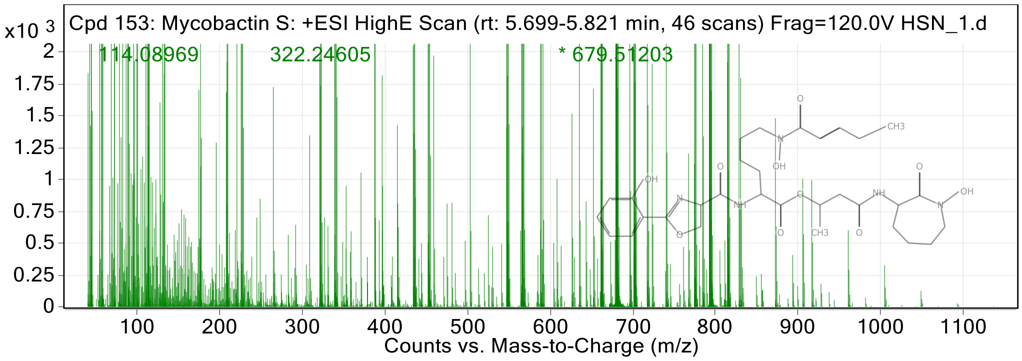

Peak List

m/z	z	Abund
45.03323		67961.52
89.05851	1	80658.18
114.08969	1	86002.1
133.08392	1	46607.89
661.49721	1	57399.68
679.51203	1	169491.09
680.5117	1	108115.69
701.48896	1	60617.43
792.59317	1	124489.47
793.59442	1	79231.37

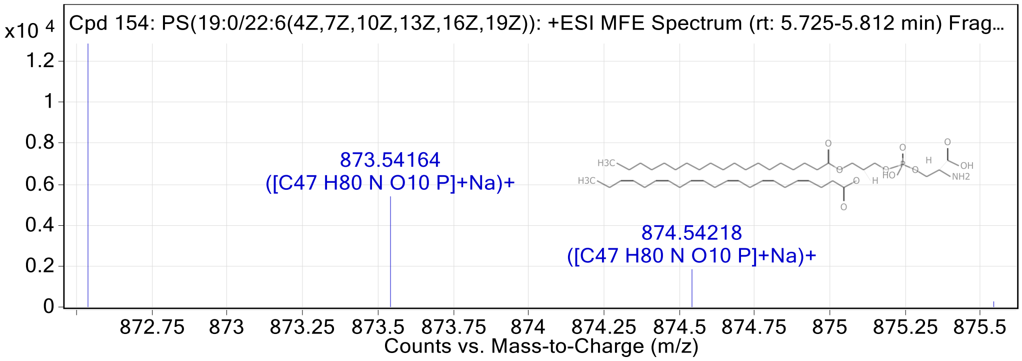

Peak List

m/z	z	Abund	Name	Formula	Ion	Score (DB)	Hits (DB)
872.53767	1	12879.14	PS(19:0/22:6(4Z,7Z,10Z,13Z,16Z,19Z))	C47 H80 N O10 P	(M+Na)+	80.22	2
873.54164	1	5409.73		C47 H80 N O10 P	(M+Na)+		
874.54218	1	1846.98		C47 H80 N O10 P	(M+Na)+		
875.54377	1	274.11		C47 H80 N O10 P	(M+Na)+		

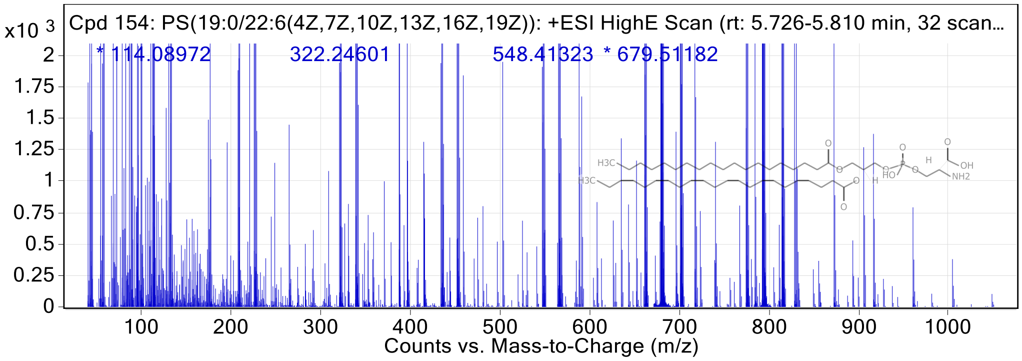

Peak List

m/z	z	Abund
45.03321		60698.74
89.05848	1	70252.06
114.08972	1	102753.88
133.08393	1	40697.71
661.49713	1	57404.33
679.51182	1	174151.08
680.51162	1	108779.07
701.48897	1	63848.65
792.59337	1	164258.55
793.59447	1	106829.14

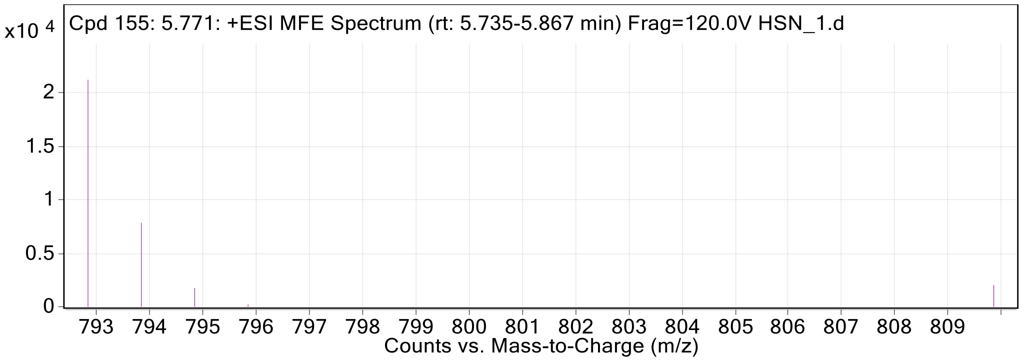

Peak List

m/z	z	Abund	Ion
792.83284	1	21249.6	(M+H)+
793.83623	1	7859.94	(M+H)+
794.83916	1	1763.1	(M+H)+
795.84158	1	223.6	(M+H)+
809.86016	1	2045.07	(M+NH4)+

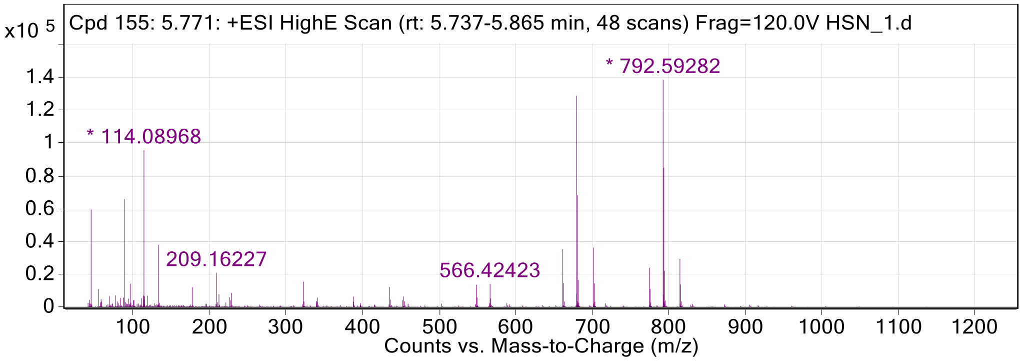

Peak List

m/z	z	Abund

Qualitative Analysis Report

45.0332		59396.7
89.05846	1	65746.03
114.08968	1	95676.38
133.08397	1	37759.28
661.49683	1	35243.78
679.50967	1	128916.65
680.51093	1	68191.38
701.48896	1	36104.06
792.59282	1	138641.61
793.59434	1	84955.8

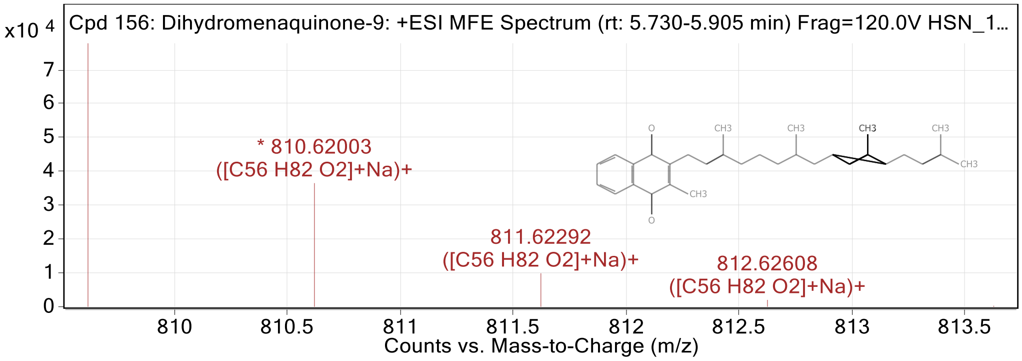

Peak List

m/z	z	Abund	Name	Formula	Ion	Score (DB)	Hits (DB)
809.61733	1	77841.87	Dihydromenaquinone-9	C56 H82 O2	(M+Na)+	75.61	1
810.62003	1	36527.66		C56 H82 O2	(M+Na)+		
811.62292	1	9880.91		C56 H82 O2	(M+Na)+		
812.62608	1	2026.38		C56 H82 O2	(M+Na)+		
813.62826	1	288.36		C56 H82 O2	(M+Na)+		

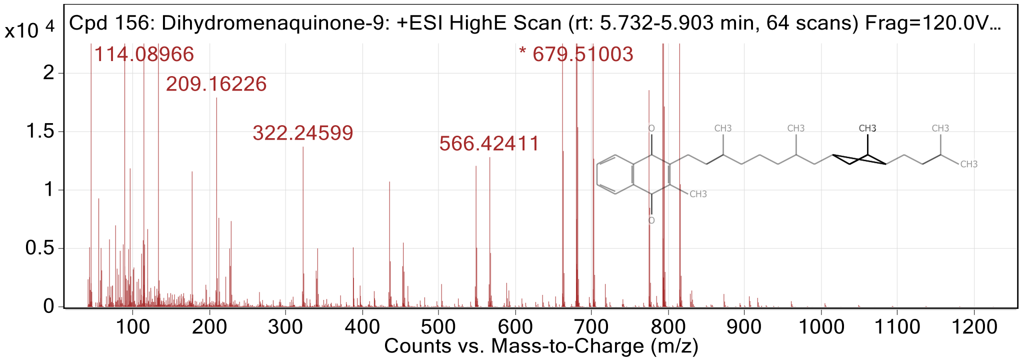

Peak List

m/z	z	Abund
45.03319		58582.86
89.05847	1	64127.64
114.08966	1	81148.38
133.08397	1	36859.63
661.49693	1	31792.58
679.51003	1	113844.41
680.51114	1	61394.86
701.48894	1	32043.72
792.5927	1	109969.07
793.59431	1	66522.98

Qualitative Analysis Report

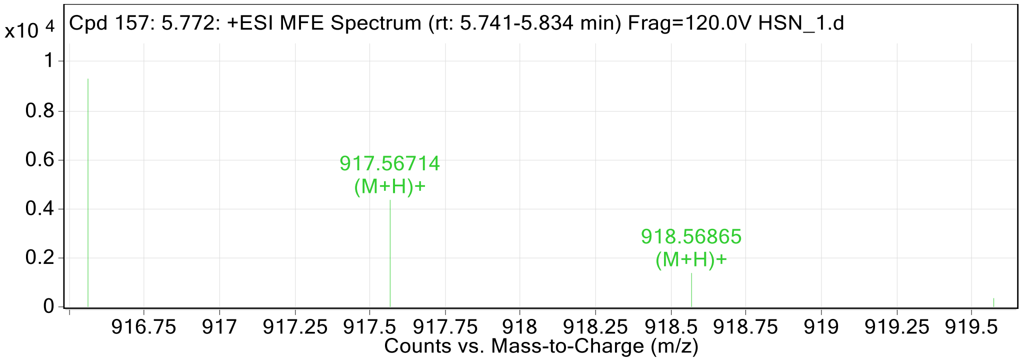

Peak List

m/z	z	Abund	Ion
916.56309	1	9306.32	(M+H)+
917.56714	1	4362.59	(M+H)+
918.56865	1	1380.83	(M+H)+

m/z	z	Abund	Ion
919.57286	1	356.21	(M+H)+

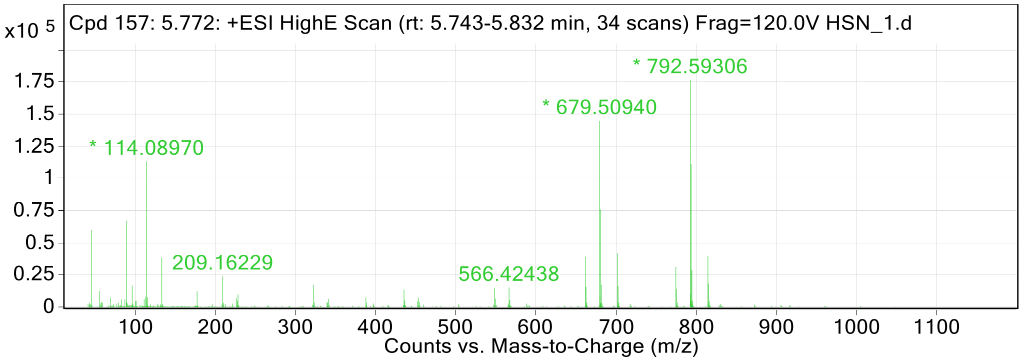

Peak List

m/z	z	Abund
45.0332		59804.47
89.05848	1	67052.45
114.0897	1	113078.89
661.49671	1	39103.41
679.5094	1	144698.14
680.51084	1	75806.05
701.48897	1	41680.11
792.59306	1	176433.63
793.5944	1	110913.29
814.57261	1	39434.62

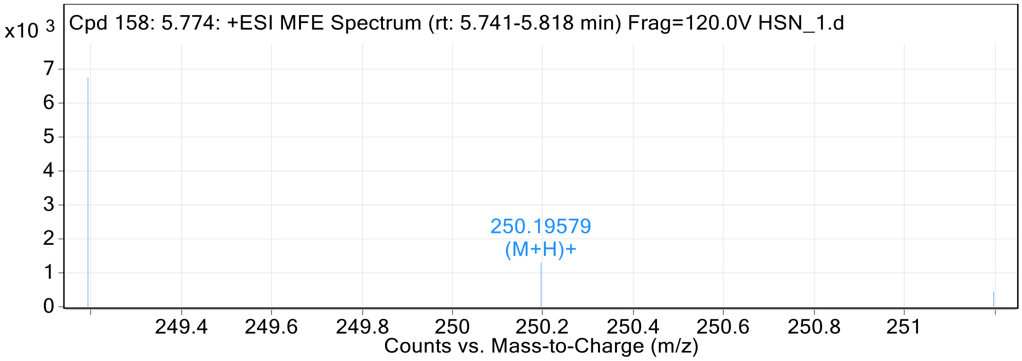

Peak List

m/z	z	Abund	Ion
249.19347	1	6731.49	(M+H)+
250.19579	1	1294.29	(M+H)+
251.19735	1	446.62	(M+H)+

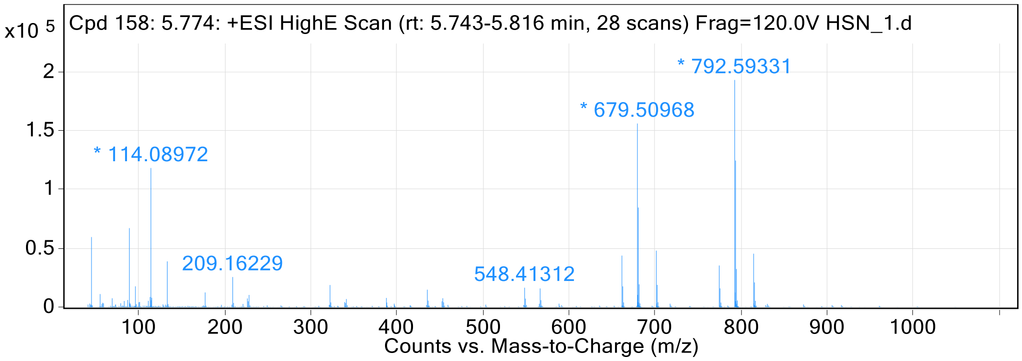

Peak List

m/z	z	Abund

45.0332		59505.09
89.05848	1	67106.11
114.08972	1	118253.29
661.4967	1	43736.01
679.50968	1	156101.14
680.51093	1	84604.04
701.48899	1	47941.44
792.59331	1	193136.05
793.59447	1	124584.23
814.57259	1	45355.04

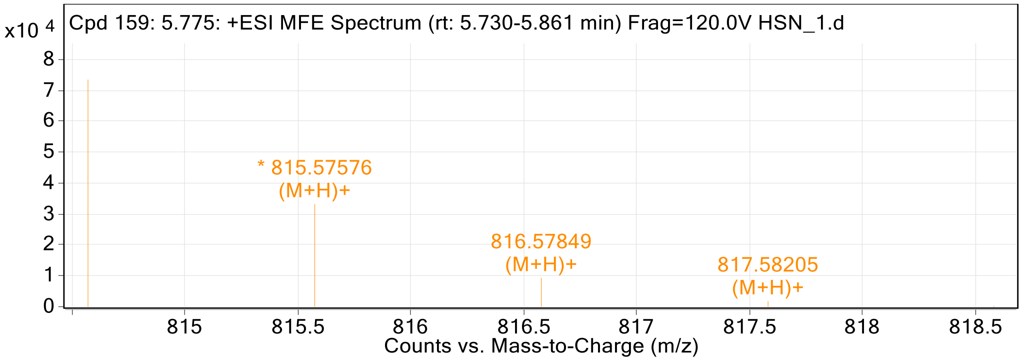

Peak List

m/z	z	Abund	Ion
814.57202	1	73472.94	(M+H)+
815.57576	1	33235.33	(M+H)+
816.57849	1	9337.43	(M+H)+
817.58205	1	1824.3	(M+H)+
818.58118	1	287.45	(M+H)+

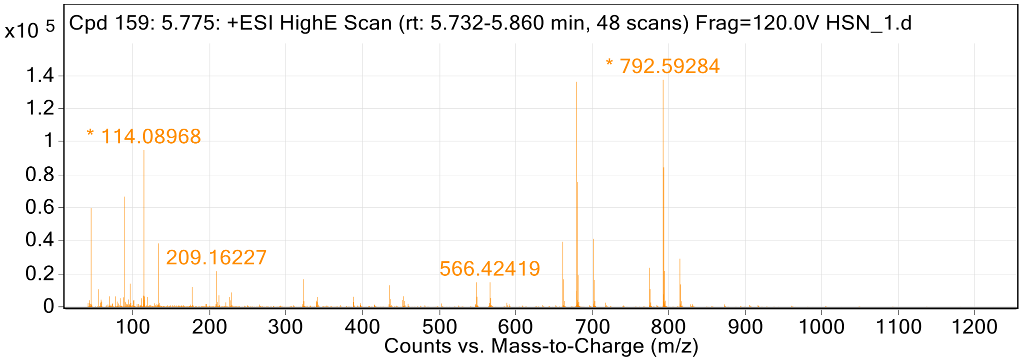

Peak List

m/z	z	Abund
45.03319		59890.29
89.05846	1	66788.39
114.08968	1	94974.12
133.08395	1	38400.85
661.49696	1	39457.46
679.51035	1	136353.39
680.51122	1	75674.57
701.48895	1	41251.77
792.59284	1	137542.73
793.59434	1	84487.07

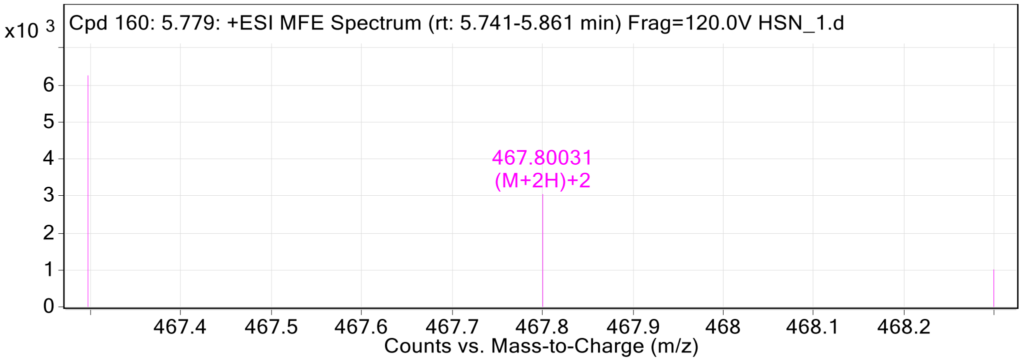

Peak List

m/z	z	Abund	Ion
467.29723	2	6260.75	(M+2H)+2
467.80031	2	3049.44	(M+2H)+2
468.29918	2	1012.18	(M+2H)+2

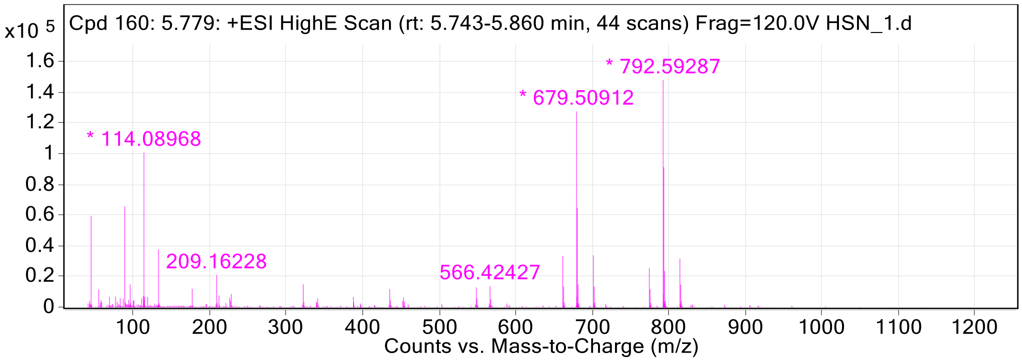

Peak List

m/z	z	Abund
45.03319		59475.7
89.05846	1	65568.01
114.08968	1	100672.57
133.08397	1	37609.09
661.49668	1	33225.46
679.50912	1	127477.57
680.51073	1	64602.39
701.48896	1	33723.84
792.59287	1	147880.98
793.59436	1	91092.28

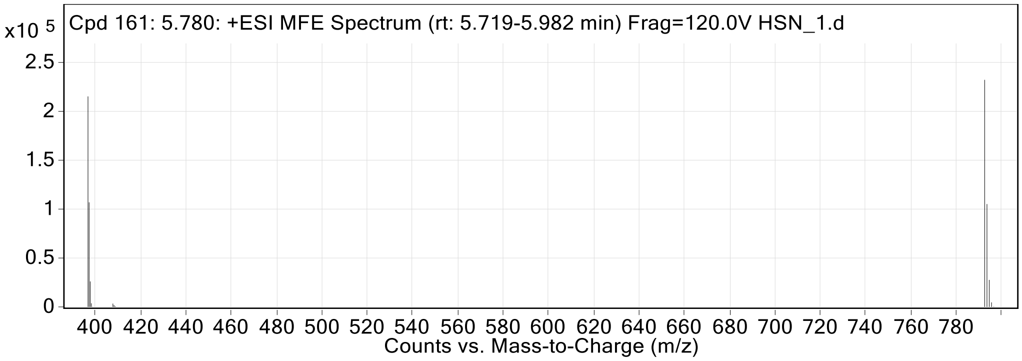

Peak List

m/z	z	Abund	Ion
396.79817	2	215813.34	(M+2H)+2
397.30004	2	107199.65	(M+2H)+2

397.8014	2	26118.51	(M+2H)+2
398.30277	2	3712.94	(M+2H)+2
407.78912	2	3397.21	(M+HNa)+2
408.28988	2	1877.33	(M+HNa)+2
792.59129	1	232798.08	(M+H)+
793.59404	1	105456.5	(M+H)+
794.59589	1	27684.89	(M+H)+
795.5984	1	4627.79	(M+H)+

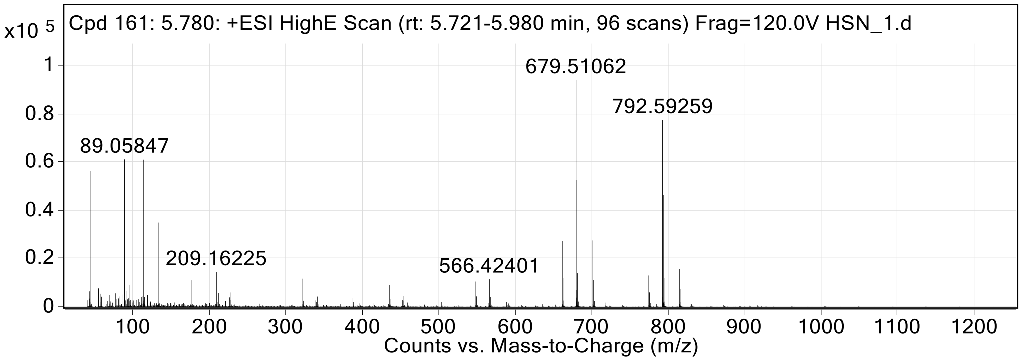

Peak List

m/z	z	Abund
45.03319		56424.79
89.05847	1	61159.77
114.08964	1	61046.96
133.08401	1	34926.34
661.49709	1	27321.45
679.51062	1	94142.09
680.51139	1	52653.25
701.48893	1	27508.84
792.59259	1	77562.43
793.59429	1	46381.21

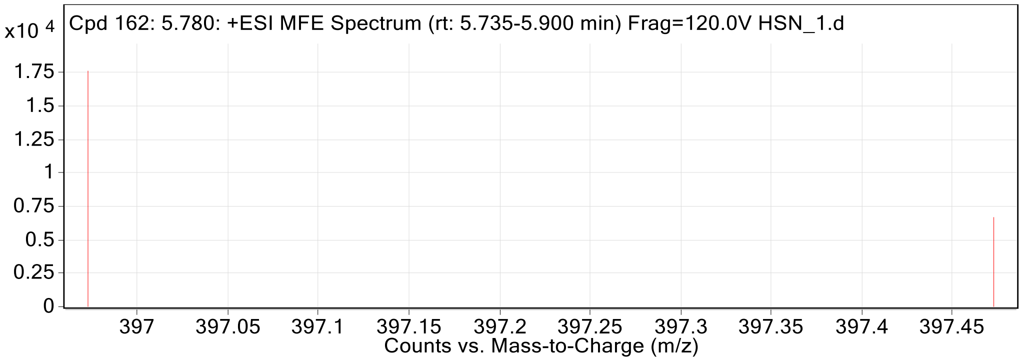

Peak List

m/z	z	Abund	Ion
396.97275	2	17608.38	(M+2H)+2
397.47287	2	6684.61	(M+2H)+2

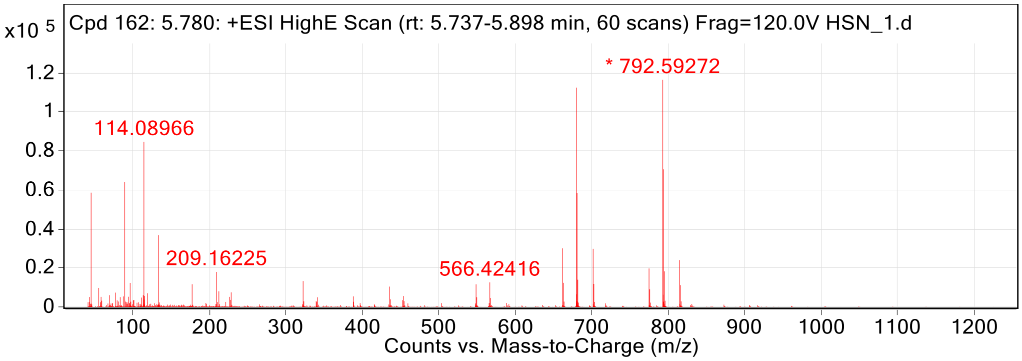

Peak List

m/z	z	Abund
45.03319		58559.84
89.05846	1	63887.87
114.08966	1	84598.15
133.08399	1	36754.31
661.4968	1	29989.28
679.50946	1	112397.96
680.51088	1	58277.07
701.48895	1	29778.86
792.59272	1	116372.01
793.59431	1	70498.01

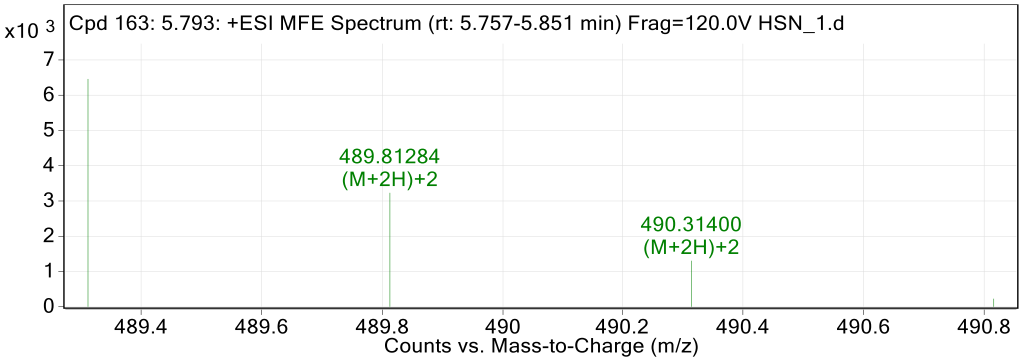

Peak List

m/z	z	Abund	Ion
489.31089	2	6465.49	(M+2H)+2
489.81284	2	3234.37	(M+2H)+2
490.314	2	1306.79	(M+2H)+2
490.81677	2	229.42	(M+2H)+2

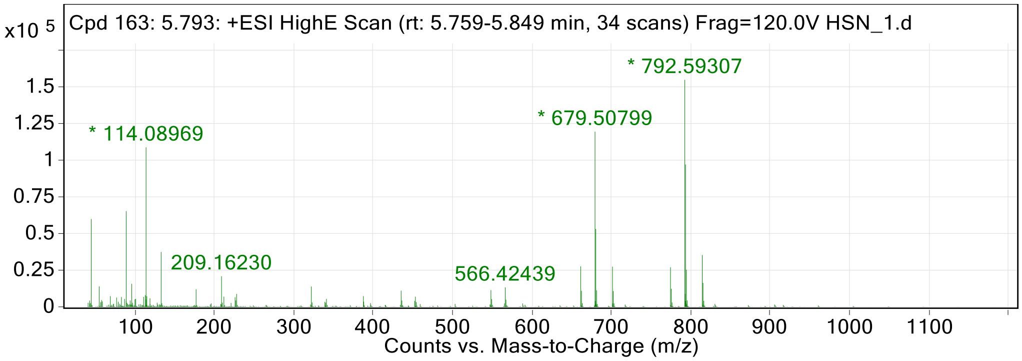

Peak List

m/z	z	Abund
45.03319		59760.7

89.05847	1	65145.69
114.08969	1	108538.13
133.08396	1	37336.05
661.49661	1	27549.15
679.50799	1	119165.02
680.5103	1	53013.78
792.59307	1	154461.2
793.59443	1	96835.72
814.57266	1	35281.76

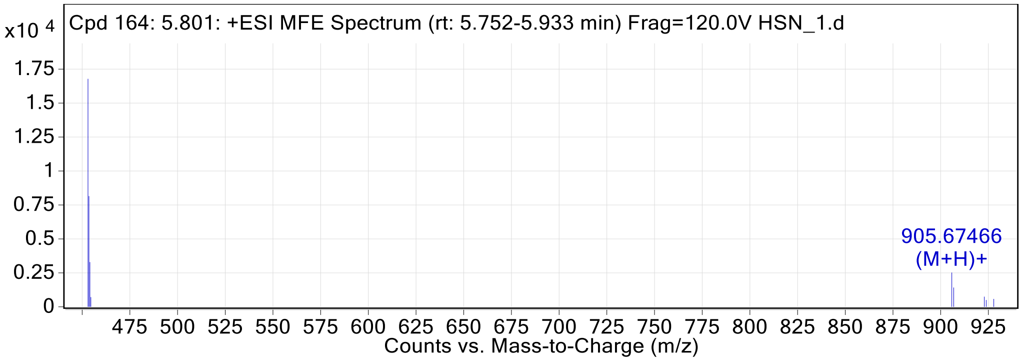

Peak List

m/z	z	Abund	Ion
453.34005	2	16791.93	(M+2H)+2
453.84176	2	8157.15	(M+2H)+2
454.3437	2	3295.27	(M+2H)+2
454.84468	2	714.94	(M+2H)+2
905.67466	1	2525.19	(M+H)+
906.67431	1	1422.9	(M+H)+
922.69912	1	748.08	(M+NH4)+
923.70019	1	501.75	(M+NH4)+
927.65667	1	582.06	(M+Na)+

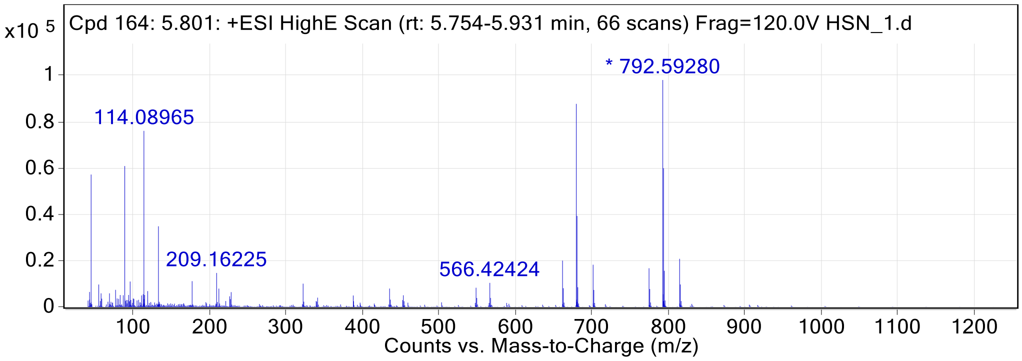

Peak List

m/z	z	Abund
45.03319		57159.95
89.05847	1	60827.41
114.08965	1	76045.11
133.08401	1	34783.8

m/z	z	Abund
661.49656	1	19951.69
679.50797	1	87713.41
680.5103	1	39269.48
792.5928	1	98025.88
793.59435	1	59884.77
814.57263	1	20717.1

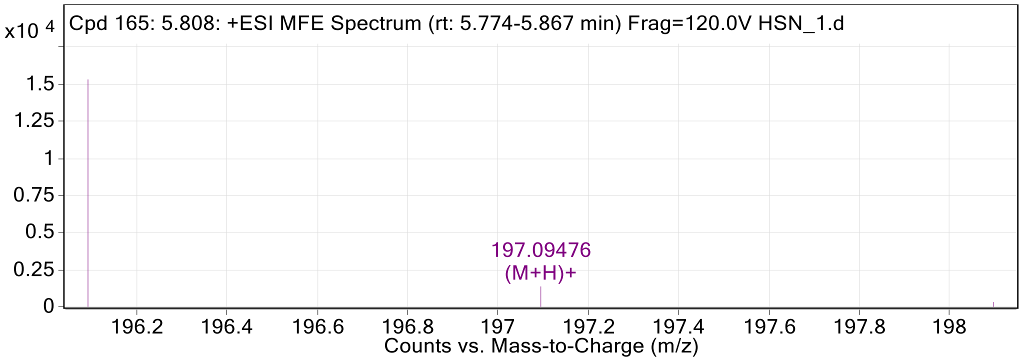

Peak List

m/z	z	Abund	Ion
196.09202	1	15311.29	(M+H)+
197.09476	1	1371.99	(M+H)+
198.09756	1	319.42	(M+H)+

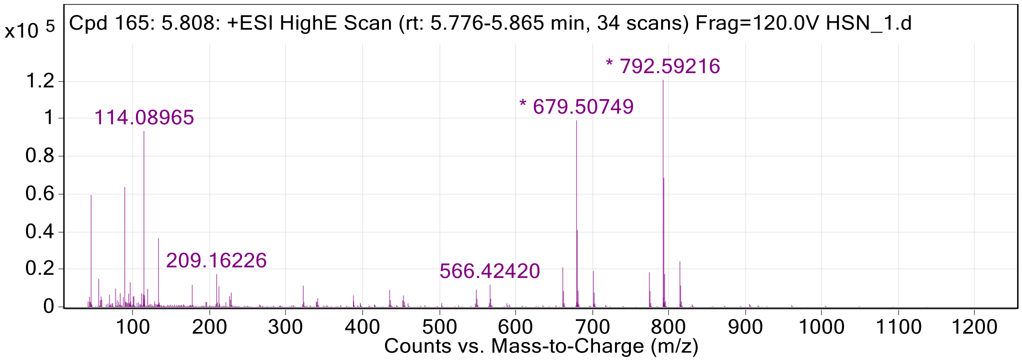

Peak List

m/z	z	Abund
45.03319		59479.22
89.05846	1	63634.31
114.08965	1	93362.59
133.08397	1	36435.67
661.4965	1	20908.56
679.50749	1	99054.32
680.51008	1	40787.85
792.59216	1	120535.45
793.59406	1	68539.59
814.5726	1	24264.66

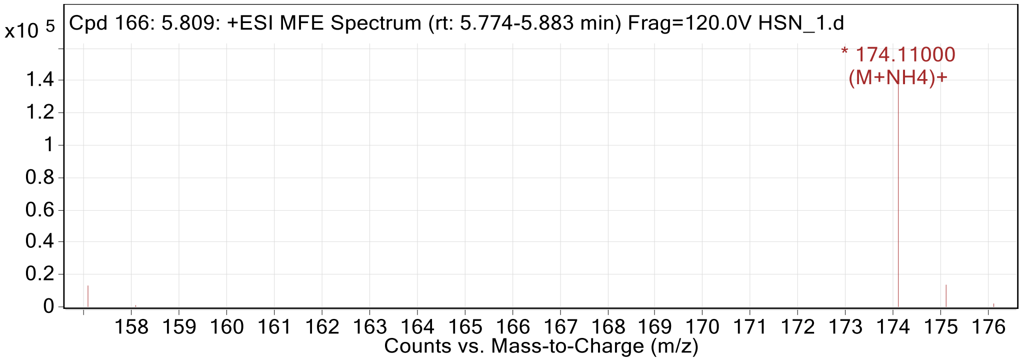

Peak List

m/z	z	Abund	Ion
157.0836	1	13203.28	(M+H)+
158.08682	1	1059.85	(M+H)+
174.11	1	140655.13	(M+NH4)+
175.11323	1	13719.6	(M+NH4)+
176.1146	1	2068.8	(M+NH4)+

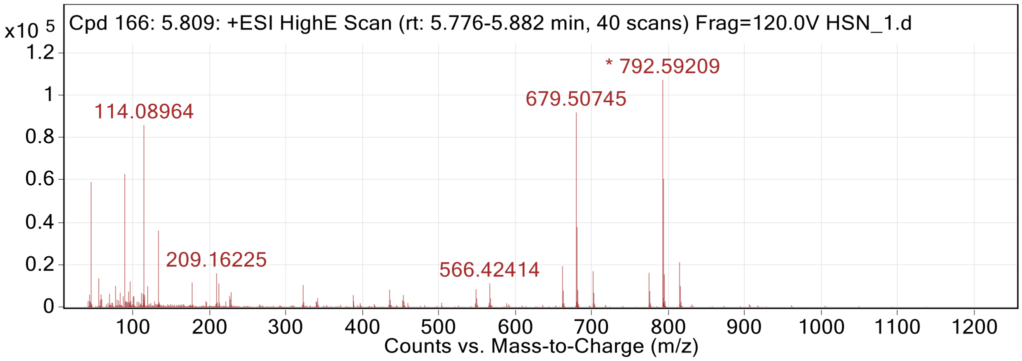

Peak List

m/z	z	Abund
45.03318		59073.67
89.05846	1	62748.12
114.08964	1	85890.23
133.08397	1	36067.75
661.49649	1	19283.21
679.50745	1	91881.95
680.51009	1	37762.94
792.59209	1	107210.11
793.59406	1	60461.76
814.57258	1	21117.46

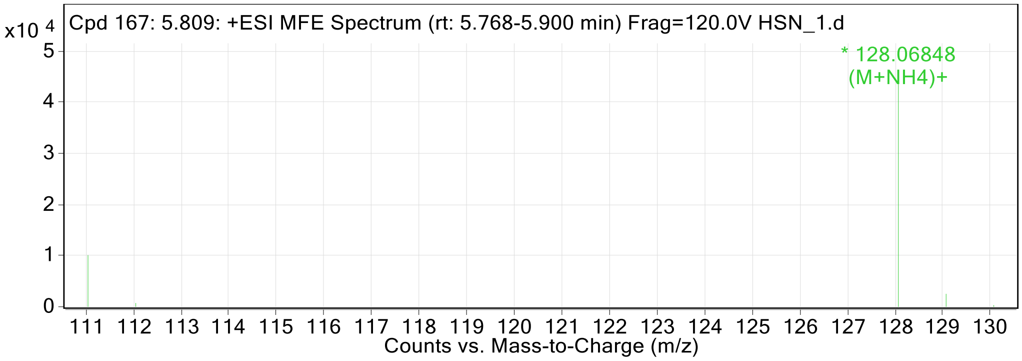

Peak List

m/z	z	Abund	Ion

111.04223	1	10092.09	(M+H)+
112.04635	1	737.96	(M+H)+
128.06848	1	44420.3	(M+NH4)+
129.07199	1	2530.3	(M+NH4)+
130.07342	1	322.66	(M+NH4)+

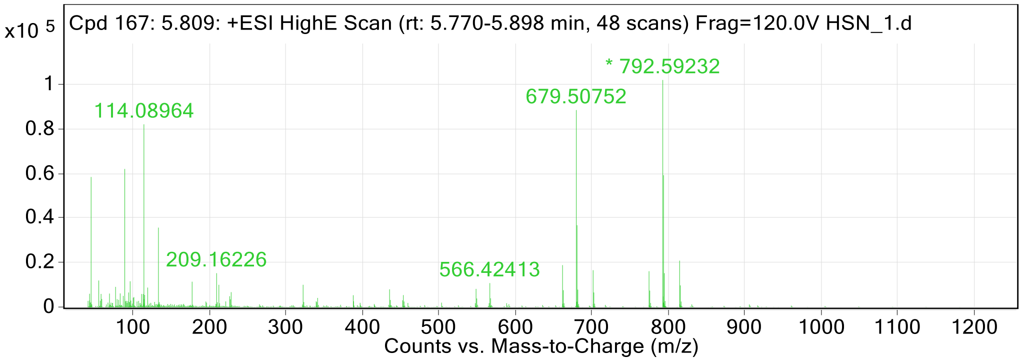

Peak List

m/z	z	Abund
45.03318		58342.18
89.05846	1	61916.91
114.08964	1	81961.49
133.08399	1	35614.9
661.49648	1	18798.55
679.50752	1	88358.59
680.5101	1	36694.88
792.59232	1	101841.43
793.59413	1	59128.32
814.57254	1	20811.48

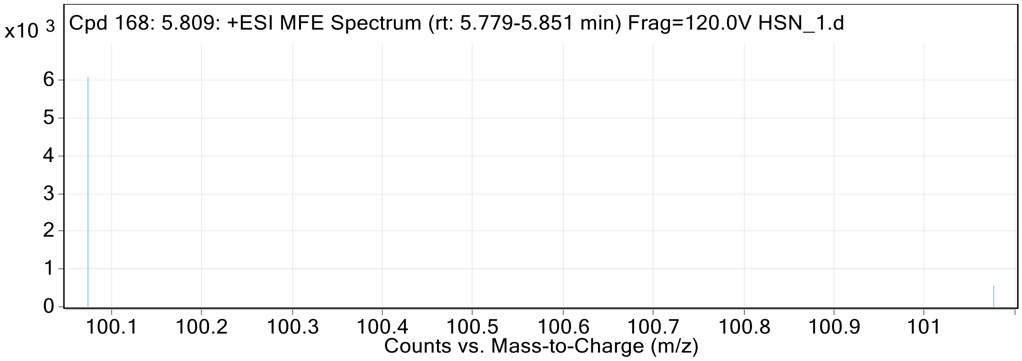

Peak List

m/z	z	Abund	Ion
100.07407	1	6091.54	(M+H)+
101.07699	1	560.57	(M+H)+

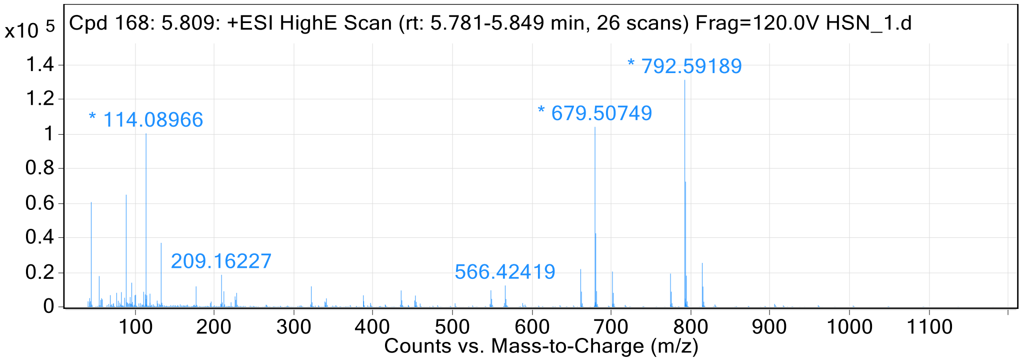

Peak List

m/z	z	Abund
45.03319		60537.06
89.05846	1	64819.69
114.08966	1	100238.02
133.08395	1	36999.9
661.49657	1	21831.2
679.50749	1	104007.16
680.51013	1	42506.56
792.59189	1	131157.86
793.59401	1	72343.89
814.57256	1	25406.93

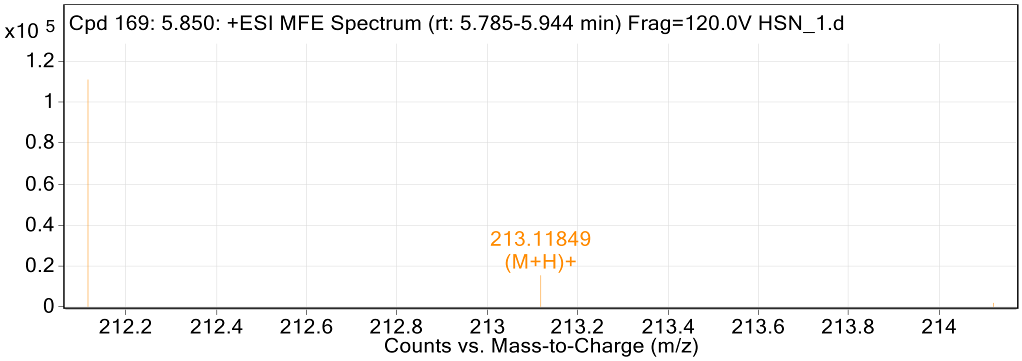

Peak List

m/z	z	Abund	Ion
212.11577	1	111147.43	(M+H)+
213.11849	1	15391.73	(M+H)+
214.12148	1	1907.28	(M+H)+

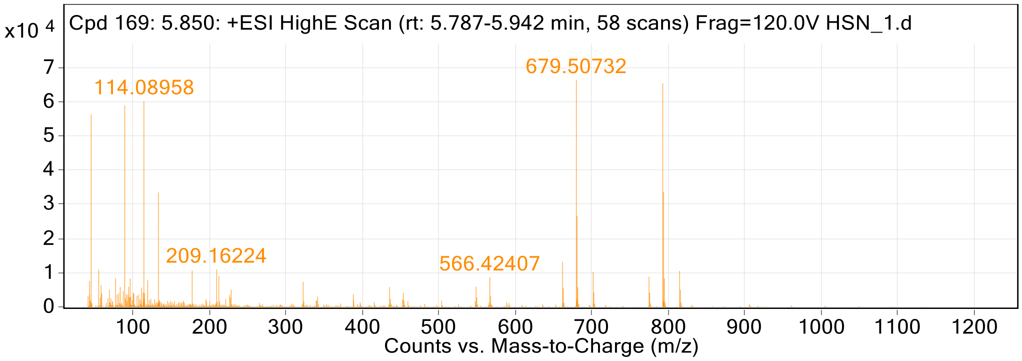

Peak List

m/z	z	Abund
45.03318		56302.66
89.05846	1	58929.68

114.08958	1	60221.02
133.08403	1	33494.17
209.16224		10971.08
661.49652	1	13169.51
679.50732	1	66410.31
680.51009	1	26635.88
792.59134	1	65384.53
793.59381	1	33587.28

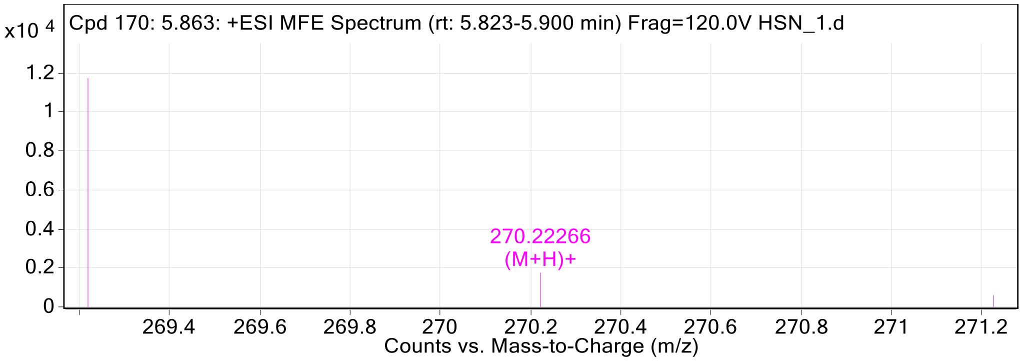

Peak List

m/z	z	Abund	Ion
269.21945	1	11720.89	(M+H)+
270.22266	1	1750.47	(M+H)+
271.22697	1	601.64	(M+H)+

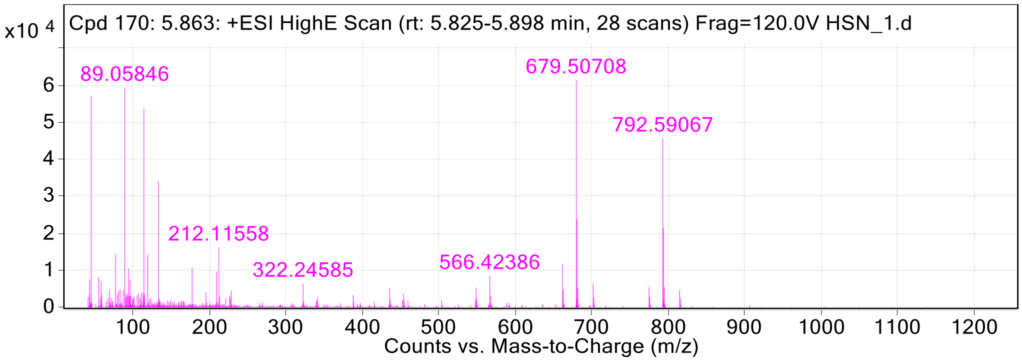

Peak List

m/z	z	Abund
45.03318		57136.88
77.03758		14319.21
89.05846	1	59346.76
114.08956	1	53646.38
133.08403	1	34106.79
212.11558	1	16160.71
679.50708	1	61378.59
680.50979	1	23809.44
792.59067	1	45629.75
793.59356	1	21312.16

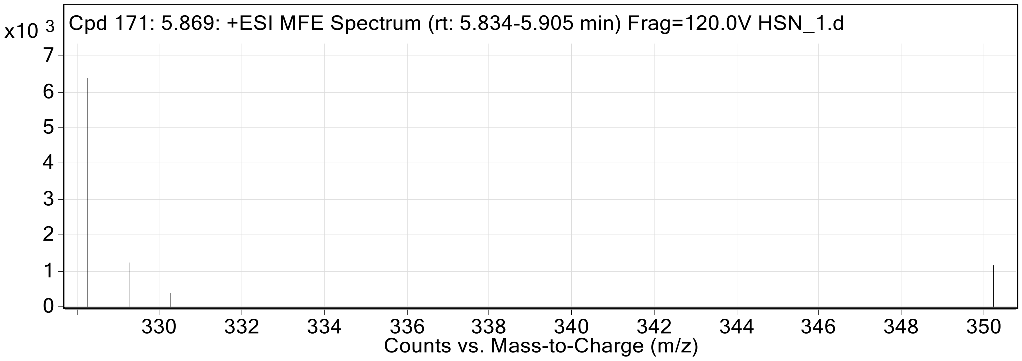

Peak List

m/z	z	Abund	Ion
328.25576	1	6387.04	(M+H)+
329.25902	1	1233.69	(M+H)+
330.25746	1	385.41	(M+H)+
350.23458	1	1157.26	(M+Na)+

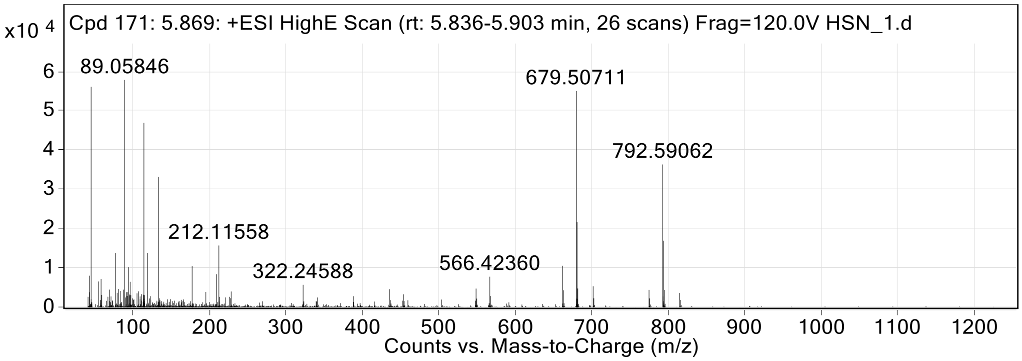

Peak List

m/z	z	Abund
45.03318		56082.62
89.05846	1	57828.29
114.08953	1	46909.63
119.05834		13804.9
133.08403	1	33183.37
212.11558	1	15664.73
679.50711	1	55013.68
680.5099	1	21615.02
792.59062	1	36307.33
793.59359	1	16888.46

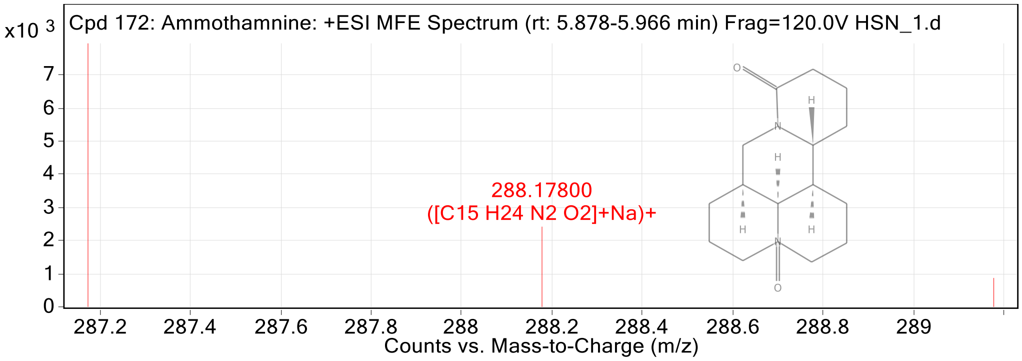

Peak List

m/z	z	Abund	Name	Formula	Ion	Score (DB)	Hits (DB)
287.17321	1	7947.91	Ammothamnine	C15 H24 N2 O2	(M+Na)+	72.31	6

288.178	1	2418.97		C15 H24 N2 O2	(M+Na)+		
289.1778	1	871.02		C15 H24 N2 O2	(M+Na)+		

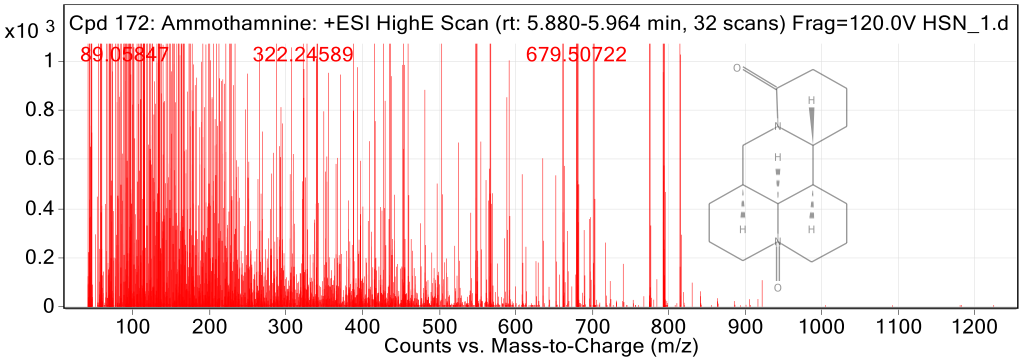

Peak List

m/z	z	Abund
43.0175		9817.17
45.03317		51122.73
89.05847	1	52263.71
91.05291		10324.58
114.08949	1	26849.33
133.08415	1	29482.68
177.11006		9299.07
679.50722	1	34182.58
680.51016	1	13528.74
792.59064	1	17945.6

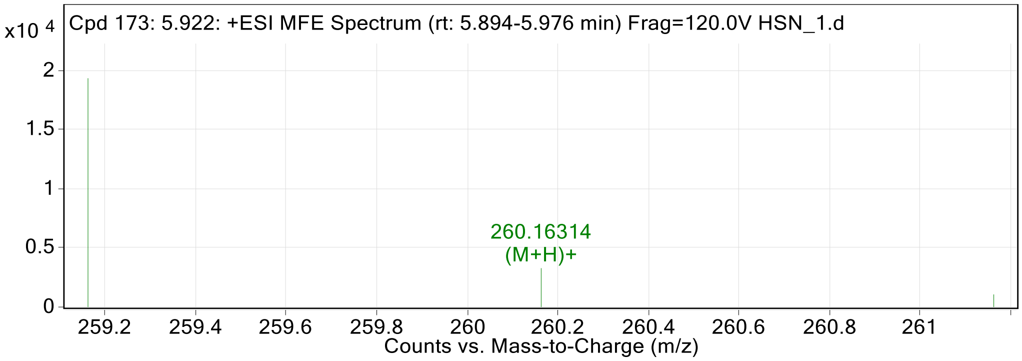

Peak List

m/z	z	Abund	Ion
259.1627	1	19316.18	(M+H)+
260.16314	1	3282.05	(M+H)+
261.16223	1	1063.12	(M+H)+

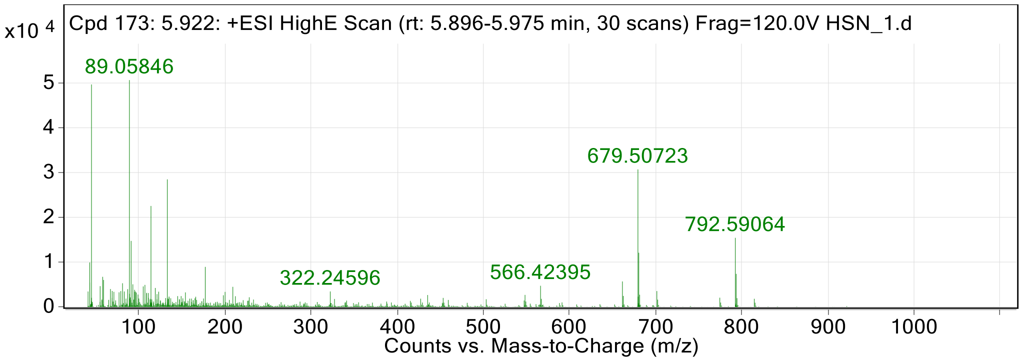

Peak List

m/z	z	Abund

43.01751		9887.78
45.03317		49584.71
89.05846	1	50564.77
91.05293	1	14697.53
114.08949	1	22492.24
133.08416	1	28418.29
177.11013		8902.41
679.50723	1	30641.4
680.51019	1	12059.67
792.59064	1	15382.77

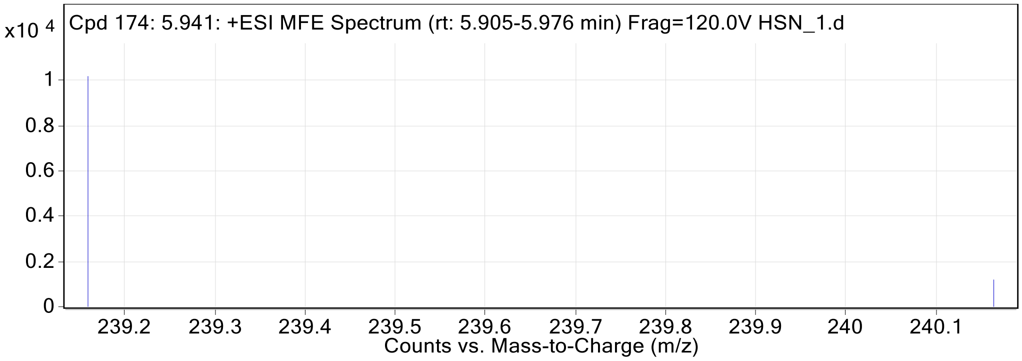

Peak List

m/z	z	Abund	Ion
239.15932	1	10182.5	(M+H)+
240.16422	1	1199.25	(M+H)+

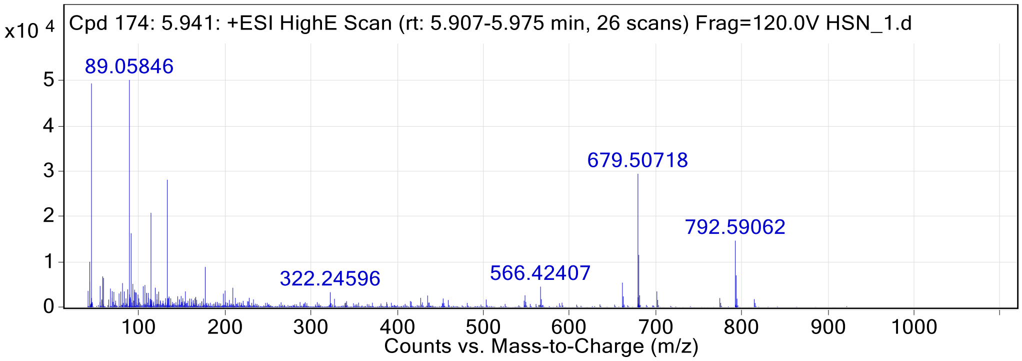

Peak List

m/z	z	Abund
43.0175		9977.16
45.03316		49336.83
89.05846	1	50105.05
91.05292	1	16267.86
114.08946	1	20781.26
133.08417	1	28088.62
177.11023		8848.65
679.50718	1	29400.3
680.51017	1	11502.38
792.59062	1	14649.65

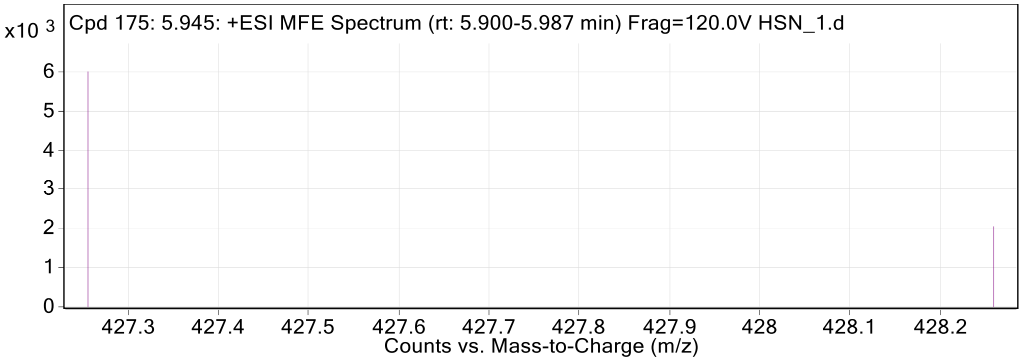

Peak List

m/z	z	Abund	Ion
427.25553	1	6008.29	(M+H)+
428.2592	1	2052.13	(M+H)+

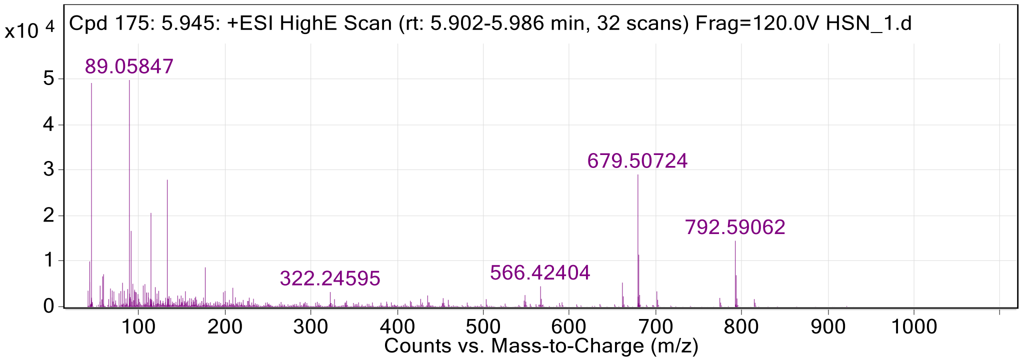

Peak List

m/z	z	Abund
43.0175		9943.73
45.03317		49152.39
89.05847	1	49810.71
91.0529	1	16654.16
114.08948	1	20608.78
133.08416	1	27895.04
177.11013		8649.15
679.50724	1	29057.44
680.51024	1	11424.94
792.59062	1	14485.25

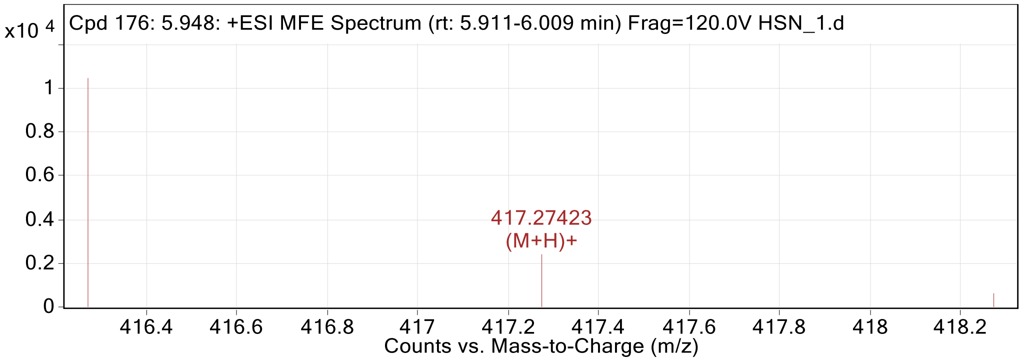

Peak List

m/z	z	Abund	Ion
416.27137	1	10480.58	(M+H)+
417.27423	1	2412.23	(M+H)+
418.27314	1	626.69	(M+H)+

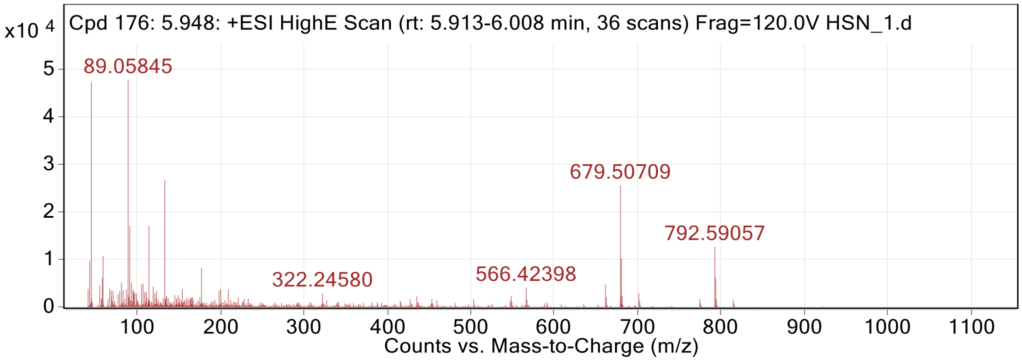

Peak List

m/z	z	Abund
43.01749		9743.09
45.03314		47314.37
59.0485		10688.47
89.05845	1	47777.48
91.05289	1	17031
114.08948		17108.01
133.08419	1	26721.23
679.50709	1	25565.82
680.51004	1	10175.95
792.59057	1	12645.38

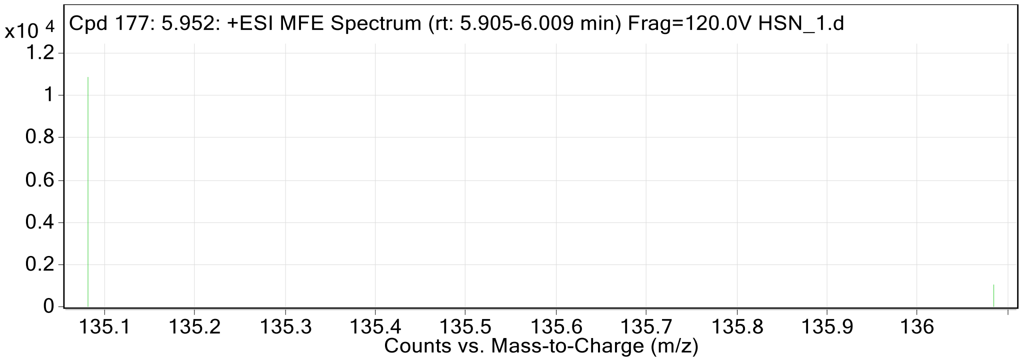

Peak List

m/z	z	Abund	Ion
135.08178	1	10885.27	(M+H)+
136.08476	1	1051.98	(M+H)+

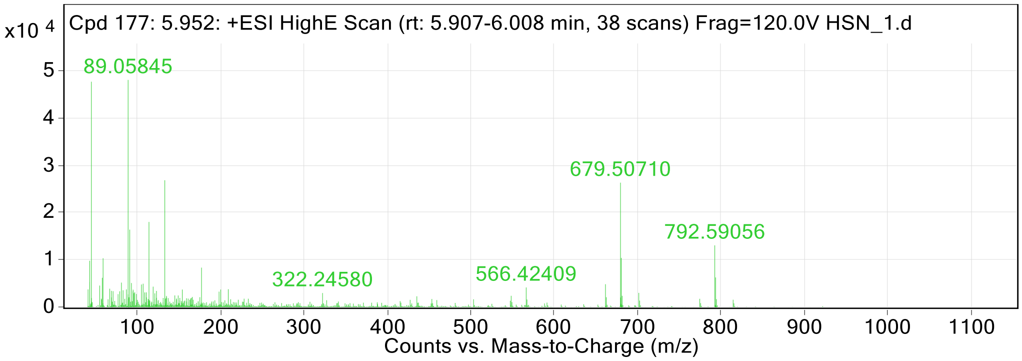

Peak List

m/z	z	Abund
43.0175		9774.08
45.03314		47661.7
59.0485		10315.15

89.05845	1	48039.2
91.05289	1	16367.11
114.08946		17988.73
133.08419	1	26808.74
679.5071	1	26303.11
680.51003	1	10381.25
792.59056	1	13036.1

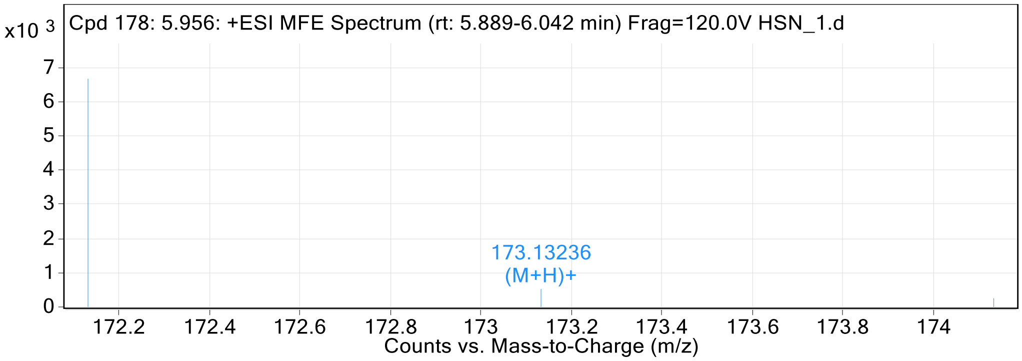

Peak List

m/z	z	Abund	Ion
172.13083	1	6667.14	(M+H)+
173.13236	1	524.23	(M+H)+
174.13327	1	255.01	(M+H)+

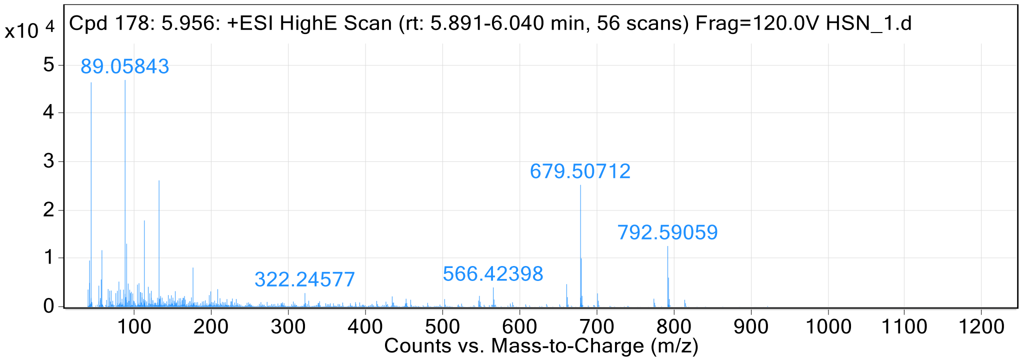

Peak List

m/z	z	Abund
43.01751		9563.25
45.03315		46416.84
59.04852		11688.44
89.05843	1	46893.09
91.0529	1	13031.44
114.08949	1	17847.18
133.08416	1	26131.4
679.50712	1	25200.44
680.51019	1	9999.27
792.59059	1	12551.18

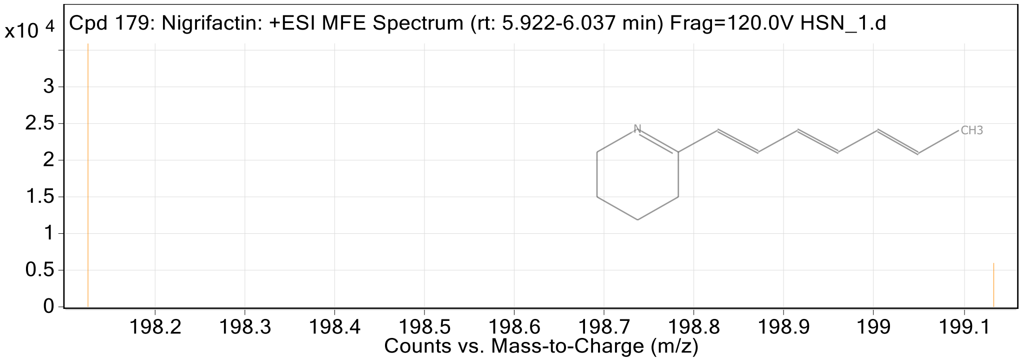

Peak List

m/z	z	Abund	Name	Formula	Ion	Score (DB)	Hits (DB)
198.12522	1	35841.89	Nigrifactin	C12 H17 N	(M+Na)+	73.61	1
199.13334	1	5973.75		C12 H17 N	(M+Na)+		

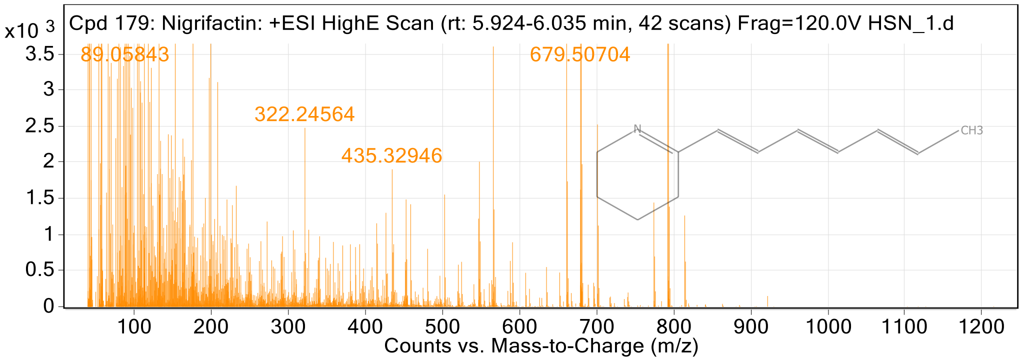

Peak List

m/z	z	Abund
43.01751		9543.06
45.03314		44999.43
59.04851		13855.01
89.05843	1	45218.25
91.0529	1	15810.76
114.08951		14171.36
133.08416	1	25175.51
679.50704	1	22284.72
680.51018	1	8858.84
792.59044	1	10847.28

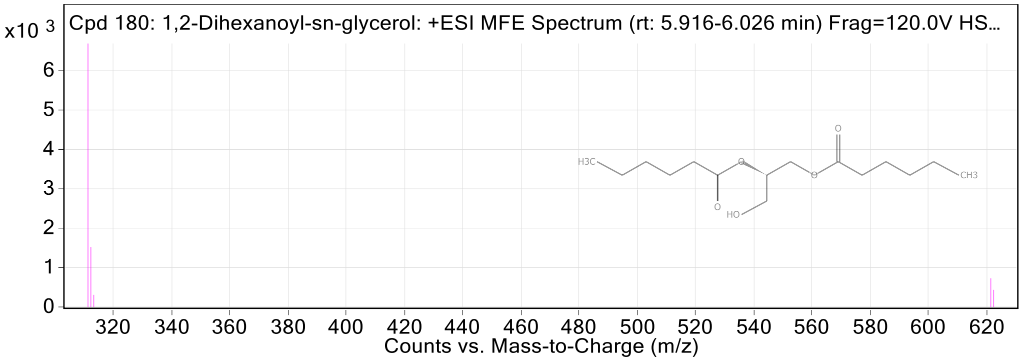

Qualitative Analysis Report

Peak List

m/z	z	Abund	Name	Formula	Ion	Score (DB)	Hits (DB)
311.18184	1	6679.78	1,2-Dihexanoyl-sn-glycerol	C15 H28 O5	(M+Na)+	85.18	2
312.18488	1	1521.88		C15 H28 O5	(M+Na)+		
313.19396	1	307.46		C15 H28 O5	(M+Na)+		
621.35846	1	726.04			(2M+H)+		
622.35552	1	434.68			(2M+H)+		

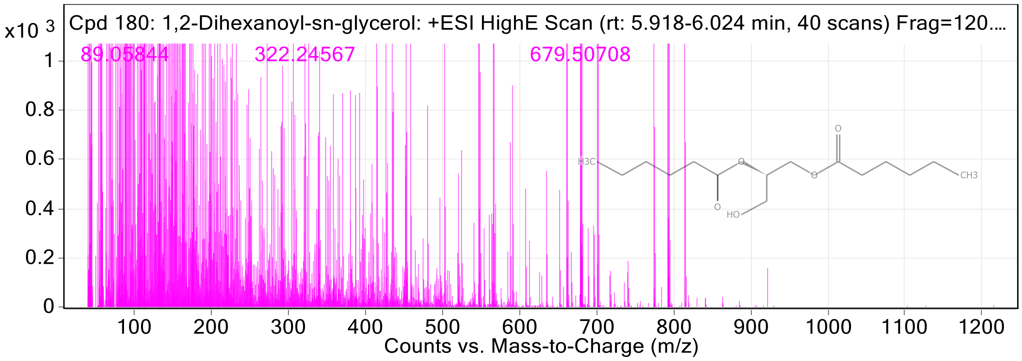

Peak List

m/z	z	Abund
43.01751		9576.25
45.03315		45766.7
59.04852		12925.95
89.05844	1	46305.33
91.0529	1	16182.52
114.08949		15246.51
133.08417	1	25712.51
679.50708	1	23535
680.51014	1	9358.11
792.59052	1	11574.69

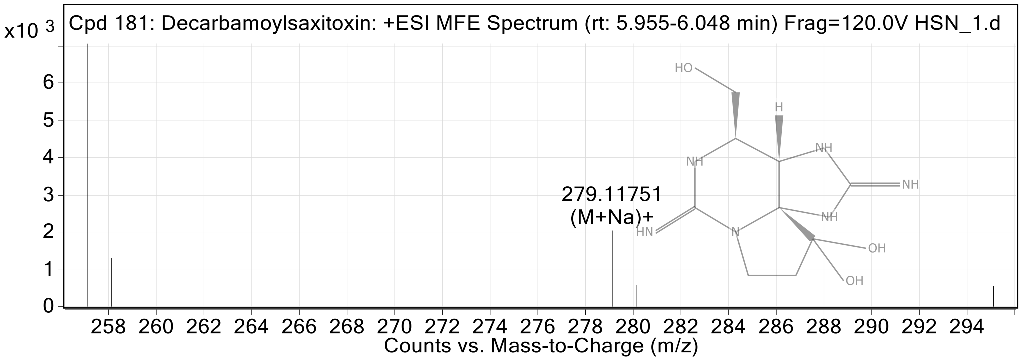

Peak List

m/z	z	Abund	Name	Formula	Ion	Score (DB)	Hits (DB)
257.13553	1	7064.15	Decarbamoylsaxitoxin	C9 H16 N6 O3	(M+H)+	70.4	1
258.13522	1	1301.27		C9 H16 N6 O3	(M+H)+		
279.11751	1	2042.87			(M+Na)+		
280.11799	1	585.76			(M+Na)+		
295.0938	1	557.39			(M+K)+		

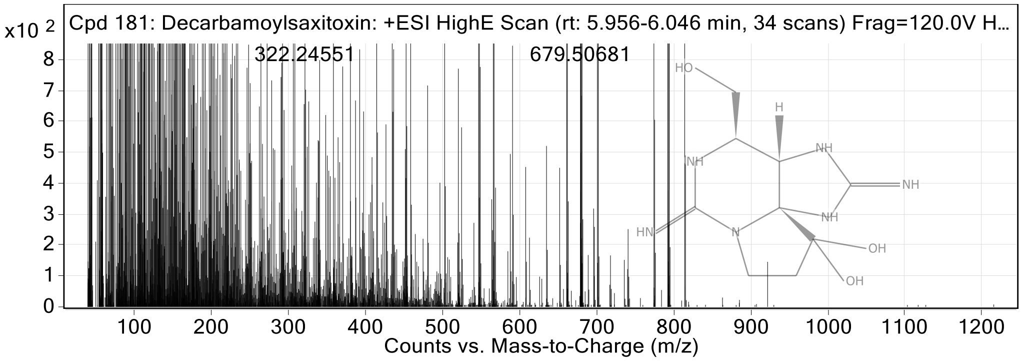

Peak List

m/z	z	Abund
43.01751		9273.76
45.03313		42817.68
59.04851		16965.79
89.0584	1	42510.06
91.05289	1	16376.08
114.08947		11336.91
133.08414	1	23579.34
679.50681	1	18589.56
680.51016	1	7488.92
792.59028	1	8885.25

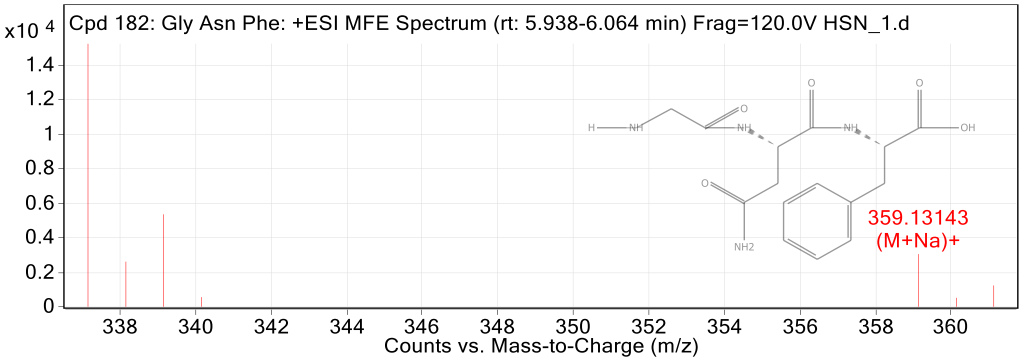

Peak List

m/z	z	Abund	Name	Formula	Ion	Score (DB)	Hits (DB)
337.14959	1	15263.64	Gly Asn Phe	C15 H20 N4 O5	(M+H)+	41.55	7
338.15324	1	2618.63		C15 H20 N4 O5	(M+H)+		
339.14702	1	5363.56		C15 H20 N4 O5	(M+H)+		
340.15101	1	562.3		C15 H20 N4 O5	(M+H)+		
359.13143	1	3055			(M+Na)+		
360.13463	1	521.21			(M+Na)+		
361.12793	1	1236.44			(M+Na)+		

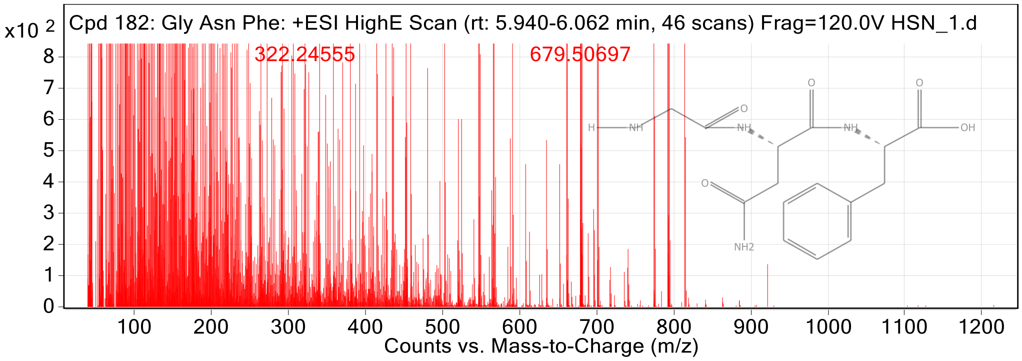

Peak List

m/z	z	Abund
43.01752		9607.51
45.03314		43671.57
59.04852		14691.78
89.05843	1	43351.24
91.0529	1	14923.64
114.08949		12066.84
133.08416	1	24137.5
679.50697	1	19580.19
680.51018	1	7865.04
792.59039	1	9456.08

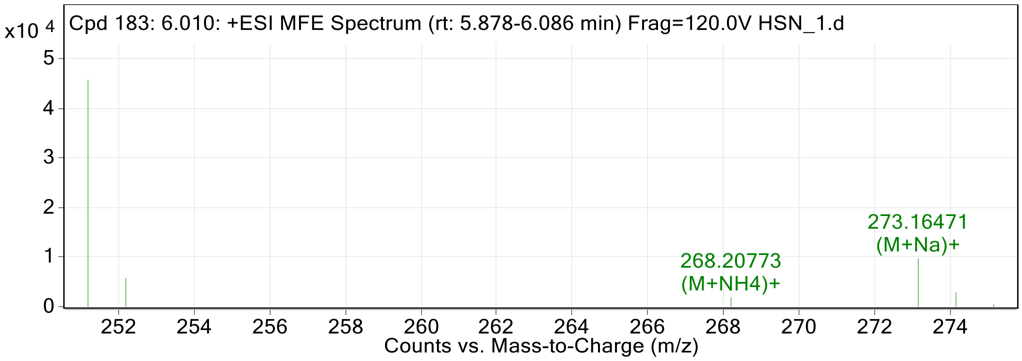

Peak List

m/z	z	Abund	Ion
251.18168	1	45783.34	(M+H)+
252.18535	1	5821.13	(M+H)+
268.20773	1	1925.42	(M+NH4)+
273.16471	1	9784.12	(M+Na)+
274.16437	1	2898.71	(M+Na)+
275.16428	1	503.15	(M+Na)+

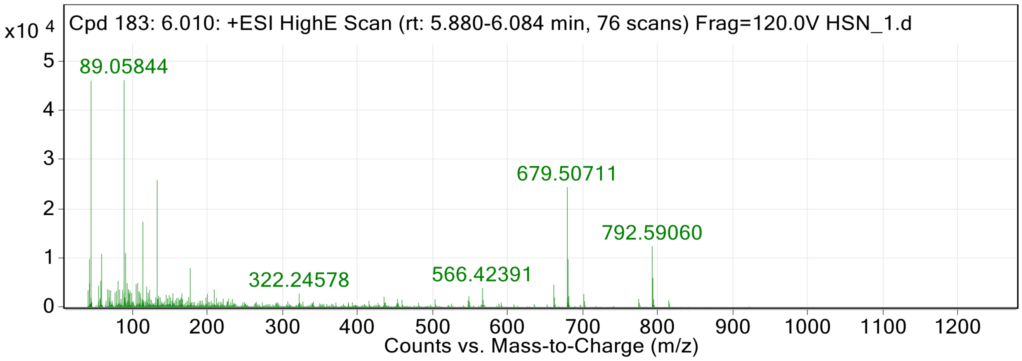

Peak List

m/z	z	Abund
43.01751		9761.87
45.03316		45980.54
59.04853		10798.77
89.05844	1	46152.85
91.0529		10933.51
114.08949		17322.2
133.08415	1	25789.83
679.50711	1	24345.85
680.51019	1	9701.03
792.5906	1	12310.13

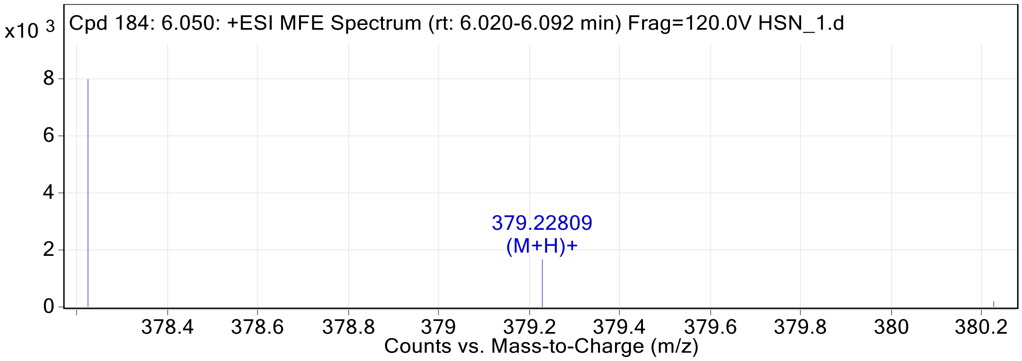

Peak List

m/z	z	Abund	Ion
378.22421	1	7987.91	(M+H)+
379.22809	1	1668.37	(M+H)+
380.22593	1	205.04	(M+H)+

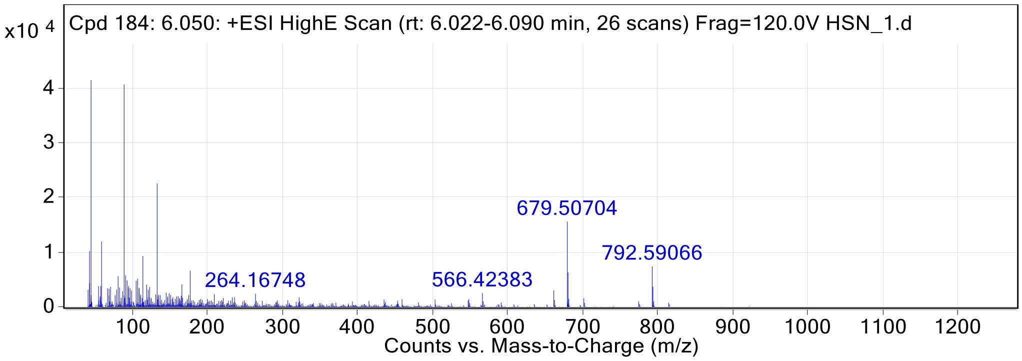

Peak List

m/z	z	Abund
43.01754		10179.89
45.03316		41462.2
59.04854		11922.14
89.05843	1	40675.34
114.0895		9288.38
133.08415	1	22542.51
177.11023		6586.01
679.50704	1	15565.54
680.51043	1	6275.86
792.59066	1	7386.29

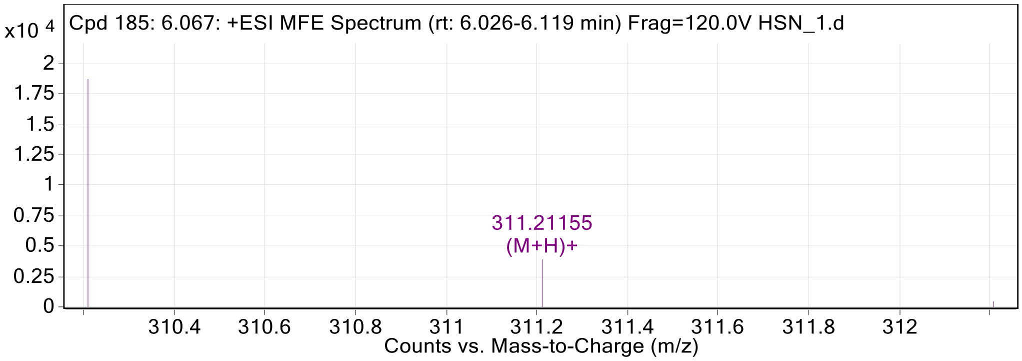

Peak List

m/z	z	Abund	Ion
310.20936	1	18717.19	(M+H)+
311.21155	1	3908	(M+H)+
312.20802	1	453.21	(M+H)+

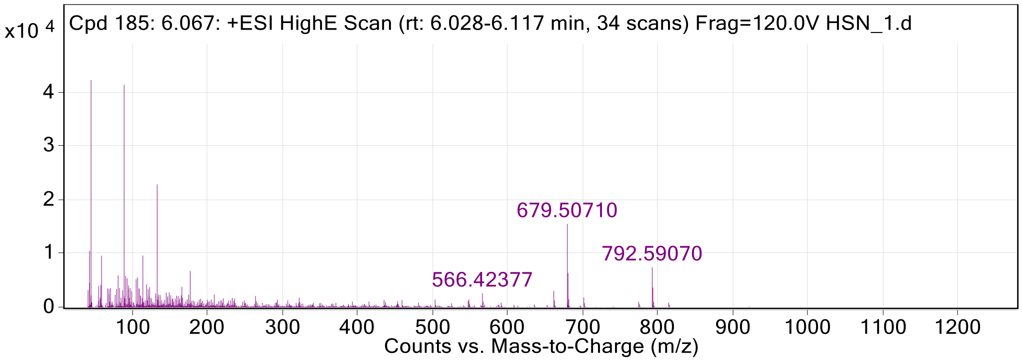

Peak List

m/z	z	Abund
43.01754		10425.23
45.03315		42243.21
59.04851		9557.34
89.05843	1	41343.46
114.08948		9587.81
133.08419	1	22805.6
177.11055		6711.68
679.5071	1	15481.24
680.51029	1	6337.13
792.5907	1	7423.08

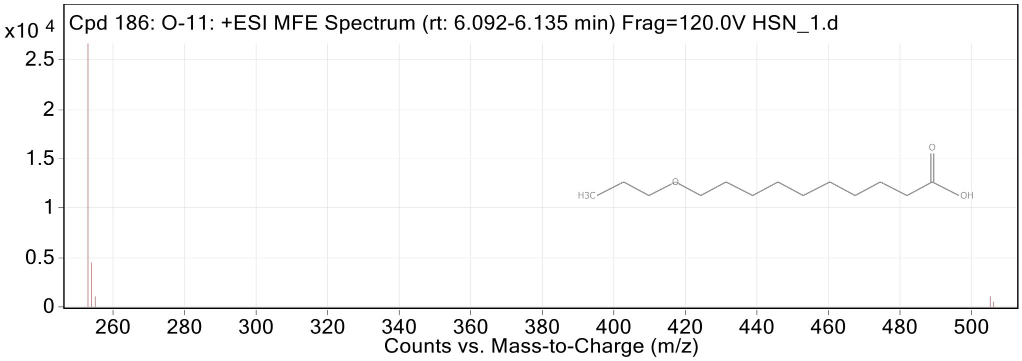

Peak List

m/z	z	Abund	Name	Formula	Ion	Score (DB)	Hits (DB)
253.17688	1	26704.98	O-11	C13 H26 O3	(M+Na)+	95.77	10
254.18025	1	4496.72		C13 H26 O3	(M+Na)+		

255.18457	1	1054.22		C13 H26 O3	(M+Na)+		
505.34687	1	1059.03			(2M+H)+		
506.34987	1	524.04			(2M+H)+		

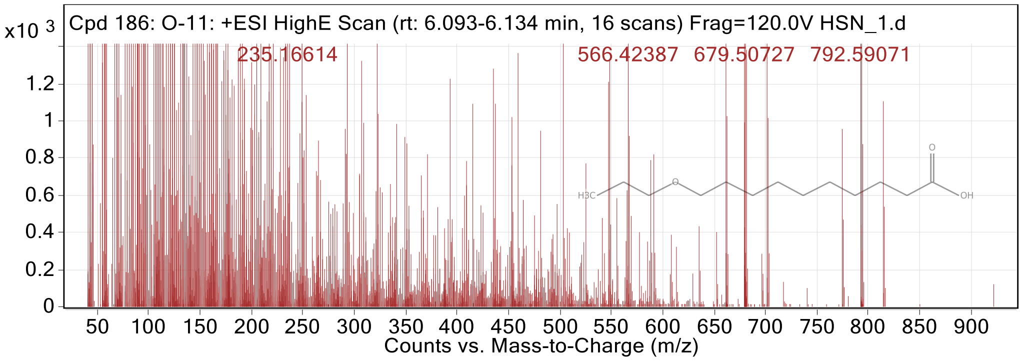

Peak List

m/z	z	Abund
43.01754		11103.24
45.03313		42639.07
81.06872		7521.04
89.05847	1	42210.02
93.06839		6733.57
114.08945		9587
133.0842	1	23118.8
177.11228		6811.8
679.50727	1	13737.29
792.59071	1	6906.34

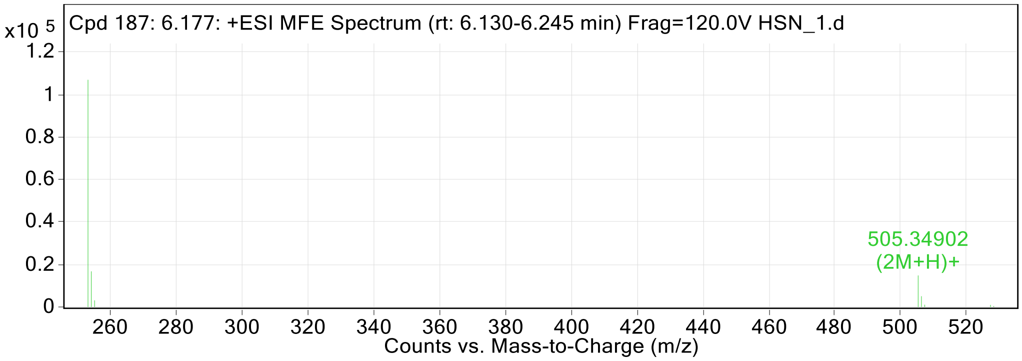

Peak List

m/z	z	Abund	Ion
253.17685	1	107040.34	(M+H)+
254.18051	1	16775.55	(M+H)+
255.18322	1	3100.94	(M+H)+
505.34902	1	14846.36	(2M+H)+
506.35234	1	5063.65	(2M+H)+
507.35541	1	1061.53	(2M+H)+
527.32823	1	981	(2M+Na)+
528.32197	1	428.45	(2M+Na)+

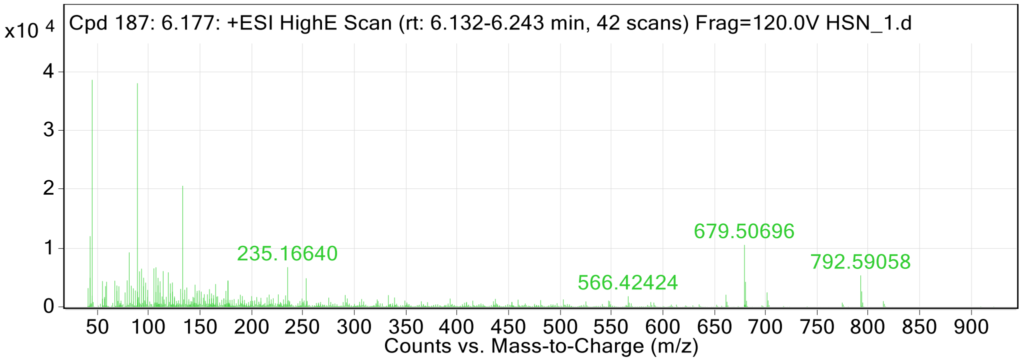

Peak List

m/z	z	Abund
43.01755		12013.3
45.03316		38576.8
81.06874		9252.5
89.05846	1	38001.05
93.06848		6490
105.06831		6539.58
107.08373		6721.28
133.08416		20579.18
235.1664		6748.5
679.50696	1	10532.78

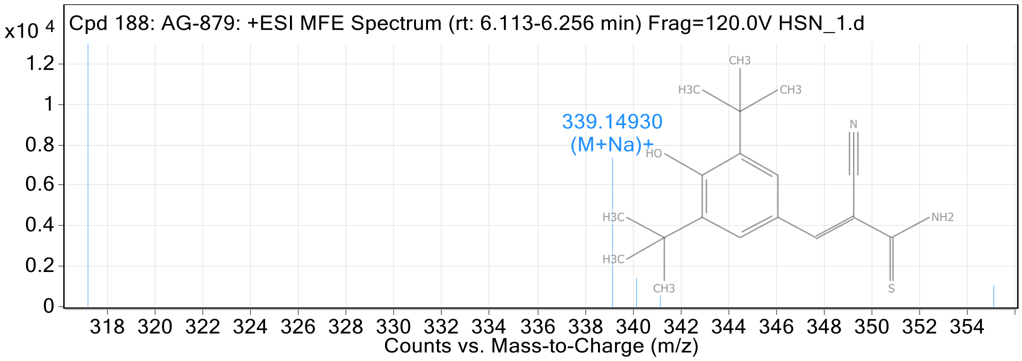

Peak List

m/z	z	Abund	Name	Formula	Ion	Score (DB)	Hits (DB)
317.1683	1	12994.78	AG-879	C18 H24 N2 O S	(M+H)+	47.59	1
339.1493	1	7346.11			(M+Na)+		
340.15651	1	1413.79			(M+Na)+		
341.15564	1	607.75			(M+Na)+		
355.12559	1	1042.53			(M+K)+		

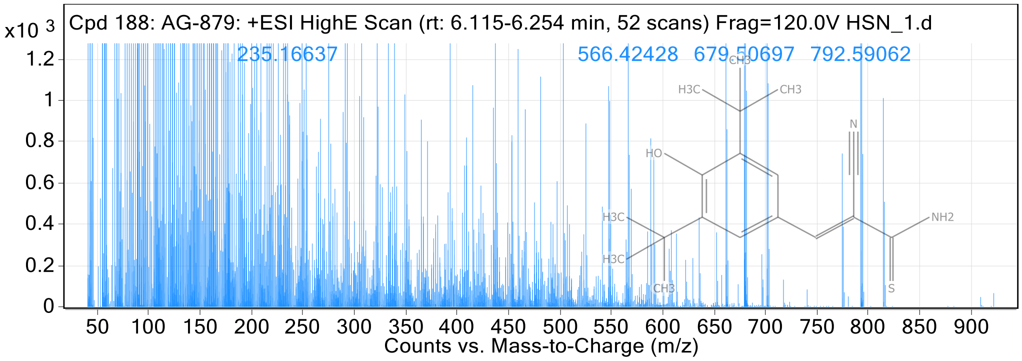

Peak List

m/z	z	Abund

43.01753		12048.17
45.03315		38982.45
81.06875		8992.72
89.05845	1	38282.55
93.06847		6543.64
105.06828		6626.55
107.08371		6689.37
114.08948		6393.76
133.08415		20817.4
679.50697	1	10805.61

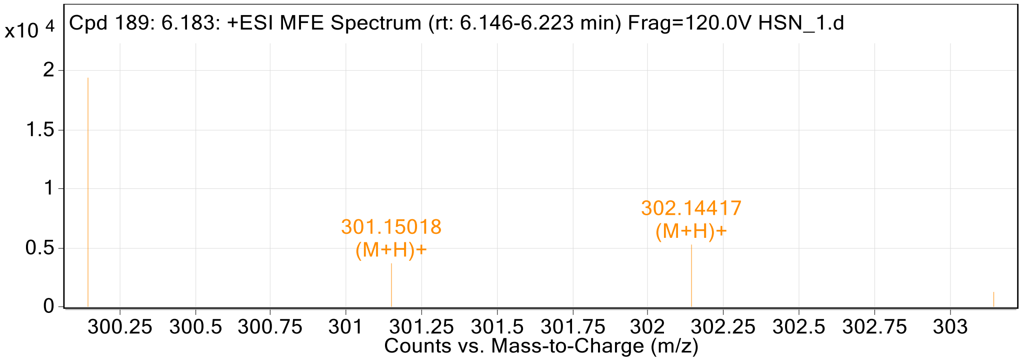

Peak List

m/z	z	Abund	Ion
300.14478	1	19422.13	(M+H)+
301.15018	1	3682.9	(M+H)+
302.14417	1	5271	(M+H)+
303.14552	1	1256.05	(M+H)+

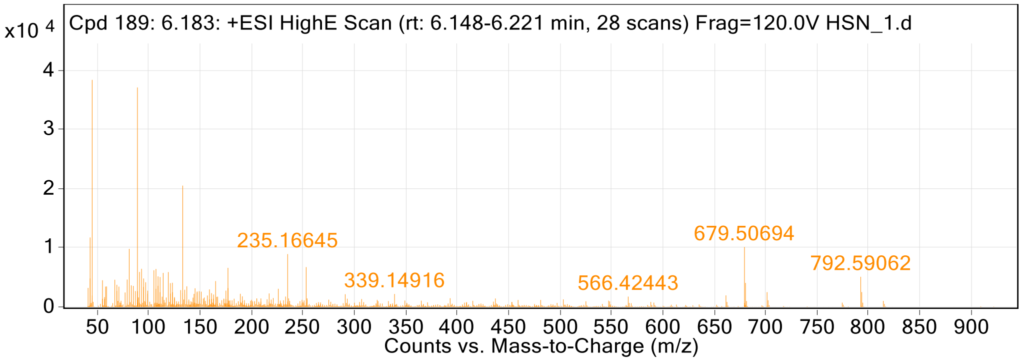

Peak List

m/z	z	Abund
43.01756		11749.34
45.03317		38420.83
81.0688		9823.67
89.05846	1	37137.72
93.06849		6448.81
133.08415		20525.65
177.12233		6608.59
235.16645	1	8934.45
253.17706		6735.6
679.50694	1	10133.17

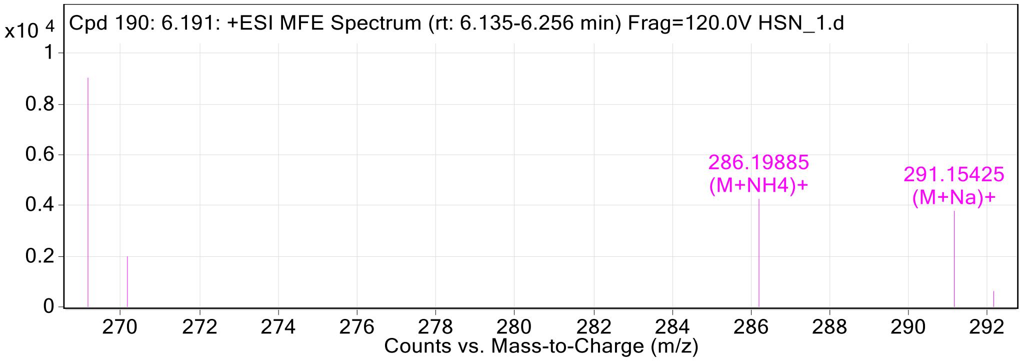

Peak List

m/z	z	Abund	Ion
269.17195	1	9037.81	(M+H)+
270.17193	1	1993	(M+H)+
286.19885	1	4263.75	(M+NH4)+
291.15425	1	3788.08	(M+Na)+
292.15691	1	623.85	(M+Na)+

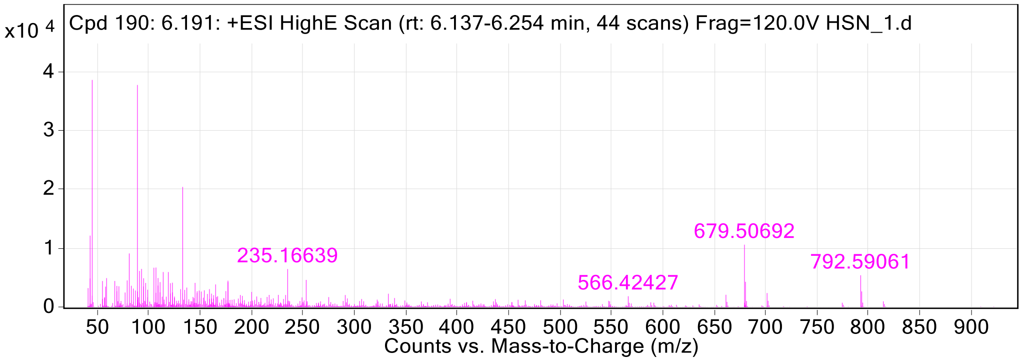

Peak List

m/z	z	Abund
43.01754		12119.2
45.03316		38568.34
81.06875		9091.29
89.05845	1	37713.85
93.0685		6464.38
105.06833		6666.29
107.08369		6696.38
133.08416		20387.91
235.16639		6439.58
679.50692	1	10577.79

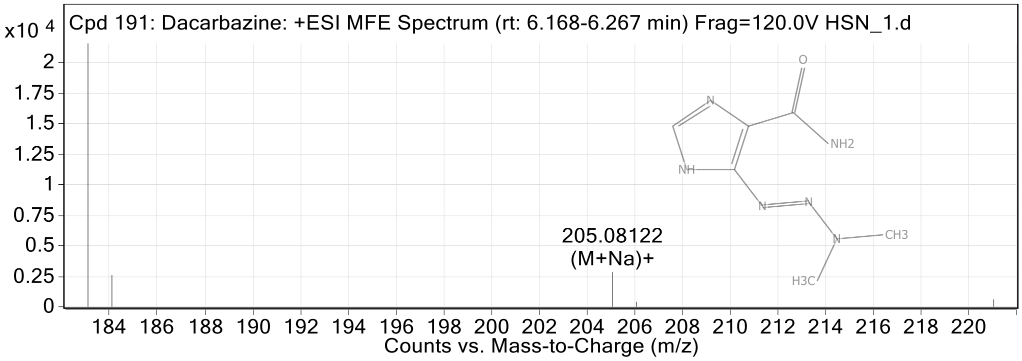

Peak List

m/z	z	Abund	Name	Formula	Ion	Score (DB)	Hits (DB)

183.09887	1	21604.78	Dacarbazine	C6 H10 N6 O	(M+H)+	78.71	1
184.10269	1	2614.03		C6 H10 N6 O	(M+H)+		
205.08122	1	2845.52			(M+Na)+		
206.08467	1	413.16			(M+Na)+		
221.05426	1	612			(M+K)+		

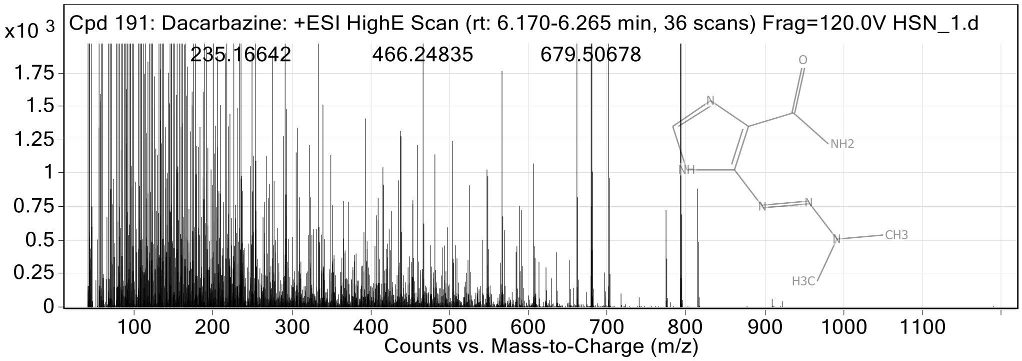

Peak List

m/z	z	Abund
43.01751		12572.92
45.03315		38007.53
59.04849		6413.35
81.06877		8498.06
89.05844	1	36773.18
93.0685		6455.11
105.06833		6908.7
107.08371		6614.4
133.08408		19828.44
679.50678	1	10299.77

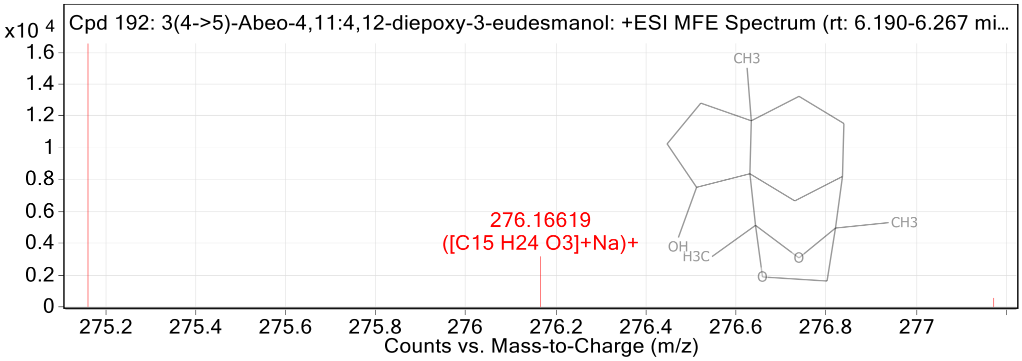

Peak List

m/z	z	Abund	Name	Formula	Ion	Score (DB)	Hits (DB)
275.16141	1	16567.18	3(4->5)-Abeo-4,11:4,12-diepoxy-3-eudesmanol	C15 H24 O3	(M+Na)+	94.1	10
276.16619	1	3168.84		C15 H24 O3	(M+Na)+		
277.1718	1	563.91		C15 H24 O3	(M+Na)+		

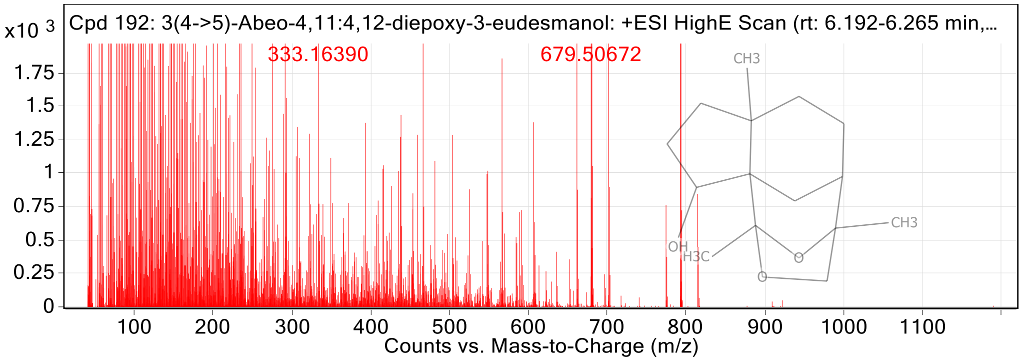

Peak List

m/z	z	Abund
43.01749		13088.09
45.03314		38128.61
59.0485		7424.3
81.06871		7552.34
89.05843	1	37194.62
105.06835		6914.24
107.08371		6618.84
133.08409		20033.58
177.11363		6667.45
679.50672	1	10806.05

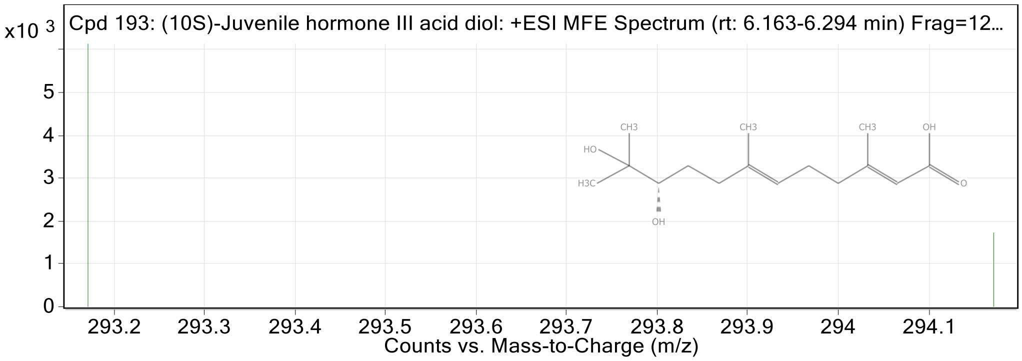

Peak List

m/z	z	Abund	Name	Formula	Ion	Score (DB)	Hits (DB)
293.17136	1	6134.6	(10S)-Juvenile hormone III acid diol	C15 H26 O4	(M+Na)+	51.77	2
294.17177	1	1730.84		C15 H26 O4	(M+Na)+		

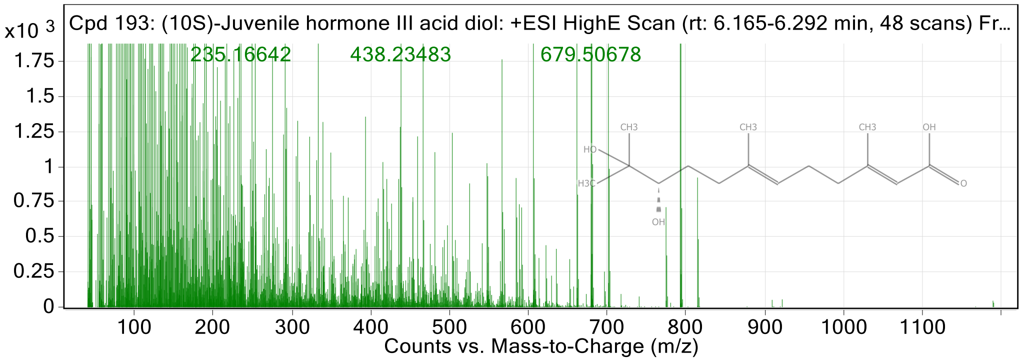

Peak List

m/z	z	Abund
43.01748		12134.09
45.03315		37957.97

59.0485		10007.37
81.06879		8351.26
89.05844	1	36734.55
93.0685		6431.87
105.06829		6617.78
107.08369		6481.81
133.08416		19887.8
679.50678	1	10157.72

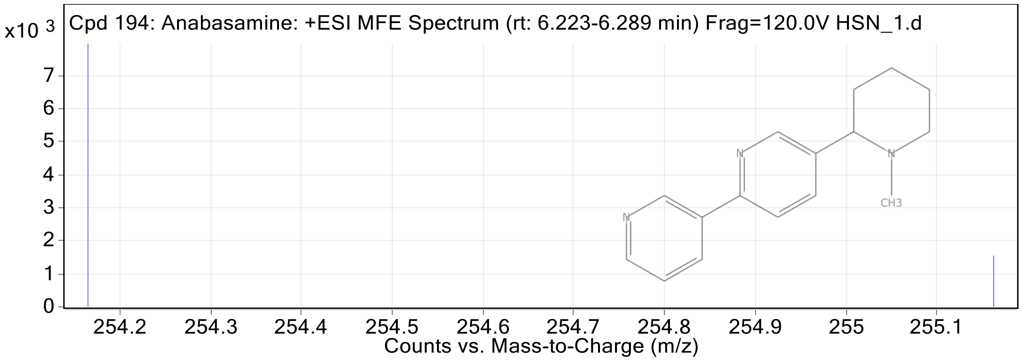

Peak List

m/z	z	Abund	Name	Formula	Ion	Score (DB)	Hits (DB)
254.16455	1	7973.37	Anabasamine	C16 H19 N3	(M+H)+	70.22	2
255.16283	1	1551.2		C16 H19 N3	(M+H)+		

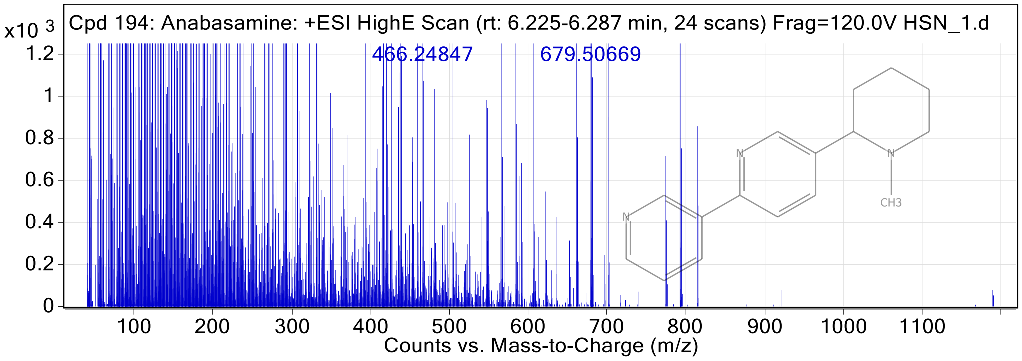

Peak List

m/z	z	Abund
43.01744		12429.84
45.03314		37860.02
59.04849		14401.01
81.06876		7151.16
89.05841	1	37156.02
93.06852		6476.76
105.06829		7140.93
107.08362		6690.93
133.08412		19289.2
679.50669	1	10563.17

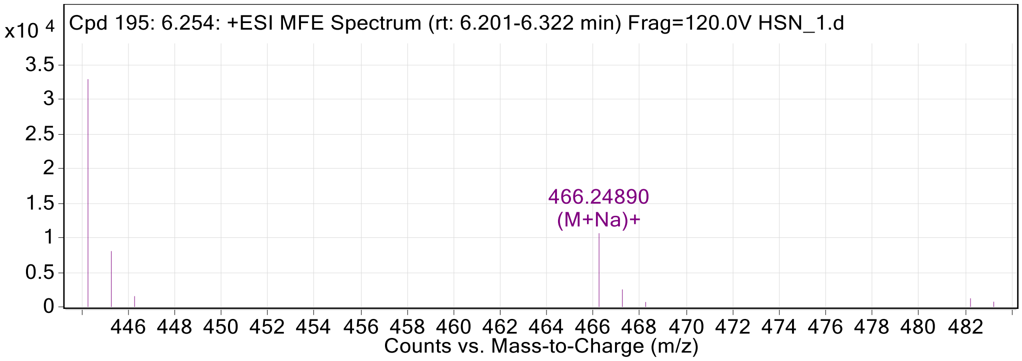

Peak List

m/z	z	Abund	Ion
444.26676	1	32914.77	(M+H)+
445.27004	1	8064.06	(M+H)+

m/z	z	Abund	Ion
446.27082	1	1550.05	(M+H)+
466.2489	1	10648.5	(M+Na)+
467.25159	1	2519.84	(M+Na)+
468.25525	1	703.02	(M+Na)+
482.22596	1	1237.79	(M+K)+
483.22662	1	746.98	(M+K)+

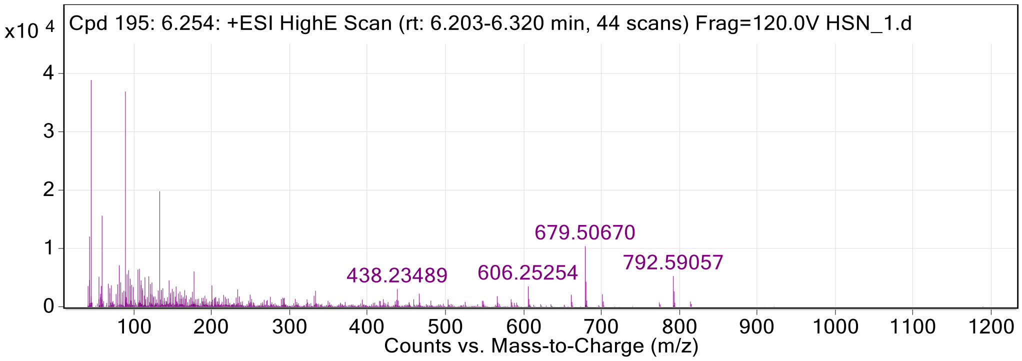

Peak List

m/z	z	Abund
43.01748		12043.49
45.03314		38873.58
59.04847		15620.69
81.06873		7113.22
89.05842	1	36903.02
93.0685		6270.23
105.06829		6379.6
107.08373		6503.8
133.08411		19800.14
679.5067	1	10389.61

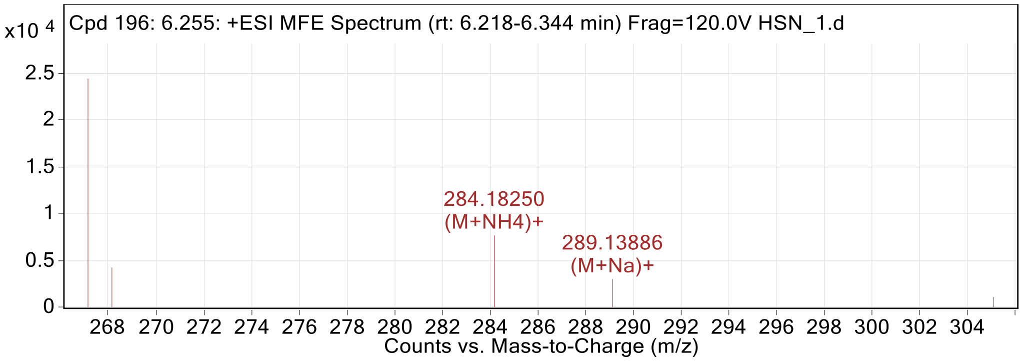

Peak List

m/z	z	Abund	Ion
267.15625	1	24445.1	(M+H)+
268.15934	1	4227.63	(M+H)+
284.1825	1	7666.2	(M+NH4)+
289.13886	1	2988.47	(M+Na)+
305.1112	1	1071.54	(M+K)+

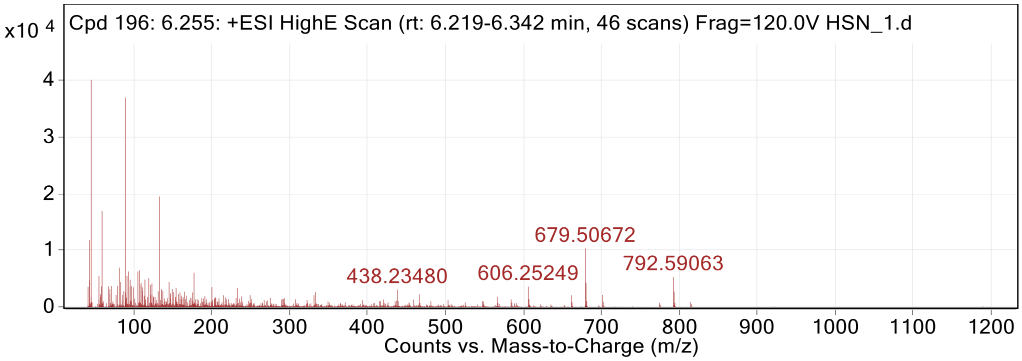

Peak List

m/z	z	Abund
43.01748		11779.38
45.03314		40154.88
59.04849		16983.35
81.06873		6930.27
89.05841	1	37033.24
93.06852		6253.21
105.06827		6254.85
107.08373		6458.36
133.0841		19518.73
679.50672	1	10317.61

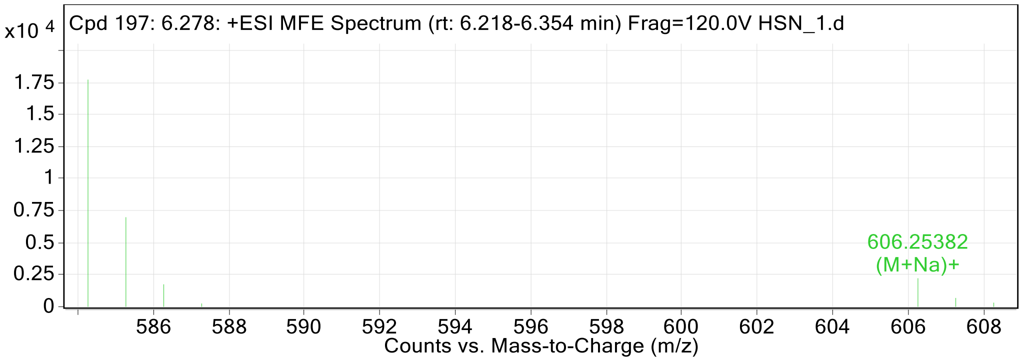

Peak List

m/z	z	Abund	Ion
584.27125	1	17716.15	(M+H)+
585.27572	1	6985.47	(M+H)+
586.27807	1	1751.88	(M+H)+
587.28348	1	257.36	(M+H)+
606.25382	1	2224.3	(M+Na)+
607.25574	1	687.96	(M+Na)+
608.26238	1	320.13	(M+Na)+

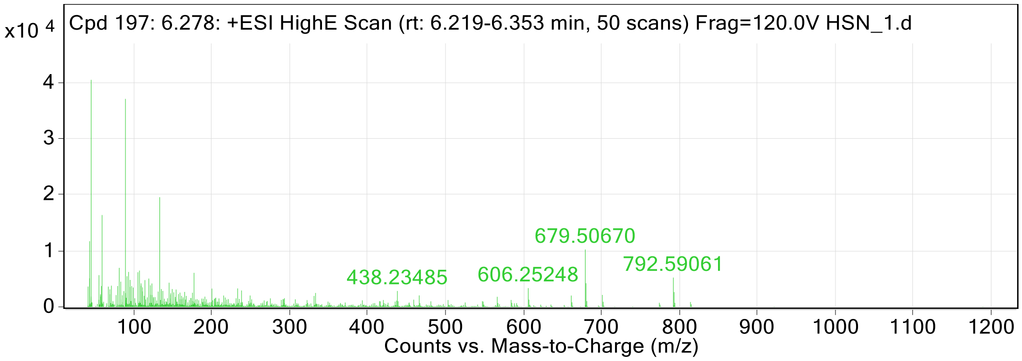

Peak List

m/z	z	Abund
43.01749		11728.49
45.03314		40519.91
59.0485		16390.64
81.06872		6964.36
89.0584	1	37123.93
93.0685		6233.08
105.06829		6186.56
107.08373		6477.66
133.08409		19579.95
679.5067	1	10237.04

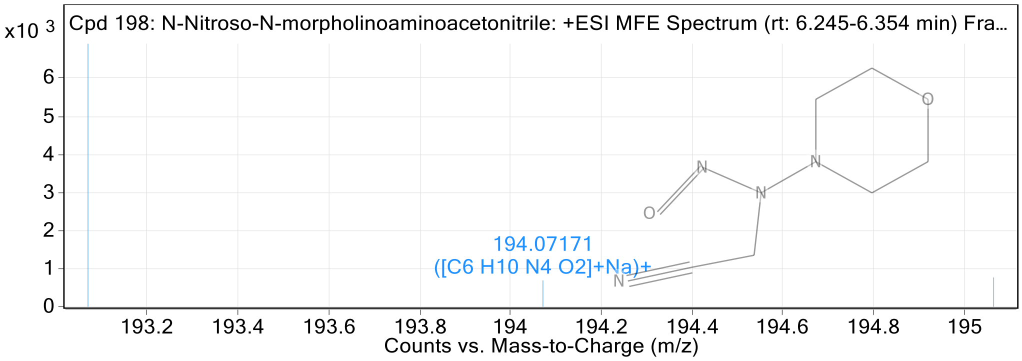

Peak List

m/z	z	Abund	Name	Formula	Ion	Score (DB)	Hits (DB)
193.06913	1	6905.98	N-Nitroso-N-morpholinoaminoacetonitrile	C6 H10 N4 O2	(M+Na)+	62.16	3
194.07171	1	692.91		C6 H10 N4 O2	(M+Na)+		
195.06513	1	769.59		C6 H10 N4 O2	(M+Na)+		

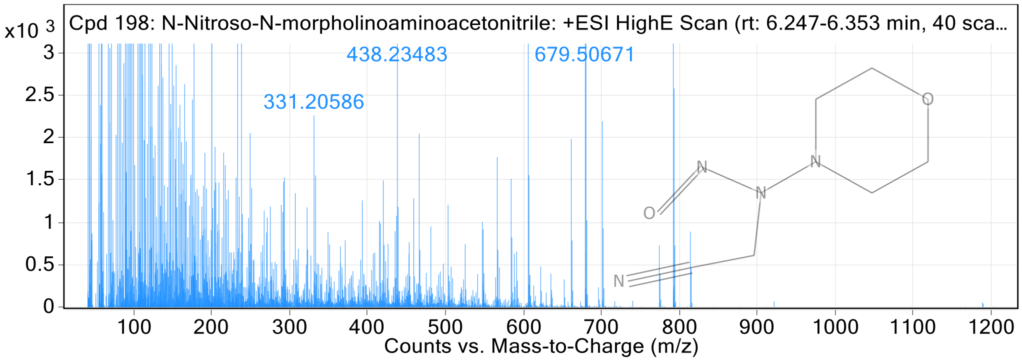

Peak List

m/z	z	Abund
43.01748		11262.83

45.03314		41071.2
59.04849		18838.53
81.06873		6940.93
89.0584	1	36824.07
93.06848		6163.34
107.08374		6252.69
133.08405		19461.66
177.11034		6030.1
679.50671	1	9980.96

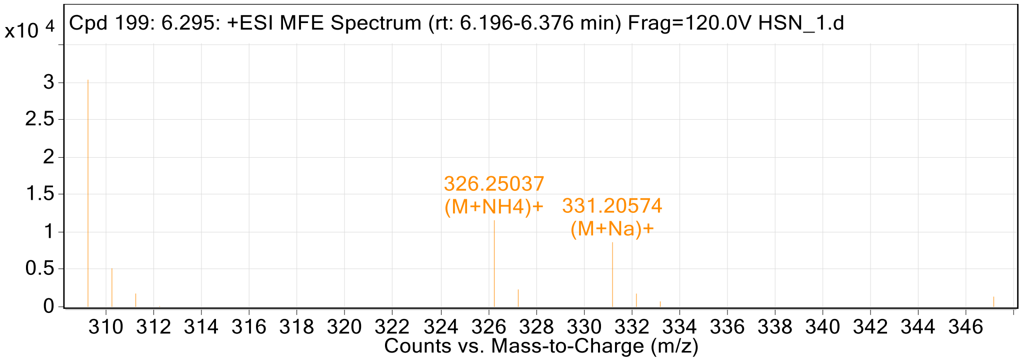

Peak List

m/z	z	Abund	Ion
309.22386	1	30324.37	(M+H)+
310.22763	1	5146.19	(M+H)+
311.22625	1	1773.47	(M+H)+
312.22798	1	89.6	(M+H)+
326.25037	1	11562.98	(M+NH4)+
327.25329	1	2320.37	(M+NH4)+
331.20574	1	8628.01	(M+Na)+
332.2065	1	1764.08	(M+Na)+
333.206	1	724.37	(M+Na)+
347.18218	1	1360.05	(M+K)+

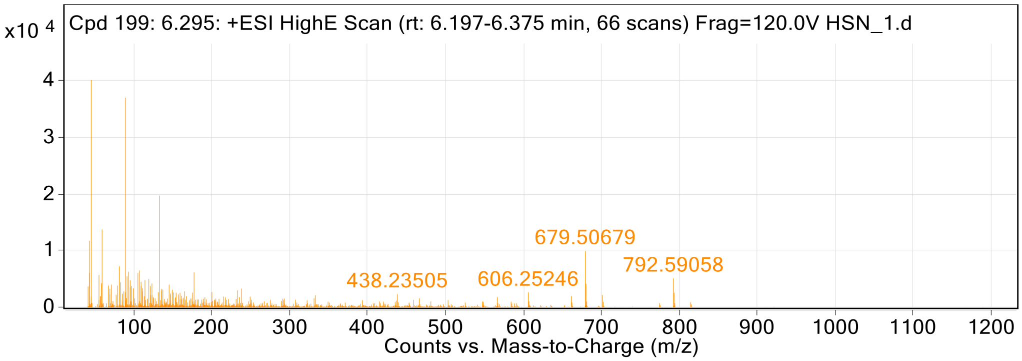

Peak List

m/z	z	Abund
43.01749		11710.99
45.03314		40098.53
59.0485		13712.24
81.06872		7217.58
89.05841	1	36986.23
93.06849		6247.69
107.08377		6465.82
133.08412		19694.22
177.11095		6139.63
679.50679	1	9922.95

Qualitative Analysis Report

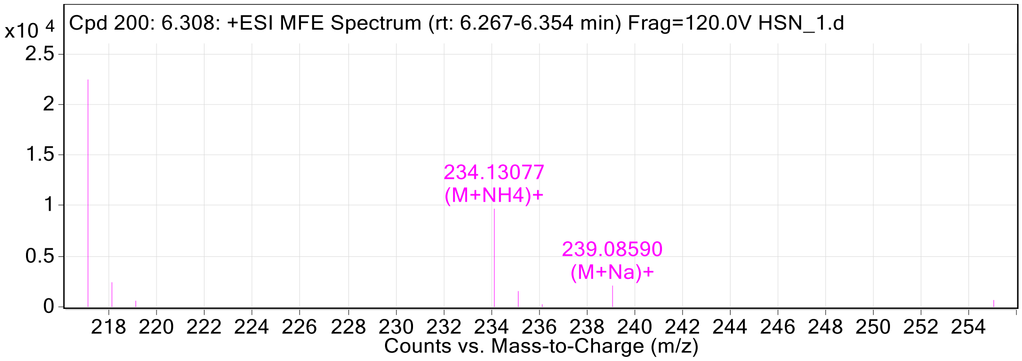

Peak List

m/z	z	Abund	Ion
217.1044	1	22470.27	(M+H)+
218.10775	1	2434.53	(M+H)+
219.10783	1	607.6	(M+H)+
234.13077	1	9703.29	(M+NH4)+
235.13308	1	1560.12	(M+NH4)+
236.13835	1	256.54	(M+NH4)+
239.0859	1	2102.93	(M+Na)+
255.06054	1	675.65	(M+K)+

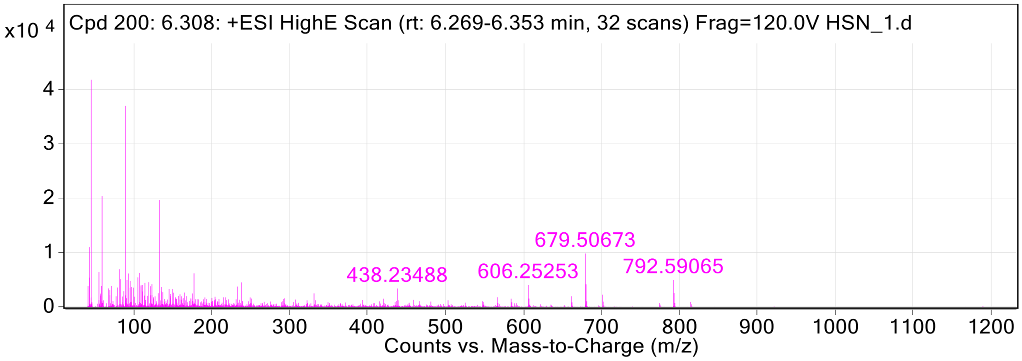

Peak List

m/z	z	Abund
43.01749		10967
45.03315		41726.41
55.05357		6397.04
59.0485		20347.23
81.06873		6892.44
89.05841	1	36913.04
107.08378		6241.16
133.0841		19652.75
177.11017		6134.75
679.50673	1	9784.78

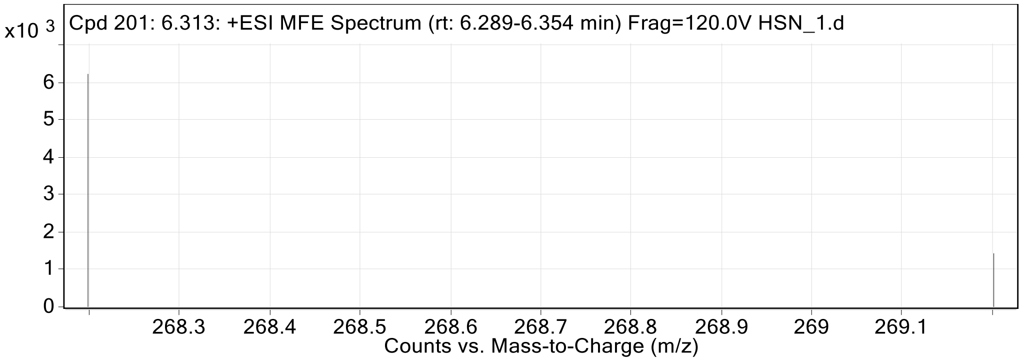

Peak List

m/z	z	Abund	Ion
268.19866	1	6221.57	(M+H)+
269.20168	1	1430.37	(M+H)+

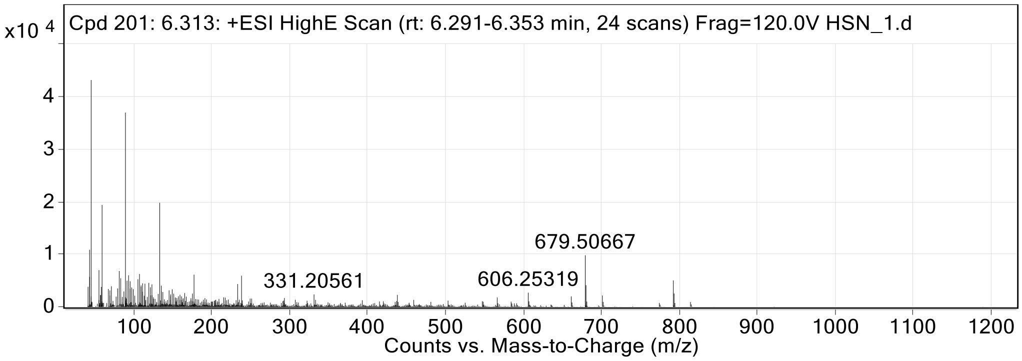

Peak List

m/z	z	Abund
43.01753		10858.68
45.03314		43184.88
55.05356		6973.14
59.04851		19377.66
81.06869		6794.75
89.0584	1	36976.8
107.08385		6234.81
133.08405		19803.22
177.11019		6106.57
679.50667	1	9799.44

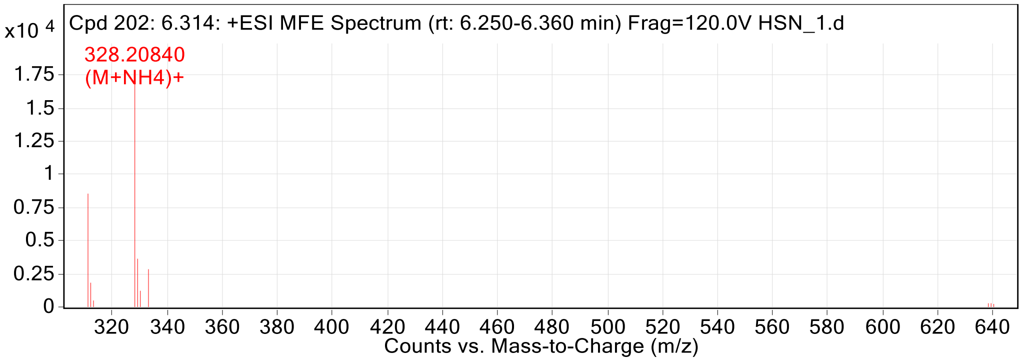

Peak List

m/z	z	Abund	Ion
311.18213	1	8555.59	(M+H)+
312.18484	1	1831.12	(M+H)+
313.18572	1	488.08	(M+H)+

328.2084	1	17170.03	(M+NH4)+
329.21139	1	3642.43	(M+NH4)+
330.21741	1	1222.55	(M+NH4)+
333.16393	1	2850.17	(M+Na)+
638.37824	1	277.23	(2M+NH4)+
639.38729	1	281.63	(2M+NH4)+
640.366	1	225.05	(2M+NH4)+

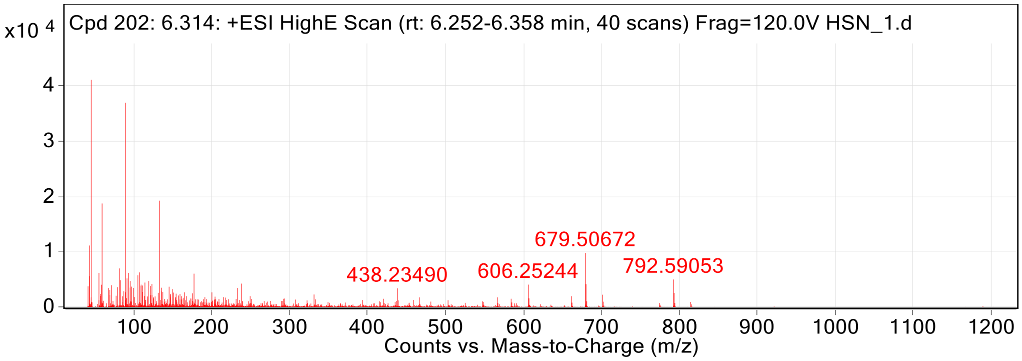

Peak List

m/z	z	Abund
43.01748		11099.99
45.03314		41056.14
55.0536		6154.34
59.04849		18700.07
81.06875		6955.78
89.05841	1	36922.05
93.06847		6161.19
107.08375		6234.52
133.08405		19215.88
679.50672	1	9762.86

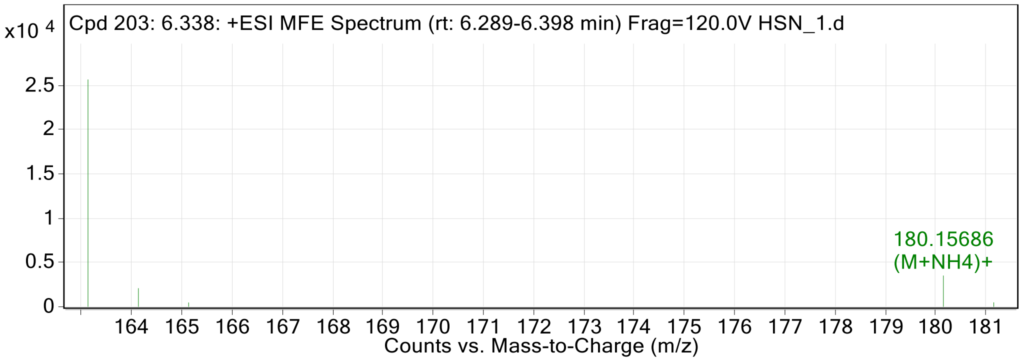

Peak List

m/z	z	Abund	Ion
163.13059	1	25635.24	(M+H)+
164.13408	1	2096.98	(M+H)+
165.13411	1	485.85	(M+H)+
180.15686	1	3524.55	(M+NH4)+
181.15977	1	500.09	(M+NH4)+

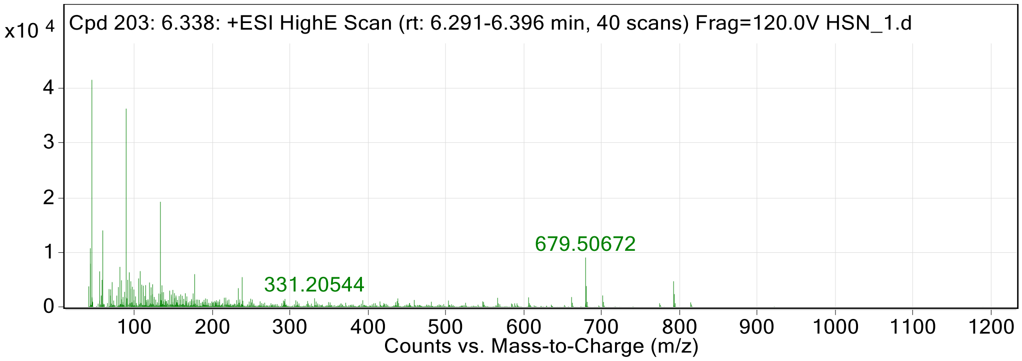

Peak List

m/z	z	Abund
43.01749		10736.88
43.05391		7901.43
45.03313		41568.13
55.05362		6502.78
59.0485		13992.04
81.06869		7318.31
89.0584	1	36299.88
107.08384		6547.78
133.08408		19238.96
679.50672	1	9031.54

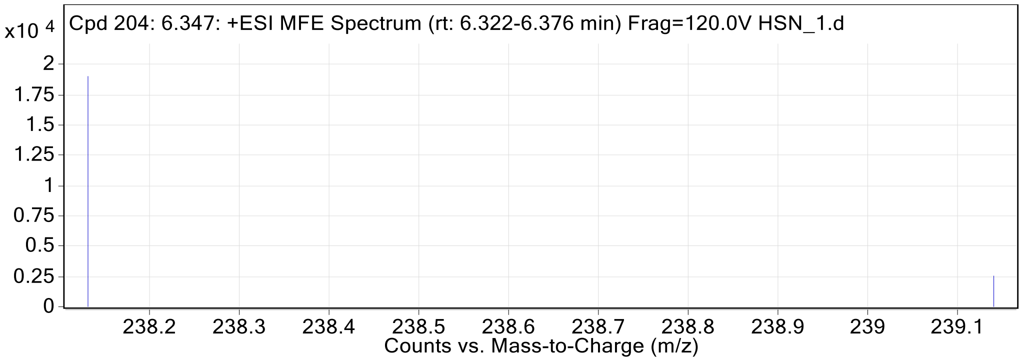

Peak List

m/z	z	Abund	Ion
238.13121	1	19047.9	(M+H)+
239.13982	1	2563.08	(M+H)+

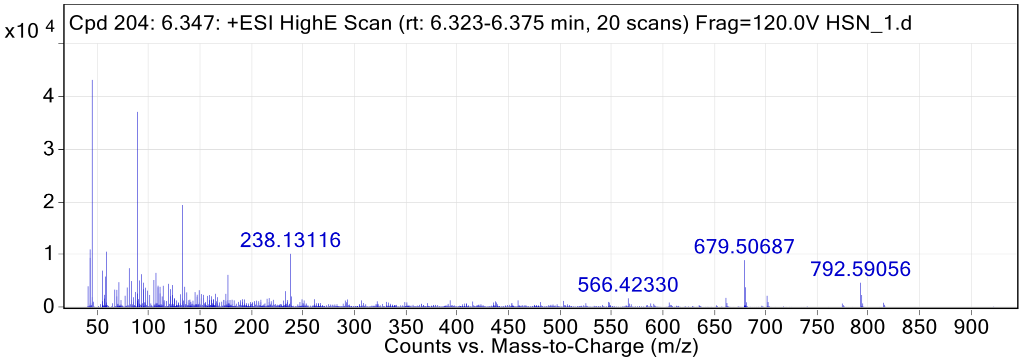

Peak List

m/z	z	Abund
43.0175		10924.29
43.05392		9342.46
45.03315		43148.81

55.05362		6907.5
59.04857		10512.97
81.06871		7359.39
89.05839	1	37087.95
133.08414		19418.18
238.13116	1	10112.38
679.50687	1	8891.41

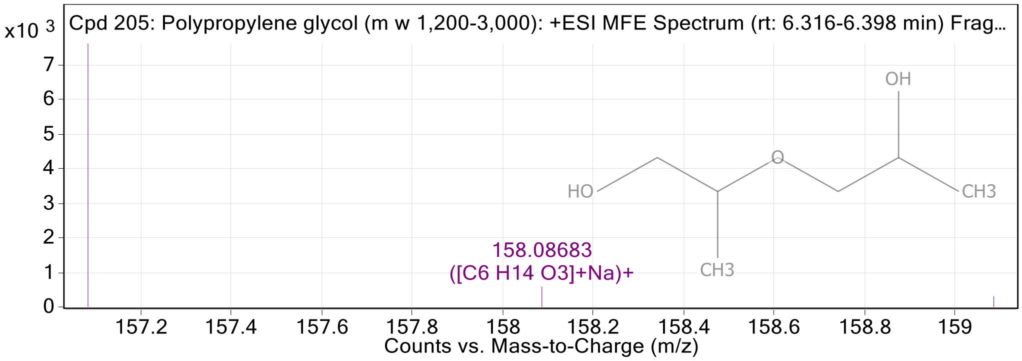

Peak List

m/z	z	Abund	Name	Formula	Ion	Score (DB)	Hits (DB)
157.08396	1	7615.64	Polypropylene glycol (m w 1,200-3,000)	C6 H14 O3	(M+Na)+	92.93	1
158.08683	1	590.84		C6 H14 O3	(M+Na)+		
159.08521	1	310.28		C6 H14 O3	(M+Na)+		

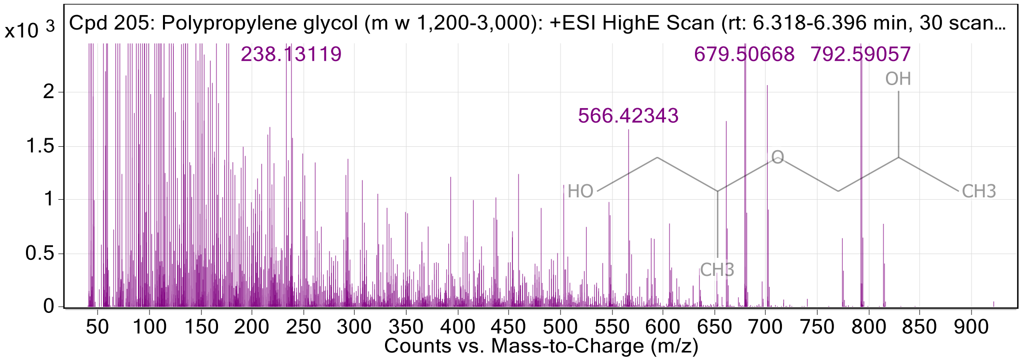

Peak List

m/z	z	Abund
43.01746		10907.99
43.0539		9096.9
45.03313		41848.68
59.04854		9733.89
81.06867		7468.1
89.05839	1	36266.87
107.08382		6658.01
133.0841		19057.19
238.13119	1	7237.75
679.50668	1	8846.25

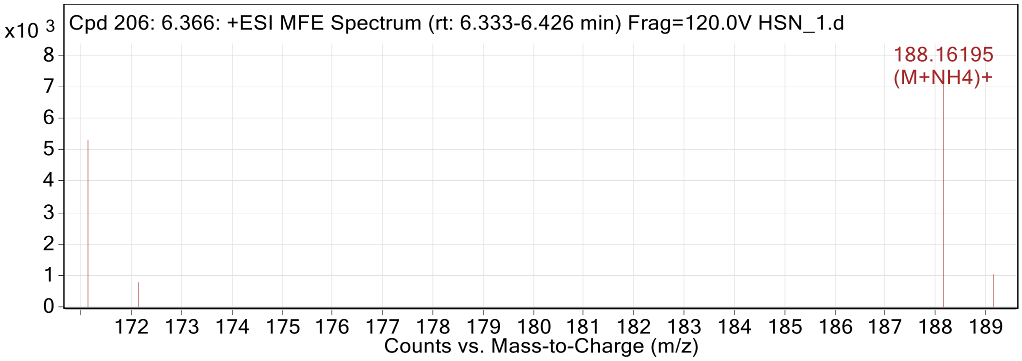

Peak List

m/z	z	Abund	Ion
171.1356	1	5322.25	(M+H)+
172.13779	1	781.01	(M+H)+
188.16195	1	7331.23	(M+NH4)+
189.16495	1	1035.53	(M+NH4)+

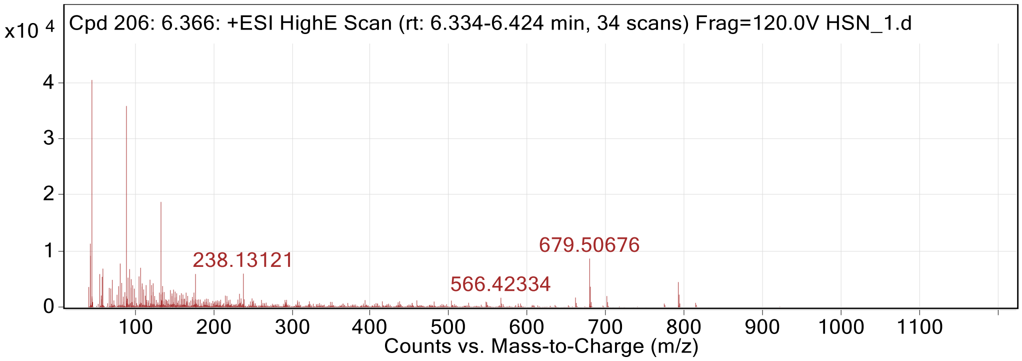

Peak List

m/z	z	Abund
43.01747		11292.76
43.05389		9117.54
45.03313		40469.4
59.04856		6843.57
81.06871		7746.18
89.05841	1	35834.05
93.06847		6760.16
107.08383		6995.9
133.08412		18733.03
679.50676	1	8637.23

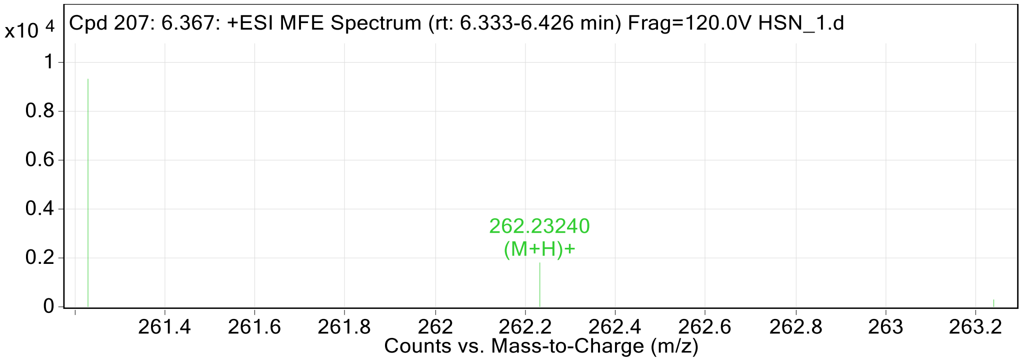

Peak List

m/z	z	Abund	Ion
261.22947	1	9304.04	(M+H)+

262.2324	1	1808.3	(M+H)+
263.23986	1	299.5	(M+H)+

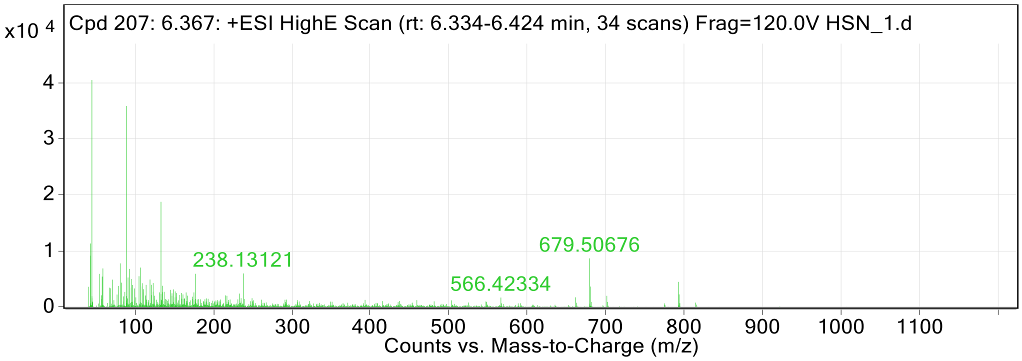

Peak List

m/z	z	Abund
43.01747		11292.76
43.05389		9117.54
45.03313		40469.4
59.04856		6843.57
81.06871		7746.18
89.05841	1	35834.05
93.06847		6760.16
107.08383		6995.9
133.08412		18733.03
679.50676	1	8637.23

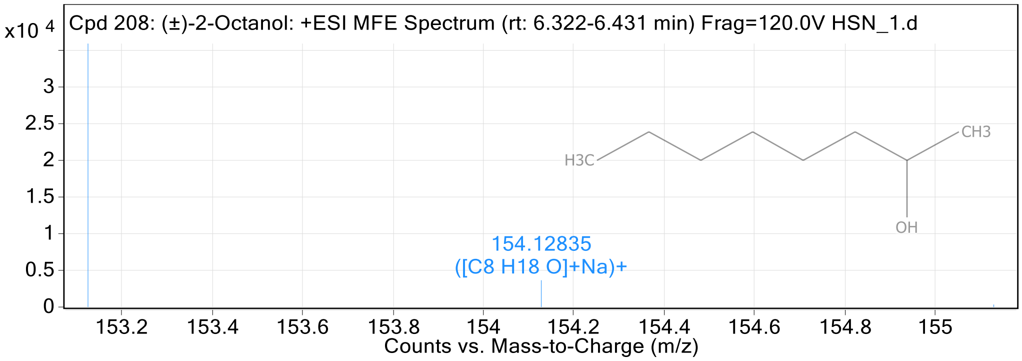

Peak List

m/z	z	Abund	Name	Formula	Ion	Score (DB)	Hits (DB)
153.12501	1	36019.67	(±)-2-Octanol	C8 H18 O	(M+Na)+	99.39	10
154.12835	1	3660.96		C8 H18 O	(M+Na)+		
155.13	1	339.78		C8 H18 O	(M+Na)+		

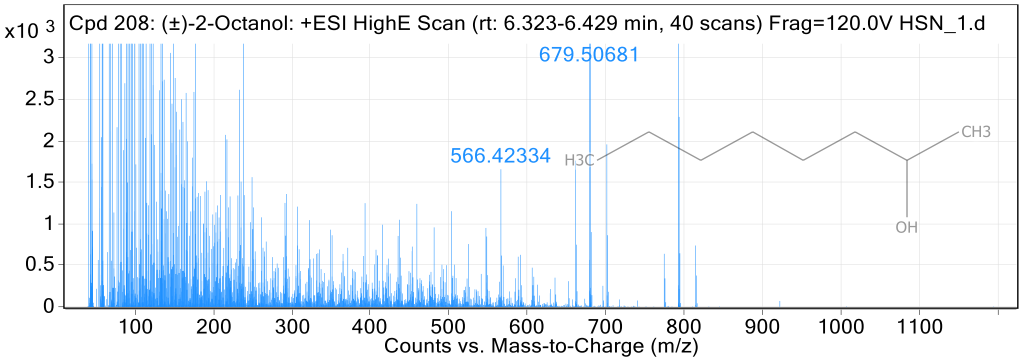

Peak List

m/z	z	Abund

43.01747		11330.6
43.0539		8584.73
45.03312		40769.63
59.04854		7716.91
81.06869		7627.34
89.0584	1	36075.7
93.06847		6743.27
107.08382		6935.46
133.08412		18796.31
679.50681	1	8906.66

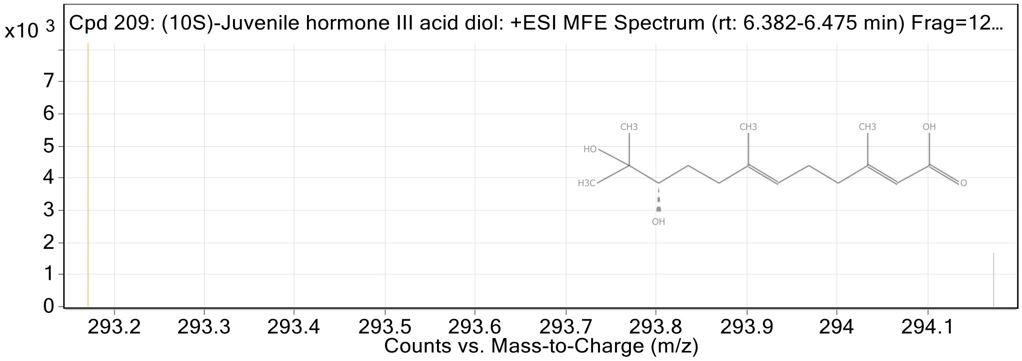

Peak List

m/z	z	Abund	Name	Formula	Ion	Score (DB)	Hits (DB)
293.17148	1	8190.79	(10S)-Juvenile hormone III acid diol	C15 H26 O4	(M+Na)+	74.58	2
294.17323	1	1675.57		C15 H26 O4	(M+Na)+		

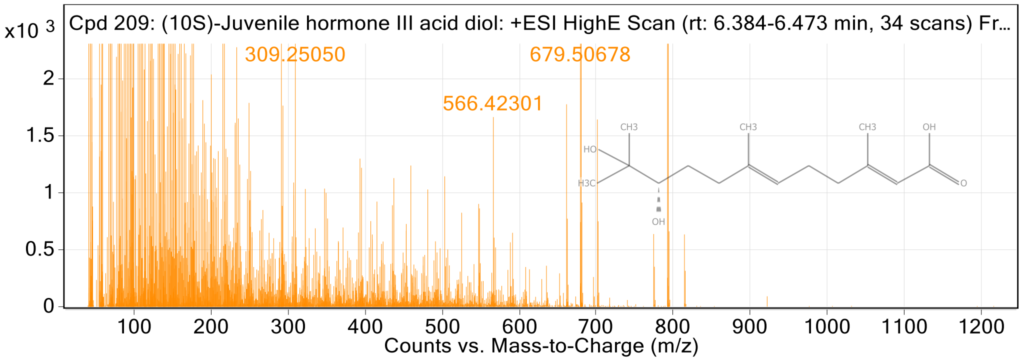

Peak List

m/z	z	Abund
43.01749		11634.24
45.03312		38666.73
58.06454		7278.16
81.06878		7945.45
89.0584		34971.11
93.06847		7469.75
107.08379		7483.76
114.0896		7686.99
133.08412		17677.29
679.50678	1	9148.87

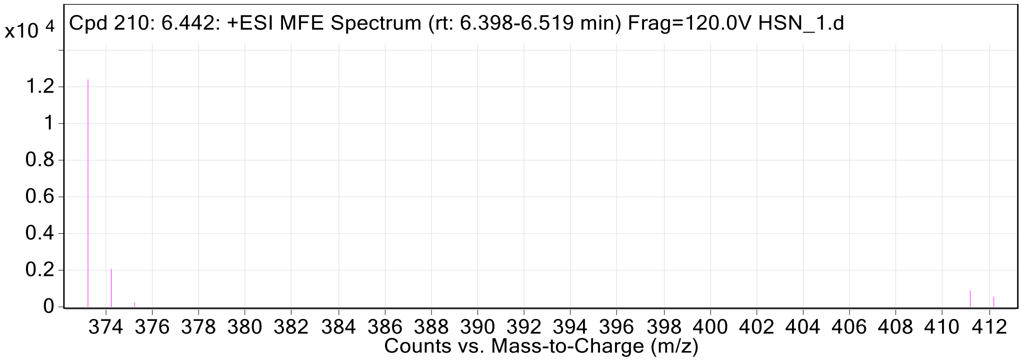

Peak List

m/z	z	Abund	Ion
373.23004	1	12447.67	(M+H)+
374.23225	1	2071.7	(M+H)+
375.23159	1	233.69	(M+H)+
411.18631	1	895.27	(M+K)+
412.19703	1	540.55	(M+K)+

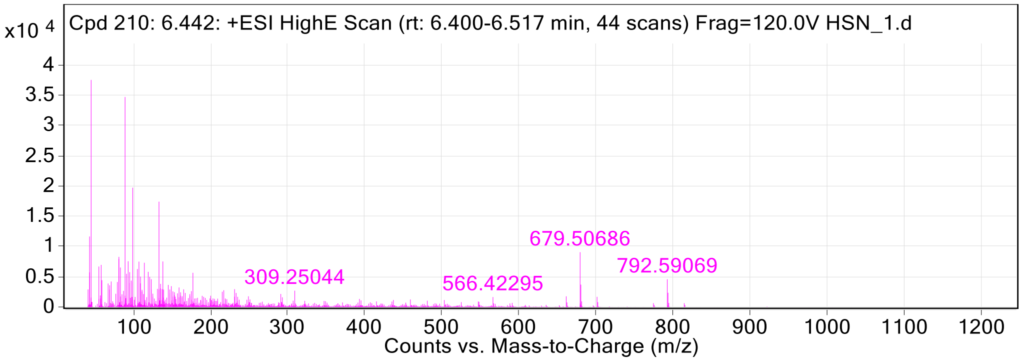

Peak List

m/z	z	Abund
43.01751		11647.82
45.03314		37536.06
80.97246		7935.19
81.06889		8285.07
89.0584		34717.11
93.06851		7557.74
98.98272		19728.24
133.08413		17412.68
138.12564		7520.89
679.50686	1	9053.7

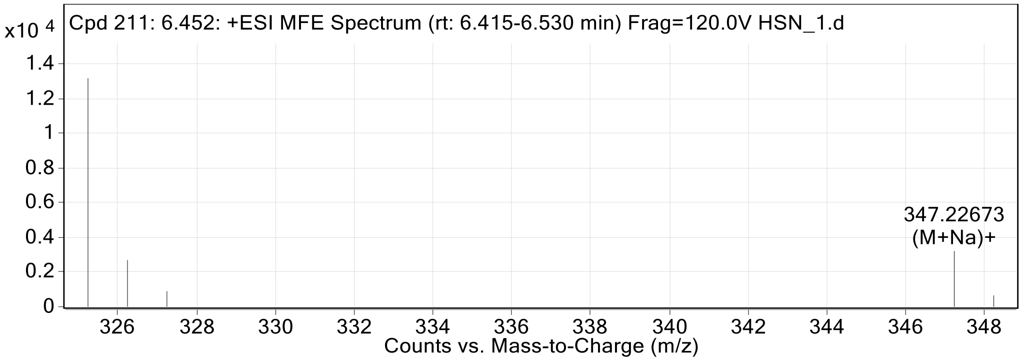

Peak List

m/z	z	Abund	Ion

325.24584	1	13194.12	(M+H)+
326.24793	1	2696.76	(M+H)+
327.24877	1	902.94	(M+H)+
347.22673	1	3224.3	(M+Na)+
348.23033	1	655.7	(M+Na)+

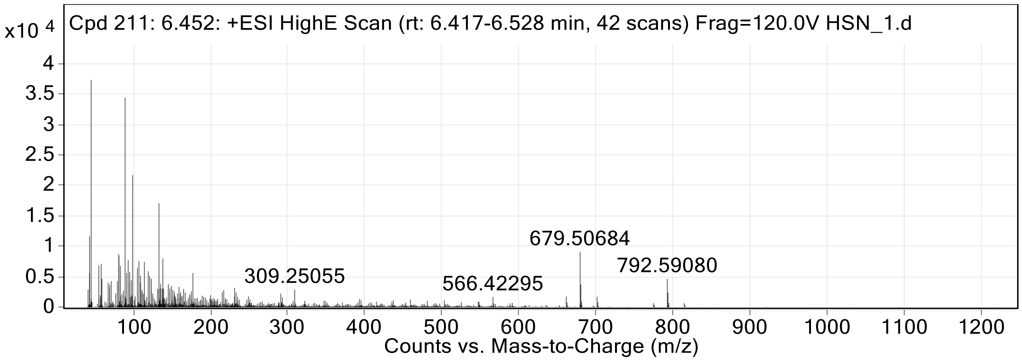

Peak List

m/z	z	Abund
43.01753		11612.81
45.03314		37296.52
80.97247		8693.45
81.0689		8417.36
89.05841		34405.82
93.06853		7681.14
98.98272		21661.27
133.08412		17040.82
138.12564		7988.5
679.50684	1	9047.22

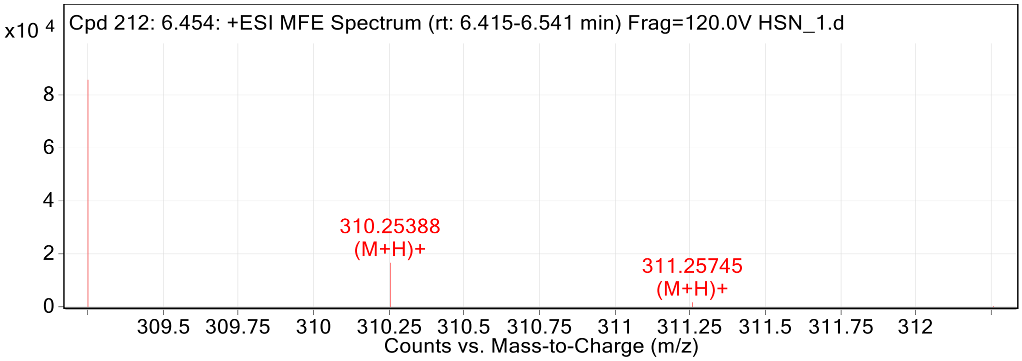

Peak List

m/z	z	Abund	Ion
309.25042	1	86019.84	(M+H)+
310.25388	1	16711.03	(M+H)+
311.25745	1	1693.23	(M+H)+
312.25797	1	290.92	(M+H)+

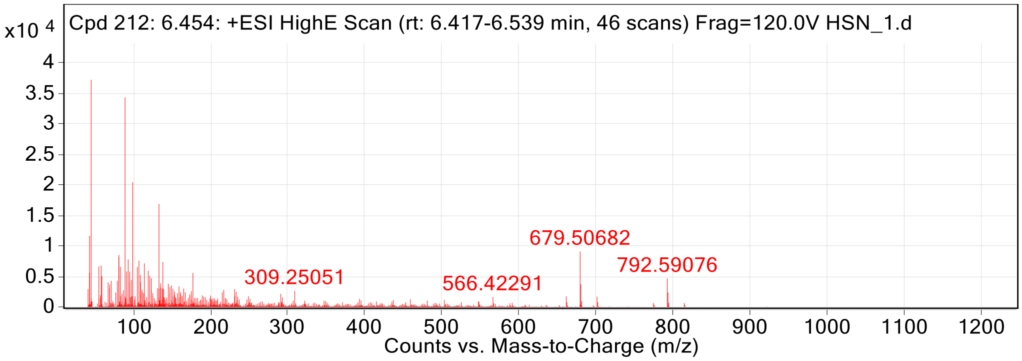

Peak List

m/z	z	Abund
43.01753		11666.56
45.03313		37211.49
80.97248		8181.79
81.06888		8513.21
89.05842		34342.52
93.06854		7792.91
98.98272		20437.84
107.08375		7589.16
133.08412		16895.56
679.50682	1	9077

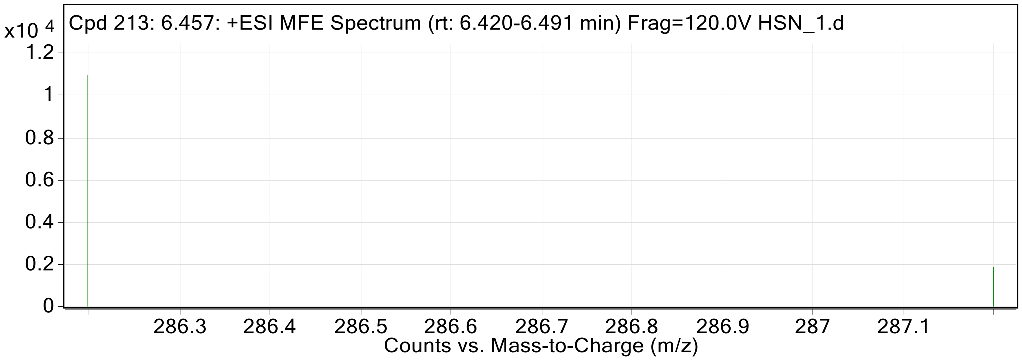

Peak List

m/z	z	Abund	Ion
286.19797	1	10966.71	(M+H)+
287.19921	1	1869.66	(M+H)+

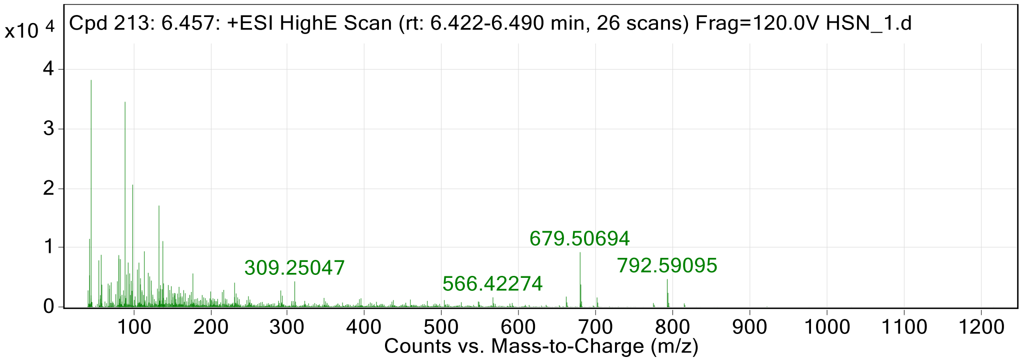

Peak List

m/z	z	Abund
43.01751		11497.27
45.03316		38313.68
58.06456		8800.65

80.97248		8746.54
89.0584		34621.35
98.98274		20640.18
114.08958		9390.2
133.08413		17111.99
138.12563		11114.2
679.50694	1	9245.96

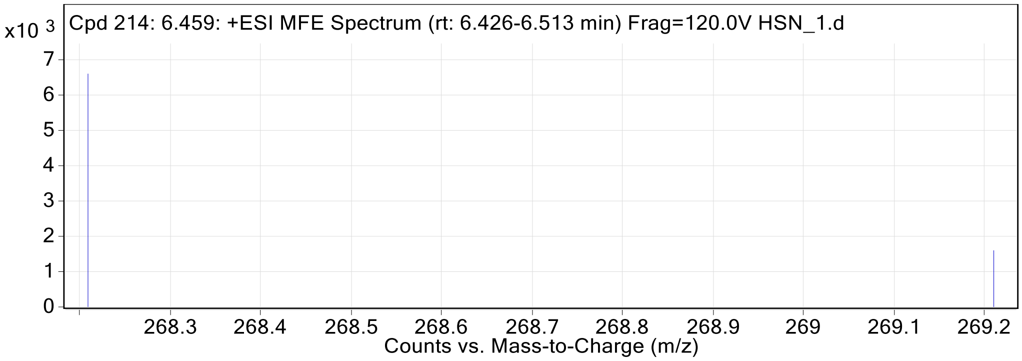

Peak List

m/z	z	Abund	Ion
268.209	1	6592.96	(M+H)+
269.21068	1	1599.05	(M+H)+

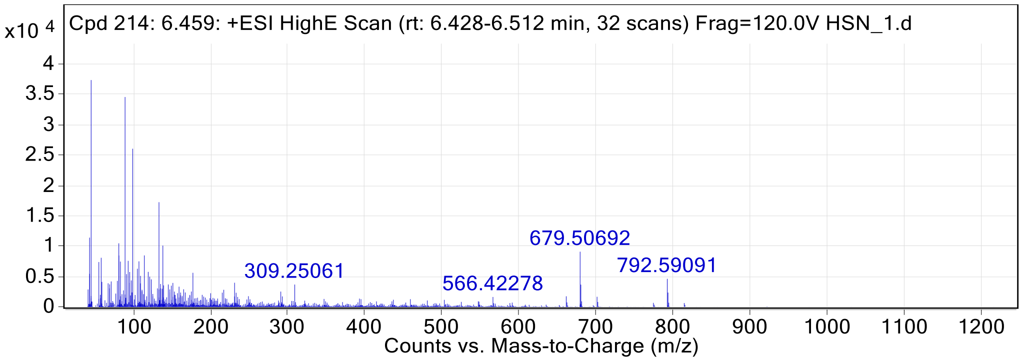

Peak List

m/z	z	Abund
43.01752		11399.5
45.03314		37371.57
80.97248		10458.58
81.06894		8441.29
89.05841		34576.35
98.98271		26051.9
114.08957		8463.45
133.08408		17238.21
138.12563		10091.67
679.50692	1	9079.22

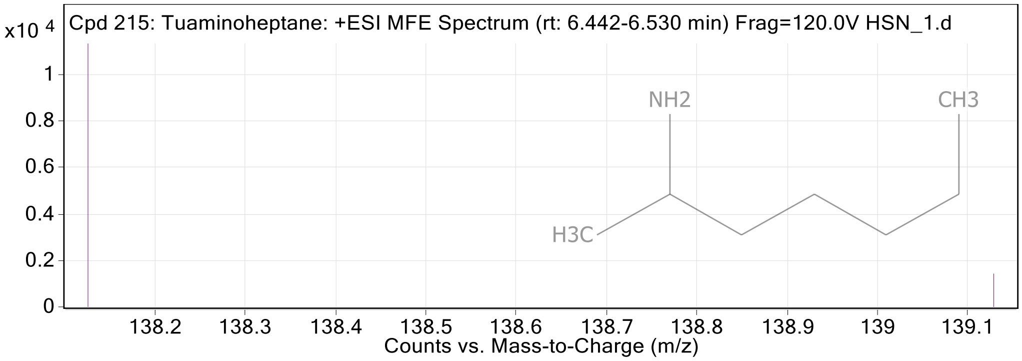

Peak List

m/z	z	Abund	Name	Formula	Ion	Score (DB)	Hits (DB)
138.12563	1	11343.99	Tuaminoheptane	C7 H17 N	(M+Na)+	81.14	3
139.12886	1	1432.54		C7 H17 N	(M+Na)+		

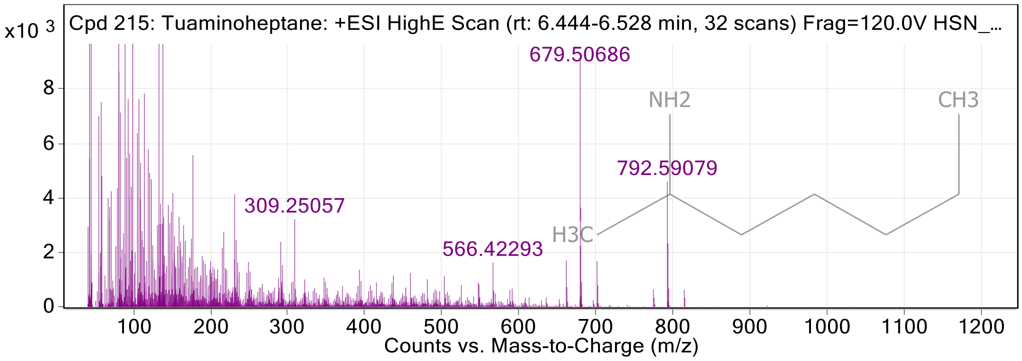

Peak List

m/z	z	Abund
43.01753		11394.69
45.03315		37073.89
80.97247		11332.54
81.06893		8650.53
89.05842		34312.47
98.98273		28269.17
114.08959		7833.93
133.08412		16946.94
138.12564		9669
679.50686	1	9055.78

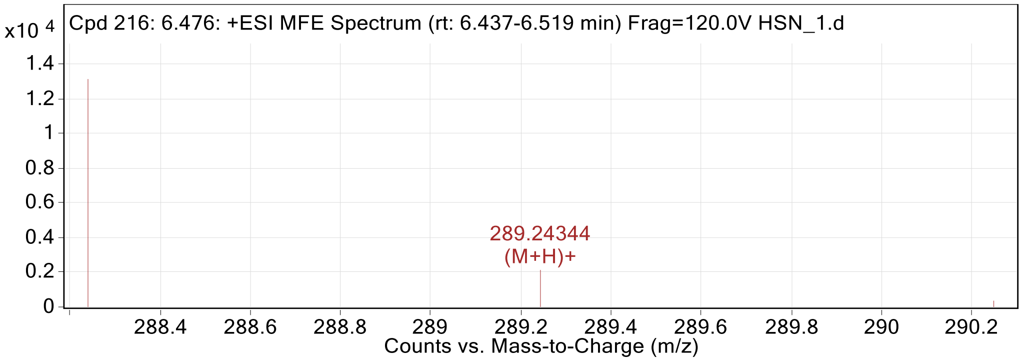

Peak List

m/z	z	Abund	Ion
288.24012	1	13151.7	(M+H)+
289.24344	1	2136.7	(M+H)+
290.24876	1	361.94	(M+H)+

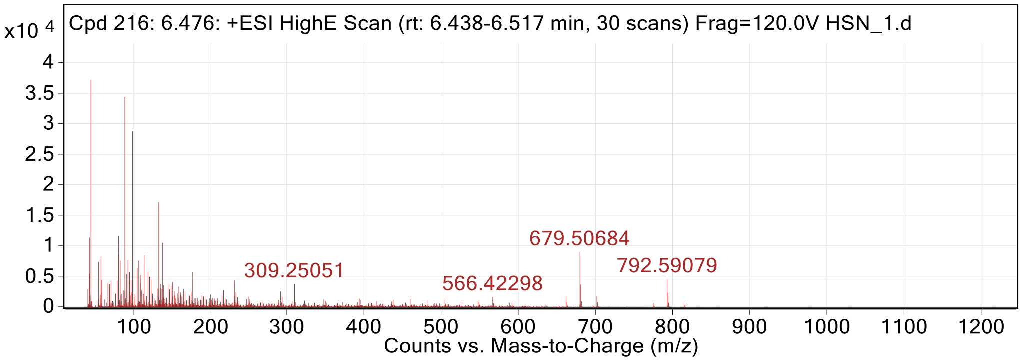

Peak List

m/z	z	Abund
43.01751		11379
45.03316		37166.91
80.97247		11577.17
81.06895		8547.16
89.05839		34424.86
98.98272		28764.89
114.08958		8432.14
133.08412		17174.79
138.12564		10491.82
679.50684	1	8988.72

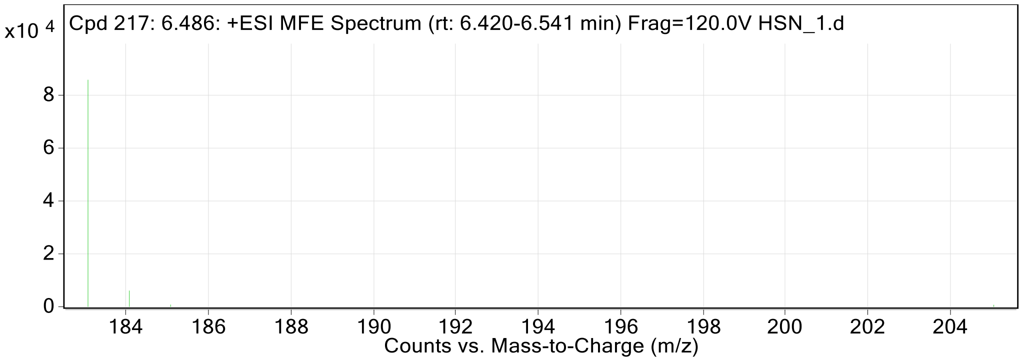

Peak List

m/z	z	Abund	Ion
183.07578	1	85882.71	(M+H)+
184.07925	1	6123.29	(M+H)+
185.08056	1	823.6	(M+H)+
205.05734	1	743.01	(M+Na)+

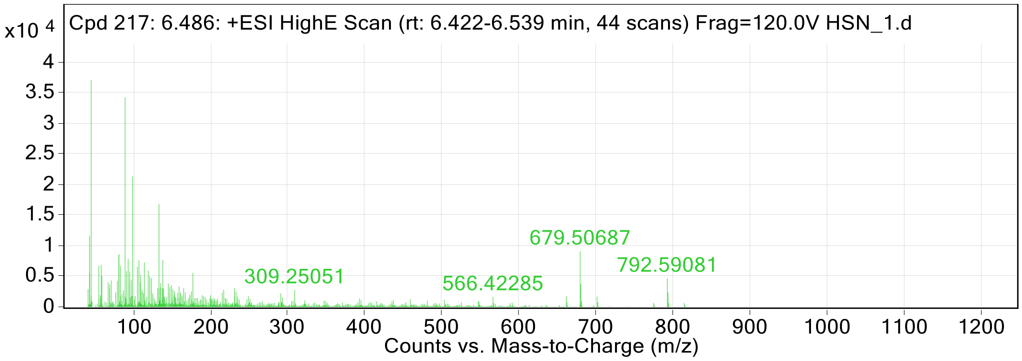

Peak List

m/z	z	Abund
43.01753		11625.68
45.03313		37135.11
80.97248		8548.1
81.06887		8549.23
89.05843		34267.93
93.06854		7808.78
98.98272		21343.71
133.08413		16824.46
138.12564		7666.08
679.50687	1	9086.07

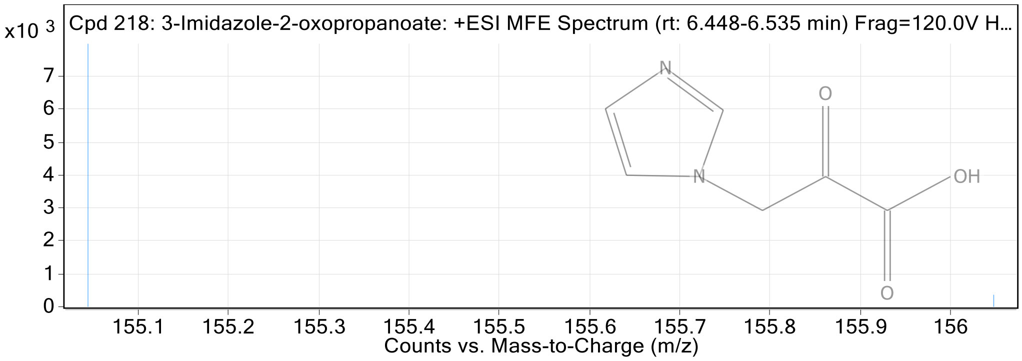

Peak List

m/z	z	Abund	Name	Formula	Ion	Score (DB)	Hits (DB)
155.04455	1	7986.2	3-Imidazole-2-oxopropanoate	C6 H6 N2 O3	(M+H)+	83.03	6
156.04787	1	364.22		C6 H6 N2 O3	(M+H)+		

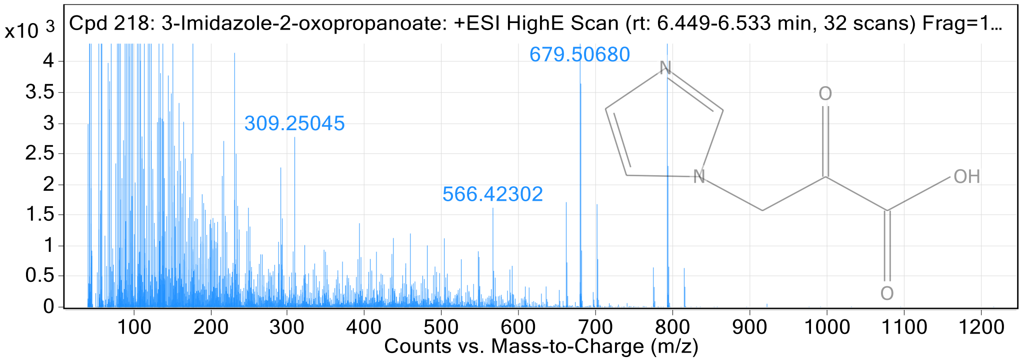

Peak List

m/z	z	Abund
43.01752		11463.69
45.03315		36894.22
80.97247		11461.16
81.06892		8733.89
89.05844		34268.8
93.06856		7670.76
98.98273		28688.98
133.08413		16743.8
138.12568		9086.86
679.5068	1	9043.41

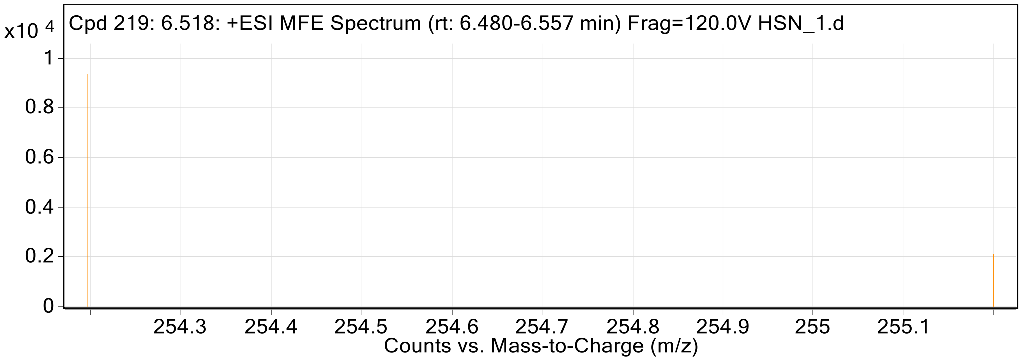

Peak List

m/z	z	Abund	Ion
254.19708	1	9356.4	(M+H)+
255.19927	1	2125.49	(M+H)+

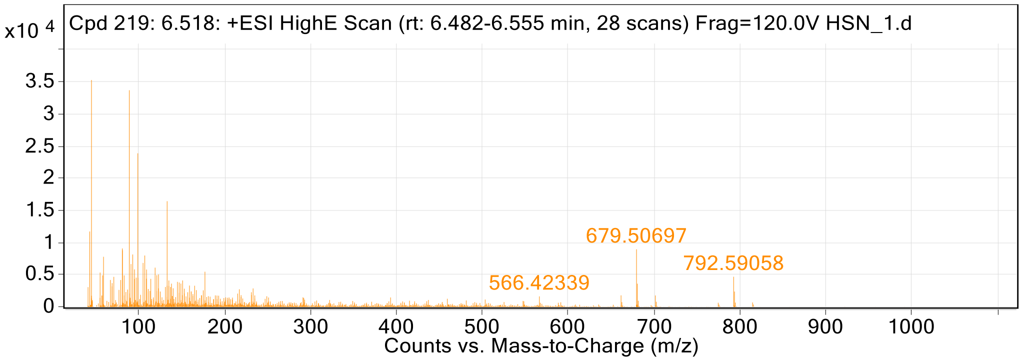

Peak List

m/z	z	Abund
43.01753		11705.78
45.03313		35278.91
80.97249		9066.19
81.06885		8793.58
89.05845		33687.23
93.06858		8126.01
98.98274		23871.5
107.08375		7966.95
133.08417		16420.31
679.50697	1	8932.67

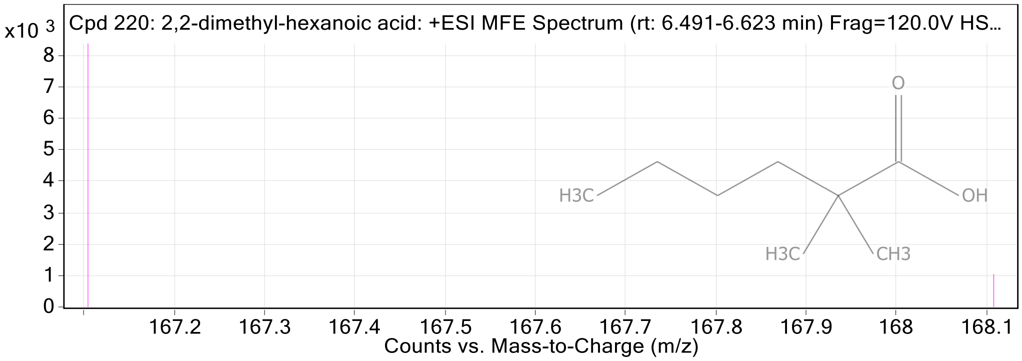

Peak List

m/z	z	Abund	Name	Formula	Ion	Score (DB)	Hits (DB)
167.10438	1	8383.59	2,2-dimethyl-hexanoic acid	C8 H16 O2	(M+Na)+	83.45	10
168.10791	1	1052.2		C8 H16 O2	(M+Na)+		

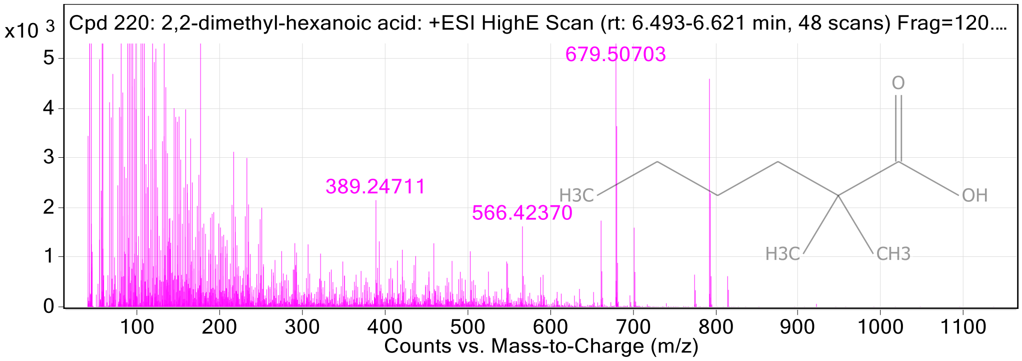

Peak List

m/z	z	Abund
43.01756		13012.34
45.03315		34333
59.04852		18723.09
81.0688		8669.34
89.05848		33027.12
93.06855		8478.39
98.98272		10229.57
107.08382		8075.01
133.08436		16137.44
679.50703	1	8889.24

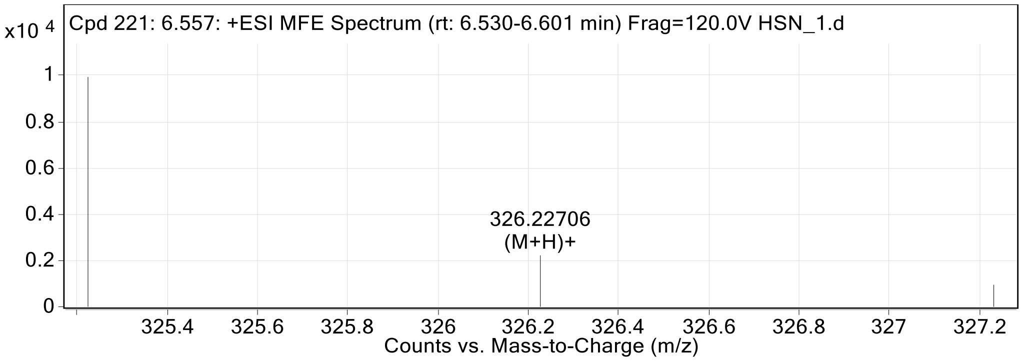

Peak List

m/z	z	Abund	Ion
325.22445	1	9933.16	(M+H)+
326.22706	1	2219.56	(M+H)+
327.23208	1	951.78	(M+H)+

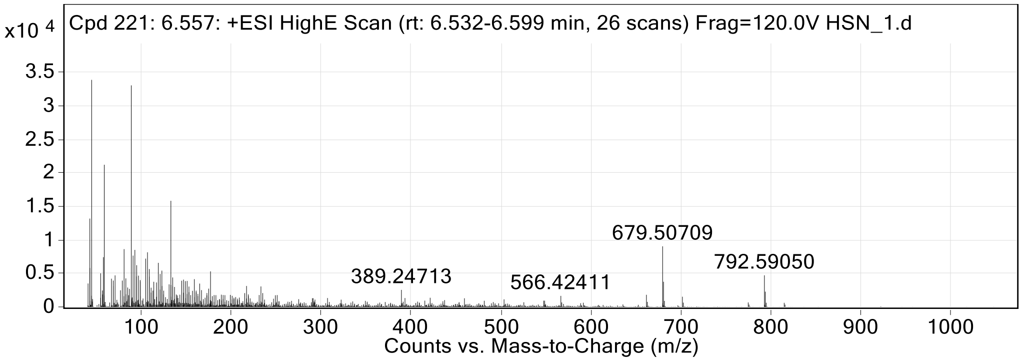

Peak List

m/z	z	Abund
43.01754		13180.1
45.03317		33879.73

59.0485		21215.7
81.06875		8621.69
89.05848		33058.02
91.05303		7661.17
93.0685		8505.26
107.08381		8149.28
133.08446		15832.64
679.50709	1	9040.46

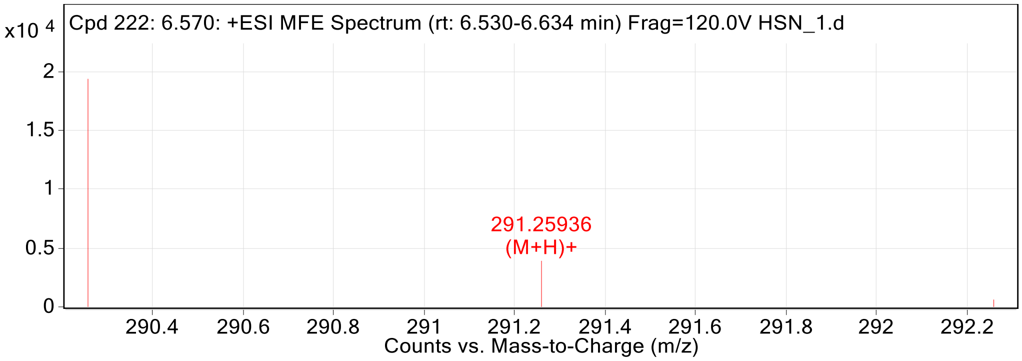

Peak List

m/z	z	Abund	Ion
290.25643	1	19393.91	(M+H)+
291.25936	1	3916.94	(M+H)+
292.25959	1	621.33	(M+H)+

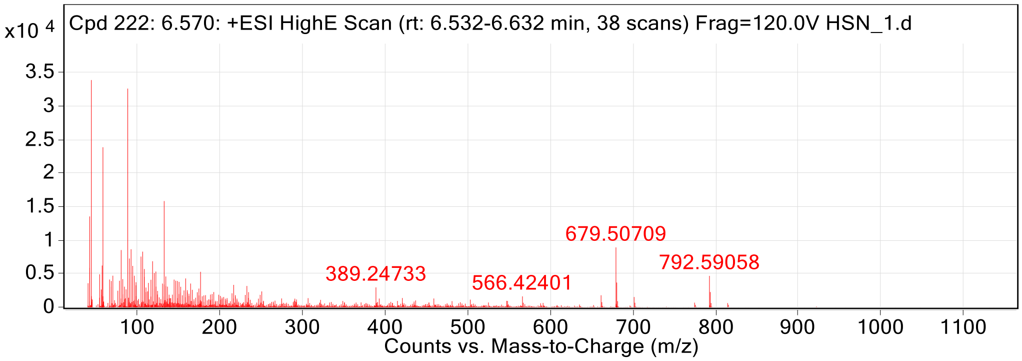

Peak List

m/z	z	Abund
43.01754		13524.87
45.03316		33872.32
59.04851		23844.53
81.06875		8507.38
89.05847		32598.31
93.0685		8630.13
105.06826		7538.69
107.08384		8272.29
133.08448		15818.98
679.50709	1	8905.93

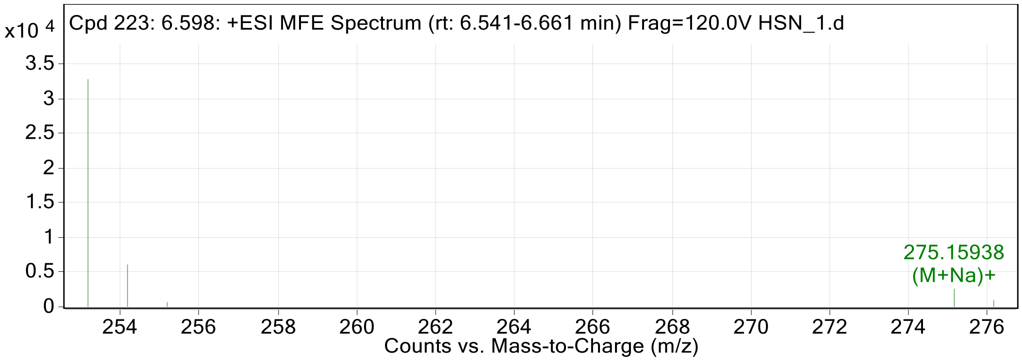

Peak List

m/z	z	Abund	Ion
253.1773	1	32781.01	(M+H)+
254.18037	1	6111.5	(M+H)+
255.18615	1	676.95	(M+H)+
275.15938	1	2607.28	(M+Na)+
276.16382	1	973.4	(M+Na)+

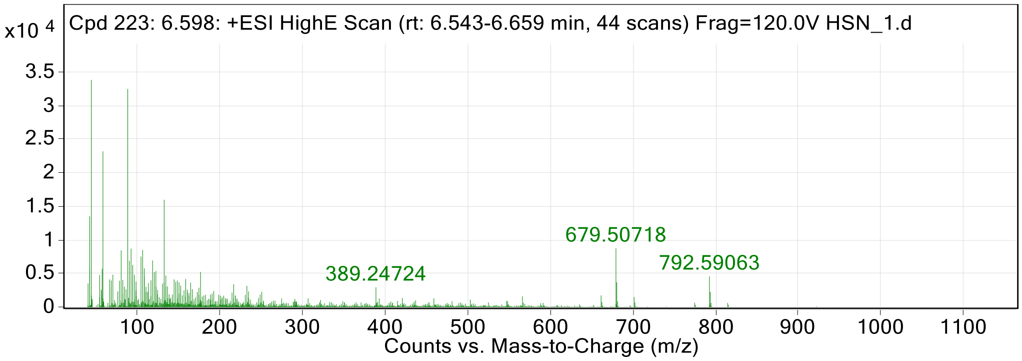

Peak List

m/z	z	Abund
43.01756		13492.98
45.03318		33774.6
59.04852		23146.97
81.06879		8428.55
89.05845		32429.16
93.06848		8678.04
105.06823		7500.84
107.08389		8465.89
133.08444		15946.34
679.50718	1	8722.64

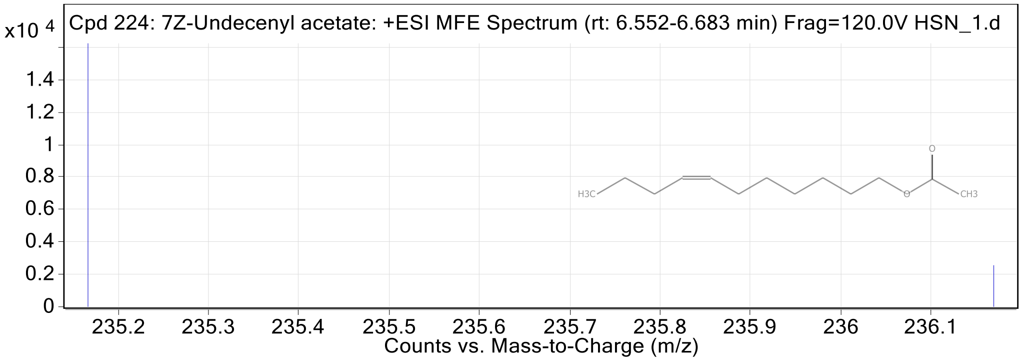

Peak List

m/z	z	Abund	Name	Formula	Ion	Score (DB)	Hits (DB)

235.16677	1	16235.1	7Z-Undecenyl acetate	C13 H24 O2	(M+Na)+	85.17	10
236.16928	1	2560.5		C13 H24 O2	(M+Na)+		

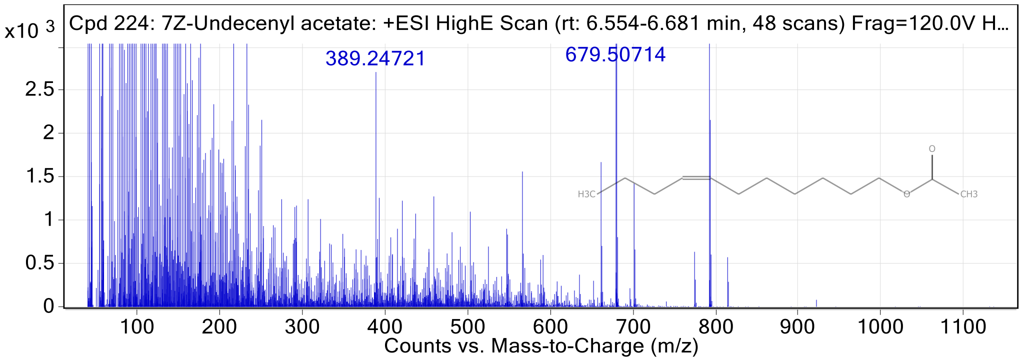

Peak List

m/z	z	Abund
43.01755		13400.32
45.03318		33521.63
59.04854		21649.88
81.06878		9334.23
89.05844		32019.4
93.0685		8767.58
105.06823		7439.81
107.08391		8638.11
133.08444		15803.51
679.50714	1	8536.24

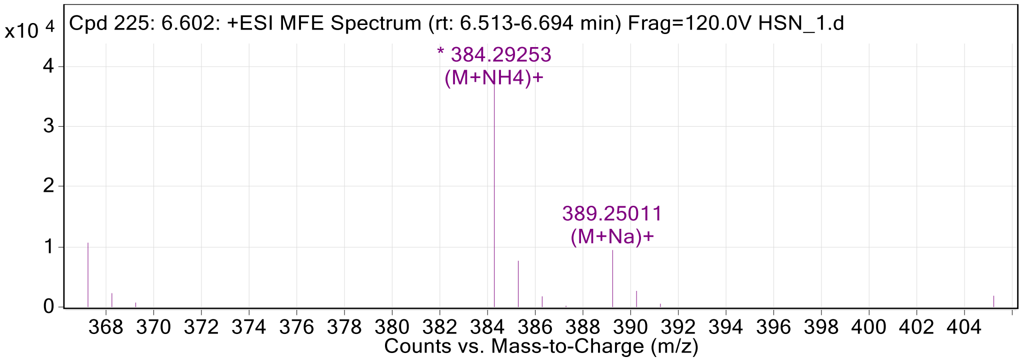

Peak List

m/z	z	Abund	Ion
367.2657	1	10698.2	(M+H)+
368.26809	1	2303.13	(M+H)+
369.26664	1	734.5	(M+H)+
384.29253	1	37774.63	(M+NH4)+
385.29538	1	7690.71	(M+NH4)+
386.29747	1	1777.3	(M+NH4)+
389.25011	1	9474.54	(M+Na)+
390.25154	1	2678.93	(M+Na)+
391.25174	1	549.04	(M+Na)+
405.22056	1	1873.67	(M+K)+

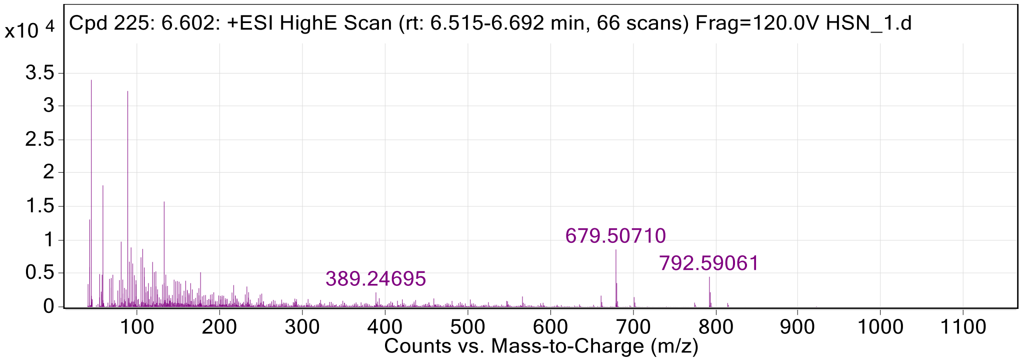

Peak List

m/z	z	Abund
43.01754		13062.5
45.03317		33934.24
59.04854		18165.75
81.06879		9748.69
89.05845		32268.24
93.0685		8887.89
105.06823		7411.3
107.08386		8662.82
133.0844		15746.87
679.5071	1	8585.14

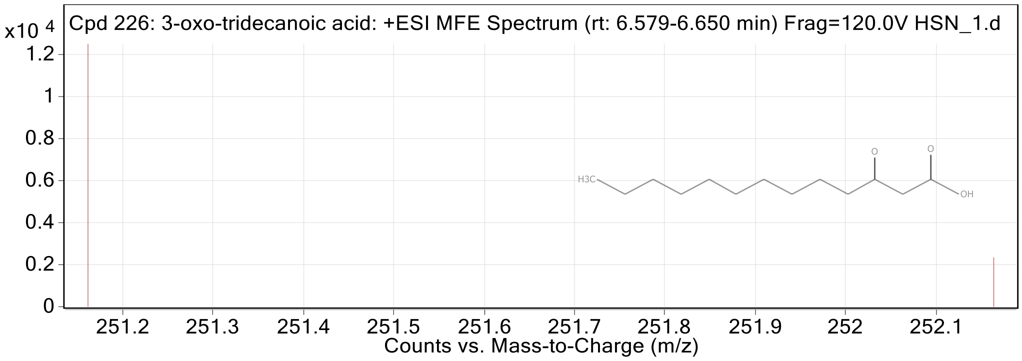

Peak List

m/z	z	Abund	Name	Formula	Ion	Score (DB)	Hits (DB)
251.16159	1	12532.48	3-oxo-tridecanoic acid	C13 H24 O3	(M+Na)+	80.6	10
252.16415	1	2349.21		C13 H24 O3	(M+Na)+		

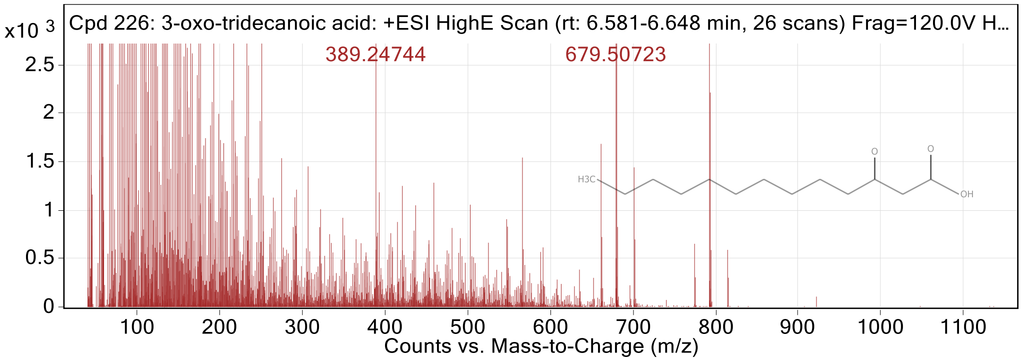

Peak List

m/z	z	Abund
43.01754		14379.58
45.03316		33613.27
59.04852		27633.2

81.06877		8391.76
89.05844		31815.35
93.06848		8847.89
105.06822		7976.2
107.08393		8512.85
133.08453		16000.19
679.50723	1	8592.24

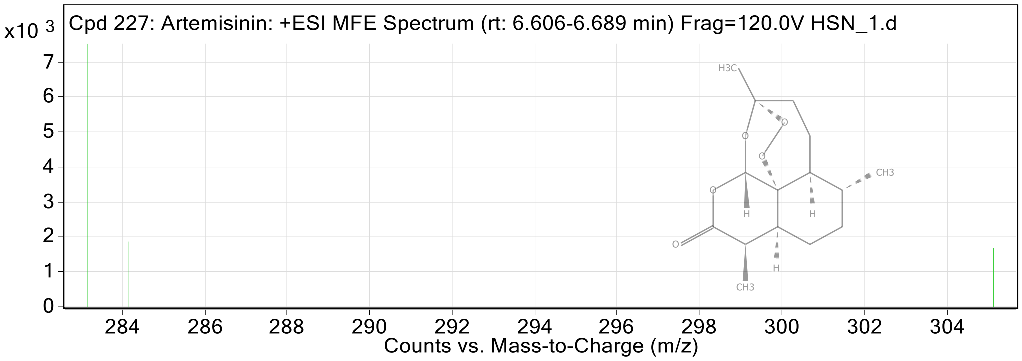

Peak List

m/z	z	Abund	Name	Formula	Ion	Score (DB)	Hits (DB)
283.15507	1	7523.6	Artemisinin	C15 H22 O5	(M+H)+	64.55	10
284.15319	1	1864.39		C15 H22 O5	(M+H)+		
305.1347	1	1686.09			(M+Na)+		

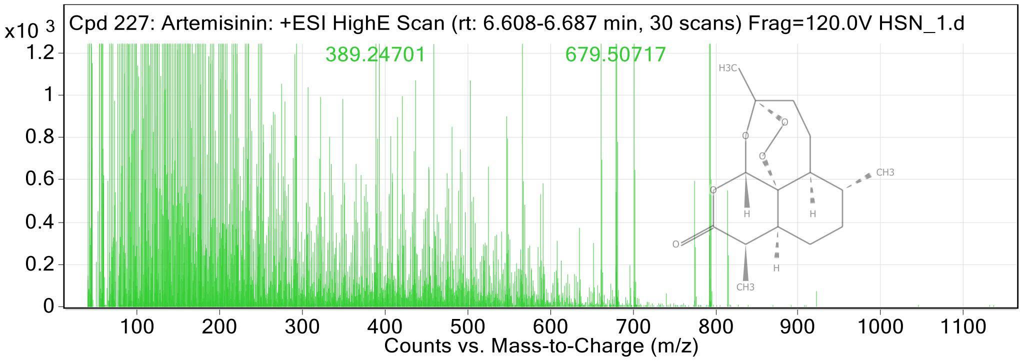

Peak List

m/z	z	Abund
43.01754		13039.31
45.03318		33799.78
59.04856		17153.93
81.0688		10493.1
89.05842		31457.33
93.0685		9135.42
105.06819		7669.54
107.08392		9035.92
133.08434		15610.51
679.50717	1	8170.53

Qualitative Analysis Report

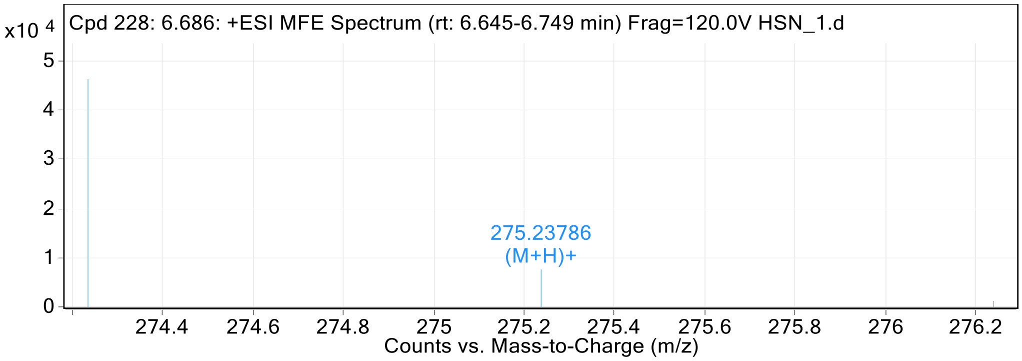

Peak List

m/z	z	Abund	Ion
274.2349	1	46303.08	(M+H)+
275.23786	1	7627.26	(M+H)+
276.23993	1	1209.5	(M+H)+

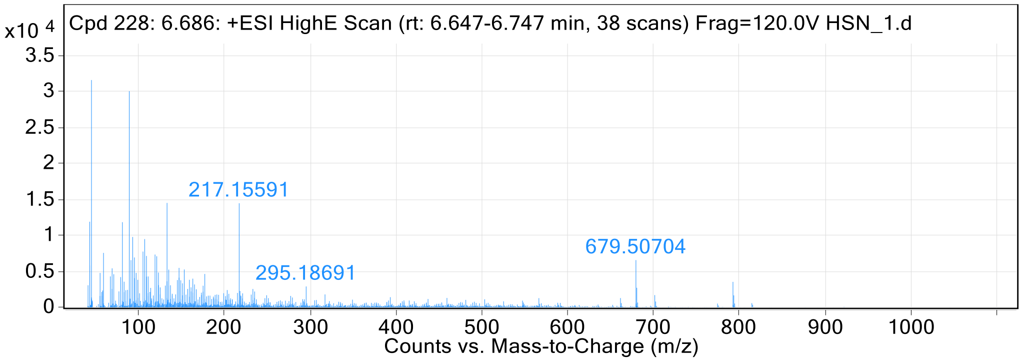

Peak List

m/z	z	Abund
43.01751		11862.48
45.03317		31552.77
59.04856		7530.22
81.06884		11798.76
89.0584		30002.96
93.06847		9743.47
105.06821		7714.93
107.08377		9445.22
133.08437		14474.38
217.15591	1	14428.2

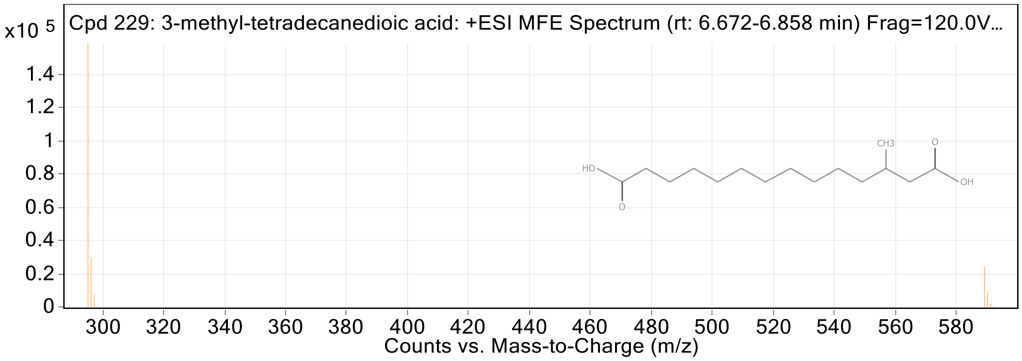

Peak List

m/z	z	Abund	Name	Formula	Ion	Score (DB)	Hits (DB)
295.18755	1	158475.45	3-methyl-tetradecanedioic acid	C15 H28 O4	(M+Na)+	96.53	4

296.1906	1	29831.69		C15 H28 O4	(M+Na)+		
297.19384	1	7189.31		C15 H28 O4	(M+Na)+		
298.19919	1	435.27		C15 H28 O4	(M+Na)+		
589.36936	1	24293.18			(2M+H)+		
590.37304	1	8765.75			(2M+H)+		
591.37441	1	2157.4			(2M+H)+		
592.3765	1	616.03			(2M+H)+		

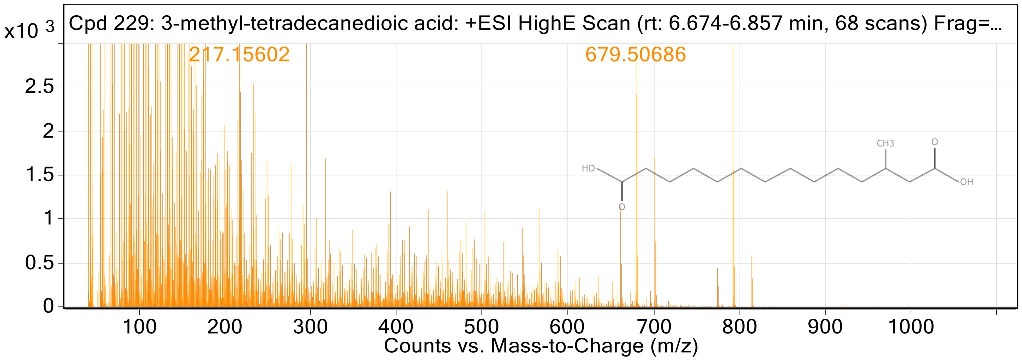

Peak List

m/z	z	Abund
43.01751		11853.84
45.03315		30450.47
81.06879		11063.44
89.05842		28945.34
93.06846		9955.15
105.06823		7901.21
107.08379		9601.21
109.09938		7621.03
133.08447		13762.79
217.15602	1	15932.21

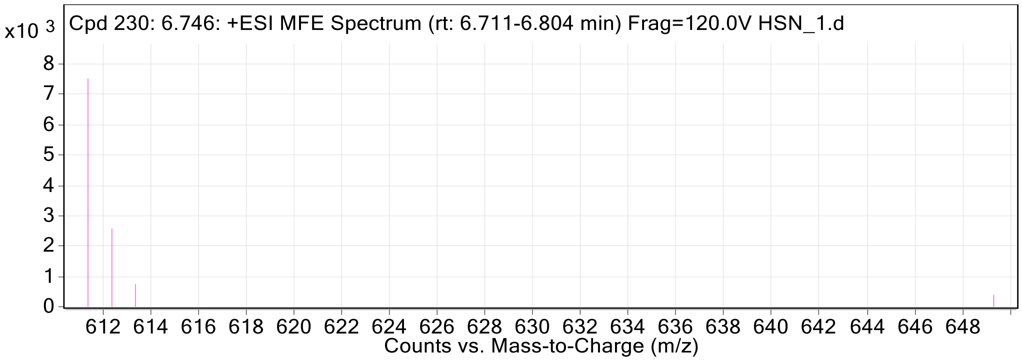

Peak List

m/z	z	Abund	Ion
611.35087	1	7528.38	(M+H)+
612.35513	1	2584.17	(M+H)+
613.33975	1	744.54	(M+H)+
649.31204	1	394.31	(M+K)+

Qualitative Analysis Report

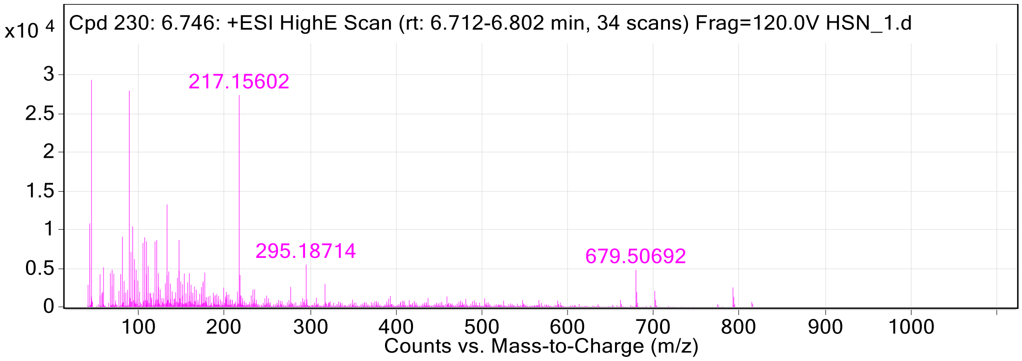

Peak List

m/z	z	Abund
43.01751		10803.45
45.03317		29401.73
81.06881		9077.5
89.05839		27994.28
93.06849		10423.57
107.08374		8997.2
121.09916		8661.31
133.0845		13268.2
147.07815		8663.04
217.15602	1	27421.77

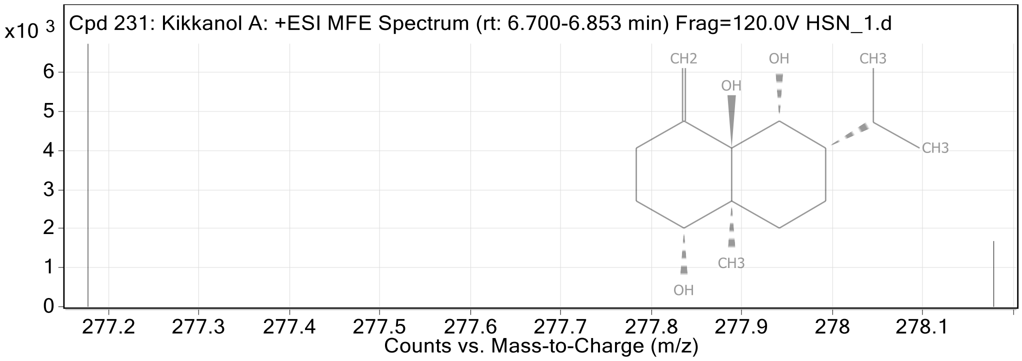

Peak List

m/z	z	Abund	Name	Formula	Ion	Score (DB)	Hits (DB)
277.17698	1	6740.52	Kikkanol A	C15 H26 O3	(M+Na)+	68.49	10
278.17879	1	1683.59		C15 H26 O3	(M+Na)+		

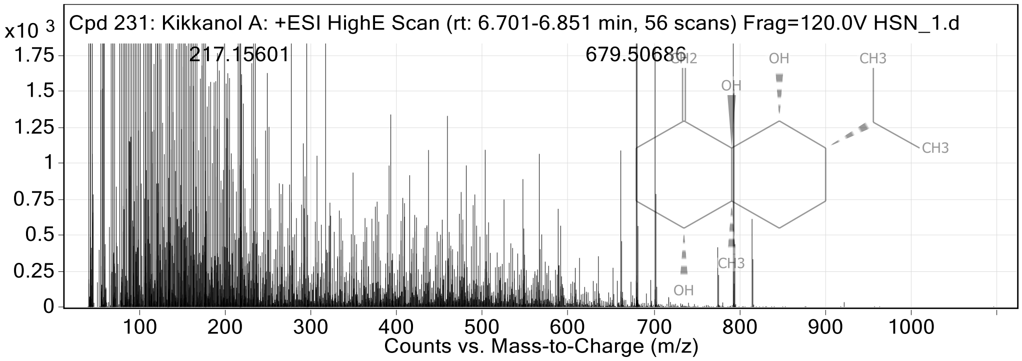

Peak List

m/z	z	Abund
43.01751		11706.03
45.03315		30070.07
81.06878		9984.09

89.05841		28757.76
93.06845		9976.79
105.06822		7983.14
107.08378		9452.17
109.09937		7875.18
133.08448		13623.35
217.15601	1	18631.91

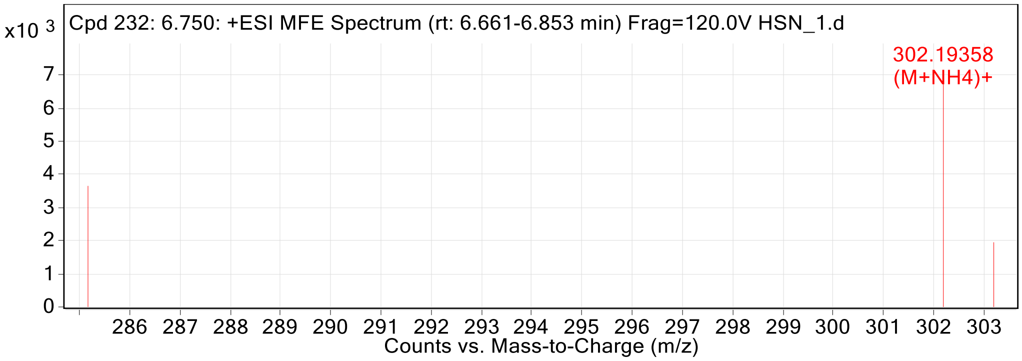

Peak List

m/z	z	Abund	Ion
285.16712	1	3654	(M+H)+
302.19358	1	7063.71	(M+NH4)+
303.1971	1	1951.38	(M+NH4)+

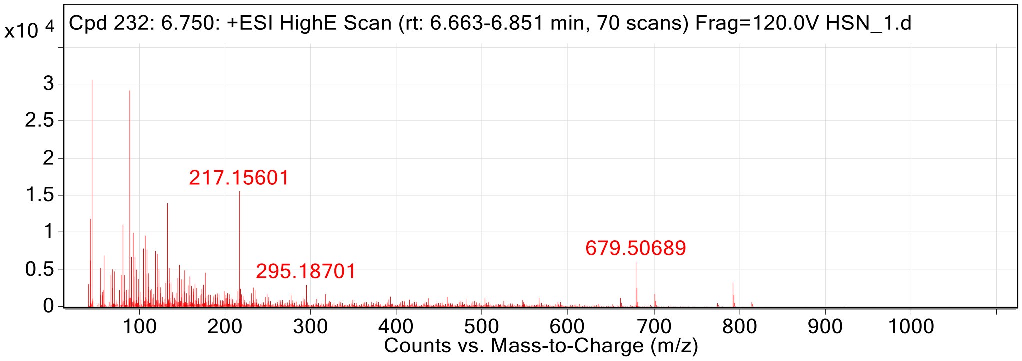

Peak List

m/z	z	Abund
43.0175		11822.02
45.03316		30590.31
81.06879		11045.52
89.05842		29142.58
93.06847		9949.85
105.06823		7811.66
107.08379		9562.88
109.09937		7575.02
133.08447		13926.77
217.15601	1	15547.2

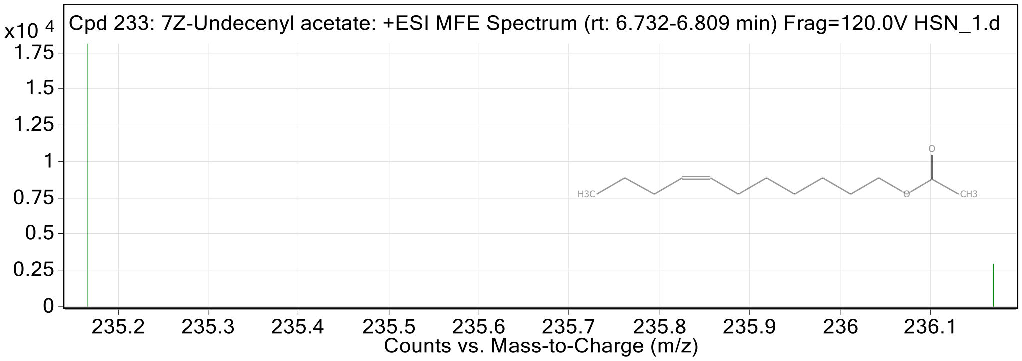

Peak List

m/z	z	Abund	Name	Formula	Ion	Score (DB)	Hits (DB)
235.16649	1	18111.83	7Z-Undecenyl acetate	C13 H24 O2	(M+Na)+	85.6	10
236.16984	1	2939.81		C13 H24 O2	(M+Na)+		

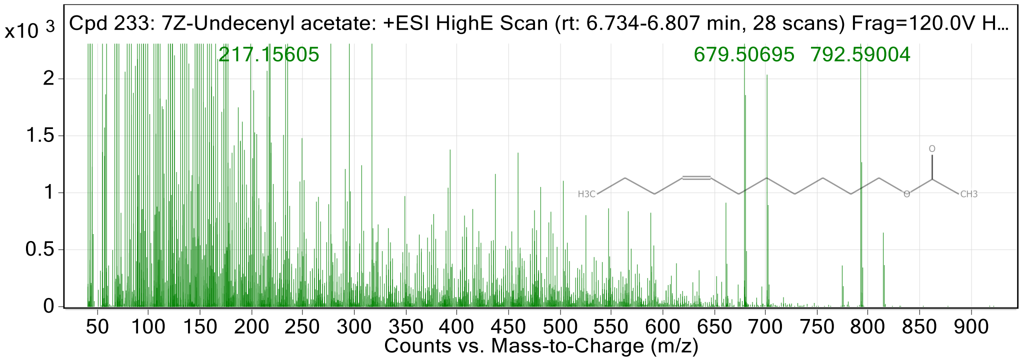

Peak List

m/z	z	Abund
43.01751		10743.58
45.03318		29133.05
81.0688		8883.84
89.0584		27704.85
93.06846		10527.26
107.0838		9085.13
109.09944		8566.08
121.09916		8606.95
133.08458		13203.43
217.15605	1	27735.63

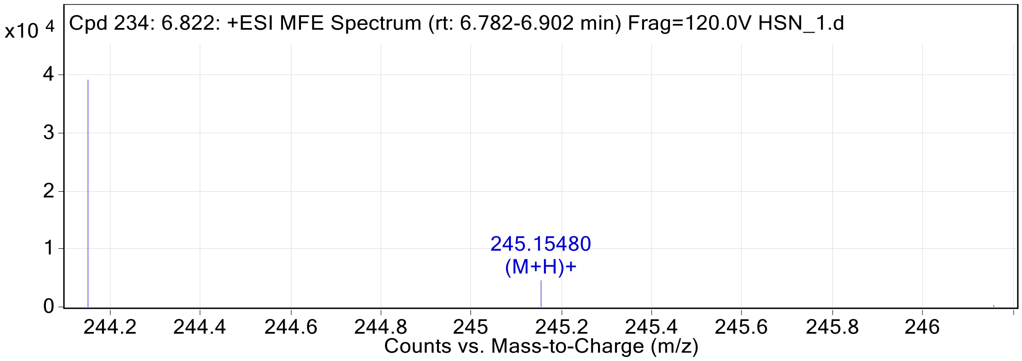

Peak List

m/z	z	Abund	Ion
244.15145	1	39179.78	(M+H)+
245.1548	1	4598.21	(M+H)+
246.15734	1	308.89	(M+H)+

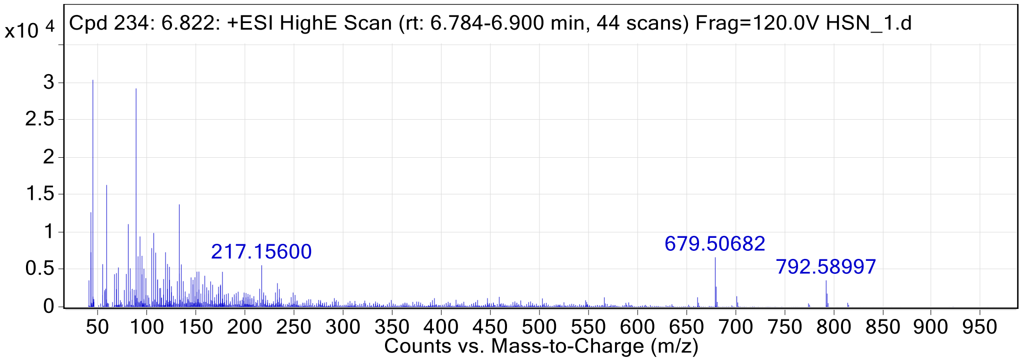

Peak List

m/z	Abund
43.01752	12658.57
45.03315	30367.85
59.04852	16307.25
81.06874	11066.59
89.05843	29222.07
93.06841	9415.78
105.06816	7863.13
107.08381	9882.64
119.08353	7305.66
133.08449	13708.98

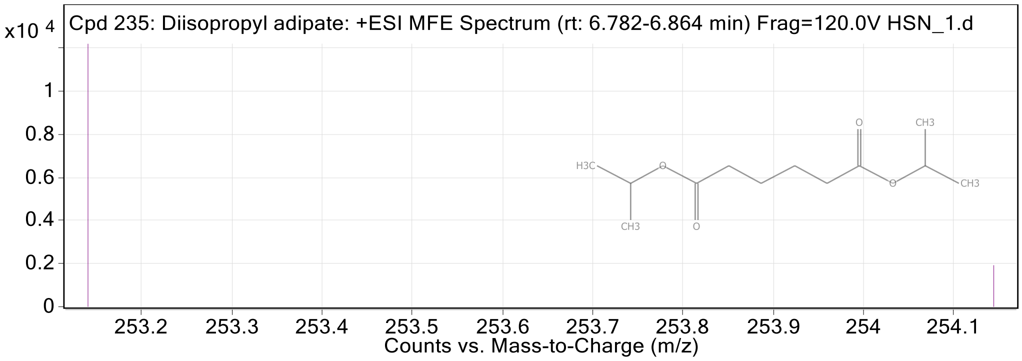

Peak List

m/z	z	Abund	Name	Formula	Ion	Score (DB)	Hits (DB)
253.1408	1	12209.81	Diisopropyl adipate	C12 H22 O4	(M+Na)+	84.91	5
254.1443	1	1928.32		C12 H22 O4	(M+Na)+		

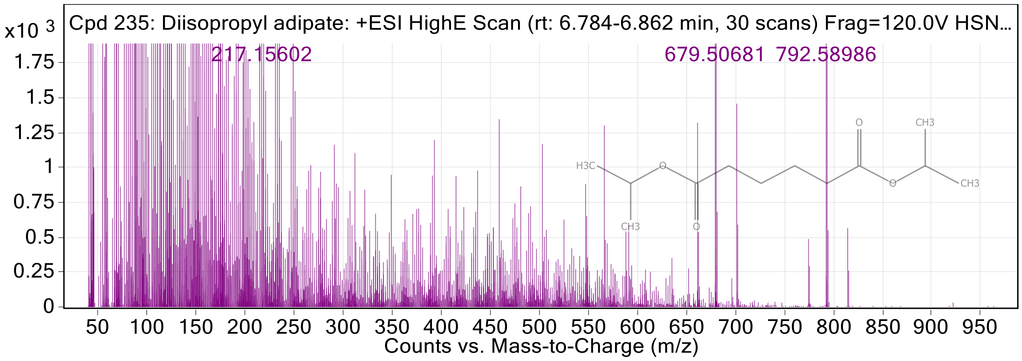

Peak List

m/z	Abund
43.01751	13007.77
43.05392	7506.05
45.03314	30661.29

59.04854	9487.48
81.06875	10765.56
89.05844	29491.07
93.06842	9556.56
105.06823	7584.76
107.08383	9965.13
133.08443	13858.59

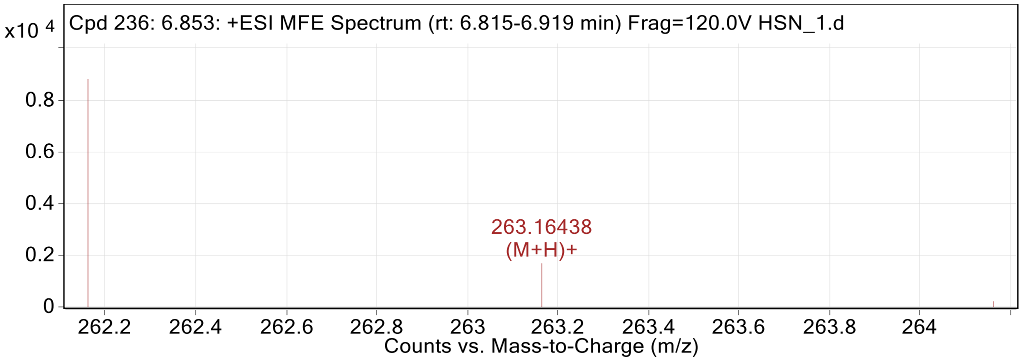

Peak List

m/z	z	Abund	Ion
262.16198	1	8802.97	(M+H)+
263.16438	1	1683.07	(M+H)+
264.16251	1	219.02	(M+H)+

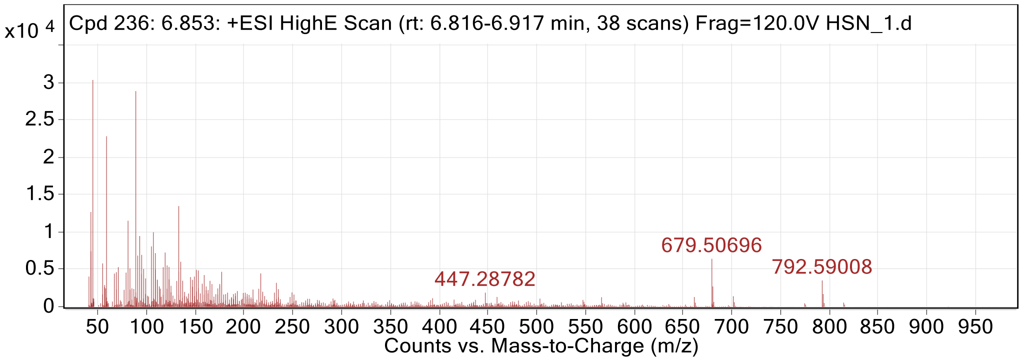

Peak List

m/z	Abund
43.01753	12669.71
43.05393	7424.02
45.03315	30333.36
59.04854	22813.7
81.06872	11503.28
89.05843	28848.03
93.06843	9460.37
105.06815	8069.74
107.08382	9963.96
133.08453	13463.56

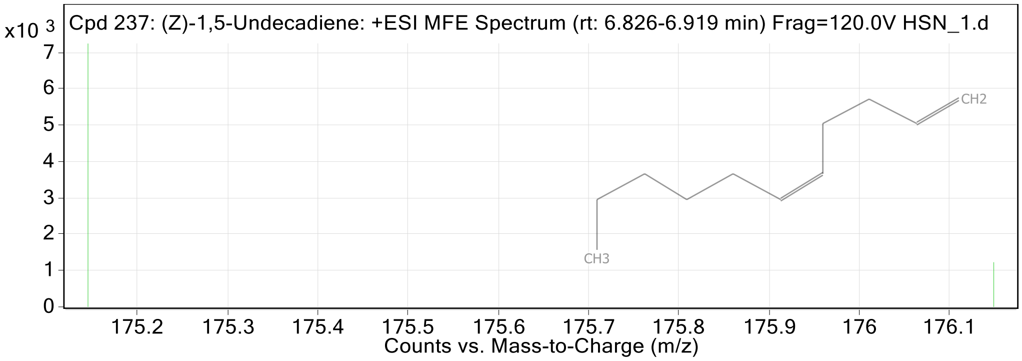

Peak List

m/z	z	Abund	Name	Formula	Ion	Score (DB)	Hits (DB)
175.14547	1	7243.78	(Z)-1,5-Undecadiene	C11 H20	(M+Na)+	81.28	4
176.149	1	1228.46		C11 H20	(M+Na)+		

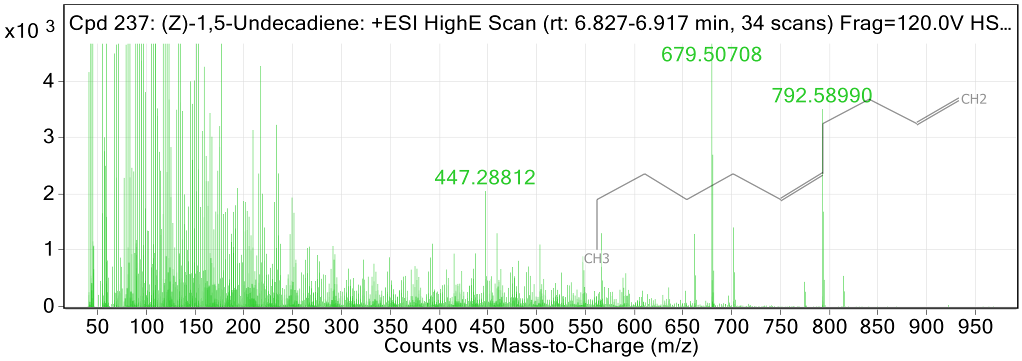

Peak List

m/z	Abund
43.01754	12729.82
45.03315	30162.03
59.04854	24588.18
81.06873	11640.96
89.05843	28732.59
93.06842	9451.85
105.06813	8235.4
107.08381	9953.27
119.08354	7297.44
133.08454	13447.73

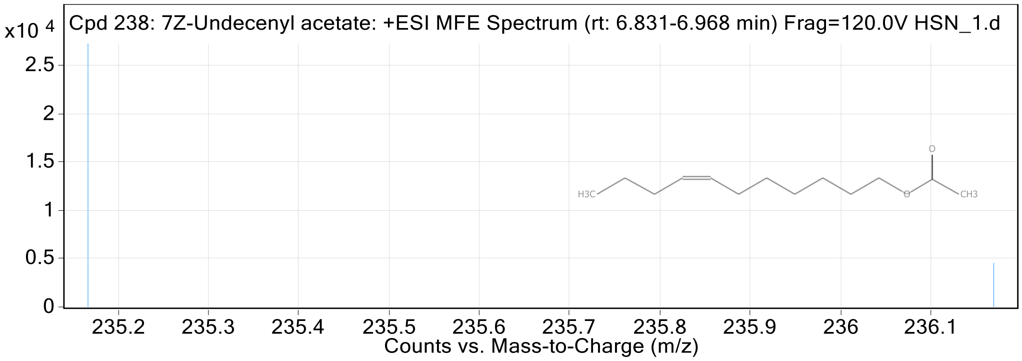

Peak List

m/z	z	Abund	Name	Formula	Ion	Score (DB)	Hits (DB)
235.16656	1	27235.82	7Z-Undecenyl acetate	C13 H24 O2	(M+Na)+	85.24	10
236.16985	1	4547.18		C13 H24 O2	(M+Na)+		

Qualitative Analysis Report

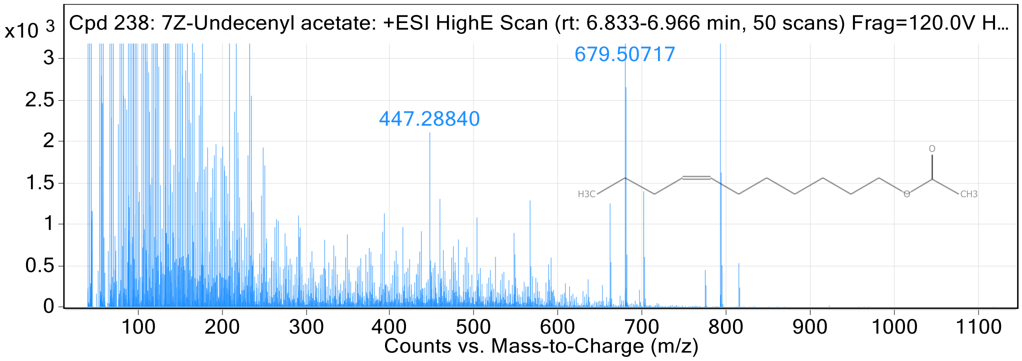

Peak List

m/z	Abund
43.01756	12496.9
43.05391	7209.44
45.03318	29638.54
59.04856	24055.36
81.06874	11393.63
89.05846	28556.44
93.06848	9813.58
105.06821	7640.13
107.08387	10037.85
133.08454	13405.72

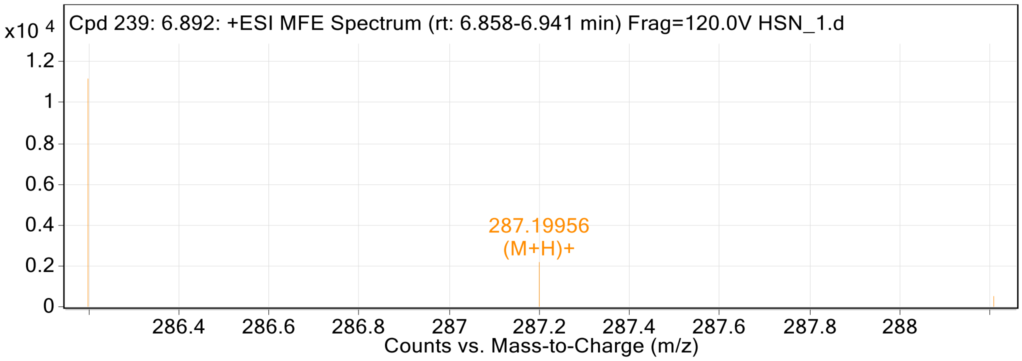

Peak List

m/z	z	Abund	Ion
286.19838	1	11173.9	(M+H)+
287.19956	1	2187.74	(M+H)+
288.20806	1	521.08	(M+H)+

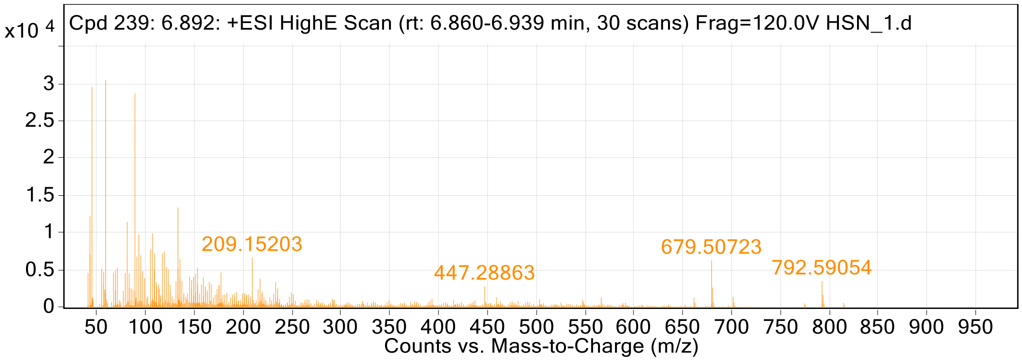

Peak List

m/z	Abund
43.01757	12240.99
45.03318	29549.14

59.04855	30508.21
81.06873	11431.22
89.05844	28675.38
93.06849	9754.83
105.06819	7693.29
107.08386	9894.98
119.08361	7451.96
133.08454	13361.97

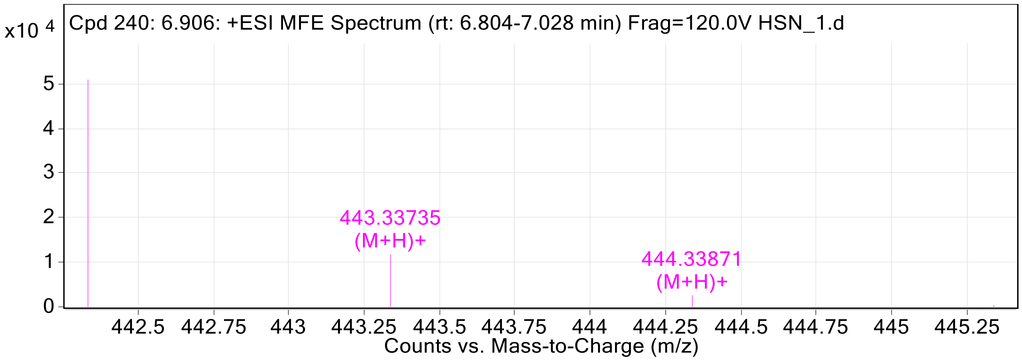

Peak List

m/z	z	Abund	Ion
442.3335	1	50910.26	(M+H)+
443.33735	1	11793.81	(M+H)+
444.33871	1	2569.59	(M+H)+
445.33909	1	394.48	(M+H)+

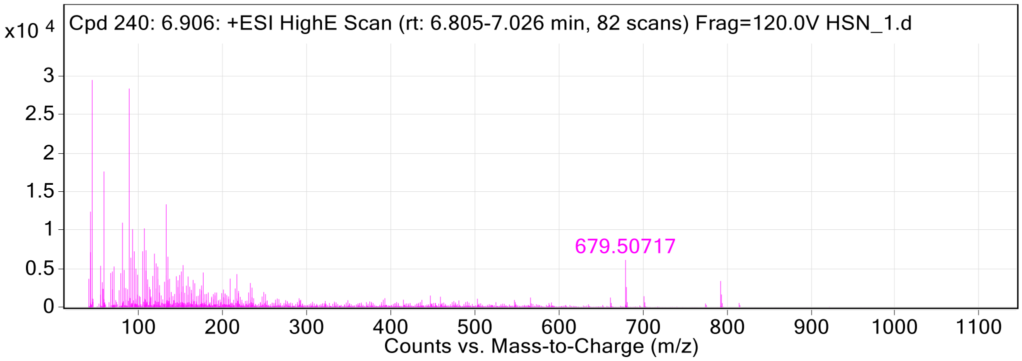

Peak List

m/z	Abund
43.01755	12397.08
45.03318	29511.21
59.04856	17617.7
81.06875	10962.91
89.05846	28399.62
93.06849	10121.56

m/z	Abund
105.06826	7245.38
107.0839	10218.33
109.09938	7377.05
133.08452	13346.25

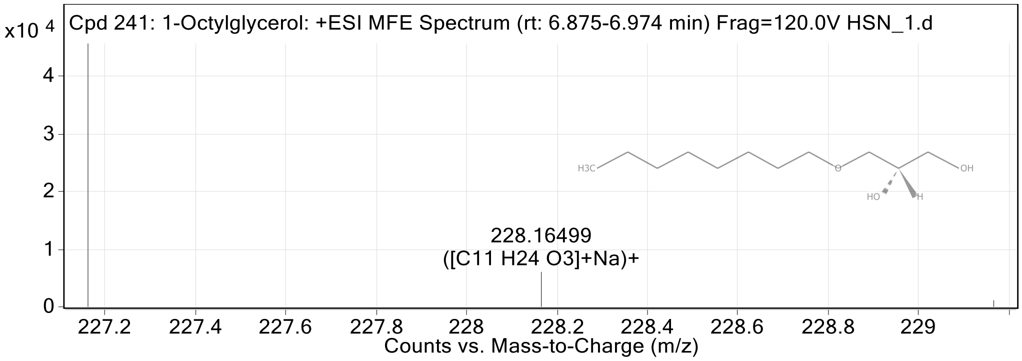

Peak List

m/z	z	Abund	Name	Formula	Ion	Score (DB)	Hits (DB)
227.16169	1	45713.3	1-Octylglycerol	C11 H24 O3	(M+Na)+	99.13	2
228.16499	1	6053.05		C11 H24 O3	(M+Na)+		
229.16621	1	1145.29		C11 H24 O3	(M+Na)+		

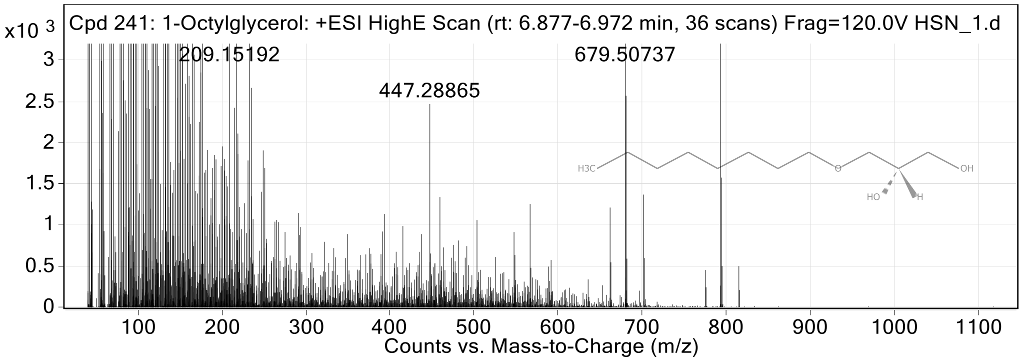

Peak List

m/z	Abund
43.01759	11898.58
45.0332	28997.58
59.04857	26554.38
81.06875	10876.4
89.05847	28325.33
93.06849	10194.82
105.06828	7121.33
107.08388	9892.73
109.09944	7213.2
133.0846	13259.56

Qualitative Analysis Report

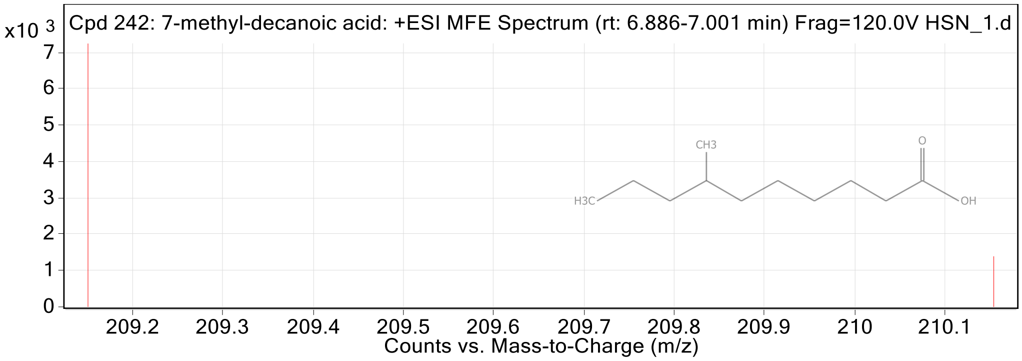

Peak List

m/z	z	Abund	Name	Formula	Ion	Score (DB)	Hits (DB)
209.15093	1	7251.31	7-methyl-decanoic acid	C11 H22 O2	(M+Na)+	75.42	10
210.15385	1	1391.81		C11 H22 O2	(M+Na)+		

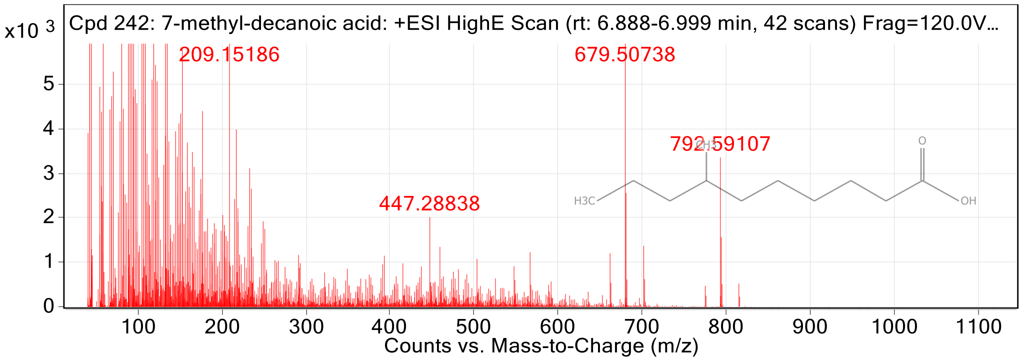

Peak List

m/z	Abund
43.01758	11996.94
45.0332	28800.07
59.04859	21807.73
81.06878	10596.6
89.05846	28162.75
93.06852	10403.97
95.08403	7067.43
107.08395	10148.92
109.09942	7306.56
133.08458	13218.83

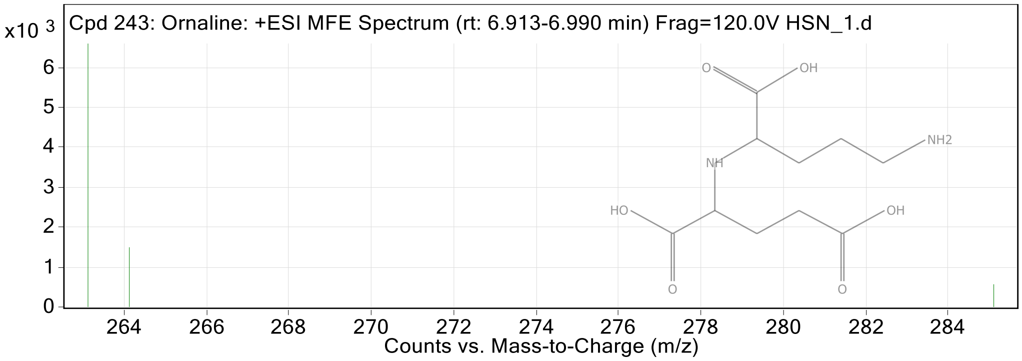

Peak List

m/z	z	Abund	Name	Formula	Ion	Score (DB)	Hits (DB)
263.12498	1	6595	Ornaline	C10 H18 N2 O6	(M+H)+	62.63	1
264.12812	1	1494.01		C10 H18 N2 O6	(M+H)+		
285.10636	1	568.27			(M+Na)+		

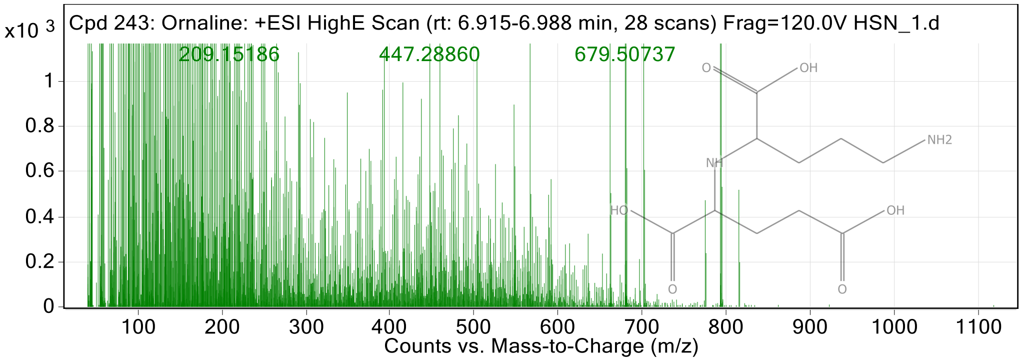

Peak List

m/z	Abund
43.01761	12183.4
45.03321	28727.53
59.04859	18665.12
81.06879	10608.16
89.05849	28118.59
93.06853	10565.26
107.08398	10177.66
109.09942	7245.16
133.08459	13224.21
135.11472	6985.64

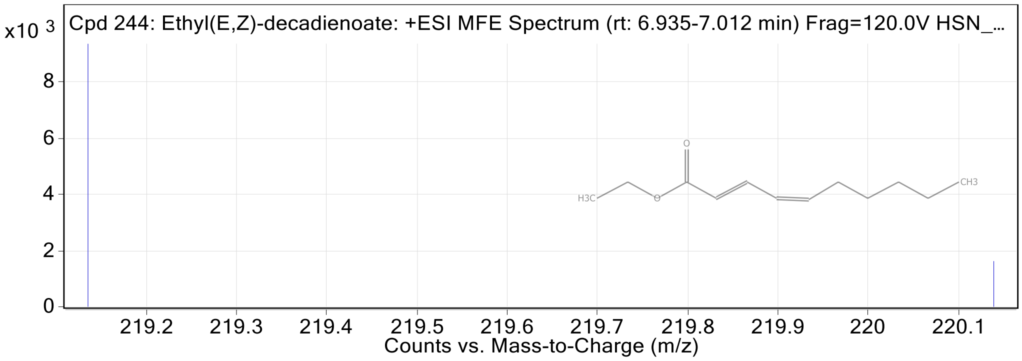

Peak List

m/z	z	Abund	Name	Formula	Ion	Score (DB)	Hits (DB)
219.13542	1	9349.48	Ethyl(E,Z)-decadienoate	C12 H20 O2	(M+Na)+	82.79	10
220.13895	1	1621.96		C12 H20 O2	(M+Na)+		

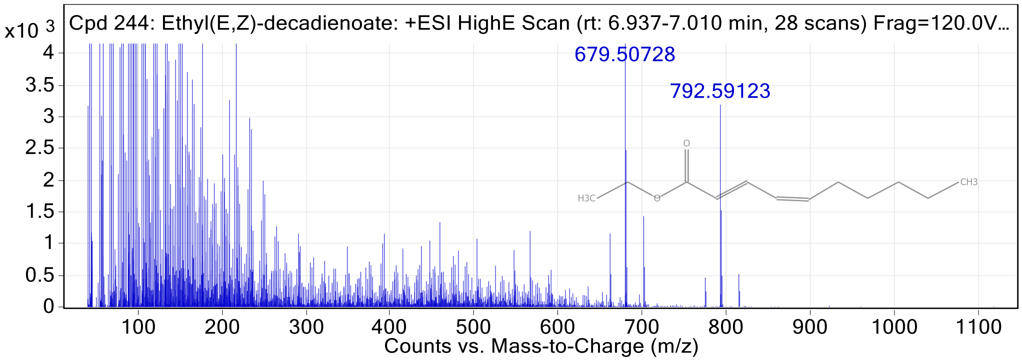

Peak List

m/z	Abund
43.01758	12064.83
45.03321	28539.44
59.04861	12077.69

81.06881	10334.8
89.05848	27624.4
93.06855	10694.28
95.08406	7458.47
107.08401	10359.04
109.09938	7650.94
133.08453	13064.13

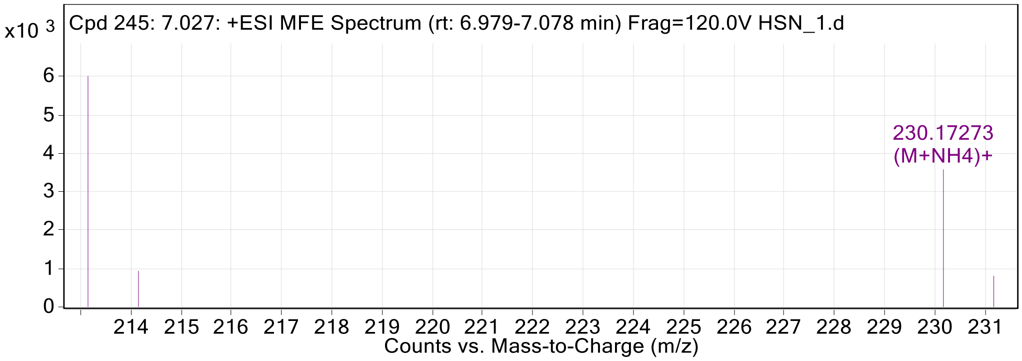

Peak List

m/z	z	Abund	Ion
213.14604	1	6019.31	(M+H)+
214.15055	1	938.85	(M+H)+
230.17273	1	3586.86	(M+NH4)+
231.17527	1	811.99	(M+NH4)+

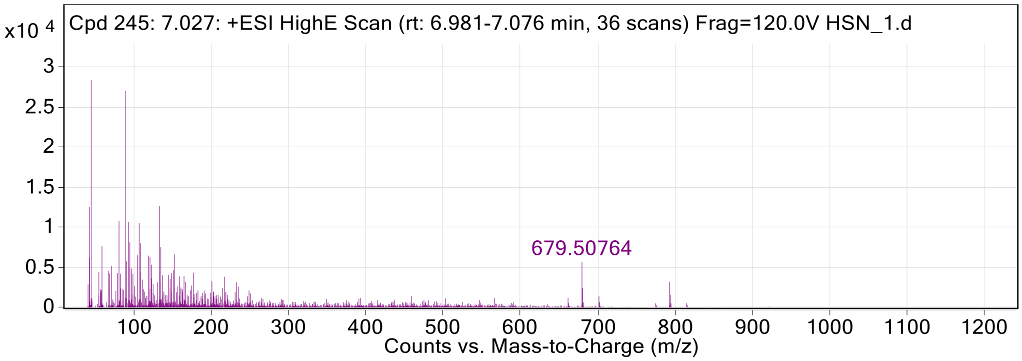

Peak List

m/z	Abund
43.01758	12504.42
45.03322	28341.95
59.0486	7607.45
81.06883	10767.9
89.05844	26962.67
93.06861	10649.39
95.08407	8090.57
107.0839	10475.45
109.09938	7949.59
133.08448	12643.73

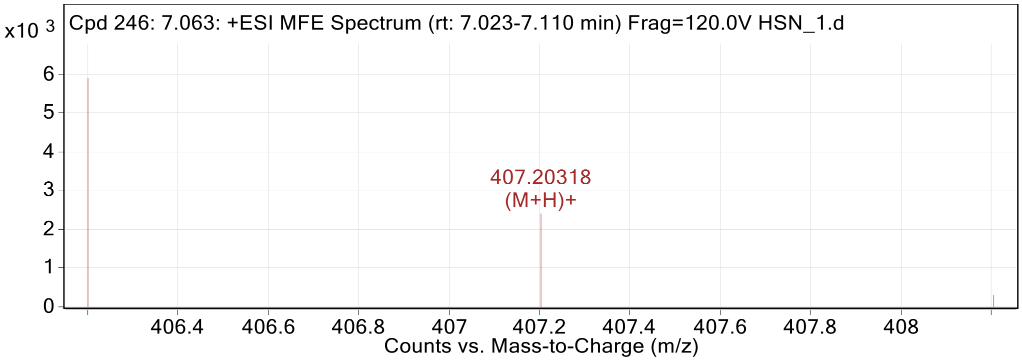

Peak List

m/z	z	Abund	Ion
406.20042	1	5892.69	(M+H)+
407.20318	1	2403.52	(M+H)+
408.20545	1	305.79	(M+H)+

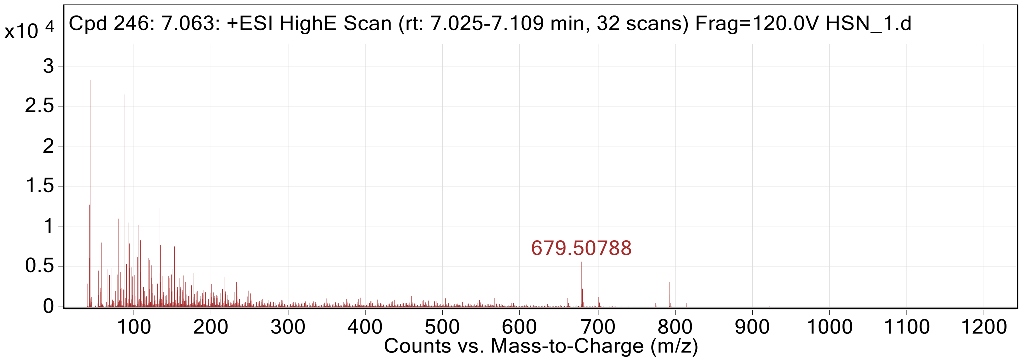

Peak List

m/z	Abund
43.01758	12760.77
45.03319	28295.21
59.04862	8030.33
81.06886	11016.06
89.05847	26521.16
93.06863	10527.31
95.08405	7914.13
107.08386	10215.52
109.09939	8311.87
133.08452	12304.37

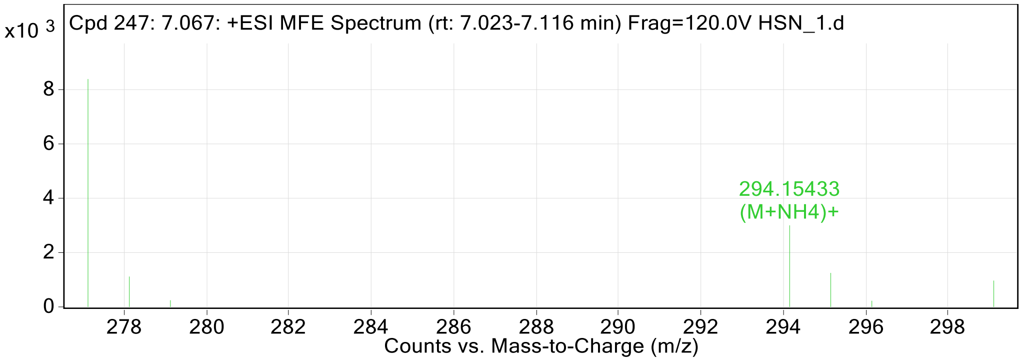

Peak List

m/z	z	Abund	Ion
277.12612	1	8384.7	(M+H)+
278.12955	1	1117.18	(M+H)+

279.12602	1	245.52	(M+H)+
294.15433	1	3000.04	(M+NH4)+
295.15194	1	1249.78	(M+NH4)+
296.14914	1	225.8	(M+NH4)+
299.10814	1	968.66	(M+Na)+

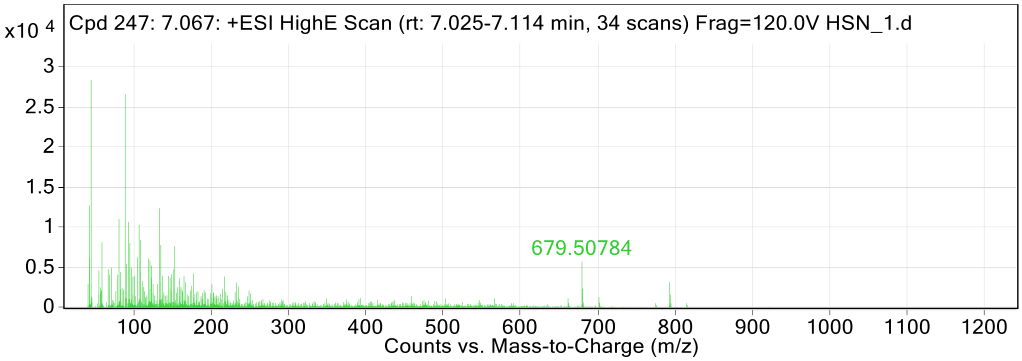

Peak List

m/z	Abund
43.01758	12685.83
45.03318	28370.84
59.04862	8091.04
81.06884	11002.15
89.05847	26597.86
93.06861	10626.56
95.08406	8019.12
107.08385	10252.15
109.0994	8344.91
133.08454	12328.31

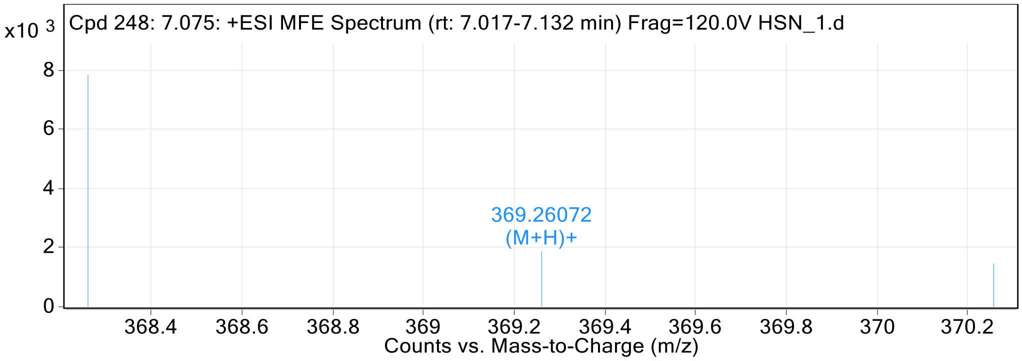

Peak List

m/z	z	Abund	Ion
368.26049	1	7855.19	(M+H)+
369.26072	1	1880.67	(M+H)+
370.25777	1	1472.75	(M+H)+

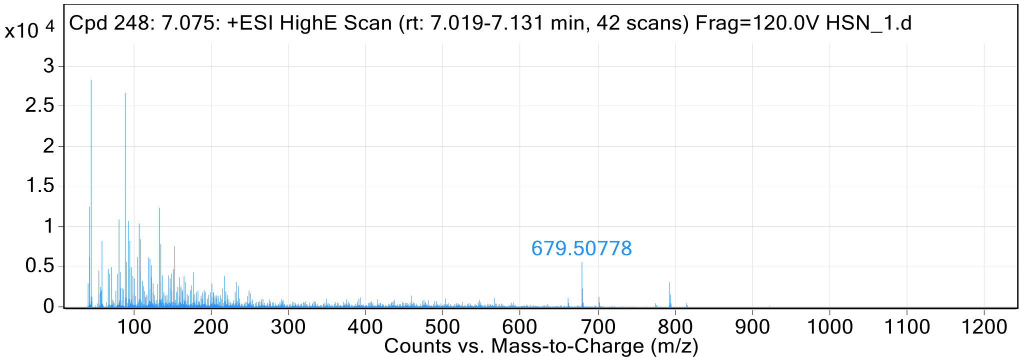

Peak List

m/z	Abund
43.01758	12463.32
45.0332	28266.87
59.04861	8167.75
81.06884	10891.74
89.0585	26623.44
93.06859	10702.5
95.0841	8226.91
107.08385	10387.7
109.0994	8435.52
133.08454	12353.74

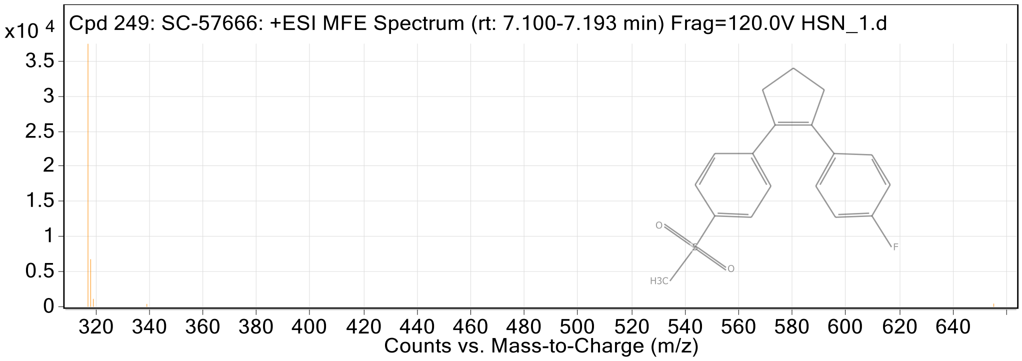

Peak List

m/z	z	Abund	Name	Formula	Ion	Score (DB)	Hits (DB)
317.09921	1	37480.65	SC-57666	C18 H17 F O2 S	(M+H)+	88.48	1
318.10259	1	6775.72		C18 H17 F O2 S	(M+H)+		
319.1054	1	1130.03		C18 H17 F O2 S	(M+H)+		
339.07946	1	406.89			(M+Na)+		
655.1762	1	450.6			(2M+Na)+		

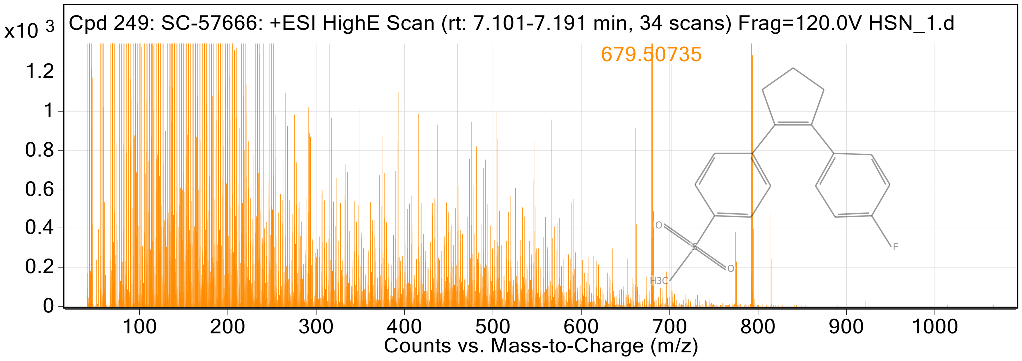

Peak List

m/z	Abund

43.01763	11411.63
45.03318	26898.27
59.0486	14076.77
81.0688	10334.82
89.05851	25435.02
93.06858	11039.39
95.08421	8421.09
107.08389	10504.67
109.09936	8784.26
133.08467	12085.07

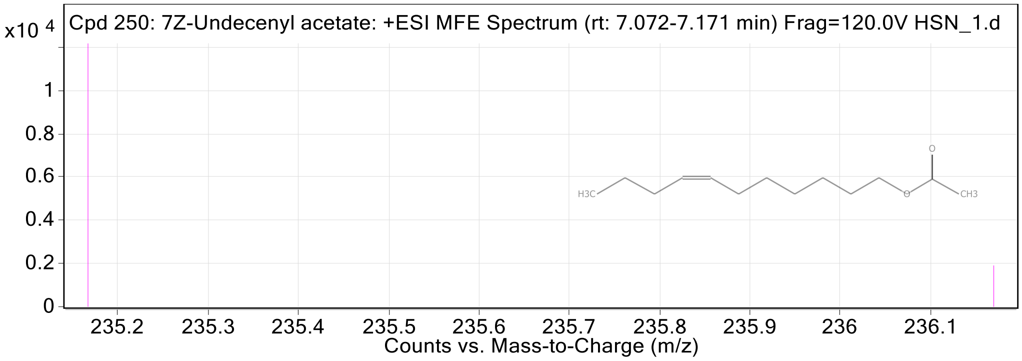

Peak List

m/z	z	Abund	Name	Formula	Ion	Score (DB)	Hits (DB)
235.1669	1	12175.18	7Z-Undecenyl acetate	C13 H24 O2	(M+Na)+	86.75	10
236.1702	1	1908.02		C13 H24 O2	(M+Na)+		

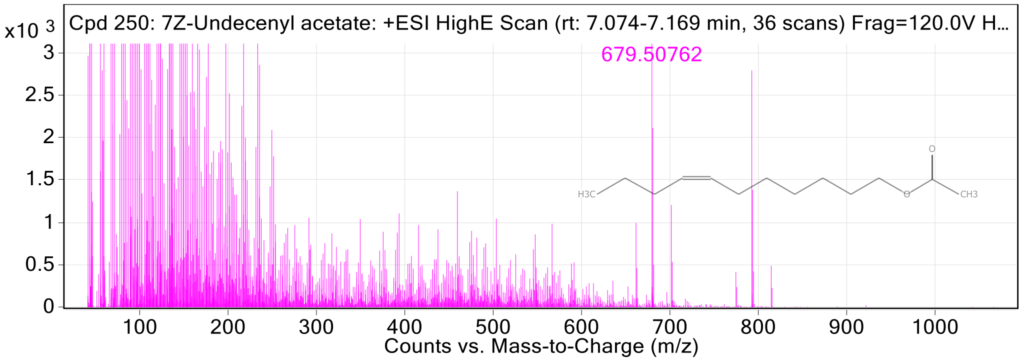

Peak List

m/z	Abund
43.01758	12008.58
45.03318	27531.37
59.04861	10442.2
81.06879	10339.97
89.05852	25882.8
93.0686	10870.12
95.08415	8165.51
107.08388	10500.45
109.09932	8702.61
133.08457	12127.11

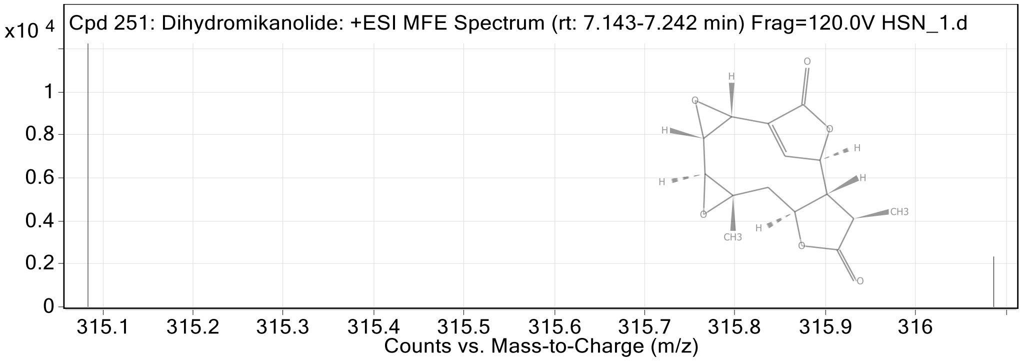

Peak List

m/z	z	Abund	Name	Formula	Ion	Score (DB)	Hits (DB)
315.08361	1	12268.36	Dihydromikanolide	C15 H16 O6	(M+Na)+	82.92	5
316.08648	1	2347.38		C15 H16 O6	(M+Na)+		

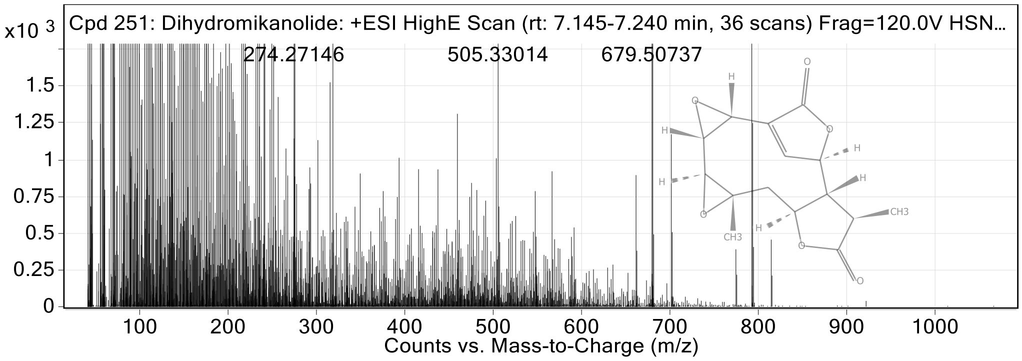

Peak List

m/z	z	Abund
43.01759		10391.84
45.03318		25857.45
59.0486		23379.87
81.06887		10381.09
89.05847		24014.96
93.0686		10566.77
107.08387		9842.65
133.08452		11785.39
240.22965	1	11523.23
274.27146	1	14186.57

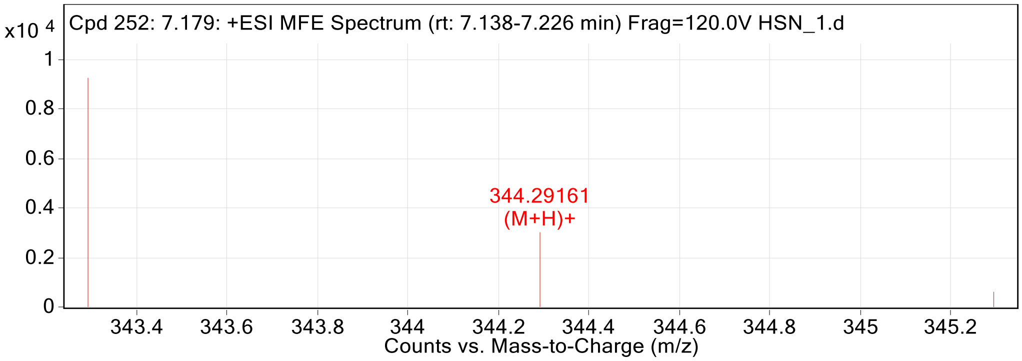

Peak List

m/z	z	Abund	Ion
343.29271	1	9262.41	(M+H)+
344.29161	1	3020.72	(M+H)+
345.29444	1	611.61	(M+H)+

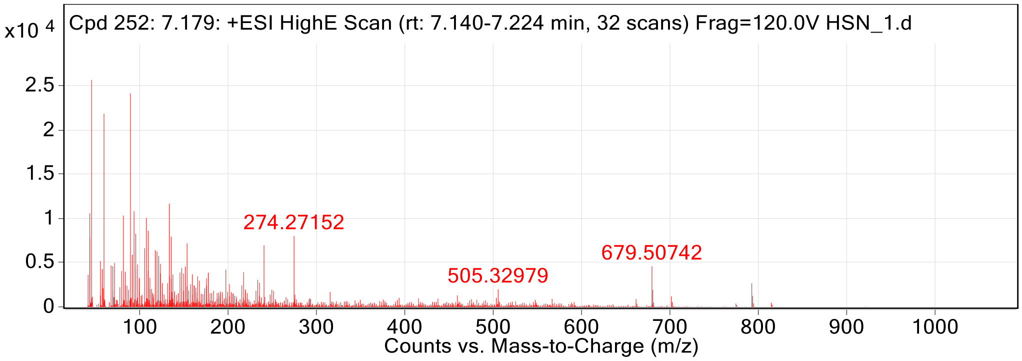

Peak List

m/z	Abund
43.01761	10594.66
45.03317	25661.38
59.04861	21826
81.06886	10325.16
89.05846	24140.01
93.0686	10815.92
95.08414	8280.9
107.08387	10061.1
109.09935	8607.11
133.08459	11663.51

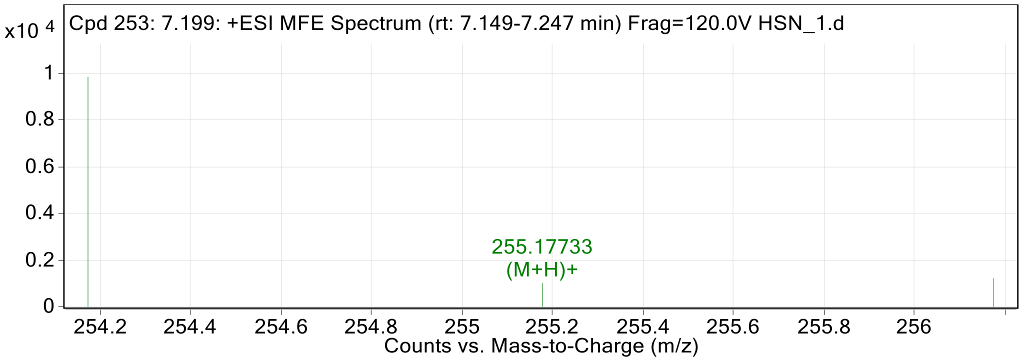

Peak List

m/z	z	Abund	Ion
254.17332	1	9860.73	(M+H)+
255.17733	1	1012.8	(M+H)+
256.17495	1	1224.09	(M+H)+

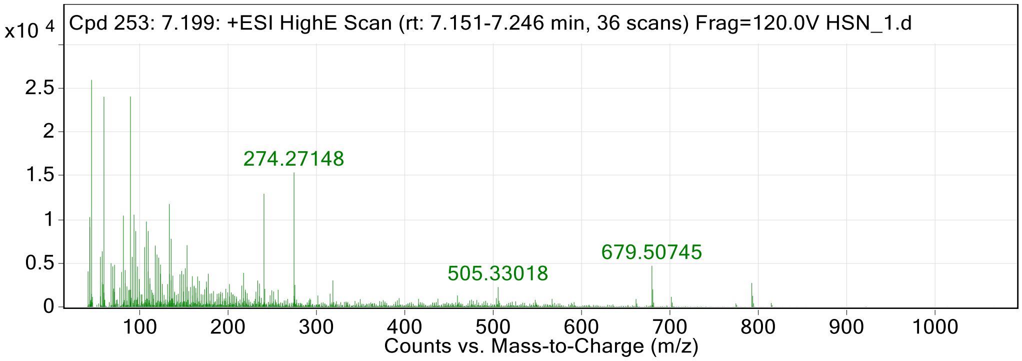

Peak List

m/z	z	Abund
43.0176		10262.62
45.03318		25966.24

59.04861		24040.17
81.06886		10449.39
89.05848		24060.71
93.0686		10549.71
107.08389		9765.1
133.08451		11771.04
240.22965	1	12943.39
274.27148	1	15384.27

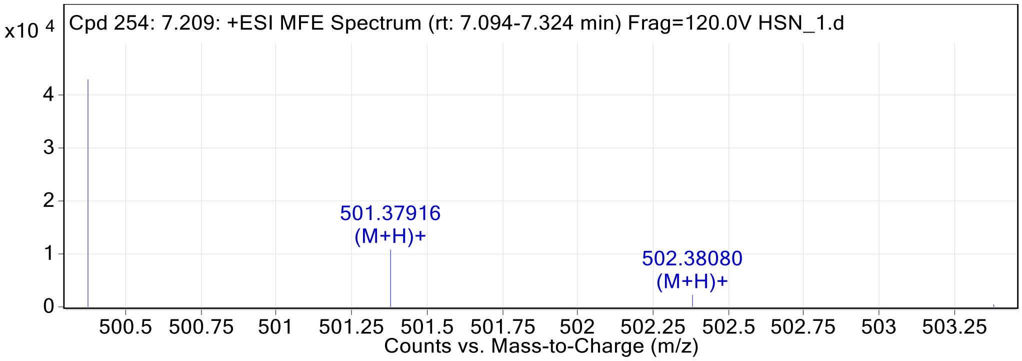

Peak List

m/z	z	Abund	Ion
500.37545	1	43038.72	(M+H)+
501.37916	1	10832.67	(M+H)+
502.3808	1	2266.02	(M+H)+
503.38025	1	468.07	(M+H)+

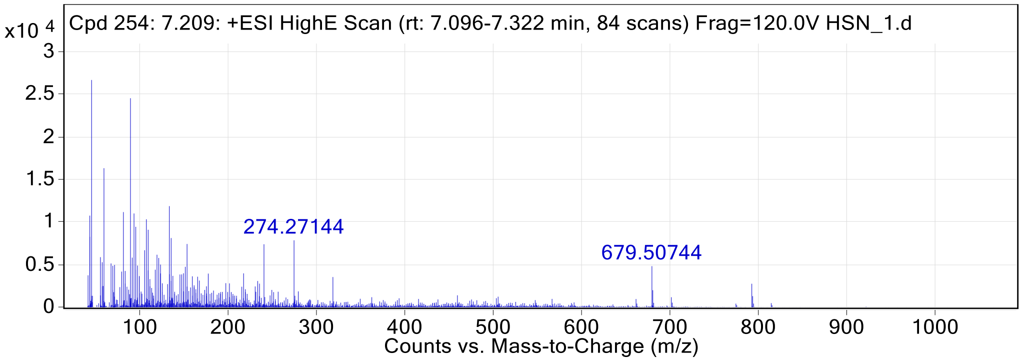

Peak List

m/z	Abund
43.01756	10737.62
45.03318	26673.73
59.0486	16310.55
81.06883	11155.28
89.05851	24532.63
93.06856	10992.24
95.0841	9426.61
107.0839	10306.79
109.09941	9077.44
133.08458	11855.12

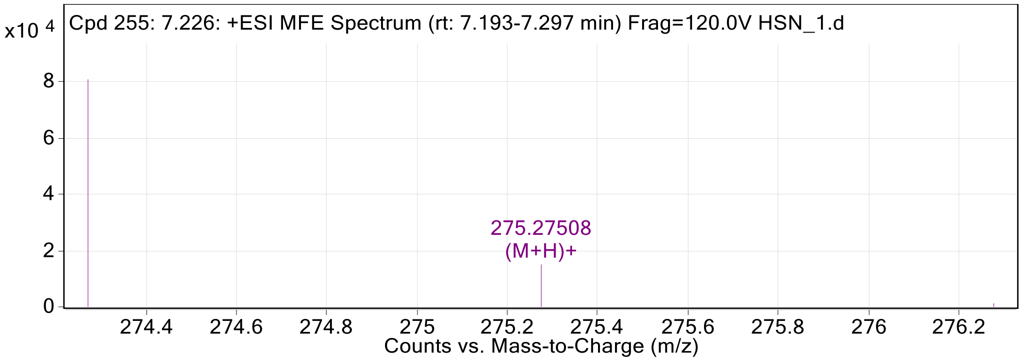

Peak List

m/z	z	Abund	Ion
274.27148	1	80828.48	(M+H)+
275.27508	1	15083.38	(M+H)+
276.27712	1	1270.07	(M+H)+

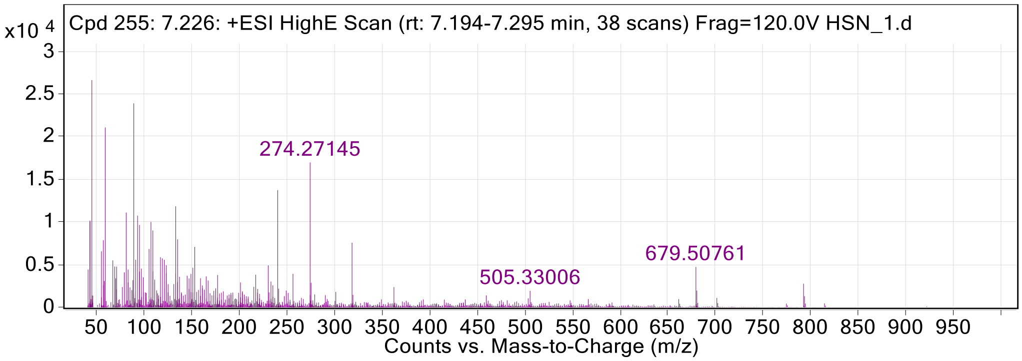

Peak List

m/z	z	Abund
43.01751		10050.16
43.05395		10147.4
45.03318		26640.04
59.04859		21069.99
81.06883		11085.26
89.05852		23900.79
93.06855		10724.43
133.08447		11826.27
240.22962	1	13730.92
274.27145	1	16970.32

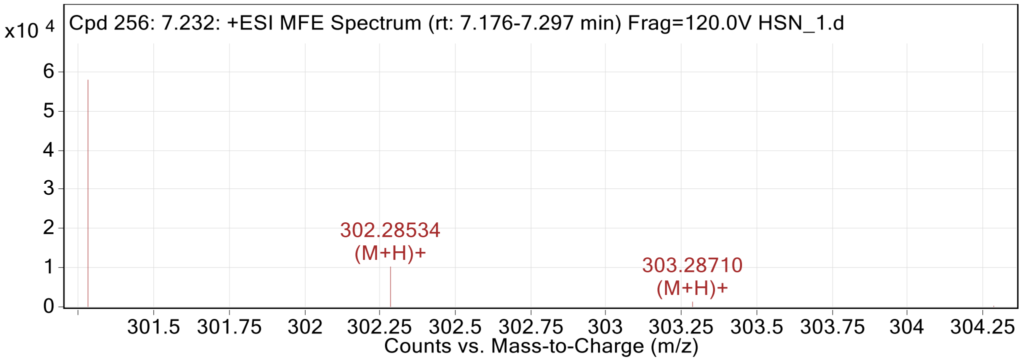

Peak List

m/z	z	Abund	Ion
301.28183	1	58011.66	(M+H)+
302.28534	1	10288.87	(M+H)+

303.2871	1	1331	(M+H)+
304.28607	1	286.52	(M+H)+

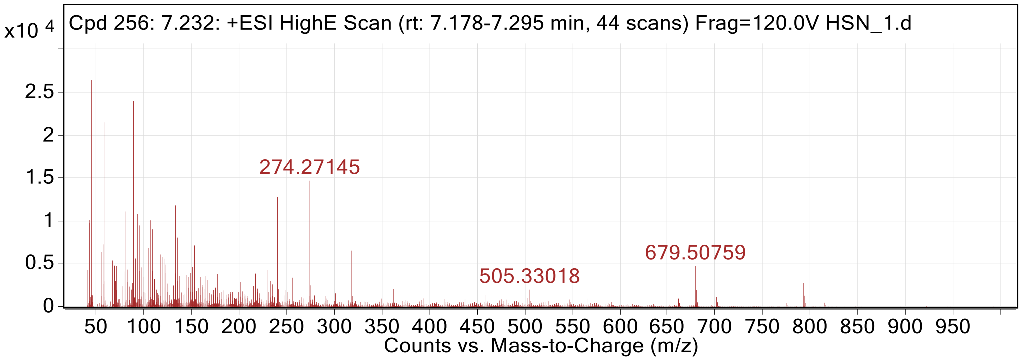

Peak List

m/z	z	Abund
43.01754		10121.82
45.03318		26447.17
59.04859		21485.32
81.06884		11085.41
89.05851		24000.98
93.06855		10779.61
107.08391		10054.4
133.08452		11793.27
240.22961	1	12786.4
274.27145	1	14682.27

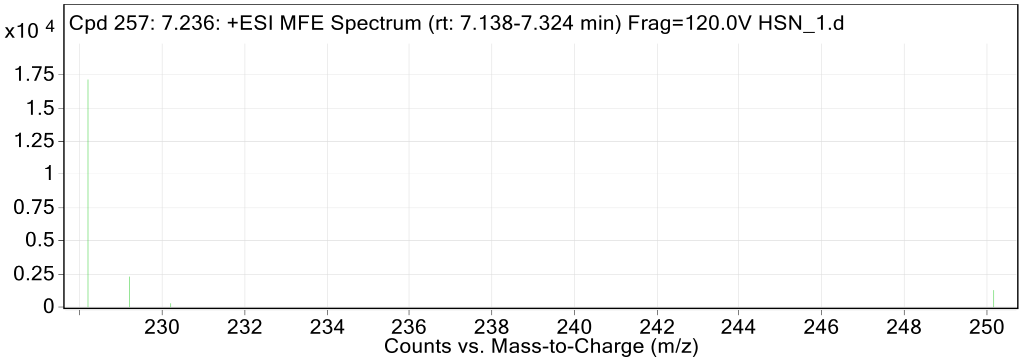

Peak List

m/z	z	Abund	Ion
228.19318	1	17161.72	(M+H)+
229.19572	1	2293.68	(M+H)+
230.20214	1	276.57	(M+H)+
250.17558	1	1275.79	(M+Na)+

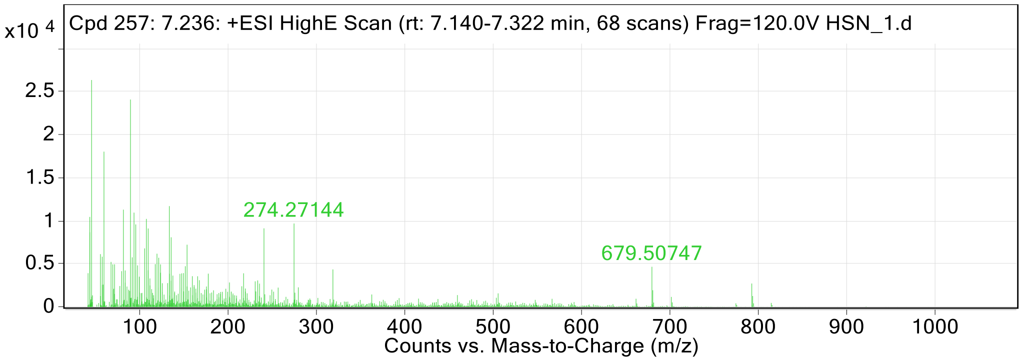

Peak List

m/z	Abund
43.01755	10437.19
45.03318	26346.22
59.0486	18023.64
81.06883	11283.35
89.0585	24079.96
93.06857	10937.12
95.08407	9566.74
107.08391	10209.03
133.08457	11694.42
274.27144	9674.09

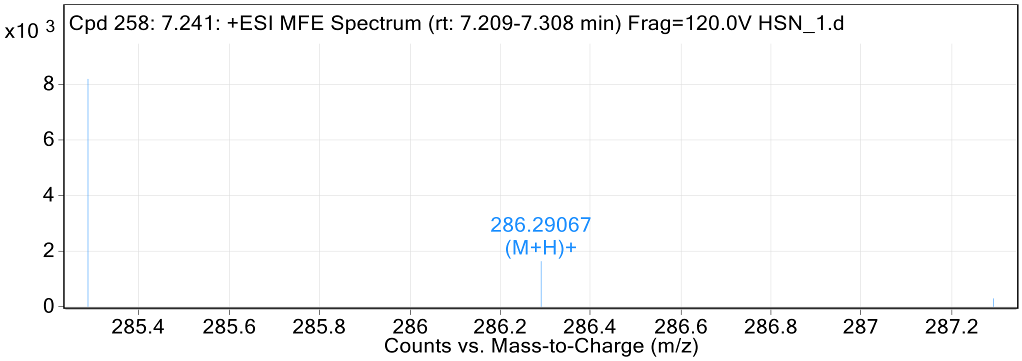

Peak List

m/z	z	Abund	Ion
285.28717	1	8179.22	(M+H)+
286.29067	1	1635.16	(M+H)+
287.2932	1	300.49	(M+H)+

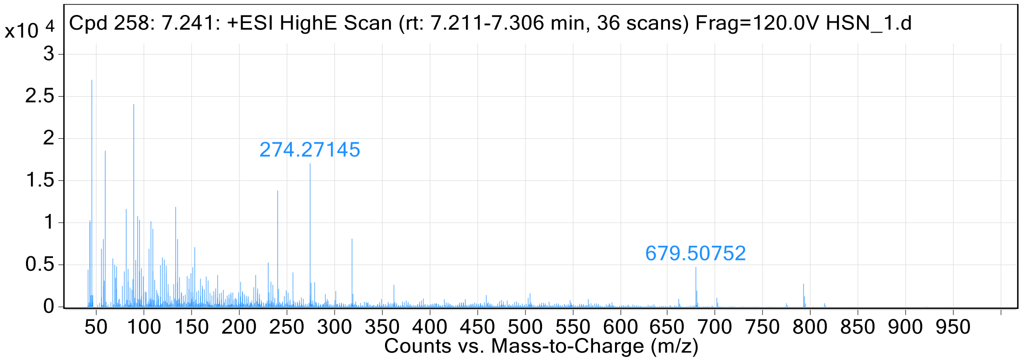

Peak List

m/z	z	Abund
43.05395		10268.43
45.03317		26908.64
59.04858		18511.79
81.06882		11619.95
89.05853		24034.64
93.06854		10771.18
95.08404		10314.16
133.08447		11848.79
240.2296	1	13785.71
274.27145	1	17022.02

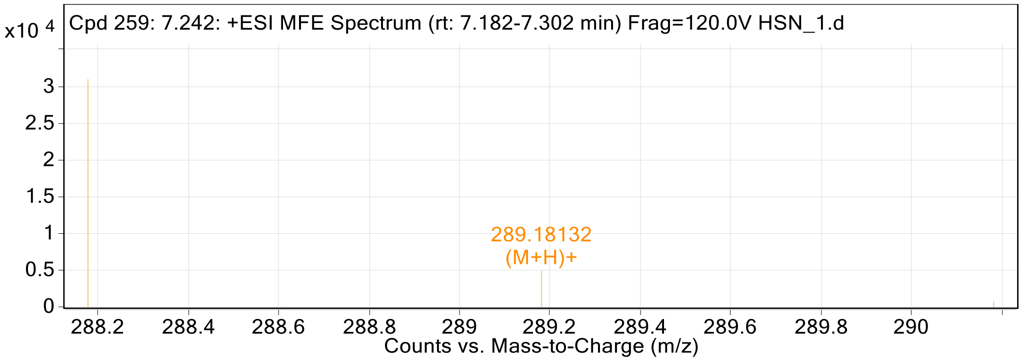

Peak List

m/z	z	Abund	Ion
288.17816	1	30971.72	(M+H)+
289.18132	1	4886.47	(M+H)+
290.18166	1	734.99	(M+H)+

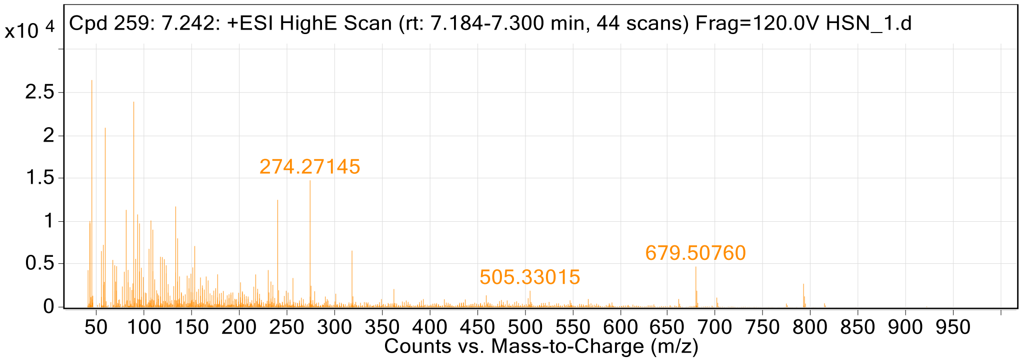

Peak List

m/z	z	Abund
43.01752		10021.68
45.03318		26460.32
59.04859		20898.88
81.06884		11329.55
89.05851		23940.84
93.06853		10795.46
107.08391		10087.5
133.08451		11723.85
240.22962	1	12497.16
274.27145	1	14776.45

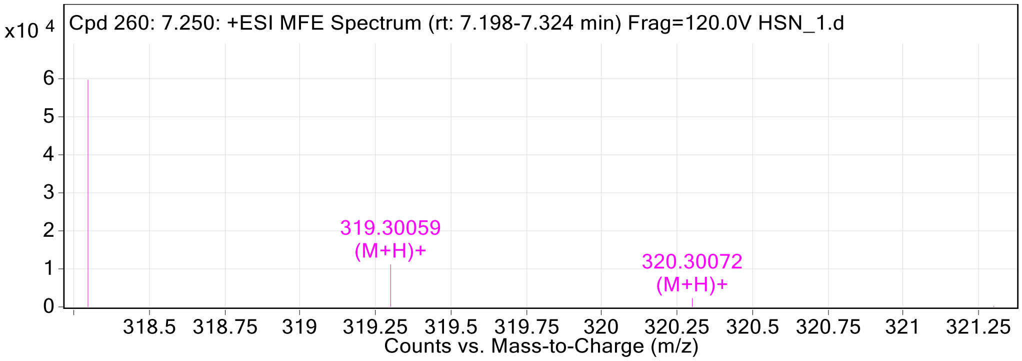

Peak List

m/z	z	Abund	Ion
318.29716	1	59902.47	(M+H)+
319.30059	1	11230.93	(M+H)+

320.30072	1	2335.17	(M+H)+
321.30032	1	236.44	(M+H)+

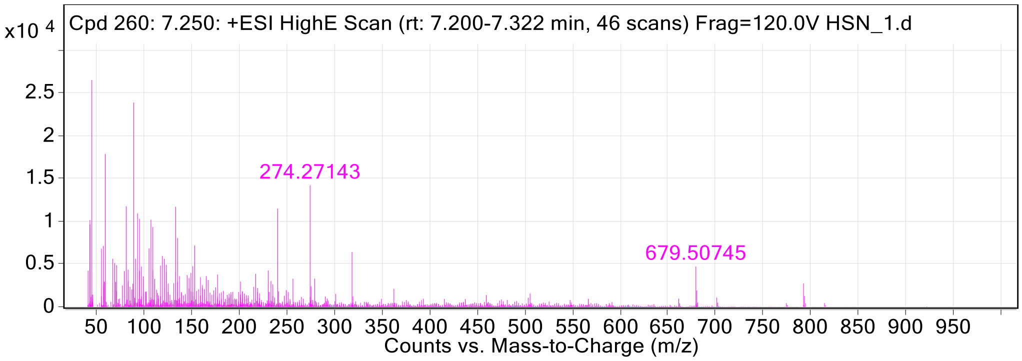

Peak List

m/z	z	Abund
45.03318		26506.37
59.0486		17871.21
81.06883		11751.24
89.0585		23877.15
93.06855		10930.82
95.08404		10282.24
107.08392		10184.83
133.08452		11695.28
240.2296	1	11475.17
274.27143	1	14196.25

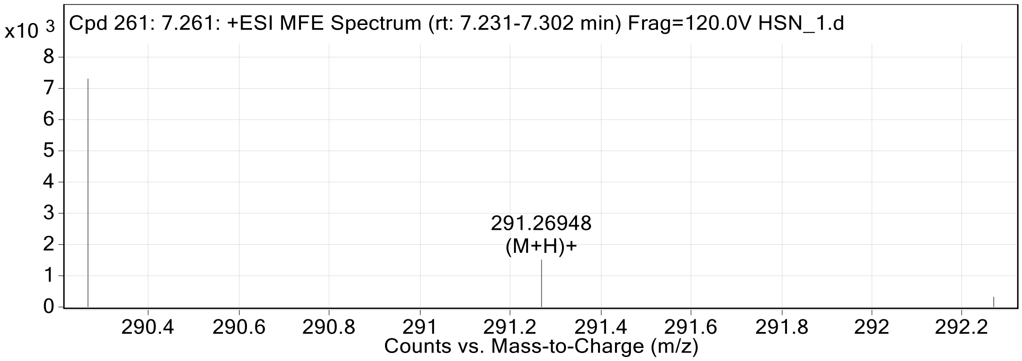

Peak List

m/z	z	Abund	Ion
290.26659	1	7322.69	(M+H)+
291.26948	1	1519.63	(M+H)+
292.26941	1	327.38	(M+H)+

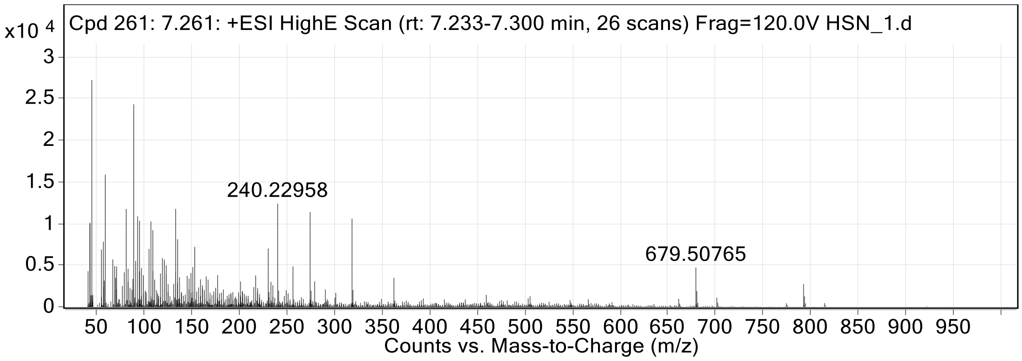

Peak List

m/z	z	Abund

45.03318		27208.54
59.04857		15862.39
81.06878		11740.22
89.05853		24306.92
93.06851		10864.94
95.08401		10329.27
133.08453		11738.93
240.22958	1	12380.42
274.27143	1	11366.66
318.29732	1	10586.17

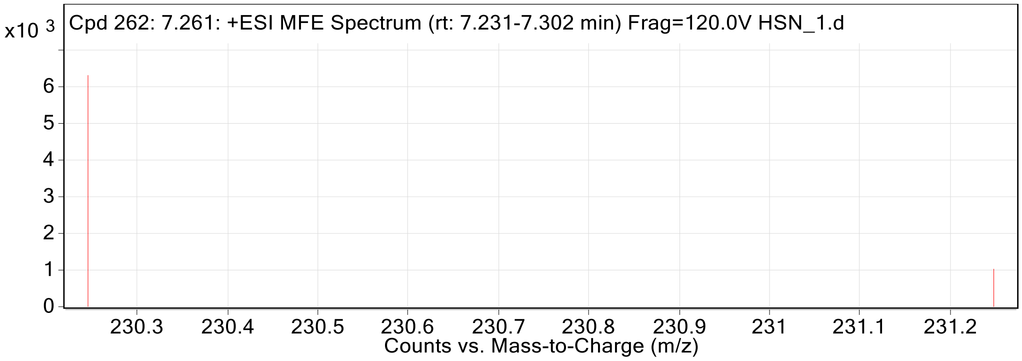

Peak List

m/z	z	Abund	Ion
230.24532	1	6320.55	(M+H)+
231.24851	1	1034.62	(M+H)+

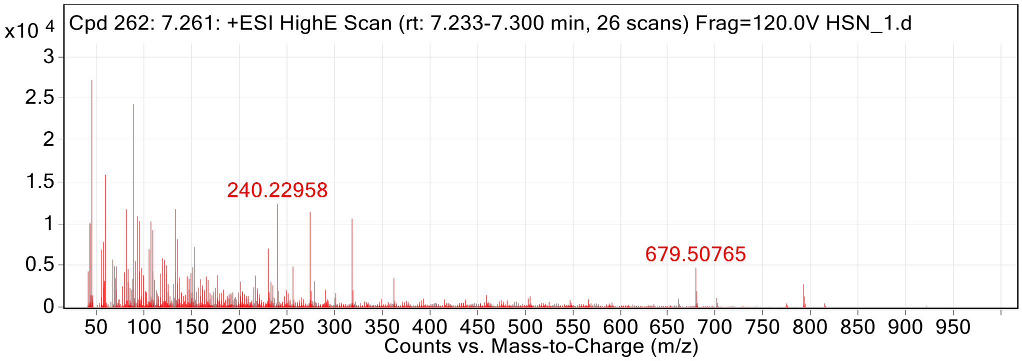

Peak List

m/z	z	Abund
45.03318		27208.54
59.04857		15862.39
81.06878		11740.22
89.05853		24306.92
93.06851		10864.94
95.08401		10329.27
133.08453		11738.93
240.22958	1	12380.42
274.27143	1	11366.66
318.29732	1	10586.17

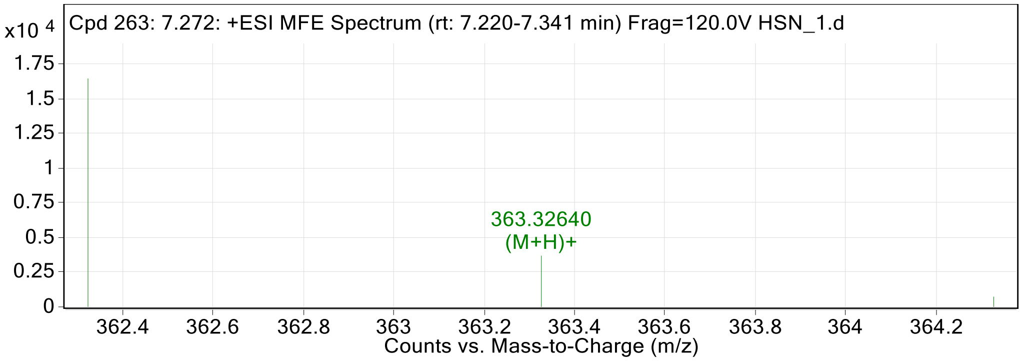

Peak List

m/z	z	Abund	Ion
362.3231	1	16478.82	(M+H)+
363.3264	1	3696.94	(M+H)+
364.32769	1	735.1	(M+H)+

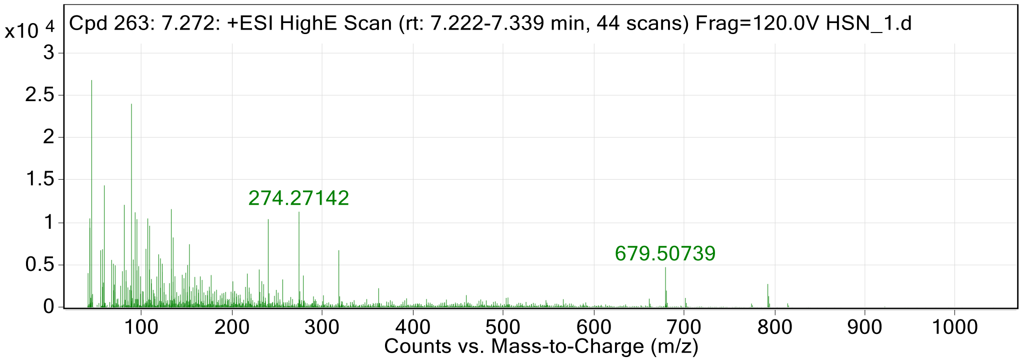

Peak List

m/z	z	Abund
43.01752		10469.59

Qualitative Analysis Report

m/z	z	Abund
45.03319		26807.86
59.04857		14370.71
81.06881		12071.55
89.05852		24006.78
93.06853		11186.82
107.08393		10463.27
133.08457		11568.31
240.22959		10381.76
274.27142	1	11261.29

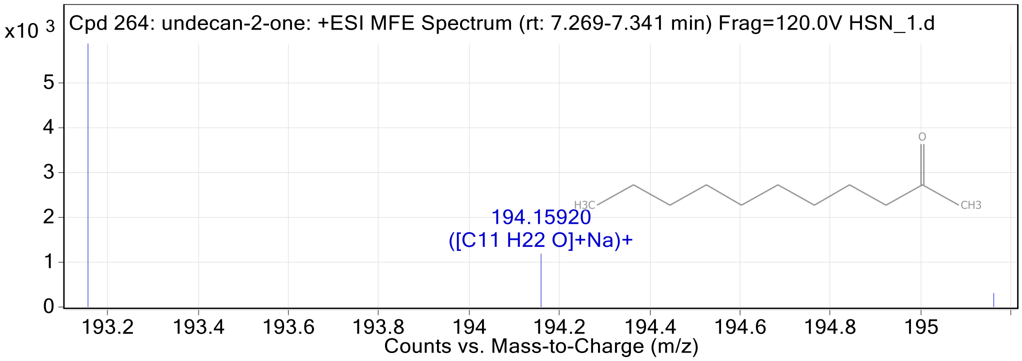

Peak List

m/z	z	Abund	Name	Formula	Ion	Score (DB)	Hits (DB)
193.15627	1	5886.87	undecan-2-one	C11 H22 O	(M+Na)+	84.37	10
194.1592	1	1193.71		C11 H22 O	(M+Na)+		
195.16135	1	311.23		C11 H22 O	(M+Na)+		

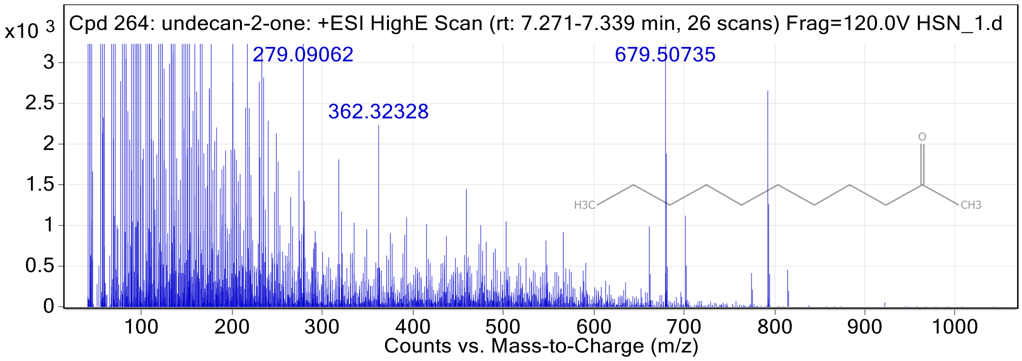

Peak List

m/z	Abund
43.01753	11100.06
45.03319	26417.93
81.0688	13007.27
89.0585	23861.63
93.06852	11995.16
95.08398	10954.94
107.08395	11161.03
109.09941	10063.47
133.08474	11093.15
135.11457	8700.31

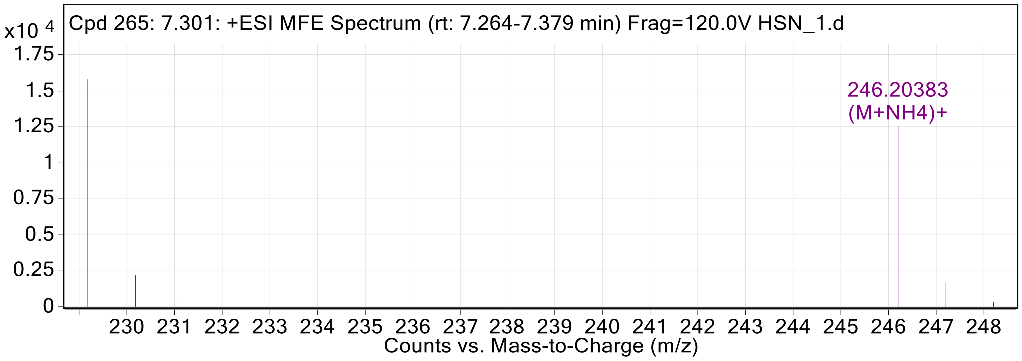

Peak List

m/z	z	Abund	Ion
229.17738	1	15769.41	(M+H)+
230.18033	1	2189.13	(M+H)+
231.17871	1	565.59	(M+H)+
246.20383	1	12522.63	(M+NH4)+
247.20747	1	1727.94	(M+NH4)+
248.2084	1	328.39	(M+NH4)+

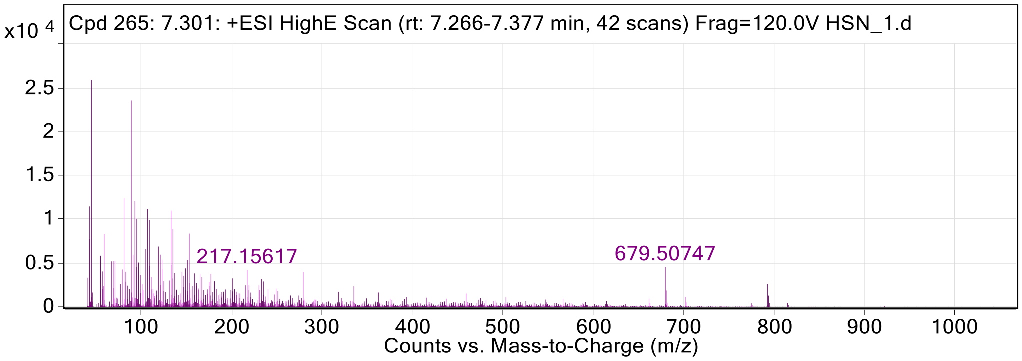

Peak List

m/z	Abund
43.01752	11451.16
45.03319	25878.6
81.06882	12381.36
89.0585	23541.4
93.06854	12056.71
95.08401	10044.54
107.0839	11180.55
109.09943	9876.15
133.08468	10975.07
135.11465	8885.23

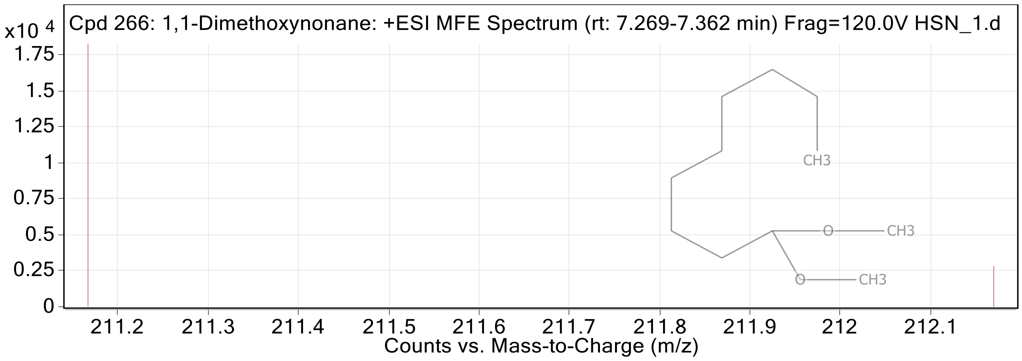

Peak List

m/z	z	Abund	Name	Formula	Ion	Score (DB)	Hits (DB)
211.167	1	18286.25	1,1-Dimethoxynonane	C11 H24 O2	(M+Na)+	83.87	2
212.16991	1	2834.92		C11 H24 O2	(M+Na)+		

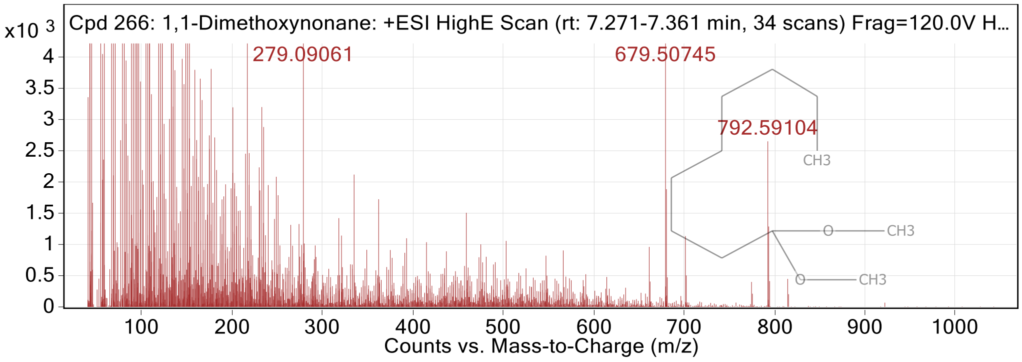

Peak List

m/z	Abund
43.01754	11394.81
45.03317	26050.22
81.06881	12673.96
89.05849	23837.57
93.06853	12026.08
95.084	10406.71
107.0839	11244.95
109.09943	9952.31
133.08469	11056.32
135.11463	8914.74

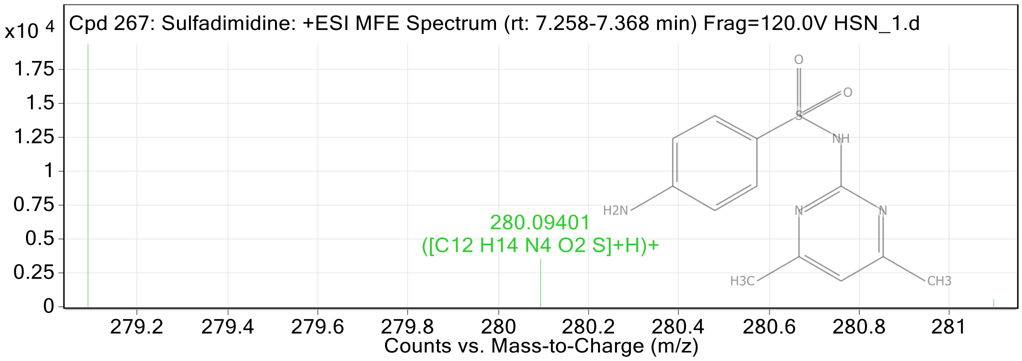

Peak List

m/z	z	Abund	Name	Formula	Ion	Score (DB)	Hits (DB)
279.09071	1	19388.77	Sulfadimidine	C12 H14 N4 O2 S	(M+H)+	89.67	2
280.09401	1	3536.17		C12 H14 N4 O2 S	(M+H)+		
281.09819	1	562.11		C12 H14 N4 O2 S	(M+H)+		

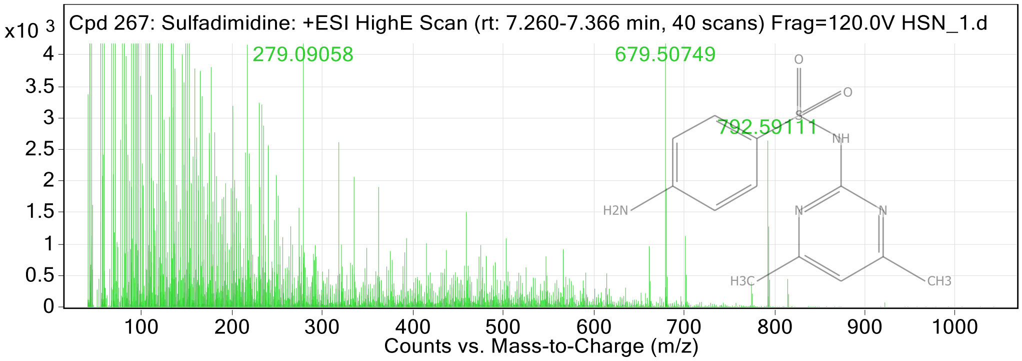

Peak List

m/z	Abund
43.01753	11304.66
45.03319	26222.41
81.06881	12447.55
89.0585	23869.74
93.06853	12007.99
95.08399	10168.11
107.08389	11140.65
109.09943	9826.27
133.08472	11069.2
135.11463	8855.42

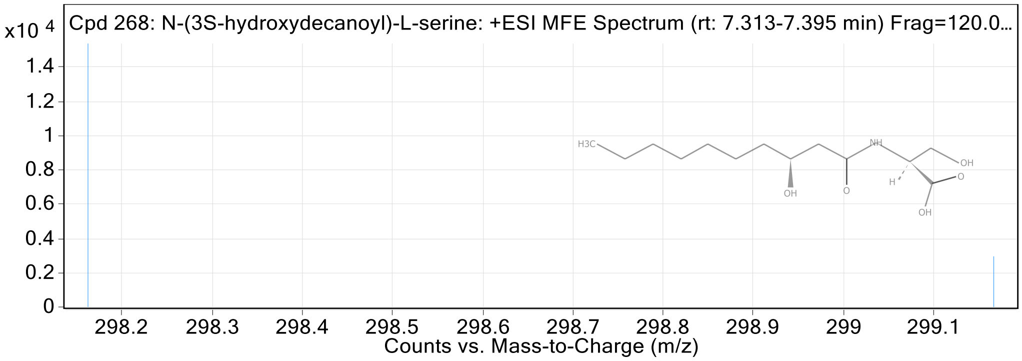

Peak List

m/z	z	Abund	Name	Formula	Ion	Score (DB)	Hits (DB)
298.16233	1	15369.87	N-(3S-hydroxydecanoyl)-L-serine	C13 H25 N O5	(M+Na)+	80.11	1
299.16664	1	2952.12		C13 H25 N O5	(M+Na)+		

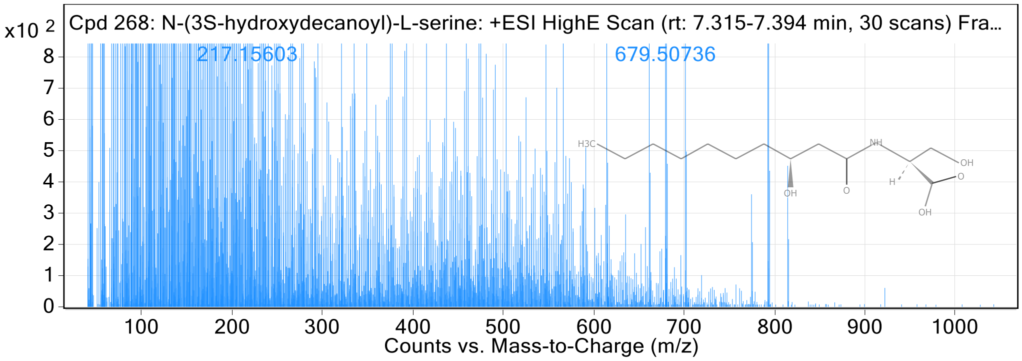

Peak List

m/z	Abund
43.01756	12175.33
45.03319	24772.29
81.06885	11940.07
89.05846	23200.82
93.06854	12528.52
107.0838	11501.28
109.09942	9761.65
133.08467	10344.5
135.11461	9081.38
153.12495	8943.92

Qualitative Analysis Report

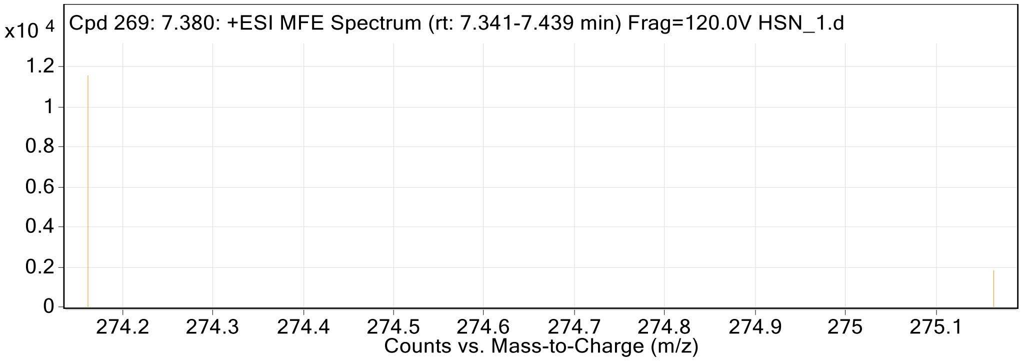

Peak List

m/z	z	Abund	Ion
274.16188	1	11585.6	(M+H)+
275.16415	1	1838.03	(M+H)+

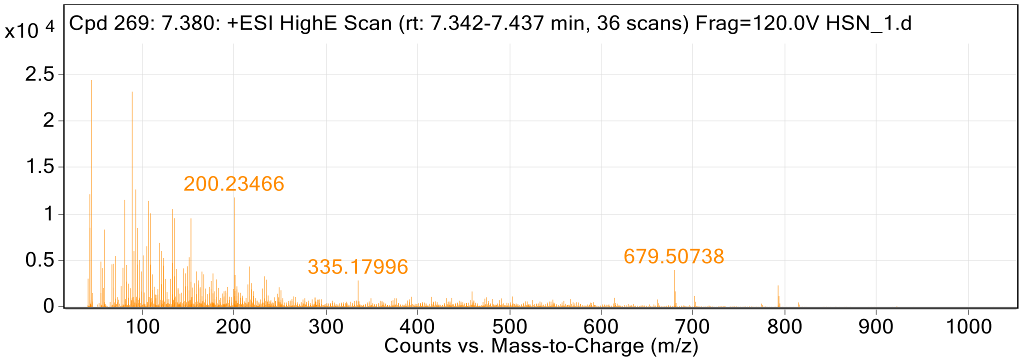

Peak List

m/z	z	Abund
43.01753		12122.43
45.03319		24428.65
81.06886		11508.86
89.05846		23167.61
93.06854		12633
107.08384		11402.42
109.09944		10067.65
133.08466		10519.22
135.11466		9539.76
200.23466	1	11774.7

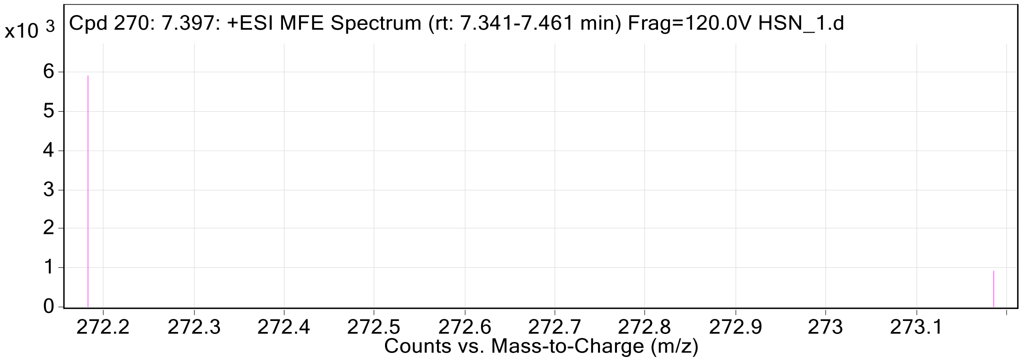

Peak List

m/z	z	Abund	Ion
272.18292	1	5920.72	(M+H)+
273.18596	1	925.34	(M+H)+

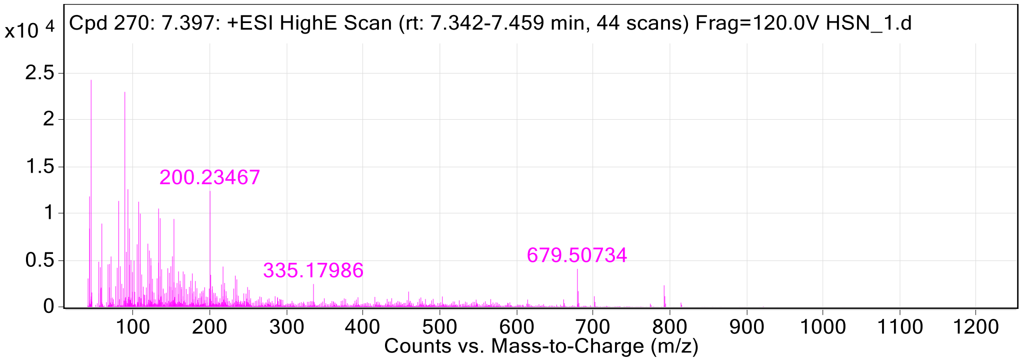

Peak List

m/z	z	Abund
43.01753		11828.77
45.0332		24314.59
81.06883		11344.1
89.05847		23025.83
93.06854		12601.08
107.08383		11265.03
109.09944		9982.84
133.08464		10531.51
135.11467		9497.68
200.23467	1	12416.46

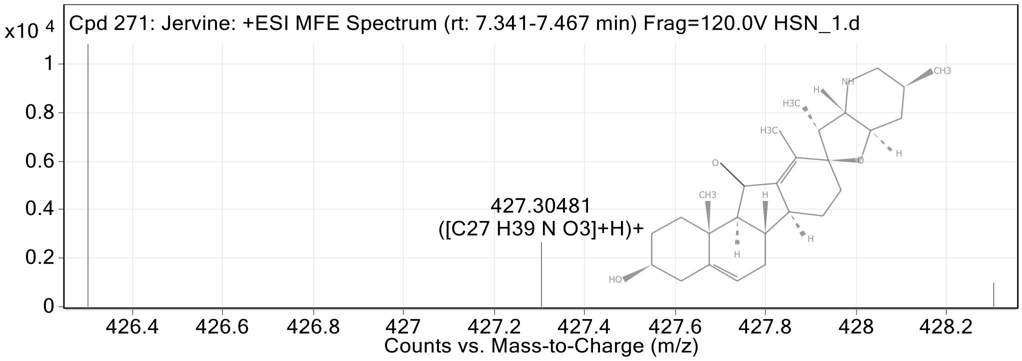

Peak List

m/z	z	Abund	Name	Formula	Ion	Score (DB)	Hits (DB)
426.30229	1	10843.95	Jervine	C27 H39 N O3	(M+H)+	79.41	1
427.30481	1	2663.23		C27 H39 N O3	(M+H)+		
428.30496	1	980.34		C27 H39 N O3	(M+H)+		

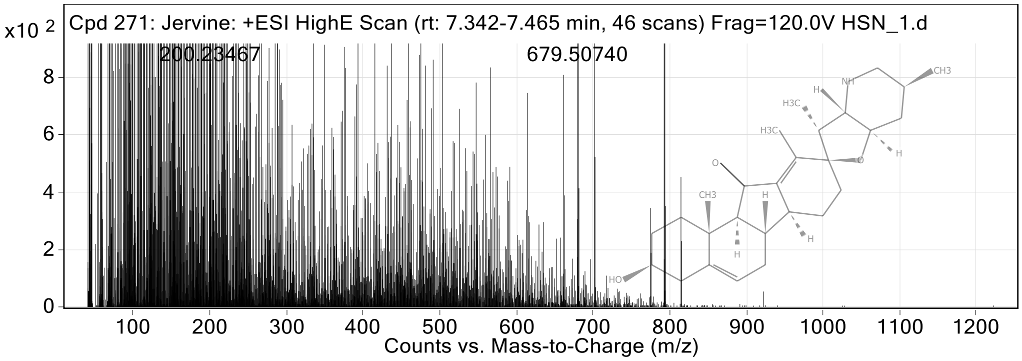

Peak List

m/z	z	Abund
43.01752		11769.57
45.0332		24346.42

81.06883		11333.83
89.05847		23030.61
93.06854		12639.62
107.08383		11284.31
109.09944		9952.97
133.08463		10558.2
135.11466		9556.56
200.23467	1	12149.16

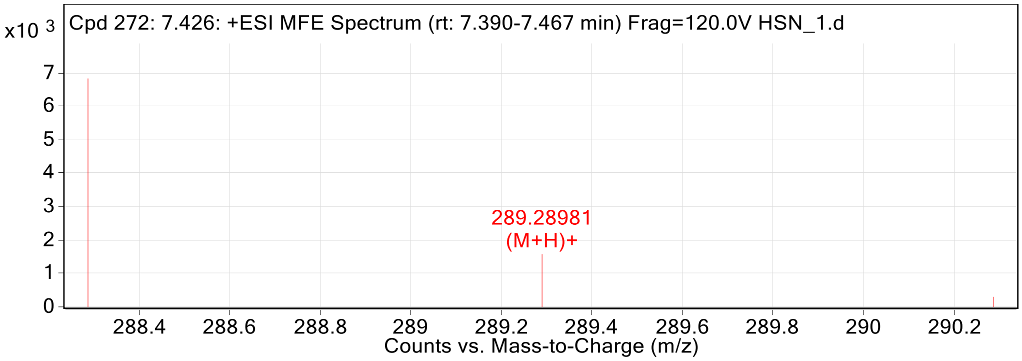

Peak List

m/z	z	Abund	Ion
288.28679	1	6826.98	(M+H)+
289.28981	1	1576.18	(M+H)+
290.2876	1	300.93	(M+H)+

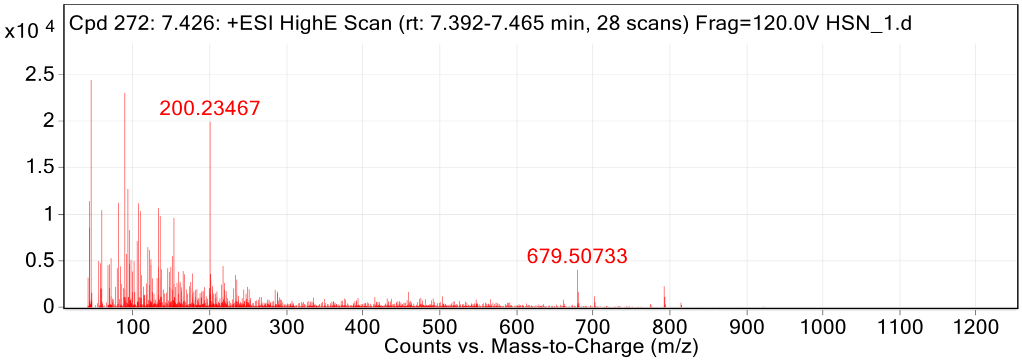

Peak List

m/z	z	Abund
43.01751		11377.29
45.0332		24422.2
59.04863		10417.3
81.06881		11188.97
89.05847		23053.1
93.06853		12730.24
107.08387		11152.45
109.09943		10296.36
133.08471		10626.98
200.23467	1	19927.09

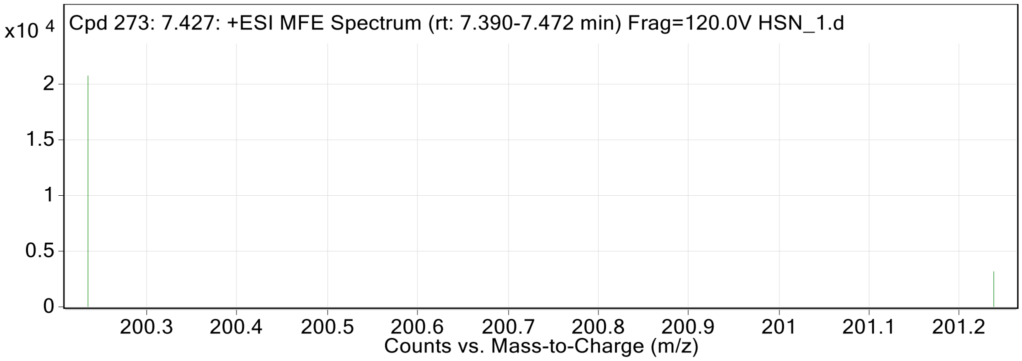

Peak List

m/z	z	Abund	Ion
200.23479	1	20804.6	(M+H)+
201.23824	1	3187.6	(M+H)+

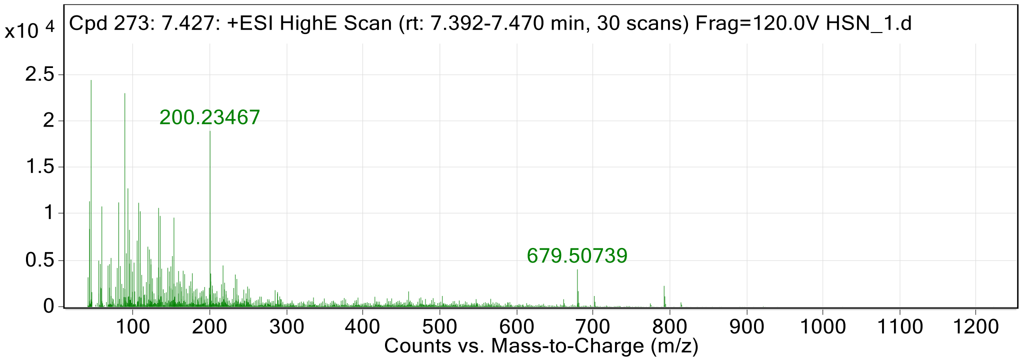

Peak List

m/z	z	Abund
43.01752		11335.26
45.0332		24373.76
59.04863		10780.52
81.0688		11213.07
89.05846		22956.3
93.06853		12726.56
107.08387		11171.96
109.09941		10261.93
133.08468		10619.82
200.23467	1	18902.11

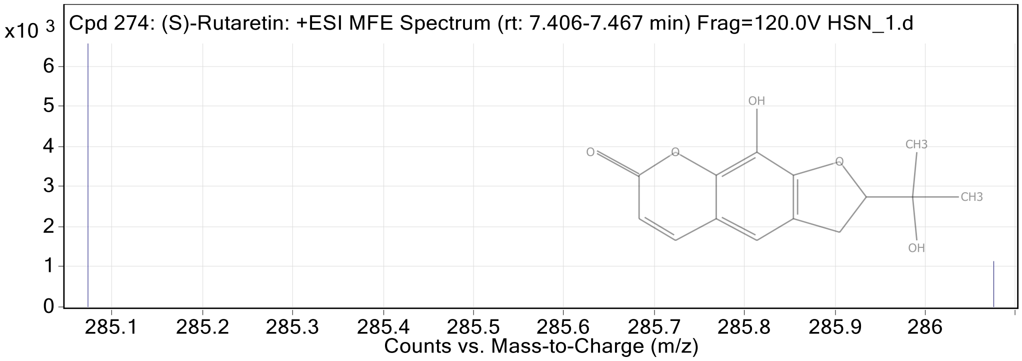

Peak List

m/z	z	Abund	Name	Formula	Ion	Score (DB)	Hits (DB)
285.07333	1	6564.88	(S)-Rutaretin	C14 H14 O5	(M+Na)+	83.7	3
286.07575	1	1142.25		C14 H14 O5	(M+Na)+		

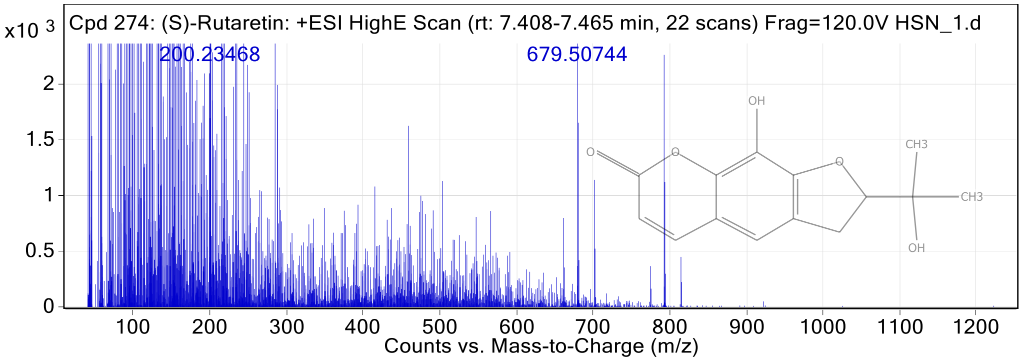

Peak List

m/z	z	Abund
43.01751		10812.7
45.03322		24265.49
59.04869		10926.98
81.06879		10874.1
89.05849		22854.59
93.06853		12444.27
107.08386		10868.54
109.09947		10159.19
133.08467		10730.76
200.23468	1	24328.46

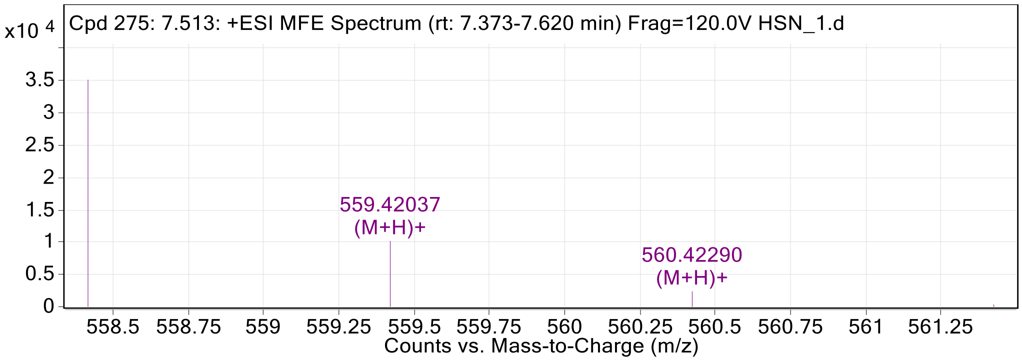

Peak List

m/z	z	Abund	Ion
558.41698	1	35120.4	(M+H)+
559.42037	1	10179.54	(M+H)+
560.4229	1	2395.7	(M+H)+
561.42408	1	346.27	(M+H)+

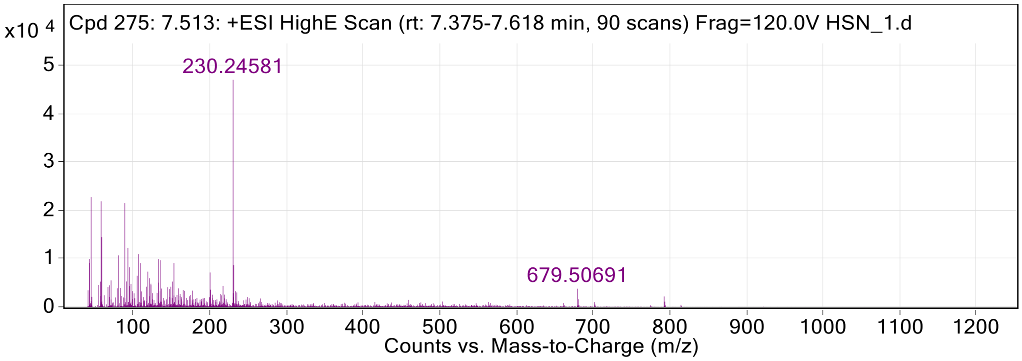

Peak List

m/z	z	Abund
43.01751		9920.2

45.03316		22699.24
58.06461		21844.02
59.04857		14426.18
81.06876		10647.76
89.05844		21480.09
93.0685		12228.06
107.0838		10919.71
133.08457		9890.78
230.24581	1	47016.37

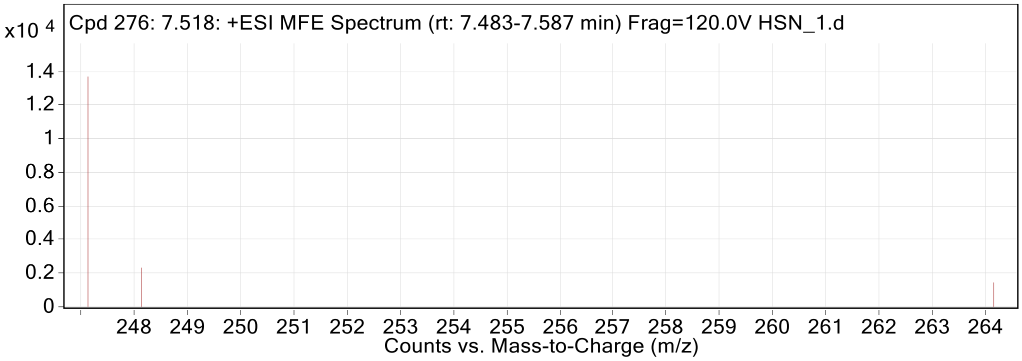

Peak List

m/z	z	Abund	Ion
247.13014	1	13687.06	(M+H)+
248.13351	1	2327.04	(M+H)+
264.15597	1	1443.98	(M+NH4)+

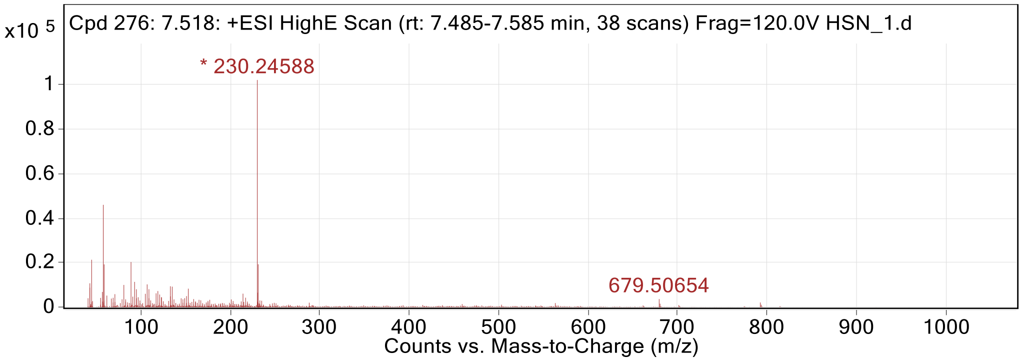

Peak List

m/z	z	Abund
43.0539		10666.72
45.03313		21262.21
58.06461	1	45951.57
59.04854		19172.55
81.06874		9930.26
89.05844		20233.56
93.06847		11308.53
107.0838		10175.04
230.24588	1	102097.58
231.24843	1	19214.38

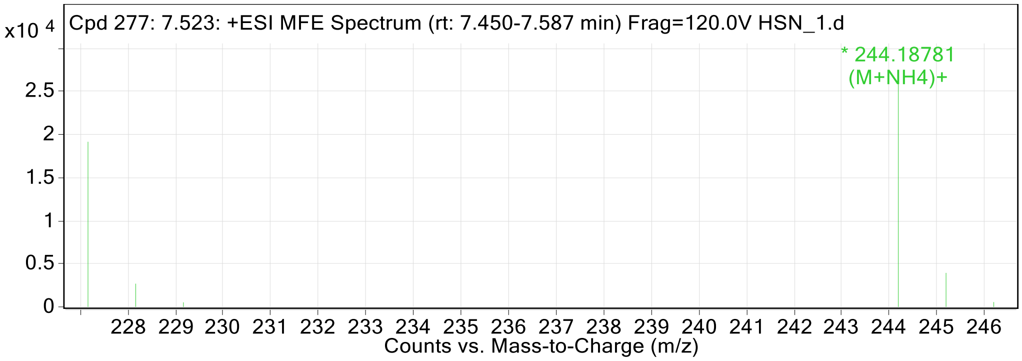

Peak List

m/z	z	Abund	Ion
227.16141	1	19146.33	(M+H)+
228.16463	1	2689.99	(M+H)+
229.16657	1	526	(M+H)+
244.18781	1	26400.9	(M+NH4)+
245.19127	1	3942.79	(M+NH4)+
246.1943	1	559.4	(M+NH4)+

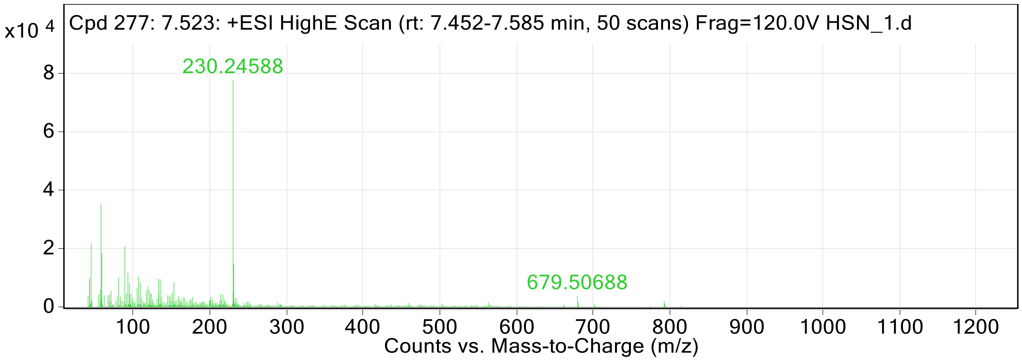

Peak List

m/z	z	Abund
43.0539		9832.94
45.03314		21838.96
58.06461	1	35471.16
59.04855		18435.62
81.06875		10165.91
89.05843		20780.68
93.06848		11709.62
107.08381		10422.8
230.24588	1	77695.41
231.24843	1	14616.58

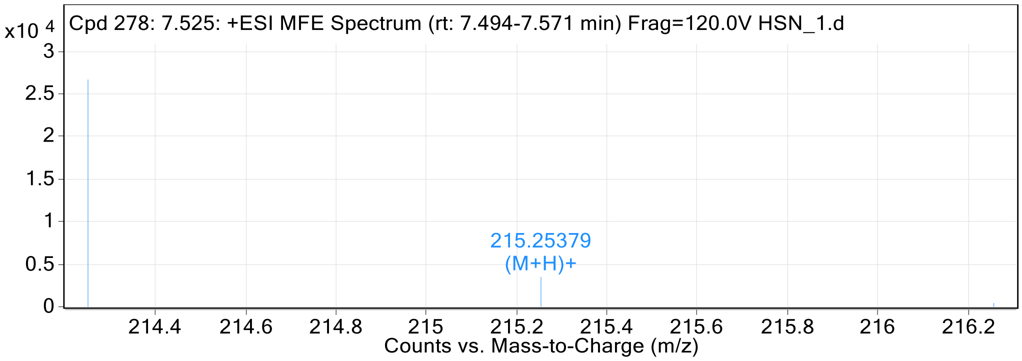

Peak List

m/z	z	Abund	Ion
214.25036	1	26696.48	(M+H)+
215.25379	1	3499.65	(M+H)+
216.25681	1	440.74	(M+H)+

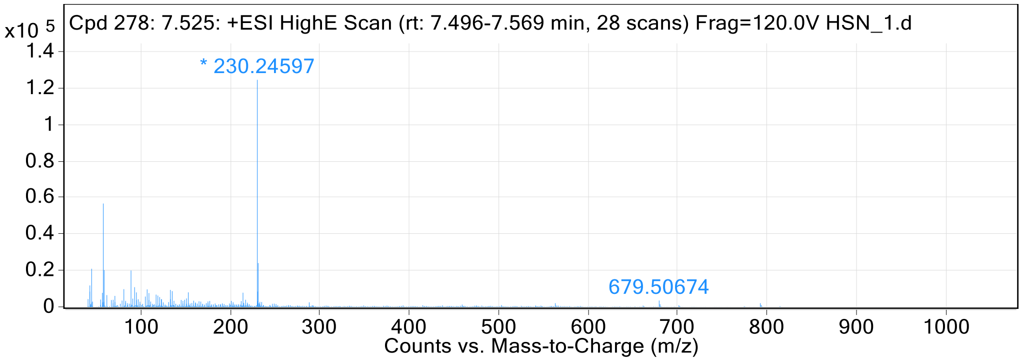

Peak List

m/z	z	Abund
43.05391		11729.58
45.03313		20885
58.06463	1	56638.02
59.04855		20157.8
81.06874		9697.26
89.05844		19914.5
93.06846		10794.46
107.08383		9569.33
230.24597	1	124441.3
231.24843	1	24010.75

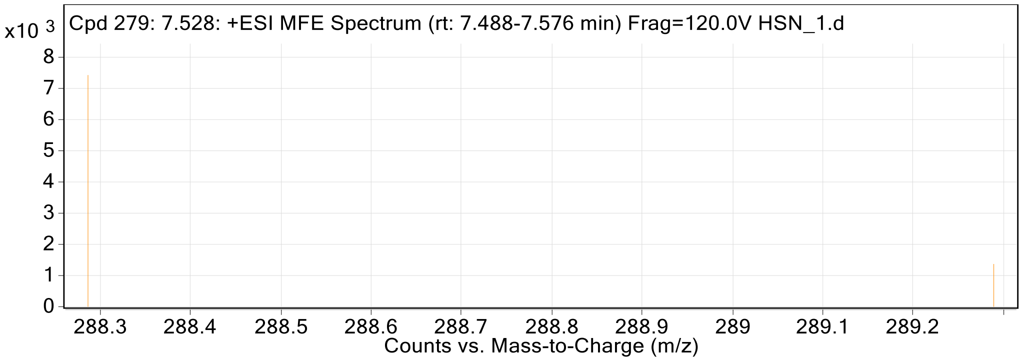

Peak List

m/z	z	Abund	Ion
288.28672	1	7408.64	(M+H)+
289.2893	1	1367.46	(M+H)+

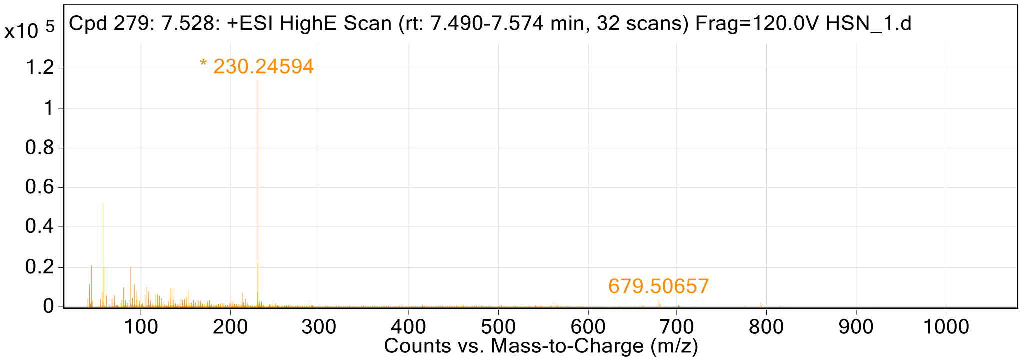

Peak List

m/z	z	Abund
43.05389		11229.91
45.03313		20967.18
58.06462	1	51752.5
59.04855		19870.97
81.06873		9816.16
89.05845		20155.78
93.0685		11031.4
107.08383		9804.93
230.24594	1	114173.69
231.24843	1	21806.2

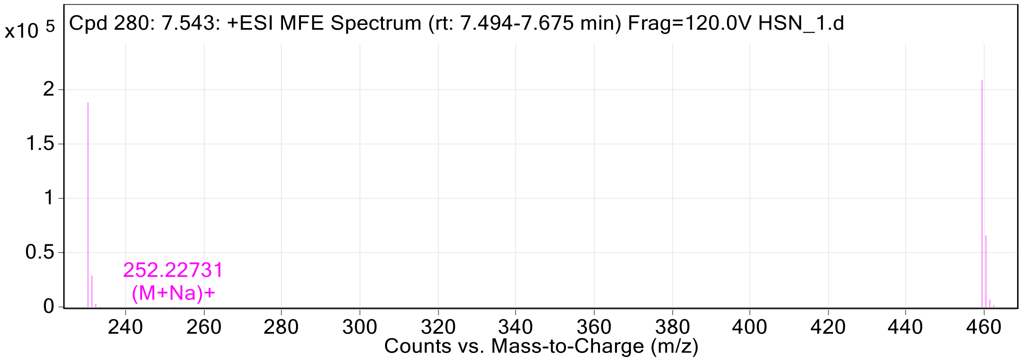

Peak List

m/z	z	Abund	Ion
230.2451	1	187975.95	(M+H)+
231.24828	1	28793.83	(M+H)+
232.25134	1	2829.96	(M+H)+
233.25313	1	105.77	(M+H)+
252.22731	1	461.08	(M+Na)+
459.484	1	208575.97	(2M+H)+
460.48772	1	65910.74	(2M+H)+
461.4908	1	6776.64	(2M+H)+
462.49248	1	1505.76	(2M+H)+

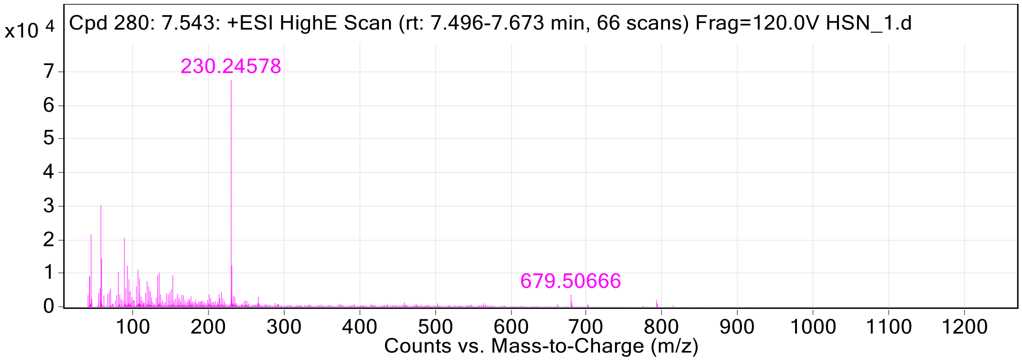

Peak List

m/z	z	Abund
45.03315		21563.07
58.0646		30322.57
59.04856		14404.24
81.06876		10382.86
89.05842		20487.9
93.06848		12265.29
107.08381		11065.58
135.1146		10137.84
230.24578	1	67572.81

231.24844	1	12338.36

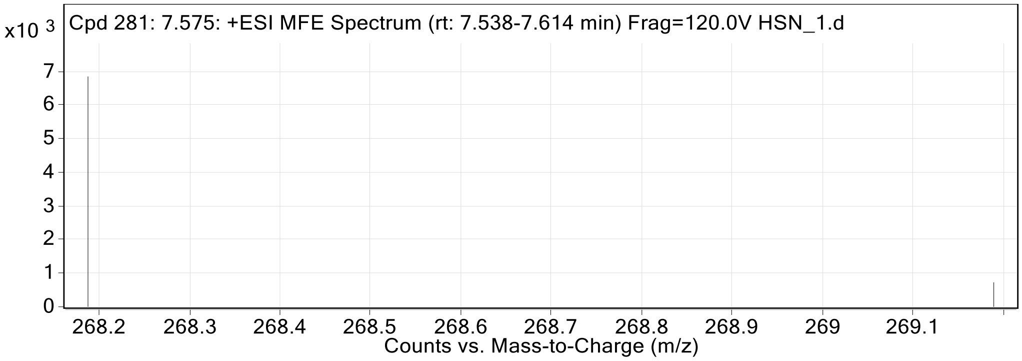

Peak List

m/z	z	Abund	Ion
268.1878	1	6849.82	(M+H)+
269.18904	1	726.93	(M+H)+

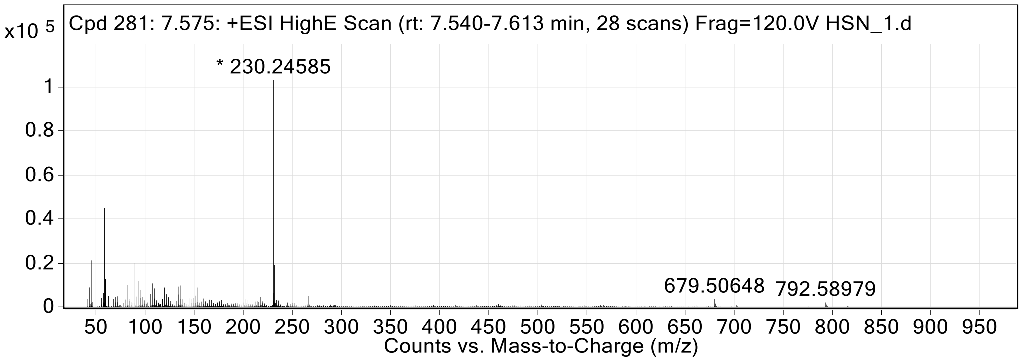

Peak List

m/z	z	Abund
45.03314		21103.5
58.06459	1	44768.09
59.04854		12690.43
81.06872		9924.61
89.05844		19771.31
93.06847		11675.71
107.08376		10649.65
135.11462		9708.7
230.24585	1	102896.59
231.2484	1	19121.81

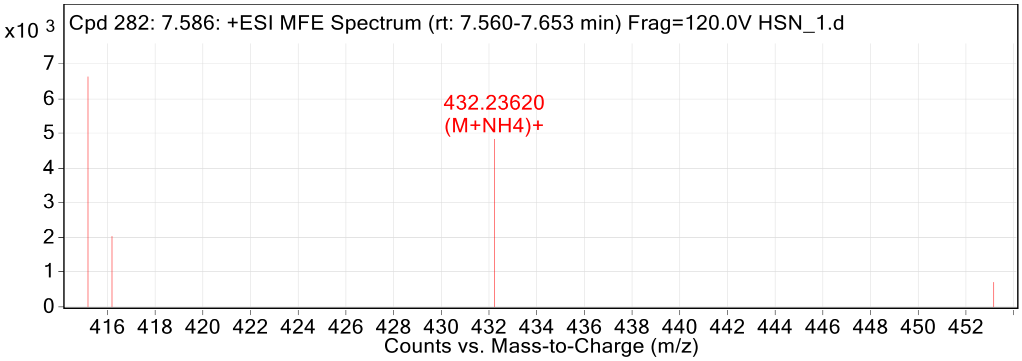

Peak List

m/z	z	Abund	Ion
415.20788	1	6646.96	(M+H)+

416.21369	1	2039.21	(M+H)+
432.2362	1	4840.53	(M+NH4)+
453.16547	1	715.69	(M+K)+

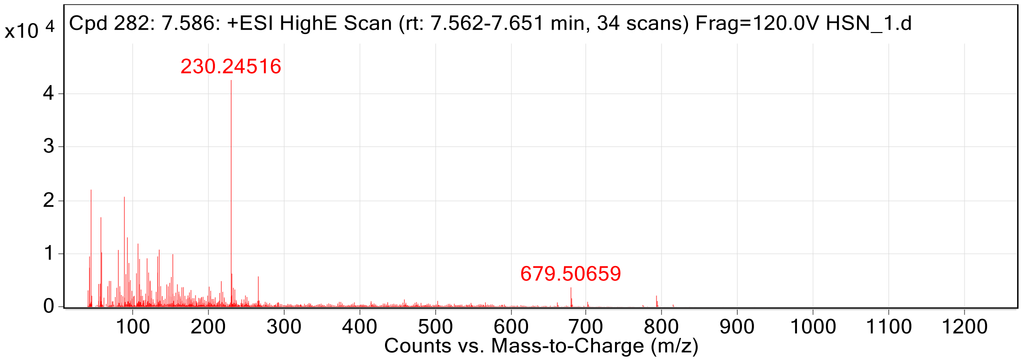

Peak List

m/z	z	Abund
45.03314		22002.95
58.06453		16816.56
59.04857		10217.95
81.06876		10666.22
89.05841		20662.4
93.06849		13032.58
107.08376		11857
135.11459		10725.85
153.12499		9871.71
230.24516	1	42604.5

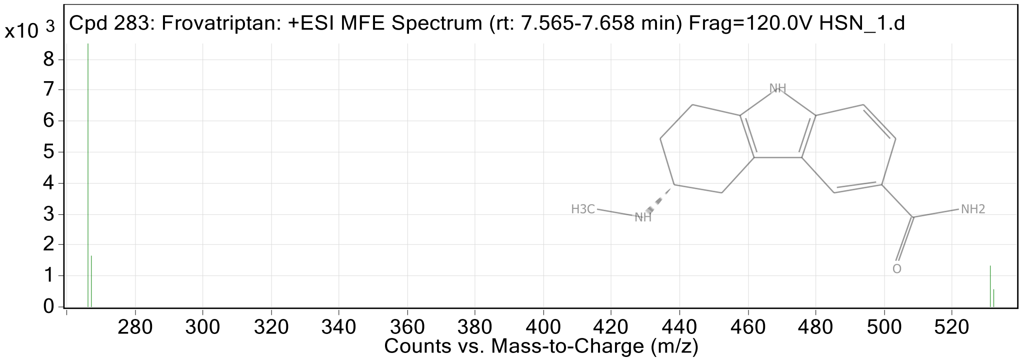

Peak List

m/z	z	Abund	Name	Formula	Ion	Score (DB)	Hits (DB)
266.12614	1	8504.52	Frovatriptan	C14 H17 N3 O	(M+Na)+	83.88	1
267.1286	1	1655.47		C14 H17 N3 O	(M+Na)+		
531.24878	1	1334.75			(2M+H)+		
532.25021	1	572.49			(2M+H)+		

Qualitative Analysis Report

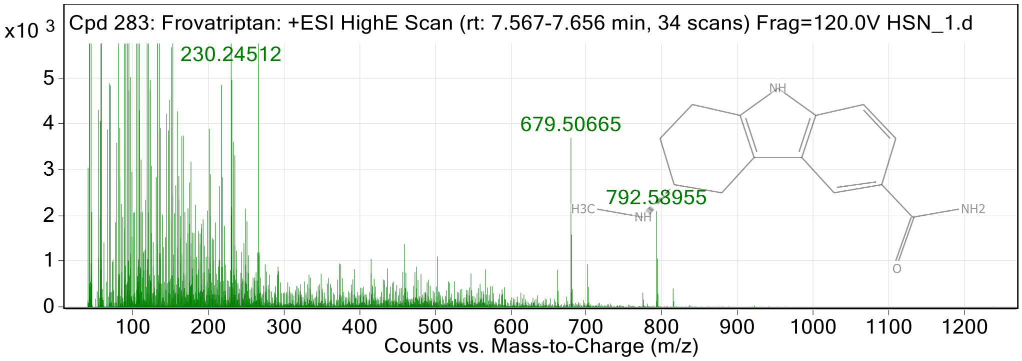

Peak List

m/z	z	Abund
45.03314		22115.81
58.06451		13859.26
59.04857		10021.25
81.06875		10775.6
89.05841		20773.53
93.0685		13171.24
107.08377		12040.39
135.11458		10912.5
153.12501		10028.83
230.24512	1	34328.48

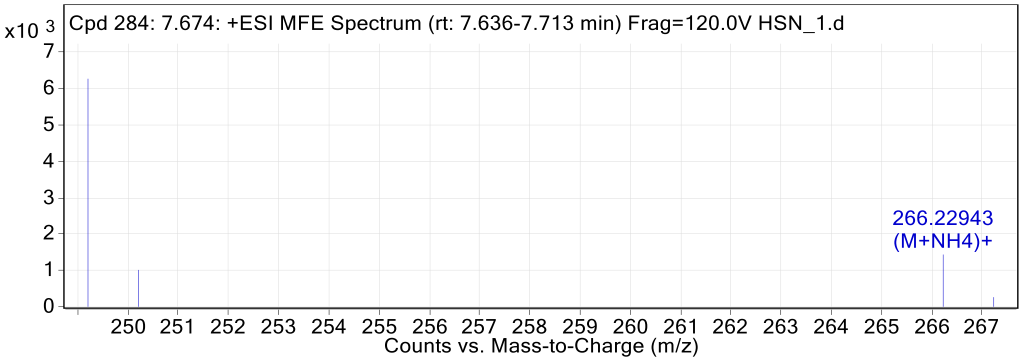

Peak List

m/z	z	Abund	Ion
249.20321	1	6266.46	(M+H)+
250.20645	1	1012.76	(M+H)+
266.22943	1	1435.64	(M+NH4)+
267.23704	1	264.12	(M+NH4)+

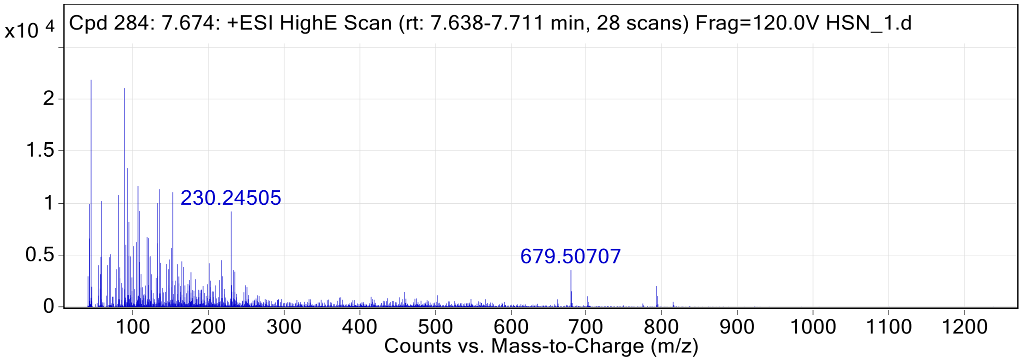

Peak List

m/z	Abund
43.01758	9918.78

45.03319	21875.67
59.04858	10191.56
81.06878	10757.79
89.05845	21067.94
93.0685	13351.76
107.08389	11665.19
133.08435	9984.14
135.11456	11324.83
153.12504	11034.97

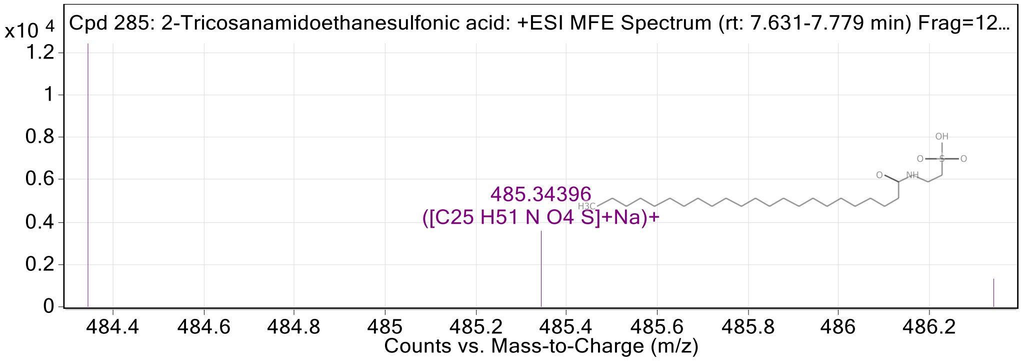

Peak List

m/z	z	Abund	Name	Formula	Ion	Score (DB)	Hits (DB)
484.34411	1	12440.88	Tricosanamidoethanesulfonic acid	C25 H51 N O4 S	(M+Na)+	89.31	1
485.34396	1	3593.17		C25 H51 N O4 S	(M+Na)+		
486.34216	1	1331.78		C25 H51 N O4 S	(M+Na)+		

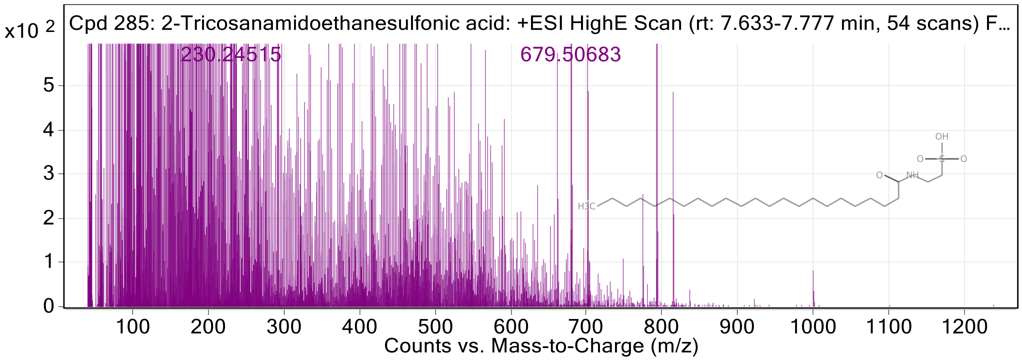

Peak List

m/z	Abund
43.01756	9153.73
45.03321	19960.64
59.04861	9536.51
81.06882	10619.08
89.05846	19183.69
93.06851	13122.66
107.08388	11206.14
133.08456	9057.05
135.11463	10947.42
153.1251	10603.55

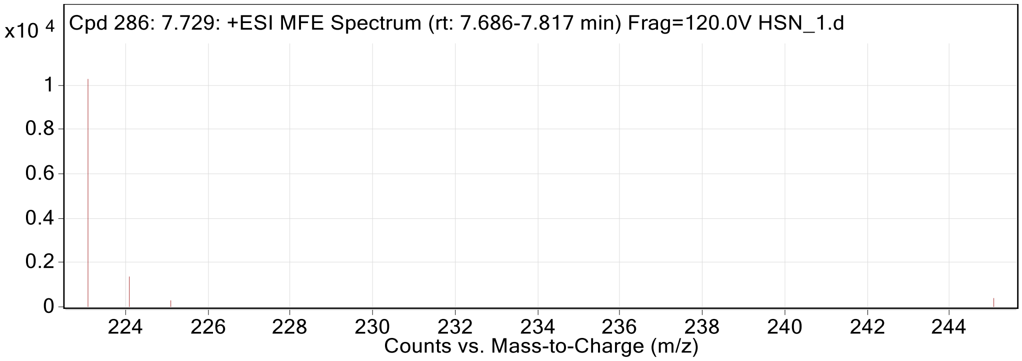

Peak List

m/z	z	Abund	Ion
223.09398	1	10268.63	(M+H)+
224.09781	1	1366.55	(M+H)+
225.10336	1	292.77	(M+H)+
245.07601	1	395.97	(M+Na)+

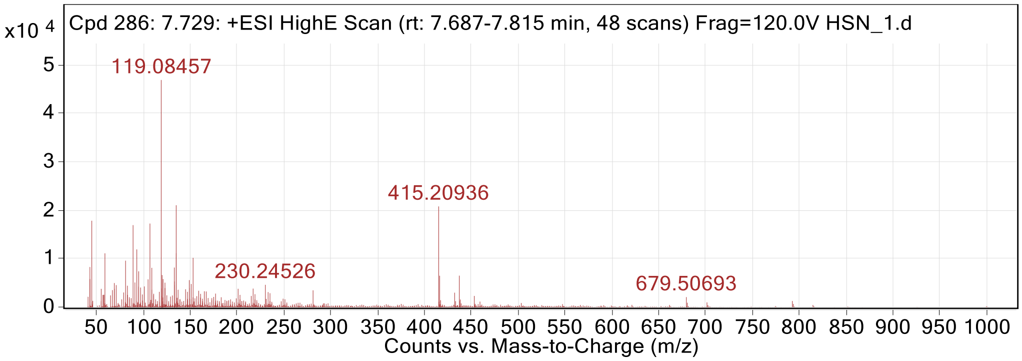

Peak List

m/z	z	Abund
45.0332		17819.12
59.0486		11090.95
89.0585		16912.55
93.06852		11894.38
107.08395		17235.9
119.08457	1	46881.19
135.07853	1	21016.24
135.11456		11107.44
153.12512		10161.92
415.20936	1	20774.9

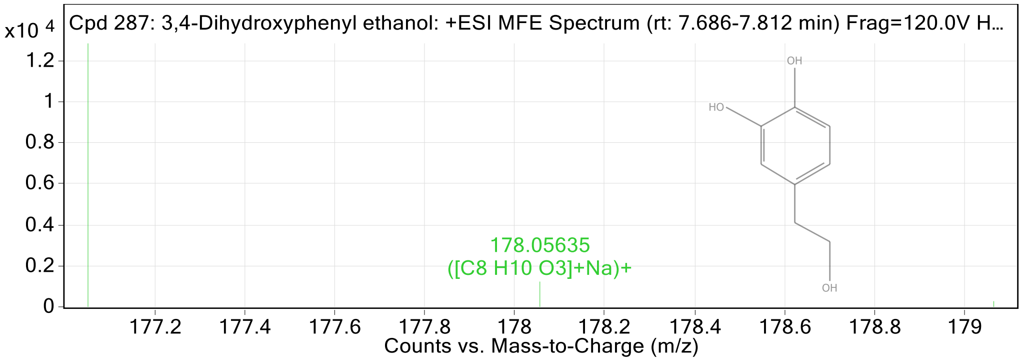

Peak List

m/z	z	Abund	Name	Formula	Ion	Score (DB)	Hits (DB)

177.05224	1	12840.17	3,4-Dihydroxyphenyl ethanol	C8 H10 O3	(M+Na)+	95.81	8
178.05635	1	1230.47		C8 H10 O3	(M+Na)+		
179.06497	1	276.6		C8 H10 O3	(M+Na)+		

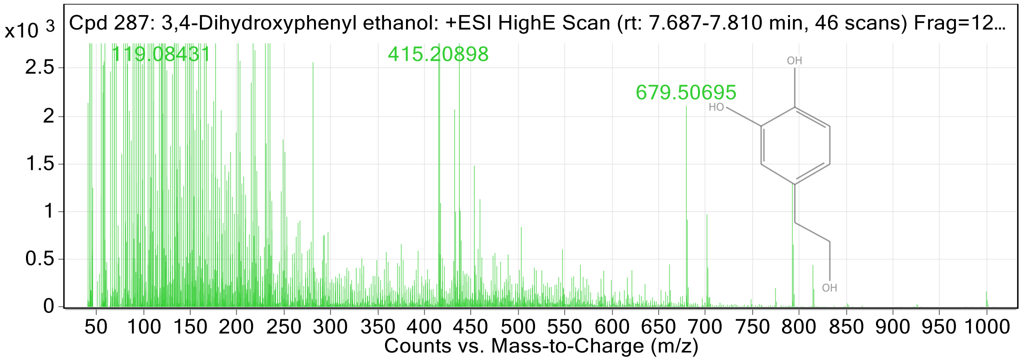

Peak List

m/z	z	Abund
45.0332		17820.47
59.0486		10708.23
89.0585		16950.35
93.06851		12178.46
107.08393		15623.51
119.08431	1	38820.63
135.07848		15838.64
135.11459		11037.64
153.12512		10266.37
415.20898	1	15464.34

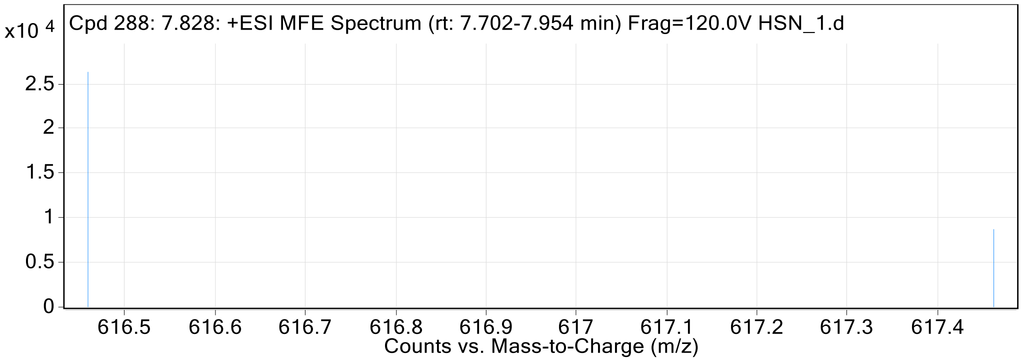

Peak List

m/z	z	Abund	Ion
616.45909	1	26336.34	(M+H)+
617.46252	1	8723.54	(M+H)+

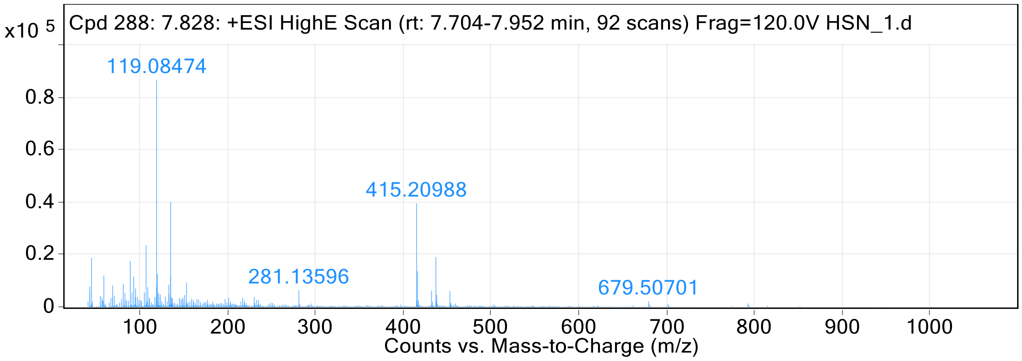

Qualitative Analysis Report

Peak List

m/z	z	Abund
45.03322		18627.44
59.04866		11935.26
89.05854		17494.98
107.08401	1	23513.04
119.08474		86665.77
120.0871		12547.03
135.0786	1	40064.9
415.20988	1	39520.21
416.21201	1	13629.11
437.1904	1	18952.68

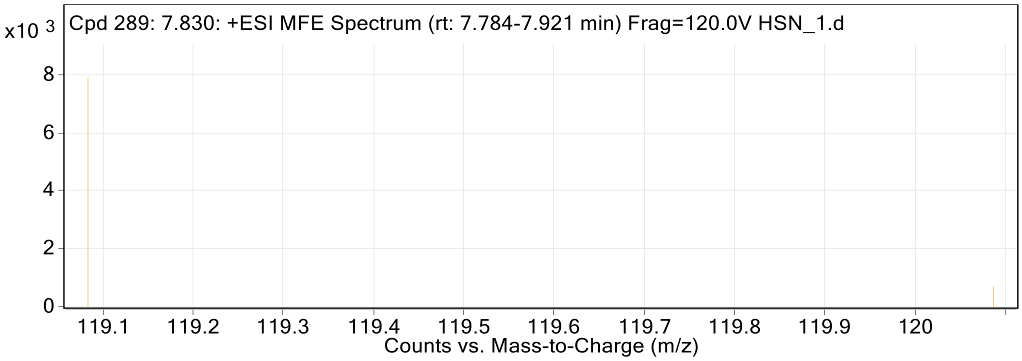

Peak List

m/z	z	Abund	Ion
119.08383	1	7914.43	(M+H)+
120.08759	1	705.8	(M+H)+

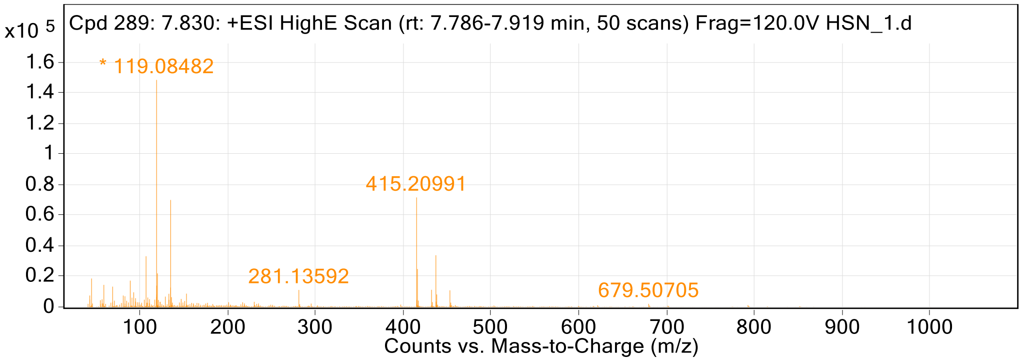

Peak List

m/z	z	Abund
45.03321		18591.9
59.04866		14438.81
89.05856		17197.11
107.08405	1	33156.43
119.08482		148361.81
120.08711		21928.01
135.07861	1	69915.13
415.20991	1	71579.17
416.212	1	24793.63
437.19038	1	33800.95

Qualitative Analysis Report

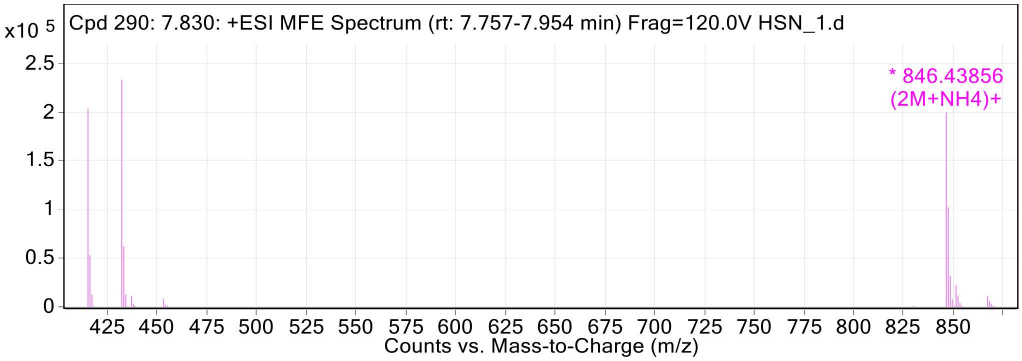

Peak List

m/z	z	Abund	Ion
415.20857	1	203327.11	(M+H)+
416.21198	1	53127.34	(M+H)+
417.21485	1	12646.07	(M+H)+
432.23532	1	233033.72	(M+NH4)+
433.23903	1	62381.27	(M+NH4)+
434.24081	1	12556.15	(M+NH4)+
846.43856	1	199553.38	(2M+NH4)+
847.44056	1	102372.22	(2M+NH4)+
848.44438	1	31958.05	(2M+NH4)+
851.39363	1	22359.87	(2M+Na)+

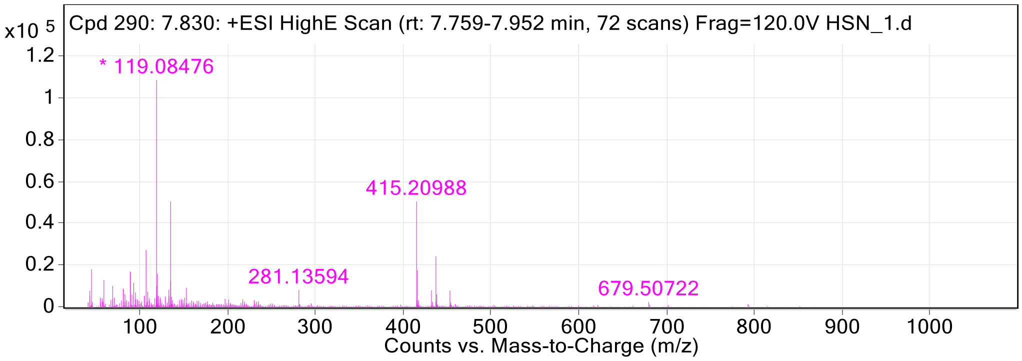

Peak List

m/z	z	Abund
45.03321		18004.7
59.04866		12865.56
89.05854		16822.21
107.08402	1	27249.41
119.08476		108704.13
120.0871		15804.45
135.07861	1	50483.68
415.20988	1	50441.35
416.21201	1	17409.19
437.1904	1	24207.39

Qualitative Analysis Report

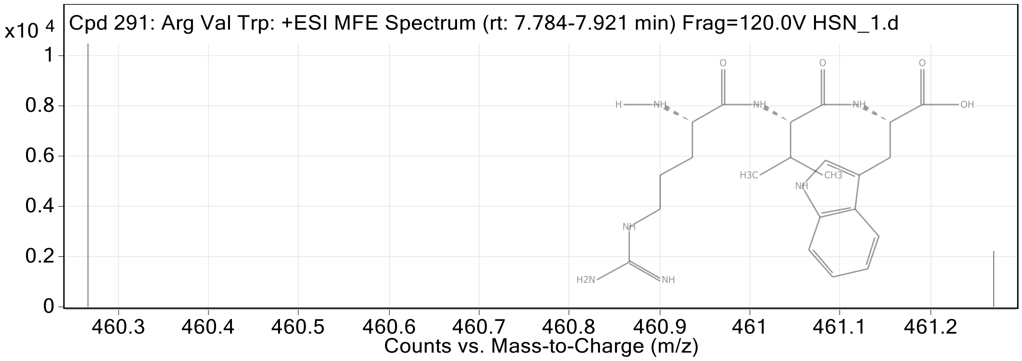

Peak List

m/z	z	Abund	Name	Formula	Ion	Score (DB)	Hits (DB)
460.26646	1	10487.71	Arg Val Trp	C22 H33 N7 O4	(M+H)+	78.9	6
461.27028	1	2226.75		C22 H33 N7 O4	(M+H)+		

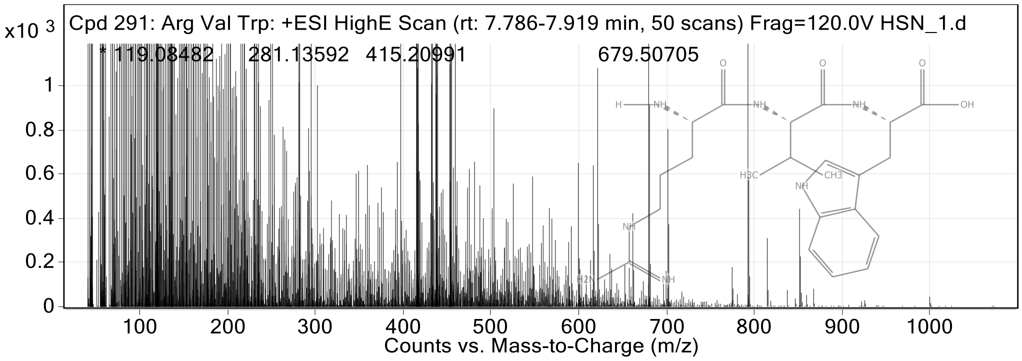

Peak List

m/z	z	Abund
45.03321		18591.9
59.04866		14438.81
89.05856		17197.11
107.08405	1	33156.43
119.08482		148361.81
120.08711		21928.01
135.07861	1	69915.13
415.20991	1	71579.17
416.212	1	24793.63
437.19038	1	33800.95

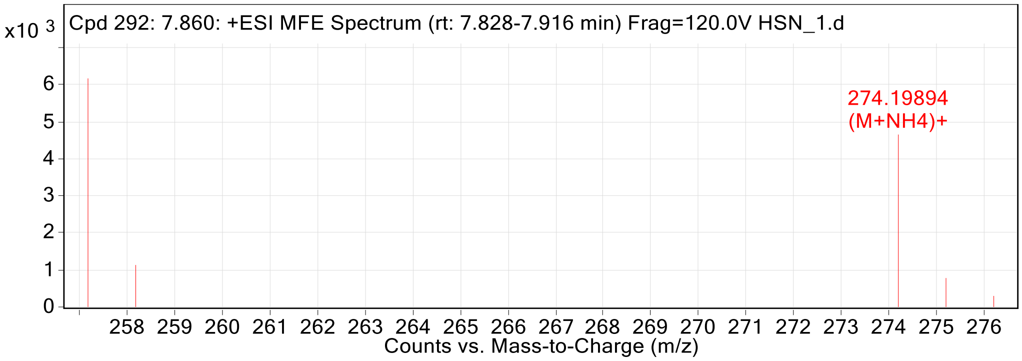

Peak List

m/z	z	Abund	Ion
257.1727	1	6167.61	(M+H)+
258.17613	1	1132.15	(M+H)+
274.19894	1	4653	(M+NH4)+

275.20086	1	780.75	(M+NH4)+
276.20467	1	297.6	(M+NH4)+

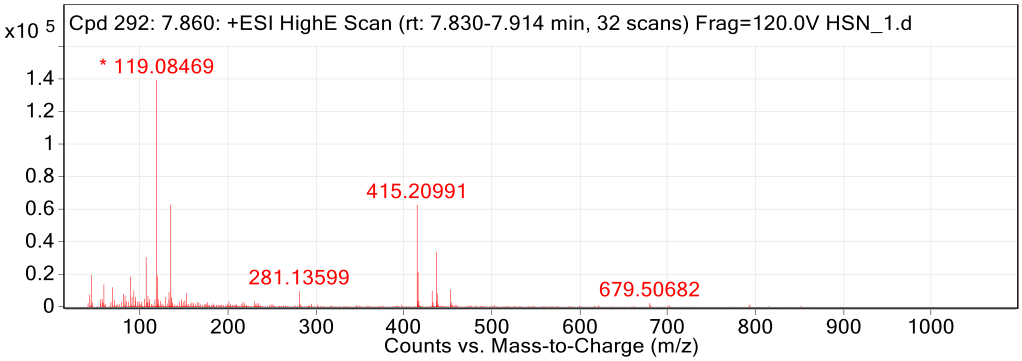

Peak List

m/z	z	Abund
45.03324		19576.66
59.0487		13654.67
89.0586		18443.38
107.08406	1	30506.47
119.08469	1	139052.91
120.0871	1	19212.94
135.07863	1	62622.81
415.20991	1	62513.73
416.21208	1	21259.92
437.19042	1	33831.79

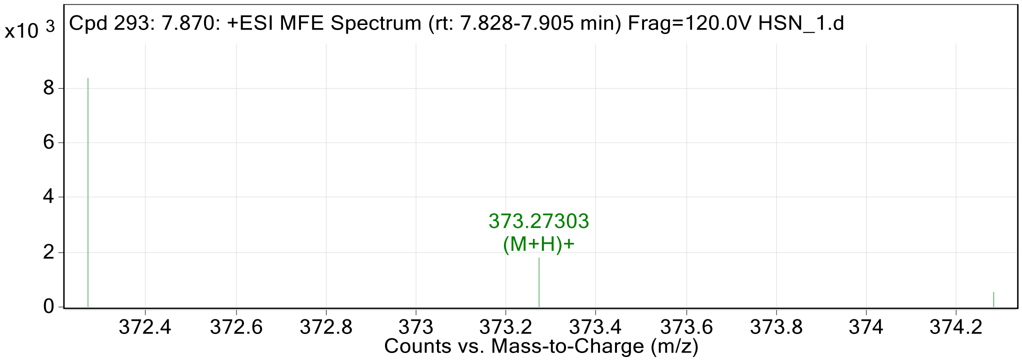

Peak List

m/z	z	Abund	Ion
372.27119	1	8359.02	(M+H)+
373.27303	1	1804.1	(M+H)+
374.28299	1	547.98	(M+H)+

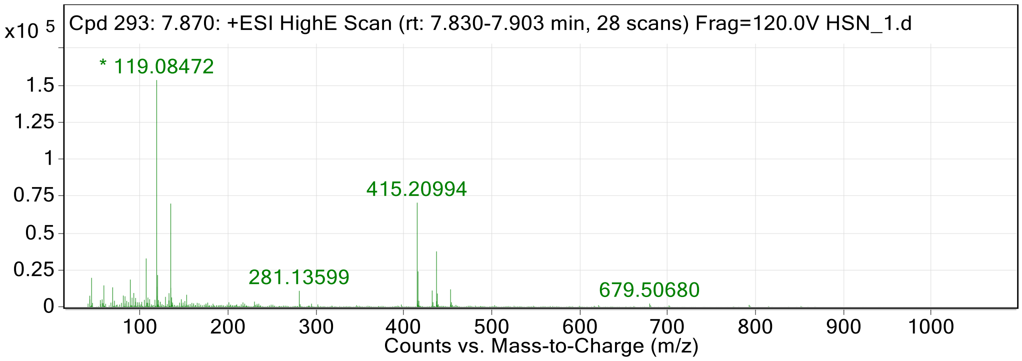

Peak List

m/z	z	Abund

45.03324		19604.65
59.0487		14500.05
89.05858		18423.7
107.08405	1	32735.65
119.08472	1	153515.52
120.08709	1	21488.09
135.07863	1	69887.73
415.20994	1	70509.53
416.21208	1	24013.74
437.19041	1	37533.21

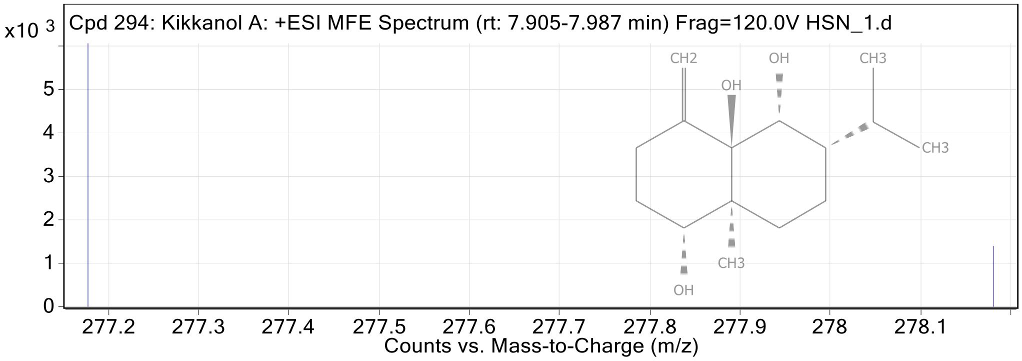

Peak List

m/z	z	Abund	Name	Formula	Ion	Score (DB)	Hits (DB)
277.1776	1	6052.83	Kikkanol A	C15 H26 O3	(M+Na)+	77.72	10
278.18097	1	1396.13		C15 H26 O3	(M+Na)+		

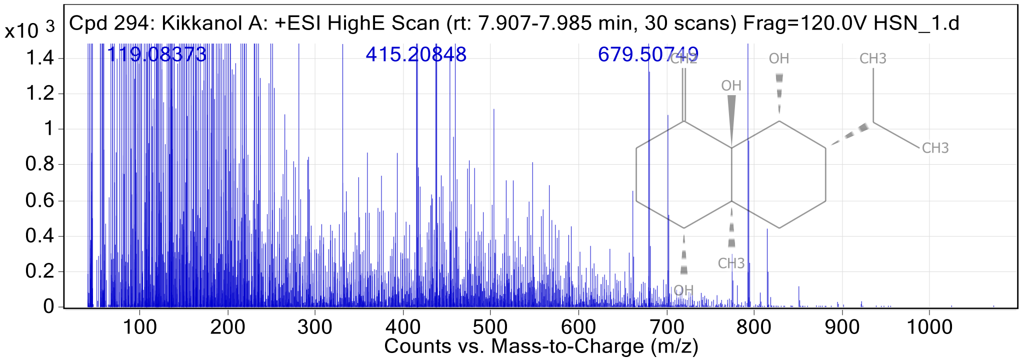

Peak List

m/z	z	Abund
45.03323		20711.96
81.06886		10489.03
89.05855		19591.01
93.06859		15828.74
107.08391		16062.58
109.09954		9837.04
119.08373	1	40126.86
135.07846		10633.2
135.11475		11631.94
197.04211		9745.13

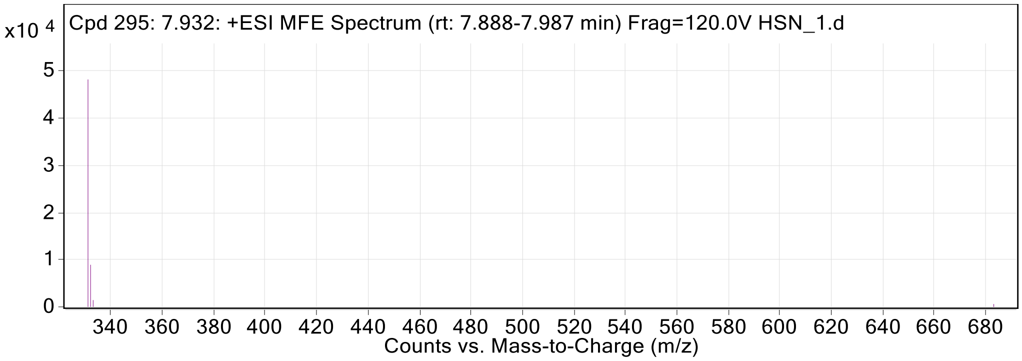

Peak List

m/z	z	Abund	Ion
331.11502	1	48202.47	(M+H)+
332.11841	1	8924.6	(M+H)+
333.12156	1	1439.51	(M+H)+
683.20444	1	601.06	(2M+Na)+

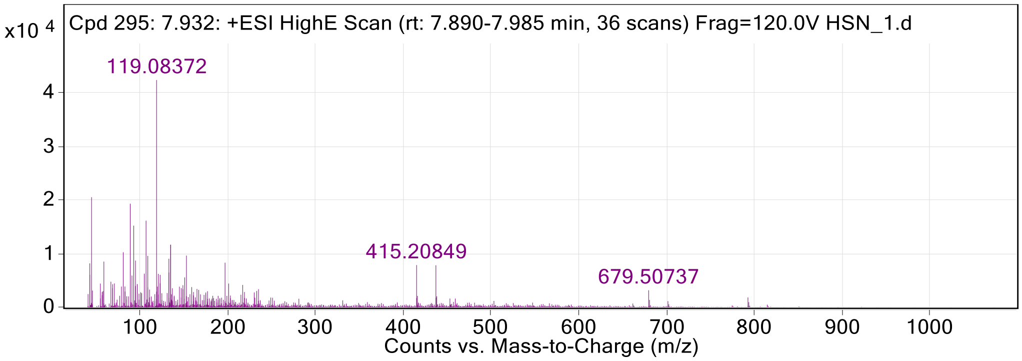

Peak List

m/z	z	Abund
45.03324		20510.46
81.06885		10231.84
89.05854		19294.96
93.06862		15191.09
107.08393		16129.03
109.09958		9545.25
119.08372	1	42393.77
135.07845		11649.74
135.1147		11605.67
153.12518		9603.96

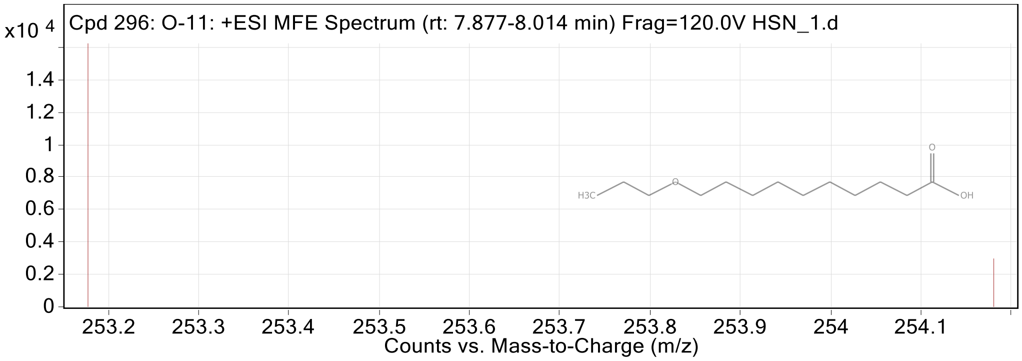

Peak List

m/z	z	Abund	Name	Formula	Ion	Score (DB)	Hits (DB)
253.17748	1	16235.19	O-11	C13 H26 O3	(M+Na)+	82.79	10

254.1806	1	2978.69		C13 H26 O3	(M+Na)+		

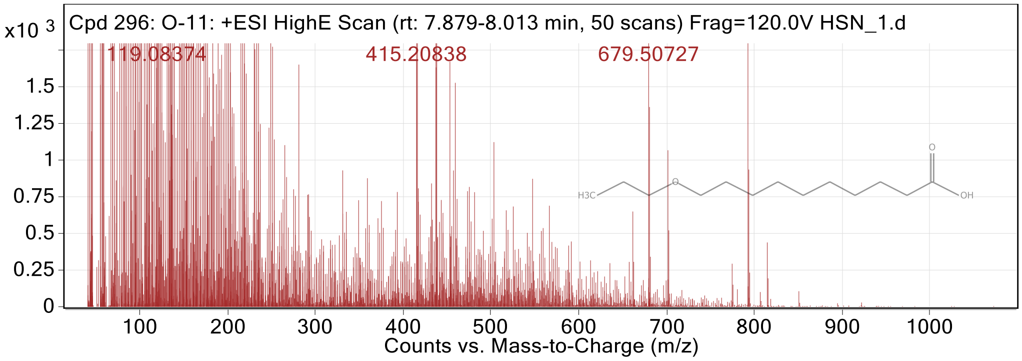

Peak List

m/z	z	Abund
45.03323		20581.33
81.06884		10263.51
89.05856		19484.36
93.06862		15023.64
107.08395		15887.12
109.09954		9624.39
119.08374	1	43410.25
135.07847		12085.4
135.11473		11816.39
153.12516		9798.58

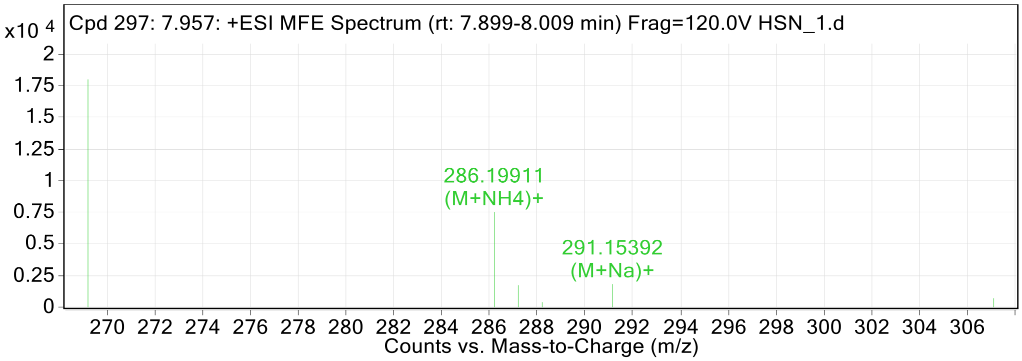

Peak List

m/z	z	Abund	Ion
269.17248	1	18016.13	(M+H)+
286.19911	1	7518.66	(M+NH4)+
287.20138	1	1730.61	(M+NH4)+
288.2048	1	390.38	(M+NH4)+
291.15392	1	1821.68	(M+Na)+
307.12946	1	698.23	(M+K)+

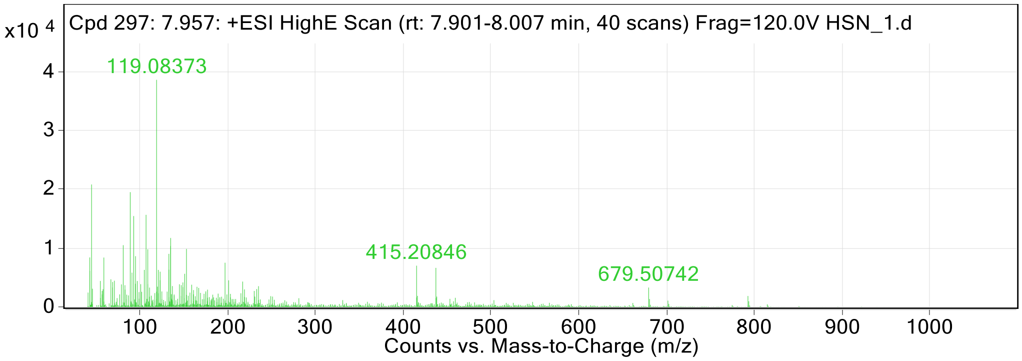

Peak List

m/z	z	Abund
45.03323		20834.01
81.06884		10505.38
89.05855		19525.71
93.0686		15461.61
107.08394		15668.45
109.09952		9805.66
119.08373	1	38643.71
135.07846		10364.21
135.11476		11724.15
153.12515		9859.41

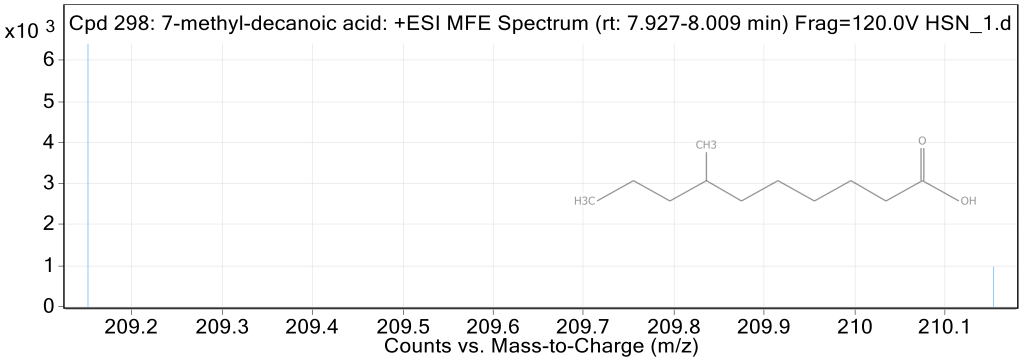

Peak List

m/z	z	Abund	Name	Formula	Ion	Score (DB)	Hits (DB)
209.15155	1	6413.18	7-methyl-decanoic acid	C11 H22 O2	(M+Na)+	83.15	10
210.1541	1	977.78		C11 H22 O2	(M+Na)+		

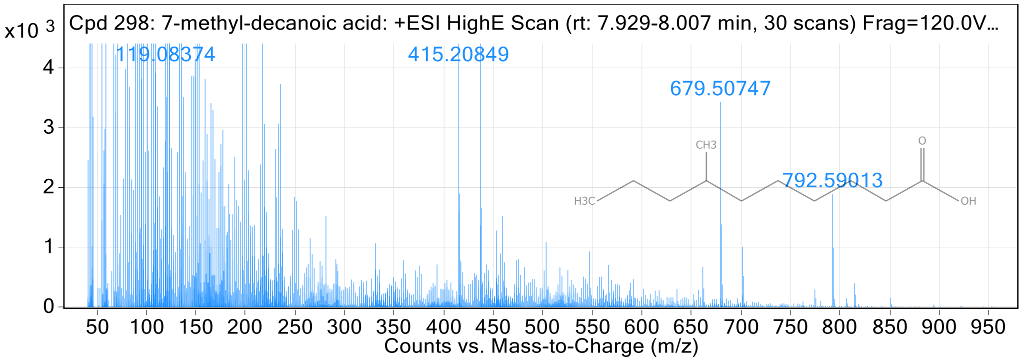

Peak List

m/z	z	Abund
45.03322		21084.25
81.06883		10831.27
89.05853		19882.48

93.06858		15954.85
107.0839		15888.76
109.09954		10092.63
119.08374	1	39337.88
135.07845		9989.76
135.11478		11891.41
153.12513		10046.67

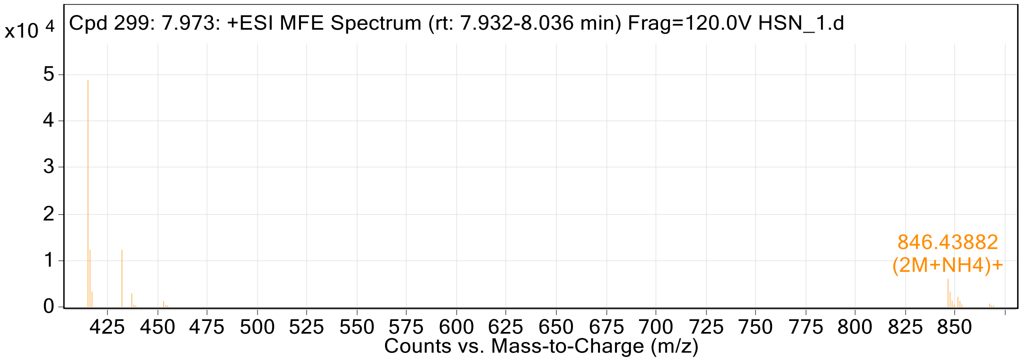

Peak List

m/z	z	Abund	Ion
415.20861	1	48910.51	(M+H)+
416.2121	1	12325.44	(M+H)+
417.21589	1	3292.77	(M+H)+
432.23493	1	12340.39	(M+NH4)+
437.19152	1	2898.9	(M+Na)+
846.43882	1	6100.98	(2M+NH4)+
847.44206	1	3305.46	(2M+NH4)+
848.44681	1	1374.32	(2M+NH4)+
851.3971	1	2191.19	(2M+Na)+
852.39561	1	1297.96	(2M+Na)+

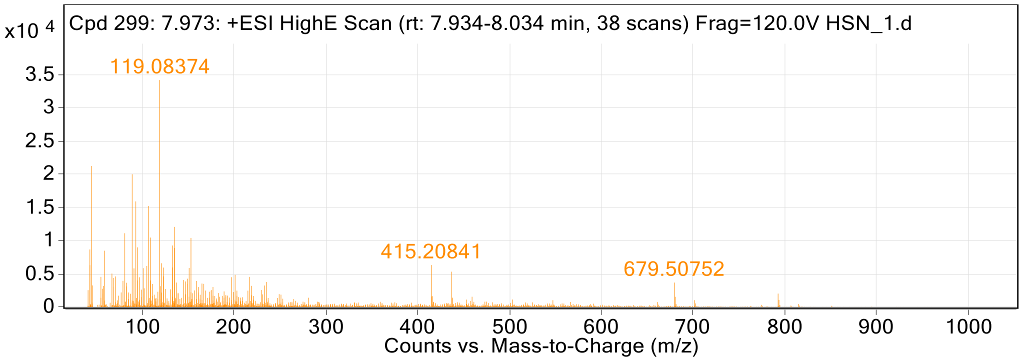

Peak List

m/z	z	Abund
45.03323		21222.88
81.06888		11088.57
89.05855		19981.97
93.06861		15890.15
107.08393		15193
109.09955		10435.35
119.08374	1	34175.95
133.0849		9240.95
135.11478		12043.11
153.12516		10373.69

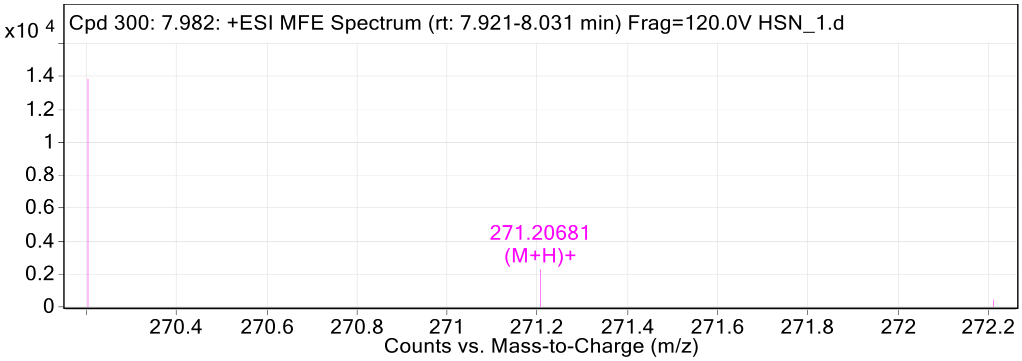

Peak List

m/z	z	Abund	Ion
270.20378	1	13861.67	(M+H)+
271.20681	1	2288	(M+H)+
272.21178	1	440.41	(M+H)+

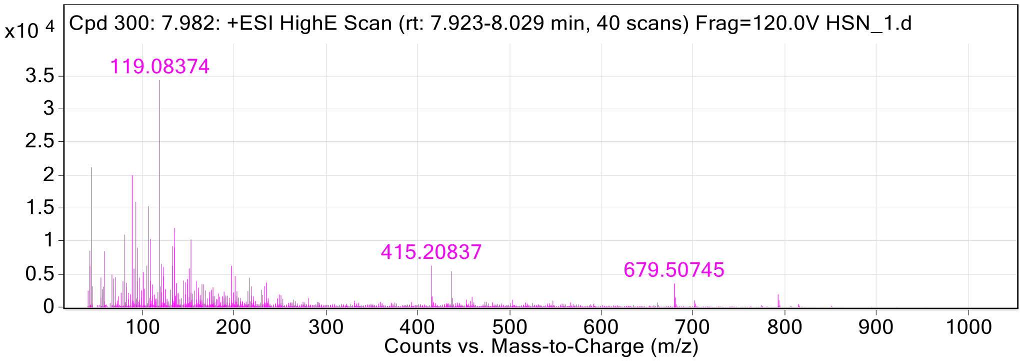

Peak List

m/z	z	Abund
45.03323		21146.76
81.06885		10963.35
89.05854		19933.94
93.0686		15910.53
107.08392		15246.25
109.09956		10313.86
119.08374	1	34343.44
133.08484		9230.03
135.11477		11971.51
153.12514		10233.1

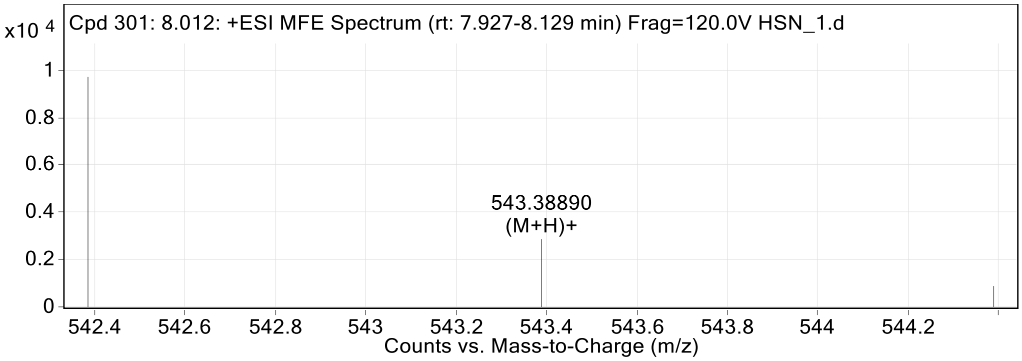

Peak List

m/z	z	Abund	Ion
542.38553	1	9718.24	(M+H)+
543.3889	1	2862.27	(M+H)+

544.38901	1	883.59	(M+H)+

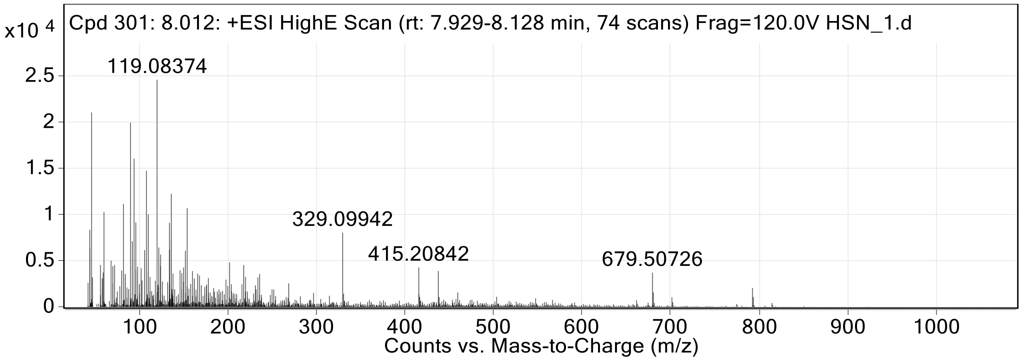

Peak List

m/z	z	Abund
45.03324		21073.44
59.04866		10289.58
81.06886		11150.45
89.05855		19961.78
93.0686		16071.68
107.08395		14762.43
109.09952		10024.63
119.08374	1	24623.91
135.11477		12255.17
153.12513		10690.55

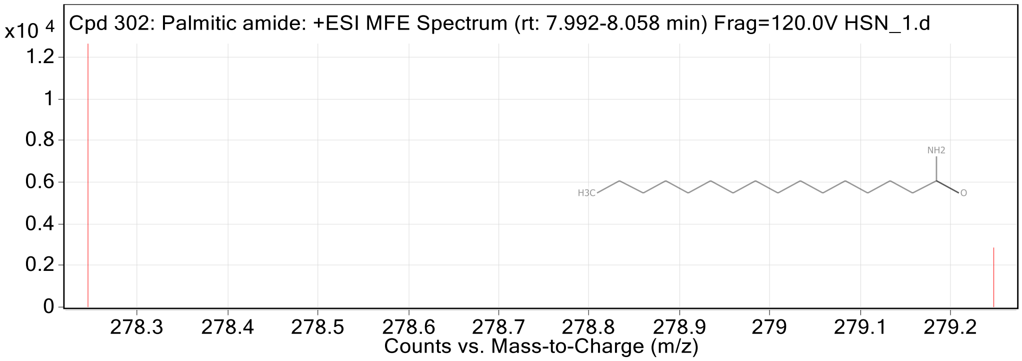

Peak List

m/z	z	Abund	Name	Formula	Ion	Score (DB)	Hits (DB)
278.2452	1	12666.97	Palmitic amide	C16 H33 N O	(M+Na)+	81.43	1
279.2481	1	2862.24		C16 H33 N O	(M+Na)+		

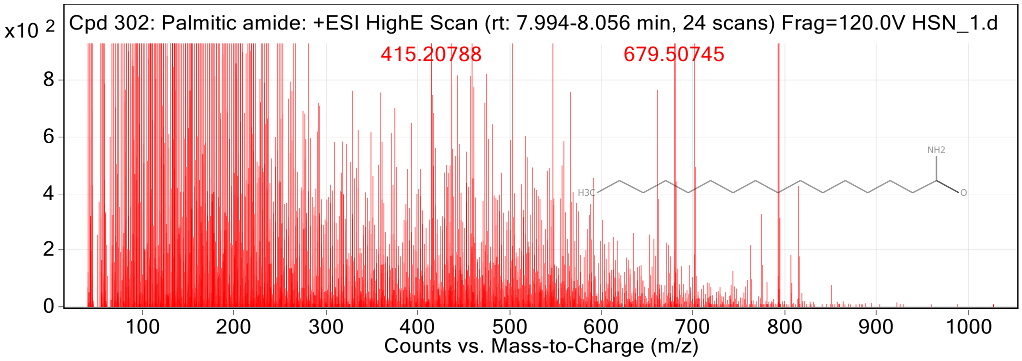

Peak List

m/z	z	Abund
45.03326		21190.13
81.06887		11738.62

89.05858		19836.38
93.06863		15595.04
95.08417		9130.68
107.08396		13227.4
109.09951		10555.03
119.08373	1	18222.51
135.1148		12407.82
153.12509		11427.67

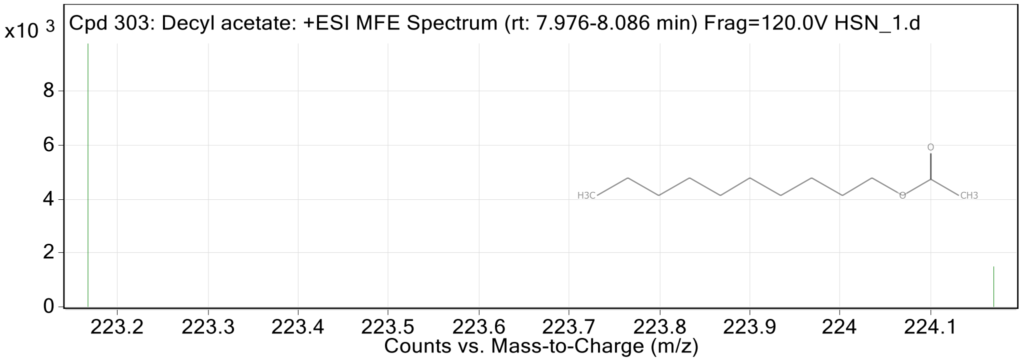

Peak List

m/z	z	Abund	Name	Formula	Ion	Score (DB)	Hits (DB)
223.16703	1	9789.72	Decyl acetate	C12 H24 O2	(M+Na)+	85.98	10
224.17024	1	1501.18		C12 H24 O2	(M+Na)+		

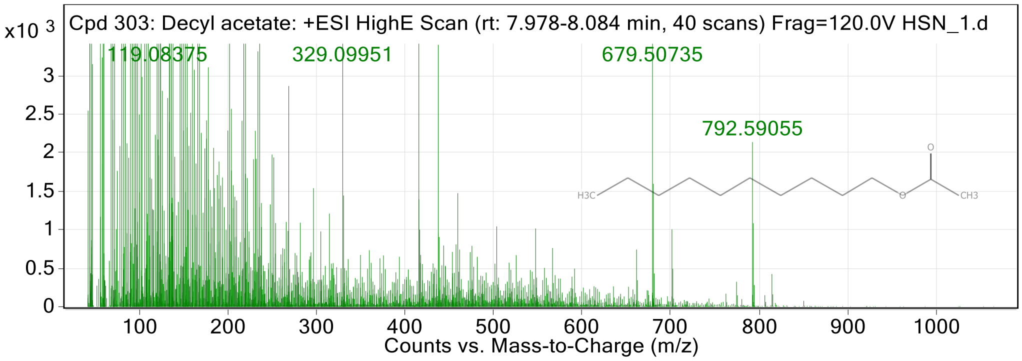

Peak List

m/z	z	Abund
45.03323		21084.63
81.06887		11300.12
89.05859		19846.53
93.06862		15116.87
107.08394		13355.03
109.09951		10022.76
119.08375	1	22675.27
133.08479		9112.28
135.1148		12196.74
153.12511		11263.99

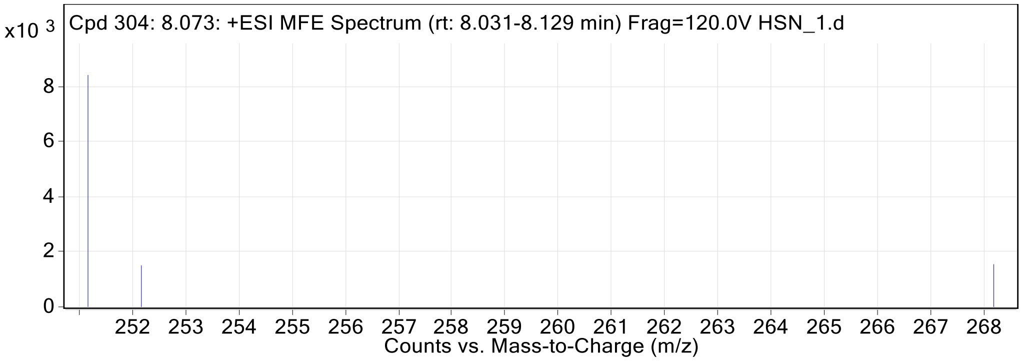

Peak List

m/z	z	Abund	Ion
251.16198	1	8410.98	(M+H)+
252.16547	1	1503.85	(M+H)+
268.18832	1	1547.89	(M+NH4)+

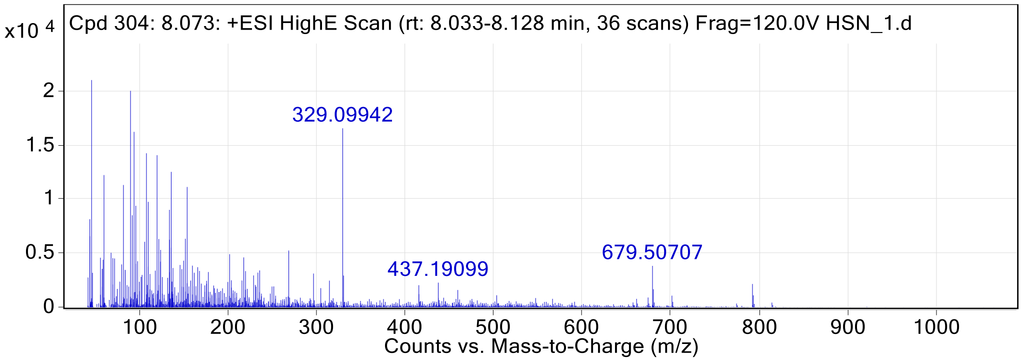

Peak List

m/z	z	Abund
45.03325		20984.6
59.04862		12203.22
81.06886		11284.05
89.05856		19986.29
93.0686		16195.92
107.08397		14217.47
119.08373		14040.3
135.11476		12497.62
153.12511		11100.74
329.09942	1	16524.3

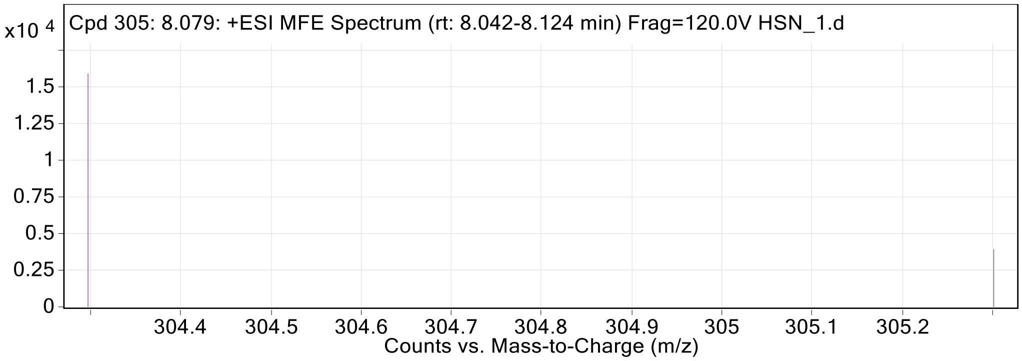

Peak List

m/z	z	Abund	Ion
304.29732	1	15897.69	(M+H)+
305.30116	1	3930.23	(M+H)+

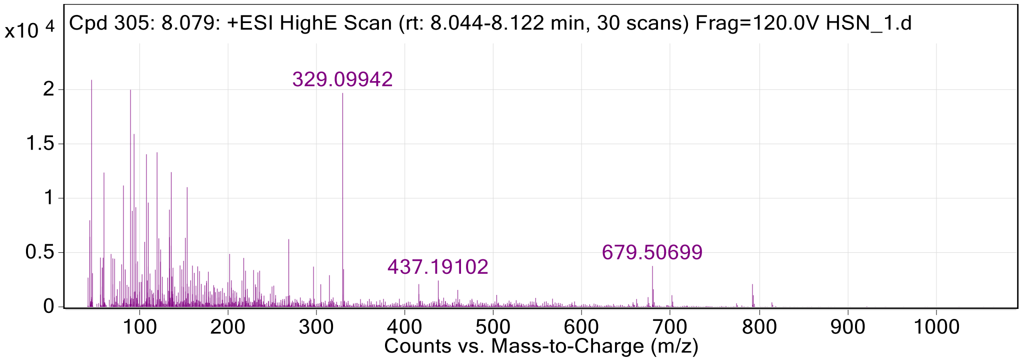

Peak List

m/z	z	Abund
45.03325		20887.43
59.04862		12357.63
81.06884		11167.16
89.05856		19974.61
93.06861		15908.03
107.08396		14042.62
119.08374		14222.46
135.11475		12398.54
153.12507		11013.27
329.09942	1	19680.21

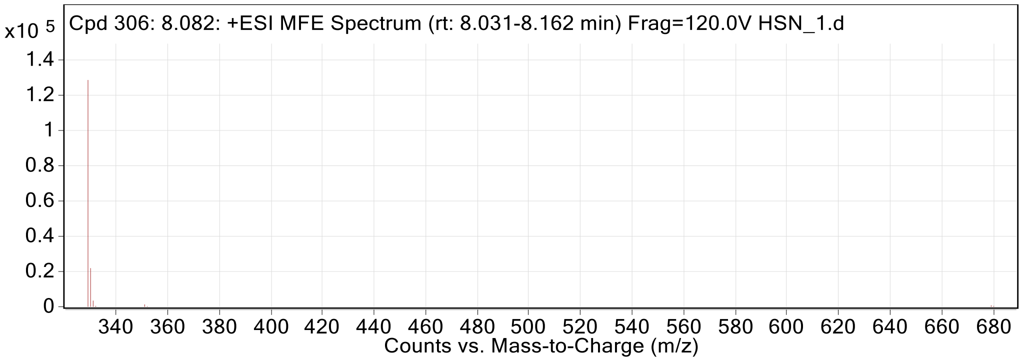

Peak List

m/z	z	Abund	Ion
329.09966	1	128955.49	(M+H)+
330.10266	1	21974.7	(M+H)+
331.10593	1	3512.94	(M+H)+
332.1063	1	505.75	(M+H)+
351.08117	1	1335.33	(M+Na)+
352.08273	1	293.13	(M+Na)+
679.17439	1	807.42	(2M+Na)+
680.17931	1	493.51	(2M+Na)+

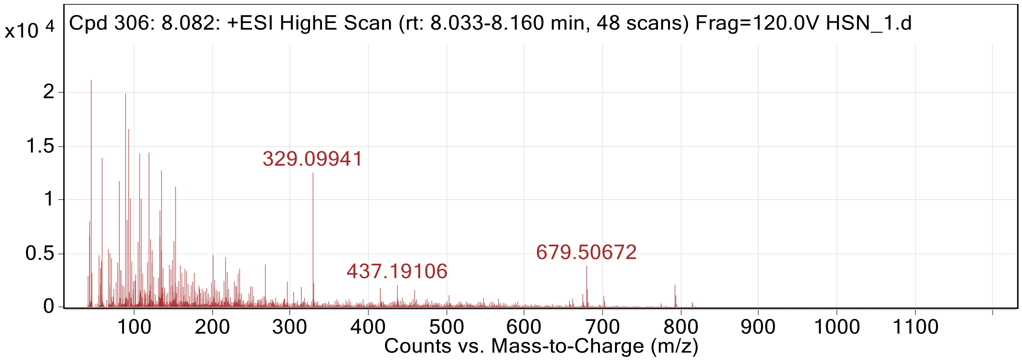

Peak List

m/z	z	Abund
45.03323		21227.15
59.04865		13935.24
81.06887		11776.9
89.05857		19974.38
93.06863		16629.04
107.08394		14325.13
119.08372		14425.48
135.11477		12744.64
153.12517		11226.94
329.09941	1	12571.25

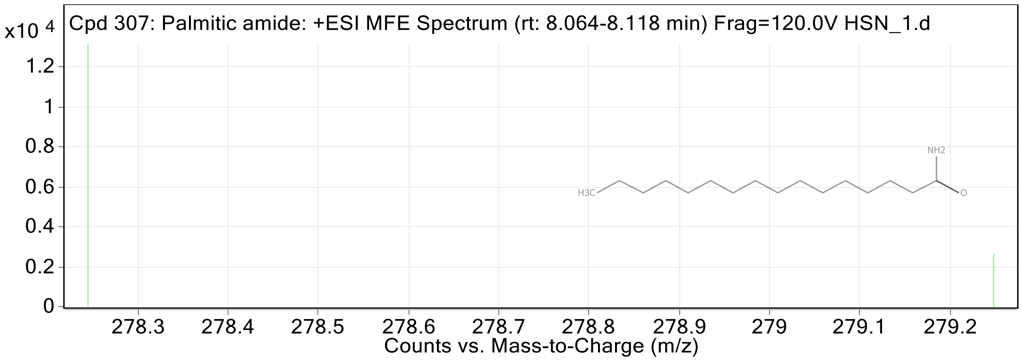

Peak List

m/z	z	Abund	Name	Formula	Ion	Score (DB)	Hits (DB)
278.24501	1	13184.85	Palmitic amide	C16 H33 N O	(M+Na)+	85.05	1
279.24835	1	2646.93		C16 H33 N O	(M+Na)+		

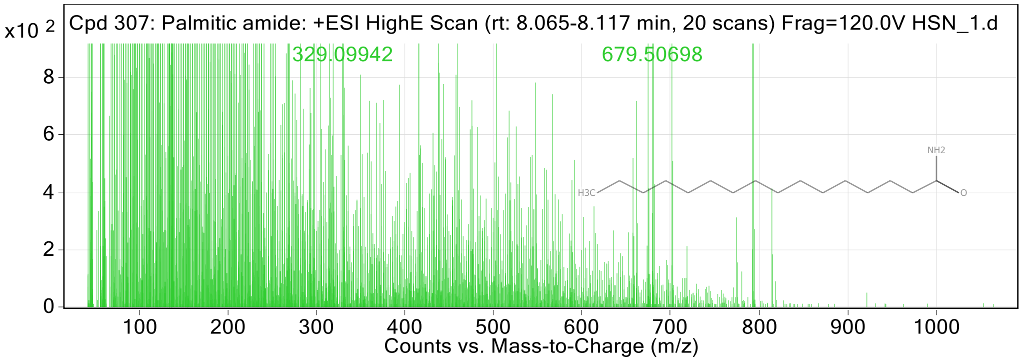

Peak List

m/z	z	Abund
45.03324		20696.18
59.04863		13292.66
81.06888		10818.35

89.05856		20018.29
93.06859		15662.85
107.084		14081.25
119.08375		14738.69
135.11471		12163.2
153.1252		10576.93
329.09942	1	27227.41

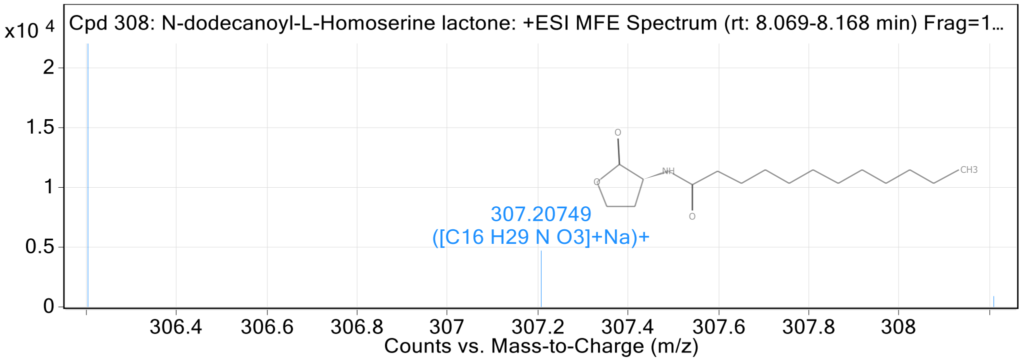

Peak List

m/z	z	Abund	Name	Formula	Ion	Score (DB)	Hits (DB)
306.20377	1	22074.71	N-dodecanoyl-L-Homoserine lactone	C16 H29 N O3	(M+Na)+	96.7	3
307.20749	1	4719.69		C16 H29 N O3	(M+Na)+		
308.20959	1	906.53		C16 H29 N O3	(M+Na)+		

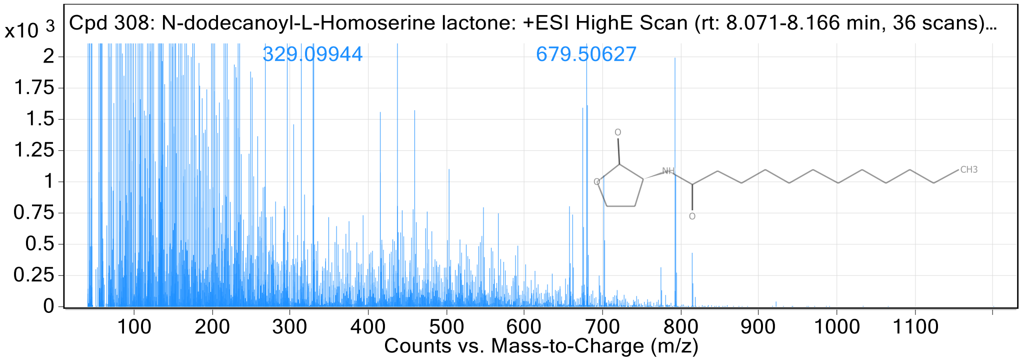

Peak List

m/z	z	Abund
45.03322		21260.23
59.04864		16060.27
81.06888		11781.48
89.05857		19940.45
93.06863		16979
107.08393		14742.87
119.0837		14693.49
135.11475		12861.12
153.12521		11005.45
329.09944	1	14365.84

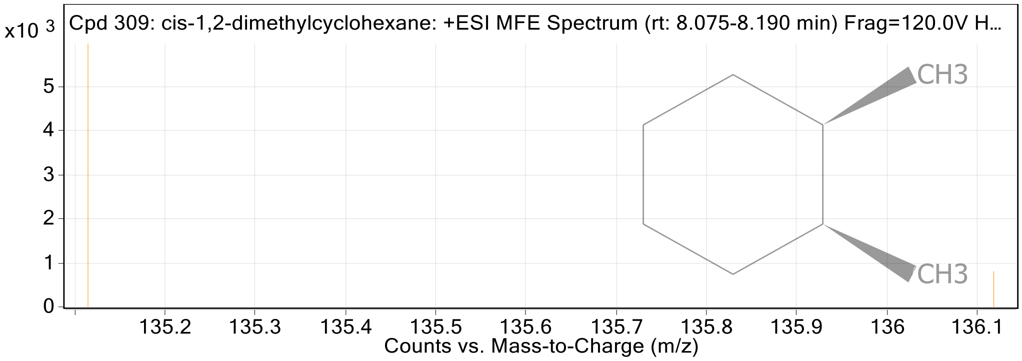

Peak List

m/z	z	Abund	Name	Formula	Ion	Score (DB)	Hits (DB)
135.11493	1	5976.65	cis-1,2-dimethylcyclohexane	C8 H16	(M+Na)+	79.78	6
136.11814	1	810.22		C8 H16	(M+Na)+		

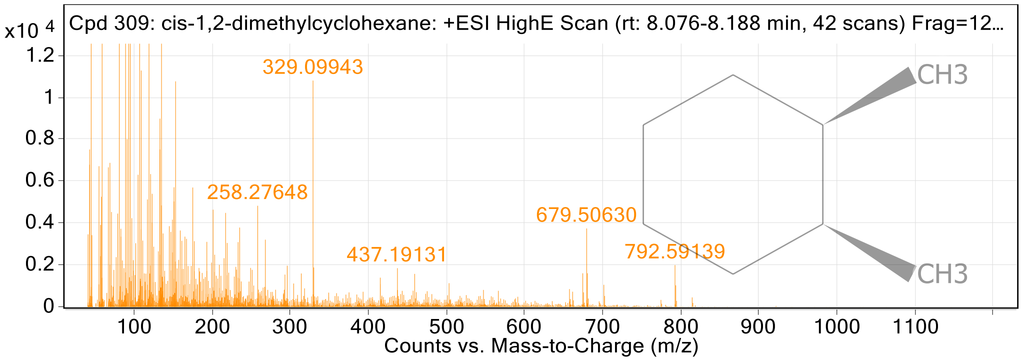

Peak List

m/z	Abund
45.03322	21088.15
59.04863	16304.57
81.06888	14124.01
89.05857	19858.64
93.06863	16864.71
95.08421	15280.35
107.08393	14334.84
109.09946	11277.07
119.08368	14786.76
135.11474	12561.02

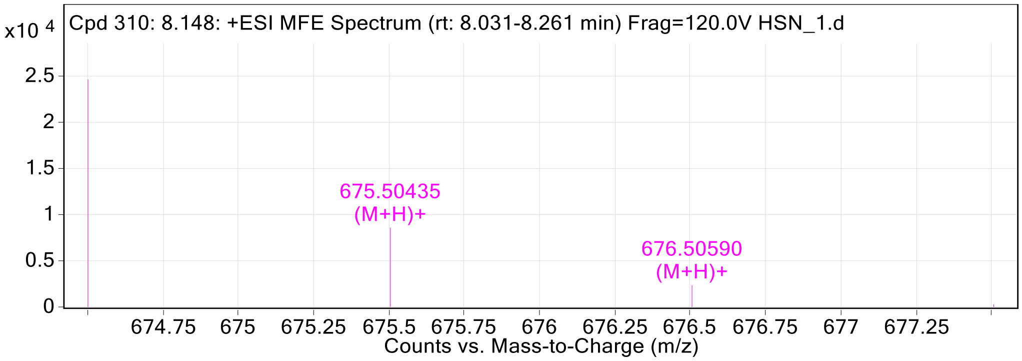

Peak List

m/z	z	Abund	Ion
674.50085	1	24568.5	(M+H)+
675.50435	1	8559.17	(M+H)+
676.5059	1	2354.24	(M+H)+

677.50754	1	293.8	(M+H)+

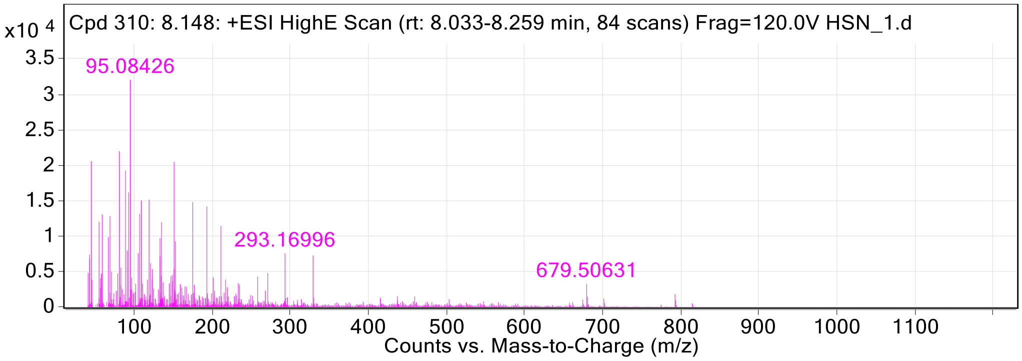

Peak List

m/z	z	Abund
45.03323		20554.56
81.0689		21940.82
89.05858		19230.08
93.06862		16159.01
95.08426	1	32047.09
109.09952		15014.43
119.08373	1	15126.22
151.14603	1	20470.71
175.14536	1	14769.1
193.15639	1	14162.47

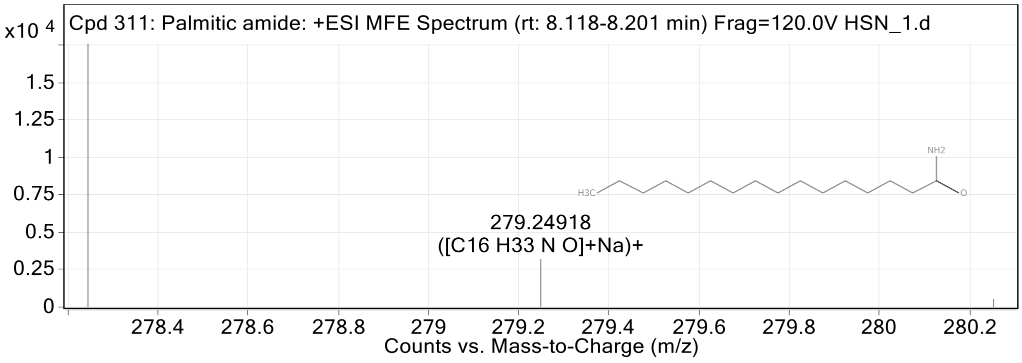

Peak List

m/z	z	Abund	Name	Formula	Ion	Score (DB)	Hits (DB)
278.24487	1	17604.94	Palmitic amide	C16 H33 N O	(M+Na)+	97.06	1
279.24918	1	3222.36		C16 H33 N O	(M+Na)+		
280.25359	1	544.65		C16 H33 N O	(M+Na)+		

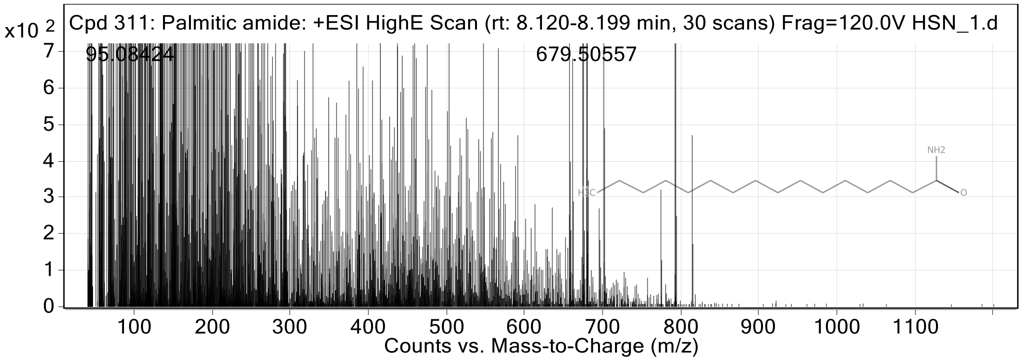

Peak List

m/z	z	Abund
45.03321		21327.08

59.04864		17184.23
81.06888		20816.4
89.05858		19562.24
93.06864		17037.29
95.08424	1	28168.15
107.08388		13736.77
109.09948		14277.53
119.0837	1	15135.8
151.14591	1	15849.04

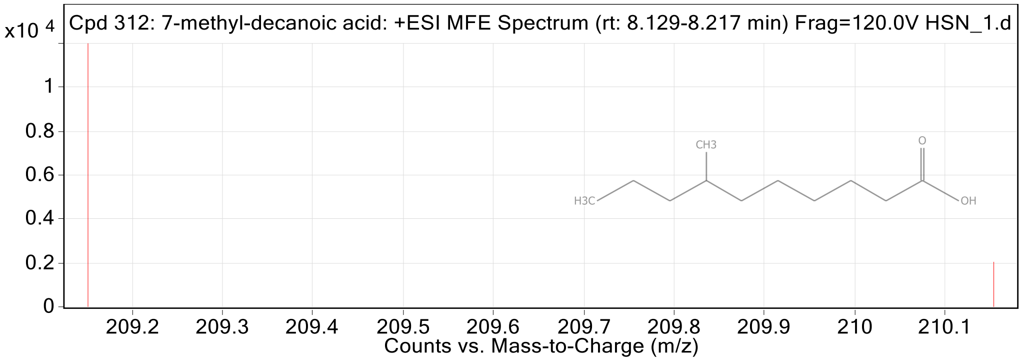

Peak List

m/z	z	Abund	Name	Formula	Ion	Score (DB)	Hits (DB)
209.15083	1	12012	7-methyl-decanoic acid	C11 H22 O2	(M+Na)+	80.58	10
210.15434	1	2050.92		C11 H22 O2	(M+Na)+		

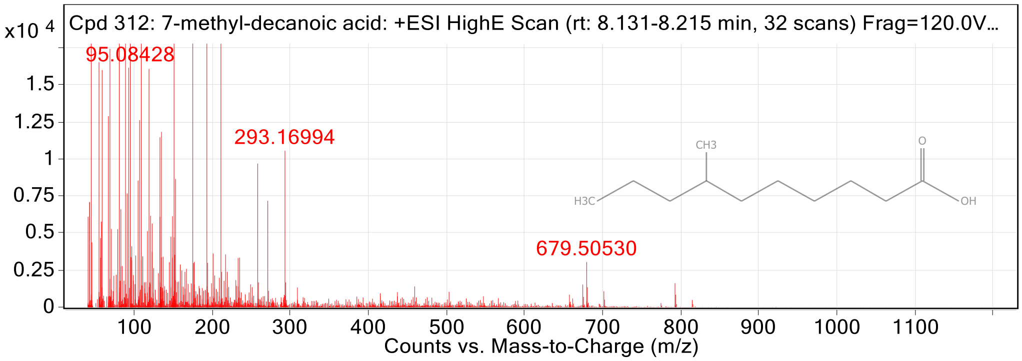

Peak List

m/z	z	Abund
45.03319		20476.02
69.06915		17439.74
81.0689	1	28381.5
89.0586		19096.46
95.08428	1	45214.63
109.09954		17965.62
151.14604	1	31650.45
175.14562	1	21298.53
193.15644	1	22075.93
211.16702	1	17797.92

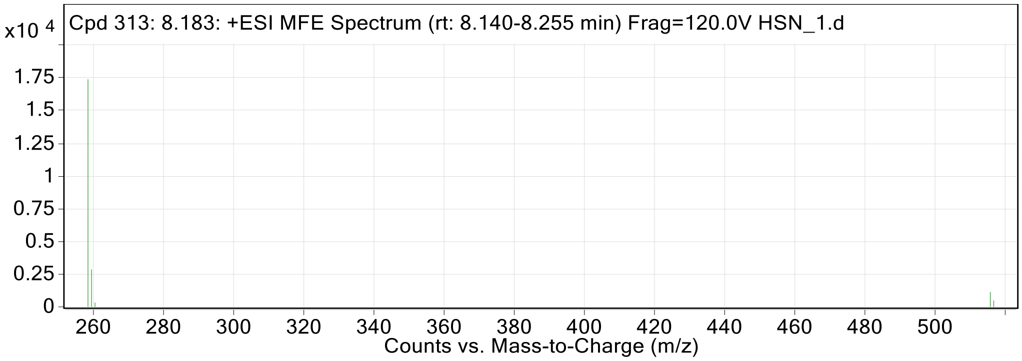

Peak List

m/z	z	Abund	Ion
258.27642	1	17395.19	(M+H)+
259.27988	1	2864.72	(M+H)+
260.28417	1	328.8	(M+H)+
515.54713	1	1128.33	(2M+H)+
516.55233	1	485.46	(2M+H)+

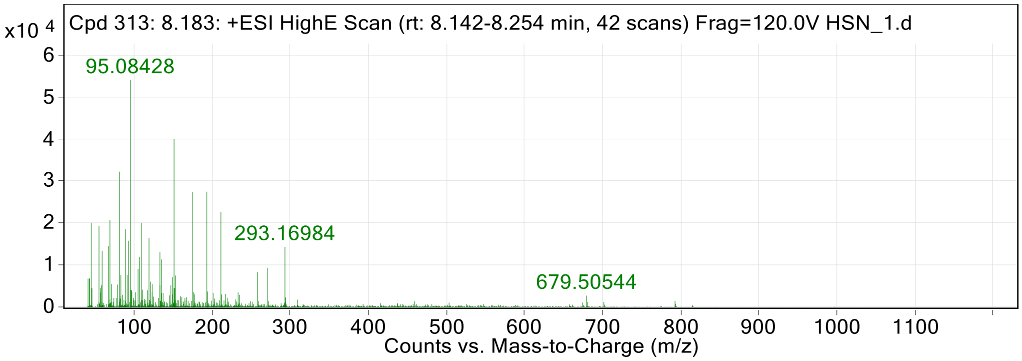

Peak List

m/z	z	Abund
45.03321		19978.85
55.05379		19334.44
69.06914		20794.27
81.06891	1	32289.07
95.08428	1	54167.22
109.09956	1	20090.85
151.14605	1	40044.41
175.14554	1	27439.53
193.15642	1	27506.55
211.16703	1	22601.36

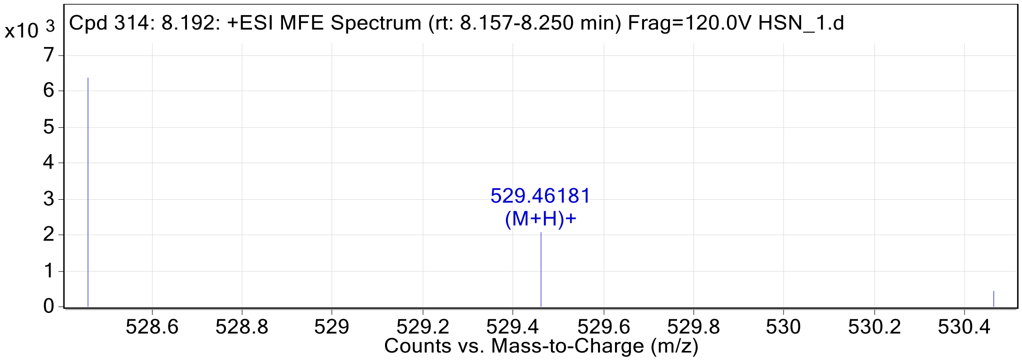

Peak List

m/z	z	Abund	Ion

528.45857	1	6377.86	(M+H)+
529.46181	1	2074.81	(M+H)+
530.46445	1	442.37	(M+H)+

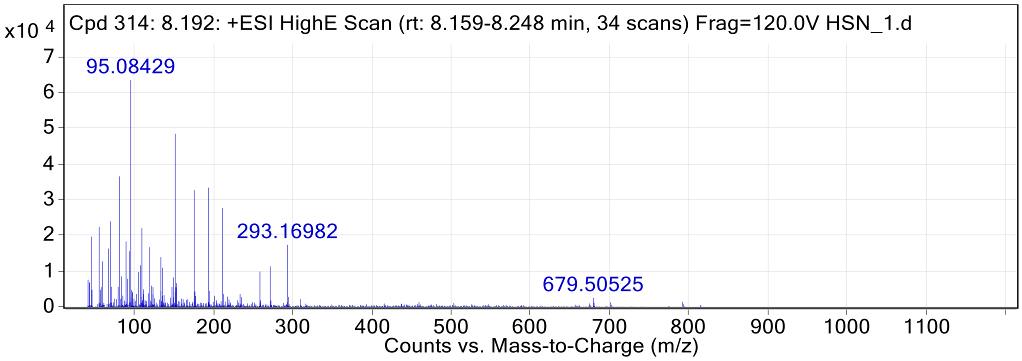

Peak List

m/z	z	Abund
45.0332		19619.56
55.05379		22345.19
69.06914		23911.95
81.06891	1	36473.19
95.08429	1	63427.02
109.09958	1	21968.55
151.14605	1	48392.18
175.14567	1	32618.82
193.15642	1	33314.44
211.16704	1	27627.22

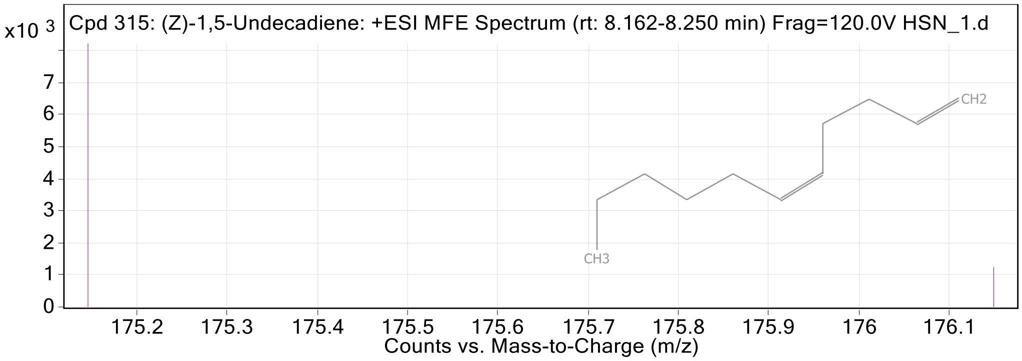

Peak List

m/z	z	Abund	Name	Formula	Ion	Score (DB)	Hits (DB)
175.14575	1	8215.1	(Z)-1,5-Undecadiene	C11 H20	(M+Na)+	85.09	4
176.14945	1	1236.25		C11 H20	(M+Na)+		

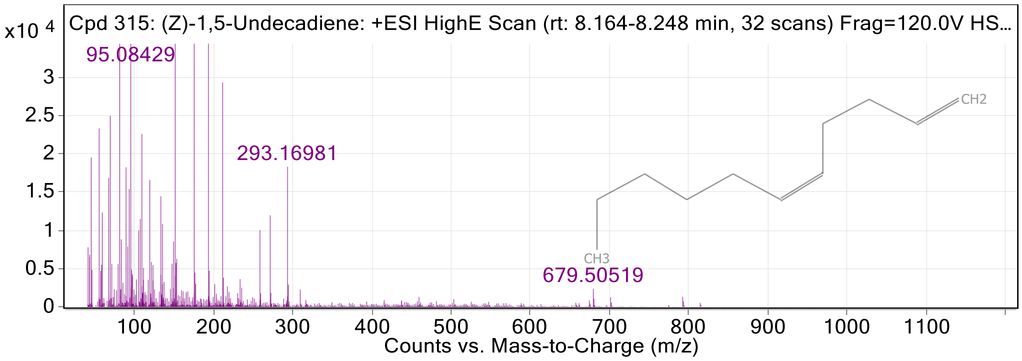

Peak List

m/z	z	Abund

45.03322		19505.5
55.05379		23315.13
69.06914		24961.32
81.06891	1	37856.75
95.08429	1	66486.89
109.09959	1	22589.46
151.14606	1	51241.91
175.1457	1	34378.8
193.15642	1	35271.5
211.16704	1	29303.79

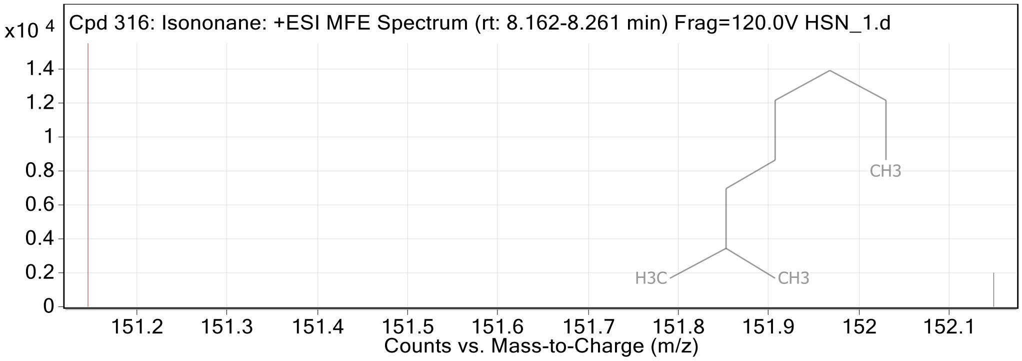

Peak List

m/z	z	Abund	Name	Formula	Ion	Score (DB)	Hits (DB)
151.14598	1	15507.37	Isononane	C9 H20	(M+Na)+	84.76	10
152.14934	1	2007.12		C9 H20	(M+Na)+		

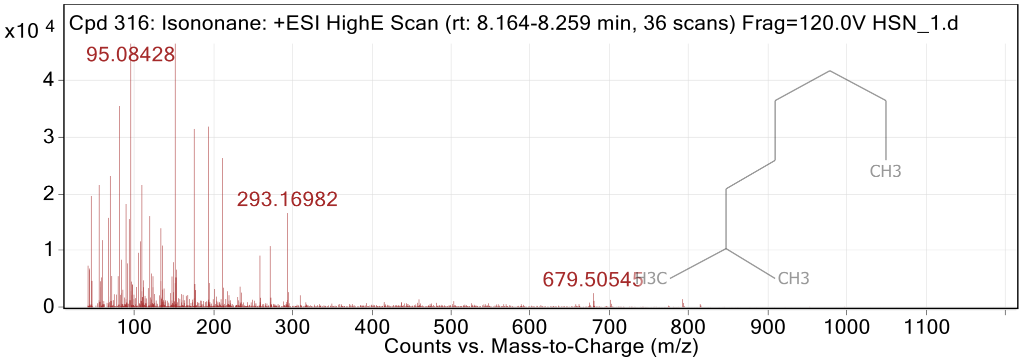

Peak List

m/z	z	Abund
45.03321		19657.78
55.05379		21602.85
69.06914		23192.24
81.06891	1	35492.7

m/z	z	Abund
95.08428	1	61258.74
109.09957	1	21561.3
151.14605	1	46551.57
175.14569	1	31439.79
193.15642	1	31888.38
211.16704	1	26287.93

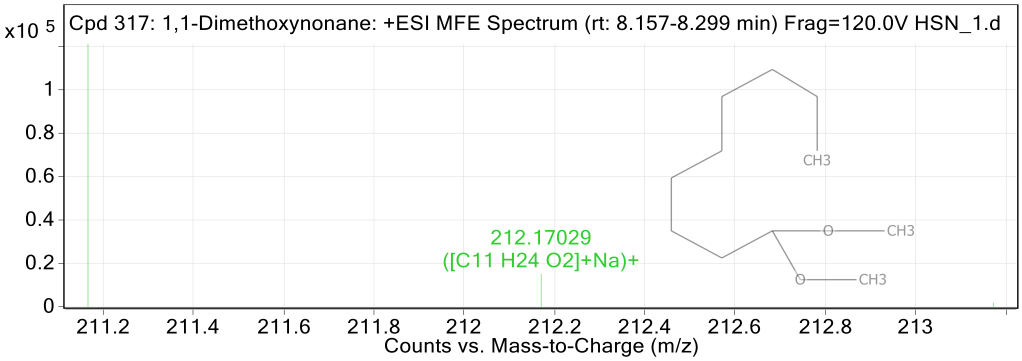

Peak List

m/z	z	Abund	Name	Formula	Ion	Score (DB)	Hits (DB)
211.16667	1	121544.93	1,1-Dimethoxynonane	C11 H24 O2	(M+Na)+	99.69	2
212.17029	1	15152.61		C11 H24 O2	(M+Na)+		
213.17284	1	1933.05		C11 H24 O2	(M+Na)+		

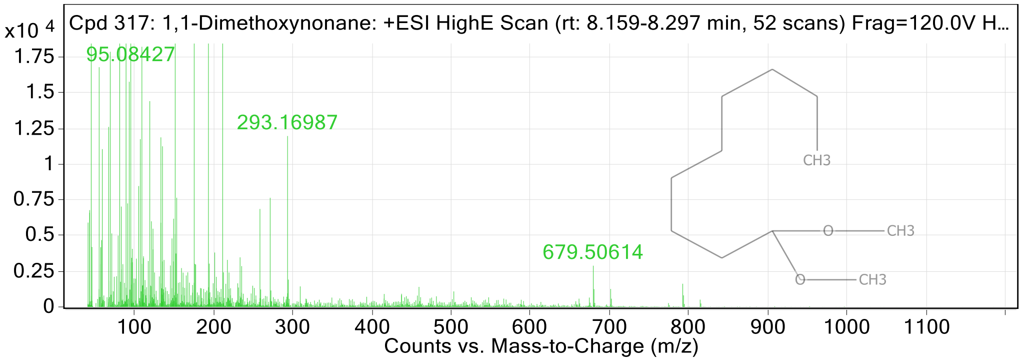

Peak List

m/z	z	Abund
45.03321		19863.64
69.06914		17873.33
81.0689	1	28599.41
89.05857		18502.4
95.08427	1	46488.79
109.09954	1	18224.11
151.14603	1	33444.12
175.14565	1	23068.22
193.15641	1	22715.12
211.16703	1	18456.82

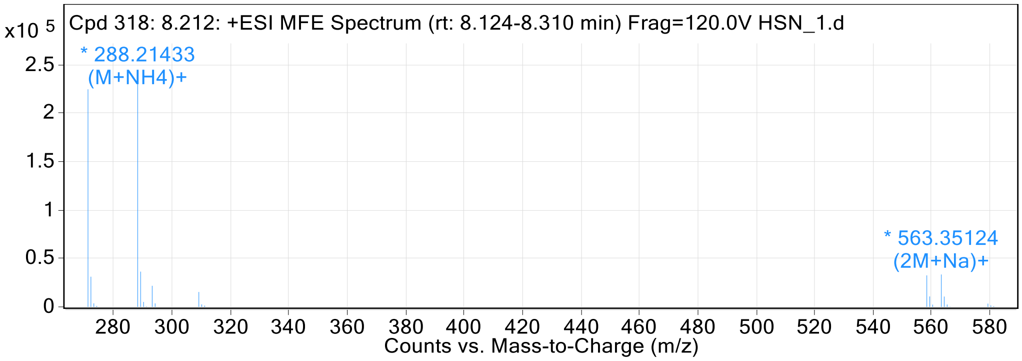

Peak List

m/z	z	Abund	Ion
271.18753	1	224678.16	(M+H)+
272.19117	1	31158.56	(M+H)+
288.21433	1	234465.89	(M+NH4)+
289.21748	1	36387.8	(M+NH4)+
293.16995	1	21768.4	(M+Na)+
309.1438	1	15370.99	(M+K)+
558.39615	1	32532.71	(2M+NH4)+
559.39947	1	10775.8	(2M+NH4)+
563.35124	1	33452.5	(2M+Na)+
564.35506	1	10665	(2M+Na)+

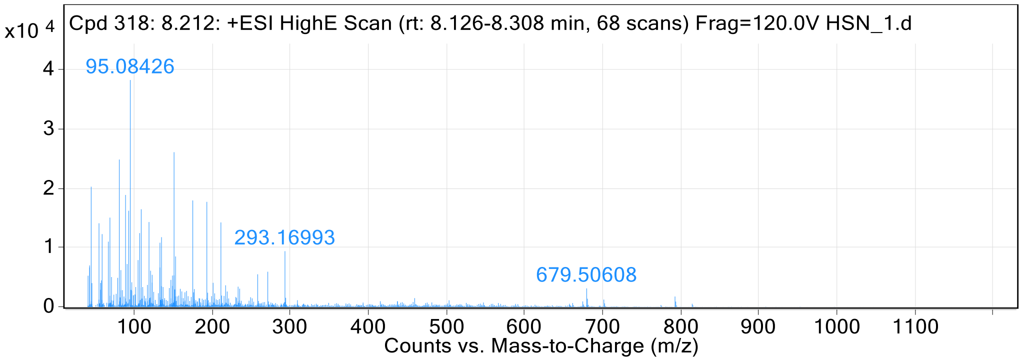

Peak List

m/z	z	Abund
45.03321		20245.44
69.06914		15032.96
81.0689	1	24834.56
89.05857		18856.08
93.06863		16192.55
95.08426	1	38247.04
109.09952		16447.81
151.14602	1	26071.86
175.14565	1	17937.67
193.1564	1	17691.03

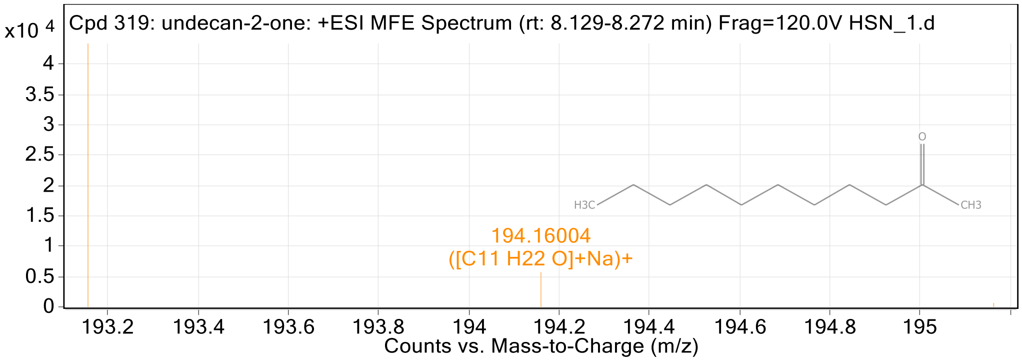

Peak List

m/z	z	Abund	Name	Formula	Ion	Score (DB)	Hits (DB)
193.15618	1	43469.58	undecan-2-one	C11 H22 O	(M+Na)+	99.18	10
194.16004	1	5730.66		C11 H22 O	(M+Na)+		
195.16345	1	650.76		C11 H22 O	(M+Na)+		

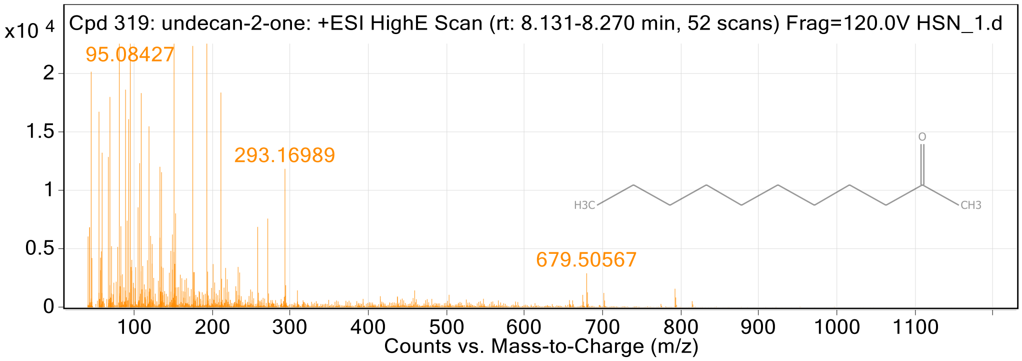

Peak List

m/z	z	Abund
45.03321		20196.13
69.06915		18032.84
81.06891	1	28702.41
89.05859		18662.69
95.08427	1	46478.75
109.09953	1	18366.09
151.14604	1	33119.2
175.14567	1	22410.62
193.15642	1	22611.37
211.16703	1	18414.61

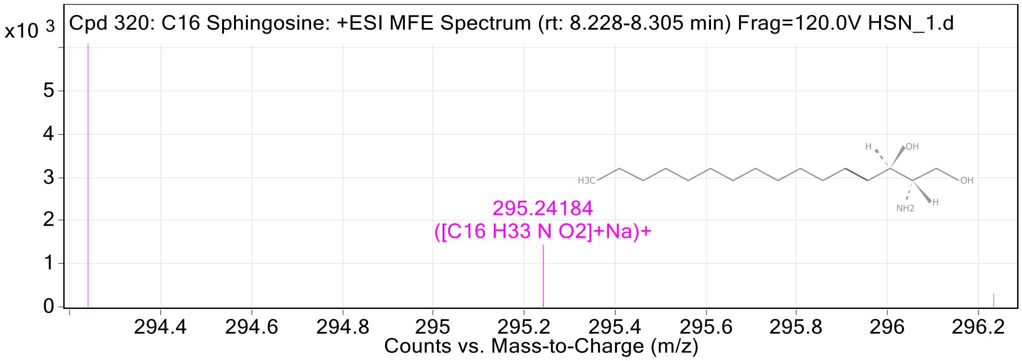

Peak List

m/z	z	Abund	Name	Formula	Ion	Score (DB)	Hits (DB)
294.2398	1	6103.98	C16 Sphingosine	C16 H33 N O2	(M+Na)+	76.68	8
295.24184	1	1440.26		C16 H33 N O2	(M+Na)+		

296.23358	1	299.31		C16 H33 N O2	(M+Na)+		

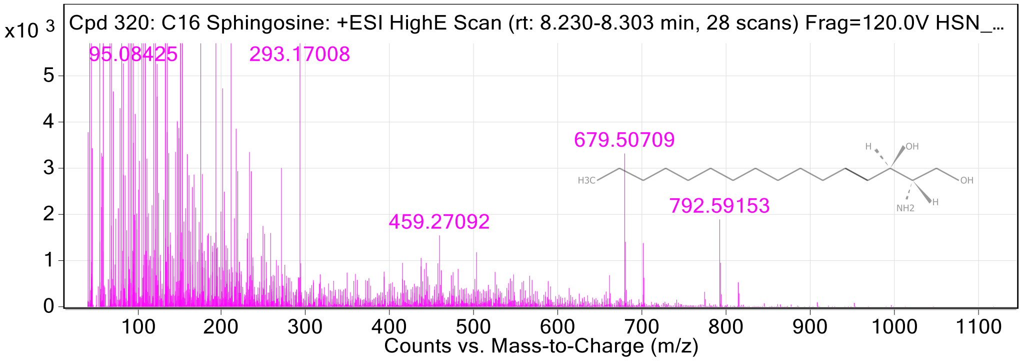

Peak List

m/z	z	Abund
45.03323		19959.7
81.06887		18824.87
89.05857		18687
93.06863		16131.26
95.08425	1	25563.72
107.08386		12007.8
109.09951		13648.47
119.08367		12011.7
135.11483		11696.55
151.14595	1	15084.18

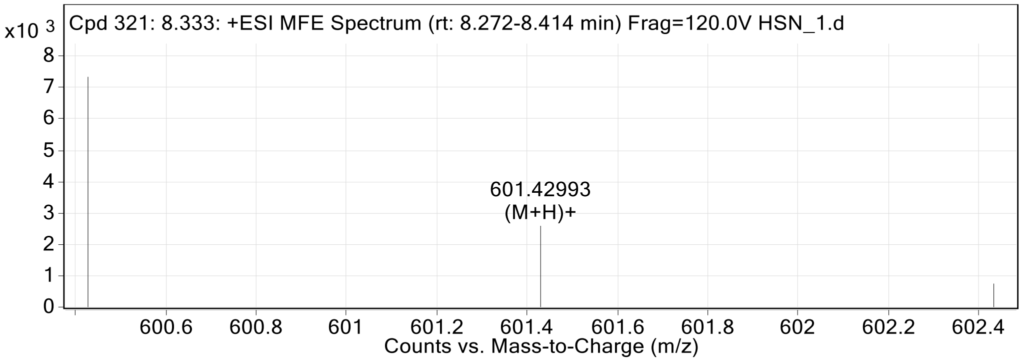

Peak List

m/z	z	Abund	Ion
600.42803	1	7343.74	(M+H)+
601.42993	1	2591.97	(M+H)+
602.433	1	748.43	(M+H)+

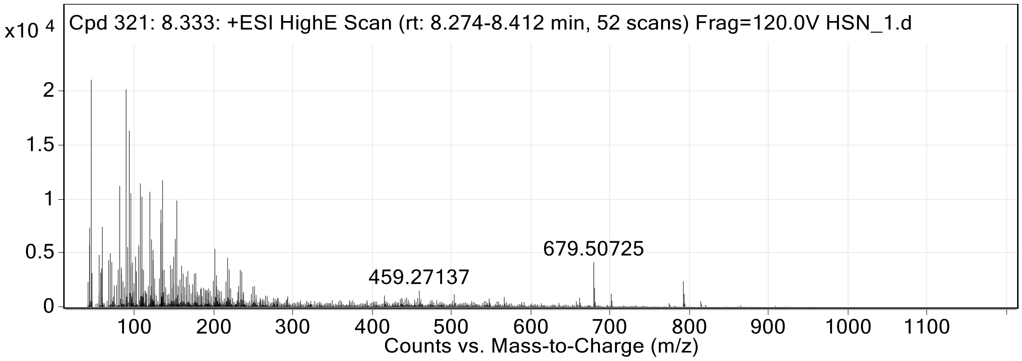

Peak List

m/z	Abund
45.03319	21060.54

81.06882	11202.01
89.05851	20158.35
93.06856	16339.96
95.08416	10518.95
107.08385	11446.08
109.09941	10213.14
119.08362	10676.54
135.11474	11720.86
153.12511	9858.17

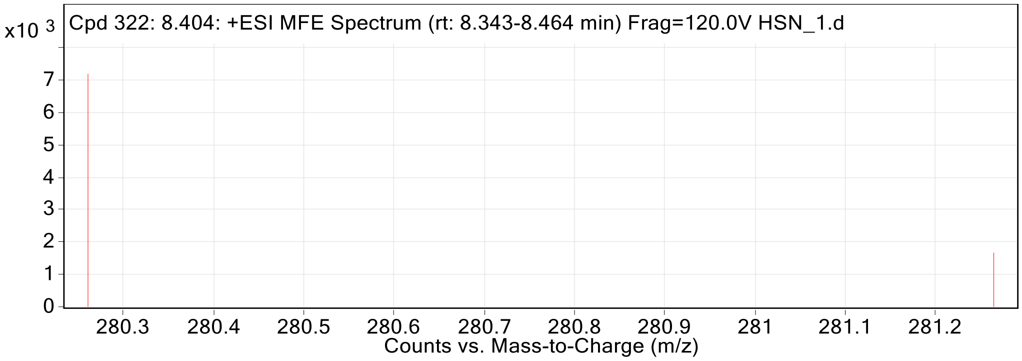

Peak List

m/z	z	Abund	Ion
280.26064	1	7189.48	(M+H)+
281.26447	1	1668.91	(M+H)+

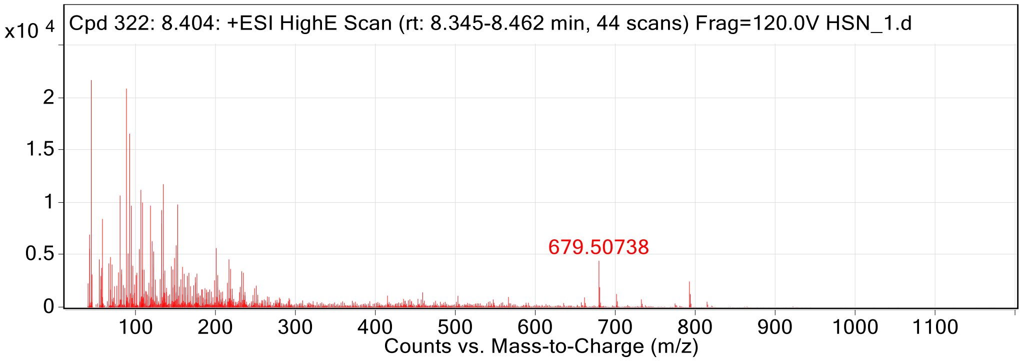

Peak List

m/z	Abund
45.03318	21644.31
81.06885	10634.86
89.0585	20825.41
93.06859	16529.44
95.08415	9658.01
107.0839	11172.04
109.09944	9964.41
119.08362	9670.22
135.11468	11721.24
153.12509	9778.75

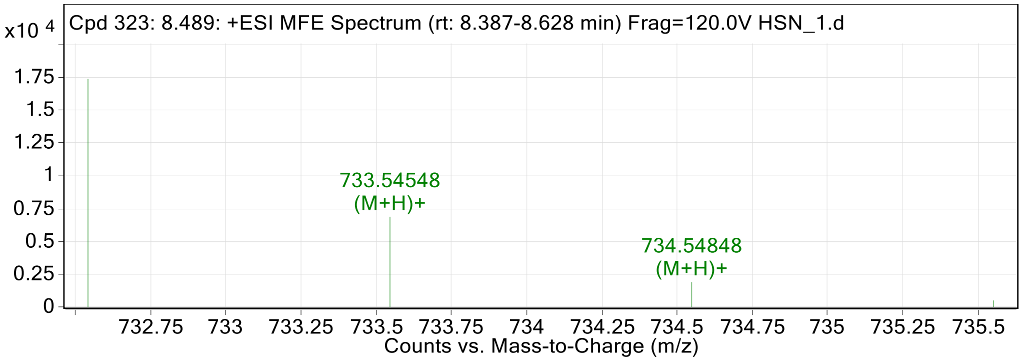

Peak List

m/z	z	Abund	Ion
732.54191	1	17389.89	(M+H)+
733.54548	1	6876.86	(M+H)+
734.54848	1	1892.77	(M+H)+
735.55191	1	491.07	(M+H)+

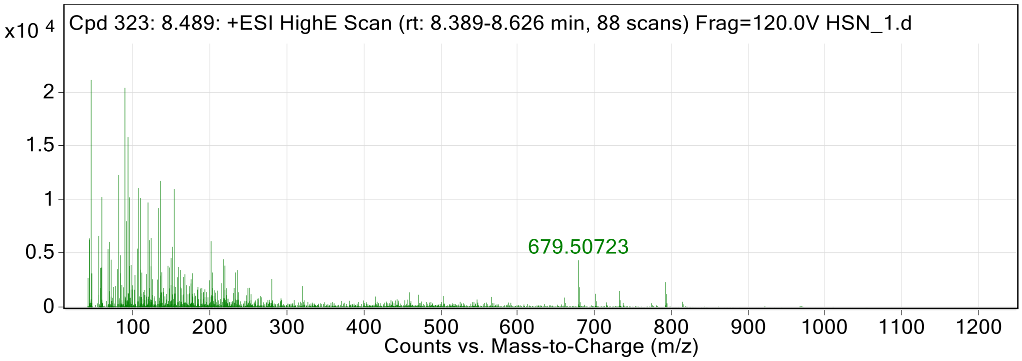

Peak List

m/z	Abund
45.03321	21096.91
59.04861	10244.45
81.06888	12272.92
89.05852	20363.97
93.06862	15777.53
95.08412	10186.15
107.08389	11039.17
109.09952	10125.78
135.11472	11735.14
153.12517	10960.8

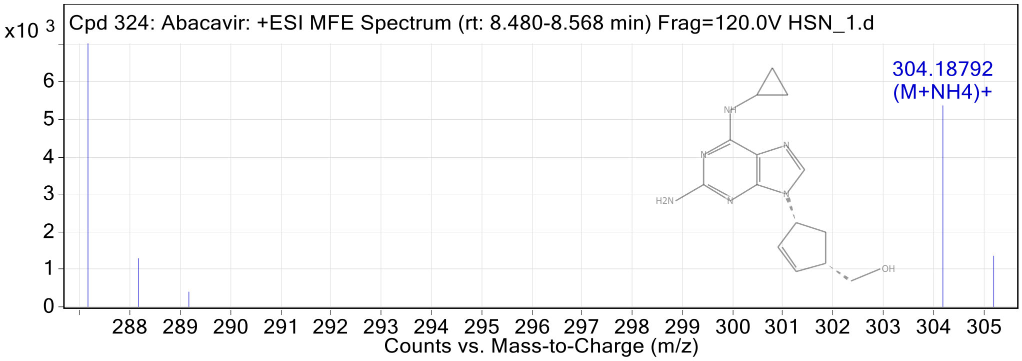

Peak List

m/z	z	Abund	Name	Formula	Ion	Score (DB)	Hits (DB)
287.16147	1	7026.95	Abacavir	C14 H18 N6 O	(M+H)+	89.8	1

288.16505	1	1295.29		C14 H18 N6 O	(M+H)+		
289.17109	1	403.34		C14 H18 N6 O	(M+H)+		
304.18792	1	5372.51			(M+NH4)+		
305.20081	1	1362.43			(M+NH4)+		

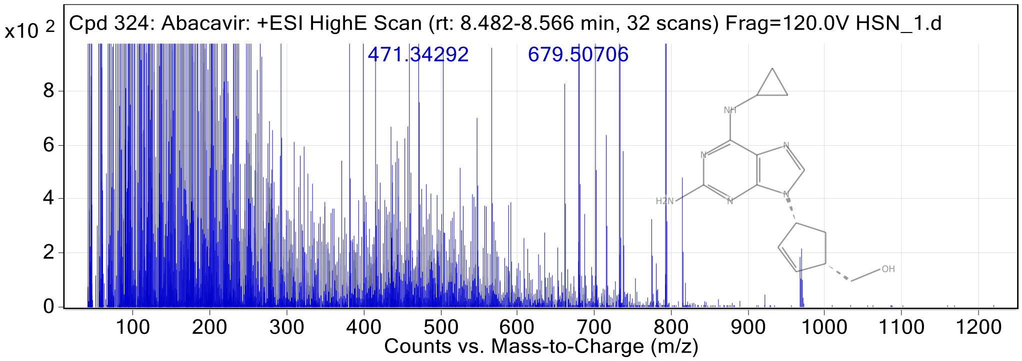

Peak List

m/z	Abund
45.03319	20843.8
59.04859	12451.07
81.06891	12062.77
89.05851	20040.13
93.06862	15796.02
107.08385	11539.84
109.09953	9603.97
119.08377	11870.39
135.11475	12151.83
153.12516	13383.28

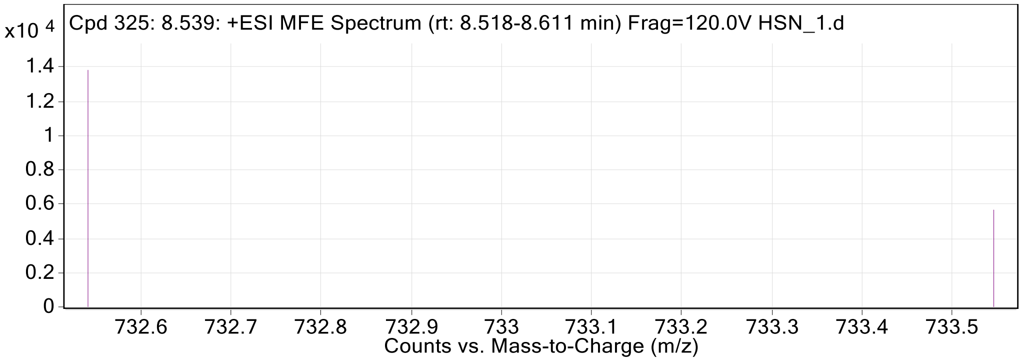

Peak List

m/z	z	Abund	Ion
732.54159	1	13833.1	(M+H)+
733.54606	1	5671.76	(M+H)+

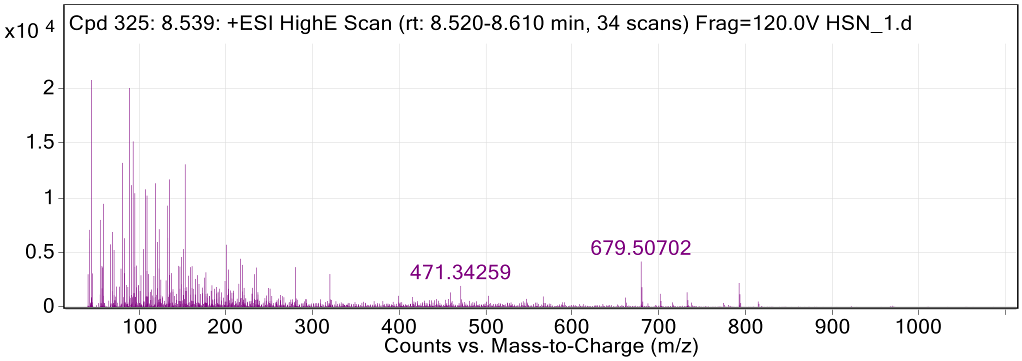

Peak List

m/z	Abund
45.03324	20791.45
81.0689	13193.45
89.05853	20064.18
91.05308	11148.45
93.0686	15160.73
95.08411	10408.53
107.08391	10767.99
119.0838	11321.54
135.11474	11675.61
153.12522	13058.59

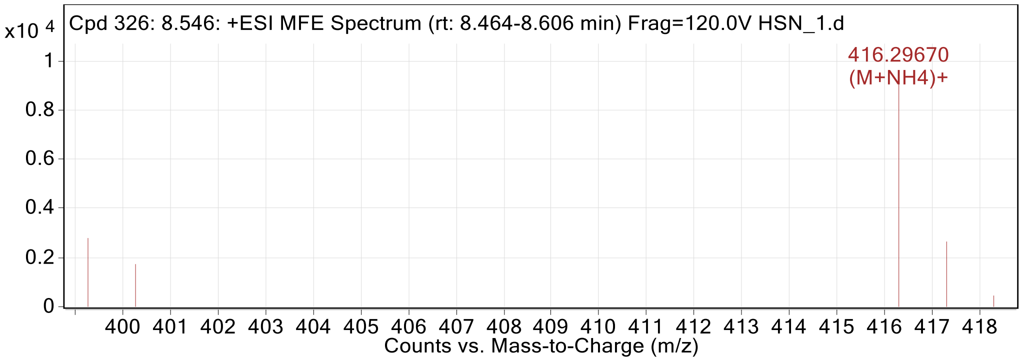

Peak List

m/z	z	Abund	Ion
399.27002	1	2796.04	(M+H)+
400.26882	1	1735.62	(M+H)+
416.2967	1	9284.89	(M+NH4)+
417.30104	1	2653.28	(M+NH4)+
418.2906	1	455.95	(M+NH4)+

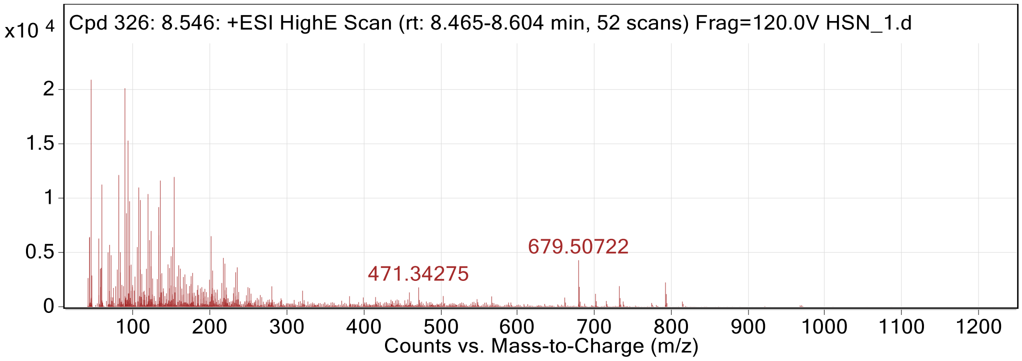

Peak List

m/z	Abund
45.03321	20861.51
59.0486	11229.16
81.06888	12107.11
89.05852	20081.77
93.06864	15267.88
107.08389	10960.71
109.09953	9809.31
119.0838	10358.95
135.11474	11596.99
153.12522	11927.7

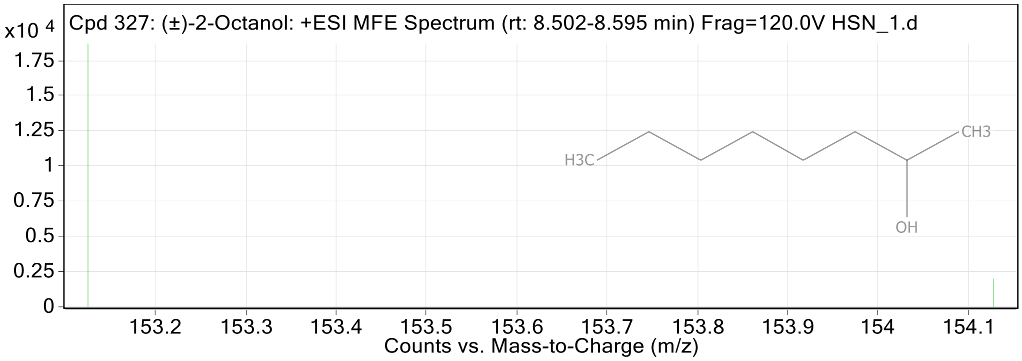

Peak List

m/z	z	Abund	Name	Formula	Ion	Score (DB)	Hits (DB)
153.12524	1	18710.42	(±)-2-Octanol	C8 H18 O	(M+Na)+	85.9	10
154.12818	1	2008.32		C8 H18 O	(M+Na)+		

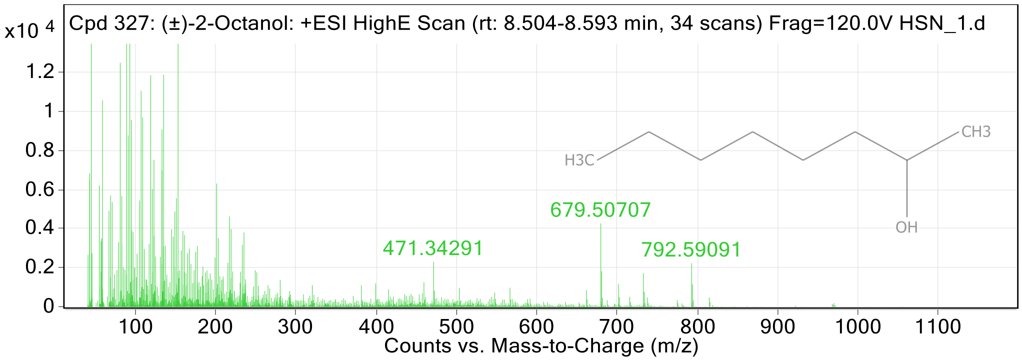

Peak List

m/z	Abund
45.03321	20824.37
59.04859	10565.21
81.0689	12488.98
89.05853	19873.48
93.06859	15360.92
107.08389	11051.49
109.09952	9690.93
119.08378	11851.12
135.11471	11867.13
153.1252	13468.79

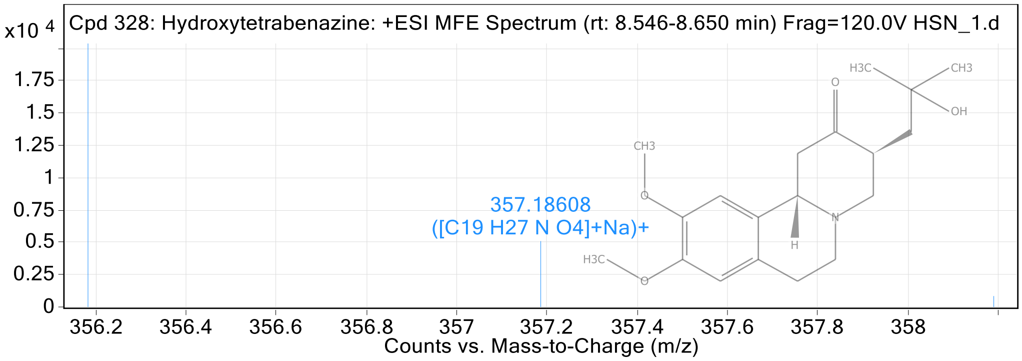

Peak List

m/z	z	Abund	Name	Formula	Ion	Score (DB)	Hits (DB)
356.18299	1	20384.92	Hydroxytetrabenazine	C19 H27 N O4	(M+Na)+	96.98	2
357.18608	1	5093.82		C19 H27 N O4	(M+Na)+		
358.18975	1	844.55		C19 H27 N O4	(M+Na)+		

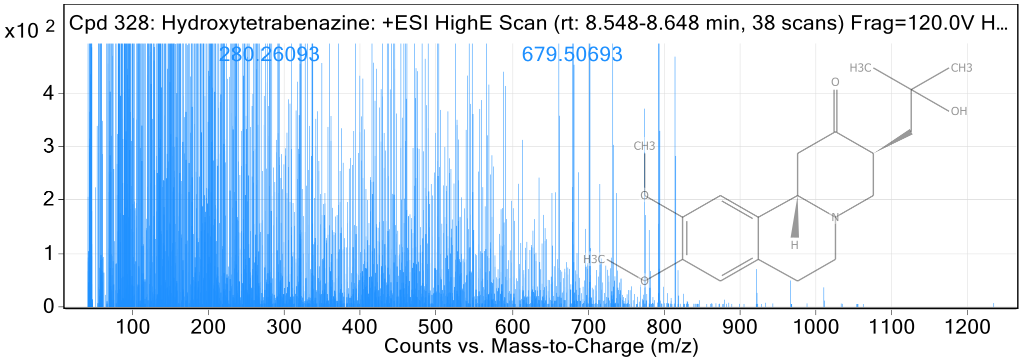

Peak List

m/z	Abund
45.03327	20369.46
55.05379	11919.63
81.06887	15745.16
89.05855	19828.58
91.05303	11702.9
93.06861	15314.49
95.08416	13521.4
109.09956	12434.06
135.11481	11380.54
153.12523	11306.41

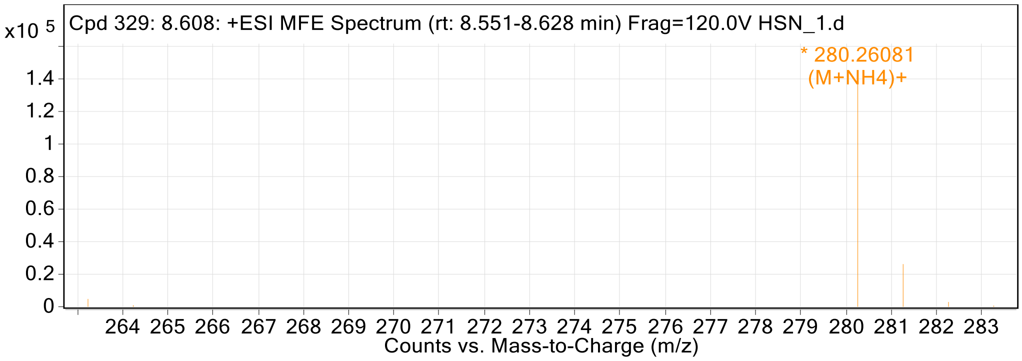

Peak List

m/z	z	Abund	Ion
263.23429	1	4815.92	(M+H)+
264.23595	1	945.32	(M+H)+
280.26081	1	139313.59	(M+NH4)+
281.26399	1	26206.22	(M+NH4)+
282.26733	1	2958.1	(M+NH4)+
283.26872	1	724.59	(M+NH4)+

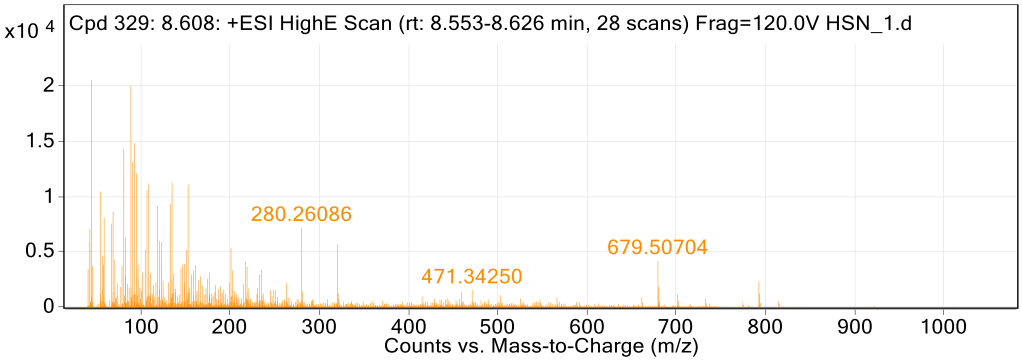

Peak List

m/z	Abund
45.03326	20525.26
81.06887	14334.9
89.05853	20046.28
91.05303	13122.58
93.0686	14821.92
95.0841	12064.79
107.08391	10542.85
109.09956	11169.5
135.11478	11255.74
153.12523	11050.37

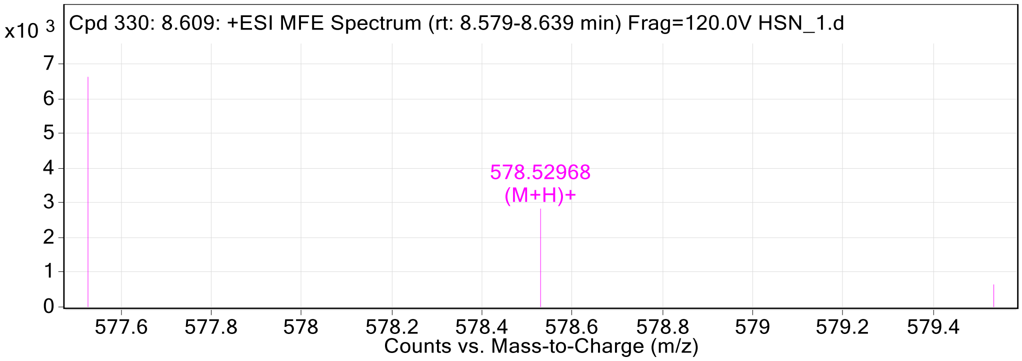

Peak List

m/z	z	Abund	Ion
577.52636	1	6638.68	(M+H)+
578.52968	1	2836.23	(M+H)+
579.53432	1	649.8	(M+H)+

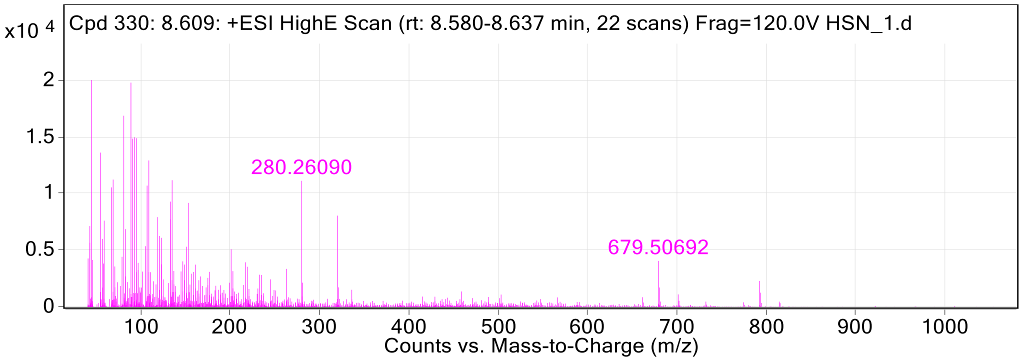

Peak List

m/z	Abund
45.03329	20010.81
55.05379	13605.31
69.06911	11224.77
81.06885	16857.94
89.05856	19782.17
91.05303	14832.61
93.0686	14951.75
95.08415	14875.69
109.09955	12912.49
135.11476	11168.54

Qualitative Analysis Report

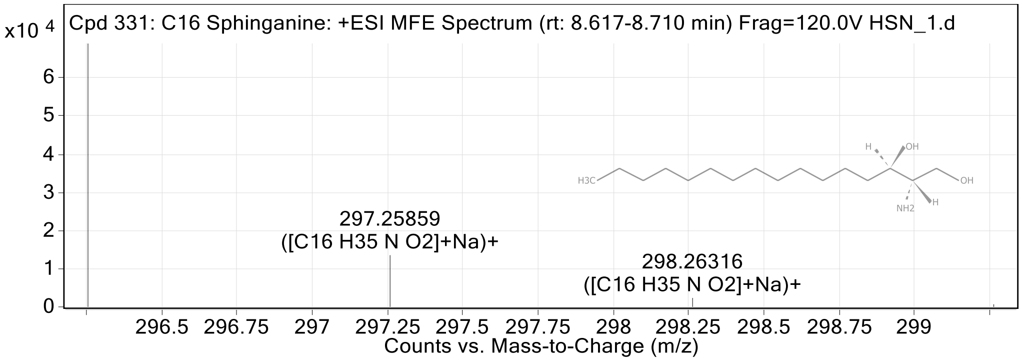

Peak List

m/z	z	Abund	Name	Formula	Ion	Score (DB)	Hits (DB)
296.25536	1	69006.46	C16 Sphinganine	C16 H35 N O2	(M+Na)+	97.3	1
297.25859	1	13545.97		C16 H35 N O2	(M+Na)+		
298.26316	1	2339.09		C16 H35 N O2	(M+Na)+		
299.26339	1	681.01		C16 H35 N O2	(M+Na)+		

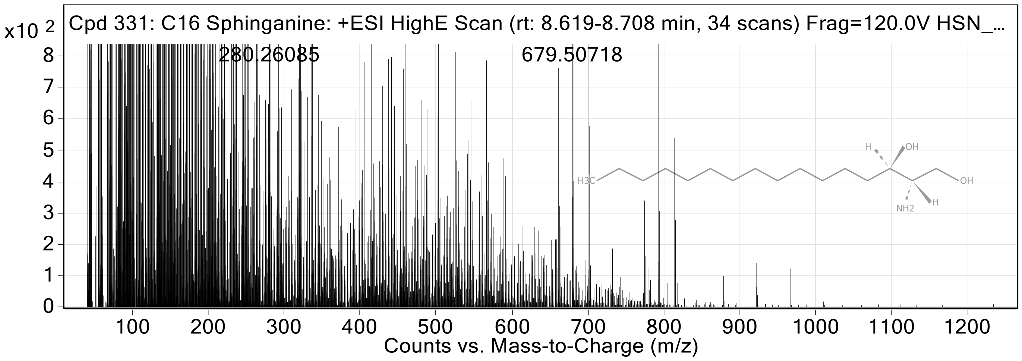

Peak List

m/z	z	Abund
45.03331		19725.08
55.05381		15874.83
67.05356		14214.04
69.06913		13042.56
81.06892		18852.28
89.05859		18983.57
93.06866		15741.71
95.0842		16780.74
109.09954		15854.94
280.26085	1	15413.39

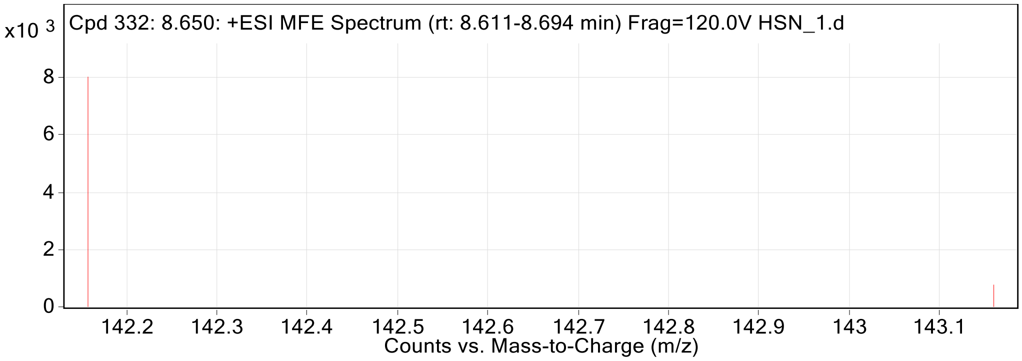

Peak List

m/z	z	Abund	Ion
142.15699	1	8033.57	(M+H)+

143.16058	1	774.15	(M+H)+

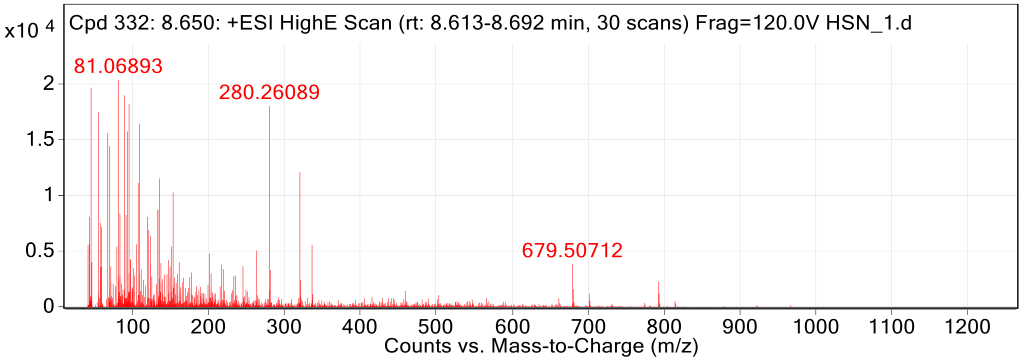

Peak List

m/z	z	Abund
45.0333		19721
55.05379		17500.95
67.05356		15618.88
69.06915		14462.98
81.06893		20436.63
89.05859		19030.1
93.06862		15785.01
95.08422		18225
109.09956		16484.63
280.26089	1	18081.68

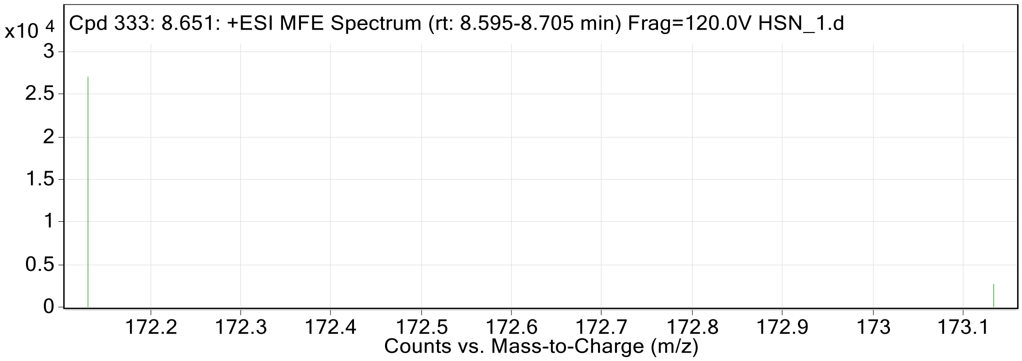

Peak List

m/z	z	Abund	Ion
172.13102	1	27051.48	(M+H)+
173.13396	1	2722.82	(M+H)+

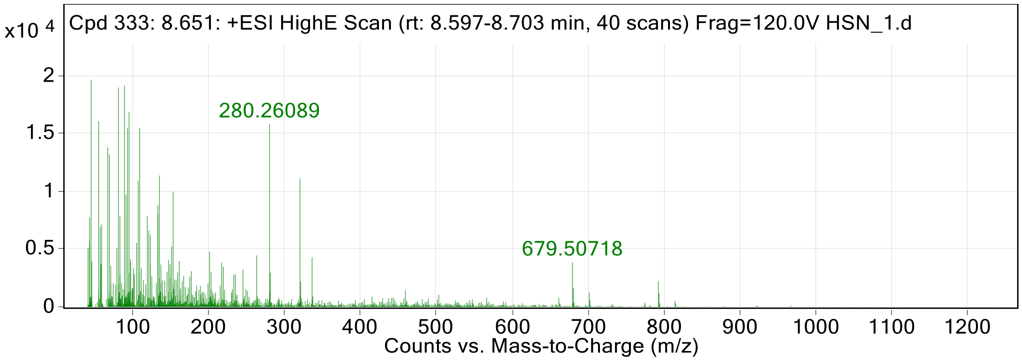

Peak List

m/z	z	Abund
45.0333		19654.93
55.0538		16074.82

67.05356		13809.15
69.06913		13178.16
81.06892		18987.52
89.05856		19152.54
93.06864		15515.53
95.08418		16870.11
109.09955		15492.6
280.26089	1	15814.87

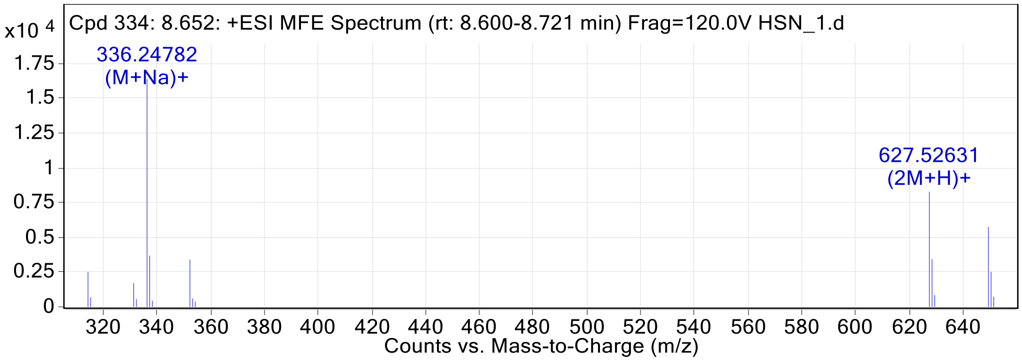

Peak List

m/z	z	Abund	Ion
314.26613	1	2522.91	(M+H)+
331.29239	1	1702.94	(M+NH4)+
336.24782	1	16361.24	(M+Na)+
337.24868	1	3681.49	(M+Na)+
352.22303	1	3399.38	(M+K)+
627.52631	1	8279.25	(2M+H)+
628.52834	1	3429.79	(2M+H)+
629.5337	1	843.99	(2M+H)+
649.50834	1	5747.36	(2M+Na)+
650.50943	1	2523.8	(2M+Na)+

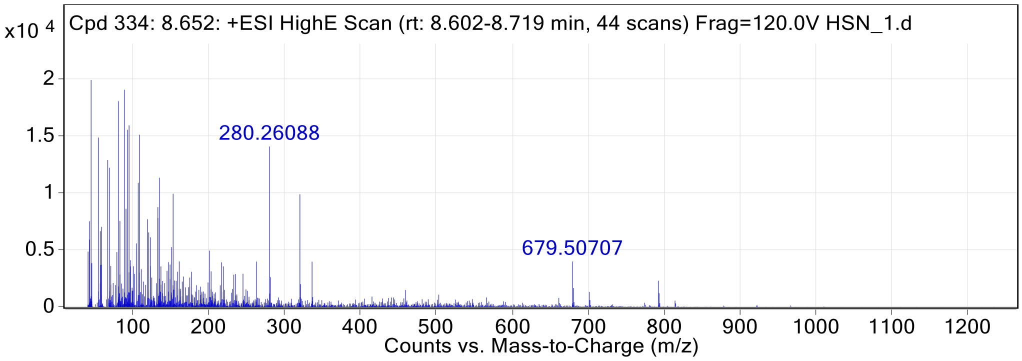

Peak List

m/z	z	Abund
45.0333		19889.74
55.0538		14840.17
67.05355		12875.17
69.06914		12191.04
81.06892		18036.57
89.05857		19038.42
93.06864		15524.76
95.08419		15915.08
109.09956		15085.98
280.26088	1	14058.81

Qualitative Analysis Report

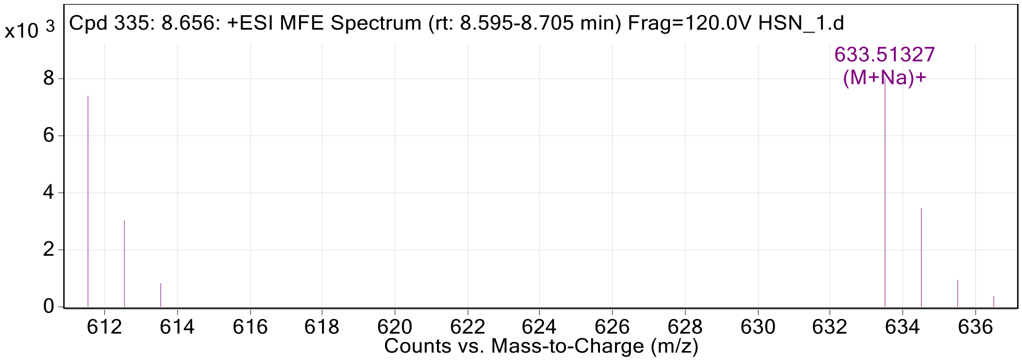

Peak List

m/z	z	Abund	Ion
611.53175	1	7392.75	(M+H)+
612.53558	1	3022.65	(M+H)+
613.53912	1	830.25	(M+H)+
633.51327	1	7992.79	(M+Na)+
634.5154	1	3453.4	(M+Na)+
635.51884	1	946.03	(M+Na)+
636.51283	1	373.74	(M+Na)+

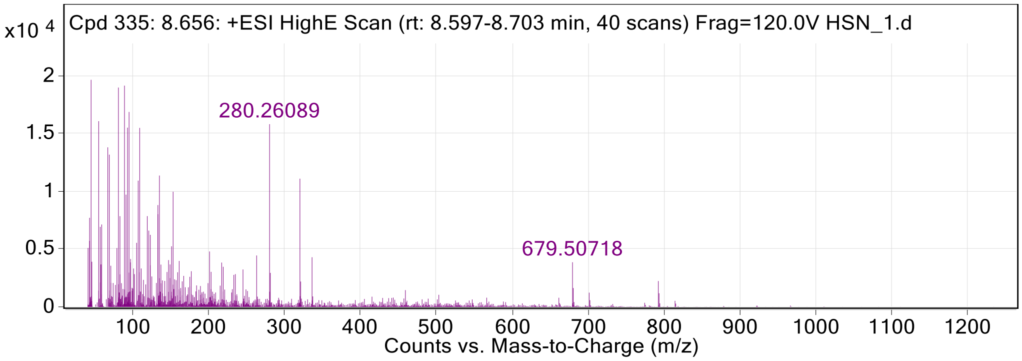

Peak List

m/z	z	Abund
45.0333		19654.93
55.0538		16074.82
67.05356		13809.15
69.06913		13178.16
81.06892		18987.52
89.05856		19152.54
93.06864		15515.53
95.08418		16870.11
109.09955		15492.6
280.26089	1	15814.87

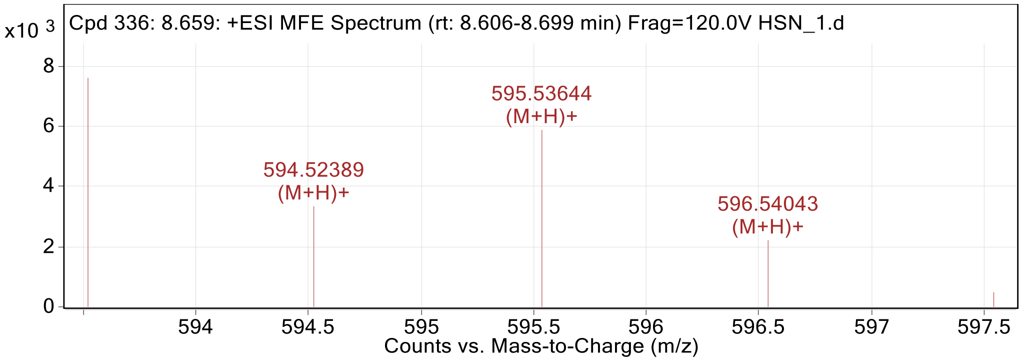

Peak List

m/z	z	Abund	Ion
593.52131	1	7620.1	(M+H)+
594.52389	1	3347.91	(M+H)+
595.53644	1	5887.11	(M+H)+
596.54043	1	2215.91	(M+H)+
597.5423	1	487.13	(M+H)+

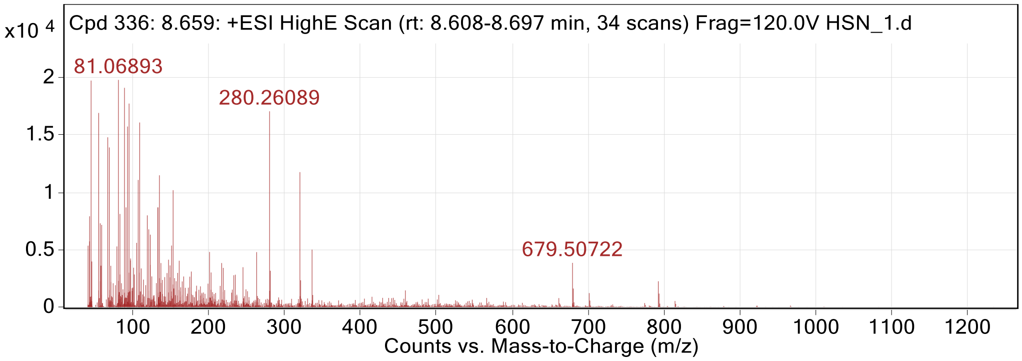

Peak List

m/z	z	Abund
45.0333		19742.44
55.0538		16916.28
67.05356		14794.67
69.06915		13910.54
81.06893		19797.15
89.05858		19106.95
93.06863		15734.35
95.0842		17734.49
109.09956		16085.44
280.26089	1	17062.68

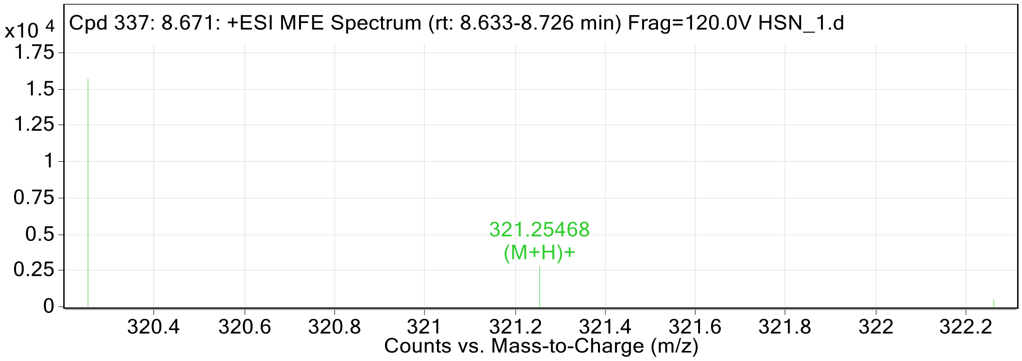

Peak List

m/z	z	Abund	Ion

320.25307	1	15712.67	(M+H)+
321.25468	1	2811.07	(M+H)+
322.26149	1	514.02	(M+H)+

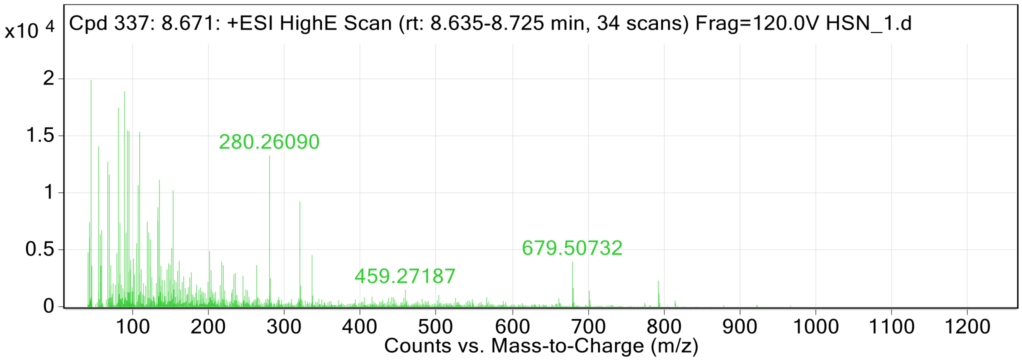

Peak List

m/z	z	Abund
45.0333		19877.62
55.05381		14061.95
67.05355		12711.87
69.06915		11571.3
81.06894		17456.06
89.05856		18909.28
93.06868		15455.69
95.0842		15386.19
109.09954		15308.68
280.2609	1	13261.07

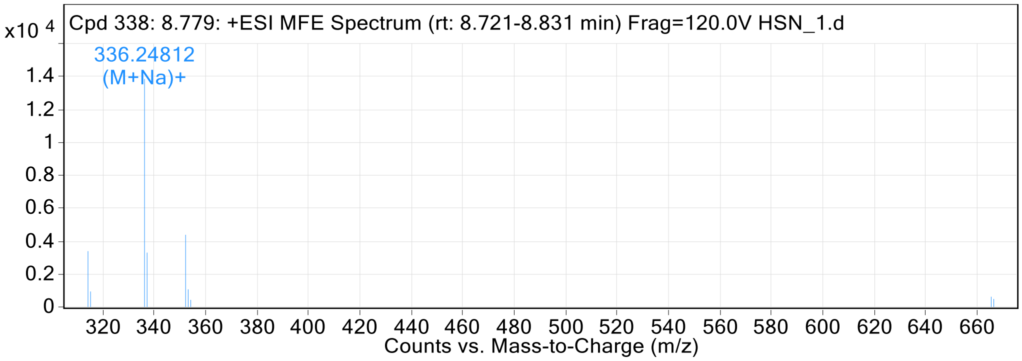

Peak List

m/z	z	Abund	Ion
314.26631	1	3394.57	(M+H)+
315.26835	1	940.94	(M+H)+
336.24812	1	13865.07	(M+Na)+
337.25025	1	3304.24	(M+Na)+
352.22305	1	4389.6	(M+K)+
353.22737	1	1071.05	(M+K)+
354.22347	1	431.77	(M+K)+
665.48195	1	622.32	(2M+K)+
666.48395	1	486.4	(2M+K)+

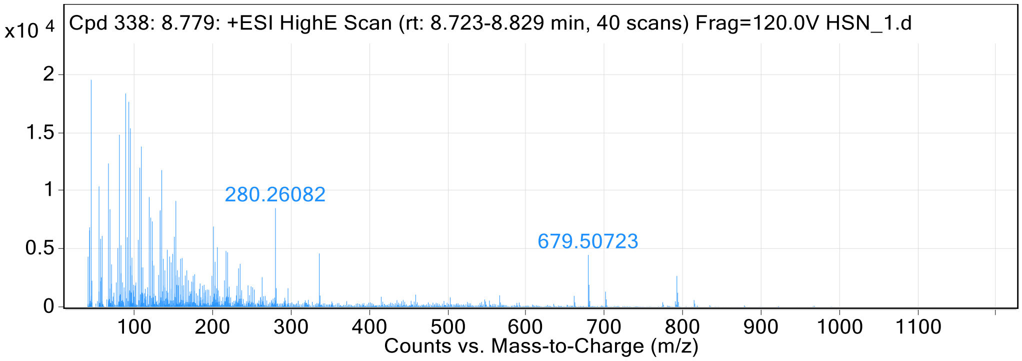

Peak List

m/z	Abund
45.03321	19524.81
55.05377	10362.51
67.05355	12334.86
81.06883	14801.31
89.05854	18357.16
93.0686	17636.5
95.08419	15353.38
107.08392	11963.35
109.09955	13786.7
135.1148	11767.34

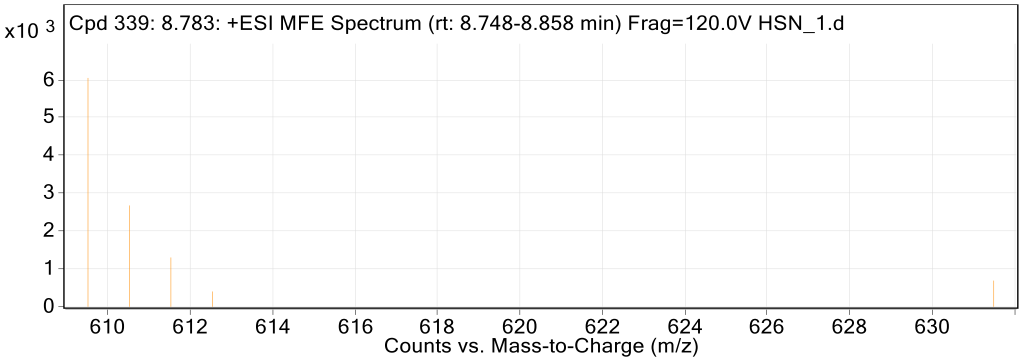

Peak List

m/z	z	Abund	Ion
609.51544	1	6046.09	(M+H)+
610.51846	1	2677.65	(M+H)+
611.52739	1	1302.85	(M+H)+
612.53135	1	404.02	(M+H)+

m/z	z	Abund	Ion
631.49212	1	691.78	(M+Na)+

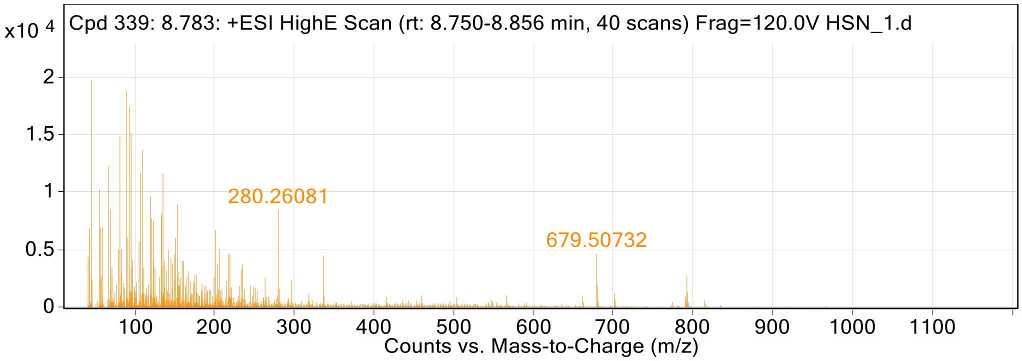

Peak List

m/z	Abund
45.03321	19758.97
55.05376	10230.71
67.05354	12238.2
81.06885	14852.33
89.05856	18814.9
93.06859	17423.49
95.08416	15132.96
107.08393	11697.43
109.09956	13636.58
135.11475	11632.57

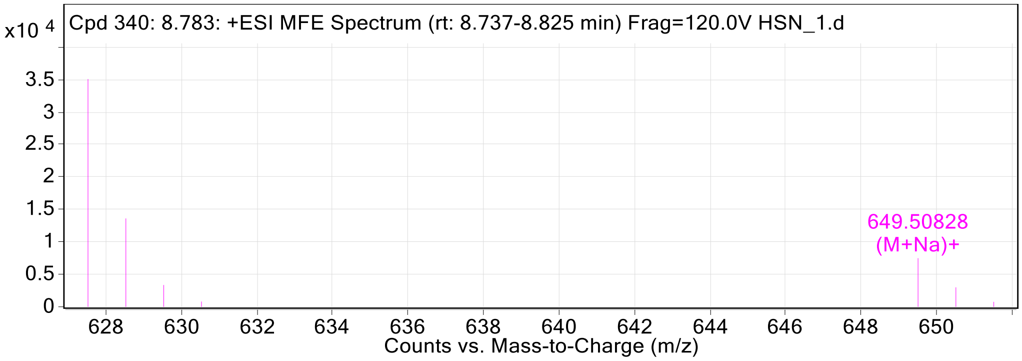

Peak List

m/z	z	Abund	Ion
627.52648	1	35076.63	(M+H)+
628.52974	1	13609.03	(M+H)+
629.53236	1	3356.77	(M+H)+
630.53222	1	807.04	(M+H)+
649.50828	1	7492.74	(M+Na)+
650.51087	1	2998.32	(M+Na)+
651.5141	1	766.57	(M+Na)+

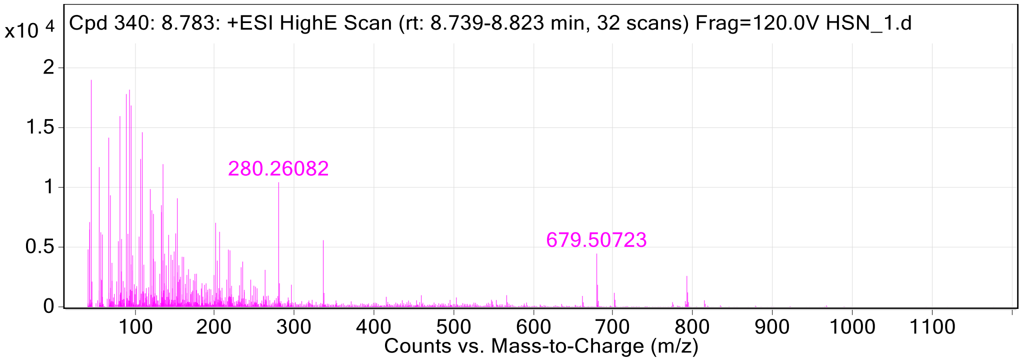

Peak List

m/z	Abund
45.03322	19031.14
55.05376	11716.8
67.05354	14186.09
81.06882	15988.38
89.05855	17843.43
93.06859	18206.99
95.08418	16882.45
107.08393	12399.9
109.09954	14639.1
135.11478	11965.35

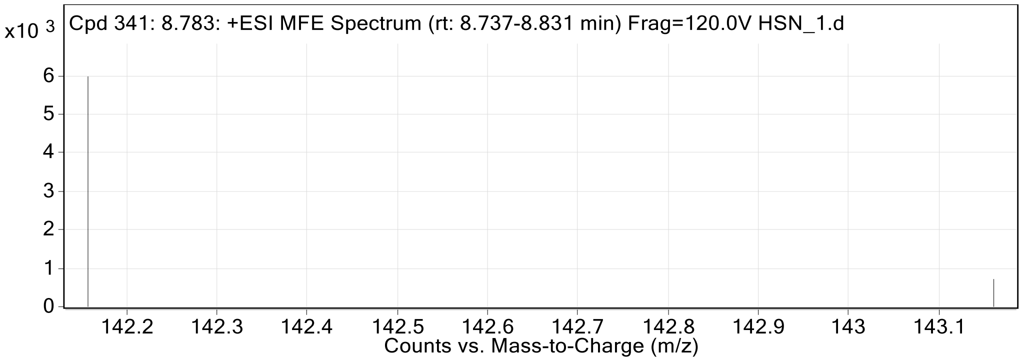

Peak List

m/z	z	Abund	Ion
142.15688	1	5981.12	(M+H)+
143.16098	1	719.69	(M+H)+

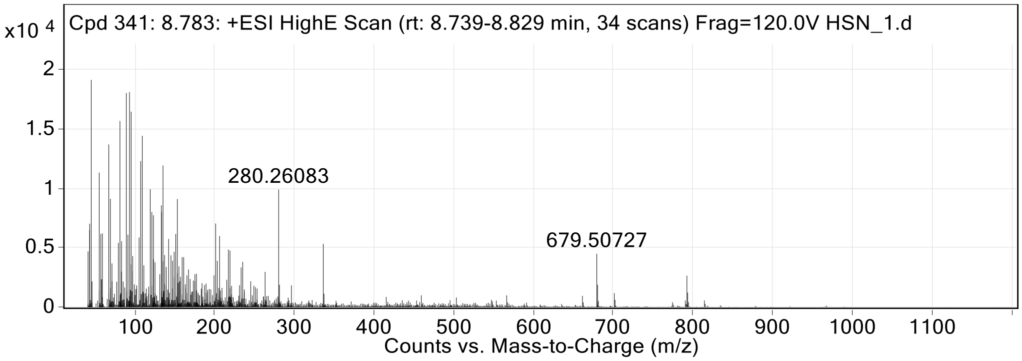

Peak List

m/z	Abund
45.03321	19125.64
55.05377	11316.92
67.05354	13686.38

81.06882	15662.45
89.05856	18019.07
93.06859	18104.77
95.08419	16451.93
107.08392	12290.3
109.09955	14418.25
135.11478	11919.91

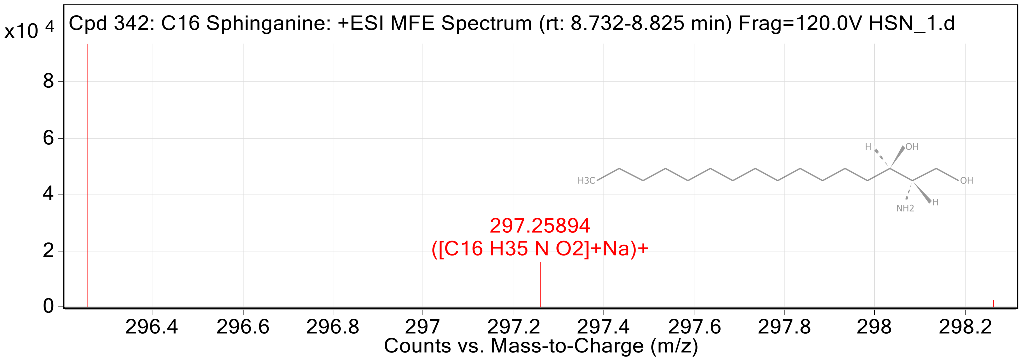

Peak List

m/z	z	Abund	Name	Formula	Ion	Score (DB)	Hits (DB)
296.25636	1	93684.8	C16 Sphinganine	C16 H35 N O2	(M+Na)+	98.47	1
297.25894	1	15908.84		C16 H35 N O2	(M+Na)+		
298.2626	1	2460.23		C16 H35 N O2	(M+Na)+		

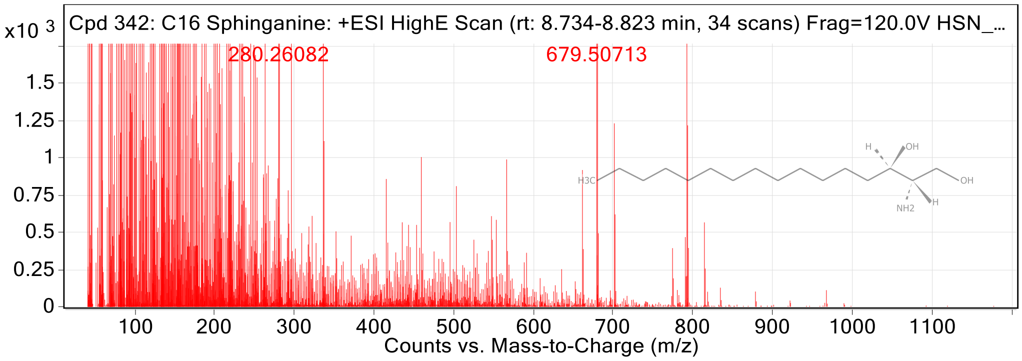

Peak List

m/z	Abund
45.03321	19222.96
55.05376	11309.98
67.05355	13648.88
81.06882	15645.72
89.05856	17990.56
93.06858	18034.93
95.08418	16425.36
107.08393	12260.81
109.09954	14359.96
135.11478	11889.79

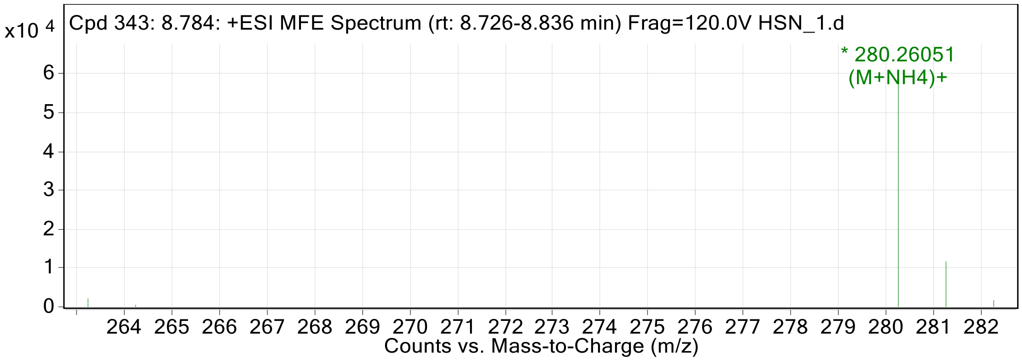

Peak List

m/z	z	Abund	Ion
263.23481	1	2186.32	(M+H)+
264.23669	1	516.49	(M+H)+
280.26051	1	58509.87	(M+NH4)+
281.26426	1	11701.26	(M+NH4)+
282.26786	1	1720.71	(M+NH4)+

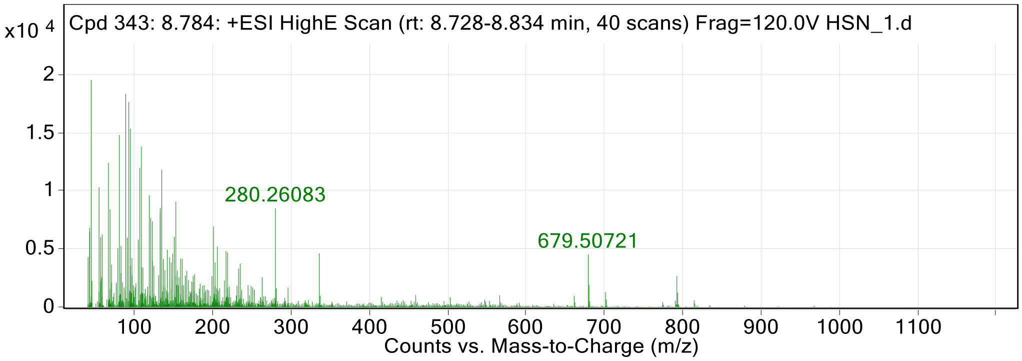

Peak List

m/z	Abund
45.03321	19515.15
55.05378	10287.42
67.05355	12379.24
81.06882	14780.83
89.05855	18319.51
93.06859	17624.21
95.08418	15324.71
107.08393	11958.58
109.09956	13781.38
135.1148	11789.68

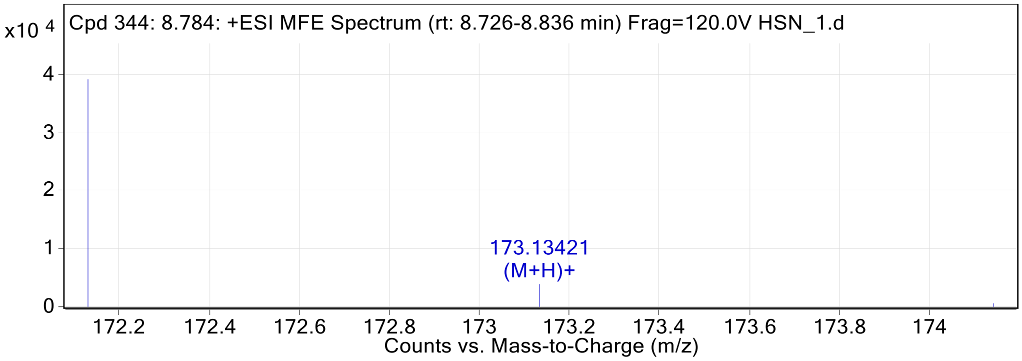

Peak List

m/z	z	Abund	Ion

172.13085	1	39116.8	(M+H)+
173.13421	1	3898.62	(M+H)+
174.14318	1	591.63	(M+H)+

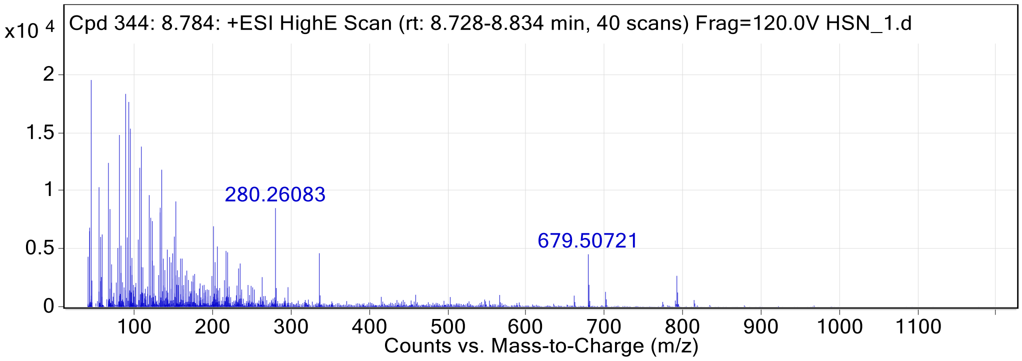

Peak List

m/z	Abund
45.03321	19515.15
55.05378	10287.42
67.05355	12379.24
81.06882	14780.83
89.05855	18319.51
93.06859	17624.21
95.08418	15324.71
107.08393	11958.58
109.09956	13781.38
135.1148	11789.68

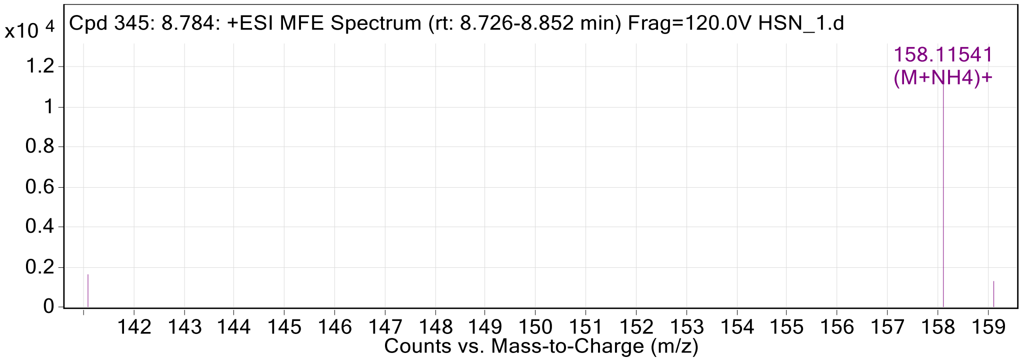

Peak List

m/z	z	Abund	Ion
141.08899	1	1631.37	(M+H)+
158.11541	1	11524.61	(M+NH4)+
159.1182	1	1294.22	(M+NH4)+

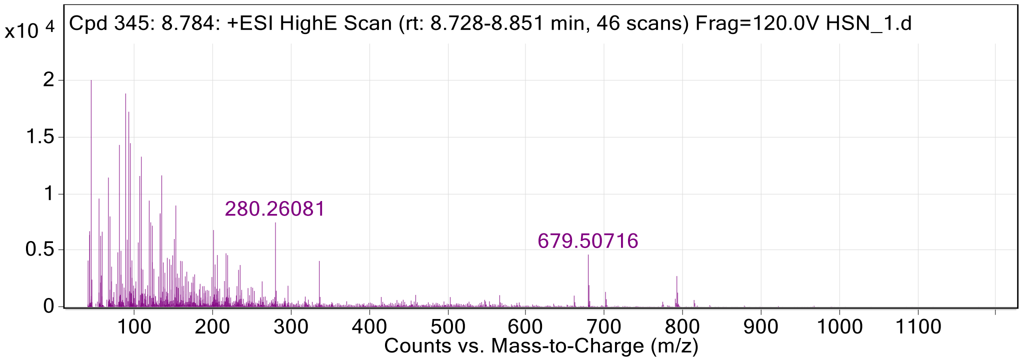

Peak List

m/z	Abund
45.03321	20035.68
55.05377	9560.63
67.05354	11414.38
81.06885	14298.85
89.05856	18845.57
93.06858	17231.57
95.08417	14457.08
107.08395	11555.65
109.09957	13260.79
135.1148	11607.54

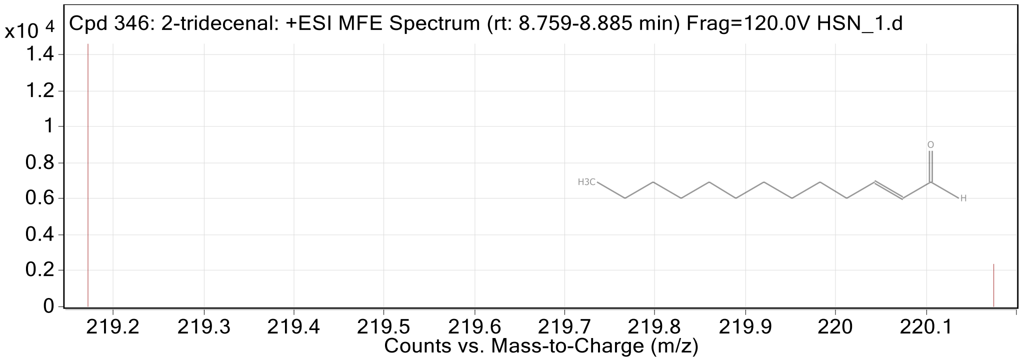

Peak List

m/z	z	Abund	Name	Formula	Ion	Score (DB)	Hits (DB)
219.1718	1	14634.72	2-tridecenal	C13 H24 O	(M+Na)+	86.2	10
220.17504	1	2382.58		C13 H24 O	(M+Na)+		

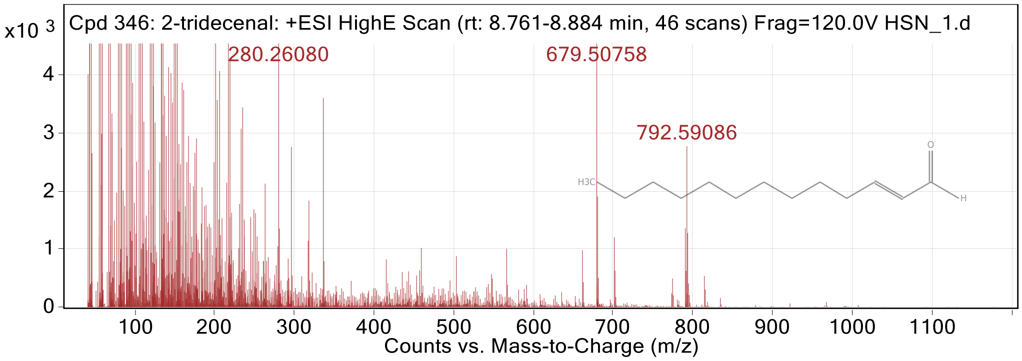

Peak List

m/z	Abund
45.03324	20882.88
55.05376	9126.38
67.05354	10840.54
81.06887	13927.01
89.05859	20639.58
93.0686	16492.14
95.0842	13660.71
107.08394	11054.41
109.09956	12629.82
135.11476	11114.45

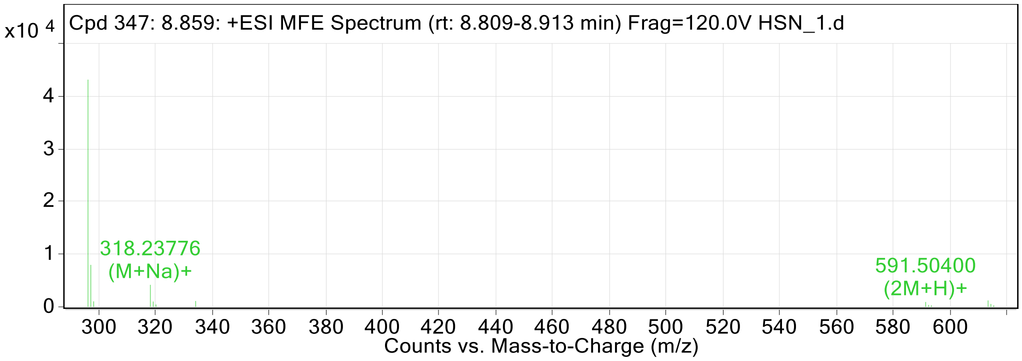

Peak List

m/z	z	Abund	Ion
296.25586	1	43142.15	(M+H)+
297.25918	1	7974.81	(M+H)+
298.26323	1	1048.49	(M+H)+
318.23776	1	4188.7	(M+Na)+
319.23549	1	1015.14	(M+Na)+
320.22612	1	454.96	(M+Na)+
334.21054	1	1117.74	(M+K)+
591.504	1	934.29	(2M+H)+
613.48538	1	1225.44	(2M+Na)+
614.48529	1	558.36	(2M+Na)+

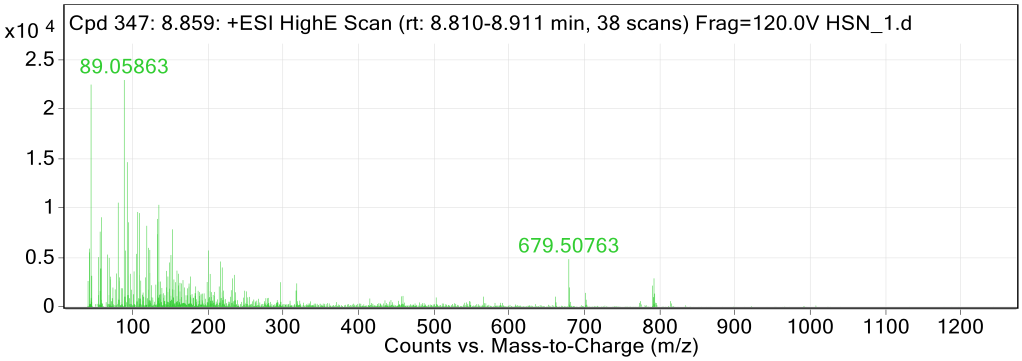

Peak List

m/z	Abund
45.03324	22463.66
59.04863	9048.1
81.06892	10523.81
89.05863	22924.6
93.06862	14618.67
95.08421	8537.33
107.08396	9582.43
109.0996	9471.44
133.08495	8876.99
135.11476	10303.29

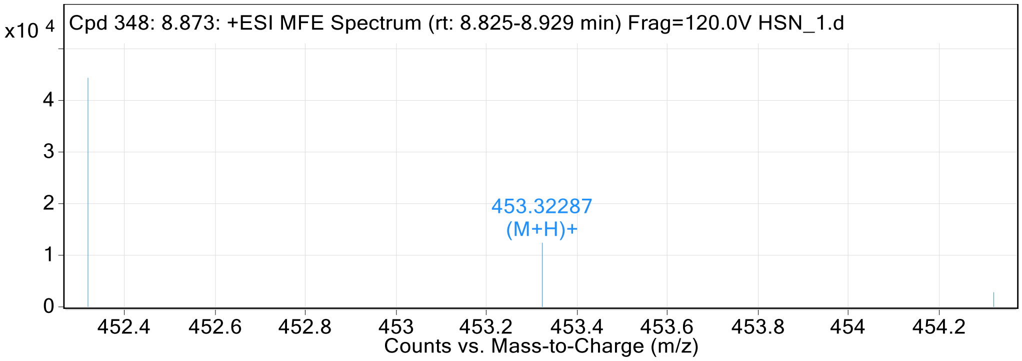

Peak List

m/z	z	Abund	Ion
452.31833	1	44317.94	(M+H)+
453.32287	1	12412.33	(M+H)+
454.32132	1	2842.02	(M+H)+

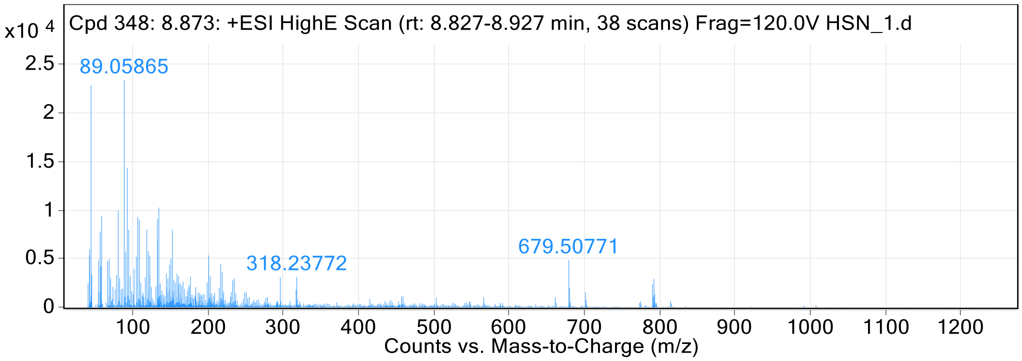

Peak List

m/z	Abund
45.03325	22839.43
59.04866	9419.43
81.06898	9959.31
89.05865	23390.09
93.06864	14350.33
107.08398	9260.71
109.09965	8994.96
119.08382	7961.02
133.08506	9070.08
135.11483	10247.98

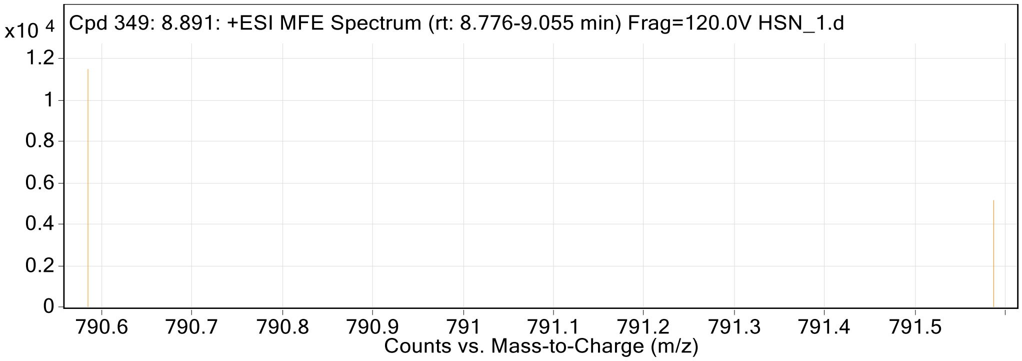

Peak List

m/z	z	Abund	Ion
790.58465	1	11488.61	(M+H)+
791.58732	1	5160.04	(M+H)+

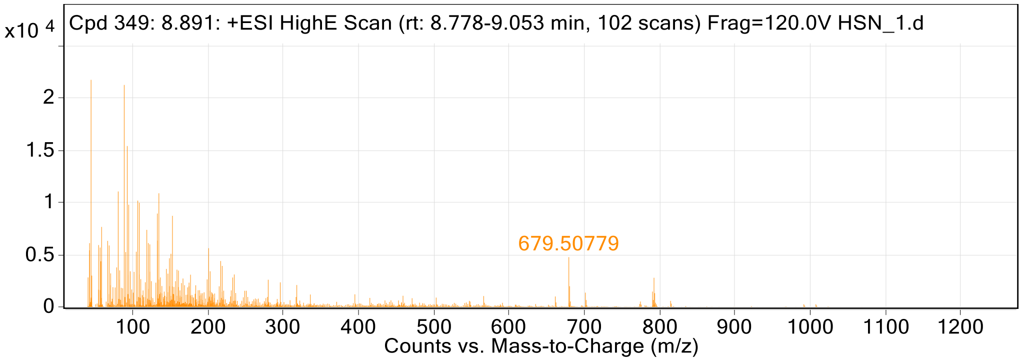

Peak List

m/z	Abund
45.03324	21774.73
81.0689	11067.54
89.05861	21285.11
93.06865	15426.44
95.08422	9801.31
107.08399	10192.99
109.0996	9986.23
133.085	8953.67
135.11485	10894.06
153.1253	8734.48

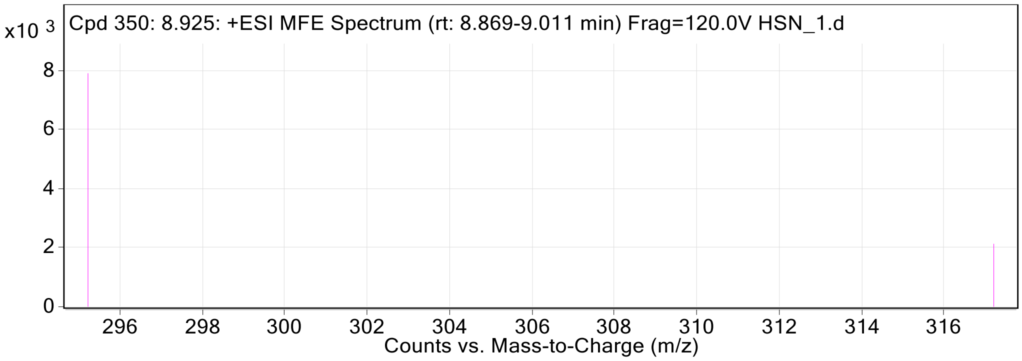

Peak List

m/z	z	Abund	Ion
295.22308	1	7898.9	(M+H)+
317.20733	1	2130.31	(M+Na)+

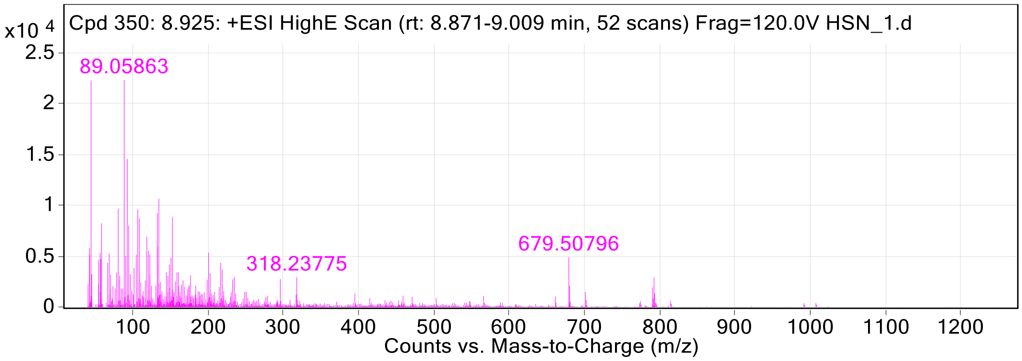

Peak List

m/z	Abund
45.03325	22309.06
59.0487	8229.64
81.06895	9673.29

89.05863	22354.12
93.06867	14584.44
107.084	9607.05
109.09962	8730.79
133.08503	9242.75
135.1149	10664.79
153.12538	8861.04

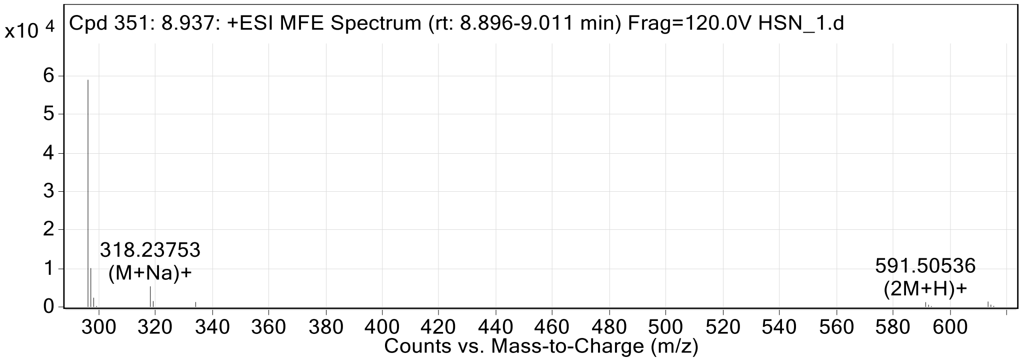

Peak List

m/z	z	Abund	Ion
296.25576	1	59045.63	(M+H)+
297.25925	1	10086.71	(M+H)+
298.26824	1	2394.72	(M+H)+
318.23753	1	5355.95	(M+Na)+
319.23633	1	1519.19	(M+Na)+
334.21139	1	1274.08	(M+K)+
591.50536	1	1248.87	(2M+H)+
592.50839	1	575.38	(2M+H)+
613.48593	1	1399.01	(2M+Na)+
614.48637	1	588.73	(2M+Na)+

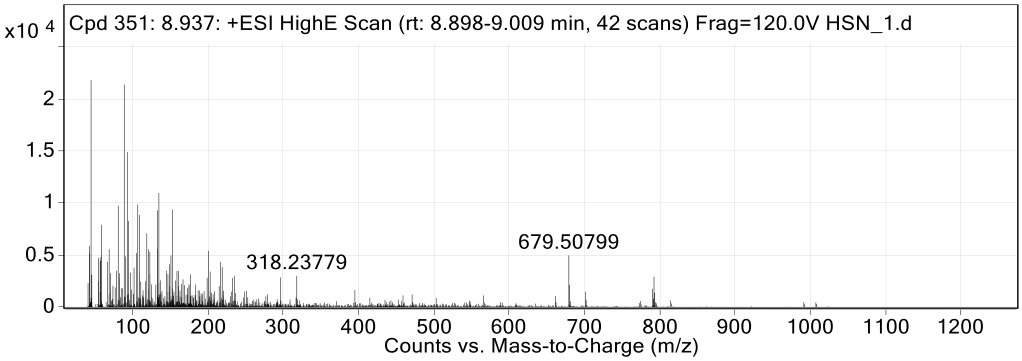

Peak List

m/z	Abund
45.03324	21784.52
81.06896	9719.17
89.05863	21363.37
93.06868	14858.54
95.08422	8243.25
107.08403	9827.79
109.09965	8831.64
133.08503	9265.58
135.11489	10937.79
153.1254	9359.07

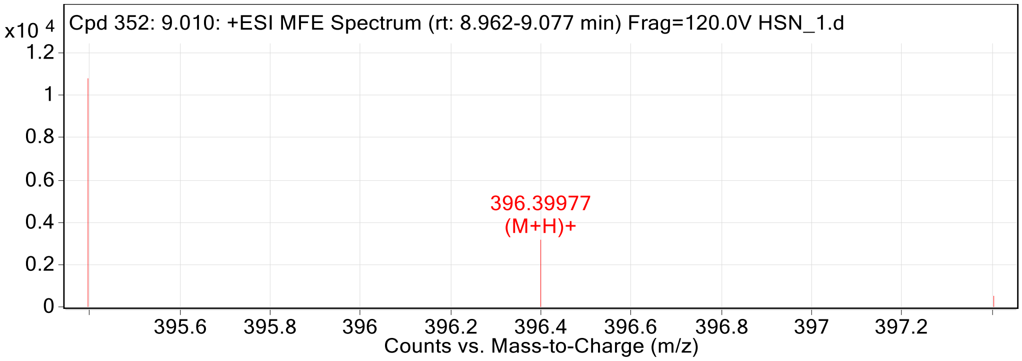

Peak List

m/z	z	Abund	Ion
395.3967	1	10802.98	(M+H)+
396.39977	1	3174.02	(M+H)+
397.40344	1	520.73	(M+H)+

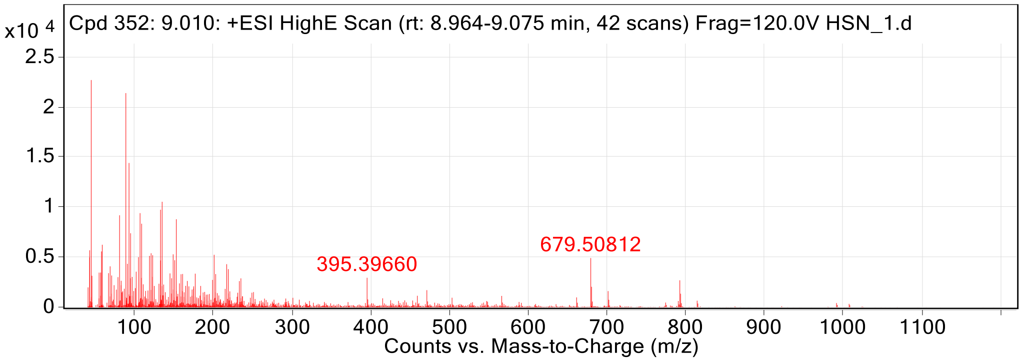

Peak List

m/z	Abund
45.03327	22693.18
81.0689	9174.94
89.05862	21387.6
93.06871	14402.97
95.08427	7384.67
107.08405	9390.5
109.09959	8334.49
133.08504	9732.53
135.1149	10526.51
153.12529	8780.25

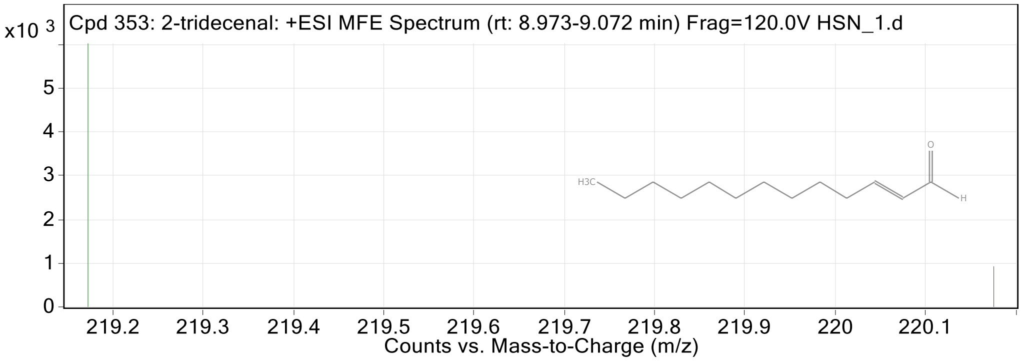

Peak List

m/z	z	Abund	Name	Formula	Ion	Score (DB)	Hits (DB)
219.17229	1	6022.71	2-tridecenal	C13 H24 O	(M+Na)+	86.59	10
220.17562	1	925.74		C13 H24 O	(M+Na)+		

Qualitative Analysis Report

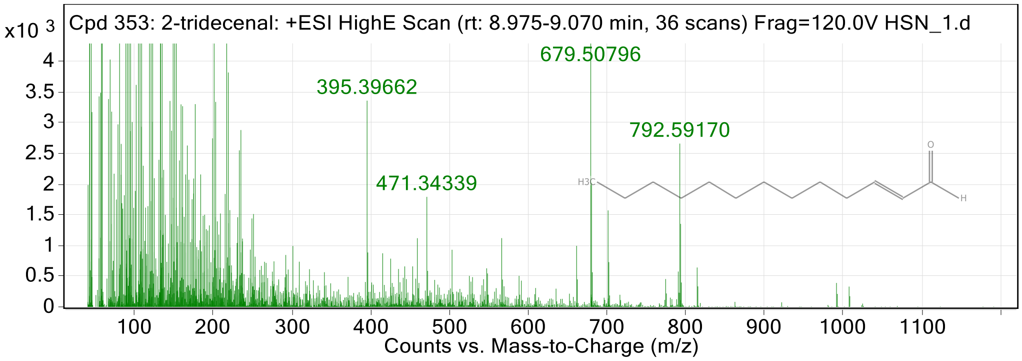

Peak List

m/z	Abund
45.03327	22835.41
81.06891	9204.31
89.05862	21375.68
93.06872	14616.27
95.08429	7362.47
107.08404	9458.6
109.09959	8264.38
133.08504	9822.1
135.11488	10584.83
153.1253	8714.42

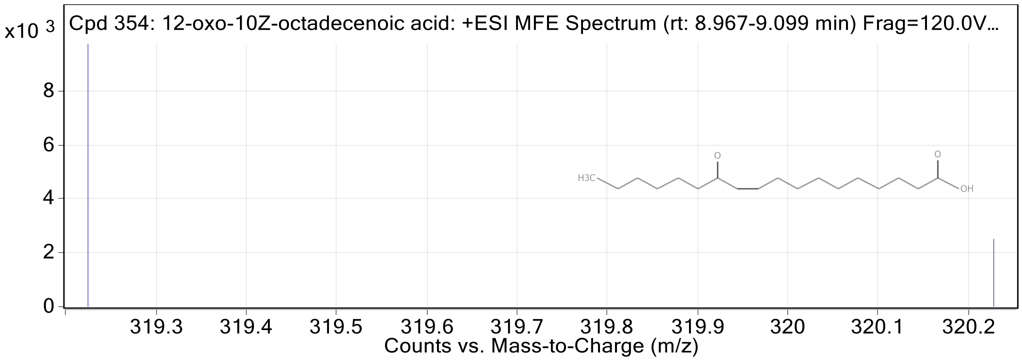

Peak List

m/z	z	Abund	Name	Formula	Ion	Score (DB)	Hits (DB)
319.22448	1	9749.82	12-oxo-10Z-octadecenoic acid	C18 H32 O3	(M+Na)+	78.44	10
320.22829	1	2514.2		C18 H32 O3	(M+Na)+		

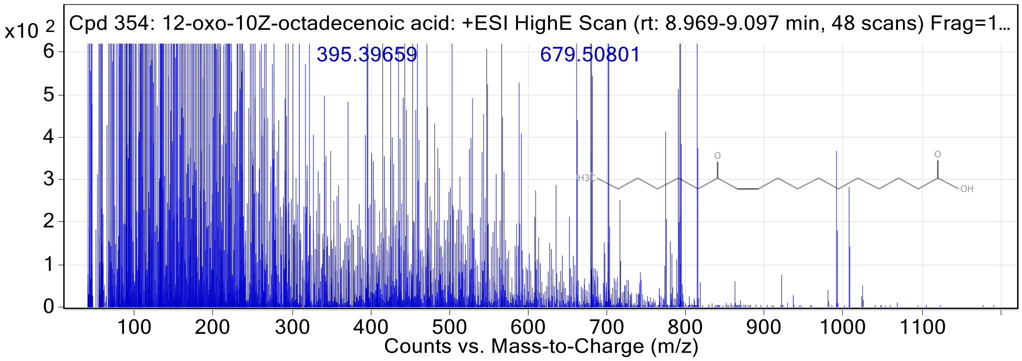

Peak List

m/z	Abund
45.03327	22791.76
81.06893	9049.66

89.0586	21493.13
93.06872	14317.39
95.08427	7297.88
107.08402	9172.26
109.0996	8361.73
133.08504	9743.32
135.11489	10288.03
153.12528	8529.32

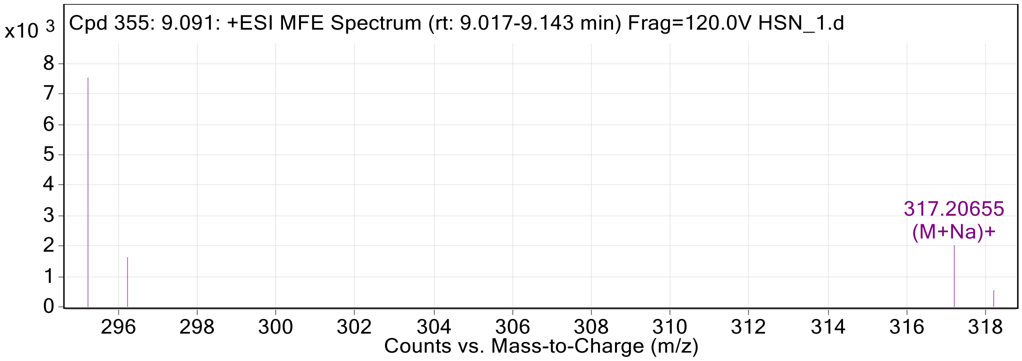

Peak List

m/z	z	Abund	Ion
295.22422	1	7533.62	(M+H)+
296.22732	1	1631.2	(M+H)+
317.20655	1	2024.65	(M+Na)+
318.21059	1	548.56	(M+Na)+

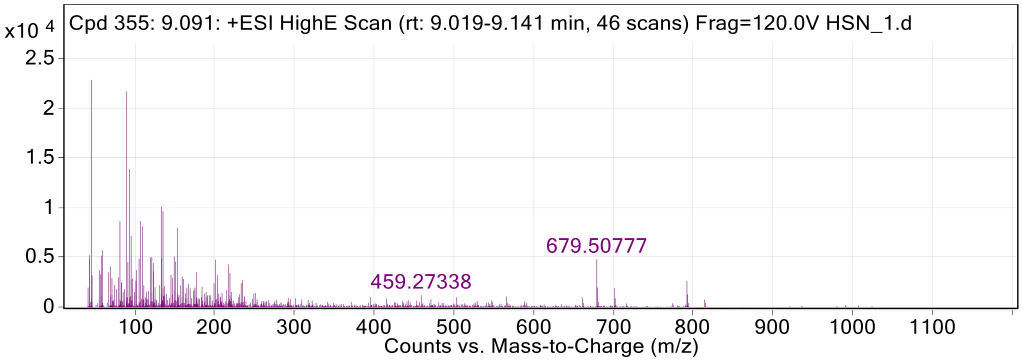

Peak List

m/z	Abund
45.03326	22856.07
81.06891	8658.35
89.05862	21696.76
93.06871	13915.2
95.08422	7158.97
107.08401	8673.1
109.09962	8110.46
133.08487	10110.11
135.1149	9634.88
153.12523	7952.38

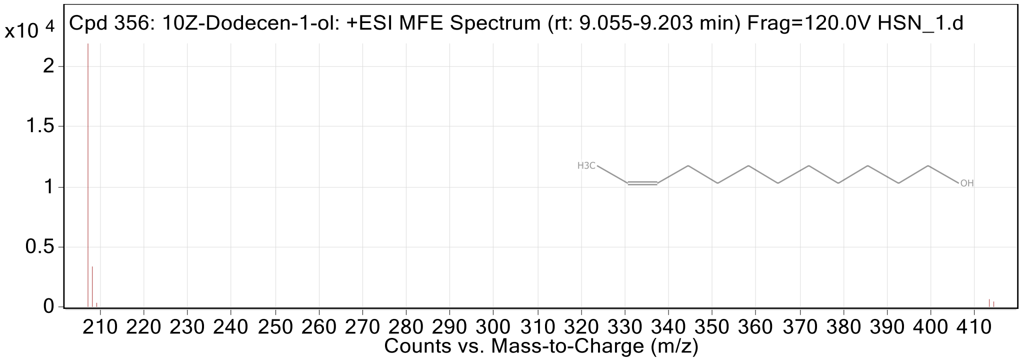

Peak List

m/z	z	Abund	Name	Formula	Ion	Score (DB)	Hits (DB)
207.17185	1	21924.69	10Z-Dodecen-1-ol	C12 H24 O	(M+Na)+	98.22	10
208.17486	1	3360.98		C12 H24 O	(M+Na)+		
209.17595	1	338.44		C12 H24 O	(M+Na)+		
413.33211	1	642.23			(2M+H)+		
414.34654	1	446.63			(2M+H)+		

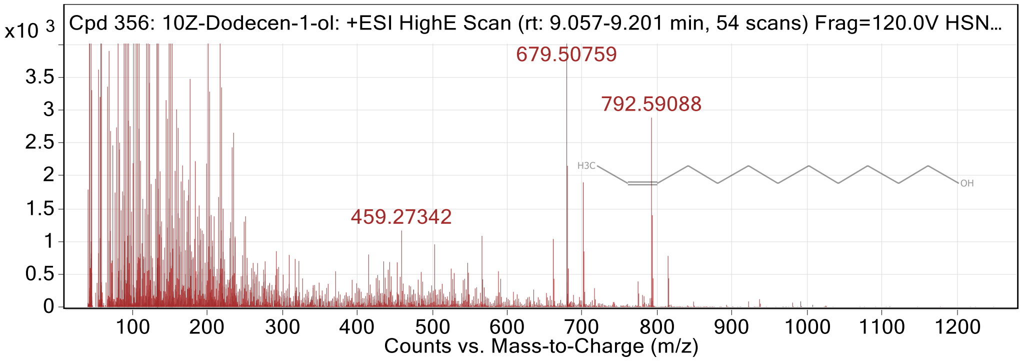

Peak List

m/z	Abund
45.03326	22657.35
81.06895	8070.47
89.0586	21922.67
93.06867	13693.79
95.08416	7052.04
107.08399	8240.51
109.0996	8099.55
133.0849	9953.69
135.11486	9511.7
153.12523	7487.92

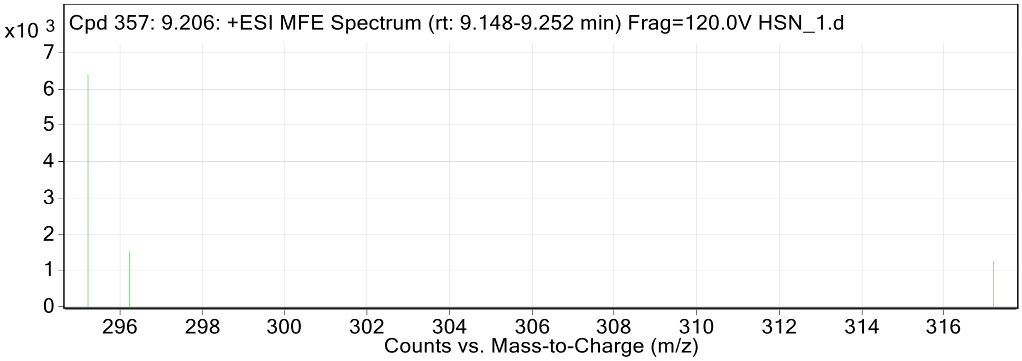

Peak List

m/z	z	Abund	Ion

295.22384	1	6399.58	(M+H)+
296.22722	1	1520.05	(M+H)+
317.20739	1	1258.05	(M+Na)+

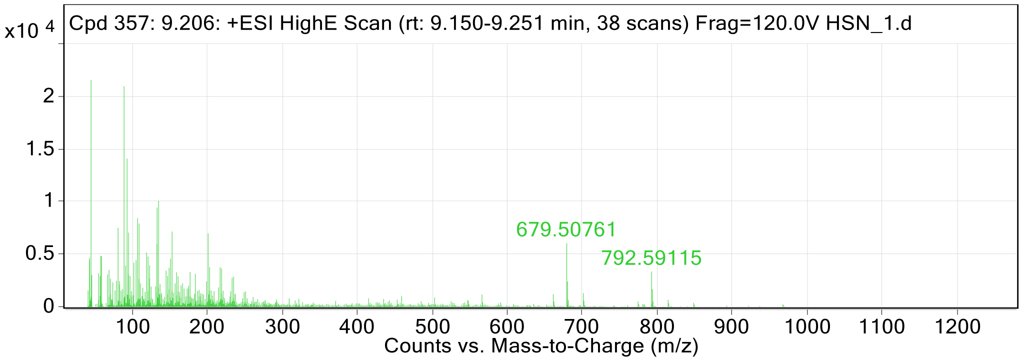

Peak List

m/z	Abund
45.03324	21563.65
81.0689	7504.79
89.05857	20970.66
93.0687	14084.33
95.08416	7051.63
107.08399	8420.46
109.09963	7899.82
133.08485	9419.49
135.11479	10077.17
153.12532	7154.59

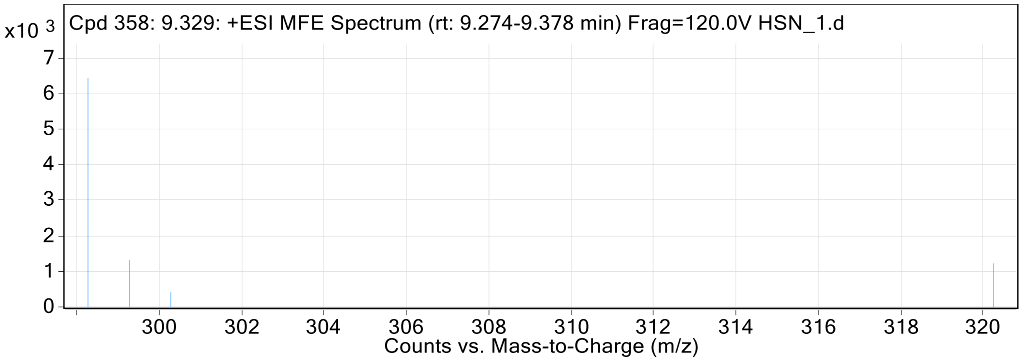

Peak List

m/z	z	Abund	Ion
298.27127	1	6439.71	(M+H)+
299.27513	1	1311.44	(M+H)+
300.28236	1	421.03	(M+H)+
320.25334	1	1222.83	(M+Na)+

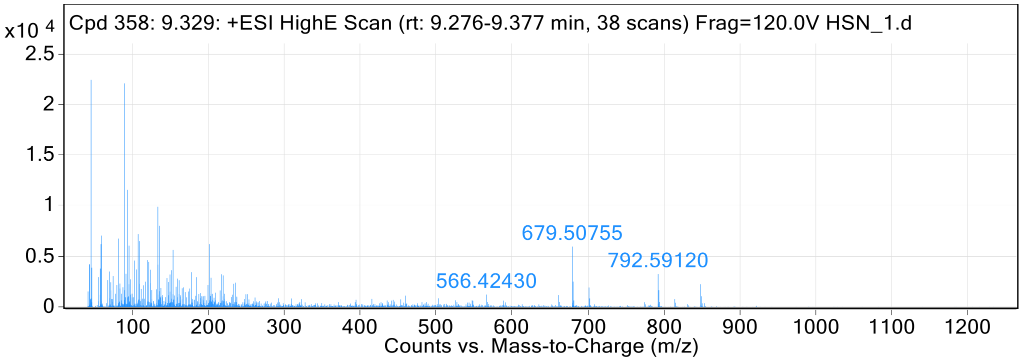

Peak List

m/z	Abund
45.03328	22409.72
59.04869	7036.13
81.0689	6740.17
89.0586	22063.07
93.06868	11566.75
107.084	7185.89
109.09956	6489.02
133.08467	9891.23
135.11487	8010.99
201.16144	6191.65

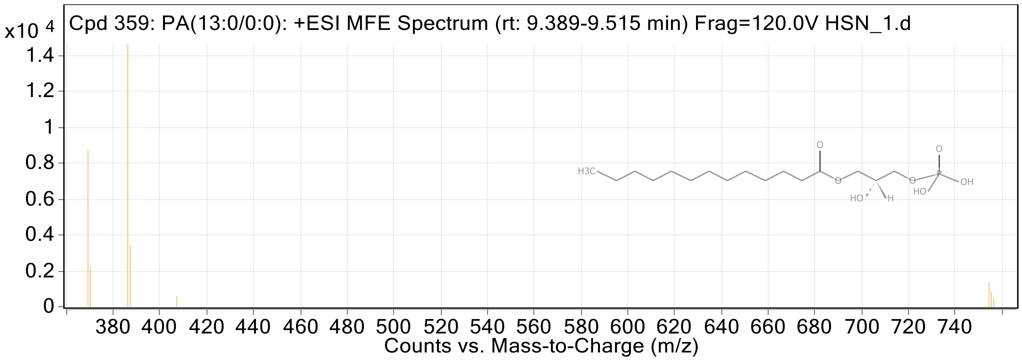

Peak List

m/z	z	Abund	Name	Formula	Ion	Score (DB)	Hits (DB)
369.20374	1	8719.92			(M+H)+		
370.20762	1	2211.77			(M+H)+		
386.23012	1	14642.46	PA(13:0/0:0)	C16 H33 O7 P	(M+NH4)+	77.47	1
387.23456	1	3444.42		C16 H33 O7 P	(M+NH4)+		
407.16317	1	594.33			(M+K)+		
754.42842	1	1376.09			(2M+NH4)+		
755.43075	1	845.79			(2M+NH4)+		
756.44285	1	482.47			(2M+NH4)+		

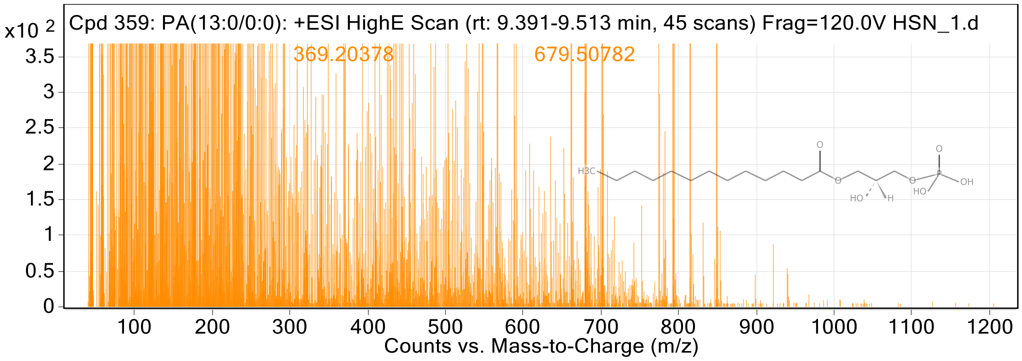

Peak List

m/z	z	Abund
45.03329		23875.7
81.06896		6981.68
89.05867		23379.72
93.06867		11548.34
95.08428		6304.41
107.08409		7742.59
109.09953		6668.39
119.08386	1	12049.69
133.08477		10917.22
135.11489		7885.91

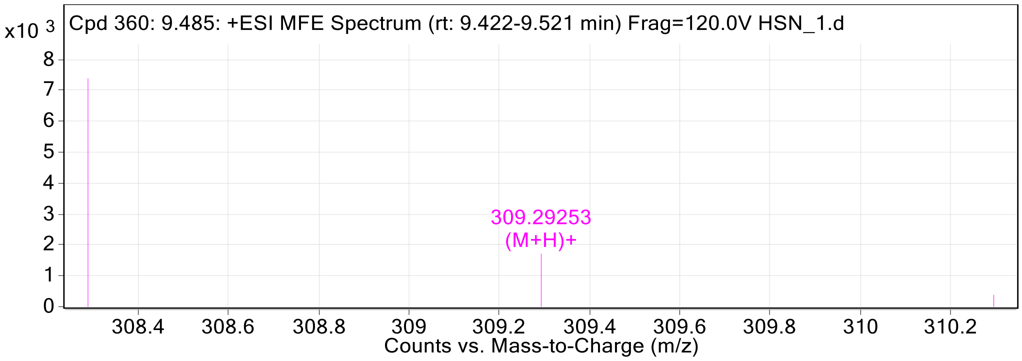

Peak List

m/z	z	Abund	Ion
308.28843	1	7383.31	(M+H)+
309.29253	1	1715.37	(M+H)+
310.2953	1	387.24	(M+H)+

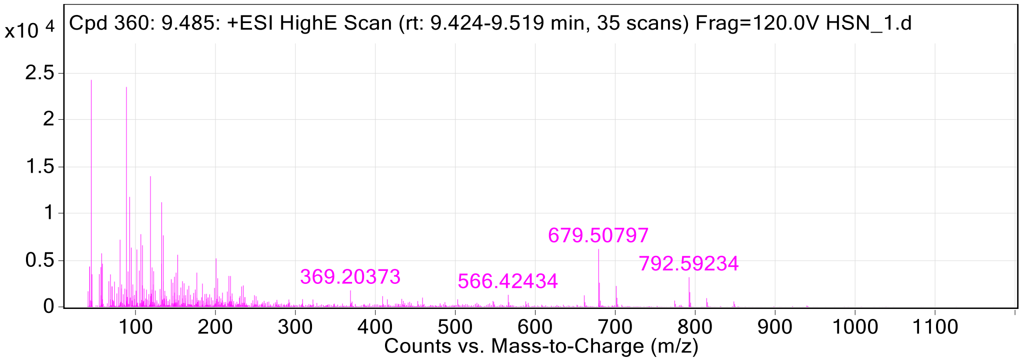

Peak List

m/z	z	Abund
45.03328		24256.45
81.06898		7180.36

89.05868		23502.51
93.06867		11753.87
95.08426		6334.69
107.08413		7769.28
109.09961		6582.86
119.08386	1	13963.35
133.08474		11182.89
135.1149		7643.28

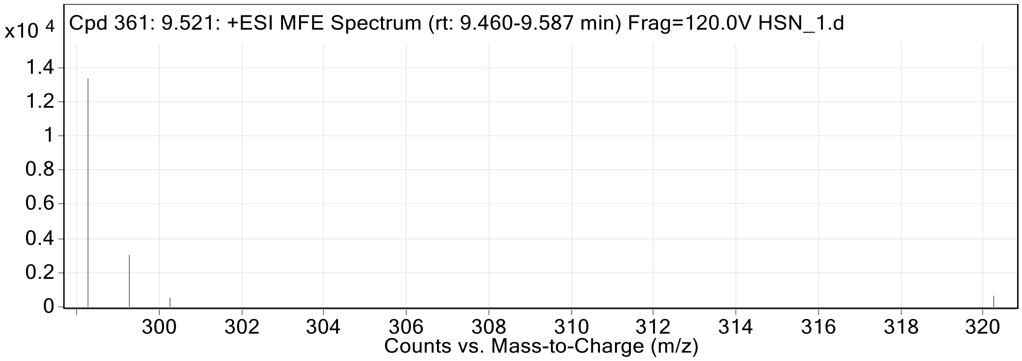

Peak List

m/z	z	Abund	Ion
298.27146	1	13358.91	(M+H)+
299.27414	1	3034.33	(M+H)+
300.26197	1	536.51	(M+H)+
320.25281	1	654.01	(M+Na)+

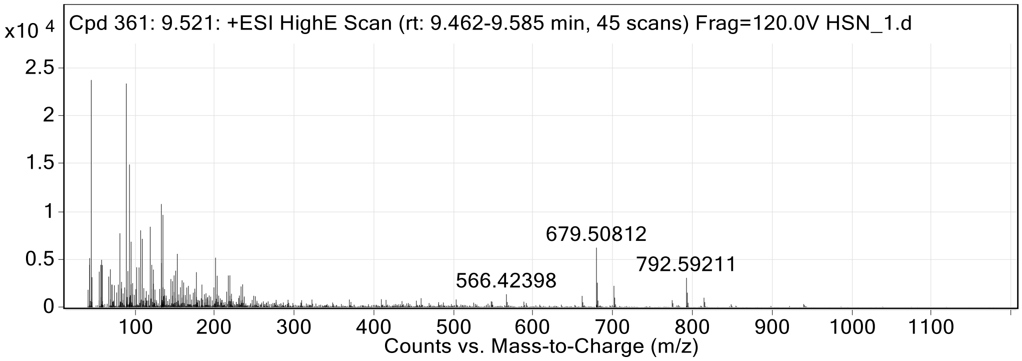

Peak List

m/z	z	Abund
45.03331		23737.82
81.06898		7710.79
89.05866		23368.21
93.06869	1	14902.07
95.08429		6827.33
107.08414		8017.22
109.09965		7132.19
119.08383		8394.64
133.0848		10768.6
135.115		9635.24

Qualitative Analysis Report

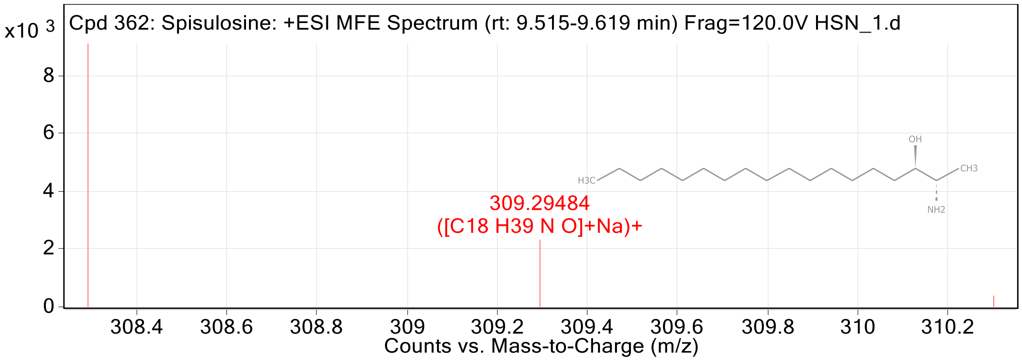

Peak List

m/z	z	Abund	Name	Formula	Ion	Score (DB)	Hits (DB)
308.29206	1	9110.62	Spisulosine	C18 H39 N O	(M+Na)+	92.76	1
309.29484	1	2327.04		C18 H39 N O	(M+Na)+		
310.3015	1	387.25		C18 H39 N O	(M+Na)+		

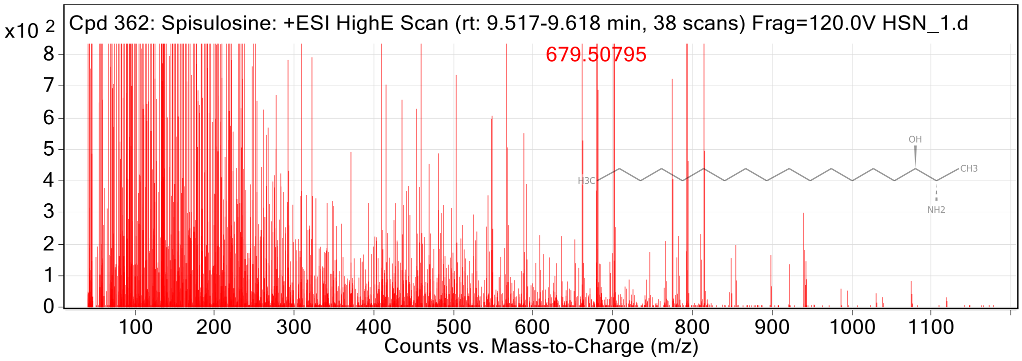

Peak List

m/z	z	Abund
45.03332		23277.27
81.06898		7207.31
89.05866		23052.98
93.06869	1	18477.34
95.0843		6685.44
107.08412		8702.09
109.09966		6974.06
133.08475		10774.74
135.11502	1	13085.08
679.50795	1	6229.85

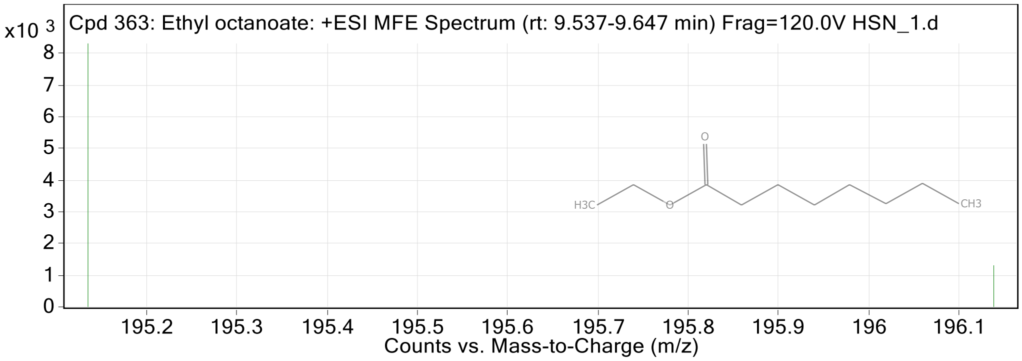

Peak List

m/z	z	Abund	Name	Formula	Ion	Score (DB)	Hits (DB)
195.13529	1	8318.53	Ethyl octanoate	C10 H20 O2	(M+Na)+	80.7	10
196.1383	1	1312.71		C10 H20 O2	(M+Na)+		

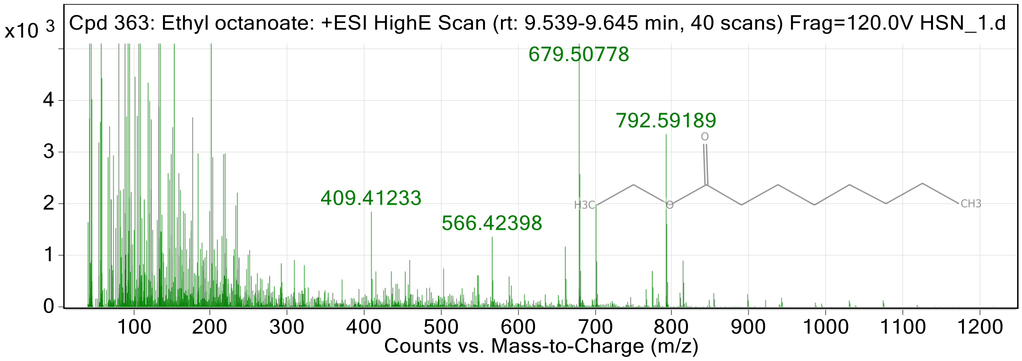

Peak List

m/z	z	Abund
45.03332		23761.69
58.06478		6489.9
81.06902		6775.85
89.05864		23558.4
93.06867	1	17905.65
107.08414		8293.63
109.09969		6579.27
133.08468		11468.87
135.11499	1	12961.91
679.50778	1	6288.48

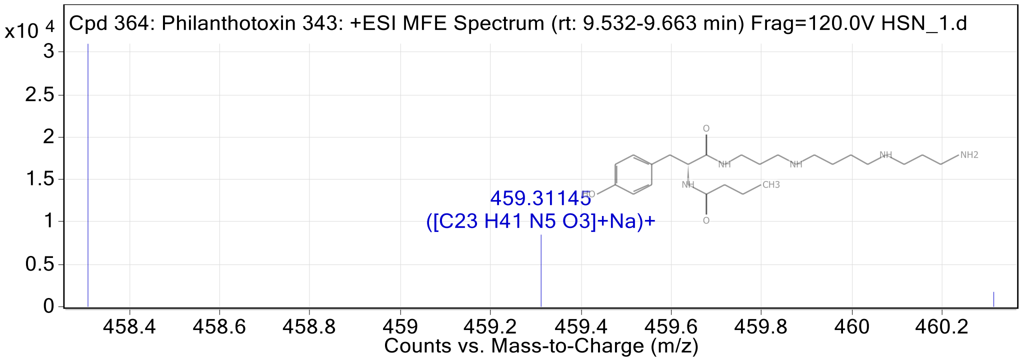

Peak List

m/z	z	Abund	Name	Formula	Ion	Score (DB)	Hits (DB)
458.30836	1	30995.6	Philanthotoxin 343	C23 H41 N5 O3	(M+Na)+	92.07	2
459.31145	1	8496.33		C23 H41 N5 O3	(M+Na)+		
460.31357	1	1754.17		C23 H41 N5 O3	(M+Na)+		

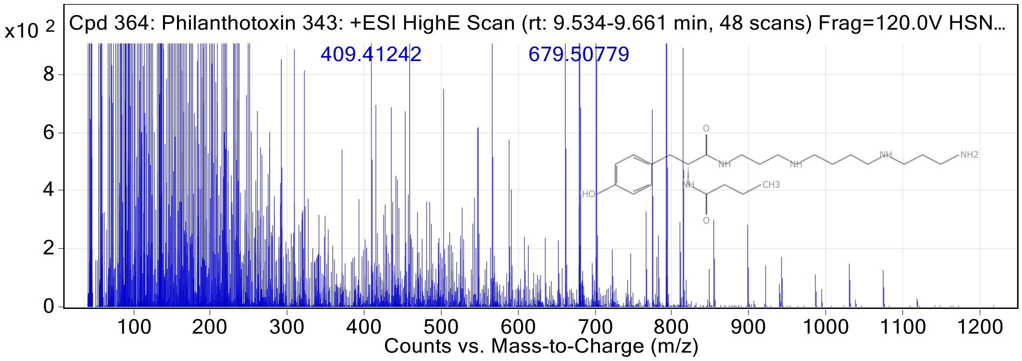

Peak List

m/z	z	Abund
45.0333		23819.59
58.06477		6501.82

81.06899		6735.72
89.05864		23643.34
93.06868	1	16960.41
107.08411		7945.16
109.09965		6557.69
133.08474		11364.7
135.11499	1	11997.6
679.50779	1	6338.39

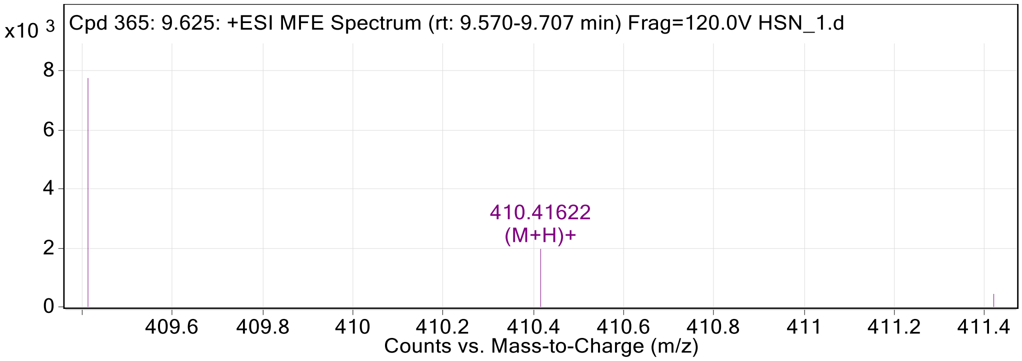

Peak List

m/z	z	Abund	Ion
409.4124	1	7754.67	(M+H)+
410.41622	1	1966.96	(M+H)+
411.42093	1	439.58	(M+H)+

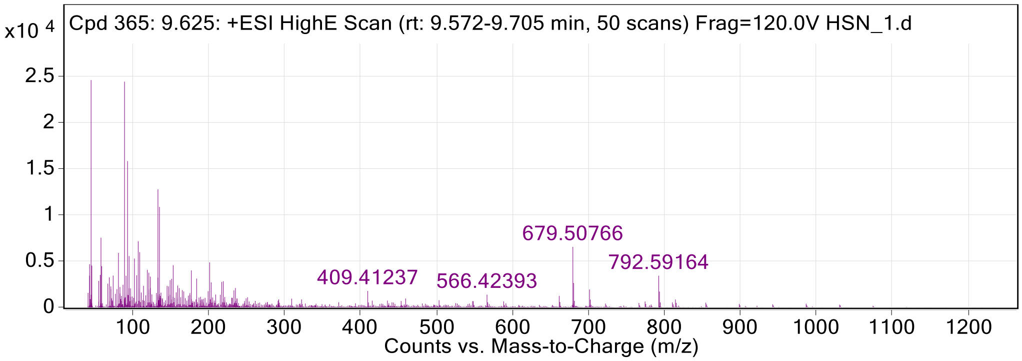

Peak List

m/z	z	Abund
45.03327		24625.16
58.06476		7526.22
81.06899		5884.19
89.05863	1	24453.52
93.0687	1	15833.75
107.08406		7143.28
109.09962		5959.83
133.08492		12768.66
135.115	1	10862.71
679.50766	1	6529.09

Qualitative Analysis Report

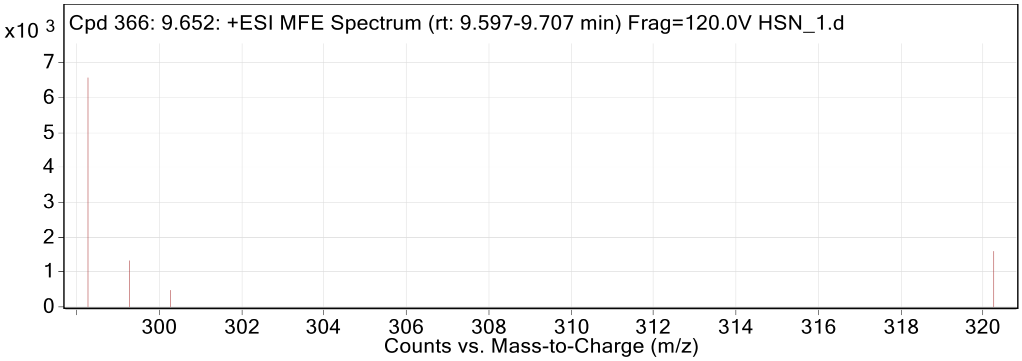

Peak List

m/z	z	Abund	Ion
298.27165	1	6579.6	(M+H)+
299.27504	1	1328.97	(M+H)+
300.2792	1	482.18	(M+H)+
320.25376	1	1597.28	(M+Na)+

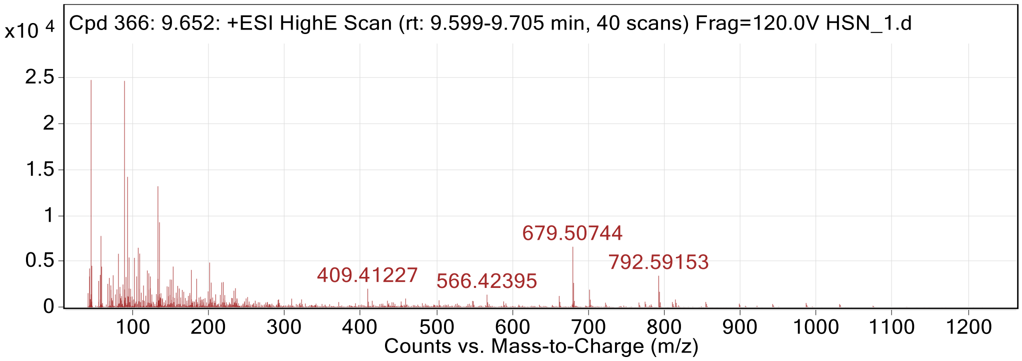

Peak List

m/z	z	Abund
45.03326		24795.03
58.06478		7760.16
81.06899		5815.63
89.05862	1	24693.98
93.06869	1	14217.75
107.08404		6470.73
109.09959		5836.7
133.085		13188.47
135.11496	1	9251.53
679.50744	1	6582.06

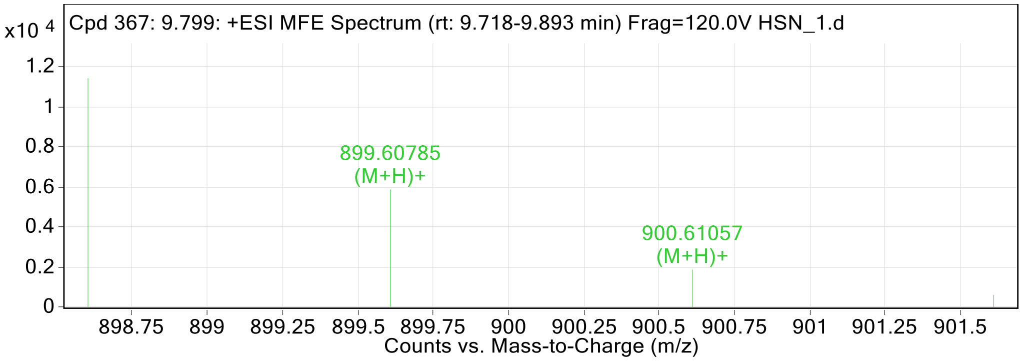

Peak List

m/z	z	Abund	Ion
898.60444	1	11420.92	(M+H)+

899.60785	1	5860.32	(M+H)+
900.61057	1	1858.45	(M+H)+
901.61062	1	600.21	(M+H)+

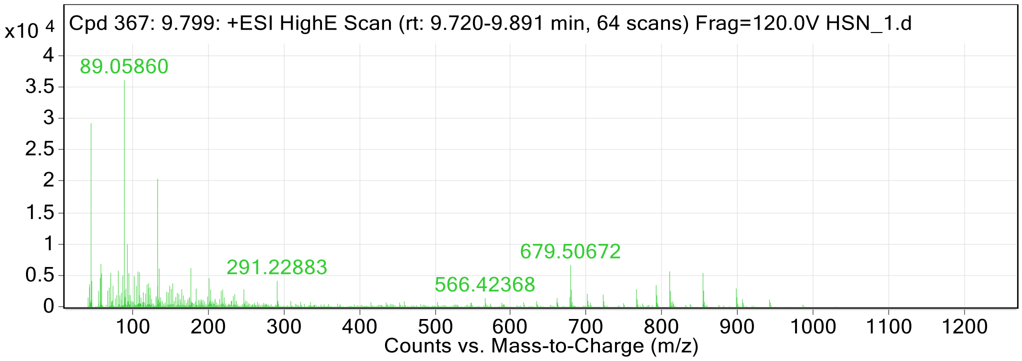

Peak List

m/z	z	Abund
45.03324		29252.38
58.06465		6802.17
81.06884		5739.48
89.0586	1	36133.73
93.0686		9946.62
133.08477		20392.93
135.11475		6092.37
177.11068		6157.53
679.50672	1	6638.34
810.55254	1	5628.06

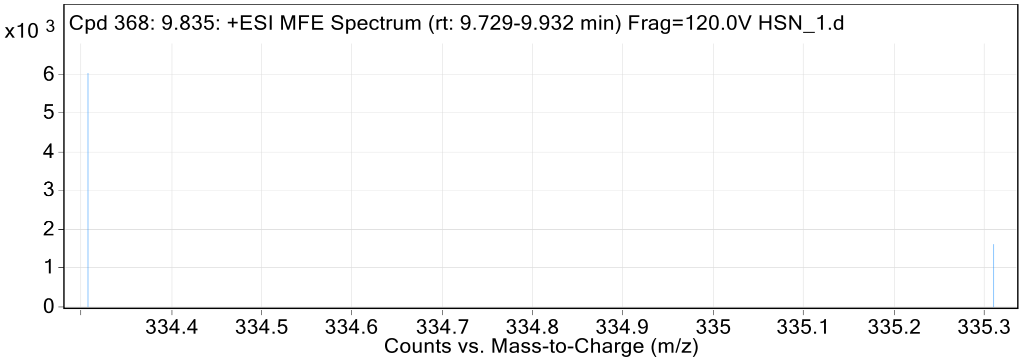

Peak List

m/z	z	Abund	Ion
334.3077	1	6024.6	(M+H)+
335.31082	1	1614.44	(M+H)+

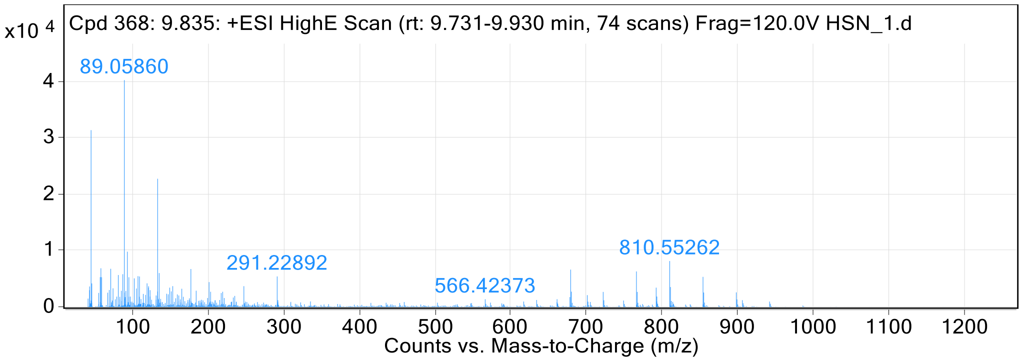

Peak List

m/z	z	Abund

45.03325		31295.57
58.06467		6825.97
71.08476		6732.18
89.0586	1	40167.25
93.06861		9763.98
133.08467	1	22686.78
177.11061		6696.41
679.5065	1	6589.59
766.52667	1	6265.24
810.55262	1	8104.87

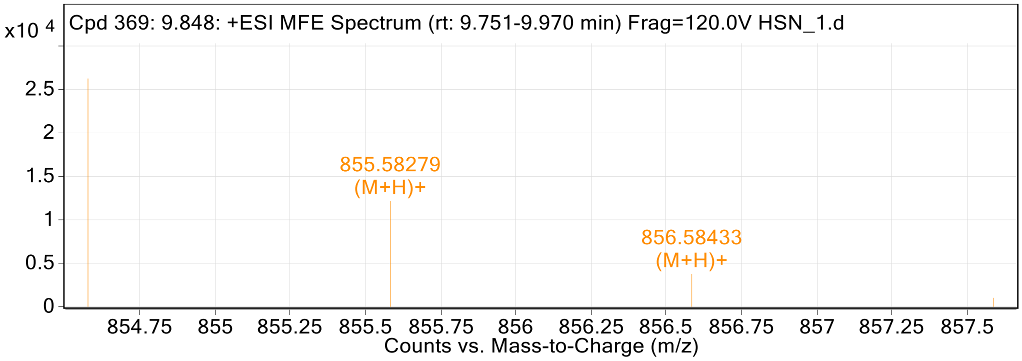

Peak List

m/z	z	Abund	Ion
854.57857	1	26329.71	(M+H)+
855.58279	1	12212.32	(M+H)+
856.58433	1	3794.01	(M+H)+
857.58736	1	1031.96	(M+H)+

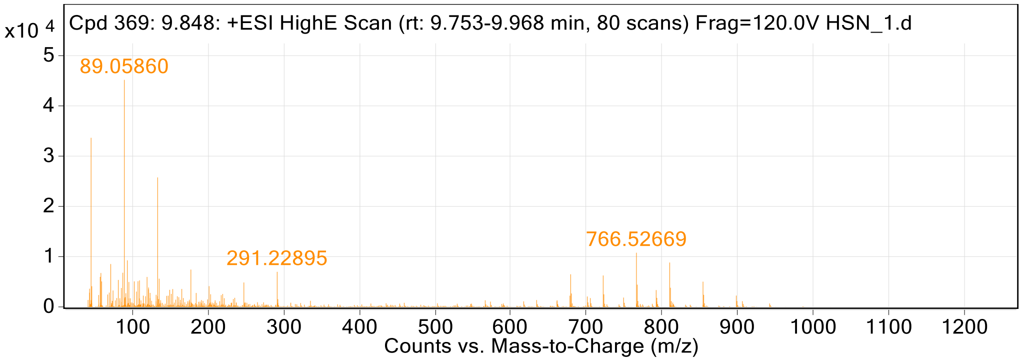

Peak List

m/z	z	Abund
45.03326		33737.98
71.08473		8553.11

m/z	z	Abund
87.04292		6813.57
89.0586	1	45287.39
93.06861		9288.49
133.08453	1	25826.73
177.11053		7445.2
291.22895	1	7000.95
766.52669	1	10808.69
810.55267	1	8852.41

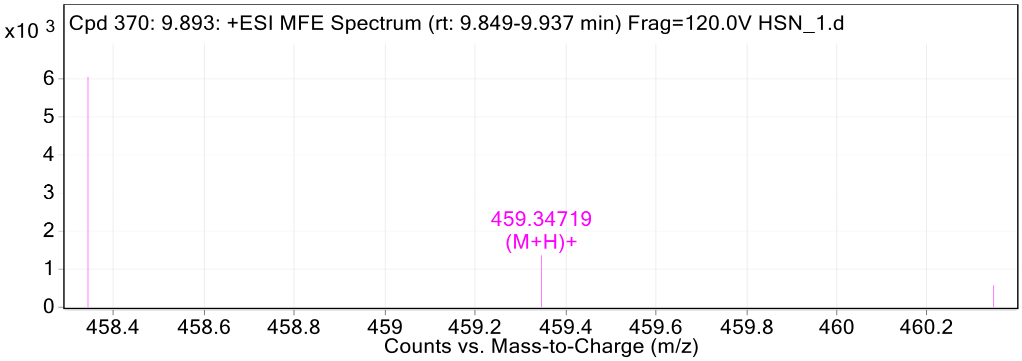

Peak List

m/z	z	Abund	Ion
458.3442	1	6041.99	(M+H)+
459.34719	1	1357.65	(M+H)+
460.3469	1	574.2	(M+H)+

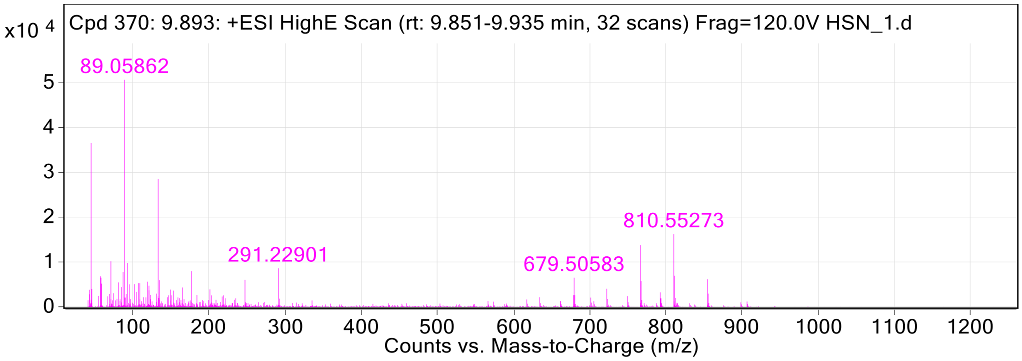

Peak List

m/z	z	Abund
45.03326		36480.52
71.08477		10138.7
87.04296		7795.54
89.05862	1	50621.72
93.06863		9801.04
133.08457	1	28470.75
177.11053		7963.6
291.22901	1	8575.36
766.52675	1	13763.5
810.55273	1	16213.53

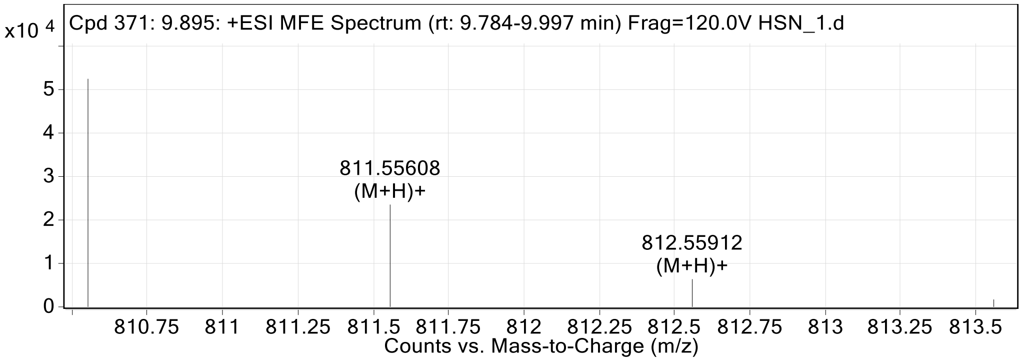

Peak List

m/z	z	Abund	Ion
810.55279	1	52412.91	(M+H)+
811.55608	1	23515.63	(M+H)+
812.55912	1	6365.37	(M+H)+
813.56027	1	1705.99	(M+H)+

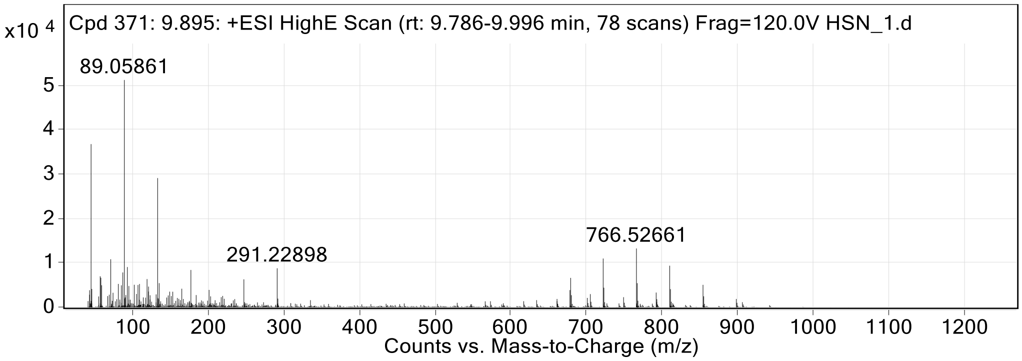

Peak List

m/z	z	Abund
45.03327		36760.09
71.08473		10778.72
89.05861	1	51222.75
93.06862		9023.6
133.08446	1	29093.85
177.11049		8358.54
291.22898	1	8744.31
722.50029	1	10940.27
766.52661	1	13207.03
810.55266	1	9324.57

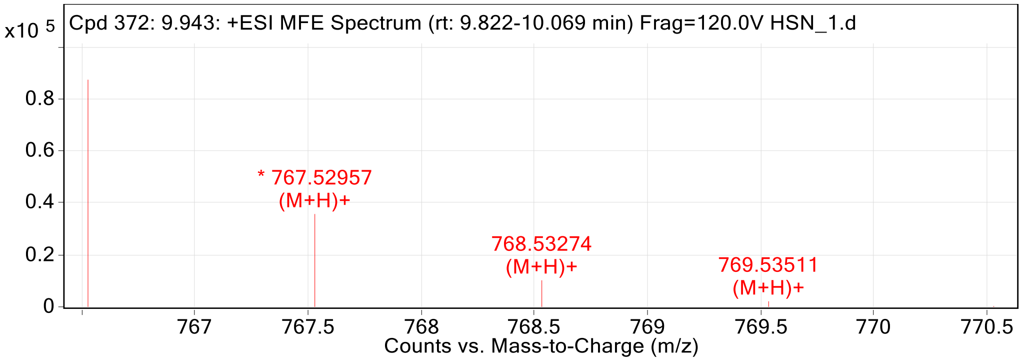

Peak List

m/z	z	Abund	Ion
766.52704	1	87445.7	(M+H)+

767.52957	1	35738.02	(M+H)+
768.53274	1	10220.75	(M+H)+
769.53511	1	2133.53	(M+H)+
770.53014	1	300.5	(M+H)+

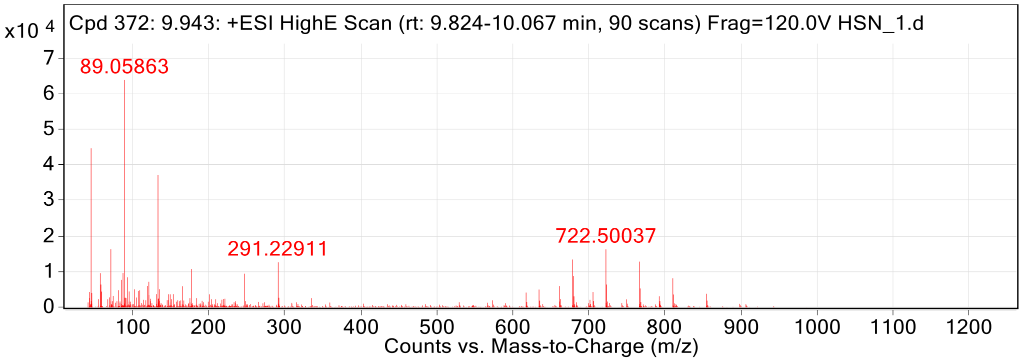

Peak List

m/z	z	Abund
45.03329		44617.94
71.08474		16258.04
87.04296		9574.85
89.05863	1	63797.11
133.08433	1	37050.77
177.11036		10724.27
291.22911	1	12576.04
678.47454	1	13360.77
722.50037	1	16212.97
766.52656	1	12772.51

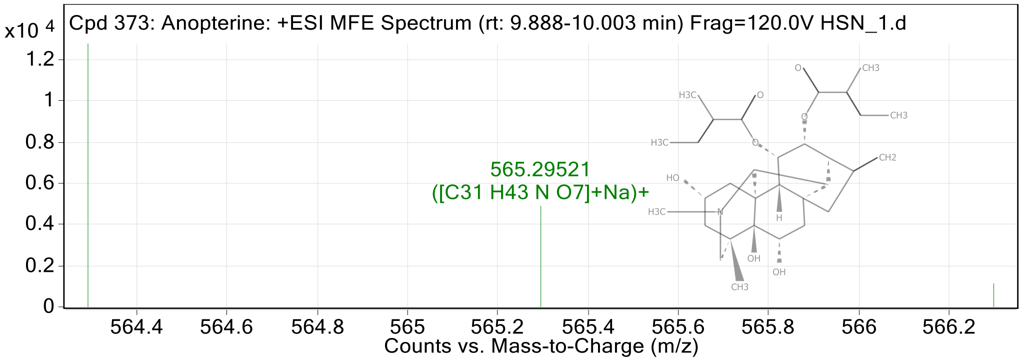

Peak List

m/z	z	Abund	Name	Formula	Ion	Score (DB)	Hits (DB)
564.2915	1	12790.8	Anopterine	C31 H43 N O7	(M+Na)+	92.97	1
565.29521	1	4906.03		C31 H43 N O7	(M+Na)+		
566.29913	1	1140.32		C31 H43 N O7	(M+Na)+		

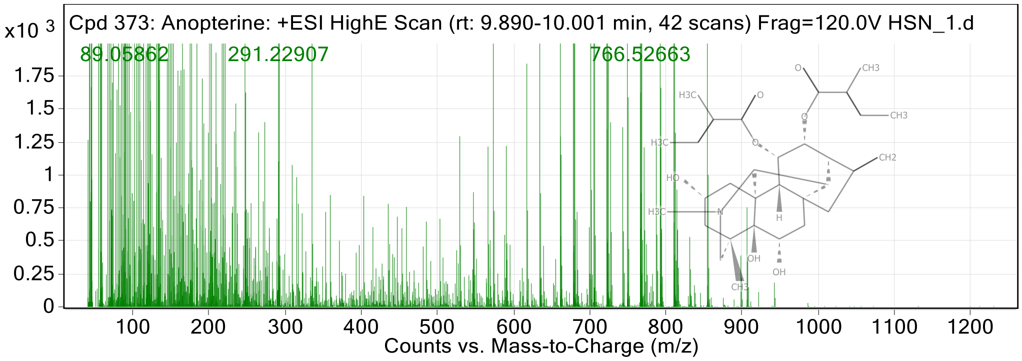

Peak List

m/z	z	Abund
45.03329		43436.39
71.08474		15317.17
87.04291		9953.3
89.05862	1	63204.71
133.08424	1	36176.97
177.11041		10099.26
291.22907	1	12392.92
722.50026	1	19998.84
766.52663	1	22383.19
810.55271	1	10495.45

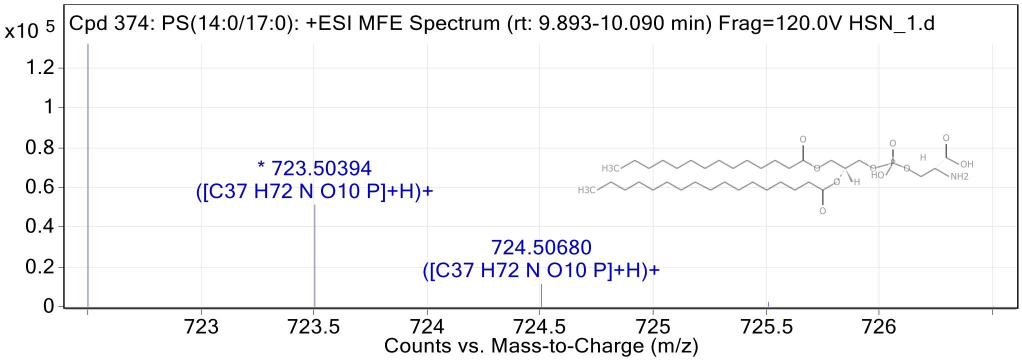

Peak List

m/z	z	Abund	Name	Formula	Ion	Score (DB)	Hits (DB)
722.50011	1	132250.94	PS(14:0/17:0)	C37 H72 N O10 P	(M+H)+	84.62	8
723.50394	1	51255.63		C37 H72 N O10 P	(M+H)+		
724.5068	1	11511.86		C37 H72 N O10 P	(M+H)+		
725.50979	1	2452.25		C37 H72 N O10 P	(M+H)+		
726.50731	1	227.34		C37 H72 N O10 P	(M+H)+		

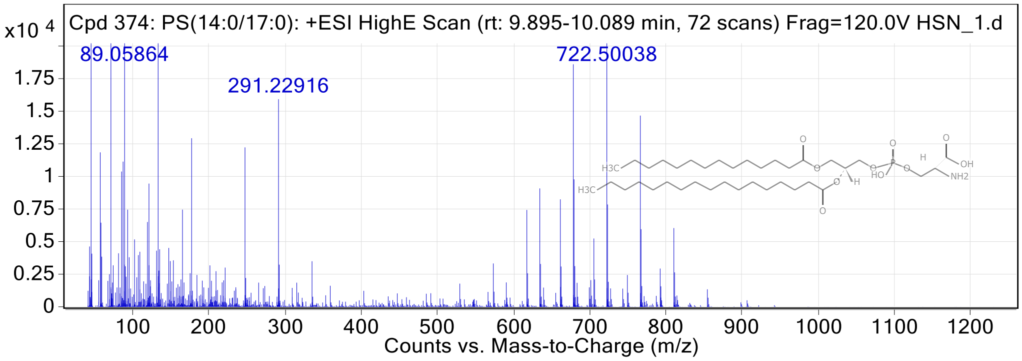

Peak List

m/z	z	Abund

45.0333		51667.63
71.08475		21233.5
89.05864	1	74959.63
133.08421	1	44101.12
177.11029		12920.54
247.20313	1	12220.07
291.22916	1	15912.9
678.47458	1	18591.63
722.50038	1	20197.47
766.52656	1	14657.5

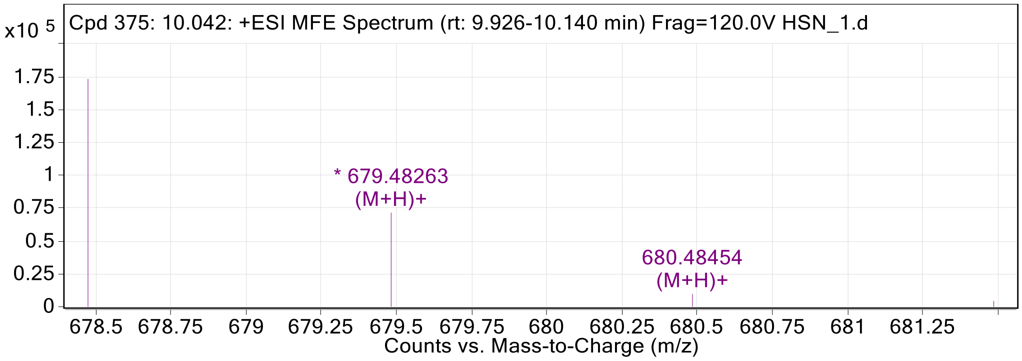

Peak List

m/z	z	Abund	Ion
678.4746	1	173301.91	(M+H)+
679.48263	1	71595.96	(M+H)+
680.48454	1	9760.43	(M+H)+
681.48646	1	4448.54	(M+H)+

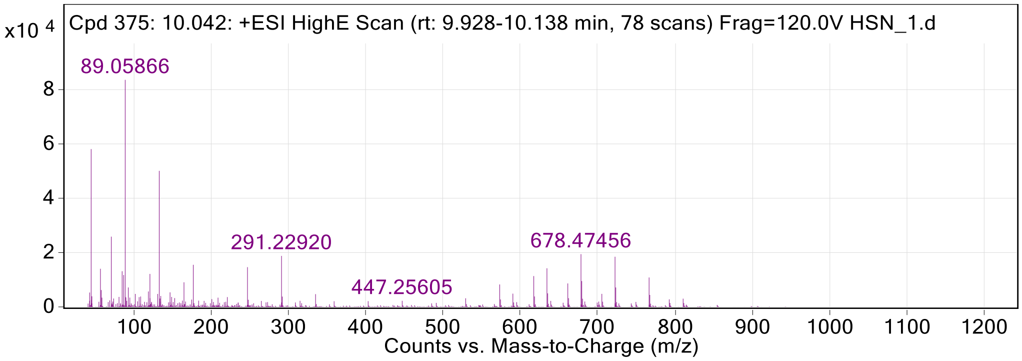

Peak List

m/z	z	Abund
45.03331		58039.89
71.08477		25836.44
89.05866	1	83499.25
133.08418	1	50037.09
177.1103		15485.44
247.20317	1	14617.55
291.2292	1	18751.02
634.44849	1	14204.93
678.47456	1	19408.61
722.50038	1	18449.64

Qualitative Analysis Report

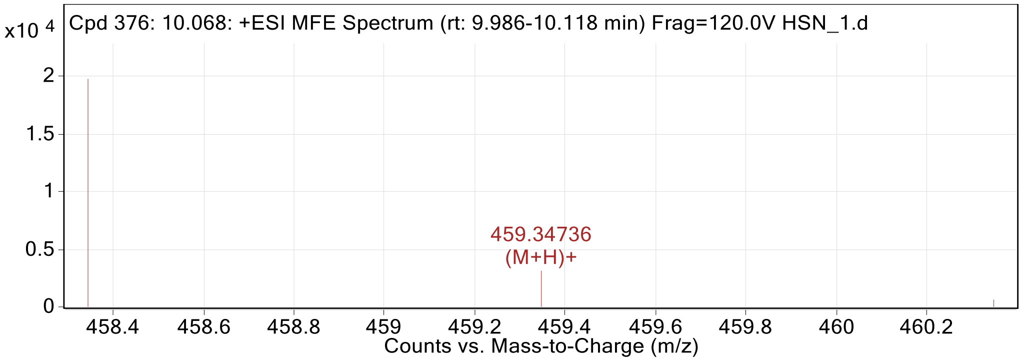

Peak List

m/z	z	Abund	Ion
458.34428	1	19797.05	(M+H)+
459.34736	1	3136.25	(M+H)+
460.34849	1	633.13	(M+H)+

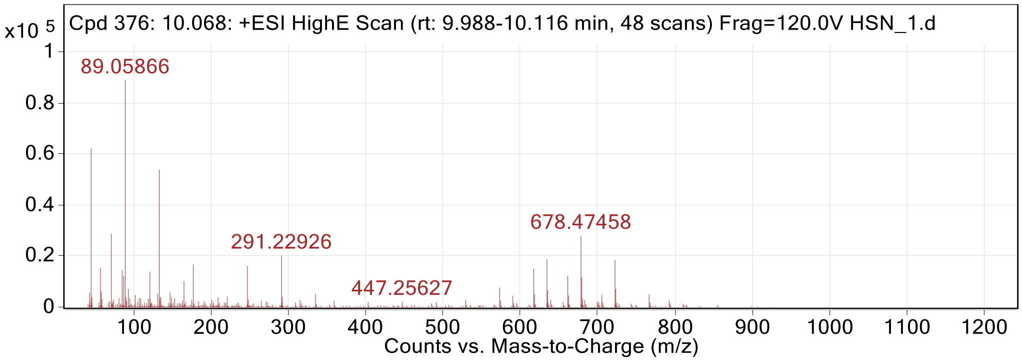

Peak List

m/z	z	Abund
45.03331		62073.78
71.08478		28633.07
89.05866	1	88887.2
133.08418	1	53717.51
177.11023		16741.04
247.20316	1	16121.55
291.22926	1	20246.4
634.4485	1	18694.9
678.47458	1	28005.84
722.50046	1	18406.31

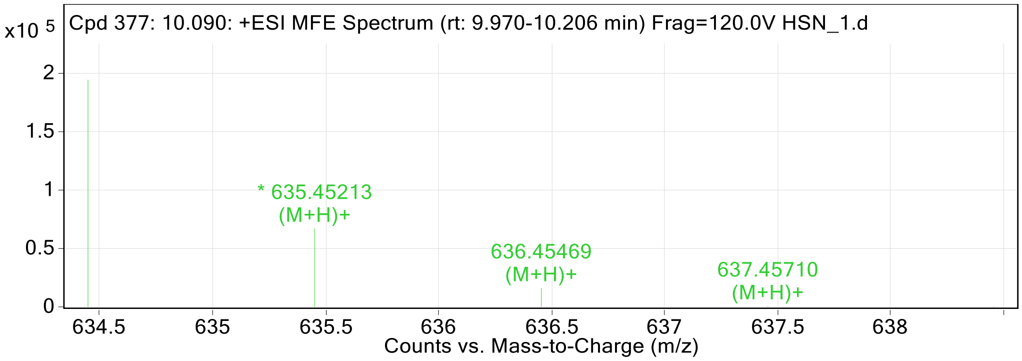

Peak List

m/z	z	Abund	Ion
634.44868	1	194975.72	(M+H)+
635.45213	1	67259.51	(M+H)+

636.45469	1	16252.76	(M+H)+
637.4571	1	714.88	(M+H)+
638.45629	1	463.86	(M+H)+

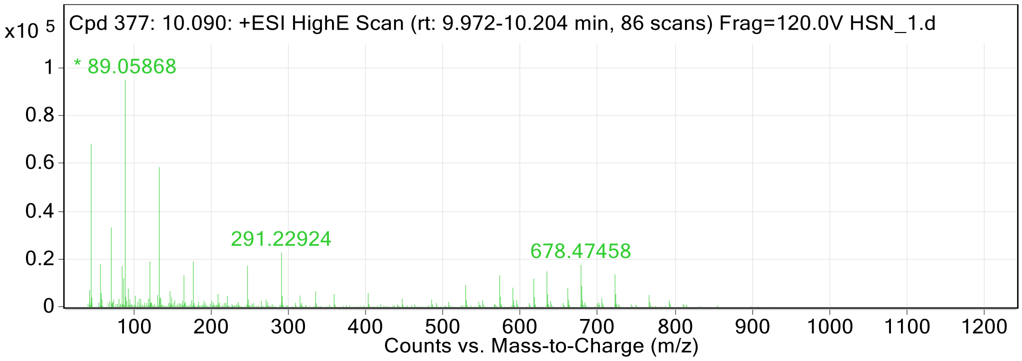

Peak List

m/z	z	Abund
45.03334		68197.91
57.0694		17937.56
71.08481		33184.4
89.05868	1	94867.2
121.06304		18996.91
133.0842	1	58388.96
177.11025		18942.25
247.20316	1	17228.68
291.22924	1	22672.47
678.47458	1	17560.26

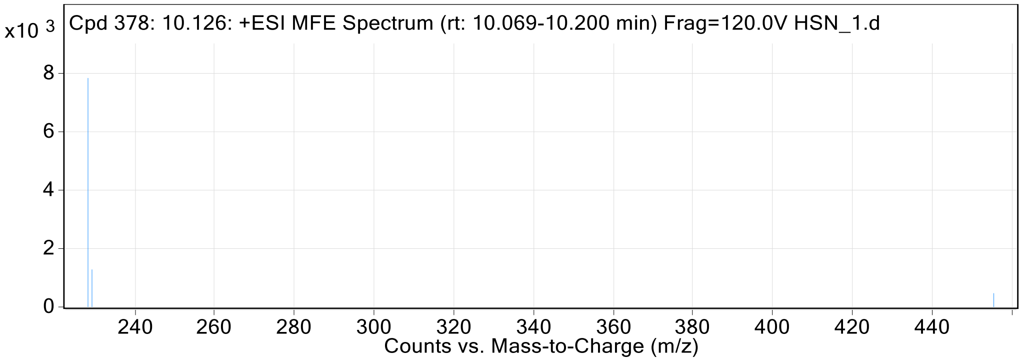

Peak List

m/z	z	Abund	Ion
228.23008	1	7824.51	(M+H)+
229.23304	1	1281.46	(M+H)+
455.45076	1	470.48	(2M+H)+

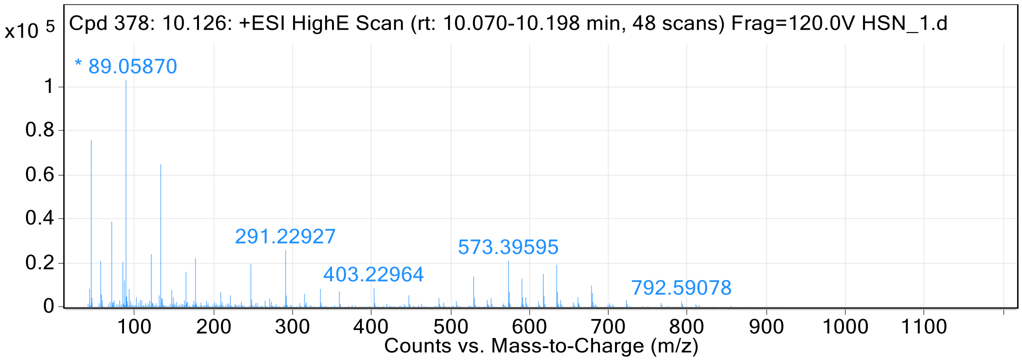

Peak List

m/z	z	Abund
45.03336	1	75778.82
57.0694		20812.38
71.08483	1	38668.01
85.1001		20418.07
89.0587	1	103108.09
121.06305	1	23923.38
133.08421	1	64734.2
177.11028	1	22118.23
291.22927	1	25761.89
573.39595	1	20936.53

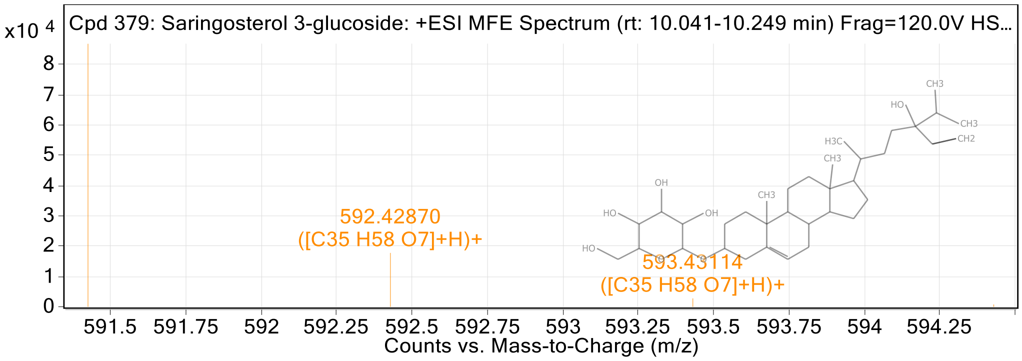

Peak List

m/z	z	Abund	Name	Formula	Ion	Score (DB)	Hits (DB)
591.42636	1	86790.02	Saringosterol 3-glucoside	C35 H58 O7	(M+H)+	74.69	3
592.4287	1	17768.45		C35 H58 O7	(M+H)+		
593.43114	1	2761.11		C35 H58 O7	(M+H)+		
594.42844	1	761.47		C35 H58 O7	(M+H)+		

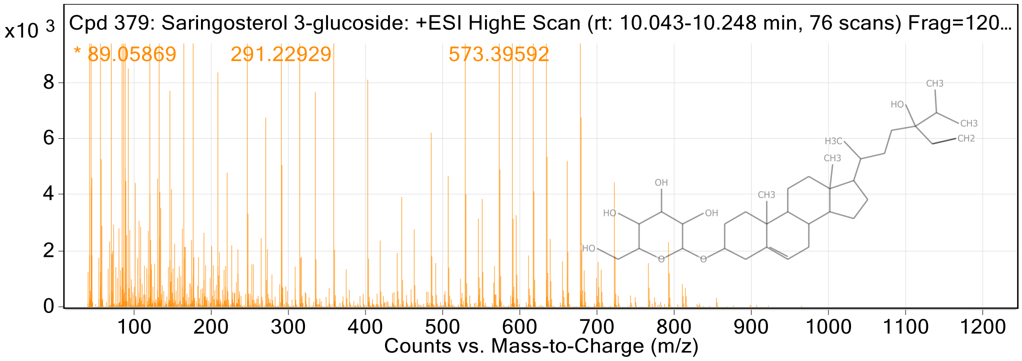

Peak List

m/z	z	Abund
45.03337	1	79446.7
57.06943		22846.13
71.08483	1	41868.32
85.10011		21579.29
89.05869	1	105660.36
121.06306	1	29950.38
133.08421	1	66065.63
165.08886	1	20152.26
177.11019	1	21655.64
291.22929	1	25692.77

Qualitative Analysis Report

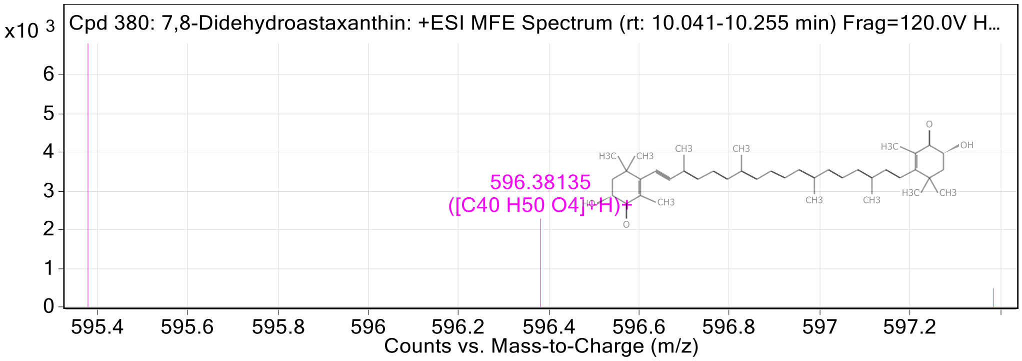

Peak List

m/z	z	Abund	Name	Formula	Ion	Score (DB)	Hits (DB)
595.37824	1	6813.8	7,8-Didehydroastaxanthin	C40 H50 O4	(M+H)+	90.46	3
596.38135	1	2282.08		C40 H50 O4	(M+H)+		
597.38554	1	480.12		C40 H50 O4	(M+H)+		

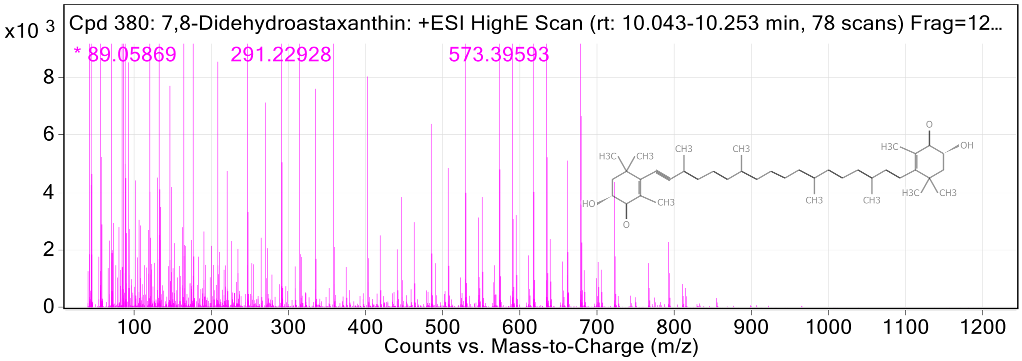

Peak List

m/z	z	Abund
45.03337	1	79818.27
57.06944		23127.12
71.08483	1	42234.93
85.10011		21735.17
89.05869	1	105865.04
121.06306	1	30602.49
133.08421	1	66158.74
165.08886	1	20661.01
177.11019	1	21624.08
291.22928	1	25674.71

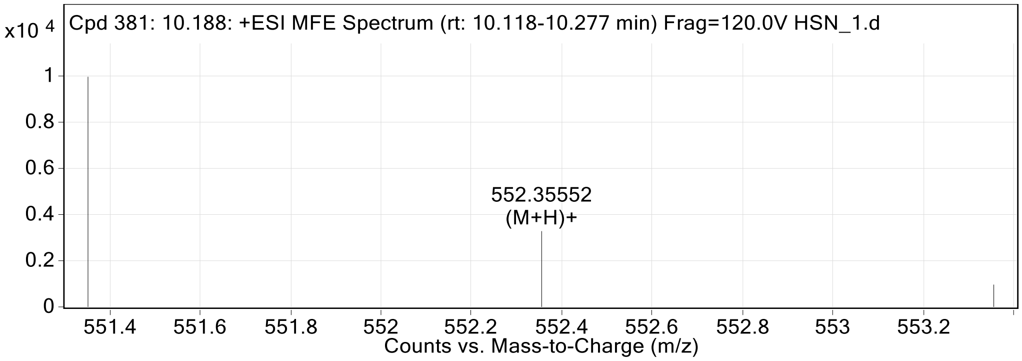

Peak List

m/z	z	Abund	Ion
551.35184	1	9954.68	(M+H)+
552.35552	1	3277.91	(M+H)+

553.35571	1	964.61	(M+H)+

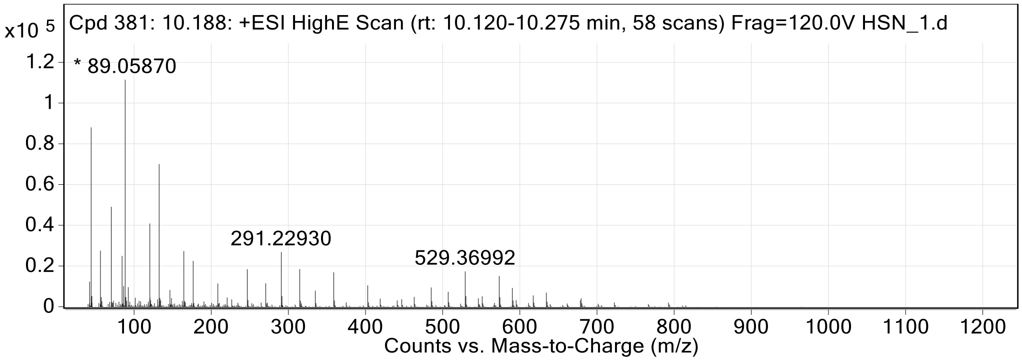

Peak List

m/z	z	Abund
45.03339	1	87993.42
57.06944		27556.28
71.08483	1	49054.69
85.10014		24931.69
89.0587	1	111367.17
121.06304	1	40840.1
133.08423	1	69957.09
165.08887	1	27348.63
177.11015	1	22470.31
291.2293	1	26825.76

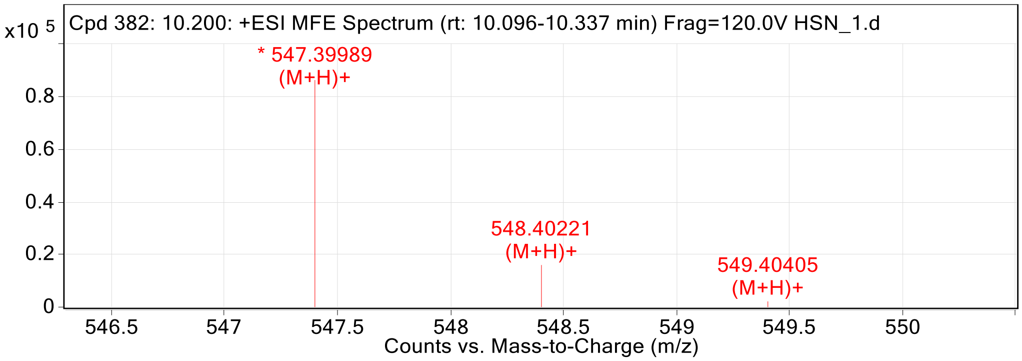

Peak List

m/z	z	Abund	Ion
546.39586	1	0	(M+H)+
547.39989	1	86380.7	(M+H)+
548.40221	1	15999.98	(M+H)+
549.40405	1	2165.23	(M+H)+
550.40359	1	0	(M+H)+

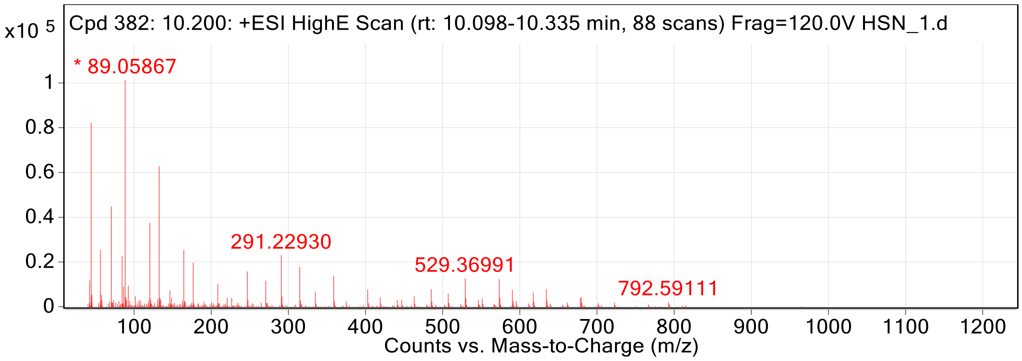

Qualitative Analysis Report

Peak List

m/z	z	Abund
45.03336	1	82199.32
57.06943		25411.09
71.08482	1	44785.28
85.10012		22724.78
89.05867	1	101226.42
121.06303	1	37566.11
133.08421	1	62813.6
165.08888	1	25436.53
177.11016		19666.65
291.2293	1	22926.3

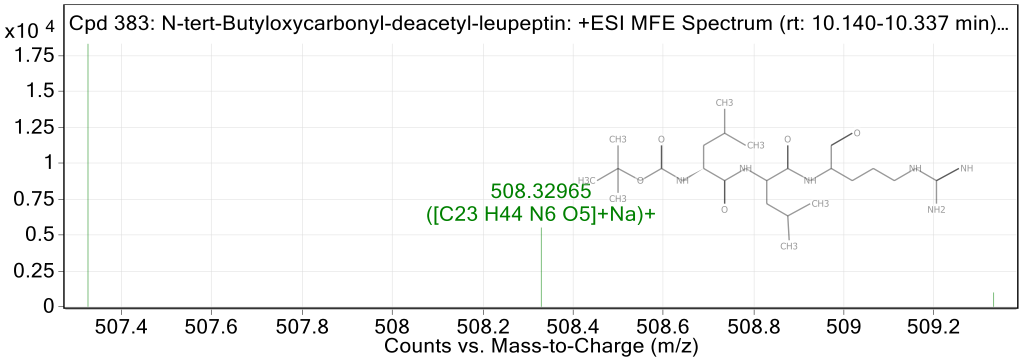

Peak List

m/z	z	Abund	Name	Formula	Ion	Score (DB)	Hits (DB)
507.32561	1	18323.86	N-tert-Butyloxycarbonyl-deacetyl-leupeptin	C23 H44 N6 O5	(M+Na)+	96.86	2
508.32965	1	5519.34		C23 H44 N6 O5	(M+Na)+		
509.33232	1	997.09		C23 H44 N6 O5	(M+Na)+		

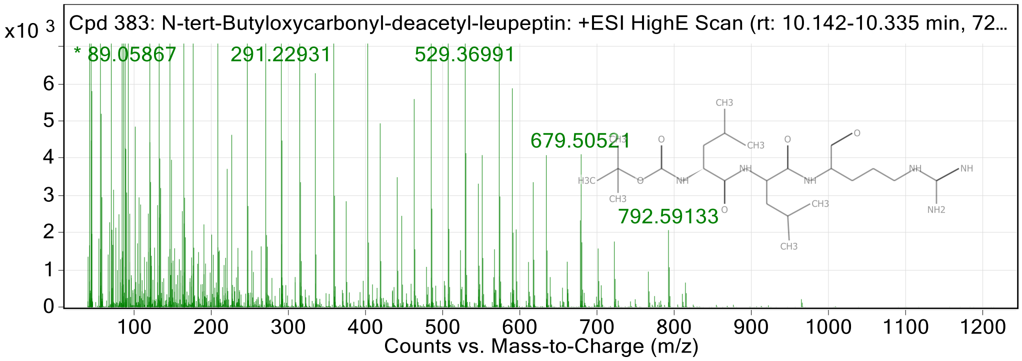

Peak List

m/z	z	Abund
45.03336	1	84684.28
57.06944		26941.29
71.08482	1	46989.7
85.10013		23617.06
89.05867	1	101886.98
121.06303	1	41873.93
133.08421	1	63252.77
165.08888	1	28286.95
291.22931	1	22633.82
315.17762	1	21079.12

Qualitative Analysis Report

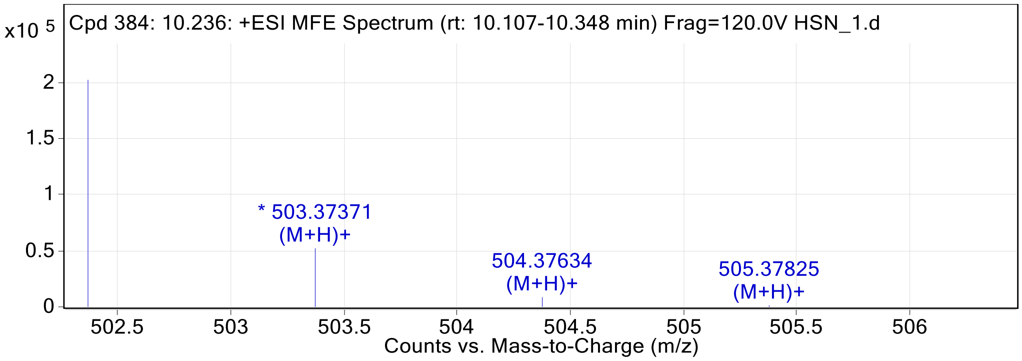

Peak List

m/z	z	Abund	Ion
502.3702	1	202226.83	(M+H)+
503.37371	1	52142.28	(M+H)+
504.37634	1	8659	(M+H)+
505.37825	1	1344.76	(M+H)+
506.37042	1	47.31	(M+H)+

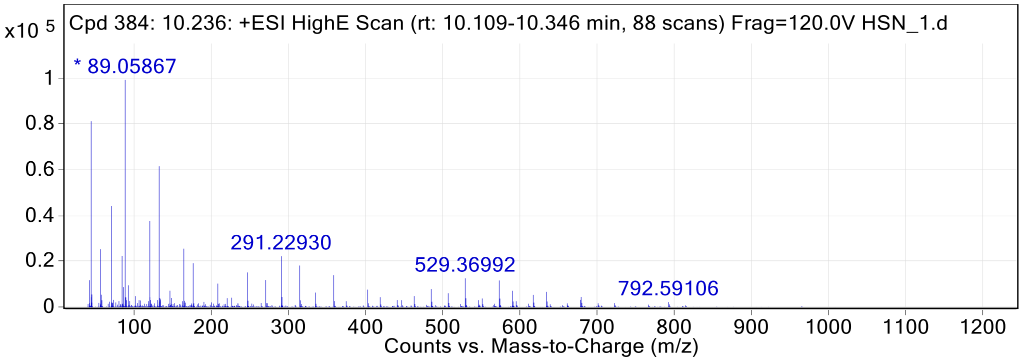

Peak List

m/z	z	Abund
45.03336	1	81156.61
57.06943		25246.96
71.08482	1	44185.19
85.10012		22341
89.05867	1	99225.32
121.06302	1	37633.25
133.08421	1	61472.26
165.08888	1	25476.72
177.11013		19120.78
291.2293	1	22173.78

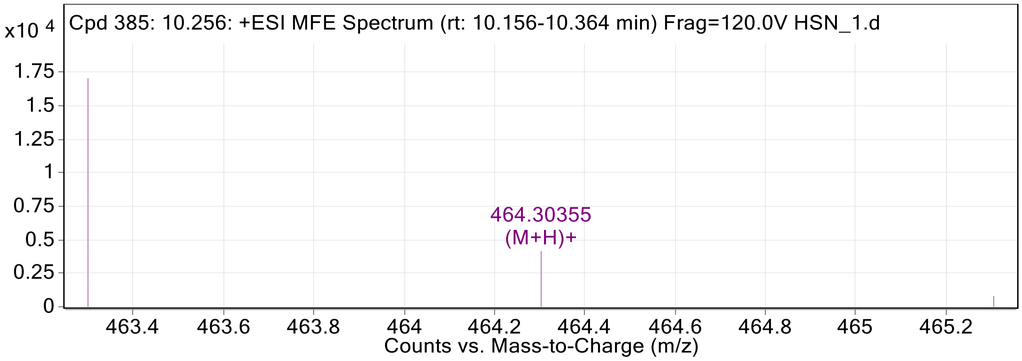

Peak List

m/z	z	Abund	Ion

463.29973	1	17043.08	(M+H)+
464.30355	1	4151.89	(M+H)+
465.30616	1	801.18	(M+H)+

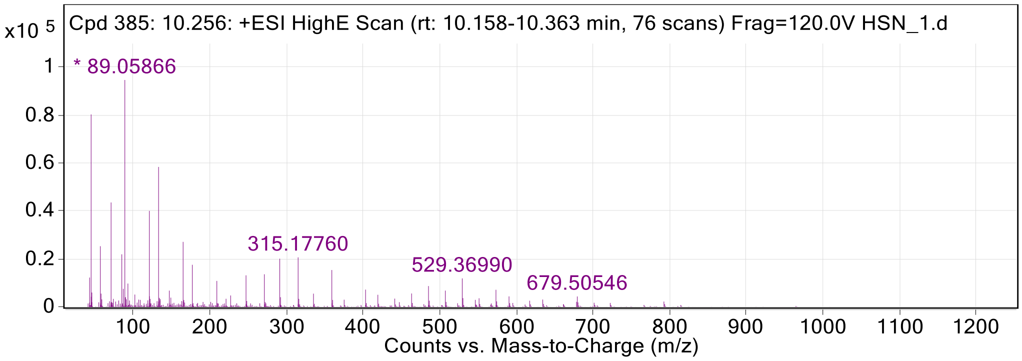

Peak List

m/z	z	Abund
45.03335	1	80333.44
57.06944		25279.45
71.0848	1	43552.35
85.10012		21897.42
89.05866	1	94710.15
121.06301	1	40030.07
133.0842	1	58383.34
165.08889	1	27090.67
291.22932	1	20053.88
315.1776	1	20622.9

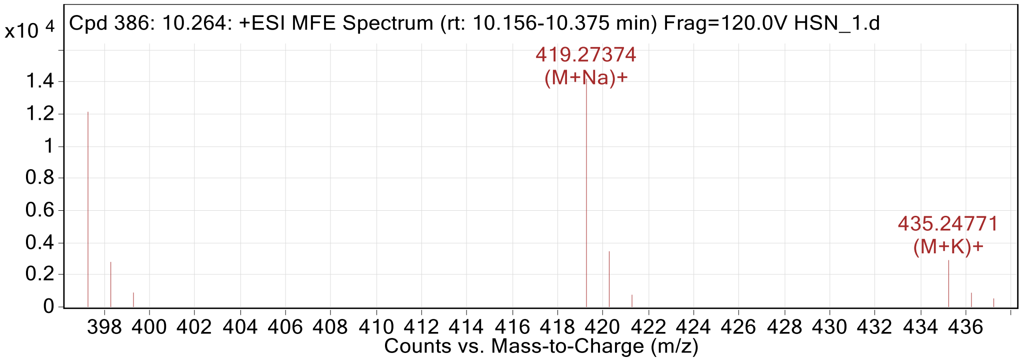

Peak List

m/z	z	Abund	Ion
397.29252	1	12150.88	(M+H)+
398.29881	1	2803.35	(M+H)+
399.29802	1	896.39	(M+H)+
419.27374	1	14205.91	(M+Na)+
420.27781	1	3465.86	(M+Na)+
421.2821	1	760.34	(M+Na)+
435.24771	1	2917	(M+K)+
436.25084	1	885.57	(M+K)+
437.23529	1	531.68	(M+K)+

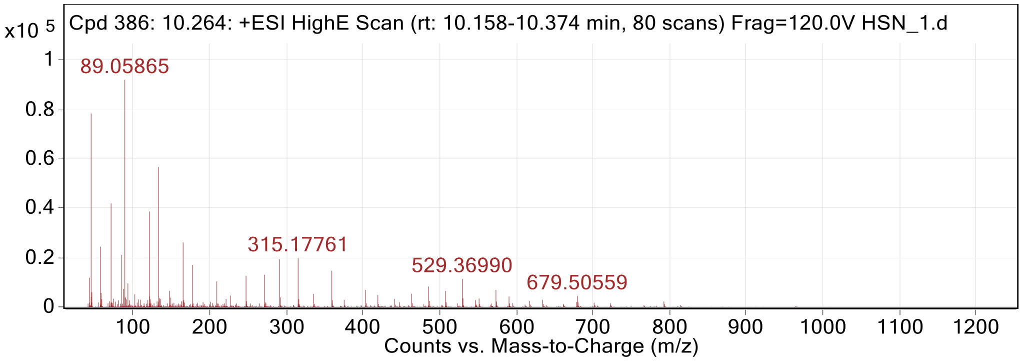

Peak List

m/z	z	Abund
45.03335	1	78370.7
57.06944		24429
71.0848	1	41944.76
85.10012		21096.41
89.05865	1	92008.58
121.06301	1	38630.66
133.0842	1	56692.64
165.08889	1	26112.17
291.22931	1	19314.12
315.17761	1	19780.45

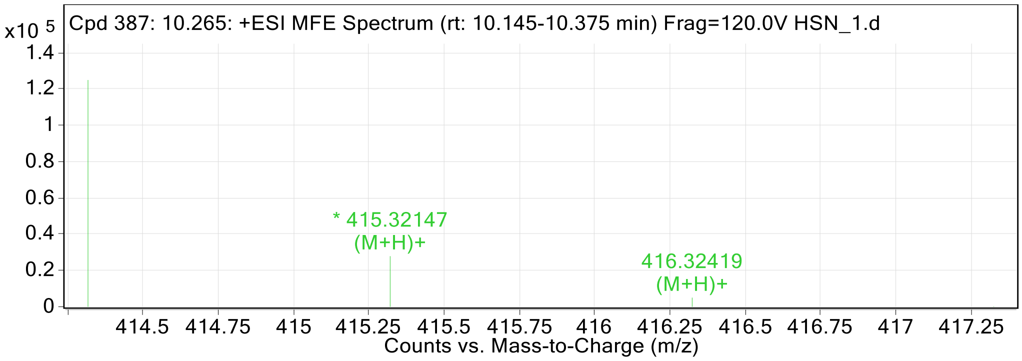

Peak List

m/z	z	Abund	Ion
414.31819	1	124904.52	(M+H)+
415.32147	1	27875.42	(M+H)+
416.32419	1	5040.81	(M+H)+
417.32556	1	224.59	(M+H)+

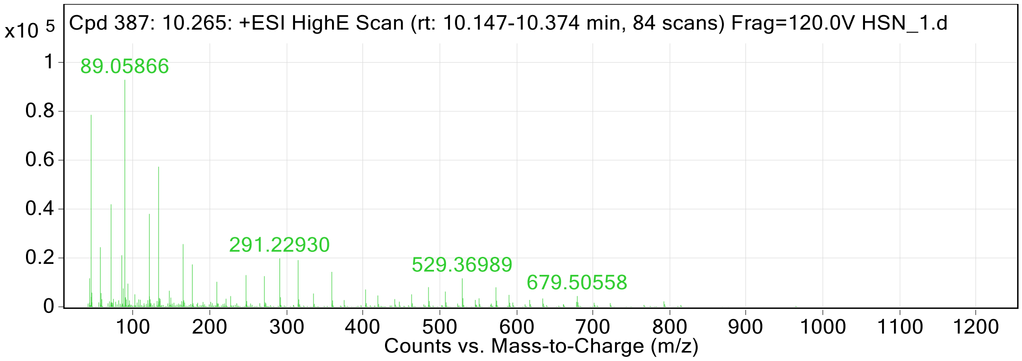

Peak List

m/z	z	Abund
45.03335	1	78362.92

57.06943		24338.36
71.08481	1	41851.71
85.10012		21059.07
89.05866	1	92652.35
121.06302	1	37936.98
133.0842	1	57196.54
165.08888	1	25611.05
291.2293	1	19758.44
315.17761	1	19069.9

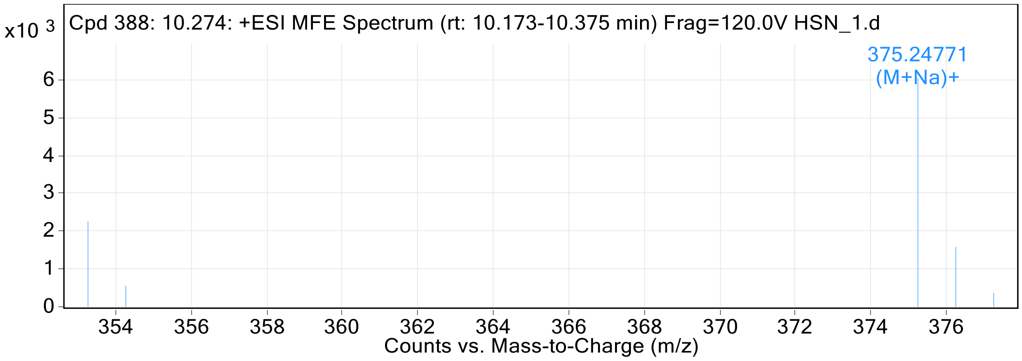

Peak List

m/z	z	Abund	Ion
353.26543	1	2267.57	(M+H)+
354.26749	1	557.52	(M+H)+
375.24771	1	6054.4	(M+Na)+
376.25066	1	1586.37	(M+Na)+
377.25599	1	364.72	(M+Na)+

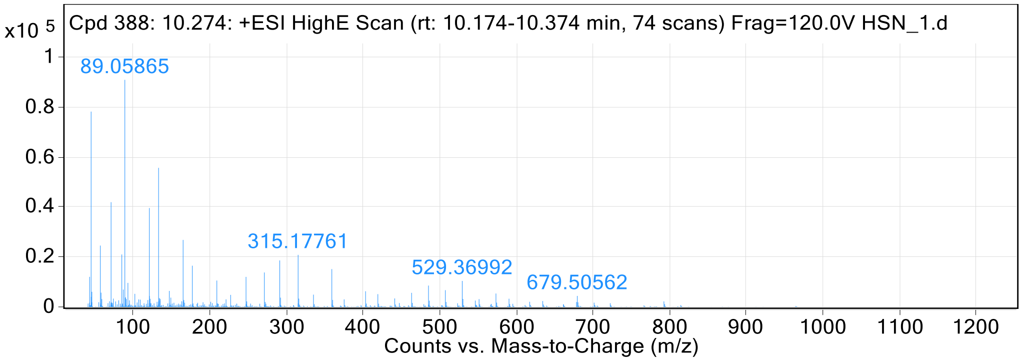

Peak List

m/z	z	Abund
45.03335	1	78142.1
57.06944		24536.64
71.0848	1	41900.29
85.10012		20979.98
89.05865	1	90841.58
121.06302	1	39554.13
133.0842	1	55638.82
165.08888	1	26798.26
291.22933	1	18593.98
315.17761	1	20830.35

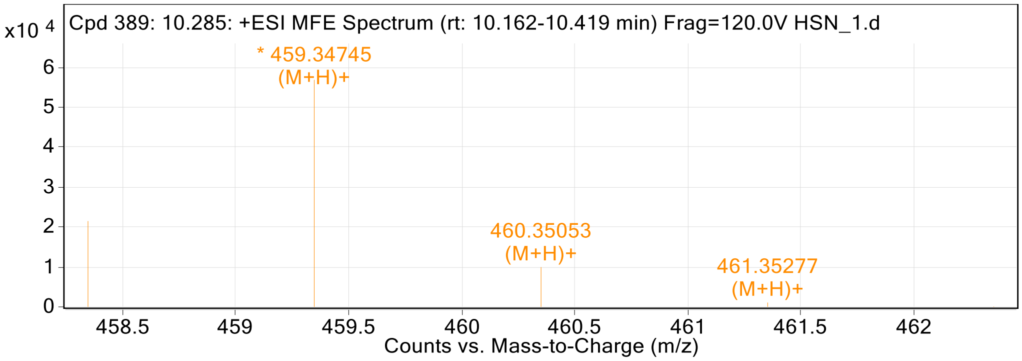

Peak List

m/z	z	Abund	Ion
458.34695	1	21501.3	(M+H)+
459.34745	1	56923.63	(M+H)+
460.35053	1	9963.36	(M+H)+
461.35277	1	1086.28	(M+H)+
462.35264	1	112.93	(M+H)+

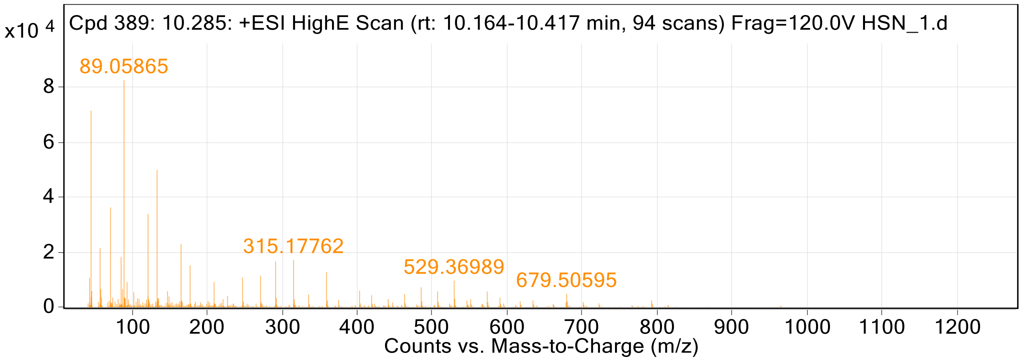

Peak List

m/z	z	Abund
45.03334	1	71458.94
57.06944		21450.97
71.0848	1	36216.4
85.10011		18167.46
89.05865	1	82628.76
121.06301	1	33861.86
133.08427	1	49907.47
165.08888	1	22837.35
291.2293	1	16564.2
315.17762	1	17156.67

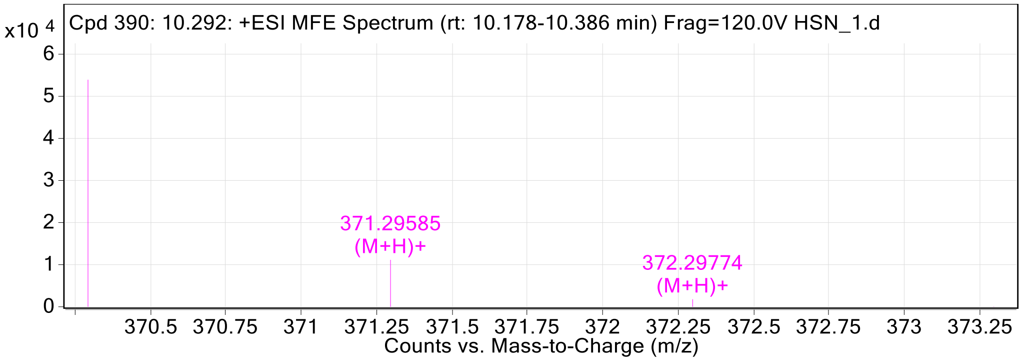

Peak List

m/z	z	Abund	Ion

370.2926	1	53884.24	(M+H)+
371.29585	1	11138.08	(M+H)+
372.29774	1	1791.78	(M+H)+
373.29622	1	65.75	(M+H)+

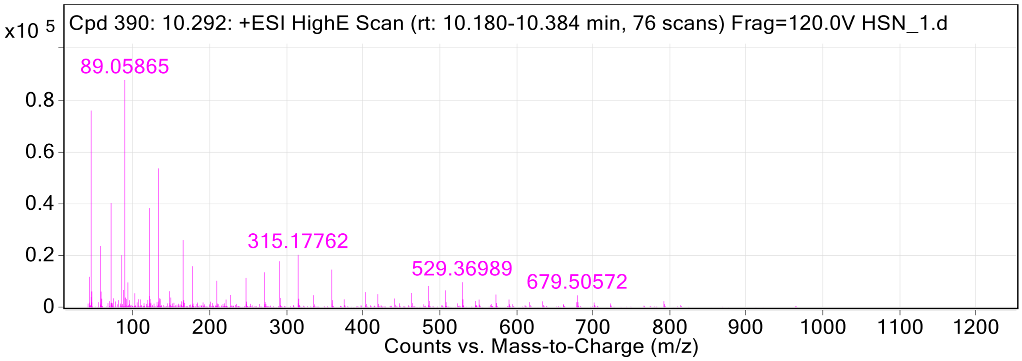

Peak List

m/z	z	Abund
45.03335	1	75973.16
57.06944		23680.54
71.0848	1	40158.93
85.10012		20124.35
89.05865	1	87738.69
121.06302	1	38289.03
133.08421	1	53589.36
165.08889	1	25914.13
291.22931	1	17671.39
315.17762	1	20224.36

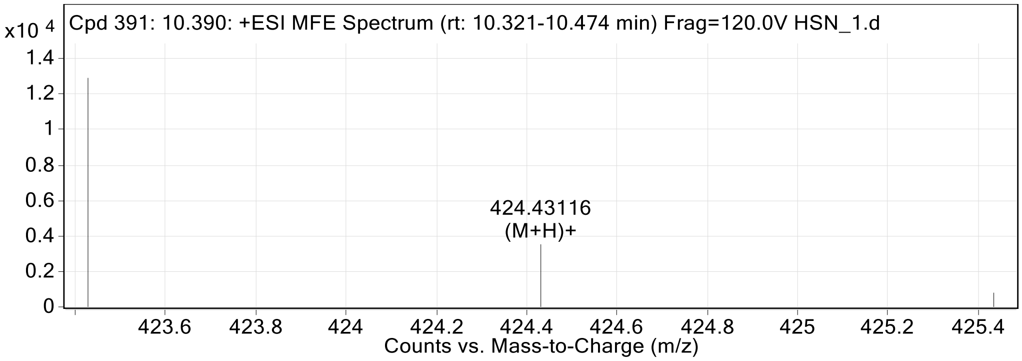

Peak List

m/z	z	Abund	Ion
423.42803	1	12916.58	(M+H)+
424.43116	1	3530.36	(M+H)+
425.43452	1	800.56	(M+H)+

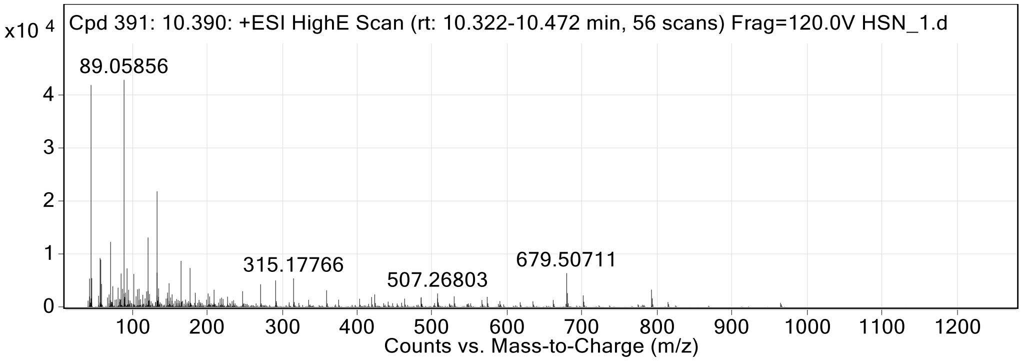

Peak List

m/z	z	Abund
45.03325		42023.8
57.06938		9251.46
58.06474		8949.37
71.08474		12299.38
89.05856	1	42988.94
93.06863		7290.32
121.0629	1	13140.62
133.08531	1	21878.42
165.08887		8717.77
177.1101		7359.68

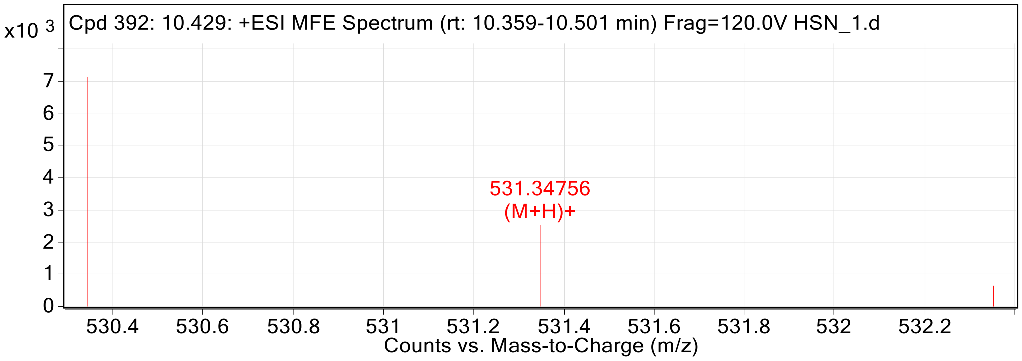

Peak List

m/z	z	Abund	Ion
530.34382	1	7138.76	(M+H)+
531.34756	1	2538.77	(M+H)+
532.3532	1	647.78	(M+H)+

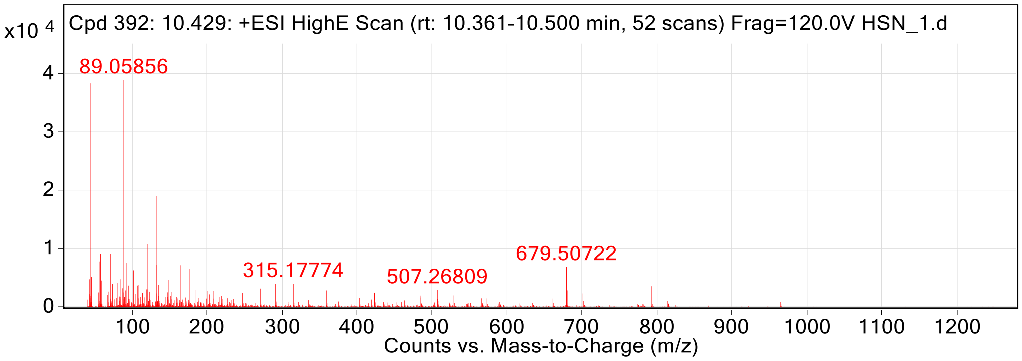

Peak List

m/z	z	Abund
45.03327		38252.23
57.06934		7720.56

58.06474		9012.69
71.08474		8990.47
89.05856	1	38816.96
93.06863		7521.26
121.06291		10707.06
133.08569		18975.19
133.09761		7102.23
165.0889		7080.18

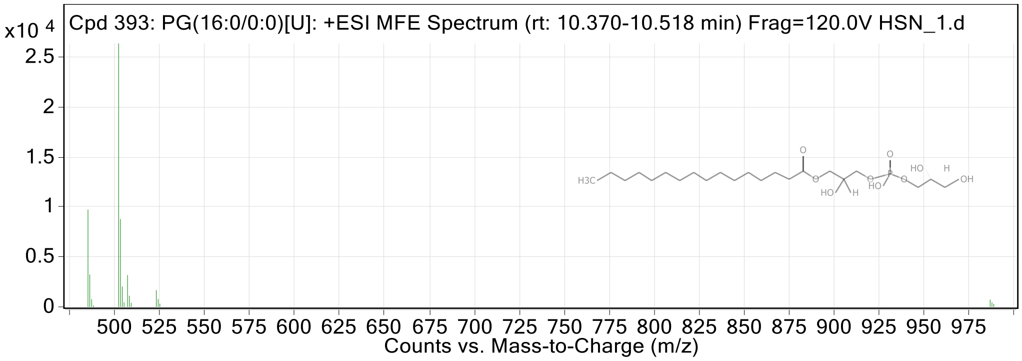

Peak List

m/z	z	Abund	Name	Formula	Ion	Score (DB)	Hits (DB)
485.2865	1	9747.13			(M+H)+		
486.29068	1	3241.85			(M+H)+		
487.29331	1	776.68			(M+H)+		
502.31282	1	26387.37	PG(16:0/0:0)[U]	C22 H45 O9 P	(M+NH4)+	88	2
503.31566	1	8797.29		C22 H45 O9 P	(M+NH4)+		
504.32011	1	2050.98		C22 H45 O9 P	(M+NH4)+		
507.26869	1	3188.76			(M+Na)+		
508.27331	1	1116.15			(M+Na)+		
523.24266	1	1675.11			(M+K)+		
524.245	1	789.45			(M+K)+		

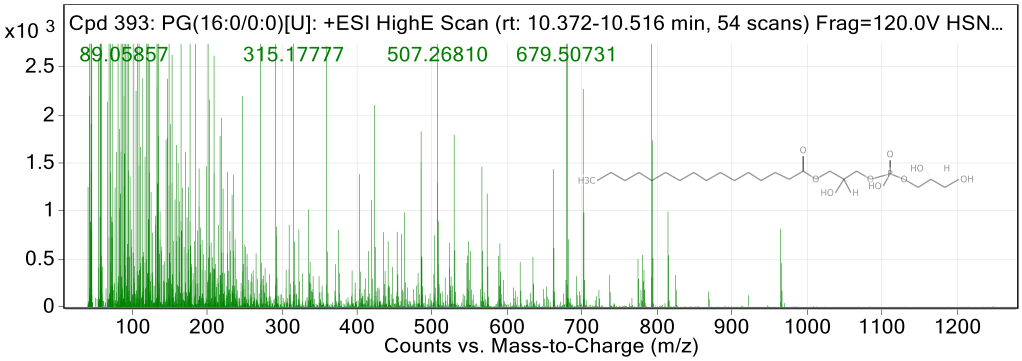

Peak List

m/z	z	Abund
45.03327		37676.62
57.06935		7512.05
58.06474		8870.99
71.08473		8531.05
89.05857	1	38058.91
93.06862		7744.35
121.06293		10321.91
133.08573		18388.44
165.08891		6895.35
679.50731	1	6966.92

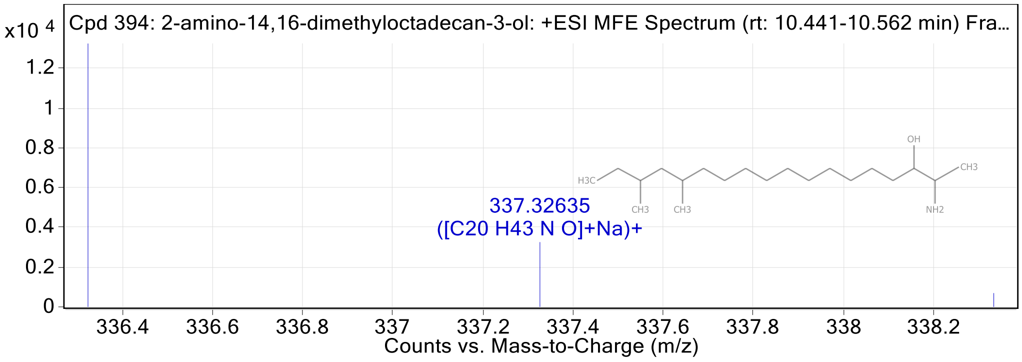

Peak List

m/z	z	Abund	Name	Formula	Ion	Score (DB)	Hits (DB)
336.32339	1	13250.37	2-amino-14,16-dimethyloctadecan-3-ol	C20 H43 N O	(M+Na)+	95.47	1
337.32635	1	3257.43		C20 H43 N O	(M+Na)+		
338.33352	1	693.69		C20 H43 N O	(M+Na)+		

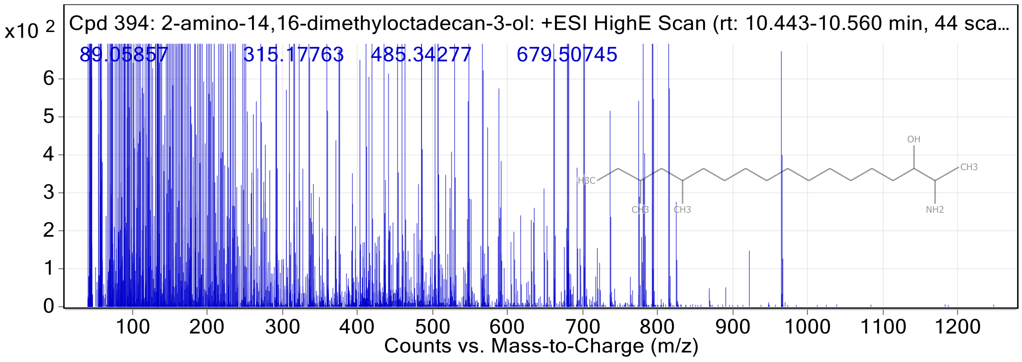

Peak List

m/z	z	Abund
45.03325		35808.94
57.06936		6916.89
58.06476		7875.18
71.08472		7256.66
89.05857	1	35811.05
93.06858		8547.93
121.06295		9622.94
133.08489		17583.04
165.08889		6543.45
679.50745	1	7344.77

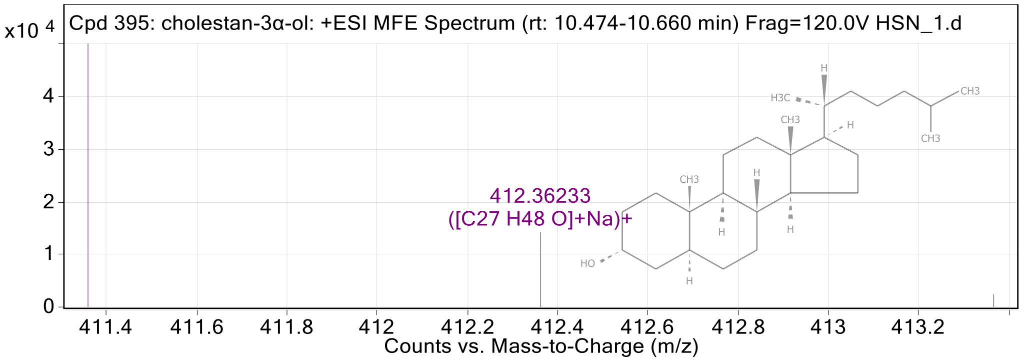

Peak List

m/z	z	Abund	Name	Formula	Ion	Score (DB)	Hits (DB)
411.35947	1	50093.63	cholestan-3α-ol	C27 H48 O	(M+Na)+	98.85	7

412.36233	1	14212.16		C27 H48 O	(M+Na)+		
413.3662	1	2435.83		C27 H48 O	(M+Na)+		

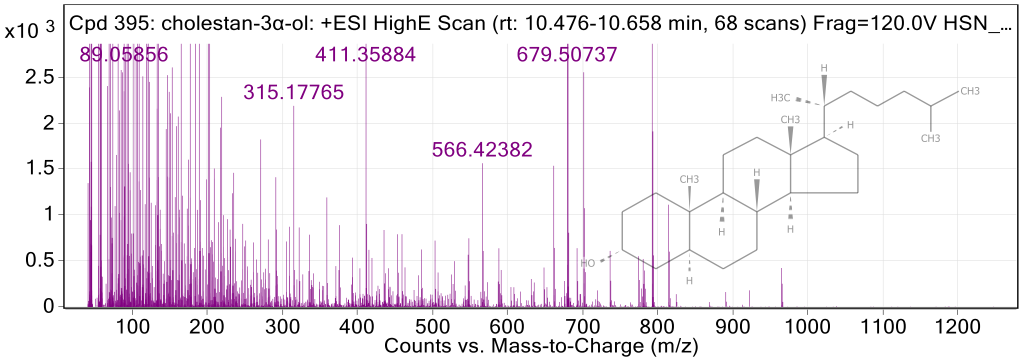

Peak List

m/z	z	Abund
45.03325		30690.9
58.06473		8207.47
81.06883		5178.77
89.05856	1	30989.4
93.06858		8127.39
95.08413		5381.96
102.1263		5557.99
121.06296		5814.29
133.08436	1	15614.52
679.50737	1	7437.63

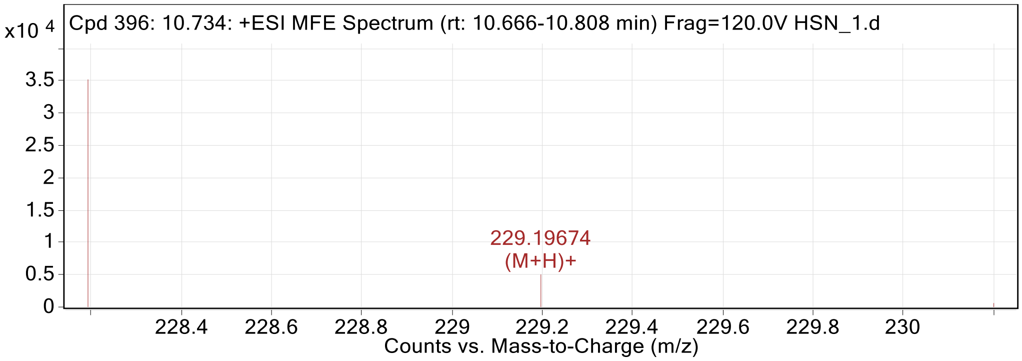

Peak List

m/z	z	Abund	Ion
228.19337	1	35224.06	(M+H)+
229.19674	1	5036.16	(M+H)+
230.20011	1	604.99	(M+H)+

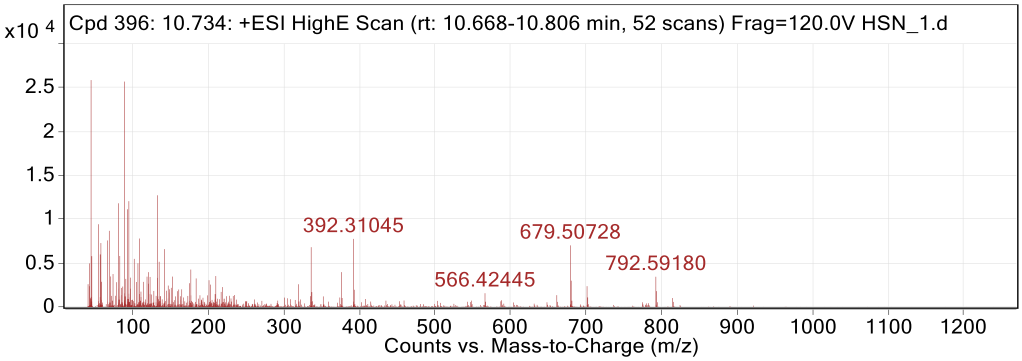

Peak List

m/z	z	Abund

45.03327		25858.57
55.0538		9416.46
69.06913		8665.47
81.06889		11806.32
89.05858	1	25686.88
93.06864		11124.69
95.08425		12049.11
109.09965		7788.79
133.08466		12718.84
392.31045	1	7745.43

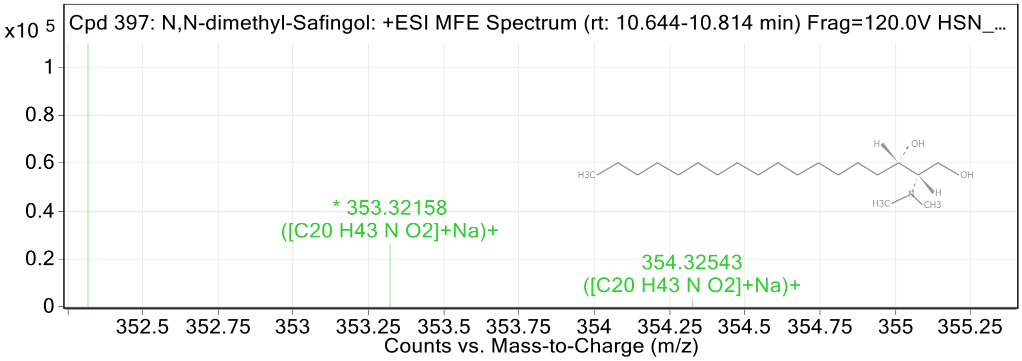

Peak List

m/z	z	Abund	Name	Formula	Ion	Score (DB)	Hits (DB)
352.31809	1	110147.18	N,N-dimethyl-Safingol	C20 H43 N O2	(M+Na)+	98.97	1
353.32158	1	26162.87		C20 H43 N O2	(M+Na)+		
354.32543	1	3327.61		C20 H43 N O2	(M+Na)+		
355.32751	1	290.13		C20 H43 N O2	(M+Na)+		

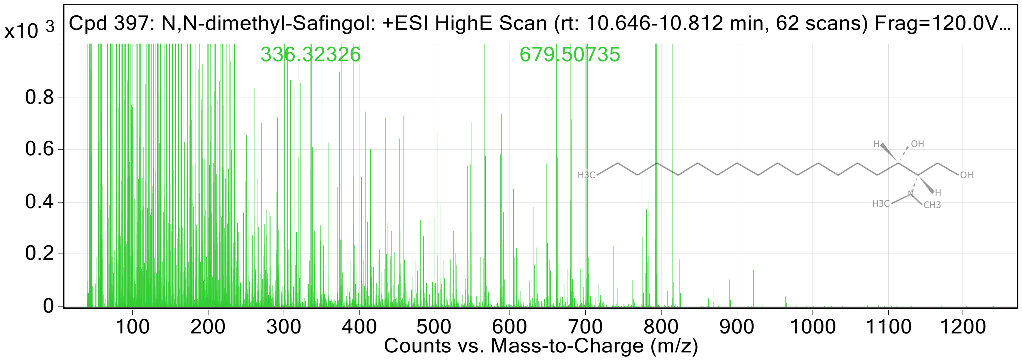

Peak List

m/z	z	Abund
45.03327		26157.36
55.05381		8825.79
58.06474		7468.28
69.06914		8161.38
81.06887		11033.19
89.05858	1	25998.26
93.06864		10872.46
95.08424		11185.05
109.09964		7412.29
133.08466		12926.56

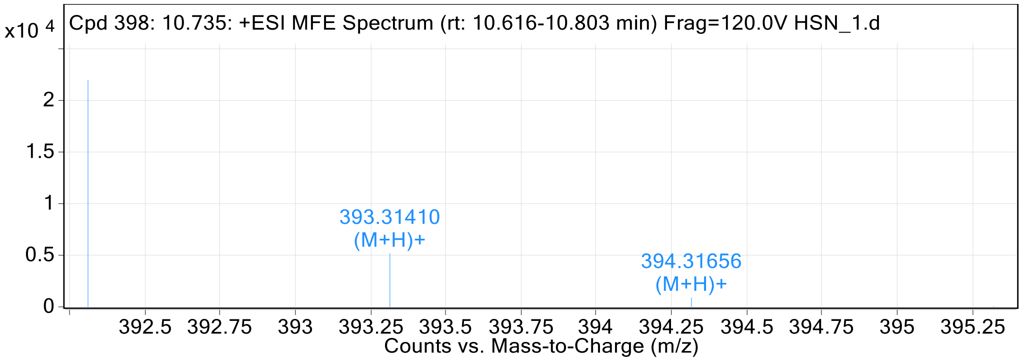

Peak List

m/z	z	Abund	Ion
392.31061	1	22000.93	(M+H)+
393.3141	1	5173.17	(M+H)+
394.31656	1	899.29	(M+H)+
395.32143	1	59.15	(M+H)+

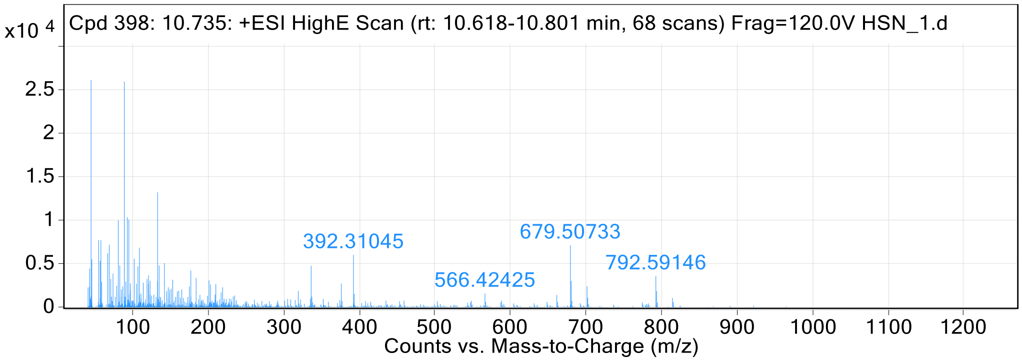

Peak List

m/z	z	Abund
45.03327		26176.99
55.0538		7710.37
58.06473		7720.24
69.06913		7163.43
81.06886		9984.36
89.05857	1	26001.48
93.06862		10347.87
95.08424		10078.3
133.08461		13227.04
679.50733	1	7123.74

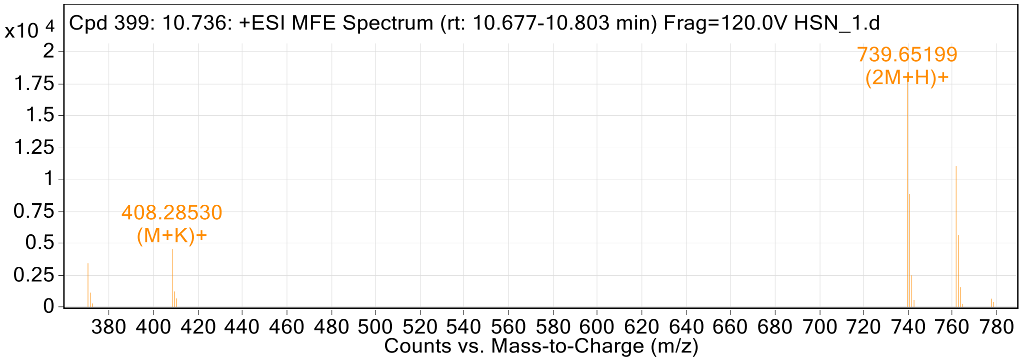

Peak List

m/z	z	Abund	Ion
370.32878	1	3420.31	(M+H)+

371.33231	1	1114.76	(M+H)+
408.2853	1	4542.31	(M+K)+
409.28852	1	1205.21	(M+K)+
739.65199	1	17858.31	(2M+H)+
740.65559	1	8876.09	(2M+H)+
741.6582	1	2477.38	(2M+H)+
761.63445	1	11038.38	(2M+Na)+
762.63723	1	5636.9	(2M+Na)+
763.64061	1	1548.78	(2M+Na)+

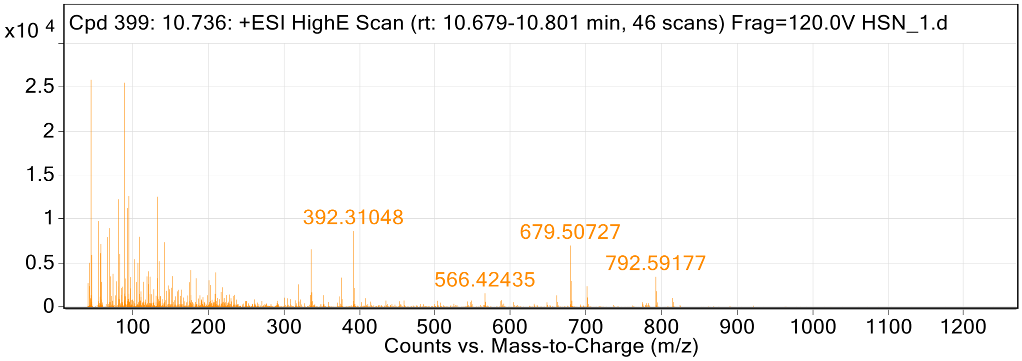

Peak List

m/z	z	Abund
45.03326		25874.39
55.0538		9777.99
69.06914		8964.12
81.06889		12242.18

m/z	z	Abund
89.05859	1	25549.49
93.06864		11251.54
95.08424		12627.39
109.09964		7984.96
133.0847		12550.76
392.31048	1	8643.23

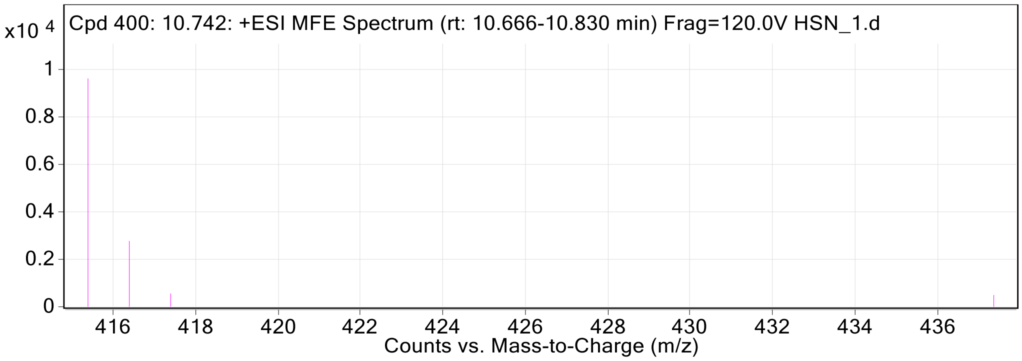

Peak List

m/z	z	Abund	Ion
415.38604	1	9632.05	(M+H)+
416.38894	1	2777.44	(M+H)+
417.39179	1	558.73	(M+H)+
437.36867	1	495.82	(M+Na)+

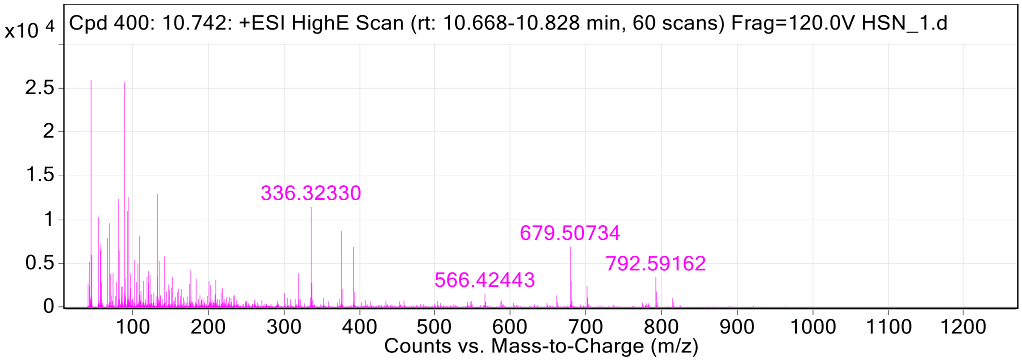

Peak List

m/z	z	Abund
45.03326		25980.95
55.05379		10324.86
69.06914		9549.92
81.06888		12375.23
89.05858	1	25702.32
93.06864		10958.44
95.08423		12553.21
133.08465		12922.64
336.3233	1	11455.29
376.31562	1	8642.55

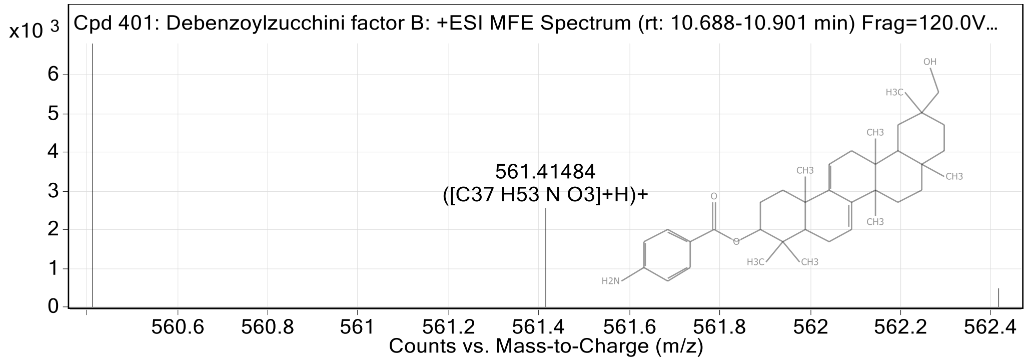

Peak List

m/z	z	Abund	Name	Formula	Ion	Score (DB)	Hits (DB)
560.41216	1	6807.18	Debenzoylzucchini factor B	C37 H53 N O3	(M+H)+	90.42	1
561.41484	1	2553.88		C37 H53 N O3	(M+H)+		
562.41701	1	480.37		C37 H53 N O3	(M+H)+		

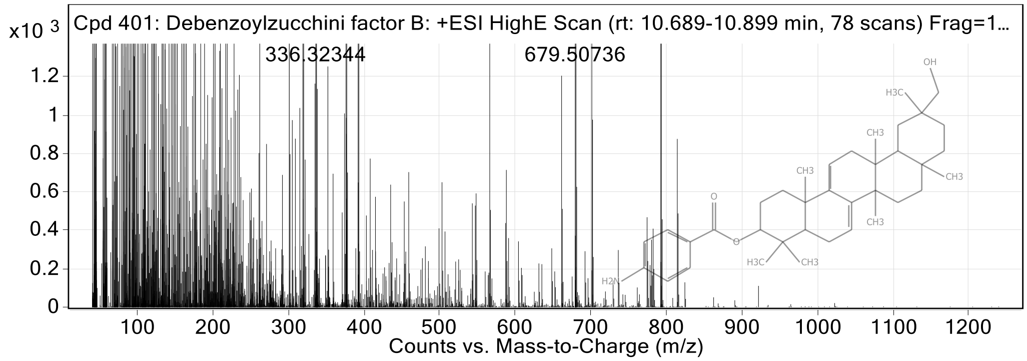

Peak List

m/z	z	Abund
45.03324		25531.53
55.05381		13762.69
69.06916		12954.98
81.06889		15655.17
89.0586		25201.33
93.06864		10047.36
95.08425	1	15851.24
133.08475		12364.96
336.32344	1	27855.21
376.31565	1	23165.86

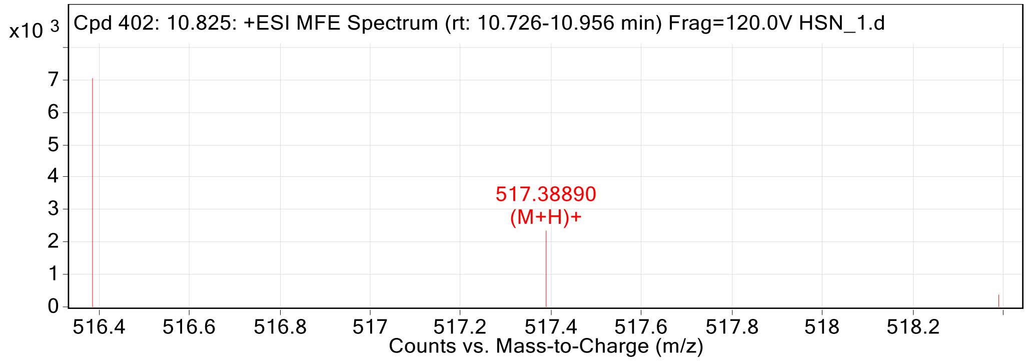

Peak List

m/z	z	Abund	Ion
516.38582	1	7047.32	(M+H)+
517.3889	1	2351.47	(M+H)+

518.3897	1	378.74	(M+H)+

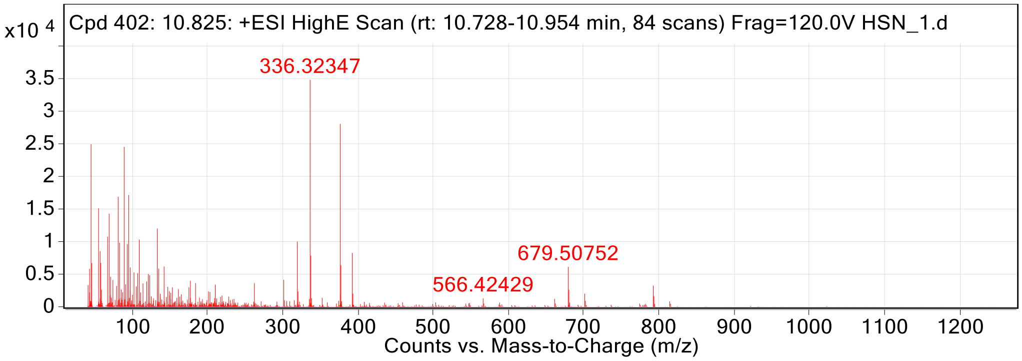

Peak List

m/z	z	Abund
45.03323		24883.7
55.05383		15082.38
67.05352		10731.76
69.06916		14244.3
81.06889		16835.7
89.05858		24480.42
95.08423	1	17112.16
133.08477		11974.93
336.32347	1	34760.47
376.31563	1	27998.59

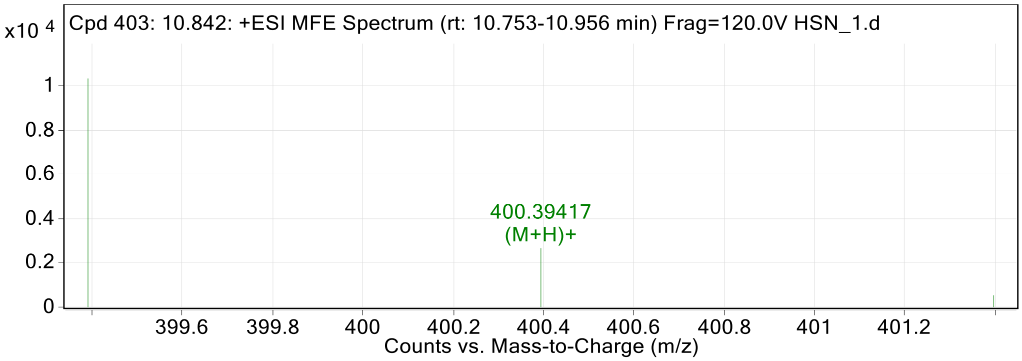

Peak List

m/z	z	Abund	Ion
399.39123	1	10359.38	(M+H)+
400.39417	1	2670.21	(M+H)+
401.39694	1	532.66	(M+H)+

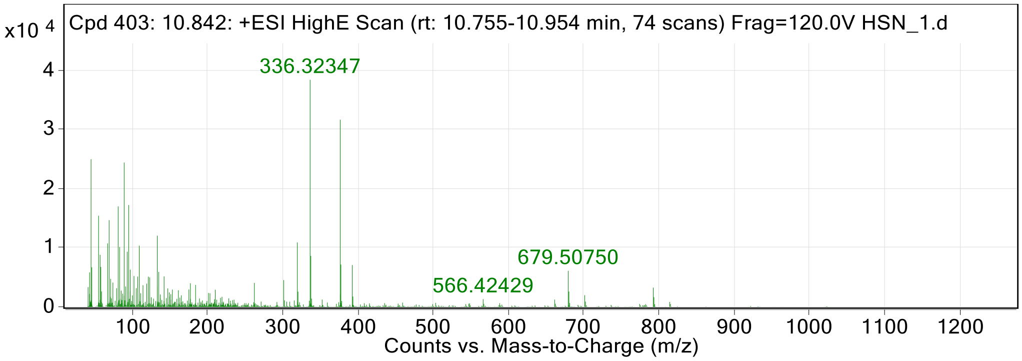

Peak List

m/z	z	Abund
45.03324		24937.41

55.05383		15392.73
69.06917		14644.78
81.06889		16962.83
89.05858		24382.58
95.08424	1	17201.85
133.08477		12023.98
319.29688	1	10874.69
336.32347	1	38369.89
376.31563	1	31617.97

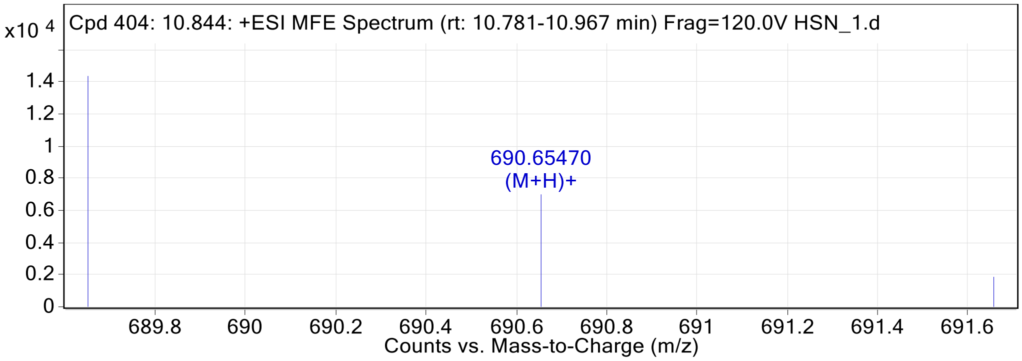

Peak List

m/z	z	Abund	Ion
689.65139	1	14357.98	(M+H)+
690.6547	1	6992.67	(M+H)+
691.65707	1	1863.88	(M+H)+

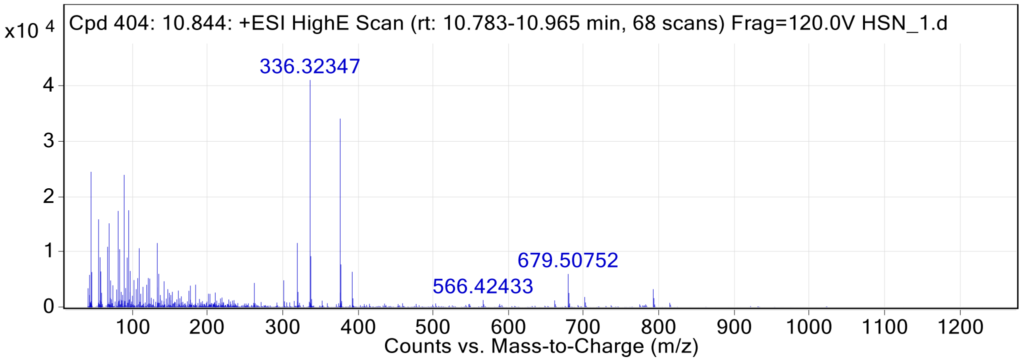

Peak List

m/z	z	Abund
45.03324		24503.08
55.05383		15887.75
69.06917		15162.93
81.06889		17409.46
89.05858		23945.99
95.08424	1	17528.12
133.08481		11595.17
319.29689	1	11603.74
336.32347	1	41134.77
376.31563	1	34141.53

Qualitative Analysis Report

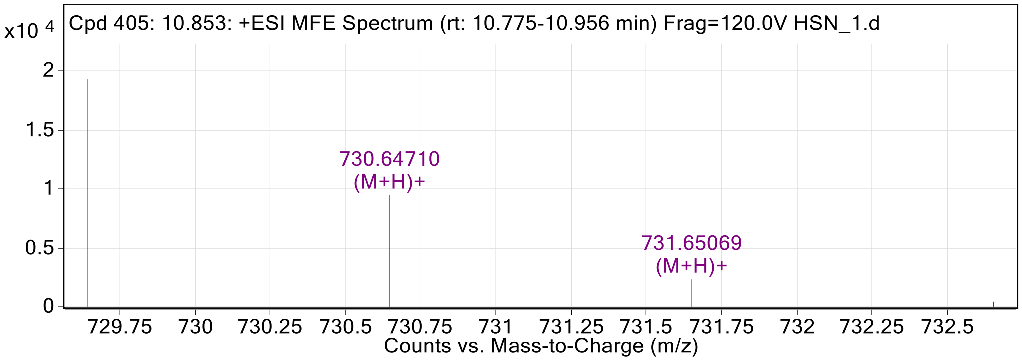

Peak List

m/z	z	Abund	Ion
729.64399	1	19326.58	(M+H)+
730.6471	1	9470.55	(M+H)+
731.65069	1	2323.18	(M+H)+
732.65336	1	431.5	(M+H)+

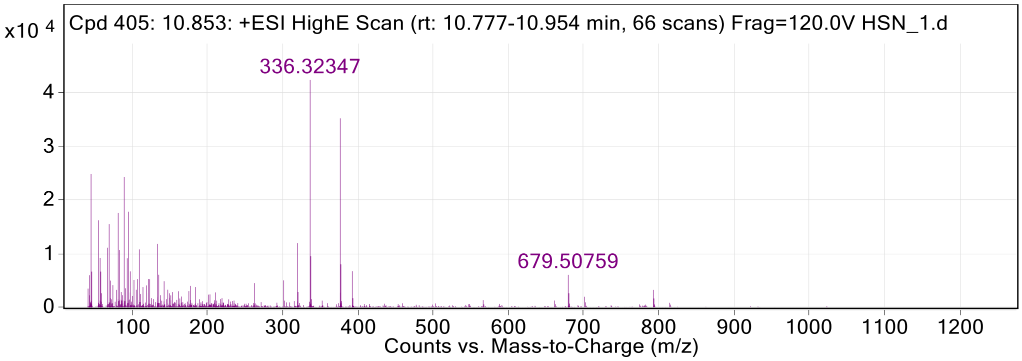

Peak List

m/z	z	Abund
45.03323		24829.92
55.05383		16153.36
69.06917		15445.04
81.0689		17573.23
89.05858		24243.52
95.08424	1	17780.21
133.08482		11789.77
319.29689	1	11924.98
336.32347	1	42332.41
376.31563	1	35183.76

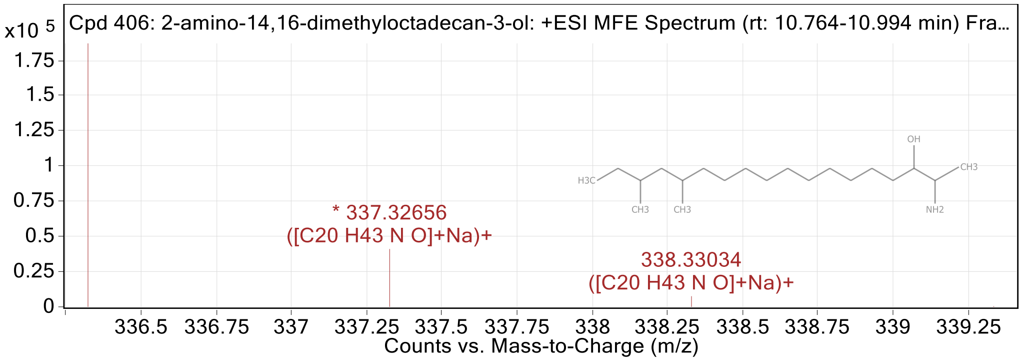

Peak List

m/z	z	Abund	Name	Formula	Ion	Score (DB)	Hits (DB)

336.32333	1	187097.5	2-amino-14,16-dimethyloctadecan-3-ol	C20 H43 N O	(M+Na)+	98.76	1
337.32656	1	41068.27		C20 H43 N O	(M+Na)+		
338.33034	1	7562.31		C20 H43 N O	(M+Na)+		
339.33562	1	480.53		C20 H43 N O	(M+Na)+		

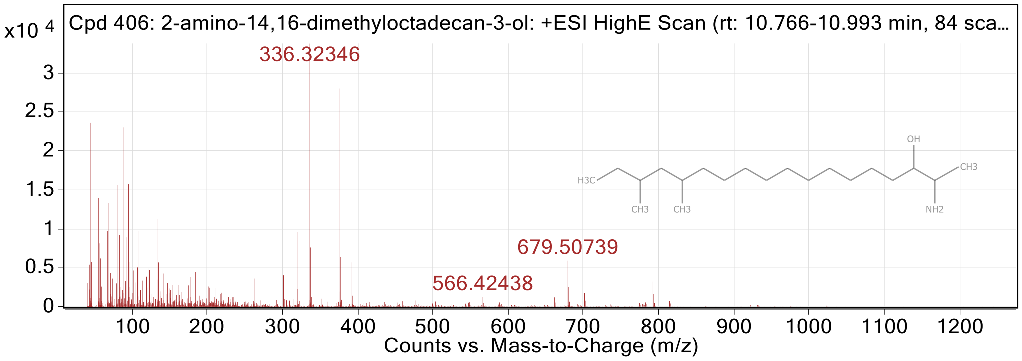

Peak List

m/z	z	Abund
45.03323		23569.18
55.05383		13902.59
69.06916		13306.65
81.06888		15562.36
89.05855		22972.28
95.08422	1	15677
109.09961		9690.95
133.08475		11236.64
336.32346	1	33739.63
376.31563	1	27954.11

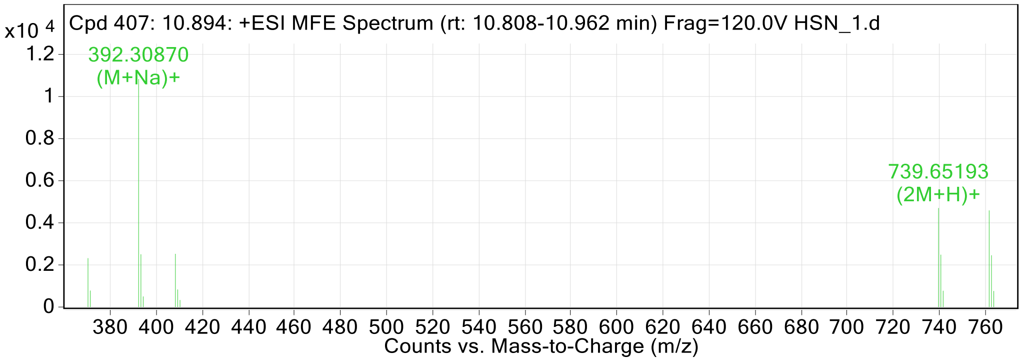

Peak List

m/z	z	Abund	Ion
370.32789	1	2321.31	(M+H)+
371.33215	1	781.85	(M+H)+
392.3087	1	10829.96	(M+Na)+
393.31272	1	2505.18	(M+Na)+
408.28483	1	2525.77	(M+K)+
409.28919	1	833.12	(M+K)+
739.65193	1	4702.76	(2M+H)+
740.65522	1	2486.57	(2M+H)+
761.63252	1	4589.74	(2M+Na)+
762.63537	1	2458.08	(2M+Na)+

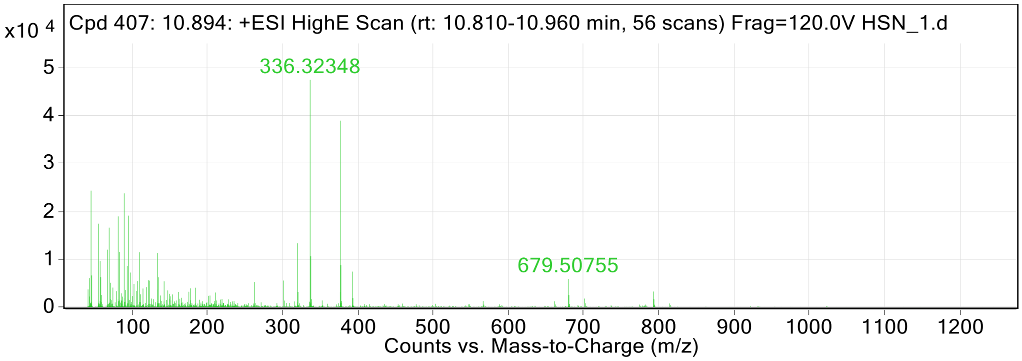

Peak List

m/z	z	Abund
45.03323		24332.65
55.05383		17417.23
67.05353		11975.76
69.06917		16583.8
81.06889		18934.33
89.05857		23756.09
95.08422	1	19100.83
319.29687	1	13299.2
336.32348	1	47519.54
376.31563	1	38989.77

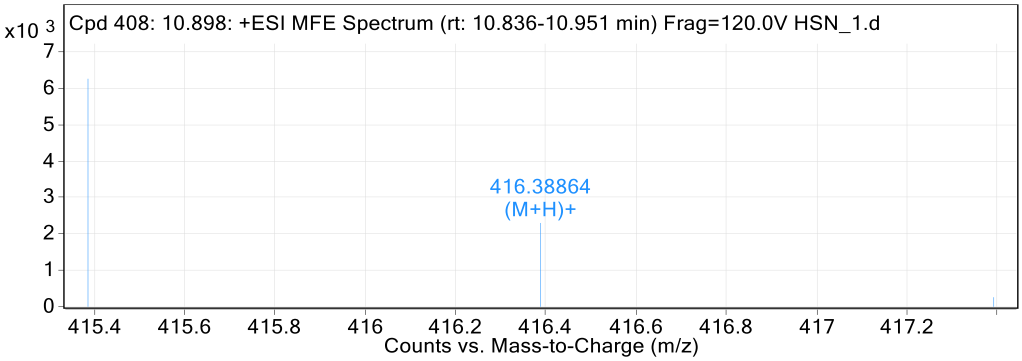

Peak List

m/z	z	Abund	Ion
415.38606	1	6259.12	(M+H)+
416.38864	1	2300.29	(M+H)+
417.39231	1	262.17	(M+H)+

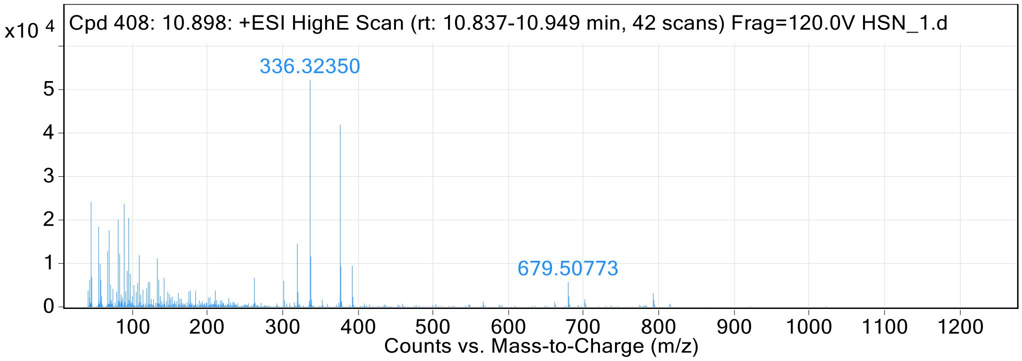

Peak List

m/z	z	Abund
45.03323		24142.51
55.05384		18369.88

67.05354		12846.26
69.06918		17566.1
81.06889		20074.12
89.05858		23673.78
95.08424	1	20418.08
319.29686	1	14527.69
336.3235	1	52148.13
376.31562	1	41850.7

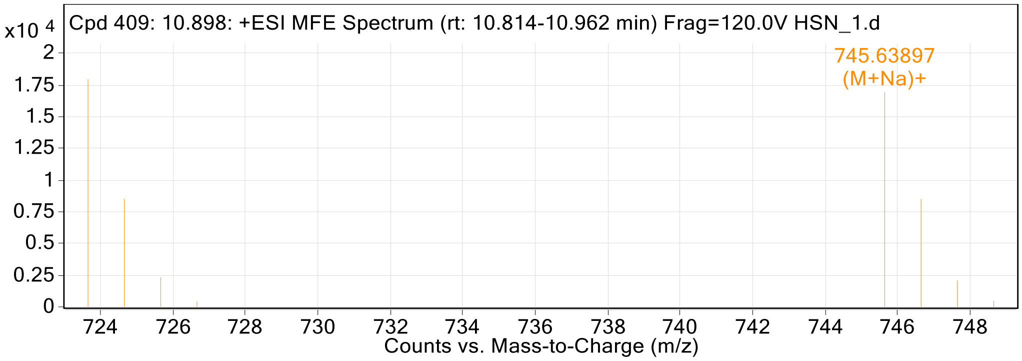

Peak List

m/z	z	Abund	Ion
723.65667	1	17979.82	(M+H)+
724.66006	1	8529.39	(M+H)+
725.6632	1	2350.17	(M+H)+
726.66306	1	437.6	(M+H)+
745.63897	1	16955.66	(M+Na)+
746.64219	1	8522.48	(M+Na)+
747.64465	1	2096.94	(M+Na)+
748.64867	1	485.71	(M+Na)+

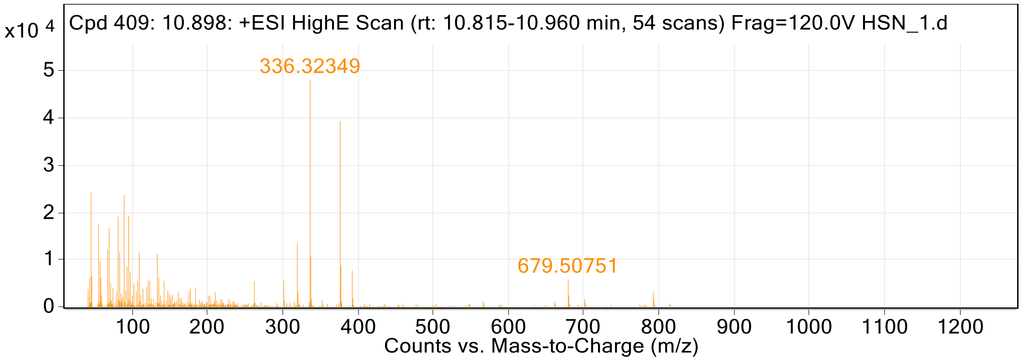

Peak List

m/z	z	Abund
45.03323		24205.47
55.05382		17519.04
67.05353		12097.28
69.06917		16716.7
81.06889		19114.6
89.05857		23660.66
95.08422	1	19276.63
319.29686	1	13457.54
336.32349	1	48143.98
376.31562	1	39311.75

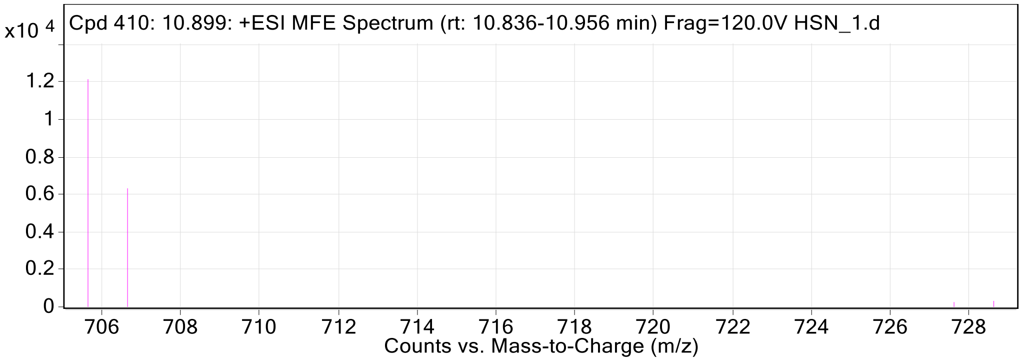

Peak List

m/z	z	Abund	Ion
705.64615	1	12152.57	(M+H)+
706.65008	1	6326.98	(M+H)+
727.62461	1	256.25	(M+Na)+
728.63315	1	319.02	(M+Na)+

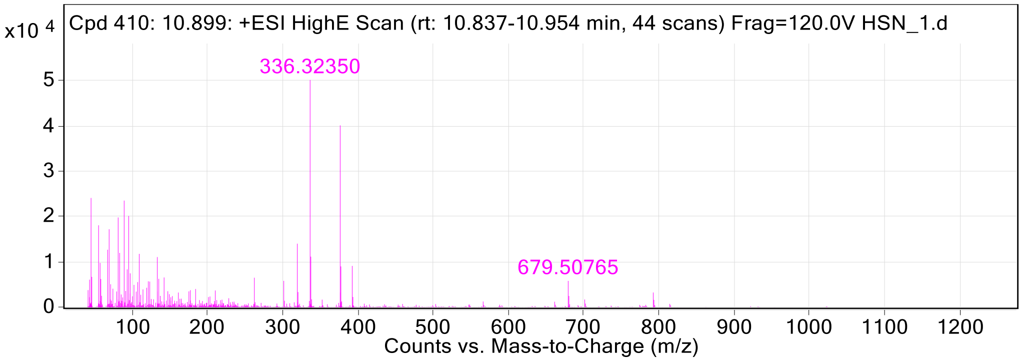

Peak List

m/z	z	Abund
45.03323		24085.38
55.05384		18049.55
67.05353		12654.74
69.06918		17178.83
81.0689		19779.94
89.05857		23503.3
95.08423	1	20111.07
319.29685	1	13988.36
336.3235	1	50140.85
376.31562	1	40114.48

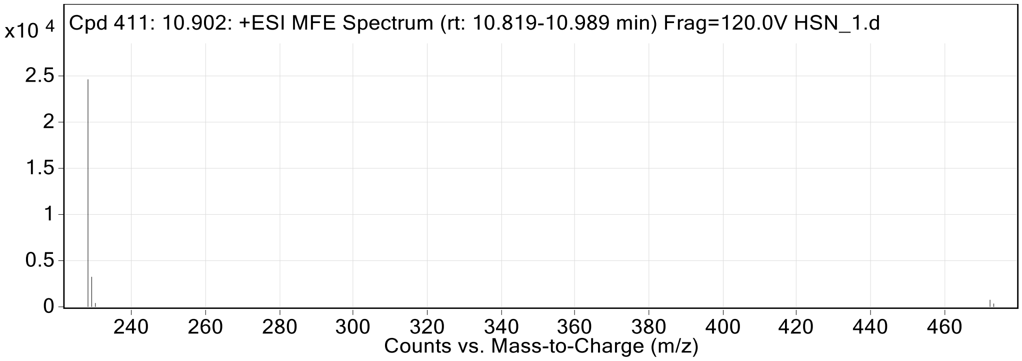

Peak List

m/z	z	Abund	Ion
228.19359	1	24588.41	(M+H)+

229.19717	1	3246.47	(M+H)+
230.19841	1	411.59	(M+H)+
472.40817	1	762.59	(2M+NH4)+
473.4246	1	353.09	(2M+NH4)+

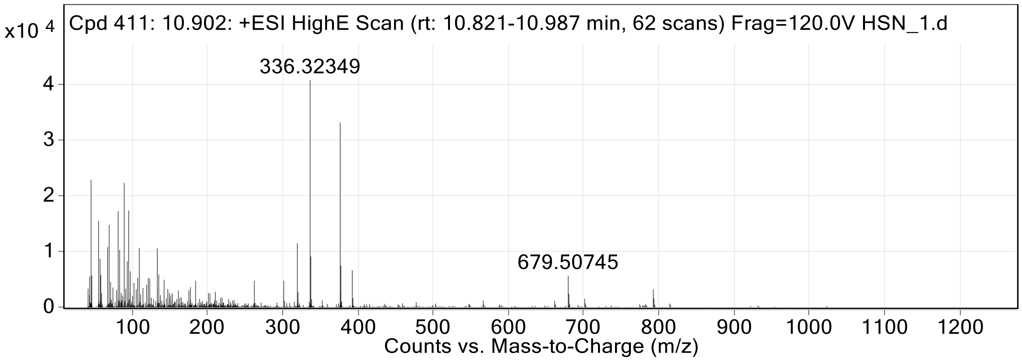

Peak List

m/z	z	Abund
45.03323		22907.61
55.05383		15514.66
67.05352		10802.3
69.06916		14798.65
81.06888		17246.11
89.05855		22324.72
95.08422	1	17360.37
319.29688	1	11465.28
336.32349	1	40826.79
376.31562	1	33212.17

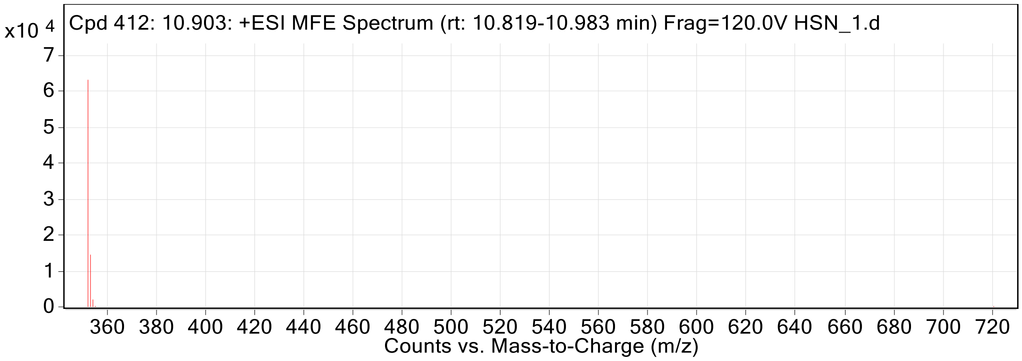

Peak List

m/z	z	Abund	Ion
352.31827	1	63149.4	(M+H)+
353.32153	1	14523.43	(M+H)+
354.32642	1	2057.62	(M+H)+
355.32985	1	244.16	(M+H)+
720.65044	1	208.01	(2M+NH4)+

Qualitative Analysis Report

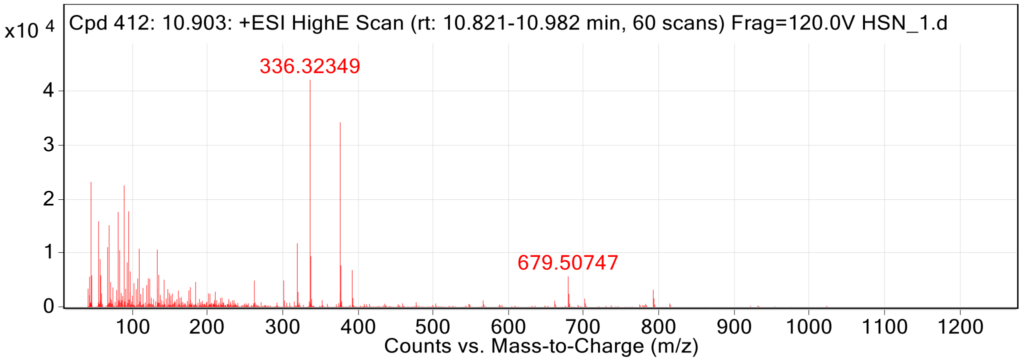

Peak List

m/z	z	Abund
45.03323		23221.7
55.05384		15888.62
67.05353		11080.53
69.06917		15167.14
81.06888		17626.17
89.05855		22566.21
95.08422	1	17750.29
319.29688	1	11829.73
336.32349	1	42169.1
376.31562	1	34297.41

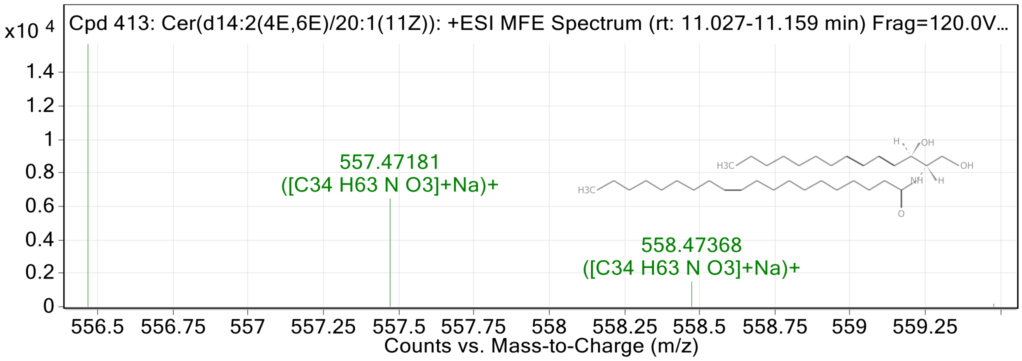

Peak List

m/z	z	Abund	Name	Formula	Ion	Score (DB)	Hits (DB)
556.4682	1	15711.87	Cer(d14:2(4E,6E)/20:1(11Z))	C34 H63 N O3	(M+Na)+	92.35	1
557.47181	1	6471.9		C34 H63 N O3	(M+Na)+		
558.47368	1	1508.74		C34 H63 N O3	(M+Na)+		
559.47702	1	186.97		C34 H63 N O3	(M+Na)+		

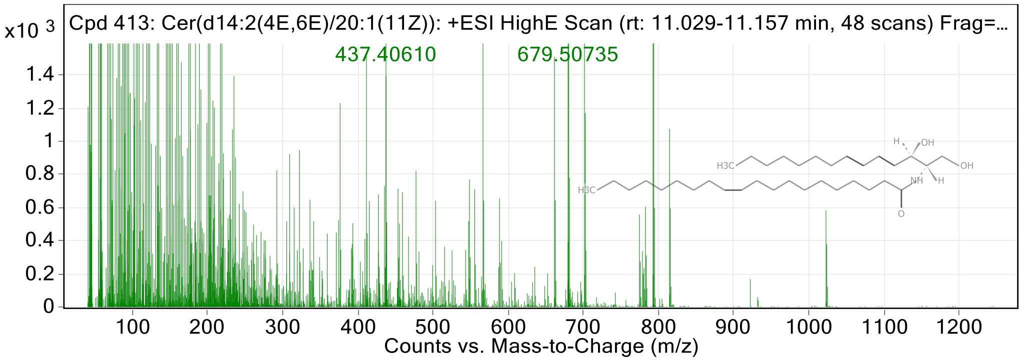

Peak List

m/z	z	Abund

45.03321		24080.21
58.06461		7998.38
81.06883		4918.54
89.05856	1	23382.31
93.06865		6097.56
95.08421		5085.42
102.12627	1	19515.21
109.09953		4597.79
133.08439		11800
679.50735	1	7538.62

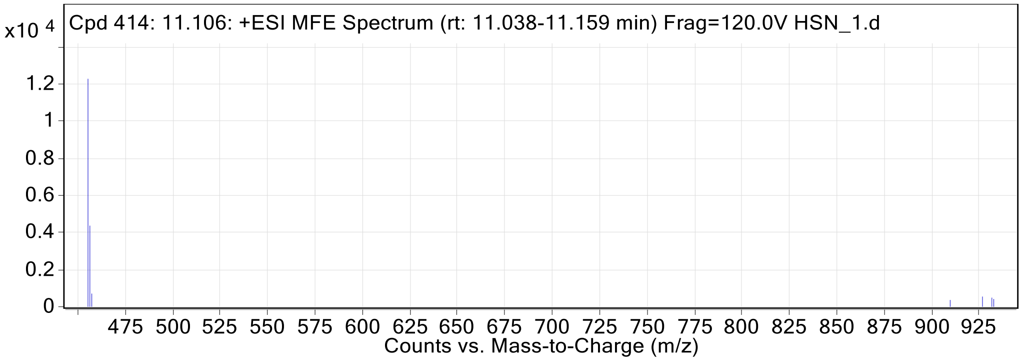

Peak List

m/z	z	Abund	Ion
455.34828	1	12276.44	(M+H)+
456.35174	1	4369.3	(M+H)+
457.35582	1	709.92	(M+H)+
909.69104	1	365.25	(2M+H)+
926.71569	1	549	(2M+NH4)+
931.66904	1	489.99	(2M+Na)+
932.66853	1	416.92	(2M+Na)+

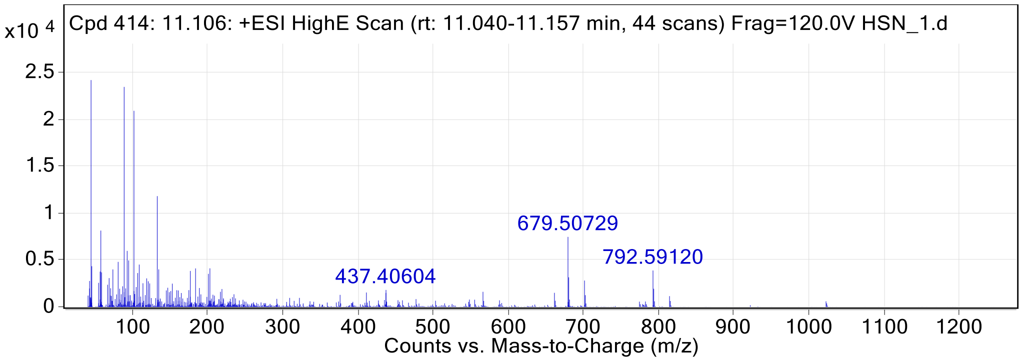

Peak List

m/z	z	Abund
45.03321		24195.08
58.0646		8134.18
81.06883		4793.1
89.05856	1	23464.99
93.06864		5966.69

m/z	z	Abund
95.08422		4948.96
102.12627	1	20912.6
109.09955		4505.24
133.08437		11808.56
679.50729	1	7460.29

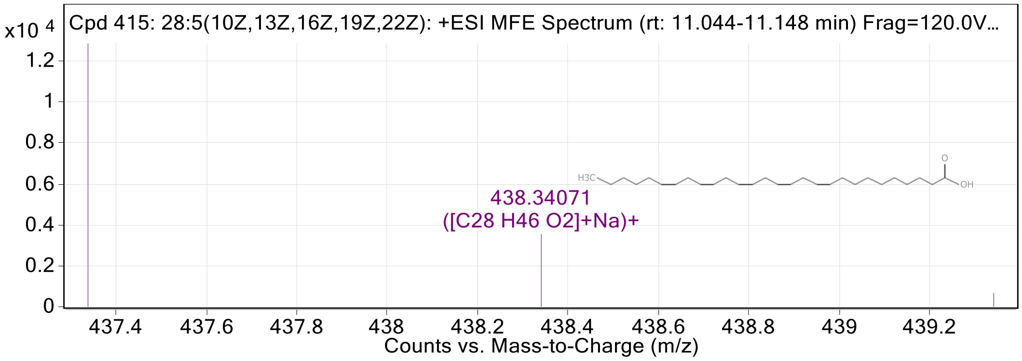

Peak List

m/z	z	Abund	Name	Formula	Ion	Score (DB)	Hits (DB)
437.33768	1	12870.27	28:5(10Z,13Z,16Z,19Z,22Z)	C28 H46 O2	(M+Na)+	92.28	10
438.34071	1	3545.01		C28 H46 O2	(M+Na)+		
439.34231	1	676.38		C28 H46 O2	(M+Na)+		

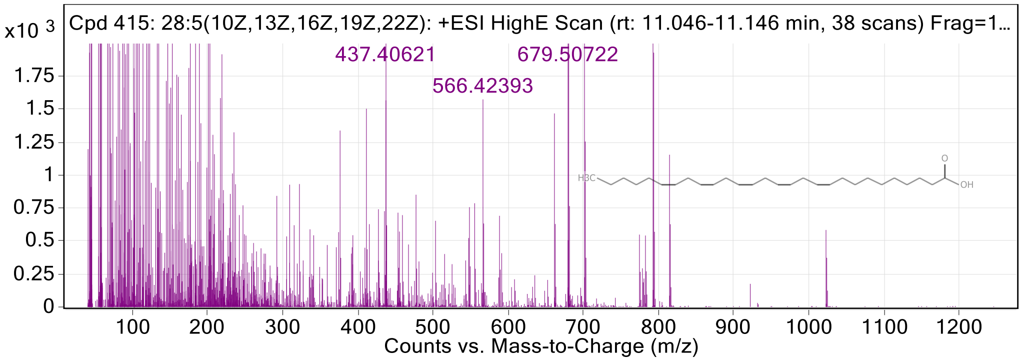

Peak List

m/z	z	Abund
45.03322		24080.93
58.06462		8170.35
81.06883		4793.75
89.05856	1	23519.37
93.06863		5874.62
95.08424		4936.94
102.12625	1	22442.3
109.09954		4562.78
133.08435		12093.57
679.50722	1	7449.94

Qualitative Analysis Report

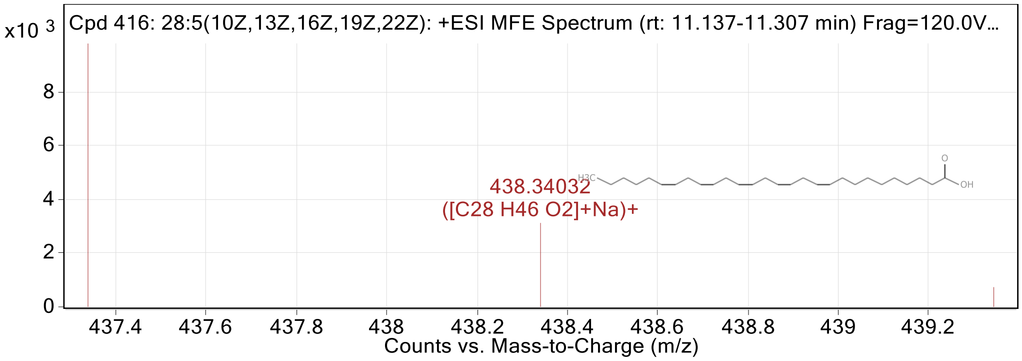

Peak List

m/z	z	Abund	Name	Formula	Ion	Score (DB)	Hits (DB)
437.33807	1	9799.92	28:5(10Z,13Z,16Z,19Z,22Z)	C28 H46 O2	(M+Na)+	93.49	10
438.34032	1	3122.63		C28 H46 O2	(M+Na)+		
439.34415	1	734.39		C28 H46 O2	(M+Na)+		

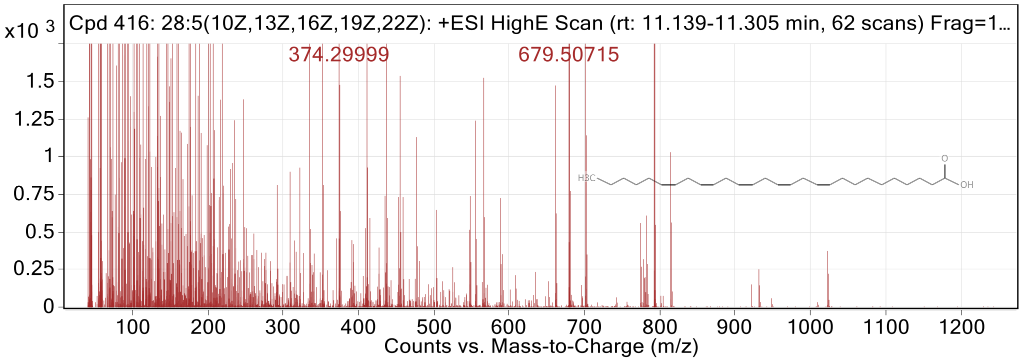

Peak List

m/z	z	Abund
45.03323		23424.67
58.06465		7728.17
81.06884		7619.53
89.05854	1	22579.45
93.06862		6344.22
95.08421		7996.53
102.12629	1	17301.21
133.08441		10761.31
374.29999	1	5898.19
679.50715	1	7205.71

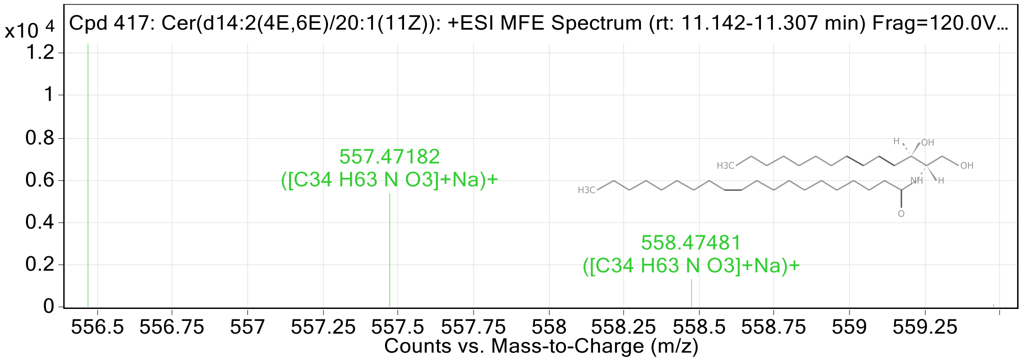

Peak List

m/z	z	Abund	Name	Formula	Ion	Score (DB)	Hits (DB)
556.46879	1	12471.7	Cer(d14:2(4E,6E)/20:1(11Z))	C34 H63 N O3	(M+Na)+	93.26	1

557.47182	1	5388.23		C34 H63 N O3	(M+Na)+		
558.47481	1	1316.45		C34 H63 N O3	(M+Na)+		
559.47914	1	133.56		C34 H63 N O3	(M+Na)+		

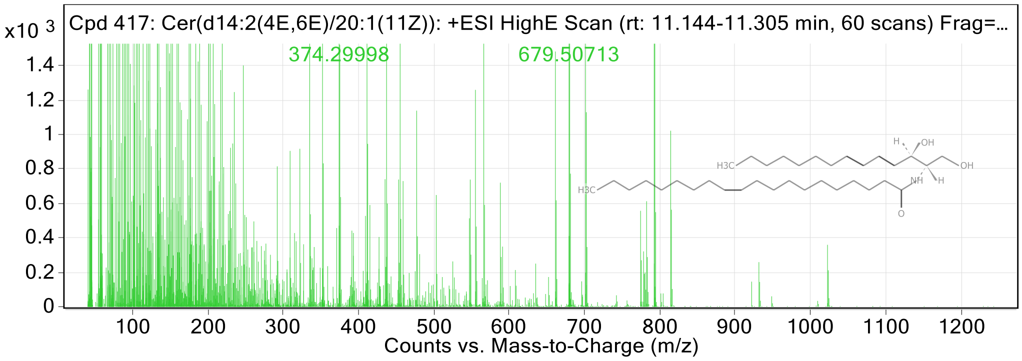

Peak List

m/z	z	Abund
45.03323		23420.64
58.06464		7719.23
81.06884		7729.71
89.05854	1	22553.95
93.06861		6350.87
95.08421		8110.26
102.12629	1	17252.41
133.08442		10650.53
374.29998	1	6073.53
679.50713	1	7190.62

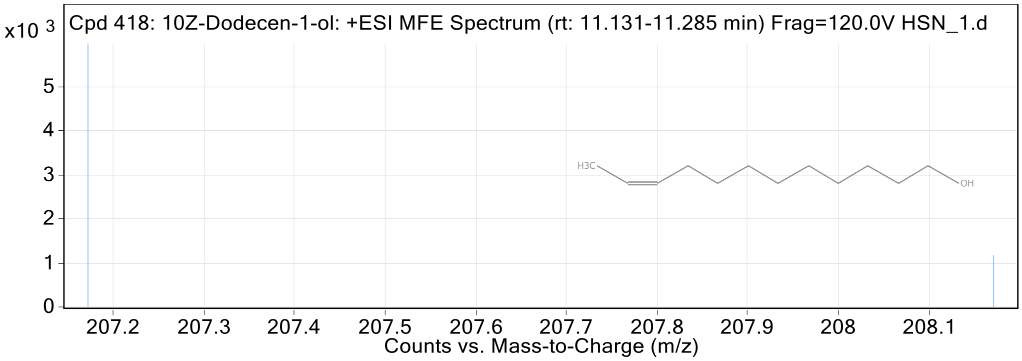

Peak List

m/z	z	Abund	Name	Formula	Ion	Score (DB)	Hits (DB)
207.17196	1	5971.81	10Z-Dodecen-1-ol	C12 H24 O	(M+Na)+	67.4	10
208.17166	1	1176.15		C12 H24 O	(M+Na)+		

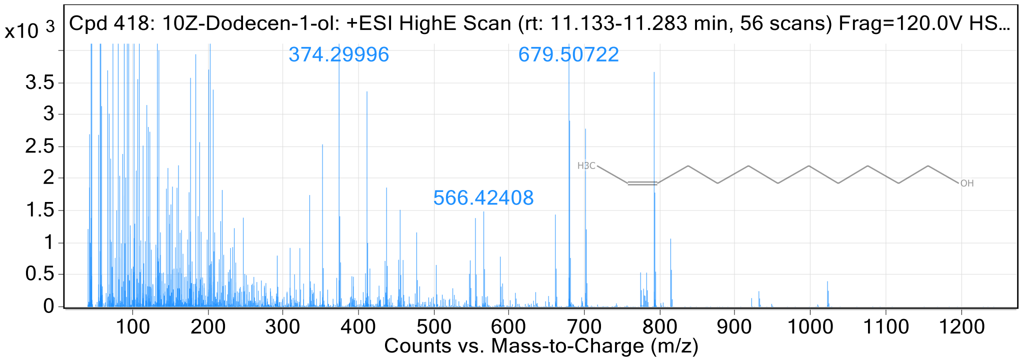

Peak List

m/z	z	Abund

45.03323		23501.65
58.06465		8024.6
81.06883		6798.22
89.05854	1	22737.58
93.06862		6273.14
95.08422		7237.09
102.12629	1	19006.23
133.08438		10971.71
374.29996	1	5648.11
679.50722	1	6996.41

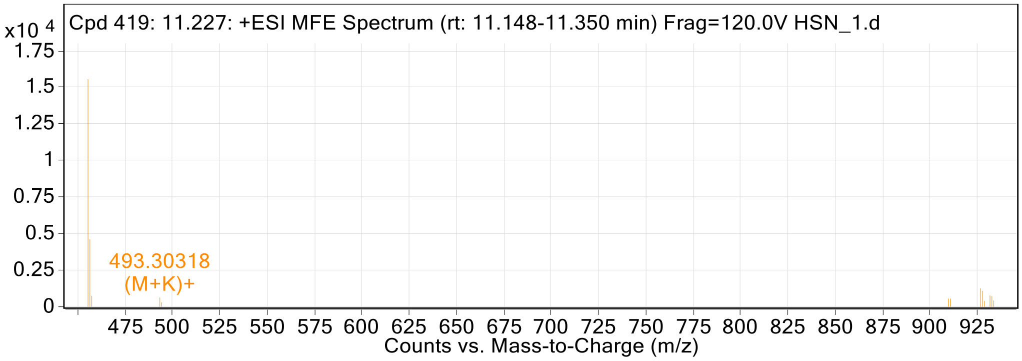

Peak List

m/z	z	Abund	Ion
455.34875	1	15563.66	(M+H)+
456.35199	1	4611.91	(M+H)+
457.35568	1	744.7	(M+H)+
493.30318	1	632.66	(M+K)+
909.69105	1	556.43	(2M+H)+
910.69457	1	551.32	(2M+H)+
926.71838	1	1258.79	(2M+NH4)+
927.72179	1	1084.2	(2M+NH4)+
931.67236	1	769.81	(2M+Na)+
932.68054	1	731.16	(2M+Na)+

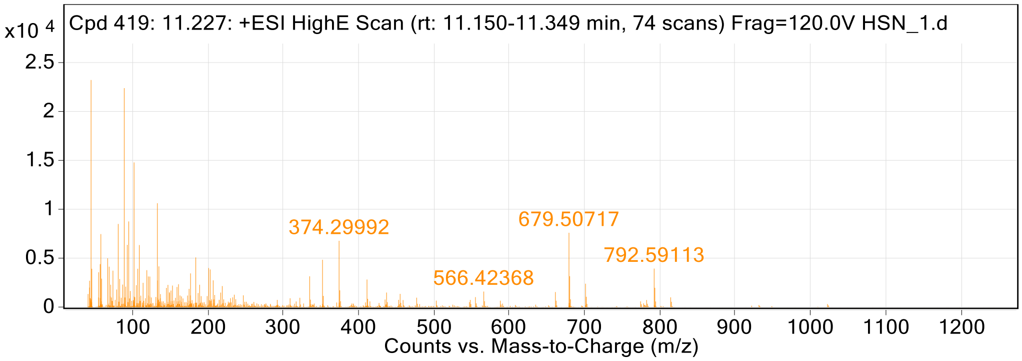

Peak List

m/z	z	Abund
45.03323		23183.99
58.06466		7449.51
81.06882		8483.69
89.05851	1	22355.89
93.06862		6351.85
95.08418		8740.7
102.12629	1	14768.01
133.0844		10602.8
374.29992	1	6762.3

679.50717	1	7583.66

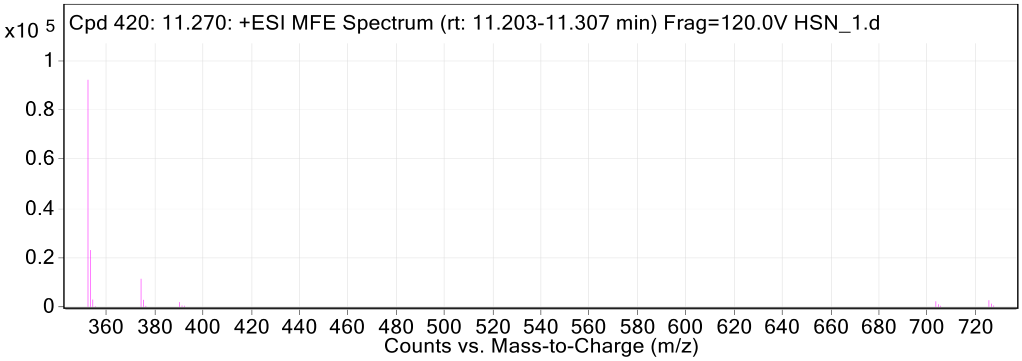

Peak List

m/z	z	Abund	Ion
352.31859	1	92373.26	(M+H)+
353.32135	1	23104.97	(M+H)+
354.32486	1	2934.49	(M+H)+
374.29981	1	11423.31	(M+Na)+
375.30338	1	2853.97	(M+Na)+
390.27265	1	1910.79	(M+K)+
703.63048	1	2214.94	(2M+H)+
704.63259	1	1017.84	(2M+H)+
725.61312	1	2655.02	(2M+Na)+
726.61383	1	1276.09	(2M+Na)+

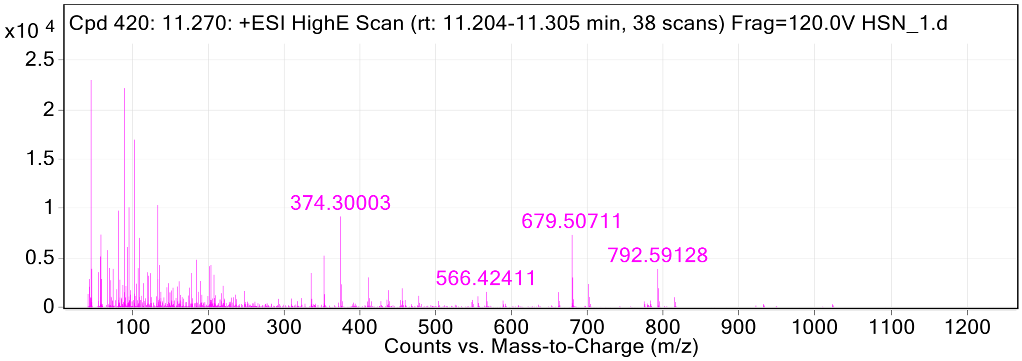

Peak List

m/z	z	Abund
45.03322		23000.86
58.06471		7343.74
81.06883		9775.16
89.05855	1	22172.79
95.0842		10097.06
102.1263	1	16970.28
109.09954		7023.32
133.08448		10333.32
374.30003	1	9177.86
679.50711	1	7324.95

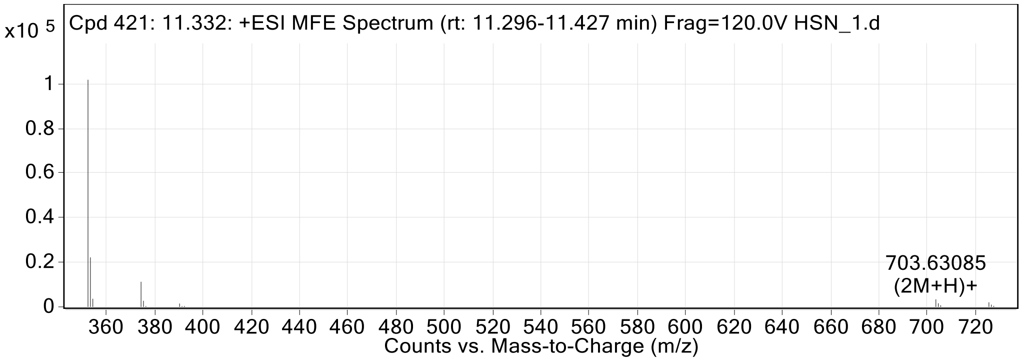

Peak List

m/z	z	Abund	Ion
352.31845	1	101780.27	(M+H)+
353.32135	1	22180.06	(M+H)+
354.32429	1	3642.25	(M+H)+
374.30002	1	11250.7	(M+Na)+
375.30376	1	2724.33	(M+Na)+
390.27158	1	1494.03	(M+K)+
703.63085	1	3374.52	(2M+H)+
704.63338	1	1609.74	(2M+H)+
725.6107	1	2047.66	(2M+Na)+
726.61391	1	1006.83	(2M+Na)+

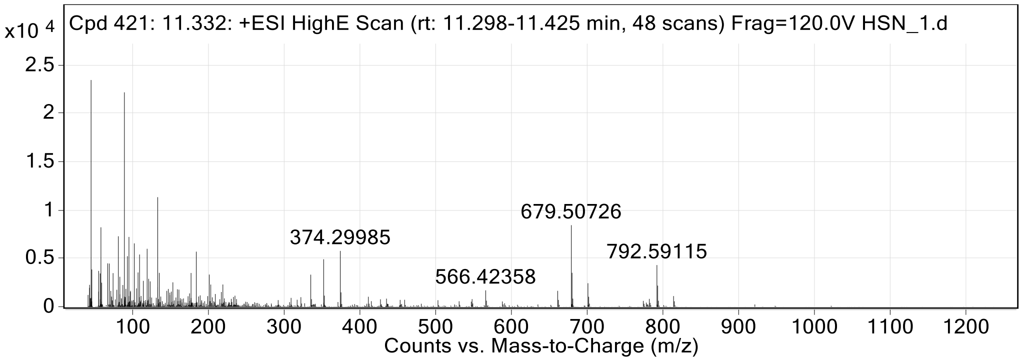

Peak List

m/z	z	Abund
45.03321		23456.11
58.06466		8218.83
81.06883		7290.15
89.05848	1	22182.83
95.08412		7221.85
102.12624		6554.79
119.08374		5994.81
133.08423		11335.43
374.29985	1	5776.18
679.50726	1	8438.63

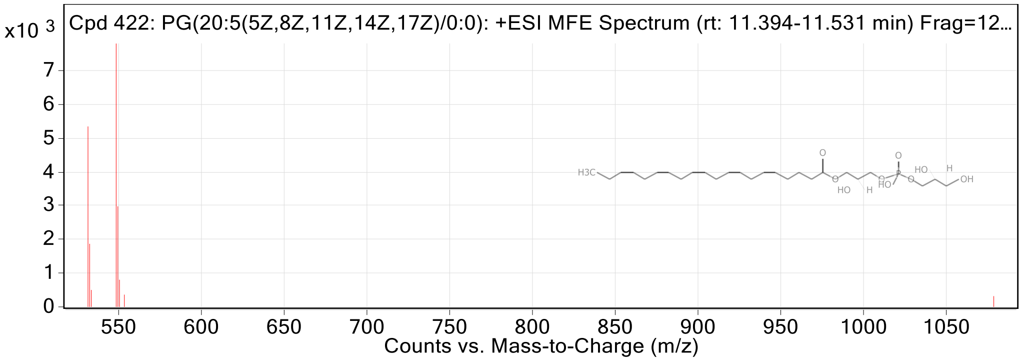

Peak List

m/z	z	Abund	Name	Formula	Ion	Score (DB)	Hits (DB)
531.27074	1	5349.44			(M+H)+		
532.27388	1	1870.38			(M+H)+		
533.28027	1	502.21			(M+H)+		
548.2971	1	7810.84	PG(20:5(5Z,8Z,11Z,14Z,17Z)/0:0)	C26 H43 O9 P	(M+NH4)+	87.56	1
549.30067	1	2981.18		C26 H43 O9 P	(M+NH4)+		
550.30334	1	804.72		C26 H43 O9 P	(M+NH4)+		
553.25148	1	363.06			(M+Na)+		
1078.56306	1	318.88			(2M+NH4)+		

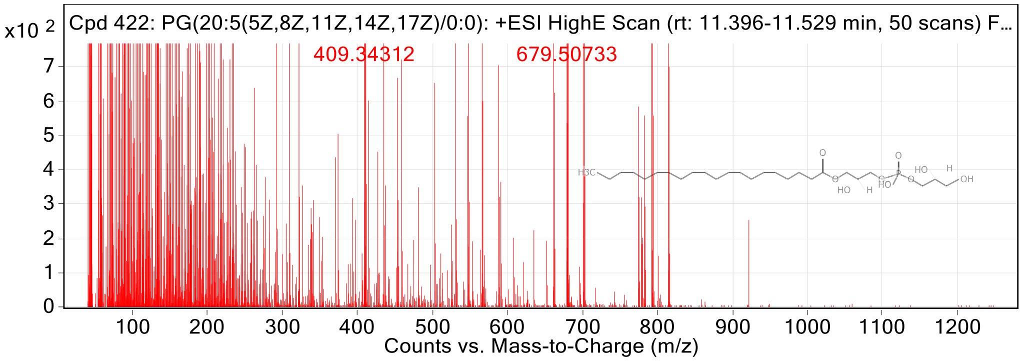

Peak List

m/z	z	Abund
45.03323		23095.15
58.06465		9399.77
89.05853	1	22068.65
93.06869		4233.45
102.12628		6560.99
119.08371	1	9113.19
133.08419	1	11117.22
184.07106		4251.2
189.16147	1	4465.79
679.50733	1	7740.84

Qualitative Analysis Report

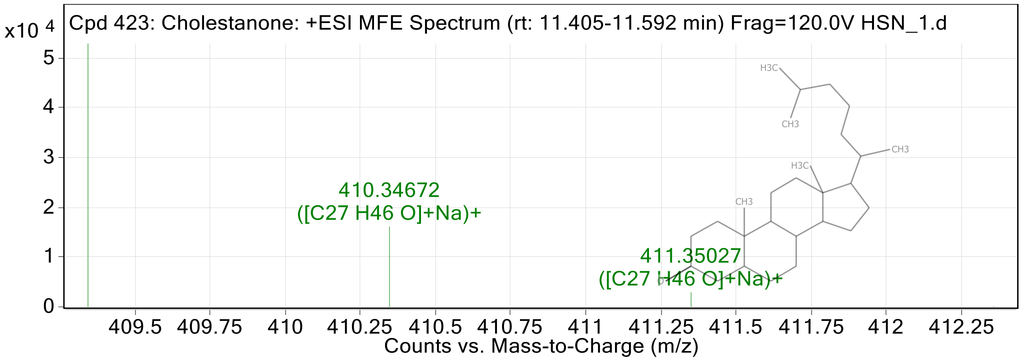

Peak List

m/z	z	Abund	Name	Formula	Ion	Score (DB)	Hits (DB)
409.34352	1	52877.48	Cholestanone	C27 H46 O	(M+Na)+	98.63	10
410.34672	1	16125.37		C27 H46 O	(M+Na)+		
411.35027	1	2939.4		C27 H46 O	(M+Na)+		
412.35749	1	128.73		C27 H46 O	(M+Na)+		

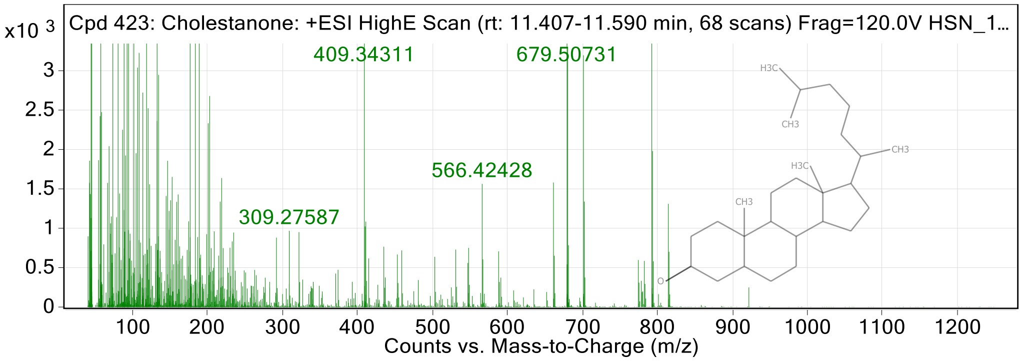

Peak List

m/z	z	Abund
45.03324		22800.61
46.06489		4306.89
58.06465		9685.63
89.05853	1	21834.18
93.06869		4485.48
102.12626		6514.21
119.08373	1	7130.46
133.08424	1	11157.27
184.07101		4264.26
679.50731	1	7830.78

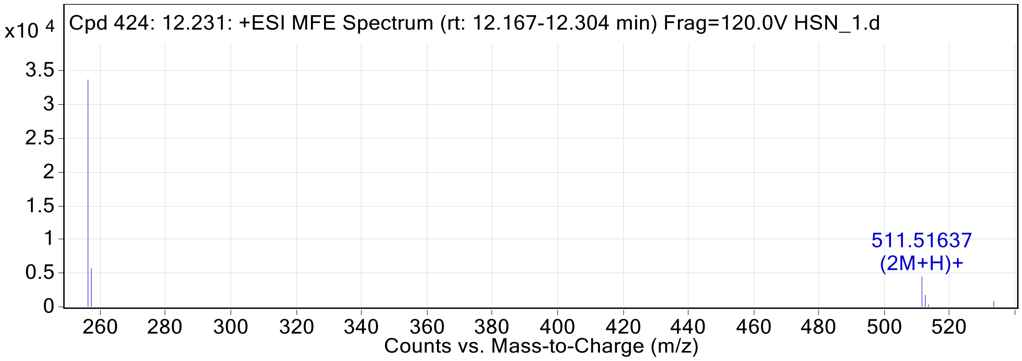

Peak List

m/z	z	Abund	Ion
256.26079	1	33727.7	(M+H)+

257.26471	1	5732.98	(M+H)+
511.51637	1	4460.59	(2M+H)+
512.51961	1	1761.23	(2M+H)+
513.52378	1	354.25	(2M+H)+
533.49815	1	841.58	(2M+Na)+

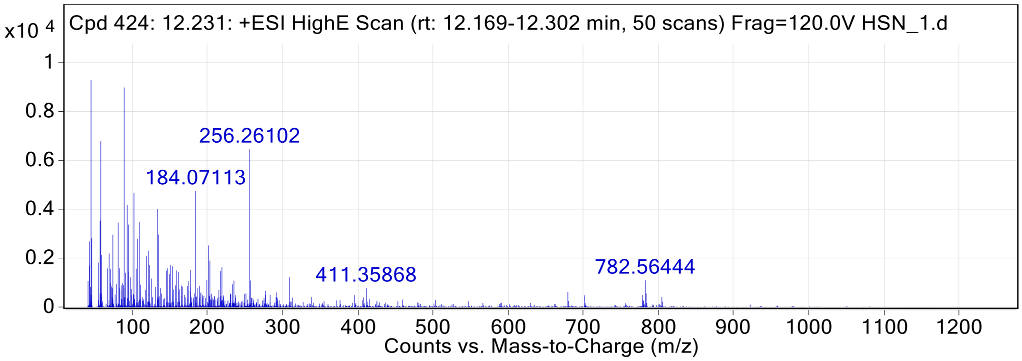

Peak List

m/z	z	Abund
45.0332		9267.39
57.06936		3528.18
58.06469		6782.24
89.05849		8962.95
93.06872		4159.02
102.12628		4666.38
109.09949		3469.29
133.08451		3999.55
184.07113		4730.68
256.26102	1	6437.62

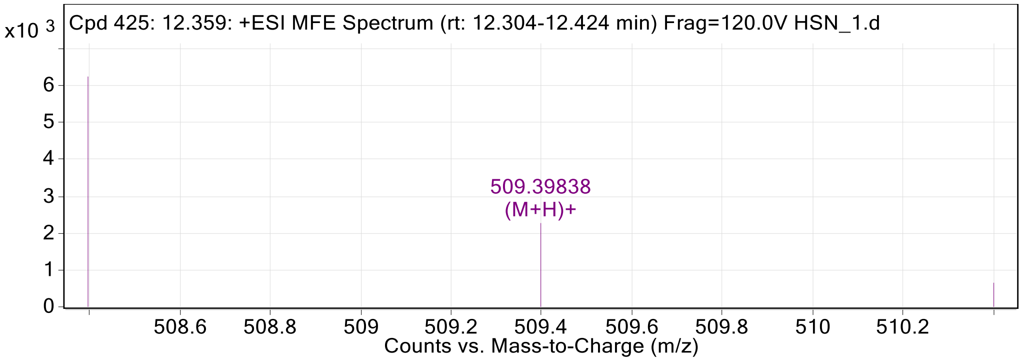

Peak List

m/z	z	Abund	Ion
508.39637	1	6244.08	(M+H)+
509.39838	1	2271.24	(M+H)+
510.40076	1	652.82	(M+H)+

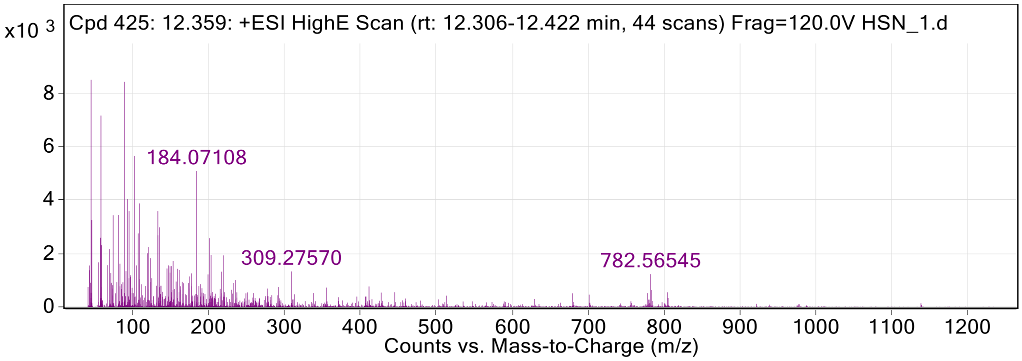

Peak List

m/z	Abund
45.03318	8510.91
58.06463	7173.37
81.06885	3448.3
89.05849	8434.64
93.06868	4044.05
95.08423	3589.43
102.1263	5654.06
109.09953	3876.79
133.08433	3585.54
184.07108	5091.86

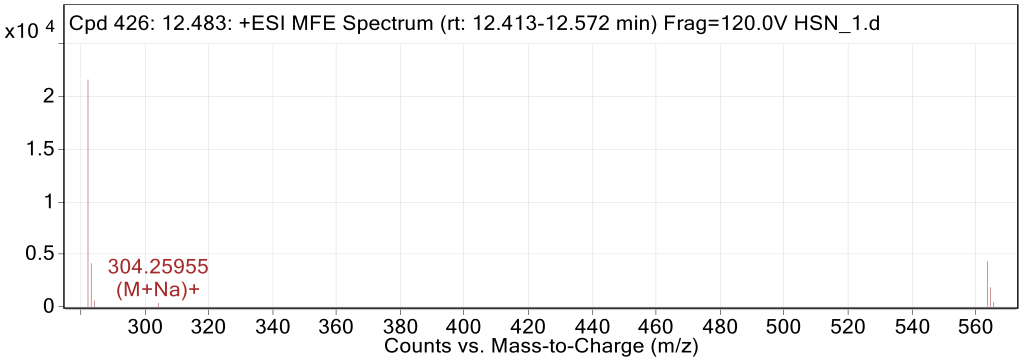

Peak List

m/z	z	Abund	Ion
282.27641	1	21629.89	(M+H)+
283.28011	1	4148.11	(M+H)+
284.28497	1	595.46	(M+H)+
304.25955	1	366.9	(M+Na)+
563.54777	1	4345.79	(2M+H)+
564.54971	1	1854.08	(2M+H)+
565.5557	1	444.62	(2M+H)+

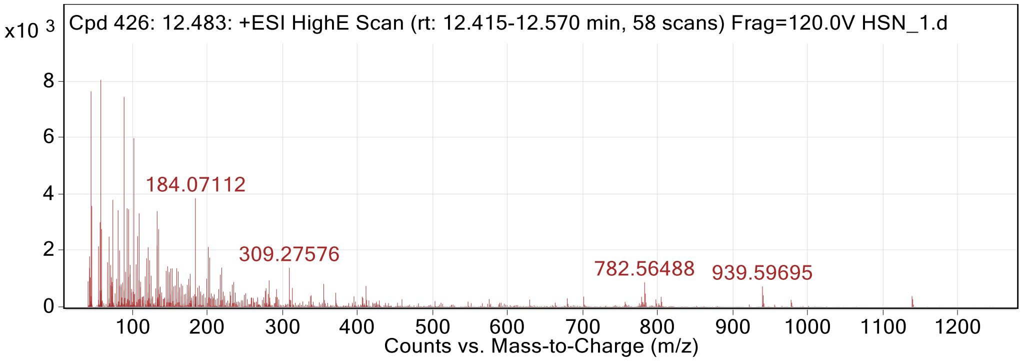

Peak List

m/z	Abund
45.03317	7644.69
46.06481	3579.37
58.06462	8052.47
74.09554	3795.85
81.06887	3425.63
89.05854	7447.66
93.06863	3495.69
95.08423	3461.76
102.12631	5986.77
184.07112	3853.72

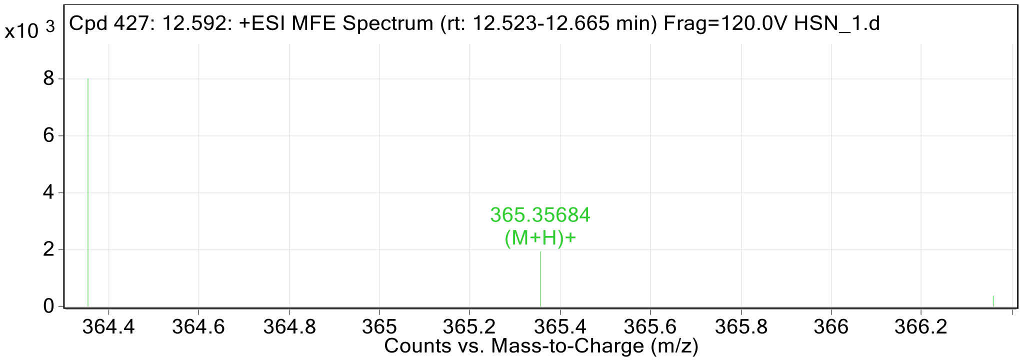

Peak List

m/z	z	Abund	Ion
364.35468	1	8001.92	(M+H)+
365.35684	1	1945.52	(M+H)+
366.36006	1	393.64	(M+H)+

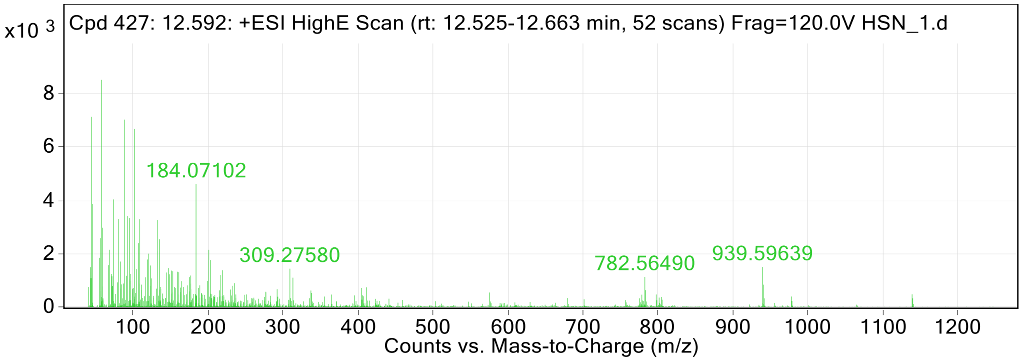

Peak List

m/z	Abund
45.03316	7139.52
46.06484	3869.97

58.0646	8525.01
74.09557	4034.8
81.06888	3295
89.05852	7033.9
93.06859	3410.7
95.08415	3344.6
102.12625	6679.35
184.07102	4609.66

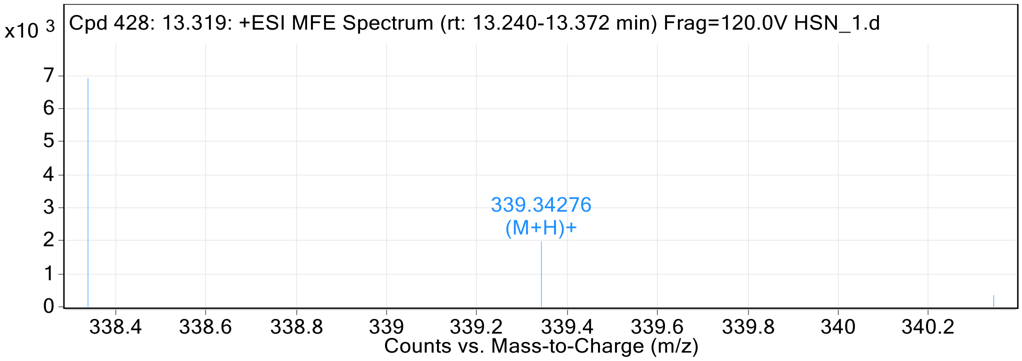

Peak List

m/z	z	Abund	Ion
338.33876	1	6928.52	(M+H)+
339.34276	1	1988.39	(M+H)+
340.34436	1	355.36	(M+H)+

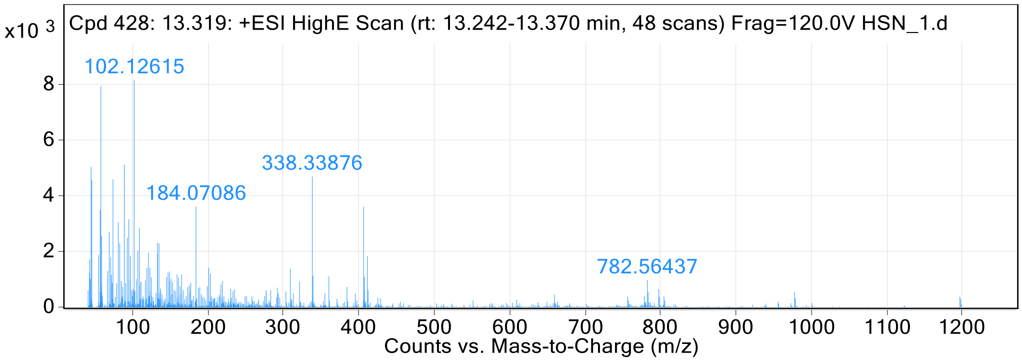

Peak List

m/z	z	Abund
45.03312		5039.19
46.06478		4563.94
57.06928		3474.49
58.06458		7923.85
74.09561		4586.27
89.05846		5103.88
102.12615	1	8141.81
184.07086		3604.32
338.33876	1	4683.94
406.32572	1	3597.45

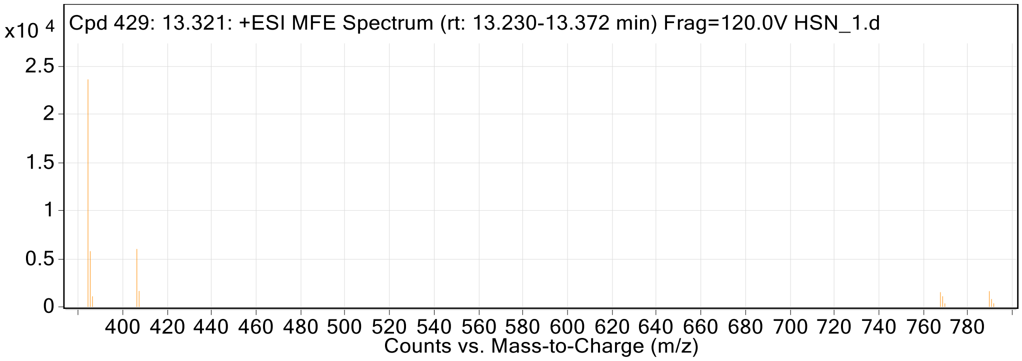

Peak List

m/z	z	Abund	Ion
384.34413	1	23610.07	(M+H)+
385.34732	1	5781.28	(M+H)+
386.35044	1	1098.35	(M+H)+
406.32551	1	6008.73	(M+Na)+
407.32922	1	1650.7	(M+Na)+
767.68108	1	1526.14	(2M+H)+
768.68357	1	1102.37	(2M+H)+

m/z	z	Abund	Ion
789.66323	1	1631.7	(2M+Na)+
790.66648	1	814.15	(2M+Na)+
791.66899	1	368.68	(2M+Na)+

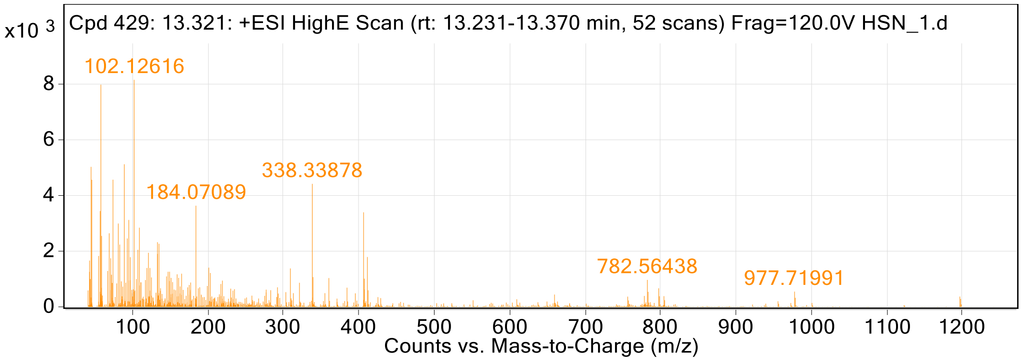

Peak List

m/z	z	Abund
45.03312		5017.45
46.0648		4554.43
57.06928		3433.77
58.06459		7961.56
74.09559		4557.17
89.05847		5109.26
102.12616	1	8135.64
184.07089		3625.42
338.33878	1	4407.87
406.32569	1	3387.12

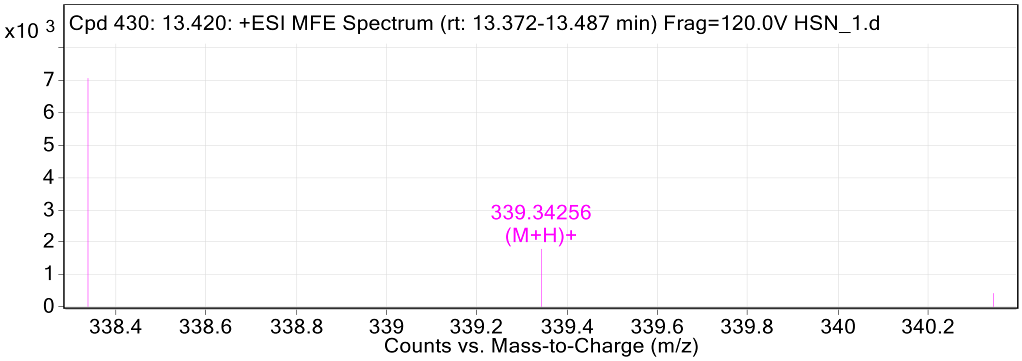

Peak List

m/z	z	Abund	Ion
338.33814	1	7076.43	(M+H)+
339.34256	1	1791.42	(M+H)+
340.34557	1	419.54	(M+H)+

Qualitative Analysis Report

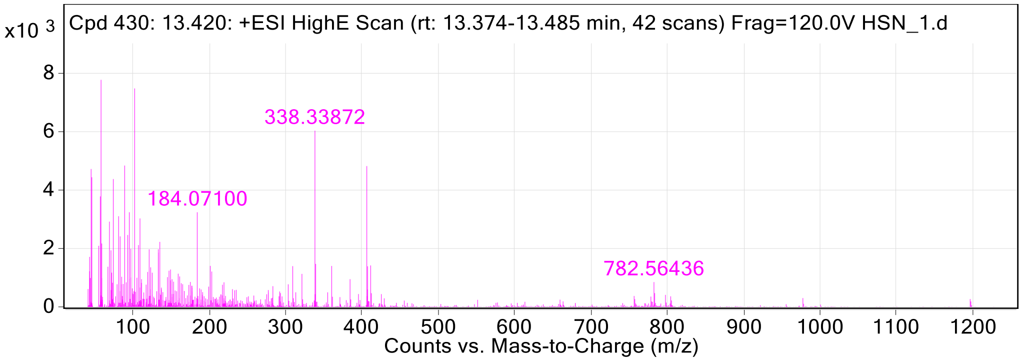

Peak List

m/z	z	Abund
45.03314		4711.81
46.06482		4429.18
57.06935		3775.62
58.06463		7759.82
74.09557		4368.68
89.05848		4830.32
95.08403		3233.93
102.12626	1	7466.34
338.33872	1	6023.13
406.32578	1	4812.37

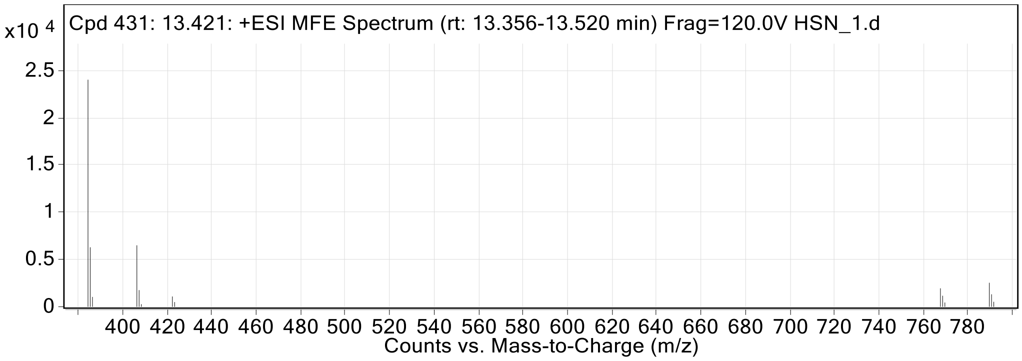

Peak List

m/z	z	Abund	Ion
384.3442	1	24013.84	(M+H)+
385.34715	1	6292.2	(M+H)+
386.35141	1	1041.67	(M+H)+
406.32563	1	6500.43	(M+Na)+
407.32987	1	1761.9	(M+Na)+
422.29879	1	1089.01	(M+K)+
767.6822	1	1951.94	(2M+H)+
768.68512	1	1165.59	(2M+H)+
789.66401	1	2539.04	(2M+Na)+
790.66497	1	1319.62	(2M+Na)+

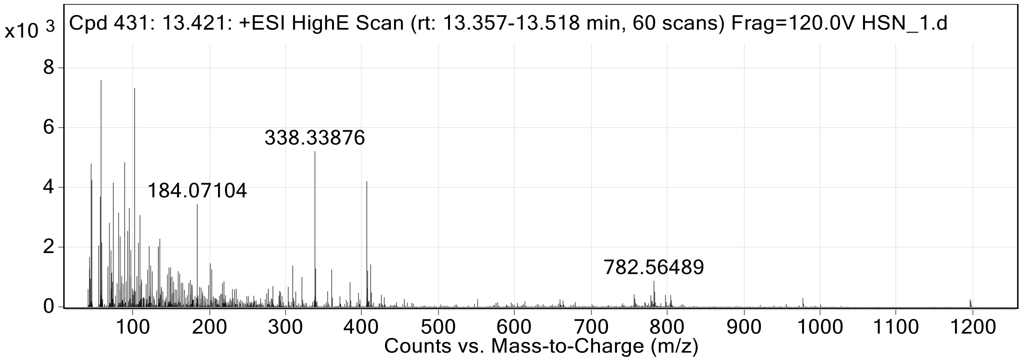

Peak List

m/z	z	Abund
45.03313		4781.93
46.06481		4227.89
57.06932		3673.58
58.06461		7578.52
74.09557		4147.3
89.05849		4825.68
102.12626		7302.32
184.07104		3429.74
338.33876	1	5194.23
406.32586	1	4198.31

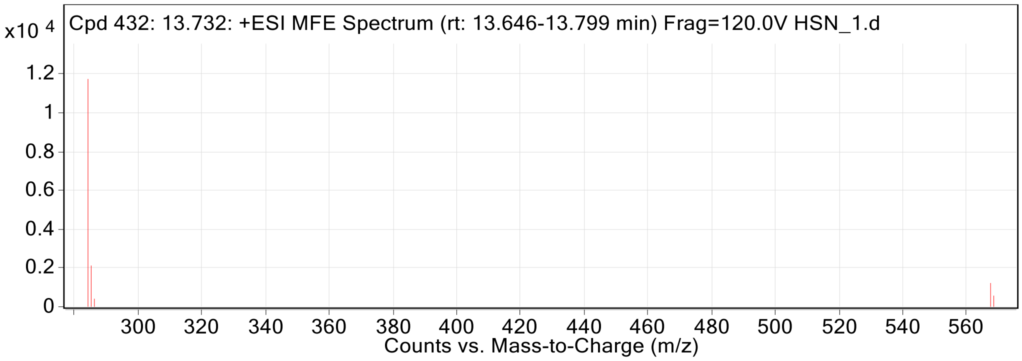

Peak List

m/z	z	Abund	Ion
284.29204	1	11752.13	(M+H)+
285.29513	1	2127.62	(M+H)+
286.30085	1	415.21	(M+H)+
567.57747	1	1225.42	(2M+H)+
568.57819	1	573.81	(2M+H)+

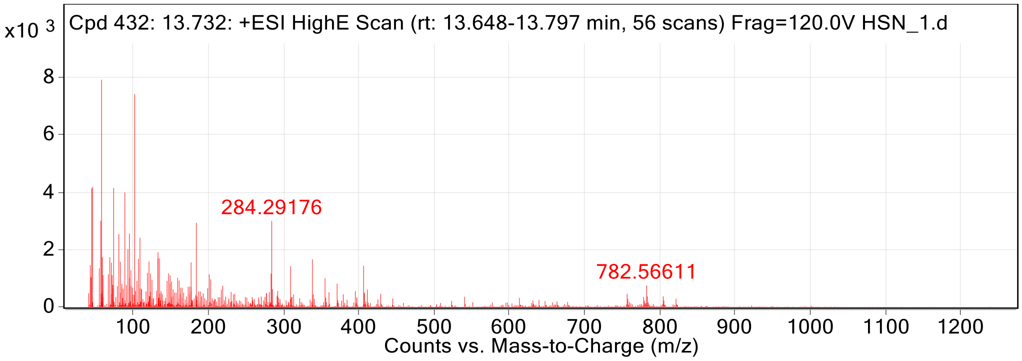

Peak List

m/z	z	Abund

45.03311		4131.56
46.06472		4179
57.0693		2998.59
58.06458	1	7920.69
74.09553		4146.86
89.05842		3989.86
95.08394		2557.11
102.12608	1	7421.03
184.07084		2931.69
284.29176	1	2994.8

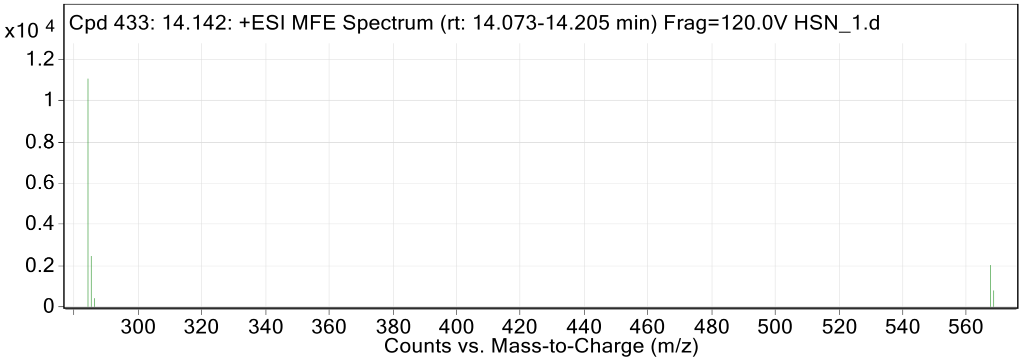

Peak List

m/z	z	Abund	Ion
284.29273	1	11069.38	(M+H)+
285.29597	1	2466.26	(M+H)+
286.30122	1	414.03	(M+H)+
567.58044	1	2025.57	(2M+H)+
568.58169	1	788.45	(2M+H)+

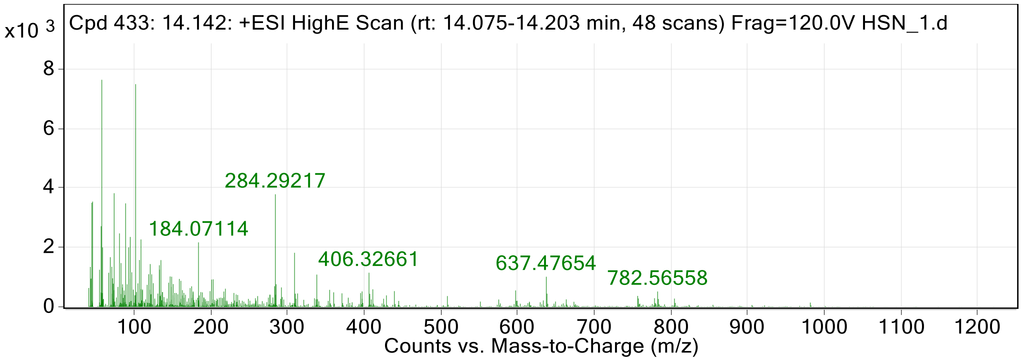

Peak List

m/z	z	Abund
45.03314		3502.64
46.06488		3542.54
57.06939		2714.05
58.06464	1	7640.97
74.09553		3823.84
81.06887		2471.58
89.05851		3481.8
95.08412		2352.21
102.12632	1	7494
284.29217	1	3787.42

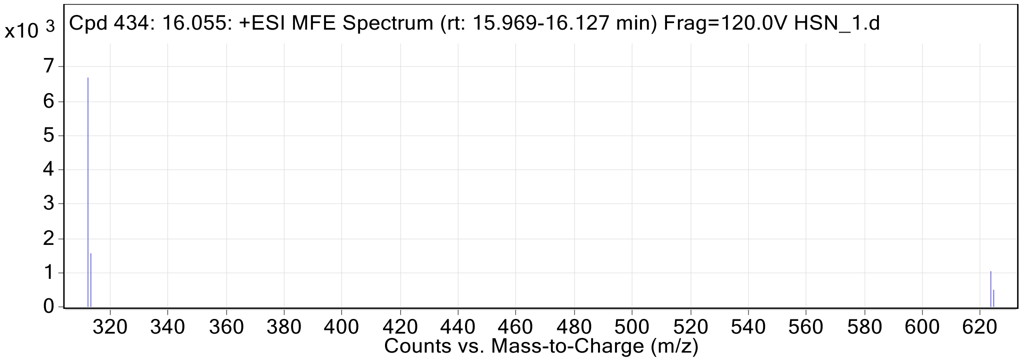

Peak List

m/z	z	Abund	Ion
312.3246	1	6696.49	(M+H)+
313.3271	1	1565.25	(M+H)+
623.64249	1	1042.9	(2M+H)+
624.6454	1	499.42	(2M+H)+

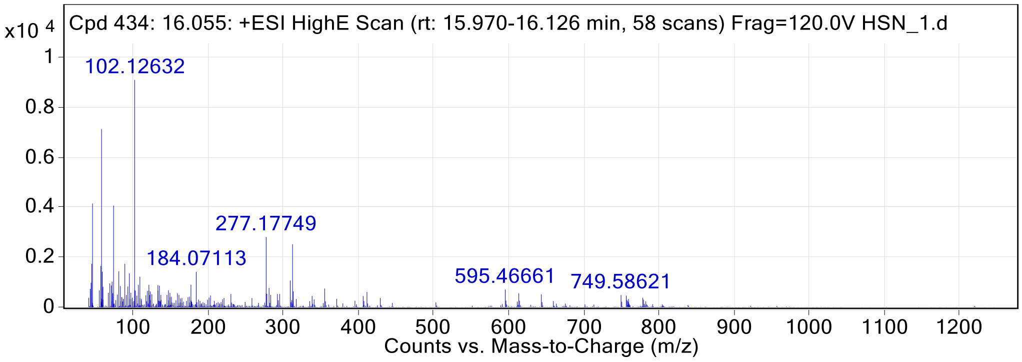

Peak List

m/z	z	Abund
45.03319		1731.35
46.06477		4144.96
57.06931		1644.8
58.06462		7132.02
74.09559		4064.1
81.06879		1434.33
89.05851		1729.7
102.12632	1	9097.38
277.17749	1	2804.46
312.32361	1	2516.88

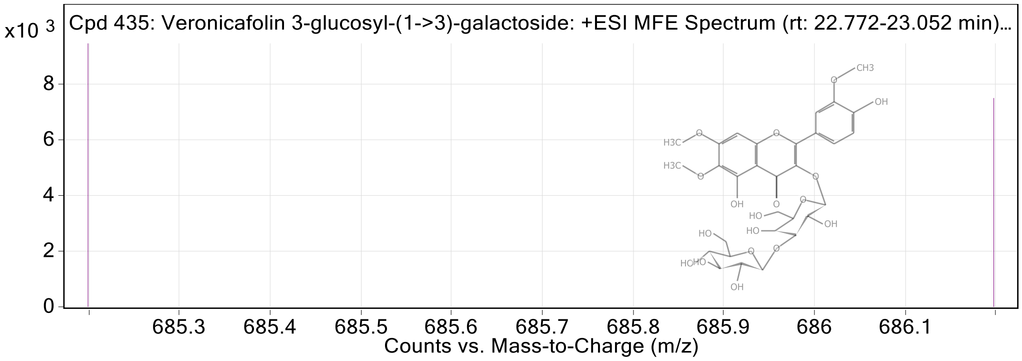

Peak List

m/z	z	Abund	Name	Formula	Ion	Score (DB)	Hits (DB)

685.19911	1	9459.87	Veronicafolin 3-glucosyl-(1->3)-galactoside	C30 H36 O18	(M+H)+	51.67	6
686.19749	1	7496.97		C30 H36 O18	(M+H)+		

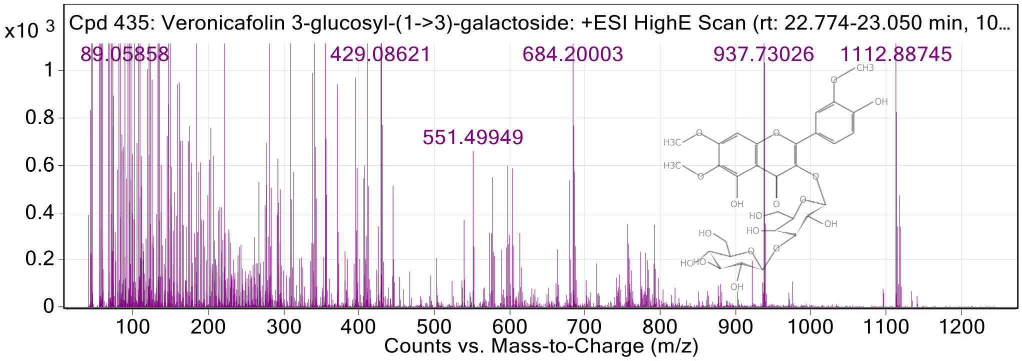

Peak List

m/z	z	Abund
45.03331		3797.67
46.06484		2355.34
57.06941		2216.93
81.06896		2453.5
89.05858		3883.73
95.08429		2606.53
102.1263		2257.57
109.09963		2622.65
184.07127		2528.98
429.08621	1	2675.79

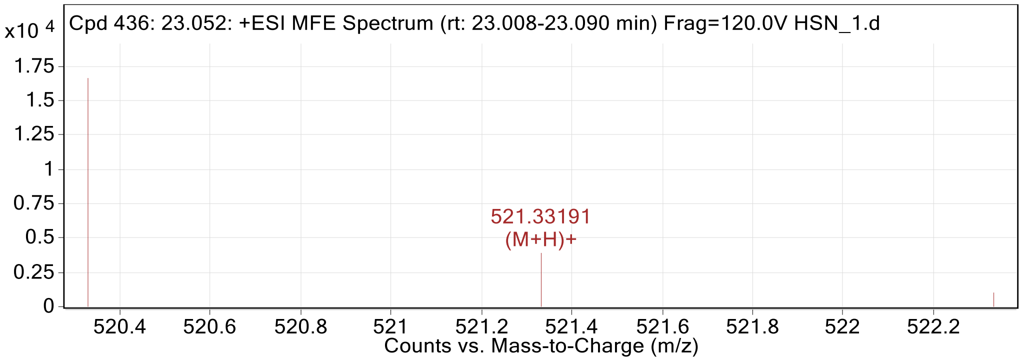

Peak List

m/z	z	Abund	Ion
520.32888	1	16642.01	(M+H)+
521.33191	1	3914.65	(M+H)+
522.33338	1	1029.23	(M+H)+

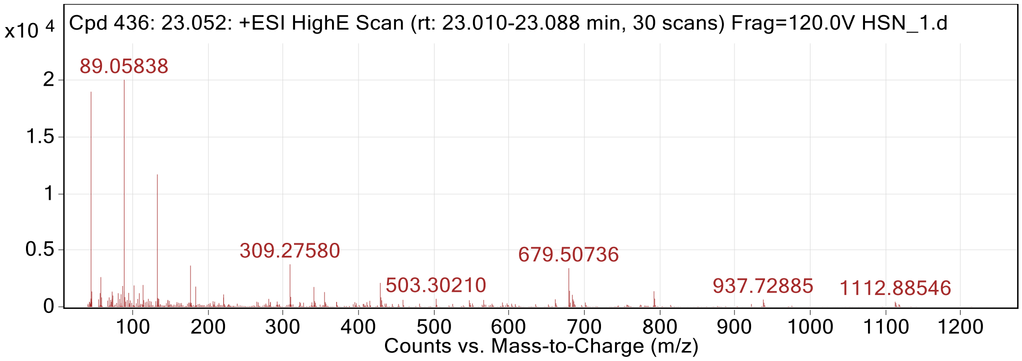

Qualitative Analysis Report

Peak List

m/z	z	Abund
45.03311	1	18994.47
58.06458		2625.07
89.05838	1	20045.21
102.12612		1888.98
114.0894		1930.02

m/z	z	Abund
133.08391	1	11692.41
177.10979		3643.17
309.2758	1	3757.86
429.08551	1	2112.59
679.50736	1	3414.45

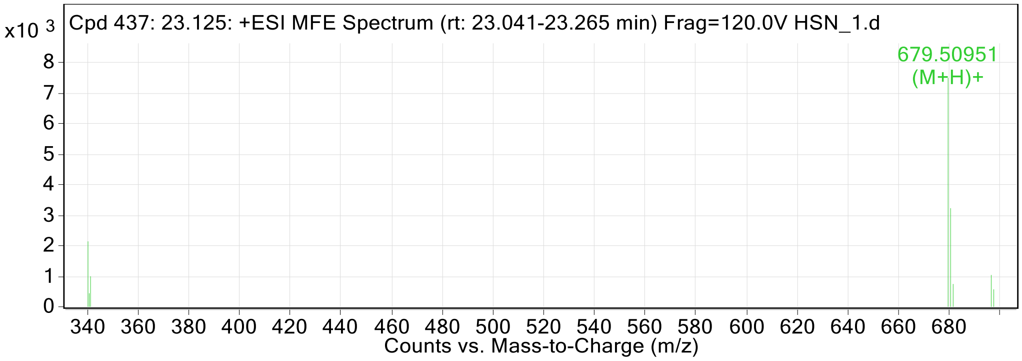

Peak List

m/z	z	Abund	Ion
340.25658	2	2141.92	(M+2H)+2
340.76061	2	442.77	(M+2H)+2
341.26143	2	999.69	(M+2H)+2
679.50951	1	7495.06	(M+H)+
680.51225	1	3228.8	(M+H)+
681.51515	1	746.49	(M+H)+
696.53378	1	1041.81	(M+NH4)+
697.54168	1	570.6	(M+NH4)+

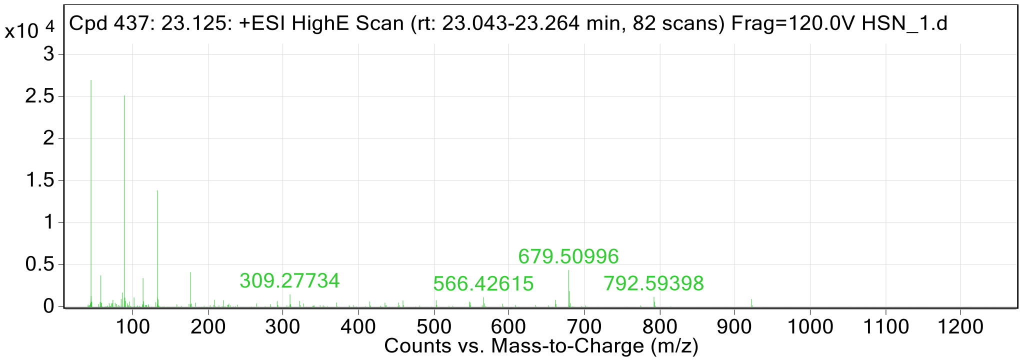

Peak List

m/z	z	Abund
45.03343	1	27016.73
58.06491		3735.35
87.04327		1683.28
89.05892	1	25175.36
114.09008	1	3414.96
133.08461	1	13882.57
177.11068	1	4123.48
309.27734	1	1485.45
679.50996	1	4371.85
680.51311	1	1855.78

--- End Of Report ---

Qualitative Analysis Report

**Data Filename**	HSN_2.d	**Sample Name**	HSN_2
**Sample Type**	Sample	**Position**	P1-A5
**Instrument Name**	6530B LC Q-TOF	**User Name**	Hasan CAN (hcan)
**Acq Method**	Unknown_3-Expt_(neg).m	**Acquired Time**	3/24/2025 5:21:51 PM
**IRM Calibration Status**	Success	**DA Method**	Default.m
**Comment**			

**Sample Group**		**Info.**	
**Stream Name**	LC 1	**Method Version**	2025-0324-1207-54604
**Override DA Method Version**		**Data File Version**	2025-0324-1421-47776
**Acquisition Workstation**	DESKTOP-L73MD3C	**DA Workstation**	DESKTOP-L73MD3C
**Acquisition SW Version**	6200 series TOF/6500 series Q-TOF (11.0.203.0)		

User Chromatograms

Fragmentor Voltage 120 **Collision Energy** 0 **Ionization Mode** ESI

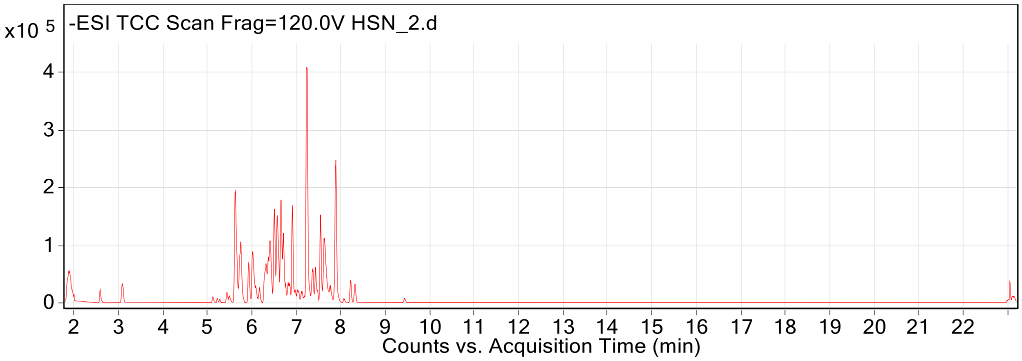

Compounds

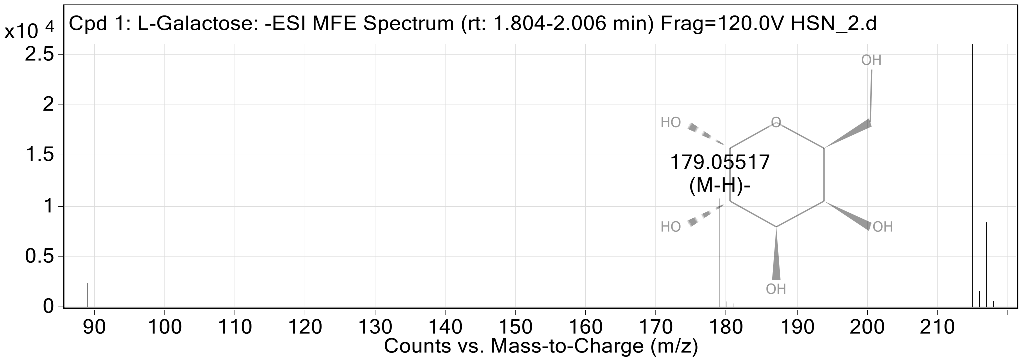

Peak List

m/z	z	Abund	Name	Formula	Ion	Score (DB)	Hits (DB)
89.02399	-2	2371.08			(M-2H)-2		
179.05517	-1	10751.2			(M-H)-		
180.0586	-1	538.6			(M-H)-		
181.06063	-1	340.35			(M-H)-		
215.03194	-1	26091.64	L-Galactose	C6 H12 O6	(M+Cl)-	95.77	10
216.03539	-1	1561.39		C6 H12 O6	(M+Cl)-		
217.02917	-1	8390.8		C6 H12 O6	(M+Cl)-		
218.03293	-1	587.11		C6 H12 O6	(M+Cl)-		

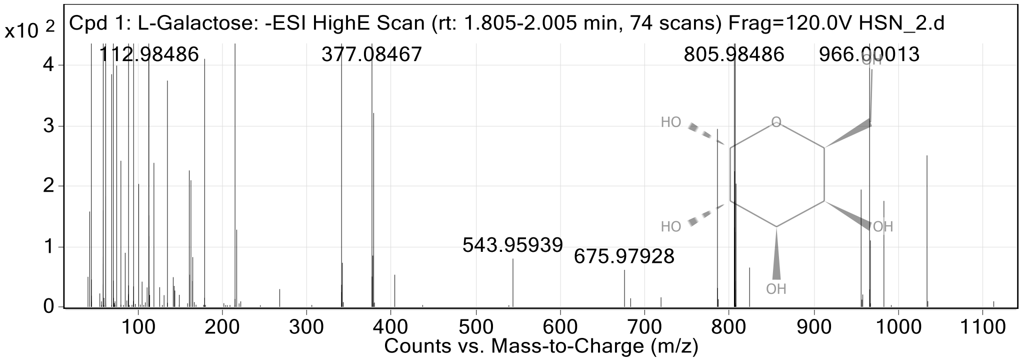

Peak List

m/z	z	Abund
44.99812		1393.73
59.01376	1	3203.62
61.98824		3431.68
71.01371		1181.87
89.02399		1115.73
112.98486	1	4242.92
341.10781	1	778.05
377.08467	1	829.89
805.98486	1	7567.51
806.98747	1	1385.38

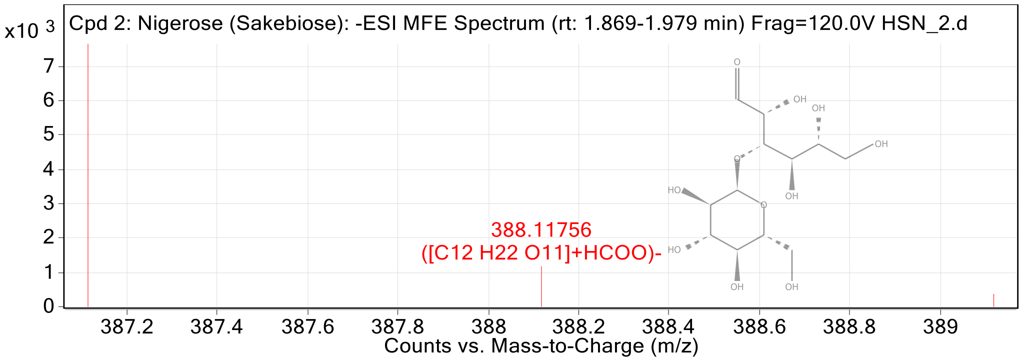

Peak List

m/z	z	Abund	Name	Formula	Ion	Score (DB)	Hits (DB)
387.11343	-1	7661.8	Nigerose (Sakebiose)	C12 H22 O11	(M+HCOO)-	96.54	10
388.11756	-1	1179.35		C12 H22 O11	(M+HCOO)-		
389.11902	-1	373.83		C12 H22 O11	(M+HCOO)-		

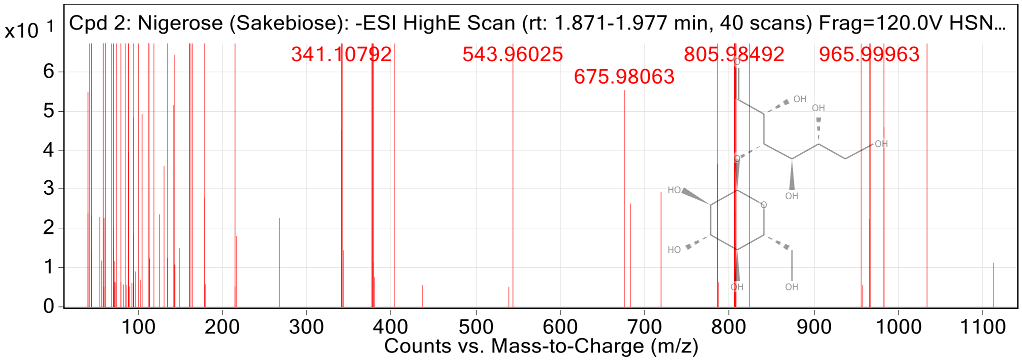

Peak List

m/z	z	Abund
44.99815		1520.49
59.01378	1	4290.16

61.98826		4383.08
71.01367		1615.3
89.024		1651.27
112.98481	1	4226.73
341.10792	1	1357.38
377.08485	1	1243.62
805.98492	1	7499.77
806.98797	1	1370.05

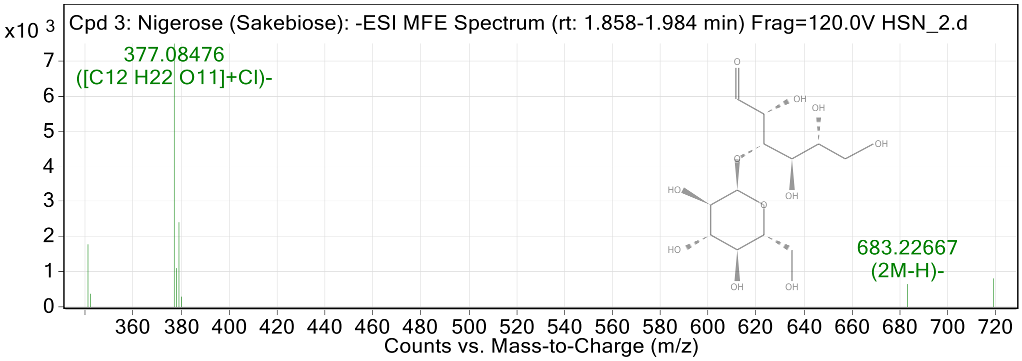

Peak List

m/z	z	Abund	Name	Formula	Ion	Score (DB)	Hits (DB)
341.10761	-1	1778.61			(M-H)-		
342.11407	-1	374.44			(M-H)-		
377.08476	-1	7501.47	Nigerose (Sakebiose)	C12 H22 O11	(M+Cl)-	96.16	10
378.08811	-1	1103.54		C12 H22 O11	(M+Cl)-		
379.0836	-1	2406.42		C12 H22 O11	(M+Cl)-		
380.08295	-1	291.24		C12 H22 O11	(M+Cl)-		
683.22667	-1	649.96			(2M-H)-		
719.20315	-1	808.71			(2M+Cl)-		

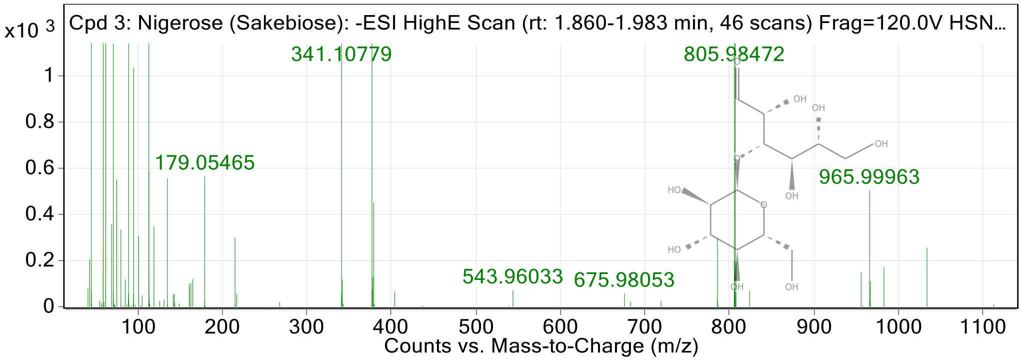

Peak List

m/z	z	Abund
44.99815		1521.65
59.01375	1	4212.46
61.98822		4173.9
71.01365		1575.4
89.02399		1565.11
112.98478	1	4210.24
341.10779	1	1192
377.08469	1	1139.28
805.98472	1	7531.63
806.98773	1	1356.16

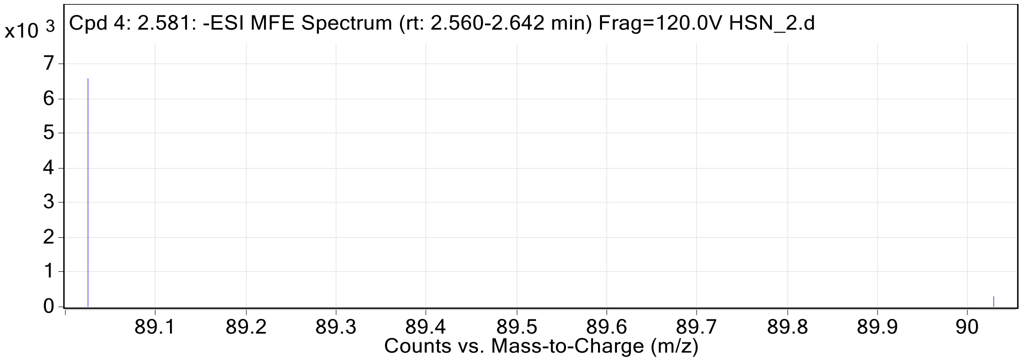

Peak List

m/z	z	Abund	Ion
89.0249	-1	6590.74	(M-H)-
90.0286	-1	304.83	(M-H)-

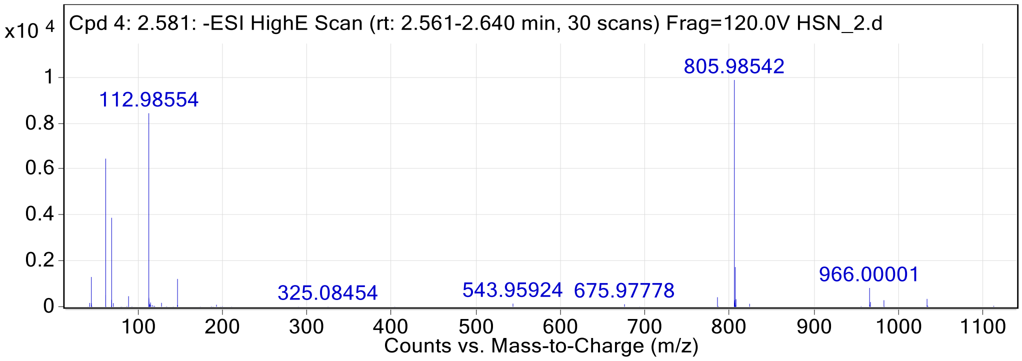

Peak List

m/z	z	Abund
44.99863		1294.79
61.98864		6448.84
62.00517		506.41
68.99611		3873.21
89.02439		457.77
112.98554	1	8427.03
146.96574		1210.28
805.98542	1	9875.14
806.9882	1	1725.86
966.00001	1	816.99

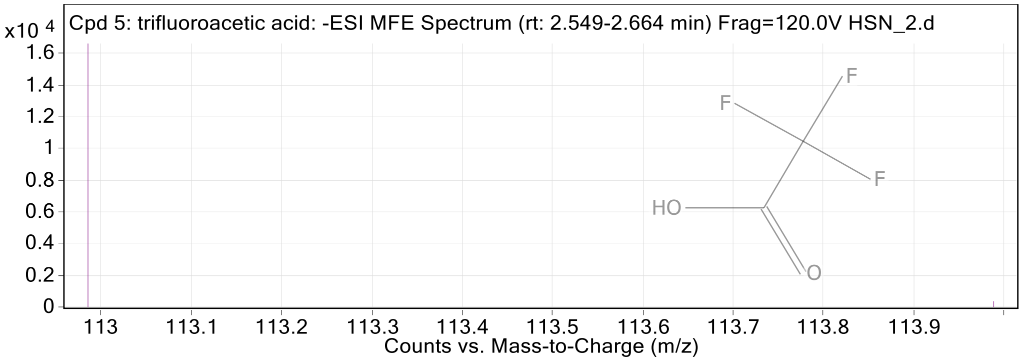

Peak List

m/z	z	Abund	Name	Formula	Ion	Score (DB)	Hits (DB)
112.98559	-1	16613.25	trifluoroacetic acid	C2 H F3 O2	(M-H)-	88	1
113.98879	-1	362.16		C2 H F3 O2	(M-H)-		

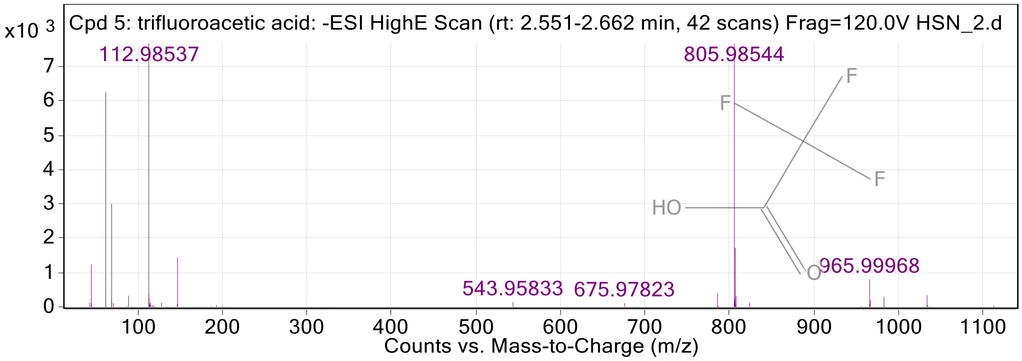

Peak List

m/z	z	Abund
44.99853		1236.65
61.98856		6243.65
62.00504		460.07
68.99597		3000.38
112.98537	1	7668.55
113.00512		398.92
146.96564		1435.84
805.98544	1	9750.18
806.98807	1	1723.62
965.99968	1	800.22

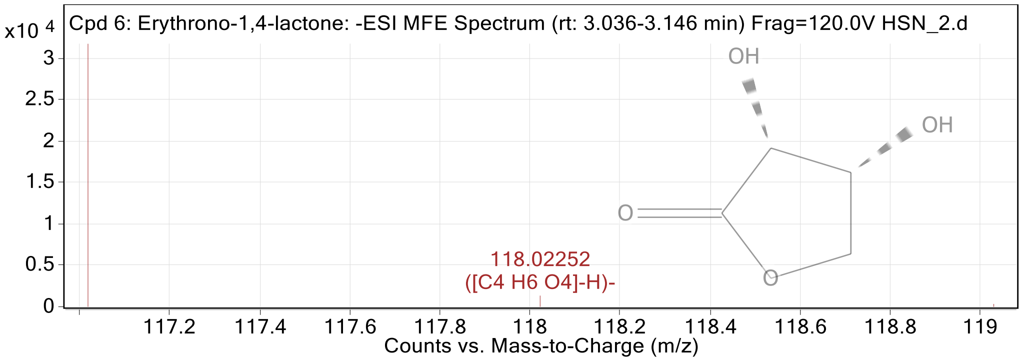

Peak List

m/z	z	Abund	Name	Formula	Ion	Score (DB)	Hits (DB)
117.01877	-1	31753.02	Erythrono-1,4-lactone	C4 H6 O4	(M-H)-	97.8	8
118.02252	-1	1346.87		C4 H6 O4	(M-H)-		
119.02962	-1	340		C4 H6 O4	(M-H)-		

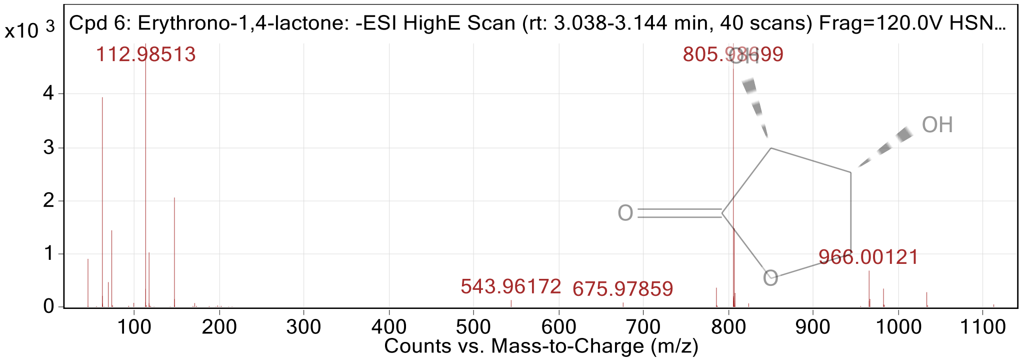

Peak List

m/z	z	Abund
44.99833		904.27
61.98837		3944.87

68.99563		466.15
73.02953	1	1440.51
112.98513	1	4956.72
117.01864	1	1025.08
146.96522		2057.88
805.98699	1	8508.36
806.99079	1	1479.93
966.00121	1	682.29

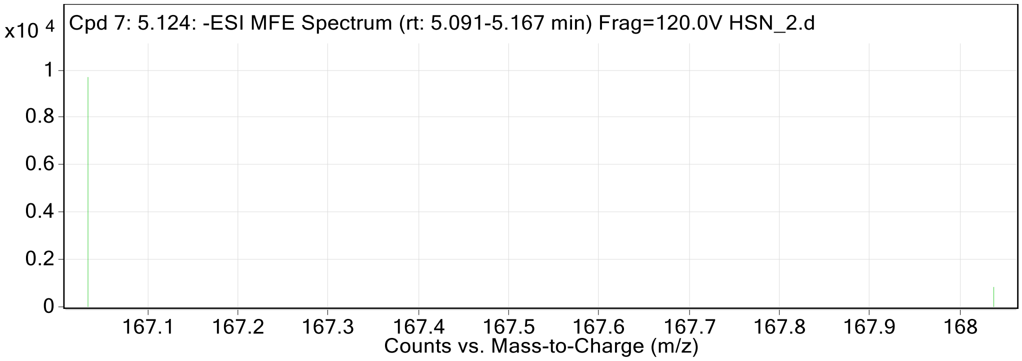

Peak List

m/z	z	Abund	Ion
167.03374	-1	9699.7	(M-H)-
168.03753	-1	839.45	(M-H)-

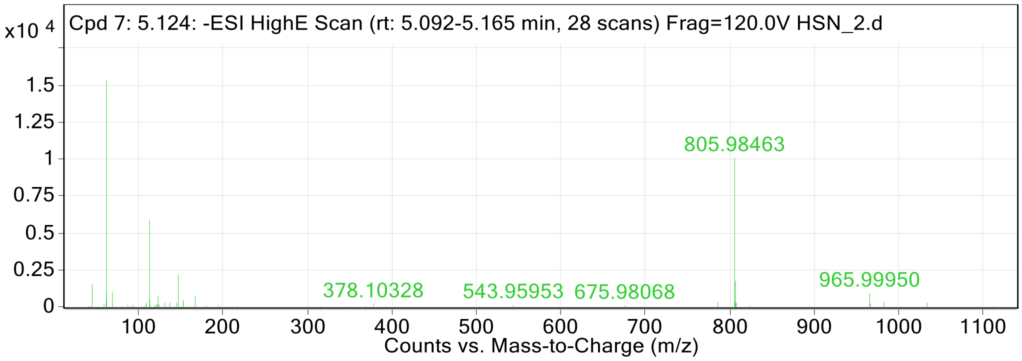

Peak List

m/z	z	Abund
44.99816		1558.73
61.98828		15354.68
62.00548		901.35
68.99545		1023.83
112.98478	1	5912.83
123.04429		754.38
146.96531		2149.88
805.98463	1	10077.42
806.98783	1	1753.88
965.9995	1	906.77

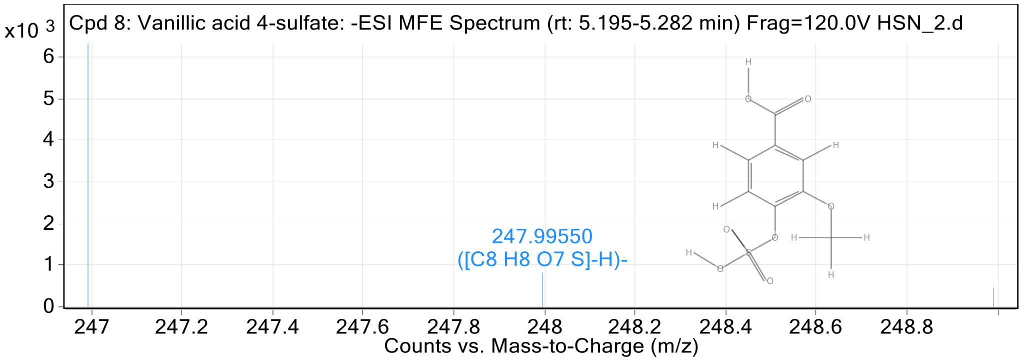

Peak List

m/z	z	Abund	Name	Formula	Ion	Score (DB)	Hits (DB)
246.99267	-1	6332.39	Vanillic acid 4-sulfate	C8 H8 O7 S	(M-H)-	93.97	3
247.9955	-1	819.4		C8 H8 O7 S	(M-H)-		

m/z	z	Abund	Name	Formula	Ion	Score (DB)	Hits (DB)
248.9911	-1	462.94		C8 H8 O7 S	(M-H)-		

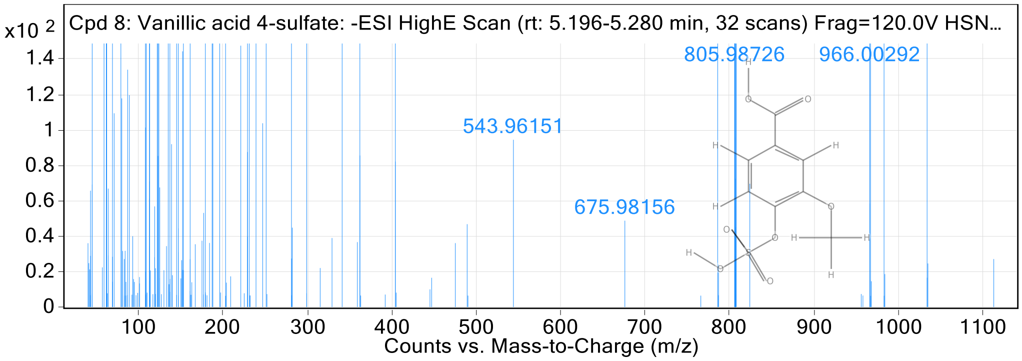

Peak List

m/z	z	Abund
44.99838		1783.77
61.98868		16320.5
62.00503		1464.56
68.99609		861.69
108.04554		592.55
112.98554		4473.47
146.96596		1413.81
805.98726	1	7700.4
806.99062	1	1377.98
966.00292	1	785.02

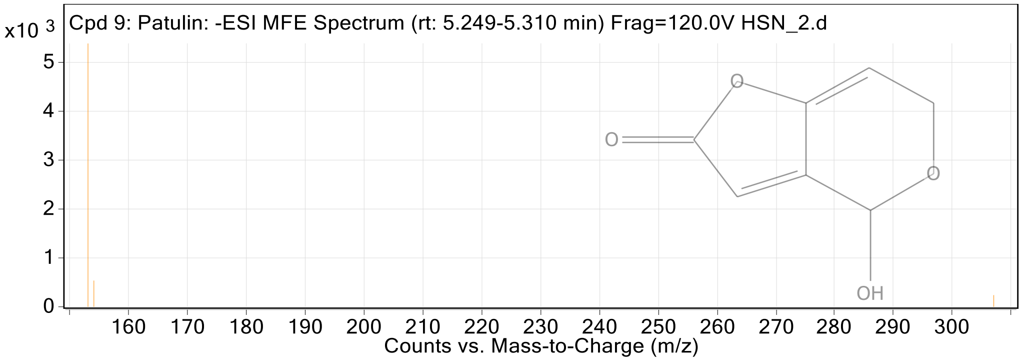

Peak List

m/z	z	Abund	Name	Formula	Ion	Score (DB)	Hits (DB)
153.01955	-1	5376.81	Patulin	C7 H6 O4	(M-H)-	85.46	9
154.0227	-1	535.22		C7 H6 O4	(M-H)-		
307.04802	-1	238.63			(2M-H)-		

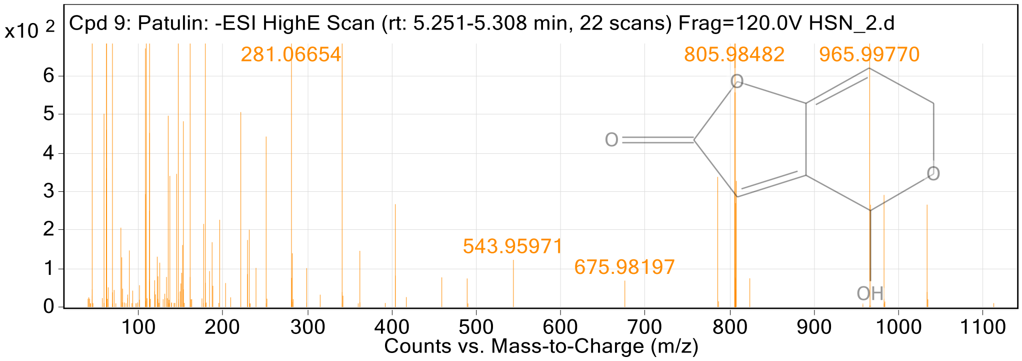

Peak List

m/z	z	Abund

44.99858		1718.12
61.98878		16253.79
62.00527		1411.15
68.99627		1092.52
109.02948		1349.78
112.9854		5488.38
146.96591		1377.18
179.03462		1026.27
805.98482	1	9613.9
806.98793	1	1707.94

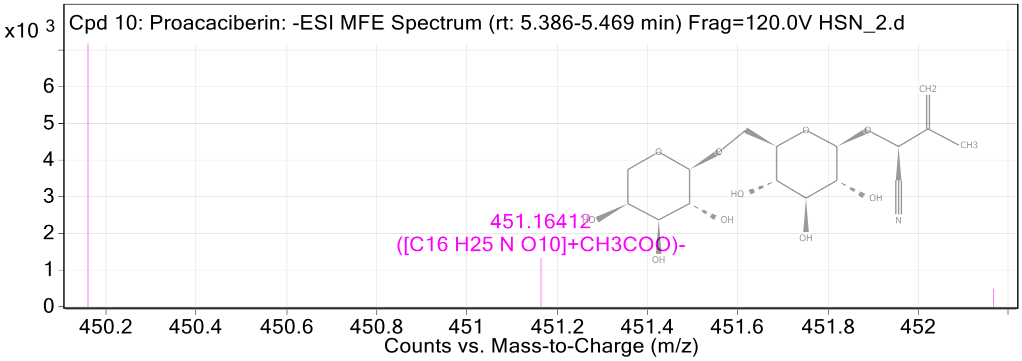

Peak List

m/z	z	Abund	Name	Formula	Ion	Score (DB)	Hits (DB)
450.16059	-1	7172.4	Proacaciberin	C16 H25 N O10	(M+CH3COO)-	93.9	1
451.16412	-1	1329.85		C16 H25 N O10	(M+CH3COO)-		
452.16723	-1	491.14		C16 H25 N O10	(M+CH3COO)-		

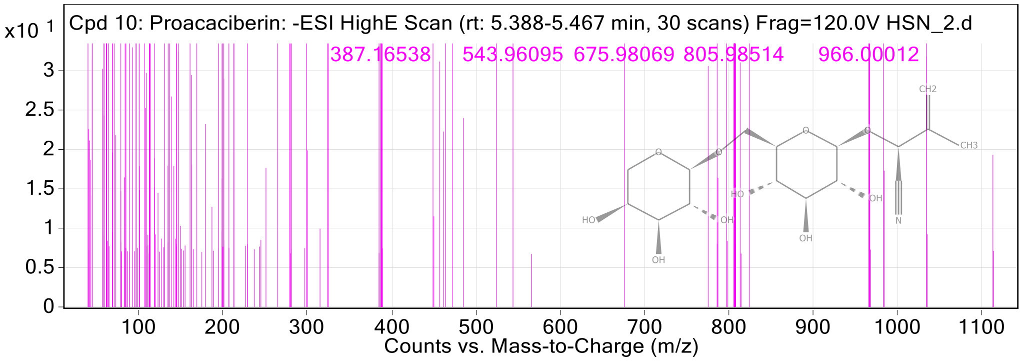

Peak List

m/z	z	Abund
44.99831		1701.52
59.01381		1238.5
61.98837		19510.48
62.00451		2080.38
68.99552		964.02
112.98506	1	5708.22
146.96555		1059.16
387.16538	1	3445.17
805.98514	1	9746.78
806.98801	1	1800.32

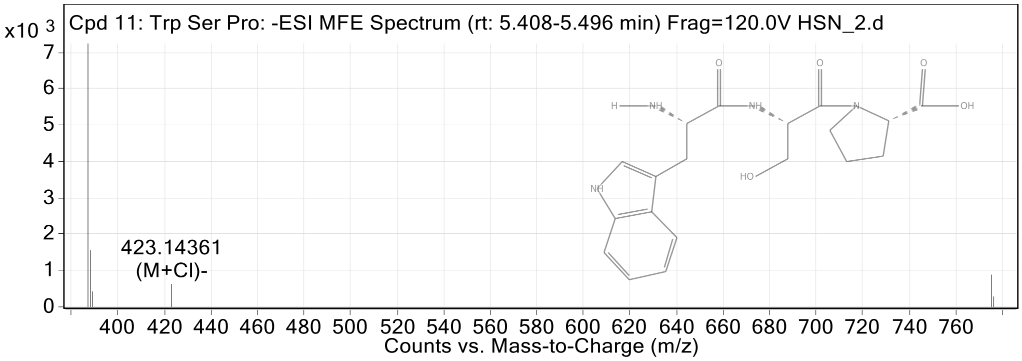

Peak List

m/z	z	Abund	Name	Formula	Ion	Score (DB)	Hits (DB)
387.16714	-1	7247.8	Trp Ser Pro	C19 H24 N4 O5	(M-H)-	97.62	10
388.17008	-1	1557.97		C19 H24 N4 O5	(M-H)-		
389.17237	-1	424.61		C19 H24 N4 O5	(M-H)-		
423.14361	-1	631.84			(M+Cl)-		
775.33515	-1	887.14			(2M-H)-		
776.3429	-1	285.79			(2M-H)-		

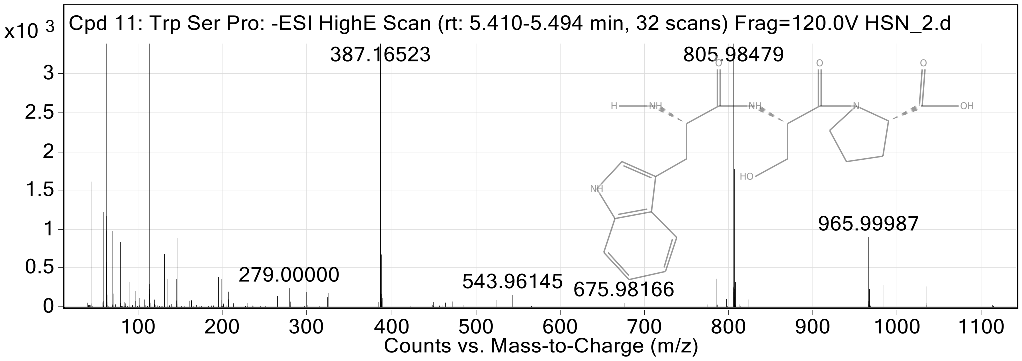

Peak List

m/z	z	Abund
44.99825		1612.07
59.01374		1217.49
61.9883		21087.44
62.0036		1166.22
62.00592		981.97
68.99543		977.59
112.98493	1	5661.3
387.16523	1	3390.19
805.98479	1	9856.29
806.98791	1	1777.12

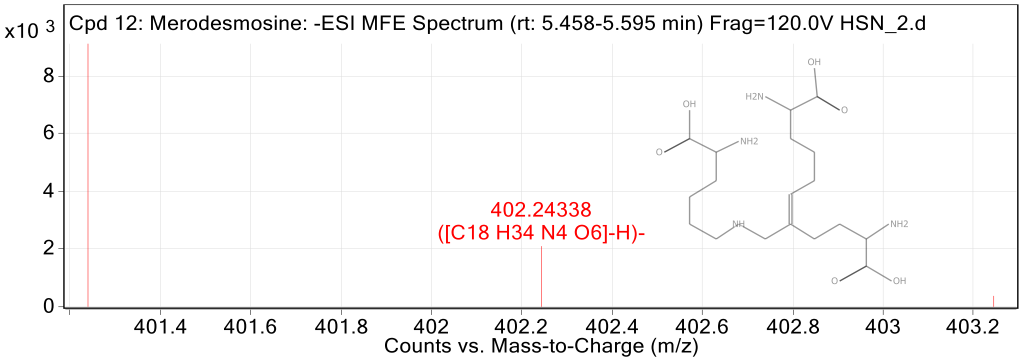

Peak List

m/z	z	Abund	Name	Formula	Ion	Score (DB)	Hits (DB)
401.23991	-1	9117.14	Merodesmosine	C18 H34 N4 O6	(M-H)-	98.22	10
402.24338	-1	2102.22		C18 H34 N4 O6	(M-H)-		
403.24469	-1	378		C18 H34 N4 O6	(M-H)-		

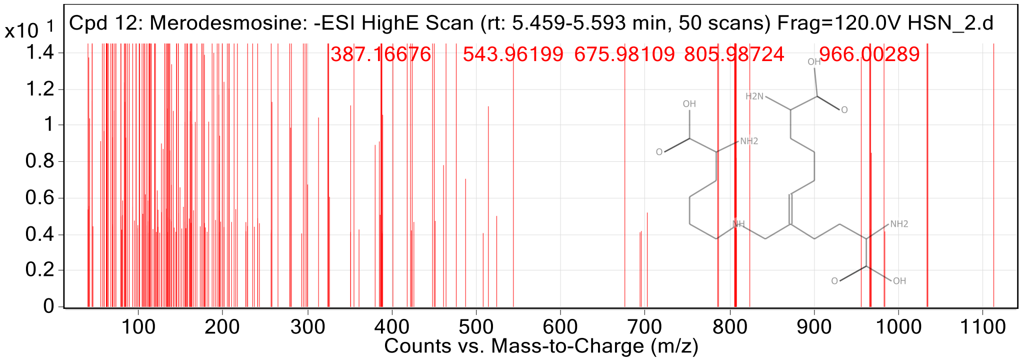

Peak List

m/z	z	Abund
44.99836		1881.52
61.98844		22221.77
62.0051		1826.99
68.9957		917.12
112.98526	1	4944.56
130.99204		1275.16
146.96579		932.58
805.98724	1	8701.66
806.99045	1	1555.26
966.00289	1	857.09

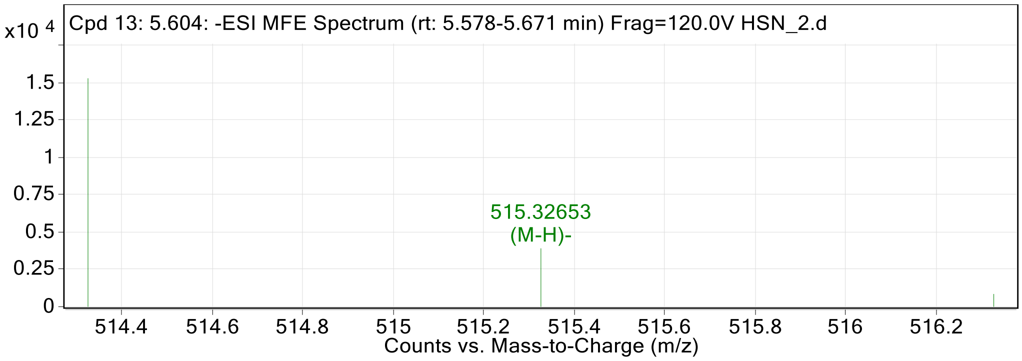

Peak List

m/z	z	Abund	Ion
514.32474	-1	15309.7	(M-H)-
515.32653	-1	3915	(M-H)-
516.32817	-1	856.38	(M-H)-

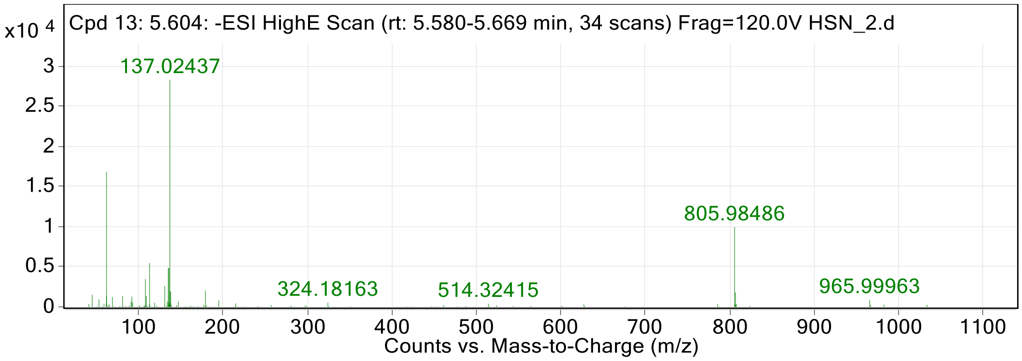

Peak List

m/z	z	Abund
61.98883		16836.48
108.02184		3474.57
112.98575		5467.86
130.99258		2607.41
135.04511	1	4839.94
136.01663		4850.21
137.02437	1	28267.49
137.05042	1	1968.13
179.03503	1	2051.32
805.98486	1	9932.96

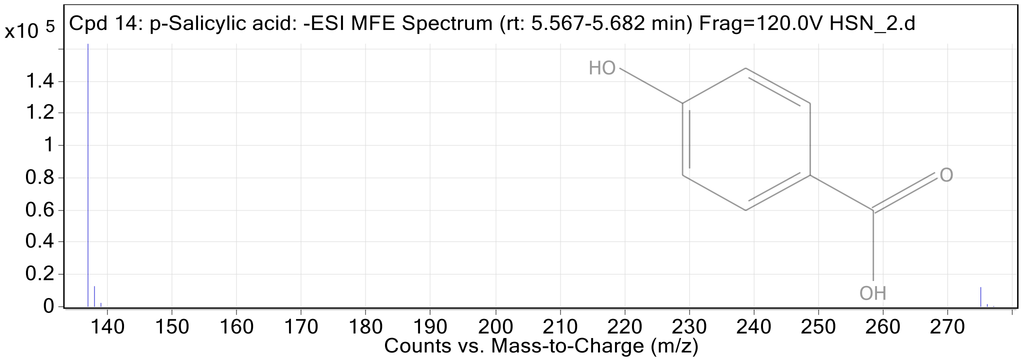

Peak List

m/z	z	Abund	Name	Formula	Ion	Score (DB)	Hits (DB)
137.02403	-1	163275.3	p-Salicylic acid	C7 H6 O3	(M-H)-	98.11	9
138.02828	-1	12781.99		C7 H6 O3	(M-H)-		
139.0296	-1	2403.33		C7 H6 O3	(M-H)-		
275.05658	-1	12193.26			(2M-H)-		
276.05911	-1	1641.18			(2M-H)-		
277.06119	-1	427.94			(2M-H)-		

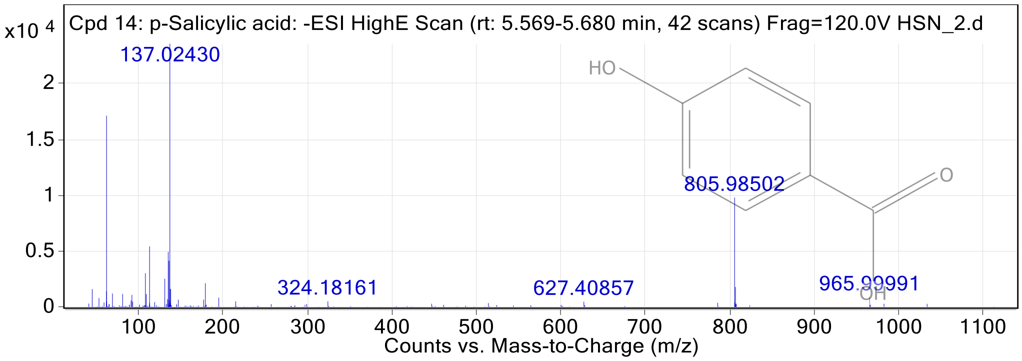

Peak List

m/z	z	Abund
61.98879		17119.66
108.02181		3005.31
112.98569		5413.05
130.99258		2514.6
135.04506	1	4930.76
136.01657		4121.14
137.0243	1	23565.5
179.03491	1	2103.81
805.98502	1	9785.45
806.9883	1	1771.27

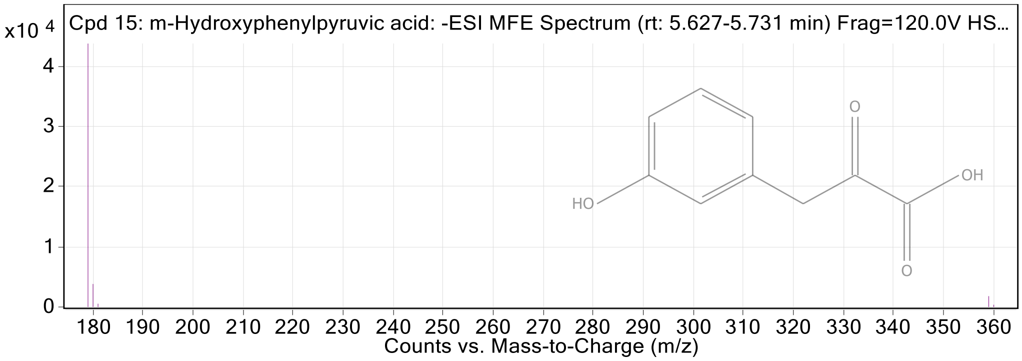

Peak List

m/z	z	Abund	Name	Formula	Ion	Score (DB)	Hits (DB)
179.03475	-1	43806.33	m-Hydroxyphenylpyruvic acid	C9 H8 O4	(M-H)-	99.11	10
180.03843	-1	3824.47		C9 H8 O4	(M-H)-		
181.03944	-1	543.81		C9 H8 O4	(M-H)-		
359.07713	-1	1800.84			(2M-H)-		
360.0806	-1	372.15			(2M-H)-		

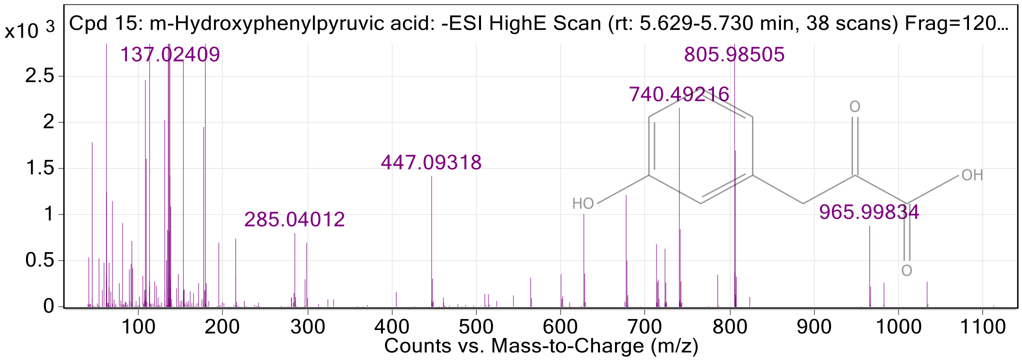

Peak List

m/z	z	Abund
61.98862		16770.56
108.02165		2453.94
112.98549		5353.6
135.04482	1	6760.06
136.01646		3053.62
137.02409	1	16333.91
153.01904	1	2678.43
179.03463	1	2847.17
740.49216	1	2151.9
805.98505	1	9500.03

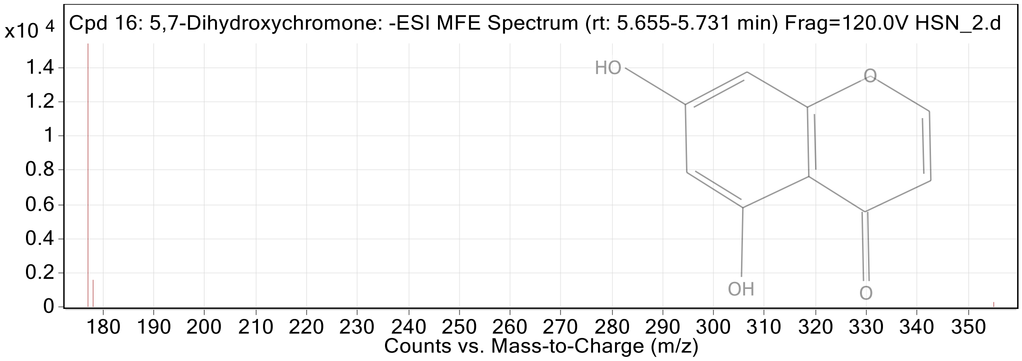

Peak List

m/z	z	Abund	Name	Formula	Ion	Score (DB)	Hits (DB)
177.01875	-1	15438.27	5,7-Dihydroxychromone	C9 H6 O4	(M-H)-	85.62	6
178.02214	-1	1585		C9 H6 O4	(M-H)-		
355.04681	-1	284.19			(2M-H)-		

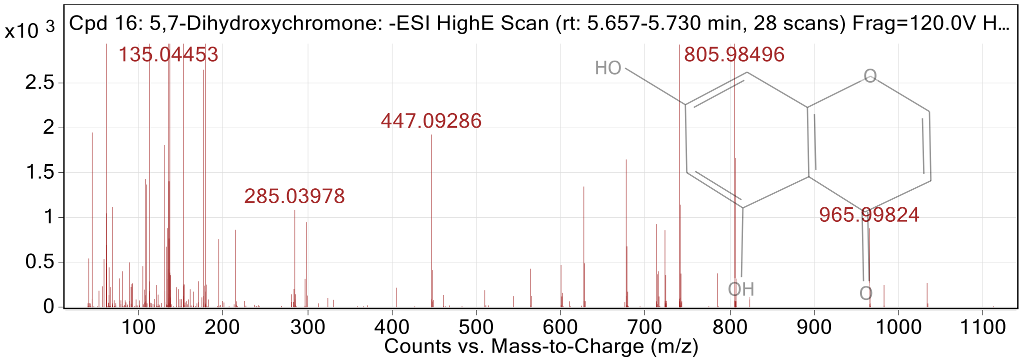

Peak List

m/z	z	Abund
44.99835		1942.26
61.9884		18659.61
112.98521		5401.09
135.04453	1	6963.6
137.02386	1	5475.68
153.01872	1	3635.01
177.01864	1	2639.83
179.03436	1	2930.54
740.49195	1	2920.44
805.98496	1	9517.05

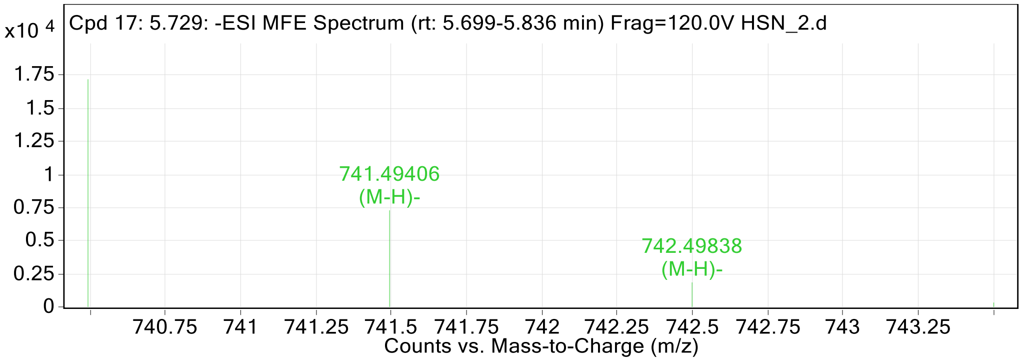

Peak List

m/z	z	Abund	Ion
740.49276	-1	17196.95	(M-H)-
741.49406	-1	7296.98	(M-H)-

742.49838	-1	1850.6	(M-H)-
743.50006	-1	327.53	(M-H)-

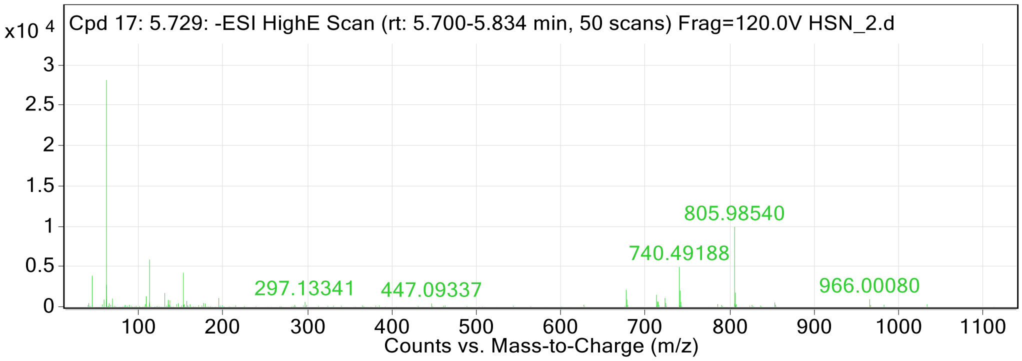

Peak List

m/z	z	Abund
44.9982		3863.9
61.98824		28141.38
62.00434		2713.26
112.98493		5853.65
153.01846	1	4214.16
677.49639	1	2118.52
740.49188	1	4953.88
741.49507	1	2011.84
805.9854	1	9907.84
806.98823	1	1751.21

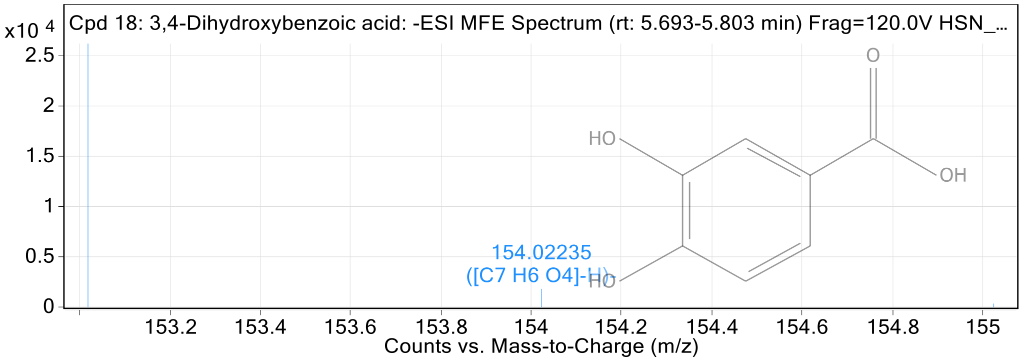

Peak List

m/z	z	Abund	Name	Formula	Ion	Score (DB)	Hits (DB)
153.0186	-1	26201.32	3,4-Dihydroxybenzoic acid	C7 H6 O4	(M-H)-	96.75	7
154.02235	-1	1806.93		C7 H6 O4	(M-H)-		
155.0243	-1	340.6		C7 H6 O4	(M-H)-		

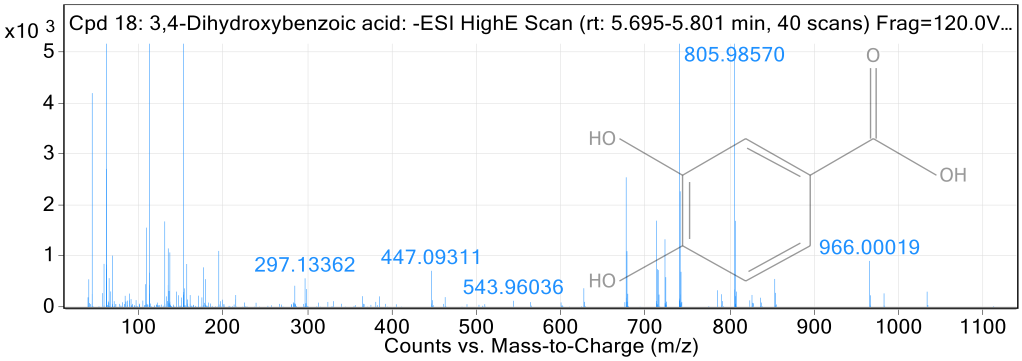

Peak List

m/z	z	Abund

44.99826		4199.05
61.98827		28066.64
62.00449		2710.17
112.98503		5739.41
153.01852	1	5169.78
677.49677	1	2544.63
713.47343	1	1692.35
740.4923	1	5614.73
741.49543	1	2269.27
805.9857	1	9700.75

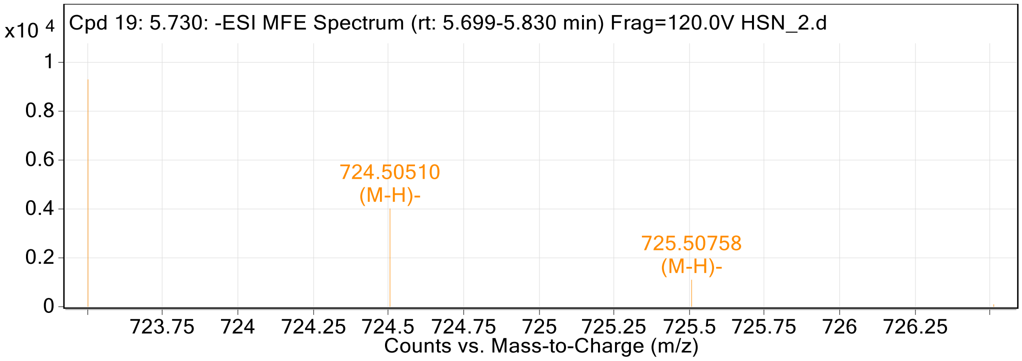

Peak List

m/z	z	Abund	Ion
723.50138	-1	9305.79	(M-H)-
724.5051	-1	4019.76	(M-H)-
725.50758	-1	1105.41	(M-H)-
726.51175	-1	99.85	(M-H)-

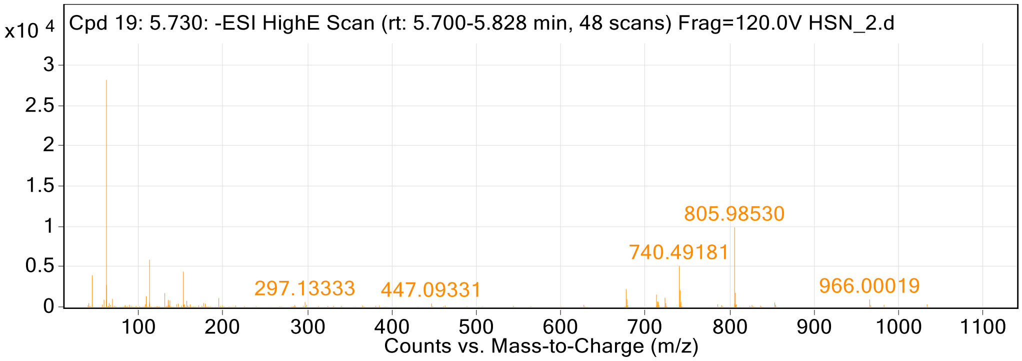

Peak List

m/z	z	Abund
44.9982		3924.9
61.98824		28167.01
62.00436		2705.19
112.98491		5838.06
153.01844	1	4371.54
677.49626	1	2201.18
740.49181	1	5087.04
741.49499	1	2071.06
805.9853	1	9863.01
806.98811	1	1743.78

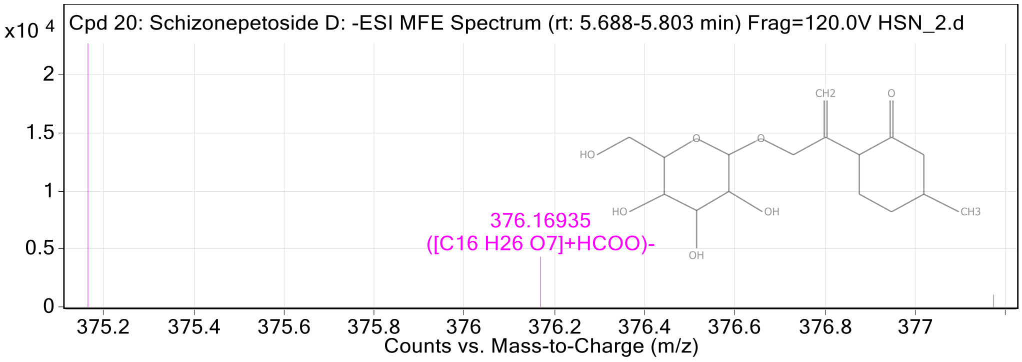

Peak List

m/z	z	Abund	Name	Formula	Ion	Score (DB)	Hits (DB)
375.1655	-1	22706.13	Schizonepetoside D	C16 H26 O7	(M+HCOO)-	97.85	10
376.16935	-1	4300.53		C16 H26 O7	(M+HCOO)-		
377.17423	-1	1050.09		C16 H26 O7	(M+HCOO)-		

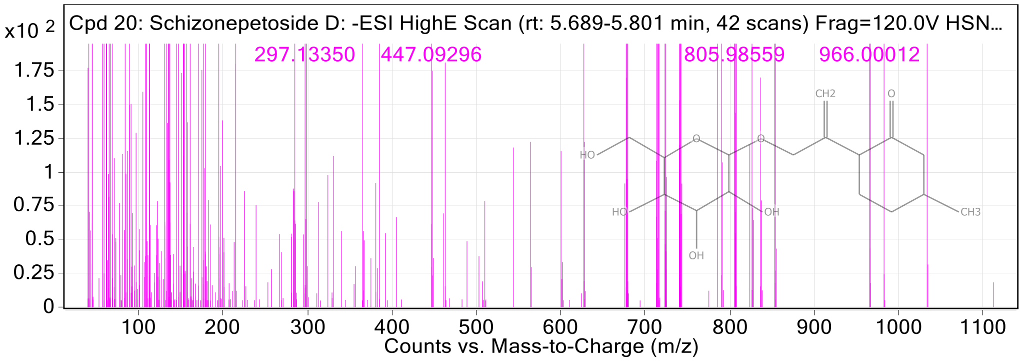

Peak List

m/z	z	Abund
44.99826		4085.78
61.98826		27562.81
62.00449		2623.94
112.98501		5747.67
153.01851	1	4929
677.49669	1	2423.46
740.49221	1	5347.36
741.49534	1	2161.21
805.98559	1	9708.33
806.98824	1	1692.95

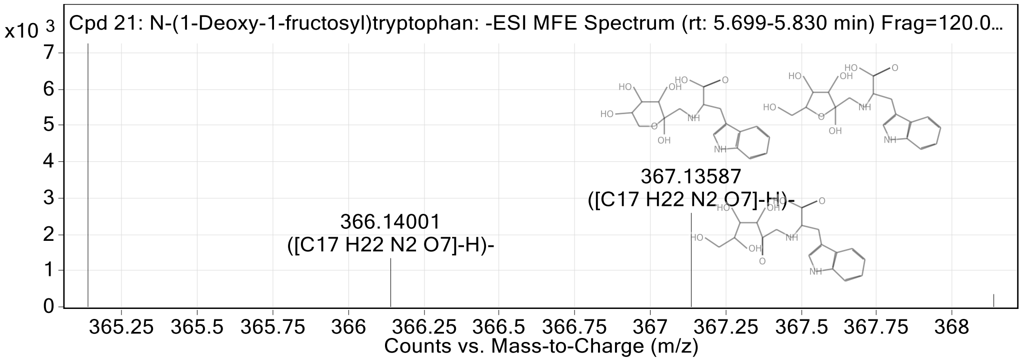

Peak List

m/z	z	Abund	Name	Formula	Ion	Score (DB)	Hits (DB)
365.13692	-1	7257.04	N-(1-Deoxy-1-fructosyl)tryptophan	C17 H22 N2 O7	(M-H)-	62.77	5

366.14001	-1	1342.54		C17 H22 N2 O7	(M-H)-		
367.13587	-1	2589.55		C17 H22 N2 O7	(M-H)-		
368.13819	-1	352.03		C17 H22 N2 O7	(M-H)-		

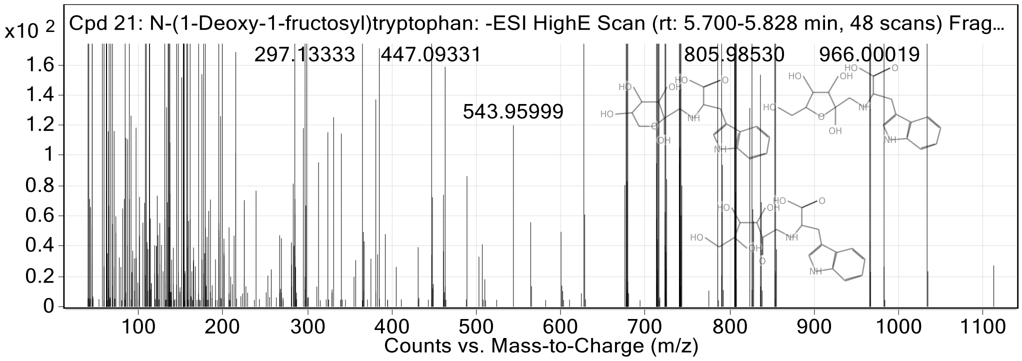

Peak List

m/z	z	Abund
44.9982		3924.9
61.98824		28167.01
62.00436		2705.19
112.98491		5838.06
153.01844	1	4371.54
677.49626	1	2201.18
740.49181	1	5087.04
741.49499	1	2071.06
805.9853	1	9863.01
806.98811	1	1743.78

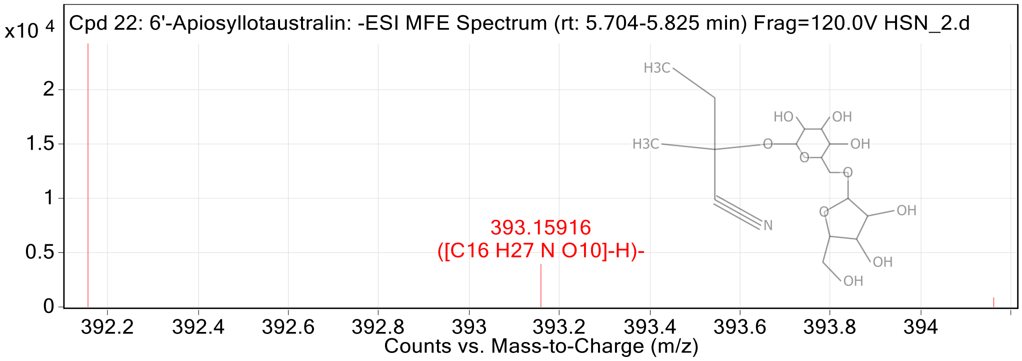

Peak List

m/z	z	Abund	Name	Formula	Ion	Score (DB)	Hits (DB)
392.15546	-1	24204.38	6'-Apiosyllotaustralin	C16 H27 N O10	(M-H)-	97.46	1
393.15916	-1	3944.97		C16 H27 N O10	(M-H)-		
394.16261	-1	879.15		C16 H27 N O10	(M-H)-		

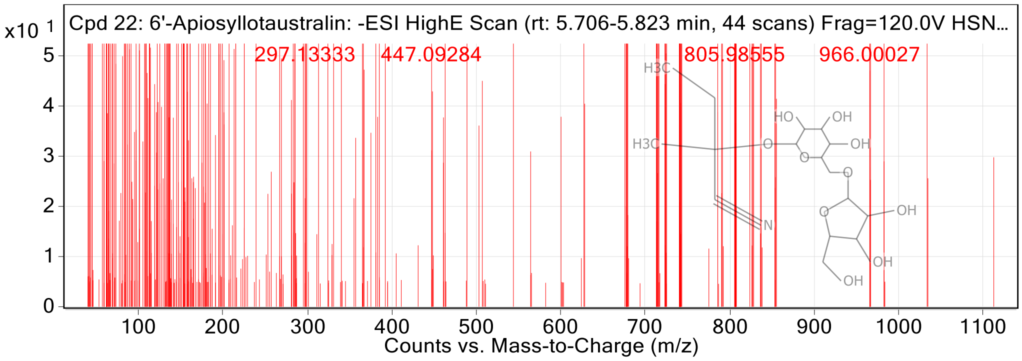

Peak List

m/z	z	Abund
44.99823		4098.68
61.98825		28567.03
62.00436		2772.99
112.98493		5829.17
153.01848	1	4696.25
677.49646	1	2348.55
740.49194	1	5382.33
741.49516	1	2189.2
805.98555	1	9823.28
806.98836	1	1755.94

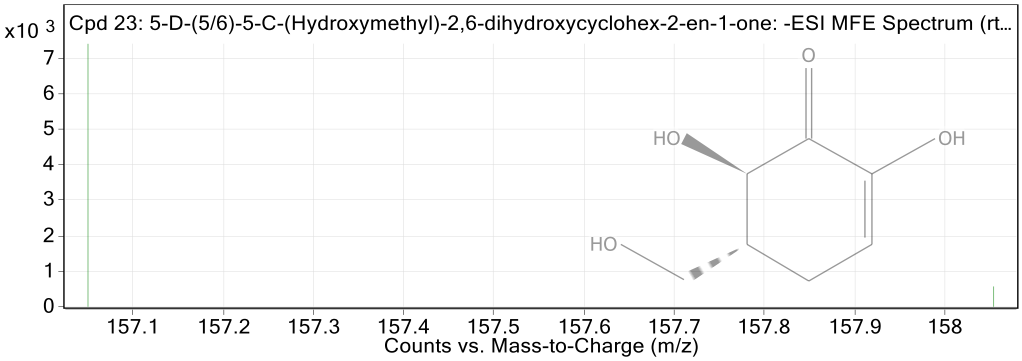

Peak List

m/z	z	Abund	Name	Formula	Ion	Score (DB)	Hits (DB)
157.05013	-1	7410.77	5-D-(5/6)-5-C-(Hydroxymethyl)-2,6-dihydroxycyclohex-2-en-1-one	C7 H10 O4	(M-H)-	85.89	10
158.05416	-1	572.72		C7 H10 O4	(M-H)-		

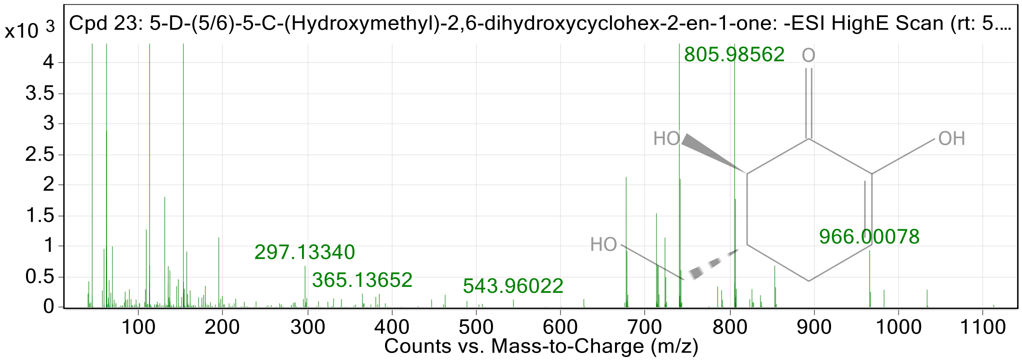

Peak List

m/z	z	Abund
44.99824		4395.18
61.98827		29624.3
62.00442		2885.47
112.98492		5880.17
130.99184		1806.49
153.01847	1	4320.23
677.49647	1	2132.9
740.49194	1	5151.62
741.49524	1	2102.75
805.98562	1	9917.35

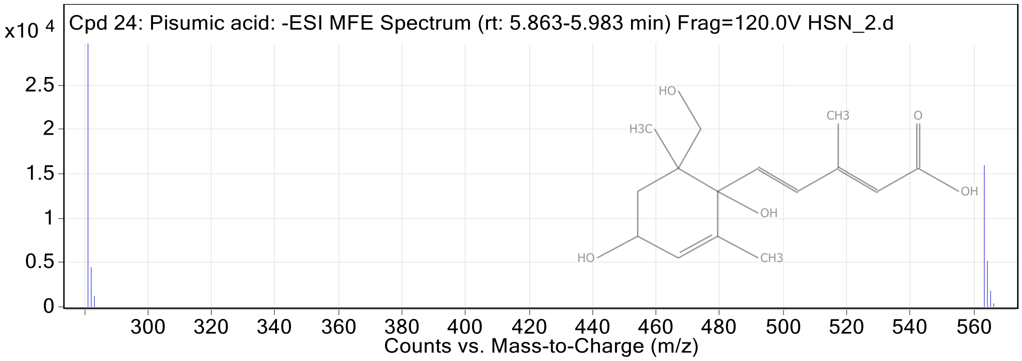

Peak List

m/z	z	Abund	Name	Formula	Ion	Score (DB)	Hits (DB)
281.1388	-1	29685.72	Pisumic acid	C15 H22 O5	(M-H)-	95.15	10
282.14229	-1	4481.31		C15 H22 O5	(M-H)-		
283.14874	-1	1240.28		C15 H22 O5	(M-H)-		
563.28441	-1	15959.72			(2M-H)-		
564.28904	-1	5178.61			(2M-H)-		
565.29218	-1	1834.08			(2M-H)-		
566.29197	-1	393.48			(2M-H)-		

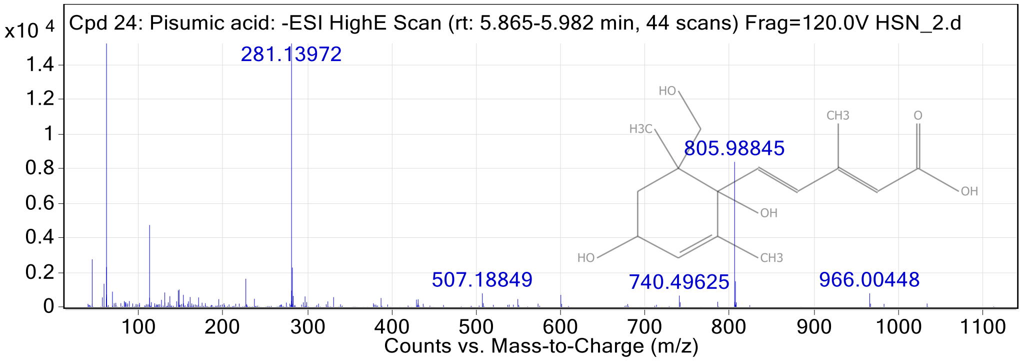

Peak List

m/z	z	Abund
44.99832		2750.24
59.01389		1333.79
61.98846		26178.16
62.00527		2301.56
112.98522		4733.89
227.00167	1	1624.99
281.13972	1	15237.56
282.14305	1	2271.95
805.98845	1	8383.17
806.99117	1	1479.16

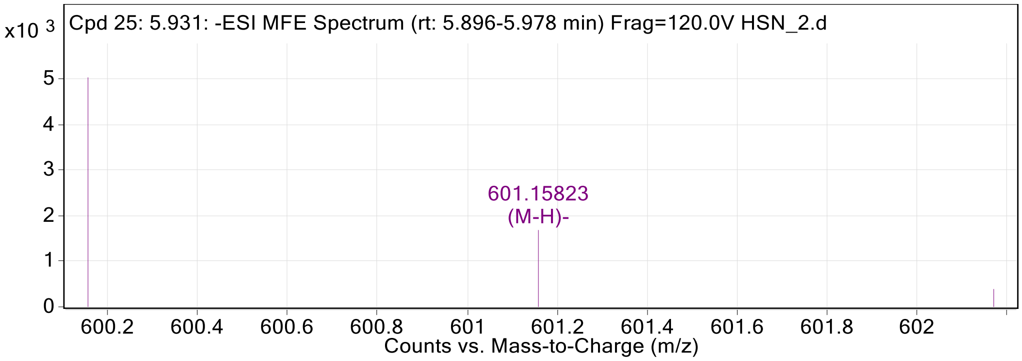

Peak List

m/z	z	Abund	Ion
600.15646	-1	5041.23	(M-H)-
601.15823	-1	1688.33	(M-H)-
602.17108	-1	391.93	(M-H)-

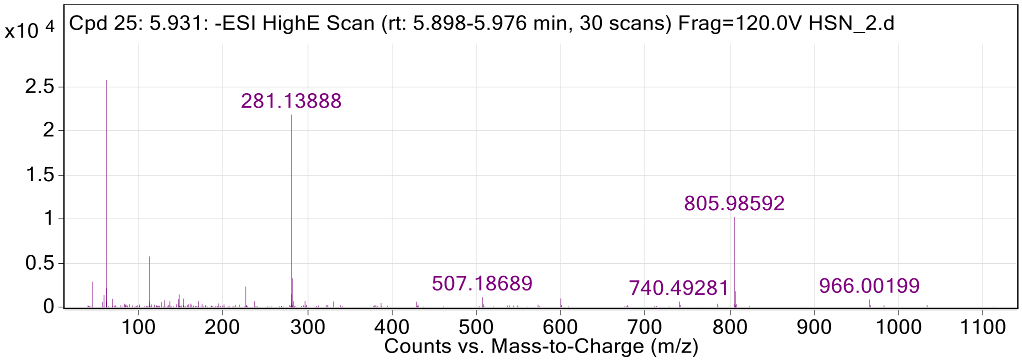

Peak List

m/z	z	Abund
44.99819		2883.95
61.98828		25740.79
62.00512		2115.63
112.98487		5735.5
227.00099	1	2327.64
281.13888	1	21826.66
281.17678	1	1468.03
282.14221	1	3264.26
805.98592	1	10209.43
806.98876	1	1789.01

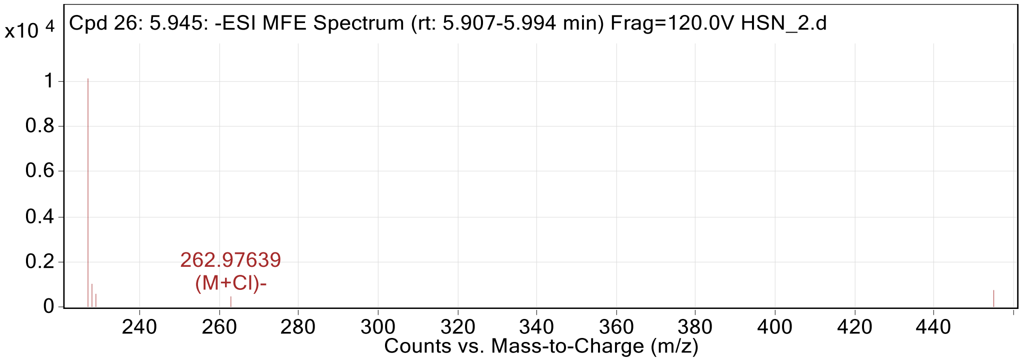

Peak List

m/z	z	Abund	Ion
227.00144	-1	10121	(M-H)-
228.00545	-1	1021.88	(M-H)-

228.9997	-1	579.49	(M-H)-
262.97639	-1	465.42	(M+Cl)-
455.00982	-1	745.23	(2M-H)-

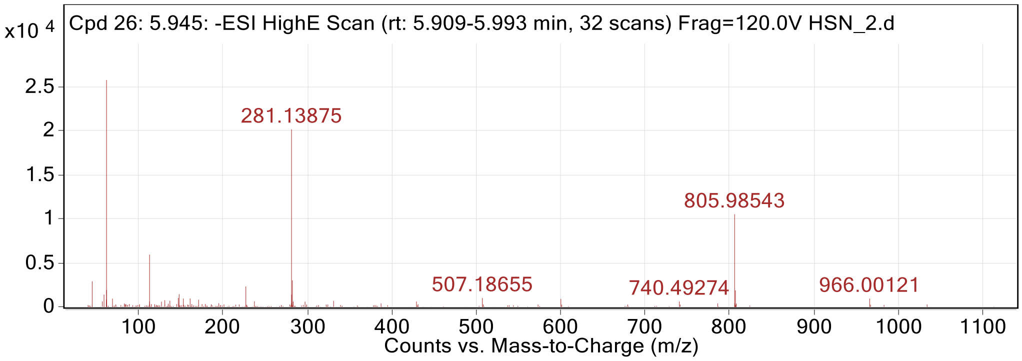

Peak List

m/z	z	Abund
44.99816		2890.33
61.98827		25729.28
62.005		1902.44
112.98491		5915.06
148.01596		1415.79
227.00091	1	2300.45
281.13875	1	20127.75
282.14211	1	2995.58
805.98543	1	10496.58
806.98821	1	1855.76

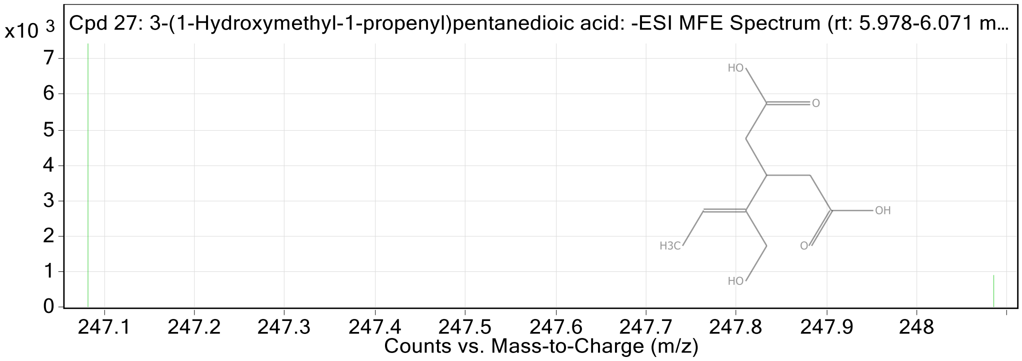

Peak List

m/z	z	Abund	Name	Formula	Ion	Score (DB)	Hits (DB)
247.08201	-1	7433.47	3-(1-Hydroxymethyl-1-propenyl)pentanedioic acid	C9 H14 O5	(M+HCOO)-	85.5	4
248.0852	-1	899.88		C9 H14 O5	(M+HCOO)-		

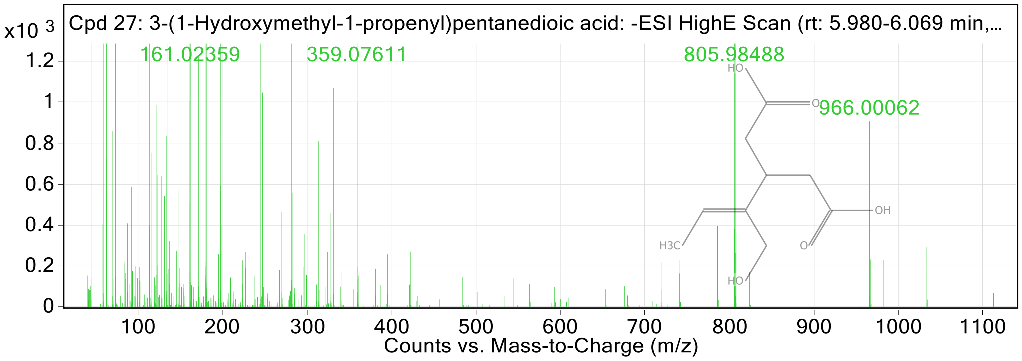

Peak List

m/z	z	Abund
44.99824		2553.33
61.98826		19779.35
112.98509		5954.51
161.02359	1	15721.08
179.03413	1	2265.74
180.98867		2154.34
197.04468	1	4000.9
281.1388	1	3234.93
359.07611	1	5558.28
805.98488	1	10336.32

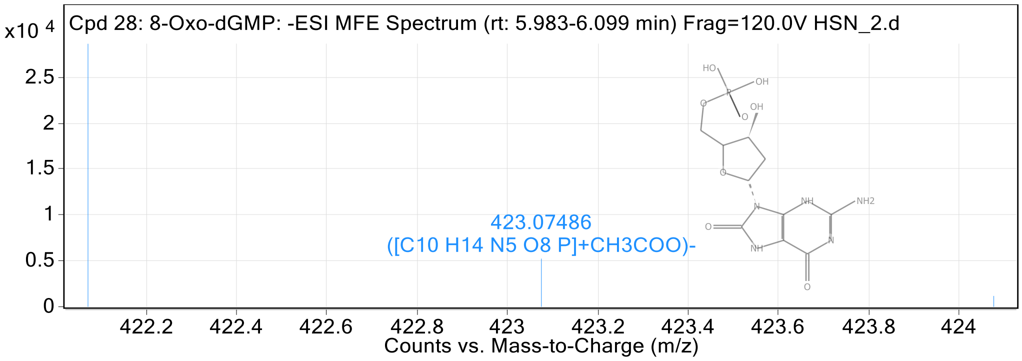

Peak List

m/z	z	Abund	Name	Formula	Ion	Score (DB)	Hits (DB)
422.07091	-1	28658.35	8-Oxo-dGMP	C10 H14 N5 O8 P	(M+CH3COO)-	94.61	4
423.07486	-1	5242.84		C10 H14 N5 O8 P	(M+CH3COO)-		
424.07658	-1	1179.62		C10 H14 N5 O8 P	(M+CH3COO)-		

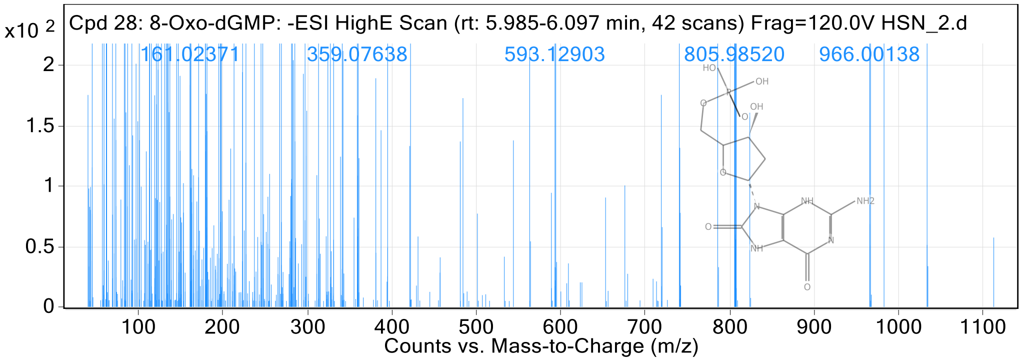

Peak List

m/z	z	Abund
44.99826		2764.65
61.98834		20244.09
112.98514		5911.74
161.02371	1	13184.15
179.03424	1	1986.84
180.98876		2119.06
197.04482	1	3349.73
281.13895	1	2785.07
359.07638	1	4669.39
805.9852	1	10526.72

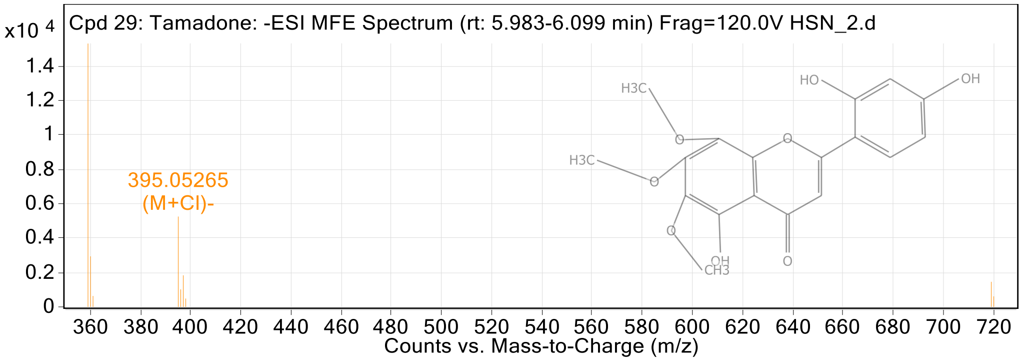

Peak List

m/z	z	Abund	Name	Formula	Ion	Score (DB)	Hits (DB)
359.07628	-1	15323.91	Tamadone	C18 H16 O8	(M-H)-	97.1	10
360.08017	-1	2940.25		C18 H16 O8	(M-H)-		
361.0818	-1	634.75		C18 H16 O8	(M-H)-		
395.05265	-1	5253.5			(M+Cl)-		
396.0566	-1	1018.79			(M+Cl)-		
397.05061	-1	1835.03			(M+Cl)-		
398.05327	-1	484.44			(M+Cl)-		
719.1596	-1	1458.38			(2M-H)-		
720.1625	-1	613.08			(2M-H)-		

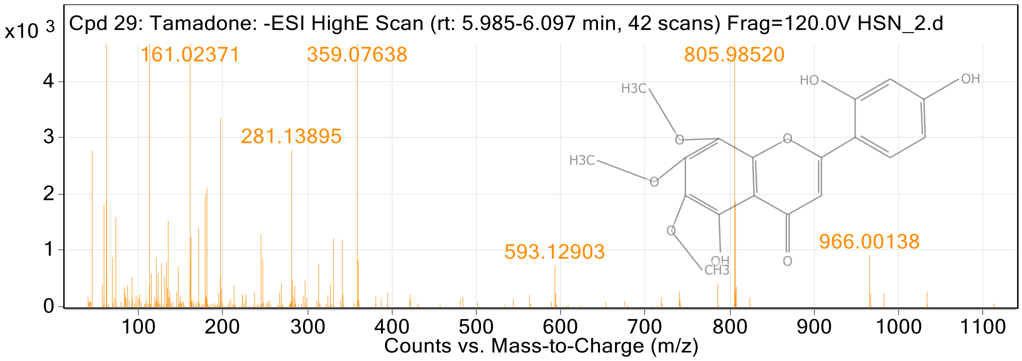

Peak List

m/z	z	Abund
44.99826		2764.65
61.98834		20244.09
112.98514		5911.74
161.02371	1	13184.15
179.03424	1	1986.84
180.98876		2119.06
197.04482	1	3349.73
281.13895	1	2785.07
359.07638	1	4669.39
805.9852	1	10526.72

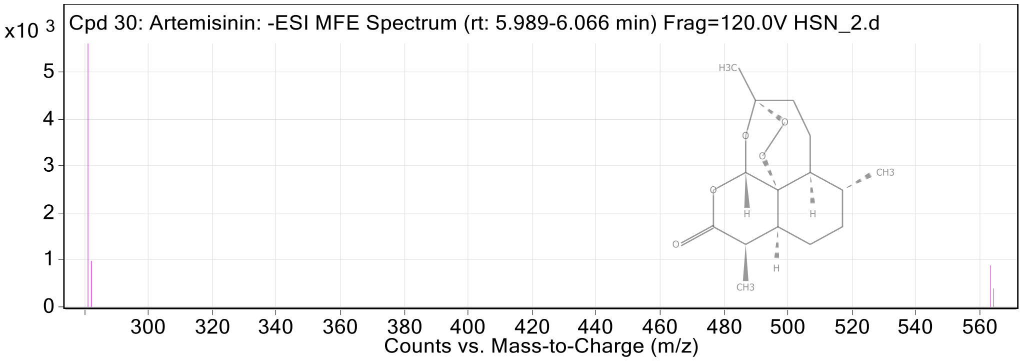

Peak List

m/z	z	Abund	Name	Formula	Ion	Score (DB)	Hits (DB)
281.13814	-1	5615.59	Artemisinin	C15 H22 O5	(M-H)-	80.09	10
282.1418	-1	978.71		C15 H22 O5	(M-H)-		
563.28188	-1	883.24			(2M-H)-		
564.28635	-1	390.67			(2M-H)-		

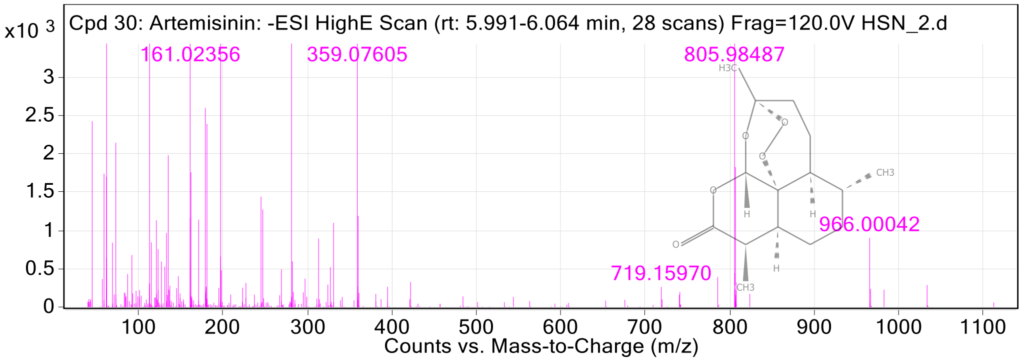

Peak List

m/z	z	Abund
44.99825		2427.65
61.98824		18268.75
112.9851		5918.33
161.02356	1	18517.62
179.03406	1	2602.44
180.98861		2391.64
197.04464	1	4712.54
281.1388	1	3442.17
359.07605	1	6563.01
805.98487	1	10203.35

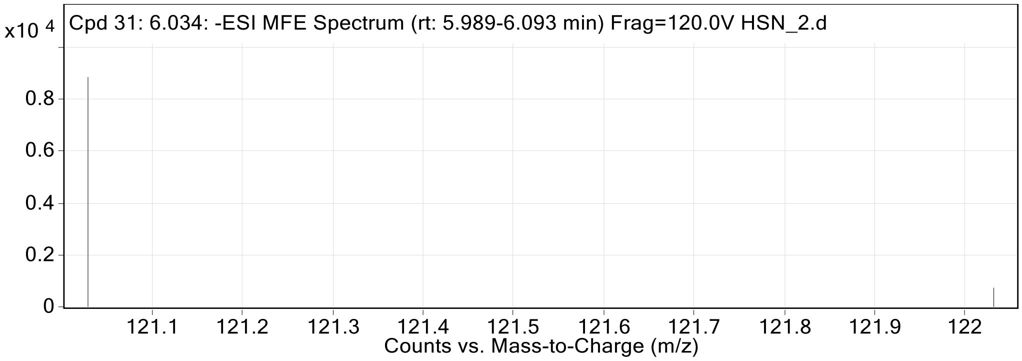

Peak List

m/z	z	Abund	Ion
121.02883	-1	8856.65	(M-H)-

122.03177	-1	737.66	(M-H)-

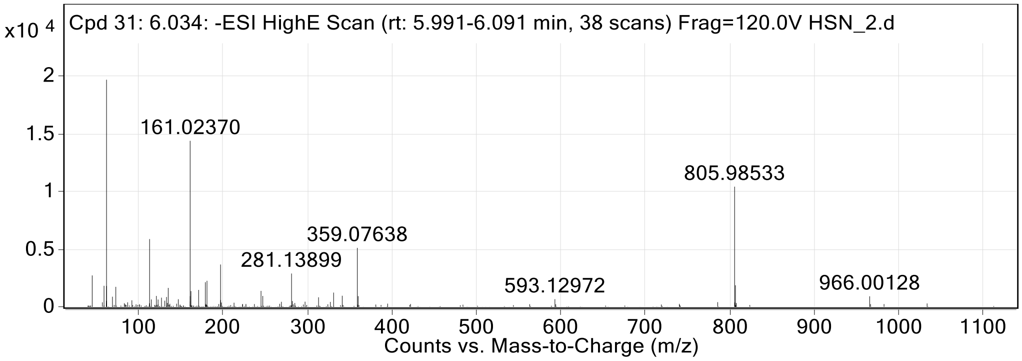

Peak List

m/z	z	Abund
44.99827		2717.49
61.98833		19692.15
112.98518		5871.42
161.0237	1	14394.45
179.03424	1	2132.75
180.98878		2251.75
197.0448	1	3664.31
281.13899	1	2882.31
359.07638	1	5107.88
805.98533	1	10416.26

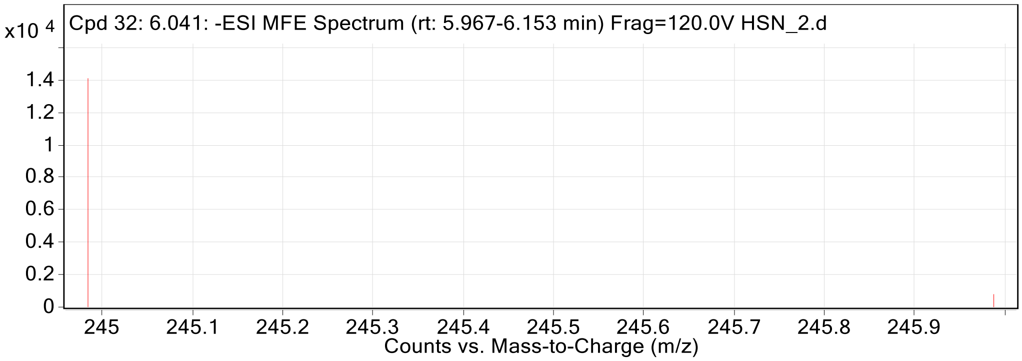

Peak List

m/z	z	Abund	Ion
244.98442	-1	14104.46	(M-H)-
245.98784	-1	795.24	(M-H)-

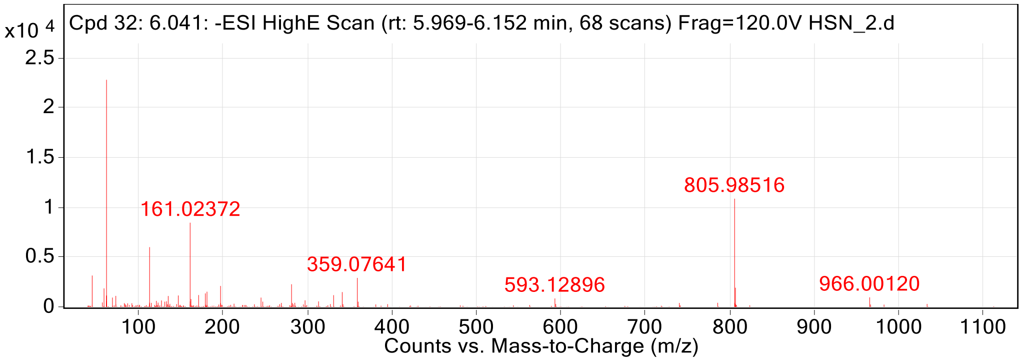

Peak List

m/z	z	Abund
44.99824		3146.05
59.01393		1854.62

61.98832		22806.02
112.985		5996.96
161.02372	1	8454.74
197.04485	1	2098.44
281.13891	1	2270.47
359.07641	1	2907.69
805.98516	1	10863.68
806.98764	1	1920.4

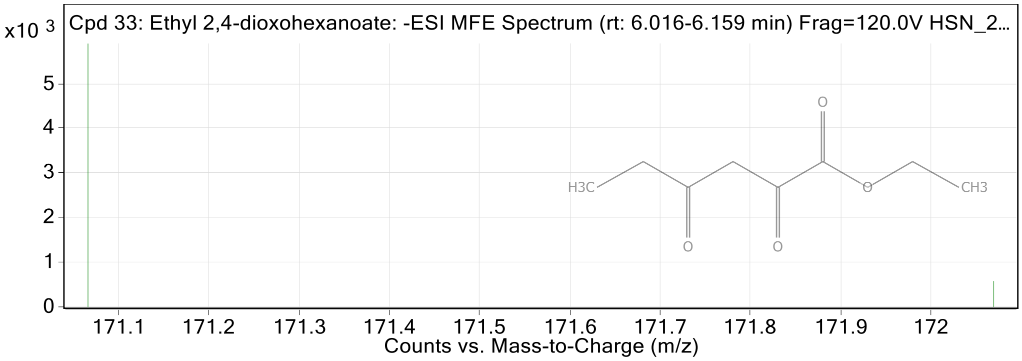

Peak List

m/z	z	Abund	Name	Formula	Ion	Score (DB)	Hits (DB)
171.06595	-1	5898.37	Ethyl 2,4-dioxohexanoate	C8 H12 O4	(M-H)-	86.45	10
172.06969	-1	578.97		C8 H12 O4	(M-H)-		

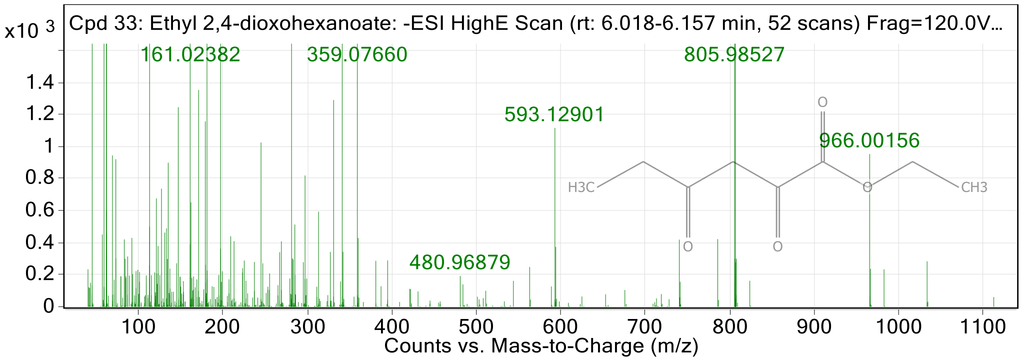

Peak List

m/z	z	Abund
44.99824		3425.89
59.01389		1916.57
61.9883		23151.16
62.00473		2319.87
112.98497		5875.4
161.02382	1	6790.54
341.15951	1	1918.17
359.0766	1	2380.66
805.98527	1	10893.12
806.98779	1	1928.42

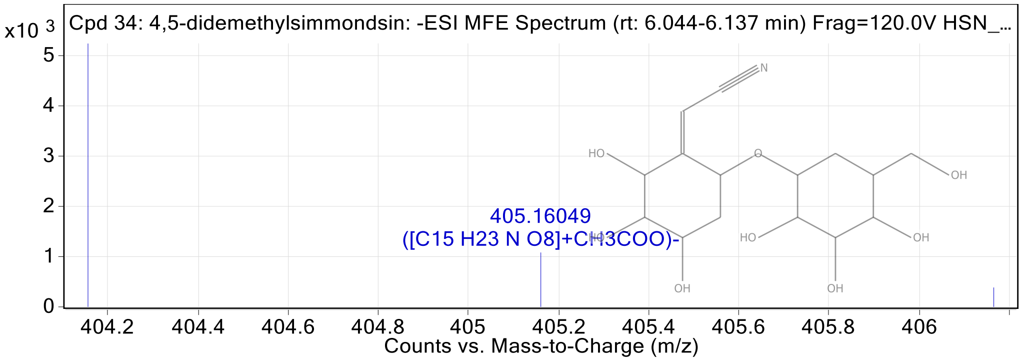

Peak List

m/z	z	Abund	Name	Formula	Ion	Score (DB)	Hits (DB)
404.1556	-1	5229.92	4,5-didemethylsimmondsin	C15 H23 N O8	(M+CH3COO)-	89.79	10
405.16049	-1	1079.84		C15 H23 N O8	(M+CH3COO)-		
406.16564	-1	385.01		C15 H23 N O8	(M+CH3COO)-		

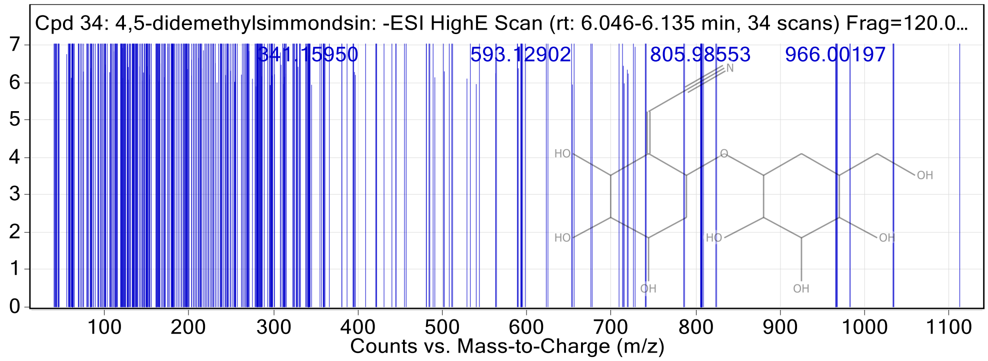

Peak List

m/z	z	Abund
44.99822		3338.35
59.0139		1927.54
61.98829		24057.2
62.00466		2408.85
112.9849		5962.18
161.02391	1	3582.14
171.06538		1672.35
341.1595	1	2758.83
805.98553	1	11043.96
806.9879	1	1945.92

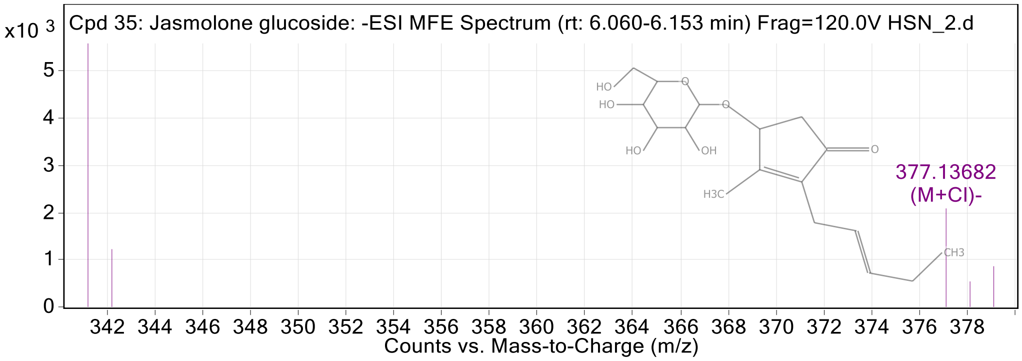

Peak List

m/z	z	Abund	Name	Formula	Ion	Score (DB)	Hits (DB)
341.15991	-1	5588.07	Jasmolone glucoside	C17 H26 O7	(M-H)-	79.73	1
342.16263	-1	1224.78		C17 H26 O7	(M-H)-		

377.13682	-1	2088.58			(M+Cl)-		
378.14087	-1	540.07			(M+Cl)-		
379.13577	-1	862.91			(M+Cl)-		

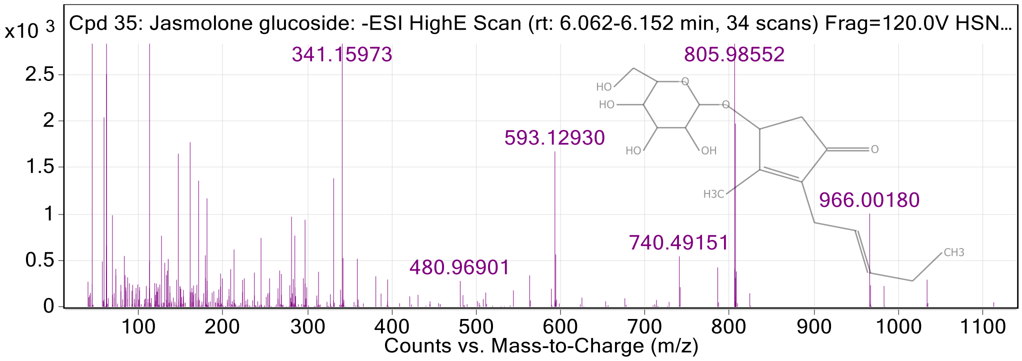

Peak List

m/z	z	Abund
44.99829		3806.8
59.01403		2041.66
61.9884		25290.21
62.00514		2507.74
112.98499		5988.36
161.02386		1774.22
341.15973	1	2837.14
593.1293	1	1675.16
805.98552	1	11281.59
806.98752	1	1974.46

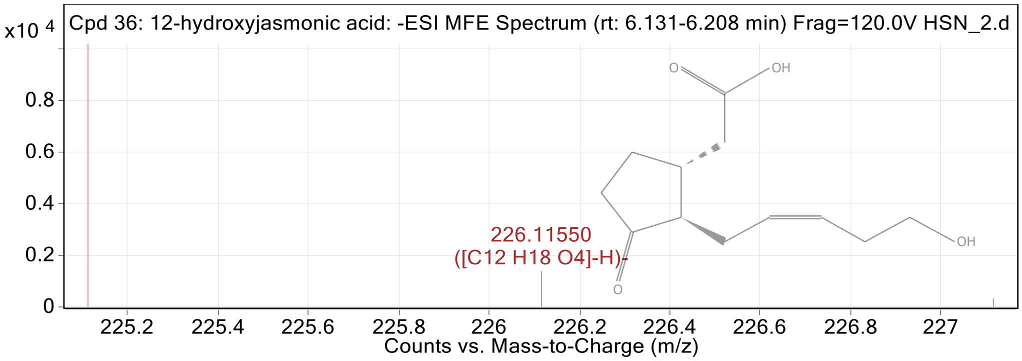

Peak List

m/z	z	Abund	Name	Formula	Ion	Score (DB)	Hits (DB)
225.11248	-1	10204.81	12-hydroxyjasmonic acid	C12 H18 O4	(M-H)-	95.56	10
226.1155	-1	1388.62		C12 H18 O4	(M-H)-		
227.11652	-1	331.82		C12 H18 O4	(M-H)-		

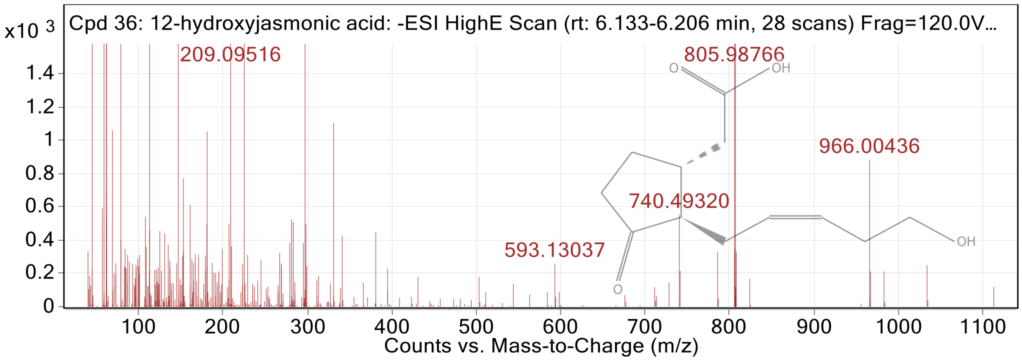

Peak List

m/z	z	Abund
44.99838		5061.74
59.01405		1948.18
61.98851		26300.79
62.00547		2471.64
78.95913		1842.25
112.9853		5539.97
146.96589		1854.22
209.09516	1	3797.93
297.13462	1	3340.7
805.98766	1	10382.95

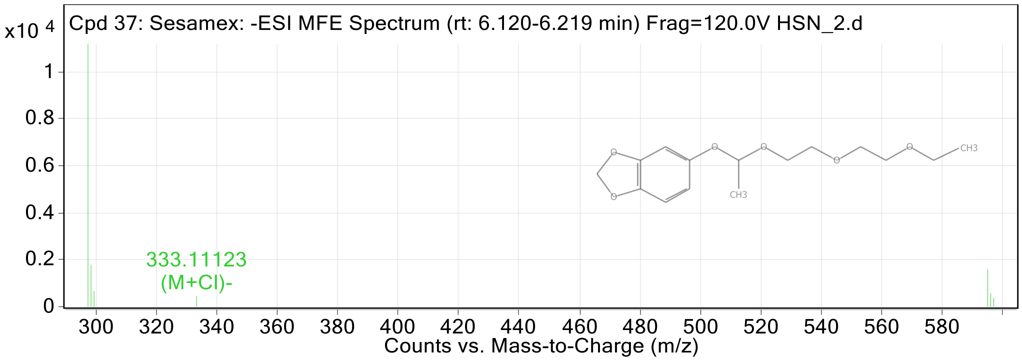

Peak List

m/z	z	Abund	Name	Formula	Ion	Score (DB)	Hits (DB)
297.13429	-1	11191.73	Sesamex	C15 H22 O6	(M-H)-	87.63	5
298.13899	-1	1785.67		C15 H22 O6	(M-H)-		
299.14595	-1	669.84		C15 H22 O6	(M-H)-		
333.11123	-1	458.84			(M+Cl)-		
595.27689	-1	1588.25			(2M-H)-		
596.2781	-1	576.93			(2M-H)-		
597.2807	-1	382.83			(2M-H)-		

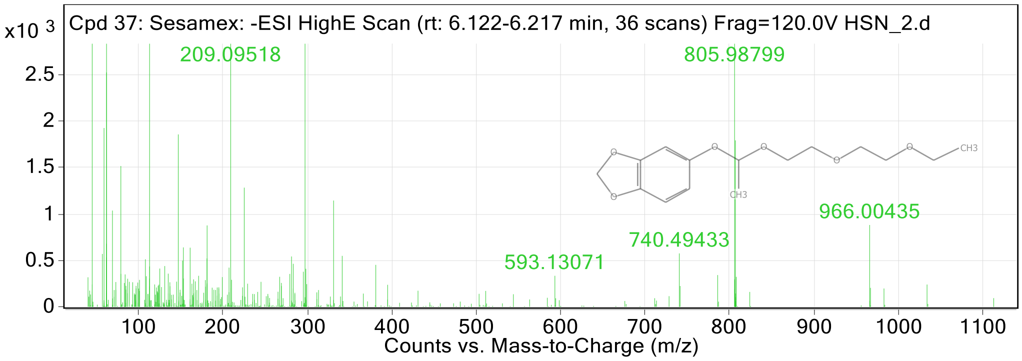

Peak List

m/z	z	Abund
44.99835		4712.35
59.01403		1927.77
61.98851		26008.24
62.00537		2526.95
112.98532		5418.9
146.9659		1858.66
209.09518	1	3140.6
297.13465	1	2837.72
805.98799	1	10054.3
806.99074	1	1794.32

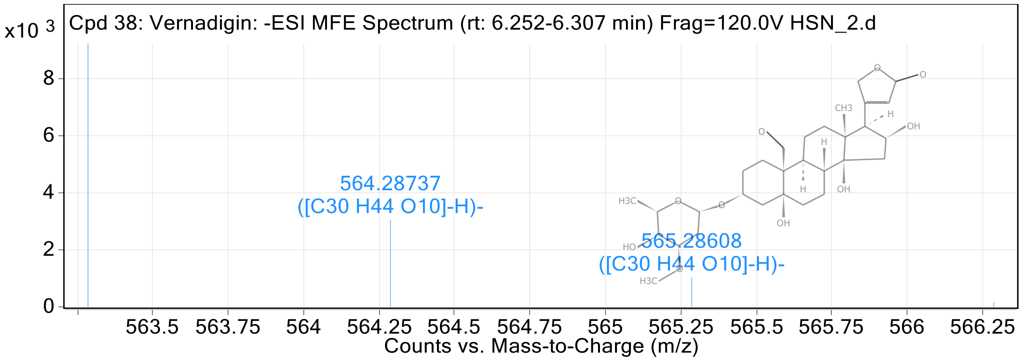

Peak List

m/z	z	Abund	Name	Formula	Ion	Score (DB)	Hits (DB)
563.28462	-1	9245.15	Vernadigin	C30 H44 O10	(M-H)-	84	8
564.28737	-1	3054.84		C30 H44 O10	(M-H)-		
565.28608	-1	1037.04		C30 H44 O10	(M-H)-		
566.28739	-1	161.77		C30 H44 O10	(M-H)-		

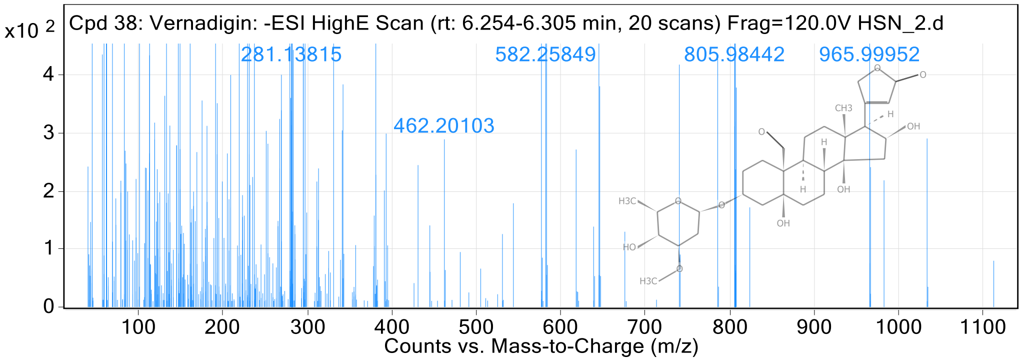

Peak List

m/z	z	Abund
44.99798		3381.26
59.01371		1887.21
61.98822		21039.42
62.0039		1987.19
112.98471		6318
230.98519	1	4130.1
281.13815	1	13178.67
282.14199	1	2045.53
805.98442	1	11318.76
806.98764	1	1964.14

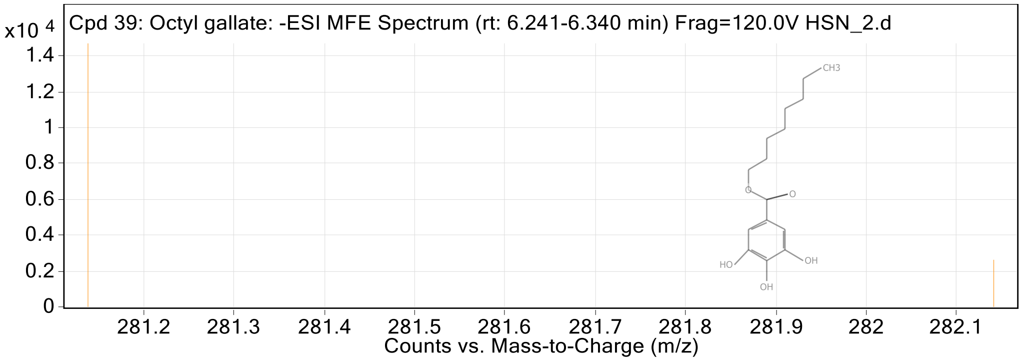

Peak List

m/z	z	Abund	Name	Formula	Ion	Score (DB)	Hits (DB)
281.13833	-1	14699.01	Octyl gallate	C15 H22 O5	(M-H)-	80.01	10

Qualitative Analysis Report

282.14119	-1	2624.77		C15 H22 O5	(M-H)-		

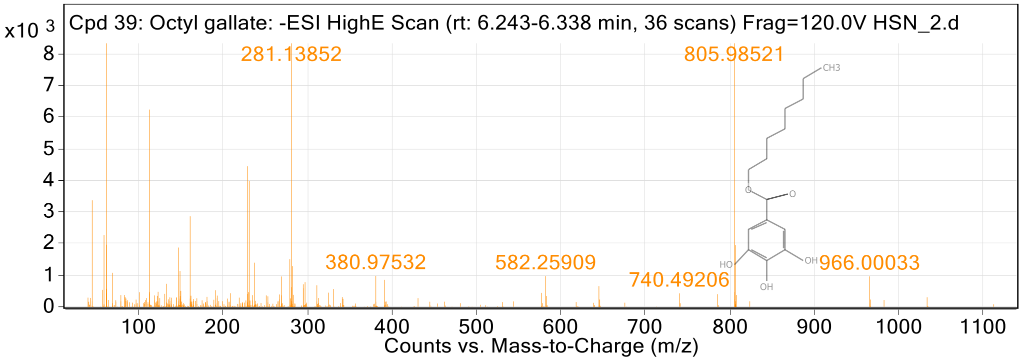

Peak List

m/z	z	Abund
44.99818		3367.64
59.01383		2270.7
61.98832		22262.88
62.00415		1964.02
112.98495		6242.09
161.02365	1	2861.73
229.01668	1	4444.99
230.98557		3976.69
281.13852	1	8338.61
805.98521	1	11161.85

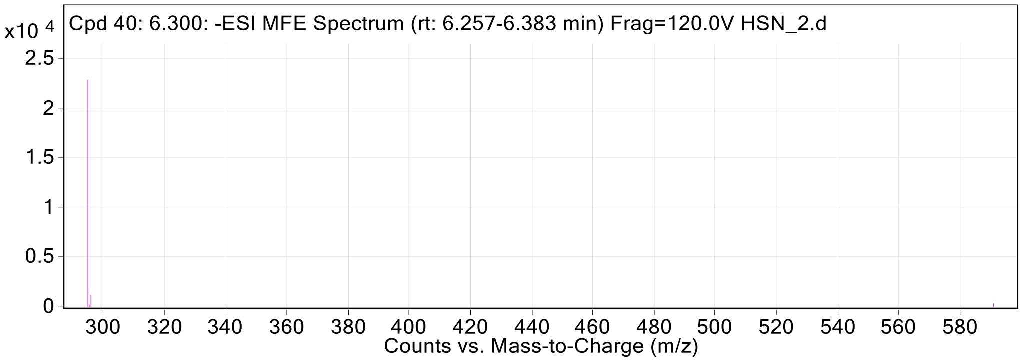

Peak List

m/z	z	Abund	Ion
294.98137	-2	22859.62	(M-2H)-2
295.47709	-2	231.28	(M-2H)-2
295.98505	-2	1225.69	(M-2H)-2
590.97259	-1	330.81	(M-H)-

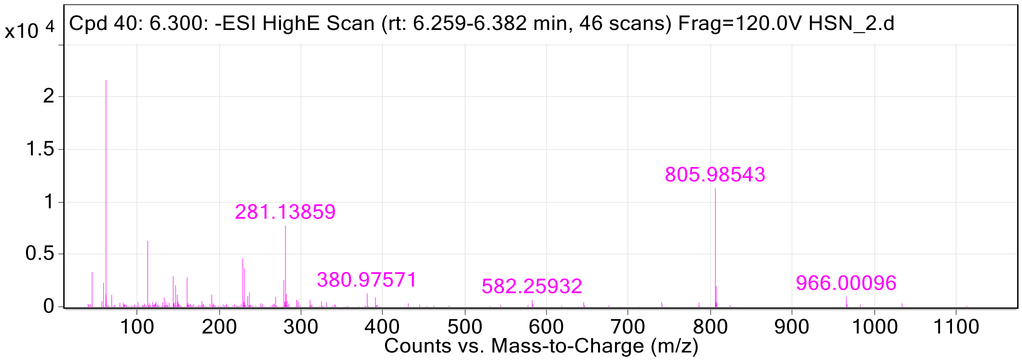

Peak List

m/z	z	Abund

44.99824		3321.22
61.98832		21633.95
112.98494		6286.79
144.04482	1	2916.76
161.02368	1	2799.97
229.01682	1	4621.05
230.98565		3675.34
279.1232	1	2553.96
281.13859	1	7759.14
805.98543	1	11321.57

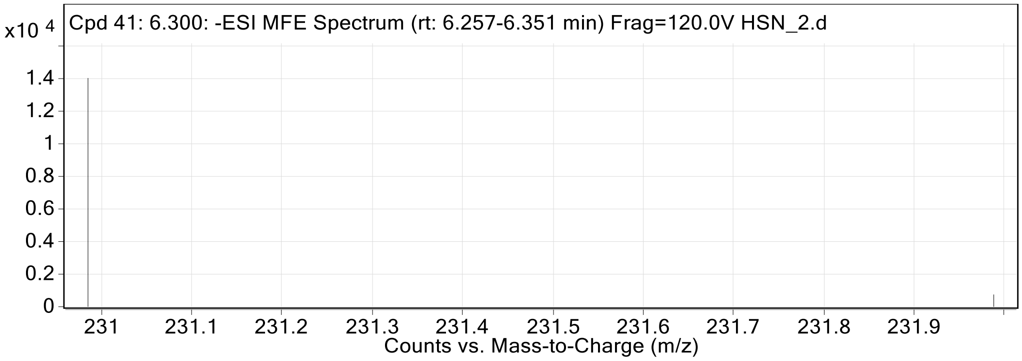

Peak List

m/z	z	Abund	Ion
230.98529	-1	14034.41	(M-H)-
231.98818	-1	751.63	(M-H)-

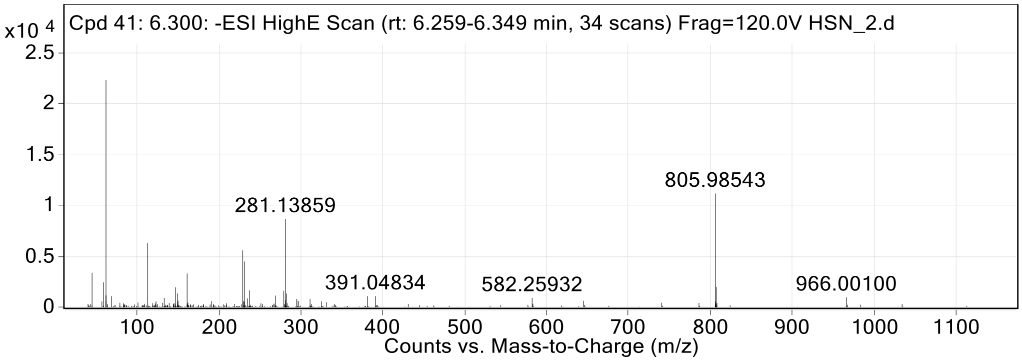

Peak List

m/z	z	Abund
44.99822		3368.41
59.01387		2428.34
61.98836		22313.11
112.98497		6285.4
161.02373	1	3292.19
229.01681	1	5563.04
230.98571		4471.35
281.13859	1	8659.72
805.98543	1	11155.74
806.9883	1	1972.96

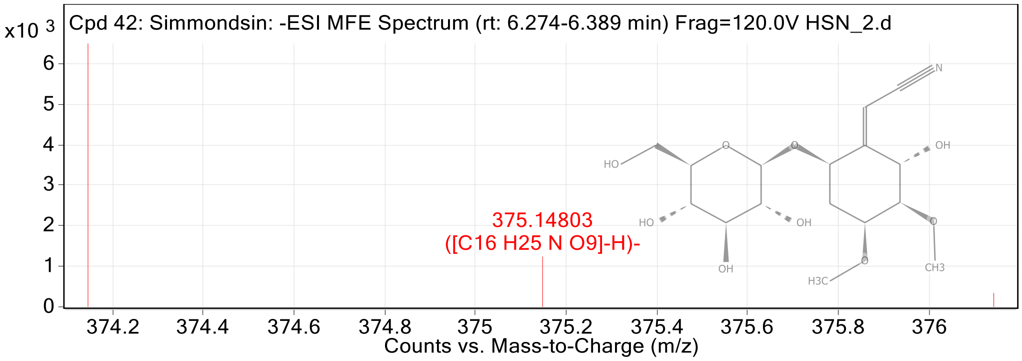

Peak List

m/z	z	Abund	Name	Formula	Ion	Score (DB)	Hits (DB)
374.1452	-1	6494.21	Simmondsin	C16 H25 N O9	(M-H)-	91.49	10
375.14803	-1	1240.5		C16 H25 N O9	(M-H)-		
376.14338	-1	345.44		C16 H25 N O9	(M-H)-		

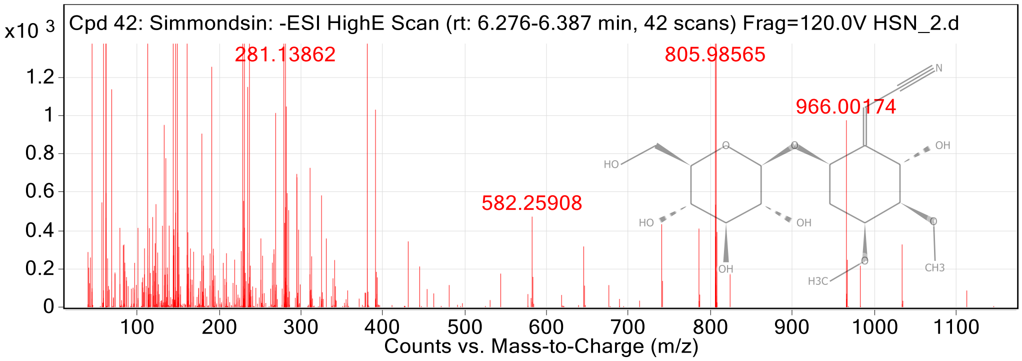

Peak List

m/z	z	Abund
44.99829		3322.53
61.98833		21629.5
112.98497		6377.72
144.04487	1	3528
161.02369	1	3044.19
229.01688	1	5151.99
230.98573		3969.42
279.1233	1	2530.05
281.13862	1	6575.36
805.98565	1	11438.07

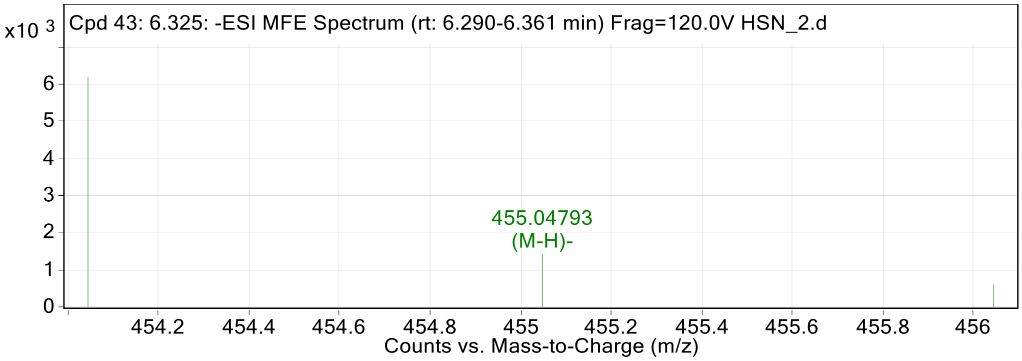

Peak List

m/z	z	Abund	Ion
454.04445	-1	6206	(M-H)-
455.04793	-1	1417.18	(M-H)-

456.04493	-1	608.33	(M-H)-

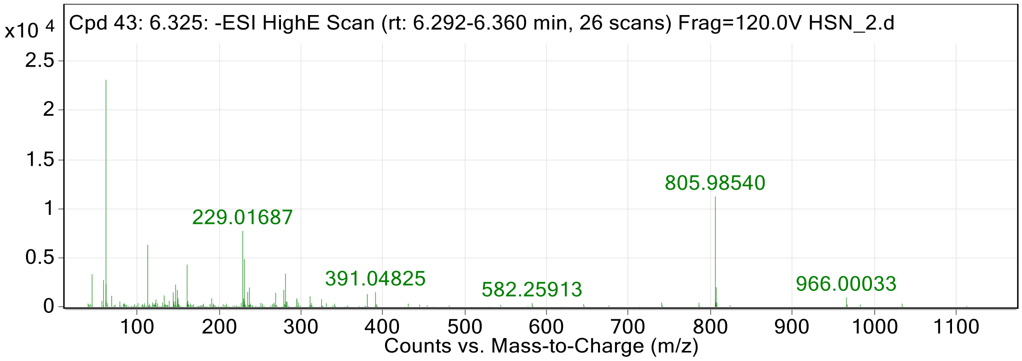

Peak List

m/z	z	Abund
44.99833		3318.17
59.01391		2721.21
61.98838		23126.65
62.00453		2285.3
112.9851		6320.1
161.02376	1	4294.22
229.01687	1	7729.92
230.98569		4852.62
281.13886	1	3367.98
805.9854	1	11230.03

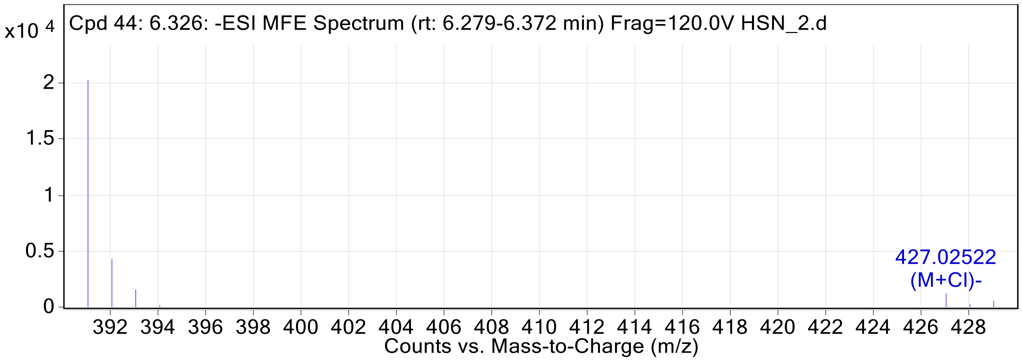

Peak List

m/z	z	Abund	Ion
391.04917	-1	20301.75	(M-H)-
392.05294	-1	4299.7	(M-H)-
393.05195	-1	1562.7	(M-H)-
394.05955	-1	174.45	(M-H)-
427.02522	-1	1209.04	(M+Cl)-
428.02781	-1	297.23	(M+Cl)-
429.02382	-1	557.12	(M+Cl)-

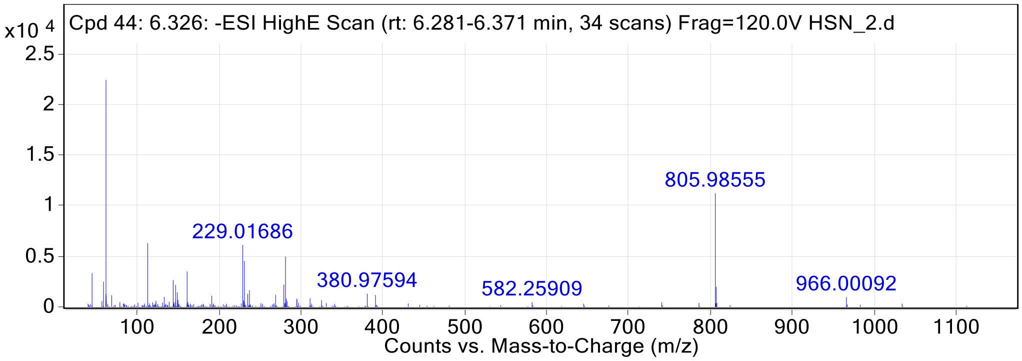

Peak List

m/z	z	Abund
44.99832		3336.28
59.0139		2490.95
61.98837		22439.04
112.98507		6290.32
144.04488	1	2645.16
161.0237	1	3497.78
229.01686	1	6116.41
230.98568		4538.27
281.13866	1	4961.37
805.98555	1	11223.49

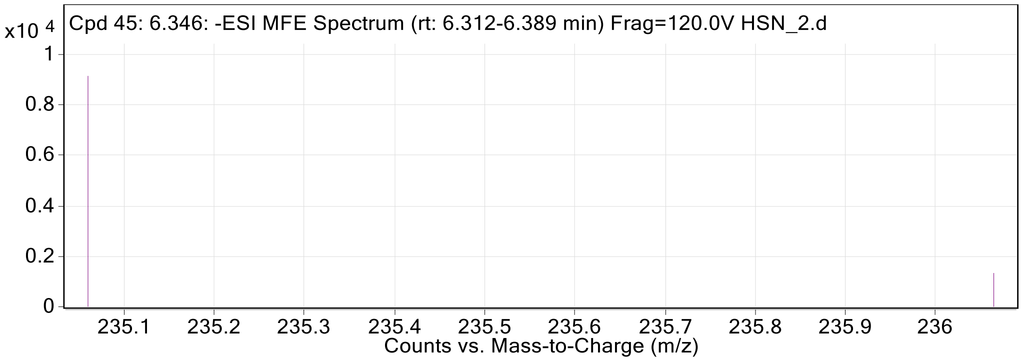

Peak List

m/z	z	Abund	Ion
235.05997	-1	9142.61	(M-H)-
236.06445	-1	1336.91	(M-H)-

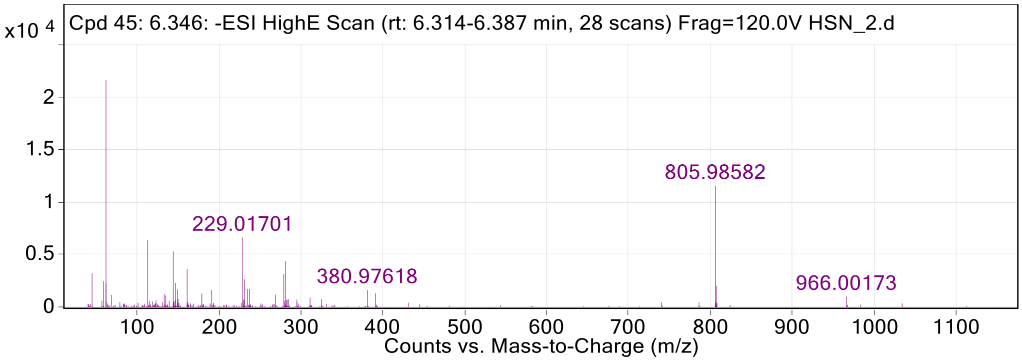

Peak List

m/z	z	Abund
44.99842		3214
61.98836		21653.67
112.9851		6374.57

144.04497	1	5292
161.02375	1	3631.77
229.01701	1	6628.33
230.98589		2637.03
279.12339	1	3160.12
281.13875	1	4378.72
805.98582	1	11567.45

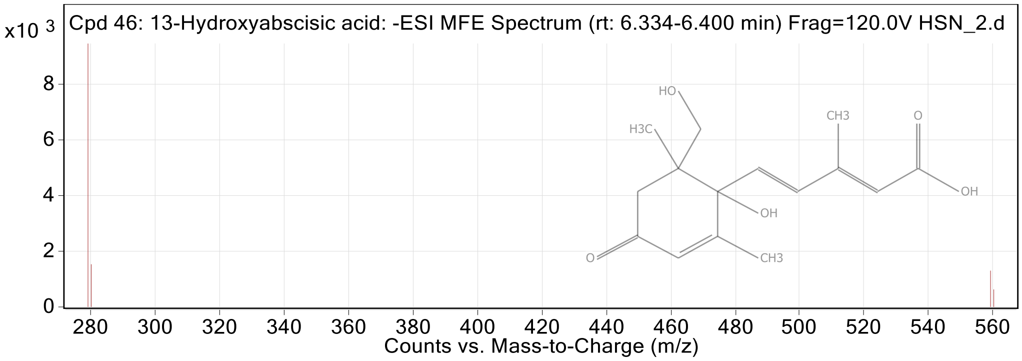

Peak List

m/z	z	Abund	Name	Formula	Ion	Score (DB)	Hits (DB)
279.1232	-1	9477.51	13-Hydroxyabscisic acid	C15 H20 O5	(M-H)-	84.87	10
280.12656	-1	1534.95		C15 H20 O5	(M-H)-		
559.25222	-1	1306.06			(2M-H)-		
560.25891	-1	629.89			(2M-H)-		

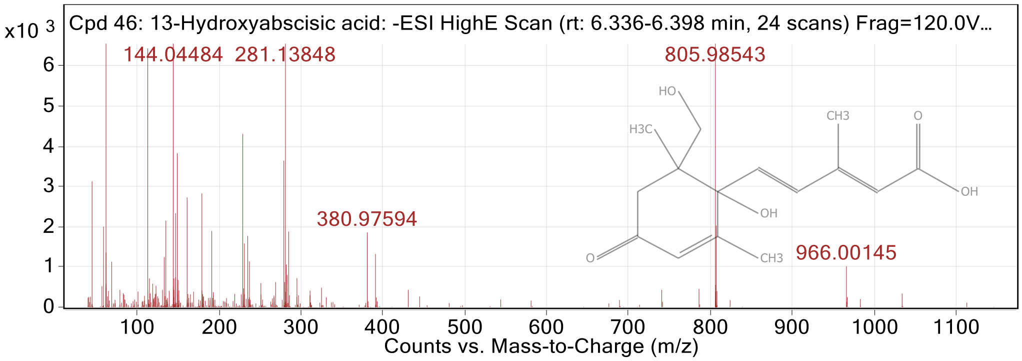

Peak List

m/z	z	Abund
44.99827		3126.35
61.98825		19260.25
112.98489		6428.96
144.04484	1	6795.89
149.02379	1	3825.25
179.07055	1	2824.98
229.01707	1	4306.96
279.12314	1	3638.59
281.13848	1	6549.9
805.98543	1	11824.49

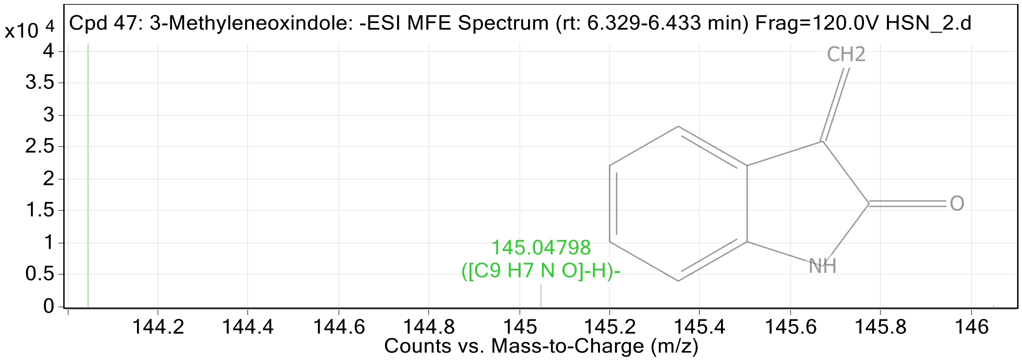

Peak List

m/z	z	Abund	Name	Formula	Ion	Score (DB)	Hits (DB)
144.04487	-1	41175.18	3-Methyleneoxindole	C9 H7 N O	(M-H)-	96.97	9
145.04798	-1	3578.97		C9 H7 N O	(M-H)-		
146.05078	-1	300.14		C9 H7 N O	(M-H)-		

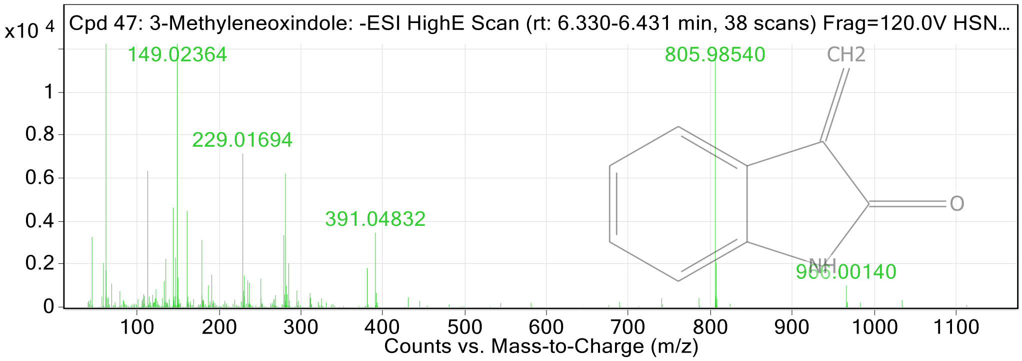

Peak List

m/z	z	Abund
61.98827		17209.55
112.98496		6344.52
144.04488	1	4627.44
149.02364	1	12262.02
161.02365	1	4472.47
229.01694	1	7150.3
279.12324	1	3351.22
281.13861	1	6215.84
391.04832	1	3466
805.9854	1	11617

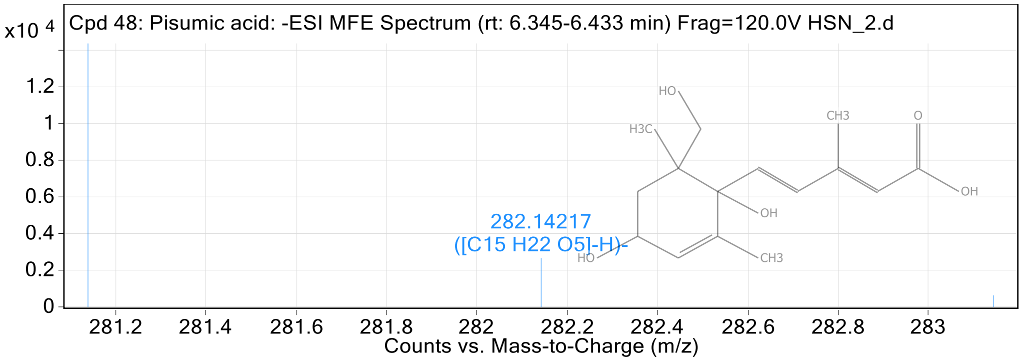

Peak List

m/z	z	Abund	Name	Formula	Ion	Score (DB)	Hits (DB)
281.13892	-1	14371.25	Pisumic acid	C15 H22 O5	(M-H)-	96.88	10
282.14217	-1	2667.45		C15 H22 O5	(M-H)-		

283.14433	-1	628.39		C15 H22 O5	(M-H)-		

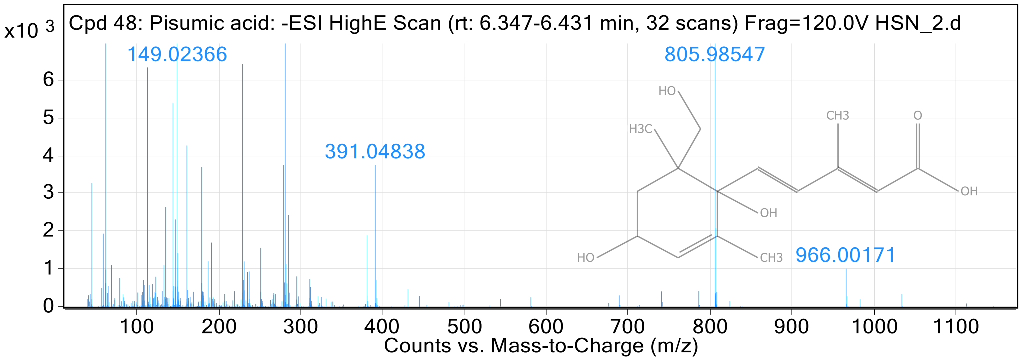

Peak List

m/z	z	Abund
61.98828		16005.22
112.98497		6329.62
144.04489	1	5395.67
149.02366	1	14234.71
161.0237	1	4262.89
229.01698	1	6416.98
279.1233	1	3742.11
281.13866	1	6963
391.04838	1	3744.76
805.98547	1	11680.69

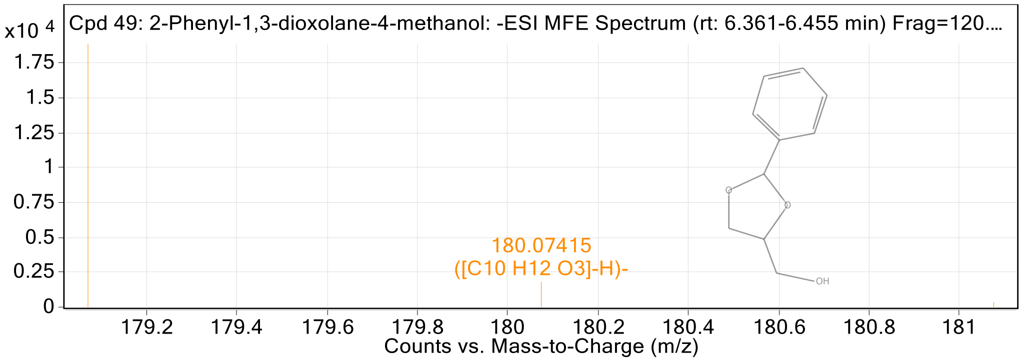

Peak List

m/z	z	Abund	Name	Formula	Ion	Score (DB)	Hits (DB)
179.07077	-1	18907.25	2-Phenyl-1,3-dioxolane-4-methanol	C10 H12 O3	(M-H)-	97.1	10
180.07415	-1	1810.87		C10 H12 O3	(M-H)-		
181.07591	-1	363.27		C10 H12 O3	(M-H)-		

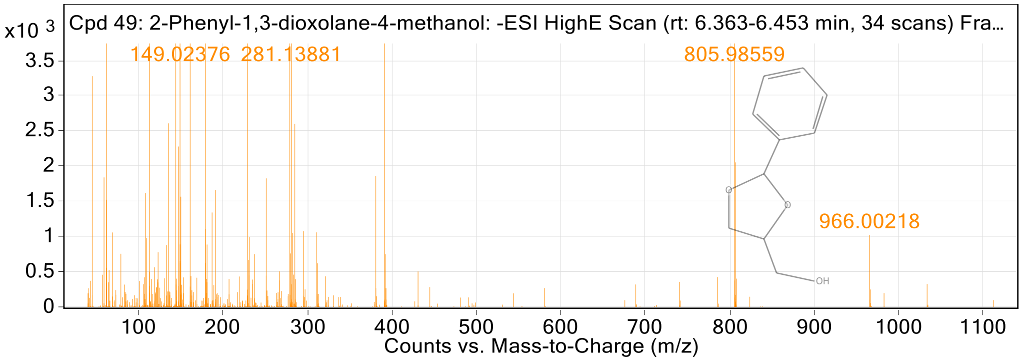

Peak List

m/z	z	Abund

61.9884		15225.94
112.98503		6434.28
144.04494	1	4157.21
149.02376	1	15608.29
161.02383	1	4372.62
229.01708	1	6269.32
279.12334	1	4577.03
281.13881	1	6485.15
391.04857	1	3958.13
805.98559	1	11925.95

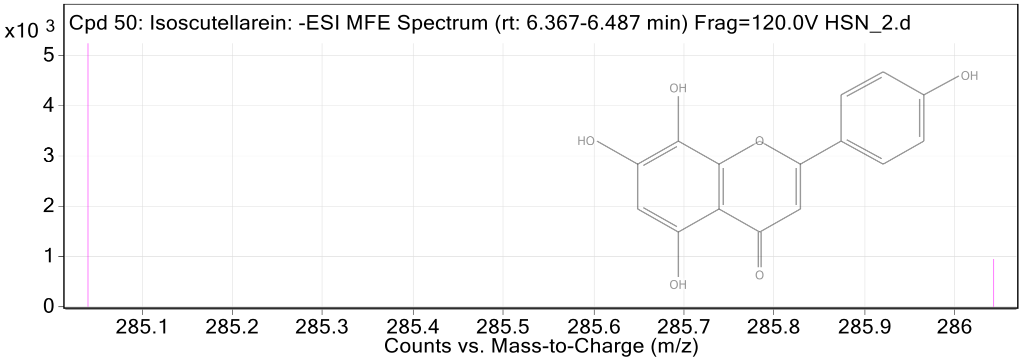

Peak List

m/z	z	Abund	Name	Formula	Ion	Score (DB)	Hits (DB)
285.04028	-1	5230.8	Isoscutellarein	C15 H10 O6	(M-H)-	84.04	10
286.04306	-1	950.64		C15 H10 O6	(M-H)-		

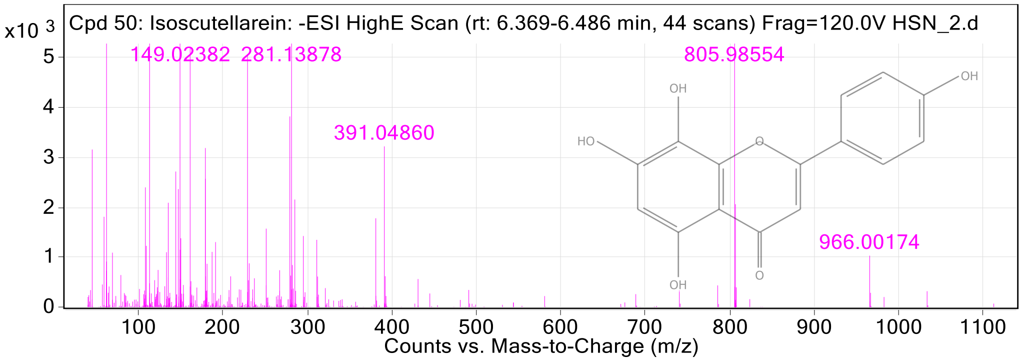

Peak List

m/z	z	Abund
61.98843		16066.32
112.98509		6446.51
149.02382	1	12872.11
161.02389	1	5189.05
179.07028	1	3189.08
229.01708	1	5067.13
279.1234	1	3822.78
281.13878	1	5284.76
391.0486	1	3222.38
805.98554	1	12032.89

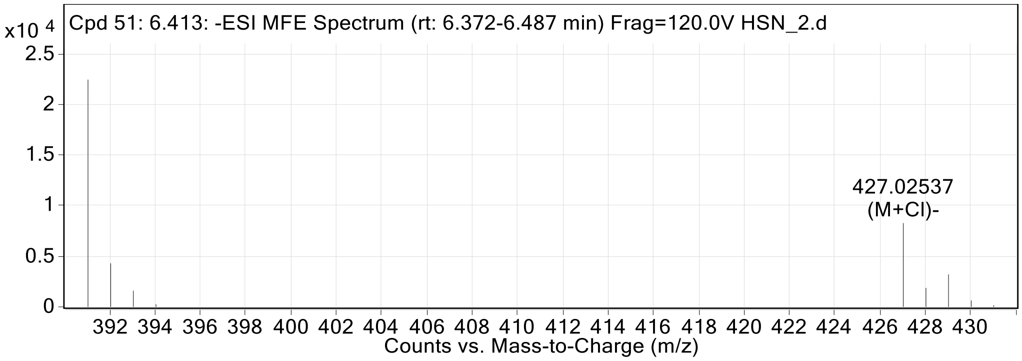

Peak List

m/z	z	Abund	Ion
391.0488	-1	22439.11	(M-H)-
392.05196	-1	4295.38	(M-H)-
393.04922	-1	1596.89	(M-H)-
394.04954	-1	257.82	(M-H)-
427.02537	-1	8272.3	(M+Cl)-
428.02911	-1	1872.72	(M+Cl)-
429.02287	-1	3208.4	(M+Cl)-
430.02531	-1	633.85	(M+Cl)-
431.02024	-1	163.1	(M+Cl)-

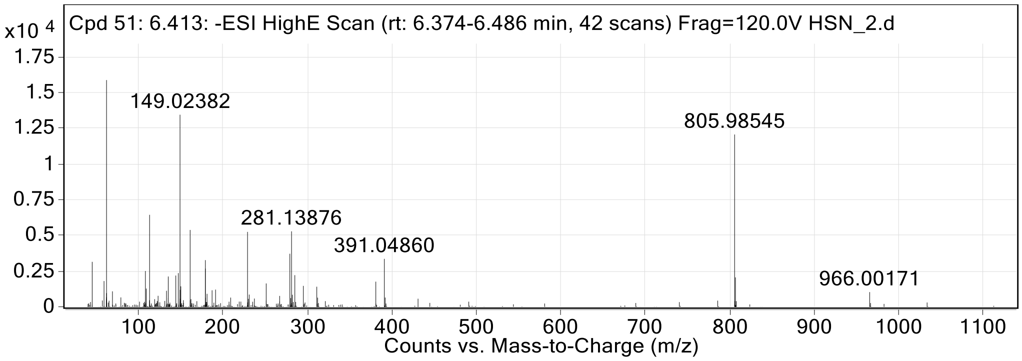

Peak List

m/z	z	Abund
61.98843		15889.28
112.98509		6445.02
149.02382	1	13461.26
161.0239	1	5384.92
179.07026	1	3270.51
229.01707	1	5248.63
279.12337	1	3717.29
281.13876	1	5288.76
391.0486	1	3359.14
805.98545	1	12076.47

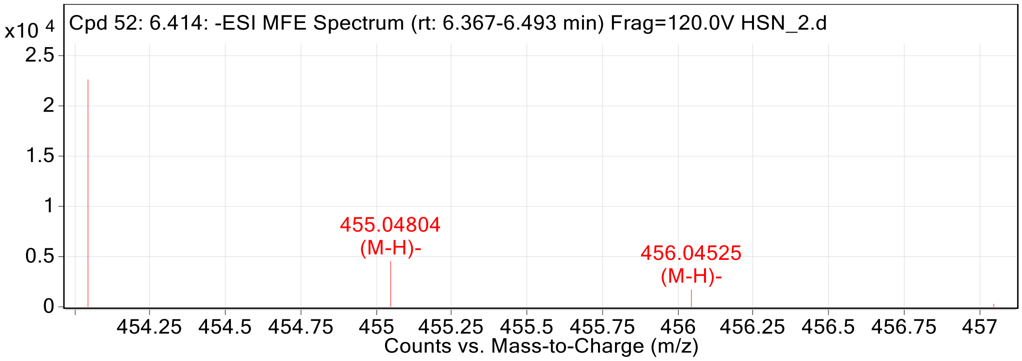

Peak List

m/z	z	Abund	Ion
454.04406	-1	22672.87	(M-H)-
455.04804	-1	4556.66	(M-H)-
456.04525	-1	1737.04	(M-H)-
457.04803	-1	299.7	(M-H)-

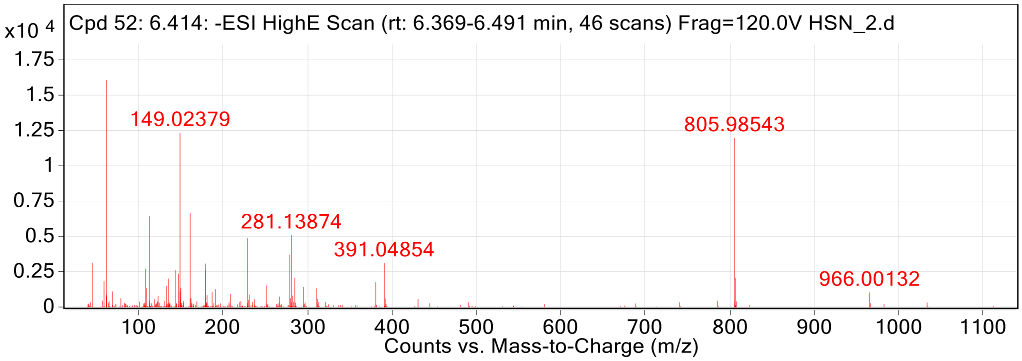

Peak List

m/z	z	Abund
44.99828		3145.53
61.98841		16110.08
112.98508		6435.81
149.02379	1	12363.14
161.02383	1	6661.96
229.01704	1	4871.14
279.12335	1	3711.02
281.13874	1	5093.67
391.04854	1	3113.87
805.98543	1	11995.37

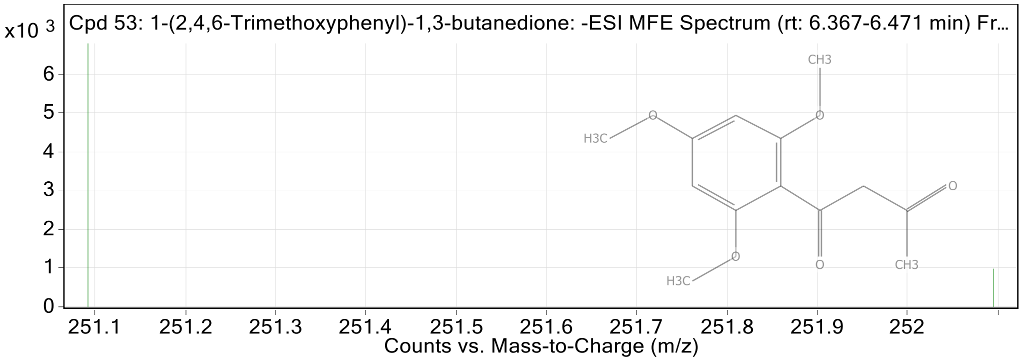

Peak List

m/z	z	Abund	Name	Formula	Ion	Score (DB)	Hits (DB)

251.09181	-1	6788.68	1-(2,4,6-Trimethoxyphenyl)-1,3-butanedione	C13 H16 O5	(M-H)-	84.58	10
252.09575	-1	983.65		C13 H16 O5	(M-H)-		

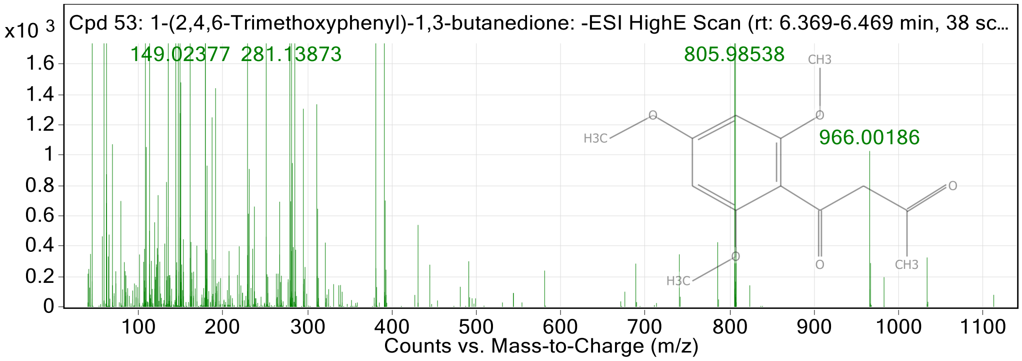

Peak List

m/z	z	Abund
61.9884		15614.62
112.98503		6446.67
149.02377	1	14560.16
161.02388	1	4293.27
179.07037	1	3545.05
229.01703	1	5736.9
279.1233	1	4230.58
281.13873	1	5908.02
391.04853	1	3649.89
805.98538	1	12017.62

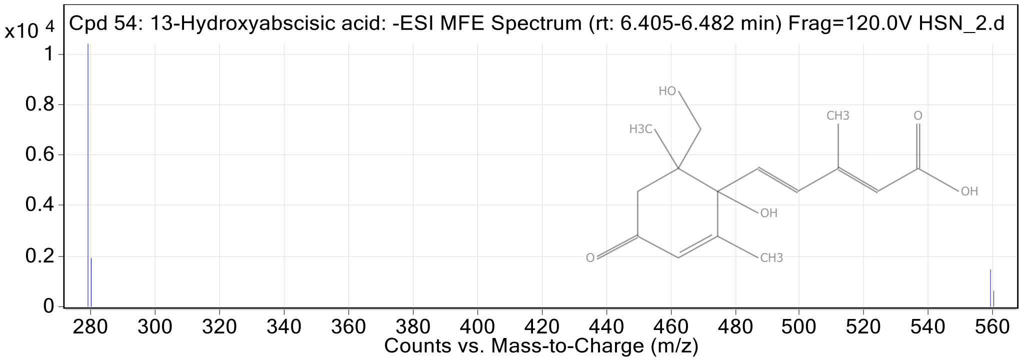

Peak List

m/z	z	Abund	Name	Formula	Ion	Score (DB)	Hits (DB)
279.12304	-1	10408.7	13-Hydroxyabscisic acid	C15 H20 O5	(M-H)-	81.51	10
280.12575	-1	1924.24		C15 H20 O5	(M-H)-		
559.25322	-1	1466.22			(2M-H)-		
560.25644	-1	623.89			(2M-H)-		

Qualitative Analysis Report

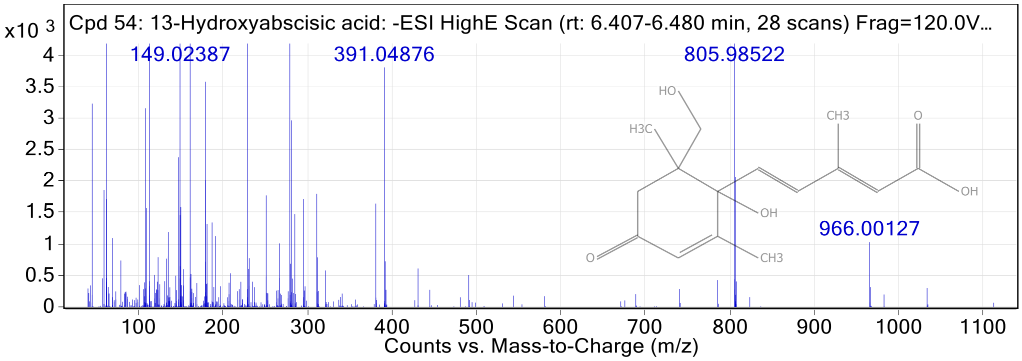

Peak List

m/z	z	Abund
44.99836		3230.41
61.9885		16022.31
112.98524		6428.46
149.02387	1	15002.31
161.02391	1	5165.81
179.03462	1	3577.18
229.0171	1	5805.35
279.12337	1	4185.82
391.04876	1	3805.13
805.98522	1	12040.8

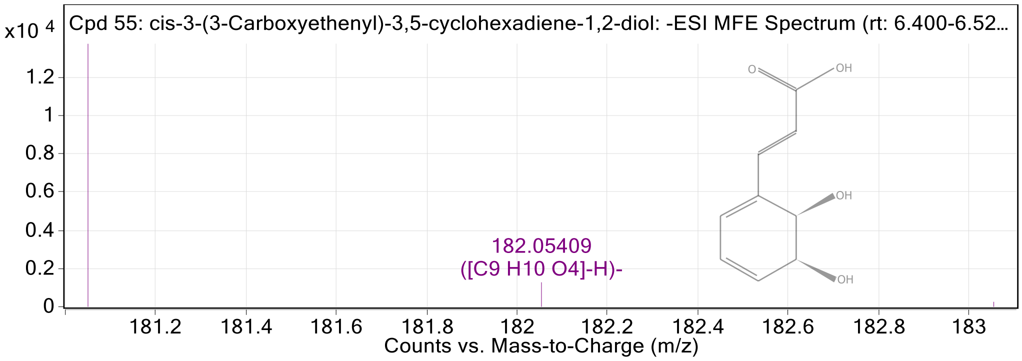

Peak List

m/z	z	Abund	Name	Formula	Ion	Score (DB)	Hits (DB)
181.05008	-1	13771.81	cis-3-(3-Carboxyethenyl)-3,5-cyclohexadiene-1,2-diol	C9 H10 O4	(M-H)-	97.75	10
182.05409	-1	1281.22		C9 H10 O4	(M-H)-		
183.05553	-1	259.49		C9 H10 O4	(M-H)-		

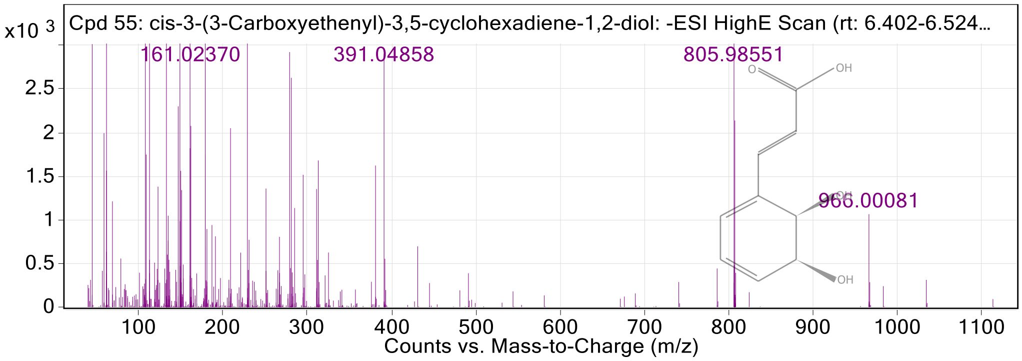

Peak List

m/z	z	Abund
44.99822		3013.07

61.98834		16589.71
108.02122		4039.86
112.98505		6773.78
133.02893	1	5727.94
149.02366	1	10889.88
161.0237	1	24024.61
179.03438	1	3019.86
229.01691	1	4165.52
805.98551	1	12667.33

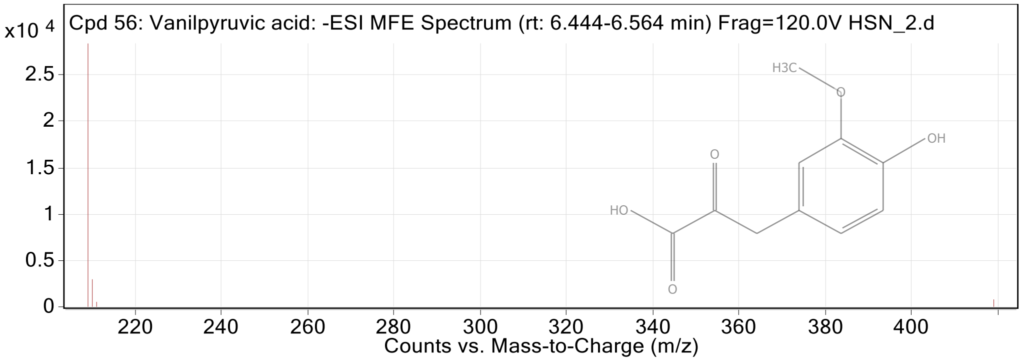

Peak List

m/z	z	Abund	Name	Formula	Ion	Score (DB)	Hits (DB)
209.04478	-1	28402.86	Vanilpyruvic acid	C10 H10 O5	(M-H)-	95.24	10
210.04734	-1	2970.14		C10 H10 O5	(M-H)-		
211.04938	-1	536.87		C10 H10 O5	(M-H)-		
419.09431	-1	799.88			(2M-H)-		

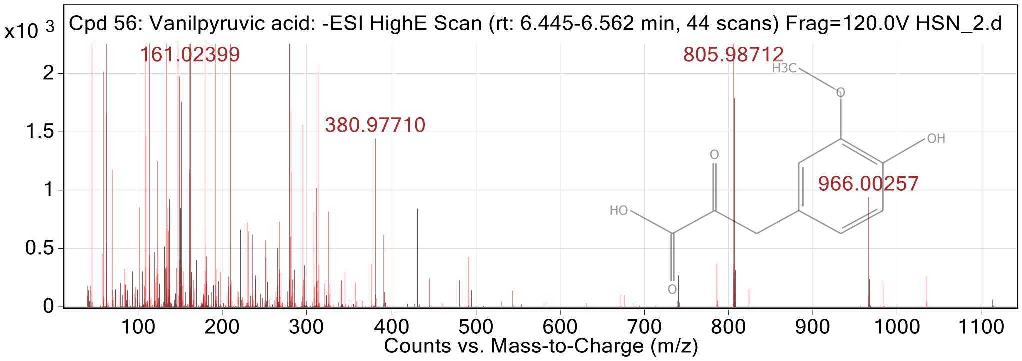

Peak List

m/z	z	Abund
44.99828		3073.64
61.98846		18940.08
108.02144		3748.71
112.98525		5852.63
133.02915	1	6793.59
161.02399	1	27997.12
179.0347	1	3164.58
191.14407	1	2756.7
279.12354	1	4084.83
805.98712	1	10833.76

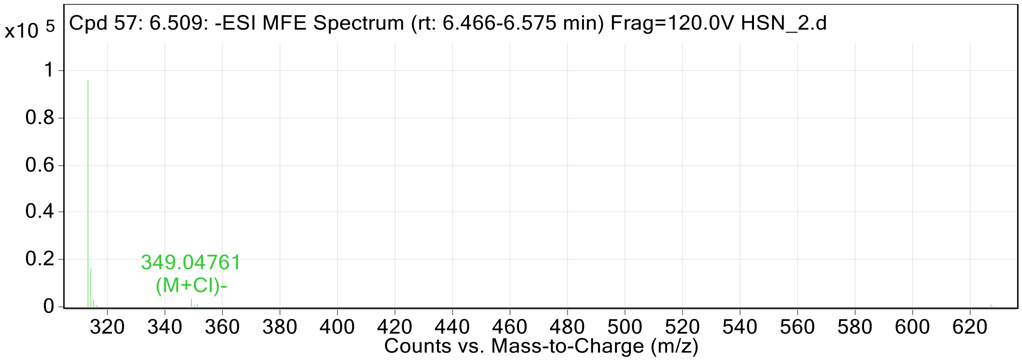

Peak List

m/z	z	Abund	Ion
313.07404	-1	96062.21	(M-H)-
314.07441	-1	16234.48	(M-H)-
315.07776	-1	2739.47	(M-H)-
316.07773	-1	648.89	(M-H)-
349.04761	-1	3423.05	(M+Cl)-
350.05138	-1	700.22	(M+Cl)-
351.04525	-1	1070.45	(M+Cl)-
627.14987	-1	973.84	(2M-H)-
628.14945	-1	366.92	(2M-H)-

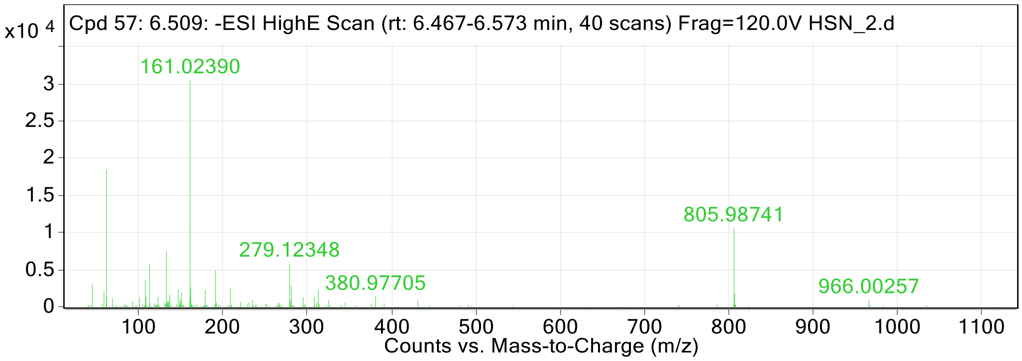

Peak List

m/z	z	Abund
44.9982		3109.62
61.98836		18478.43
108.02135		3578.35
112.98514		5804.96
133.02905	1	7481.6
161.0239	1	30520.41
191.14381	1	4898.06
279.12348	1	5844.35
280.98268		2819.32
805.98741	1	10596.07

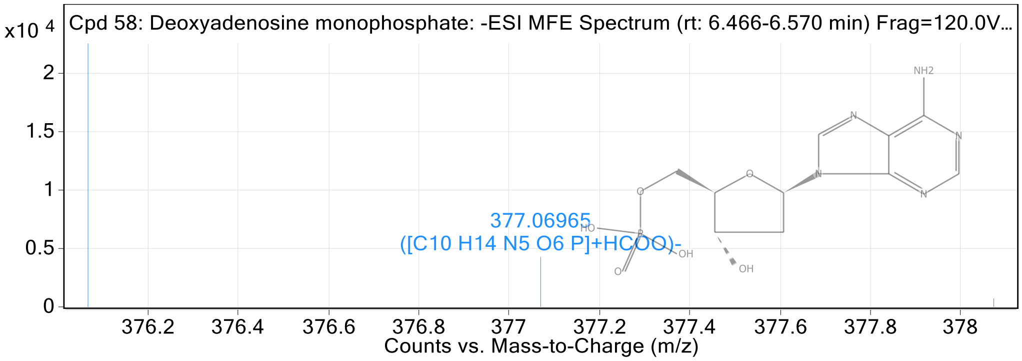

Peak List

m/z	z	Abund	Name	Formula	Ion	Score (DB)	Hits (DB)
376.06692	-1	22587.52	Deoxyadenosine monophosphate	C10 H14 N5 O6 P	(M+HCOO)-	93.31	1
377.06965	-1	4274.08		C10 H14 N5 O6 P	(M+HCOO)-		
378.07329	-1	726.26		C10 H14 N5 O6 P	(M+HCOO)-		

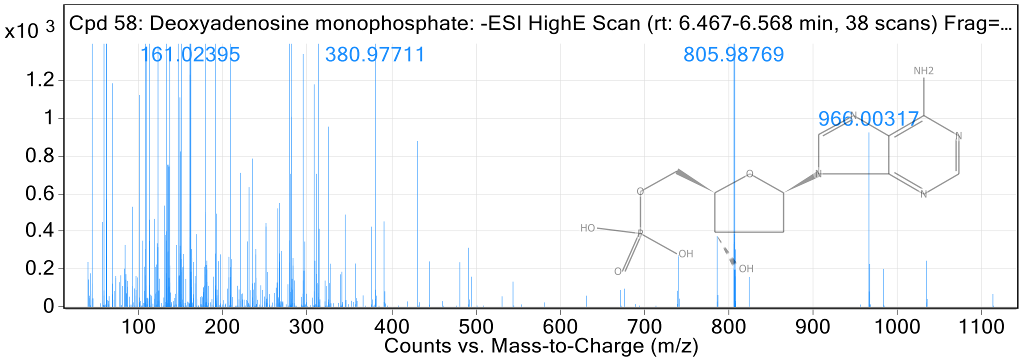

Peak List

m/z	z	Abund
44.99822		3127.08
61.98839		18719.07
108.02139		3750.71
112.9852		5763.34
133.0291	1	7841.81
161.02395	1	31986.05
162.02732	1	2737.19
191.14395	1	4050.8
279.12363	1	4952.1
805.98769	1	10547.71

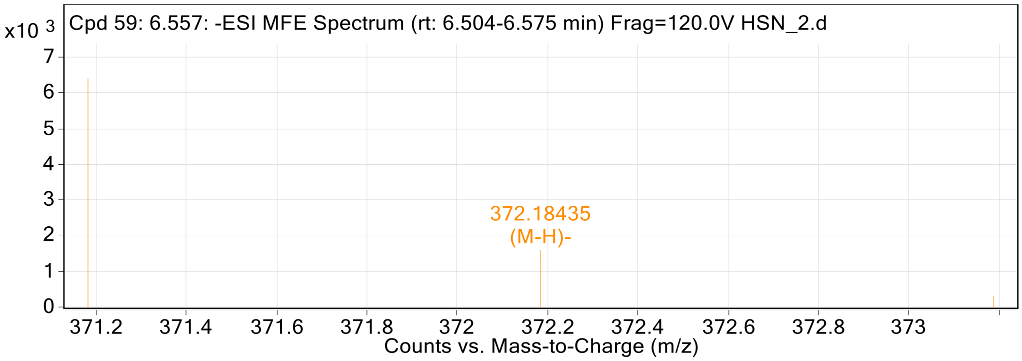

Peak List

m/z	z	Abund	Ion
371.1821	-1	6407	(M-H)-

372.18435	-1	1596.99	(M-H)-
373.18809	-1	310.49	(M-H)-

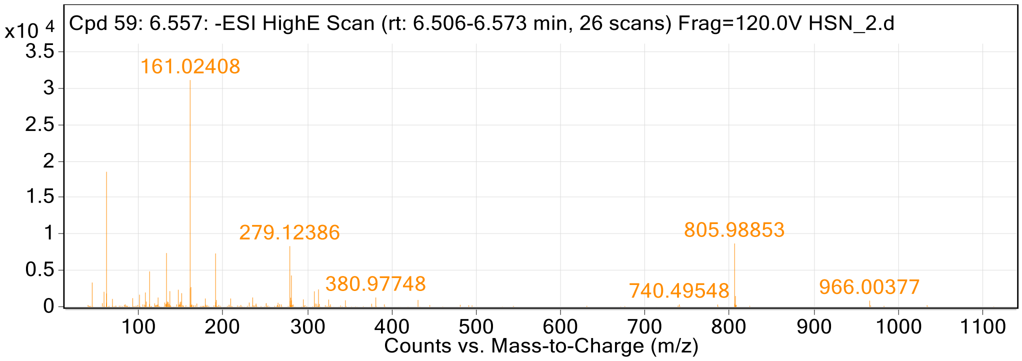

Peak List

m/z	z	Abund
44.99822		3326.23
61.98843		18518.41
112.98522		4855.82
133.02926	1	7370.97
161.02408	1	31102.86
162.02744	1	2704.73
191.14407	1	7315.03
279.12386	1	8319.25
280.98306	1	4294.88
805.98853	1	8683.27

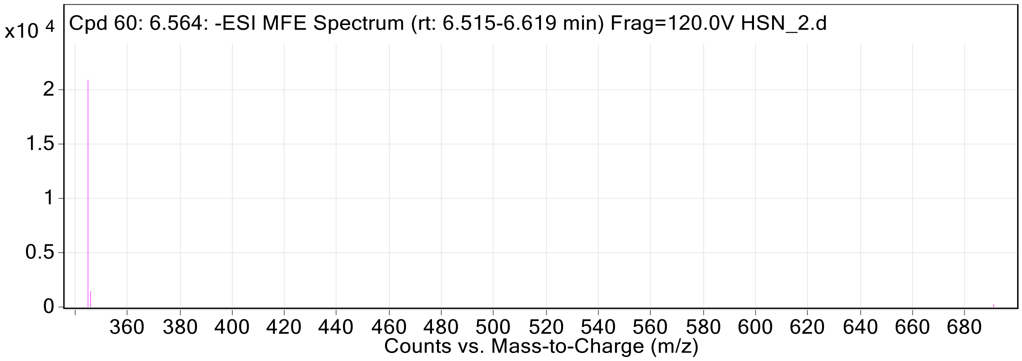

Peak List

m/z	z	Abund	Ion
344.97799	-1	20942.22	(M-H)-
345.98261	-1	1454.42	(M-H)-
690.96583	-1	257.7	(2M-H)-

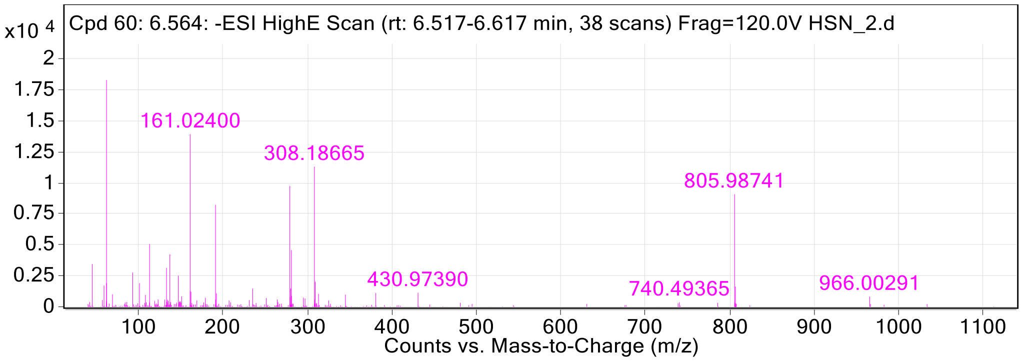

Peak List

m/z	z	Abund

44.99824		3453.32
61.98841		18296.08
112.98523		5064.68
137.02406	1	4235.04
161.024	1	13929.93
191.14394	1	8234.35
279.12355	1	9755.1
280.98295	1	4579.32
308.18665	1	11304.57
805.98741	1	9081.99

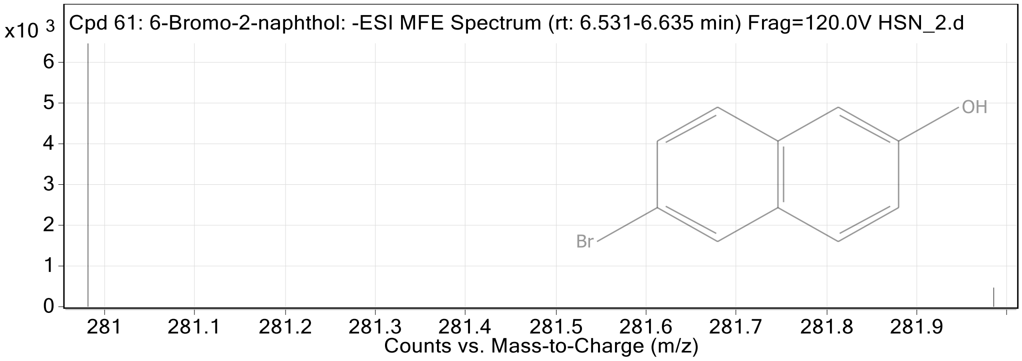

Peak List

m/z	z	Abund	Name	Formula	Ion	Score (DB)	Hits (DB)
280.9819	-1	6457.71	6-Bromo-2-naphthol	C10 H7 Br O	(M+CH3COO)-	59.52	1
281.98524	-1	469.74		C10 H7 Br O	(M+CH3COO)-		

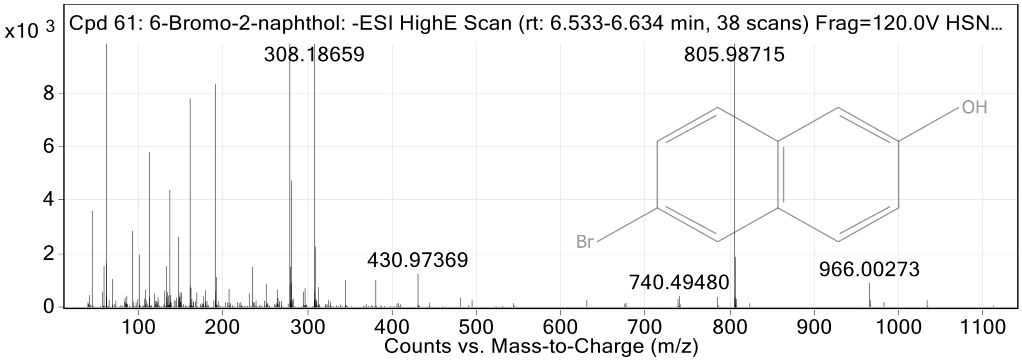

Peak List

m/z	z	Abund
44.99829		3605.13
61.9884		18521.75
112.98522		5801.19
137.02406	1	4368.78
161.02391	1	7810.36
191.14392	1	8343.13
279.12348	1	9860.59
280.98295	1	4731.49
308.18659	1	12595.06
805.98715	1	10419.47

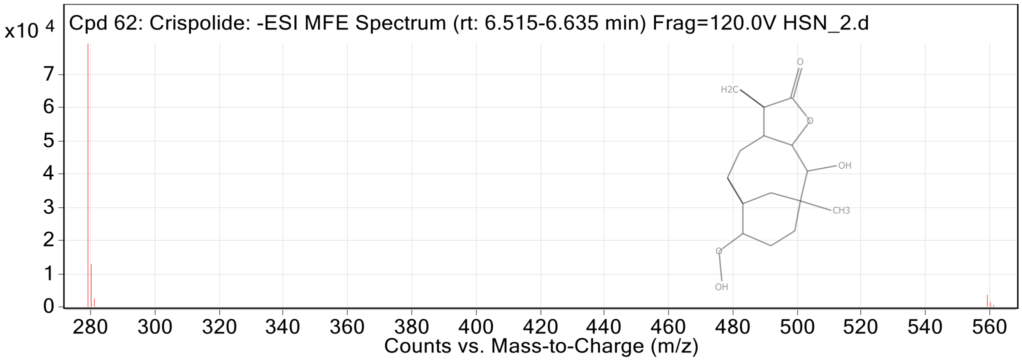

Peak List

m/z	z	Abund	Name	Formula	Ion	Score (DB)	Hits (DB)
279.12396	-1	79414.87	Crispolide	C15 H20 O5	(M-H)-	98.92	10
280.12679	-1	12858.57		C15 H20 O5	(M-H)-		
281.13101	-1	2572.42		C15 H20 O5	(M-H)-		
559.25434	-1	3649.91			(2M-H)-		
560.25746	-1	1377.75			(2M-H)-		
561.2645	-1	613.34			(2M-H)-		

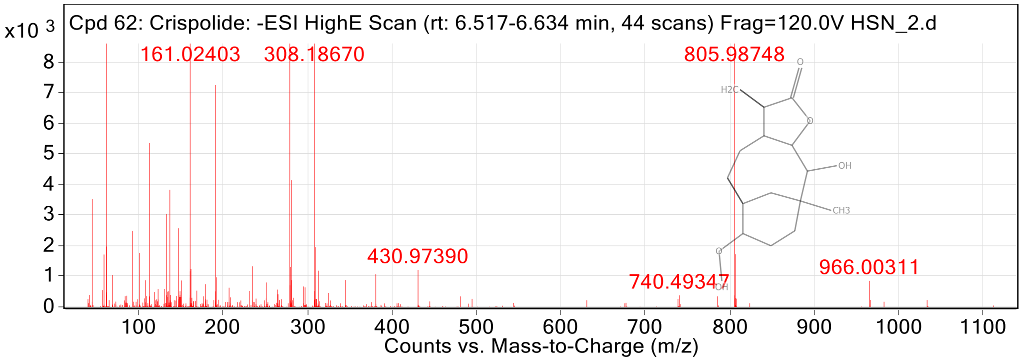

Peak List

m/z	z	Abund
44.99829		3520.75
61.98843		18619.28
112.98526		5352.42
137.02411	1	3830.51
161.02403	1	14316.01
191.14398	1	7248.6
279.12359	1	8612.47
280.98302		4139.13
308.1867	1	10882.21
805.98748	1	9552.6

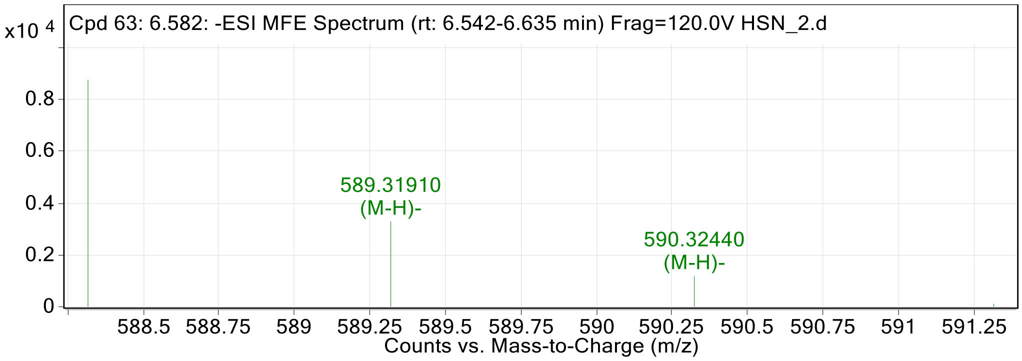

Peak List

m/z	z	Abund	Ion
588.31632	-1	8757.47	(M-H)-
589.3191	-1	3291.81	(M-H)-
590.3244	-1	1193.11	(M-H)-
591.31613	-1	116.41	(M-H)-

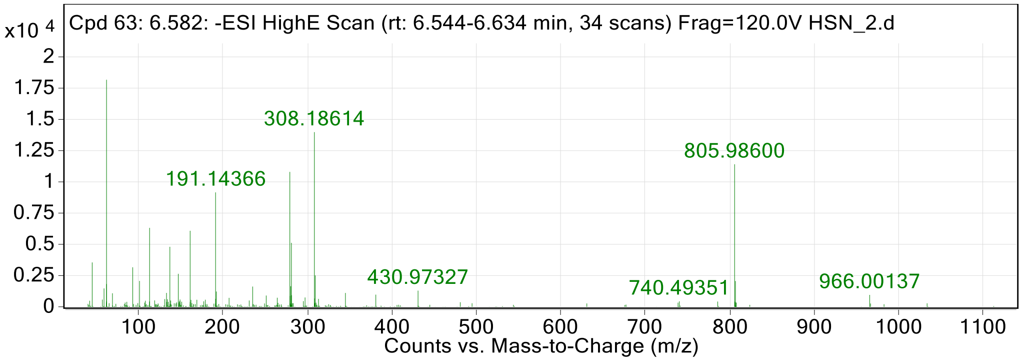

Peak List

m/z	z	Abund
44.99825		3551.45
61.9883		18170.82
112.98506		6317.75
137.02388	1	4801.26
161.02371	1	6084.18
191.14366	1	9157.52
279.1231	1	10797.55
280.9826	1	5119.63
308.18614	1	13981.51
805.986	1	11406.91

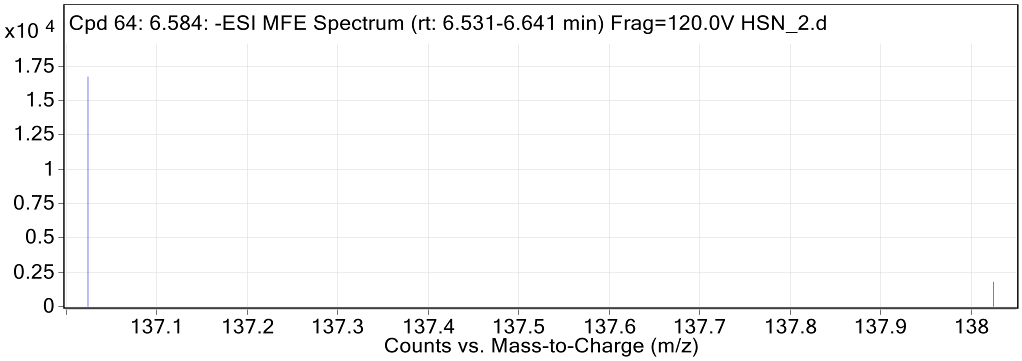

Peak List

m/z	z	Abund	Ion
137.02372	-1	16751.27	(M-H)-
138.02537	-1	1812.11	(M-H)-

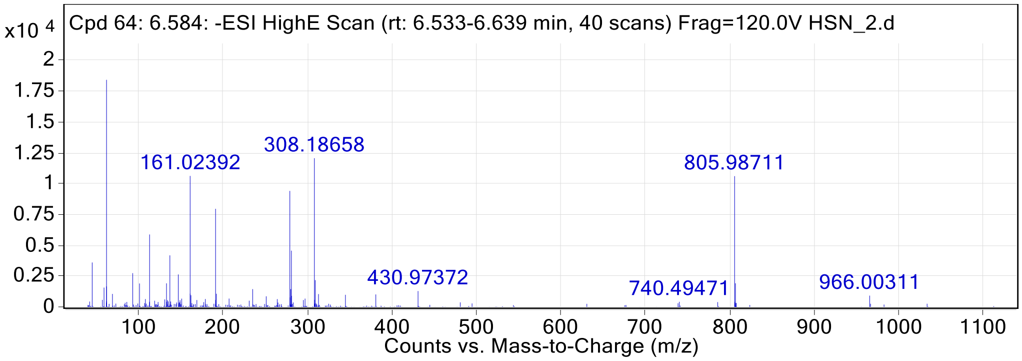

Peak List

m/z	z	Abund
44.99829		3601.53
61.98839		18418.82
112.98521		5873.01
137.02404	1	4180.03
161.02392	1	10623.61
191.14391	1	7956.1
279.12347	1	9406.5
280.98293		4560.97
308.18658	1	12062.92
805.98711	1	10606

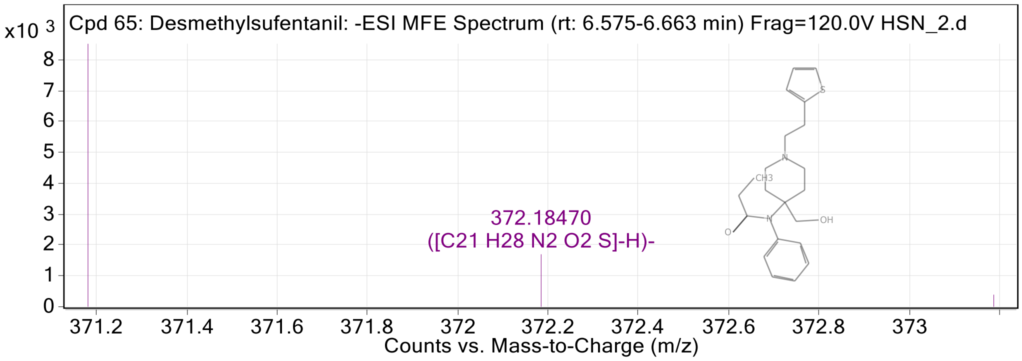

Peak List

m/z	z	Abund	Name	Formula	Ion	Score (DB)	Hits (DB)
371.18119	-1	8521.3	Desmethylsufentanil	C21 H28 N2 O2 S	(M-H)-	84.9	2
372.1847	-1	1700.55		C21 H28 N2 O2 S	(M-H)-		
373.18703	-1	391.55		C21 H28 N2 O2 S	(M-H)-		

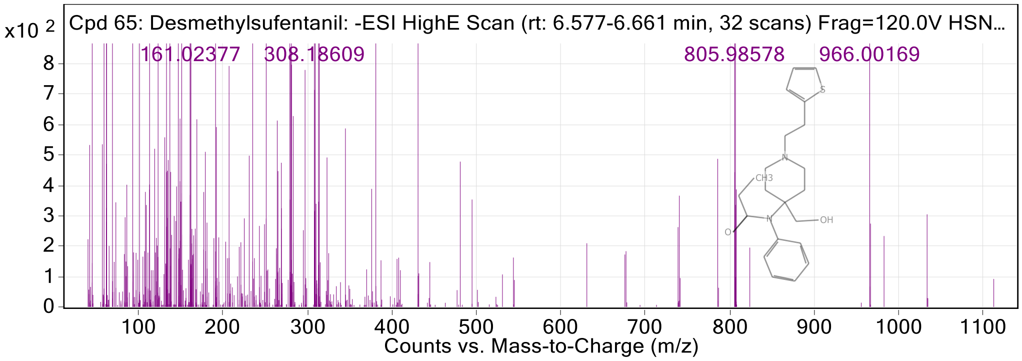

Peak List

m/z	z	Abund
61.98828		17876.87
112.98504		6863.78

133.02883	1	5338.74
137.0238	1	3734.42
161.02377	1	36823.92
191.14361	1	4244.62
279.12296	1	5492.59
308.18609	1	13525.26
313.07094	1	5527.18
805.98578	1	12504.7

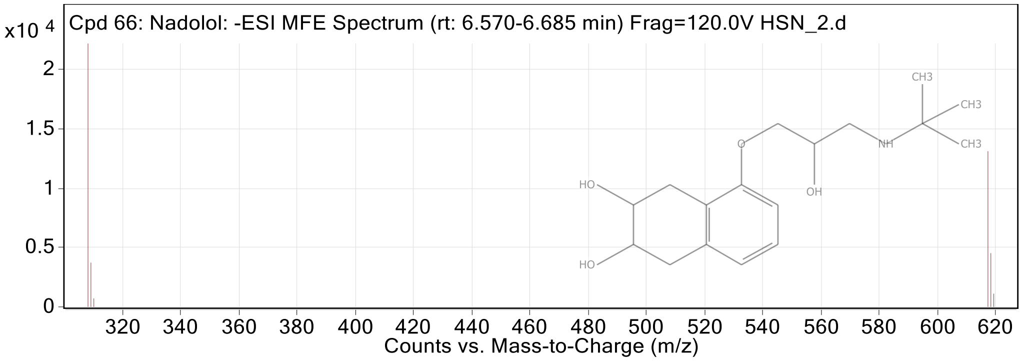

Peak List

m/z	z	Abund	Name	Formula	Ion	Score (DB)	Hits (DB)
308.18598	-1	22227.86	Nadolol	C17 H27 N O4	(M-H)-	96.45	10
309.18923	-1	3732.39		C17 H27 N O4	(M-H)-		
310.19234	-1	716.06		C17 H27 N O4	(M-H)-		
617.37964	-1	13136.45			(2M-H)-		
618.38285	-1	4535.79			(2M-H)-		
619.38578	-1	1114.89			(2M-H)-		

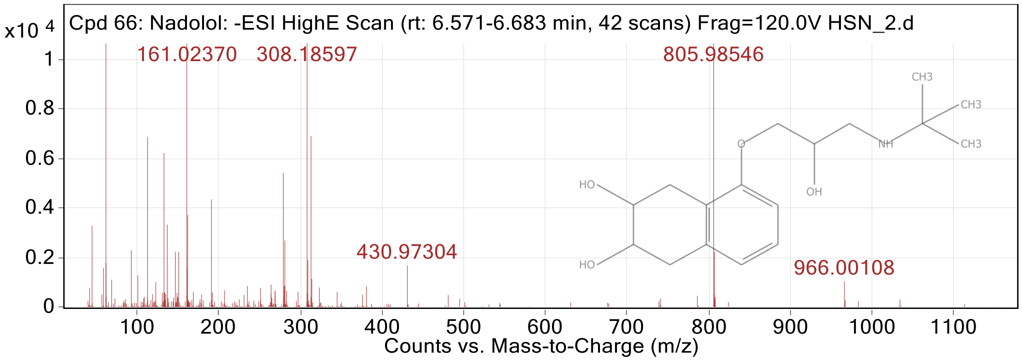

Peak List

m/z	z	Abund
61.98824		18101.91
112.98497		6866.08
133.02878	1	6213.93
161.0237	1	44319.03
162.02703	1	3709.99
191.14347	1	4339.48
279.12287	1	5416.89
308.18597	1	10640.88
313.07093	1	6901.19
805.98546	1	12431.19

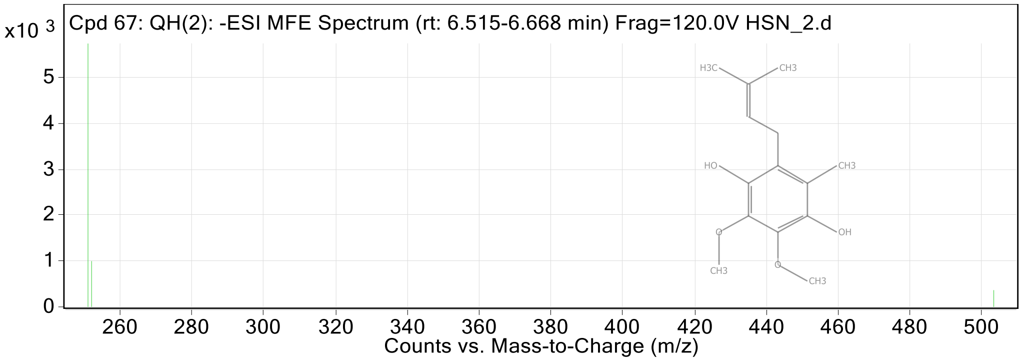

Peak List

m/z	z	Abund	Name	Formula	Ion	Score (DB)	Hits (DB)
251.12864	-1	5742.16	QH(2)	C14 H20 O4	(M-H)-	85.27	10
252.13208	-1	997.67		C14 H20 O4	(M-H)-		
503.26343	-1	364.88			(2M-H)-		

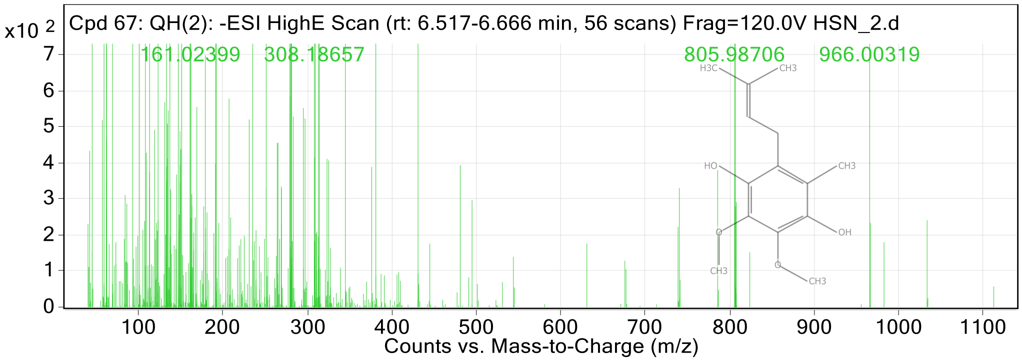

Peak List

m/z	z	Abund
61.98838		18126.82
112.98519		5702.53
133.02904	1	5566.47
161.02399	1	33794.01
191.1439	1	5830.73
279.12348	1	6975.45
280.9829		3581.81
308.18657	1	8725.04
313.07141	1	4493.46
805.98706	1	10213.91

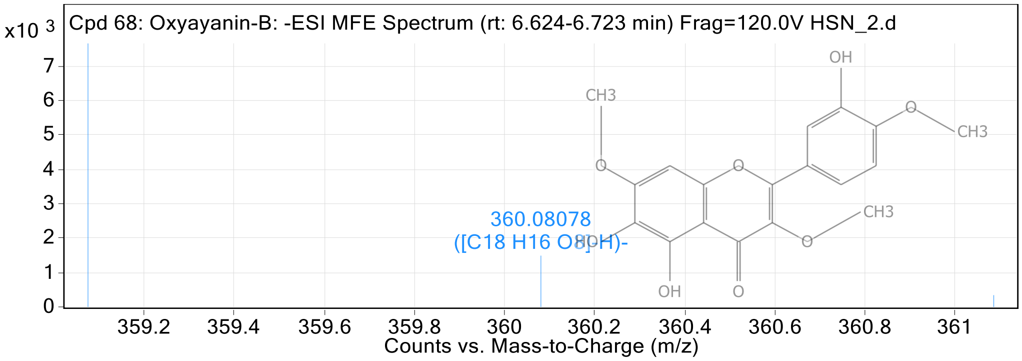

Peak List

m/z	z	Abund	Name	Formula	Ion	Score (DB)	Hits (DB)
359.07609	-1	7661.92	Oxyayanin-B	C18 H16 O8	(M-H)-	94.55	10
360.08078	-1	1491.06		C18 H16 O8	(M-H)-		

361.0853	-1	341.73		C18 H16 O8	(M-H)-		

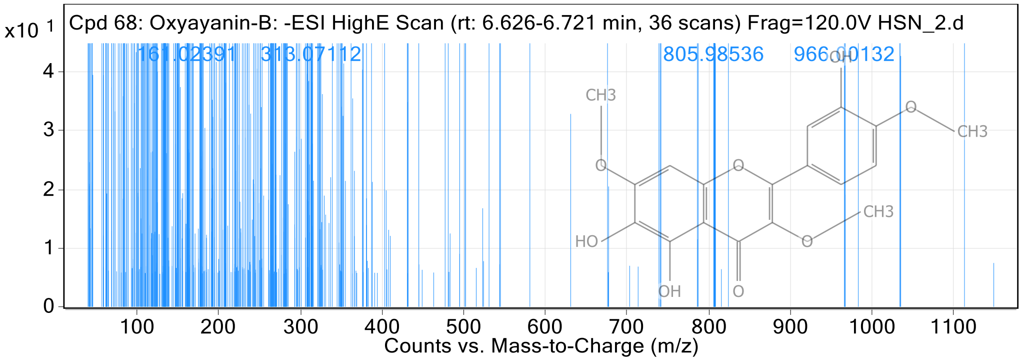

Peak List

m/z	z	Abund
61.98846		18985.82
112.98518		6962.3
133.02904		9713.34
135.04448	1	3152.26
161.02391	1	57469.87
162.02727	1	4873.69
207.06596	1	6771.98
264.16033	1	4173.29
313.07112	1	8837.98
805.98536	1	12787.92

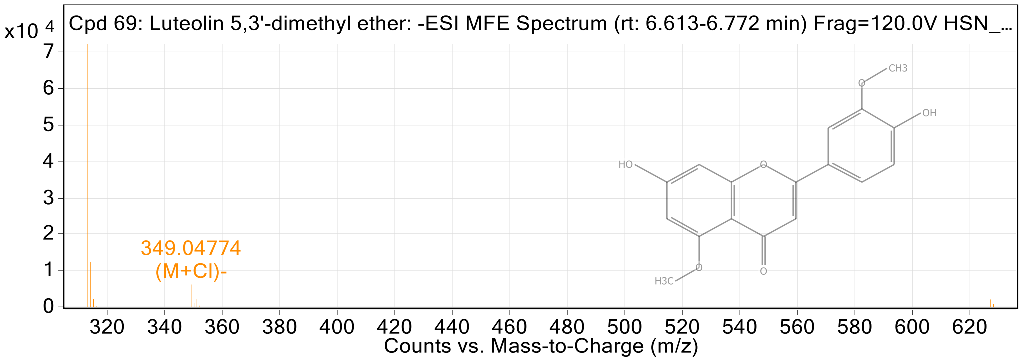

Peak List

m/z	z	Abund	Name	Formula	Ion	Score (DB)	Hits (DB)
313.07128	-1	72412.8	Luteolin 5,3'-dimethyl ether	C17 H14 O6	(M-H)-	98.61	10
314.0747	-1	12397.65		C17 H14 O6	(M-H)-		
315.07728	-1	2108.72		C17 H14 O6	(M-H)-		
316.08175	-1	113.93		C17 H14 O6	(M-H)-		
349.04774	-1	6164.78			(M+Cl)-		
350.05114	-1	1148.41			(M+Cl)-		
351.04506	-1	2214.38			(M+Cl)-		
352.0506	-1	354.71			(M+Cl)-		
627.15298	-1	2053.81			(2M-H)-		
628.15458	-1	772.7			(2M-H)-		

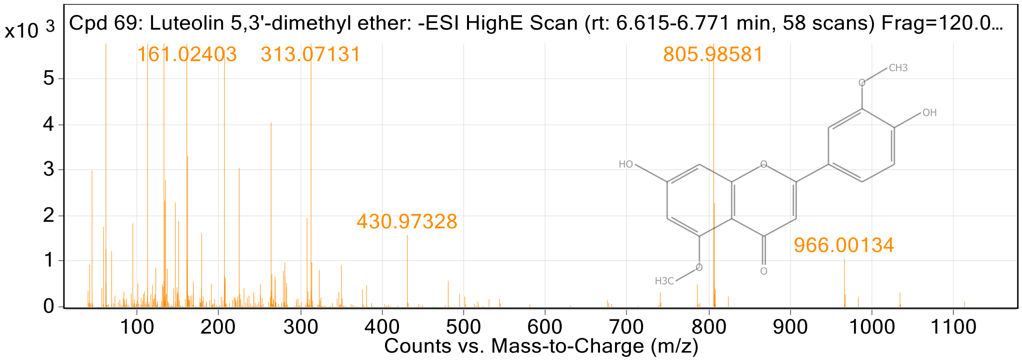

Peak List

m/z	z	Abund
61.98856		19375.83
112.98535		7039.62
133.02913		6763.63
161.02403	1	37928.97
162.02746	1	3307.44
207.06606	1	5634.76
225.07164	1	3047.36
264.16036	1	4042.76
313.07131	1	5774.39
805.98581	1	12961.14

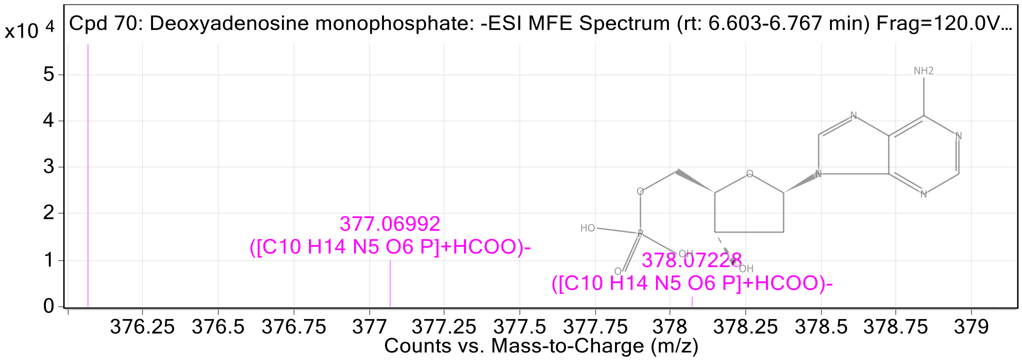

Peak List

m/z	z	Abund	Name	Formula	Ion	Score (DB)	Hits (DB)
376.06676	-1	56592.39	Deoxyadenosine monophosphate	C10 H14 N5 O6 P	(M+HCOO)-	95.6	1
377.06992	-1	9996.17		C10 H14 N5 O6 P	(M+HCOO)-		
378.07228	-1	2181.26		C10 H14 N5 O6 P	(M+HCOO)-		
379.07347	-1	91.33		C10 H14 N5 O6 P	(M+HCOO)-		

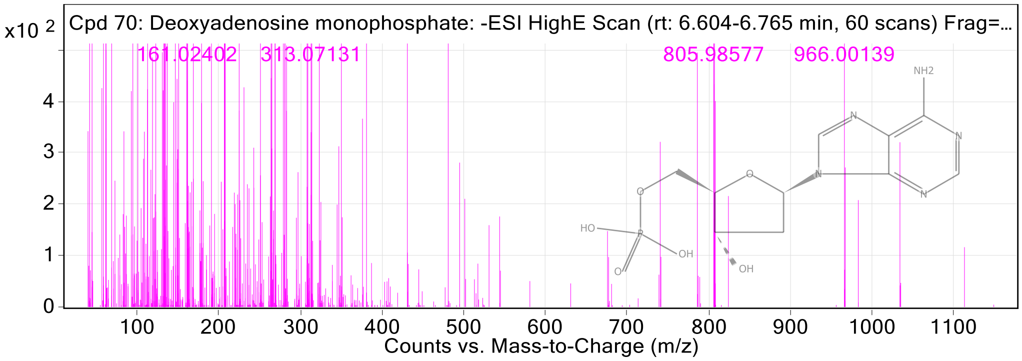

Peak List

m/z	z	Abund

61.98855		19421.01
112.98535		7029.15
133.02912		6543.74
161.02402	1	36643.69
162.02746	1	3188.76
207.06605	1	5413.44
264.16035	1	3881.76
308.18638	1	4035.23
313.07131	1	5573.19
805.98577	1	12907.15

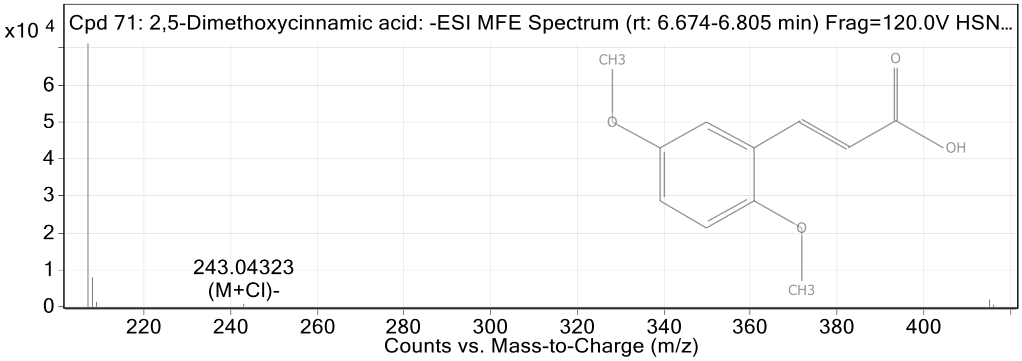

Peak List

m/z	z	Abund	Name	Formula	Ion	Score (DB)	Hits (DB)
207.06661	-1	71270.55	2,5-Dimethoxycinnamic acid	C11 H12 O4	(M-H)-	98.35	10
208.0693	-1	7975.54		C11 H12 O4	(M-H)-		
209.07229	-1	1350.45		C11 H12 O4	(M-H)-		
243.04323	-1	819.86			(M+Cl)-		
415.13879	-1	1944.67			(2M-H)-		
416.14288	-1	594.19			(2M-H)-		

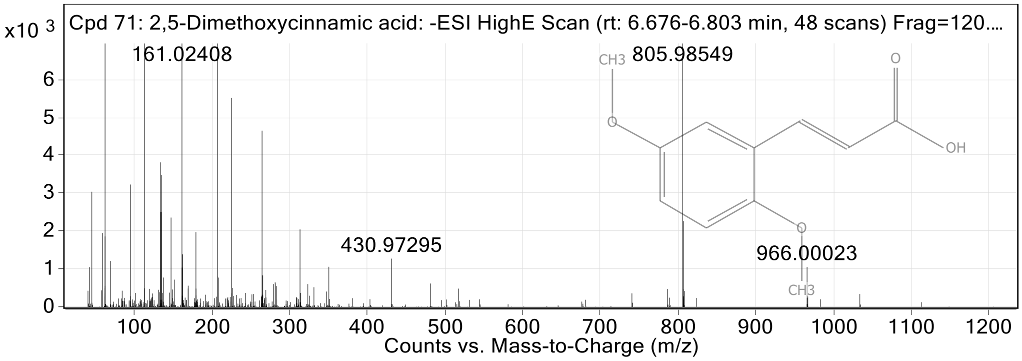

Peak List

m/z	z	Abund
61.98865		20680.66
94.93621		3228.35
112.98543		6986.49
133.02925		3813.49
135.04467	1	3474.45
161.02408	1	14263.64
207.06605	1	6956.31
225.07163	1	5512.24
264.16032	1	4651.04
805.98549	1	13244.93

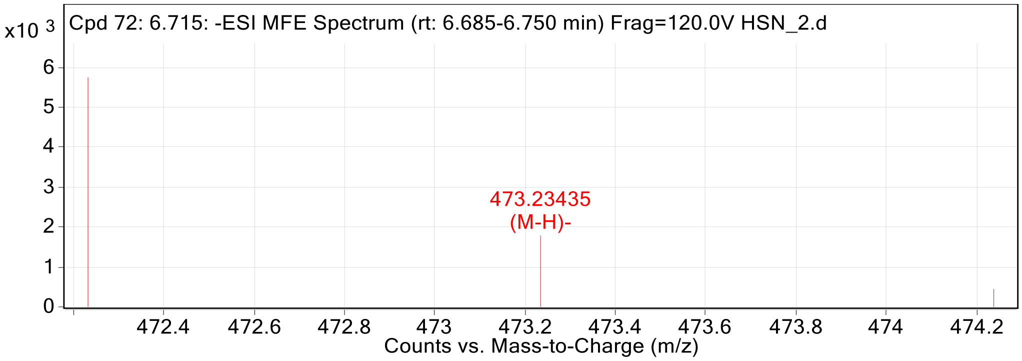

Peak List

m/z	z	Abund	Ion
472.23171	-1	5746.5	(M-H)-
473.23435	-1	1787.43	(M-H)-
474.23831	-1	448.32	(M-H)-

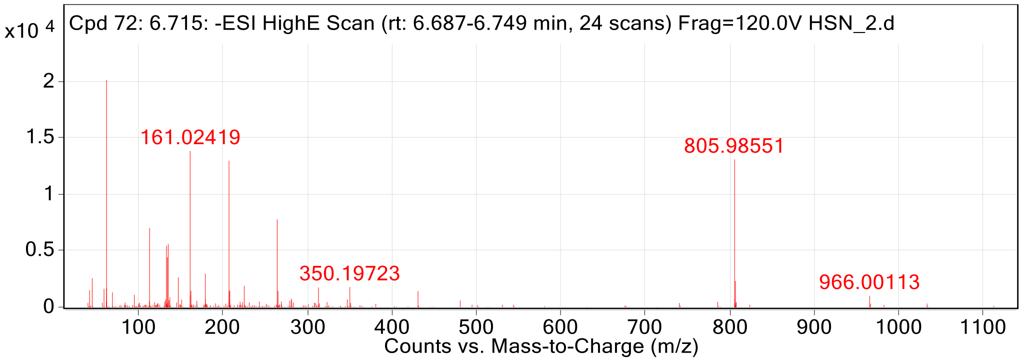

Peak List

m/z	z	Abund
61.98875		20124.05
112.98555		7011.21
133.02938		5434.02
134.03664		4409.47
135.04475	1	5587.06
161.02419	1	13839.31
179.03487	1	2964.06
207.06616	1	12971.62
264.1604	1	7772.19
805.98551	1	13087.05

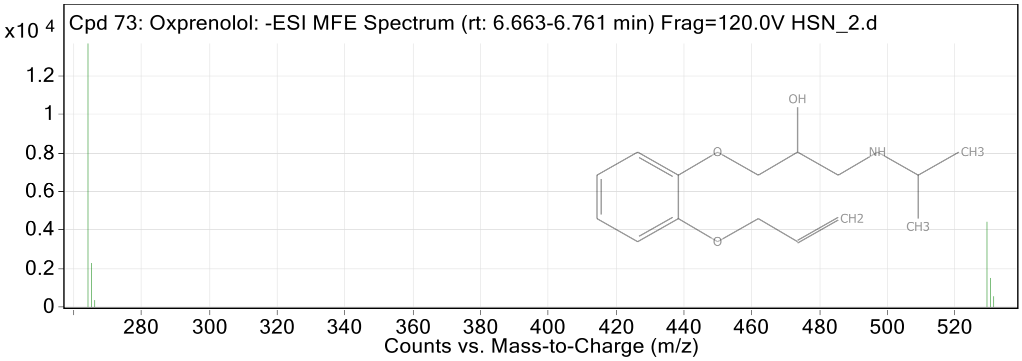

Peak List

m/z	z	Abund	Name	Formula	Ion	Score (DB)	Hits (DB)
264.16007	-1	13696.94	Oxprenolol	C15 H23 N O3	(M-H)-	94.4	10
265.16173	-1	2287.95		C15 H23 N O3	(M-H)-		

266.16426	-1	355.29		C15 H23 N O3	(M-H)-		
529.32685	-1	4430.41			(2M-H)-		
530.32914	-1	1509.57			(2M-H)-		
531.33163	-1	551.37			(2M-H)-		

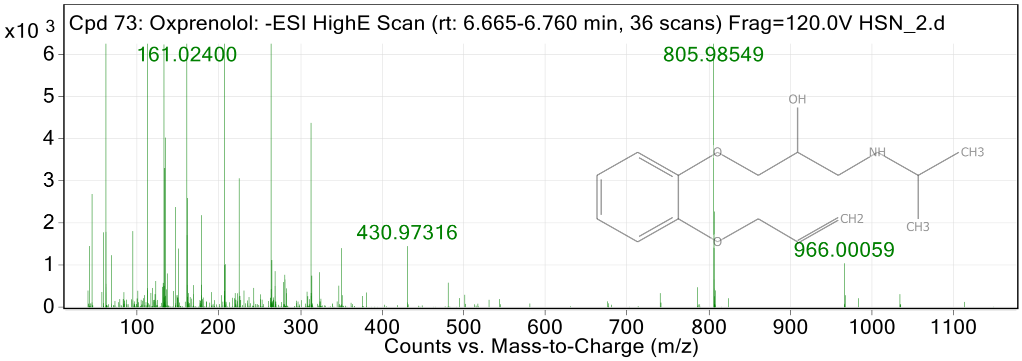

Peak List

m/z	z	Abund
61.9886		19923.66
112.98538		7071.73
133.02917		6237.84
134.03624		3297.05
135.04458	1	4027.07
161.024	1	28679.22
207.06601	1	8951.63
264.16028	1	6257.79
313.07142	1	4377.52
805.98549	1	13041.07

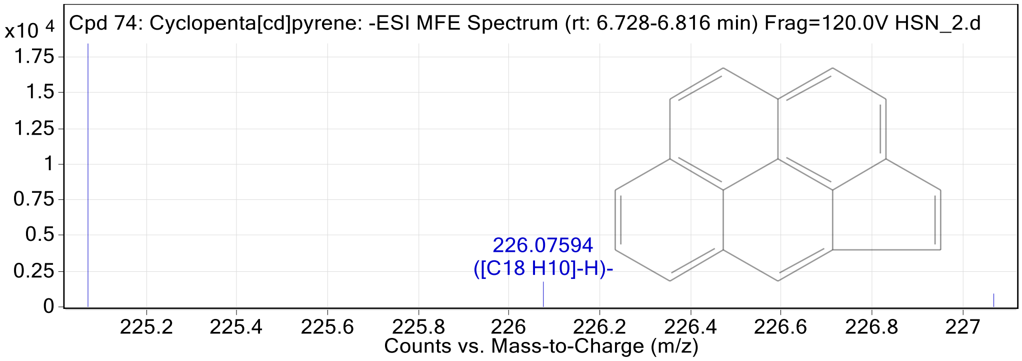

Peak List

m/z	z	Abund	Name	Formula	Ion	Score (DB)	Hits (DB)
225.07203	-1	18472.98	Cyclopenta[cd]pyrene	C18 H10	(M-H)-	69.04	2
226.07594	-1	1769.74		C18 H10	(M-H)-		
227.06882	-1	934.04		C18 H10	(M-H)-		

Qualitative Analysis Report

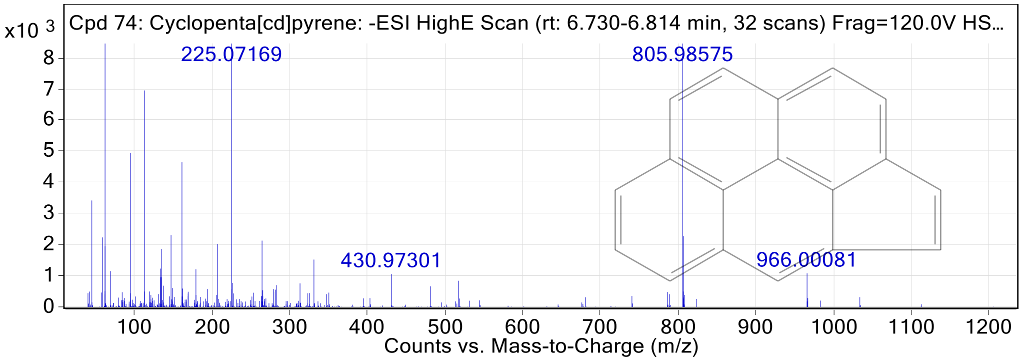

Peak List

m/z	z	Abund
44.99847		3417.92
59.0142		2230.79
61.98863		20869.31
94.93622		4945.21
112.98546		6951.61
146.96573		2303.52
161.02404	1	4644.81
225.07169	1	8462.04
805.98575	1	13466.41
806.98856	1	2270.67

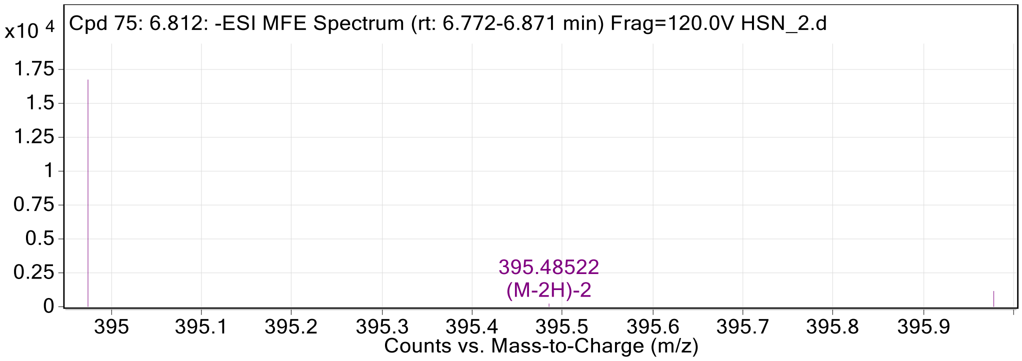

Peak List

m/z	z	Abund	Ion
394.97426	-2	16763.76	(M-2H)-2
395.48522	-2	236.68	(M-2H)-2
395.97811	-2	1162.61	(M-2H)-2

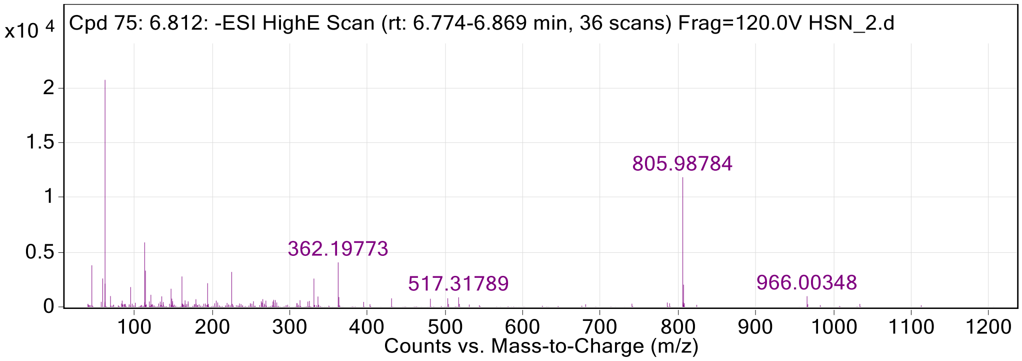

Peak List

m/z	z	Abund
44.99838		3798.65
59.01409		2593.46

61.98853		20725.62
112.98545		5887.09
114.05582		3308.73
161.02412	1	2778.78
225.07204	1	3190.87
330.97989		2585.29
362.19773	1	4063.04
805.98784	1	11836.5

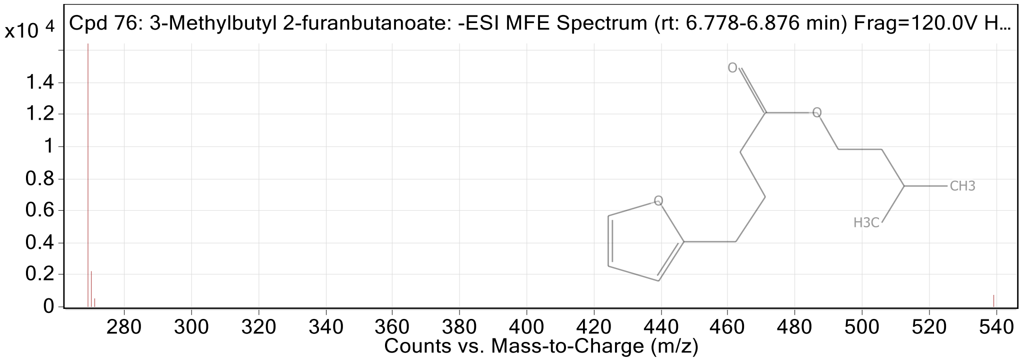

Peak List

m/z	z	Abund	Name	Formula	Ion	Score (DB)	Hits (DB)
269.13899	-1	16447.28	3-Methylbutyl 2-furanbutanoate	C13 H20 O3	(M+HCOO)-	96.86	10
270.14246	-1	2230.81		C13 H20 O3	(M+HCOO)-		
271.14819	-1	528.48		C13 H20 O3	(M+HCOO)-		
539.28457	-1	747			(2M-H)-		

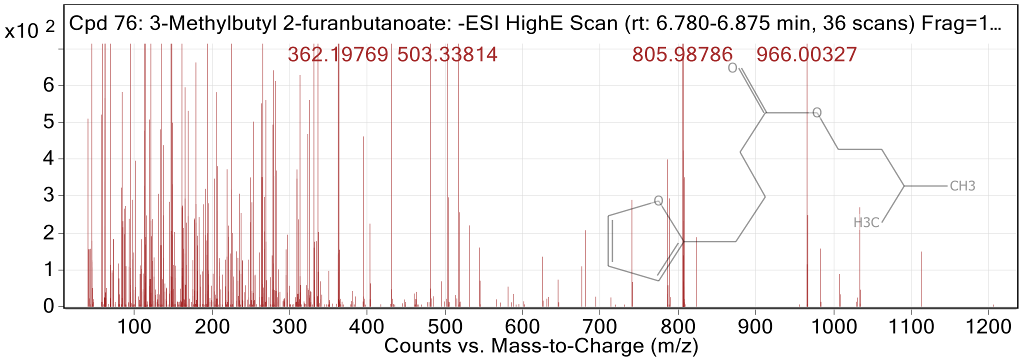

Peak List

m/z	z	Abund
44.9984		3791.61
59.01412		2620.18
61.98854		20771.85
112.98544		5896.14
114.05585	1	3610.7
161.02417	1	2755.02
225.07211		2419.97
330.97987		2623.23
362.19769	1	4458.53
805.98786	1	11817.28

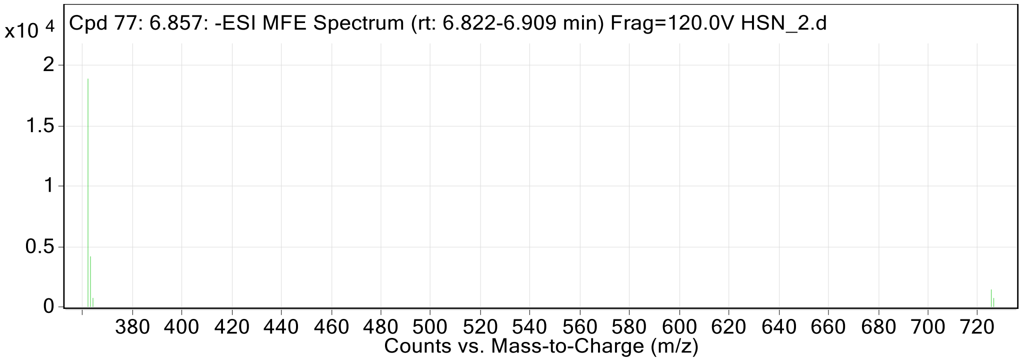

Peak List

m/z	z	Abund	Ion
362.20075	-1	18925.65	(M-H)-
363.20328	-1	4187.19	(M-H)-
364.20653	-1	743.82	(M-H)-
725.40937	-1	1442.75	(2M-H)-
726.41214	-1	736.06	(2M-H)-

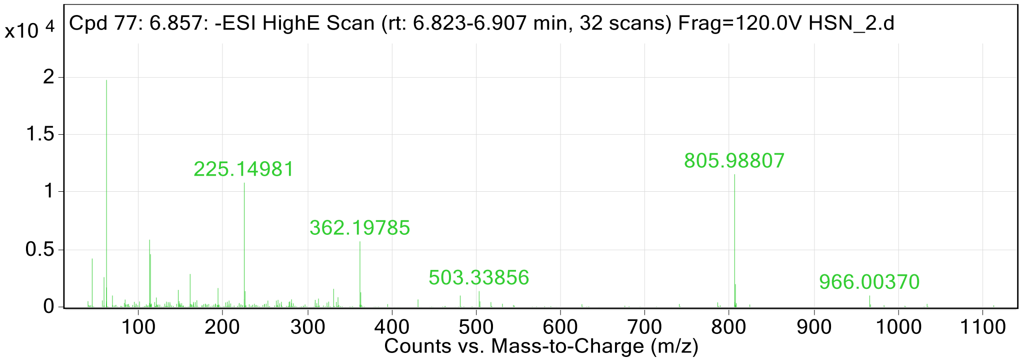

Peak List

m/z	z	Abund
44.99848		4215.2
59.01414		2595.44
61.98858		19762.96
112.98557		5856.54
114.0559	1	4592.9
161.02443	1	2870.89
225.14981	1	10820.22
362.19785	1	5710.11
805.98807	1	11552.34
806.99202	1	1983.61

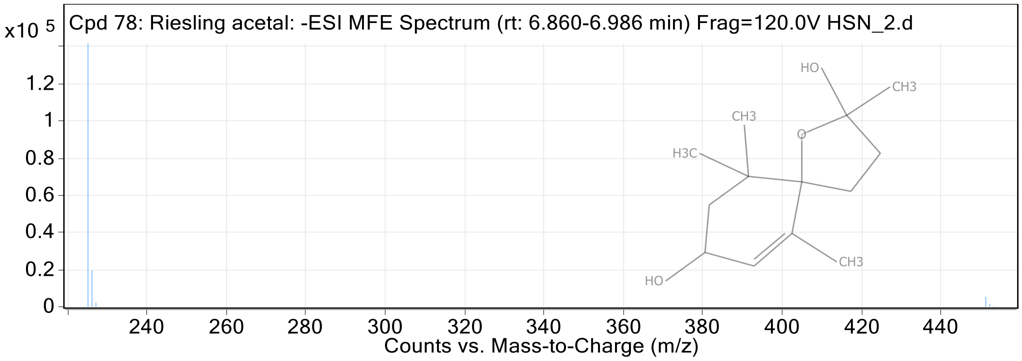

Peak List

m/z	z	Abund	Name	Formula	Ion	Score (DB)	Hits (DB)

225.14915	-1	141806.06	Riesling acetal	C13 H22 O3	(M-H)-	98.77	10
226.15306	-1	19714.66		C13 H22 O3	(M-H)-		
227.15555	-1	2258.29		C13 H22 O3	(M-H)-		
451.30646	-1	5284.64			(2M-H)-		
452.30843	-1	1420.73			(2M-H)-		
453.3101	-1	287.24			(2M-H)-		

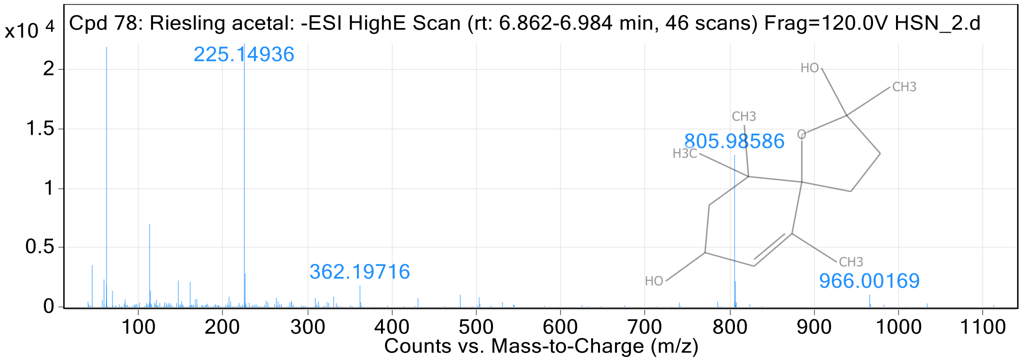

Peak List

m/z	z	Abund
44.99846		3529.09
59.0141		2324.94
61.98856		21920.31
112.98539		7009.24
146.96577		2227.31
161.02416		2105.11
225.14936	1	22216.84
226.15312	1	2825.46
805.98586	1	12828.69
806.99015	1	2175.96

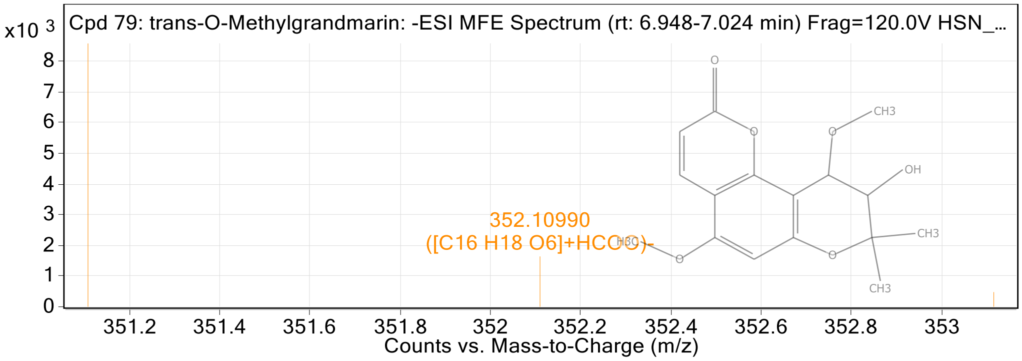

Peak List

m/z	z	Abund	Name	Formula	Ion	Score (DB)	Hits (DB)
351.10754	-1	8582.82	trans-O-Methylgrandmarin	C16 H18 O6	(M+HCOO)-	92.23	6
352.1099	-1	1640.36		C16 H18 O6	(M+HCOO)-		
353.11611	-1	478.79		C16 H18 O6	(M+HCOO)-		

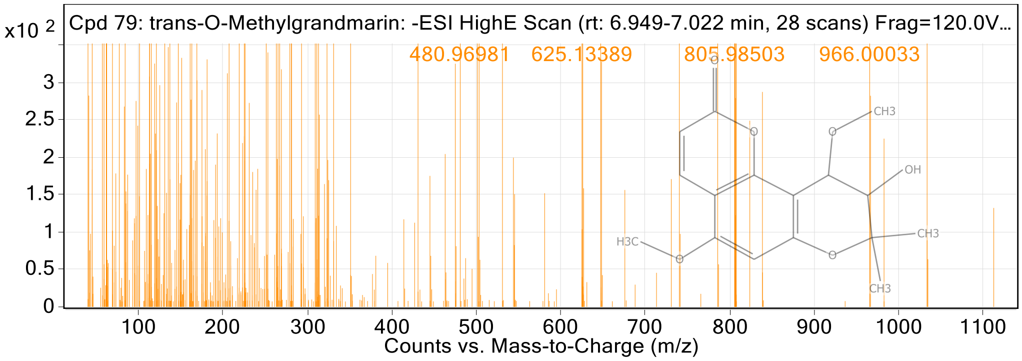

Peak List

m/z	z	Abund
44.9981		2936.55
59.01372		2341.5
61.98813		24205.71
62.00526		1701.66
112.98505	1	8021.11
146.96508		2250.62
161.02344	1	3140
225.14882	1	3059.08
805.98503	1	13700.3
806.98921	1	2278.83

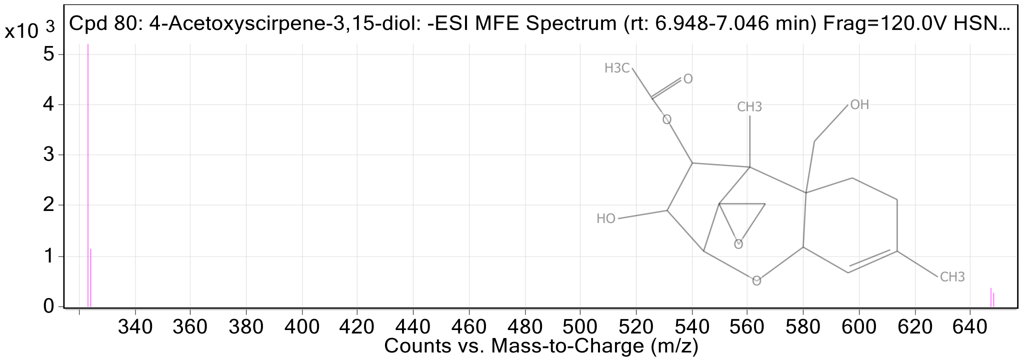

Peak List

m/z	z	Abund	Name	Formula	Ion	Score (DB)	Hits (DB)
323.14915	-1	5207.35	4-Acetoxyscirpene-3,15-diol	C17 H24 O6	(M-H)-	79.41	10
324.15217	-1	1148.11		C17 H24 O6	(M-H)-		
647.30705	-1	373.69			(2M-H)-		
648.3094	-1	277.26			(2M-H)-		

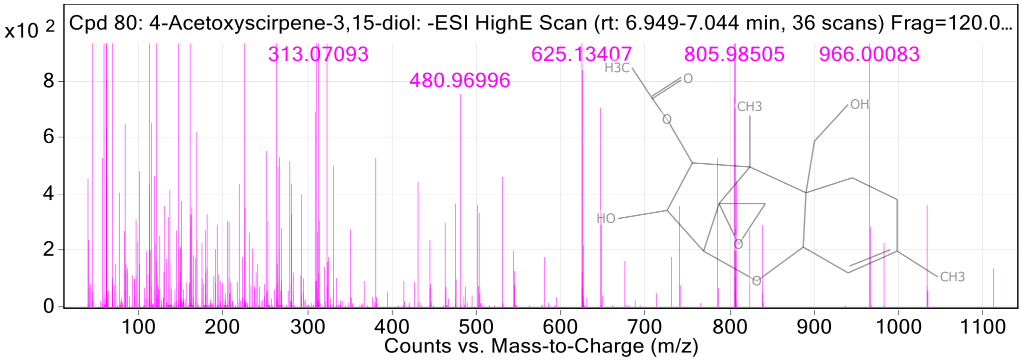

Peak List

m/z	z	Abund
44.99821		3012.4

59.01383		2191.1
61.98822		23598.98
112.98509		8212.25
146.96521		2336.9
161.0235	1	3736.9
225.14896	1	2639.12
625.13407	1	2311.51
805.98505	1	13755.03
806.989	1	2290.57

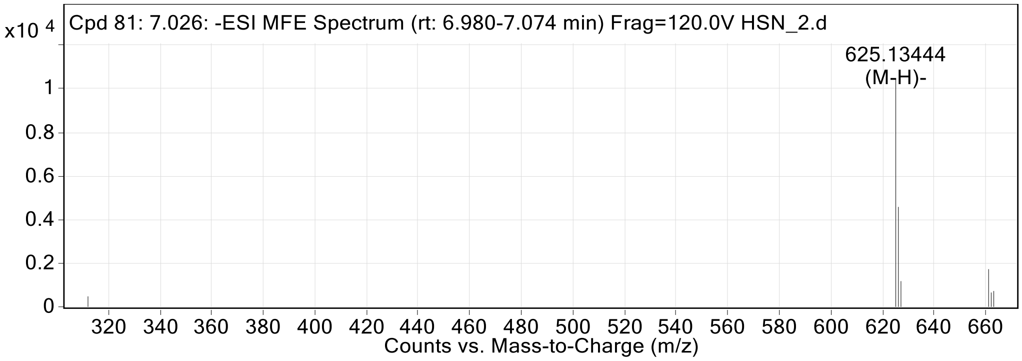

Peak List

m/z	z	Abund	Ion
312.0619	-2	479.27	(M-2H)-2
625.13444	-1	10479.06	(M-H)-
626.13725	-1	4582.73	(M-H)-
627.13764	-1	1178.47	(M-H)-
661.11106	-1	1727.36	(M+Cl)-
662.11273	-1	656.38	(M+Cl)-
663.11114	-1	725.32	(M+Cl)-

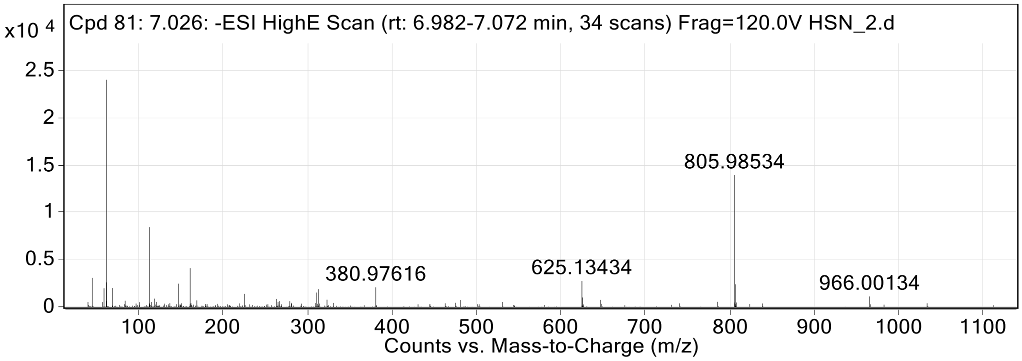

Peak List

m/z	z	Abund
44.99833		3067.97
61.98835		24041.92
62.00456		2576.02
112.98521		8419.97
146.96532		2446.79
161.02373	1	4093.64
380.97616		2054.12
625.13434	1	2735.59
805.98534	1	13934.93
806.98896	1	2373.61

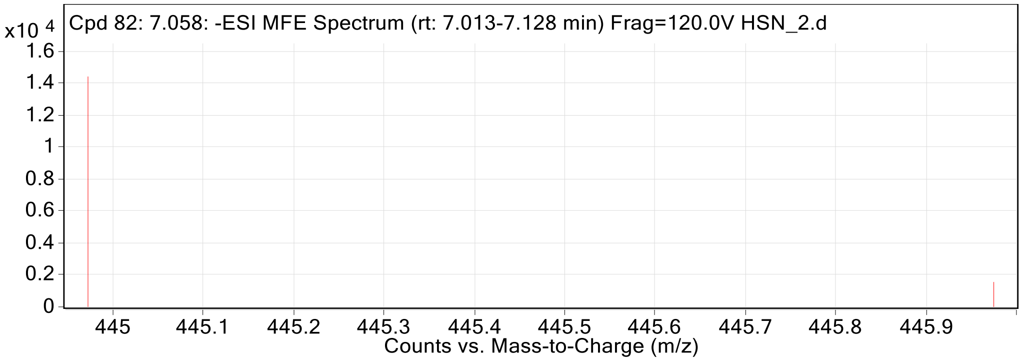

Peak List

m/z	z	Abund	Ion
444.97208	-1	14414.86	(M-H)-
445.97506	-1	1557.79	(M-H)-

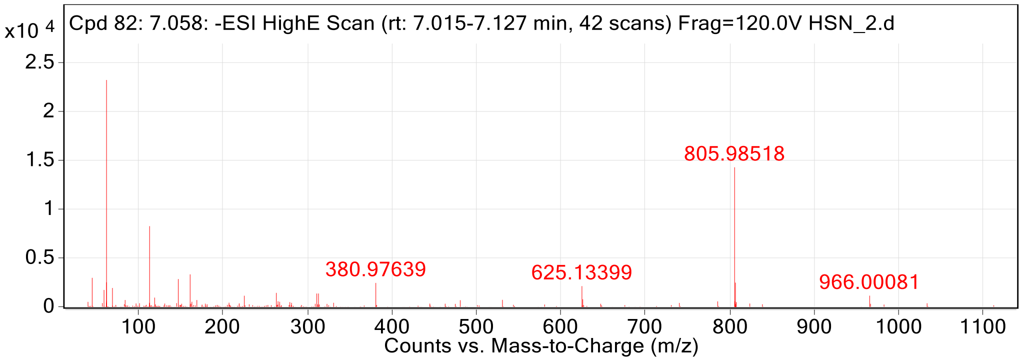

Peak List

m/z	z	Abund
44.99855		2962.56
61.98863		23252.29
62.0048		2503.51
112.98538		8263.35
146.96566		2833.46
161.02407	1	3317.79
380.97639	1	2440.09
625.13399	1	2112.77
805.98518	1	14300.47
806.98837	1	2469.51

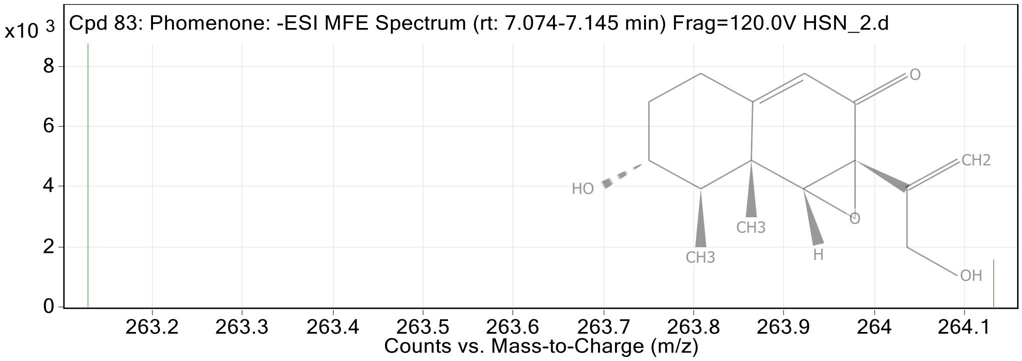

Peak List

m/z	z	Abund	Name	Formula	Ion	Score (DB)	Hits (DB)
263.12894	-1	8757.18	Phomenone	C15 H20 O4	(M-H)-	85.68	10
264.13234	-1	1579.8		C15 H20 O4	(M-H)-		

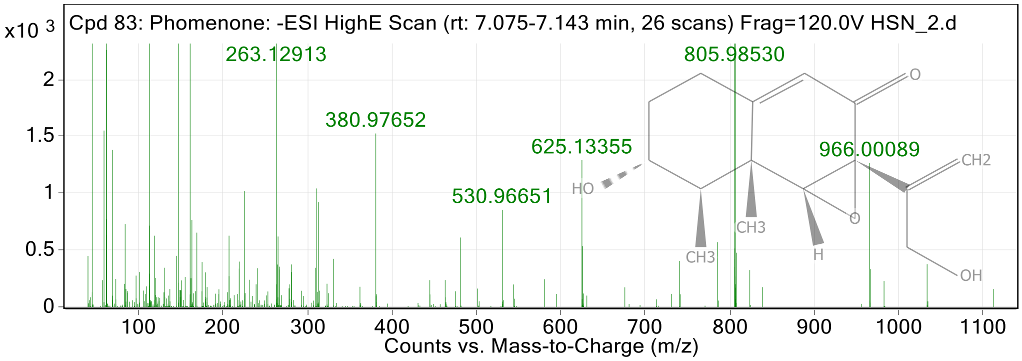

Peak List

m/z	z	Abund
44.99863		2666.87
59.0142		1550.53
61.98877		22592.52
62.00513		2259.16
112.98555		7809.34
146.96576		3044.14
161.02436	1	2480.27
263.12913	1	2316.82
805.9853	1	14727.46
806.98852	1	2554.32

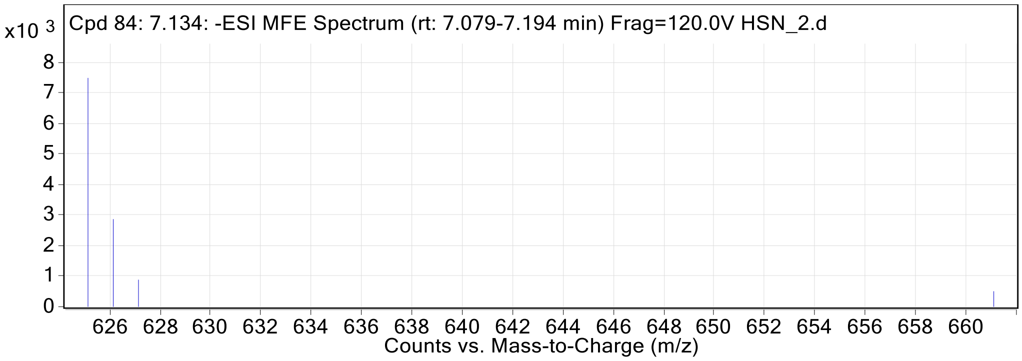

Peak List

m/z	z	Abund	Ion
625.13456	-1	7493.42	(M-H)-
626.13793	-1	2868.97	(M-H)-
627.14022	-1	886.17	(M-H)-
661.11326	-1	508.82	(M+Cl)-

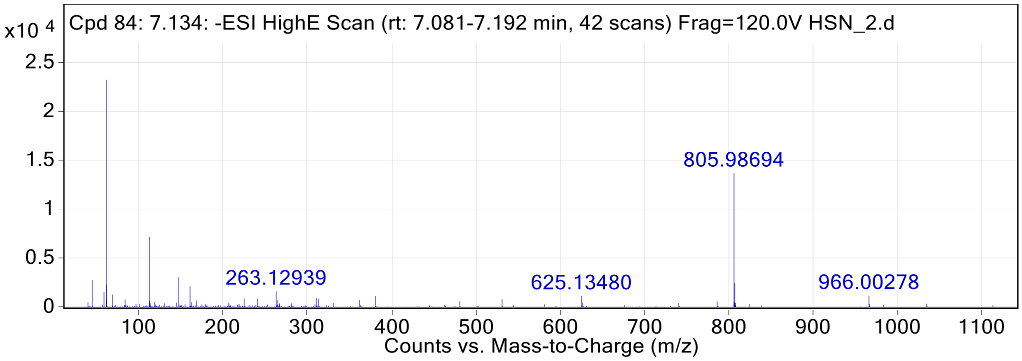

Peak List

m/z	z	Abund
44.99852		2751.9

59.0142		1506.52
61.98873		23192.85
62.00492		2272.84
112.98563		7157.94
146.96581		3016.87
161.02451		2098.5
263.12939	1	1575.64
805.98694	1	13646.71
806.98996	1	2402.79

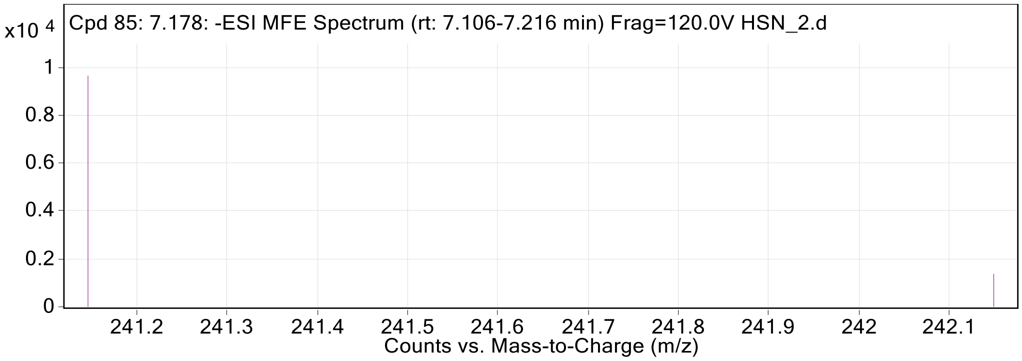

Peak List

m/z	z	Abund	Ion
241.14578	-1	9648.42	(M-H)-
242.1497	-1	1376.18	(M-H)-

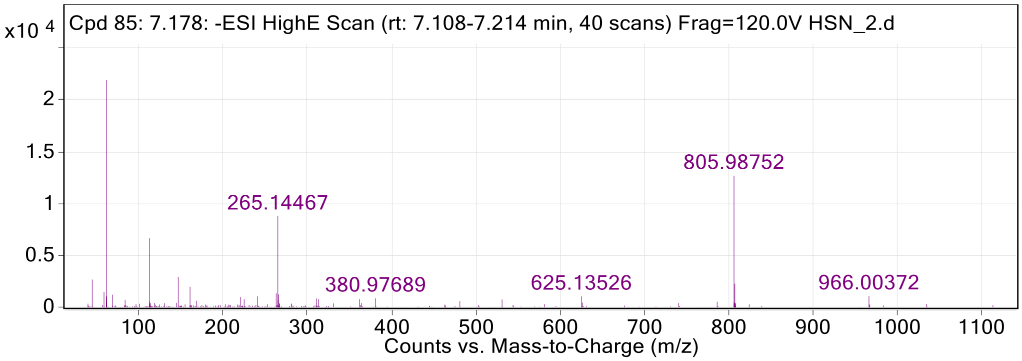

Peak List

m/z	z	Abund
44.99844		2655.28
59.01407		1454.79
61.98865		21917.97
112.98556		6652.02
146.96567		2916.4
161.02448	1	1956.55
263.12937	1	1329.32
265.14467	1	8769.4
805.98752	1	12684.99
806.99014	1	2255.16

Qualitative Analysis Report

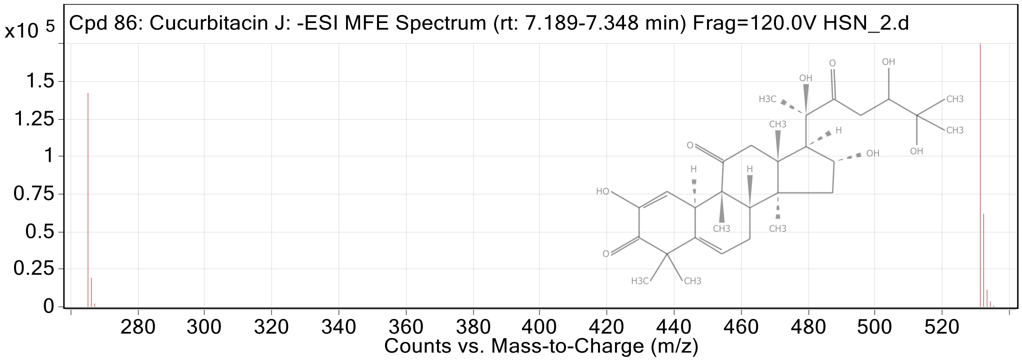

Peak List

m/z	z	Abund	Name	Formula	Ion	Score (DB)	Hits (DB)
265.14433	-1	142228.58			(M-H)-		
266.14738	-1	19352.13			(M-H)-		
267.15023	-1	2264.87			(M-H)-		
268.15216	-1	107.77			(M-H)-		
531.2945	-1	175123.61	Cucurbitacin J	C30 H44 O8	(M-H)-	93	10
532.29767	-1	61897.28		C30 H44 O8	(M-H)-		
533.30202	-1	11416.57		C30 H44 O8	(M-H)-		
534.30462	-1	3589.12		C30 H44 O8	(M-H)-		
535.31058	-1	890.63		C30 H44 O8	(M-H)-		

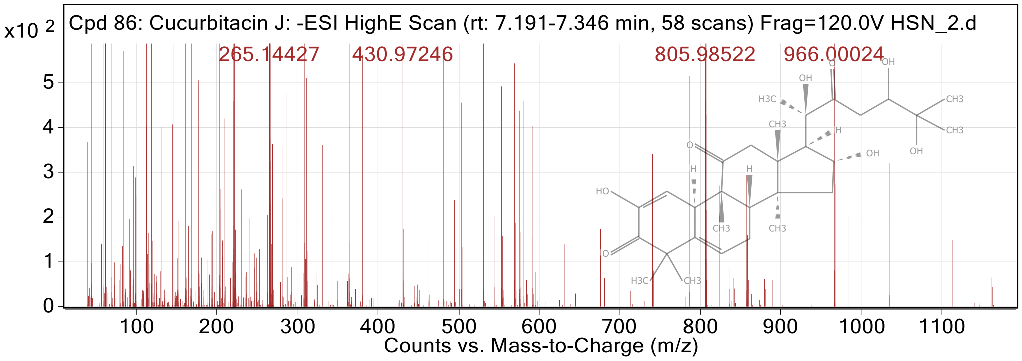

Peak List

m/z	z	Abund
44.99821		2289.54
61.98839		18010.2
112.98504		7177.82
146.96523		2277.22
221.15406	1	7078.77
265.14427	1	63938.97
265.28562	1	2101.04
266.14733	1	10607.37
805.98522	1	13669.42
806.98797	1	2386.24

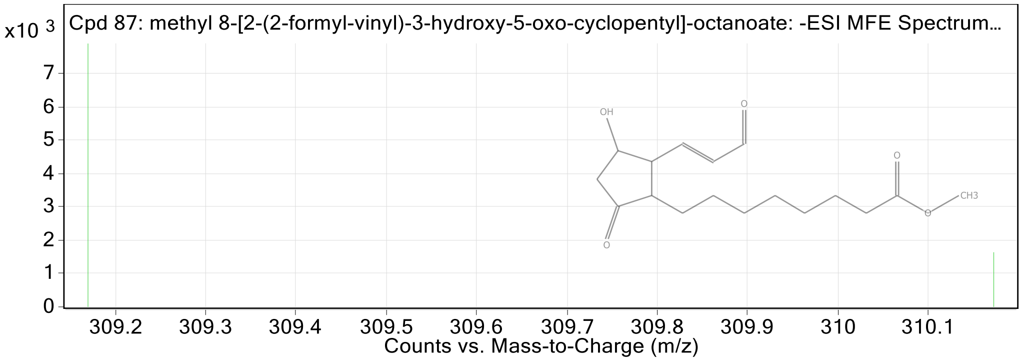

Peak List

m/z	z	Abund	Name	Formula	Ion	Score (DB)	Hits (DB)
309.1699	-1	7916.08	methyl 8-[2-(2-formyl-vinyl)-3-hydroxy-5-oxo-cyclopentyl]-octanoate	C17 H26 O5	(M-H)-	79.69	10
310.17232	-1	1641.32		C17 H26 O5	(M-H)-		

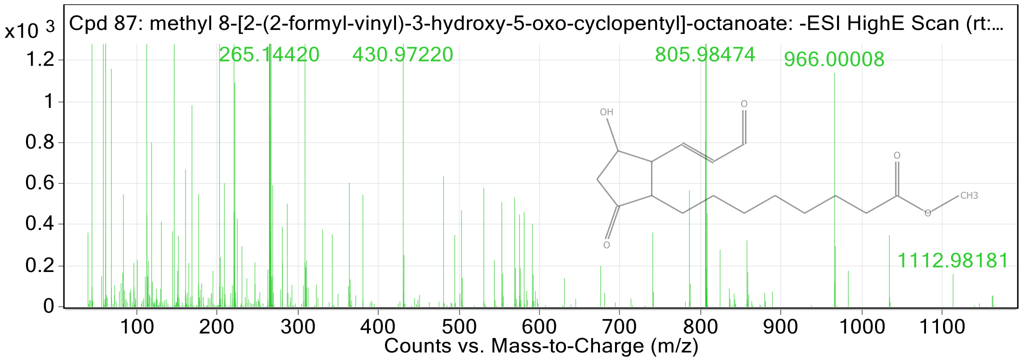

Peak List

m/z	z	Abund
61.98835		17913.42
112.98497	1	7616.73
146.96522		2285.34
221.15401	1	7982.82
265.1442	1	71702.02
265.28552	1	2747.29
266.1472	1	12131.39
430.9722	1	2541.47
805.98474	1	14531.94
806.98763	1	2514.58

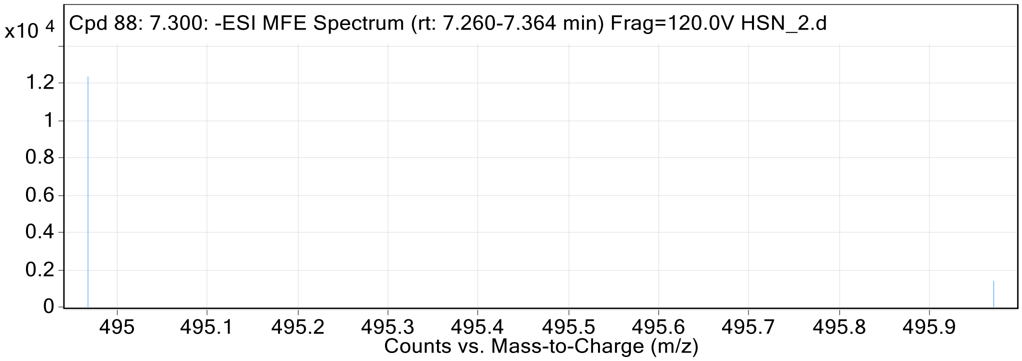

Peak List

m/z	z	Abund	Ion
494.96768	-1	12347.7	(M-H)-

495.97157	-1	1398.73	(M-H)-

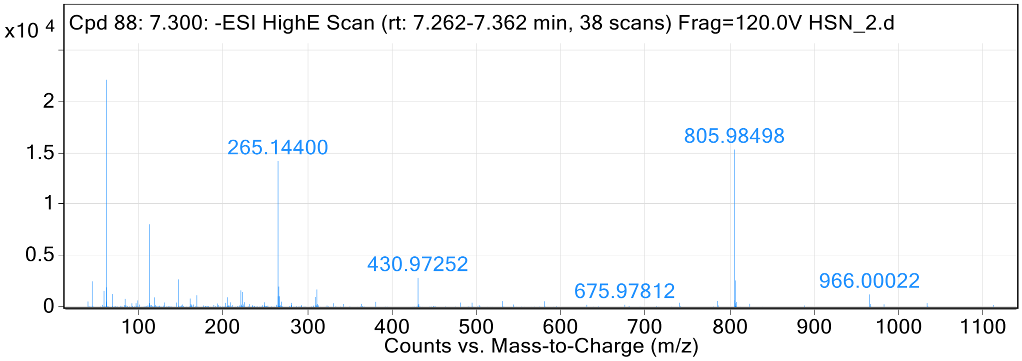

Peak List

m/z	z	Abund
44.99836		2470.01
61.98851		22121.05
62.00505		1883.58
112.98521	1	8026.94
146.96539		2659.82
265.144	1	14196.3
266.1475	1	1963.66
430.97252	1	2821.49
805.98498	1	15323.99
806.98811	1	2559.94

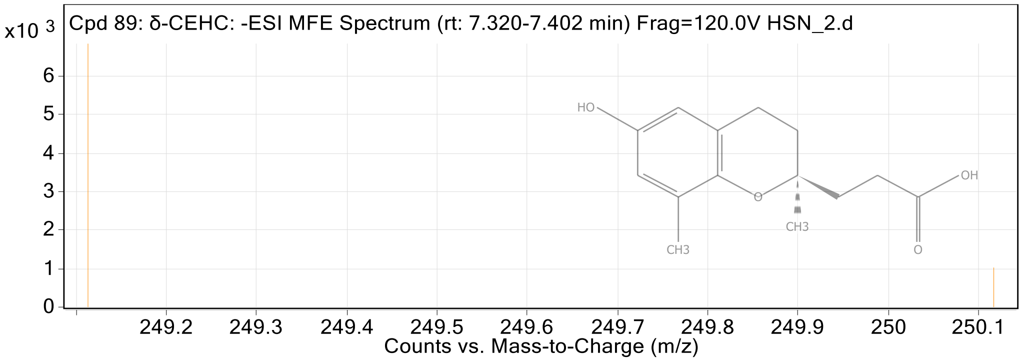

Peak List

m/z	z	Abund	Name	Formula	Ion	Score (DB)	Hits (DB)
249.11309	-1	6840.52	δ-CEHC	C14 H18 O4	(M-H)-	86.55	10
250.11678	-1	1026.78		C14 H18 O4	(M-H)-		

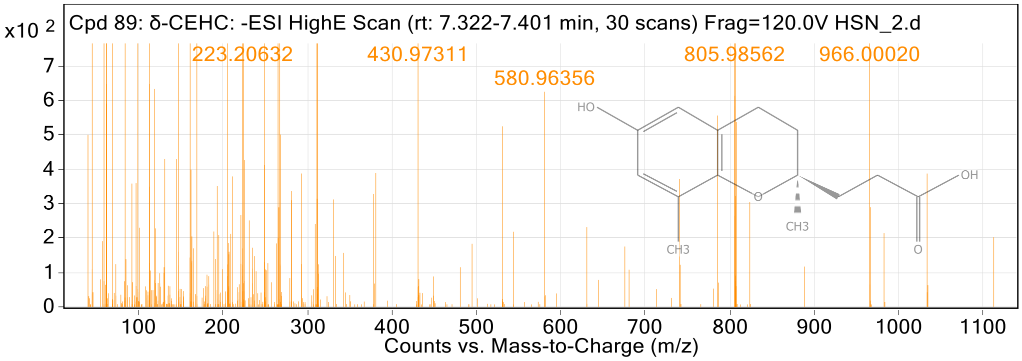

Peak List

m/z	z	Abund
44.99834		2518.57
61.98847		24566.08

62.00413		1745.8
112.98515		8000.61
146.96541		2552.77
223.20632	1	6098.35
267.19594	1	2830.33
311.18592	1	4634.2
805.98562	1	15859.86
806.98904	1	2638.06

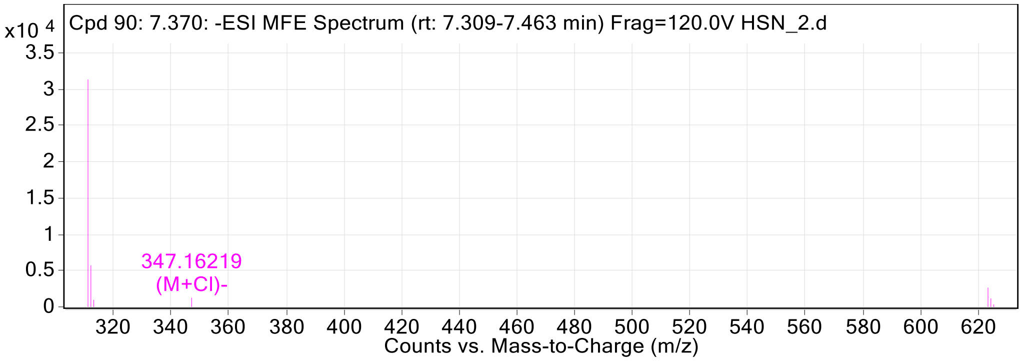

Peak List

m/z	z	Abund	Ion
311.18605	-1	31347.4	(M-H)-
312.18949	-1	5723.51	(M-H)-
313.19027	-1	950.13	(M-H)-
347.16219	-1	1259.27	(M+Cl)-
623.37842	-1	2649.46	(2M-H)-
624.37946	-1	1167.47	(2M-H)-
625.38408	-1	334.37	(2M-H)-

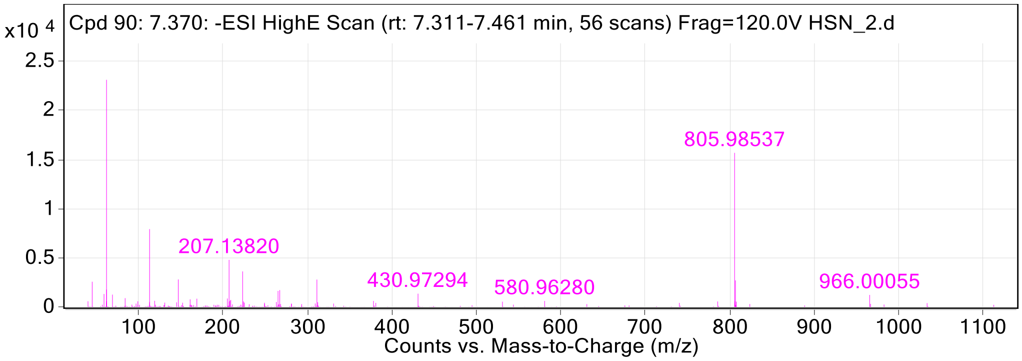

Peak List

m/z	z	Abund
44.9982		2558.08
61.9884		23122.96
62.00419		1760.91
112.98513		7915.8
146.96529		2789.99
207.1382	1	4785.3
223.20624	1	3607.49
311.18582	1	2777.85
805.98537	1	15683.76
806.98891	1	2693.21

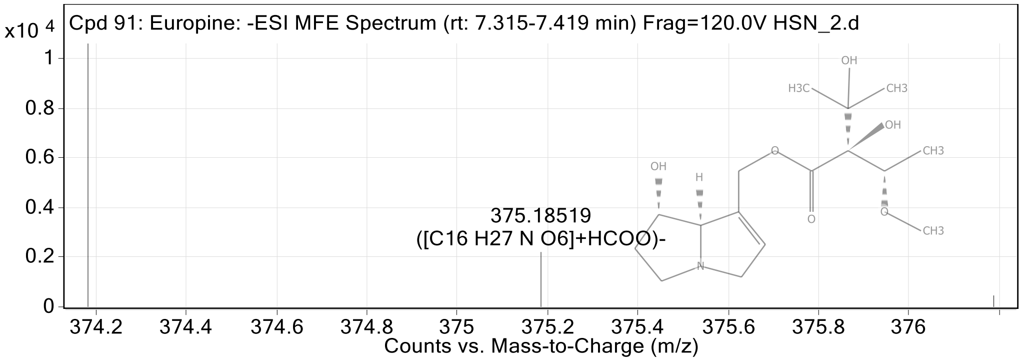

Peak List

m/z	z	Abund	Name	Formula	Ion	Score (DB)	Hits (DB)
374.18157	-1	10626.97	Europine	C16 H27 N O6	(M+HCOO)-	98.55	1
375.18519	-1	2213.47		C16 H27 N O6	(M+HCOO)-		
376.18828	-1	460.98		C16 H27 N O6	(M+HCOO)-		

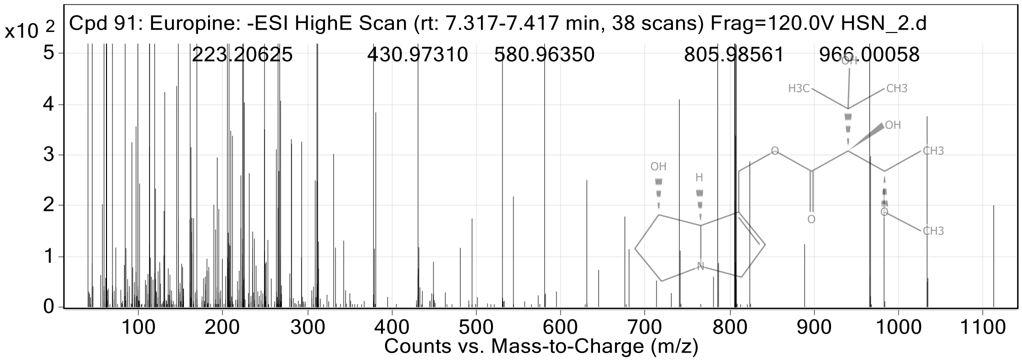

Peak List

m/z	z	Abund
44.9982		2560
61.98841		23982.44
62.00416		1830.73
112.98508		7931.05
146.96526		2644.99
223.20625	1	5080.69
267.19584	1	2367.56
311.18583	1	3851.4
805.98561	1	15632.31
806.98898	1	2609.58

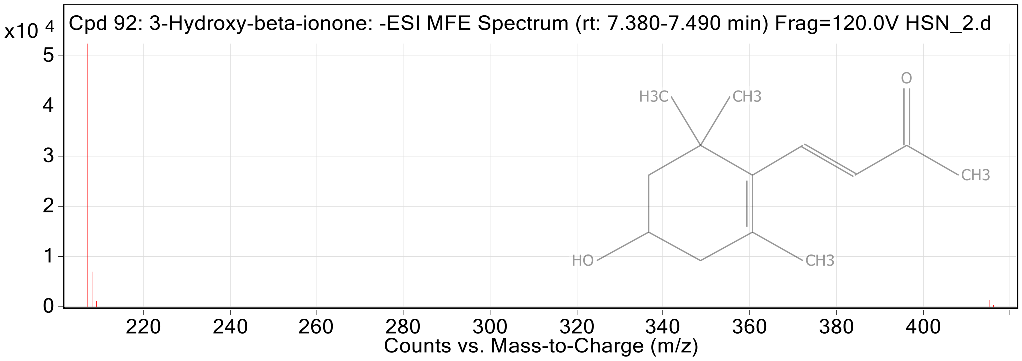

Peak List

m/z	z	Abund	Name	Formula	Ion	Score (DB)	Hits (DB)
207.13889	-1	52298.09	3-Hydroxy-beta-ionone	C13 H20 O2	(M-H)-	98.84	10
208.14198	-1	6966.79		C13 H20 O2	(M-H)-		

209.14734	-1	1140.05		C13 H20 O2	(M-H)-		
415.28603	-1	1380.31			(2M-H)-		
416.28913	-1	311.78			(2M-H)-		

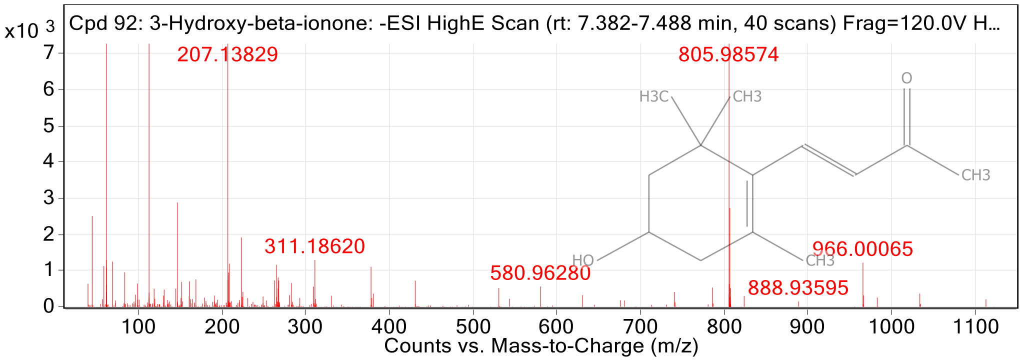

Peak List

m/z	z	Abund
44.99818		2510.39
61.98838		22884.93
62.00601		1291.23
112.98517		7804.14
146.96532		2883.6
207.13829	1	7280.72
223.20649	1	1919.28
311.1862	1	1294.9
805.98574	1	15540.08
806.98915	1	2728.18

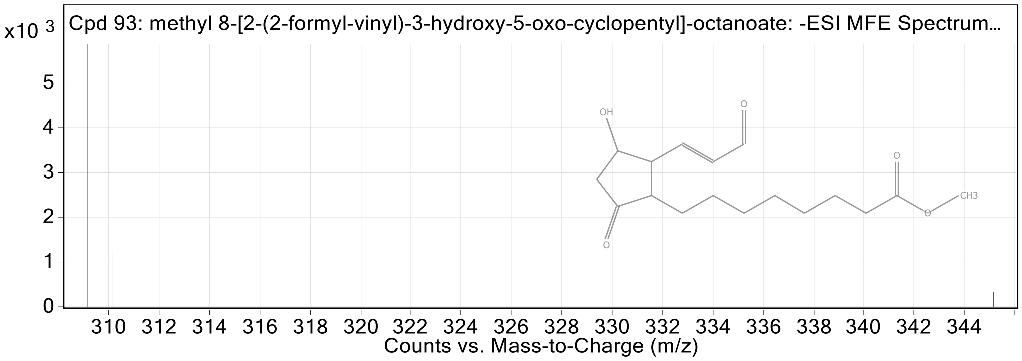

Peak List

m/z	z	Abund	Name	Formula	Ion	Score (DB)	Hits (DB)
309.17075	-1	5887.74	methyl 8-[2-(2-formyl-vinyl)-3-hydroxy-5-oxo-cyclopentyl]-octanoate	C17 H26 O5	(M-H)-	82.42	4
310.17333	-1	1269.32		C17 H26 O5	(M-H)-		
345.14859	-1	332.73			(M+Cl)-		

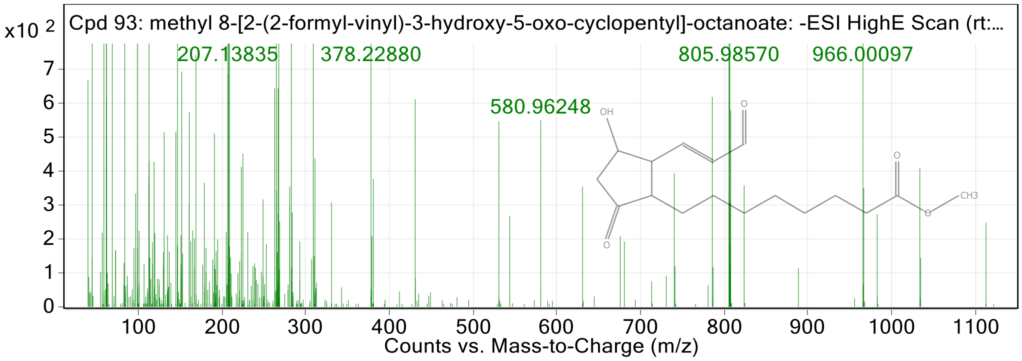

Peak List

m/z	z	Abund
44.99837		2568.37
61.98844		23029.24
62.00488		2304.42
112.98526		8984.65
146.96551		2898.87
207.13835	1	6317.09
268.03756	1	1535.27
283.06094	1	1460.23
805.9857	1	17888.35
806.98893	1	3103.09

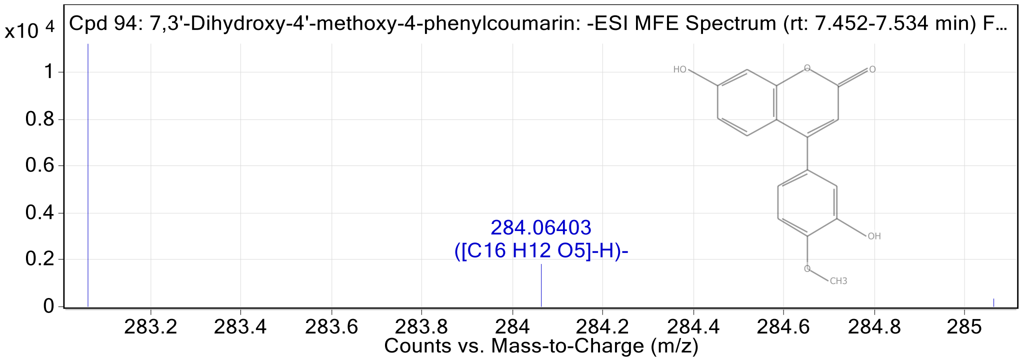

Peak List

m/z	z	Abund	Name	Formula	Ion	Score (DB)	Hits (DB)
283.06112	-1	11233.25	7,3'-Dihydroxy-4'-methoxy-4-phenylcoumarin	C16 H12 O5	(M-H)-	98.58	10
284.06403	-1	1826.47		C16 H12 O5	(M-H)-		
285.06486	-1	349.25		C16 H12 O5	(M-H)-		

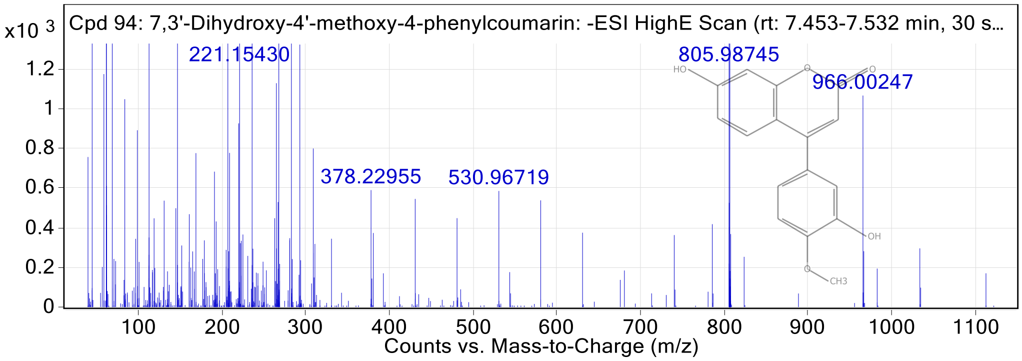

Peak List

m/z	z	Abund
44.99846		2478.57

61.9885		22406.73
62.00484		2056.04
68.9958		1904.13
112.98537		7679.09
146.96573		2602.66
207.13879	1	2147.17
221.1543	1	2342.62
805.98745	1	13296.02
806.99103	1	2258.66

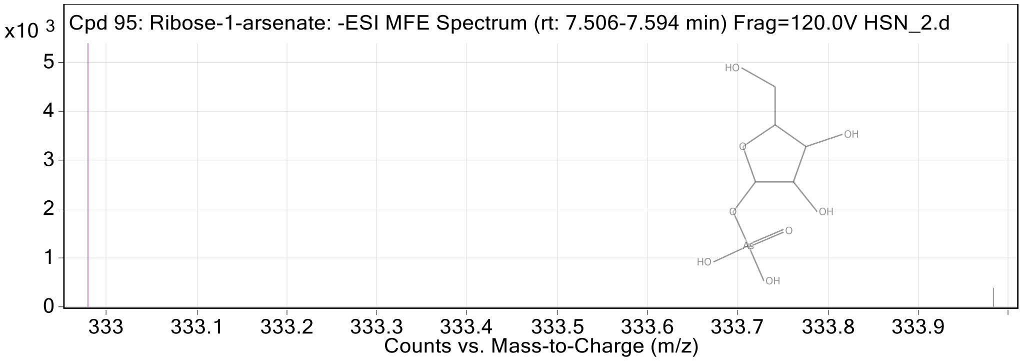

Peak List

m/z	z	Abund	Name	Formula	Ion	Score (DB)	Hits (DB)
332.9796	-1	5382.67	Ribose-1-arsenate	C5 H11 As O8	(M+CH3COO)-	80.16	1
333.98391	-1	385.94		C5 H11 As O8	(M+CH3COO)-		

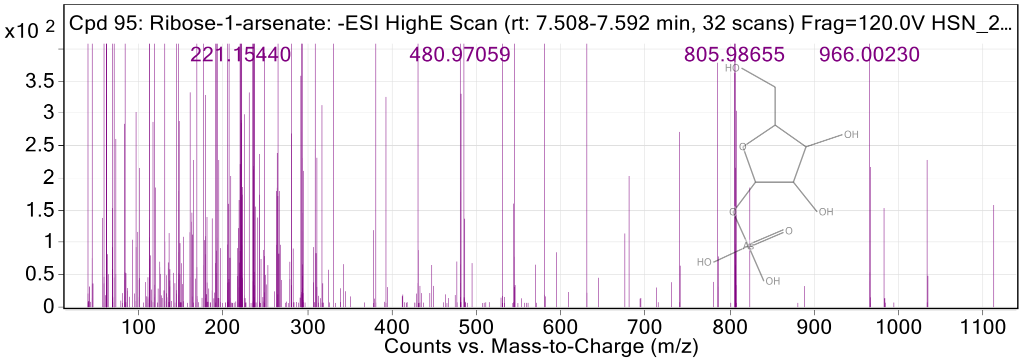

Peak List

m/z	z	Abund
61.98862		20952.9
68.99597		2659.92
112.98538		8059.15
146.96556		2270.62
220.14681		4362.93
221.1544	1	11331.55
236.10535	1	9611.45
293.17592	1	8864.58
480.97059	1	3074.99
805.98655	1	10473.73

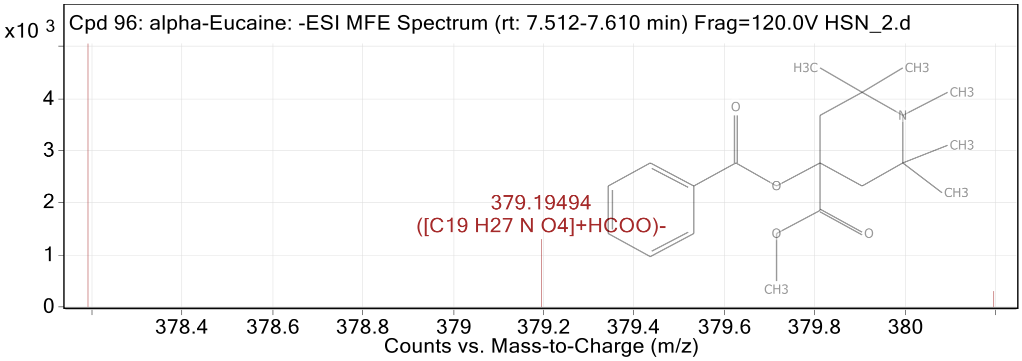

Peak List

m/z	z	Abund	Name	Formula	Ion	Score (DB)	Hits (DB)
378.19169	-1	5060.35	alpha-Eucaine	C19 H27 N O4	(M+HCOO)-	95.83	3
379.19494	-1	1302.9		C19 H27 N O4	(M+HCOO)-		
380.19638	-1	304.02		C19 H27 N O4	(M+HCOO)-		

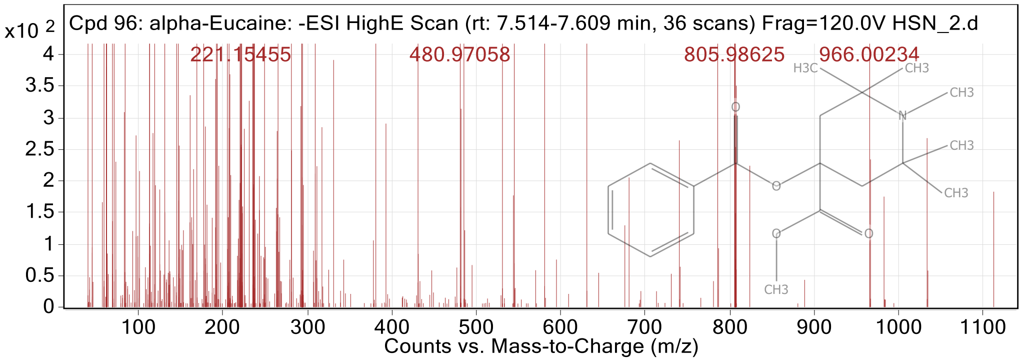

Peak List

m/z	z	Abund
61.98873		20520.47
68.99612		2554.52
112.9855		8405.25
207.13883	1	2958.43
220.14692		3941.93
221.15455	1	10208.52
236.1055	1	8673.53
293.17603	1	8284.77
480.97058	1	2905.42
805.98625	1	11667.79

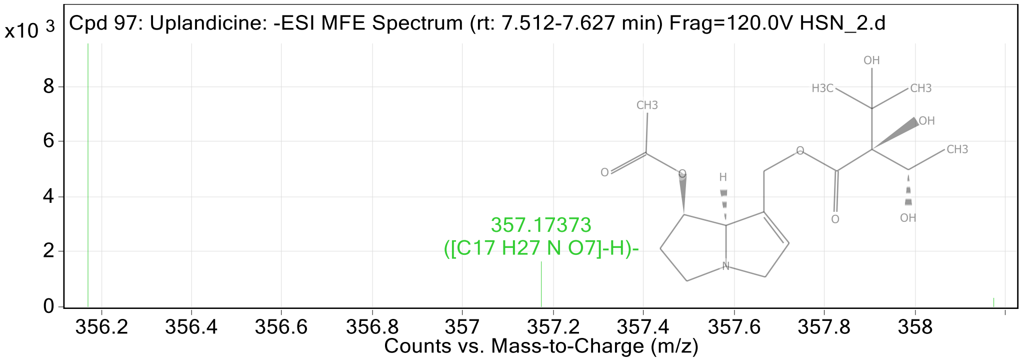

Peak List

m/z	z	Abund	Name	Formula	Ion	Score (DB)	Hits (DB)
356.17088	-1	9561.49	Uplandicine	C17 H27 N O7	(M-H)-	96.98	2
357.17373	-1	1649.51		C17 H27 N O7	(M-H)-		

358.17454	-1	327.38		C17 H27 N O7	(M-H)-		

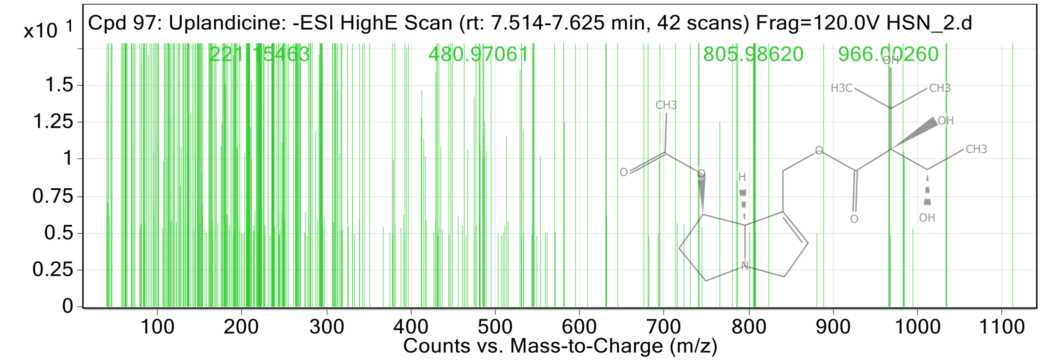

Peak List

m/z	z	Abund
61.98881		20016.34
68.9962		2389.46
112.98561		8400.75
207.13908	1	7841.62
220.14702		3430.6
221.15463	1	8874.66
236.10558	1	7512.95
293.17611	1	7255.46
480.97061	1	2621.6
805.9862	1	12213.27

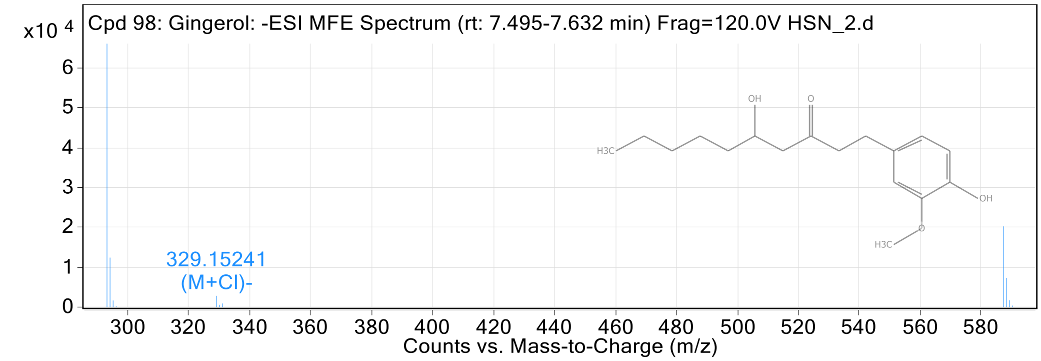

Peak List

m/z	z	Abund	Name	Formula	Ion	Score (DB)	Hits (DB)
293.17641	-1	66211.11	Gingerol	C17 H26 O4	(M-H)-	98.56	7
294.1791	-1	12429.41		C17 H26 O4	(M-H)-		
295.18113	-1	1658.81		C17 H26 O4	(M-H)-		
329.15241	-1	2829.88			(M+Cl)-		
330.15658	-1	568.32			(M+Cl)-		
331.15033	-1	902.93			(M+Cl)-		
587.35875	-1	20279.79			(2M-H)-		
588.36194	-1	7345.79			(2M-H)-		
589.36578	-1	1721.58			(2M-H)-		
590.3645	-1	382.2			(2M-H)-		

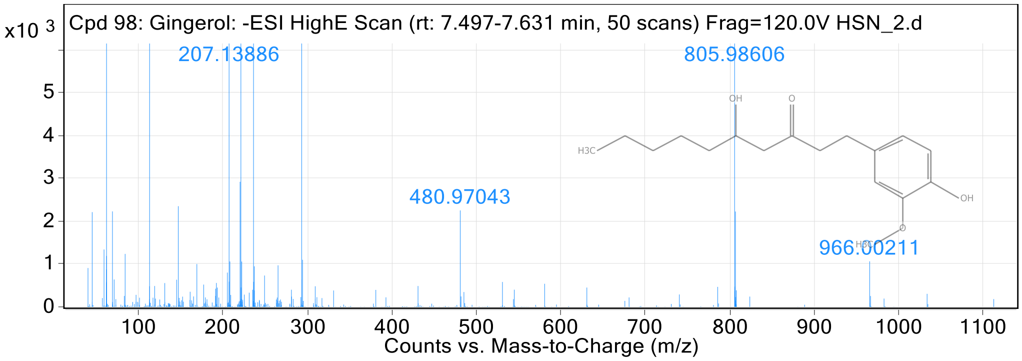

Peak List

m/z	z	Abund
61.9887		20309.55
112.98546		8316.89
146.96565		2350.16
207.13886	1	8458.12
220.14684		2920.25
221.15444	1	7560.04
236.10538	1	6354.83
293.17592	1	6149.15
480.97043	1	2251.39
805.98606	1	12837.71

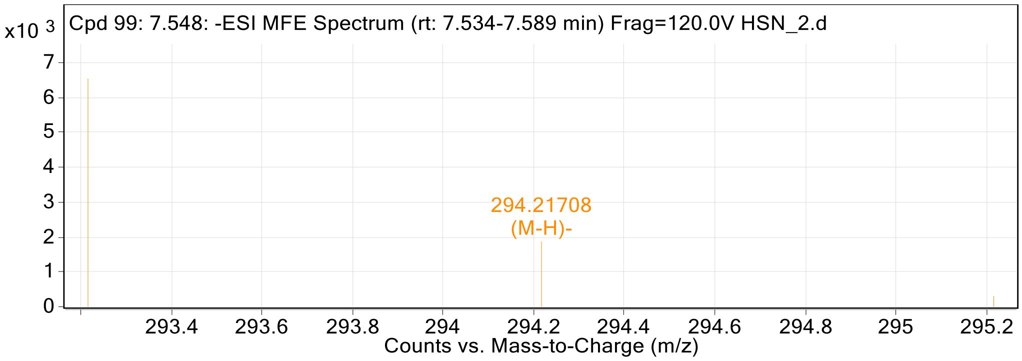

Peak List

m/z	z	Abund	Ion
293.21534	-1	6544.92	(M-H)-
294.21708	-1	1871.38	(M-H)-
295.21621	-1	304.3	(M-H)-

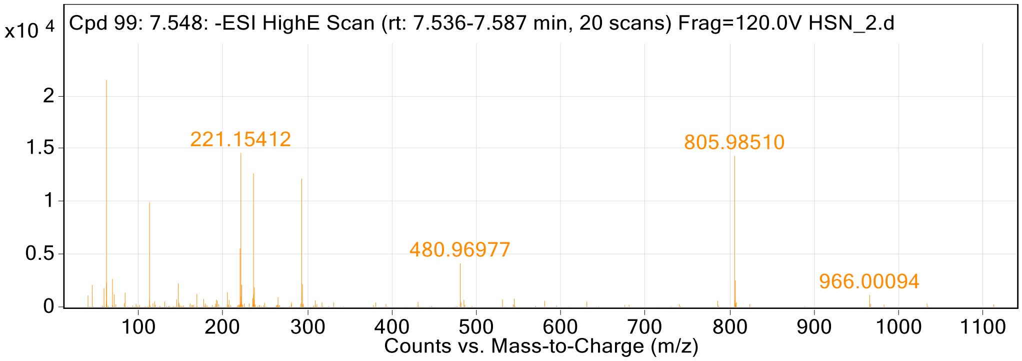

Peak List

m/z	z	Abund
61.98858		21513.34
68.99596		2655.26

112.98529		9925.82
220.14651		5550.77
221.15412	1	14589.42
236.10505	1	12648.91
293.17547	1	12148.8
480.96977	1	4131.16
805.9851	1	14309.67
806.98845	1	2514.16

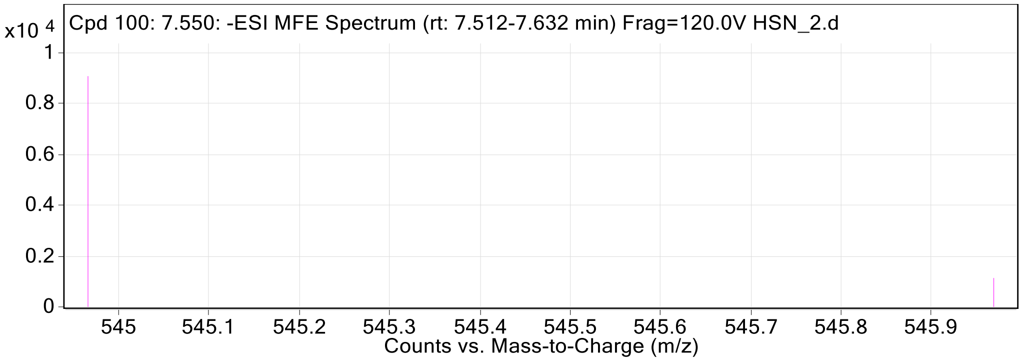

Peak List

m/z	z	Abund	Ion
544.96589	-1	9079.26	(M-H)-
545.96896	-1	1135.99	(M-H)-

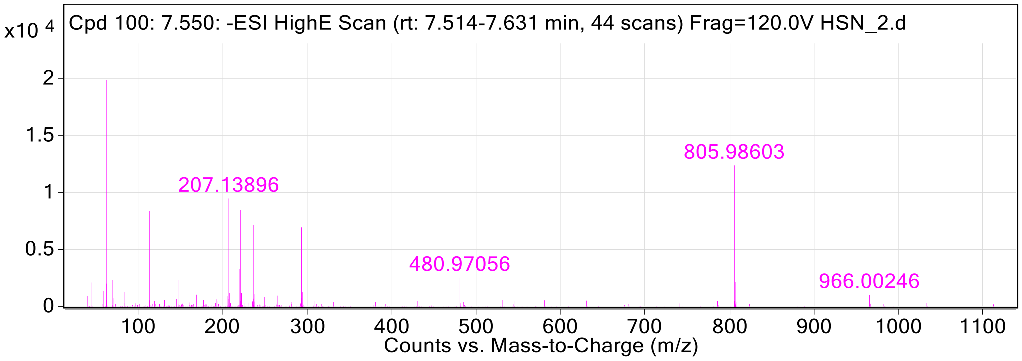

Peak List

m/z	z	Abund
61.98876		19953.86
68.99614		2347.2
112.98554		8385.02
207.13896	1	9505.16
220.14694		3288.89
221.15454	1	8511.75
236.10549	1	7191.91
293.17602	1	6961.63
480.97056	1	2542.24
805.98603	1	12416.71

Qualitative Analysis Report

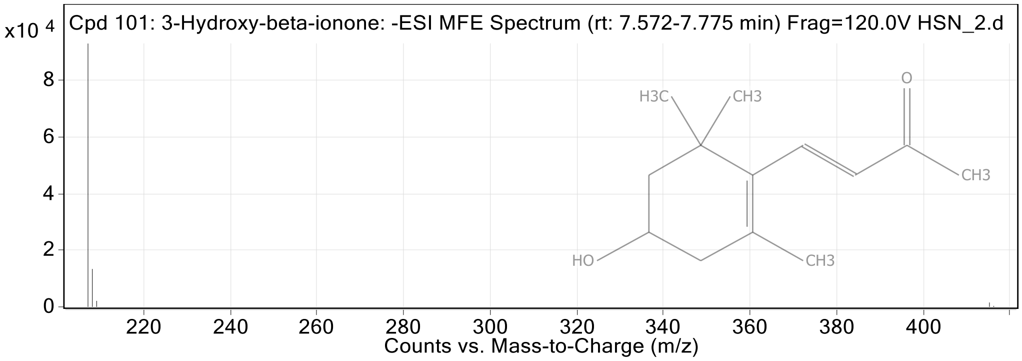

Peak List

m/z	z	Abund	Name	Formula	Ion	Score (DB)	Hits (DB)
207.13869	-1	93162.17	3-Hydroxy-beta-ionone	C13 H20 O2	(M-H)-	99.11	10
208.14218	-1	13407.6		C13 H20 O2	(M-H)-		
209.14511	-1	2124.34		C13 H20 O2	(M-H)-		
415.28512	-1	1551.56			(2M-H)-		
416.29034	-1	347.41			(2M-H)-		

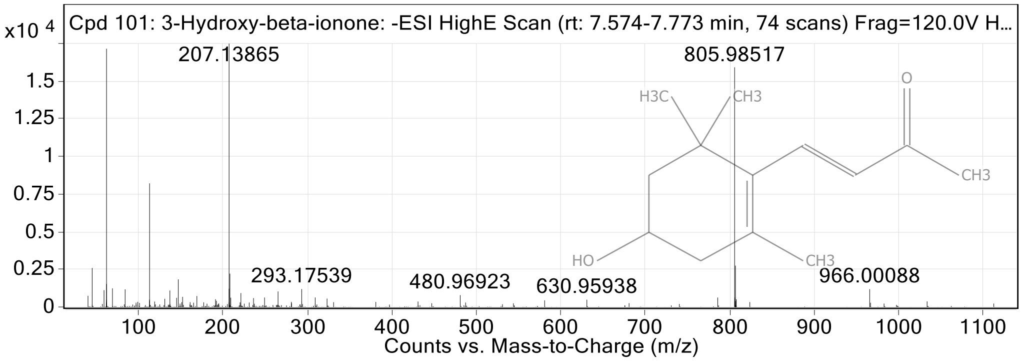

Peak List

m/z	z	Abund
44.99845		2589.41
61.98852		17158.57
62.00501		1523.5
68.9959		1238.13
112.98526		8211.63
146.96539		1834.02
207.13865	1	17504.45
208.14217	1	2213.7
805.98517	1	15921.39
806.98838	1	2744.61

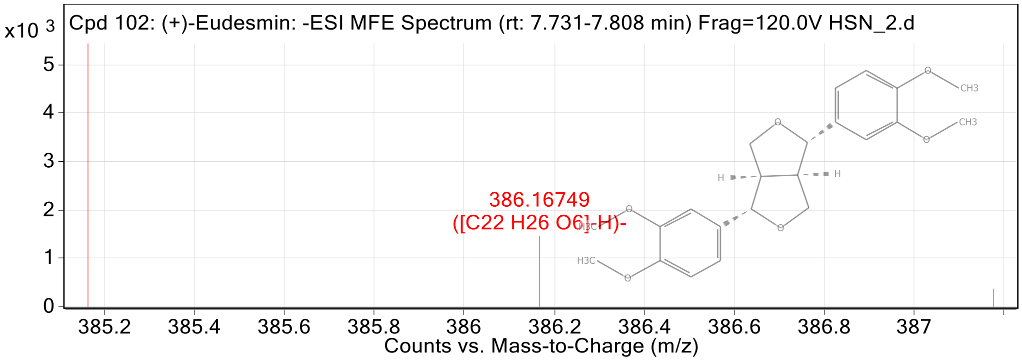

Peak List

m/z	z	Abund	Name	Formula	Ion	Score (DB)	Hits (DB)

385.16433	-1	5438.5	(+)-Eudesmin	C22 H26 O6	(M-H)-	88.67	10
386.16749	-1	1458.05		C22 H26 O6	(M-H)-		
387.17625	-1	373.71		C22 H26 O6	(M-H)-		

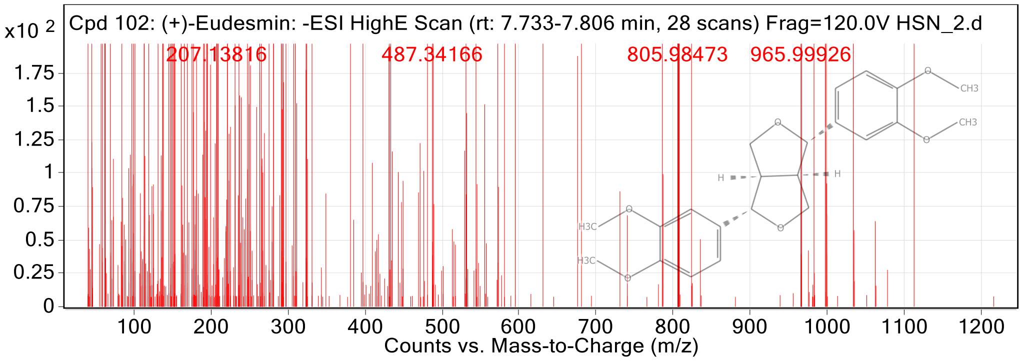

Peak List

m/z	z	Abund
44.99817		2775.33
59.01362		1416.61
61.98818		18291.65
112.98483		7876.16
137.02355	1	3389.6
207.13816	1	4379.71
323.18569	1	2705.6
487.34166	1	1806.45
805.98473	1	16299.71
806.98791	1	2769.43

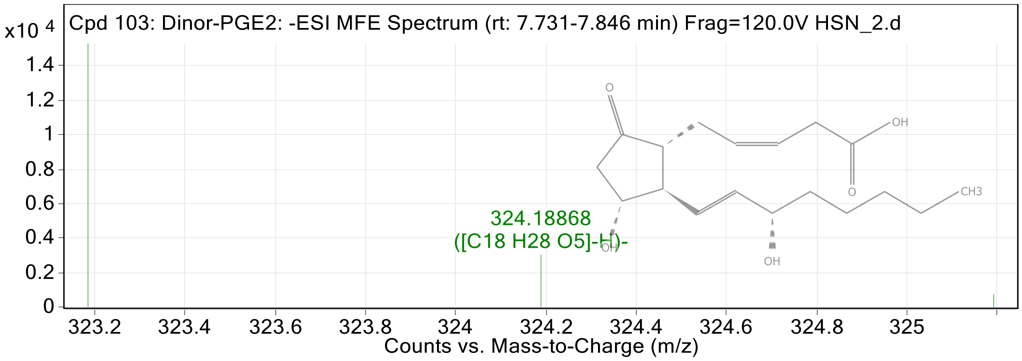

Peak List

m/z	z	Abund	Name	Formula	Ion	Score (DB)	Hits (DB)
323.18491	-1	15291.21	Dinor-PGE2	C18 H28 O5	(M-H)-	92.51	5
324.18868	-1	3031.88		C18 H28 O5	(M-H)-		
325.19209	-1	746.37		C18 H28 O5	(M-H)-		

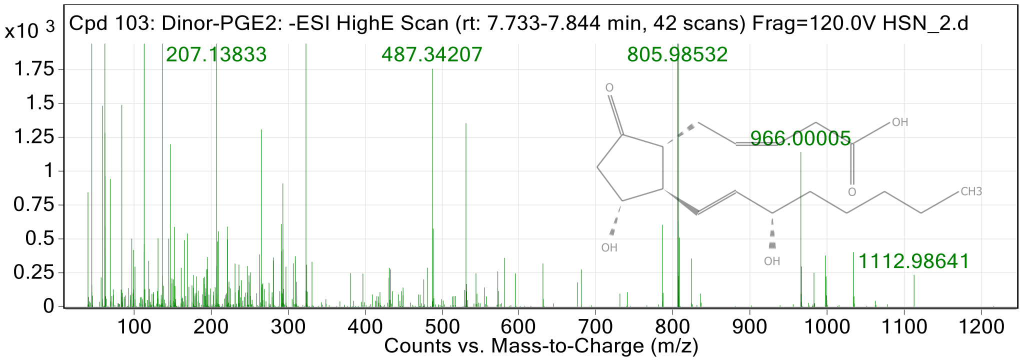

Peak List

m/z	z	Abund
44.99815		3131.91
61.98821		23259.19
84.00861		1490.35
112.98493		8115.06
137.02364		2351.96
207.13833	1	3580.76
323.18598	1	1942.61
487.34207	1	1757.01
805.98532	1	16665.65
806.98837	1	2817.47

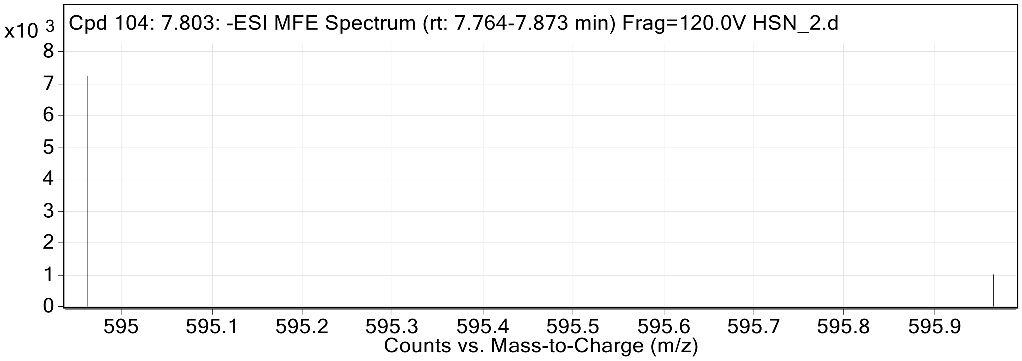

Peak List

m/z	z	Abund	Ion
594.96249	-1	7249.76	(M-H)-
595.96521	-1	1008.45	(M-H)-

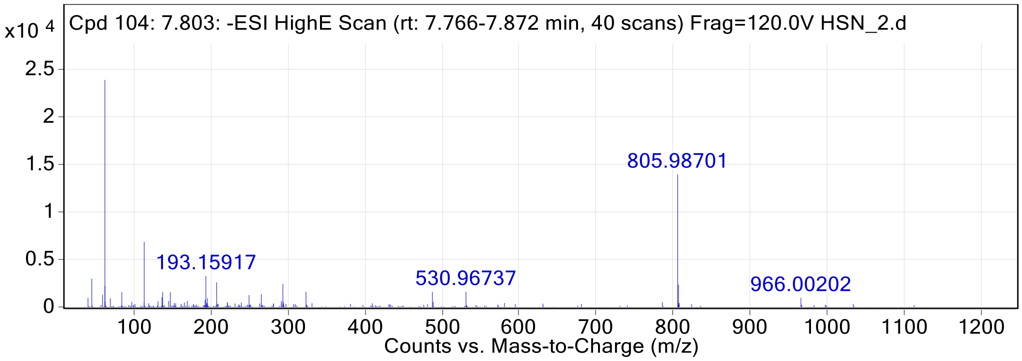

Peak List

m/z	z	Abund
44.99828		2988.27
61.98834		23942.01
62.0049		2212.92
112.98514		6866.35
193.15917	1	3270.1
207.13884	1	2597.88
293.17577	1	2423.98
323.18667	1	1601.77
805.98701	1	13980.43
806.98999	1	2348.57

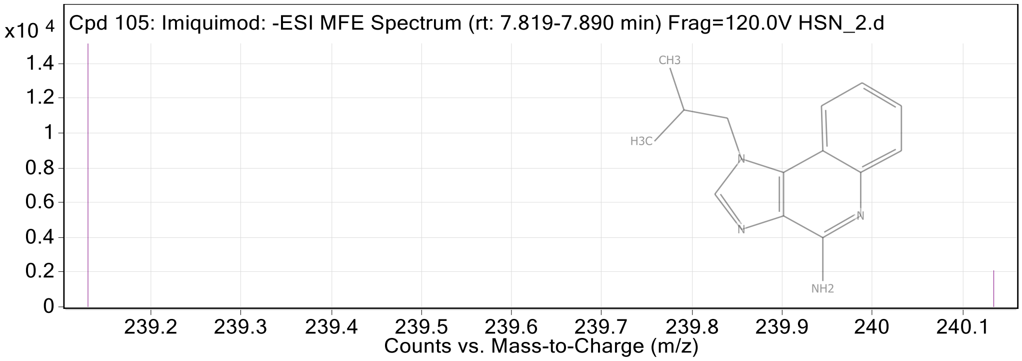

Peak List

m/z	z	Abund	Name	Formula	Ion	Score (DB)	Hits (DB)
239.13023	-1	15162.27	Imiquimod	C14 H16 N4	(M-H)-	81.96	1
240.13461	-1	2102.17		C14 H16 N4	(M-H)-		

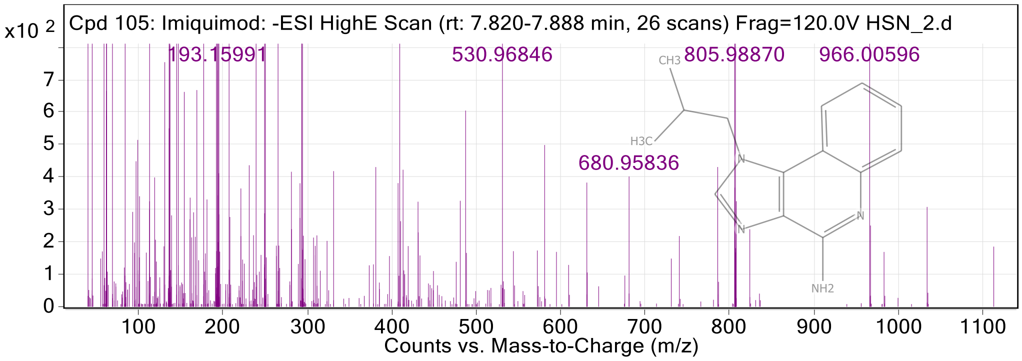

Peak List

m/z	z	Abund
44.99838		2553.11
61.98847		24175.11
112.98547		6199.8
136.08929	1	4859.09
192.11575	1	3635.09
193.15991	1	16866.37
207.13932	1	2198.02
249.18631	1	6043.42
293.17626	1	10860.86
805.9887	1	12265.16

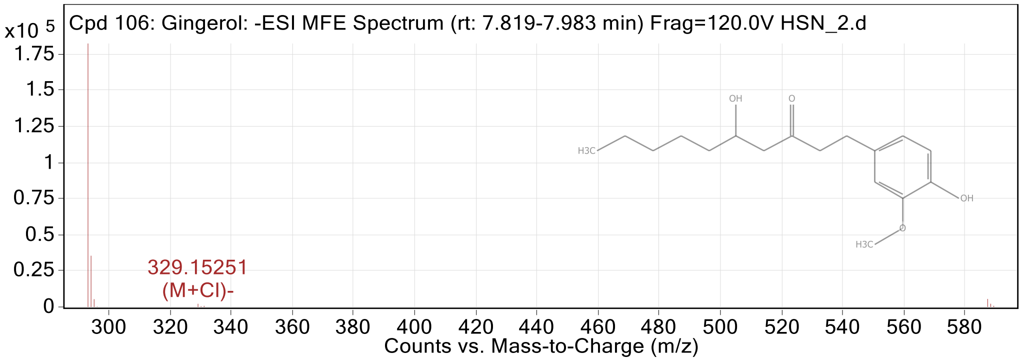

Peak List

m/z	z	Abund	Name	Formula	Ion	Score (DB)	Hits (DB)
293.17675	-1	182194.91	Gingerol	C17 H26 O4	(M-H)-	95.82	7
294.17882	-1	35442.16		C17 H26 O4	(M-H)-		
295.18084	-1	5397.45		C17 H26 O4	(M-H)-		

296.18301	-1	227.3		C17 H26 O4	(M-H)-		
329.15251	-1	2196.79			(M+Cl)-		
330.15479	-1	507.06			(M+Cl)-		
331.14871	-1	772.37			(M+Cl)-		
587.35756	-1	5558.48			(2M-H)-		
588.36077	-1	2231.77			(2M-H)-		
589.35358	-1	801.64			(2M-H)-		

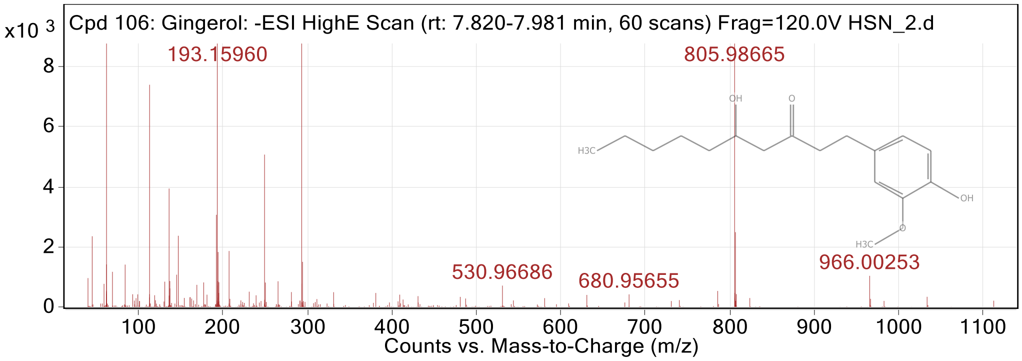

Peak List

m/z	z	Abund
61.9885		19671.14
112.98534		7404.92
136.08913	1	3946.19
146.96561		2371.62
192.11527	1	3070.83
193.1596	1	14086.83
249.18587	1	5077.34
293.17579	1	8782.56
805.98665	1	14383.92
806.99026	1	2490.93

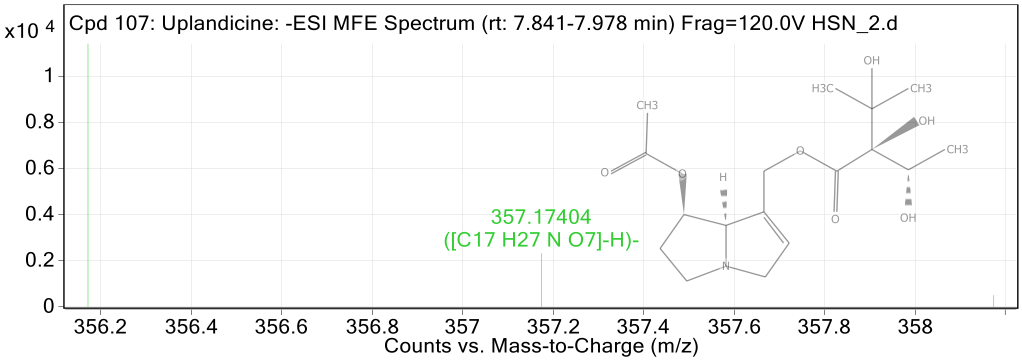

Peak List

m/z	z	Abund	Name	Formula	Ion	Score (DB)	Hits (DB)
356.17108	-1	11394.16	Uplandicine	C17 H27 N O7	(M-H)-	98.09	2
357.17404	-1	2316.51		C17 H27 N O7	(M-H)-		
358.17477	-1	501.22		C17 H27 N O7	(M-H)-		

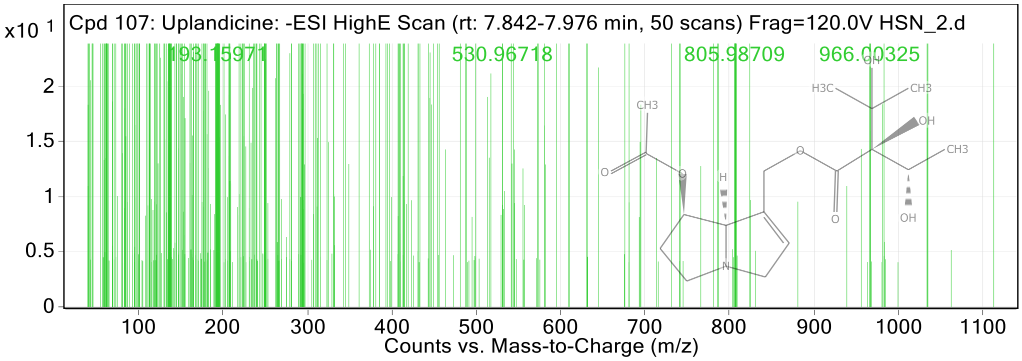

Peak List

m/z	z	Abund
61.98856		17386.79
112.98541		6836.65
136.08922	1	4710.34
146.96571		2418.1
192.11539	1	3659.47
193.15971	1	16807.13
249.18601	1	6026.64
293.17594	1	10400.99
805.98709	1	13129.63
806.99073	1	2306.2

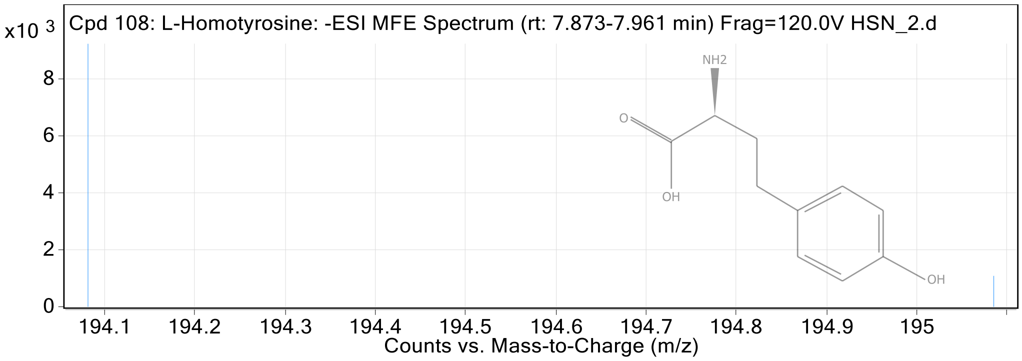

Peak List

m/z	z	Abund	Name	Formula	Ion	Score (DB)	Hits (DB)
194.08184	-1	9215.87	L-Homotyrosine	C10 H13 N O3	(M-H)-	86.5	10
195.08534	-1	1082.3		C10 H13 N O3	(M-H)-		

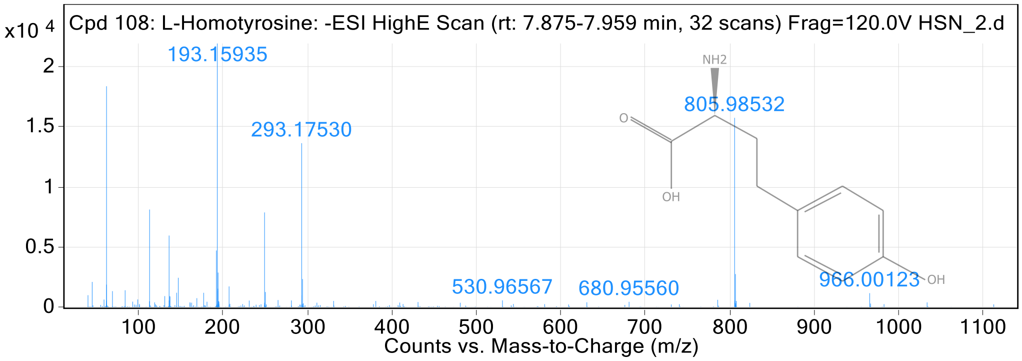

Peak List

m/z	z	Abund
61.98845		18377.83
112.98521		8112.16
136.08898	1	5947.01

192.11496	1	4699.84
193.15935	1	21935.93
194.16252	1	2863.51
249.18554	1	7871.3
293.1753	1	13635.02
805.98532	1	15745.72
806.98894	1	2737.62

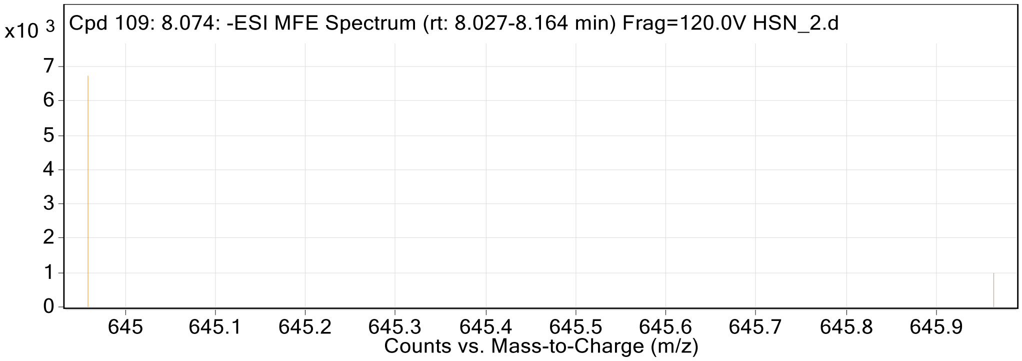

Peak List

m/z	z	Abund	Ion
644.95853	-1	6734.41	(M-H)-
645.96383	-1	988.26	(M-H)-

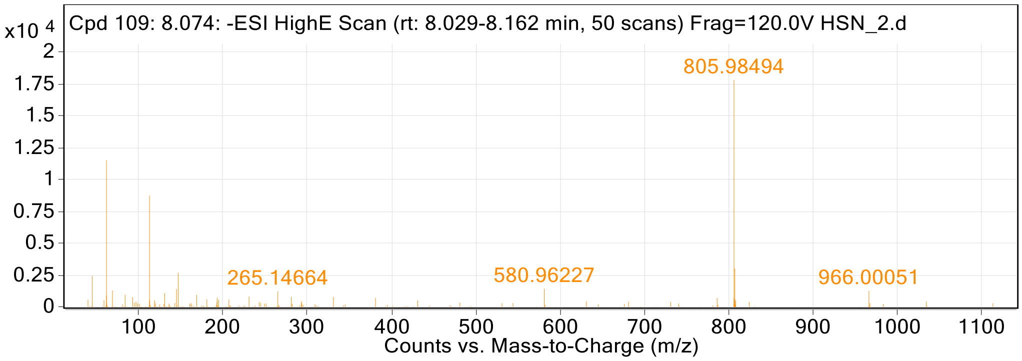

Peak List

m/z	z	Abund
44.99823		2430.52
61.98846		11496.72
68.99597		1278.86
112.98509	1	8722.53
144.9914		1397.72
146.96547		2659
580.96227	1	1413.41
805.98494	1	17790.19
806.98846	1	3007.89
966.00051	1	1248.26

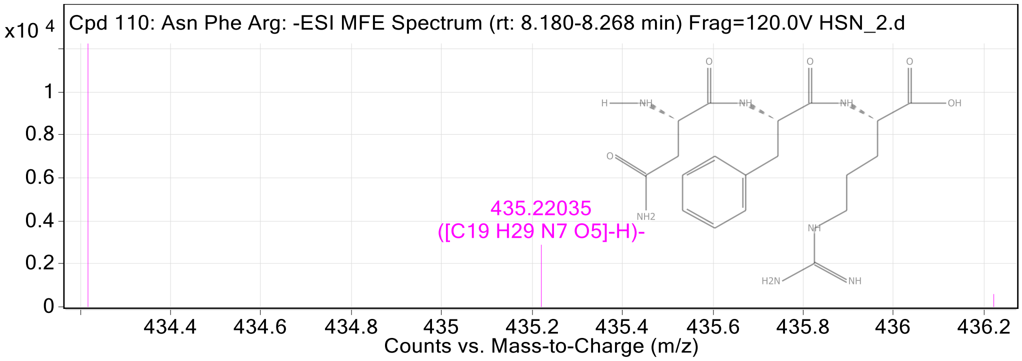

Peak List

m/z	z	Abund	Name	Formula	Ion	Score (DB)	Hits (DB)
434.21722	-1	12240.51	Asn Phe Arg	C19 H29 N7 O5	(M-H)-	93.98	6
435.22035	-1	2886.95		C19 H29 N7 O5	(M-H)-		
436.22163	-1	595.44		C19 H29 N7 O5	(M-H)-		

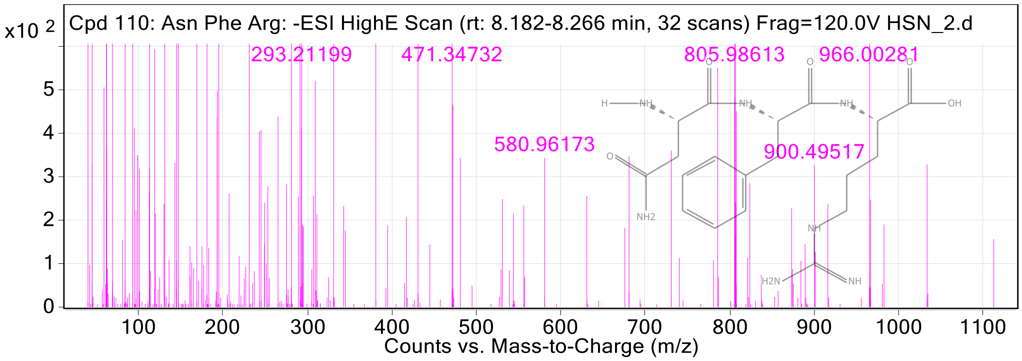

Peak List

m/z	z	Abund
44.99837		5095.17
61.98841		15280.78
62.00446		1406.13
84.00899		1376.57
112.98524		7319.05
144.99148		1746.03
146.96567		1375.45
471.34732	1	1381.52
805.98613	1	13705.56
806.98978	1	2307.15

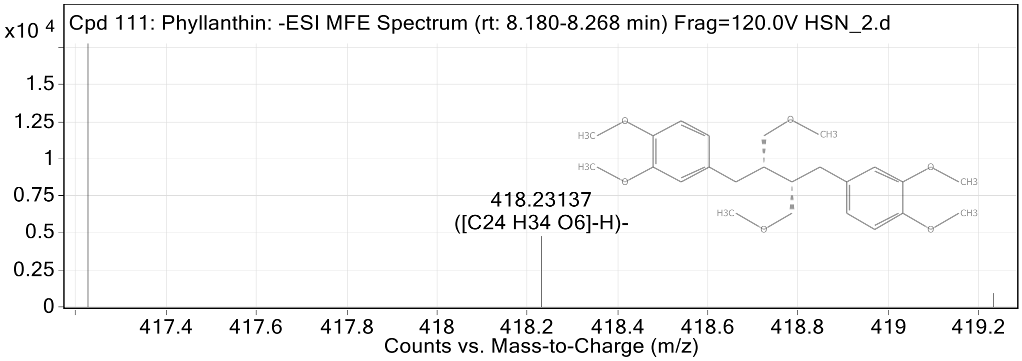

Peak List

m/z	z	Abund	Name	Formula	Ion	Score (DB)	Hits (DB)
417.22727	-1	17790.82	Phyllanthin	C24 H34 O6	(M-H)-	97.4	10
418.23137	-1	4780.71		C24 H34 O6	(M-H)-		

419.23305	-1	934.11		C24 H34 O6	(M-H)-		

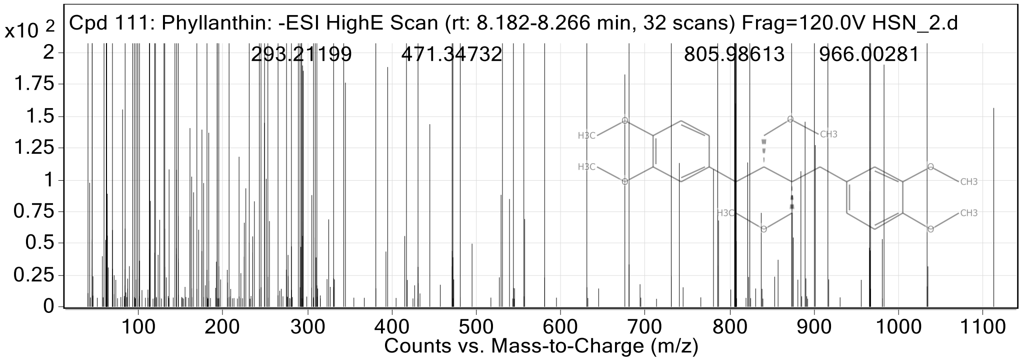

Peak List

m/z	z	Abund
44.99837		5095.17
61.98841		15280.78
62.00446		1406.13
84.00899		1376.57
112.98524		7319.05
144.99148		1746.03
146.96567		1375.45
471.34732	1	1381.52
805.98613	1	13705.56
806.98978	1	2307.15

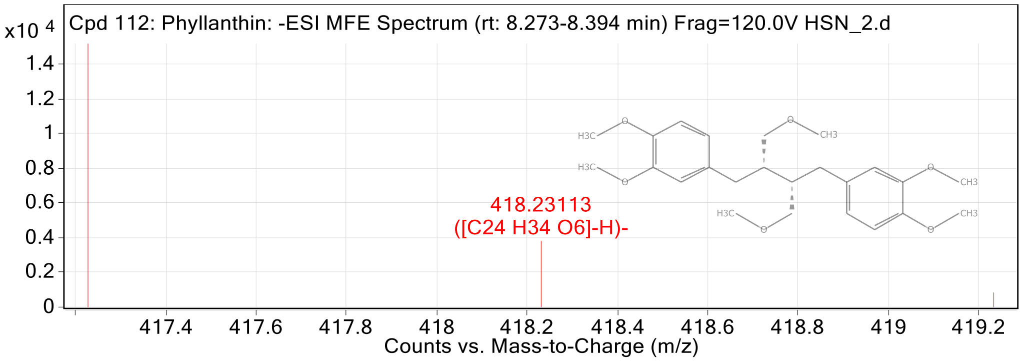

Peak List

m/z	z	Abund	Name	Formula	Ion	Score (DB)	Hits (DB)
417.22727	-1	15209.32	Phyllanthin	C24 H34 O6	(M-H)-	97	10
418.23113	-1	3810.89		C24 H34 O6	(M-H)-		
419.23383	-1	836.85		C24 H34 O6	(M-H)-		

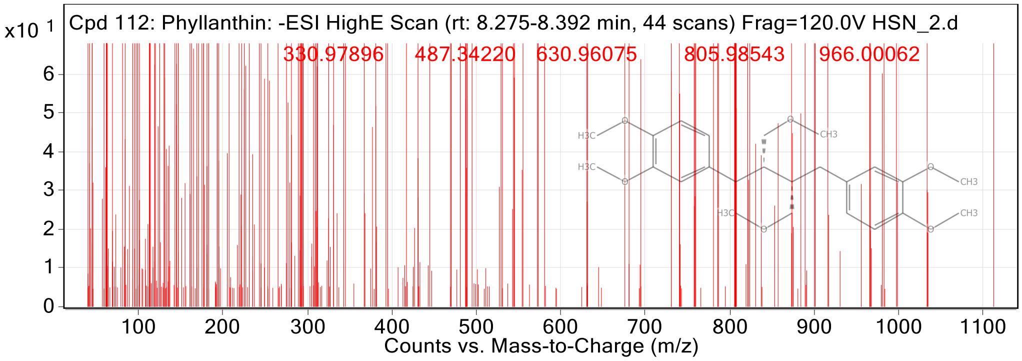

Peak List

m/z	z	Abund
44.99833		4494.57

61.98841		14704.98
68.99592		1225.16
84.00906		1295.46
112.98519	1	8371.12
144.99146		1615.32
146.96551		1464.2
487.3422	1	1328.09
805.98543	1	15995.17
806.98884	1	2702.33

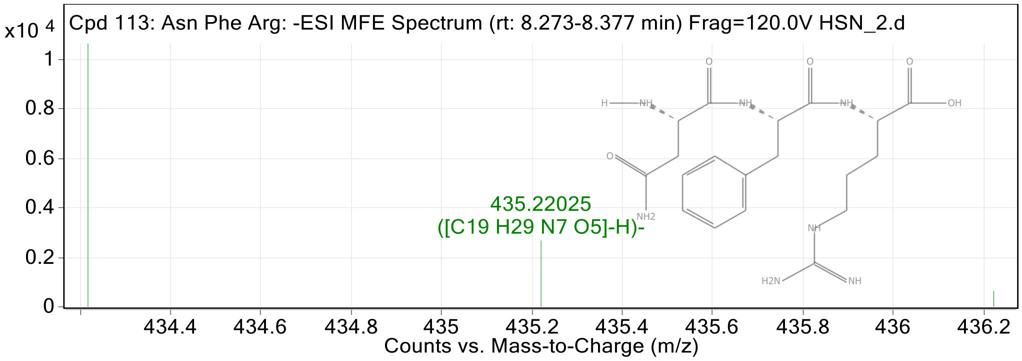

Peak List

m/z	z	Abund	Name	Formula	Ion	Score (DB)	Hits (DB)
434.21743	-1	10634.56	Asn Phe Arg	C19 H29 N7 O5	(M-H)-	91.82	6
435.22025	-1	2680.7		C19 H29 N7 O5	(M-H)-		
436.22226	-1	636.88		C19 H29 N7 O5	(M-H)-		

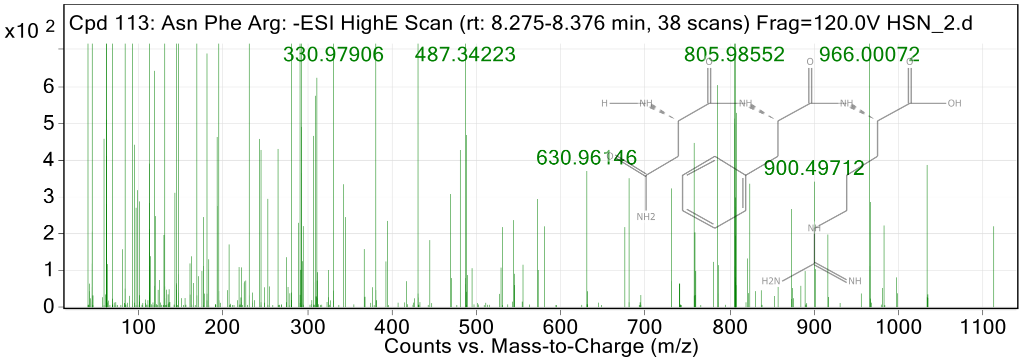

Peak List

m/z	z	Abund
44.99837		4810.54
61.98845		15236.35
68.99594		1227.5
84.00918		1337.46
112.98528	1	8474.35
144.99145		1672.53
146.96564		1310.12
487.34223	1	1459.8
805.98552	1	16024.38
806.98896	1	2687.97

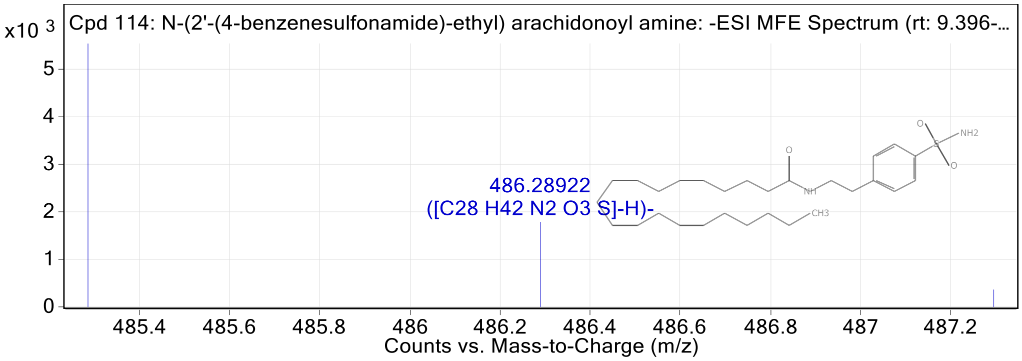

Peak List

m/z	z	Abund	Name	Formula	Ion	Score (DB)	Hits (DB)
485.2854	-1	5541.04	N-(2'-(4-benzenesulfonamide)-ethyl) arachidonoyl amine	C28 H42 N2 O3 S	(M-H)-	83.24	1
486.28922	-1	1787.8		C28 H42 N2 O3 S	(M-H)-		
487.29582	-1	363.87		C28 H42 N2 O3 S	(M-H)-		

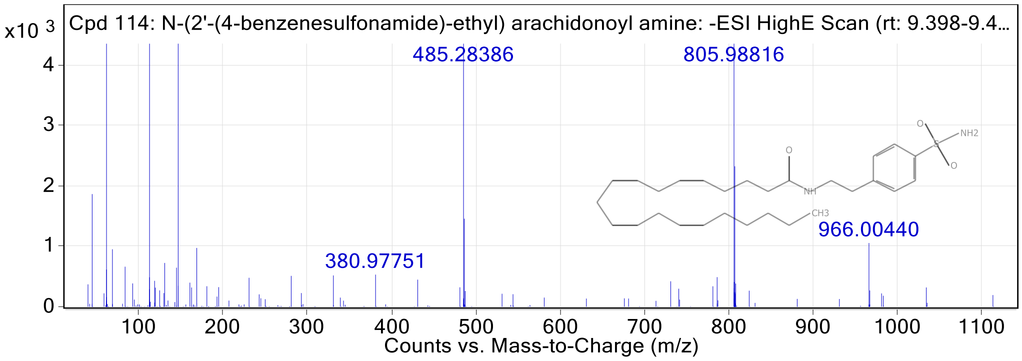

Peak List

m/z	z	Abund
44.99835		1865.58
61.98856		8111.19
112.9854	1	6433.56
146.96584		4546.61
168.98801		973.2
485.28386	1	4357.77
486.28683	1	1457.61
805.98816	1	13930.15
806.99164	1	2326.32
966.0044	1	1054.92

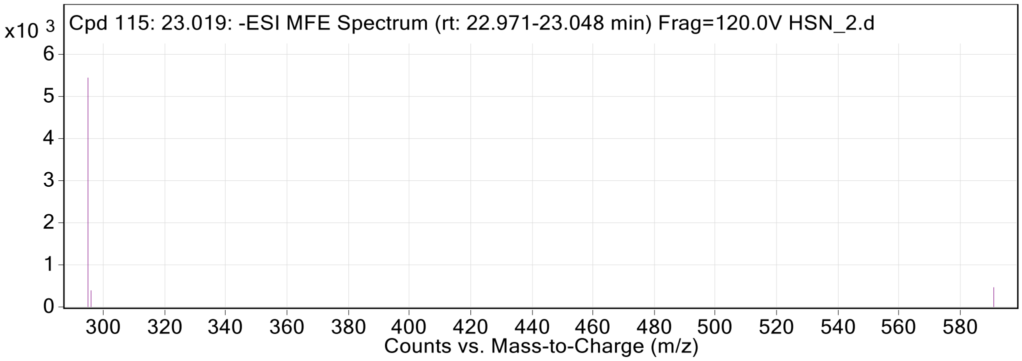

Peak List

m/z	z	Abund	Ion

294.981	-1	5438.03	(M-H)-
295.9838	-1	393.68	(M-H)-
590.97151	-1	466.59	(2M-H)-

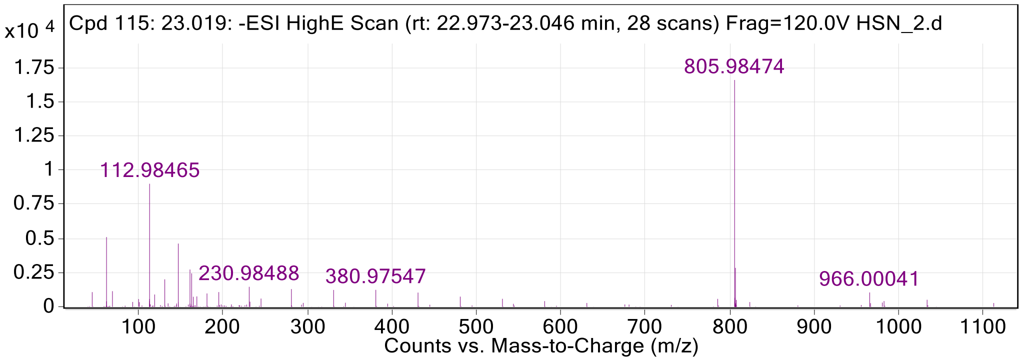

Peak List

m/z	z	Abund
61.98804		5096.78
112.98465	1	8997.26
130.9916		2003.17
146.96494		4619.48
160.84108		2721.99
162.83806		2439.64
230.98488		1455.77
280.98145		1289.62
805.98474	1	16590.46
806.98793	1	2840.23

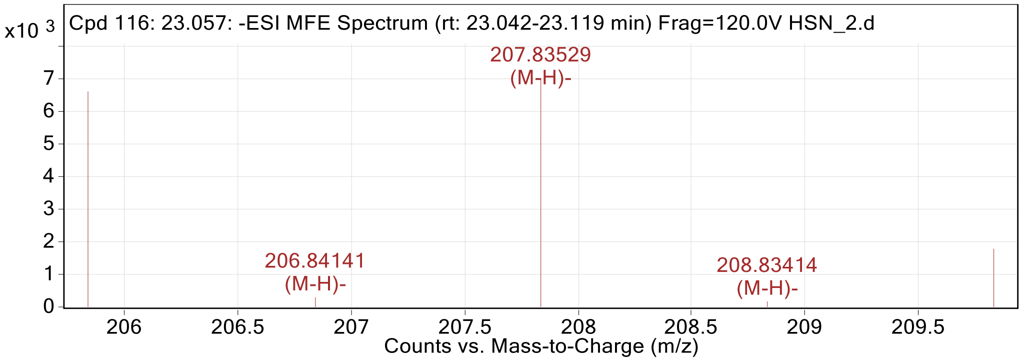

Peak List

m/z	z	Abund	Ion
205.83854	-1	6600.71	(M-H)-
206.84141	-1	299.49	(M-H)-
207.83529	-1	6975.09	(M-H)-
208.83414	-1	166.93	(M-H)-
209.83261	-1	1782.74	(M-H)-

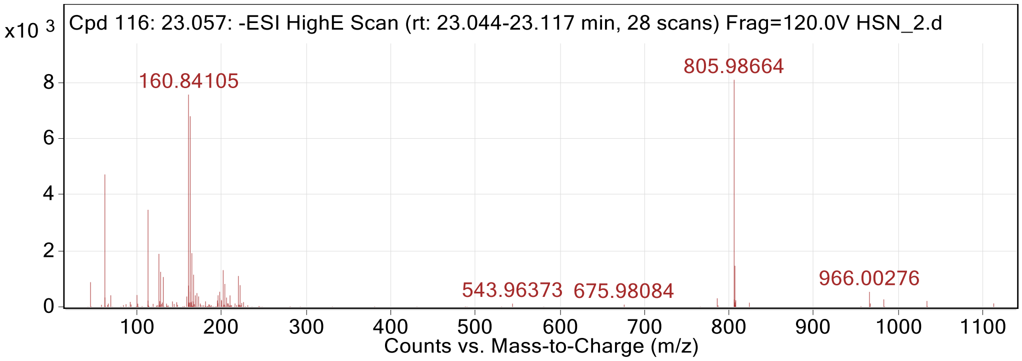

Peak List

m/z	z	Abund
61.98818		4722.27
112.985		3462.54
125.87248	1	1890.46
127.86949	1	1249.75
160.84105	1	7569.99
162.83818	1	6798.67
164.8353	1	1917.2
201.83431	1	1310.16
805.98664	1	8097.28
806.98989	1	1467.06

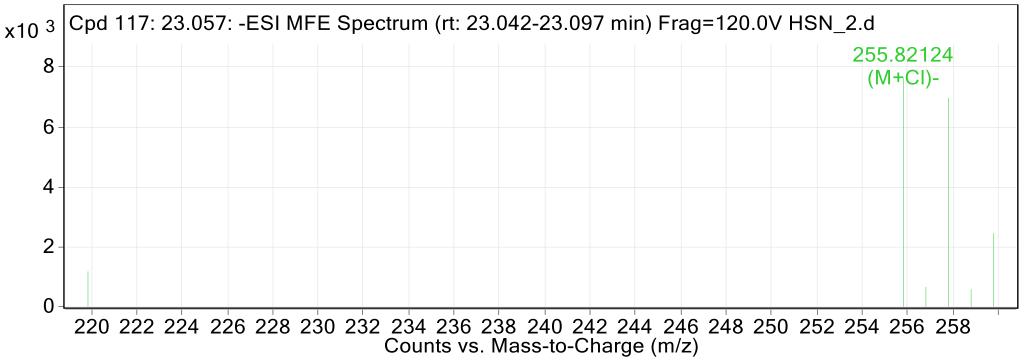

Peak List

m/z	z	Abund	Ion
219.84648	-1	1185.25	(M-H)-
255.82124	-1	7657.32	(M+Cl)-
256.82032	-1	662.42	(M+Cl)-
257.81755	-1	6975.56	(M+Cl)-
258.81504	-1	590.63	(M+Cl)-
259.81454	-1	2456.9	(M+Cl)-

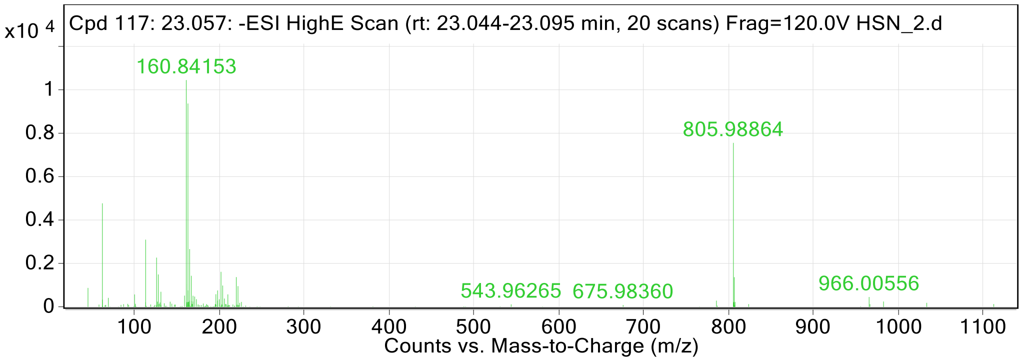

Peak List

m/z	z	Abund
61.9884		4777.4
112.98531		3098.8
125.87291	1	2271.1
127.86989	1	1493.68
160.84153	1	10466.84
162.83865	1	9385.26
164.83577	1	2665.04
166.86563	1	1431.41
201.83487	1	1614.56
805.98864	1	7567.56

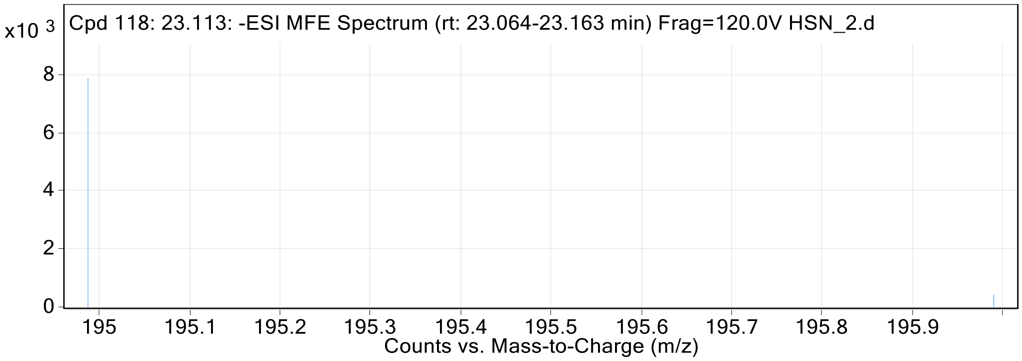

Peak List

m/z	z	Abund	Ion
194.98709	-1	7875.44	(M-H)-
195.99063	-1	437.24	(M-H)-

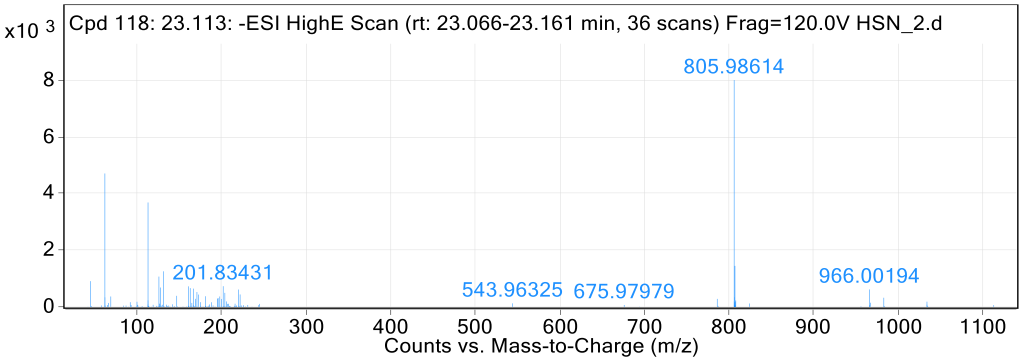

Peak List

m/z	z	Abund
44.99796		906.46
61.988		4707.22
112.9847		3683.81
125.87215	1	1067.27
127.86893	1	681.46
130.99143		1253.27
160.8409		722.62
201.83431		728.19
805.98614	1	8004.67
806.98867	1	1444.98

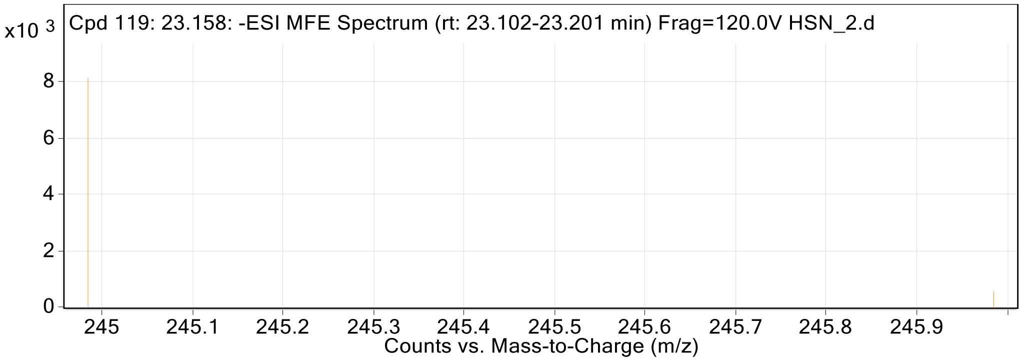

Peak List

m/z	z	Abund	Ion
244.98416	-1	8140.78	(M-H)-
245.98501	-1	554.04	(M-H)-

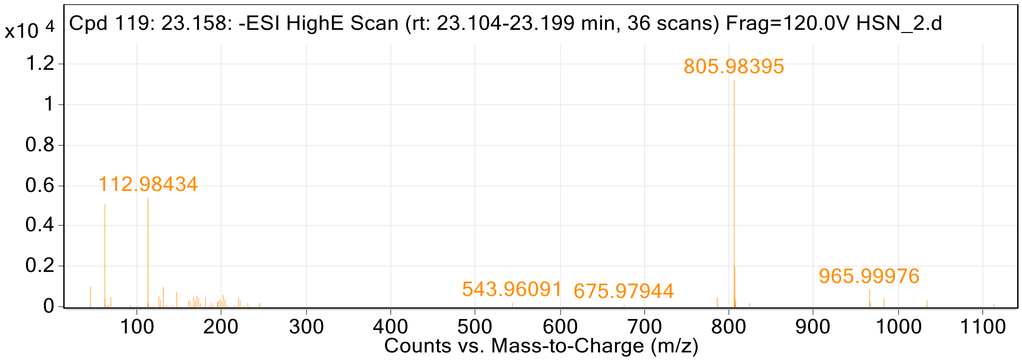

Peak List

m/z	z	Abund
44.99776	1	994.54
61.98784		5072.56
112.98434	1	5389.58
130.99102		985.59
146.96434		737.91
170.83162		536.35
201.83419		574.03
805.98395	1	11213.9
806.98615	1	1968.41
965.99976	1	838.64

--- End Of Report ---

Qualitative Analysis Report

**Data Filename**	HSN_2.d	**Sample Name**	HSN_2
**Sample Type**	Sample	**Position**	P1-A5
**Instrument Name**	6530B LC Q-TOF	**User Name**	Hasan CAN (hcan)
**Acq Method**	Unknown_3-Expt_(pos).m	**Acquired Time**	3/25/2025 12:06:40 PM
**IRM Calibration Status**	Success	**DA Method**	Default.m
**Comment**			

**Sample Group**		**Info.**	
**Stream Name**	LC 1	**Method Version**	2025-0325-0557-37078
**Override DA Method Version**		**Data File Version**	2025-0325-0906-36664
**Acquisition Workstation**	DESKTOP-L73MD3C	**DA Workstation**	DESKTOP-L73MD3C
**Acquisition SW Version**	6200 series TOF/6500 series Q-TOF (11.0.203.0)		

User Chromatograms

Fragmentor Voltage 120 **Collision Energy** 0 **Ionization Mode** ESI

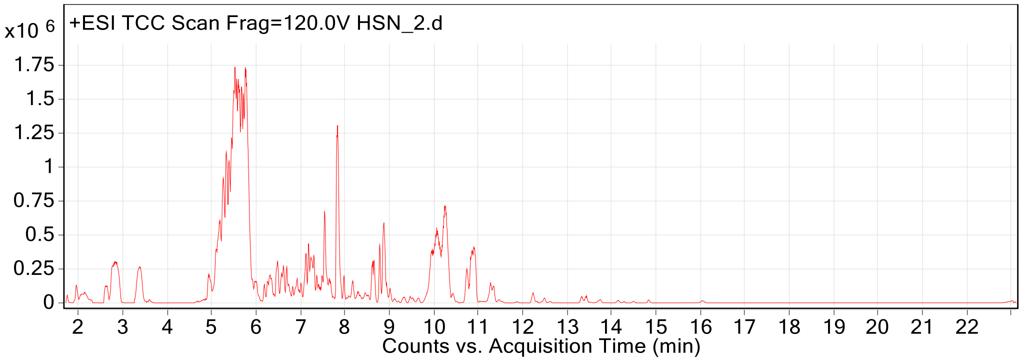

Compounds

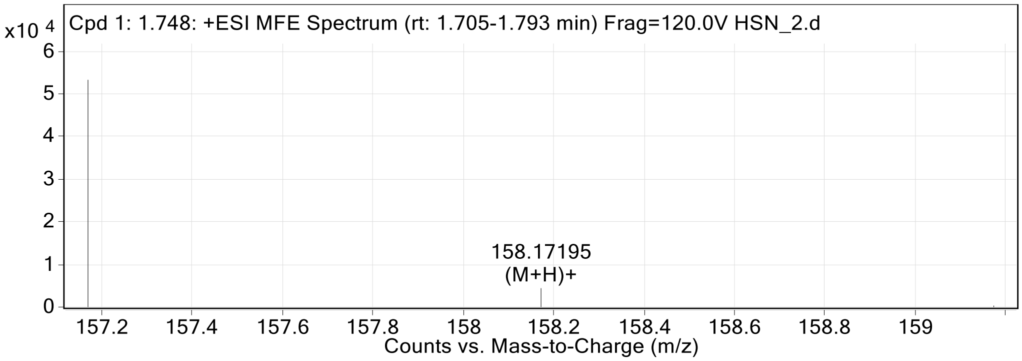

Peak List

m/z	z	Abund	Ion
157.16913	1	53262.69	(M+H)+
158.17195	1	4426.63	(M+H)+
159.17414	1	334.65	(M+H)+

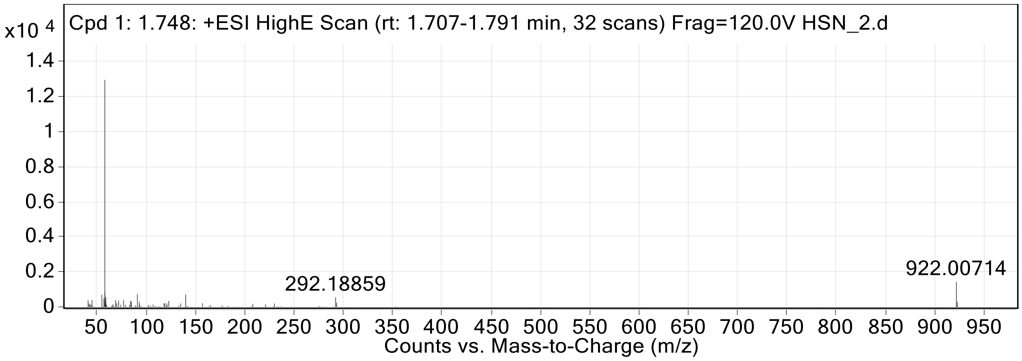

Peak List

m/z	z	Abund
45.03346		397.93
55.05407	1	703.36
57.06963		481.91
58.06488	1	12959.04
58.08116		815.86
59.07155		560.48
91.05356		731.2
140.14146	1	706.6
292.18859	1	540.28
922.00714	1	1429.78

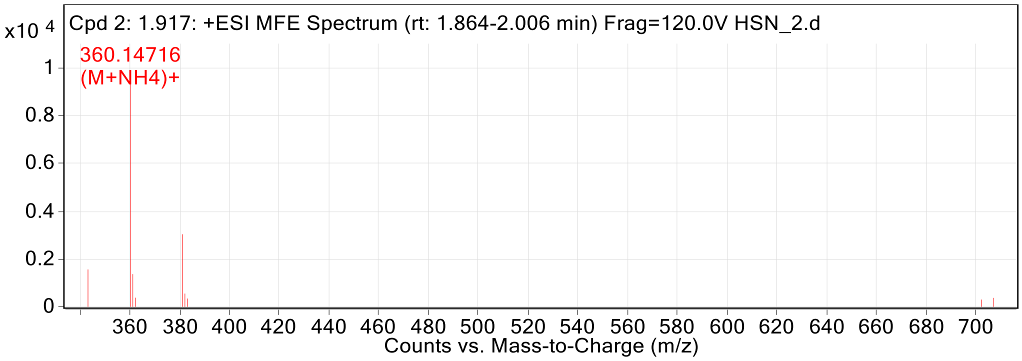

Peak List

m/z	z	Abund	Ion
343.12035	1	1564.76	(M+H)+
360.14716	1	9544.18	(M+NH4)+
361.15043	1	1368.79	(M+NH4)+
362.1536	1	380.96	(M+NH4)+
381.07611	1	3039.55	(M+K)+
382.079	1	552.98	(M+K)+
383.07507	1	344.91	(M+K)+
702.26304	1	302.01	(2M+NH4)+
707.2185	1	372.97	(2M+Na)+

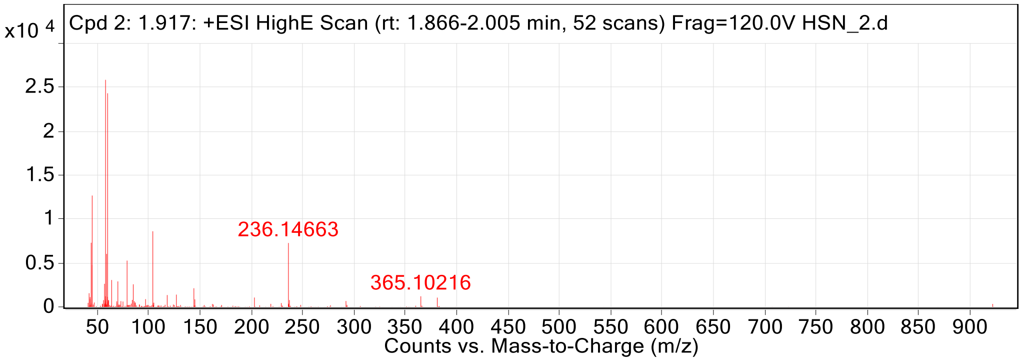

Peak List

m/z	z	Abund
44.04903		7305.89
45.03304	1	12681.7
45.05656		4769.49
58.06444	1	25857.95
59.07199		6033.1
60.0801	1	24327.13
63.99698		3062.73
79.02026		5271.69
104.10535	1	8610.4
236.14663	1	7276.47

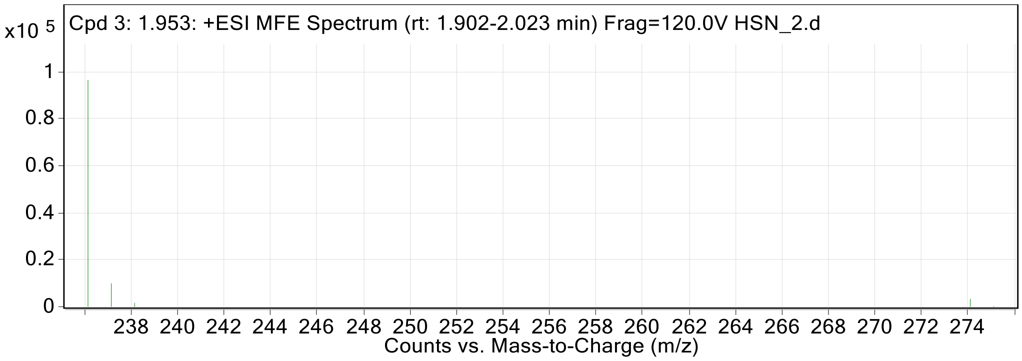

Peak List

m/z	z	Abund	Ion
236.14666	1	96523.33	(M+H)+
237.1497	1	9977.37	(M+H)+
238.15152	1	1608.72	(M+H)+
274.10243	1	3367.25	(M+K)+
275.11369	1	295.03	(M+K)+

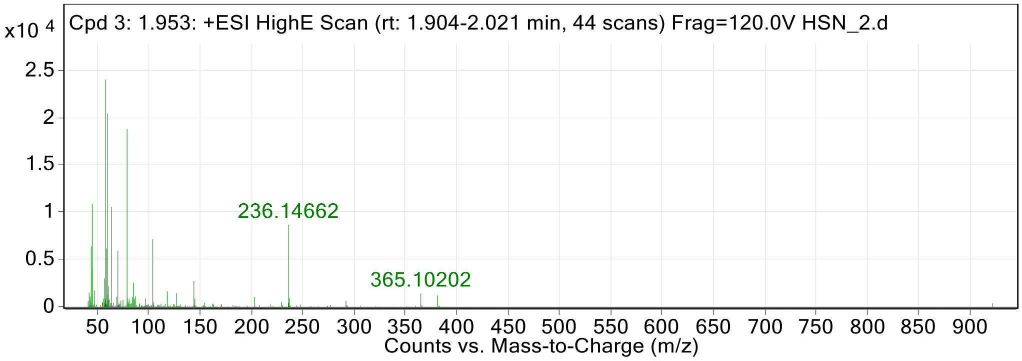

Peak List

m/z	z	Abund

44.04903	1	6368.59
45.03306		10864.07
58.06446		23988.24
59.07194		6159.18
60.08012	1	20422.55
63.99698		10542.29
70.06422	1	5930.33
79.0203	1	18792.54
104.10532	1	7148.35
236.14662	1	8674.99

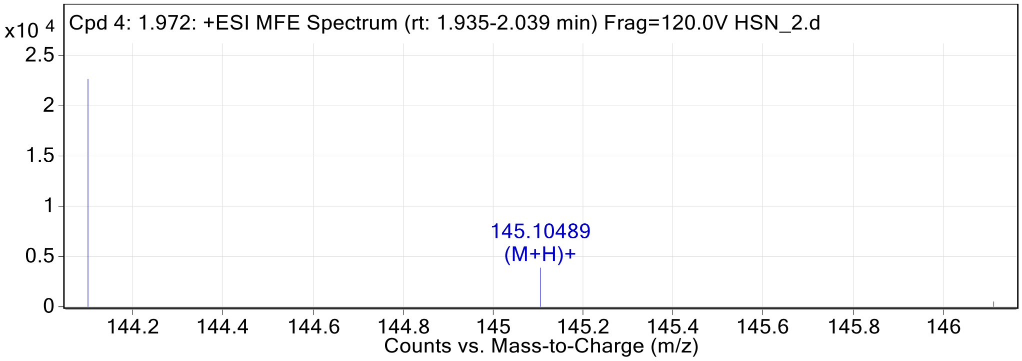

Peak List

m/z	z	Abund	Ion
144.09975	1	22699.68	(M+H)+
145.10489	1	3896.68	(M+H)+
146.11229	1	521.49	(M+H)+

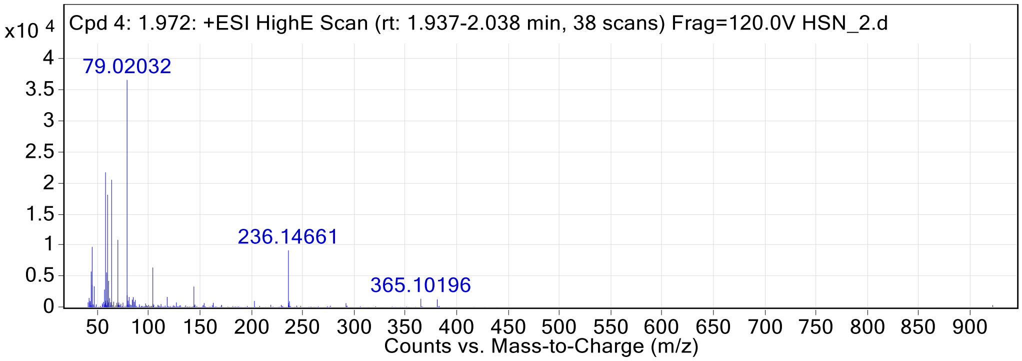

Peak List

m/z	z	Abund
44.04901		5717.55
45.03307		9689.52
58.06446		21719.45
59.07197		5571.39
60.08012	1	18099.37
63.99701	1	20509.64
70.06425	1	10815.78
79.02032	1	36624.3
104.10534	1	6362.73
236.14661	1	9133.81

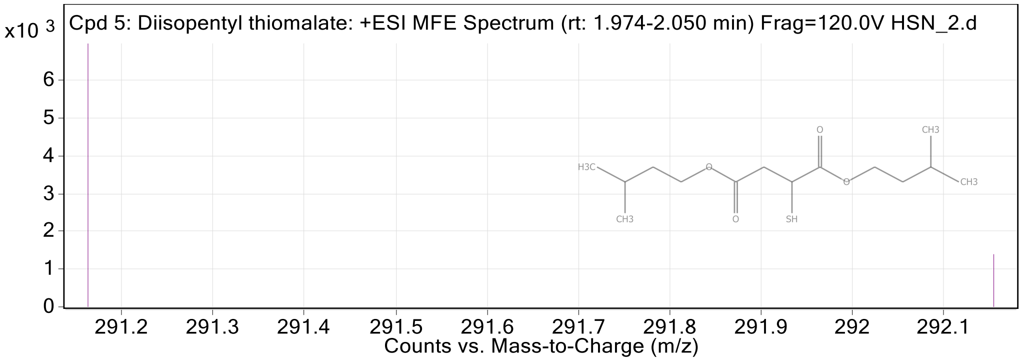

Peak List

m/z	z	Abund	Name	Formula	Ion	Score (DB)	Hits (DB)
291.16324	1	6987.69	Diisopentyl thiomalate	C14 H26 O4 S	(M+H)+	58.88	1
292.15445	1	1397.28		C14 H26 O4 S	(M+H)+		

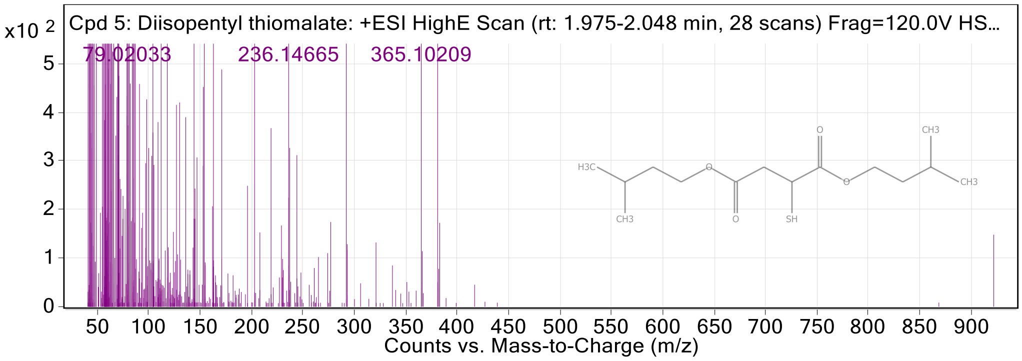

Peak List

m/z	z	Abund
44.04901		4670.66
45.0331		7097.08
46.99456		5771.34
58.06447		14448.11
60.08013	1	13481.13
61.00999		7281.73
63.99702	1	35537.3
70.06427	1	18554.6
79.02033	1	63533.86
104.10534	1	4818.54

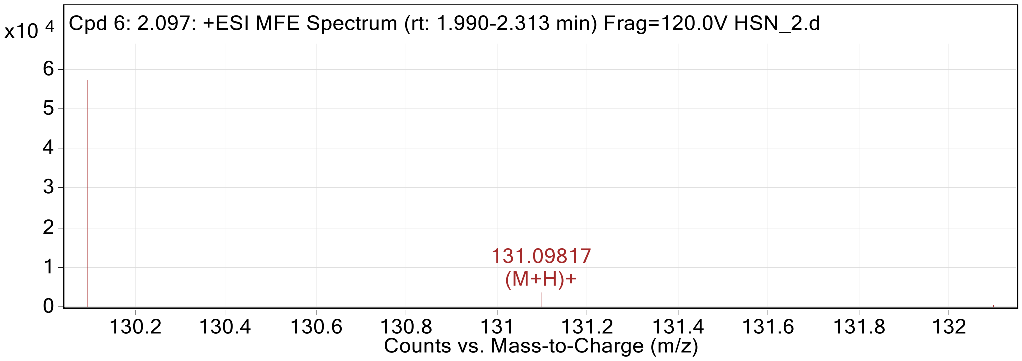

Peak List

m/z	z	Abund	Ion
130.09553	1	57217.13	(M+H)+
131.09817	1	3621.74	(M+H)+
132.09863	1	400.22	(M+H)+

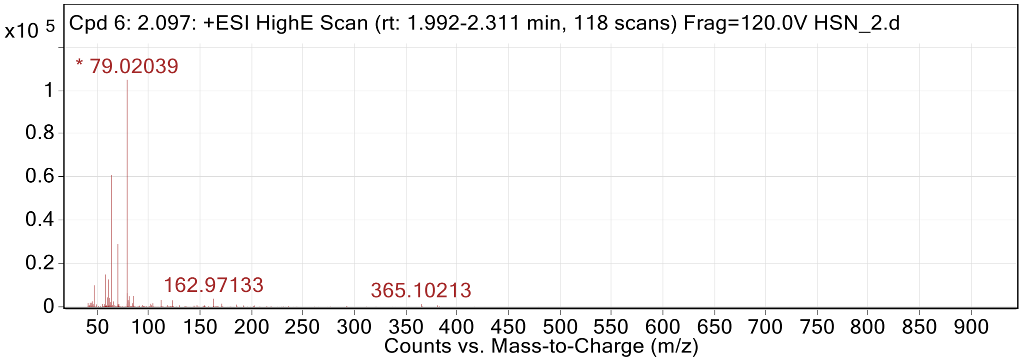

Peak List

m/z	z	Abund
46.99455		9945.68
58.0645	1	14932.3
60.08013	1	4348.18
61.00995		12637.55
63.99704	1	60838.54
70.06428	1	29098.06
79.02039	1	104816.16
79.09859		6329.8
81.01601	1	4952.11
84.95851		5067.62

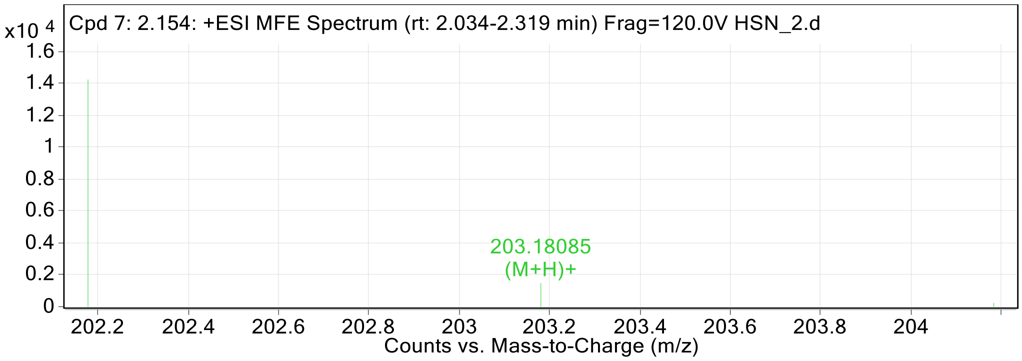

Peak List

m/z	z	Abund	Ion
202.17767	1	14240.38	(M+H)+
203.18085	1	1494.5	(M+H)+
204.18406	1	239.89	(M+H)+

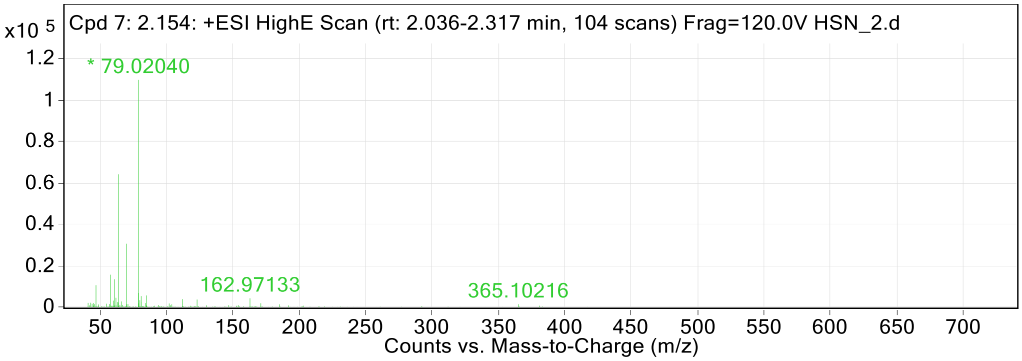

Peak List

m/z	z	Abund
46.99455		10490.29
58.0645	1	15579.05

61.00995		13328.06
62.01755		4417.6
63.99704	1	64072.77

m/z	z	Abund
70.06429	1	30557.94
79.0204	1	109758.66
79.09859		6746.89
81.01602	1	5233.47
84.95851		5520.19

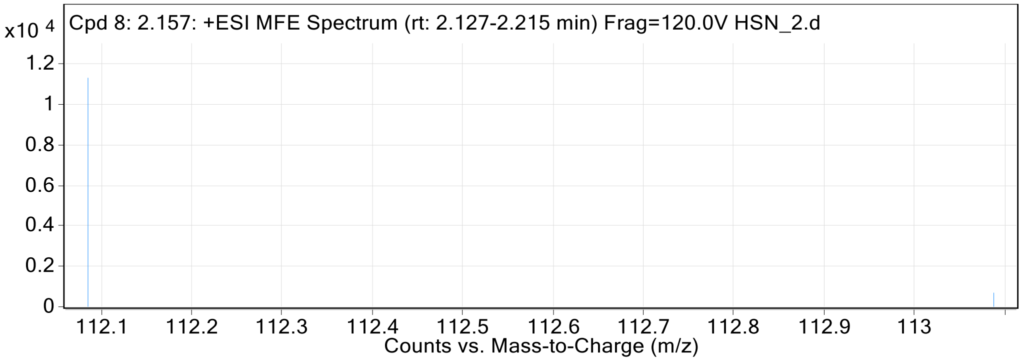

Peak List

m/z	z	Abund	Ion
112.0852	1	11327.19	(M+H)+
113.08803	1	696.35	(M+H)+

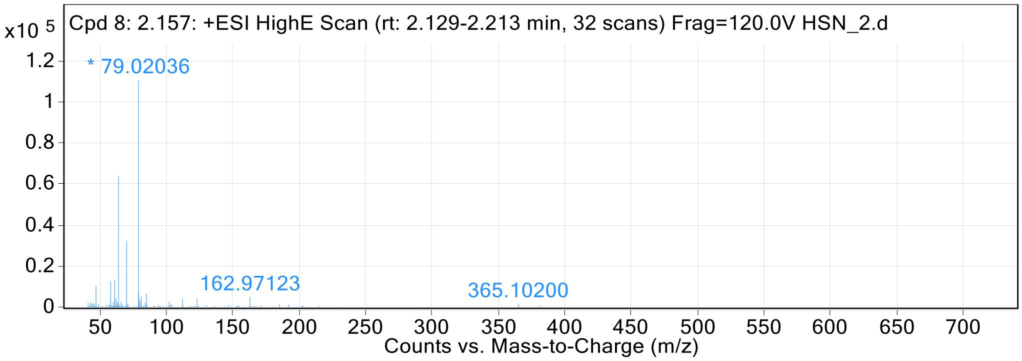

Peak List

m/z	z	Abund
46.99453		10407.05
58.06448	1	12634.94
61.00991		13255.67
63.99703	1	63921.43
70.06428	1	32371.94
79.02036	1	110726.27
79.09857		6777.2
81.01597	1	5210.06
84.95852		6493.04
162.97123		4859.8

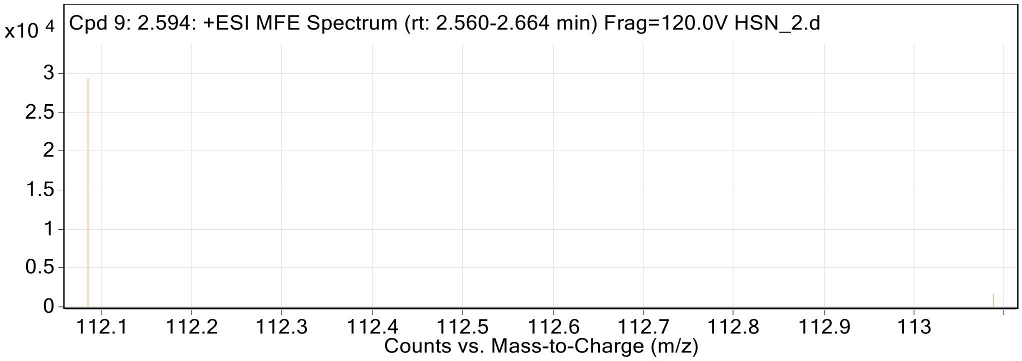

Peak List

m/z	z	Abund	Ion
112.08519	1	29393.33	(M+H)+
113.08821	1	1639.07	(M+H)+

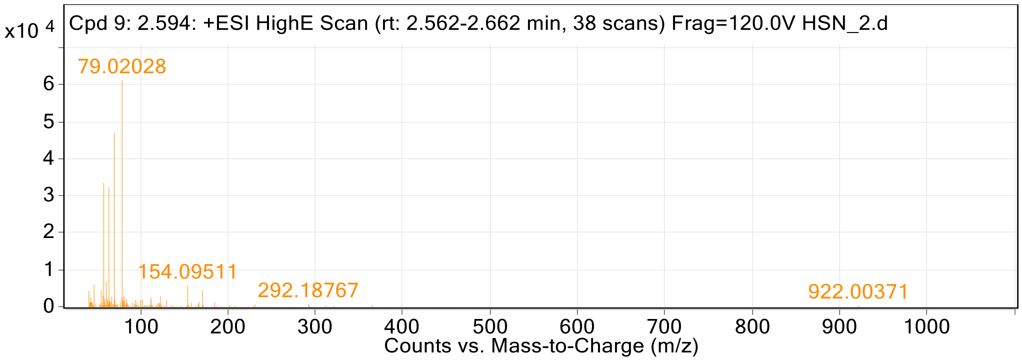

Peak List

m/z	z	Abund
41.0383		4322.33
46.99457		5939.52
55.05362		4403.84
58.06454	1	33529.75
61.01002		6846.93
63.99701	1	32245.24
70.06433	1	46959.21
79.02028	1	61250.45
154.09511	1	5782.31
171.14687	1	4509.41

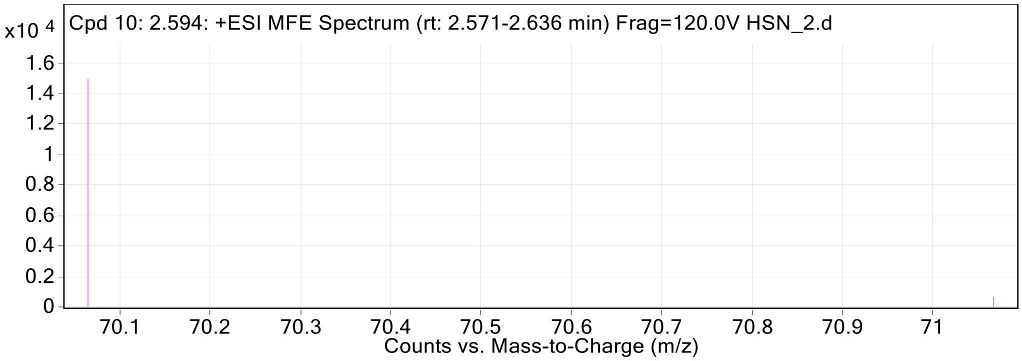

Peak List

m/z	z	Abund	Ion
70.06447	1	15002.06	(M+H)+
71.06774	1	662.24	(M+H)+

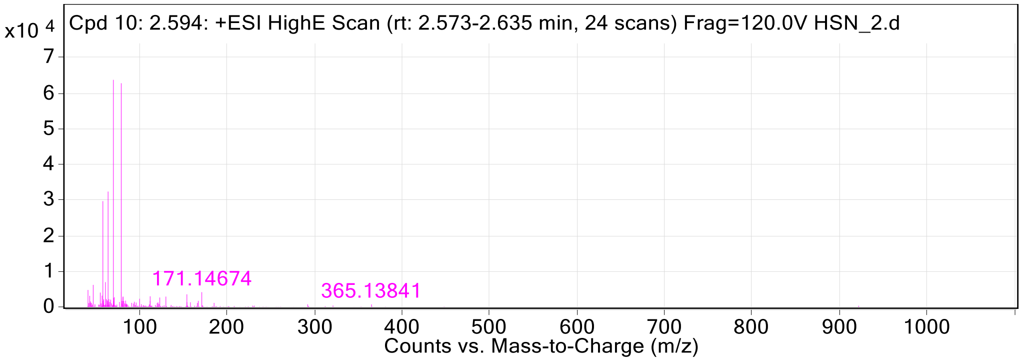

Peak List

m/z	z	Abund
41.03829		4750.03
46.99458		6171.3
55.05362		4008.29
58.06456	1	29667.29
61.01004		6945.47
63.99701	1	32363.47
70.06434	1	63700.58
79.02027	1	62749.73
79.04936		4283.6
171.14674	1	4129.29

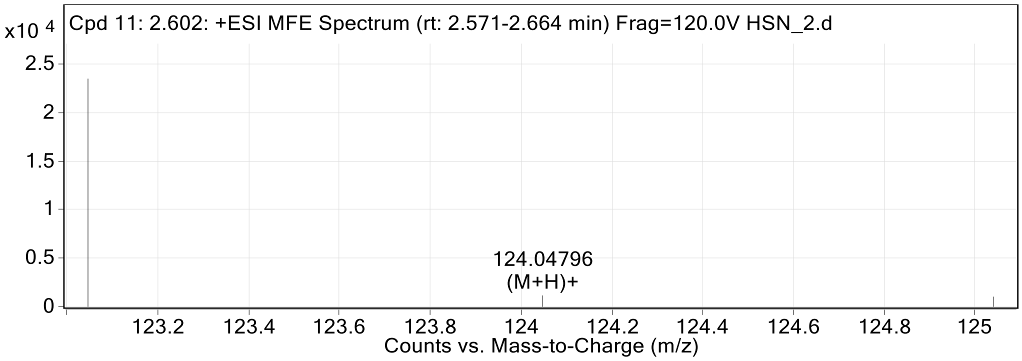

Peak List

m/z	z	Abund	Ion
123.04553	1	23479.54	(M+H)+
124.04796	1	1167.33	(M+H)+
125.04205	1	1041.85	(M+H)+

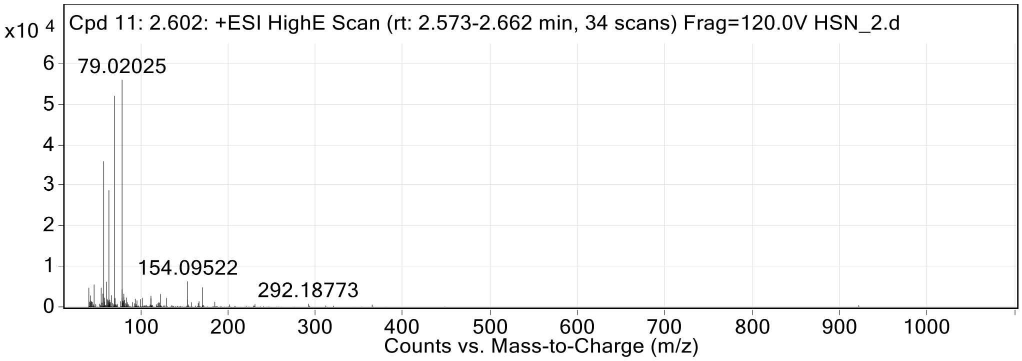

Peak List

m/z	z	Abund
41.0383		4702.66
46.99459		5498.57

55.05363		4707.63
58.06454	1	35918.46
61.01002		6165.68
63.997	1	28781.12
70.06433	1	52047.05
79.02025	1	56003.18
154.09522	1	6282.46
171.14687	1	4840.56

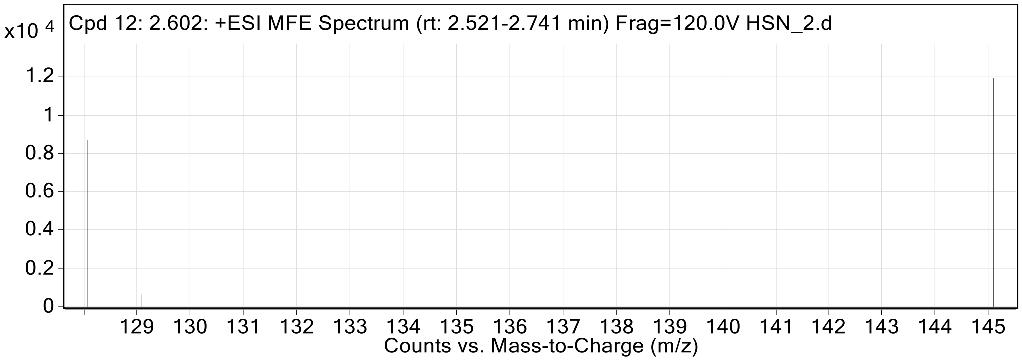

Peak List

m/z	z	Abund	Ion
128.06883	1	8688.01	(M+H)+
129.07213	1	641.76	(M+H)+
145.09502	1	11923.54	(M+NH4)+

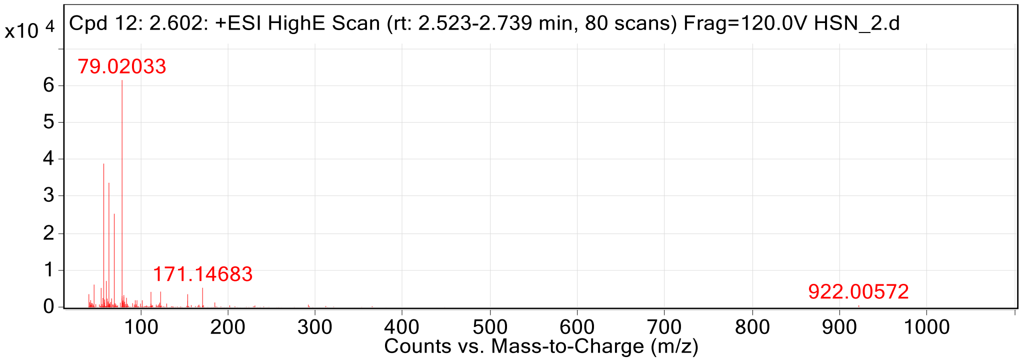

Peak List

m/z	z	Abund
46.9946		6084.5
55.05371		5171.72
58.06455	1	38822.89
61.01003		7066.89
63.99706	1	33646.96
70.06435	1	25262.01
79.02033	1	61436.13
112.07437		4113.66
123.11484	1	4185.73
171.14683	1	5224.63

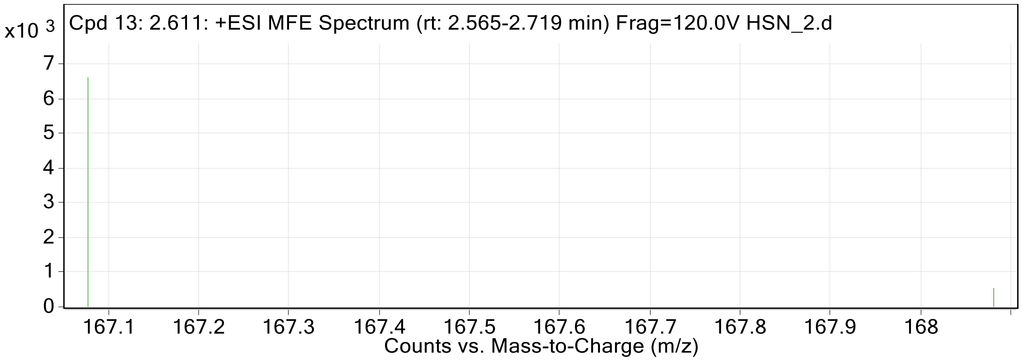

Peak List

m/z	z	Abund	Ion
167.07733	1	6613.74	(M+H)+
168.08096	1	545.7	(M+H)+

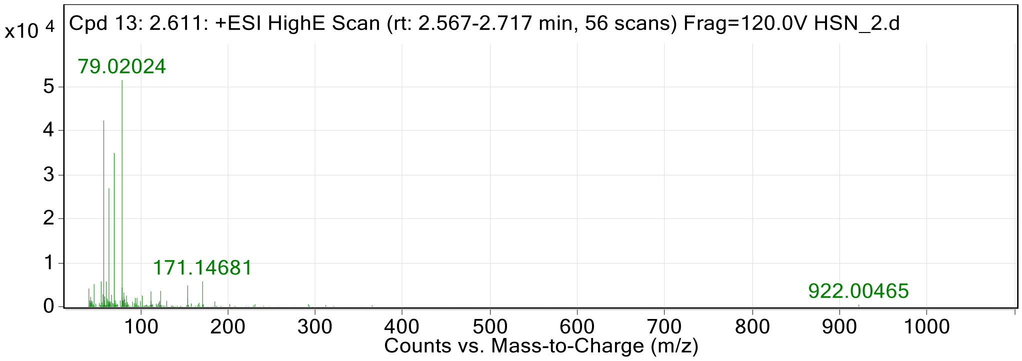

Peak List

m/z	z	Abund
46.99457		5151.71
55.05368		5752.83
58.06455	1	42332.07
61.00999		5728.25
63.99701	1	26942.92
70.06433	1	34935.13
79.02024	1	51540.9
79.04986		4367.54
154.09503	1	4835.65
171.14681	1	5778.63

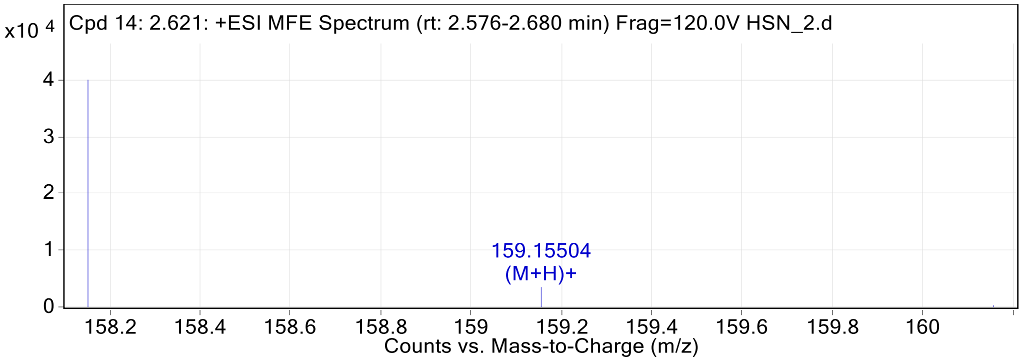

Peak List

m/z	z	Abund	Ion
158.15171	1	40033.59	(M+H)+
159.15504	1	3502.4	(M+H)+
160.15717	1	289.02	(M+H)+

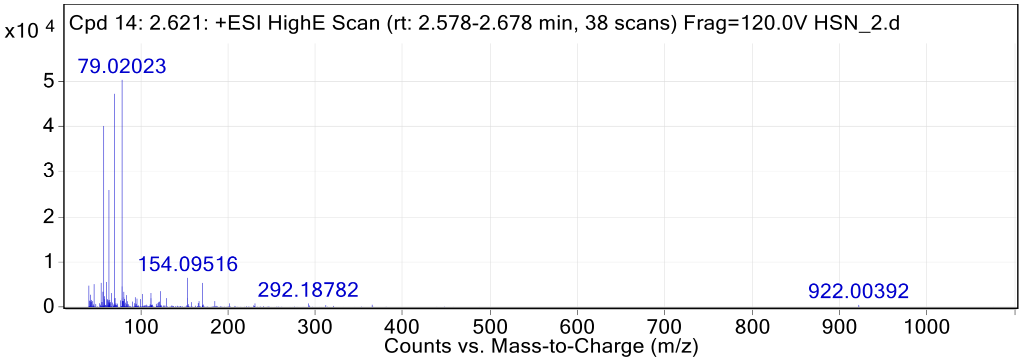

Peak List

m/z	z	Abund
41.03829		4706.57
46.99462		5043.44
55.05365		5333.04
58.06454	1	40076.95
61.01004		5539
63.99701	1	25948.57
70.06434	1	47238.38
79.02023	1	50315.69
154.09516	1	6449.46
171.14685	1	5325.45

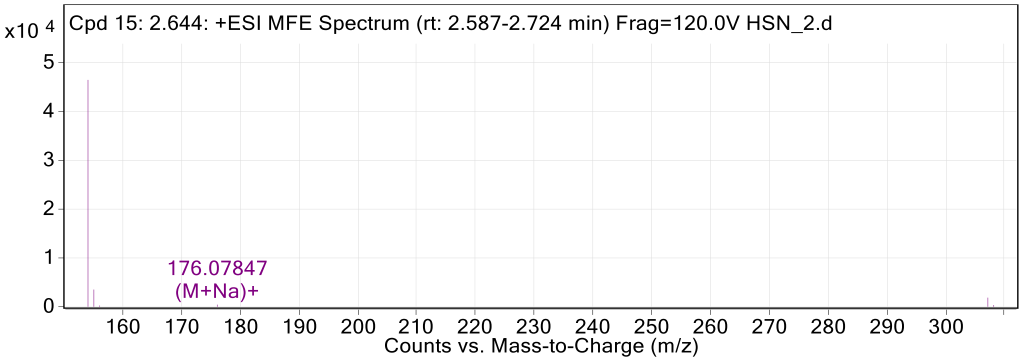

Peak List

m/z	z	Abund	Ion
154.09555	1	46365.39	(M+H)+
155.09809	1	3520.93	(M+H)+
156.09847	1	256.75	(M+H)+
176.07847	1	438.84	(M+Na)+
307.18486	1	1867.01	(2M+H)+
308.18825	1	351.76	(2M+H)+

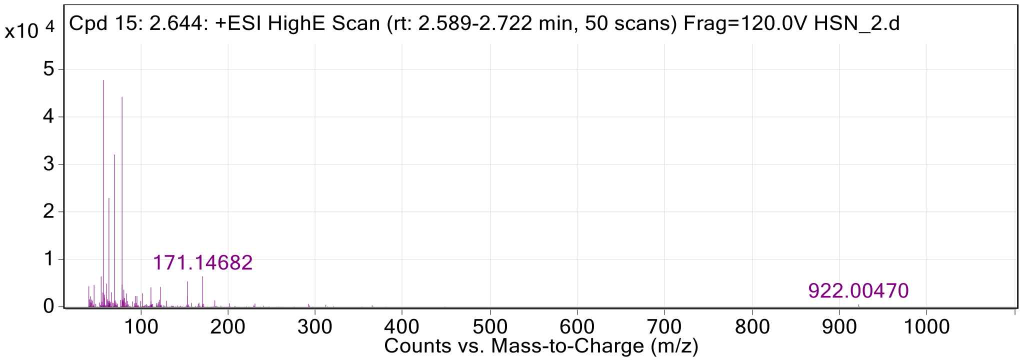

Peak List

m/z	z	Abund
46.99459		4588.6
55.05369		6402.67
58.06454	1	47820.34
61.00999		4895.66
63.99702	1	22956.44
70.06433	1	32127.3
79.02021	1	44274.77
79.04989		4678.96
154.09507	1	5369.36
171.14682	1	6437.96

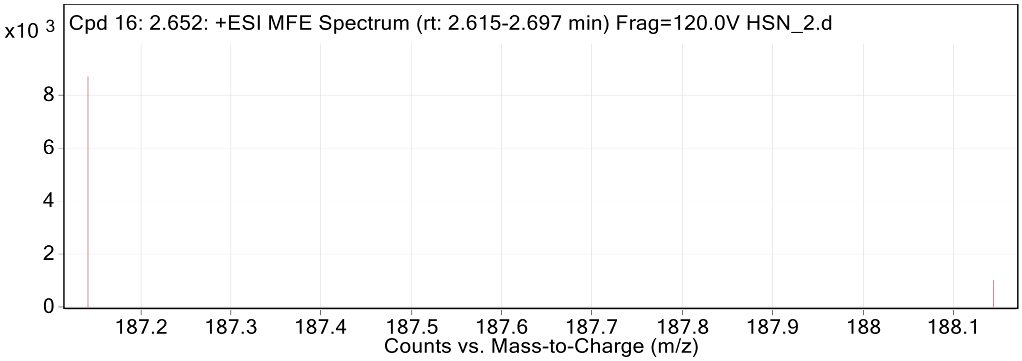

Peak List

m/z	z	Abund	Ion
187.14176	1	8688.4	(M+H)+
188.14501	1	1002.08	(M+H)+

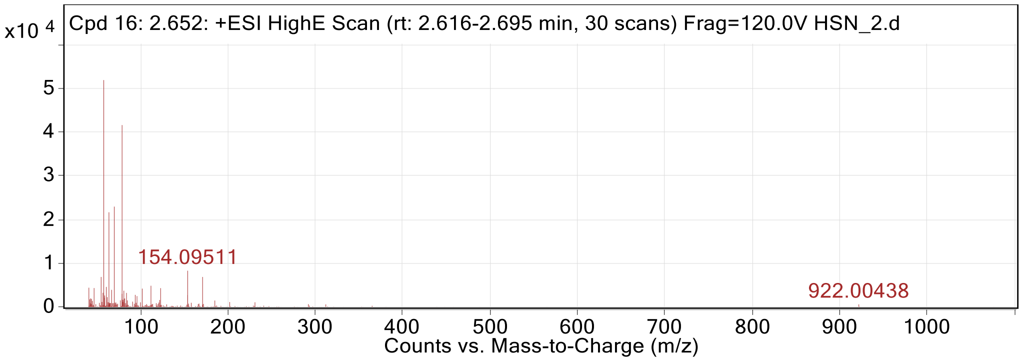

Peak List

m/z	z	Abund
41.03827		4372.57
55.05368		6831.69
58.06455	1	51904.43
61.00995		4558.52
63.99704	1	21645.85
70.06432	1	22937.62
79.02021	1	41604.34
112.07473		4824.16
154.09511	1	8263.89
171.14687	1	6834.08

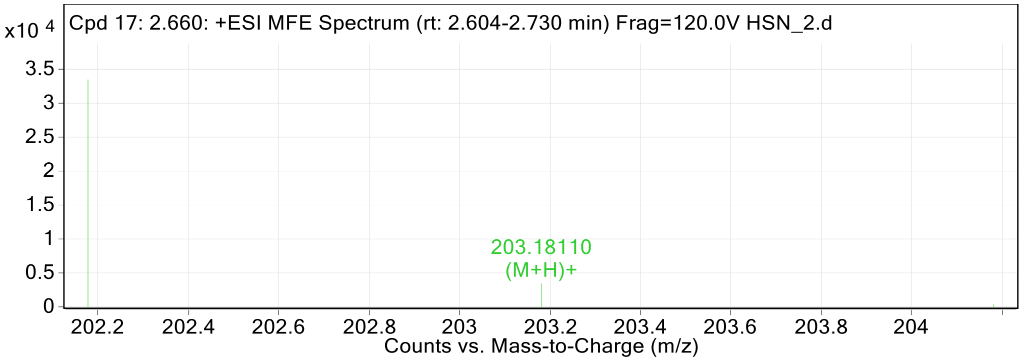

Peak List

m/z	z	Abund	Ion
202.17794	1	33408.41	(M+H)+
203.1811	1	3434.3	(M+H)+
204.18171	1	386.99	(M+H)+

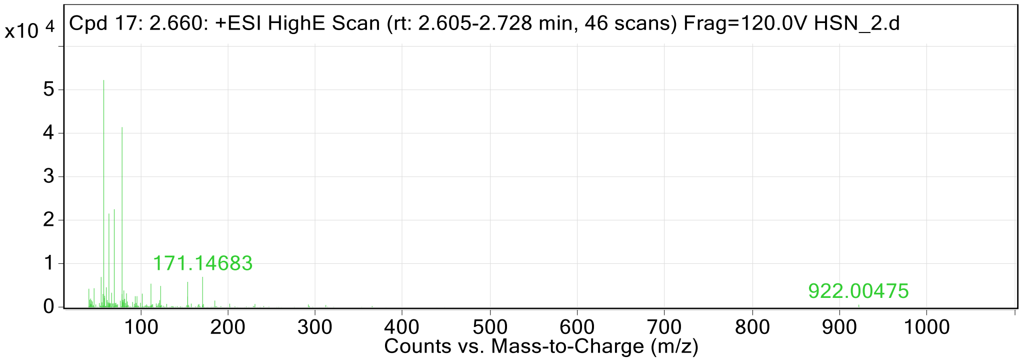

Peak List

m/z	z	Abund
55.0537		6911.73
58.06453	1	52172.53
61.00998		4552.34
63.99703	1	21490.76
70.06431	1	22490.06
79.0202	1	41353.61
112.07504		5374.52
123.11477	1	4856.25
154.09504	1	5792.37
171.14683	1	6947.5

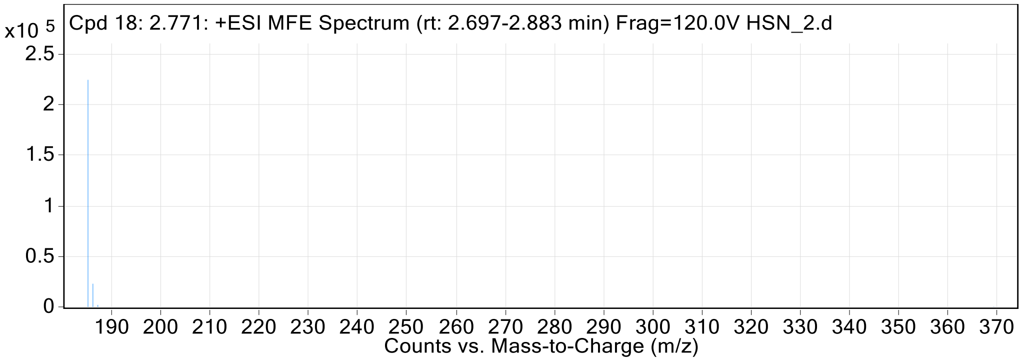

Peak List

m/z	z	Abund	Ion
185.16259	1	224468.17	(M+H)+
186.16573	1	22931.18	(M+H)+

187.16827	1	1985.39	(M+H)+
369.3203	1	245.08	(2M+H)+

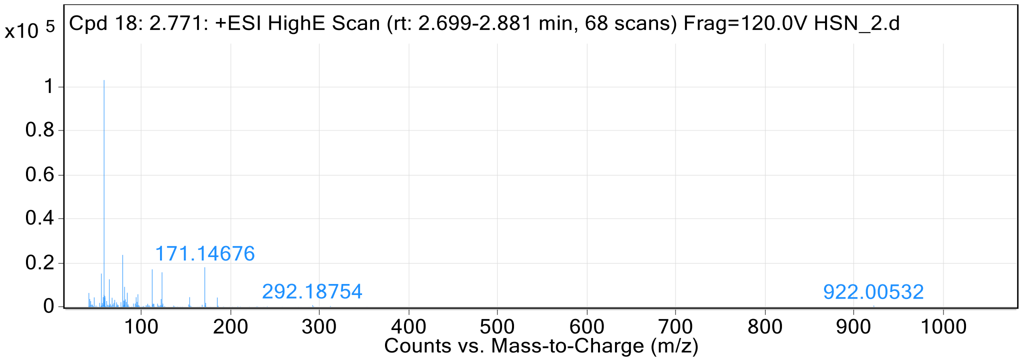

Peak List

m/z	z	Abund
41.03822		6286.35
55.05367		15091.99
58.06464	1	103144.31
63.997		12512.67
79.02014	1	23587.15
81.0686		9061.89
84.07955		6378.91
112.07394	1	17031.98
123.11483	1	15690.34
171.14676	1	17982.58

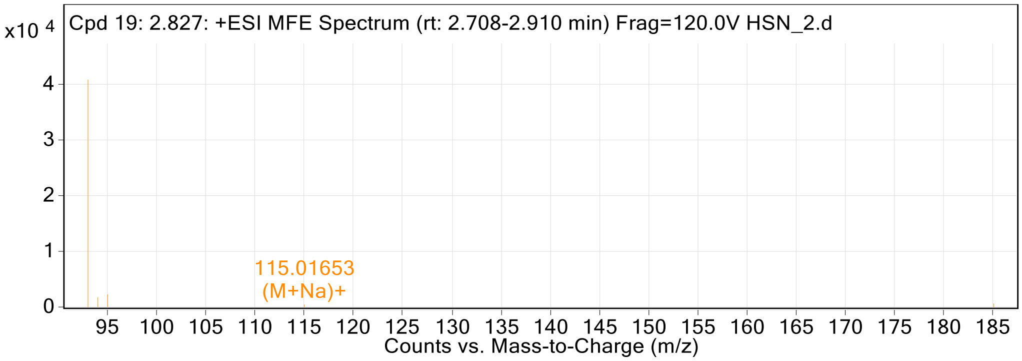

Peak List

m/z	z	Abund	Ion
93.03558	1	40721.86	(M+H)+
94.0383	1	1738.67	(M+H)+
95.03163	1	2273.39	(M+H)+
115.01653	1	441.33	(M+Na)+
185.06384	1	636.98	(2M+H)+

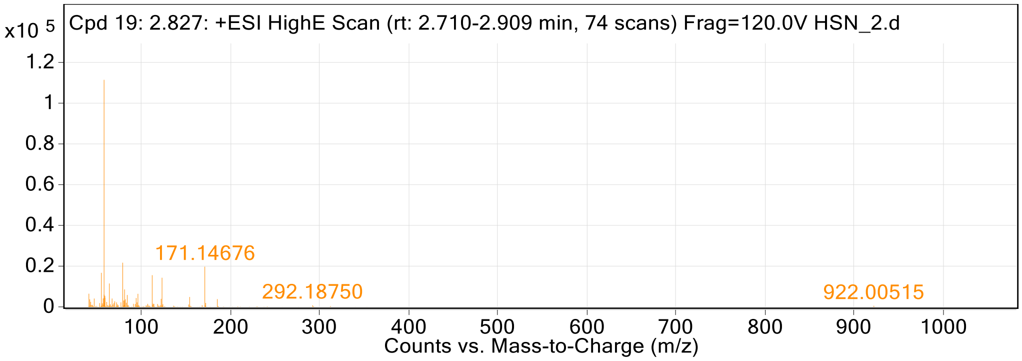

Peak List

m/z	z	Abund
41.0382		6460.91
55.05367		16734.43
58.06465	1	111494.26
63.997		11495.73
79.02013	1	21703.77
81.06856		8568.12
96.07941		6419.4
112.07394	1	15570.73
123.11483	1	14319.94
171.14676	1	19732.19

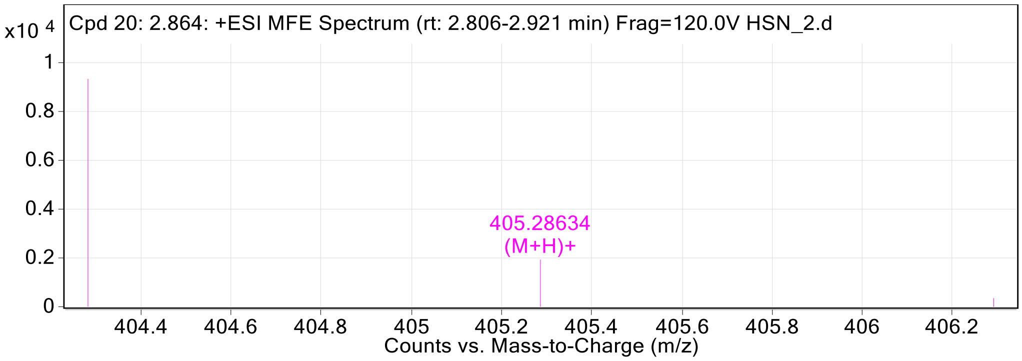

Peak List

m/z	z	Abund	Ion
404.28317	1	9351.65	(M+H)+
405.28634	1	1936.37	(M+H)+
406.29159	1	351.44	(M+H)+

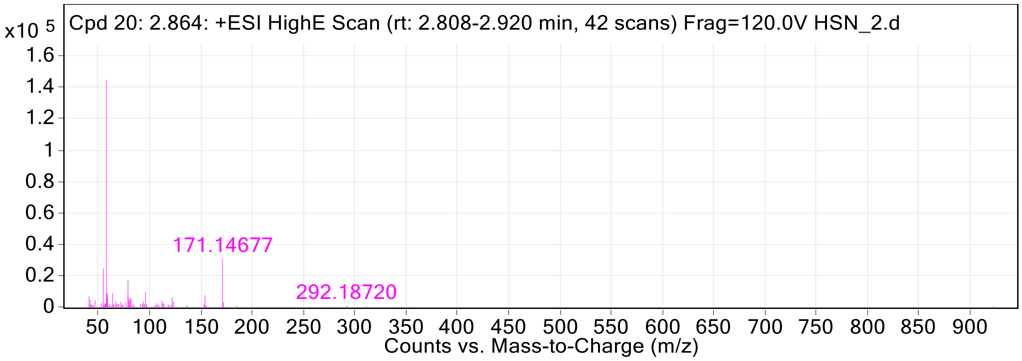

Peak List

m/z	z	Abund
41.03817		6795.01
55.05367		24464.05

58.06468	1	144813.78
58.1318		8519.86
59.0485		8345.29
63.99699		8966.74
79.02011	1	16885.93
96.07937		9735.75
154.12036	1	7385.6
171.14677	1	30761.35

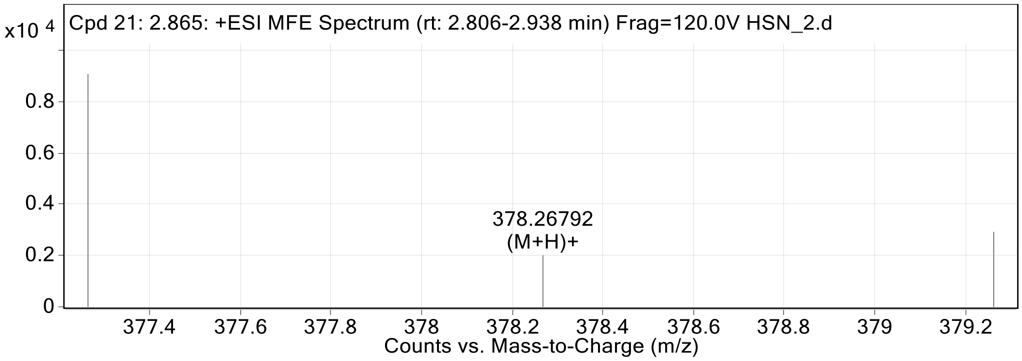

Peak List

m/z	z	Abund	Ion
377.2644	1	9069.48	(M+H)+
378.26792	1	2008.24	(M+H)+
379.26226	1	2923.17	(M+H)+

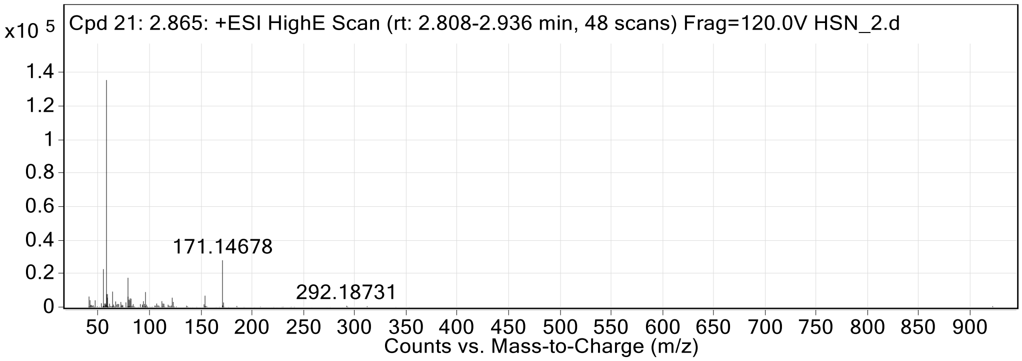

Peak List

m/z	z	Abund
41.03816		6290.77
55.05366		22675.83
58.06467	1	135662.78
58.1318		7736.71
59.0485		7750.05
63.997		9249.92
79.0201	1	17507.65
96.07937		8975.88
154.12036	1	6807.56
171.14678	1	27991.48

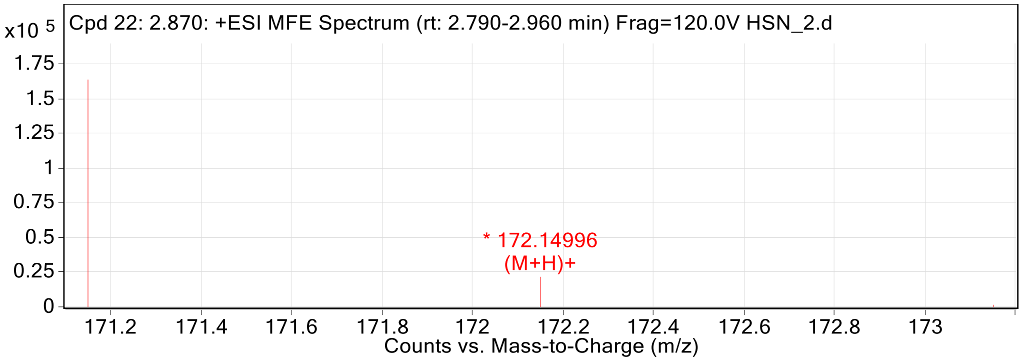

Peak List

m/z	z	Abund	Ion
171.15003	1	163680.66	(M+H)+
172.14996	1	21672.36	(M+H)+
173.15253	1	1511.53	(M+H)+

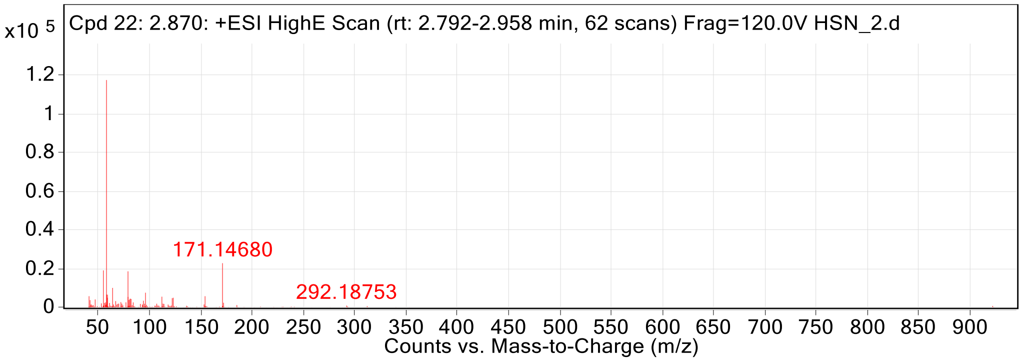

Peak List

m/z	z	Abund
41.03816		5649.52
55.05366		19006.55
58.06467	1	117637.64
58.13181		6478.88
59.04852		6423.33
63.997		9946.36
79.02011	1	18521.44
96.0794		7450.45
154.12036	1	5638.55
171.1468	1	22752.02

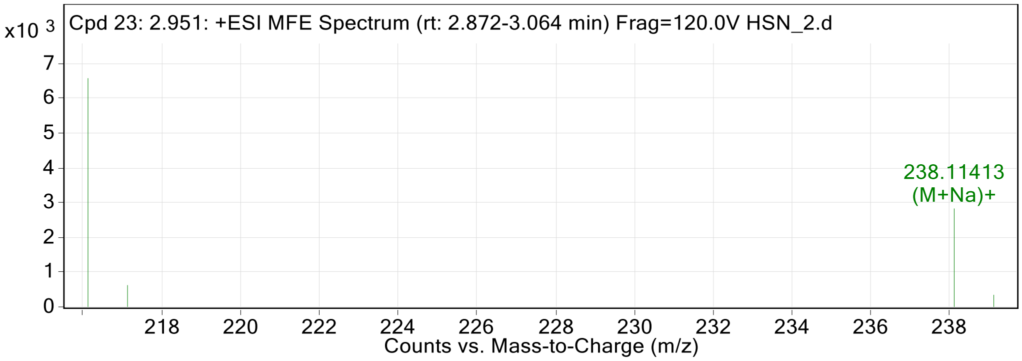

Peak List

m/z	z	Abund	Ion
216.13237	1	6581.31	(M+H)+
217.13558	1	626.44	(M+H)+

238.11413	1	2831.51	(M+Na)+
239.11822	1	347.32	(M+Na)+

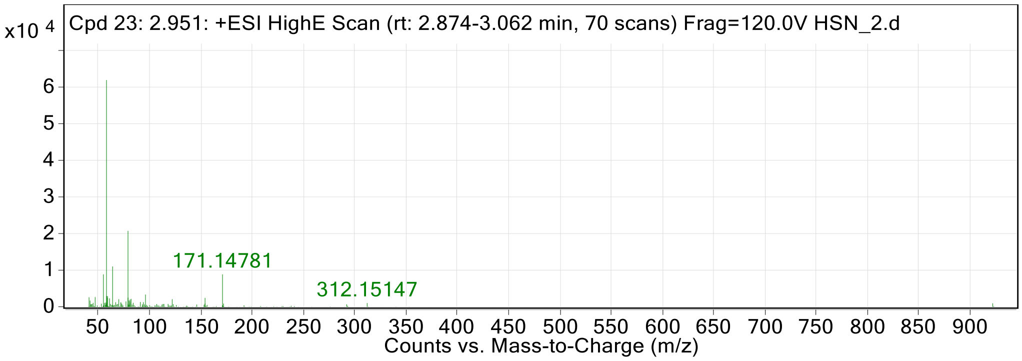

Peak List

m/z	z	Abund
41.03852		2596.37
46.995		2632.98
55.05416		8857.45
58.06512	1	61862.16
58.1323		2949.29
59.04911		2937.21
63.99751	1	11013.66
79.0207	1	20715.75
96.08009		3336.75
171.14781	1	8856.01

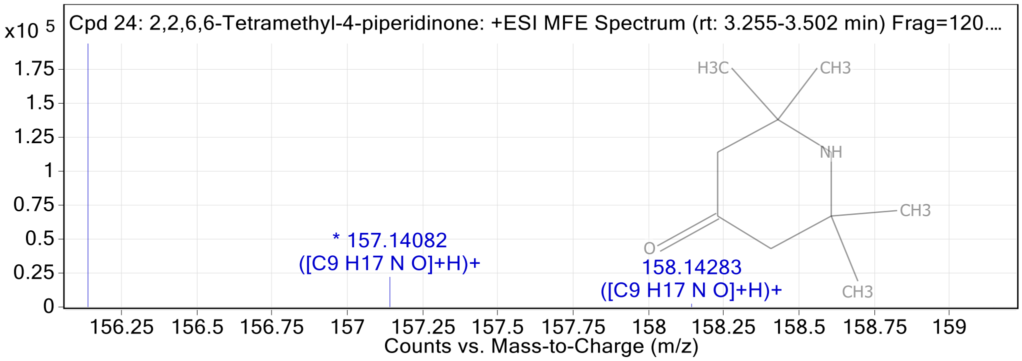

Peak List

m/z	z	Abund	Name	Formula	Ion	Score (DB)	Hits (DB)
156.1382	1	194091.95	2,2,6,6-Tetramethyl-4-piperidinone	C9 H17 N O	(M+H)+	98.62	4
157.14082	1	22194.56		C9 H17 N O	(M+H)+		
158.14283	1	2397.64		C9 H17 N O	(M+H)+		
159.14626	1	22.03		C9 H17 N O	(M+H)+		

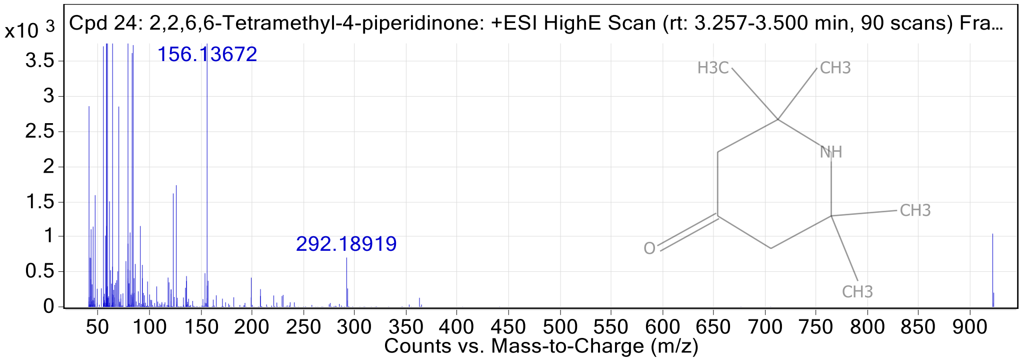

Peak List

m/z	z	Abund
41.03849		2862.59
55.05404	1	3716.25
58.06496	1	98462.05
58.13213		5041.28
59.06878	1	3762.92
63.99729	1	7020.65
79.02055	1	12347.38
83.0484	1	3620.35
84.08	1	3733.93
156.13672	1	3757.82

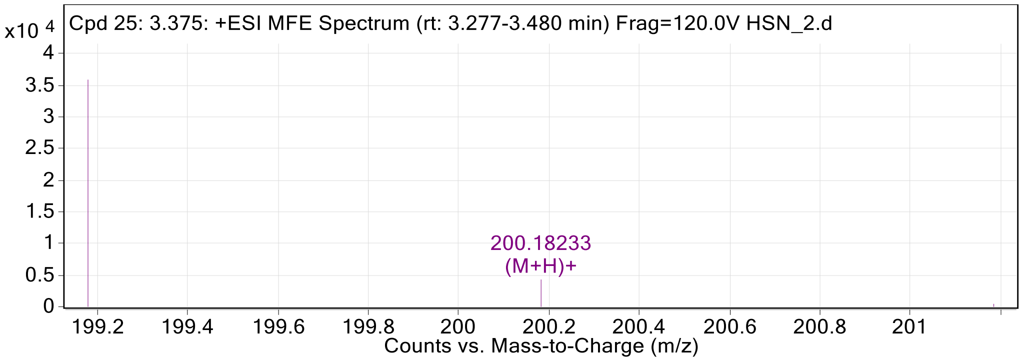

Peak List

m/z	z	Abund	Ion
199.17871	1	35872.06	(M+H)+
200.18233	1	4307.3	(M+H)+
201.18504	1	458.23	(M+H)+

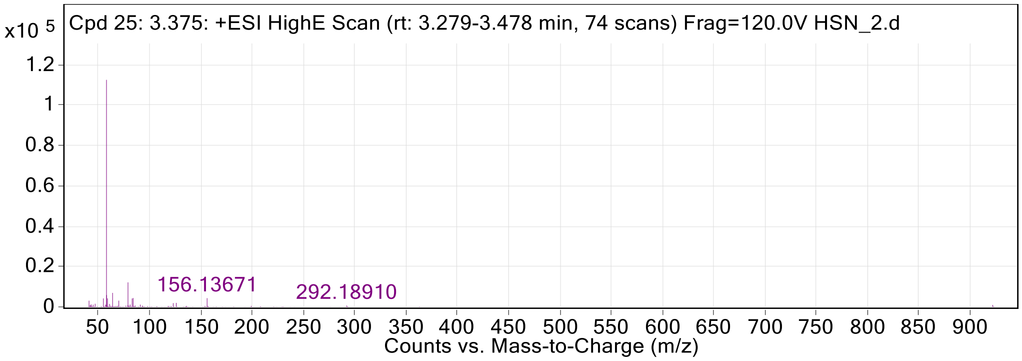

Peak List

m/z	z	Abund
55.05404	1	4201.24
58.06494	1	112442.83

58.13211		5873.29
59.06865	1	4229.43
63.99728		6938.25
70.06461		3098.32
79.02052	1	12154.96
83.04837	1	4179.57
84.07997	1	4384.73
156.13671	1	4331.32

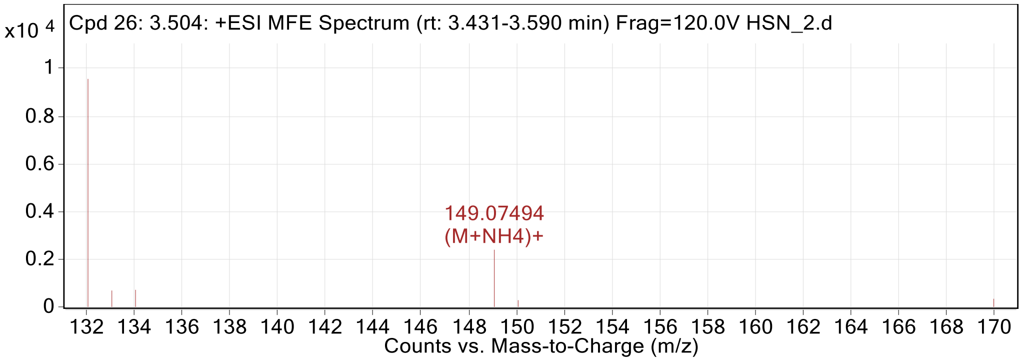

Peak List

m/z	z	Abund	Ion
132.04883	1	9560.25	(M+H)+
133.04892	1	686.38	(M+H)+
134.04212	1	715.42	(M+H)+
149.07494	1	2396.85	(M+NH4)+
150.07534	1	281.9	(M+NH4)+
170.0057	1	339.96	(M+K)+

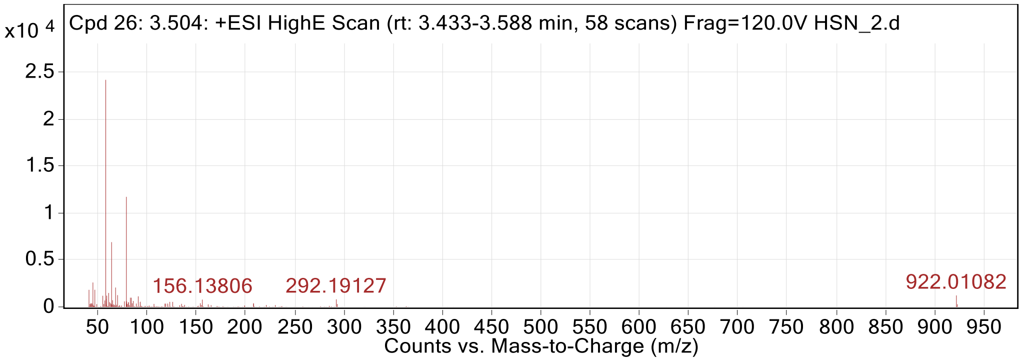

Peak List

m/z	z	Abund
41.03922		1821.05
45.03418		2602.45
46.9957		1826.99
58.06578	1	24202.25
61.0112		1486.35
63.99826		6904.28
68.04995		2065.38
70.06555		1253.33
79.02168	1	11732.83
922.01082	1	1223.09

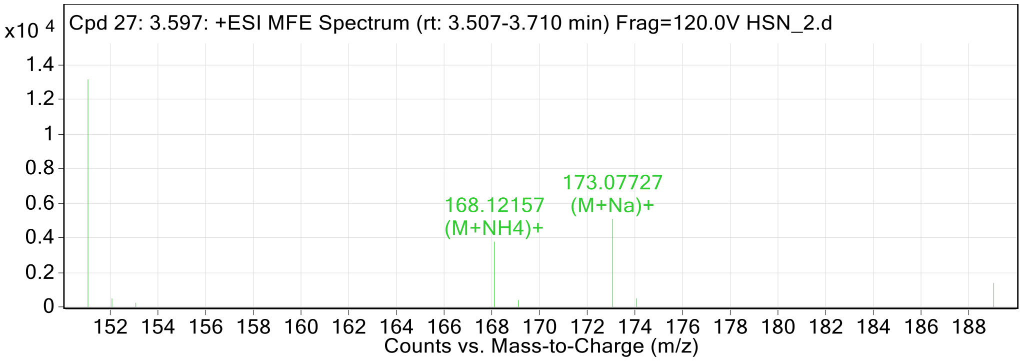

Peak List

m/z	z	Abund	Ion
151.09481	1	13149.02	(M+H)+
152.09969	1	484.49	(M+H)+
153.10004	1	239.56	(M+H)+
168.12157	1	3769.22	(M+NH4)+
169.12614	1	392.78	(M+NH4)+
173.07727	1	5080.97	(M+Na)+
174.08057	1	492.22	(M+Na)+
189.05067	1	1379.35	(M+K)+

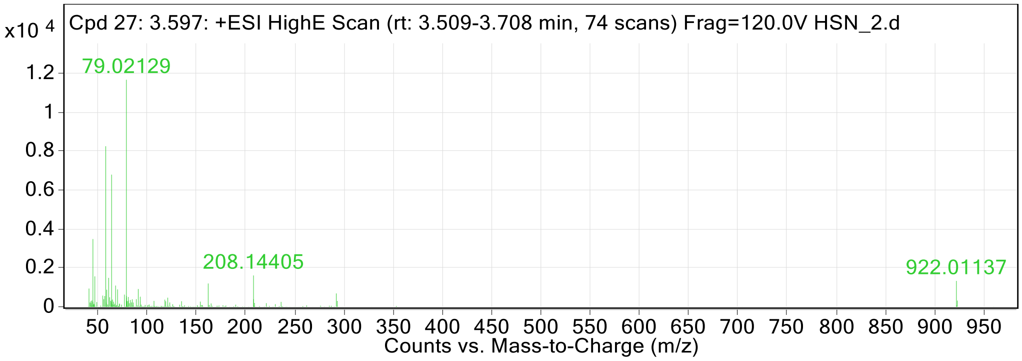

Peak List

m/z	z	Abund
45.03388		3477.3
46.9954		1561.79
58.0654	1	8242.04
61.01095		1484.83
63.99792	1	6784.8
68.04966		1089.85
79.02129	1	11656.13
162.10178	1	1195.76
208.14405	1	1608.12
922.01137	1	1327.59

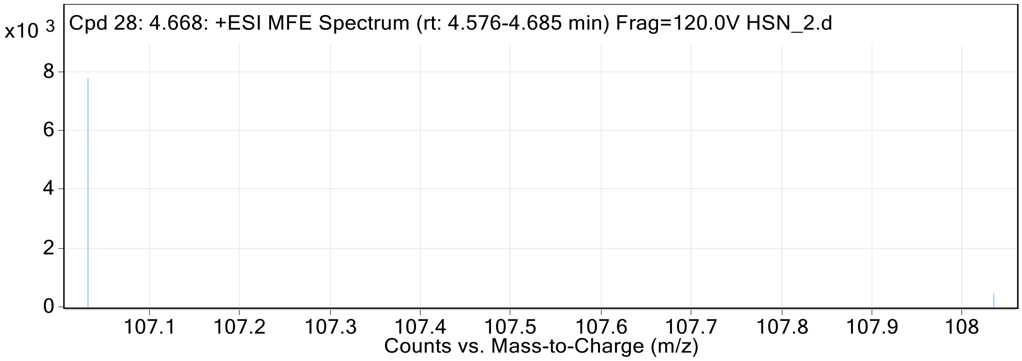

Peak List

m/z	z	Abund	Ion
107.03226	1	7778.74	(M+H)+
108.03488	1	445.56	(M+H)+

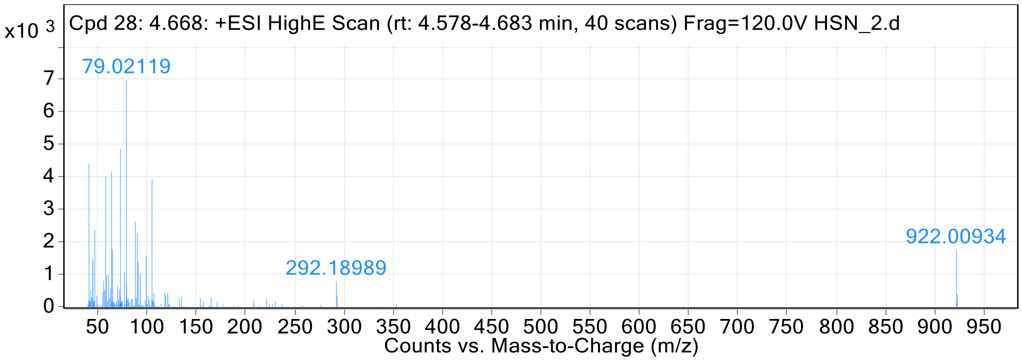

Peak List

m/z	z	Abund
41.0389	1	4408.29
46.99528		2353.46
58.06536		4018.64
63.99787		4164.98
65.00557		1763.35
73.01066	1	4864.43
79.02119	1	6985.02
88.03382	1	2618.38
90.01316		2272.88
105.03638	1	3936.72

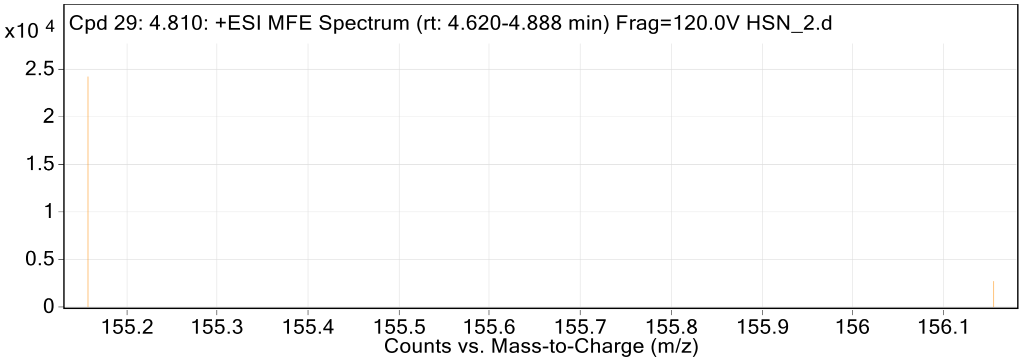

Peak List

m/z	z	Abund	Ion
155.1575	1	24203.53	(M+H)+
156.1555	1	2714	(M+H)+

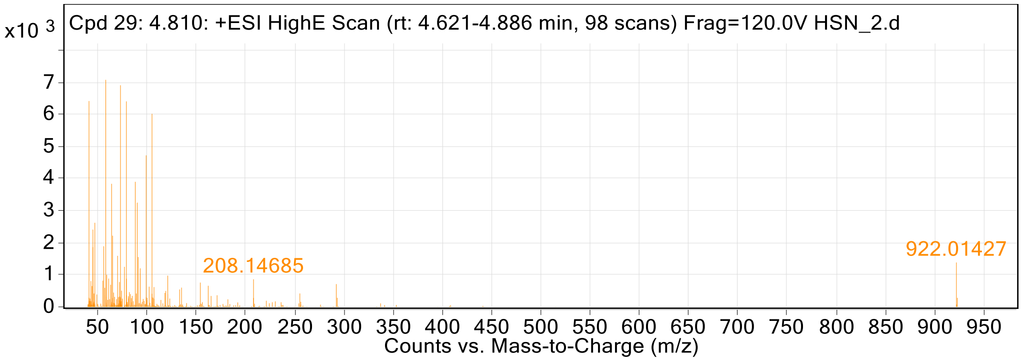

Peak List

m/z	z	Abund
41.04057	1	6412.65
46.99708		2620.16
58.06727		7072.71
63.99989		3834.24
73.01279	1	6903.98
79.0234		6401.75
88.03633	1	3896.43
90.01543		3247.4
99.09371	1	4720.6
105.03887	1	6013.66

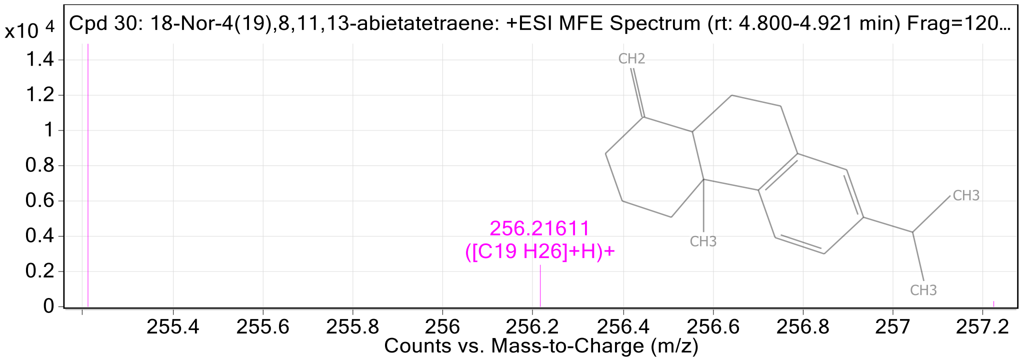

Peak List

m/z	z	Abund	Name	Formula	Ion	Score (DB)	Hits (DB)
255.21153	1	14947.74	18-Nor-4(19),8,11,13-abietatetraene	C19 H26	(M+H)+	87.44	1
256.21611	1	2383.92		C19 H26	(M+H)+		
257.22315	1	321.62		C19 H26	(M+H)+		

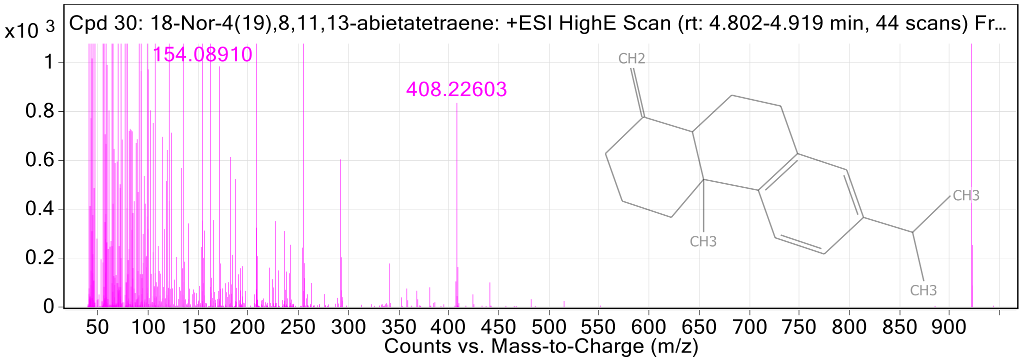

Peak List

m/z	z	Abund
41.04096		2836.06

45.03601		4089.21
56.05211		2163.09
58.06776	1	12667.23
64.00042		3586.03
70.06779		1911.84
79.02396		6263.19
91.05685		1903.26
99.09434	1	4884.31
154.0891		2445.99

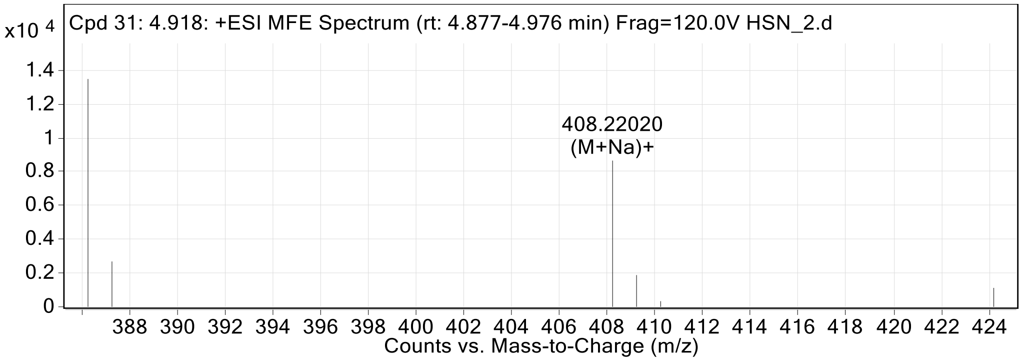

Peak List

m/z	z	Abund	Ion
386.23812	1	13486.61	(M+H)+
387.24292	1	2684.87	(M+H)+
408.2202	1	8657.18	(M+Na)+
409.22462	1	1878.46	(M+Na)+
410.23458	1	335.23	(M+Na)+
424.19288	1	1120.79	(M+K)+

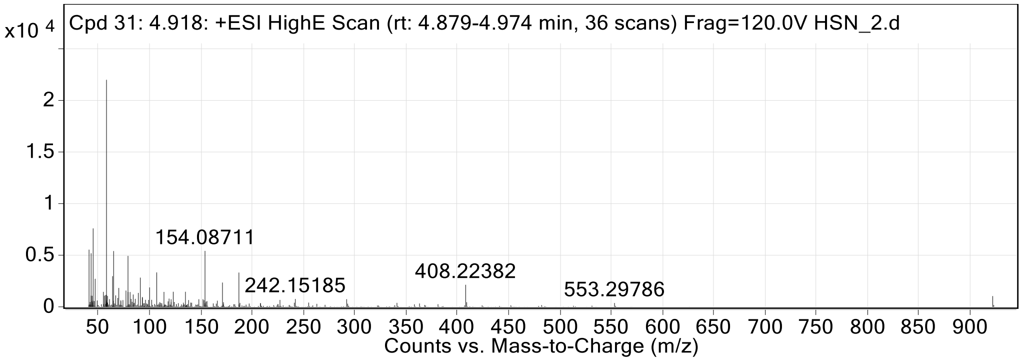

Peak List

m/z	z	Abund
41.03971		5542.43
43.05541		5193.84
45.03467		7600.86
58.06633	1	22000.84
63.99881		2972.04
65.00666		5405.94
79.02231		4936.66
107.05207		3334.61
154.08711	1	5421.65
187.14519	1	3322.09

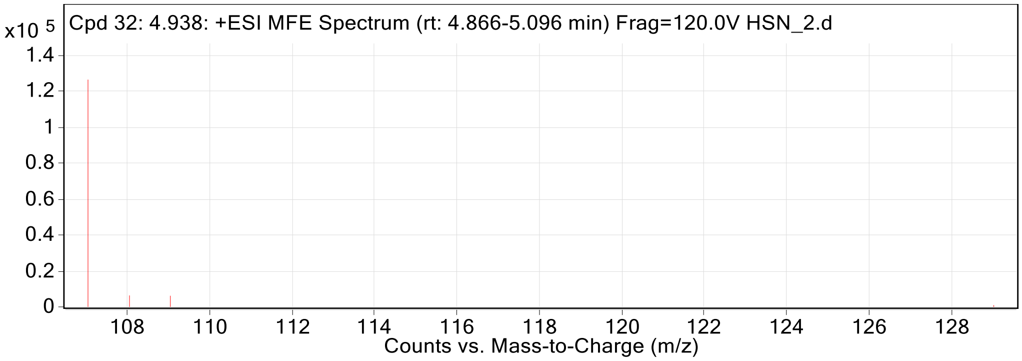

Peak List

m/z	z	Abund	Ion
107.05103	1	126551.94	(M+H)+
108.05796	1	6373.7	(M+H)+
109.04972	1	6191.56	(M+H)+
129.03264	1	965.84	(M+Na)+

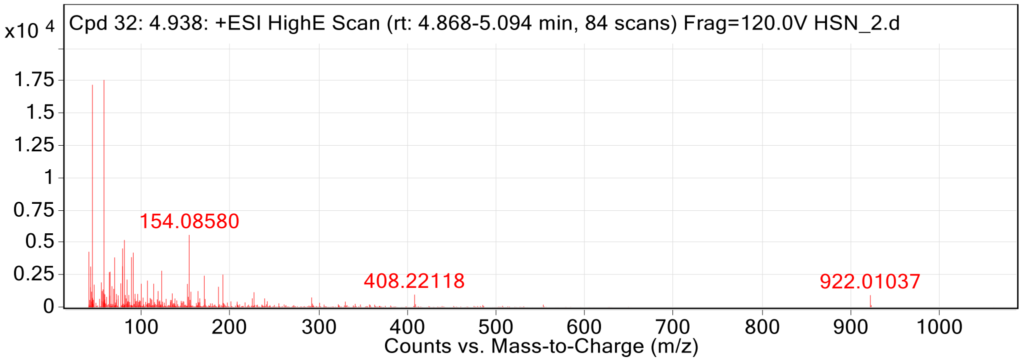

Peak List

m/z	z	Abund
41.03908		4274.72
45.03403		17187.07
58.06554	1	17558.25
70.06542		3834.9
79.02137		4527.21
79.05417		3148.21
81.07001		5184.72
89.05967		3853.42
91.05431		4206.42
154.0858	1	5577.47

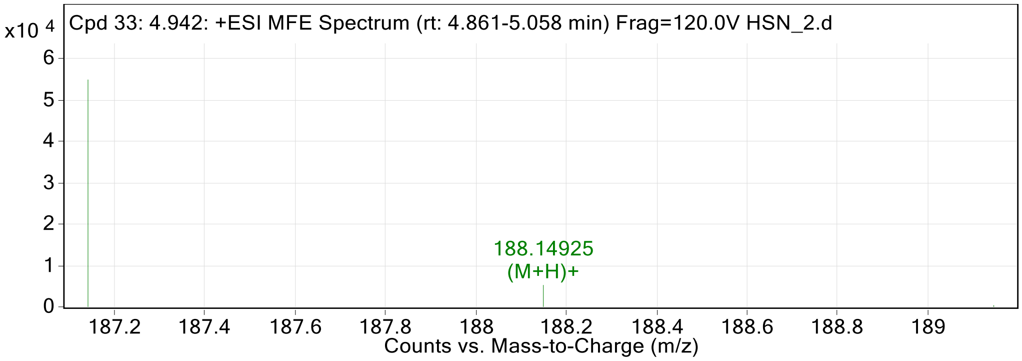

Peak List

m/z	z	Abund	Ion
187.1417	1	54932.83	(M+H)+

188.14925	1	5278.7	(M+H)+
189.14595	1	395.51	(M+H)+

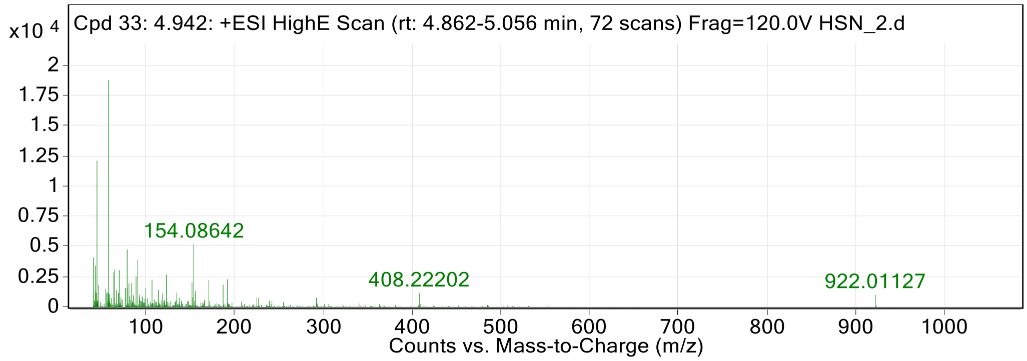

Peak List

m/z	z	Abund
41.03946		4069.23
43.0551		3391.17
45.03438		12091.71
58.06595	1	18758.47
63.99851		2832.69
65.00625		3100.15
70.06583		3040.18
79.02183		4750.07
91.05478		3861.76
154.08642	1	5167.36

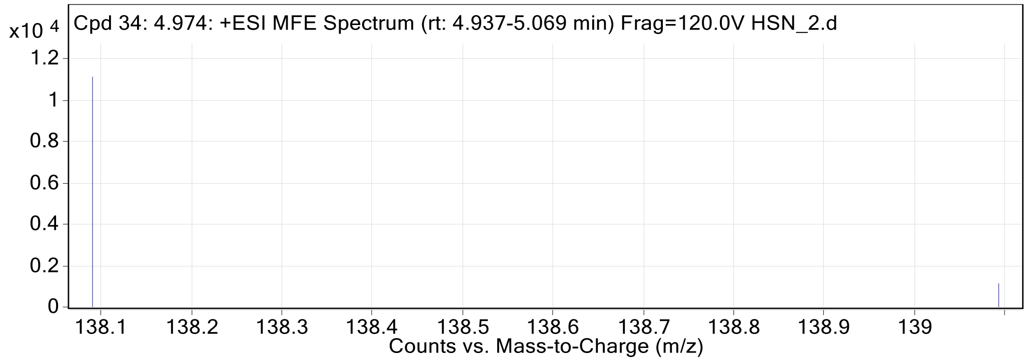

Peak List

m/z	z	Abund	Ion
138.09056	1	11145.74	(M+H)+
139.09316	1	1145.81	(M+H)+

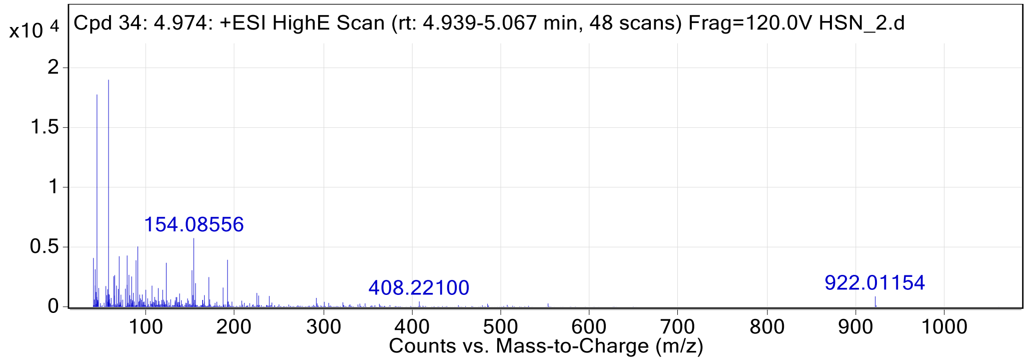

Peak List

m/z	z	Abund
41.03883		4083.36

45.03373		17764.28
58.06525	1	18996.92
70.06506		4230.23
79.02102		4291.59
89.05936		3891.48
91.05397	1	5046.45
123.11607	1	3681.94
154.08556	1	5746.19
192.11226	1	3931.59

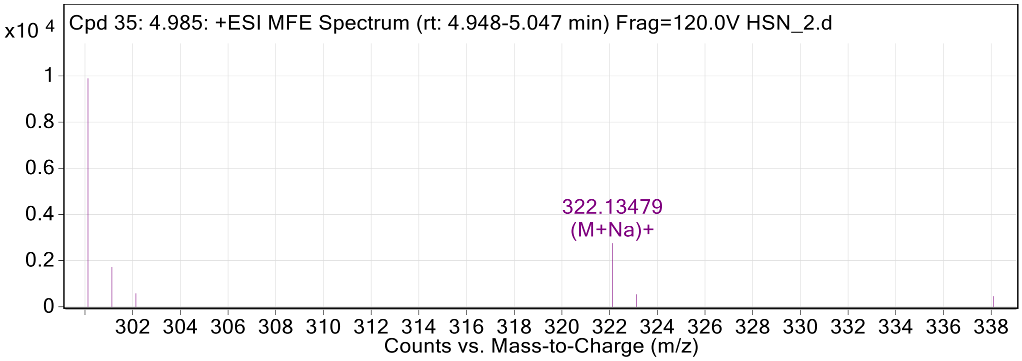

Peak List

m/z	z	Abund	Ion
300.15336	1	9908.44	(M+H)+
301.15618	1	1731.56	(M+H)+
302.16232	1	581.17	(M+H)+
322.13479	1	2756.3	(M+Na)+
323.13972	1	541.21	(M+Na)+
338.10845	1	461.84	(M+K)+

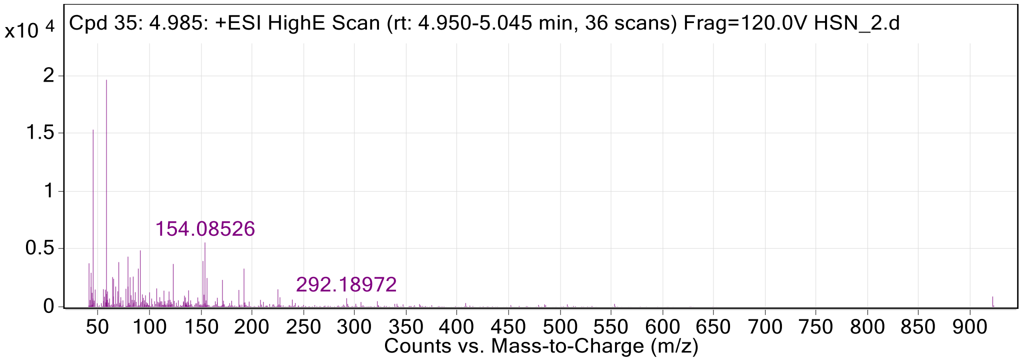

Peak List

m/z	z	Abund
41.03888		3766.16
45.03372		15326.54
58.06521	1	19636.78
70.06503		3867.13
79.02091		4339.95
89.0593		3305.15
91.05386	1	4882.5
123.11586	1	3708.12
152.10597		3965.24
154.08526	1	5566.39

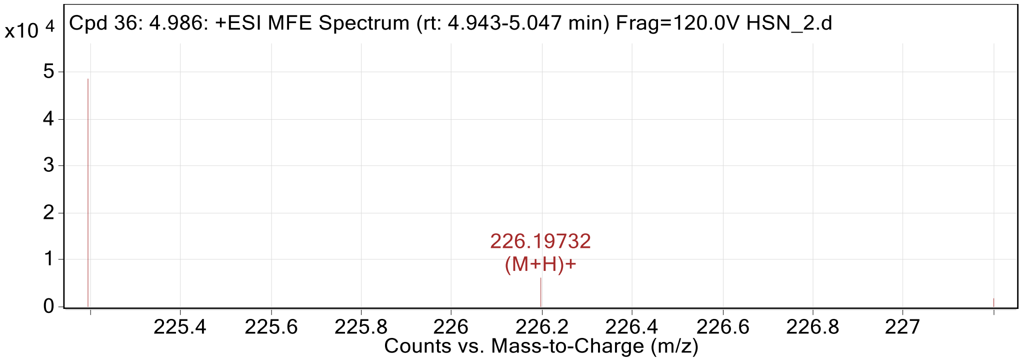

Peak List

m/z	z	Abund	Ion
225.19402	1	48537.05	(M+H)+
226.19732	1	6213.29	(M+H)+
227.20084	1	1825.04	(M+H)+

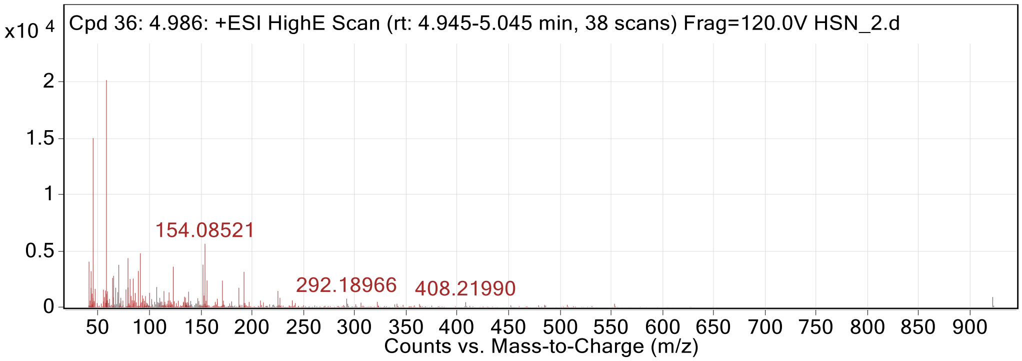

Peak List

m/z	z	Abund
41.03884		4035.9
45.03369		15018.22
58.06517	1	20176.03
70.06499		3756.66
79.02089		4351.34
89.05924		3211.03
91.05379	1	4779.78
123.1158	1	3591.17
152.10591		3771.08
154.08521	1	5625.41

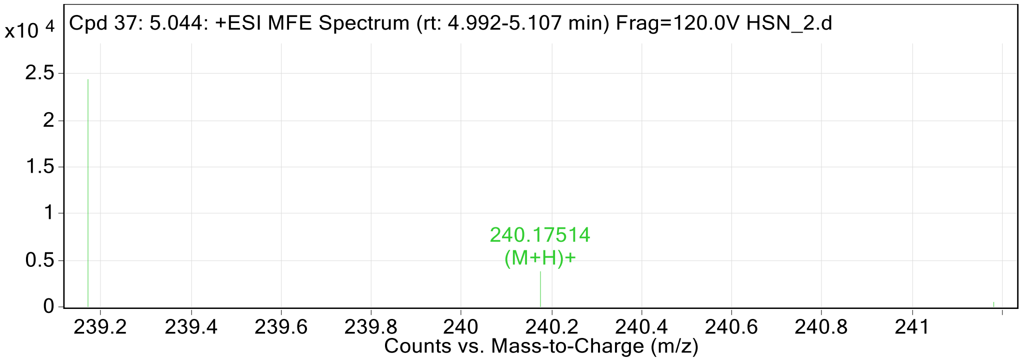

Peak List

m/z	z	Abund	Ion
239.17104	1	24408.95	(M+H)+
240.17514	1	3813.59	(M+H)+

241.18108	1	530.63	(M+H)+

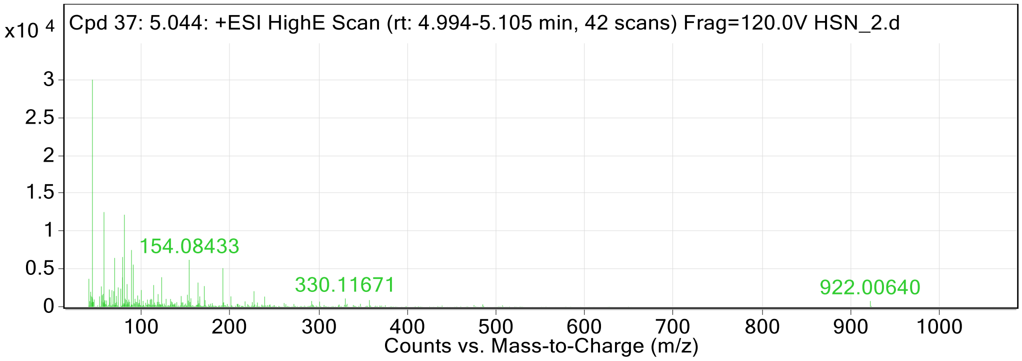

Peak List

m/z	z	Abund
45.03322	1	30024.45
58.06459	1	12519.67
70.06442		6455.97
79.0532		6574.44
81.06893	1	12172.15
89.05853		7503.13
91.05314		5569.81
123.11511		3907.48
154.08433	1	6203.61
192.11082	1	5105.58

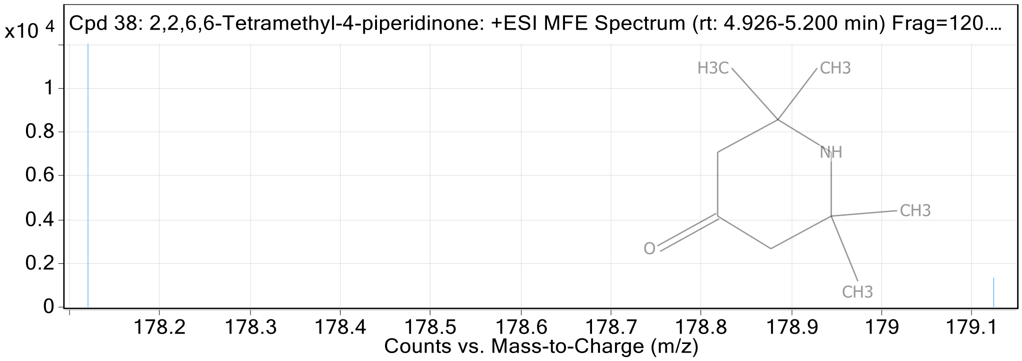

Peak List

m/z	z	Abund	Name	Formula	Ion	Score (DB)	Hits (DB)
178.12051	1	12037.19	2,2,6,6-Tetramethyl-4-piperidinone	C9 H17 N O	(M+Na)+	87	4
179.12392	1	1346.3		C9 H17 N O	(M+Na)+		

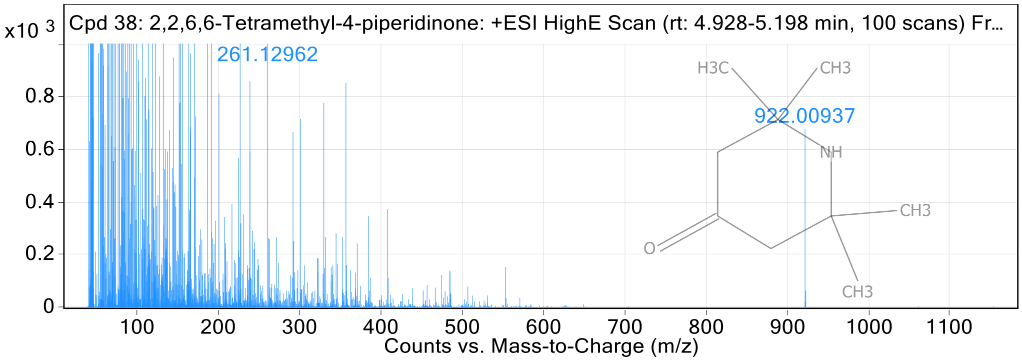

Peak List

m/z	z	Abund
41.03855		4003.2

45.03357	1	56088.6
58.06493	1	17650.04
70.06481		5126.25
79.02062		3717.68
79.05357		5098.9
81.0693		8889.92
89.05912	1	20241.58
91.05364		4107.41
154.08497	1	5488.17

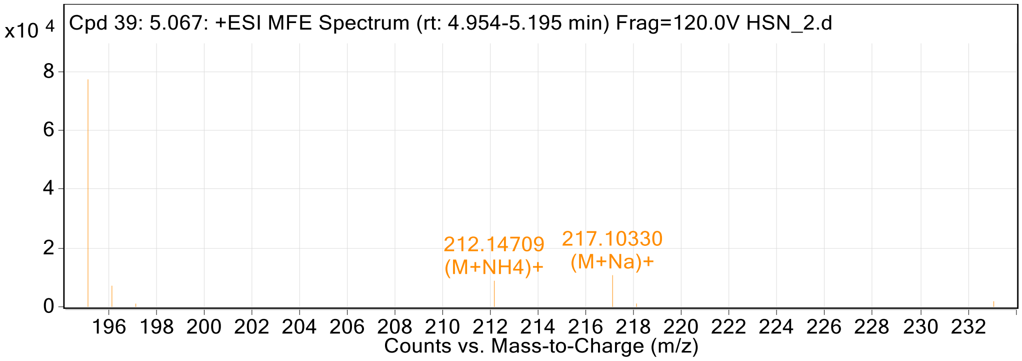

Peak List

m/z	z	Abund	Ion
195.12095	1	77415.32	(M+H)+
196.12425	1	7182.88	(M+H)+
197.1253	1	1085.29	(M+H)+
212.14709	1	8867.16	(M+NH4)+
217.1033	1	10768.88	(M+Na)+
218.10684	1	1122.58	(M+Na)+
233.07662	1	1899.2	(M+K)+

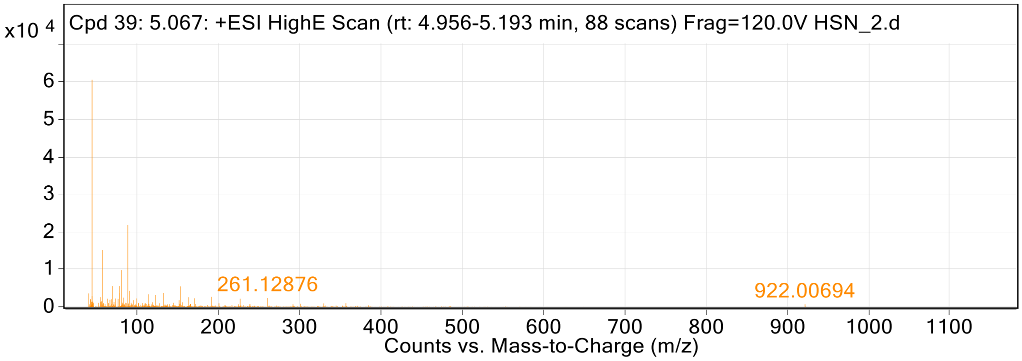

Peak List

m/z	z	Abund
45.03332	1	60568.77
58.06464	1	15194.92
70.06448		5548.4
79.02027		3667.29
79.05324		5543.73
81.06893		9809.49
89.05873	1	21874.69
91.05323		4263.92
133.08416	1	3708.04
154.08439	1	5428.77

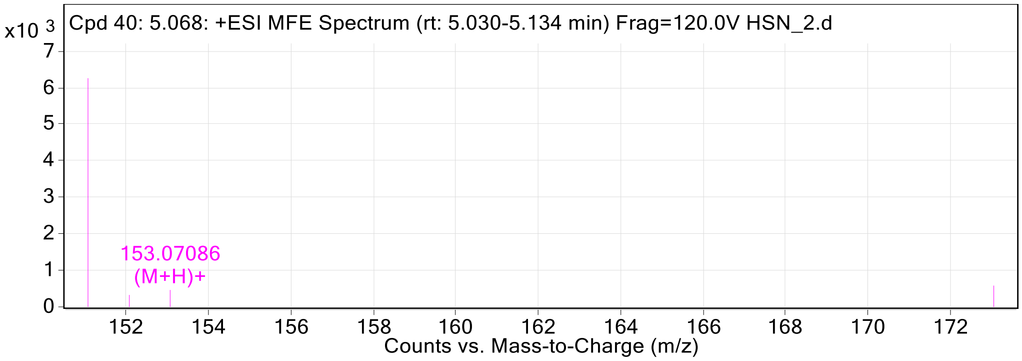

Peak List

m/z	z	Abund	Ion
151.07663	1	6256.78	(M+H)+
152.07936	1	326.52	(M+H)+
153.07086	1	463.04	(M+H)+
173.05886	1	581.91	(M+Na)+

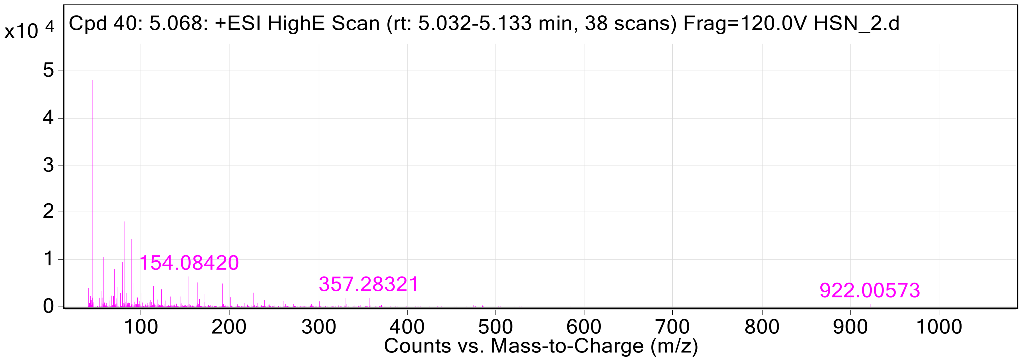

Peak List

m/z	z	Abund
45.0332	1	48021.53
58.06455	1	10507.65
70.06439		8006.96
79.0532	1	9511.38
81.06887	1	18090.26
89.05854		14419.47
91.0532		5113.75
154.0842	1	6439.31
164.04516	1	5167.45
192.11067		4940.85

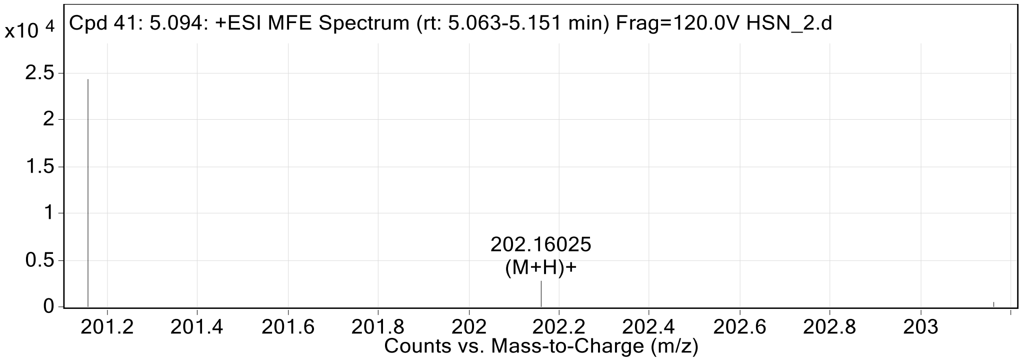

Peak List

m/z	z	Abund	Ion
201.15748	1	24353.22	(M+H)+

202.16025	1	2785.8	(M+H)+
203.16168	1	514.51	(M+H)+

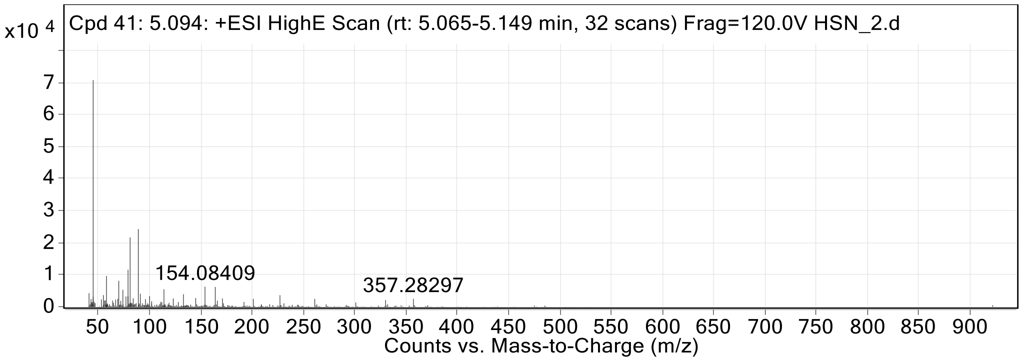

Peak List

m/z	z	Abund
45.03322	1	70756.59
58.06451		9613.02
70.06439		8106.35
74.05912		5312.47
79.05315	1	11561.47
81.06883	1	21652.6
89.05852	1	24257.01
114.08948		5464.07
154.08409	1	6275.52
164.04508	1	6189.2

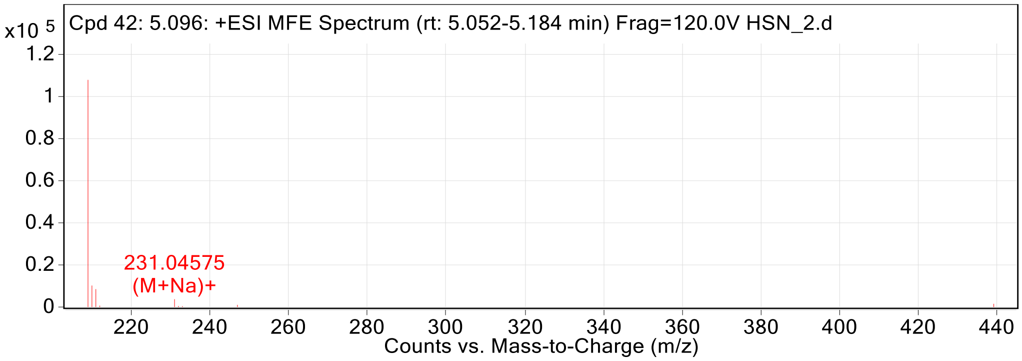

Peak List

m/z	z	Abund	Ion
209.06425	1	107833.52	(M+H)+
210.06691	1	10198.92	(M+H)+
211.0604	1	8498.98	(M+H)+
212.06362	1	661.75	(M+H)+
231.04575	1	3727.56	(M+Na)+
232.04861	1	489.53	(M+Na)+
233.04411	1	413.01	(M+Na)+
247.0198	1	1032.93	(M+K)+
439.10356	1	1529.37	(2M+Na)+

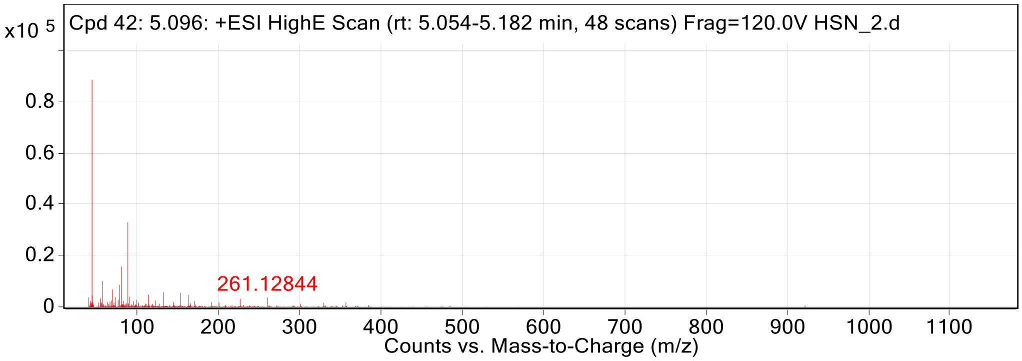

Peak List

m/z	z	Abund
45.03326	1	88425.41
58.06452	1	10038.17
70.0644		6817.28
79.05316	1	8571.64
81.06882	1	15646.81
89.0586	1	32998.89
114.08953		4740.81
133.08397		5623.64
154.08416	1	5497.88
164.04507	1	4603.96

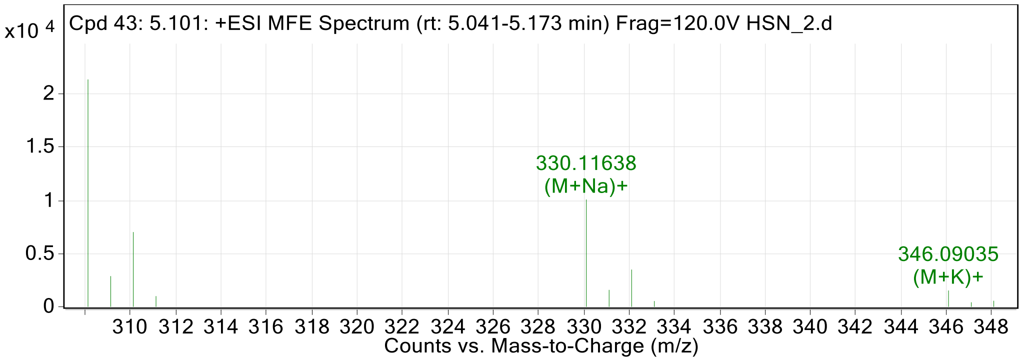

Peak List

m/z	z	Abund	Ion
308.13442	1	21366.69	(M+H)+
309.13744	1	2882.2	(M+H)+
310.13228	1	7021.57	(M+H)+
311.13492	1	1001.6	(M+H)+
330.11638	1	10079.93	(M+Na)+
331.11993	1	1588.4	(M+Na)+
332.11384	1	3495.89	(M+Na)+
333.11471	1	538.33	(M+Na)+
346.09035	1	1532.05	(M+K)+
348.0869	1	564.28	(M+K)+

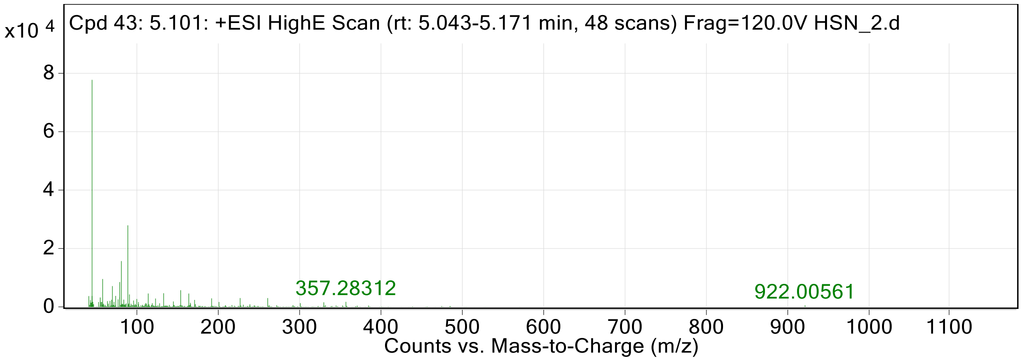

Peak List

m/z	z	Abund
45.03326	1	77851.48
58.06454		9557.18
70.06441		7133.08
79.05317	1	8515.94
81.06885	1	15708.34
89.05863	1	27959.58
114.08955		4584.83
133.08401		4686.57
154.0842	1	5727.35
164.04514	1	4572.37

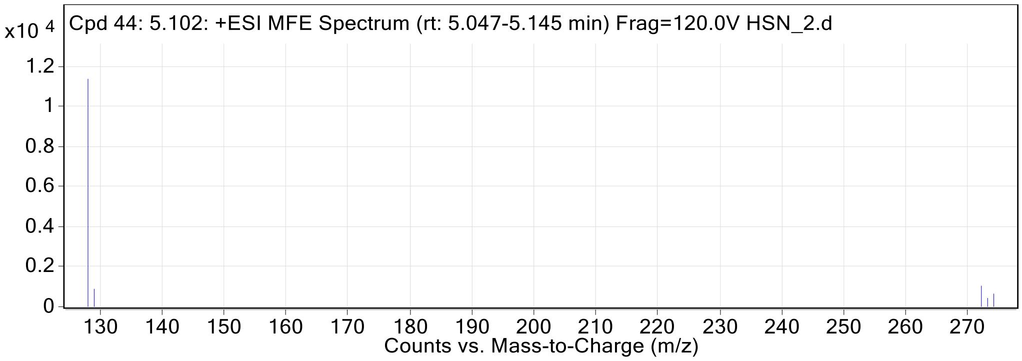

Peak List

m/z	z	Abund	Ion
128.06881	1	11365.85	(M+H)+
129.07099	1	892.67	(M+H)+
272.16048	1	1048.01	(2M+NH4)+
273.1668	1	438.1	(2M+NH4)+
274.15984	1	654.97	(2M+NH4)+

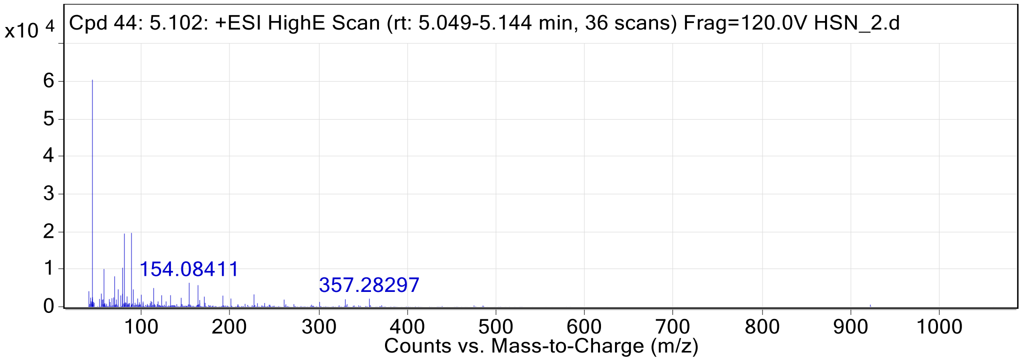

Peak List

m/z	z	Abund

45.0332	1	60418.83
58.0645	1	10052.01
70.06437		8104.39
74.05914		4647.08
79.05314	1	10387.93
81.06882	1	19478.21
89.05851	1	19653.62
114.08948		4987.62
154.08411	1	6383.18
164.04507	1	5755.14

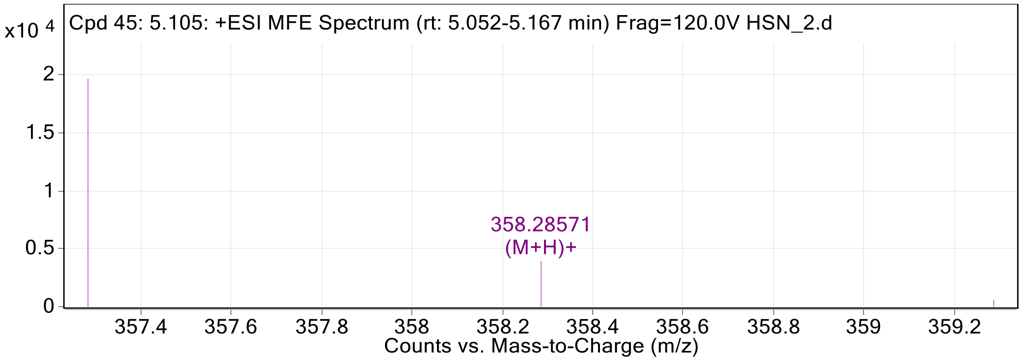

Peak List

m/z	z	Abund	Ion
357.28311	1	19693.03	(M+H)+

m/z	z	Abund	Ion
358.28571	1	3960.95	(M+H)+
359.28724	1	612.87	(M+H)+

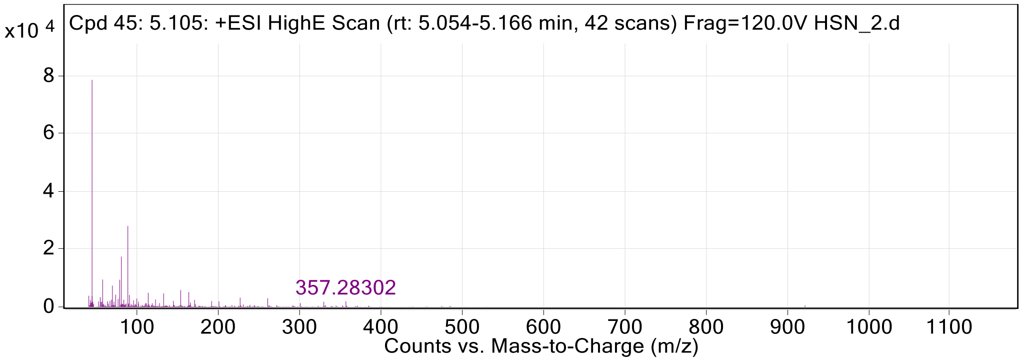

Peak List

m/z	z	Abund
45.03324	1	78647.85
58.06453		9482.46
70.0644		7381.72
79.05315	1	9430.27
81.06882	1	17441.13
89.05857	1	28038.5
114.08949		4887.44
133.08396		4709.52
154.08412	1	5845.33
164.04508	1	5156.75

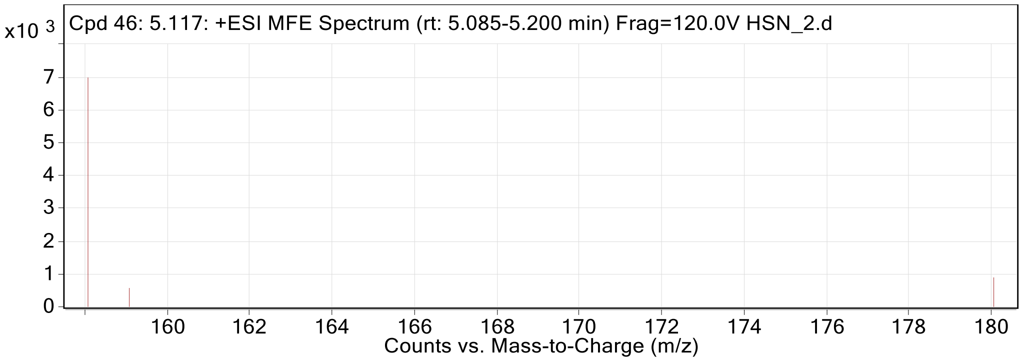

Peak List

m/z	z	Abund	Ion
158.07914	1	6987.22	(M+H)+
159.08162	1	573.57	(M+H)+
180.06083	1	895.79	(M+Na)+

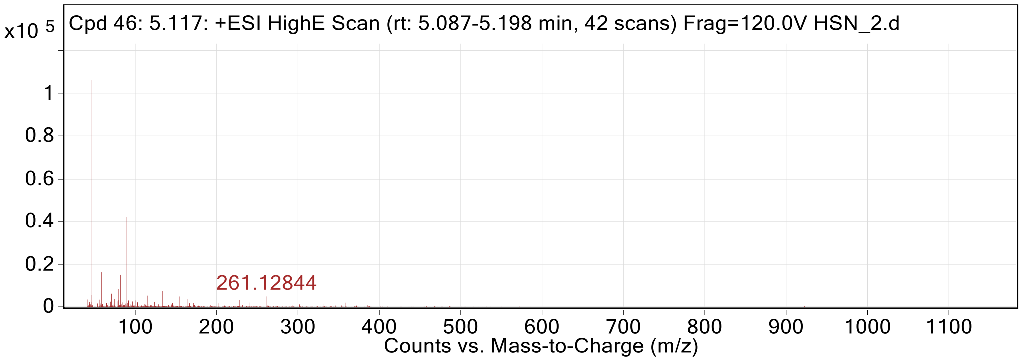

Peak List

m/z	z	Abund
45.03328	1	106351.44
45.09241		5480.7
58.06457	1	16178.14
70.06444		6152.38
79.05318	1	8323.21
81.06883	1	15025.16
89.05862	1	42133.42
114.0895		5233.27
133.084		7331.47
261.12844	1	4853.49

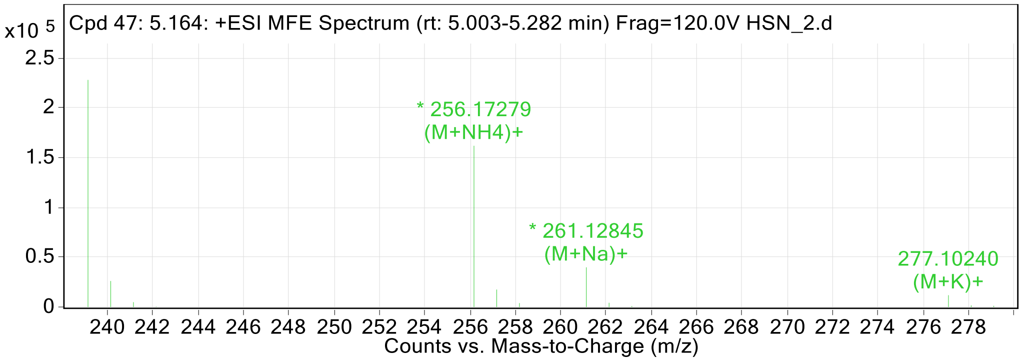

Peak List

m/z	z	Abund	Ion
239.14646	1	227857.66	(M+H)+
240.15	1	26070.61	(M+H)+
241.15178	1	4849.37	(M+H)+
256.17279	1	161768.13	(M+NH4)+
257.17635	1	17500.28	(M+NH4)+
258.17742	1	3908.01	(M+NH4)+
261.12845	1	39794.83	(M+Na)+
262.13207	1	4228.94	(M+Na)+
277.1024	1	11829.2	(M+K)+
278.10605	1	1573.29	(M+K)+

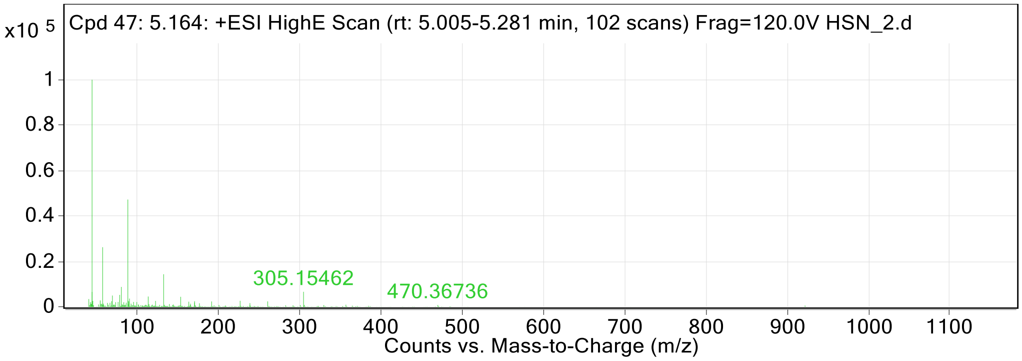

Peak List

m/z	z	Abund
45.03346	1	99852
45.09242		6518.38
58.06462	1	26198.82
70.06442		4933.25
79.05318		5217.39
81.06886		8721.55
89.05893	1	47161.73
114.08959		4495.36

133.08412	1	14308.08
305.15462	1	6581.19

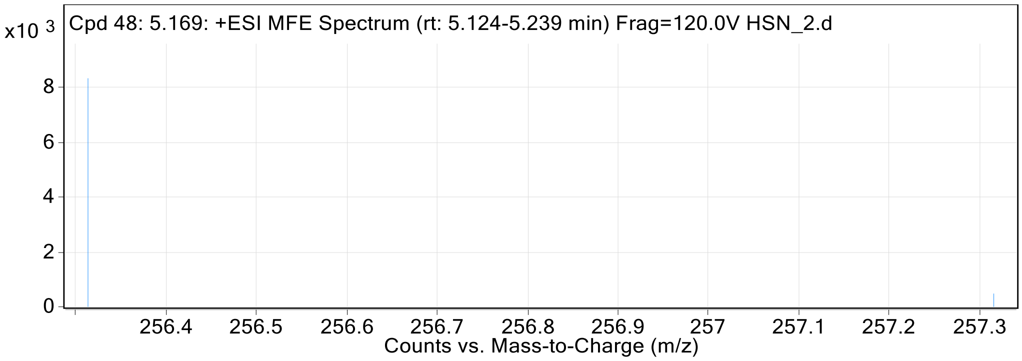

Peak List

m/z	z	Abund	Ion
256.31358	1	8342.76	(M+H)+
257.31585	1	483.64	(M+H)+

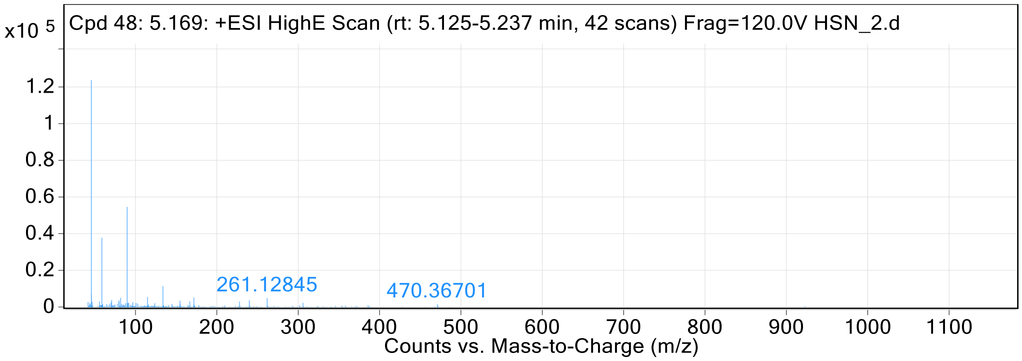

Peak List

m/z	z	Abund
45.0333	1	123461.89
45.09241		6683.66
58.06462	1	37653.07
70.06446		3799.53
81.06882		4990.27
89.05865	1	54431.38
114.08952	1	5482.7
133.08395	1	11321.2
171.1469		5082.16
261.12845	1	4829.96

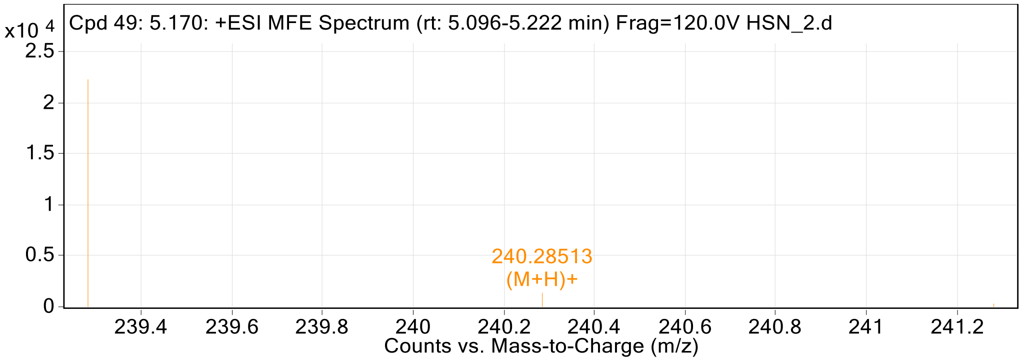

Peak List

m/z	z	Abund	Ion

239.28229	1	22302.48	(M+H)+
240.28513	1	1374.89	(M+H)+
241.28158	1	310.87	(M+H)+

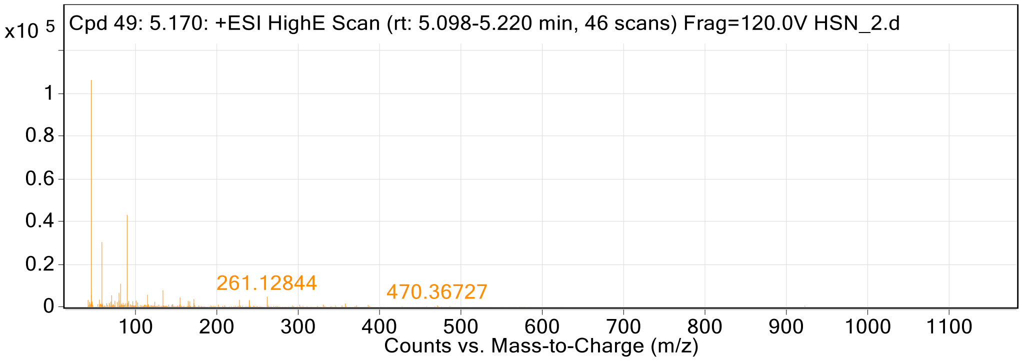

Peak List

m/z	z	Abund
45.03327	1	106228.25
45.0924		5398.59
58.06461	1	30293.54
70.06443		5362.91
79.05319		6452.38
81.06882		10810.84
89.05861	1	43044.66
114.08952		5592.52
133.084		7712.08
261.12844	1	4704.04

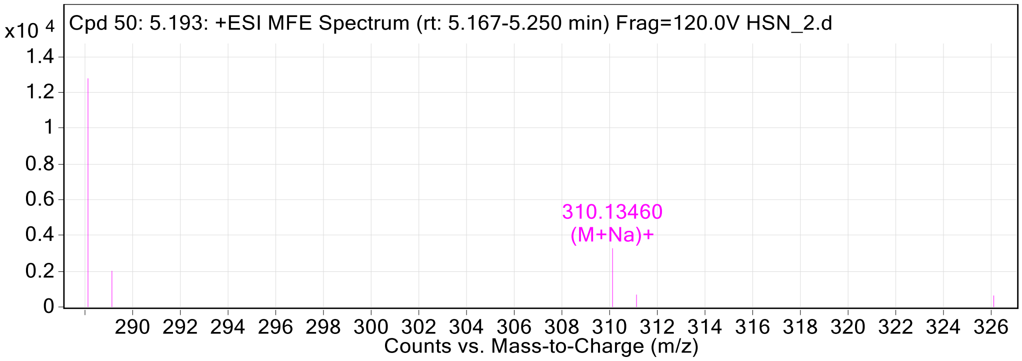

Peak List

m/z	z	Abund	Ion
288.15273	1	12779.21	(M+H)+
289.15604	1	2019.01	(M+H)+
310.1346	1	3281.77	(M+Na)+
311.13885	1	690.91	(M+Na)+
326.10971	1	639.92	(M+K)+

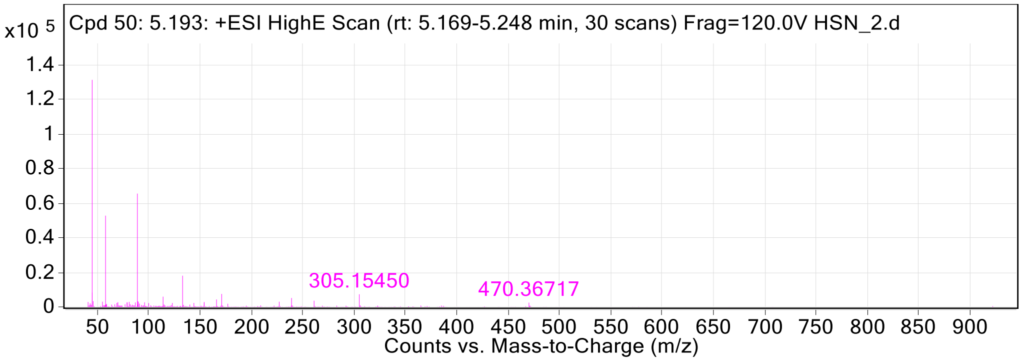

Peak List

m/z	z	Abund
45.03341	1	131397.34
45.09241		8256.69
58.06463	1	52799.46
89.05879	1	65567.8
114.08957		5916.37
133.08399	1	18041.9
166.12032		4298.38
171.14694	1	7495.86
239.20948	1	5148.9
305.1545	1	7297.63

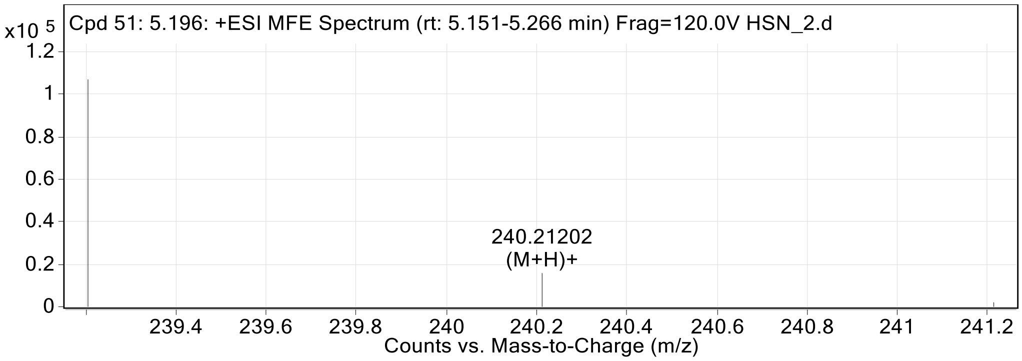

Peak List

m/z	z	Abund	Ion
239.20452	1	107051.43	(M+H)+
240.21202	1	15848.3	(M+H)+
241.21407	1	2087.38	(M+H)+

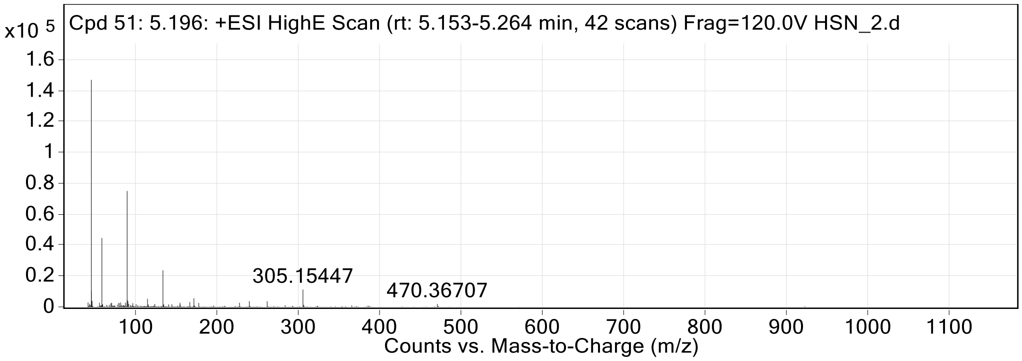

Peak List

m/z	z	Abund
45.0335	1	146817.5
45.0924		10465.37

46.03656	1	3960.52
58.06461	1	44557.73
89.05893	1	74957.21
89.14177		4655.06
114.08958		5196.97
133.08404	1	23679.22
171.14694	1	5581.88
305.15447	1	11234.45

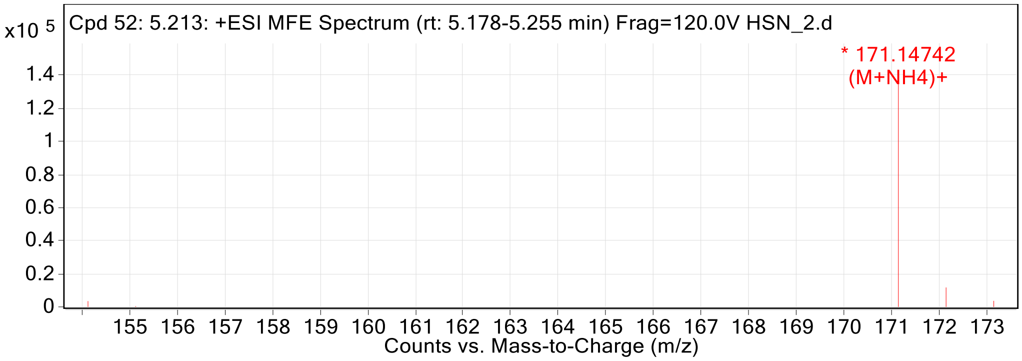

Peak List

m/z	z	Abund	Ion
154.12071	1	3487.07	(M+H)+
155.12332	1	437.4	(M+H)+
171.14742	1	137254.34	(M+NH4)+
172.15023	1	11731.07	(M+NH4)+
173.15232	1	3649.86	(M+NH4)+

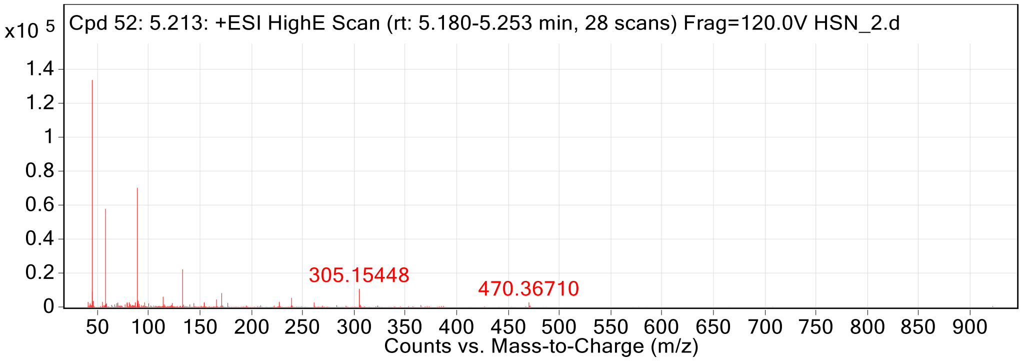

Peak List

m/z	z	Abund
45.03348	1	133520.86
45.0924		9147.16
58.06462	1	57708.45
89.05889	1	70083.08
114.08955		5997.75
133.08403	1	22151.6
166.12035		4400.25
171.14693	1	8163.63

m/z	z	Abund
239.20951	1	5345.53
305.15448	1	10689.56

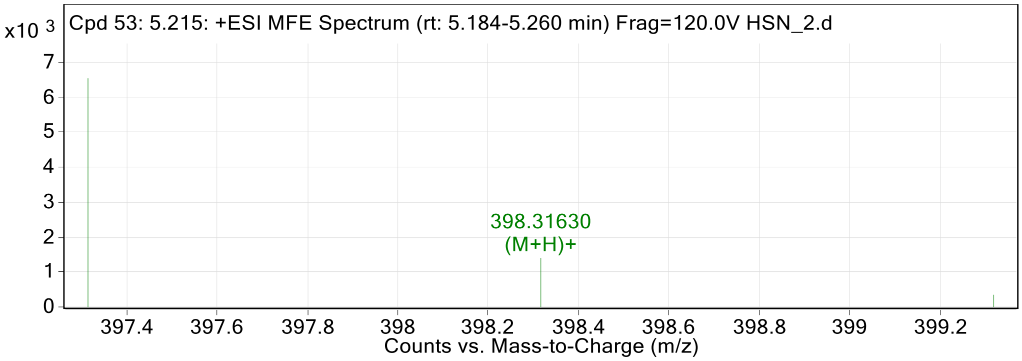

Peak List

m/z	z	Abund	Ion
397.31466	1	6540.5	(M+H)+
398.3163	1	1403.25	(M+H)+
399.31804	1	348.63	(M+H)+

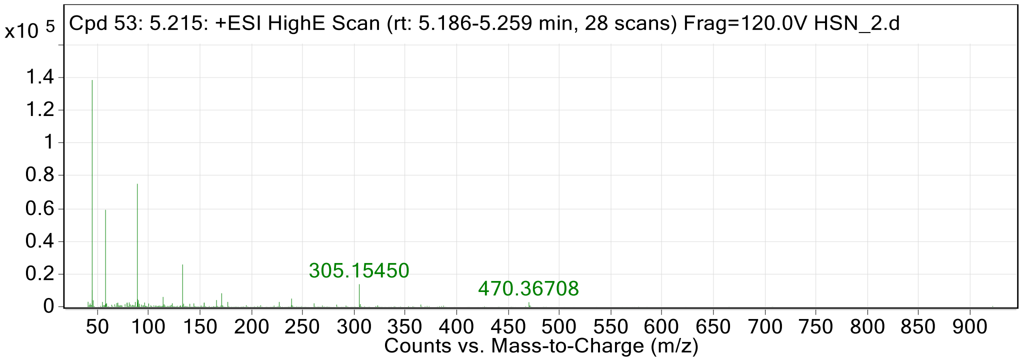

Peak List

m/z	z	Abund
45.03354	1	138500.67
45.09239		10178.46
58.06462	1	59210.64
89.05896	1	75110.1
89.14177		4719.52
114.08955		5989.56
133.08401	1	25812.36
171.14693	1	8241.79
239.20951	1	4977.47
305.1545	1	13765.83

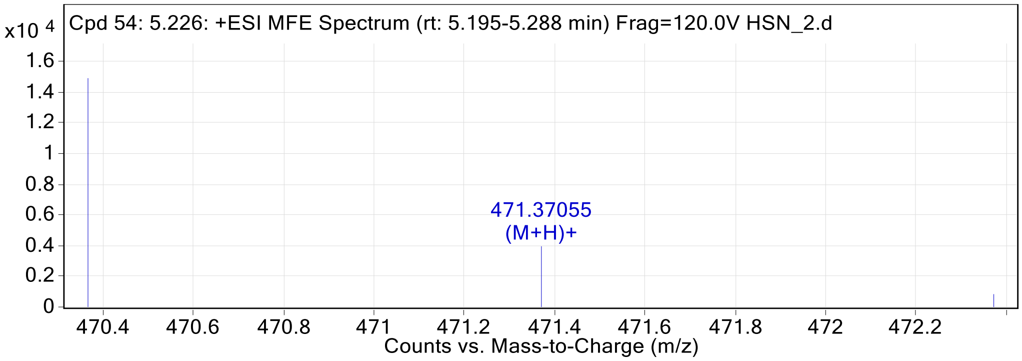

Peak List

m/z	z	Abund	Ion
470.36682	1	14895.97	(M+H)+
471.37055	1	3950.62	(M+H)+
472.372	1	835.75	(M+H)+

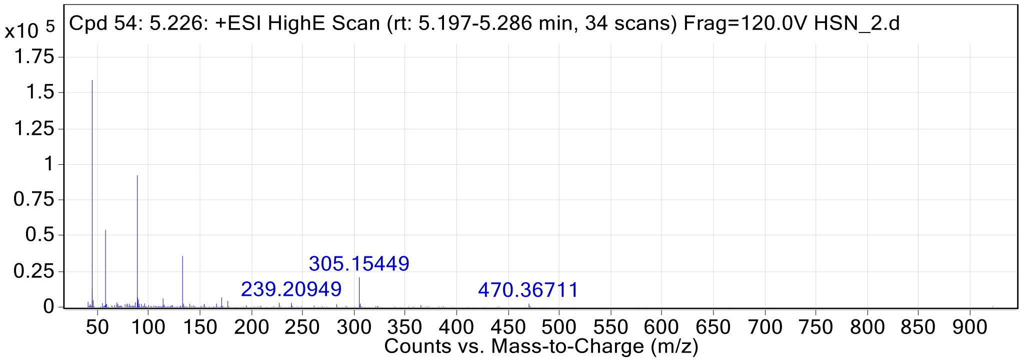

Peak List

m/z	z	Abund
45.03361	1	159074.47
45.0924		12948.63
45.10842		6412.19
58.06462	1	54016.83
89.05906	1	92370.95
89.14178		6831.65
114.08955	1	6022.1
133.08408	1	35744.71
171.14695	1	6704.41
305.15449	1	20821.48

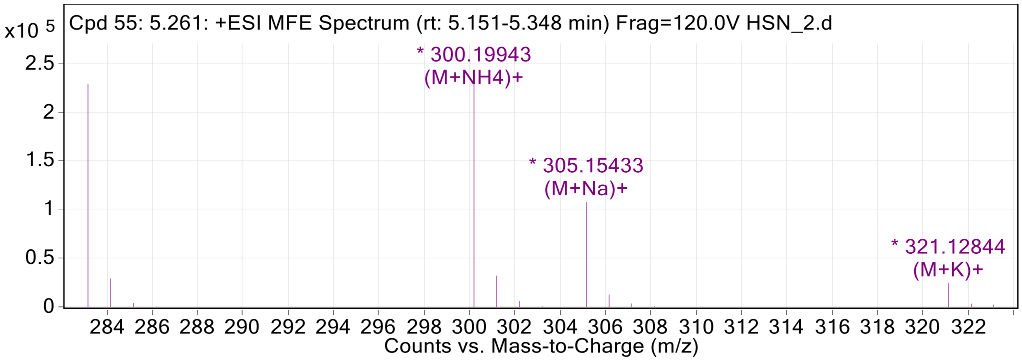

Peak List

m/z	z	Abund	Ion
283.17244	1	229180.19	(M+H)+
284.17614	1	29136.56	(M+H)+
285.17766	1	4065.48	(M+H)+
300.19943	1	233237.44	(M+NH4)+
301.20218	1	32088.43	(M+NH4)+
302.20439	1	5988.33	(M+NH4)+
305.15433	1	107797.36	(M+Na)+
306.15805	1	12631.27	(M+Na)+
307.15975	1	3468.47	(M+Na)+
321.12844	1	24374.79	(M+K)+

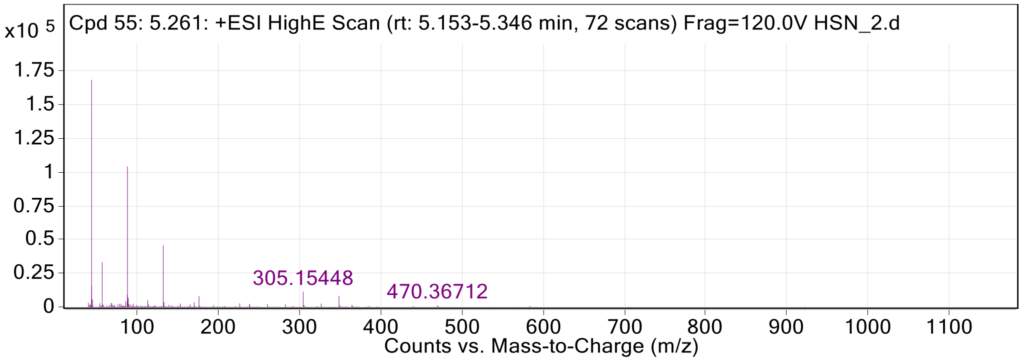

Peak List

m/z	z	Abund
45.03377	1	168358.23
45.09241		15443.85
45.10842		7275.73
58.06461	1	33033.16
89.05936	1	104125.16
89.14179		9154.8
133.08445	1	45500.29
177.10985	1	8033.14
305.15448	1	11275.44
349.18043	1	8193.98

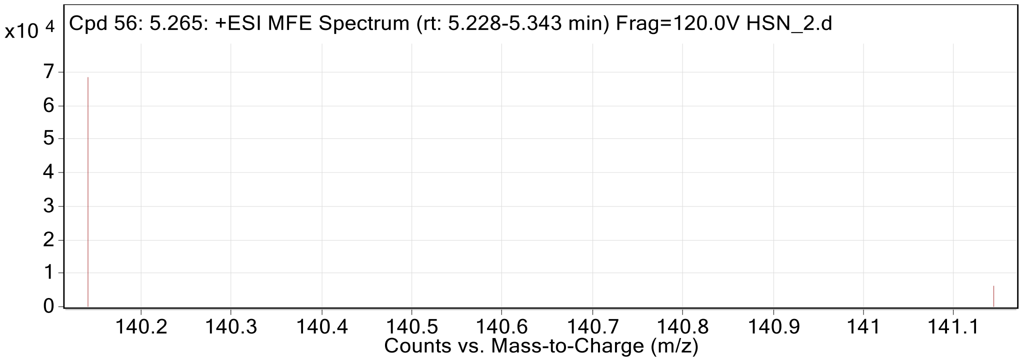

Peak List

m/z	z	Abund	Ion
140.14147	1	68493.71	(M+H)+
141.14437	1	6251.21	(M+H)+

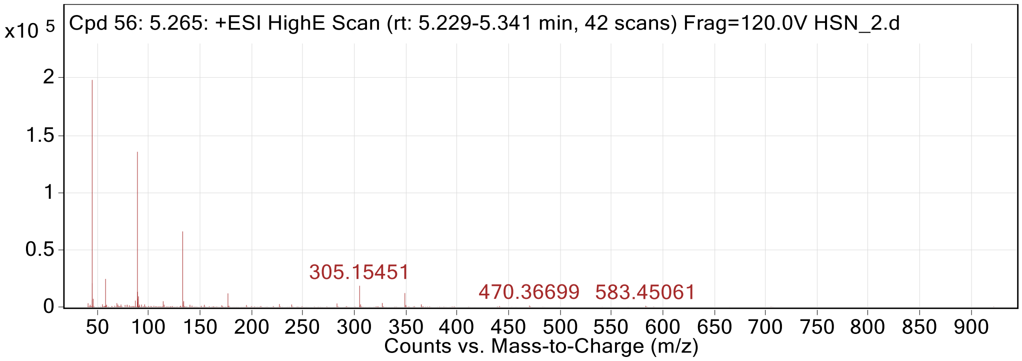

Peak List

m/z	z	Abund
45.03392	1	198317.31
45.0924		20752.56
45.10842		11408.26

58.06458	1	24516.7
89.05948	1	135539.17
89.14179		13231.41
133.08445	1	65976.67
177.10985	1	11870.2
305.15451	1	18535.18
349.18044	1	12129.32

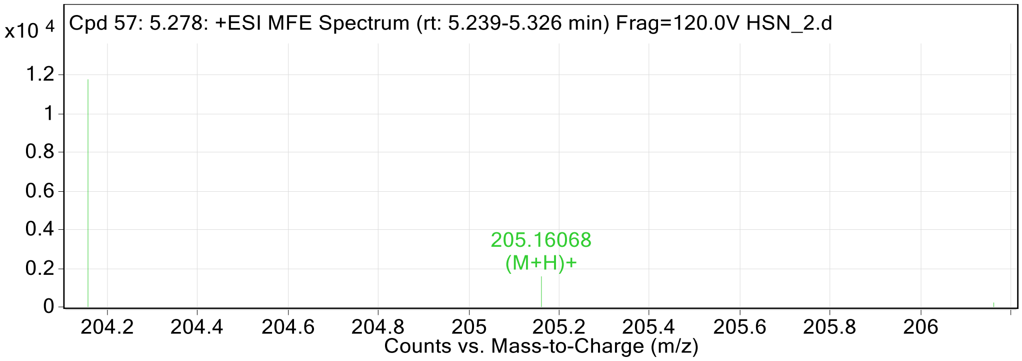

Peak List

m/z	z	Abund	Ion
204.1571	1	11770.6	(M+H)+
205.16068	1	1581.13	(M+H)+
206.16186	1	227.06	(M+H)+

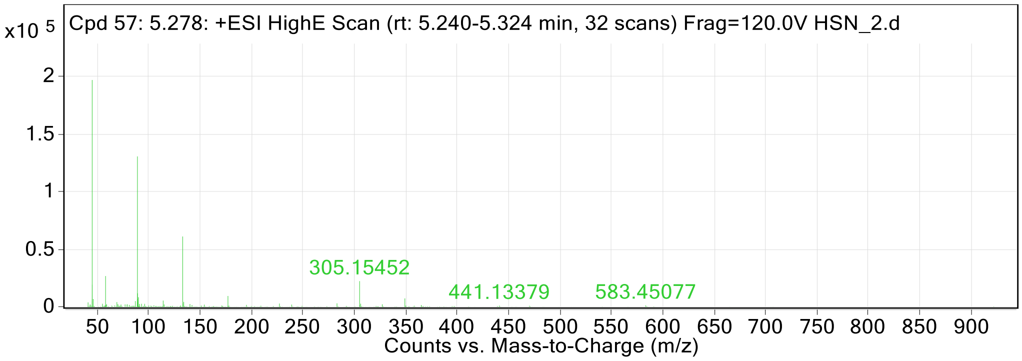

Peak List

m/z	z	Abund
45.0338	1	196918.58
45.0924		19091.23
45.10843		10199.98
58.06458	1	26644.88
89.05928	1	130534.12
89.14181		11643.74
90.06194	1	8224.36
133.08425	1	60998.94
177.10989	1	9319.68
305.15452	1	22222.85

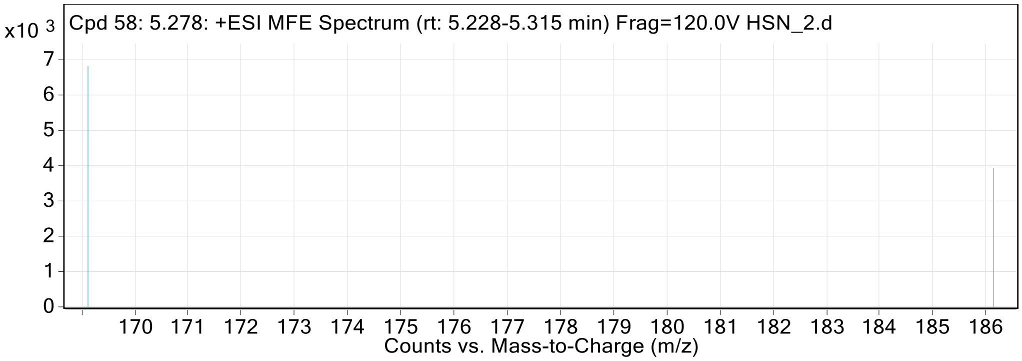

Peak List

m/z	z	Abund	Ion
169.12042	1	6824.05	(M+H)+
186.14647	1	3934.4	(M+NH4)+

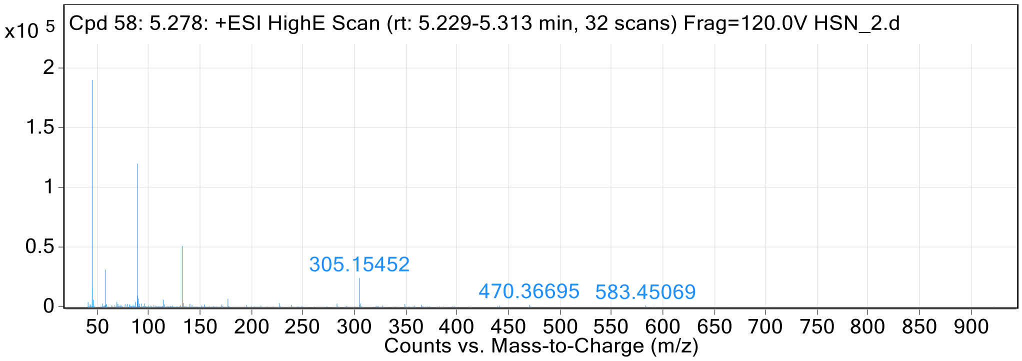

Peak List

m/z	z	Abund
45.03365	1	189606.67
45.0549		6619.63
45.0924		16324.9
45.10842		7837.37
58.06458	1	31031.21
89.05907	1	119638.38
89.14179		9334.03
90.06193	1	6840.59
133.08406	1	50713.2
305.15452	1	23996.74

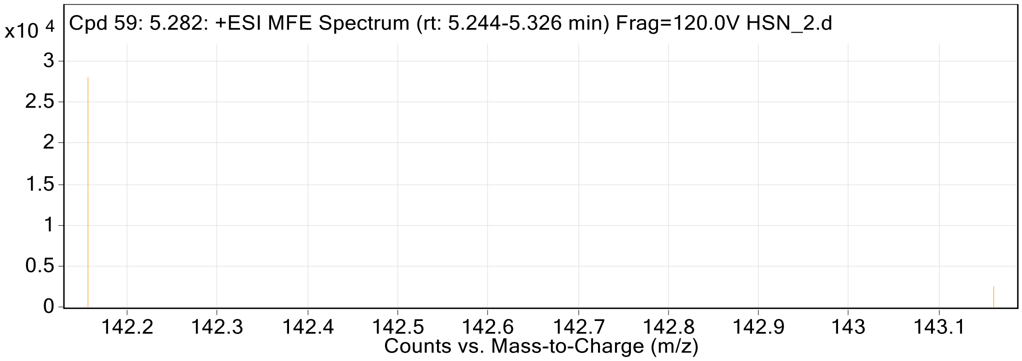

Peak List

m/z	z	Abund	Ion
142.15689	1	28068.31	(M+H)+
143.16068	1	2510.41	(M+H)+

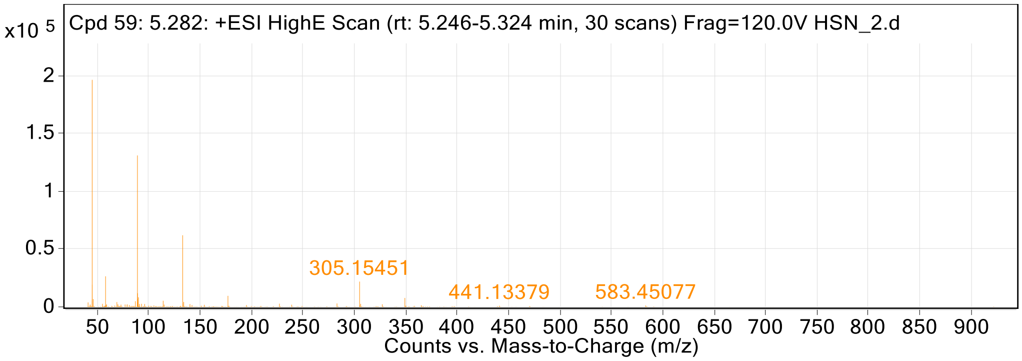

Peak List

m/z	z	Abund
45.03381	1	196331.14
45.0924		19153.71
45.10842		10394.36
58.06458	1	26439.59
89.0593	1	130952.65
89.1418		11796.33
90.06192	1	8290.48
133.08426	1	61914.99
177.10986	1	9552.71
305.15451	1	21863.18

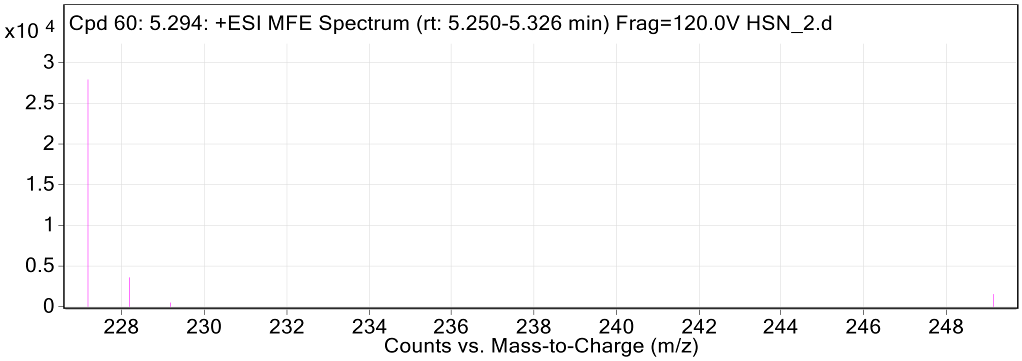

Peak List

m/z	z	Abund	Ion
227.17281	1	27984	(M+H)+
228.17617	1	3617.45	(M+H)+
229.17889	1	520.45	(M+H)+
249.15418	1	1557.5	(M+Na)+

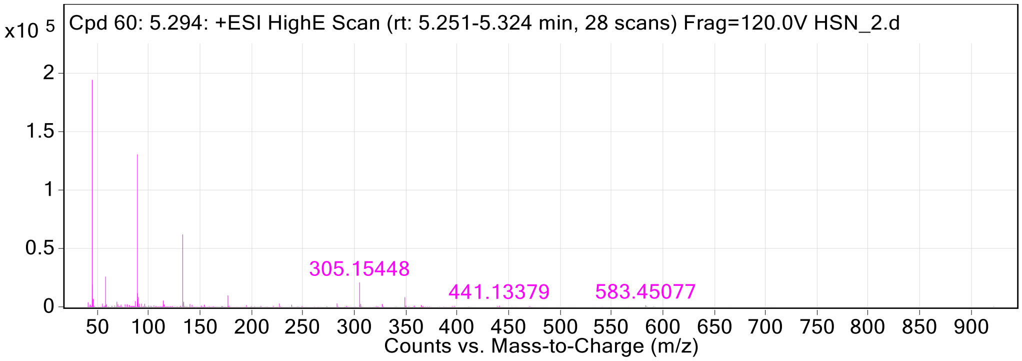

Peak List

m/z	z	Abund
45.03381	1	194886.17

45.0924		19015.64
45.10842		10150.78
58.06457	1	25938.84
89.0593	1	130929.69
89.1418		11812.84
90.06191	1	8318.98
133.08427	1	62149.14
177.10988	1	9820.38
305.15448	1	20945.74

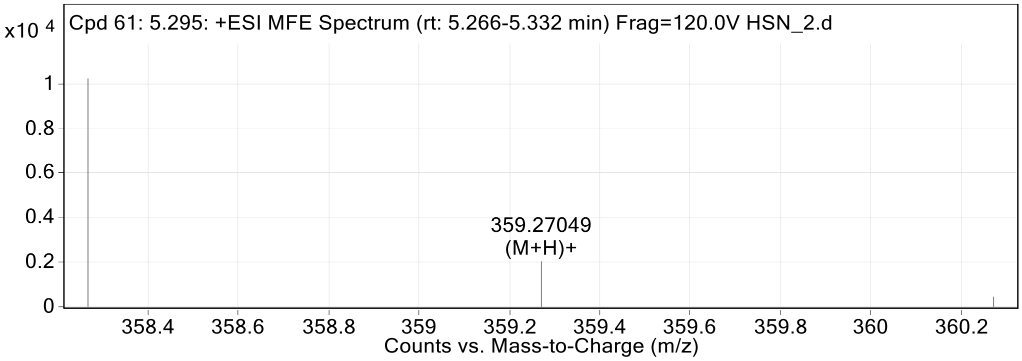

Peak List

m/z	z	Abund	Ion
358.26734	1	10238.84	(M+H)+
359.27049	1	2033.48	(M+H)+
360.27218	1	455.15	(M+H)+

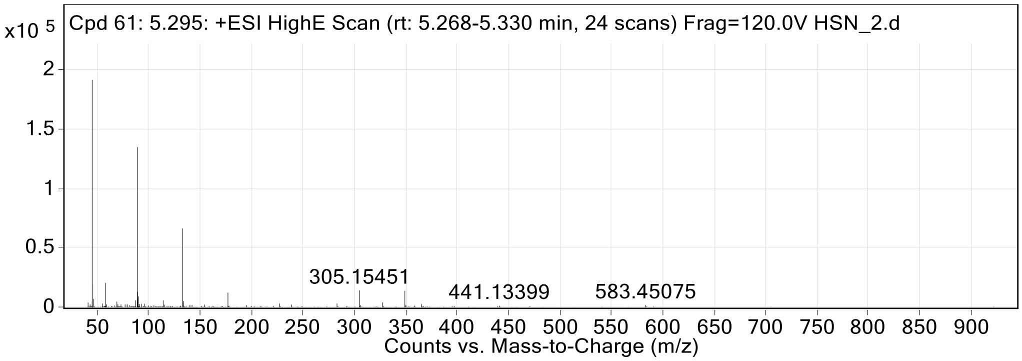

Peak List

m/z	z	Abund
45.03388	1	191565.13
45.09241		19416.38
45.10843		10112.04
58.06458	1	20384.75
89.05942	1	134839.58
89.14181		12775.75
133.08442	1	66166.51
177.10988	1	12049.23
305.15451	1	14012.64
349.18033	1	13679.14

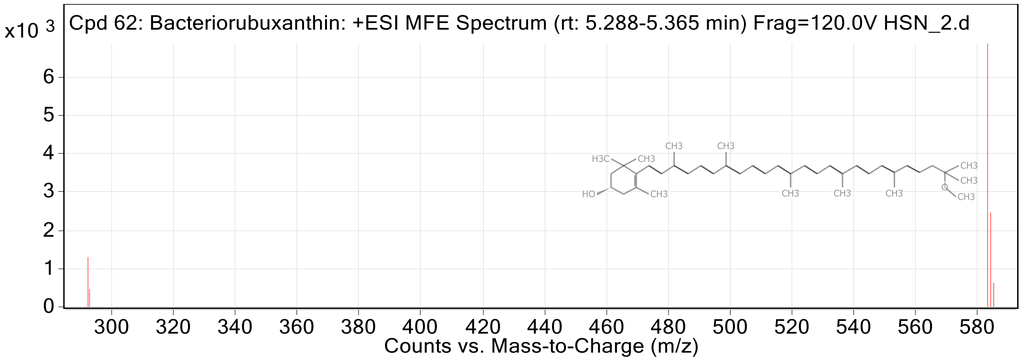

Peak List

m/z	z	Abund	Name	Formula	Ion	Score (DB)	Hits (DB)
292.22938	2	1298.88			(M+2H)+2		
292.72886	2	464.36			(M+2H)+2		
583.44994	1	6887.54	Bacteriorubuxanthin	C41 H58 O2	(M+H)+	89.01	6
584.45253	1	2469.81		C41 H58 O2	(M+H)+		
585.4554	1	629.43		C41 H58 O2	(M+H)+		

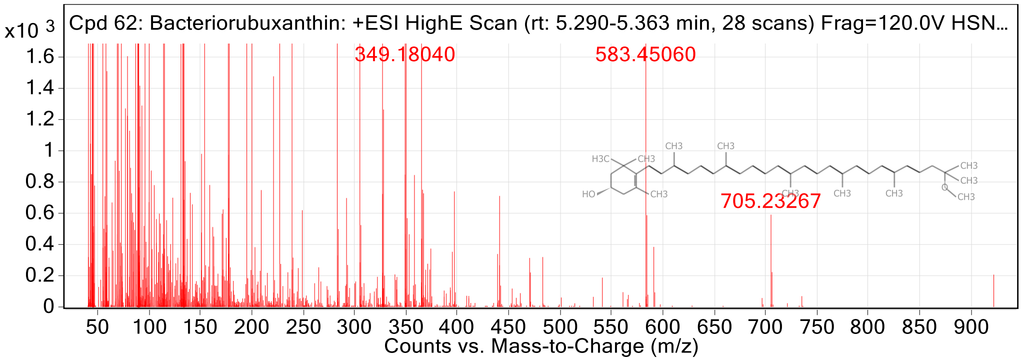

Peak List

m/z	z	Abund
45.03416	1	201511.45
45.0924		24459.59
45.10843		14314.88
89.05981	1	156425.14
89.1418		17631.29
89.16433		9592.31
90.06193	1	12057.4
133.08468	1	89786.12
177.10981	1	19775.2
349.1804	1	27002.68

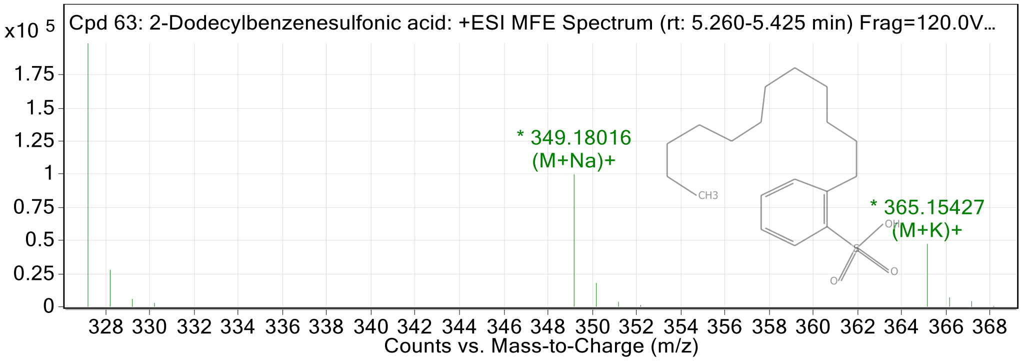

Peak List

m/z	z	Abund	Name	Formula	Ion	Score (DB)	Hits (DB)

327.19855	1	198893.11	2-Dodecylbenzenesulfonic acid	C18 H30 O3 S	(M+H)+	87.6	1
328.20203	1	27952.02		C18 H30 O3 S	(M+H)+		
329.20375	1	5932.65		C18 H30 O3 S	(M+H)+		
330.20434	1	2969.65		C18 H30 O3 S	(M+H)+		
349.18016	1	100142.01			(M+Na)+		
350.18388	1	17958.36			(M+Na)+		
351.18638	1	3799.97			(M+Na)+		
365.15427	1	47556.05			(M+K)+		
366.1577	1	7067.88			(M+K)+		
367.15411	1	4271.78			(M+K)+		

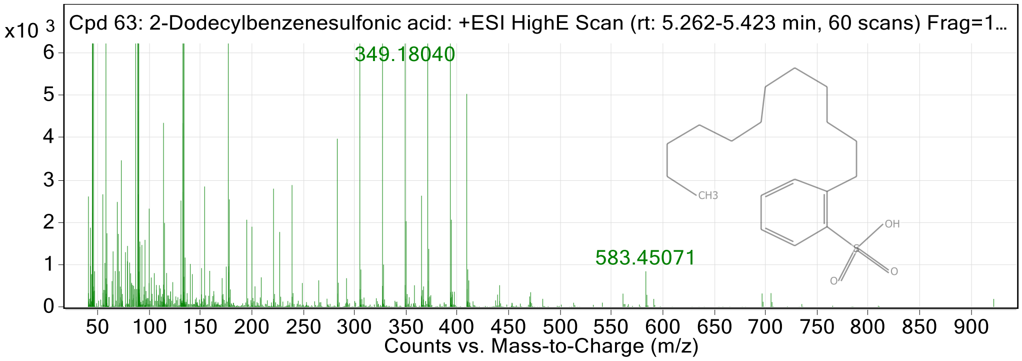

Peak List

m/z	z	Abund
45.03425	1	206200.66
45.09242		26106.88
45.10845		15708.65
89.05996	1	170418.73
89.14181		20393.5
90.06196	1	13946.91
133.08515	1	94446.16
177.11	1	30257.71
349.1804	1	14091
393.2066	1	12352.11

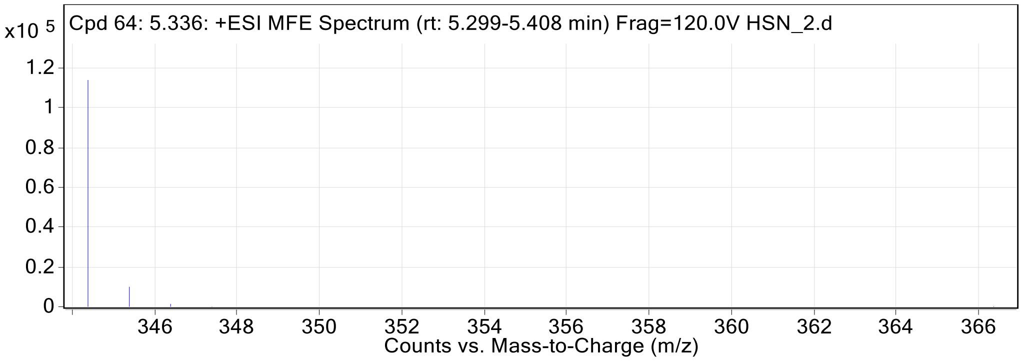

Peak List

m/z	z	Abund	Ion
344.38735	1	113962.38	(M+H)+
345.39101	1	10005.94	(M+H)+
346.39217	1	1439.44	(M+H)+
347.39626	1	158.37	(M+H)+
366.36775	1	254.42	(M+Na)+

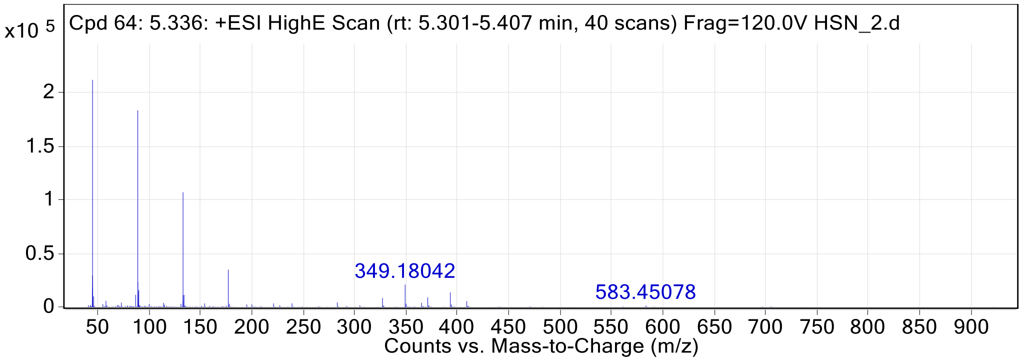

Peak List

m/z	z	Abund
45.03443	1	211977.55
45.09242		29357
45.10844		18264.04
89.06012	1	183581.39
89.14182		23294.65
89.16435		13838.58
90.06198	1	15698.68
133.08522	1	107024.64
177.10999	1	34790.26
349.18042	1	20800.84

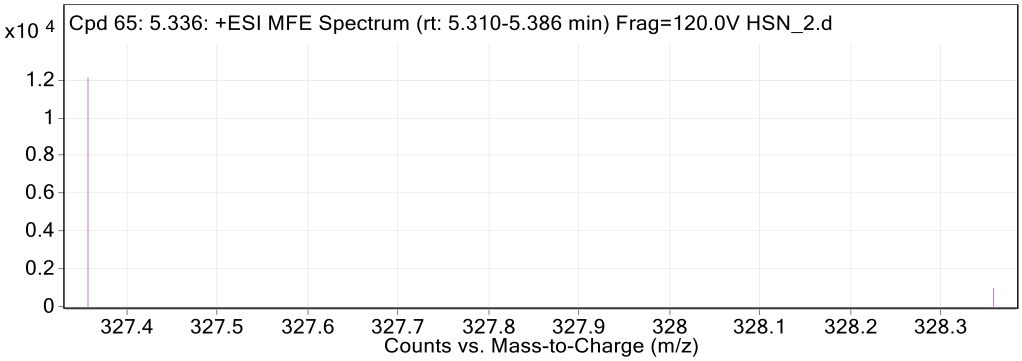

Peak List

m/z	z	Abund	Ion
327.35706	1	12121.58	(M+H)+
328.35852	1	972.79	(M+H)+

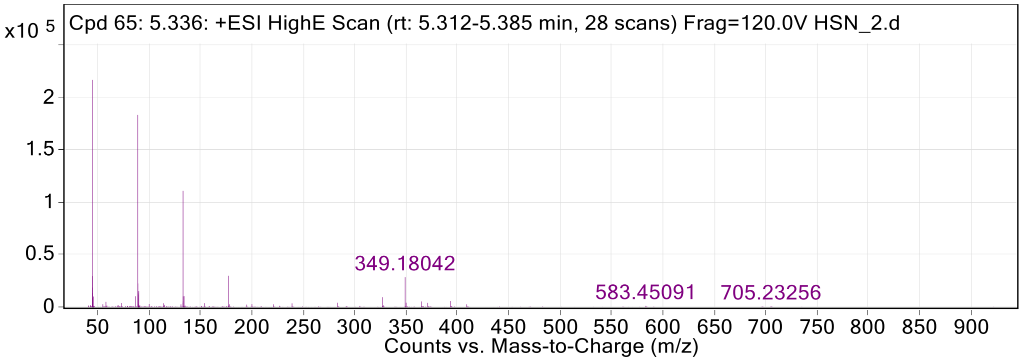

Peak List

m/z	z	Abund
45.03436	1	216429.47
45.07263		13165.35
45.09241		29450.83

45.10844		18489.06
89.06001	1	183066.48
89.14182		22362.63
90.06197	1	15109.99
133.08491	1	110836.58
177.10991	1	29750.04
349.18042	1	28426.14

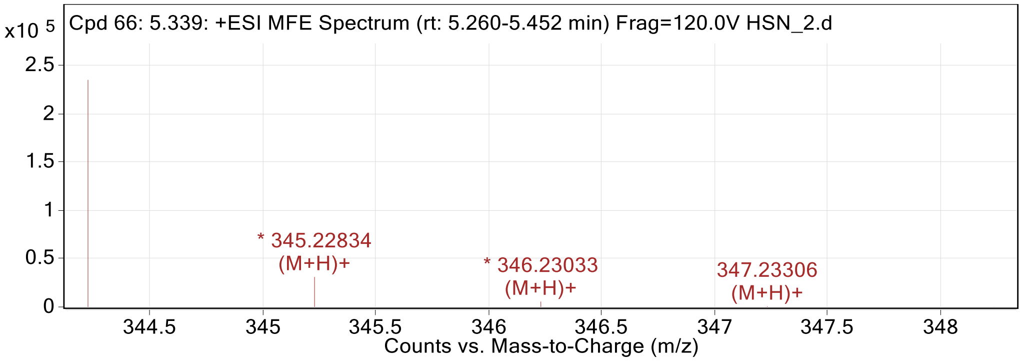

Peak List

m/z	z	Abund	Ion
344.22519	1	235120.27	(M+H)+
345.22834	1	31075.7	(M+H)+
346.23033	1	5570.68	(M+H)+
347.23306	1	575.15	(M+H)+
348.23583	1	112.4	(M+H)+

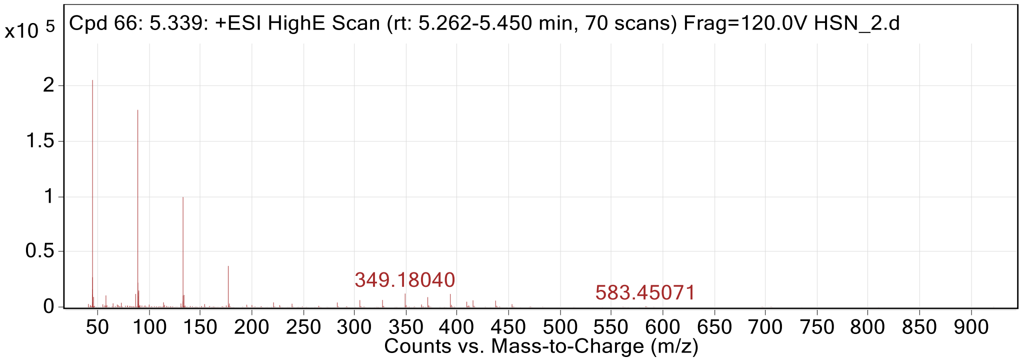

Peak List

m/z	z	Abund
45.03433	1	205607.09
45.09243		26936.02
45.10846		16089.28
89.06004	1	178559.28
89.14182		22078.52
89.16437		13223.36
90.06198	1	14948.13
133.08541	1	99566.95
177.1101	1	37208.34
349.1804	1	12182.58

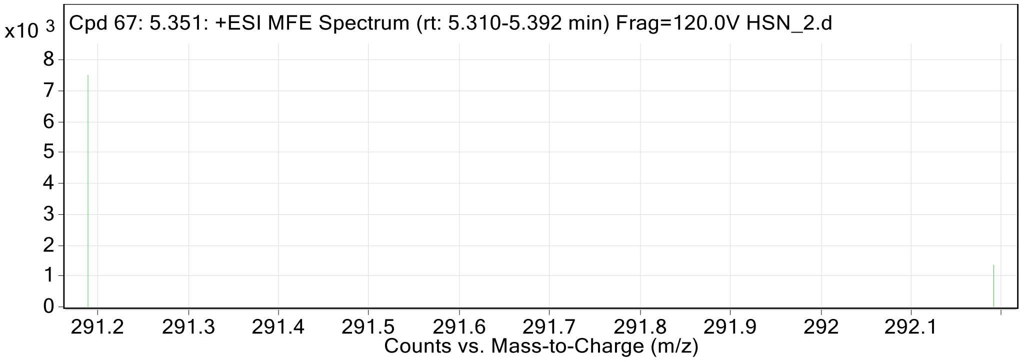

Peak List

m/z	z	Abund	Ion
291.18911	1	7523.22	(M+H)+
292.19149	1	1364.17	(M+H)+

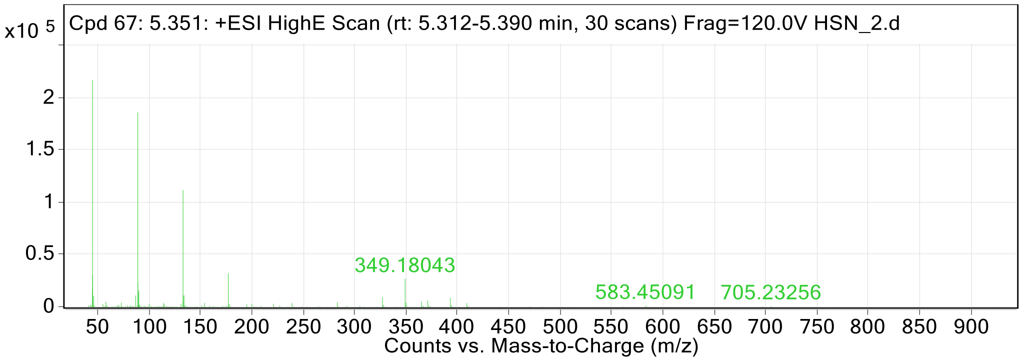

Peak List

m/z	z	Abund
45.03441	1	216757.55
45.07265		13553.27
45.09242		30093.69
45.10844		18947.84
89.06007	1	185636.17
89.14182		23224.15
90.06197	1	15621.06
133.08502	1	111652.74
177.10994	1	32141.32

m/z	z	Abund
349.18043	1	26653.42

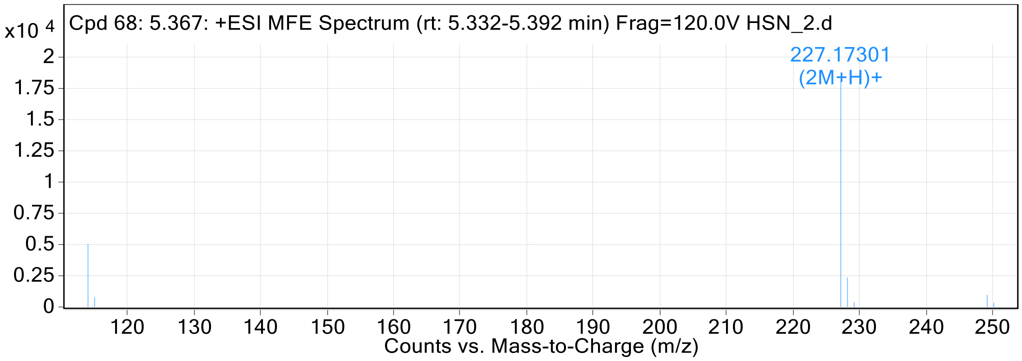

Peak List

m/z	z	Abund	Ion
114.08958	1	5029.28	(M+H)+
115.09309	1	763.93	(M+H)+
227.17301	1	18176.13	(2M+H)+
228.17662	1	2365.6	(2M+H)+
229.18082	1	364.46	(2M+H)+
249.15473	1	972.77	(2M+Na)+
250.14741	1	352.6	(2M+Na)+

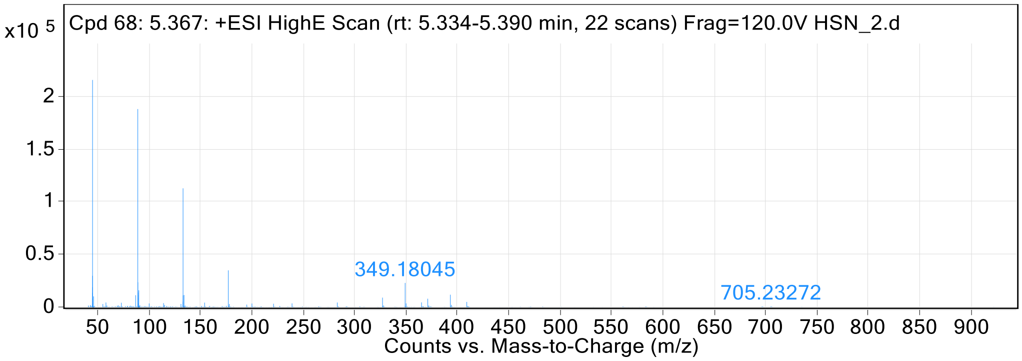

Peak List

m/z	z	Abund
45.0344	1	215402.31
45.09242		29575.32
45.10845		18713.52
89.06008	1	187778.98
89.14182		23427.41
89.16435		13759.94
90.06196	1	15851.95
133.08511	1	112569.66
177.10994	1	34765.57
349.18045	1	22816.28

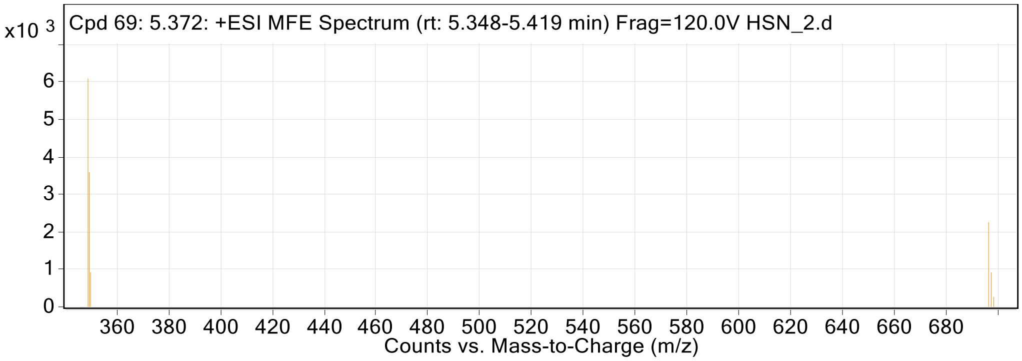

Peak List

m/z	z	Abund	Ion
348.77008	2	6093.48	(M+2H)+2
349.2714	2	3596.35	(M+2H)+2
349.77248	2	923.79	(M+2H)+2
696.53394	1	2261.02	(M+H)+
697.5377	1	920.36	(M+H)+
698.5383	1	263.54	(M+H)+

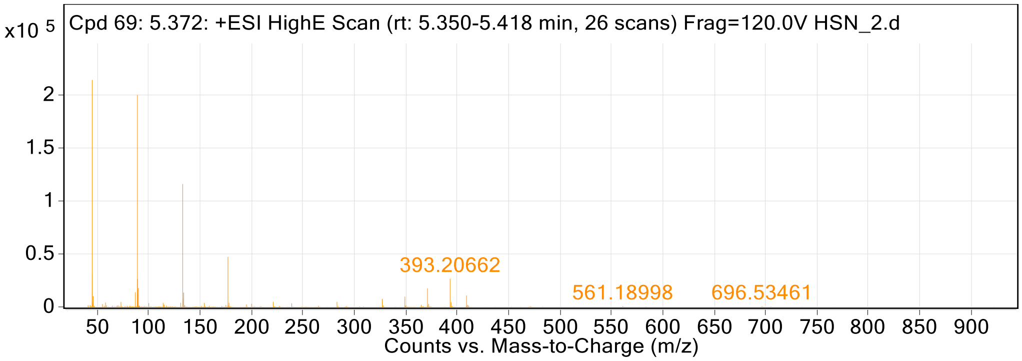

Peak List

m/z	z	Abund
45.03451	1	214146.64
45.09243		30592.55
45.10847		19536.75
89.06025	1	199866.89
89.14182		26359.83
90.06196	1	17944.95
133.08556	1	115885.66
177.11006	1	47149.57
371.22484	1	17546.51
393.20662	1	26613.4

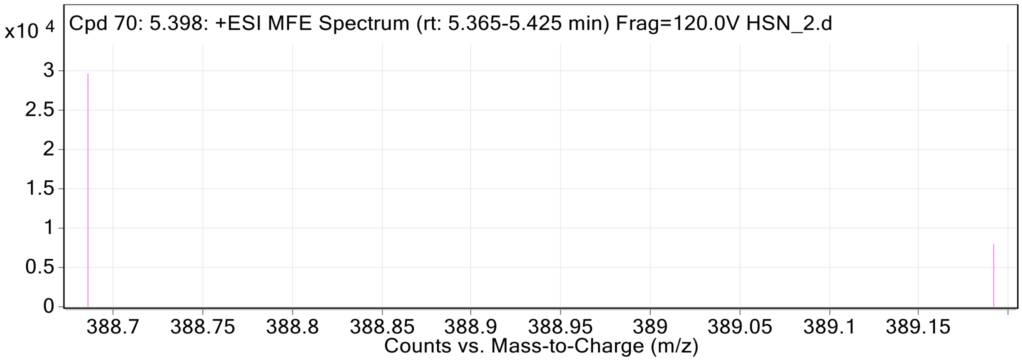

Peak List

m/z	z	Abund	Ion
388.68588	2	29710.65	(M+2H)+2
389.19195	2	7993.71	(M+2H)+2

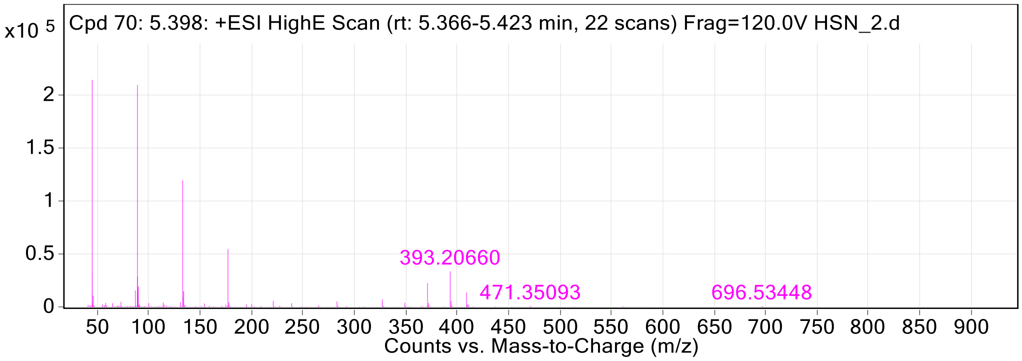

Peak List

m/z	z	Abund
45.03463	1	213753.94
45.09244		31874.54
45.10847		19985.11
89.06033	1	208886.89
89.14182	1	28452.01
90.06199	1	19220.22
133.08581	1	119109.08
177.1101	1	54397.58
371.22483	1	22395.25
393.2066	1	33550.4

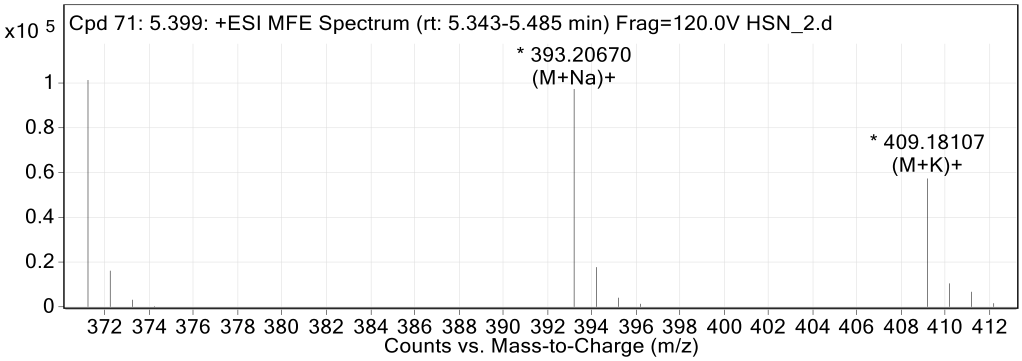

Peak List

m/z	z	Abund	Ion
371.22475	1	101461.37	(M+H)+
372.22848	1	16154.41	(M+H)+
373.23082	1	3158.77	(M+H)+
393.2067	1	97421	(M+Na)+
394.21011	1	17746.97	(M+Na)+
395.21256	1	4070.84	(M+Na)+
409.18107	1	57401.43	(M+K)+
410.18397	1	10485.85	(M+K)+
411.17982	1	6718.93	(M+K)+
412.18377	1	1603.18	(M+K)+

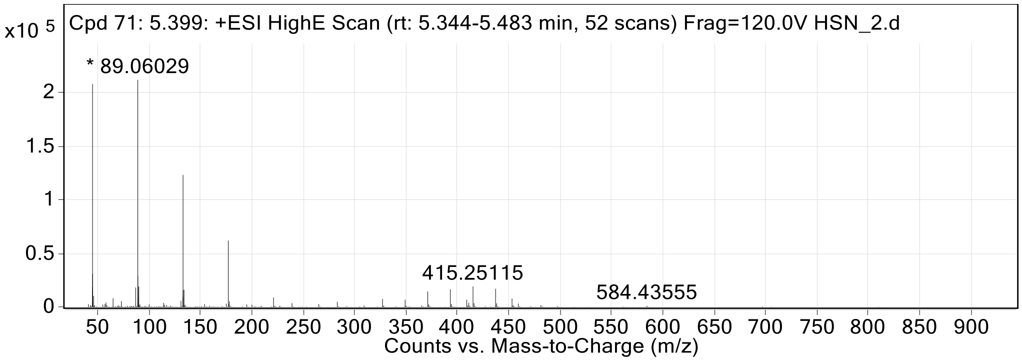

Peak List

m/z	z	Abund
45.0346	1	207741
45.09244		30707.26
45.10848		18553.61
89.06029	1	211637.27
89.14185		28853.15
89.1644		18131.45
90.06199	1	19054.39
133.086	1	123004.58
177.11021	1	61860.05
415.25115	1	19054.21

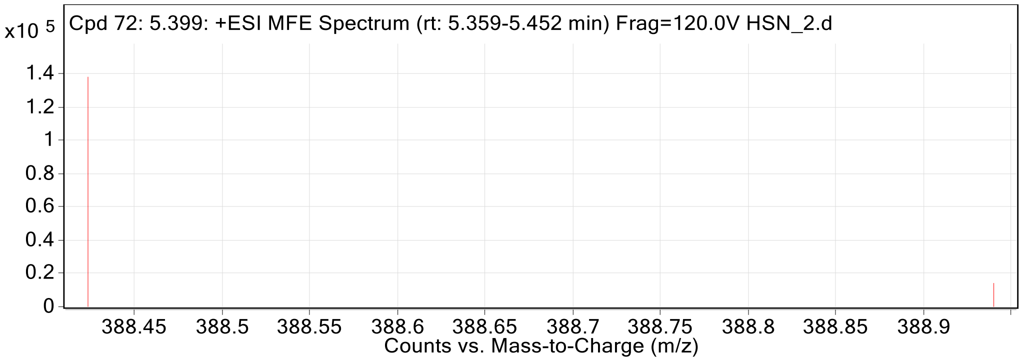

Peak List

m/z	z	Abund	Ion
388.42341	1	138010.03	(M+H)+
388.94003	1	14240.73	(M+H)+

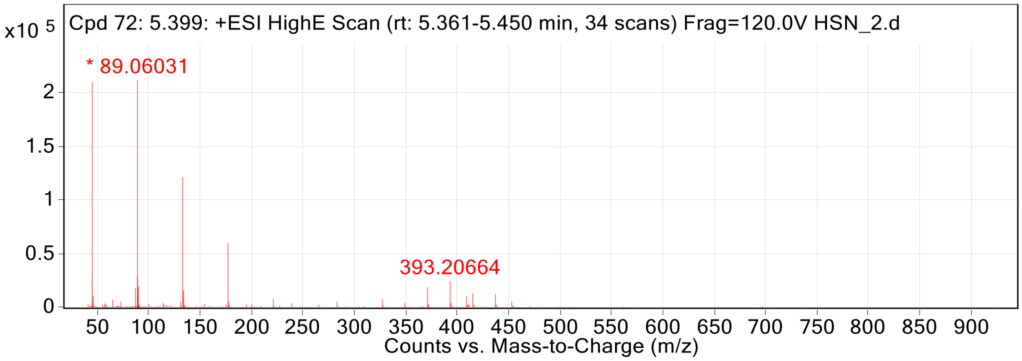

Peak List

m/z	z	Abund
45.03463	1	209707.86
45.09244		31217.94
45.10848		19113.55

89.06031	1	211609.75
89.14184		28805.98
90.06201	1	19260.13
133.08596	1	121195.45
177.11018	1	59529.53
371.2249	1	18370.97
393.20664	1	24270.61

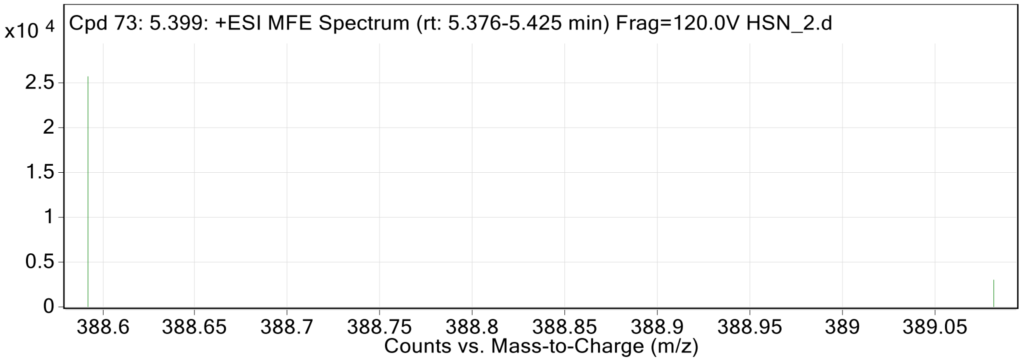

Peak List

m/z	z	Abund	Ion
388.59212	2	25752.74	(M+2H)+2
389.0817	2	3035.81	(M+2H)+2

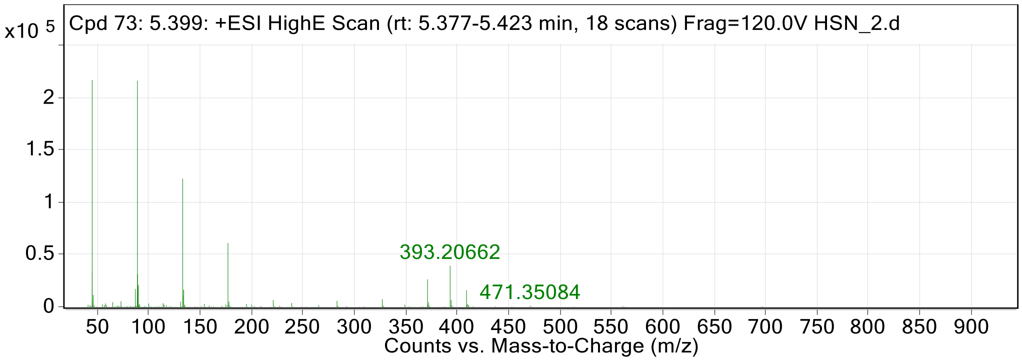

Peak List

m/z	z	Abund
45.03475	1	216858.03
45.09244		34092.18
45.10848		22039.56
89.06049	1	216179.09
89.14182	1	31117.14
90.06199	1	20939.88
133.08605	1	122506.27
177.11011	1	60987.56
371.22482	1	26219.02
393.20662	1	39306.94

Qualitative Analysis Report

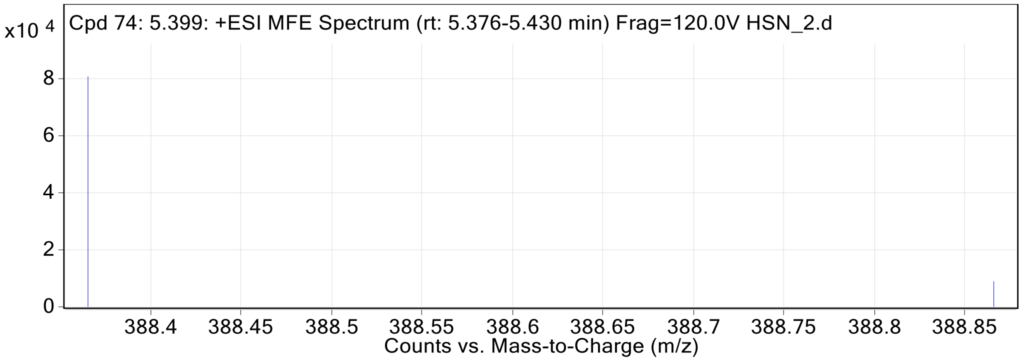

Peak List

m/z	z	Abund	Ion
388.36557	2	80987.06	(M+2H)+2
388.86603	2	9015.32	(M+2H)+2

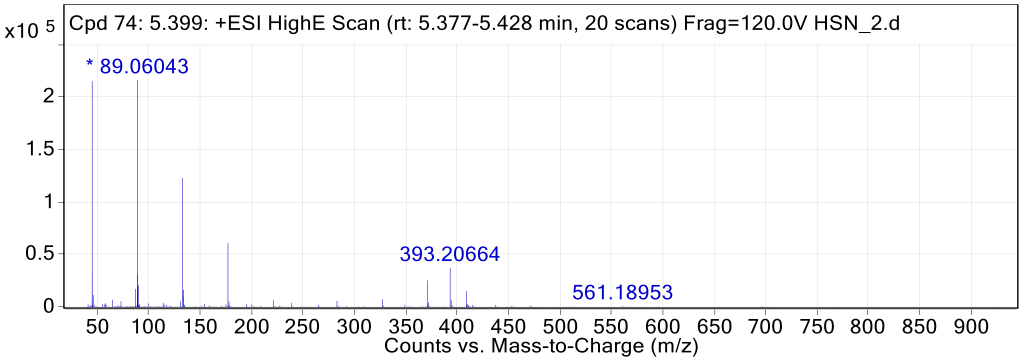

Peak List

m/z	z	Abund
45.03471	1	215133.47
45.09244		33300.68
45.10847		21234.24
89.06043	1	216170.25
89.14182	1	30503.92
90.06199	1	20555.77
133.086	1	122620.17
177.11011	1	60705.74
371.22479	1	25140.88
393.20664	1	37145.16

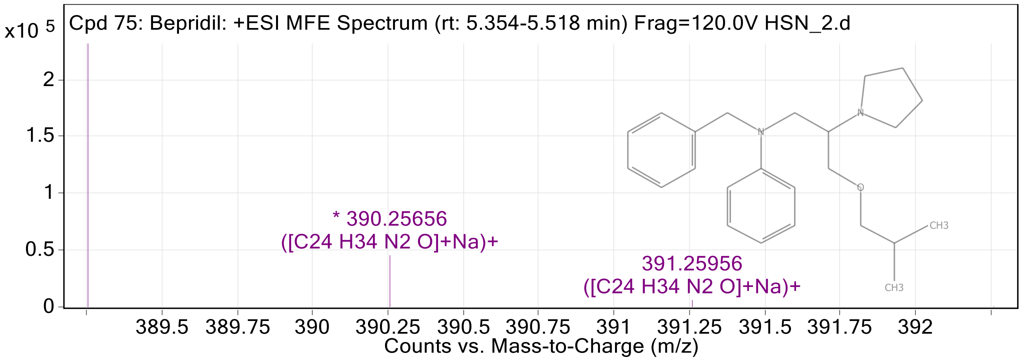

Peak List

m/z	z	Abund	Name	Formula	Ion	Score (DB)	Hits (DB)
389.25497	1	231706.36	Bepridil	C24 H34 N2 O	(M+Na)+	80.6	1
390.25656	1	45483.69		C24 H34 N2 O	(M+Na)+		
391.25956	1	6118.08		C24 H34 N2 O	(M+Na)+		

392.25953	1	481.16		C24 H34 N2 O	(M+Na)+		

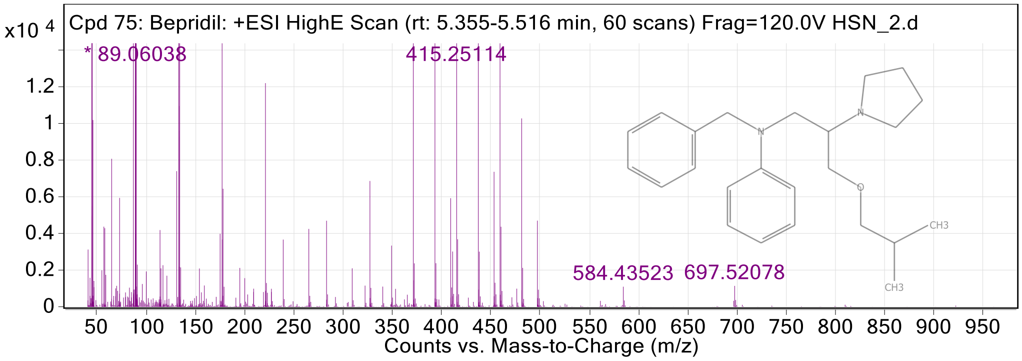

Peak List

m/z	z	Abund
45.03469	1	202358.2
45.09245		30770.06
87.04298	1	21719.36
89.06038	1	218412.91
89.14186		30667.02
90.062	1	20223.04
133.08626	1	128498.4
177.11032	1	72398.5
415.25114	1	20170.05
459.2775	1	19762.63

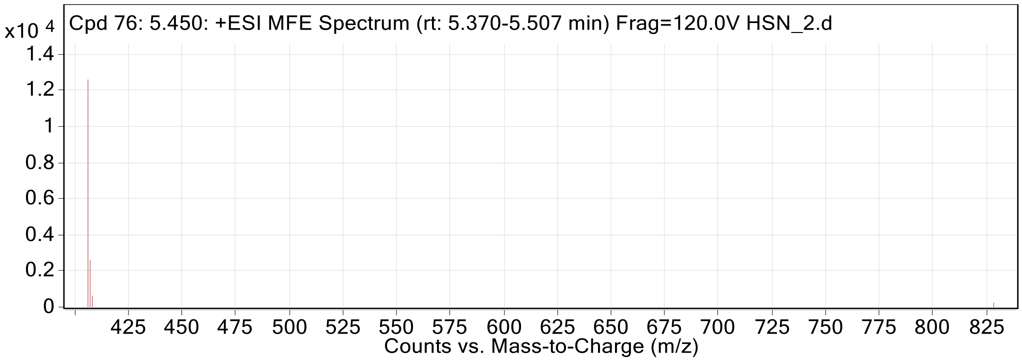

Peak List

m/z	z	Abund	Ion
406.20484	1	12597.51	(M+H)+
407.20772	1	2606.67	(M+H)+
408.21162	1	630.97	(M+H)+
828.43325	1	244.62	(2M+NH4)+

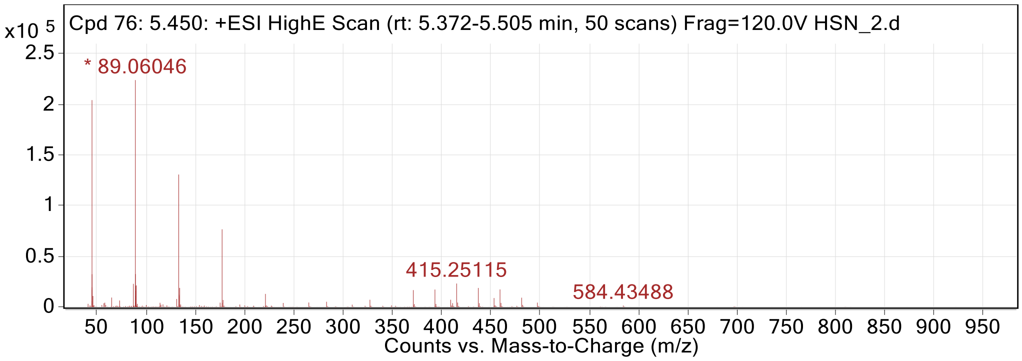

Peak List

m/z	z	Abund

45.03478	1	203913.72
45.09245		32139.47
87.04297	1	22649.73
89.06046	1	223647.56
89.14186		32272.55
89.16441		20631.38
90.06201	1	21094.02
133.08641	1	130490.8
177.11031	1	76449.85
415.25115	1	22971.51

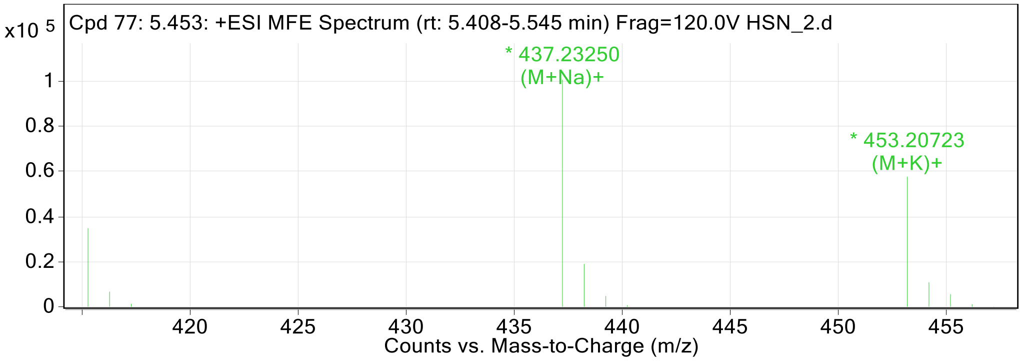

Peak List

m/z	z	Abund	Ion
415.25149	1	34894.08	(M+H)+
416.25406	1	6695.24	(M+H)+
417.25668	1	1340.98	(M+H)+
437.2325	1	100784.89	(M+Na)+
438.23645	1	19012.29	(M+Na)+
439.23982	1	4775.62	(M+Na)+
453.20723	1	57714.89	(M+K)+
454.20983	1	10864.96	(M+K)+
455.20749	1	5611.67	(M+K)+
456.20857	1	1140.56	(M+K)+

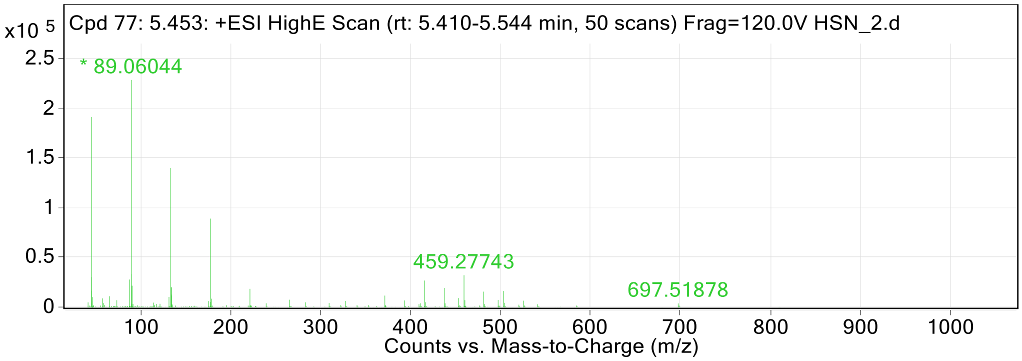

Peak List

m/z	z	Abund
45.0348	1	191449.7
45.09246		30194.63
87.04301	1	27719.16
89.06044	1	228872.09
89.14188		33149.8
90.06202	1	21517.78
133.08649	1	140047.7
177.11047	1	89167.55
415.25113	1	26698.03

459.27743	1	32044.36

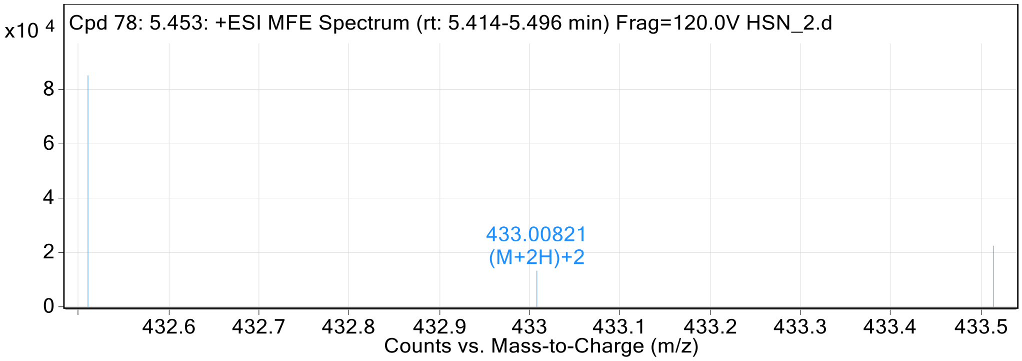

Peak List

m/z	z	Abund	Ion
432.51085	2	84995.09	(M+2H)+2
433.00821	2	13246.01	(M+2H)+2
433.51442	2	22416.94	(M+2H)+2

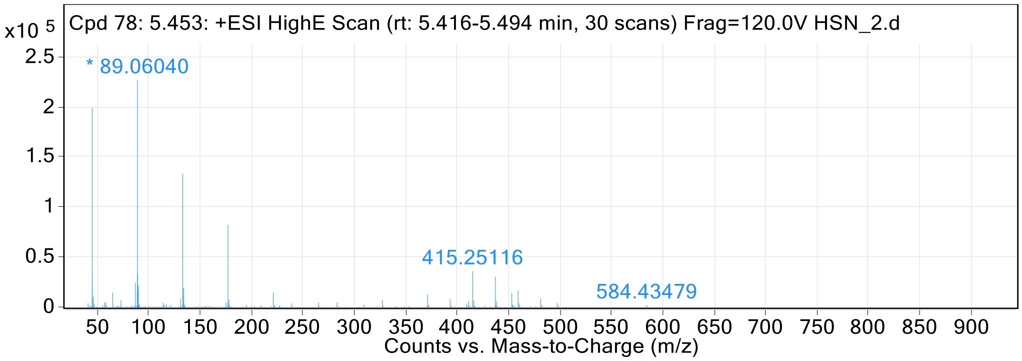

Peak List

m/z	z	Abund
45.03475	1	198954.67
45.09246		30947.06
87.043	1	24273.76
89.0604	1	226910.58
89.14188		32382.04
90.06202	1	21086.78
133.08652	1	132971.05
177.11033	1	82184.97
415.25116	1	35917.69
437.23296	1	30255.27

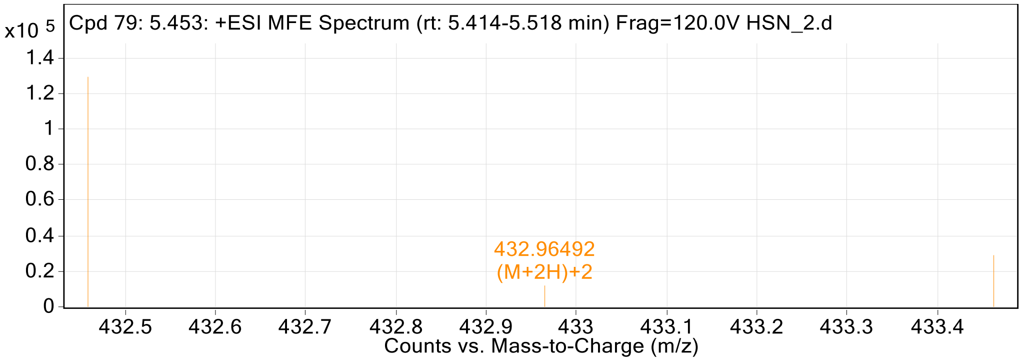

Peak List

m/z	z	Abund	Ion

432.45881	2	129262.71	(M+2H)+2
432.96492	2	11968.68	(M+2H)+2
433.46238	2	29034.87	(M+2H)+2

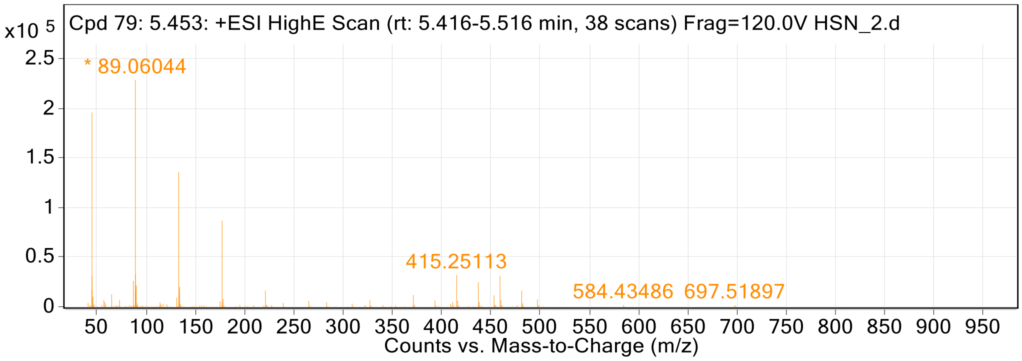

Peak List

m/z	z	Abund
45.03478	1	195702.67
45.09246		30727.18
87.043	1	26033.84
89.06044	1	228315.05
89.14188		33002.9
133.08657	1	135932.41
177.1104	1	86495.02
415.25113	1	31837.31
437.23296	1	24793.54
459.2775	1	31198.59

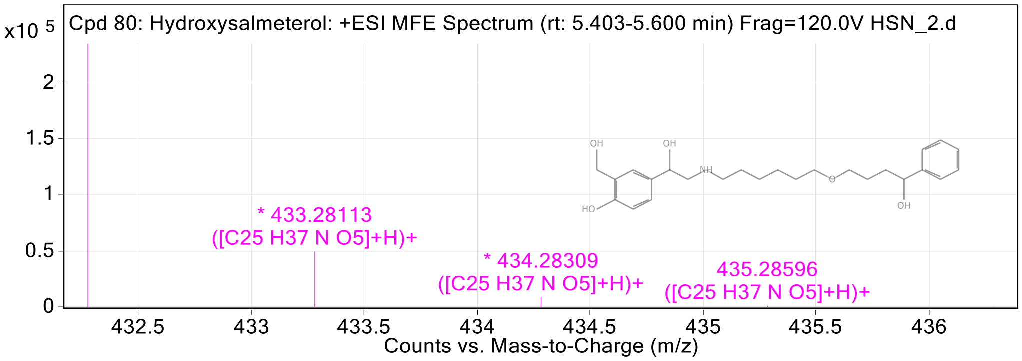

Peak List

m/z	z	Abund	Name	Formula	Ion	Score (DB)	Hits (DB)
432.27635	1	235124.5	Hydroxysalmeterol	C25 H37 N O5	(M+H)+	80.17	2
433.28113	1	49560.19		C25 H37 N O5	(M+H)+		
434.28309	1	8687.82		C25 H37 N O5	(M+H)+		
435.28596	1	999.31		C25 H37 N O5	(M+H)+		
436.28704	1	95.64		C25 H37 N O5	(M+H)+		

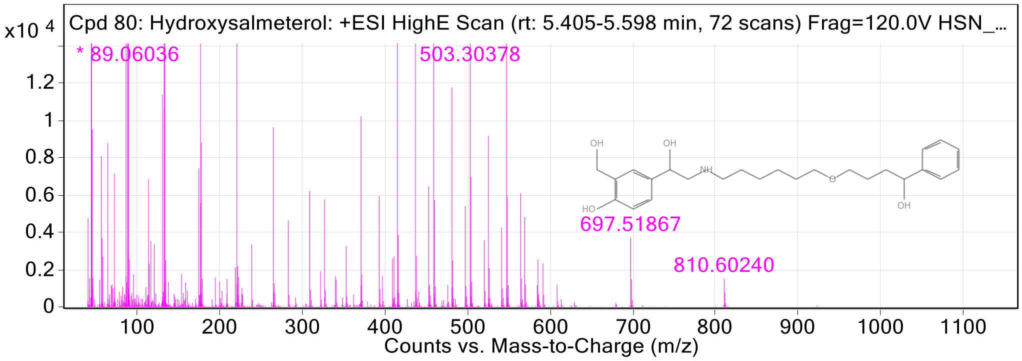

Peak List

m/z	z	Abund
45.03477	1	181829.97
45.09246		28350.92
87.04302	1	30787.77
89.06036	1	228935.94
89.14188		32370
133.08619	1	148612.13
177.11048	1	92464.17
221.1362	1	21249.58
459.27739	1	26625.73
503.30378	1	27258.59

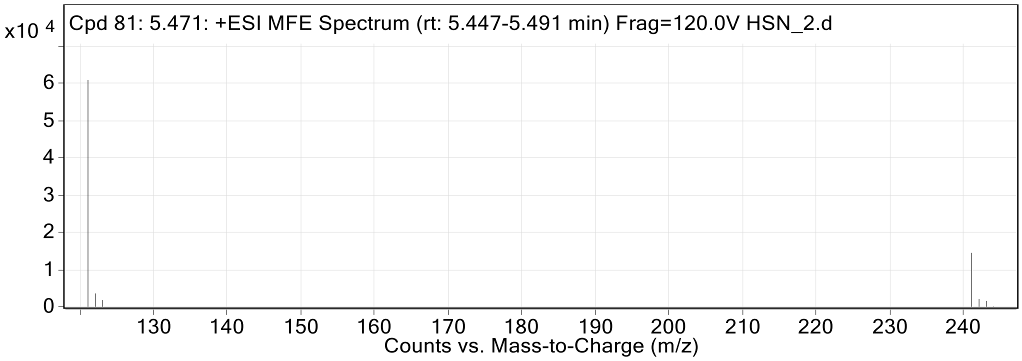

Peak List

m/z	z	Abund	Ion
121.06657	1	60959.64	(M+H)+
122.06925	1	3588.69	(M+H)+
123.06241	1	1805.36	(M+H)+
241.12697	1	14496.74	(2M+H)+
242.12935	1	2083.83	(2M+H)+
243.12319	1	1572.57	(2M+H)+
244.12874	1	98.77	(2M+H)+

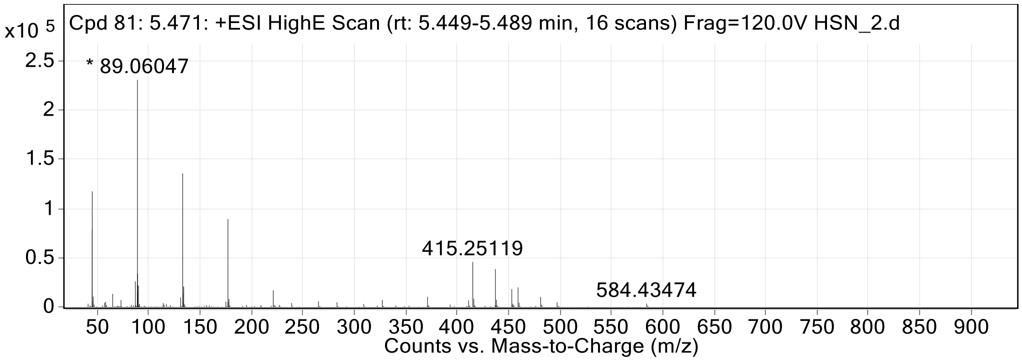

Peak List

m/z	z	Abund
45.0334		79326.16
45.03577		117495.87
45.09246		31333.27
87.04301	1	26001.61
89.06047	1	230915.59
89.14189		33941.36
133.08674	1	135708.98
177.11036	1	89337.3
415.25119	1	45633.05
437.233	1	38356.02

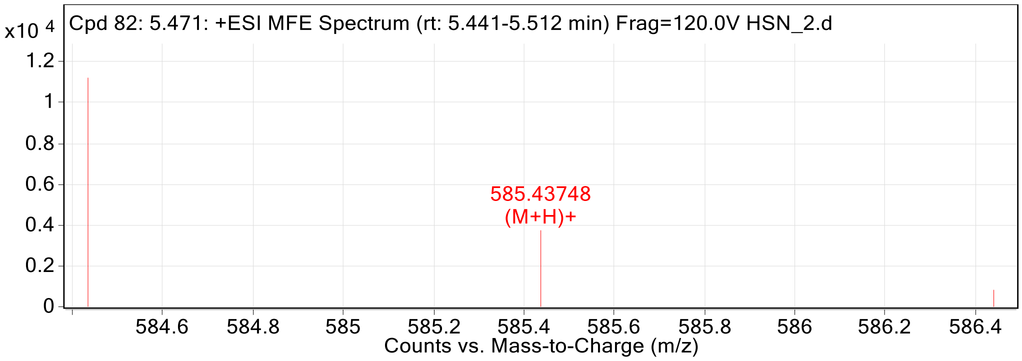

Peak List

m/z	z	Abund	Ion
584.43451	1	11217.39	(M+H)+
585.43748	1	3746.48	(M+H)+
586.4408	1	829.71	(M+H)+

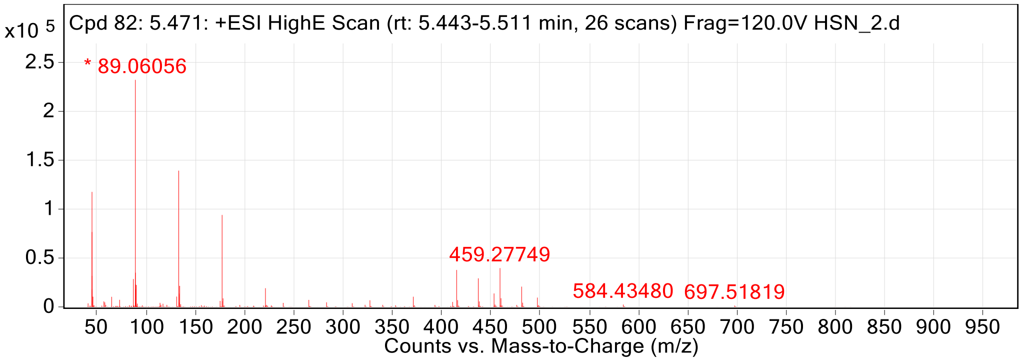

Peak List

m/z	z	Abund
45.03341		76513.66
45.03587		117502.58

45.09246		31673.96
89.06056	1	231969.5
89.14189		34973.75
133.08682	1	139218.23
177.11045	1	93939.03

m/z	z	Abund
415.25115	1	37750.52
437.23302	1	29186.85
459.27749	1	39630.68

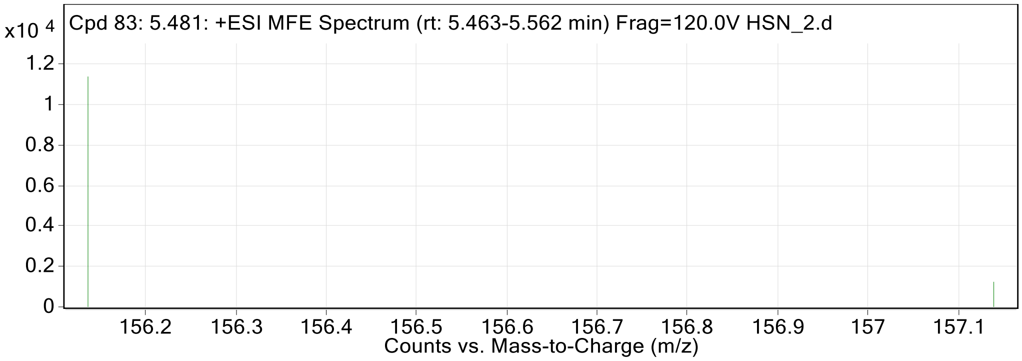

Peak List

m/z	z	Abund	Ion
156.13623	1	11375.62	(M+H)+
157.13936	1	1238.94	(M+H)+

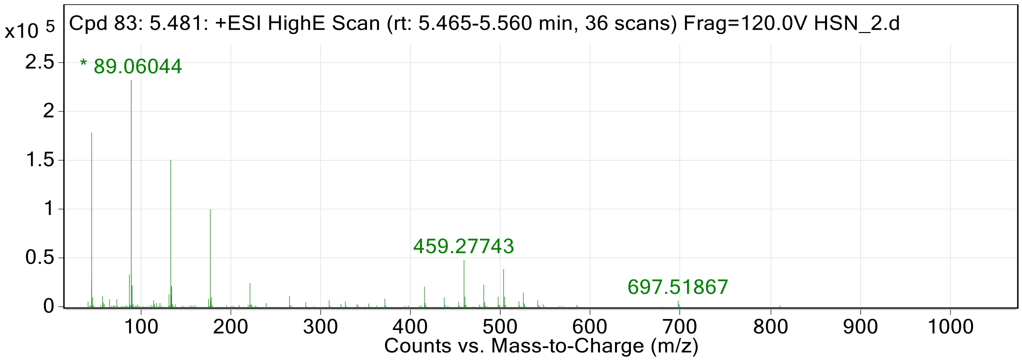

Peak List

m/z	z	Abund
45.03485	1	178810.77
45.09247		28545.91
87.04305	1	33040.9
89.06044	1	232717.23
89.1419		33900.52
133.08644	1	150925
177.11059	1	100084.72
221.13622	1	24278.03
459.27743	1	48069.55
503.30403	1	38695.12

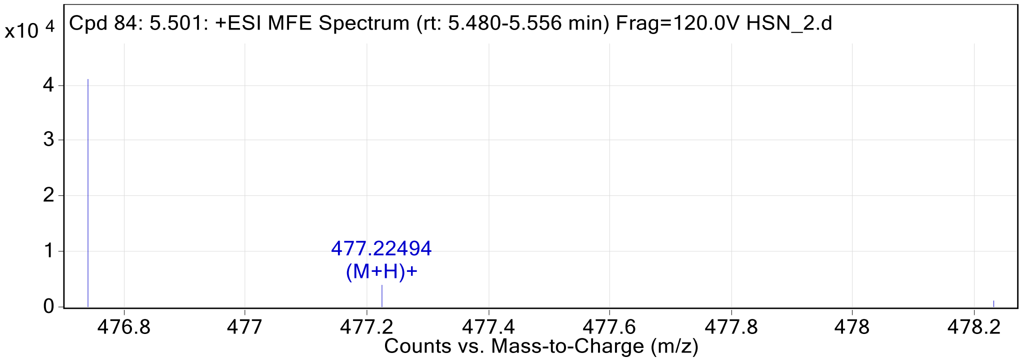

Qualitative Analysis Report

Peak List

m/z	z	Abund	Ion
476.74102	1	41062.31	(M+H)+
477.22494	1	3976.64	(M+H)+
478.23203	1	1154.64	(M+H)+

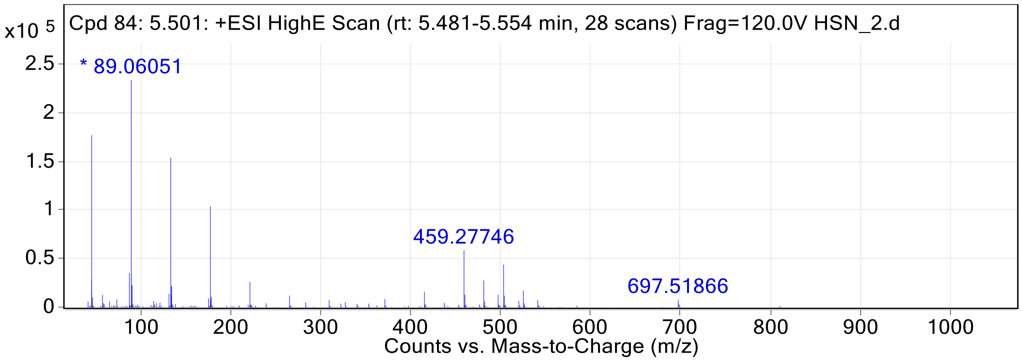

Peak List

m/z	z	Abund
45.03491		176859.89
45.09247		28752.76
87.04304	1	34942.03
89.06051	1	233553.78
89.14189		34766.16
133.08649	1	153678.09
177.11063	1	103567.31
459.27746	1	58391.16
481.25887	1	27323.36
503.30405	1	43606.24

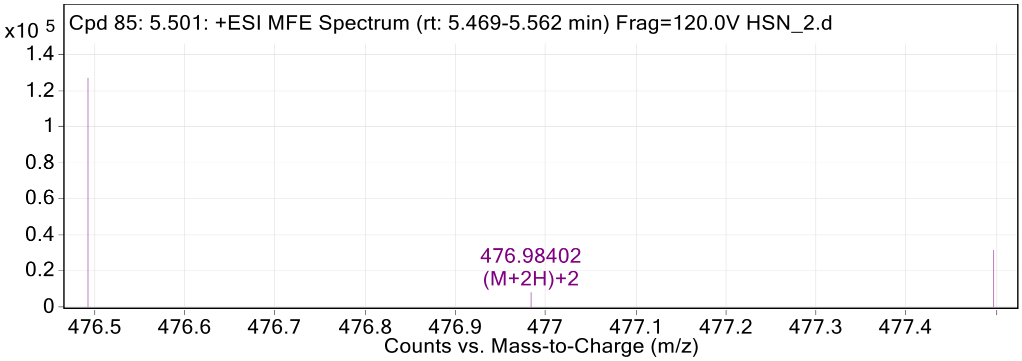

Peak List

m/z	z	Abund	Ion
476.49282	2	127056.3	(M+2H)+2
476.98402	2	8135.47	(M+2H)+2
477.49698	2	31579.25	(M+2H)+2

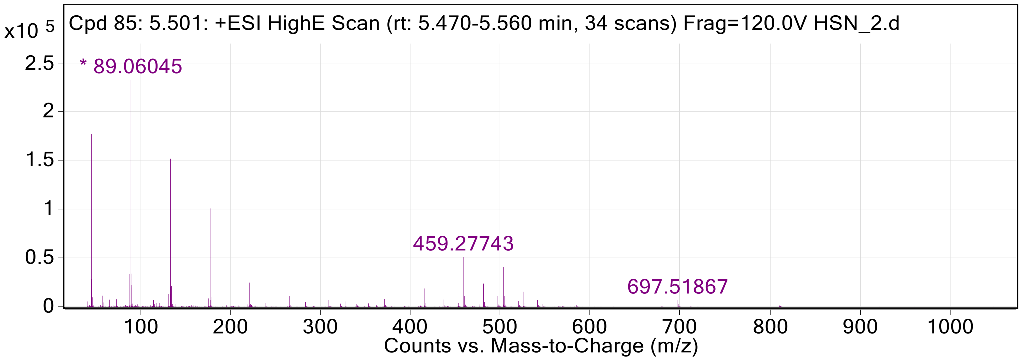

Peak List

m/z	z	Abund
45.03485		177553.22
45.09247		28392.2
87.04304	1	33667.77
89.06045	1	232892.72
89.1419		33964.78
133.08643	1	152032.34
177.1106	1	100966.48
221.13623	1	24755.63
459.27743	1	50848.78
503.30403	1	40971.3

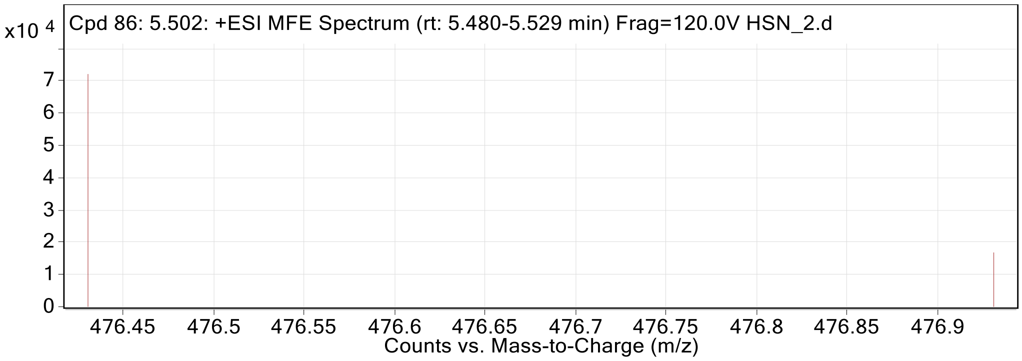

Peak List

m/z	z	Abund	Ion
476.43056	2	72133.1	(M+2H)+2
476.93117	2	16831.65	(M+2H)+2

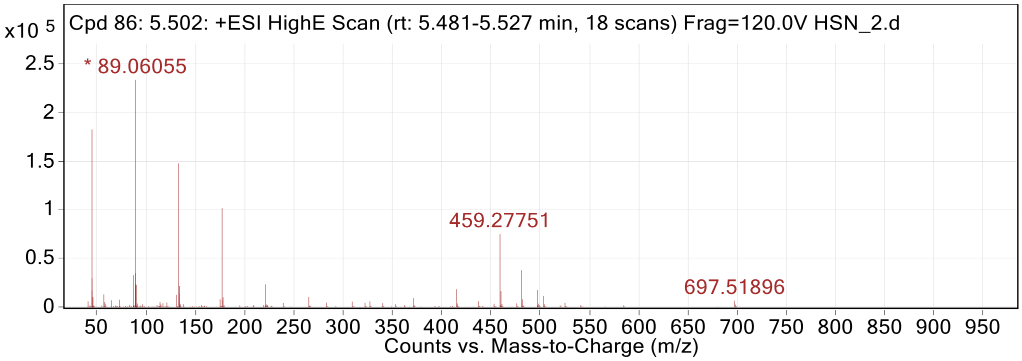

Peak List

m/z	z	Abund
45.03491		182601.39
45.09247		29750.18
87.04301		32867.8

89.06055	1	233570.5
89.14189		35033.77
133.08668	1	147671.86
177.11058	1	101420.8
221.13627	1	23152.84
459.27751	1	75047.95
481.25884	1	37761.33

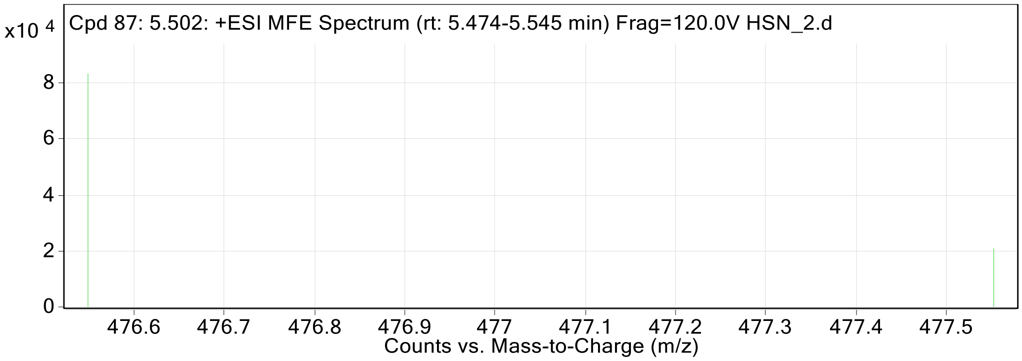

Peak List

m/z	z	Abund	Ion
476.54914	1	83490.35	(M+H)+
477.55267	1	20876.59	(M+H)+

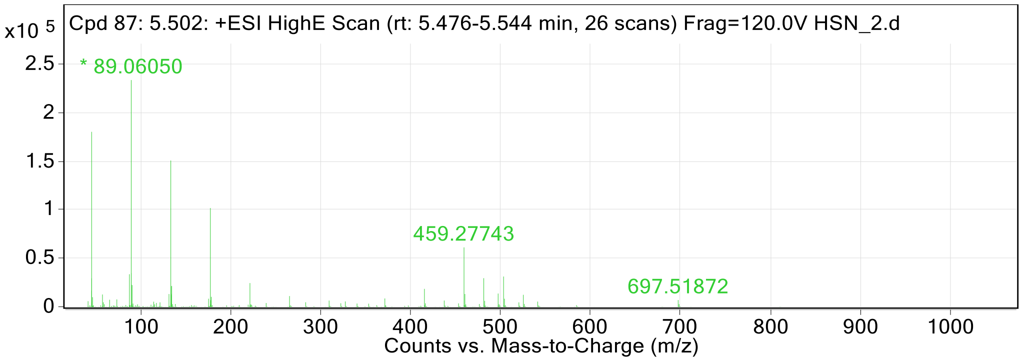

Peak List

m/z	z	Abund
45.0349		180058.64
45.09247		29308.18
87.04303	1	33520.9
89.0605	1	233361.58
89.1419		34686.18
133.08655	1	150537.92
177.11062	1	101633.58
459.27743	1	61151.14
481.25885	1	29430.33
503.30405	1	31126.89

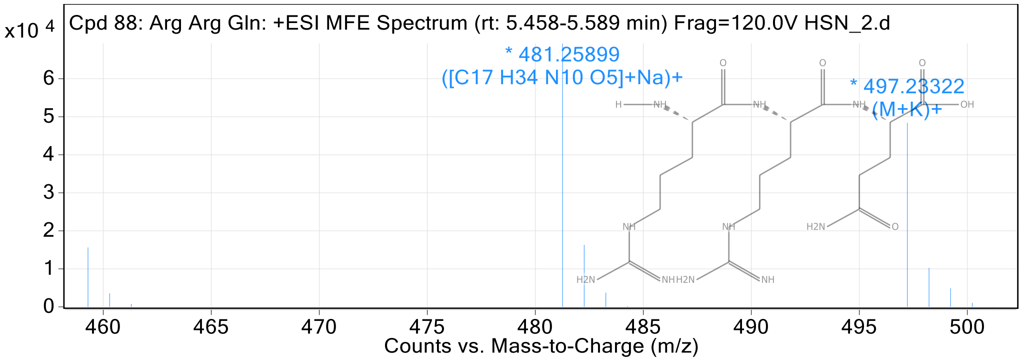

Peak List

m/z	z	Abund	Name	Formula	Ion	Score (DB)	Hits (DB)
459.27744	1	15659.62			(M+H)+		
460.2801	1	3572.86			(M+H)+		
461.28345	1	767.58			(M+H)+		
481.25899	1	69479.75	Arg Arg Gln	C17 H34 N10 O5	(M+Na)+	93.6	3
482.2627	1	16353.43		C17 H34 N10 O5	(M+Na)+		
483.2647	1	3749.46		C17 H34 N10 O5	(M+Na)+		
497.23322	1	48533.23			(M+K)+		
498.23655	1	10292.88			(M+K)+		
499.2347	1	4905.04			(M+K)+		
500.23766	1	1061.26			(M+K)+		

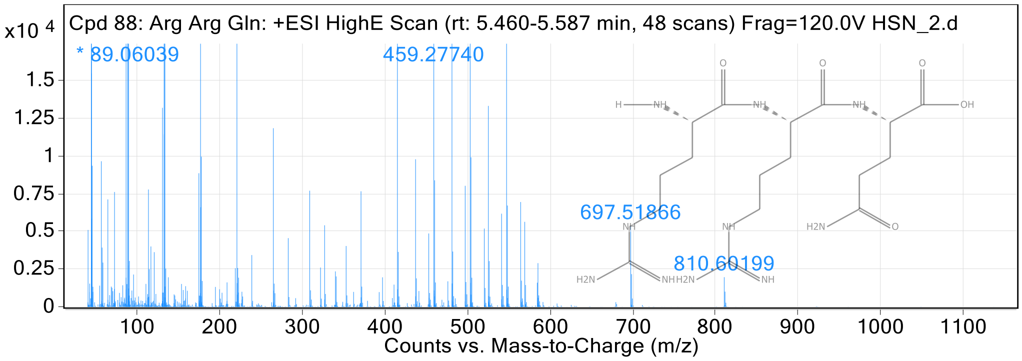

Peak List

m/z	z	Abund
45.03481	1	174402.63
45.09247		27574.48
87.04304	1	34297.24
89.06039	1	232118.11
89.14189		33260.76
133.08624	1	155277.89
177.11055	1	100578.1
221.1362	1	25275.55
459.2774	1	39110.46
503.30381	1	38892.73

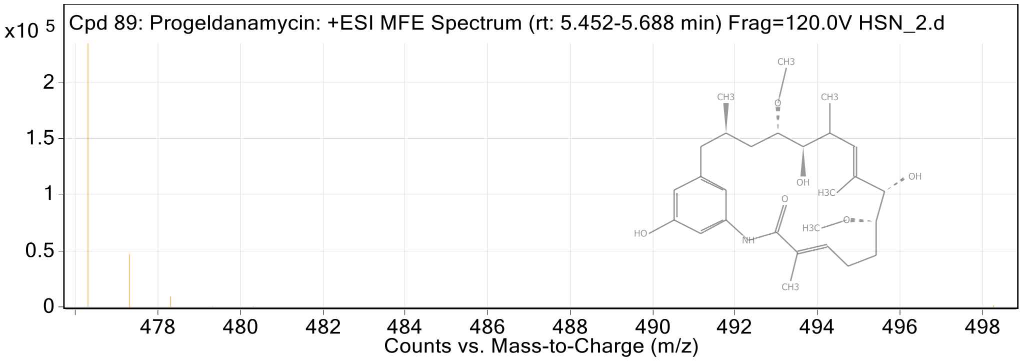

Peak List

m/z	z	Abund	Name	Formula	Ion	Score (DB)	Hits (DB)
476.30102	1	235124.38	Progeldanamycin	C27 H41 N O6	(M+H)+	79.41	2
477.30707	1	47060.22		C27 H41 N O6	(M+H)+		
478.30941	1	9359.83		C27 H41 N O6	(M+H)+		
479.31219	1	370.98		C27 H41 N O6	(M+H)+		
480.31423	1	387.68		C27 H41 N O6	(M+H)+		
498.27898	1	1479.52			(M+Na)+		

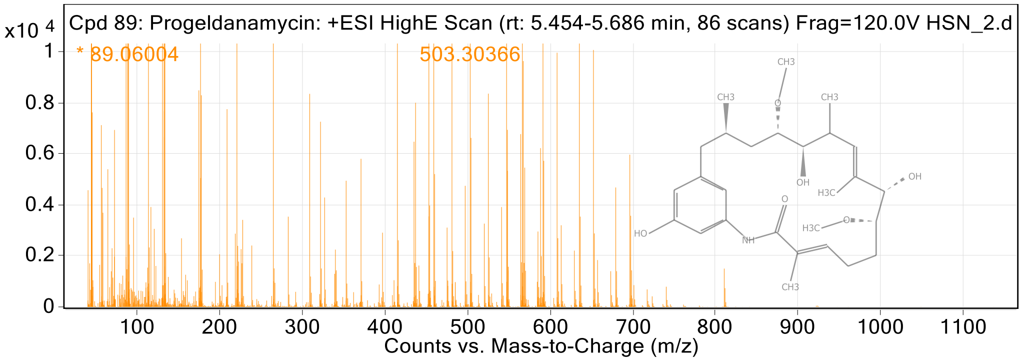

Peak List

m/z	z	Abund
45.03359		77249.15
45.03539		81842.87
87.04302		33124.36
89.06004	1	218324.95
89.14188		27459.67
133.08554	1	155201.81
177.11043	1	87576.24
459.27734	1	24158.24
503.30366	1	26114.21
547.32981	1	24867.46

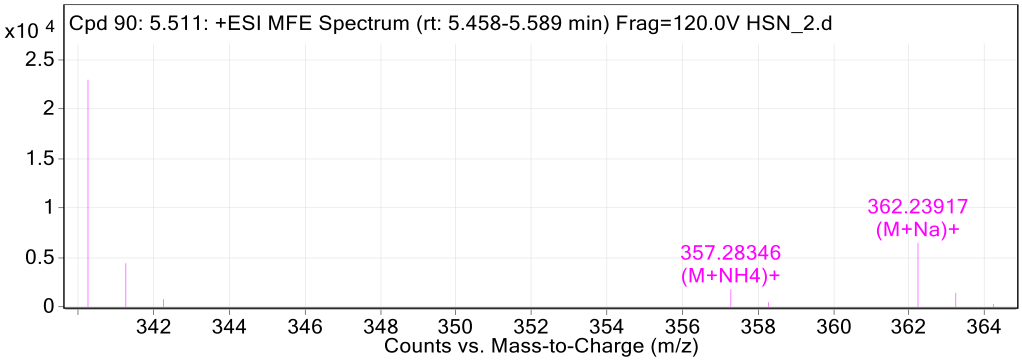

Peak List

m/z	z	Abund	Ion
340.25716	1	22938.96	(M+H)+
341.2603	1	4398.87	(M+H)+
342.26114	1	764.16	(M+H)+
357.28346	1	1782.86	(M+NH4)+
358.2856	1	465.9	(M+NH4)+
362.23917	1	6453.28	(M+Na)+
363.24189	1	1405.79	(M+Na)+
364.24655	1	263.02	(M+Na)+

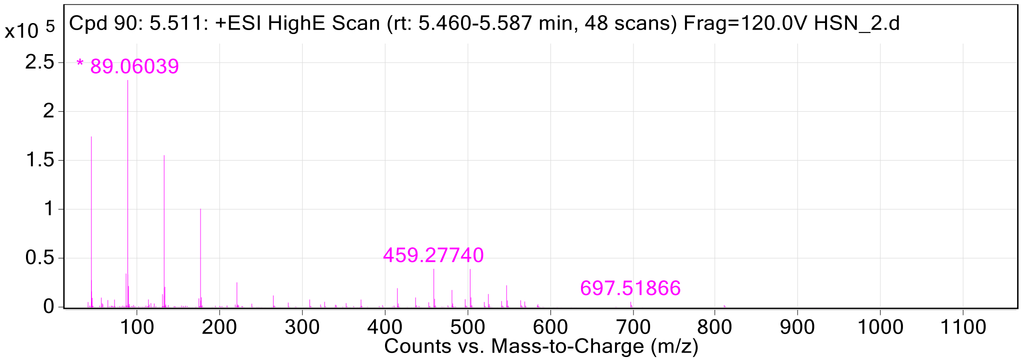

Peak List

m/z	z	Abund
45.03481	1	174402.63
45.09247		27574.48
87.04304	1	34297.24
89.06039	1	232118.11
89.14189		33260.76
133.08624	1	155277.89
177.11055	1	100578.1
221.1362	1	25275.55
459.2774	1	39110.46
503.30381	1	38892.73

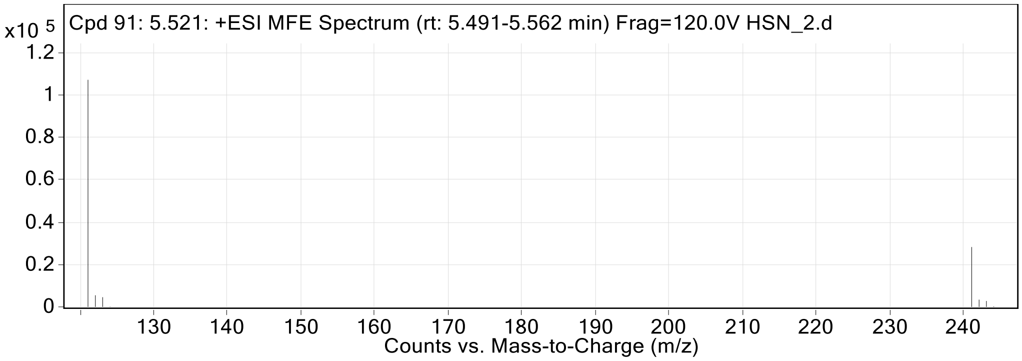

Peak List

m/z	z	Abund	Ion
121.06651	1	107074.63	(M+H)+
122.06948	1	5476.92	(M+H)+
123.06255	1	4568.86	(M+H)+
124.06678	1	117.4	(M+H)+
241.12703	1	28223.58	(2M+H)+
242.1299	1	3421.95	(2M+H)+
243.12373	1	2783.82	(2M+H)+
244.12609	1	224.89	(2M+H)+

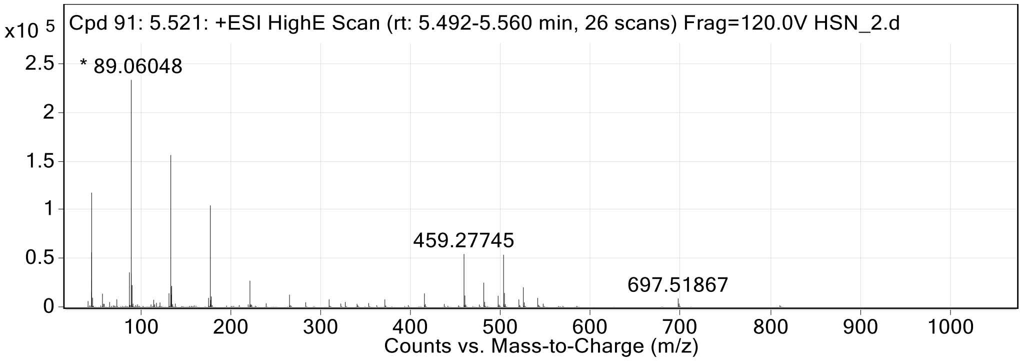

Peak List

m/z	z	Abund
45.03336		56115.82
45.03562		117487.66
45.09247		28036.78
87.04305	1	35545.94
89.06048	1	233450.39
89.1419		34313.68
133.0864	1	156150.92
177.11066	1	104365.75
459.27745	1	54395.77
503.30403	1	53561.13

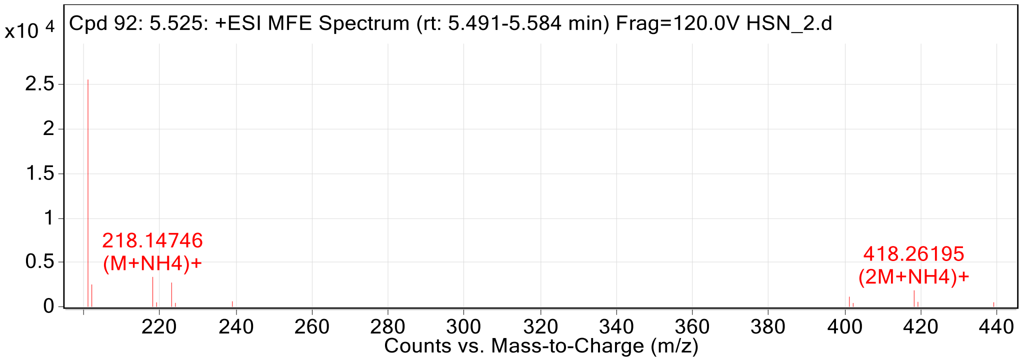

Peak List

m/z	z	Abund	Ion
201.12127	1	25584.61	(M+H)+
202.12475	1	2512.23	(M+H)+
218.14746	1	3362.53	(M+NH4)+
219.14977	1	498.49	(M+NH4)+
223.10339	1	2726.55	(M+Na)+
239.0749	1	615.72	(M+K)+
401.23622	1	1135.29	(2M+H)+
418.26195	1	1853.43	(2M+NH4)+
419.26182	1	553.28	(2M+NH4)+
439.19055	1	507.35	(2M+K)+

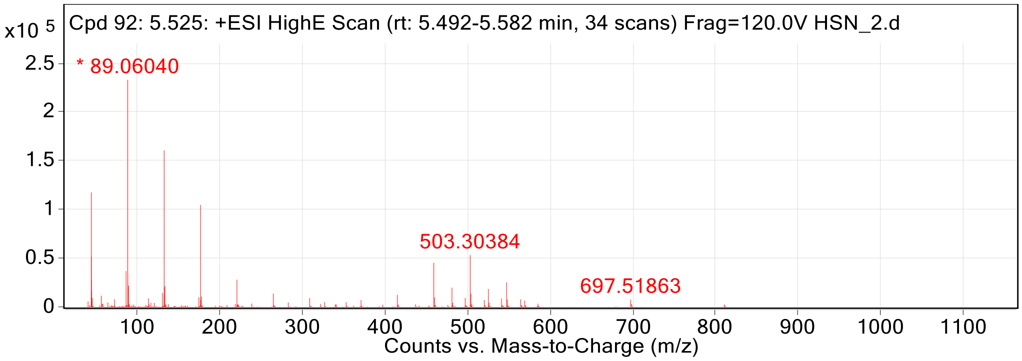

Peak List

m/z	z	Abund
45.03334		51588.18
45.0355		117440.29
87.04304	1	36831.05
89.0604	1	232873.45
89.14188		33528.4
133.08618	1	160580.45
177.11061	1	104577.03
221.13621	1	27853.25
459.27741	1	45152.82
503.30384	1	53069.49

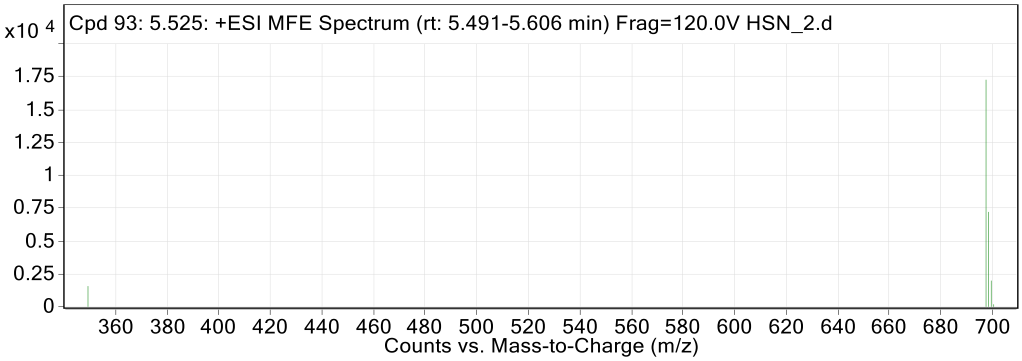

Peak List

m/z	z	Abund	Ion
349.26144	2	1573.02	(M+2H)+2
697.51894	1	17281.17	(M+H)+
698.52159	1	7220.04	(M+H)+
699.52463	1	1990.33	(M+H)+
700.52569	1	186.97	(M+H)+

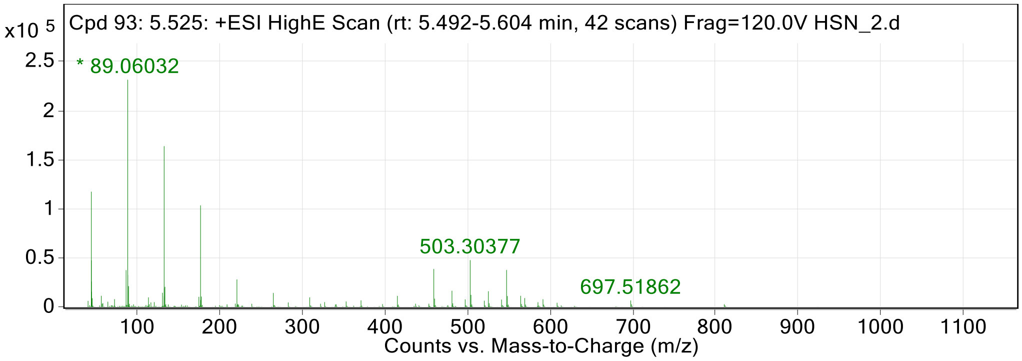

Peak List

m/z	z	Abund

45.03333		47308.53
45.03537		117332.34
87.04305		37399.18
89.06032	1	231441.58
89.14189		32370.24
133.08598	1	163796.78
177.11059	1	103378.11
459.27739	1	38500.11
503.30377	1	47582.74
547.33012	1	37523.51

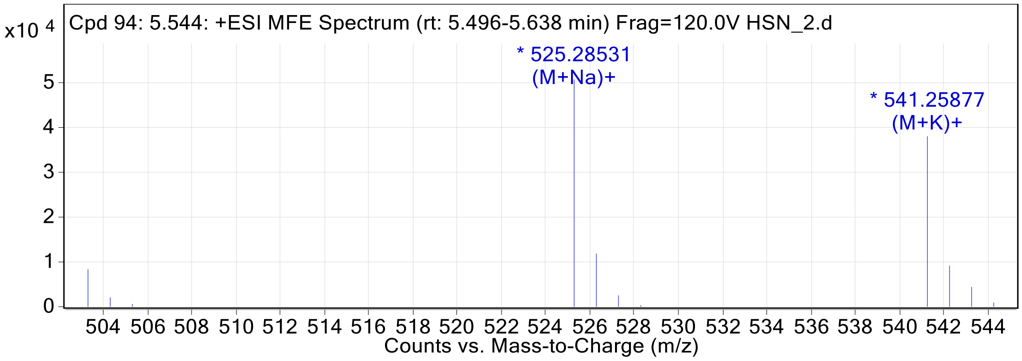

Peak List

m/z	z	Abund	Ion
503.30273	1	8355.25	(M+H)+
504.3073	1	2093.42	(M+H)+
505.30875	1	619.42	(M+H)+
525.28531	1	50723.39	(M+Na)+
526.28903	1	11872.22	(M+Na)+
527.29118	1	2548.9	(M+Na)+
541.25877	1	38061.9	(M+K)+
542.26227	1	9163.46	(M+K)+
543.2602	1	4420.13	(M+K)+
544.26162	1	947.51	(M+K)+

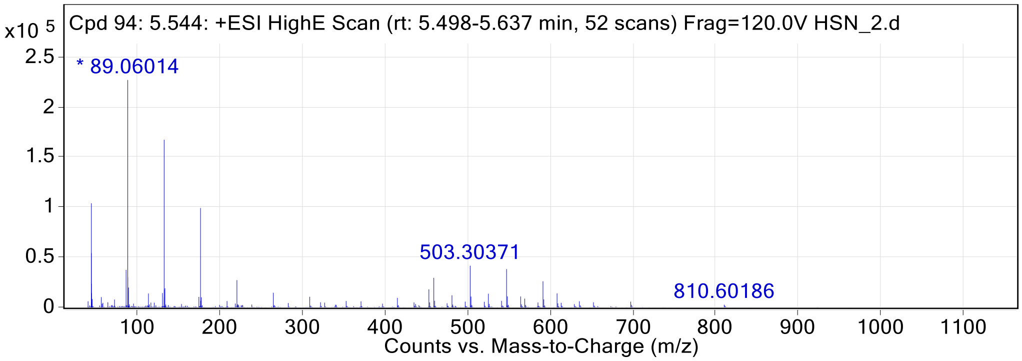

Peak List

m/z	z	Abund
45.03352		53802.63
45.03521		103651.22
87.04303		37148.18
89.06014	1	226750.52
89.14188		29830.83
133.0856	1	167099.94
177.1105	1	98908.66
459.27728	1	29153.37
503.30371	1	41247.05

547.32988	1	37889.48

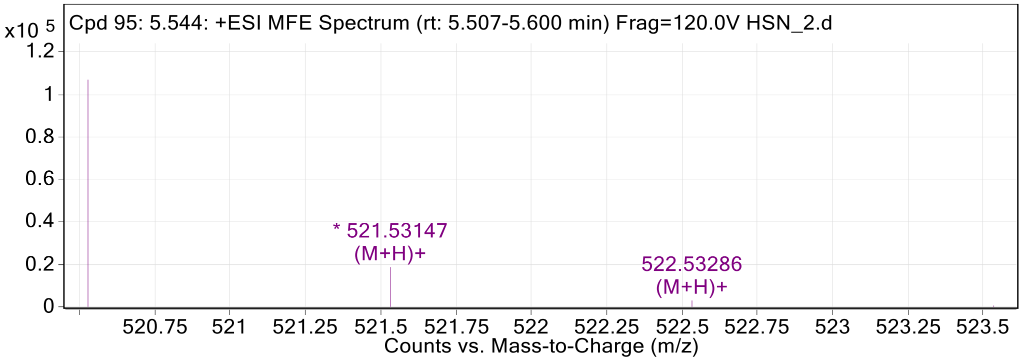

Peak List

m/z	z	Abund	Ion
520.52811	1	106953.02	(M+H)+
521.53147	1	18695.24	(M+H)+
522.53286	1	2965.84	(M+H)+
523.53453	1	569.33	(M+H)+

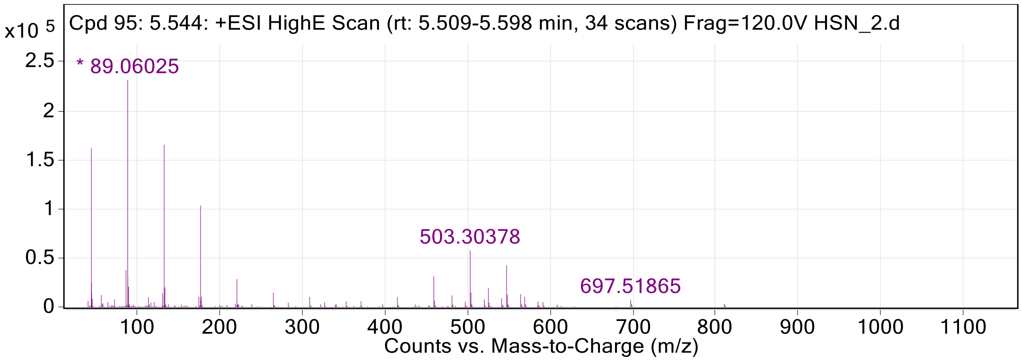

Peak List

m/z	z	Abund
45.03474	1	161693.25
87.04306	1	37717.33
89.06025	1	231448.48
89.14188		31776.86
133.08587	1	165468.22
177.11058	1	103474.02
221.13621	1	28339.92
459.27731	1	31191.1
503.30378	1	57685.82
547.33013	1	42397.83

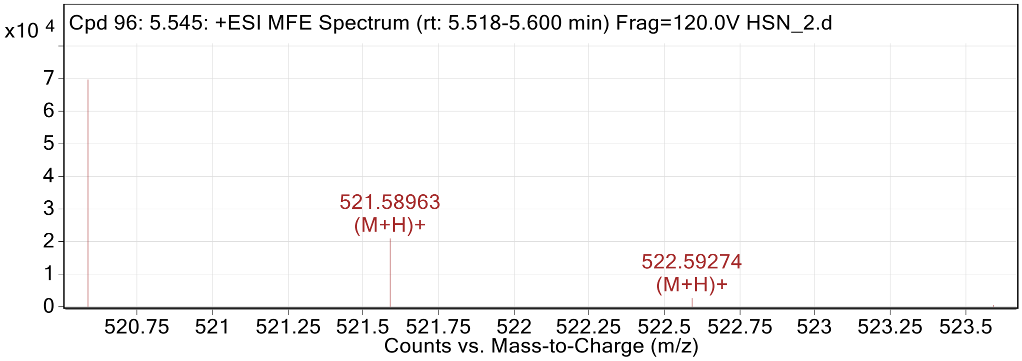

Peak List

m/z	z	Abund	Ion
520.58598	1	69638.84	(M+H)+
521.58963	1	20922.84	(M+H)+
522.59274	1	2667.9	(M+H)+
523.59483	1	544.49	(M+H)+

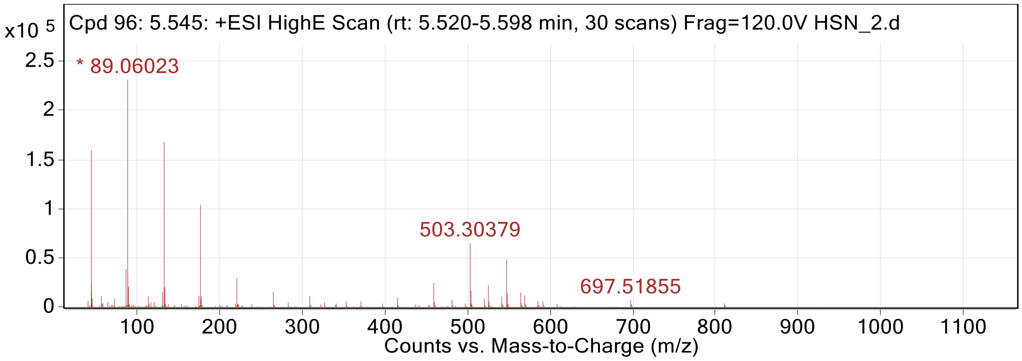

Peak List

m/z	z	Abund
45.03474	1	159333.61
45.09247		24530.78
87.04306	1	38596.46
89.06023	1	231234.36
89.14189		31565.19
133.0858	1	168082.27
177.11059	1	103913.45
221.13619	1	29197.51
503.30379	1	64805.6
547.33013	1	48050.87

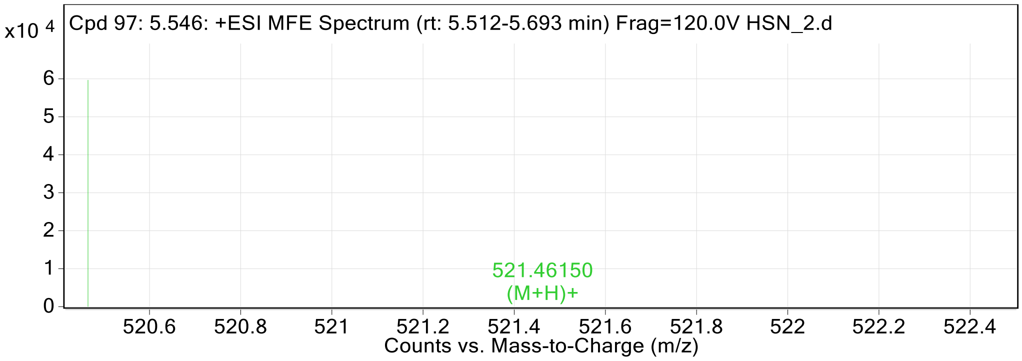

Peak List

m/z	z	Abund	Ion
520.46438	1	59710.3	(M+H)+
521.4615	1	0	(M+H)+
522.45034	1	0	(M+H)+

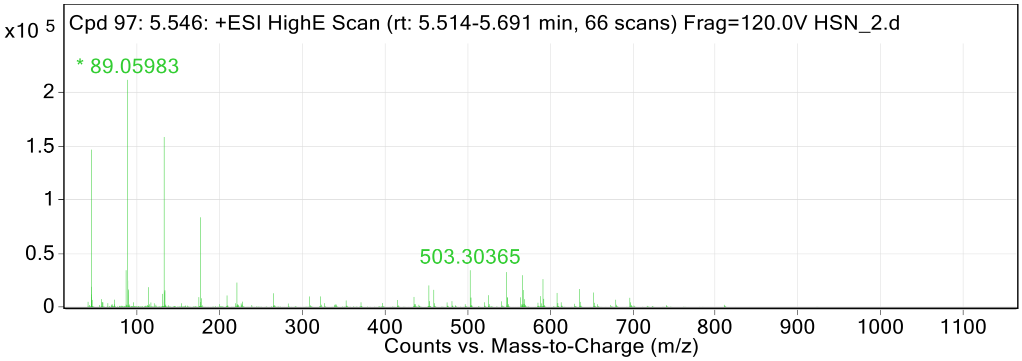

Peak List

m/z	z	Abund
45.03433	1	146801.7
87.04302		34094.19
89.05983	1	211841.27
89.14187		24474.68
133.08515	1	158399.41
177.11042	1	83490.77
503.30365	1	34086.18
547.3298	1	32521.39
566.42698	1	29508.07
591.35559	1	25926.3

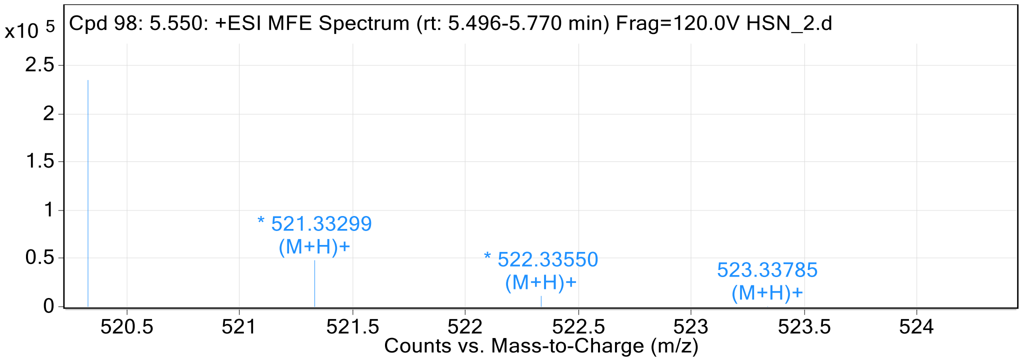

Peak List

m/z	z	Abund	Ion
520.32928	1	235129.88	(M+H)+
521.33299	1	48267.05	(M+H)+
522.3355	1	11253.32	(M+H)+
523.33785	1	439.22	(M+H)+
524.33912	1	11.34	(M+H)+

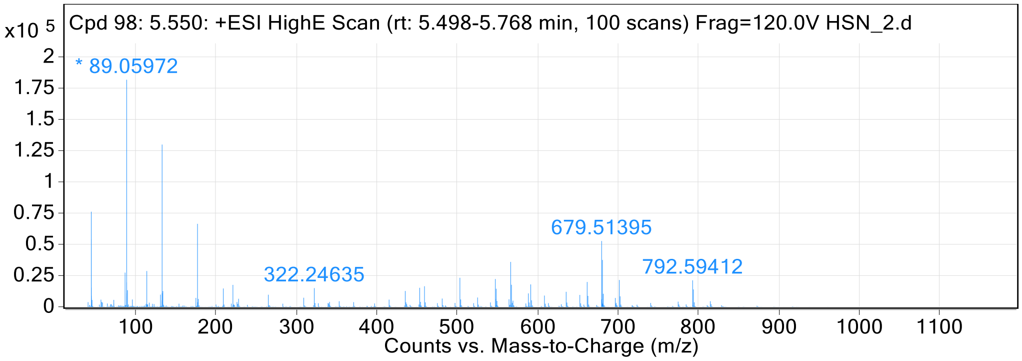

Peak List

m/z	z	Abund

45.03352		76049.33
45.03521		53898.64
87.043		27349.38
89.05972	1	181591.77
114.08977		28595.27
133.08513	1	129793.19
177.1104	1	66348.7
566.42638	1	35896.84
679.51395	1	52618.44
680.51262	1	37449.64

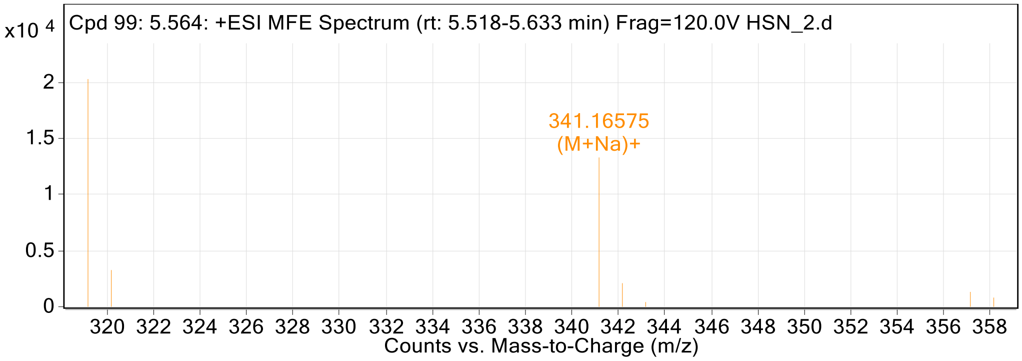

Peak List

m/z	z	Abund	Ion
319.18341	1	20305.74	(M+H)+
320.18709	1	3273.61	(M+H)+
341.16575	1	13317.65	(M+Na)+
342.16915	1	2104.87	(M+Na)+
343.17123	1	419.51	(M+Na)+
357.1375	1	1325.59	(M+K)+
358.14399	1	817.67	(M+K)+

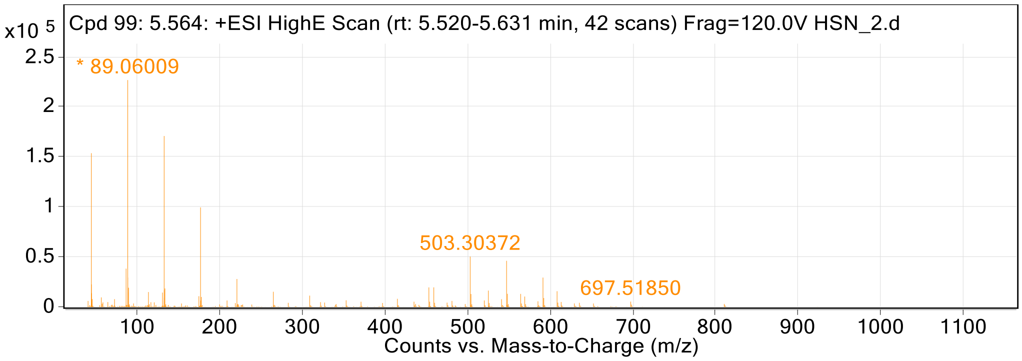

Peak List

m/z	z	Abund
45.0346	1	153520.67
87.04303		38168.95
89.06009	1	226591.41
89.14189		29253.23
133.08547	1	170686.77
177.1105	1	99340.41
221.13621	1	27787.89
503.30372	1	50217.9
547.32989	1	45958.11
591.35581	1	29216.55

Qualitative Analysis Report

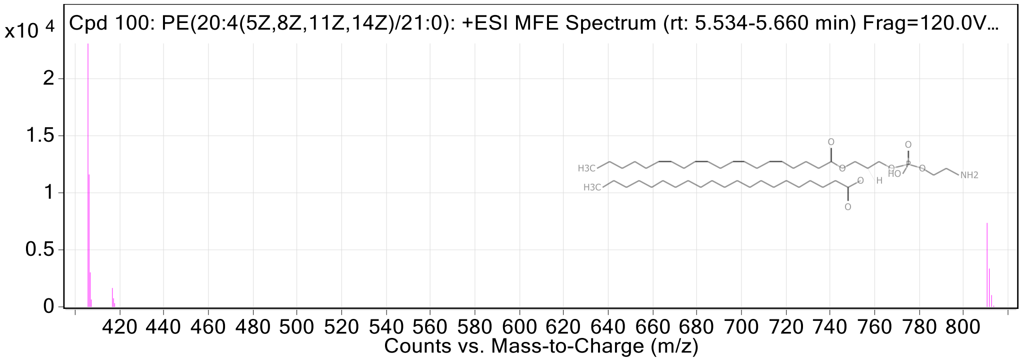

Peak List

m/z	z	Abund	Name	Formula	Ion	Score (DB)	Hits (DB)
405.80411	2	23124.93	PE(20:4(5Z,8Z,11Z,14Z)/21:0)	C46 H84 N O8 P	(M+2H)+2	99.61	4
406.30558	2	11624.95		C46 H84 N O8 P	(M+2H)+2		
406.80714	2	3031.31		C46 H84 N O8 P	(M+2H)+2		
407.3099	2	650.21		C46 H84 N O8 P	(M+2H)+2		
416.79411	2	1661.06			(M+HNa)+2		
417.29652	2	755.59			(M+HNa)+2		
417.79709	2	324.59			(M+HNa)+2		
810.602	1	7372.19			(M+H)+		
811.60543	1	3364.88			(M+H)+		
812.60971	1	1034.04			(M+H)+		

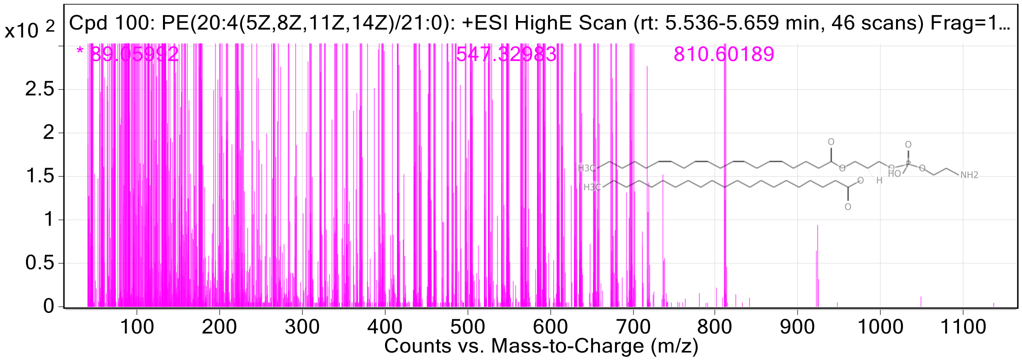

Peak List

m/z	z	Abund
45.03442	1	147497.7
87.04302		37244.14
89.05992	1	219130.05
89.14186		26367.74
133.08514	1	170269.02
177.11041	1	91589.44
221.13619	1	25876.78
503.30368	1	39324.75
547.32983	1	45110.37
591.35563	1	34482.53

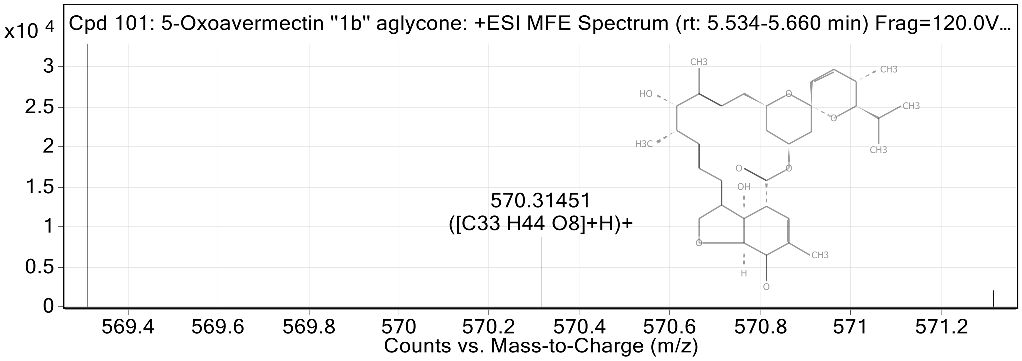

Peak List

m/z	z	Abund	Name	Formula	Ion	Score (DB)	Hits (DB)
569.3114	1	32956.31	5-Oxoavermectin "1b" aglycone	C33 H44 O8	(M+H)+	88.48	5
570.31451	1	8747.84		C33 H44 O8	(M+H)+		
571.31562	1	2043.45		C33 H44 O8	(M+H)+		

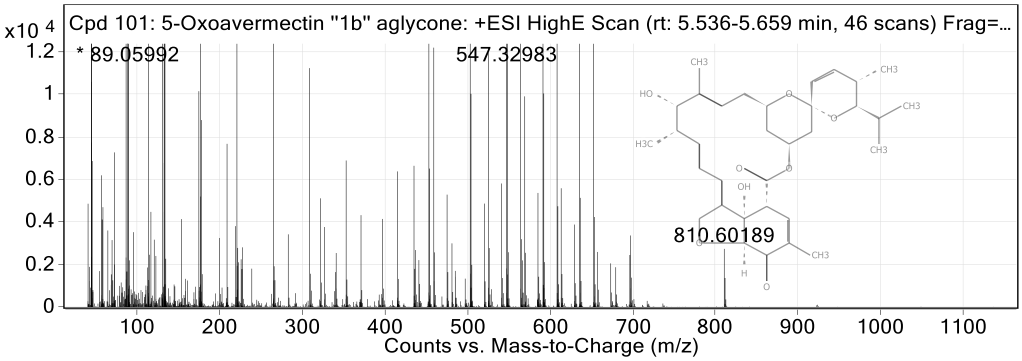

Peak List

m/z	z	Abund
45.03442	1	147497.7
87.04302		37244.14
89.05992	1	219130.05
89.14186		26367.74
133.08514	1	170269.02
177.11041	1	91589.44
221.13619	1	25876.78
503.30368	1	39324.75
547.32983	1	45110.37
591.35563	1	34482.53

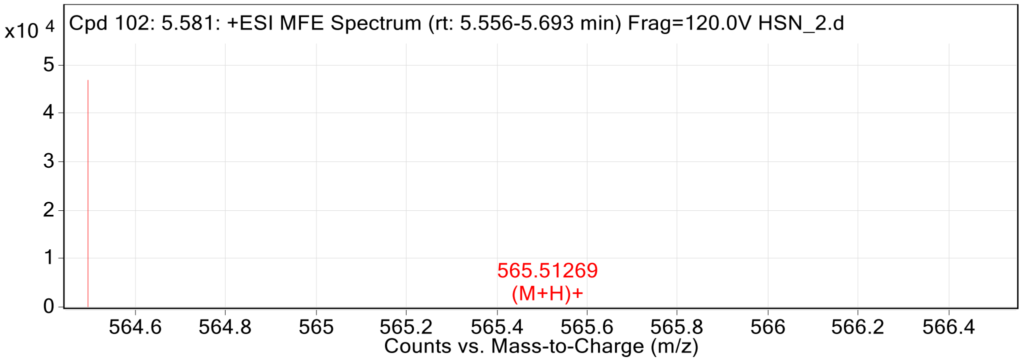

Peak List

m/z	z	Abund	Ion
564.49547	1	46887.62	(M+H)+

565.51269	1	0	(M+H)+
566.49942	1	0	(M+H)+

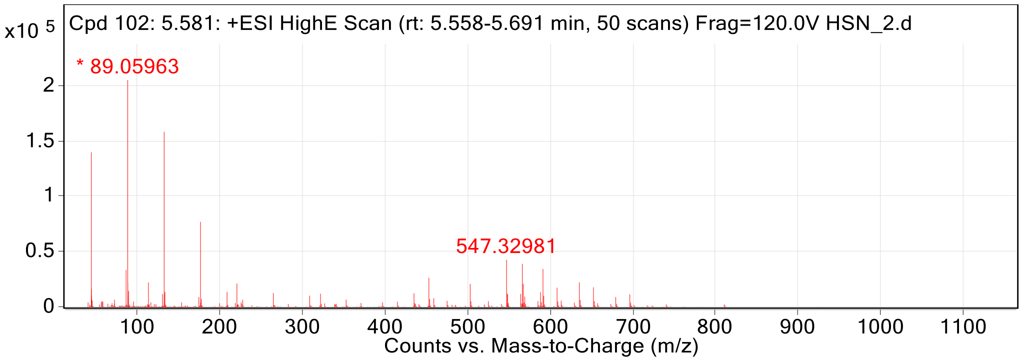

Peak List

m/z	z	Abund
45.03414	1	139623.25
87.043		33418.14
89.05963	1	204966.69
133.0848	1	158222.27
177.11031	1	76806.63
453.34065	1	26205.05
547.32981	1	42492.42
566.42699	1	38808.92
591.35559	1	34222.71
635.38134	1	22142

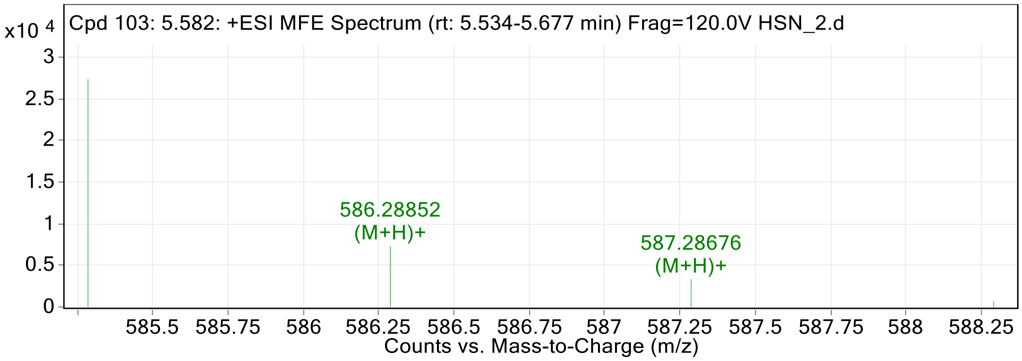

Peak List

m/z	z	Abund	Ion
585.28526	1	27351.38	(M+H)+
586.28852	1	7306.01	(M+H)+
587.28676	1	3324.03	(M+H)+
588.29136	1	706.09	(M+H)+

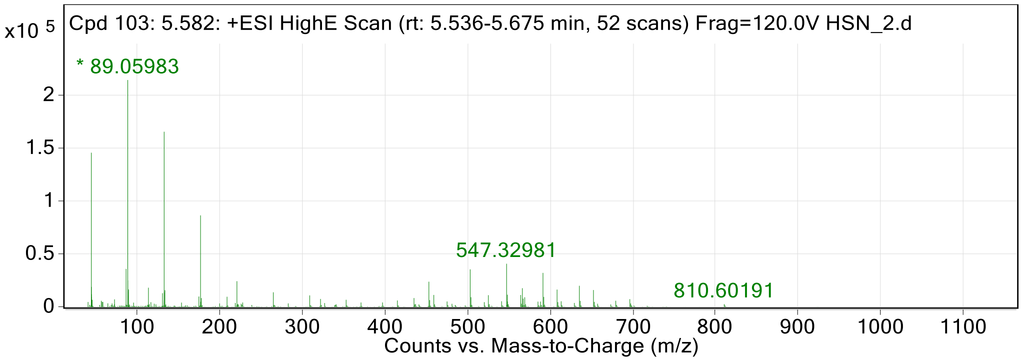

Peak List

m/z	z	Abund
45.03434	1	145849.73
87.04302		35863.97
89.05983	1	214640.97
89.14186		24897.49
133.08506	1	165676.66
177.1104	1	86507.5
221.13618	1	24196.87
503.30366	1	35382.77
547.32981	1	40714.98
591.35561	1	32052.78

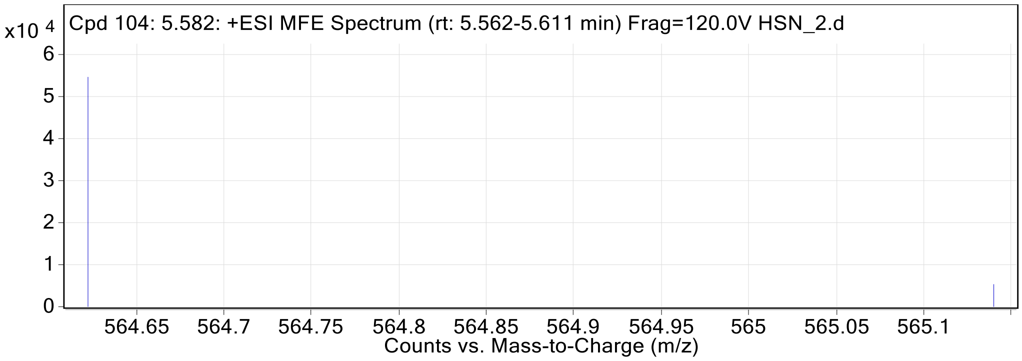

Peak List

m/z	z	Abund	Ion
564.62246	2	54708.76	(M+2H)+2
565.13988	2	5371.84	(M+2H)+2

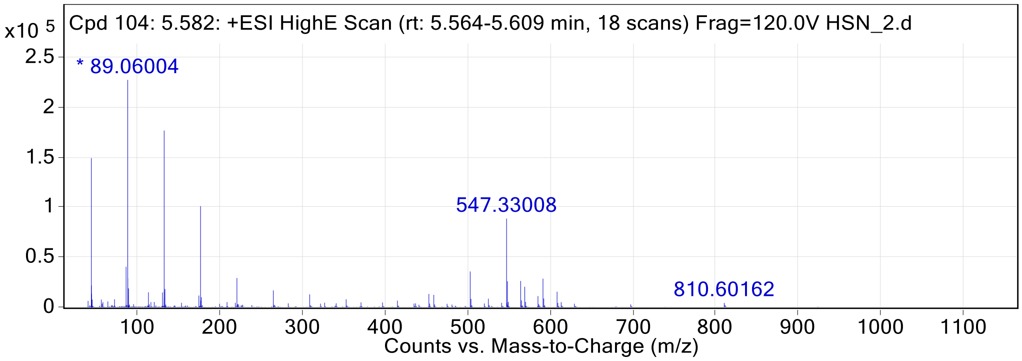

Peak List

m/z	z	Abund
45.03455	1	148981.63
87.04304	1	40289.59
89.06004	1	227261.06
89.14187		28759.54
133.08532	1	176608.69
177.11046	1	100927.6

m/z	z	Abund
221.1362	1	28944.18
503.30318	1	35518.46
547.33008	1	88525.45
591.35581	1	28332.42

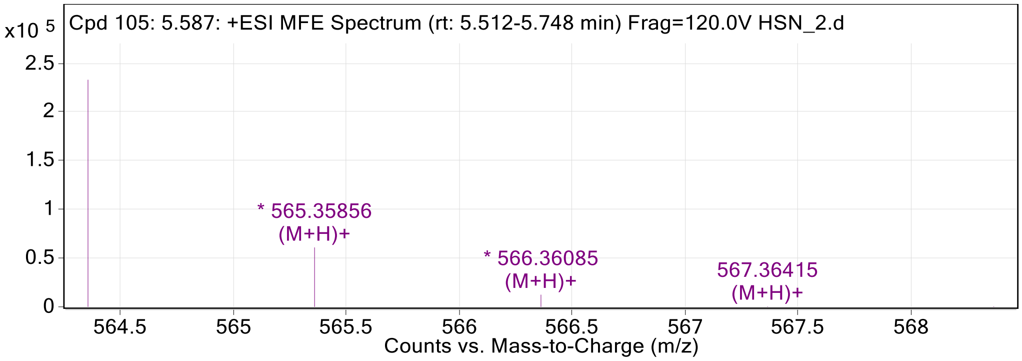

Peak List

m/z	z	Abund	Ion
564.35539	1	233010.61	(M+H)+
565.35856	1	60953.37	(M+H)+
566.36085	1	12479.53	(M+H)+
567.36415	1	443.16	(M+H)+
568.36562	1	505.86	(M+H)+

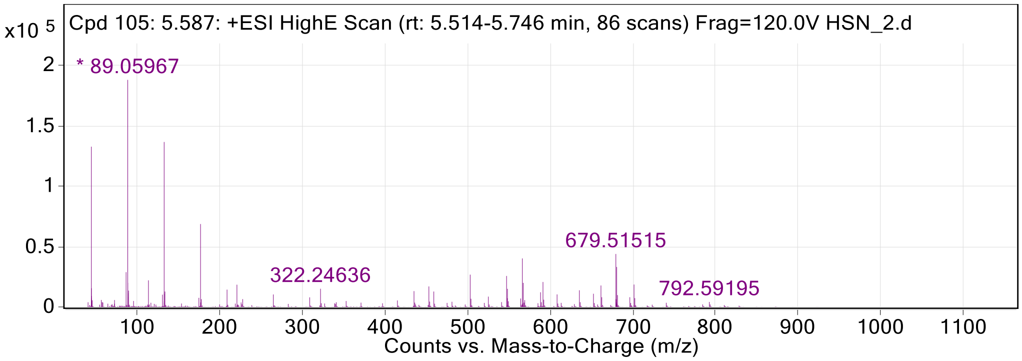

Peak List

m/z	z	Abund
45.03418	1	132825.89
87.04301		28847.76
89.05967	1	188167.47
133.08503	1	136693.55
177.11038	1	68704.15
503.30363	1	26751.21
547.32977	1	25654.49
566.42643	1	40184.23
679.51515	1	43876.72
680.51299	1	33099.63

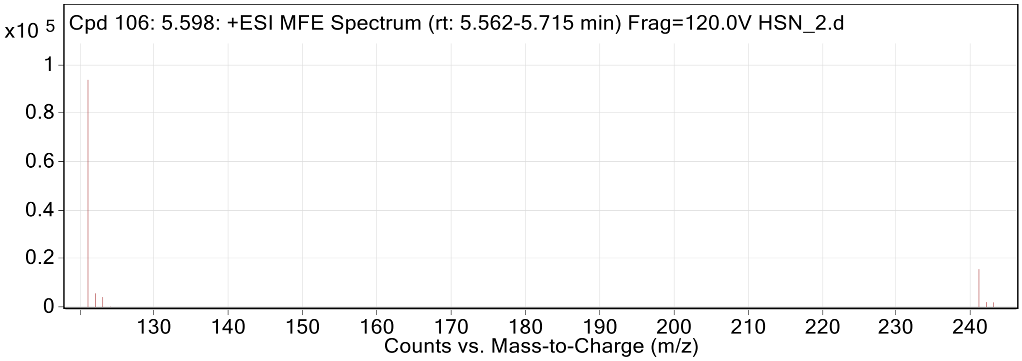

Peak List

m/z	z	Abund	Ion
121.06648	1	93725.55	(M+H)+
122.06932	1	5546.45	(M+H)+
123.06253	1	4066.87	(M+H)+
124.06642	1	78.8	(M+H)+
241.12658	1	15551.76	(2M+H)+
242.13062	1	1971.95	(2M+H)+
243.12307	1	1831.51	(2M+H)+

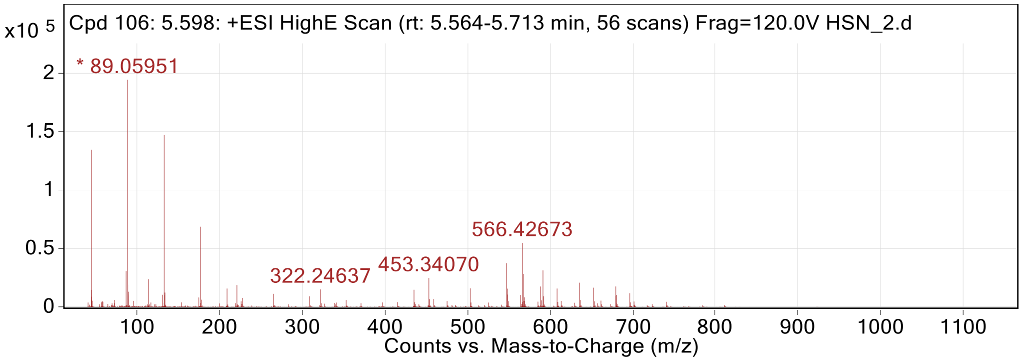

Peak List

m/z	z	Abund
45.03403	1	134559.34
87.04299		30647.91
89.05951	1	194392.08
133.08471	1	147077.22
177.11027	1	68622.19
453.3407	1	24716.89
547.32979	1	37373.47
566.42673	1	54742.32
567.42755	1	28251.4
591.35558	1	31202.23

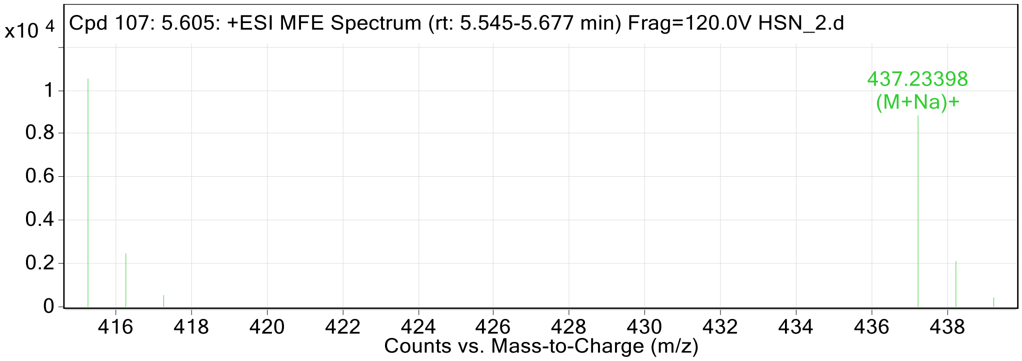

Peak List

m/z	z	Abund	Ion
415.25177	1	10548.66	(M+H)+
416.25488	1	2474.39	(M+H)+
417.25655	1	545.85	(M+H)+
437.23398	1	8846.29	(M+Na)+
438.23666	1	2114.23	(M+Na)+
439.23905	1	432.32	(M+Na)+

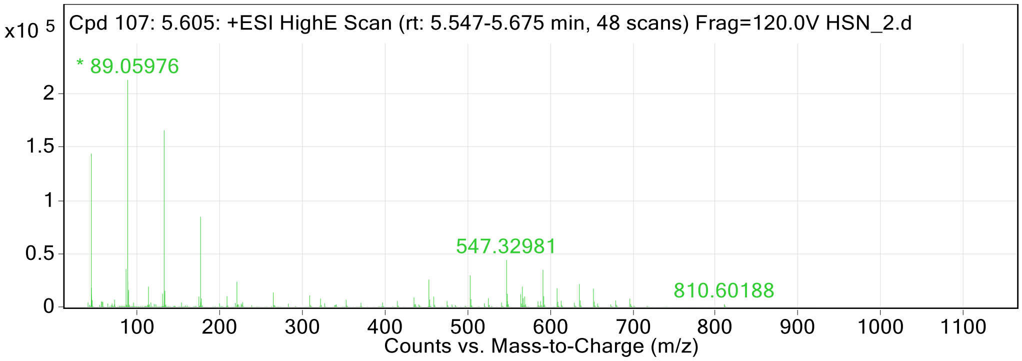

Peak List

m/z	z	Abund
45.03427	1	143902.7
87.04302		35603.33
89.05976	1	213037.88
89.14186		23996.62
133.08495	1	165711.34
177.11035	1	84630.12
453.34063	1	25653.82
503.30344	1	29603.38
547.32981	1	44088.45
591.35561	1	34723.84

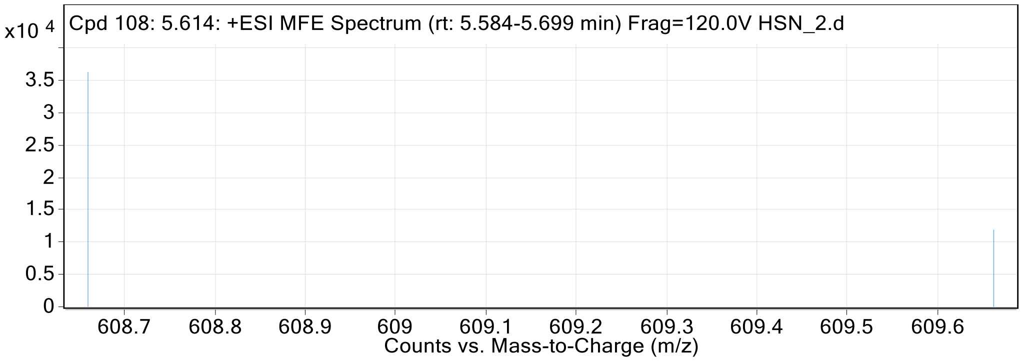

Peak List

m/z	z	Abund	Ion
608.65944	1	36260.67	(M+H)+
609.66247	1	11924.06	(M+H)+

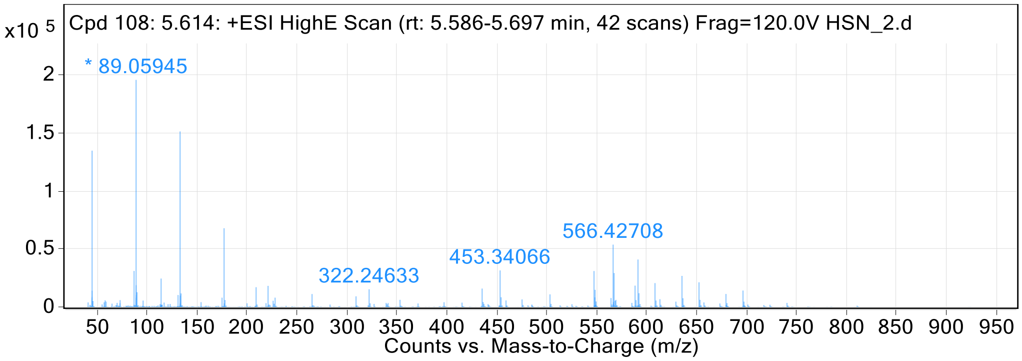

Peak List

m/z	z	Abund
45.03396	1	134866.36
87.04299		31055.04
89.05945	1	195934.61
133.08457	1	151441.02
177.11023	1	67913.66
453.34066	1	31502.71
547.32964	1	30982.11
566.42708	1	53776.8
567.42764	1	29271.87
591.35559	1	40917.73

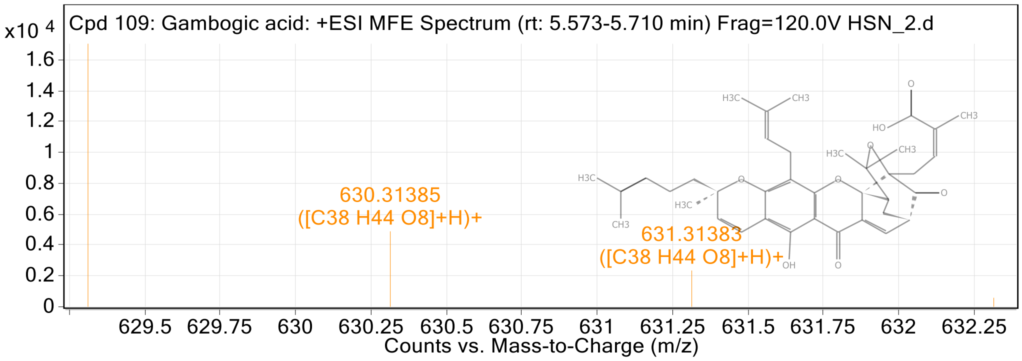

Peak List

m/z	z	Abund	Name	Formula	Ion	Score (DB)	Hits (DB)
629.31109	1	17046.15	Gambogic acid	C38 H44 O8	(M+H)+	76.69	1
630.31385	1	4890.49		C38 H44 O8	(M+H)+		
631.31383	1	2352.46		C38 H44 O8	(M+H)+		
632.31561	1	585.13		C38 H44 O8	(M+H)+		

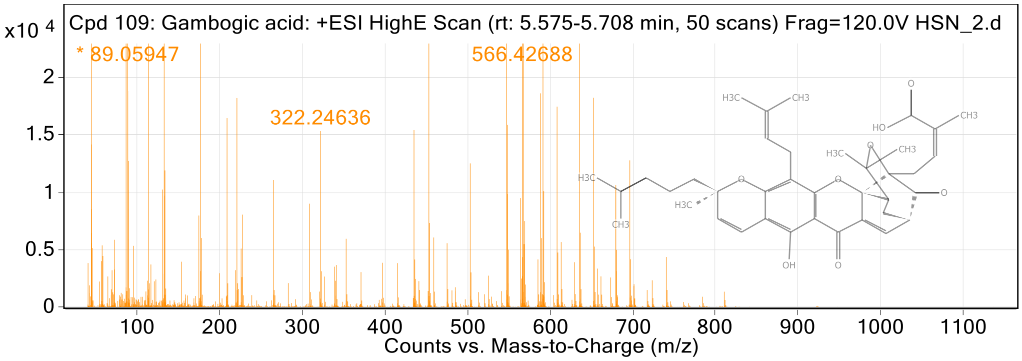

Peak List

m/z	z	Abund
45.03399	1	134358.88
87.04299		30678.84
89.05947	1	194410
133.08465	1	147934.27
177.11026	1	67584.48
453.34069	1	27307.21
547.32981	1	34945.23
566.42688	1	57214.92
567.42757	1	30353.86
591.35558	1	34779.59

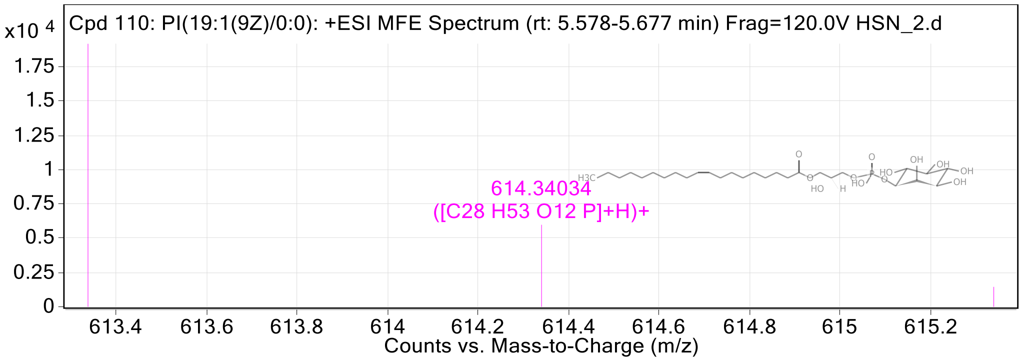

Peak List

m/z	z	Abund	Name	Formula	Ion	Score (DB)	Hits (DB)
613.33698	1	19198.19	PI(19:1(9Z)/0:0)	C28 H53 O12 P	(M+H)+	92.09	1
614.34034	1	5981.98		C28 H53 O12 P	(M+H)+		
615.34069	1	1451.99		C28 H53 O12 P	(M+H)+		

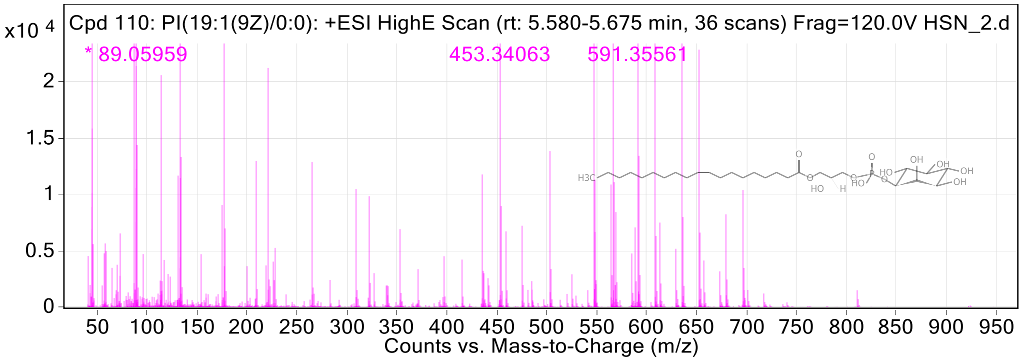

Peak List

m/z	z	Abund
45.0341	1	139194.69
87.043		34340

89.05959	1	206735.44
133.08468	1	164096.66
177.11026	1	77704.95
453.34063	1	33905.29
547.32979	1	41121.06
566.42561	1	24949.55
591.35561	1	46270.71
635.38133	1	28656.8

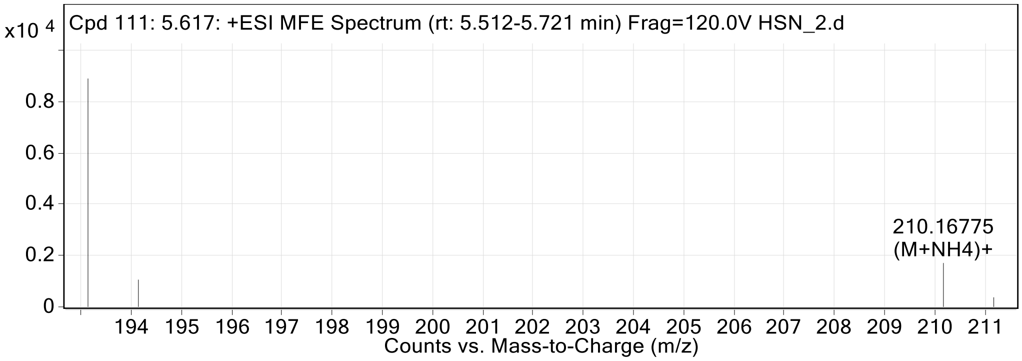

Peak List

m/z	z	Abund	Ion
193.14139	1	8905.3	(M+H)+
194.14449	1	1061.38	(M+H)+
210.16775	1	1711.16	(M+NH4)+
211.17016	1	372.44	(M+NH4)+

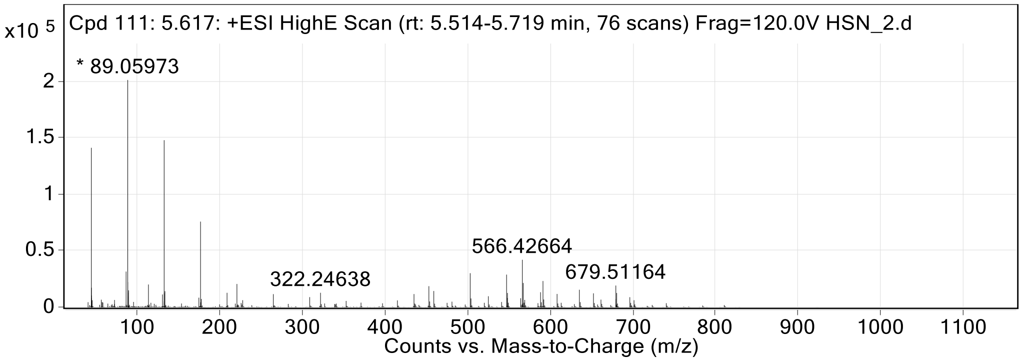

Peak List

m/z	z	Abund
45.03424	1	141166.19
87.04301		31463.94
89.05973	1	201262.19
89.14187		22118.39
133.08508	1	147898.25
177.1104	1	75722.94
503.30364	1	29997.74
547.32978	1	28729.99
566.42664	1	41889.97
591.35558	1	23069.21

Qualitative Analysis Report

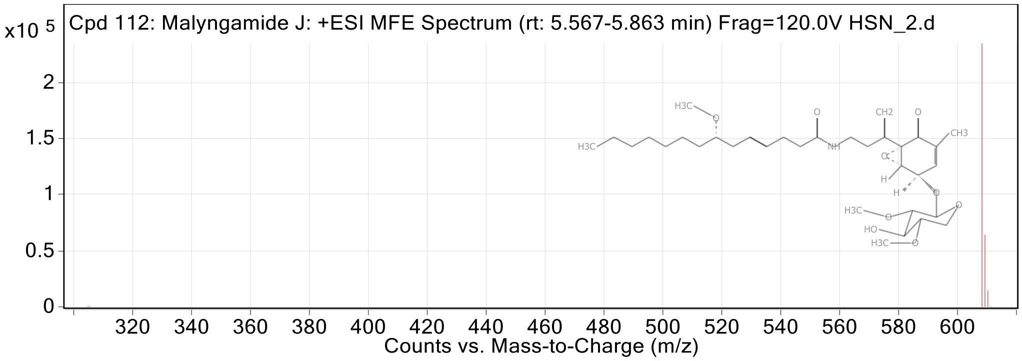

Peak List

m/z	z	Abund	Name	Formula	Ion	Score (DB)	Hits (DB)
304.69558	2	671.65			(M+2H)+2		
305.18925	2	599.36			(M+2H)+2		
608.38159	1	234689.52	Malyngamide J	C33 H53 N O9	(M+H)+	82.54	1
609.38478	1	64163.17		C33 H53 N O9	(M+H)+		
610.38732	1	14824.83		C33 H53 N O9	(M+H)+		
611.38948	1	531.06		C33 H53 N O9	(M+H)+		

m/z	z	Abund	Name	Formula	Ion	Score (DB)	Hits (DB)
612.39391	1	25.76		C33 H53 N O9	(M+H)+		

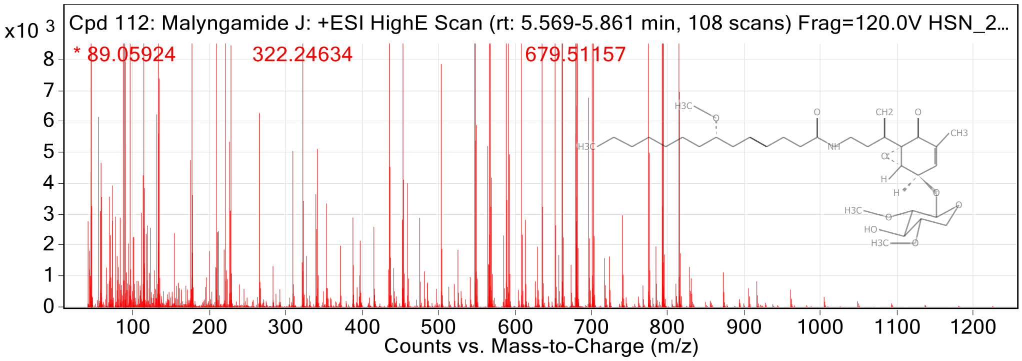

Peak List

m/z	z	Abund
45.03377	1	98960.22
89.05924	1	134049.56
114.08981	1	56972.45
133.08454	1	94790.71
177.11021	1	40492.55
566.4262	1	36985.31
679.51157	1	81513.91
680.51212	1	48201.71
792.59336	1	63549.93
793.59482	1	38600.95

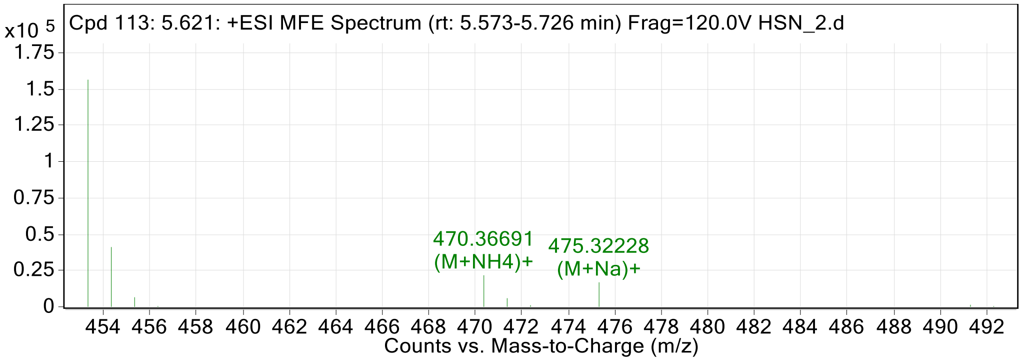

Peak List

m/z	z	Abund	Ion
453.34069	1	156337.17	(M+H)+
454.34355	1	41124.11	(M+H)+
455.34635	1	6586.68	(M+H)+
456.34847	1	541.06	(M+H)+
470.36691	1	21719.04	(M+NH4)+
471.36969	1	5924.84	(M+NH4)+
472.37428	1	1091.51	(M+NH4)+
475.32228	1	16854.94	(M+Na)+
491.2965	1	1385.65	(M+K)+
492.29877	1	503.04	(M+K)+

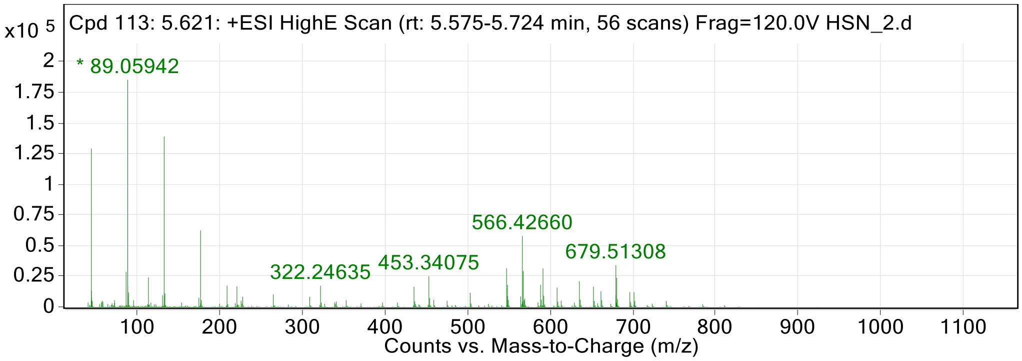

Peak List

m/z	z	Abund
45.03394	1	129235.45
87.04298		28525.47
89.05942	1	185150.36
133.08461	1	138966.63
177.11025	1	62387.9
547.3298	1	31510.32
566.4266	1	57840.62
567.42754	1	29236.04
591.35557	1	31403.73
679.51308	1	34006.71

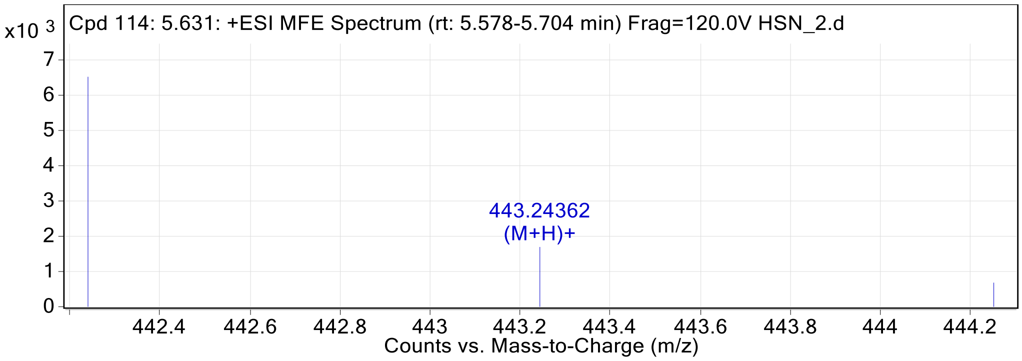

Peak List

m/z	z	Abund	Ion
442.24062	1	6538.97	(M+H)+
443.24362	1	1700.13	(M+H)+
444.25115	1	685.64	(M+H)+

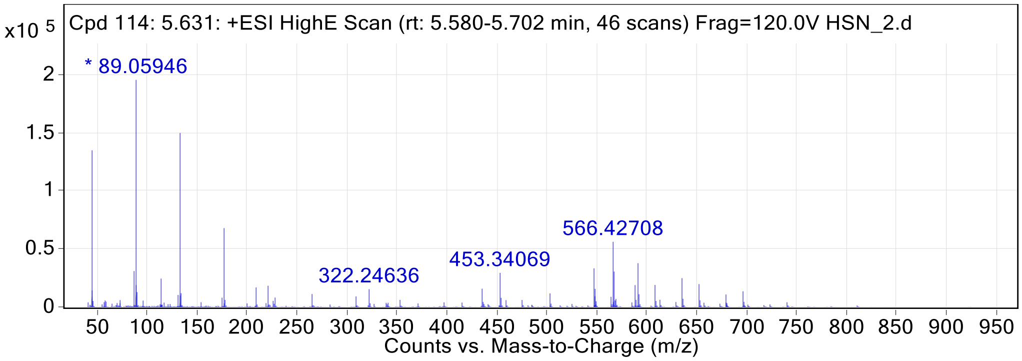

Peak List

m/z	z	Abund
45.03397	1	134692.38
87.04299		30827.09

89.05946	1	195285.72
133.08461	1	149574.77
177.11024	1	67776.5
453.34069	1	29268.41
547.32977	1	33170.57
566.42708	1	55996.09
567.42763	1	30408.21
591.35558	1	37607.43

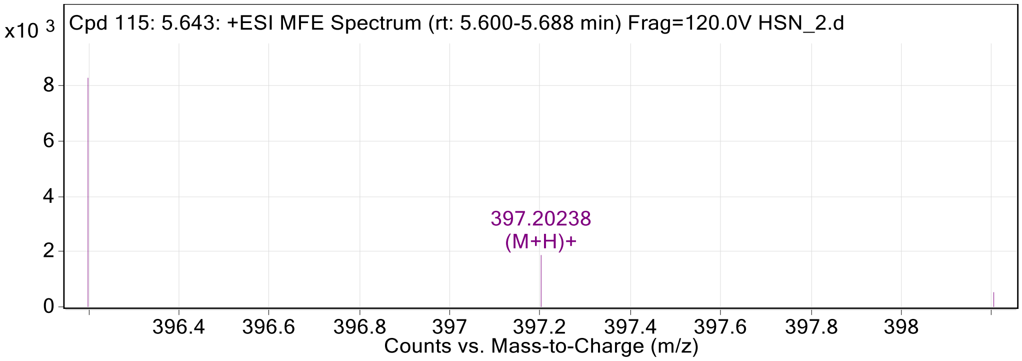

Peak List

m/z	z	Abund	Ion
396.19874	1	8282.24	(M+H)+
397.20238	1	1873.73	(M+H)+
398.2049	1	530.89	(M+H)+

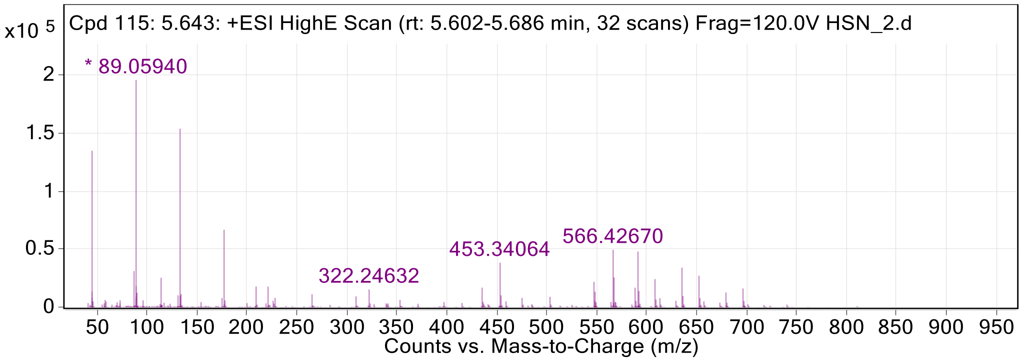

Peak List

m/z	z	Abund
45.03391	1	134936.06
87.04298		31089.22
89.0594	1	195929.33
133.08445	1	153966.77
177.11018	1	66786.33
453.34064	1	38320.72
566.4267	1	49467.05
591.35561	1	47883.67
635.38132	1	33994.08
652.40764	1	27064.93

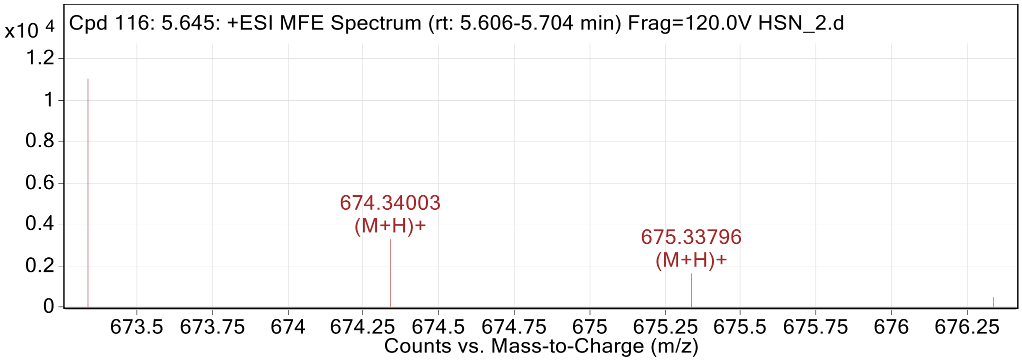

Peak List

m/z	z	Abund	Ion
673.33741	1	11036.79	(M+H)+
674.34003	1	3269.14	(M+H)+
675.33796	1	1615.18	(M+H)+
676.33948	1	462.07	(M+H)+

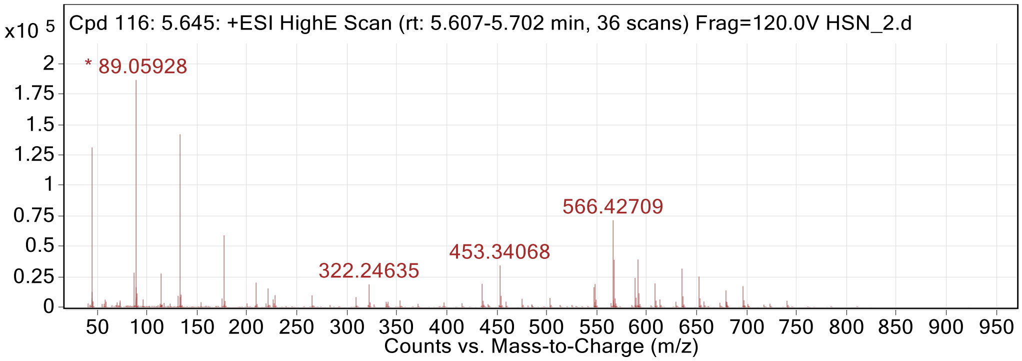

Peak List

m/z	z	Abund
45.03381	1	131222.08
87.04297		28231.23
89.05928	1	186678.98
133.08438	1	142046.36
177.11013	1	58957.85
453.34068	1	34208.11
566.42709	1	71364.03
567.42763	1	38796.05
591.35554	1	39113.27
635.38132	1	31601.59

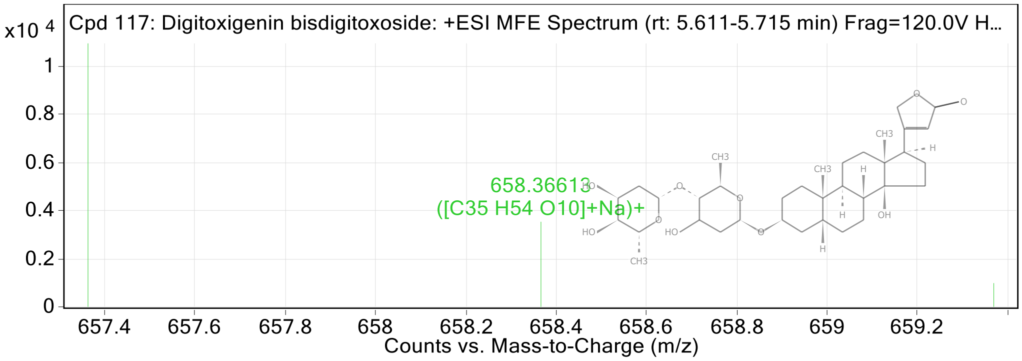

Peak List

m/z	z	Abund	Name	Formula	Ion	Score (DB)	Hits (DB)
657.36307	1	10966.16	Digitoxigenin bisdigitoxoside	C35 H54 O10	(M+Na)+	89.03	2

658.36613	1	3541.91		C35 H54 O10	(M+Na)+		
659.3689	1	987.22		C35 H54 O10	(M+Na)+		

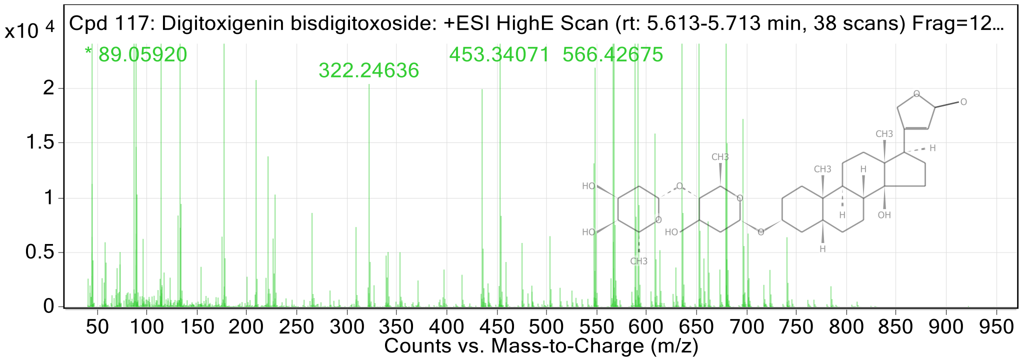

Peak List

m/z	z	Abund
45.03374	1	127727.75
89.0592	1	178822.58
114.08969		27846.05
133.08433	1	133088.58
177.1101	1	53319.63
453.34071	1	30266.63
566.42675	1	80270.29
567.42756	1	41518.55
591.35548	1	32561.62
635.38131	1	30456.86

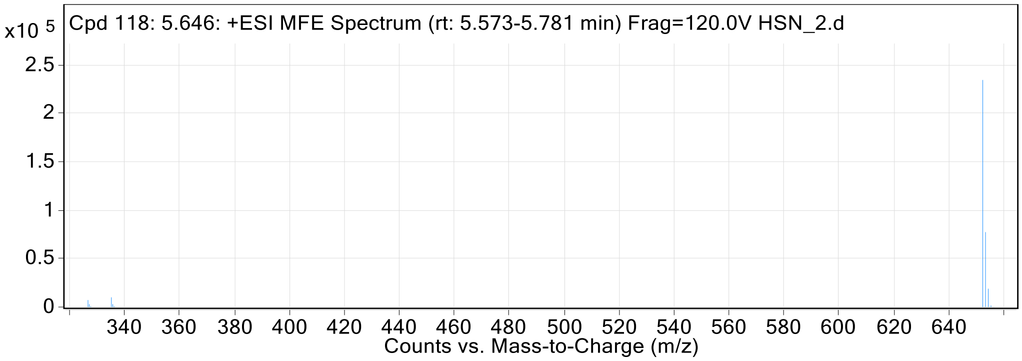

Peak List

m/z	z	Abund	Ion
326.70711	2	7193.03	(M+2H)+2
327.20209	2	2960.6	(M+2H)+2
327.70998	2	695.19	(M+2H)+2
335.22024	2	9991.23	(M+H5N)+2
335.72169	2	3002.29	(M+H5N)+2
336.22222	2	881.78	(M+H5N)+2
652.40812	1	234334.08	(M+H)+
653.41066	1	77304.61	(M+H)+
654.41306	1	18904.69	(M+H)+
655.41641	1	1498.46	(M+H)+

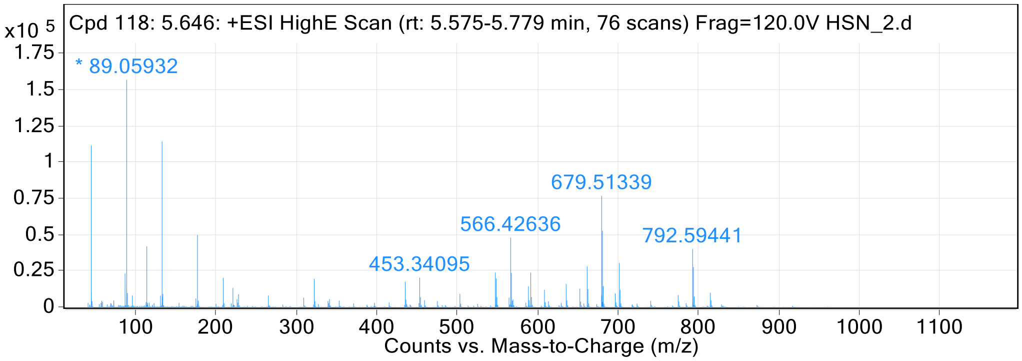

Peak List

m/z	z	Abund
45.03384	1	111491.06
89.05932	1	156752.16
114.08979	1	41879.12
133.08456	1	114242.54
177.11022	1	49585.49
566.42636	1	47767.43
679.51339	1	76670.73
680.51251	1	52524.35
701.48957	1	30329.77
792.59441	1	39903.01

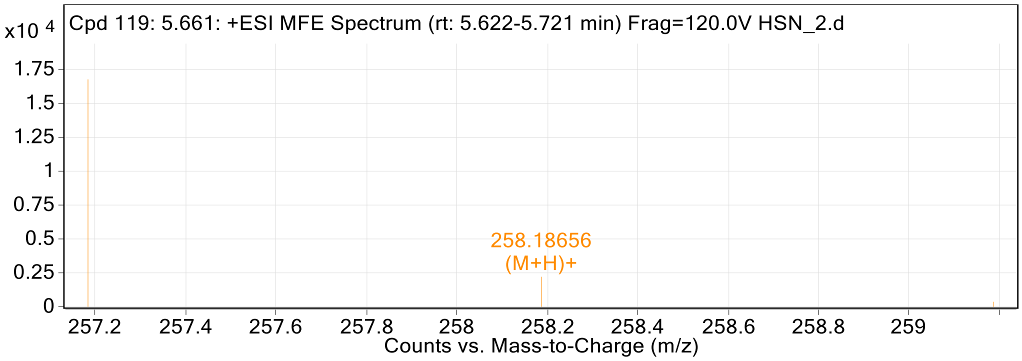

Peak List

m/z	z	Abund	Ion
257.18376	1	16721.35	(M+H)+
258.18656	1	2205.9	(M+H)+
259.18752	1	367.61	(M+H)+

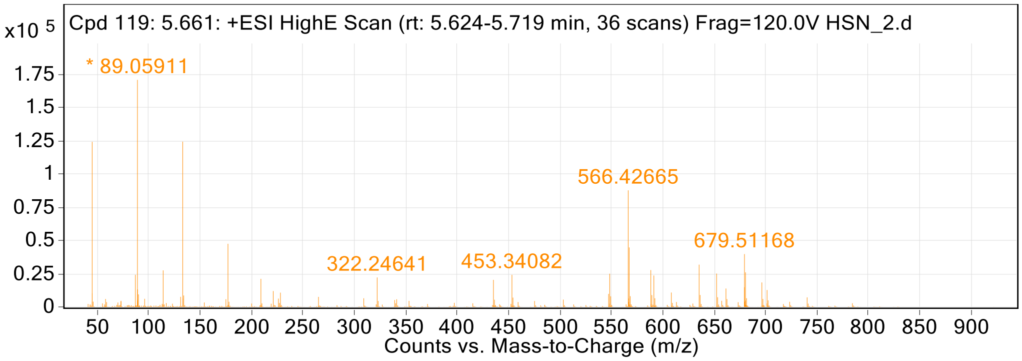

Peak List

m/z	z	Abund
45.03367	1	124190.97
89.05911	1	170796.25

114.08968		27481.19
133.08428	1	124300.3

m/z	z	Abund
177.11009	1	47475.37
566.42665	1	87685.53
567.42756	1	44742.8
588.40585	1	27709.58
635.38132	1	31776.35
679.51168	1	39736.06

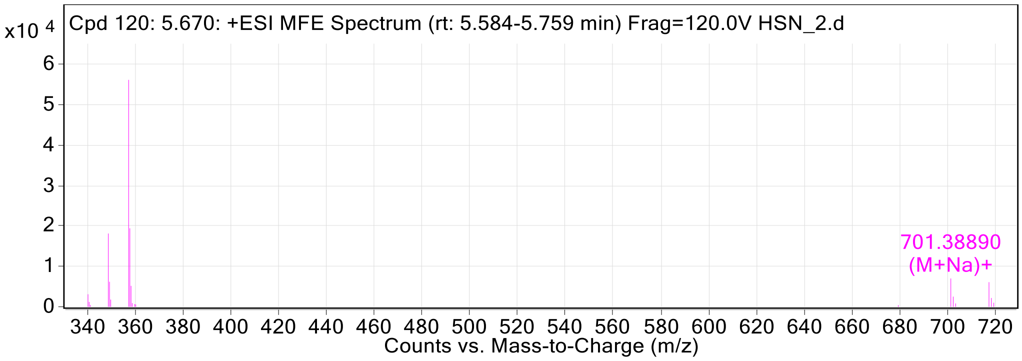

Peak List

m/z	z	Abund	Ion
340.20682	2	3108.99	(M+2H)+2
348.72024	2	18152.06	(M+H5N)+2
349.2219	2	6196.82	(M+H5N)+2
357.23294	2	56205.35	(M+2(NH4))+2
357.73473	2	19444.4	(M+2(NH4))+2
358.23613	2	5188.37	(M+2(NH4))+2
701.3889	1	7020.37	(M+Na)+
702.39327	1	2519.23	(M+Na)+
717.3623	1	6100.68	(M+K)+
718.36612	1	2185.18	(M+K)+

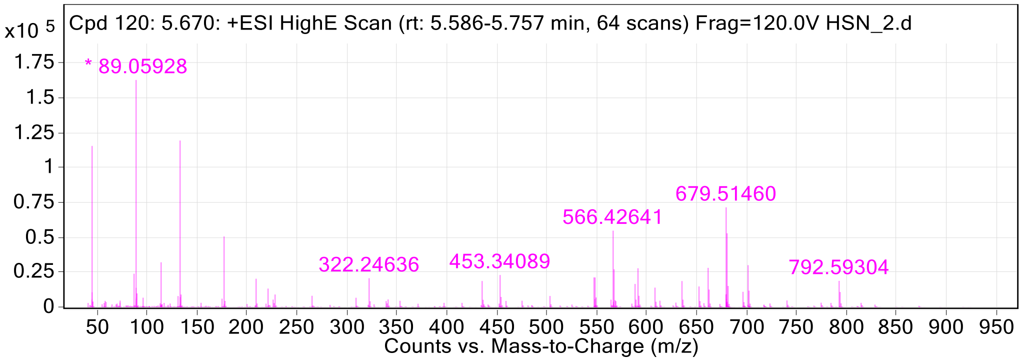

Peak List

m/z	z	Abund
45.03381	1	115625.77
89.05928	1	162968.48
114.08974		32095.32
133.08449	1	119511.21
177.11019	1	50644.36
566.42641	1	54823.54
661.49771	1	28104.53
679.5146	1	71530.88
680.51278	1	52918.25
701.48953	1	30040.88

Qualitative Analysis Report

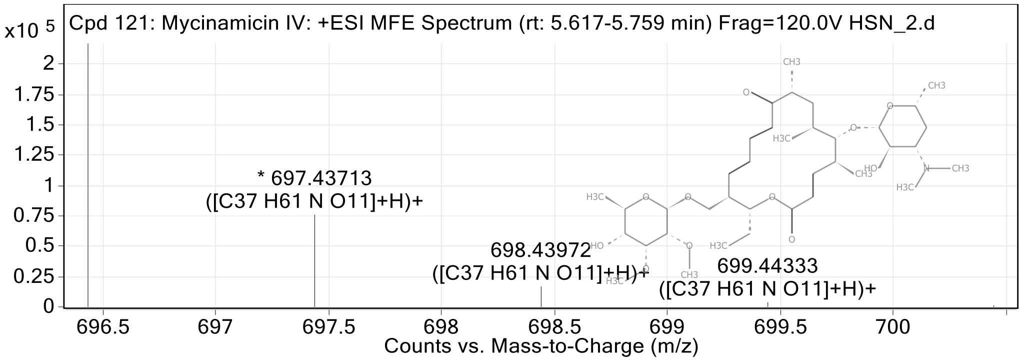

Peak List

m/z	z	Abund	Name	Formula	Ion	Score (DB)	Hits (DB)
696.43341	1	217117.78	Mycinamicin IV	C37 H61 N O11	(M+H)+	91.19	1
697.43713	1	75991.85		C37 H61 N O11	(M+H)+		
698.43972	1	16784.32		C37 H61 N O11	(M+H)+		
699.44333	1	3775.79		C37 H61 N O11	(M+H)+		
700.44355	1	1196.95		C37 H61 N O11	(M+H)+		

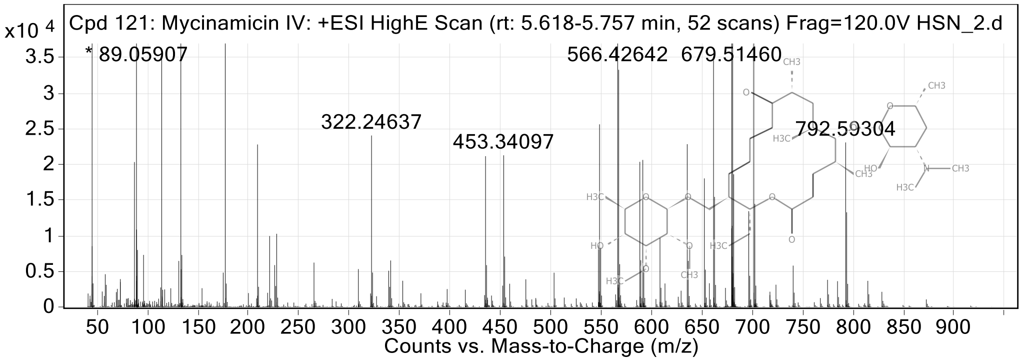

Peak List

m/z	z	Abund
45.03363	1	109108.13
89.05907	1	149080.77
114.08974		35471.9
133.08426	1	105951.58
177.11006	1	40003.38
566.42642	1	67339.45
661.49771	1	34590.19
679.5146	1	87947.12
680.51278	1	65113.96
701.48953	1	36973.39

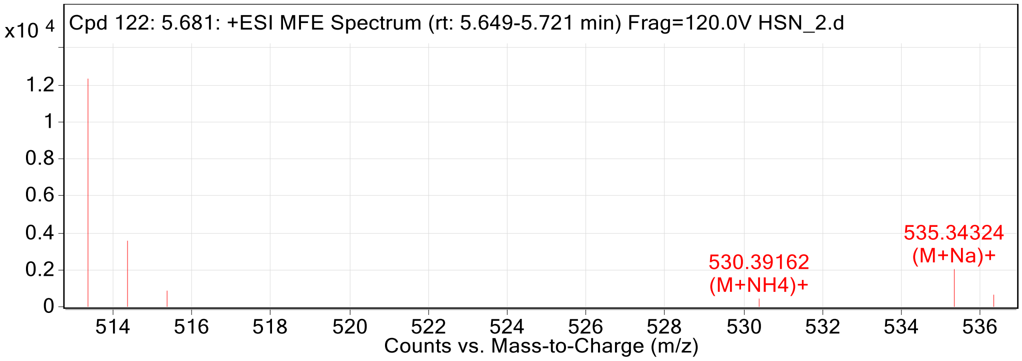

Peak List

m/z	z	Abund	Ion

513.36162	1	12346.29	(M+H)+
514.36488	1	3574.57	(M+H)+
515.36846	1	871.91	(M+H)+
530.39162	1	443.76	(M+NH4)+
535.34324	1	2037.47	(M+Na)+
536.34585	1	654.11	(M+Na)+

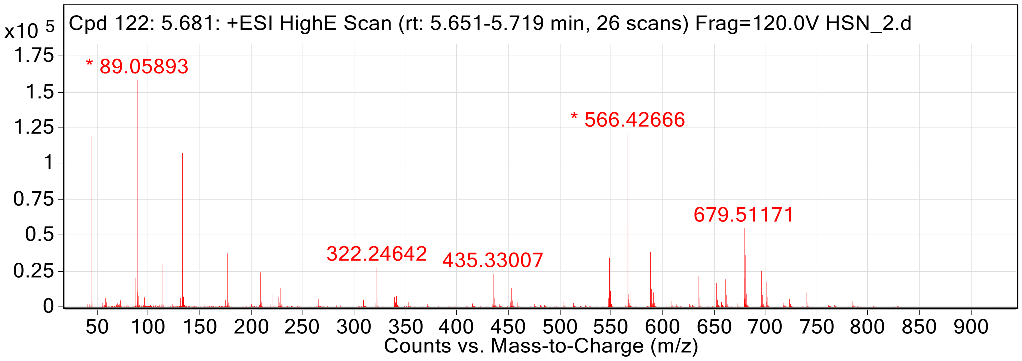

Peak List

m/z	z	Abund
45.03354	1	119571.24
89.05893	1	158316.42
133.0842	1	107230.95
177.11007	1	37253.76
548.41355	1	34288.08
566.42666	1	121114.95
567.42756	1	61863.94
588.40585	1	38334.98
679.51171	1	54809.36
680.51175	1	35935.94

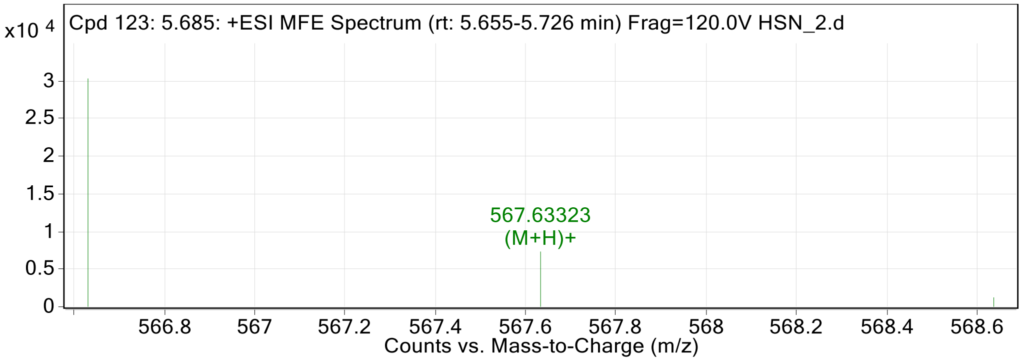

Peak List

m/z	z	Abund	Ion
566.63082	1	30321.11	(M+H)+
567.63323	1	7333.49	(M+H)+
568.63694	1	1246.41	(M+H)+

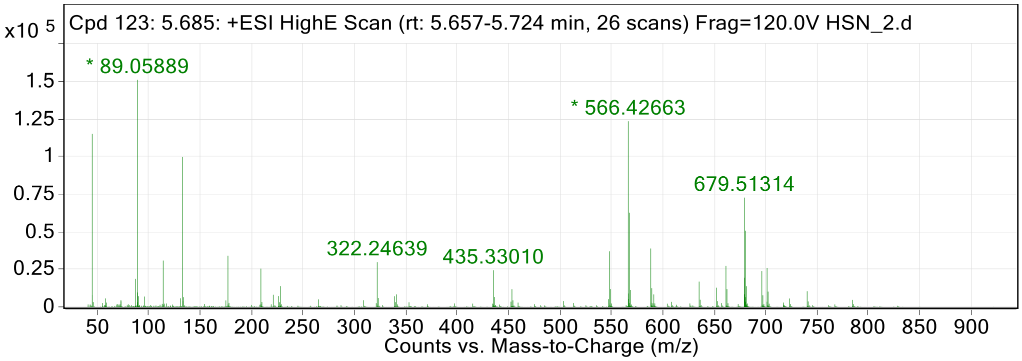

Peak List

m/z	z	Abund
45.0335	1	115090.48
89.05889	1	150927.2
133.08418	1	99701.16
177.11004	1	33997.77
548.41353	1	36899.17
566.42663	1	123387.76
567.42755	1	62621.92
588.40586	1	38786.43
679.51314	1	72642.45
680.51228	1	50653.78

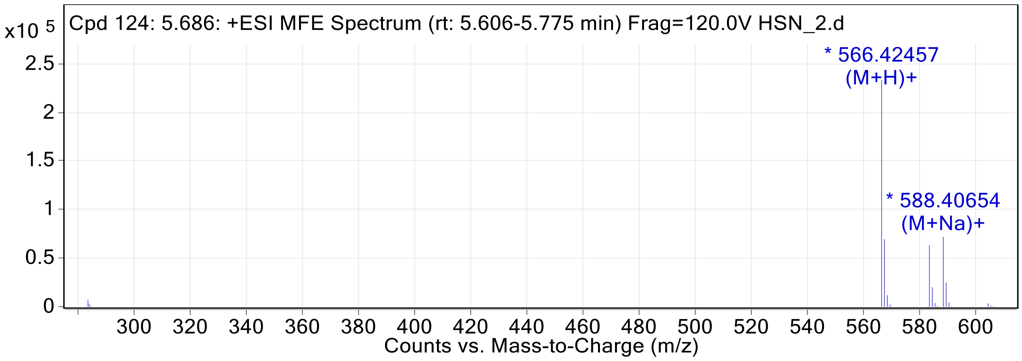

Peak List

m/z	z	Abund	Ion
283.71501	2	7422.54	(M+2H)+2
566.42457	1	233048	(M+H)+
567.4272	1	69546.59	(M+H)+
568.43043	1	11824.42	(M+H)+
583.44989	1	63156.19	(M+NH4)+
584.45379	1	20017.8	(M+NH4)+
585.45587	1	3822.31	(M+NH4)+
588.40654	1	71900.2	(M+Na)+
589.40898	1	24845.43	(M+Na)+
590.41096	1	4514.1	(M+Na)+

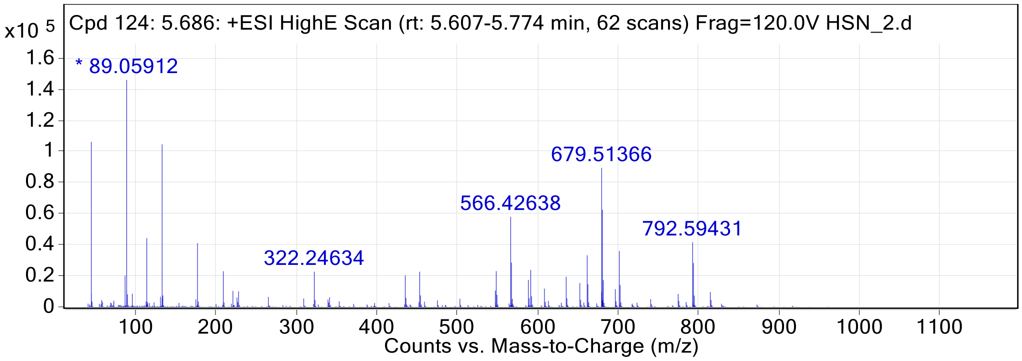

Peak List

m/z	z	Abund
45.03366	1	106046.34
89.05912	1	145941.97
114.08979	1	44215.95
133.08431	1	104497.91
177.11009	1	40866.36
566.42638	1	57943.58
679.51366	1	89346.86
680.51256	1	62434.18
701.48957	1	35937.41
792.59431	1	41570.63

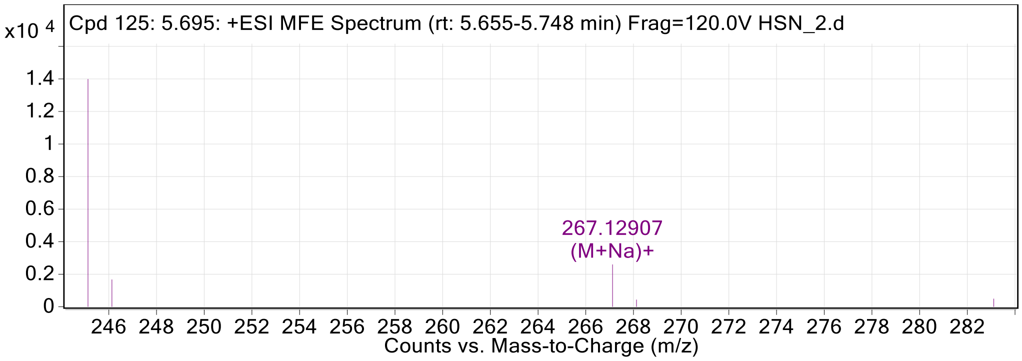

Peak List

m/z	z	Abund	Ion
245.14732	1	13969.81	(M+H)+
246.15025	1	1673.73	(M+H)+
267.12907	1	2597.45	(M+Na)+
268.13238	1	438.85	(M+Na)+
283.10402	1	496.45	(M+K)+

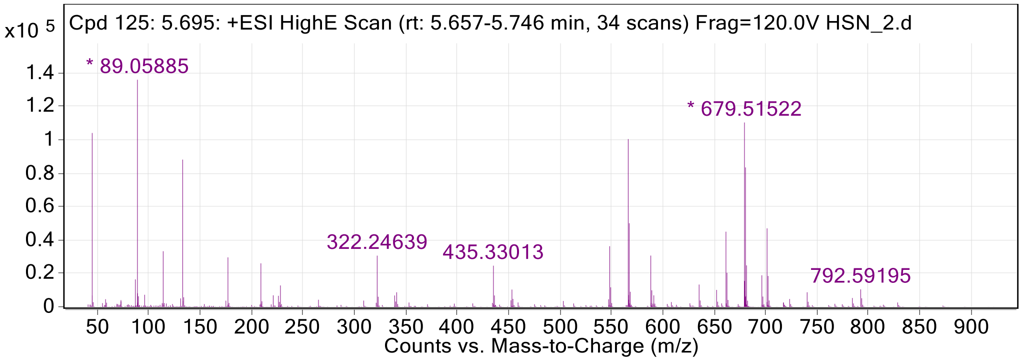

Peak List

m/z	z	Abund

45.03346	1	103946.6
89.05885	1	135777.16
133.08416	1	88024.96
548.41353	1	36229.88
566.42646	1	100287.22
567.42753	1	49963.84
661.49783	1	44919.95
679.51522	1	110233.68
680.51302	1	83348.67
701.48951	1	46900.79

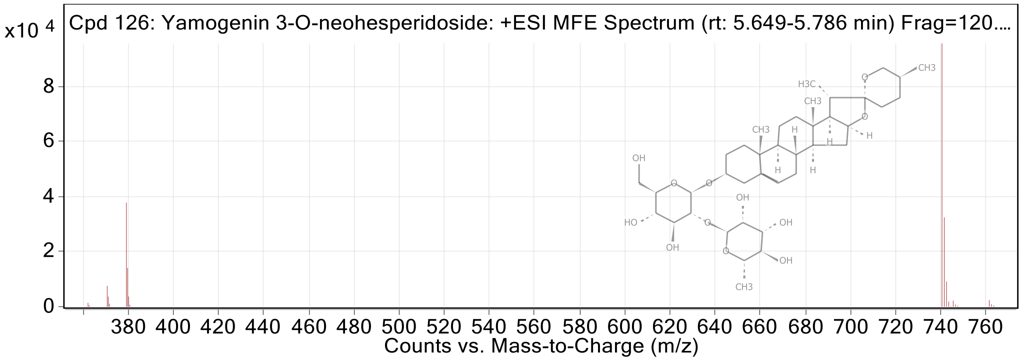

Peak List

m/z	z	Abund	Name	Formula	Ion	Score (DB)	Hits (DB)
370.73306	2	7589.79			(M+H5N)+2		
371.23177	2	3696.72			(M+H5N)+2		
379.2464	2	37781.3			(M+2(NH4))+2		
379.74797	2	14045.37			(M+2(NH4))+2		
380.24913	2	3716.18			(M+2(NH4))+2		
740.45958	1	95522.48	Yamogenin 3-O-neohesperidoside	C39 H62 O12	(M+NH4)+	88.59	3
741.46271	1	32474.65		C39 H62 O12	(M+NH4)+		
742.4655	1	9164.2		C39 H62 O12	(M+NH4)+		
745.41565	1	2180.77			(M+Na)+		
761.38961	1	2395.31			(M+K)+		

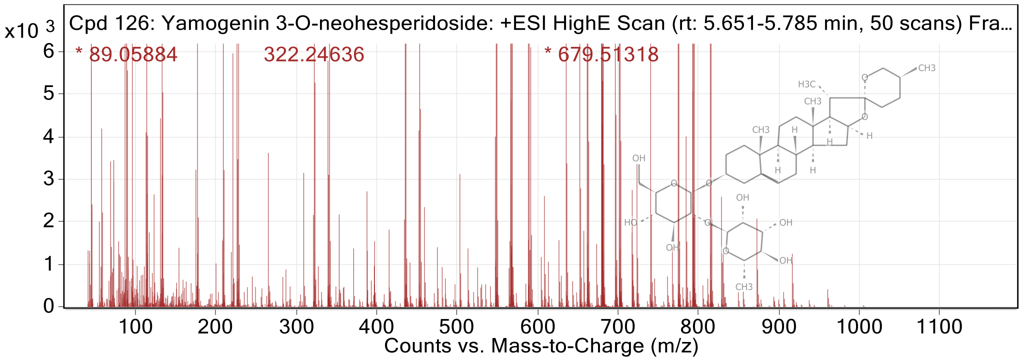

Peak List

m/z	z	Abund
45.03345	1	92363.73
89.05884	1	119966.11
114.08982	1	59004.34
133.08415	1	77727.34
566.42636	1	72793.15
679.51318	1	121795.93
680.51247	1	82080.63
701.48958	1	47646.94

792.59448	1	69699.48
793.59507	1	48228.04

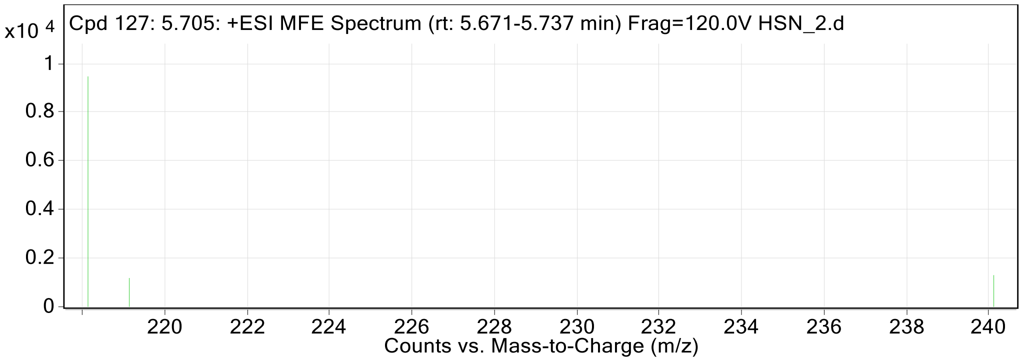

Peak List

m/z	z	Abund	Ion
218.13638	1	9467.59	(M+H)+
219.13857	1	1183.73	(M+H)+
240.11802	1	1301.54	(M+Na)+

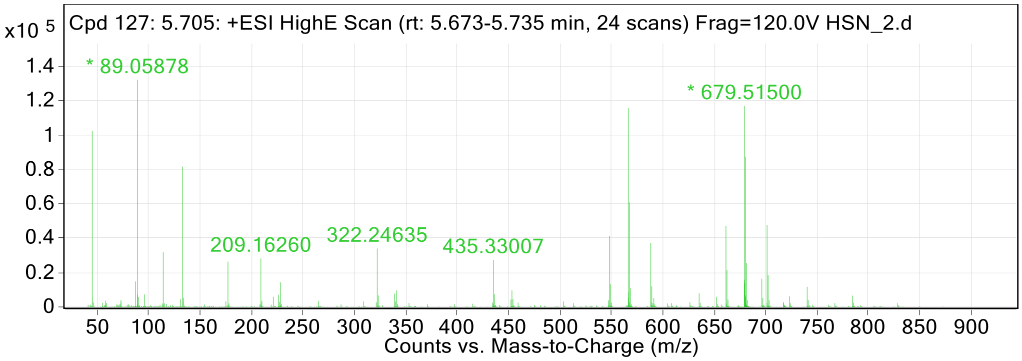

Peak List

m/z	z	Abund
45.0334	1	102591.98
89.05878	1	132170.61
133.08413	1	81768.57
548.4135	1	41345.94
566.42685	1	115911.84
567.42763	1	60873.87
661.49779	1	47227.13
679.515	1	117025.26
680.51293	1	87531.19
701.48951	1	47617.66

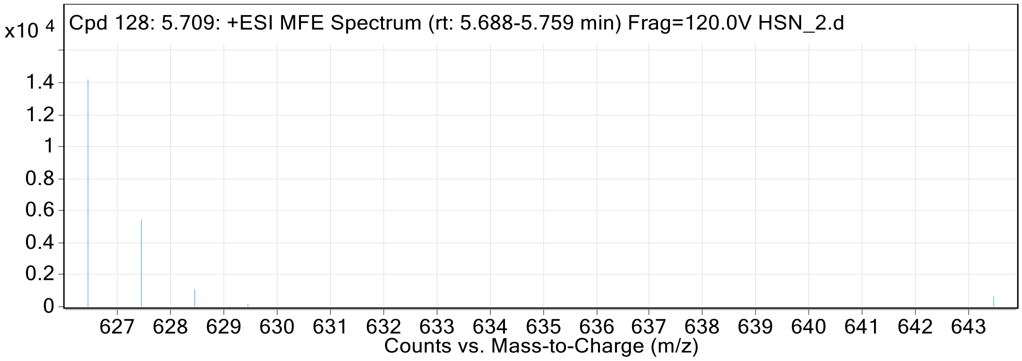

Peak List

m/z	z	Abund	Ion
626.44503	1	14211.83	(M+H)+
627.44763	1	5460.52	(M+H)+
628.45208	1	1082.95	(M+H)+
629.45092	1	176.87	(M+H)+
643.47028	1	642.37	(M+NH4)+

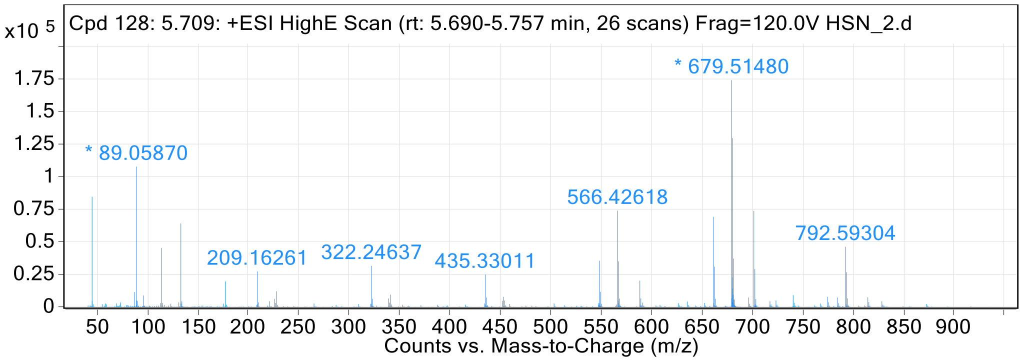

Peak List

m/z	z	Abund
45.03333		84612.95
89.0587	1	107804.79
114.08978	1	45295.2
133.08412	1	64038.8
566.42618	1	73925.02
661.49771	1	69090.05
679.5148	1	174333.81
680.51283	1	129679.03
701.48962	1	73674.33
792.59304	1	46178.72

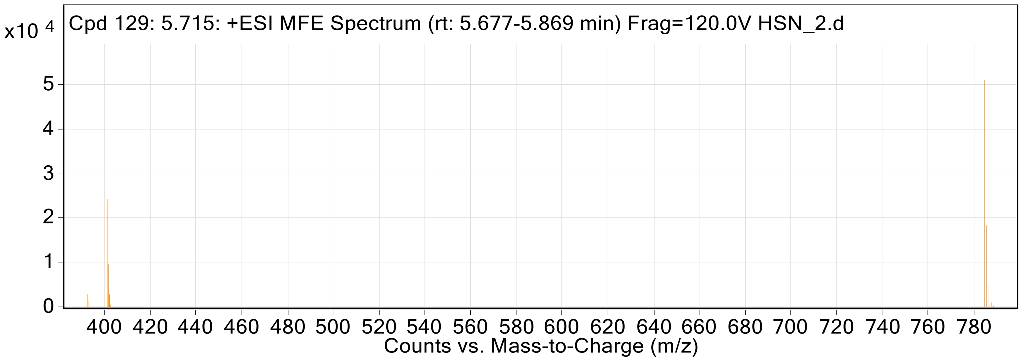

Peak List

m/z	z	Abund	Ion
392.74671	2	2869.21	(M+2H)+2
393.24503	2	1378.7	(M+2H)+2
401.2595	2	24276.88	(M+H5N)+2
401.76091	2	9558.7	(M+H5N)+2
402.26211	2	2811.58	(M+H5N)+2
402.76372	2	513.73	(M+H5N)+2
784.4858	1	51008.7	(M+H)+
785.48968	1	18315.99	(M+H)+
786.49231	1	5211.67	(M+H)+
787.49488	1	1041.16	(M+H)+

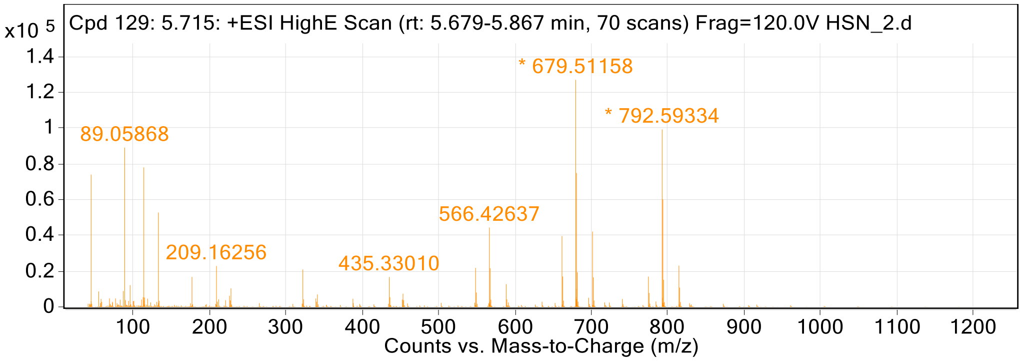

Peak List

m/z	z	Abund
45.03333		74046.02
89.05868	1	89132.47
114.08982	1	78083.13
133.0841	1	52794.91
566.42637	1	44407.16
679.51158	1	127062.54
680.51212	1	74929.68
701.48961	1	42147.74
792.59334	1	99325.2
793.59481	1	60135.85

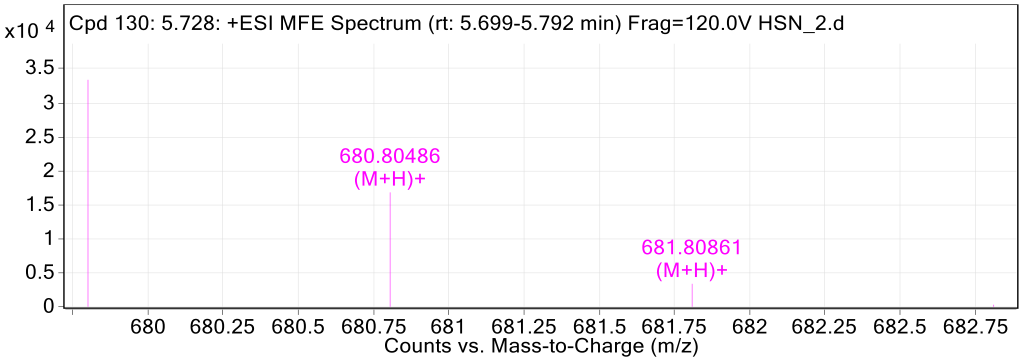

Peak List

m/z	z	Abund	Ion
679.8008	1	33365.89	(M+H)+
680.80486	1	16815.58	(M+H)+
681.80861	1	3380.01	(M+H)+
682.81128	1	302.33	(M+H)+

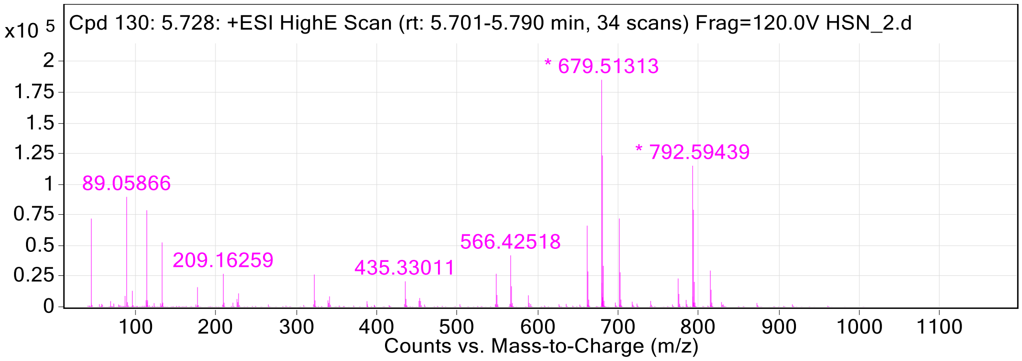

Peak List

m/z	z	Abund
45.03331		71950.85

89.05866	1	89659.16
114.08986	1	78726.27
133.08407	1	52487.69
661.49761	1	66183.88
679.51313	1	185063.53
680.51247	1	123401.02
701.48965	1	72004.06
792.59439	1	115041.69
793.59503	1	79140.07

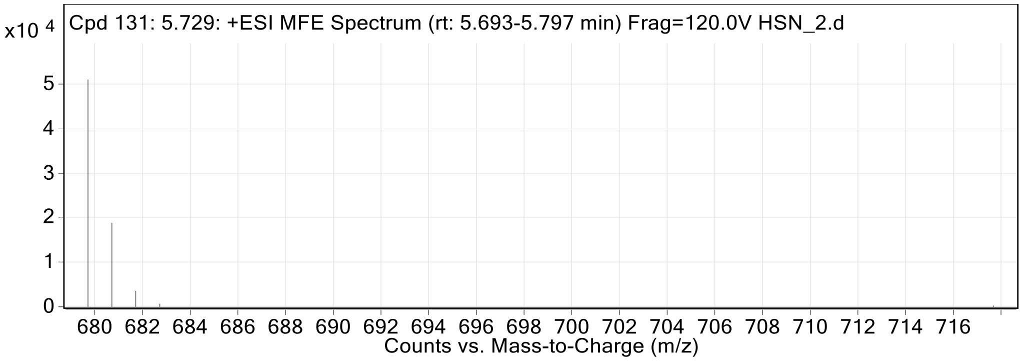

Peak List

m/z	z	Abund	Ion
679.73286	1	51018.26	(M+H)+
680.73591	1	18854.5	(M+H)+
681.73806	1	3574.35	(M+H)+
682.74521	1	696.27	(M+H)+
717.69594	1	276.92	(M+K)+

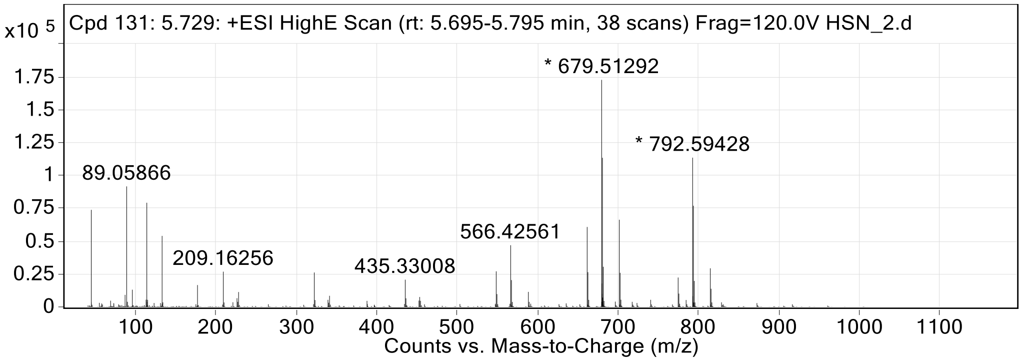

Peak List

m/z	z	Abund
45.03332		73798.91
89.05866	1	91640.69
114.08986	1	79216.8
133.08408	1	53994.67
661.4976	1	60779.66
679.51292	1	172651.53
680.51243	1	113382.16
701.48965	1	66291.84
792.59428	1	113495.5
793.59502	1	76931.73

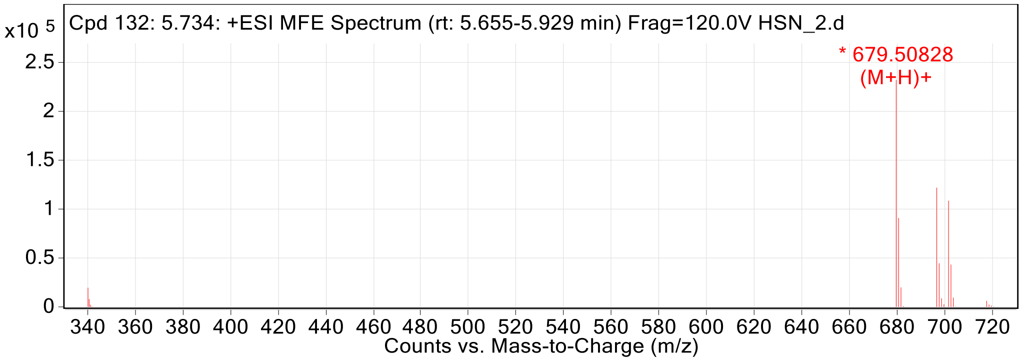

Peak List

m/z	z	Abund	Ion
340.25708	2	19532.16	(M+2H)+2
679.50828	1	231856.36	(M+H)+
680.51054	1	90834	(M+H)+
681.51366	1	19925.44	(M+H)+
696.53371	1	121744.27	(M+NH4)+
697.53617	1	44580.76	(M+NH4)+
698.54004	1	8762.16	(M+NH4)+
701.48932	1	108479.83	(M+Na)+
702.49251	1	43186.86	(M+Na)+
703.49522	1	9496.93	(M+Na)+

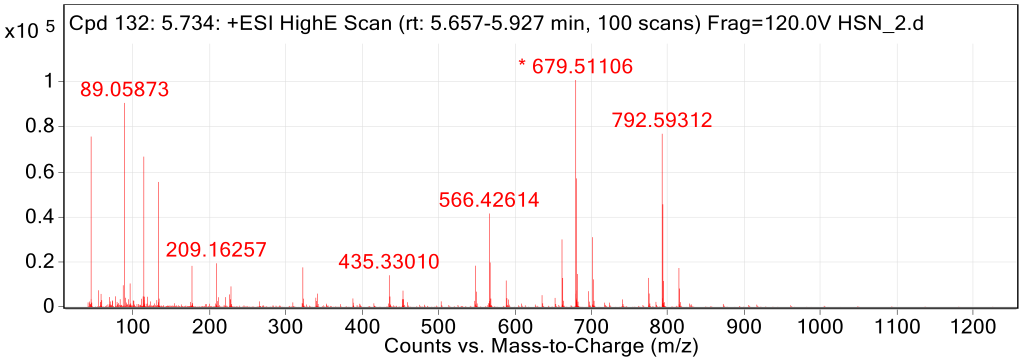

Peak List

m/z	z	Abund
45.03337		75663.8
89.05873	1	90618.55
114.0898	1	66745.25
133.08412	1	55483.9
566.42614	1	41463.98
679.51106	1	100806.16
680.51198	1	57064.23
701.48953	1	30927.95
792.59312	1	76910.51
793.59477	1	45542.53

Qualitative Analysis Report

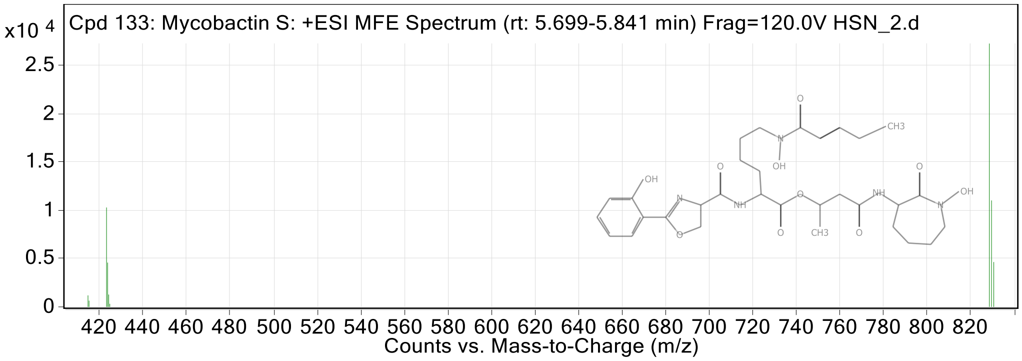

Peak List

m/z	z	Abund	Name	Formula	Ion	Score (DB)	Hits (DB)
414.75986	2	1191.11			(M+2H)+2		
415.25113	2	645.45			(M+2H)+2		
423.27266	2	10296.72			(M+H5N)+2		
423.77425	2	4587.59			(M+H5N)+2		
424.27551	2	1277.5			(M+H5N)+2		
424.77428	2	319.98			(M+H5N)+2		
828.51149	1	27294.27	Mycobactin S	C44 H69 N5 O10	(M+H)+	74.95	1
829.51555	1	11026.64		C44 H69 N5 O10	(M+H)+		
830.53674	1	4646.73		C44 H69 N5 O10	(M+H)+		

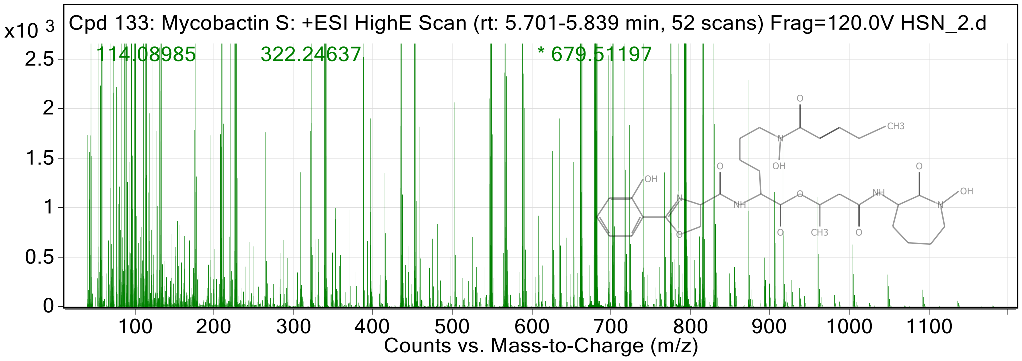

Peak List

m/z	z	Abund
45.03331		69300.05
89.05865	1	83342.99
114.08985	1	87364.01
133.08408	1	48415.46
661.49753	1	50683.88
679.51197	1	156429.09
680.51223	1	95095.13
701.48961	1	54824.91
792.59352	1	122689.99
793.59486	1	75795.53

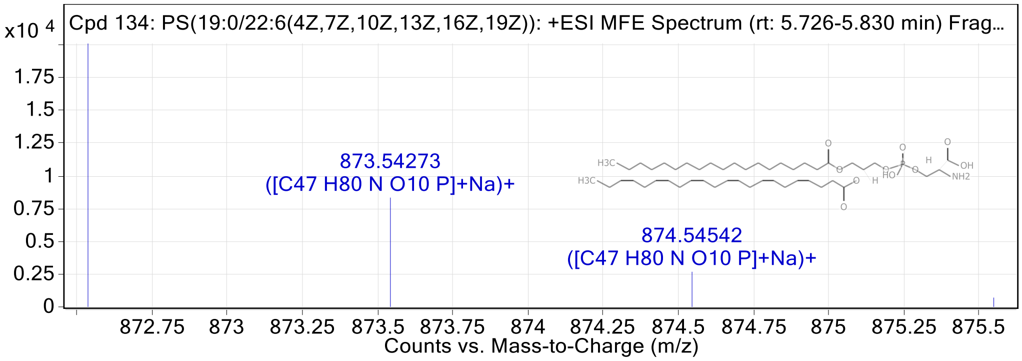

Peak List

m/z	z	Abund	Name	Formula	Ion	Score (DB)	Hits (DB)
872.5375	1	20120.35	PS(19:0/22:6(4Z,7Z,10Z,13Z,16Z,19Z))	C47 H80 N O10 P	(M+Na)+	81.23	2
873.54273	1	8344.35		C47 H80 N O10 P	(M+Na)+		
874.54542	1	2682.2		C47 H80 N O10 P	(M+Na)+		
875.54842	1	708.25		C47 H80 N O10 P	(M+Na)+		

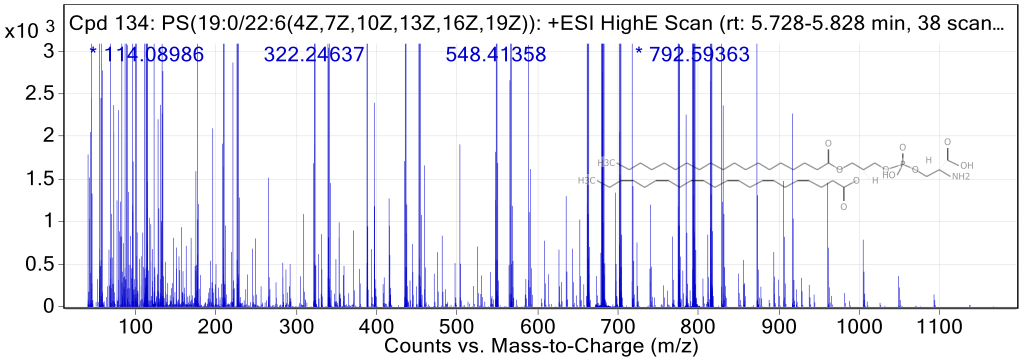

Peak List

m/z	z	Abund
45.03329		62717.58
89.05863	1	74679.2
114.08986	1	103025.04
133.08406	1	43288.34
661.49751	1	49007.27
679.51168	1	156436.59
680.51221	1	92319.67
701.4897	1	56196.47
792.59363	1	158112.38
793.59486	1	99093.74

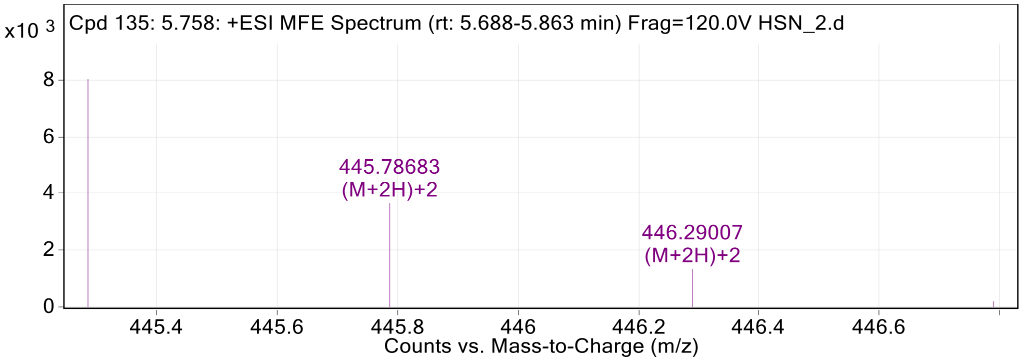

Peak List

m/z	z	Abund	Ion

445.28517	2	8024.33	(M+2H)+2
445.78683	2	3653.41	(M+2H)+2
446.29007	2	1339.66	(M+2H)+2
446.79059	2	205.34	(M+2H)+2

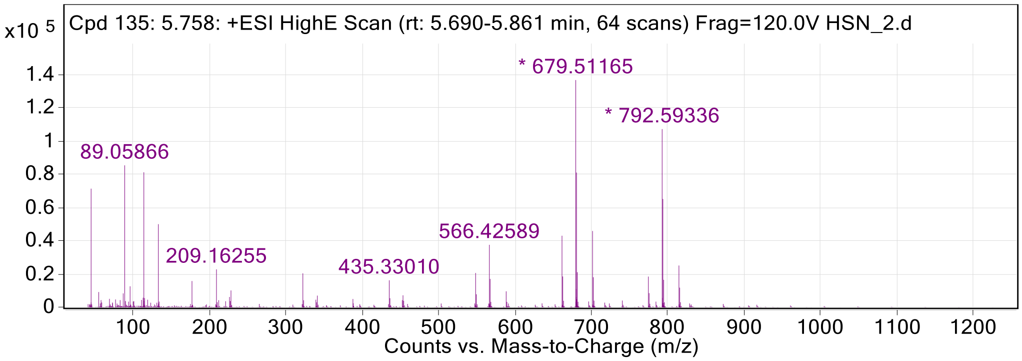

Peak List

m/z	z	Abund
45.03332		71363.26
89.05866	1	85410.01
114.08983	1	81232.69
133.0841	1	49960
661.49752	1	42991.74
679.51165	1	136793.91
680.51215	1	81052.47
701.48961	1	45835.93
792.59336	1	107240.5
793.59482	1	65139.11

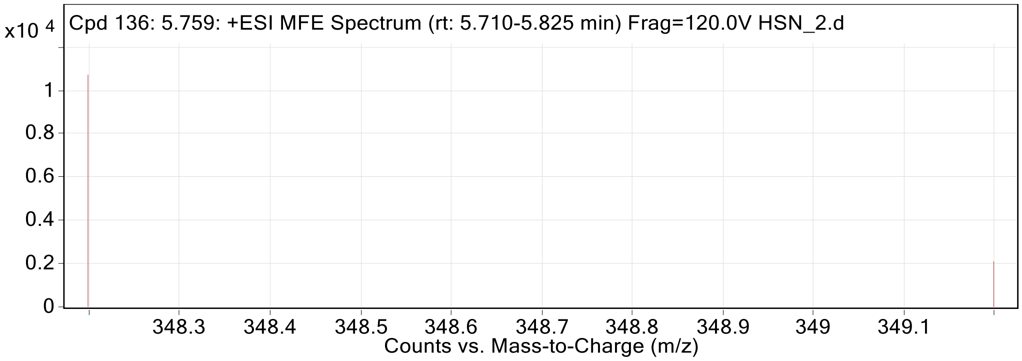

Peak List

m/z	z	Abund	Ion
348.19889	1	10728.33	(M+H)+
349.19922	1	2106.62	(M+H)+

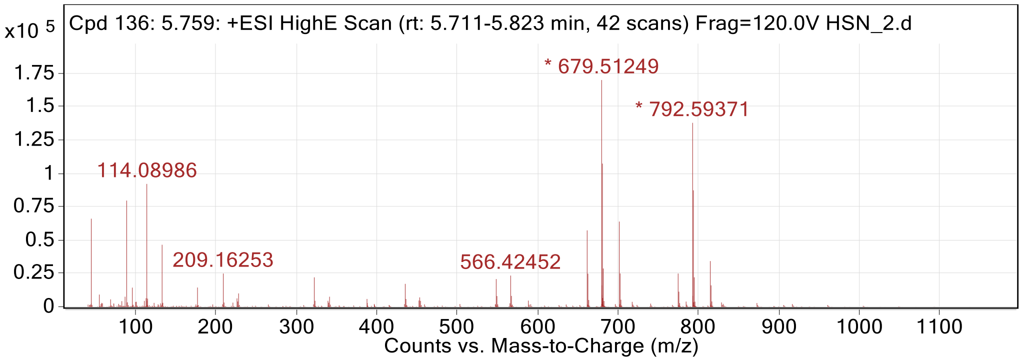

Qualitative Analysis Report

Peak List

m/z	z	Abund
45.03329		65960.54
89.05863	1	79531.41
114.08986	1	92012.41
133.08405	1	46430.2
661.49757	1	57204.29
679.51249	1	169844.83
680.51237	1	107230.41
701.48961	1	63810.05
792.59371	1	137766.33
793.59487	1	87212.49

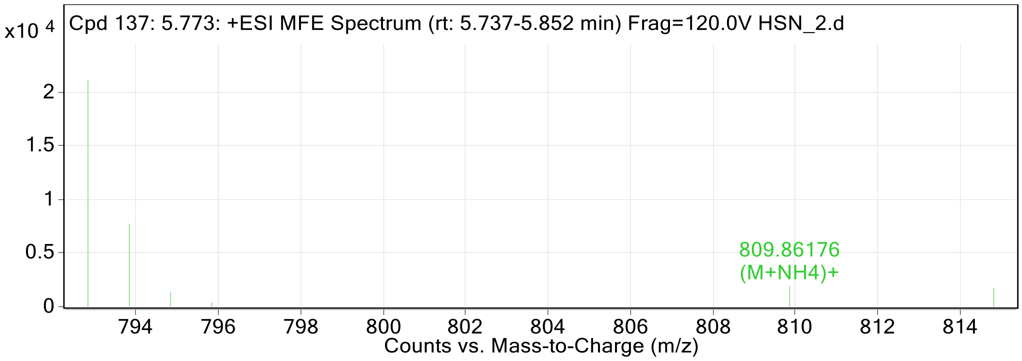

Peak List

m/z	z	Abund	Ion
792.8337	1	21188.8	(M+H)+
793.83644	1	7741.76	(M+H)+
794.83927	1	1418.28	(M+H)+
795.83702	1	403.04	(M+H)+
809.86176	1	1934.16	(M+NH4)+
814.81572	1	1782.77	(M+Na)+

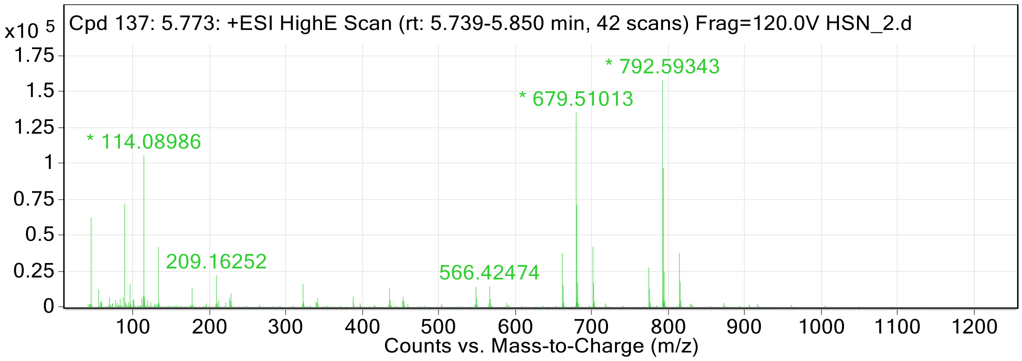

Peak List

m/z	z	Abund
45.0333		62182.35
89.05863	1	72014.84
114.08986	1	105523.89
133.08408	1	41581.8
679.51013	1	135368.14
680.51164	1	71075.61
701.48967	1	41852.43
792.59343	1	157930.92
793.59483	1	96586.44
814.57323	1	37393.02

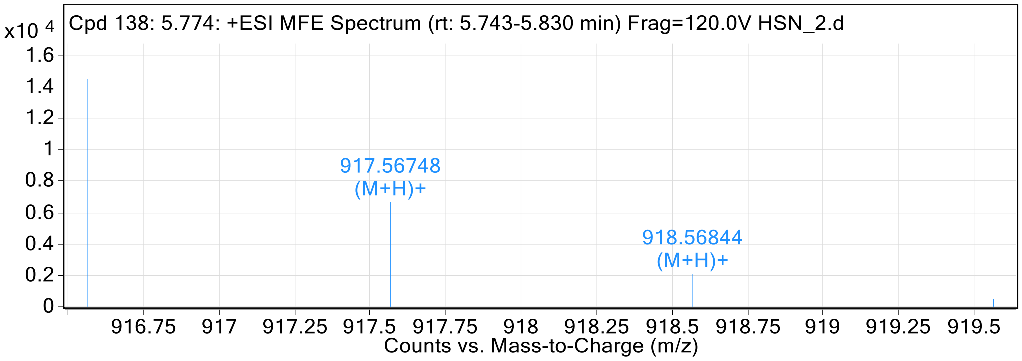

Peak List

m/z	z	Abund	Ion
916.56442	1	14509.12	(M+H)+
917.56748	1	6662.62	(M+H)+
918.56844	1	2092.77	(M+H)+
919.5648	1	490.76	(M+H)+

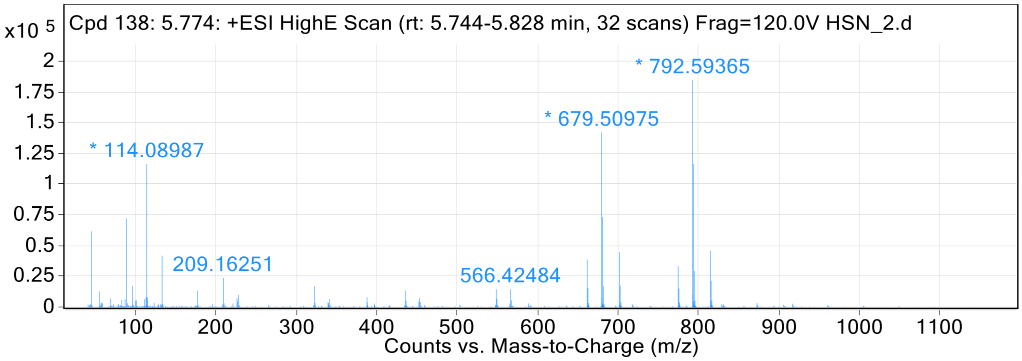

Peak List

m/z	z	Abund
45.0333		61514.24
89.05863	1	72093.91
114.08987	1	116368.66
133.08407	1	41778.24
679.50975	1	141963.17
680.5114	1	73215.58
701.48968	1	44823.31
792.59365	1	184630.59
793.59485	1	116178.34
814.57319	1	45761.61

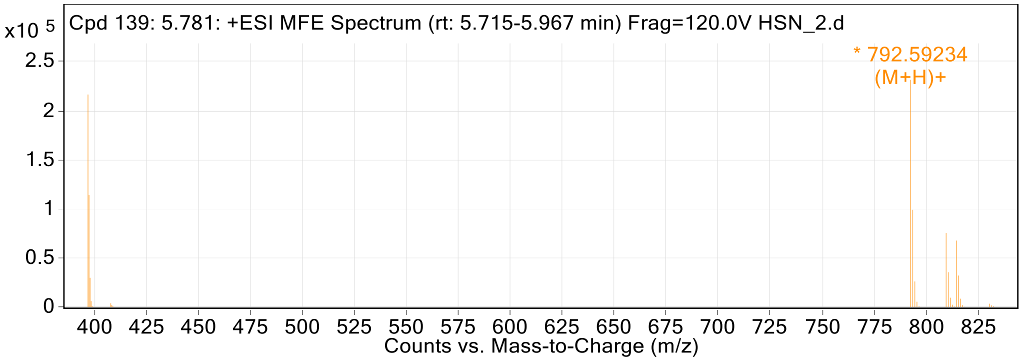

Peak List

m/z	z	Abund	Ion
396.79935	2	216483.78	(M+2H)+2

397.3004	2	114088.66	(M+2H)+2
397.80171	2	29635.89	(M+2H)+2
792.59234	1	231448.47	(M+H)+
793.59399	1	99010.98	(M+H)+
794.59809	1	25862.49	(M+H)+
809.61813	1	75397.09	(M+NH4)+
810.62096	1	35223.21	(M+NH4)+
814.57334	1	67562.16	(M+Na)+
815.57652	1	31901.2	(M+Na)+

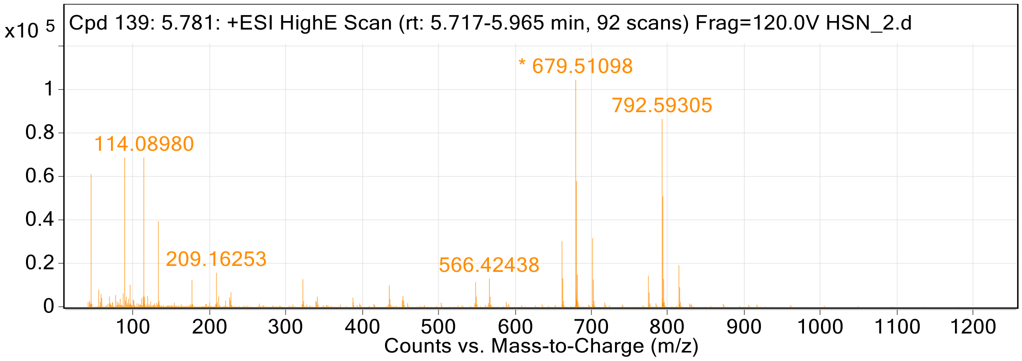

Peak List

m/z	z	Abund
45.03328		61258.49
89.0586	1	68842.56
114.0898	1	68848.72
133.08408	1	39514.48
661.49749	1	30319.86
679.51098	1	104717.01
680.512	1	58008
701.48959	1	31671.87
792.59305	1	86648.63
793.59475	1	50949.99

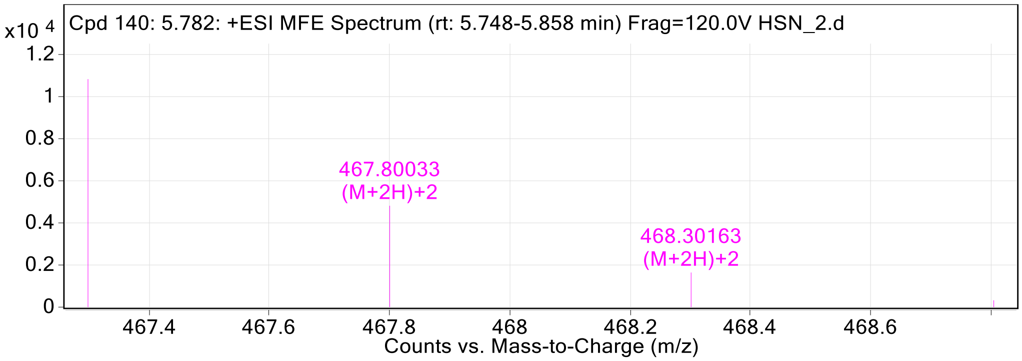

Peak List

m/z	z	Abund	Ion
467.29848	2	10811.7	(M+2H)+2
467.80033	2	4806.29	(M+2H)+2
468.30163	2	1640.45	(M+2H)+2
468.80503	2	322.28	(M+2H)+2

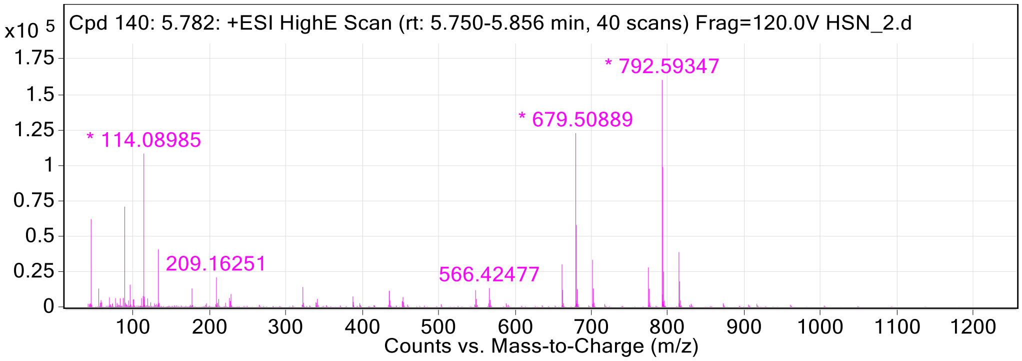

Peak List

m/z	z	Abund
45.0333		61919.52
89.05861	1	70753.91
114.08985	1	108238.23
133.08408	1	40661.84
679.50889	1	122665.81
680.51107	1	57795.68
701.4896	1	33202.64
792.59347	1	160291.48
793.59481	1	98752.5
814.57319	1	38688.77

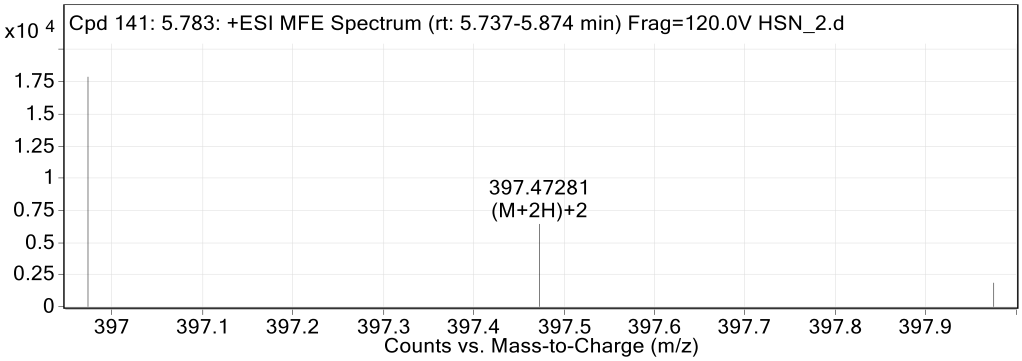

Peak List

m/z	z	Abund	Ion
396.97325	2	17891.39	(M+2H)+2
397.47281	2	6442.53	(M+2H)+2
397.97548	2	1867.22	(M+2H)+2

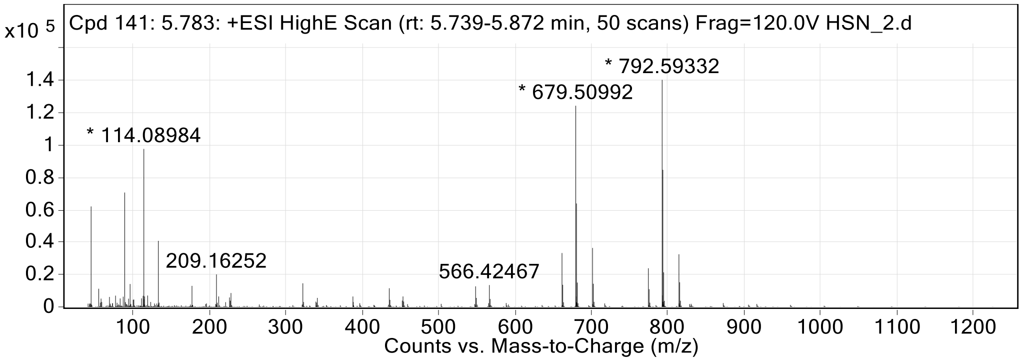

Peak List

m/z	z	Abund
45.0333		62108.83
89.05863	1	70715.41

114.08984	1	97671.51
133.08408	1	40827.41
661.49733	1	33305.62
679.50992	1	124371.91
680.51156	1	63938.16
701.48966	1	36445.75
792.59332	1	140366.34
793.5948	1	84772.44

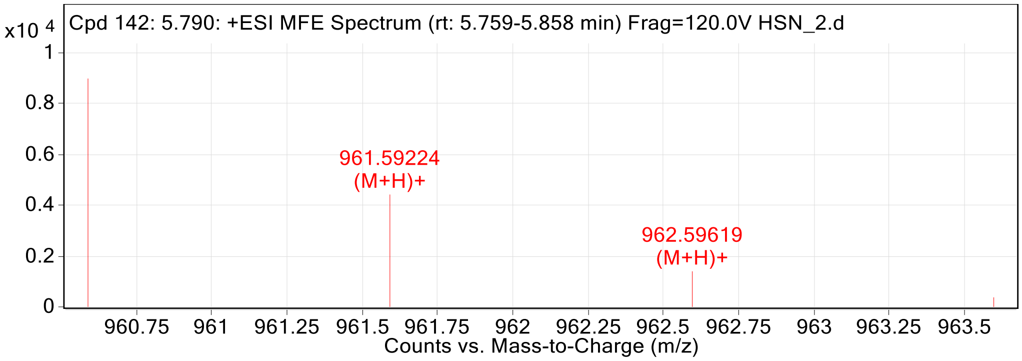

Peak List

m/z	z	Abund	Ion
960.58971	1	8995.92	(M+H)+
961.59224	1	4426.78	(M+H)+
962.59619	1	1401.35	(M+H)+
963.59732	1	375.34	(M+H)+

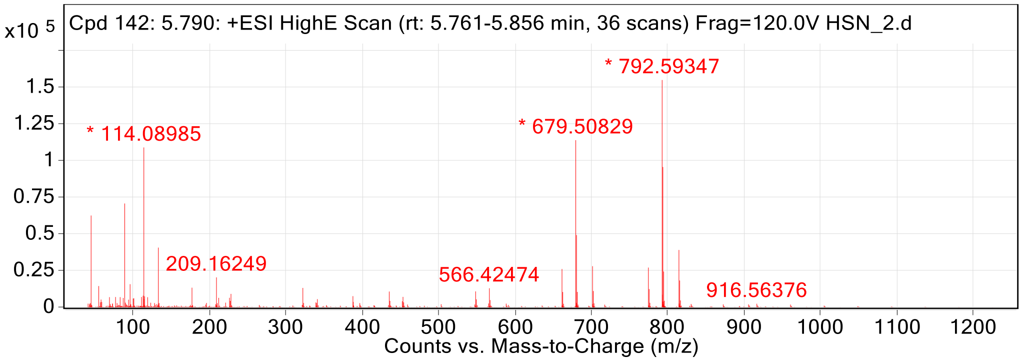

Peak List

m/z	z	Abund
45.0333		62315.17
89.05861	1	70498.16
114.08985	1	108672.23
133.08407	1	40454.05
679.50829	1	113621.54
680.5109	1	48984.4
701.48957	1	27810.21
792.59347	1	154593.06
793.59481	1	95356.88
814.57321	1	38851.41

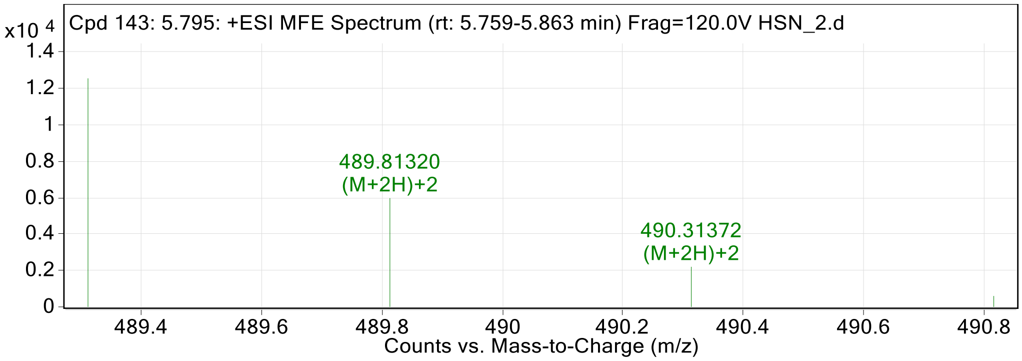

Peak List

m/z	z	Abund	Ion
489.31175	2	12563.31	(M+2H)+2
489.8132	2	5977.49	(M+2H)+2
490.31372	2	2203.32	(M+2H)+2
490.81612	2	599.12	(M+2H)+2

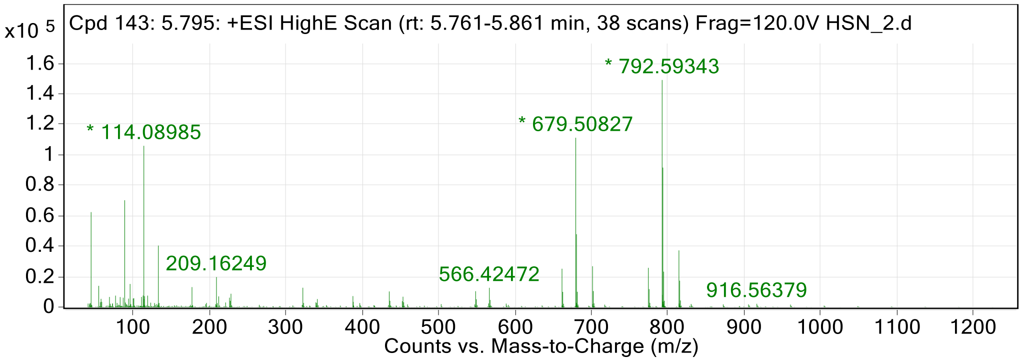

Peak List

m/z	z	Abund
45.0333		62297.68
89.05861	1	70087.27
114.08985	1	105821.48
133.08407	1	40327.14
679.50827	1	111108.73
680.51087	1	47781.7
701.48959	1	26788.59
792.59343	1	149019.58
793.5948	1	91530.52
814.57322	1	37176.39

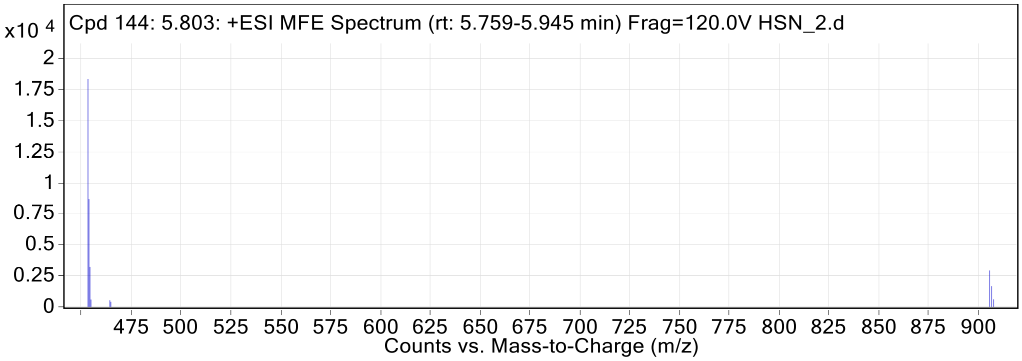

Peak List

m/z	z	Abund	Ion
453.34073	2	18355.5	(M+2H)+2

453.84239	2	8668.18	(M+2H)+2
454.34376	2	3224.23	(M+2H)+2
454.84596	2	586.82	(M+2H)+2
464.33005	2	531.33	(M+HNa)+2
464.83382	2	407.81	(M+HNa)+2
905.67467	1	2930	(M+H)+
906.67728	1	1669.82	(M+H)+
907.68512	1	601.47	(M+H)+

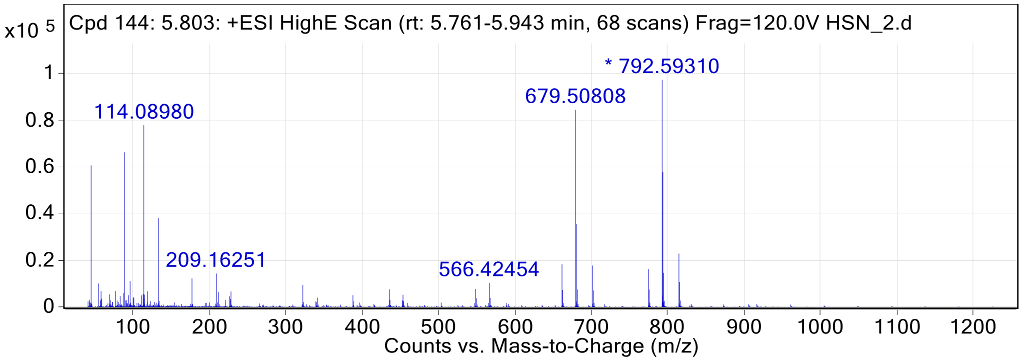

Peak List

m/z	z	Abund
45.03329		60602.64
89.0586	1	66219.77
114.0898	1	77829.77
133.08408	1	37945.07
661.4971	1	18201.38
679.50808	1	84488.44
680.51078	1	35511.51
792.5931	1	97309.41
793.59474	1	57700.07
814.57323	1	22831.17

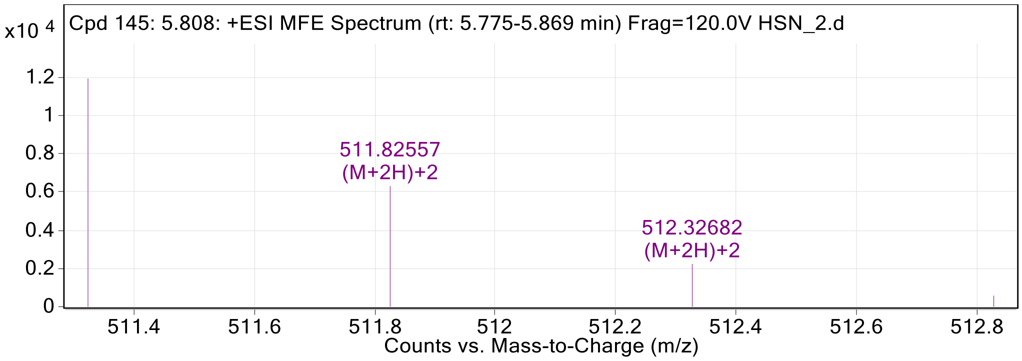

Peak List

m/z	z	Abund	Ion
511.32414	2	11941.08	(M+2H)+2
511.82557	2	6320.44	(M+2H)+2
512.32682	2	2235.72	(M+2H)+2
512.82718	2	581.4	(M+2H)+2

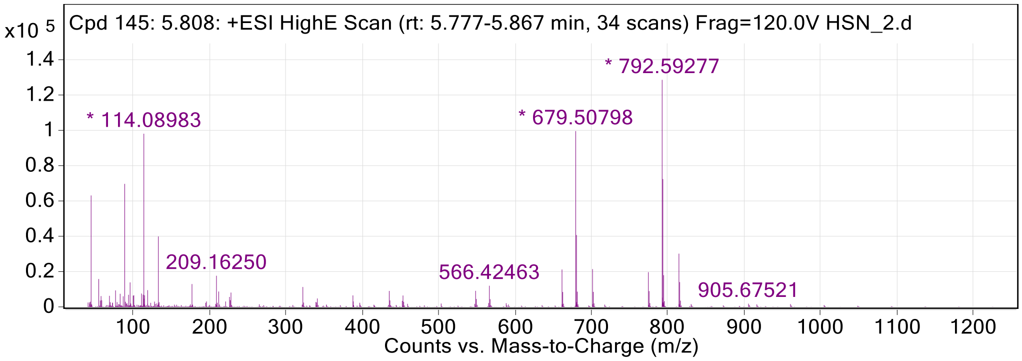

Peak List

m/z	z	Abund
45.03331		63161.84
89.05862	1	69724.9
114.08983	1	98169.96
133.08408	1	39925.98
679.50798	1	99655.19
680.51082	1	40724.79
701.48949	1	21410.12
792.59277	1	128687.77
793.59465	1	72454.22
814.57319	1	30173.33

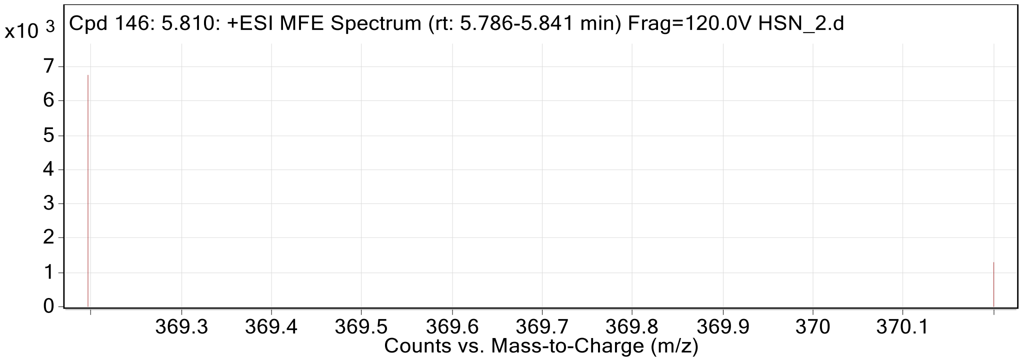

Peak List

m/z	z	Abund	Ion
369.19655	1	6762.3	(M+H)+
370.20019	1	1300.41	(M+H)+

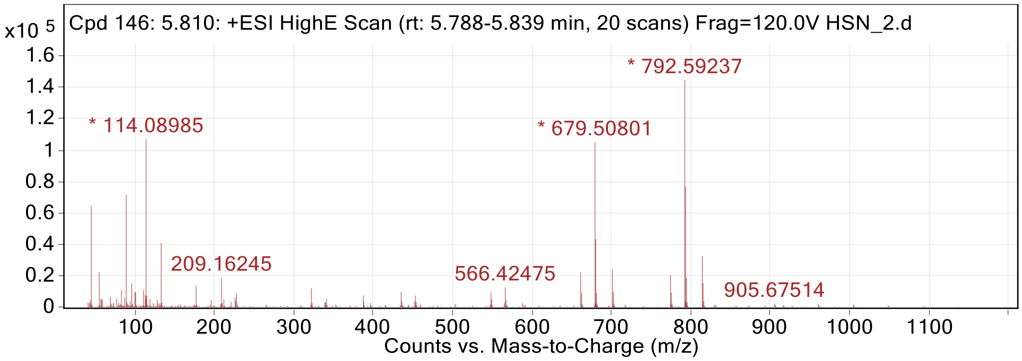

Peak List

m/z	z	Abund
45.03331		64321.88
89.05863	1	71486.39
114.08985	1	107111.27

133.0841	1	40827.54
679.50801	1	105175.36
680.51087	1	43153.24
701.48942	1	23972.15
792.59237	1	144757.14
793.59452	1	76498.27
814.5732	1	32538.66

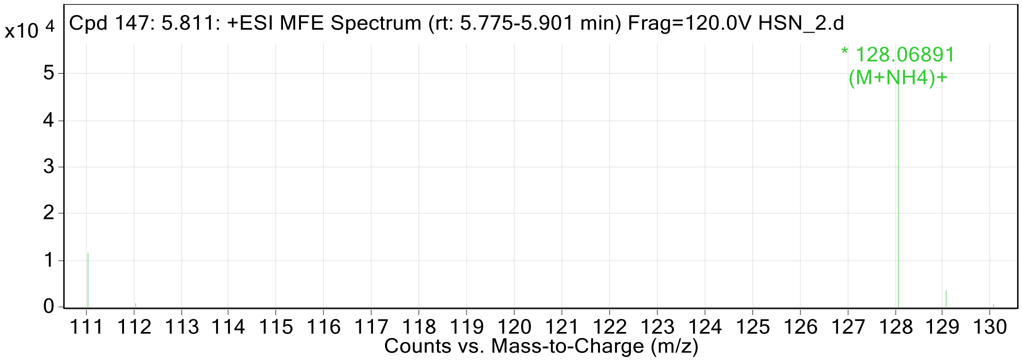

Peak List

m/z	z	Abund	Ion
111.0426	1	11621.95	(M+H)+
112.04562	1	768.17	(M+H)+
128.06891	1	48723.37	(M+NH4)+
129.0723	1	3527.41	(M+NH4)+
130.07347	1	563.99	(M+NH4)+

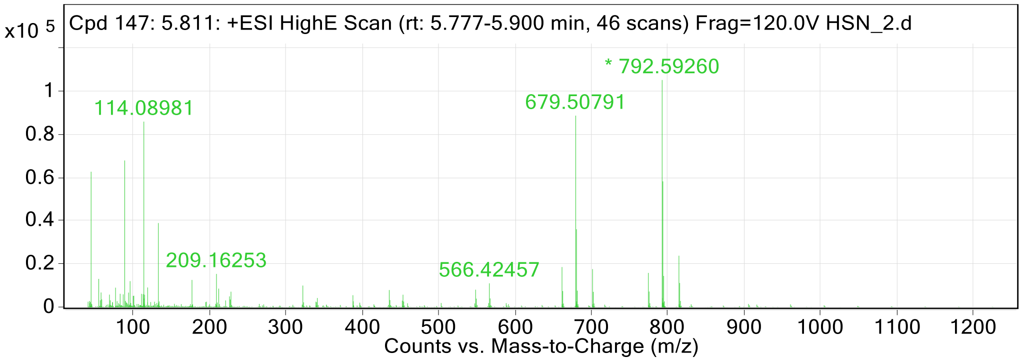

Peak List

m/z	z	Abund
45.0333		62722.05
89.05861	1	67932.76
114.08981	1	85949.48
133.08407	1	38924.8
661.4971	1	18536.32
679.50791	1	88724.02
680.51078	1	36037.16
792.5926	1	105242.05
793.59462	1	58257.59
814.57319	1	23786.26

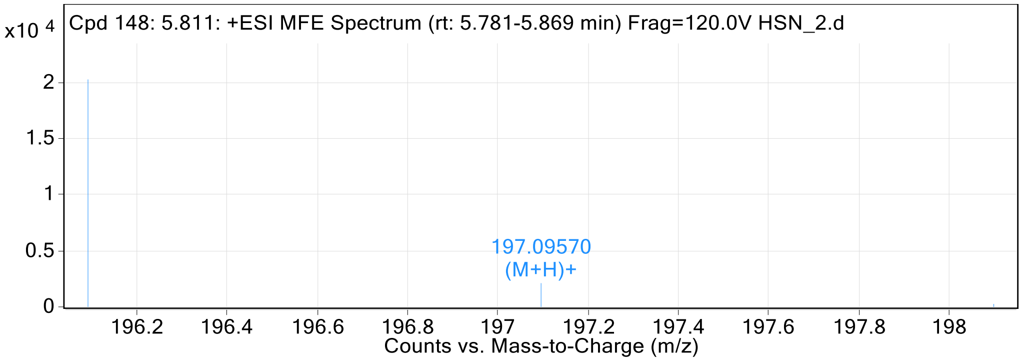

Peak List

m/z	z	Abund	Ion
196.09196	1	20267.13	(M+H)+
197.0957	1	2114.98	(M+H)+
198.0986	1	259.77	(M+H)+

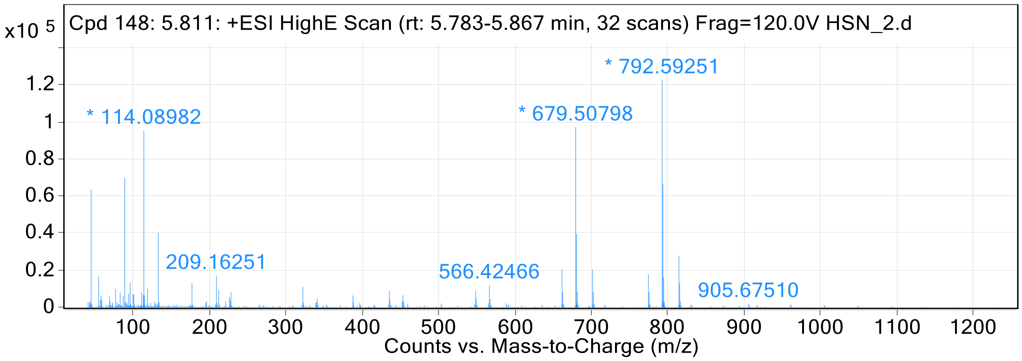

Peak List

m/z	z	Abund
45.03331		63418.93
89.05862	1	69740.92
114.08982	1	95258.6
133.08408	1	39931.51
679.50798	1	97050.38
680.51082	1	39567.3
701.48947	1	20343.8
792.59251	1	122504.2
793.59458	1	66216.5
814.5732	1	27607.67

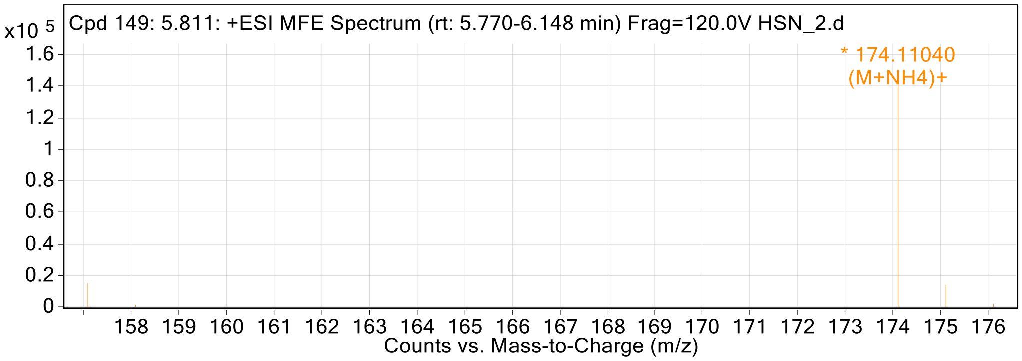

Peak List

m/z	z	Abund	Ion
157.08404	1	15008.94	(M+H)+
158.08747	1	1253.57	(M+H)+

174.1104	1	144171.22	(M+NH4)+
175.11353	1	14125.79	(M+NH4)+
176.11535	1	1753.76	(M+NH4)+

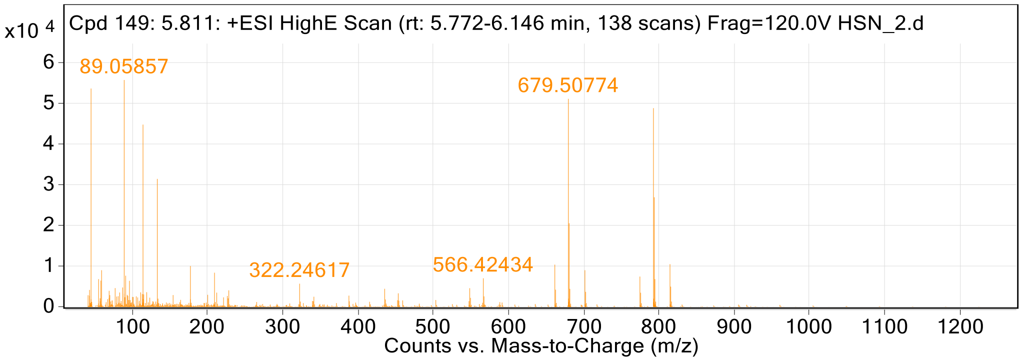

Peak List

m/z	z	Abund
45.03325		53708.24
89.05857	1	55805.16
114.08974	1	44826.77
133.08412	1	31460.34
661.497	1	10354.9
679.50774	1	51167.17
680.51066	1	20538.15
792.59246	1	48882.14
793.59455	1	26934.14
814.57311	1	10478.13

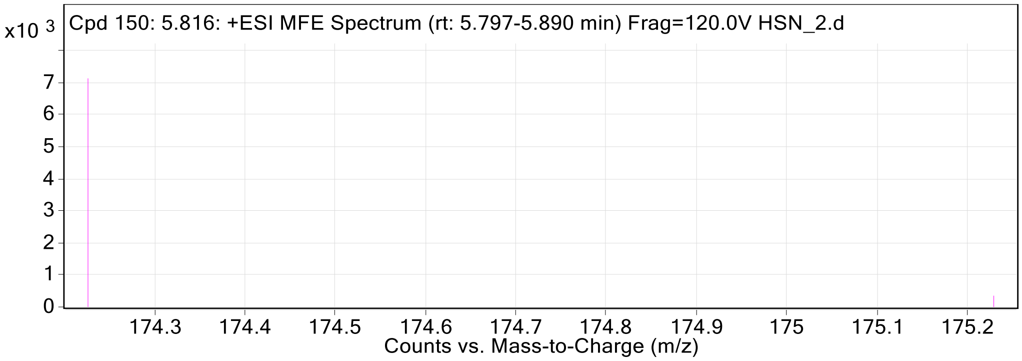

Peak List

m/z	z	Abund	Ion
174.22643	1	7127.09	(M+H)+
175.22921	1	351.01	(M+H)+

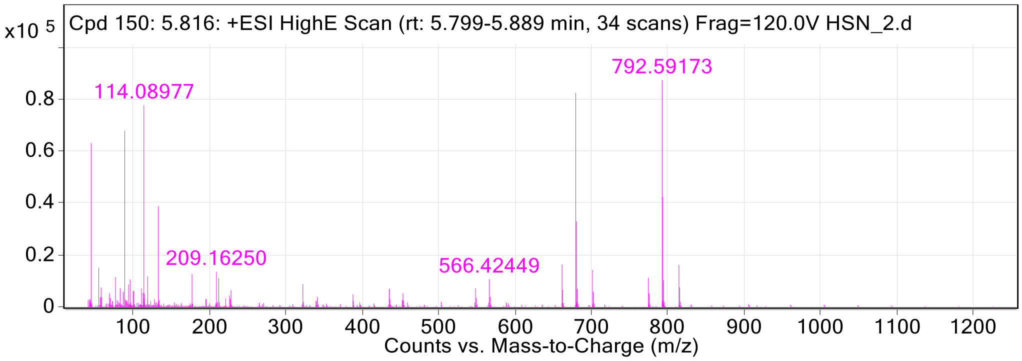

Peak List

m/z	z	Abund

45.0333		63116.17
89.05861	1	67863.91
114.08977	1	77714.22
133.08408	1	38775.7
661.49707	1	16323.54
679.50779	1	82464.23
680.51075	1	32923.13
792.59173	1	87378.24
793.59433	1	42348.21
814.57325	1	16169.14

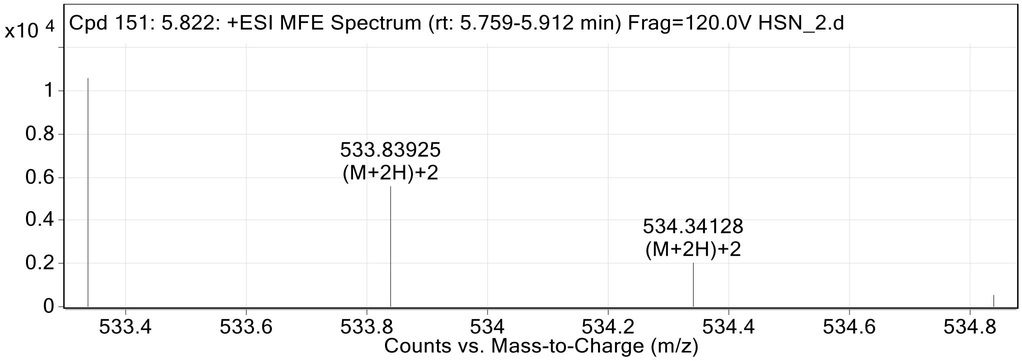

Peak List

m/z	z	Abund	Ion
533.33746	2	10587.05	(M+2H)+2
533.83925	2	5582.87	(M+2H)+2
534.34128	2	2038.84	(M+2H)+2
534.83943	2	544.08	(M+2H)+2

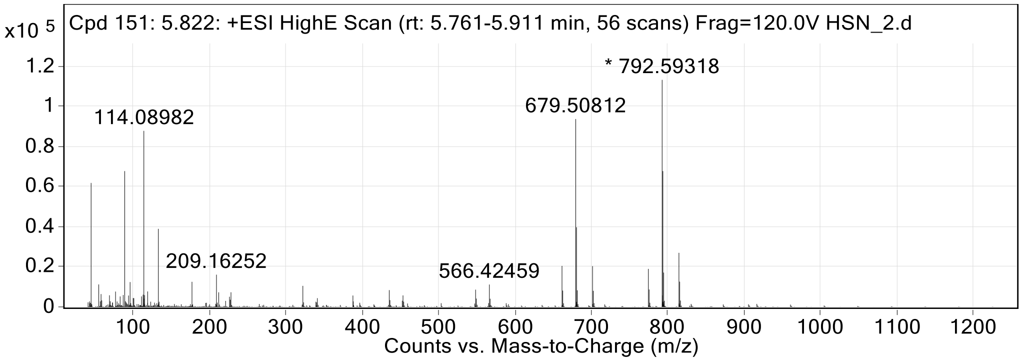

Peak List

m/z	z	Abund
45.0333		61778.54
89.0586	1	67699.73
114.08982	1	87750.46
133.08406	1	38909.88
661.4971	1	20441.36
679.50812	1	93637.74
680.5108	1	39663.72
792.59318	1	113166.76
793.59476	1	67701.95
814.57323	1	26960.28

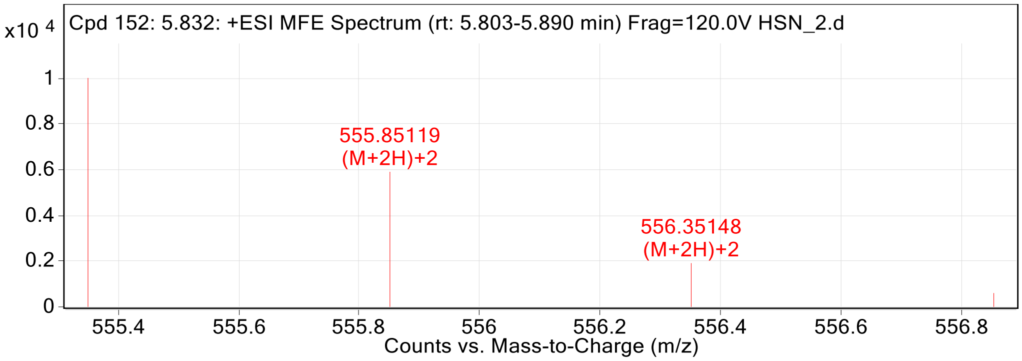

Peak List

m/z	z	Abund	Ion
555.34986	2	10015.99	(M+2H)+2
555.85119	2	5909.51	(M+2H)+2
556.35148	2	1915.1	(M+2H)+2
556.85385	2	604.85	(M+2H)+2

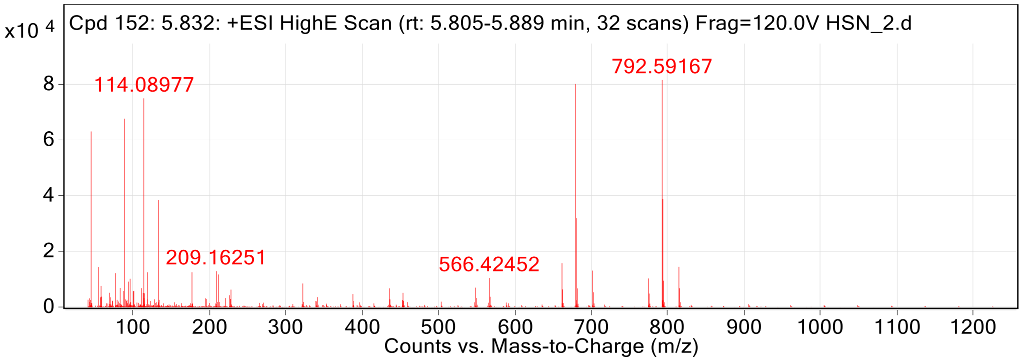

Peak List

m/z	z	Abund
45.0333		63053.61
89.05861	1	67659.36
114.08977	1	74946.76
133.08408	1	38489.02
661.49714	1	15744.04
679.50776	1	80103.02
680.51074	1	31867.38
792.59167	1	81533.13
793.59434	1	38752.68
814.5733	1	14450.68

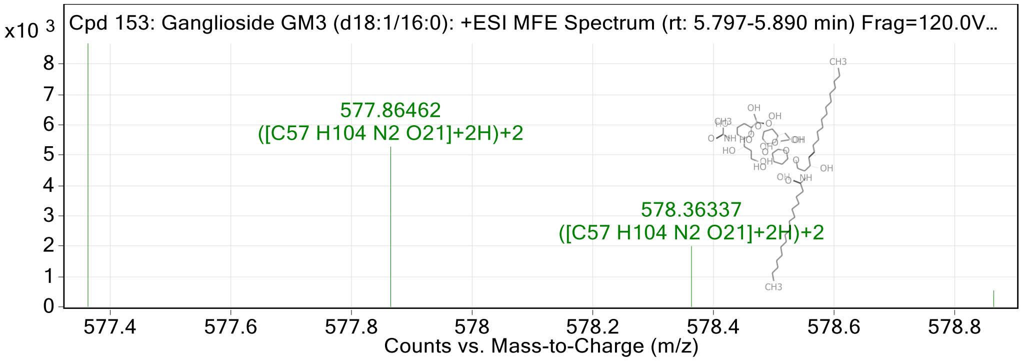

Peak List

m/z	z	Abund	Name	Formula	Ion	Score (DB)	Hits (DB)

577.36261	2	8680.72	Ganglioside GM3 (d18:1/16:0)	C57 H104 N2 O21	(M+2H)+2	92.02	2
577.86462	2	5278.15		C57 H104 N2 O21	(M+2H)+2		
578.36337	2	2000.32		C57 H104 N2 O21	(M+2H)+2		
578.86457	2	539.3		C57 H104 N2 O21	(M+2H)+2		

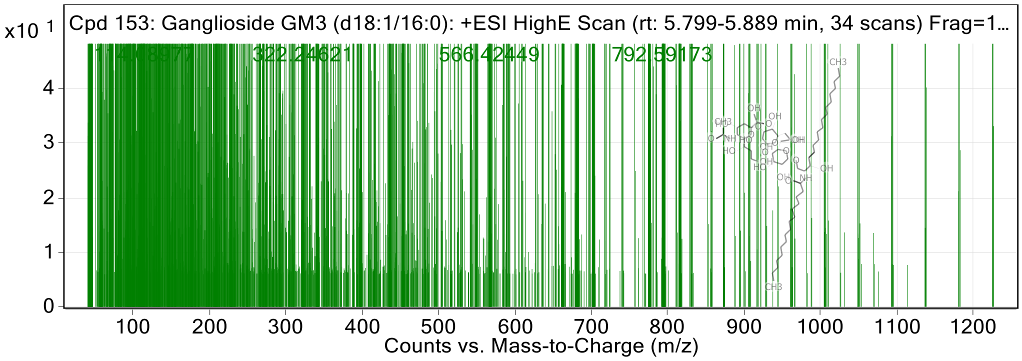

Peak List

m/z	z	Abund
45.0333		63116.17
89.05861	1	67863.91
114.08977	1	77714.22
133.08408	1	38775.7
661.49707	1	16323.54
679.50779	1	82464.23
680.51075	1	32923.13
792.59173	1	87378.24
793.59433	1	42348.21
814.57325	1	16169.14

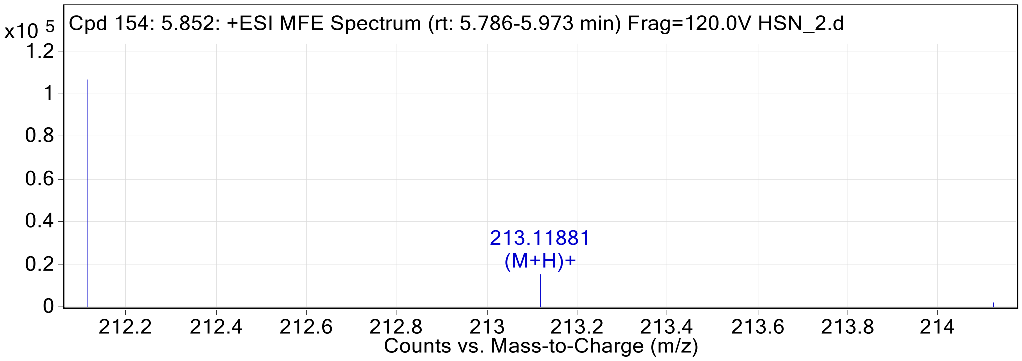

Peak List

m/z	z	Abund	Ion
212.11584	1	106760.11	(M+H)+
213.11881	1	15291.37	(M+H)+
214.1223	1	2051.25	(M+H)+

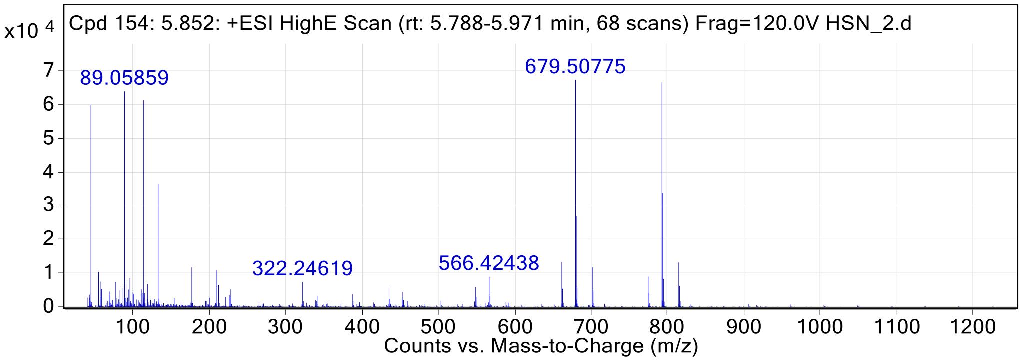

Peak List

m/z	z	Abund
45.03328		59691.79
89.05859	1	63877.29
114.08976	1	61198.65
133.0841	1	36324.03
661.49706	1	13280.43
679.50775	1	67231.8
680.51066	1	26850.17
792.59194	1	66532.56
793.59441	1	33715.65
814.57319	1	13146.69

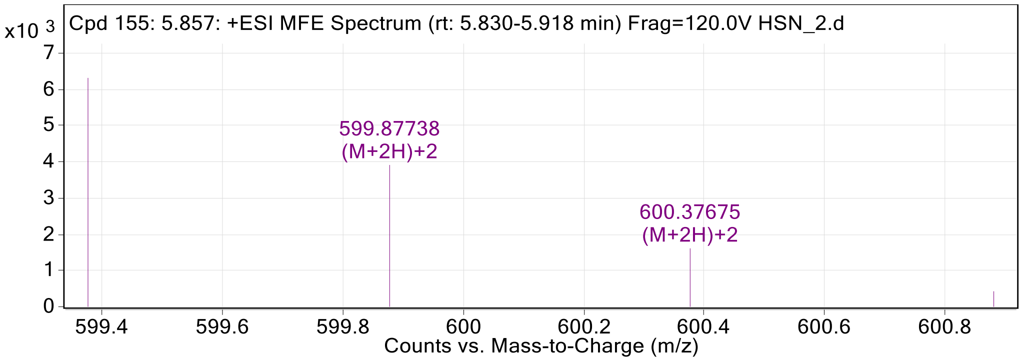

Peak List

m/z	z	Abund	Ion
599.37611	2	6313.28	(M+2H)+2
599.87738	2	3911.62	(M+2H)+2
600.37675	2	1611.55	(M+2H)+2
600.88131	2	427.56	(M+2H)+2

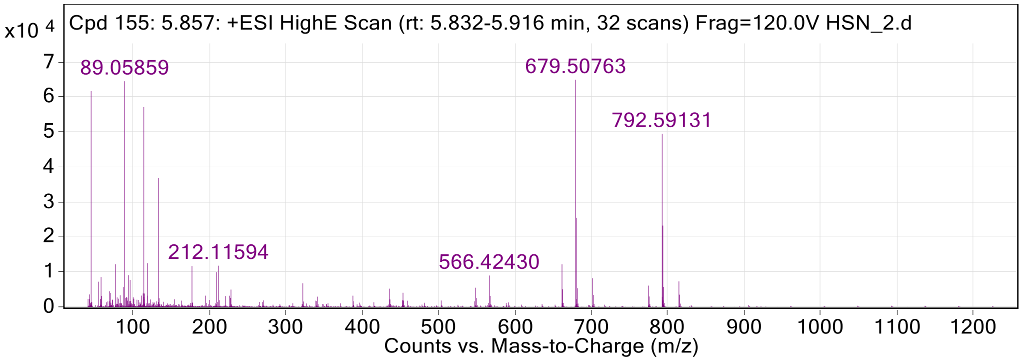

Peak List

m/z	z	Abund
45.0333		61635.07

77.03771		12169.86
89.05859	1	64474.23
114.08972	1	57062.16
119.05859		12477.89
133.08407	1	36754.13
679.50763	1	64875.21
680.51058	1	25470.64
792.59131	1	49452.55
793.59429	1	23217.99

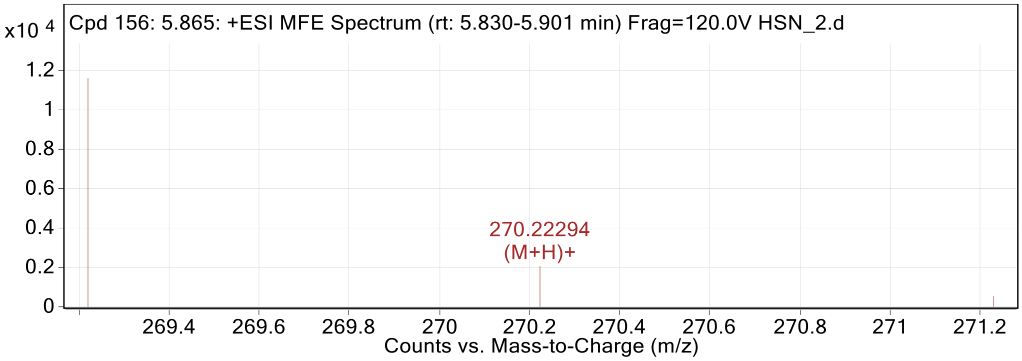

Peak List

m/z	z	Abund	Ion
269.21994	1	11631.39	(M+H)+
270.22294	1	2085.15	(M+H)+
271.22989	1	540.84	(M+H)+

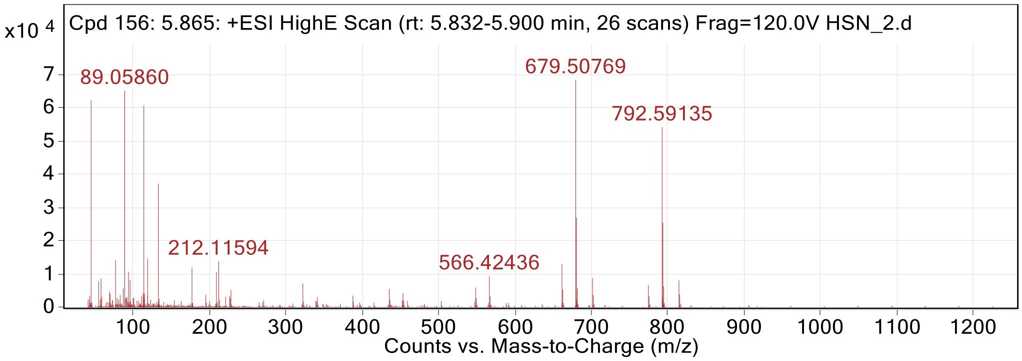

Peak List

m/z	z	Abund
45.0333		62307.17
77.0377		14186.23
89.0586	1	65111.71
114.08973	1	60686.56
119.0586		14606.92
133.08408	1	37162.03
679.50769	1	68332.66
680.51066	1	26896.19
792.59135	1	54127.97
793.59437	1	25371.13

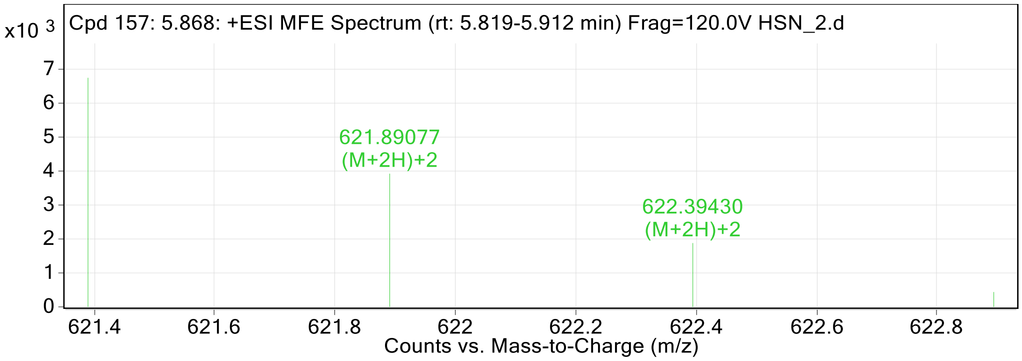

Peak List

m/z	z	Abund	Ion
621.38953	2	6759.1	(M+2H)+2
621.89077	2	3931.36	(M+2H)+2
622.3943	2	1883.06	(M+2H)+2
622.89425	2	438.33	(M+2H)+2

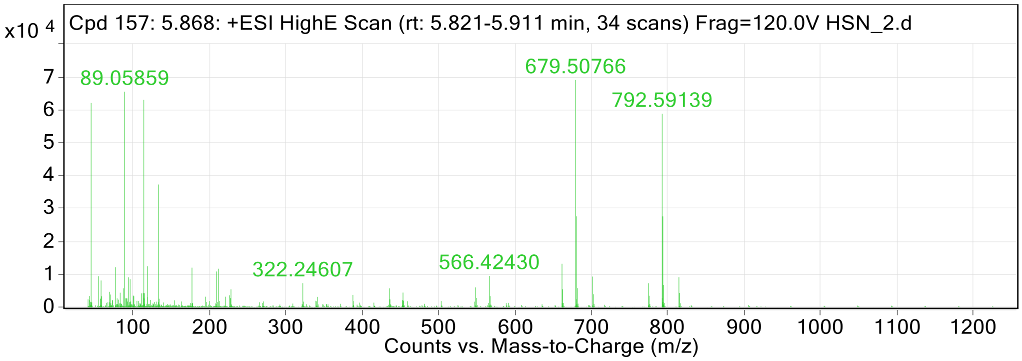

Peak List

m/z	z	Abund
45.0333		62103.88
89.05859	1	65548.93
114.08974	1	63041.21
119.05862		12402.88
133.08407	1	37285.67
661.49706	1	13170.55
679.50766	1	69101.7
680.51062	1	27593.77
792.59139	1	58847.49
793.59433	1	27572.89

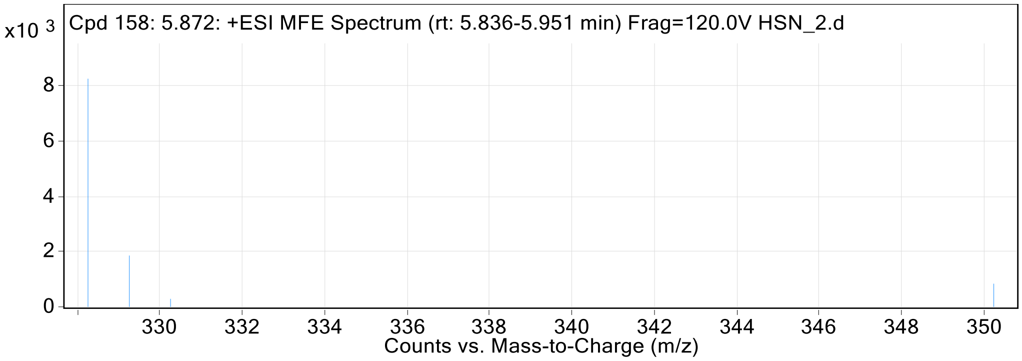

Peak List

m/z	z	Abund	Ion
328.25686	1	8267.36	(M+H)+

329.26022	1	1859.44	(M+H)+
330.2604	1	290.27	(M+H)+
350.23651	1	839.39	(M+Na)+

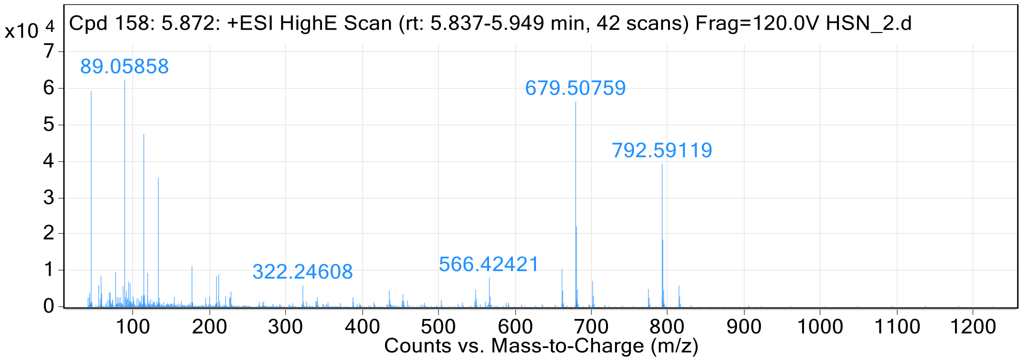

Peak List

m/z	z	Abund
45.03327		59343.17
89.05858	1	62327.83
114.08969	1	47445.13
133.08409	1	35422.55
177.10988		11275.35
661.49706	1	10461.06
679.50759	1	56385.38
680.5105	1	22112.51
792.59119	1	39246.24
793.59423	1	18469.65

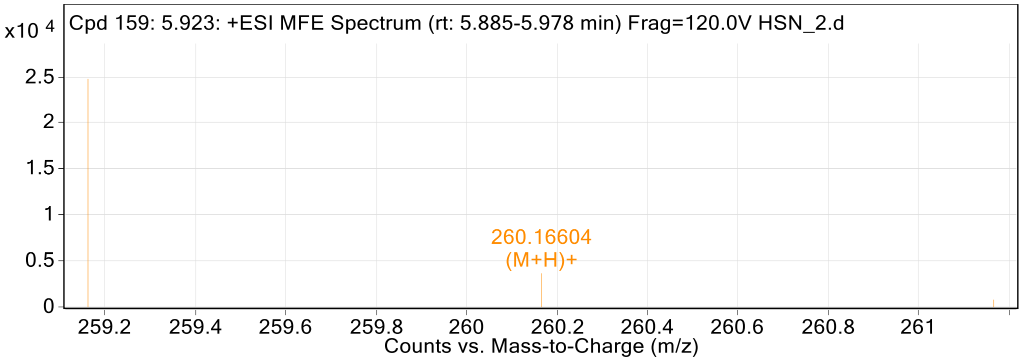

Peak List

m/z	z	Abund	Ion
259.16239	1	24782.82	(M+H)+
260.16604	1	3647.01	(M+H)+
261.16591	1	791.42	(M+H)+

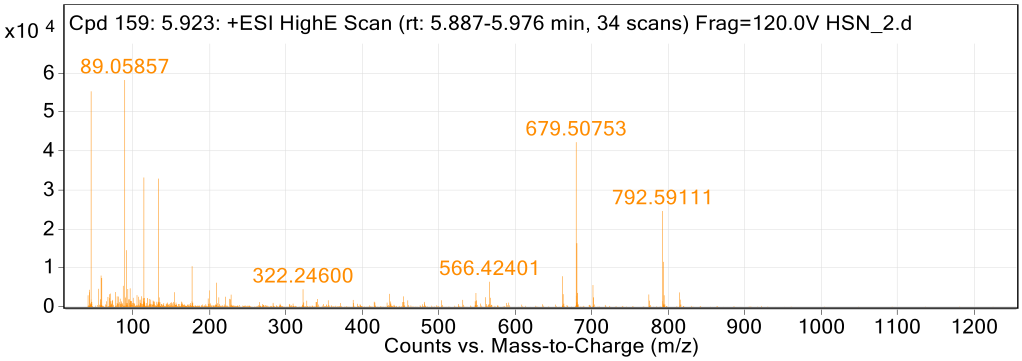

Peak List

m/z	z	Abund
45.03324		55313.3
89.05857	1	58214.7
91.05307		14525.15
114.08962	1	33187.97
133.08413	1	32903.45
177.10986		10409.06
679.50753	1	42254.95
680.51045	1	16317.99
792.59111	1	24588.14
793.59434	1	11523.45

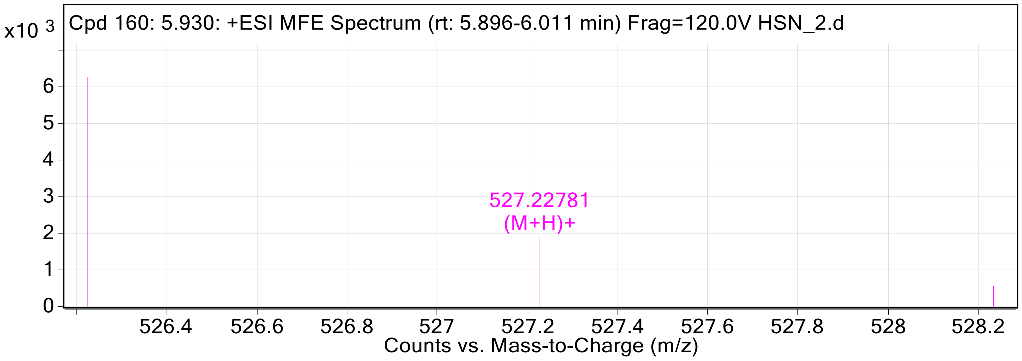

Peak List

m/z	z	Abund	Ion
526.22522	1	6253.49	(M+H)+
527.22781	1	1905.48	(M+H)+
528.23364	1	564.92	(M+H)+

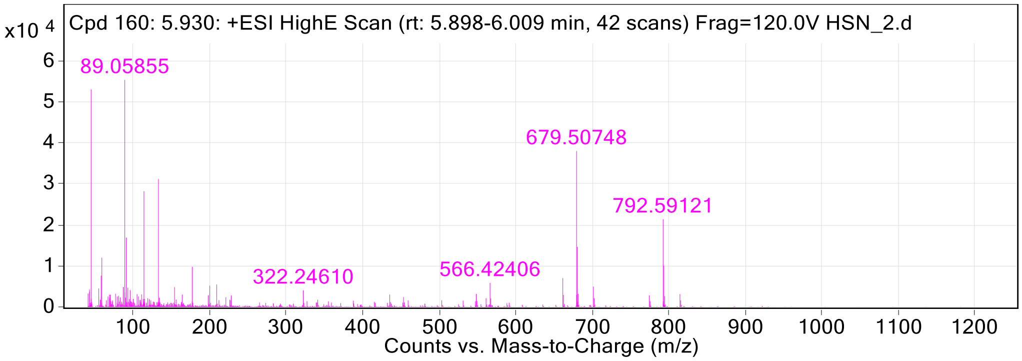

Peak List

m/z	z	Abund
45.03325		53014.81
59.04858		12008.22
89.05855	1	55316.86
91.05307	1	16874.19
114.0896	1	28197.86
133.08414	1	31170.82
679.50748	1	37988.42
680.51045	1	14633.52
792.59121	1	21352.51
793.59424	1	10029.62

Qualitative Analysis Report

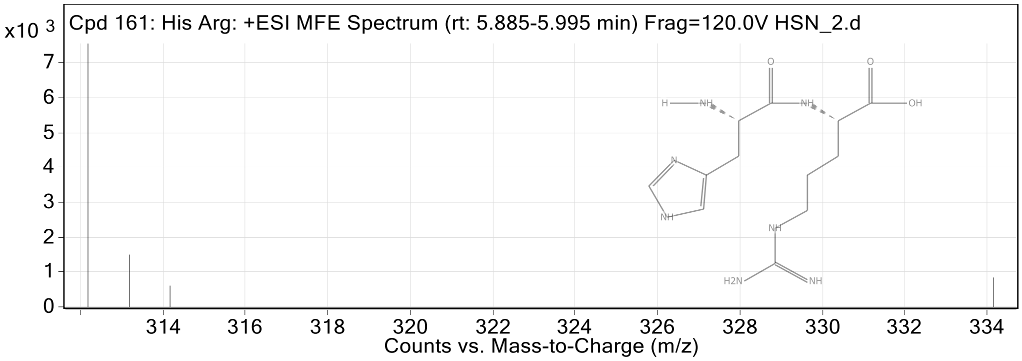

Peak List

m/z	z	Abund	Name	Formula	Ion	Score (DB)	Hits (DB)
312.17811	1	7562.75	His Arg	C12 H21 N7 O3	(M+H)+	77.47	4
313.18162	1	1500.17		C12 H21 N7 O3	(M+H)+		
314.16935	1	609.25		C12 H21 N7 O3	(M+H)+		
334.1601	1	843.1			(M+Na)+		

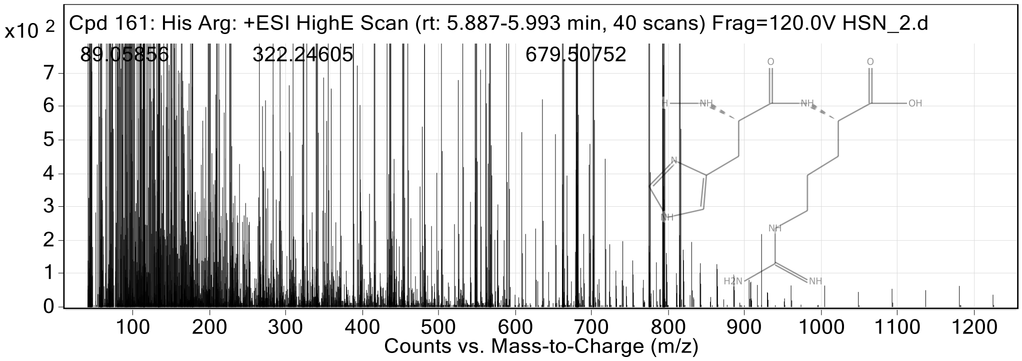

Peak List

m/z	z	Abund
45.03325		54502.26
89.05856	1	57270.71
91.05307	1	16322.99
114.08963	1	31713.43
133.08413	1	32338.38
177.10988		10160.72
679.50752	1	40954.68
680.5105	1	15765.09
792.59116	1	23657.79
793.59434	1	11065.54

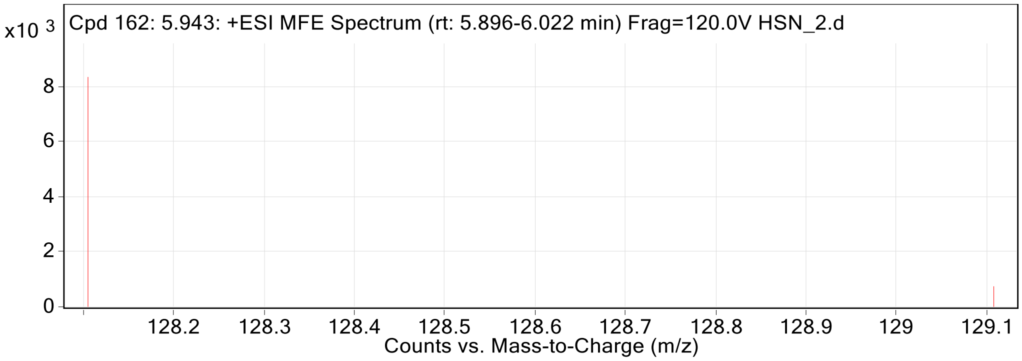

Peak List

m/z	z	Abund	Ion
128.105	1	8339.92	(M+H)+

129.10802	1	744.84	(M+H)+

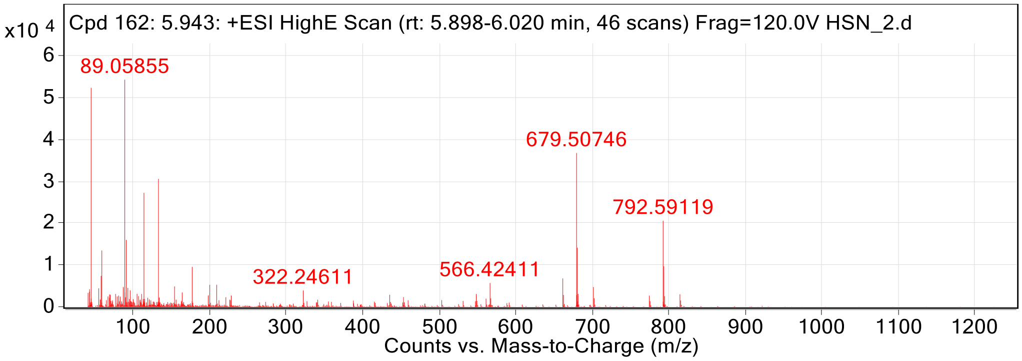

Peak List

m/z	z	Abund
45.03325		52397.16
59.04858		13460.34
89.05855	1	54324.17
91.05307	1	16011.58
114.08961	1	27257.18
133.08414	1	30611.87
679.50746	1	36795.38
680.51047	1	14152.67
792.59119	1	20592.76
793.59415	1	9687.18

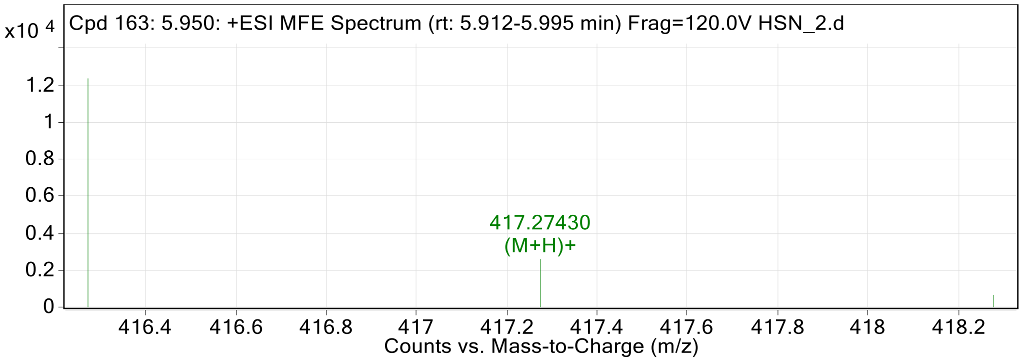

Peak List

m/z	z	Abund	Ion
416.27228	1	12366.54	(M+H)+
417.2743	1	2599.56	(M+H)+
418.27849	1	660.67	(M+H)+

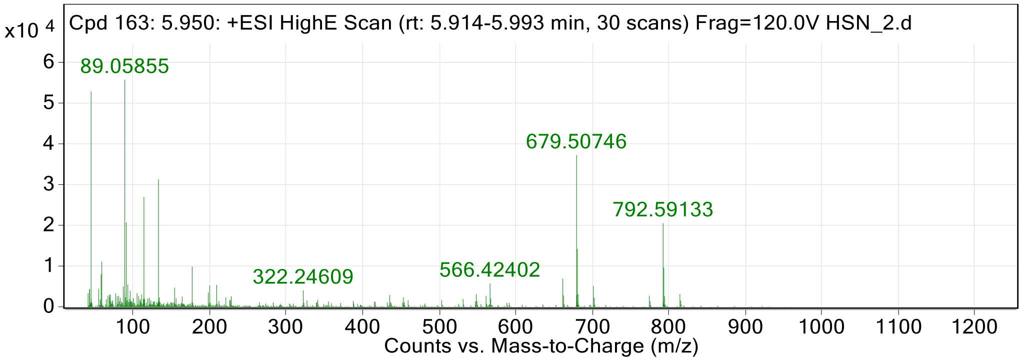

Peak List

m/z	z	Abund
45.03323		52708.59

59.04858		11073.98
89.05855	1	55596.63
91.05307	1	20637.33
114.0896	1	26891.88
133.08417	1	31190.66
177.10993		9877.24
679.50746	1	37145.71
680.51057	1	14158.2
792.59133	1	20484.92

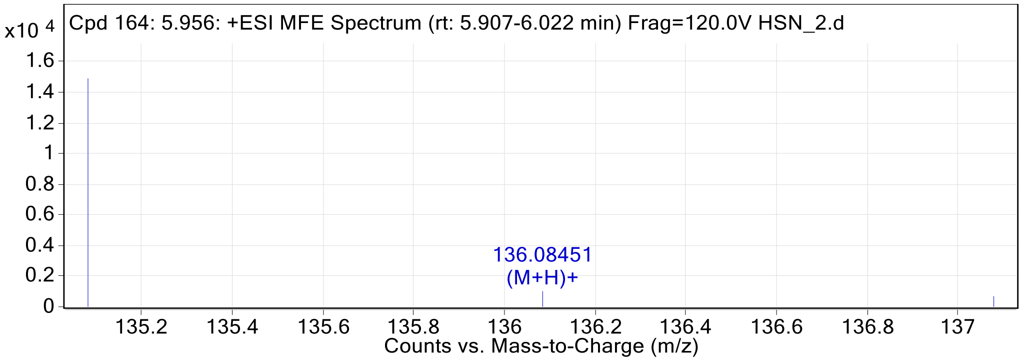

Peak List

m/z	z	Abund	Ion
135.08171	1	14903.36	(M+H)+
136.08451	1	1023.28	(M+H)+
137.07944	1	689.17	(M+H)+

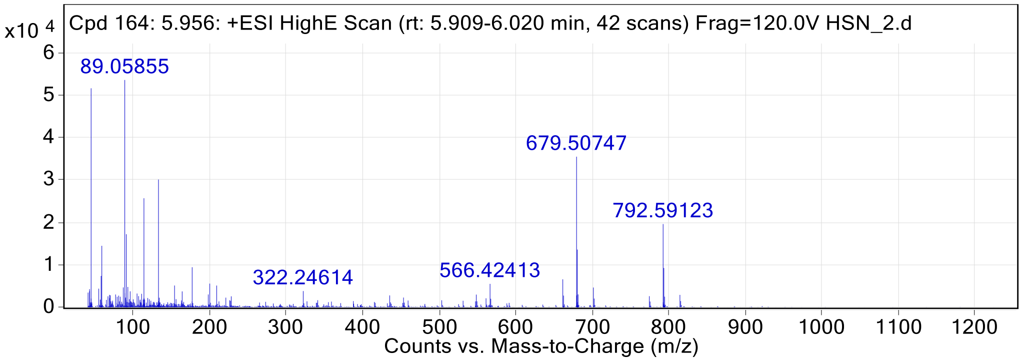

Peak List

m/z	z	Abund
45.03324		51644.02
59.04858		14449.72
89.05855	1	53601.76
91.05306	1	17191.86
114.0896	1	25681.44
133.08415	1	30098.61
177.10989		9398.23
679.50747	1	35496.43
680.51045	1	13560.7
792.59123	1	19590.13

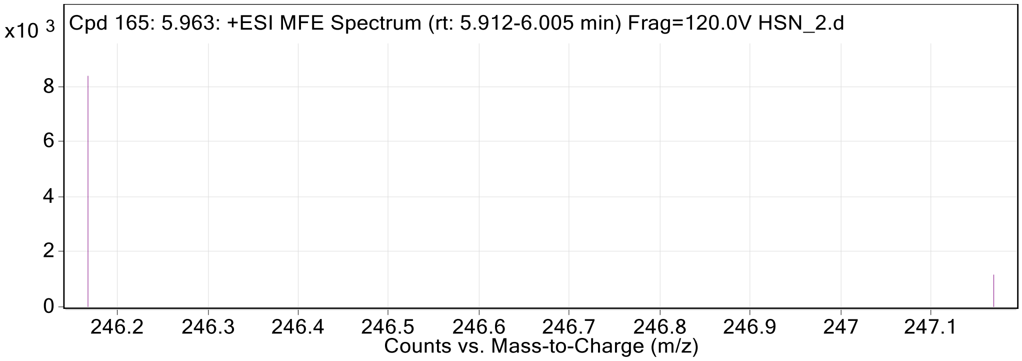

Peak List

m/z	z	Abund	Ion
246.16747	1	8396.58	(M+H)+
247.16942	1	1171.44	(M+H)+

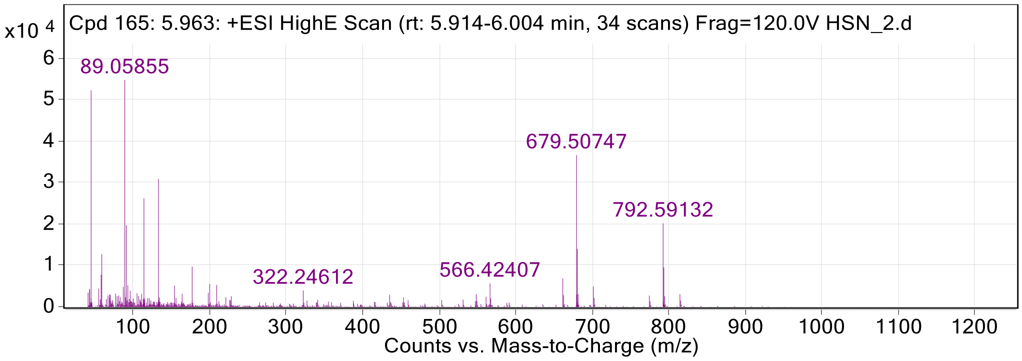

Peak List

m/z	z	Abund
45.03323		52198.67
59.04858		12678.14
89.05855	1	54688.36
91.05307	1	19660.66
114.0896	1	26145.22
133.08416	1	30824.36
177.10992		9690.88
679.50747	1	36580.77
680.51052	1	13960.03
792.59132	1	20120.22

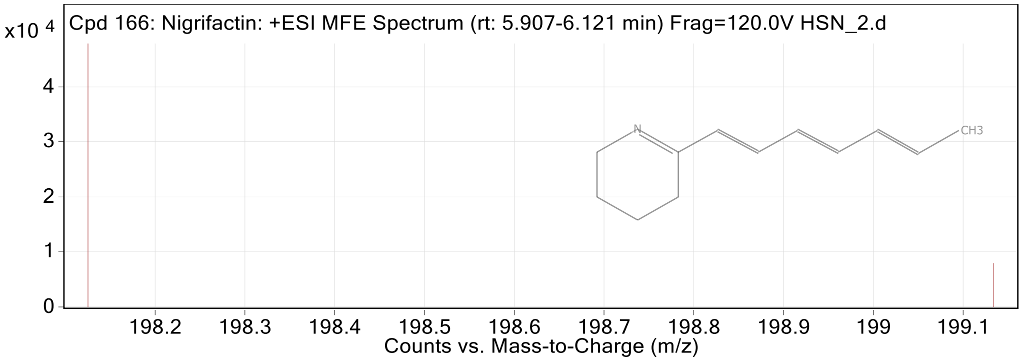

Peak List

m/z	z	Abund	Name	Formula	Ion	Score (DB)	Hits (DB)
198.12546	1	47772.78	Nigrifactin	C12 H17 N	(M+Na)+	71.73	1
199.1337	1	7955.48		C12 H17 N	(M+Na)+		

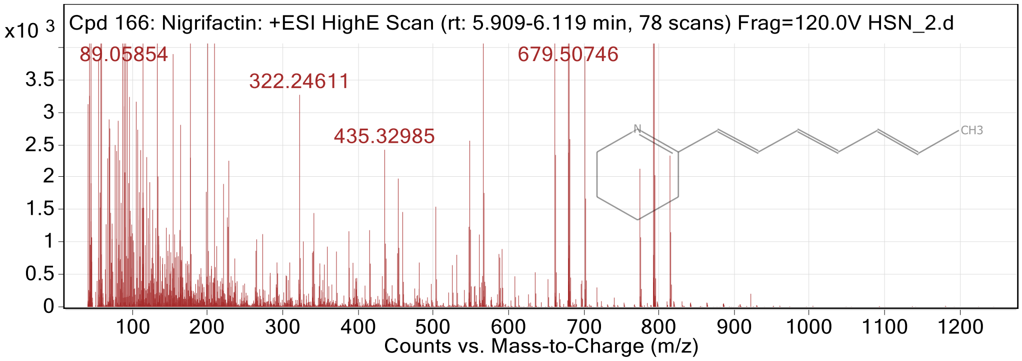

Peak List

m/z	z	Abund
45.03322		49129.48
59.04858		13146.87
89.05854	1	49542.93
91.05307		11573.1
114.08959	1	21598.93
133.08418	1	27534.97
177.10987		8666.39
679.50746	1	30163.51
680.51048	1	11675.54
792.59113	1	16199.49

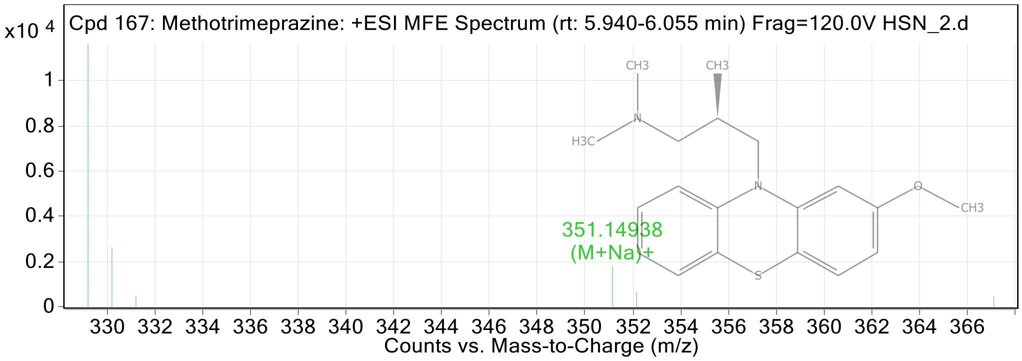

Peak List

m/z	z	Abund	Name	Formula	Ion	Score (DB)	Hits (DB)
329.16828	1	11629.49	Methotrimeprazine	C19 H24 N2 O S	(M+H)+	90.39	1
330.1706	1	2619.85		C19 H24 N2 O S	(M+H)+		
331.1774	1	485.7		C19 H24 N2 O S	(M+H)+		
351.14938	1	1798.28			(M+Na)+		
352.16041	1	660.95			(M+Na)+		
367.12847	1	459.08			(M+K)+		

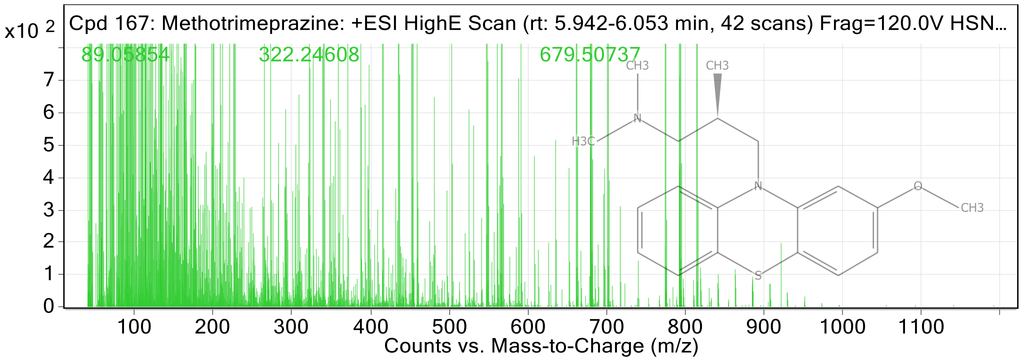

Peak List

m/z	z	Abund
45.03323		49011.33
59.0486		18955.38
89.05854	1	49148.81
91.05304	1	17411.3
114.08959	1	20717.95
133.08419	1	27366.27
177.10991		8579.39
679.50737	1	29962.9
680.51053	1	11483.03
792.59112	1	15878.88

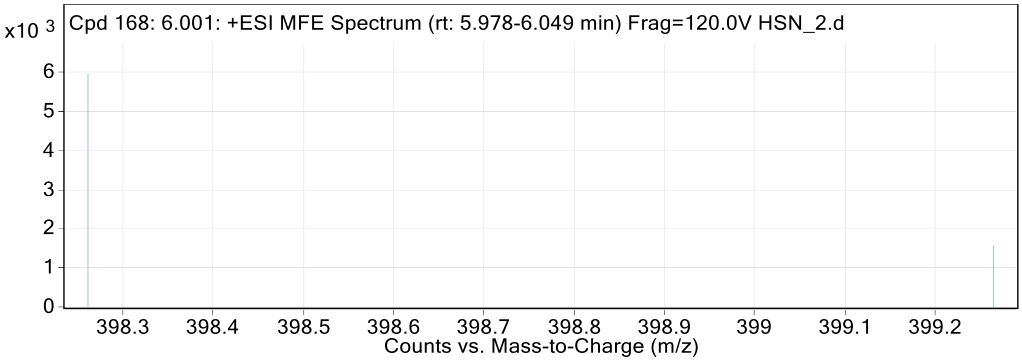

Peak List

m/z	z	Abund	Ion
398.26152	1	5974.22	(M+H)+
399.26507	1	1568.99	(M+H)+

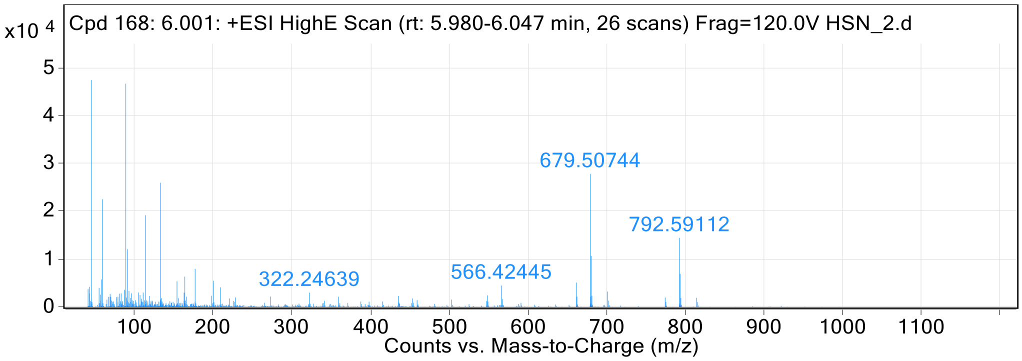

Peak List

m/z	z	Abund
45.03323		47423.62
59.0486		22515.86
89.05853	1	46675.21
91.05302	1	12042.34
114.08961	1	19166.63
133.08415	1	25957.87
177.10988		7912.64
679.50744	1	27798.89
680.51058	1	10655.77
792.59112	1	14409.9

Qualitative Analysis Report

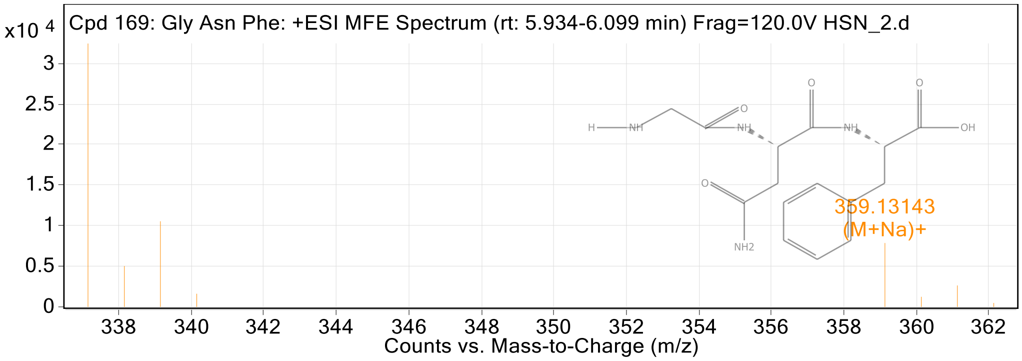

Peak List

m/z	z	Abund	Name	Formula	Ion	Score (DB)	Hits (DB)
337.14995	1	32455.81	Gly Asn Phe	C15 H20 N4 O5	(M+H)+	45.1	7
338.15313	1	5031.06		C15 H20 N4 O5	(M+H)+		
339.14728	1	10541.68		C15 H20 N4 O5	(M+H)+		
340.14986	1	1618.27		C15 H20 N4 O5	(M+H)+		
359.13143	1	7867.9			(M+Na)+		
360.13551	1	1242.2			(M+Na)+		
361.12912	1	2637.1			(M+Na)+		
362.13338	1	477.97			(M+Na)+		

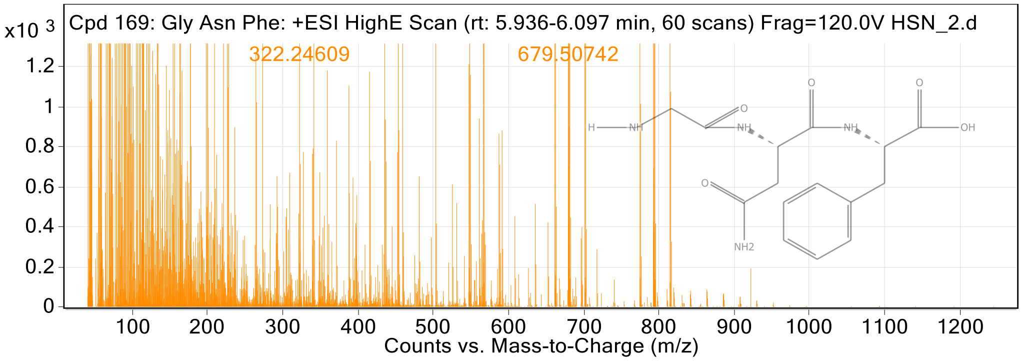

Peak List

m/z	z	Abund
45.03322		48467.96
59.04858		15774.91
89.05854	1	48298.07
91.05305	1	13698.31
114.08959	1	19877.27
133.08418	1	26832.47
177.10988		8441.43
679.50742	1	28911.53
680.51056	1	11124.69
792.59114	1	15203.77

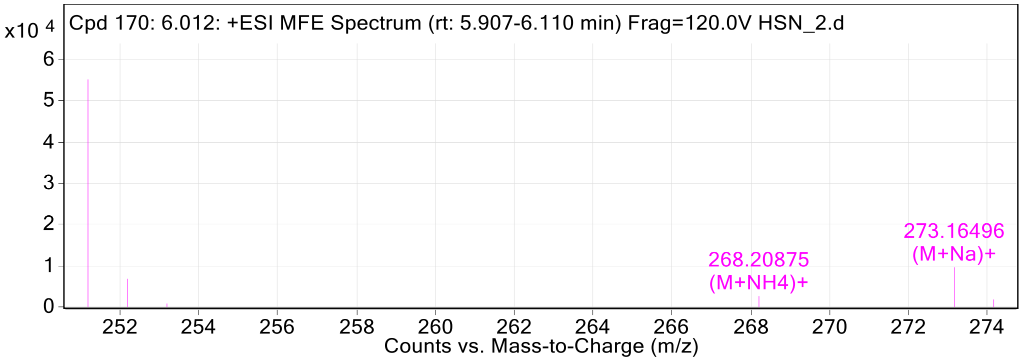

Peak List

m/z	z	Abund	Ion
251.18251	1	55197.73	(M+H)+
252.18625	1	6824.79	(M+H)+
253.18642	1	805.95	(M+H)+
268.20875	1	2620.02	(M+NH4)+
273.16496	1	9588.1	(M+Na)+
274.16692	1	1793.75	(M+Na)+

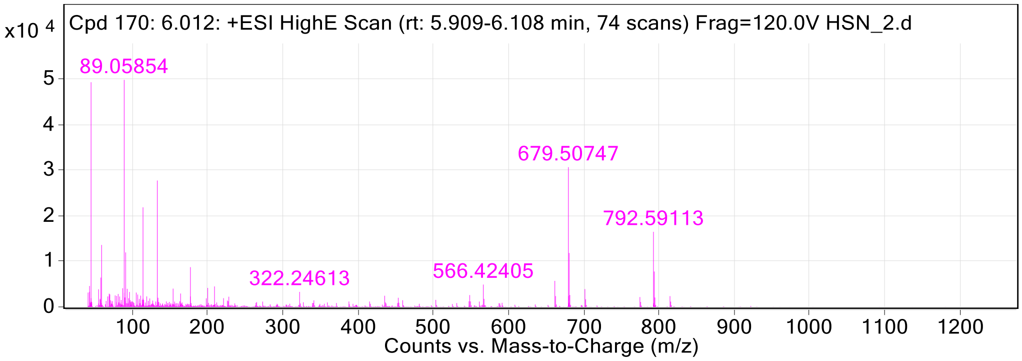

Peak List

m/z	z	Abund
45.03322		49284.27
59.04858		13588.36
89.05854	1	49798.61
91.05307		11961
114.08959	1	21842.53
133.08417	1	27721.05
177.10987		8718.14
679.50747	1	30655.46
680.51053	1	11822.58
792.59113	1	16471.61

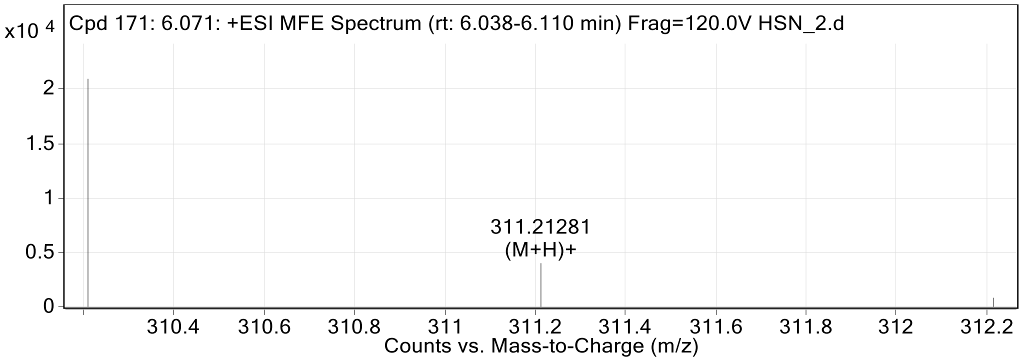

Peak List

m/z	z	Abund	Ion
310.20961	1	20925.84	(M+H)+
311.21281	1	4003.23	(M+H)+
312.2157	1	829.37	(M+H)+

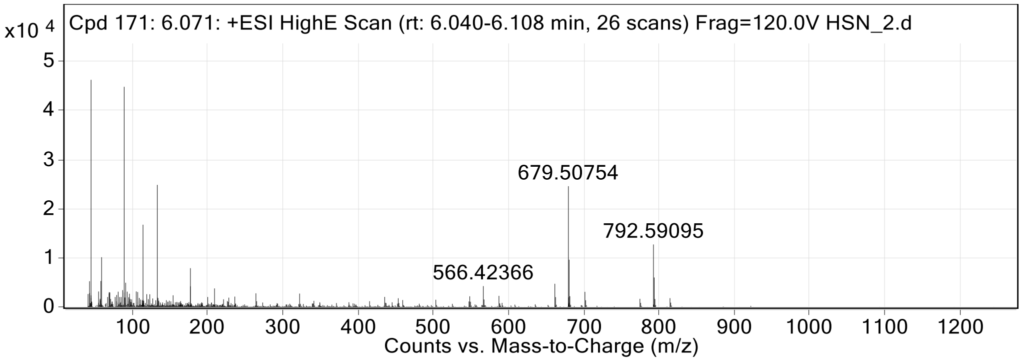

Peak List

m/z	z	Abund
45.03319	1	46267.22
59.04859		10117.19
89.05853	1	44843.71
114.08955	1	16743.48
133.0842	1	24859.31
177.10987		7882.42
679.50754	1	24587.34
680.5106	1	9611.54
792.59095	1	12711.73
793.5942	1	5980.5

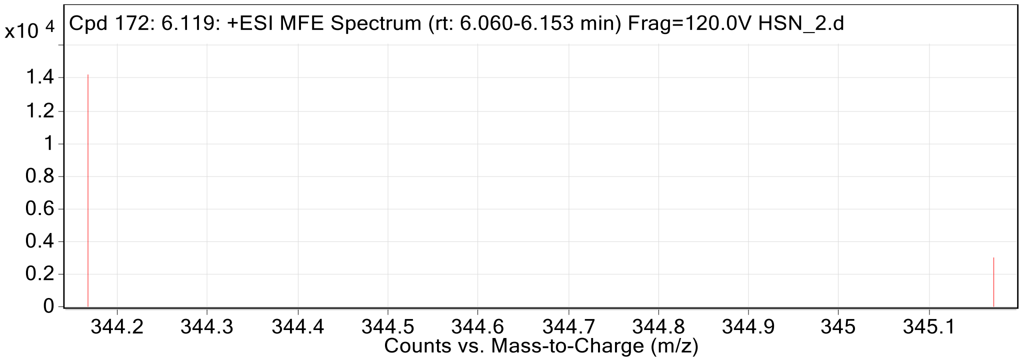

Peak List

m/z	z	Abund	Ion
344.16754	1	14228.21	(M+H)+
345.17168	1	3019.03	(M+H)+

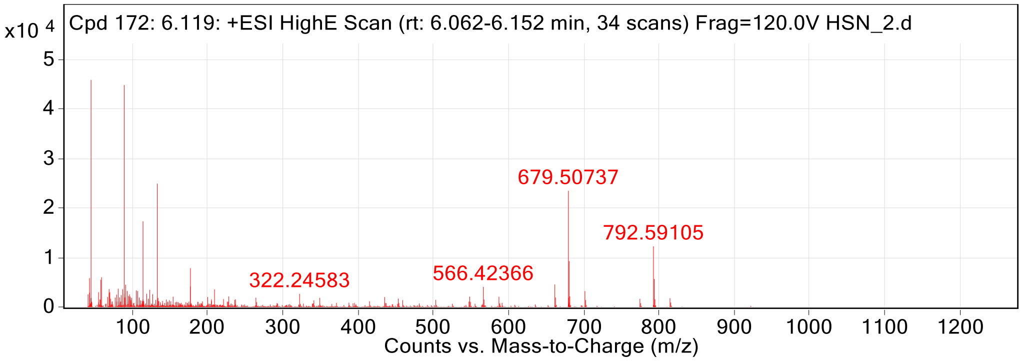

Peak List

m/z	z	Abund
43.01759		5832.01
45.0332		45936.86
59.04856		5996.66
89.05851	1	44898.59
114.08955	1	17332.06
133.08417	1	24943.77
177.10995		7846.66
679.50737	1	23498.98
680.51051	1	9271.55
792.59105	1	12277.9

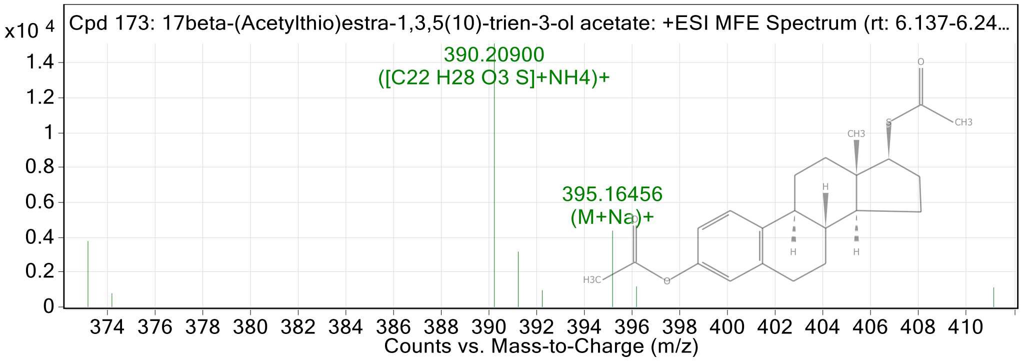

Peak List

m/z	z	Abund	Name	Formula	Ion	Score (DB)	Hits (DB)
373.18235	1	3783.36			(M+H)+		
374.18541	1	784.47			(M+H)+		
390.209	1	15124.91	17beta-(Acetylthio)estra-1,3,5(10)-trien-3-ol acetate	C22 H28 O3 S	(M+NH4)+	87.99	1
391.21239	1	3176.7		C22 H28 O3 S	(M+NH4)+		
392.21912	1	965.29		C22 H28 O3 S	(M+NH4)+		
395.16456	1	4385.89			(M+Na)+		
396.16843	1	1181.13			(M+Na)+		
411.13915	1	1124.2			(M+K)+		

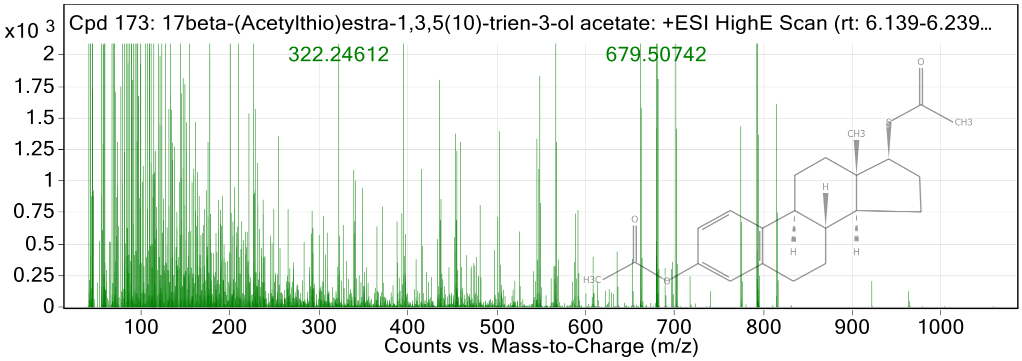

Peak List

m/z	z	Abund
43.01754		7508.1
45.03322		44441.83
89.05855	1	42538.54
114.0896		14081.09
123.1149		5438.06
133.0842	1	23723.34
177.10993		7611.65
679.50742	1	20248.22
680.51054	1	7859.65

792.59099	1	10349.28

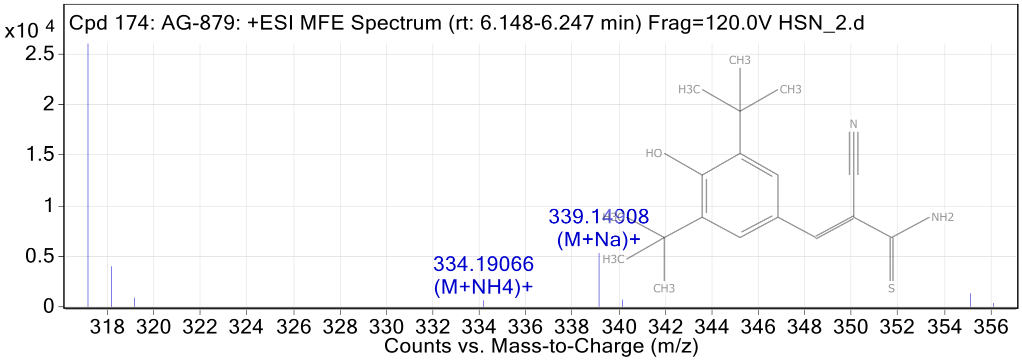

Peak List

m/z	z	Abund	Name	Formula	Ion	Score (DB)	Hits (DB)
317.16794	1	26070.51	AG-879	C18 H24 N2 O S	(M+H)+	84.09	1
318.1724	1	3997.79		C18 H24 N2 O S	(M+H)+		
319.1773	1	900.77		C18 H24 N2 O S	(M+H)+		
334.19066	1	629.9			(M+NH4)+		
339.14908	1	5326.64			(M+Na)+		
340.15143	1	709.81			(M+Na)+		
355.1231	1	1332.75			(M+K)+		
356.12686	1	381			(M+K)+		

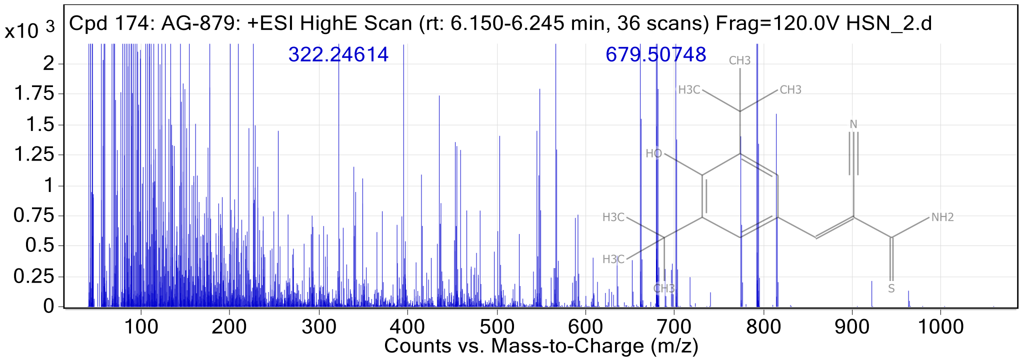

Peak List

m/z	z	Abund
43.01753		7578.03
45.03322		44127.02
89.05856	1	42185.38
105.06835		5451.82
114.08962		13438.97
133.0842	1	23598.63
177.10996		7576.12
679.50748	1	19956.05
680.51055	1	7779.12
792.59092	1	10093.31

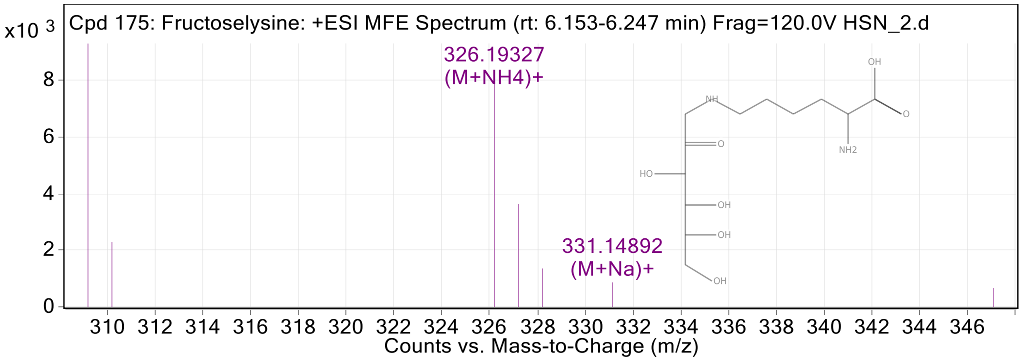

Peak List

m/z	z	Abund	Name	Formula	Ion	Score (DB)	Hits (DB)
309.16685	1	9317.47	Fructoselysine	C12 H24 N2 O7	(M+H)+	60.95	2
310.17104	1	2297.99		C12 H24 N2 O7	(M+H)+		
326.19327	1	7894.84			(M+NH4)+		
327.19763	1	3641.73			(M+NH4)+		
328.20406	1	1356.72			(M+NH4)+		
331.14892	1	864.36			(M+Na)+		
347.12535	1	668.64			(M+K)+		

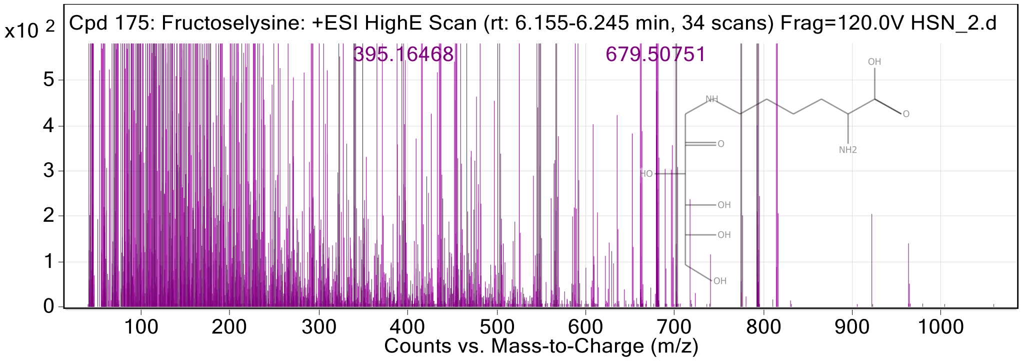

Peak List

m/z	z	Abund
43.01752		7625.52
45.03322		44139.39
89.05855	1	42116.86
105.06835		5590.9
114.08962		13242.46
133.0842	1	23496.27
177.10996		7565.19
679.50751	1	19835.92
680.51051	1	7735.39
792.59099	1	10018.21

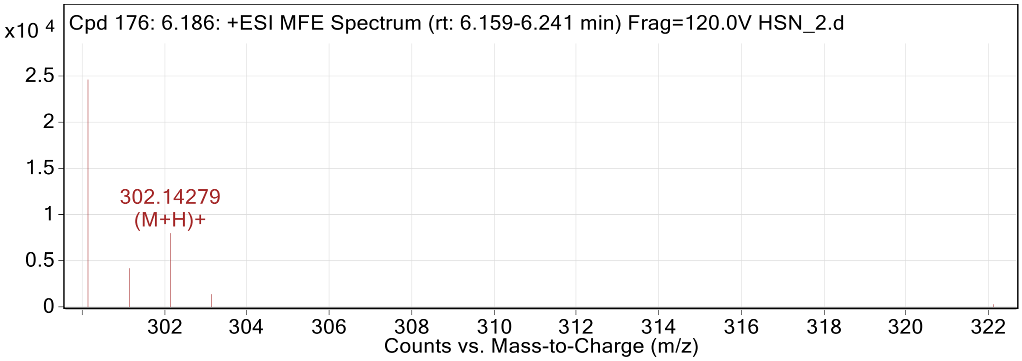

Peak List

m/z	z	Abund	Ion
300.1446	1	24628.02	(M+H)+
301.14831	1	4173.25	(M+H)+
302.14279	1	7973.32	(M+H)+
303.14528	1	1375.26	(M+H)+
322.12933	1	278.12	(M+Na)+

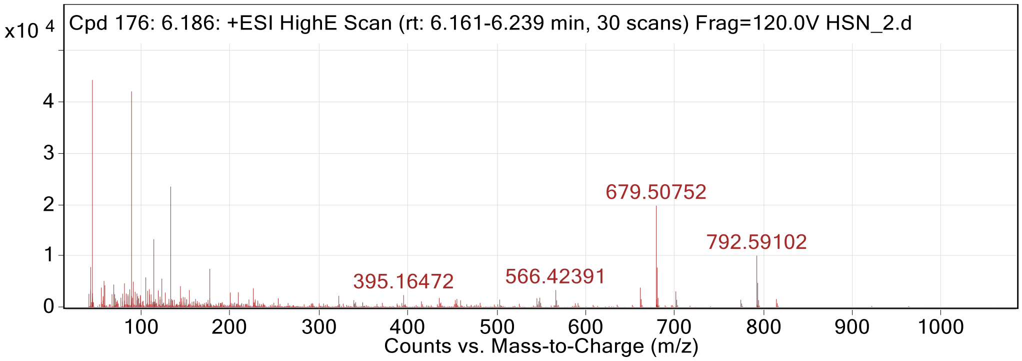

Peak List

m/z	z	Abund
43.01753		7789.5
45.03322		44325.76
89.05856	1	42089.63
105.06837		5782.56
114.08964		13212.52
133.08422	1	23490.84
177.11005		7438.52
679.50752	1	19783.59
680.5105	1	7695.24
792.59102	1	10019.52

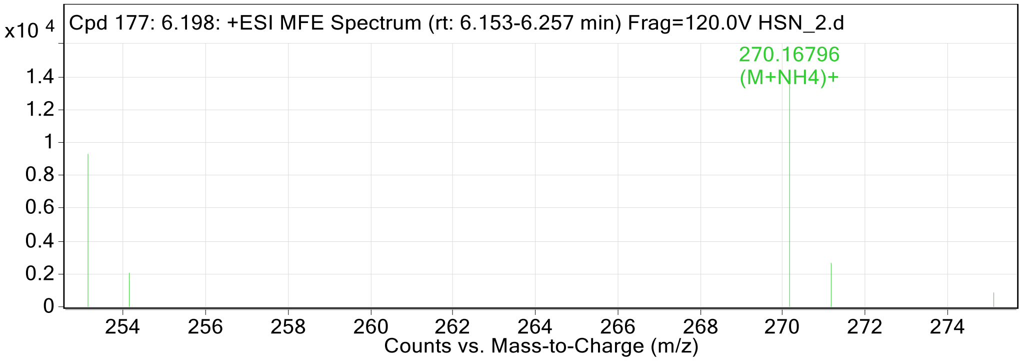

Peak List

m/z	z	Abund	Ion

253.1412	1	9300.75	(M+H)+
254.14402	1	2060.08	(M+H)+
270.16796	1	13928	(M+NH4)+
271.17719	1	2667.1	(M+NH4)+
275.1237	1	870.51	(M+Na)+

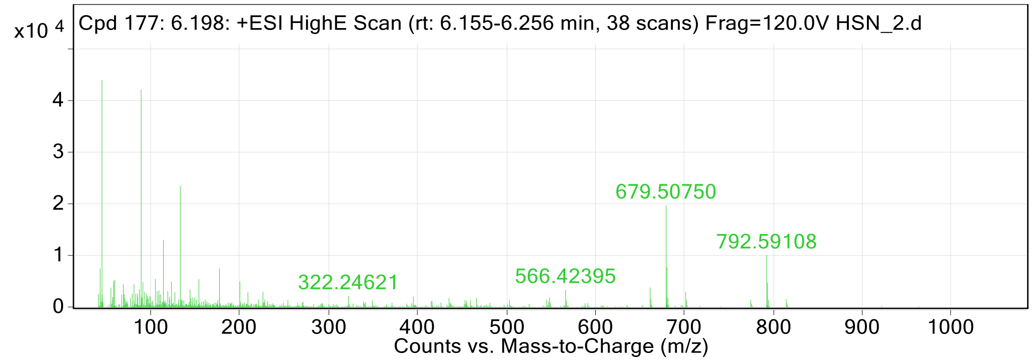

Peak List

m/z	z	Abund
43.01752		7482.87
45.03322		44054.81
89.05855	1	42158.52
105.06831		5455.48
114.08962		13047.65
133.08419	1	23488.89
177.10997		7494.56
679.5075	1	19746.75
680.5105	1	7689.2
792.59108	1	10058.06

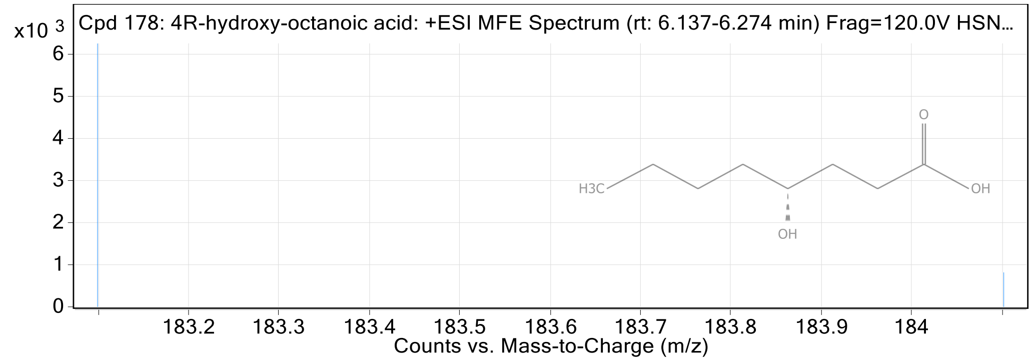

Peak List

m/z	z	Abund	Name	Formula	Ion	Score (DB)	Hits (DB)
183.09941	1	6250.99	4R-hydroxy-octanoic acid	C8 H16 O3	(M+Na)+	80.95	10
184.10201	1	816.82		C8 H16 O3	(M+Na)+		

Qualitative Analysis Report

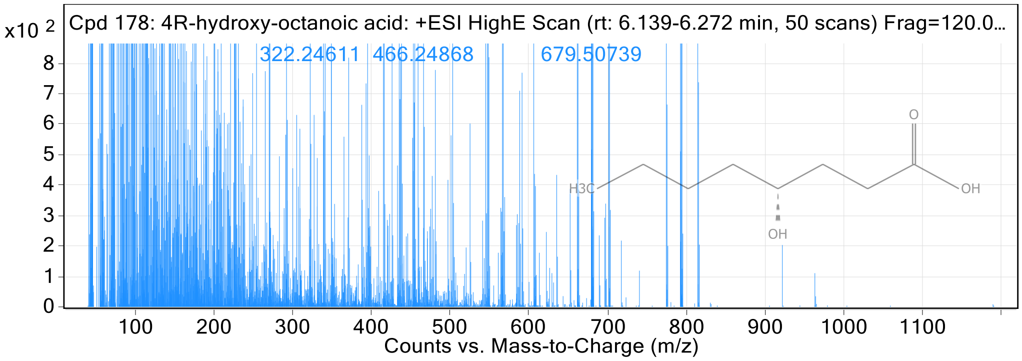

Peak List

m/z	z	Abund
43.01752		7163.88
45.03323		44029.4
59.0486		6610.03
89.05855	1	42167.93

Qualitative Analysis Report

m/z	z	Abund
114.08962		13291.88
133.08417	1	23574.45
177.10994		7428.01
679.50739	1	19853.18
680.51056	1	7746.33
792.59101	1	10142.01

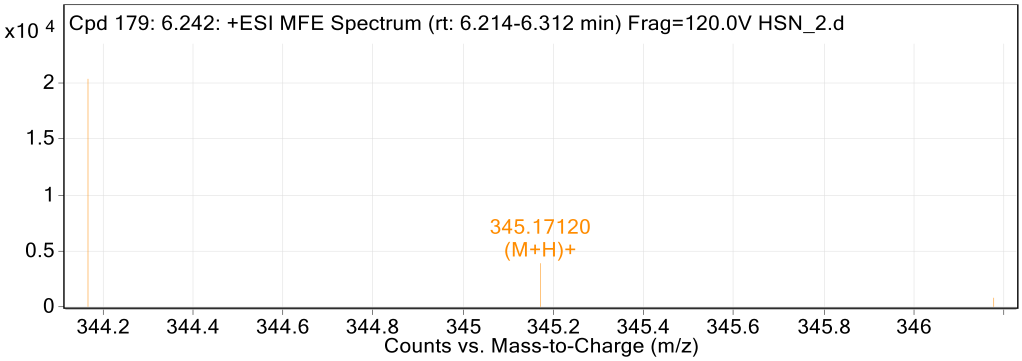

Peak List

m/z	z	Abund	Ion
344.16744	1	20392.23	(M+H)+
345.1712	1	3896.43	(M+H)+
346.17729	1	807.36	(M+H)+

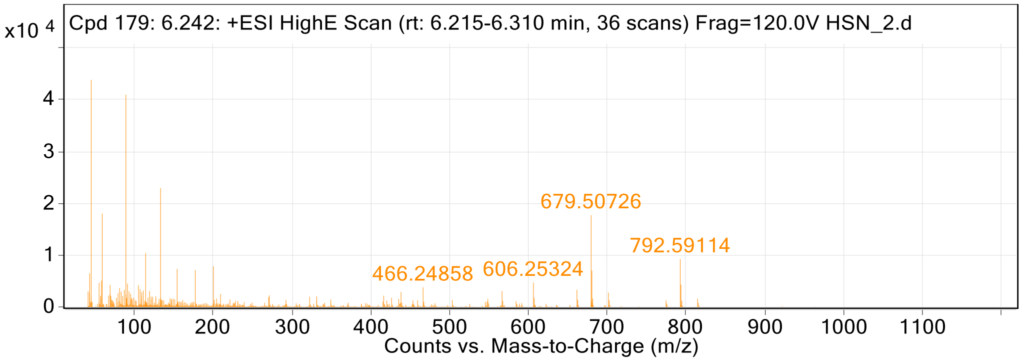

Peak List

m/z	z	Abund
45.03325		43807.14
59.04859		17986.8
89.05856	1	40952.14
114.08967		10389.68
133.08414	1	22930.56
154.08422		7321.24
177.10987		7112.29
200.12567	1	7823.41
679.50726	1	17724.05
792.59114	1	9232.89

Qualitative Analysis Report

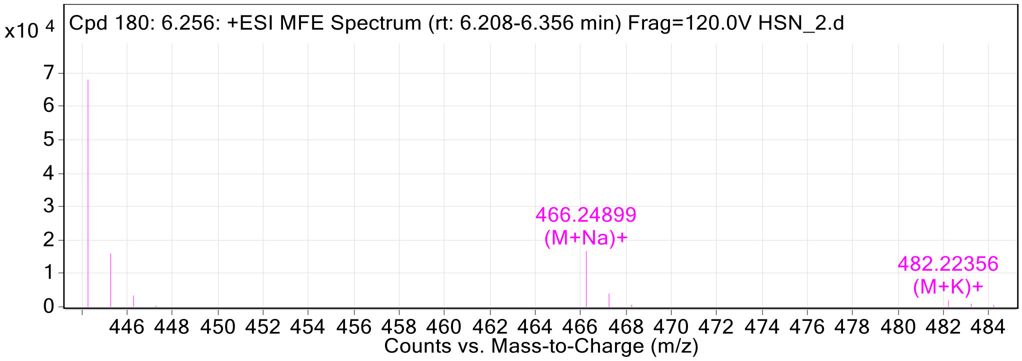

Peak List

m/z	z	Abund	Ion
444.26692	1	67945.2	(M+H)+
445.2702	1	15962.7	(M+H)+
446.2727	1	3406.77	(M+H)+
447.27051	1	379.63	(M+H)+
466.24899	1	16625.84	(M+Na)+
467.25185	1	3935.13	(M+Na)+
468.25656	1	622.42	(M+Na)+
482.22356	1	1969.93	(M+K)+
483.22836	1	903.49	(M+K)+
484.22317	1	554.52	(M+K)+

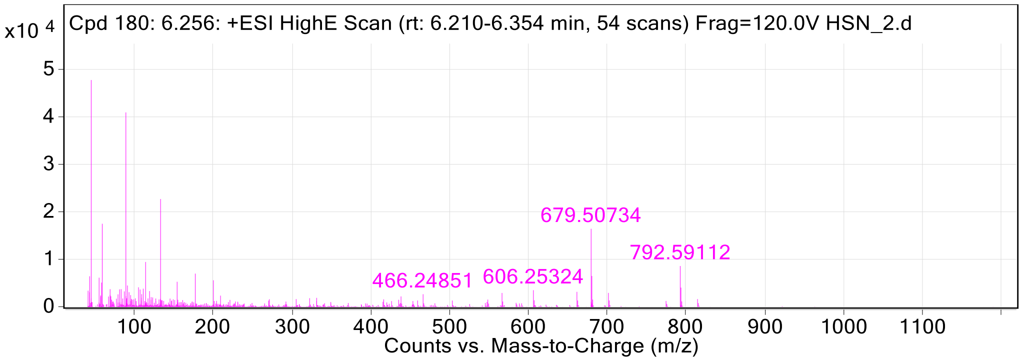

Peak List

m/z	z	Abund
43.01753		6407.45
45.03325		47669.68
59.0486		17446.77
89.05855	1	40860.39
114.08966		9413.14
133.08418	1	22651.26
177.10991		6962.69
679.50734	1	16395.73
680.51048	1	6470.52
792.59112	1	8564.4

Qualitative Analysis Report

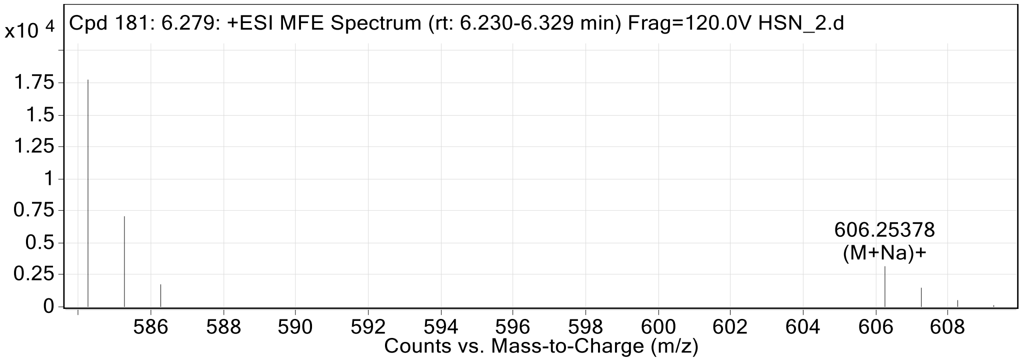

Peak List

m/z	z	Abund	Ion
584.27138	1	17750.13	(M+H)+
585.2765	1	7076.96	(M+H)+
586.27844	1	1759.33	(M+H)+
606.25378	1	3174.74	(M+Na)+
607.25746	1	1495.36	(M+Na)+
608.2615	1	525.86	(M+Na)+
609.25563	1	131.53	(M+Na)+

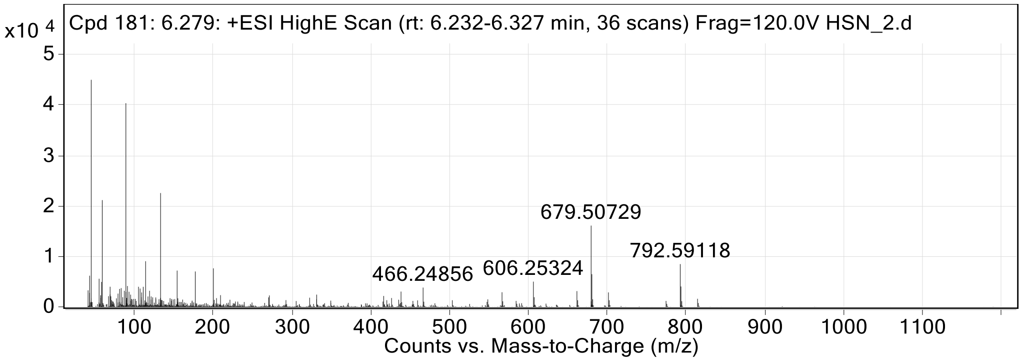

Peak List

m/z	z	Abund
45.03325		45025.35
59.04859		21165.44
89.05855	1	40391.04
114.08966		9049.19
133.08417	1	22570.35
154.08421		7188.19
177.10992		7012.88
200.12568	1	7632.52
679.50729	1	16113.21
792.59118	1	8479.83

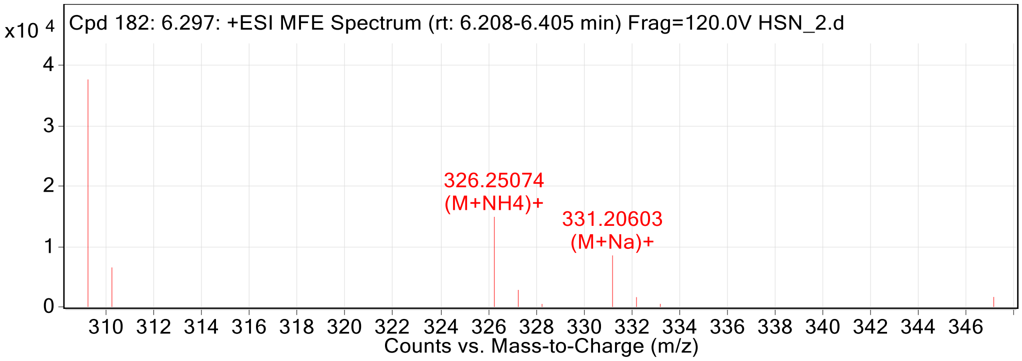

Peak List

m/z	z	Abund	Ion
309.2246	1	37734.91	(M+H)+
310.22776	1	6542.42	(M+H)+
326.25074	1	14943	(M+NH4)+
327.25347	1	2817.19	(M+NH4)+
328.25104	1	486.59	(M+NH4)+
331.20603	1	8537.53	(M+Na)+
332.20908	1	1614.86	(M+Na)+
333.20634	1	501.71	(M+Na)+
347.18051	1	1645.21	(M+K)+

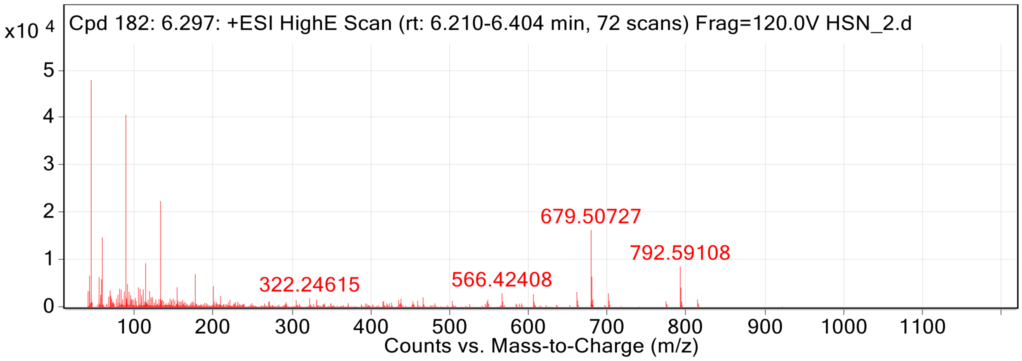

Peak List

m/z	z	Abund
43.01752		6575.07
45.03325		47907.27
59.0486		14663.7
89.05854	1	40611.07
114.08963		9221.3
133.08419	1	22350.2
177.10995		6878.31
679.50727	1	16226.57
680.51049	1	6378.23
792.59108	1	8525.11

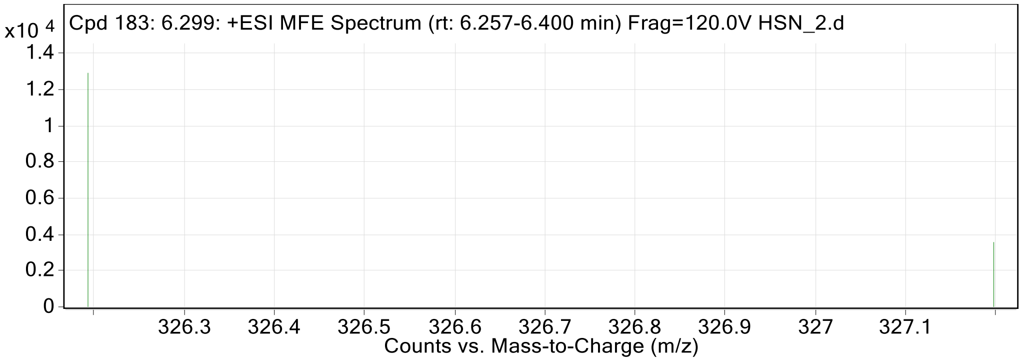

Peak List

m/z	z	Abund	Ion
326.19346	1	12928.09	(M+H)+
327.19761	1	3578.47	(M+H)+

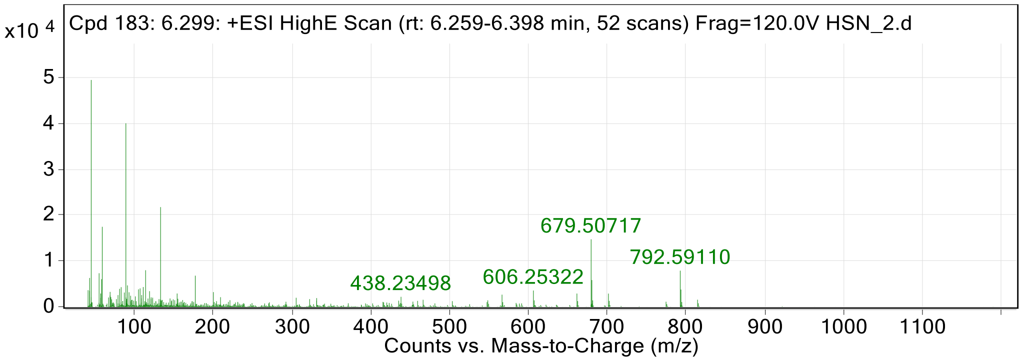

Peak List

m/z	z	Abund
43.01753		6280.74
45.03325		49497.29
55.05378		7313.47
59.0486		17445.81
89.05853	1	40043.34
114.08962		7967.57
133.08421	1	21748.56
177.10998		6776.81
679.50717	1	14723.41
792.5911	1	7871.95

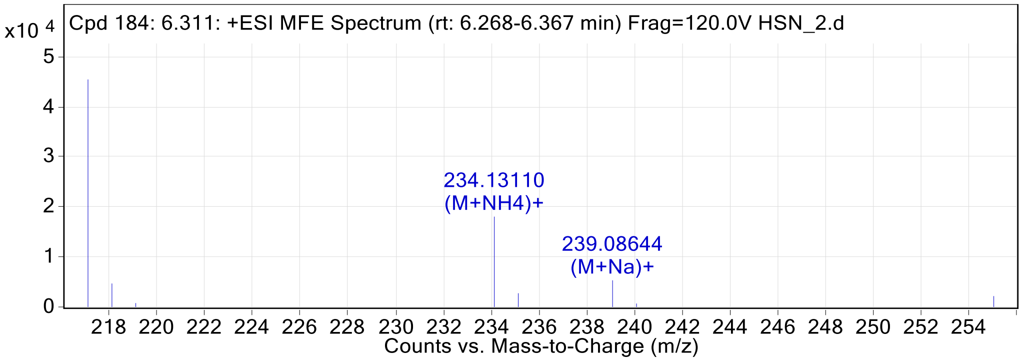

Peak List

m/z	z	Abund	Ion
217.10466	1	45434.35	(M+H)+
218.10823	1	4686.7	(M+H)+
219.10686	1	773.36	(M+H)+

234.1311	1	18040.45	(M+NH4)+
235.13474	1	2740.47	(M+NH4)+
239.08644	1	5320.64	(M+Na)+
240.09035	1	681.24	(M+Na)+
255.06016	1	2170.26	(M+K)+

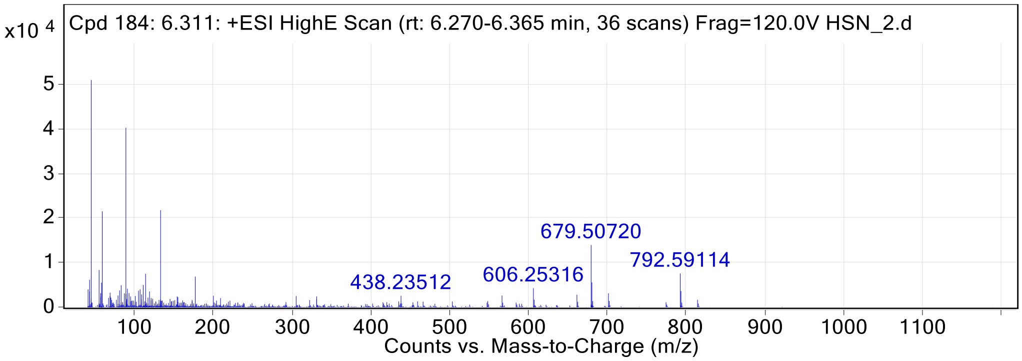

Peak List

m/z	z	Abund
43.01753		6125.61
45.03325		51005.19
55.05378		8317.2
59.0486		21487.08
89.05853	1	40274.23
114.08965		7467.71
133.08423	1	21756.73
177.10992		6822.58
679.5072	1	13939.82
792.59114	1	7539.15

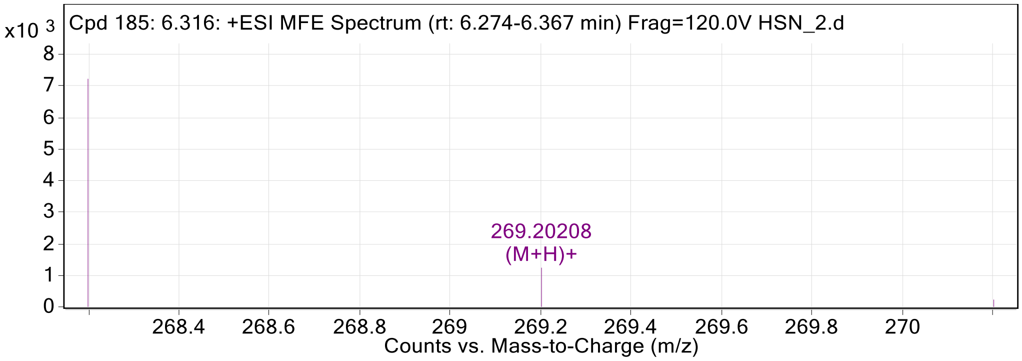

Peak List

m/z	z	Abund	Ion
268.19875	1	7222.98	(M+H)+
269.20208	1	1239.89	(M+H)+
270.20258	1	229.14	(M+H)+

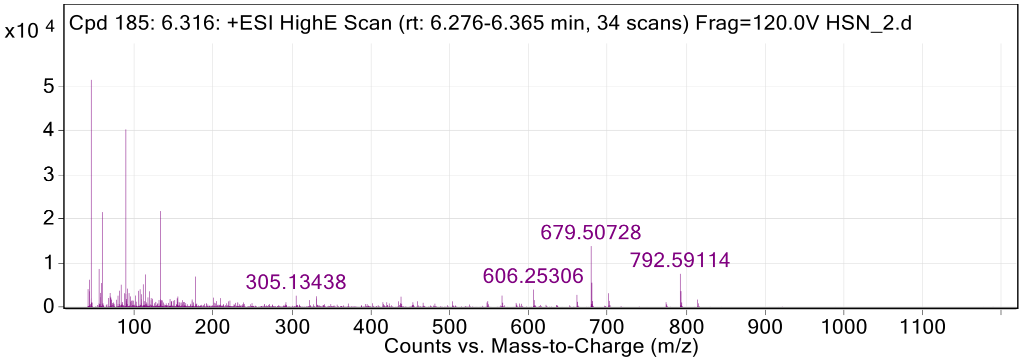

Peak List

m/z	z	Abund
43.01755		6167.04
45.03325		51503.44
55.05379		8628.05
59.0486		21463.83
89.05853	1	40259.66
114.08964		7344.52
133.08424	1	21737.63
177.10993		6871.88
679.50728	1	13800.52
792.59114	1	7514.28

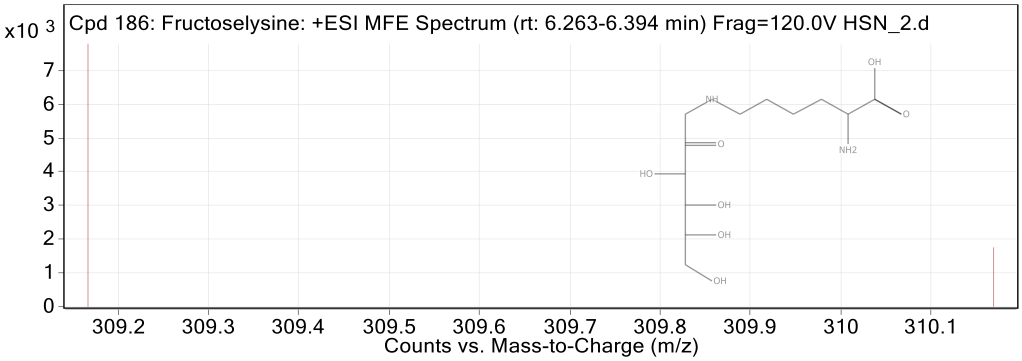

Peak List

m/z	z	Abund	Name	Formula	Ion	Score (DB)	Hits (DB)
309.16654	1	7798.56	Fructoselysine	C12 H24 N2 O7	(M+H)+	70.07	2
310.16969	1	1760.23		C12 H24 N2 O7	(M+H)+		

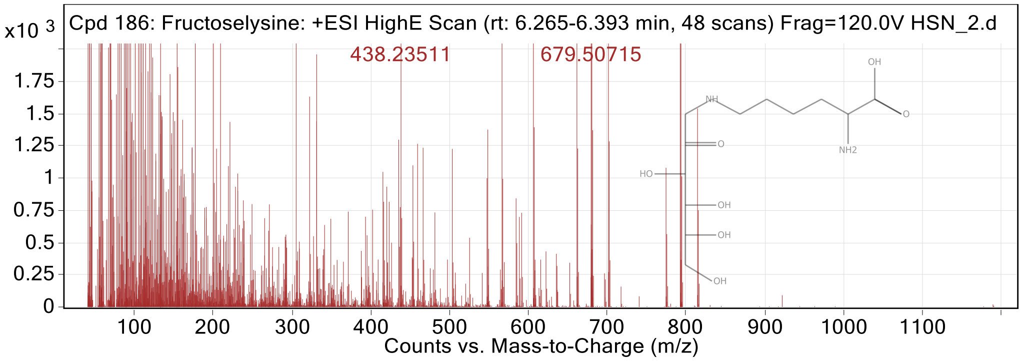

Peak List

m/z	z	Abund
43.01752		6294.71
45.03325		49876.04
55.05377		7584.63

59.0486		18031.54
89.05853	1	40072.84
114.08962		7826.56
133.0842	1	21675.12
177.11		6749.59
679.50715	1	14542.25
792.59114	1	7795.01

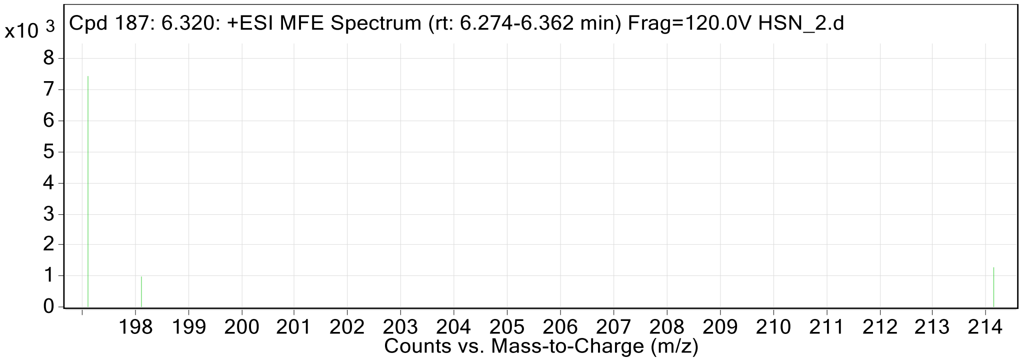

Peak List

m/z	z	Abund	Ion
197.11467	1	7451.38	(M+H)+
198.11989	1	981.09	(M+H)+
214.14432	1	1280.21	(M+NH4)+

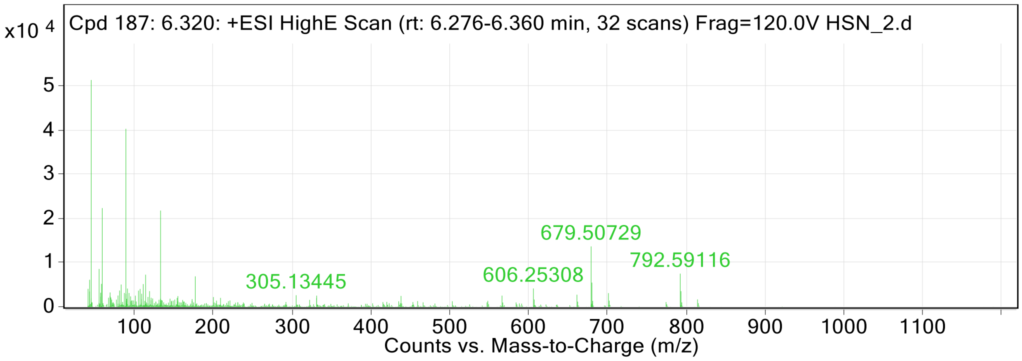

Peak List

m/z	z	Abund
43.01754		6102.15
45.03325		51339.84
55.05378		8548.48
59.0486		22332.22
89.05853	1	40285.46
114.08964		7253.85
133.08422	1	21771.63
177.10995		6874.63
679.50729	1	13659.36
792.59116	1	7490.51

Qualitative Analysis Report

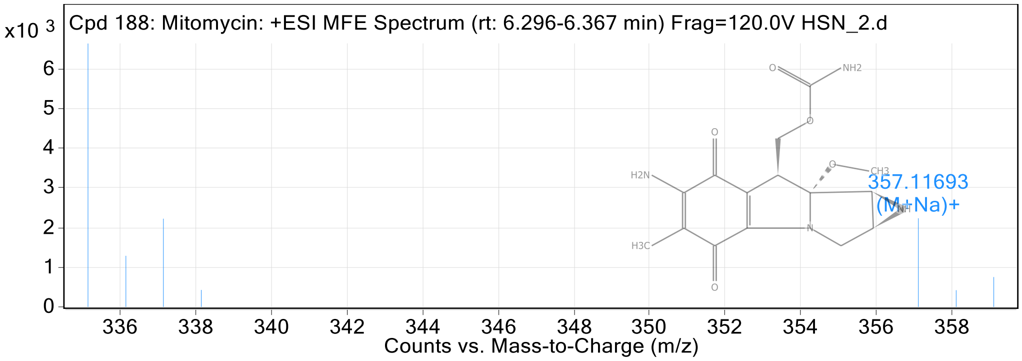

Peak List

m/z	z	Abund	Name	Formula	Ion	Score (DB)	Hits (DB)
335.13518	1	6641.07	Mitomycin	C15 H18 N4 O5	(M+H)+	51.03	1
336.13867	1	1287.47		C15 H18 N4 O5	(M+H)+		
337.13149	1	2222.87		C15 H18 N4 O5	(M+H)+		
338.13433	1	427.22		C15 H18 N4 O5	(M+H)+		
357.11693	1	2233.46			(M+Na)+		
358.11841	1	420.9			(M+Na)+		
359.11395	1	751.99			(M+Na)+		

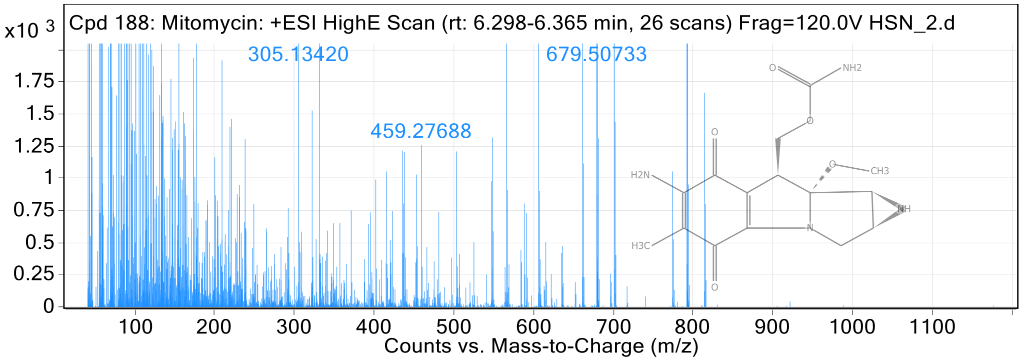

Peak List

m/z	z	Abund
43.01757		6192.03
45.03325	1	54538.85
55.05378		9758.78
59.04861		19644.71
89.05852	1	40553.42
114.08958		6985.62
133.08429	1	21756.31
177.11001		6850.18
679.50733	1	13359.82
792.59106	1	7250.22

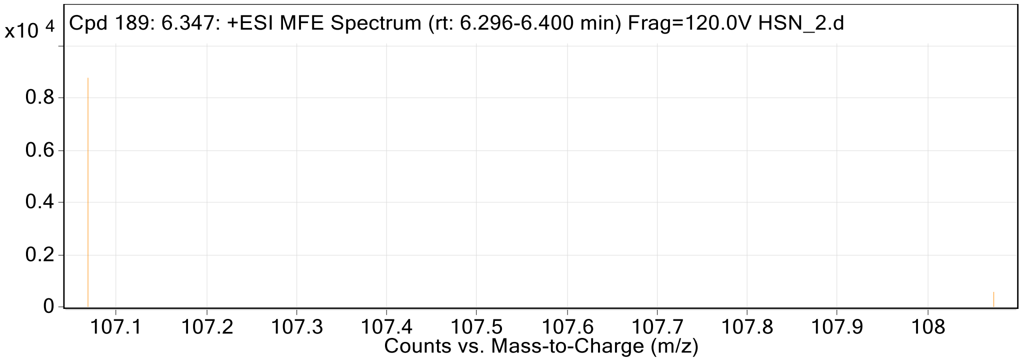

Peak List

m/z	z	Abund	Ion
107.06872	1	8769.28	(M+H)+
108.07328	1	573.26	(M+H)+

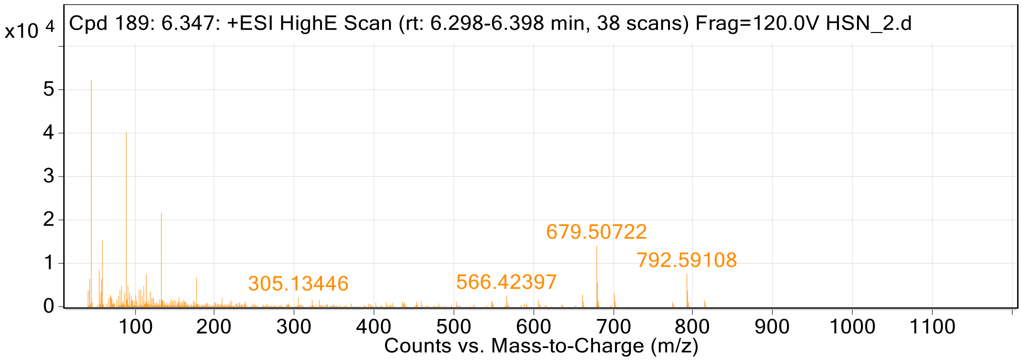

Peak List

m/z	z	Abund
43.01755		6422.63
45.03325	1	52243.41
55.05377		8480.64
59.04861		15277.39
89.05852	1	40180.26
114.08956		7525.06
133.08424	1	21621.04
177.11006		6747.73
679.50722	1	14119.71
792.59108	1	7608.93

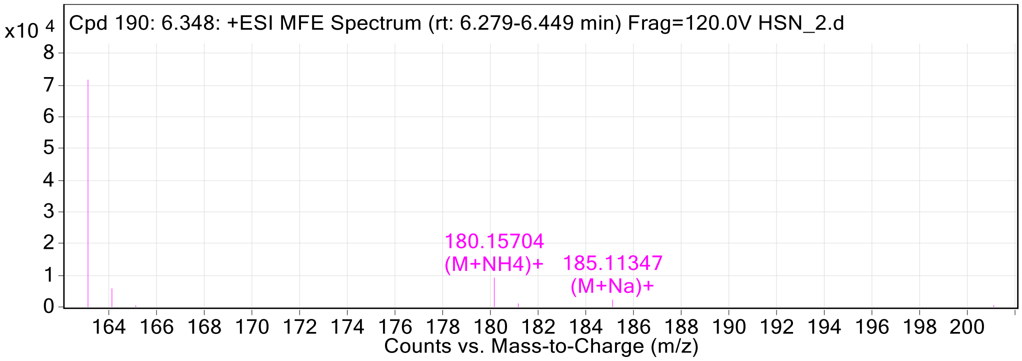

Peak List

m/z	z	Abund	Ion
163.13058	1	71709.1	(M+H)+
164.13388	1	5871.89	(M+H)+
165.13489	1	492.39	(M+H)+

180.15704	1	9232.58	(M+NH4)+
181.16013	1	1092.22	(M+NH4)+
185.11347	1	2341.12	(M+Na)+
201.08703	1	542.28	(M+K)+

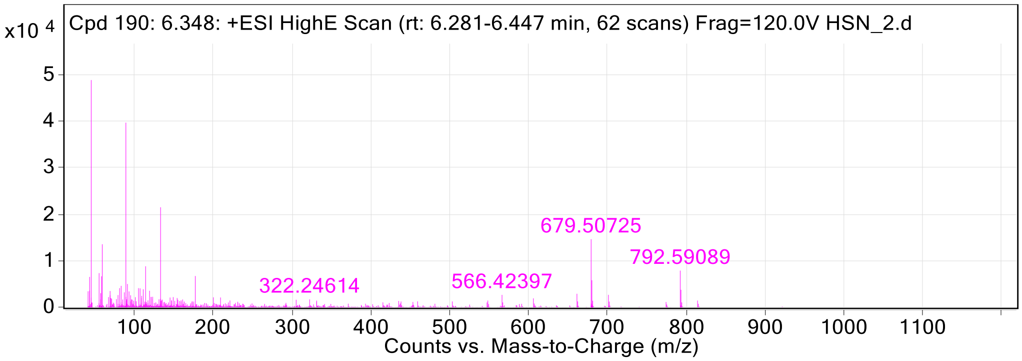

Peak List

m/z	z	Abund
45.03323		48901.91
55.05379		7318.35
58.06465		6630.72
59.0486		13536.27
89.05853	1	39734.95
114.08957		8792.69
133.08421	1	21494.84
177.10999		6689.66
679.50725	1	14622.83
792.59089	1	7858.71

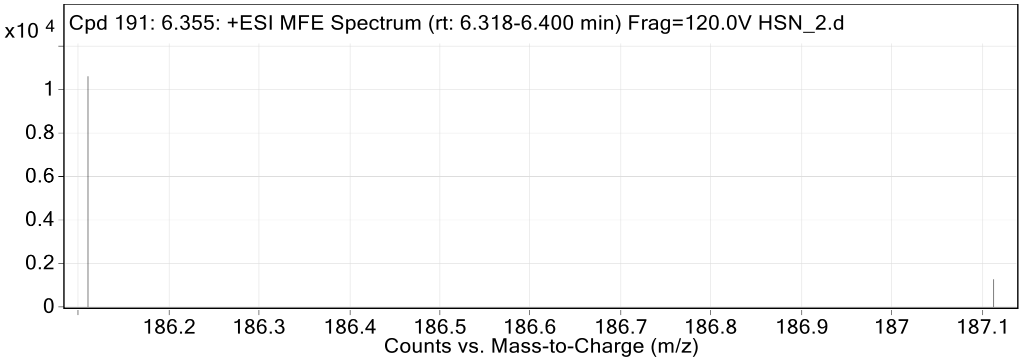

Peak List

m/z	z	Abund	Ion
186.1104	1	10637.21	(M+H)+
187.11297	1	1268.98	(M+H)+

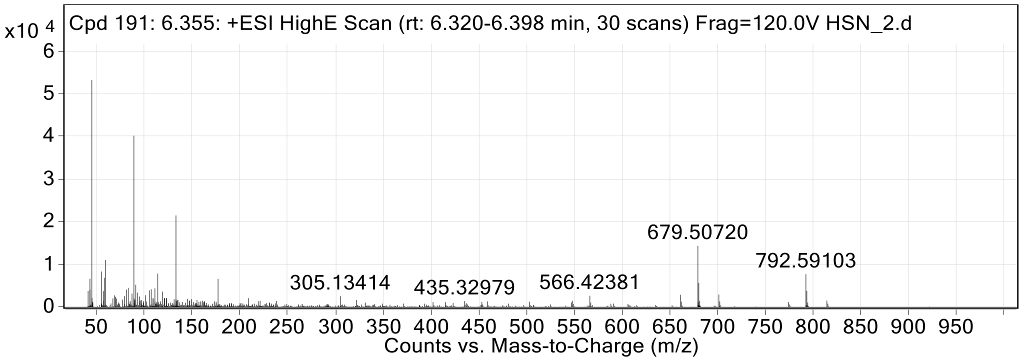

Peak List

m/z	z	Abund
43.01755		6567.67
45.03325	1	53221.14
55.05376		8267.91
58.06468		6813.01
59.04863		10988.51
89.05852	1	40148.66
114.08956		7774.97
133.08423	1	21437.04
679.5072	1	14306.75
792.59103	1	7640.07

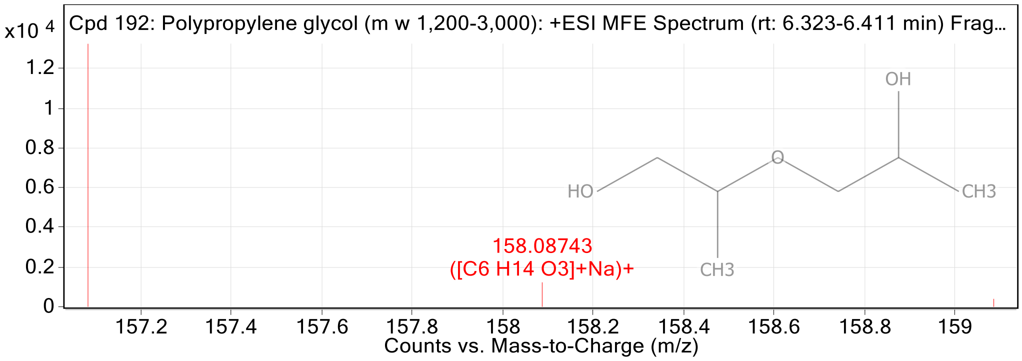

Peak List

m/z	z	Abund	Name	Formula	Ion	Score (DB)	Hits (DB)
157.08377	1	13252.33	Polypropylene glycol (m w 1,200-3,000)	C6 H14 O3	(M+Na)+	95.04	1
158.08743	1	1230.83		C6 H14 O3	(M+Na)+		
159.08528	1	398.71		C6 H14 O3	(M+Na)+		

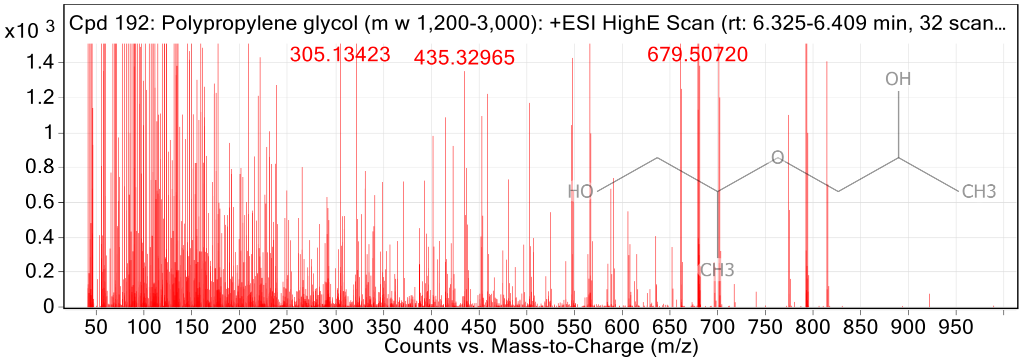

Peak List

m/z	z	Abund
43.01753		6734.98
45.03324	1	52380.2
55.05376		7736.33
58.06468		6680.61
59.04863		9485.9
89.05851	1	40158.56
114.08953		8014.21
133.08421	1	21442.9
679.5072	1	14658.73
792.59099	1	7867.84

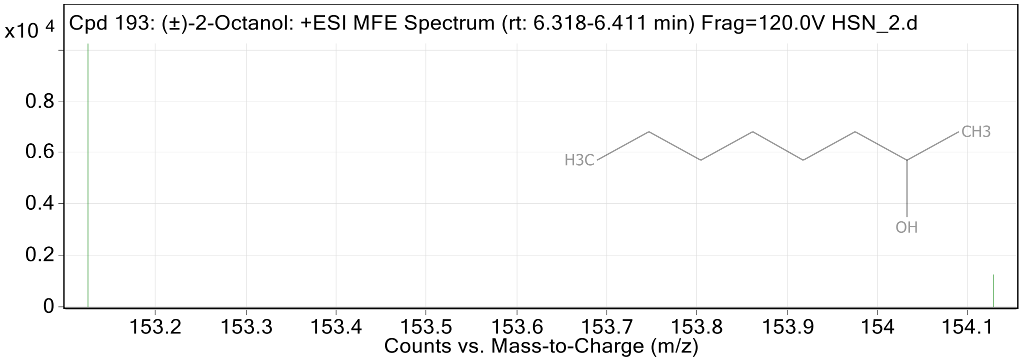

Peak List

m/z	z	Abund	Name	Formula	Ion	Score (DB)	Hits (DB)
153.1254	1	10251.01	(±)-2-Octanol	C8 H18 O	(M+Na)+	83.16	10
154.12861	1	1257.8		C8 H18 O	(M+Na)+		

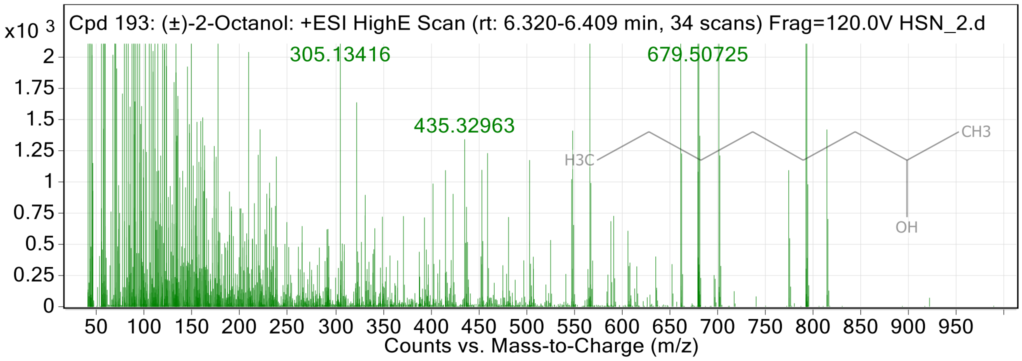

Peak List

m/z	z	Abund
43.01753		6671.81
45.03324	1	52299.5
55.05375		7797.53
58.06467		6576.46
59.04862		10385.51
89.05851	1	40132.95
114.08955		7866.49
133.08421	1	21447.7
679.50725	1	14436.15
792.59094	1	7763.53

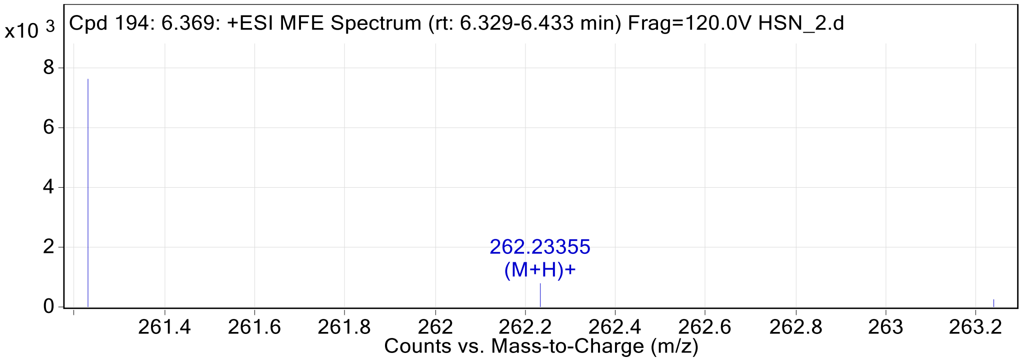

Peak List

m/z	z	Abund	Ion
261.23007	1	7630.93	(M+H)+
262.23355	1	792.94	(M+H)+
263.23943	1	256.01	(M+H)+

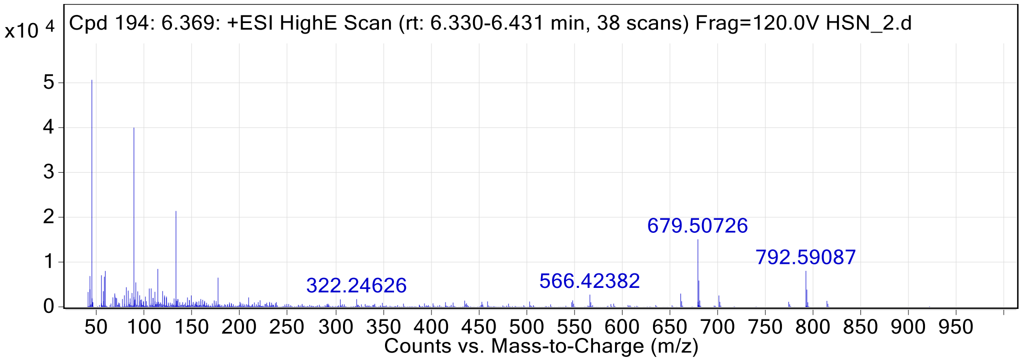

Peak List

m/z	z	Abund
43.01753		6861.38
45.03323	1	50510.23
55.05377		7036.81
58.0647		6710.7
59.04861		7990.35
89.05852	1	39884.88
114.08951		8442.42
133.08421	1	21337.1
679.50726	1	15030.37
792.59087	1	8015.98

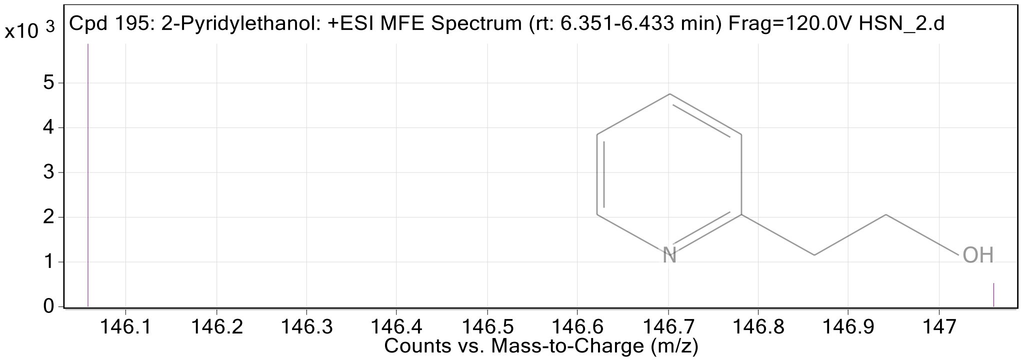

Peak List

m/z	z	Abund	Name	Formula	Ion	Score (DB)	Hits (DB)
146.05796	1	5879.18	2-Pyridylethanol	C7 H9 N O	(M+Na)+	86.55	10
147.06052	1	529.64		C7 H9 N O	(M+Na)+		

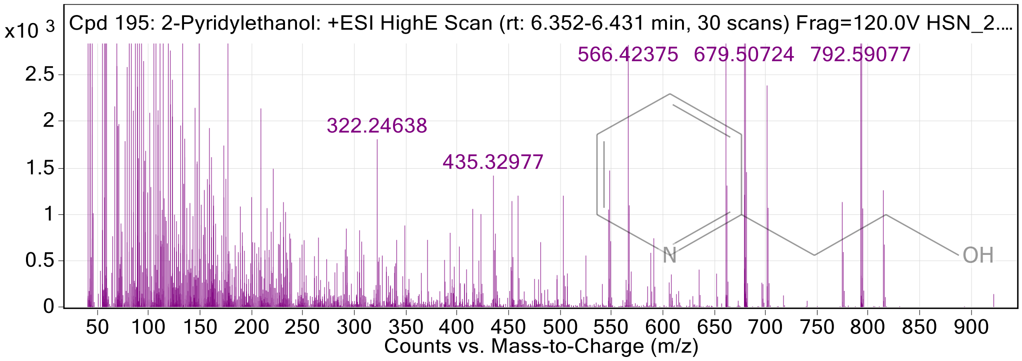

Peak List

m/z	z	Abund
43.01751		6995.87
45.03322		48088.36
55.05374		6244.72

58.0647		7226.79
89.05852	1	39683.2
114.0895		8808.28
133.08421	1	21437.71
177.10998		6559.73
679.50724	1	15621.49
792.59077	1	8295.53

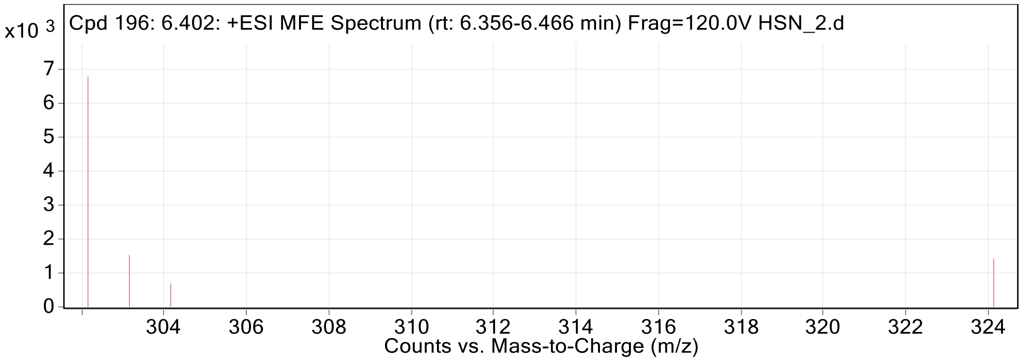

Peak List

m/z	z	Abund	Ion
302.15711	1	6771.32	(M+H)+
303.16157	1	1532.22	(M+H)+
304.16598	1	687.17	(M+H)+
324.13781	1	1422.44	(M+Na)+

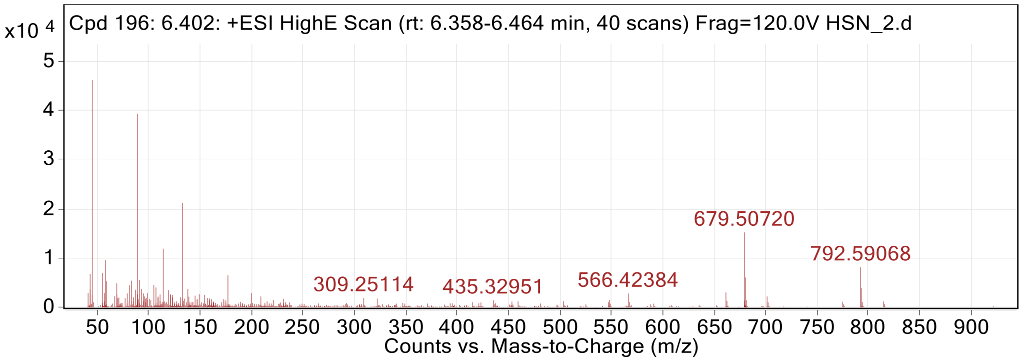

Peak List

m/z	z	Abund
43.01751		6762.88
45.03321		46209.89
55.05375		6941.55
58.06463		9568.98
89.05853	1	39355.87
114.08951		11887.16
133.08421	1	21230.37
177.11		6453.92
679.5072	1	15200
792.59068	1	8117.16

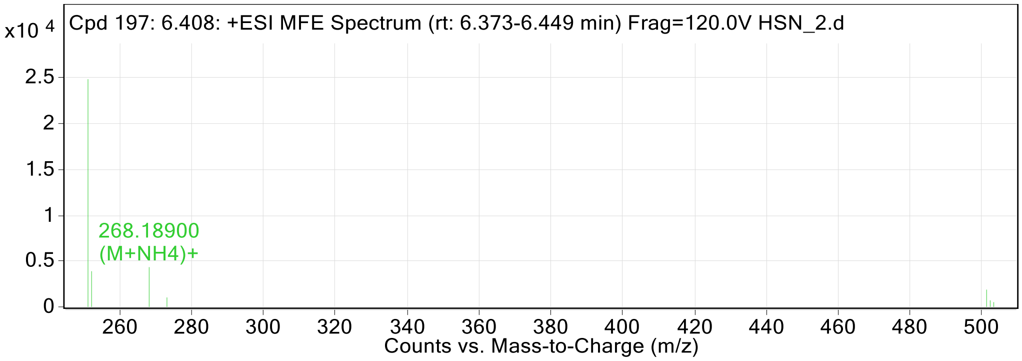

Peak List

m/z	z	Abund	Ion
251.16138	1	24844.52	(M+H)+
252.16478	1	3892.66	(M+H)+
268.189	1	4338.74	(M+NH4)+
273.1442	1	1029.64	(M+Na)+
501.31668	1	1882.03	(2M+H)+
502.31758	1	712.77	(2M+H)+
503.31454	1	518.33	(2M+H)+

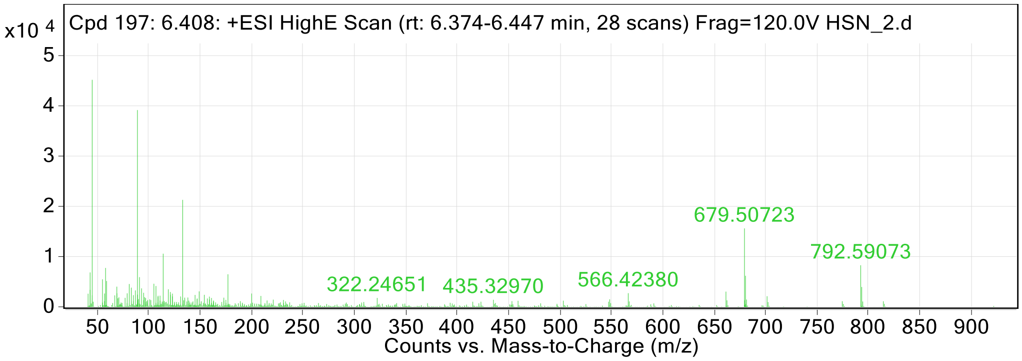

Peak List

m/z	z	Abund
43.0175		6841.36
45.0332		45128.59
58.06463		7732.65
89.05853	1	39114.5
114.08951		10563.17
133.08418	1	21263.2
177.11001		6475.55
679.50723	1	15592.7
680.51063	1	6174.36
792.59073	1	8267.94

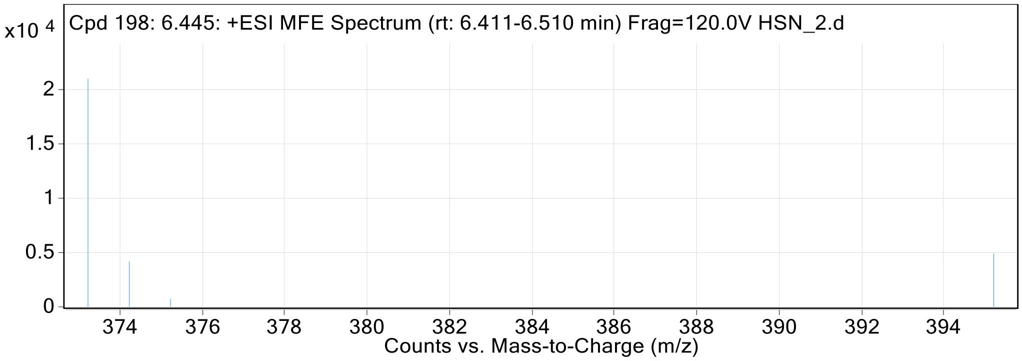

Peak List

m/z	z	Abund	Ion
373.23023	1	20959.93	(M+H)+
374.23311	1	4171.61	(M+H)+
375.23471	1	755.35	(M+H)+
395.21175	1	4905	(M+Na)+

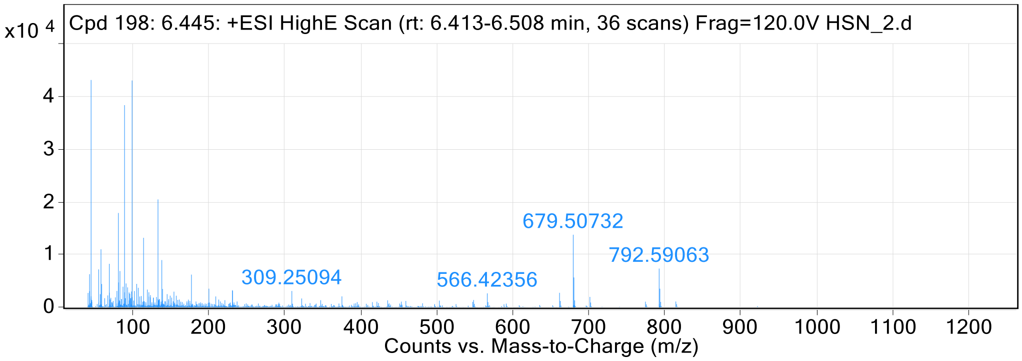

Peak List

m/z	z	Abund
45.0332		43155.17
58.0646		10976.36
69.03266		8211.1
80.97262		17873.15
89.05851	1	38375.02
98.98289		43061.54
114.08954		13155.37
133.08424	1	20450.7
138.12575	1	8925.17
679.50732	1	13741.16

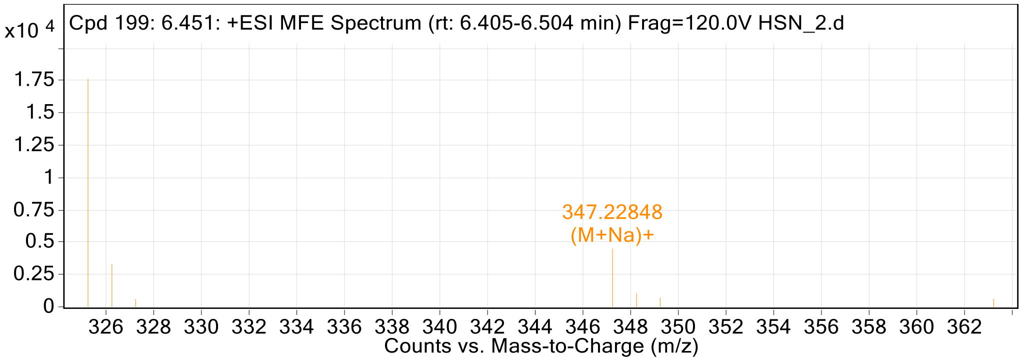

Peak List

m/z	z	Abund	Ion
325.24587	1	17648.19	(M+H)+

326.24931	1	3311.88	(M+H)+
327.24787	1	593.54	(M+H)+
347.22848	1	4511.94	(M+Na)+
348.23149	1	1040.67	(M+Na)+
349.22396	1	700.08	(M+Na)+
363.20237	1	608.48	(M+K)+

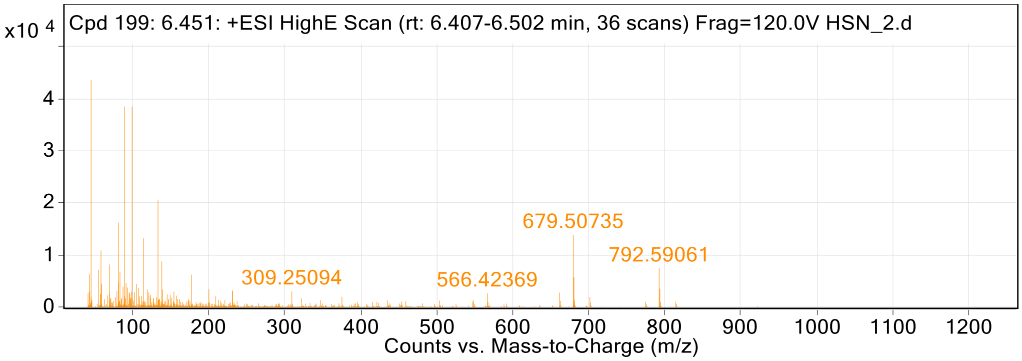

Peak List

m/z	z	Abund
45.0332		43586.02
58.0646		10836.75
69.03266		8145.6
80.97262		16193.22
89.05851	1	38467.9
98.9829		38463.49
114.08954		13199.03
133.08422	1	20458.86
138.12575	1	8760.67
679.50735	1	13852.94

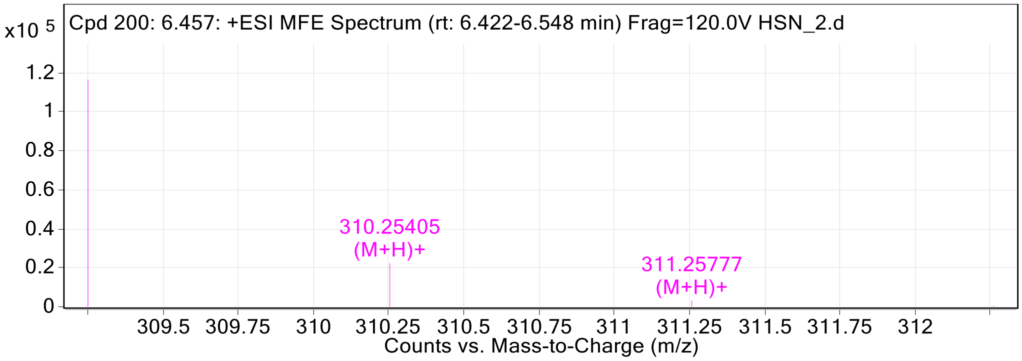

Peak List

m/z	z	Abund	Ion
309.25074	1	116449.63	(M+H)+
310.25405	1	22313.75	(M+H)+
311.25777	1	3088.11	(M+H)+
312.2613	1	520.26	(M+H)+

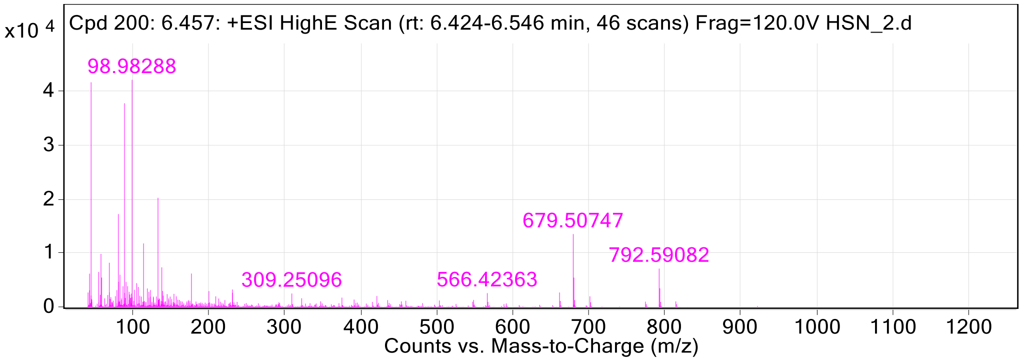

Peak List

m/z	z	Abund
45.03321		41660.89
58.06461		9829.98
69.03268		8187.06
80.97263		17216.24
89.05852	1	37766.87
98.98288		42113.81
114.08957		11766.79
133.08419	1	20241.14
138.12576		7331.81
679.50747	1	13506.19

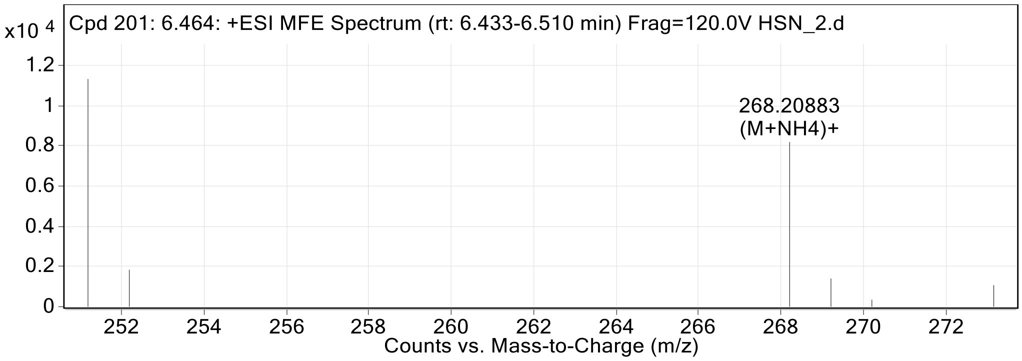

Peak List

m/z	z	Abund	Ion
251.18191	1	11324.81	(M+H)+
252.18505	1	1839.13	(M+H)+
268.20883	1	8194.16	(M+NH4)+
269.21288	1	1405.26	(M+NH4)+
270.20603	1	355.07	(M+NH4)+
273.16392	1	1077.49	(M+Na)+

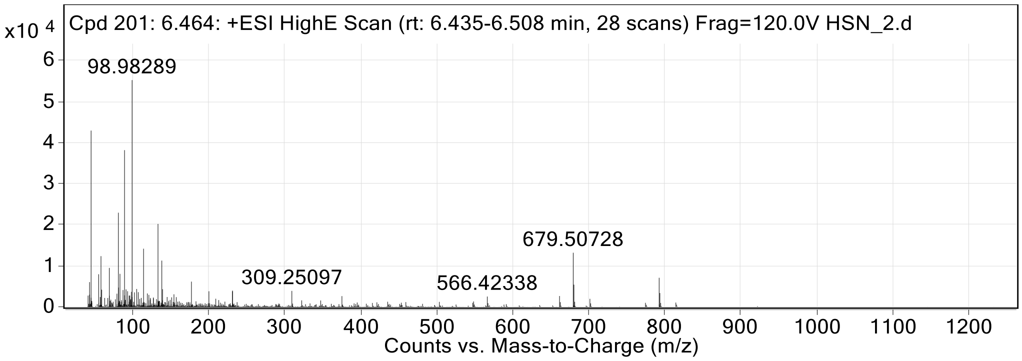

Peak List

m/z	z	Abund
45.03321		42919.57
58.06457		12337.28
69.03266		9471.72
80.97263		22916.16
89.0585	1	38151.4
98.98289		55213.08
114.08957		14145.93
133.08422	1	20175.11
138.12575	1	11251.86
679.50728	1	13164.6

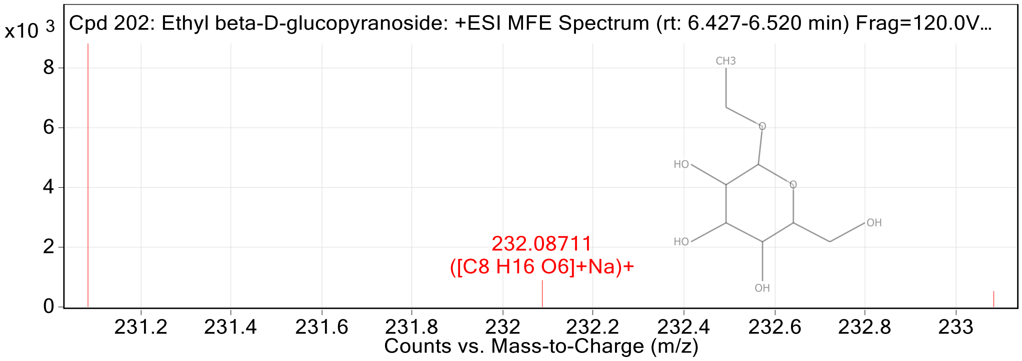

Peak List

m/z	z	Abund	Name	Formula	Ion	Score (DB)	Hits (DB)
231.08398	1	8822.58	Ethyl beta-D-glucopyranoside	C8 H16 O6	(M+Na)+	88.27	2
232.08711	1	899.59		C8 H16 O6	(M+Na)+		
233.08393	1	531.21		C8 H16 O6	(M+Na)+		

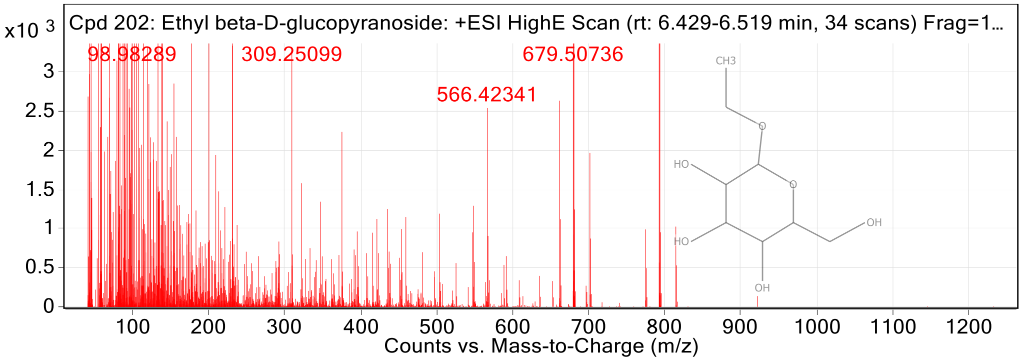

Peak List

m/z	z	Abund
45.0332		42456.24
58.06459		11347.13
69.03264		8581.75
80.97263		21124.54
89.05852	1	38016.7
98.98289		51562.98
114.08957		13056.58
133.08418	1	20205.92
138.12574	1	9596.91
679.50736	1	13388.33

Qualitative Analysis Report

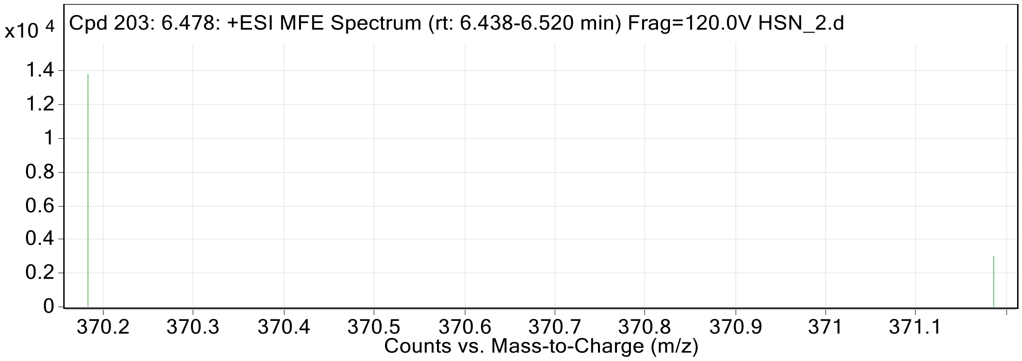

Peak List

m/z	z	Abund	Ion
370.18306	1	13824.39	(M+H)+
371.18612	1	3030.91	(M+H)+

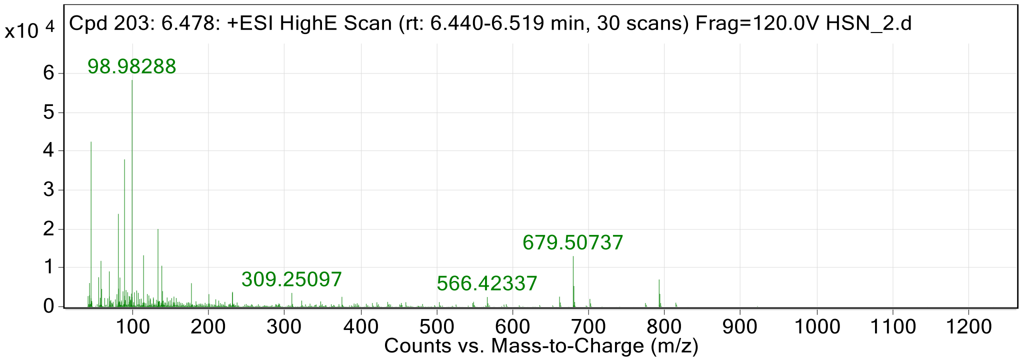

Peak List

m/z	z	Abund
45.03321		42447.42
58.06458		11791.39
69.03265		9087.75
80.97263		23880.65
89.05852	1	37906.41
98.98288		58314.1
114.08958		13233.58
133.08418	1	19990.29
138.12574	1	10537.05
679.50737	1	13017.21

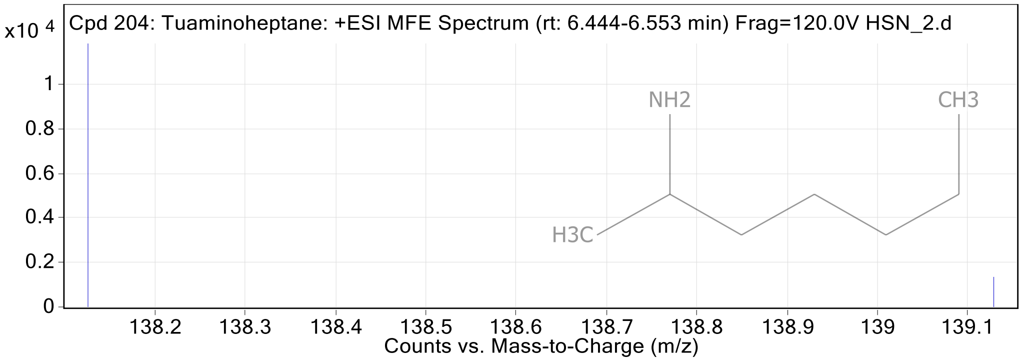

Peak List

m/z	z	Abund	Name	Formula	Ion	Score (DB)	Hits (DB)
138.1257	1	11823.31	Tuaminoheptane	C7 H17 N	(M+Na)+	83.48	3
139.12889	1	1349.12		C7 H17 N	(M+Na)+		

Qualitative Analysis Report

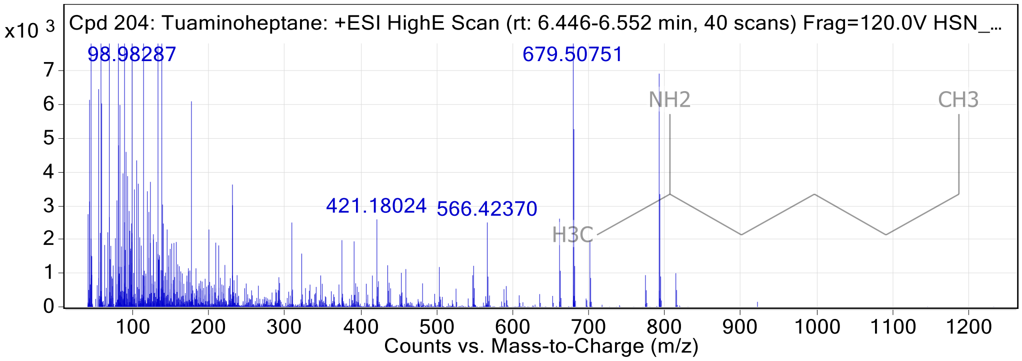

Peak List

m/z	z	Abund
45.03322		41098.31
58.06459		9925.72
69.03269		9000.5
80.97264		19905.81
89.05851	1	37460.74
98.98287		48719.54
114.0896		11375.7
133.08421	1	19977.05
138.12574		7814.85
679.50751	1	13018.33

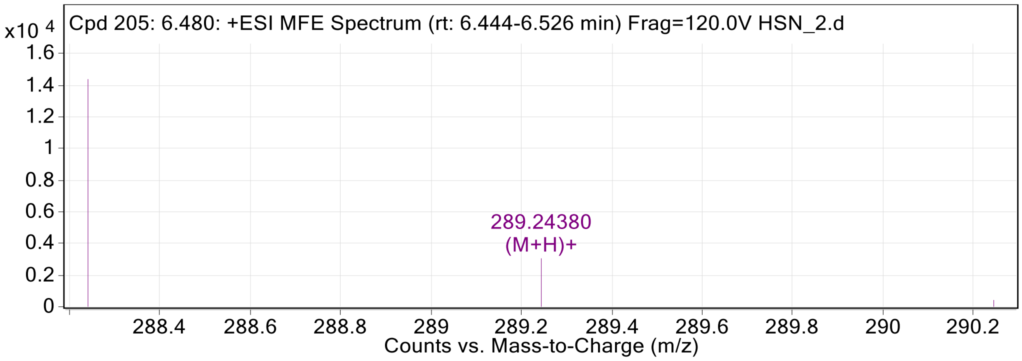

Peak List

m/z	z	Abund	Ion
288.24068	1	14390.75	(M+H)+
289.2438	1	3063.42	(M+H)+
290.24507	1	427.12	(M+H)+

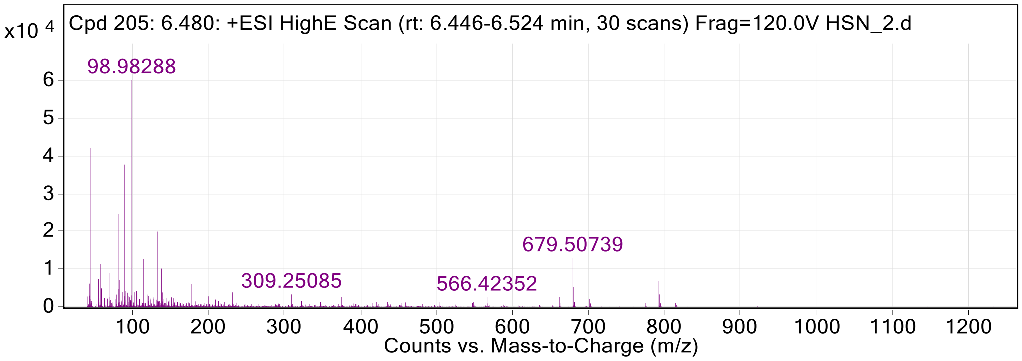

Peak List

m/z	z	Abund
45.03322		42147.6
58.06458		11277.73

69.03265		9000.54
80.97264		24657.46
89.05853	1	37709
98.98288		60161.7
114.08958		12699.03
133.08418	1	19954.57
138.12574	1	10130.83
679.50739	1	12920.97

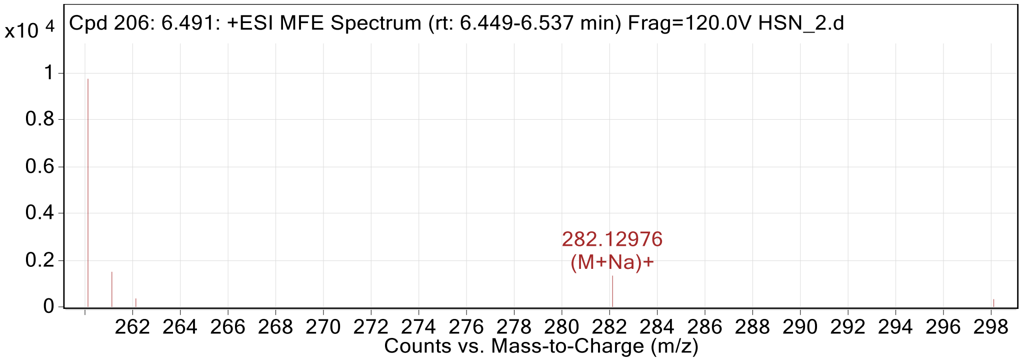

Peak List

m/z	z	Abund	Ion
260.14686	1	9745.71	(M+H)+
261.14867	1	1498.95	(M+H)+
262.15464	1	360.08	(M+H)+
282.12976	1	1335.21	(M+Na)+
298.10357	1	329.7	(M+K)+

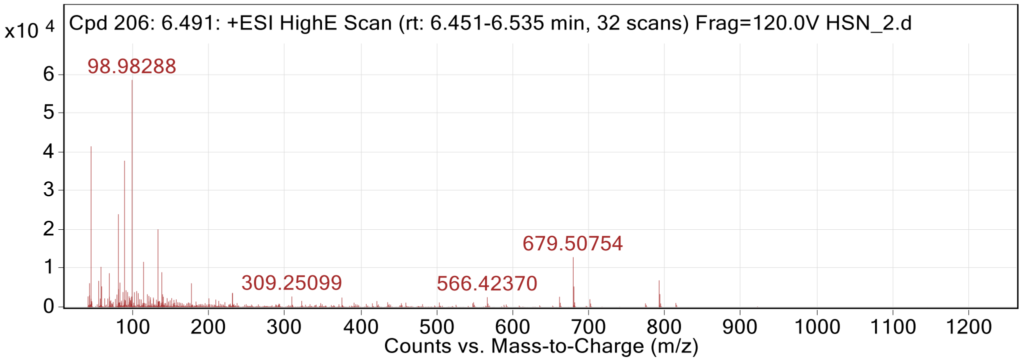

Peak List

m/z	z	Abund
45.03322		41484.86
58.06459		10355.24
69.03267		8712.6
80.97264		23951.51
89.05851	1	37774.04
98.98288		58635.53
114.08958		11644
133.08422	1	20053.14
138.12574	1	8966.16
679.50754	1	12863.19

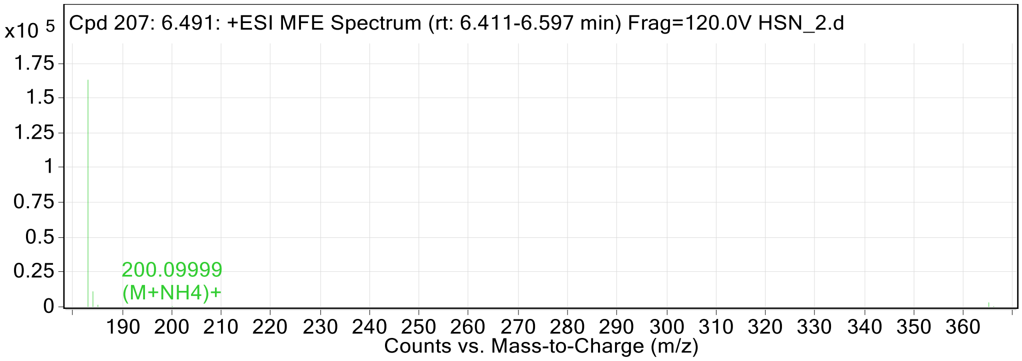

Peak List

m/z	z	Abund	Ion
183.07591	1	163141.23	(M+H)+
184.0791	1	11135.83	(M+H)+
185.08025	1	1481.99	(M+H)+
200.09999	1	516.24	(M+NH4)+
365.14766	1	3200.73	(2M+H)+
366.14802	1	480.96	(2M+H)+

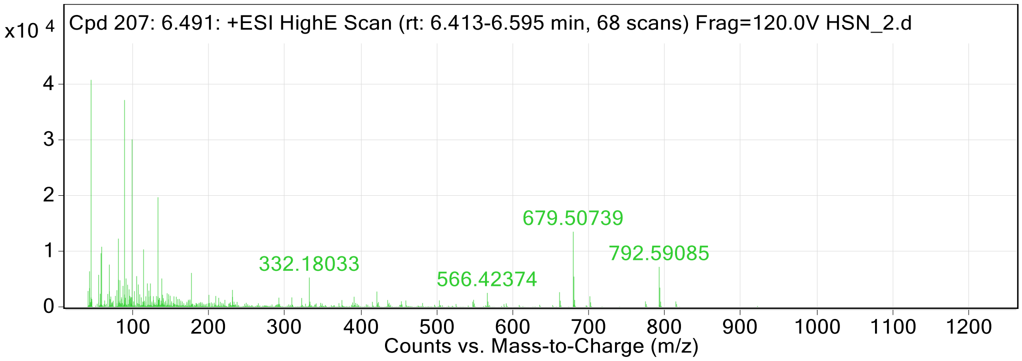

Peak List

m/z	z	Abund
45.0332		40883.18
58.06461		9662.06
59.04862		10827.26
69.0327		7611.7
80.97262		12293.86
89.05852	1	37255.95
98.98287		30160.99
114.08957		10356.42
133.08424	1	19741.33
679.50739	1	13539.57

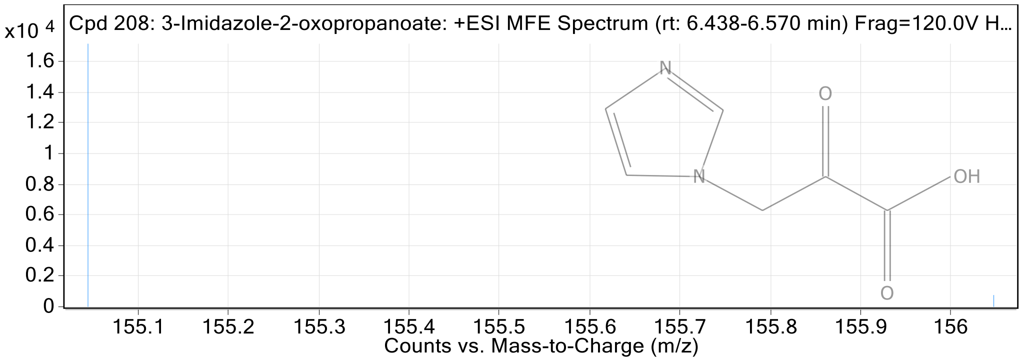

Peak List

Qualitative Analysis Report

m/z	z	Abund	Name	Formula	Ion	Score (DB)	Hits (DB)
155.04448	1	17159.11	3-Imidazole-2-oxopropanoate	C6 H6 N2 O3	(M+H)+	82.37	6
156.04729	1	766.14		C6 H6 N2 O3	(M+H)+		

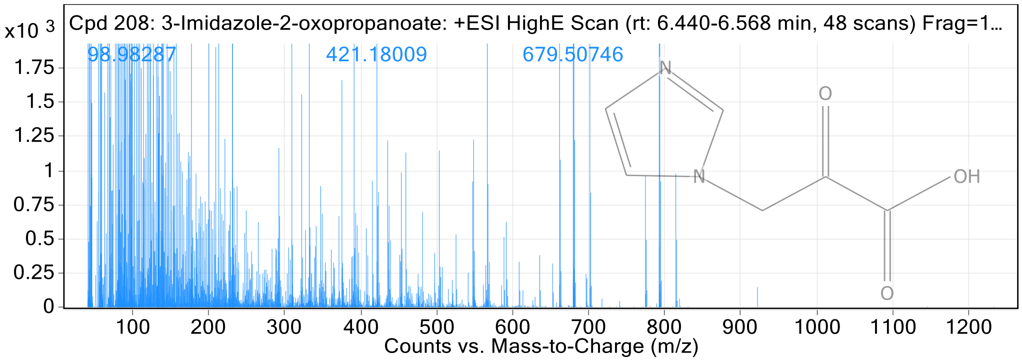

Peak List

m/z	z	Abund
45.03321		40872.85
58.0646		10167.17
59.04862		7485.65
69.0327		8938.87
80.97263		16958.28
89.05851	1	37387.3
98.98287		41531.23
114.08958		11008.47
133.08419	1	19803.6
679.50746	1	13044.66

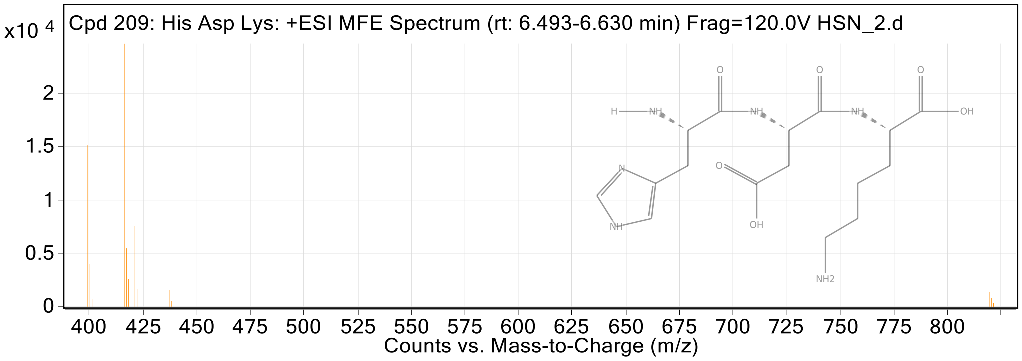

Peak List

m/z	z	Abund	Name	Formula	Ion	Score (DB)	Hits (DB)
399.19857	1	15183.17			(M+H)+		
400.20194	1	4005.64			(M+H)+		
416.22478	1	24753.7	His Asp Lys	C16 H26 N6 O6	(M+NH4)+	78.82	6
417.22814	1	5493.73		C16 H26 N6 O6	(M+NH4)+		
418.23756	1	2593.33		C16 H26 N6 O6	(M+NH4)+		
421.18022	1	7606.73			(M+Na)+		
422.1837	1	1667.2			(M+Na)+		
437.15433	1	1589.22			(M+K)+		
819.37445	1	1364.94			(2M+Na)+		
820.37907	1	793.37			(2M+Na)+		

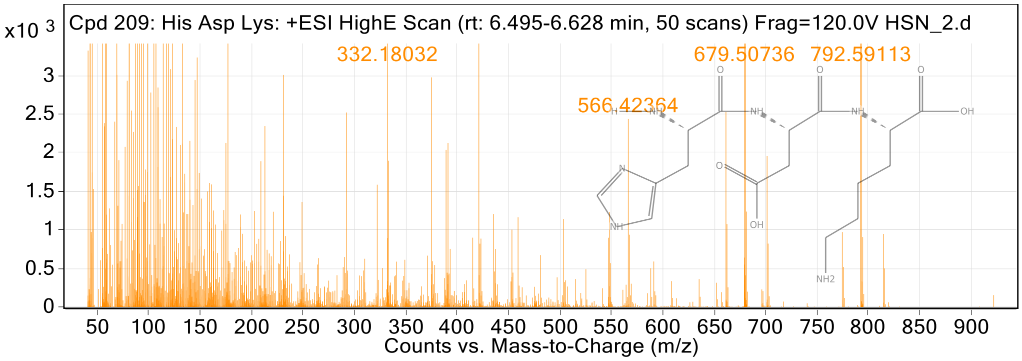

Peak List

m/z	z	Abund
45.0332		38111.88
58.06463		7394.36
59.04864		20522.74
69.03274		9226.93
80.97261		8865.61
89.05852	1	35774.28
98.98285		22353.46
133.0843	1	18827.07
332.18032	1	10360.45
679.50736	1	13159.67

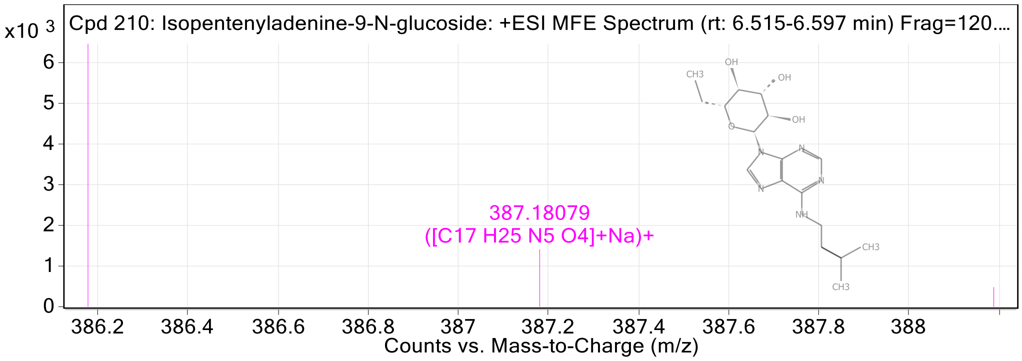

Peak List

m/z	z	Abund	Name	Formula	Ion	Score (DB)	Hits (DB)
386.17823	1	6459.77	Isopentenyladenine-9-N-glucoside	C17 H25 N5 O4	(M+Na)+	84.01	1
387.18079	1	1411.36		C17 H25 N5 O4	(M+Na)+		
388.18855	1	479.68		C17 H25 N5 O4	(M+Na)+		

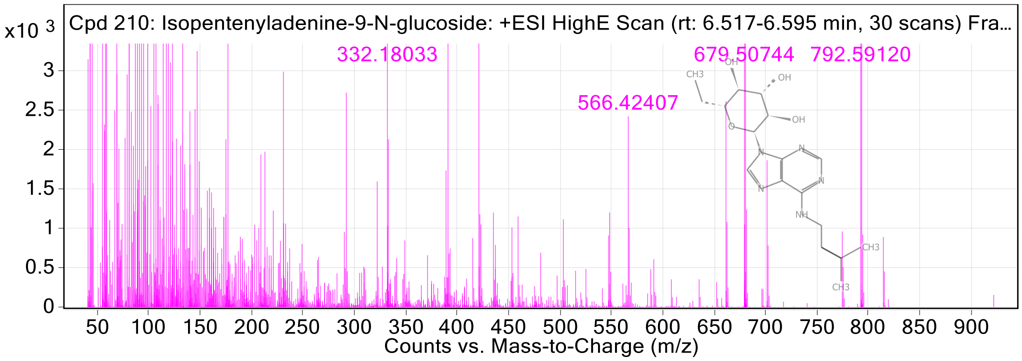

Peak List

m/z	z	Abund
45.03319		38135.44

58.06462		8332.49
59.04864		18896.47
69.03277		7101.31
89.05852	1	35940.51
98.98277		12765.98
114.0896		7274.28
133.08427	1	18943.41
332.18033	1	11933.05
679.50744	1	13312.76

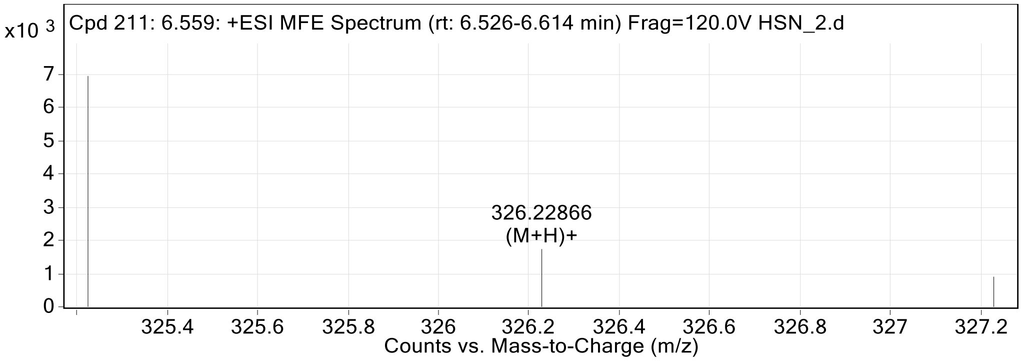

Peak List

m/z	z	Abund	Ion
325.22456	1	6941.28	(M+H)+
326.22866	1	1735.36	(M+H)+
327.22882	1	905.25	(M+H)+

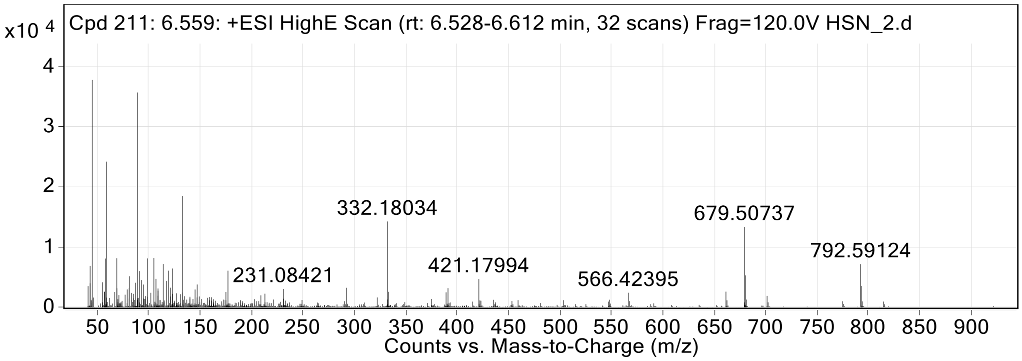

Peak List

m/z	z	Abund
45.0332		37723.4
58.06462		8051.75
59.04864		24163.39
69.03279		8091.9
89.05851	1	35650.49
98.98278		8067.74
105.06838		8159.71
133.08437		18462.5
332.18034	1	14214.02
679.50737	1	13321.01

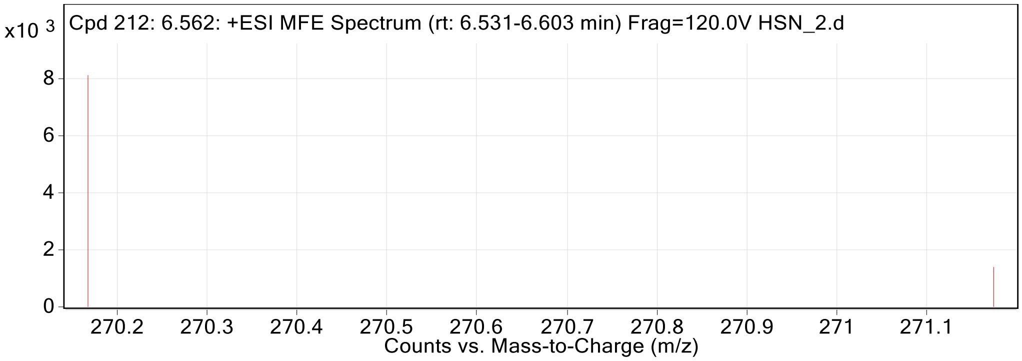

Peak List

m/z	z	Abund	Ion
270.16794	1	8117.2	(M+H)+
271.17415	1	1398.82	(M+H)+

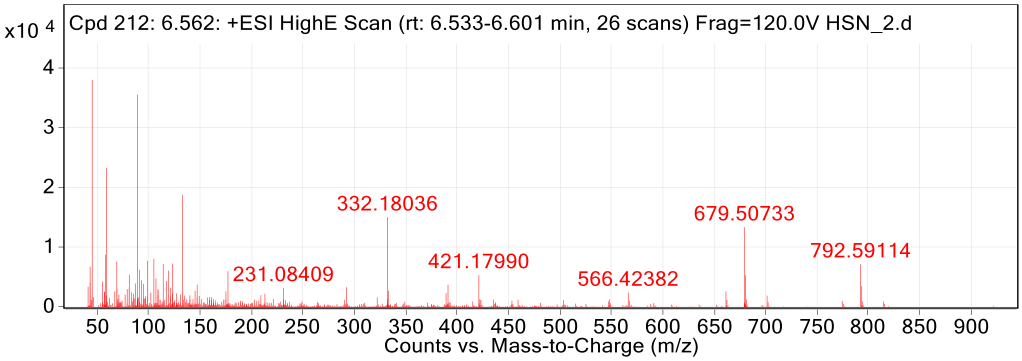

Peak List

m/z	z	Abund
45.03319		37979.93
58.0646		8708.54
59.04864		23241.02
69.03277		7583.14
89.05851	1	35542.09
98.98277		7699.75
105.06839		8025.43
133.08437		18716.9
332.18036	1	14995.69
679.50733	1	13332.85

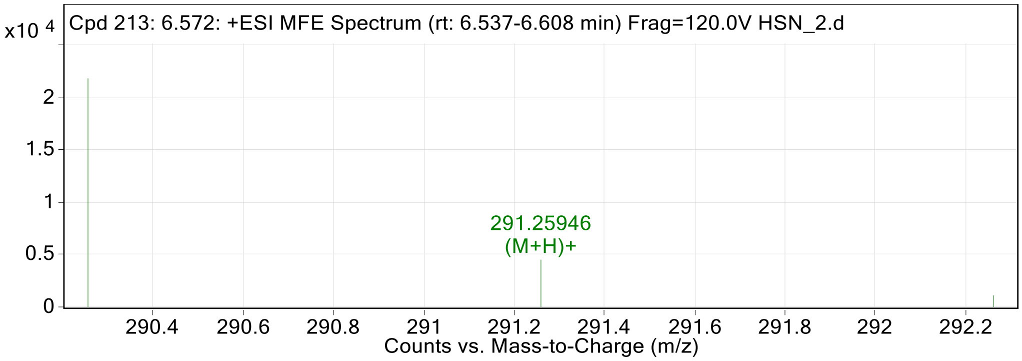

Peak List

m/z	z	Abund	Ion
290.25631	1	21812.13	(M+H)+
291.25946	1	4513.76	(M+H)+
292.26227	1	1114.51	(M+H)+

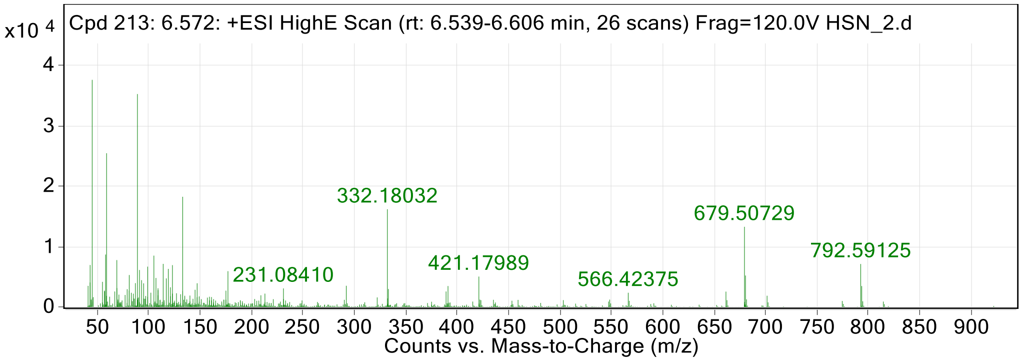

Peak List

m/z	z	Abund
45.03319		37662.02
58.06461		8681.88
59.04864		25486.57
69.03278		7762.38
89.05853	1	35306.07
105.06839		8503.12
114.0896		7134.36
133.08436		18264.07
332.18032	1	16195.39
679.50729	1	13285.67

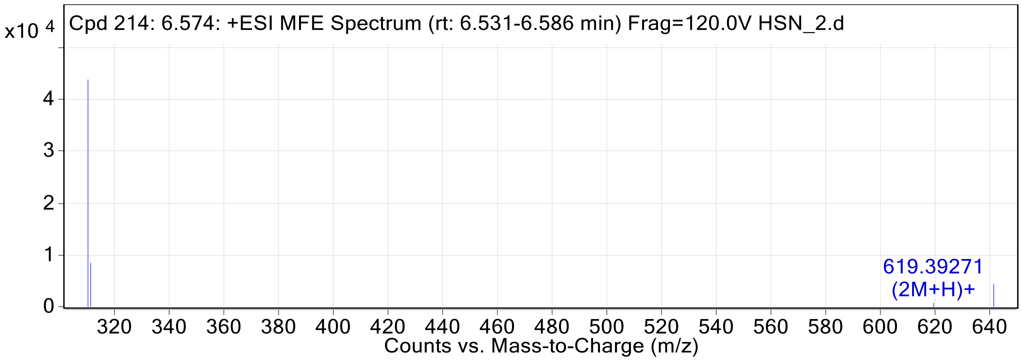

Peak List

m/z	z	Abund	Ion
310.19823	1	43878.4	(M+H)+
311.20154	1	8482.85	(M+H)+
619.39271	1	733.78	(2M+H)+
641.3724	1	4386.84	(2M+Na)+

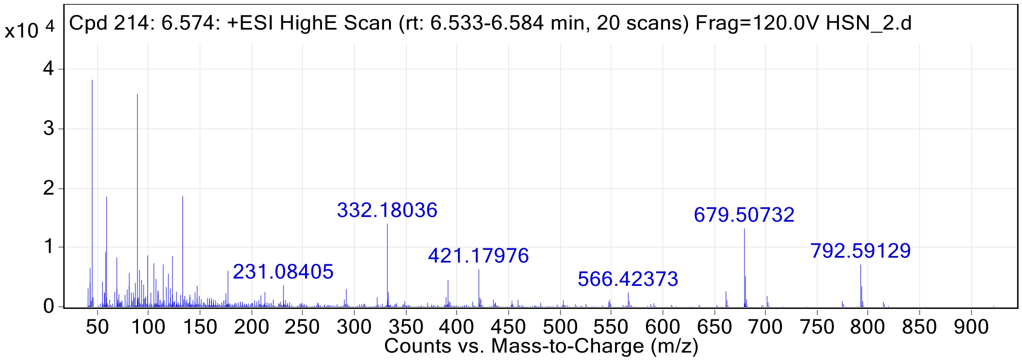

Peak List

m/z	z	Abund
45.03318		38254.7

58.06463		9203.08
59.04865		18592.77
69.03278		8312.14
89.05849	1	35892.92
98.98275		8680.24
123.11501		8546.58
133.08428	1	18709
332.18036	1	14001.14
679.50732	1	13210.95

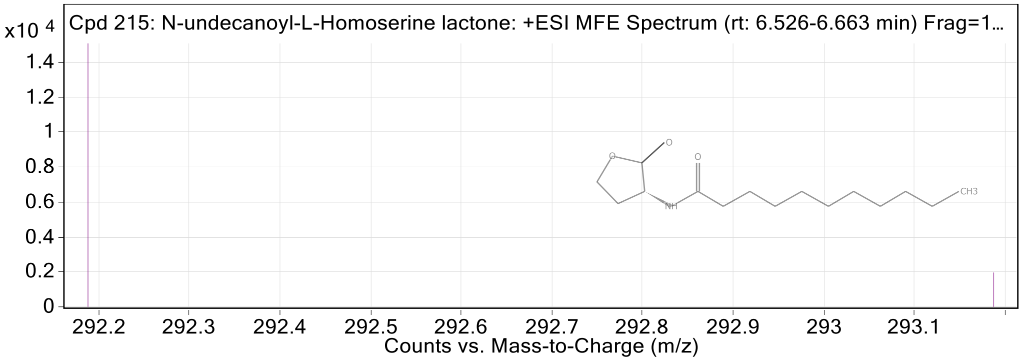

Peak List

m/z	z	Abund	Name	Formula	Ion	Score (DB)	Hits (DB)
292.18811	1	15083.59	N-undecanoyl-L-Homoserine lactone	C15 H27 N O3	(M+Na)+	73.67	2
293.18783	1	1964.31		C15 H27 N O3	(M+Na)+		

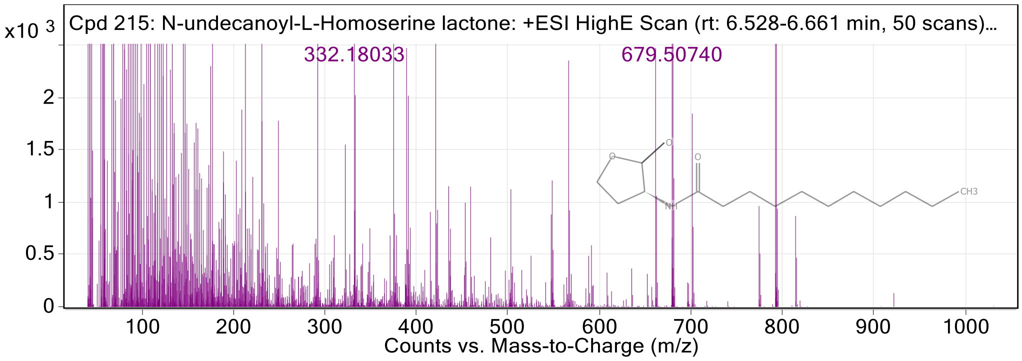

Peak List

m/z	z	Abund
43.01756		7042.12
45.0332		37271.36
59.04864		22885.97
69.03276		10878.83
89.05852	1	35119.39
105.06834		8036.14
133.08436		18552.94
332.18033	1	11012.28
679.5074	1	13068.52
792.59118	1	6988.38

Qualitative Analysis Report

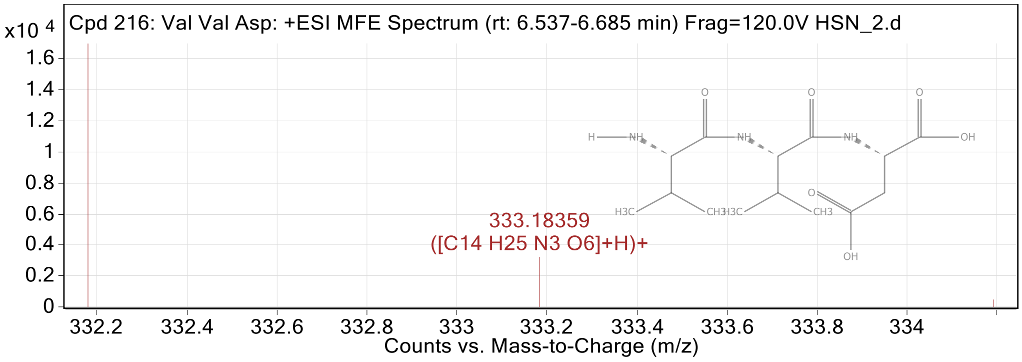

Peak List

m/z	z	Abund	Name	Formula	Ion	Score (DB)	Hits (DB)
332.18063	1	17002.53	Val Val Asp	C14 H25 N3 O6	(M+H)+	94.71	10
333.18359	1	3227.2		C14 H25 N3 O6	(M+H)+		
334.19244	1	474.54		C14 H25 N3 O6	(M+H)+		

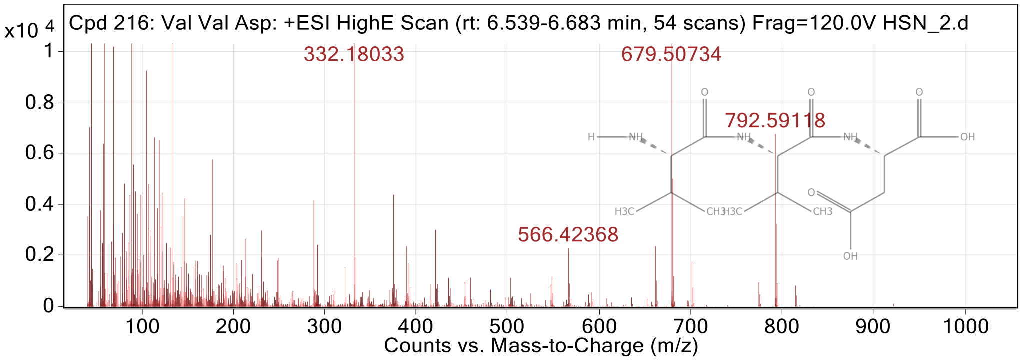

Peak List

m/z	z	Abund
43.01756		7039.46
45.03318		36703.37
59.04863		21637.82
69.03275		10196.36
89.05854	1	34389.45
105.06831		9254.19
133.08437		18039.67
332.18033	1	10327.19
679.50734	1	12576.4
792.59118	1	6765.8

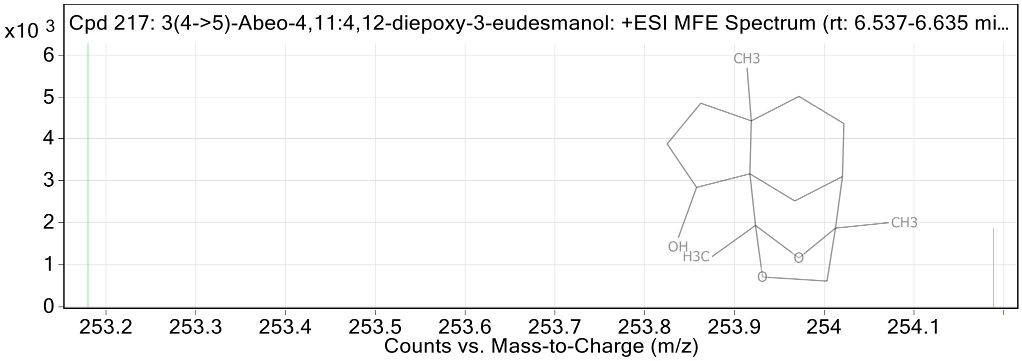

Peak List

m/z	z	Abund	Name	Formula	Ion	Score (DB)	Hits (DB)
253.17914	1	6282.25	3(4->5)-Abeo-4,11:4,12-diepoxy-3-eudesmanol	C15 H24 O3	(M+H)+	53.98	10

254.18895	1	1876.54		C15 H24 O3	(M+H)+		

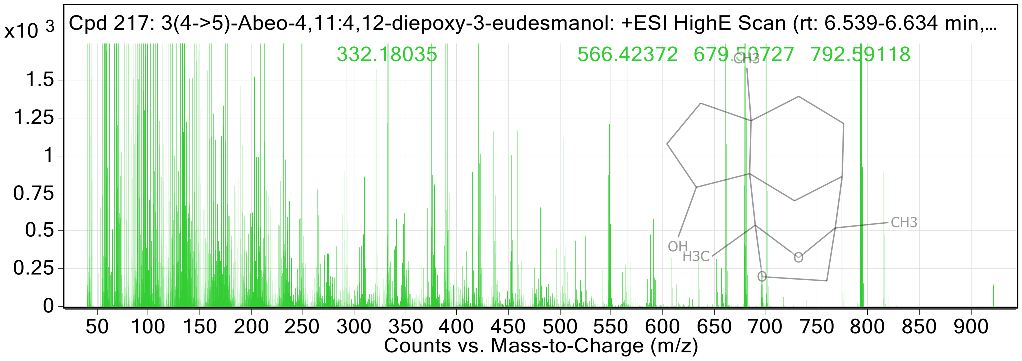

Peak List

m/z	z	Abund
43.01755		7148.79
45.03319		37179.33
58.06461		7505.15
59.04864		26658.66
69.03276		11554.5
89.05852	1	34962.76
105.06834		8786.06
133.08435		18130.29
332.18035	1	14632.31
679.50727	1	13053.04

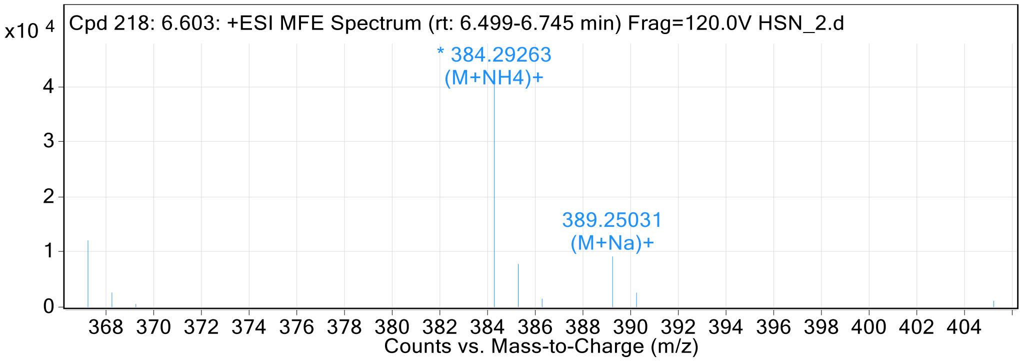

Peak List

m/z	z	Abund	Ion
367.26637	1	12109.2	(M+H)+
368.26991	1	2641.29	(M+H)+
369.27273	1	551.82	(M+H)+
384.29263	1	41305.93	(M+NH4)+
385.2964	1	7819.71	(M+NH4)+
386.29701	1	1519.37	(M+NH4)+
389.25031	1	9197.55	(M+Na)+
390.25315	1	2620.97	(M+Na)+
405.22315	1	1133.15	(M+K)+

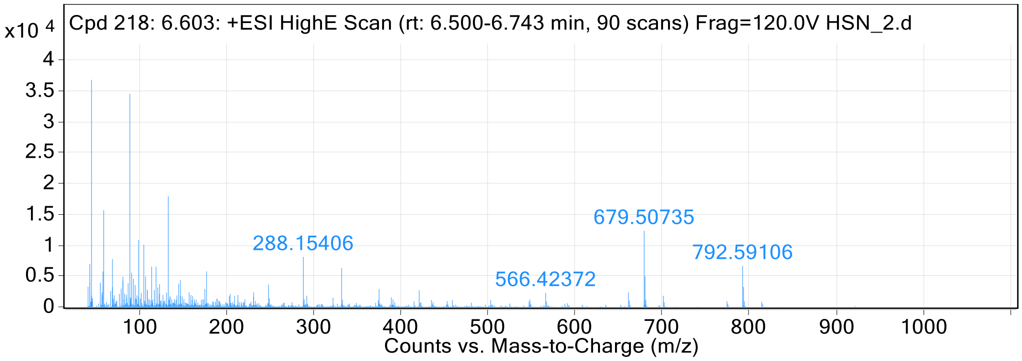

Peak List

m/z	z	Abund
43.01756		6981.02
45.03319		36819
59.04862		15678.03
69.03274		7758.27
89.05852	1	34534.9
98.98281		10852.14
105.06828		10079.76
133.08439		17932.96
288.15406	1	8110.15
679.50735	1	12344.98

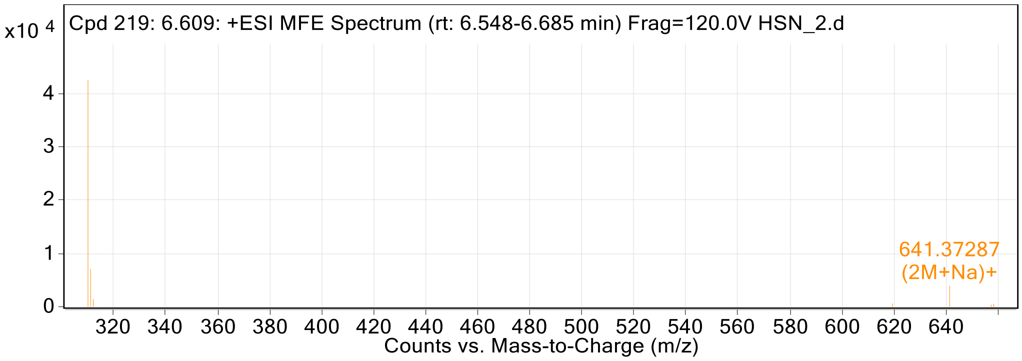

Peak List

m/z	z	Abund	Ion
310.19897	1	42598.16	(M+H)+
311.20163	1	7096.23	(M+H)+
312.20623	1	1416.64	(M+H)+
619.39062	1	625.81	(2M+H)+
641.37287	1	3934.13	(2M+Na)+
657.34323	1	410.03	(2M+K)+
658.35387	1	561.8	(2M+K)+

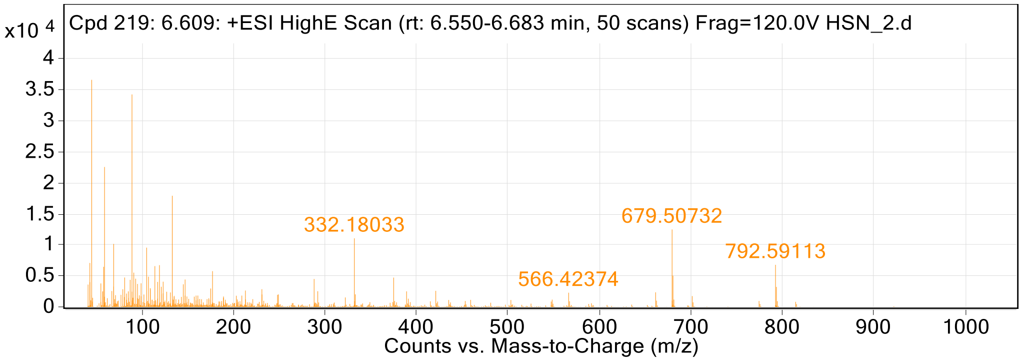

Peak List

m/z	z	Abund
43.01755		7088.67
45.03318		36623.2
59.04864		22563.99
69.03274		10153.91
89.05854	1	34269.79
105.0683		9555.53
133.08439		17926.03
332.18033	1	11058.43
679.50732	1	12487.39
792.59113	1	6785.08

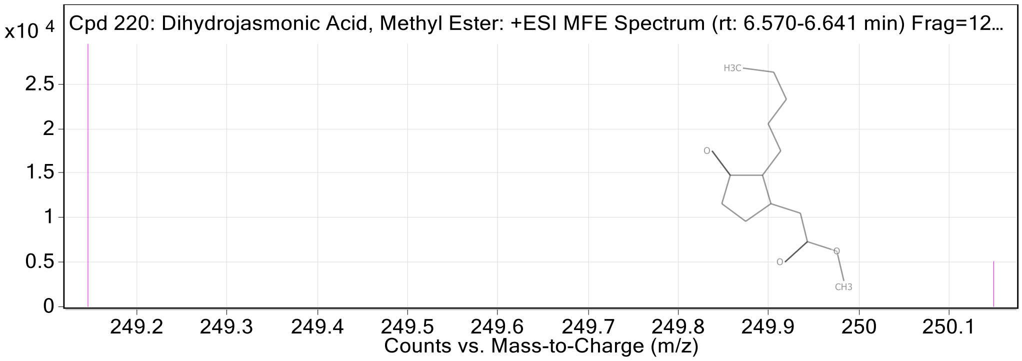

Peak List

m/z	z	Abund	Name	Formula	Ion	Score (DB)	Hits (DB)
249.14627	1	29522.2	Dihydrojasmonic Acid, Methyl Ester	C13 H22 O3	(M+Na)+	84.04	3
250.14915	1	5122.86		C13 H22 O3	(M+Na)+		

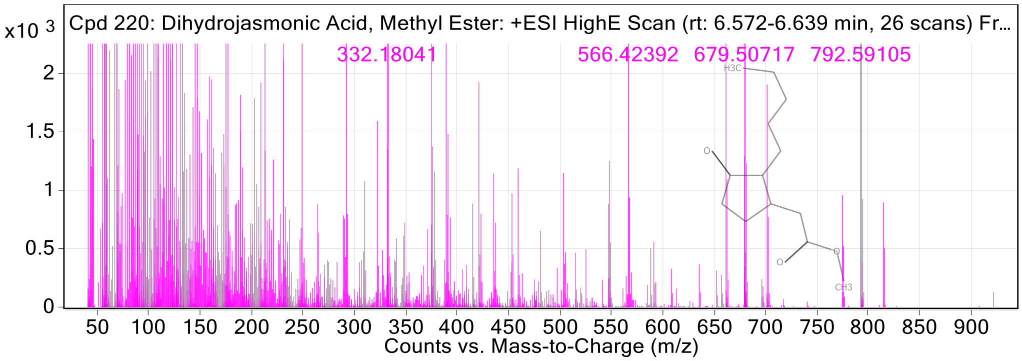

Peak List

m/z	z	Abund
43.01753		7480.08
45.03318		36692.13

59.04864		31779.14
69.03276		12603.43
89.05853	1	34501.38
105.0683		9599.99
133.08441		17844.94
332.18041	1	15939.1
679.50717	1	13090.96
792.59105	1	7154.68

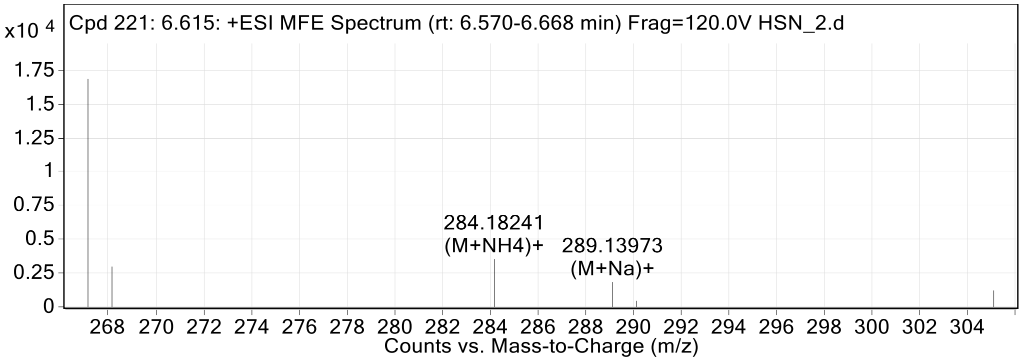

Peak List

m/z	z	Abund	Ion
267.15683	1	16856.43	(M+H)+
268.16308	1	2978.6	(M+H)+
284.18241	1	3537.61	(M+NH4)+
289.13973	1	1842.15	(M+Na)+
290.14191	1	449.64	(M+Na)+
305.11289	1	1212.49	(M+K)+

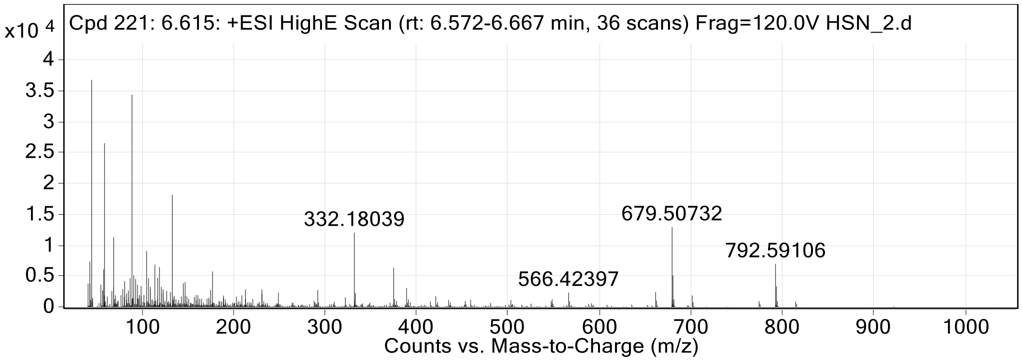

Peak List

m/z	z	Abund
43.01753		7372.75
45.03319		36772.54
59.04863		26509.98
69.03275		11291.29
89.05855	1	34378.87
105.0683		9050.64
133.0844		18143.23
332.18039	1	12017.28
679.50732	1	12925.86
792.59106	1	6945.8

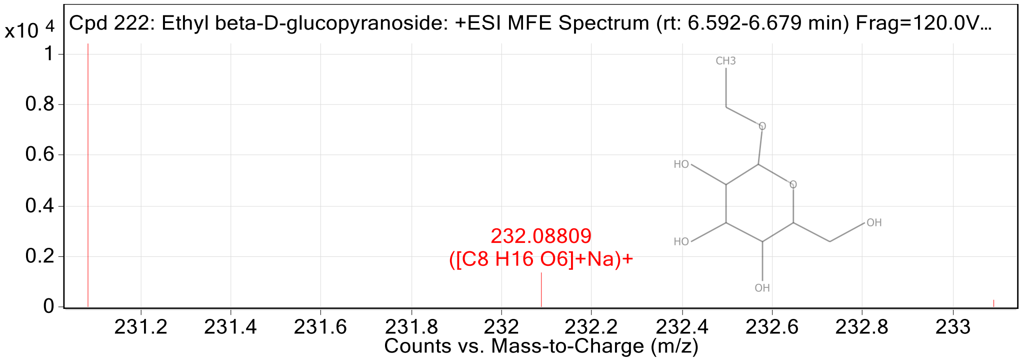

Peak List

m/z	z	Abund	Name	Formula	Ion	Score (DB)	Hits (DB)
231.08385	1	10418.33	Ethyl beta-D-glucopyranoside	C8 H16 O6	(M+Na)+	93.97	2
232.08809	1	1359.66		C8 H16 O6	(M+Na)+		
233.09007	1	280.33		C8 H16 O6	(M+Na)+		

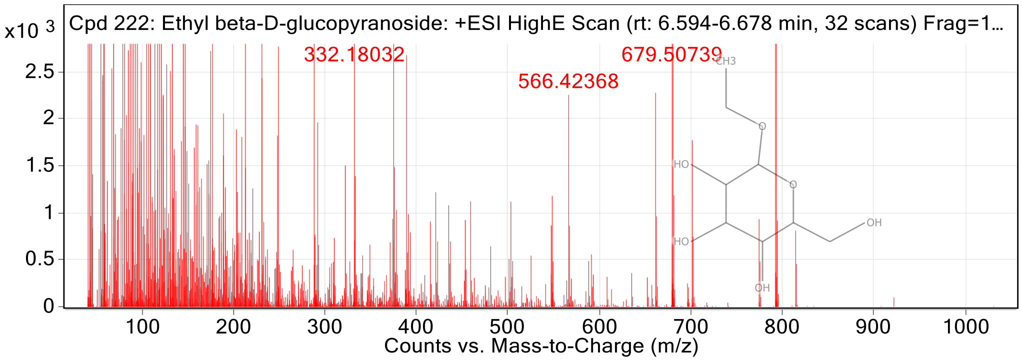

Peak List

m/z	z	Abund
43.01758		7333.29
45.03319		36046.56
59.04863		22755.35
69.03273		11994.05
89.05855	1	33806.05
105.06828		9462.24
133.08442		17815.07
332.18032	1	7378.25
375.13845	1	7250.29
679.50739	1	12363.37

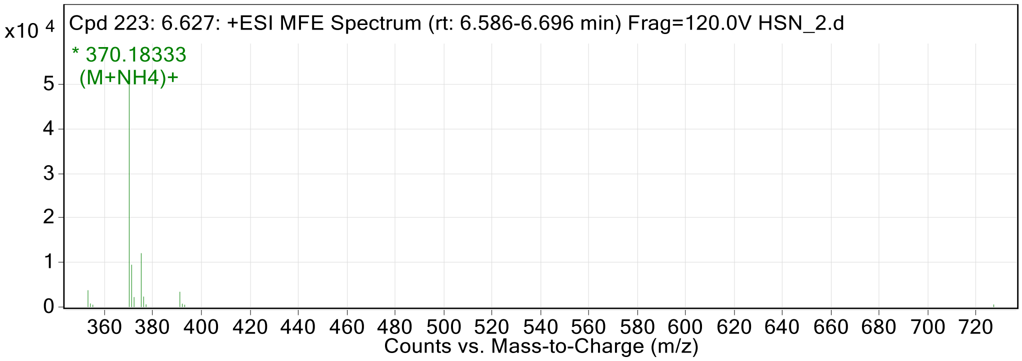

Peak List

m/z	z	Abund	Ion
353.15665	1	3769	(M+H)+

354.16175	1	795.04	(M+H)+
370.18333	1	51017.81	(M+NH4)+
371.18677	1	9487.86	(M+NH4)+
372.19037	1	2230.45	(M+NH4)+
375.13855	1	12084.02	(M+Na)+
376.14199	1	2340.81	(M+Na)+

m/z	z	Abund	Ion
377.14525	1	592.89	(M+Na)+
391.1125	1	3422.45	(M+K)+
392.11529	1	745.61	(M+K)+

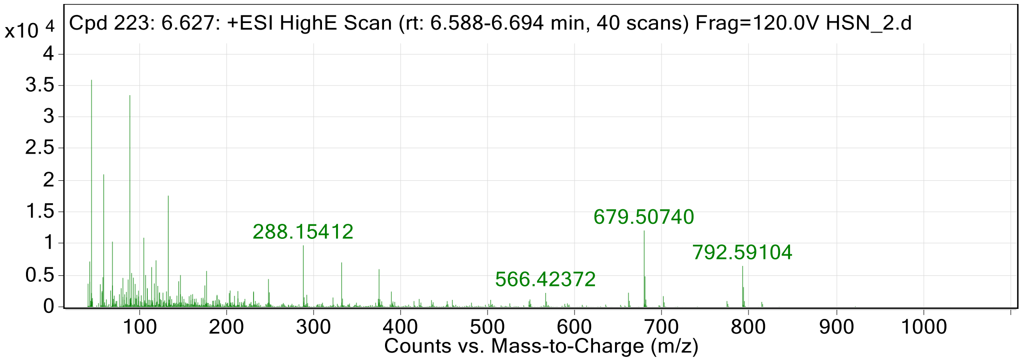

Peak List

m/z	z	Abund
43.01755		7160.53
45.03319		35861.1
59.04862		20926.12
69.03274		10298.82
89.05855	1	33438.52
105.06829		10917.18
119.08368		7344.55
133.08444		17568.36
288.15412	1	9715.07
679.5074	1	12060

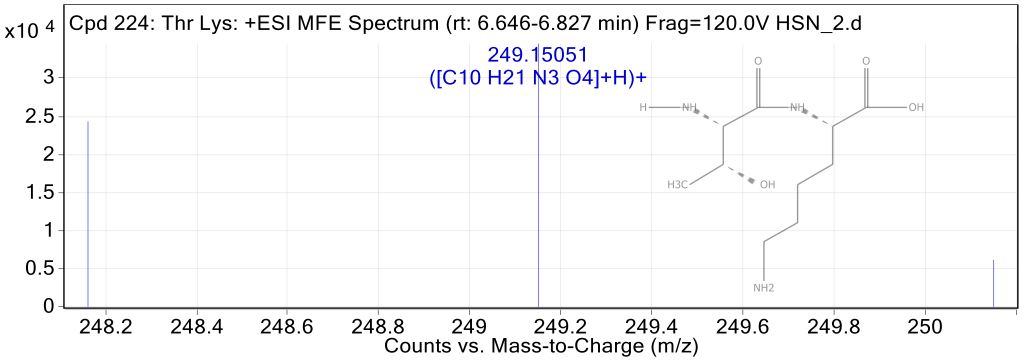

Peak List

m/z	z	Abund	Name	Formula	Ion	Score (DB)	Hits (DB)
248.16167	1	24328.03	Thr Lys	C10 H21 N3 O4	(M+H)+	5	4
249.15051	1	34534.29		C10 H21 N3 O4	(M+H)+		
250.15033	1	6176.81		C10 H21 N3 O4	(M+H)+		

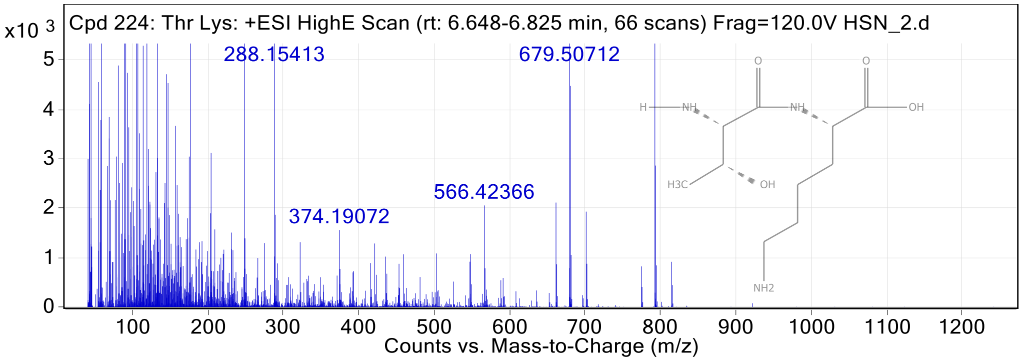

Peak List

m/z	z	Abund
43.01751		7244.76
45.03316		35486.01
59.04854		6997.91
89.0585	1	33122.44
105.06826		11492.92
119.08358		7142.2
133.08446		16928.34
288.15413	1	11780.86
679.50712	1	10917.78
792.59084	1	5925.95

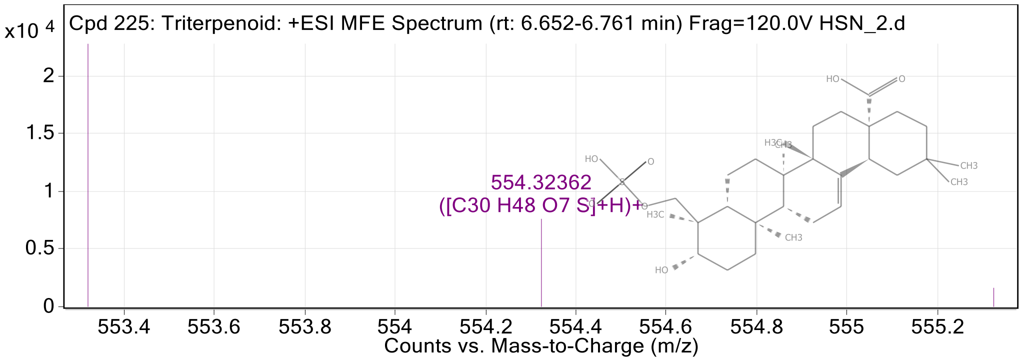

Peak List

m/z	z	Abund	Name	Formula	Ion	Score (DB)	Hits (DB)
553.32089	1	22790.36	Triterpenoid	C30 H48 O7 S	(M+H)+	90.91	2
554.32362	1	7616.94		C30 H48 O7 S	(M+H)+		
555.32397	1	1640.64		C30 H48 O7 S	(M+H)+		

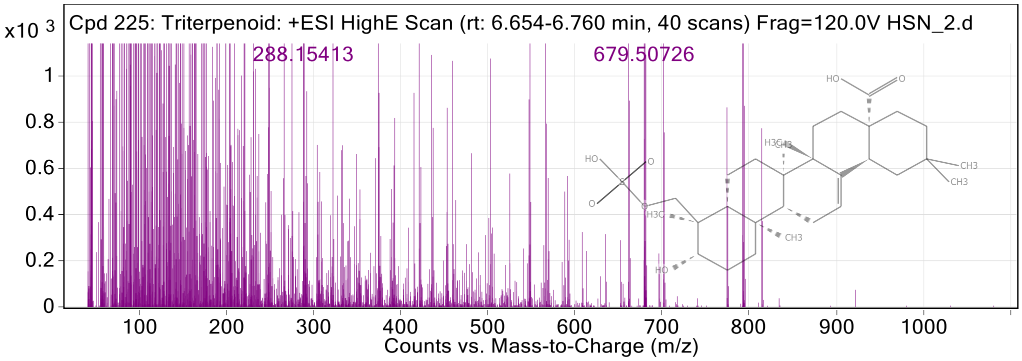

Peak List

m/z	z	Abund
43.01754		7223.73
45.03316		35543.34

59.04852		7280.33
89.05852	1	32912.62
105.06824	1	14154.56
119.08361		8080.15
133.08458		16534.96
248.16192	1	8417.12
288.15413	1	18743.22
679.50726	1	11136.68

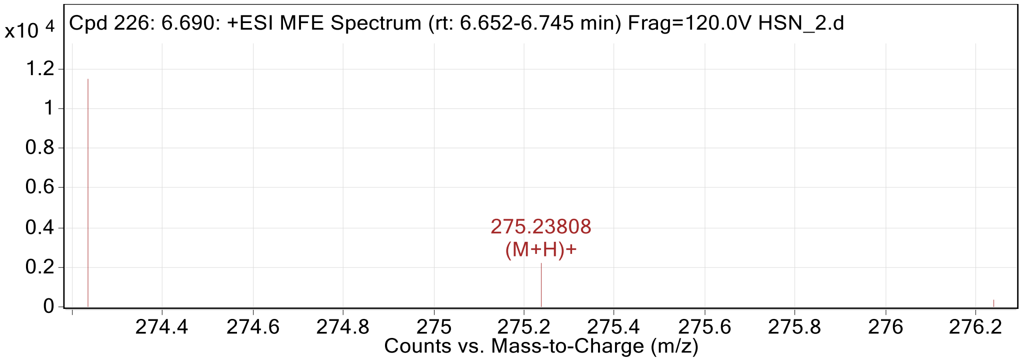

Peak List

m/z	z	Abund	Ion
274.23512	1	11499.45	(M+H)+
275.23808	1	2213.35	(M+H)+
276.2392	1	361.13	(M+H)+

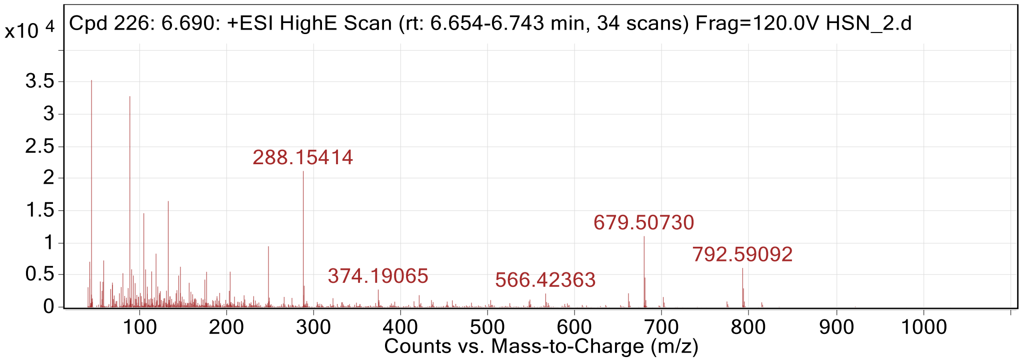

Peak List

m/z	z	Abund
43.01754		7053.08
45.03318		35336.09
59.04854		7253.06
89.05853	1	32822.55
105.06823	1	14610.68
119.08362		8311.85
133.08455		16503.72
248.1619	1	9468.15
288.15414	1	21181.21
679.5073	1	11042.42

Qualitative Analysis Report

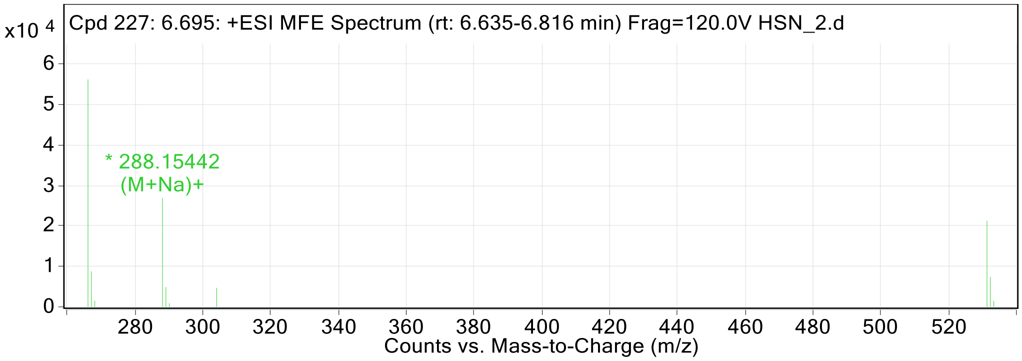

Peak List

m/z	z	Abund	Ion
266.1725	1	56187.51	(M+H)+
267.17365	1	8775.74	(M+H)+
268.17691	1	1511.37	(M+H)+
288.15442	1	26865.85	(M+Na)+
289.15589	1	4897.68	(M+Na)+
290.15971	1	898.4	(M+Na)+
304.12763	1	4702.4	(M+K)+
531.3392	1	21247.47	(2M+H)+
532.34142	1	7395.11	(2M+H)+
533.34231	1	1493.36	(2M+H)+

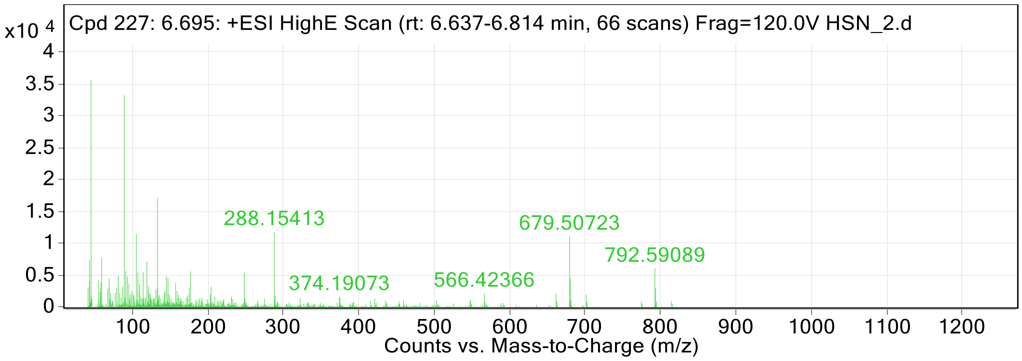

Peak List

m/z	z	Abund
43.01753		7260.05
45.03317		35583.64
59.04855		7641.19
89.05851	1	33181.79
105.06825		11445.62
119.08361		7088.8
133.08445		17068.85
288.15413	1	11748.44
679.50723	1	11033.28
792.59089	1	5996.36

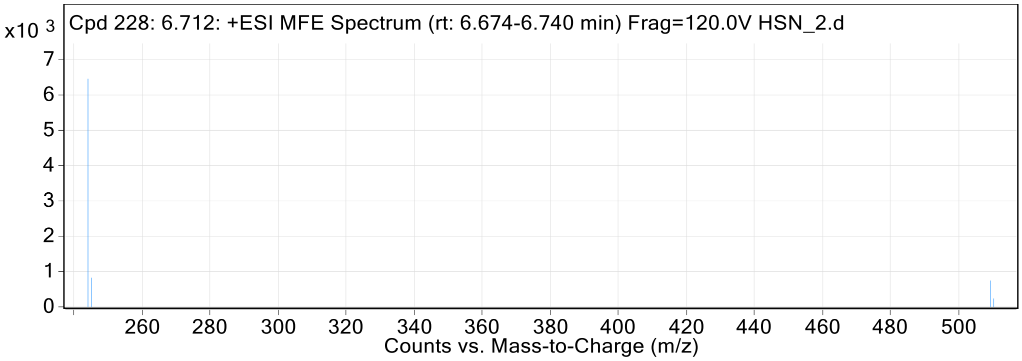

Peak List

m/z	z	Abund	Ion
244.18817	1	6453.86	(M+H)+
245.19031	1	825.54	(M+H)+
509.3554	1	744.09	(2M+Na)+
510.35477	1	236.4	(2M+Na)+

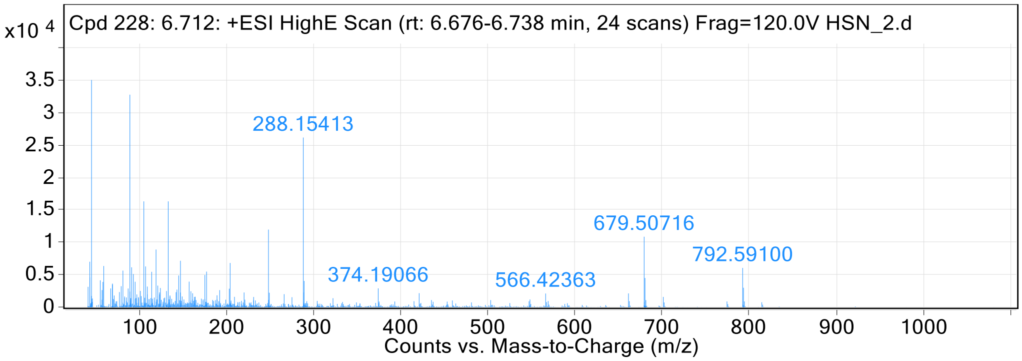

Peak List

m/z	z	Abund
43.01755		6988.02
45.03315		35033.04
89.05852	1	32763.16
105.06824	1	16300.66
119.08362		8870.6
133.08457		16301.44
147.11451		7138.92
248.16188	1	11948.73
288.15413	1	26155.45
679.50716	1	10848.12

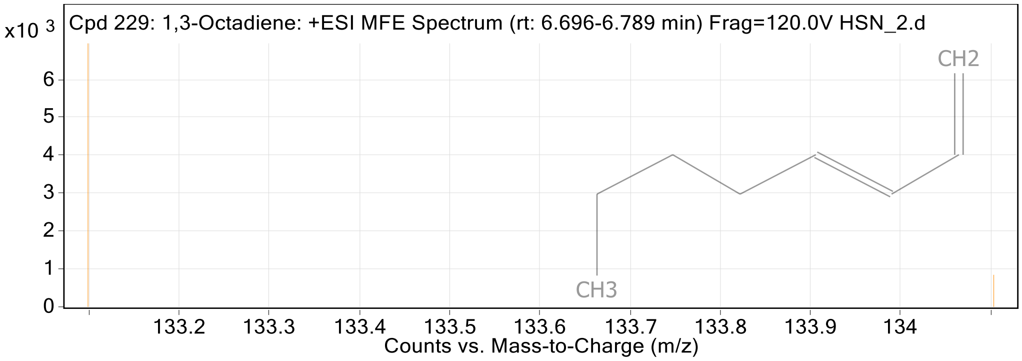

Peak List

m/z	z	Abund	Name	Formula	Ion	Score (DB)	Hits (DB)
133.099	1	6949.53	1,3-Octadiene	C8 H14	(M+Na)+	81.68	1

134.10334	1	844.97		C8 H14	(M+Na)+		

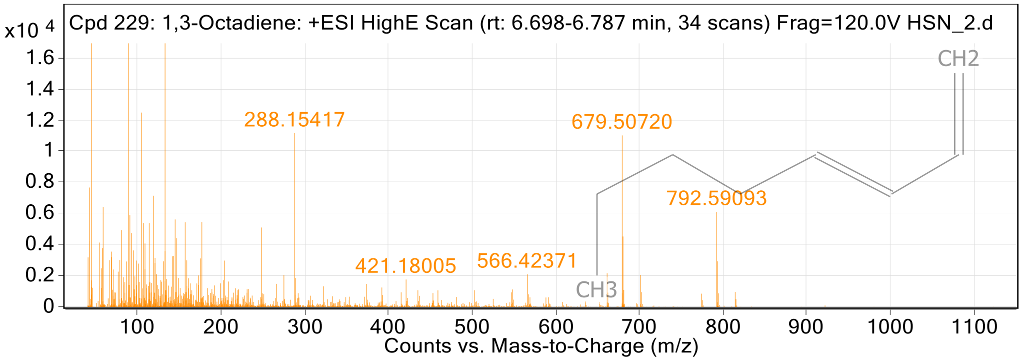

Peak List

m/z	z	Abund
43.01751		7667.7
45.03317		35696.61
59.04854		6418.11
89.05849	1	33525.04
105.06827		12487.48
119.0836		7141.02
133.08456		16940.79
288.15417	1	11160.66
679.5072	1	11018.29
792.59093	1	6103.79

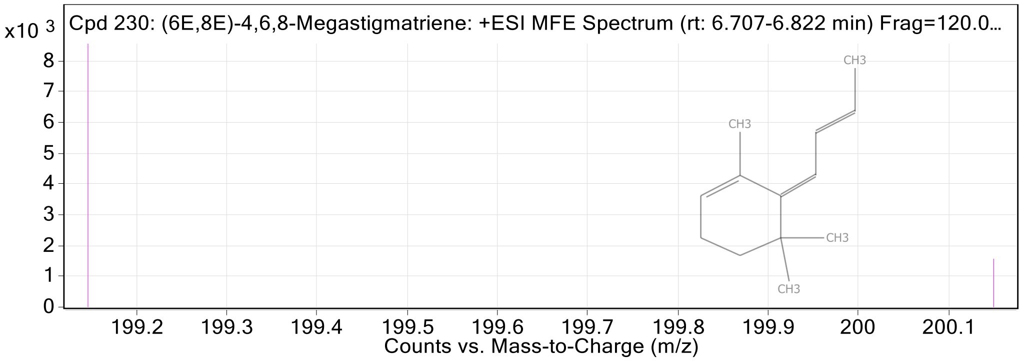

Peak List

m/z	z	Abund	Name	Formula	Ion	Score (DB)	Hits (DB)
199.14609	1	8555.87	(6E,8E)-4,6,8-Megastigmatriene	C13 H20	(M+Na)+	82.24	2
200.14979	1	1569.1		C13 H20	(M+Na)+		

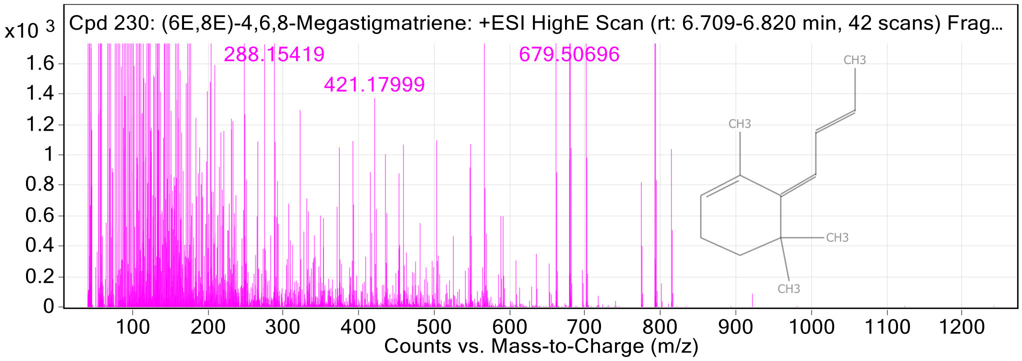

Peak List

m/z	z	Abund
43.0175		7639.22

45.03316		35650.45
59.04854		6328.71
89.05847	1	33392.64
105.06827		10620.46
119.08358		6540.37
133.08447		16884.7
288.15419		6383.66
679.50696	1	10893.46
792.59083	1	5978.77

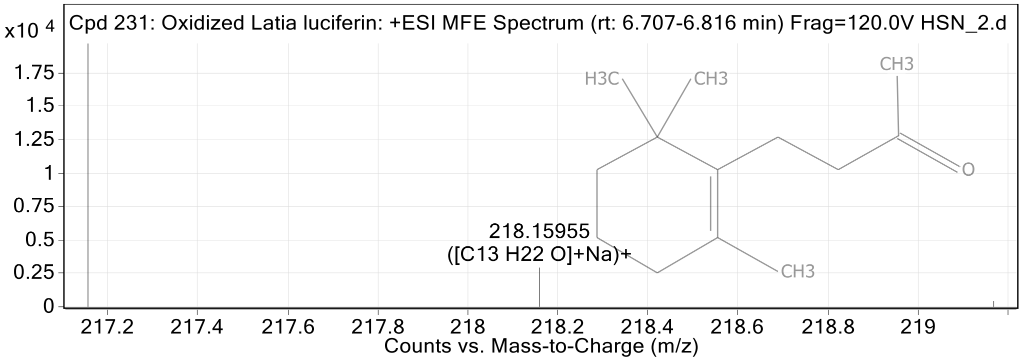

Peak List

m/z	z	Abund	Name	Formula	Ion	Score (DB)	Hits (DB)
217.15636	1	19715.68	Oxidized Latia luciferin	C13 H22 O	(M+Na)+	98.2	10
218.15955	1	2934.72		C13 H22 O	(M+Na)+		
219.16817	1	439.84		C13 H22 O	(M+Na)+		

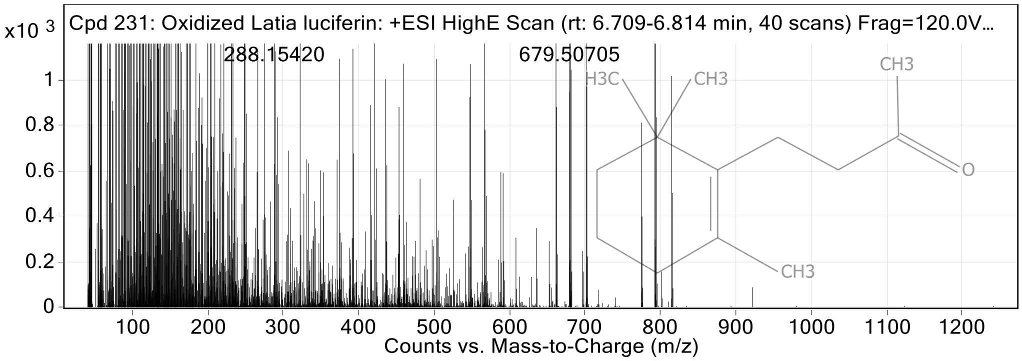

Peak List

m/z	z	Abund
43.01751		7661.46
45.03317		35619.52
59.04854		6278.17
89.05848	1	33277.79
105.06826		10781.55
119.0836		6598.07
133.08447		16827.79
288.1542		6673.51
679.50705	1	10907.33
792.59084	1	5994.16

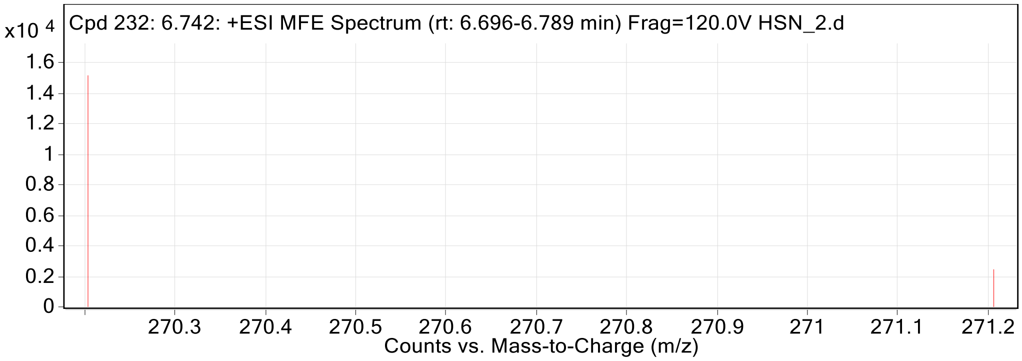

Peak List

m/z	z	Abund	Ion
270.20336	1	15159.82	(M+H)+
271.20655	1	2457.66	(M+H)+

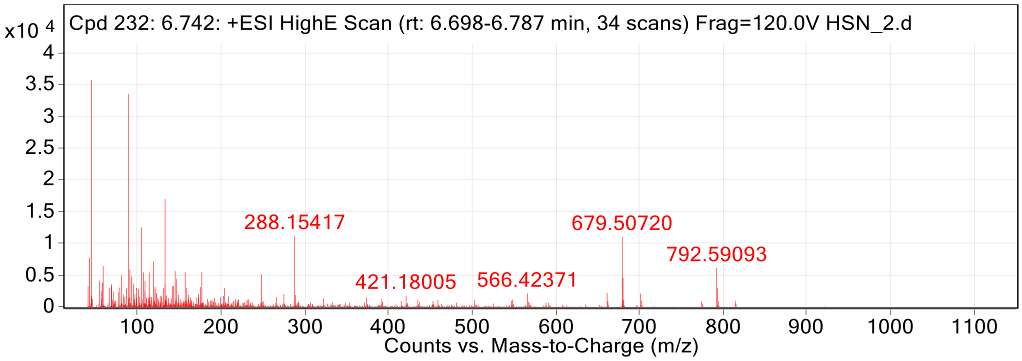

Peak List

m/z	z	Abund
43.01751		7667.7
45.03317		35696.61
59.04854		6418.11
89.05849	1	33525.04
105.06827		12487.48
119.0836		7141.02
133.08456		16940.79
288.15417	1	11160.66
679.5072	1	11018.29
792.59093	1	6103.79

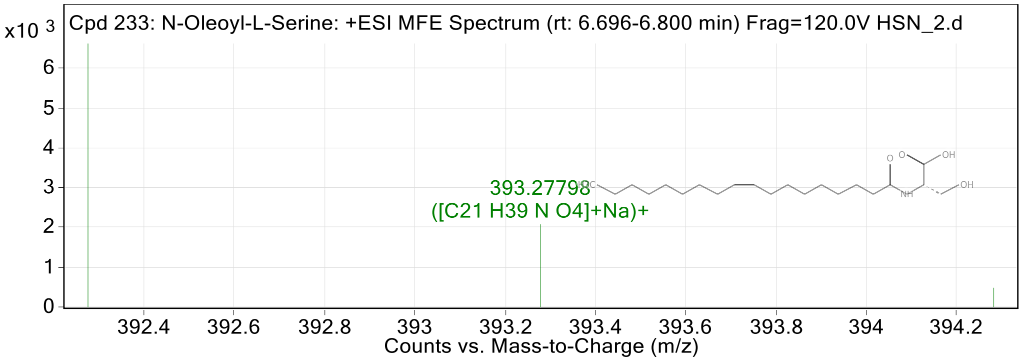

Peak List

m/z	z	Abund	Name	Formula	Ion	Score (DB)	Hits (DB)
392.27619	1	6626.64	N-Oleoyl-L-Serine	C21 H39 N O4	(M+Na)+	83.59	1
393.27798	1	2078.08		C21 H39 N O4	(M+Na)+		
394.28207	1	482.53		C21 H39 N O4	(M+Na)+		

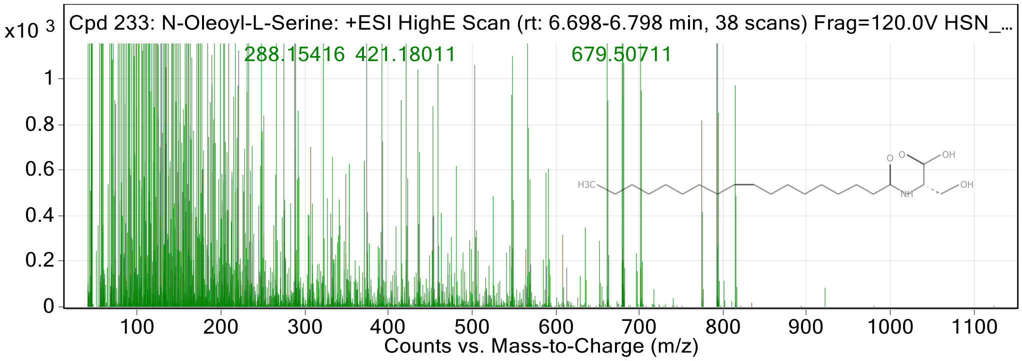

Peak List

m/z	z	Abund
43.01751		7616.1
45.03316		35616.71
59.04854		6265.72
89.05849	1	33343.59
105.06827		11984.26
119.0836		7012.99
133.08449		16929.48
288.15416	1	10067.3
679.50711	1	10909.87
792.5909	1	6085.42

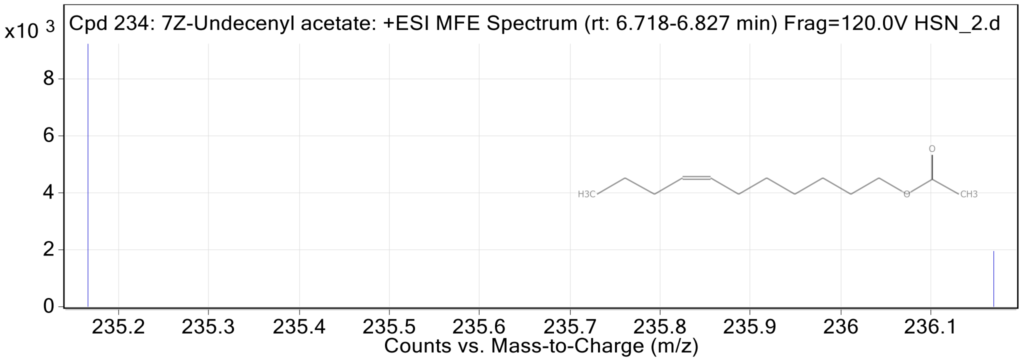

Peak List

m/z	z	Abund	Name	Formula	Ion	Score (DB)	Hits (DB)
235.16637	1	9227.68	7Z-Undecenyl acetate	C13 H24 O2	(M+Na)+	75.67	10
236.16943	1	1954.56		C13 H24 O2	(M+Na)+		

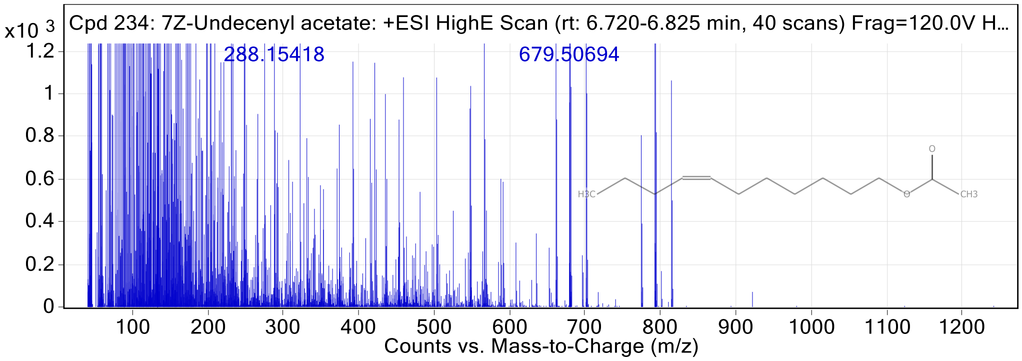

Peak List

m/z	z	Abund
43.0175		7617.93
45.03316		35697.39
59.04854		6457

89.05846	1	33395.76
91.05304		5604.02
105.06826		10149.24
119.08354		6345.99
133.08447		16769.5
679.50694	1	10895.2
792.59078	1	5910.2

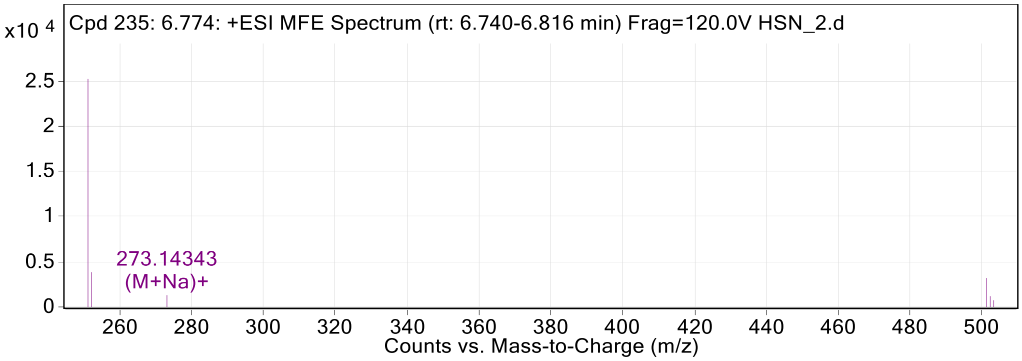

Peak List

m/z	z	Abund	Ion
251.16147	1	25213.27	(M+H)+
252.16488	1	3846.22	(M+H)+
273.14343	1	1303.34	(M+Na)+
501.31646	1	3201.65	(2M+H)+
502.31576	1	1189.77	(2M+H)+
503.31441	1	739.03	(2M+H)+

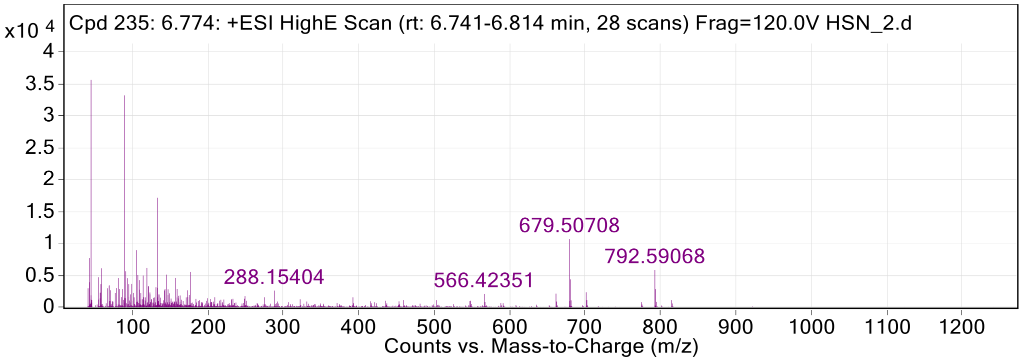

Peak List

m/z	z	Abund
43.0175		7679.01
45.03318		35579.51
59.04854		6048.62
89.05847	1	33162.04
91.05307		5585.77
105.06828		8905.41
119.08358		6128.93
133.0844		17129.29
679.50708	1	10657.06
792.59068	1	5806.96

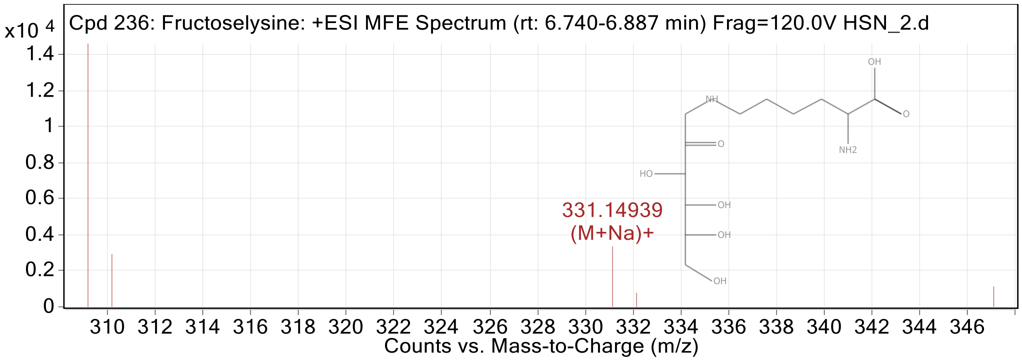

Peak List

m/z	z	Abund	Name	Formula	Ion	Score (DB)	Hits (DB)
309.16658	1	14632.59	Fructoselysine	C12 H24 N2 O7	(M+H)+	74.41	2
310.17004	1	2938.79		C12 H24 N2 O7	(M+H)+		
331.14939	1	3349.34			(M+Na)+		
332.15411	1	781.21			(M+Na)+		
347.12468	1	1130.93			(M+K)+		

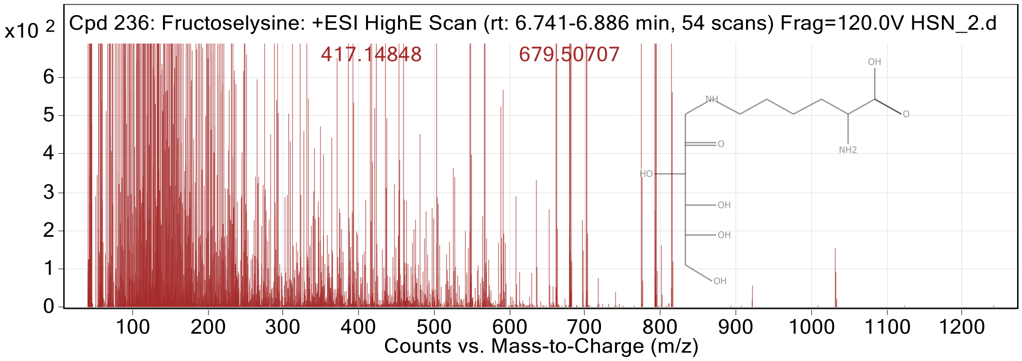

Peak List

m/z	z	Abund
43.01752		7087.6
45.03318		33742.93
59.04855		11178.36
89.05847	1	31797.3
91.05304		5292.01
105.06829		8317.94
119.08359		6078.65
133.08439		16040
177.11007		5275.36
679.50707	1	9655.88

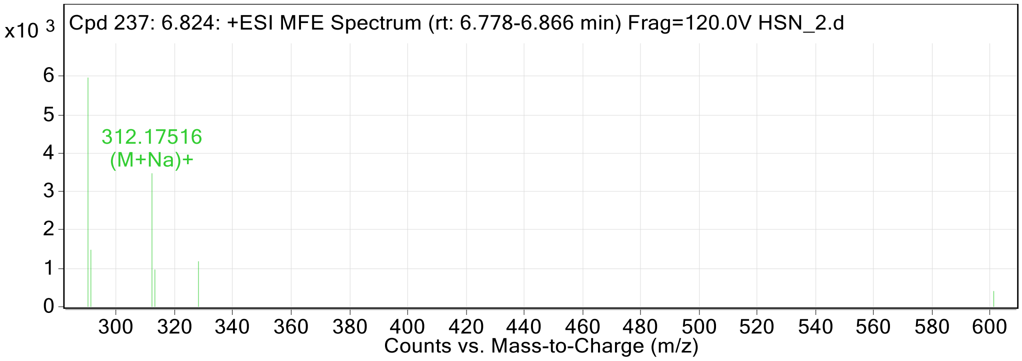

Peak List

m/z	z	Abund	Ion

290.1931	1	5968.19	(M+H)+
291.19511	1	1484.45	(M+H)+
312.17516	1	3476	(M+Na)+
313.1827	1	969.48	(M+Na)+
328.15143	1	1184.34	(M+K)+
601.35819	1	409.52	(2M+Na)+

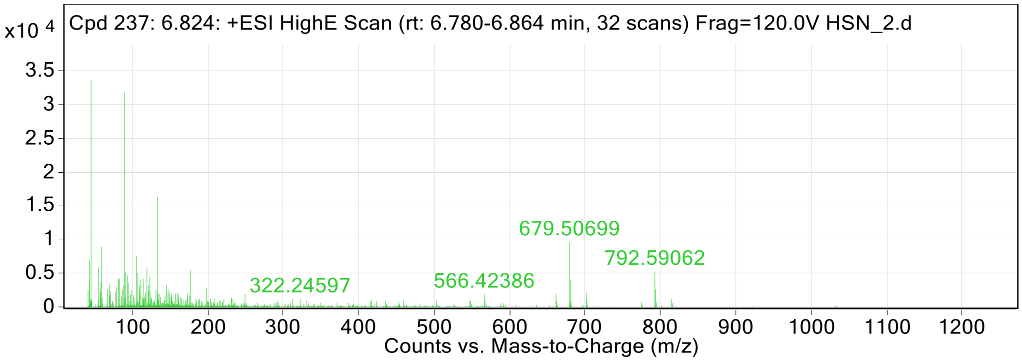

Peak List

m/z	z	Abund
43.01755		6887.77
45.03317		33603.37
55.05368		5872.56
59.04853		8884.48
89.05844	1	31780.05
105.06827		7500.74
119.08355		5684.35
133.08422		16312.12
177.11016		5446.8
679.50699	1	9597.51

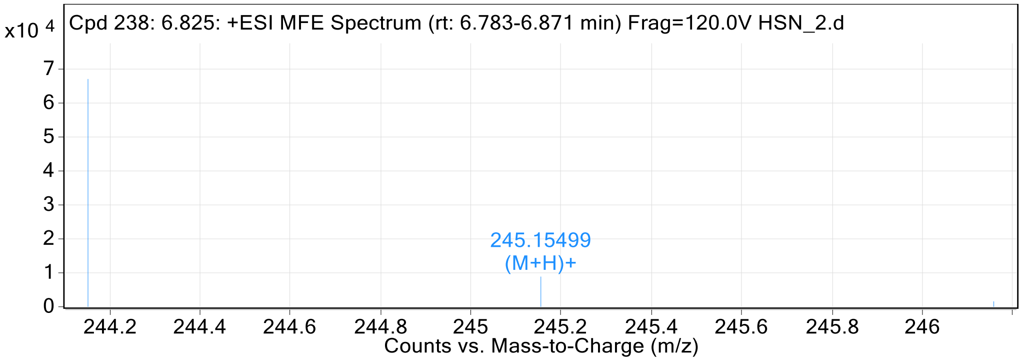

Peak List

m/z	z	Abund	Ion
244.15204	1	66984.85	(M+H)+
245.15499	1	8873.51	(M+H)+
246.15802	1	1589.79	(M+H)+

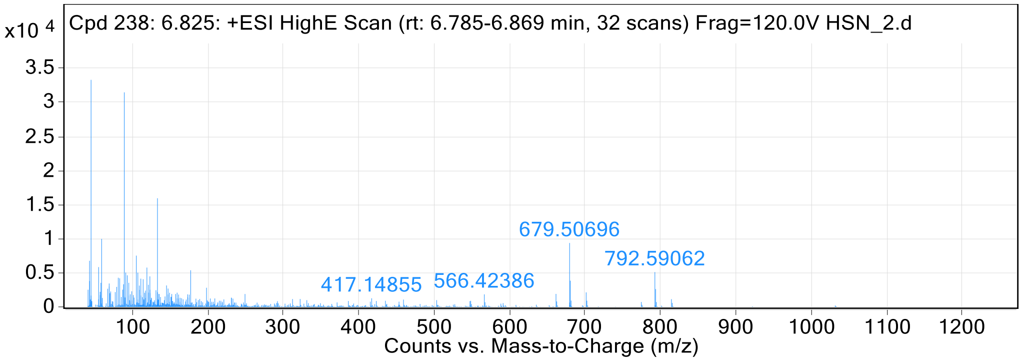

Peak List

m/z	z	Abund
43.01754		6767.64
45.03317		33315.39
55.05368		5841.9
59.04851		9978.91
89.05844	1	31479.7
105.06826		7520.92
119.08356		5767.91
133.08424		15936.32
177.11018		5373.39
679.50696	1	9375.67

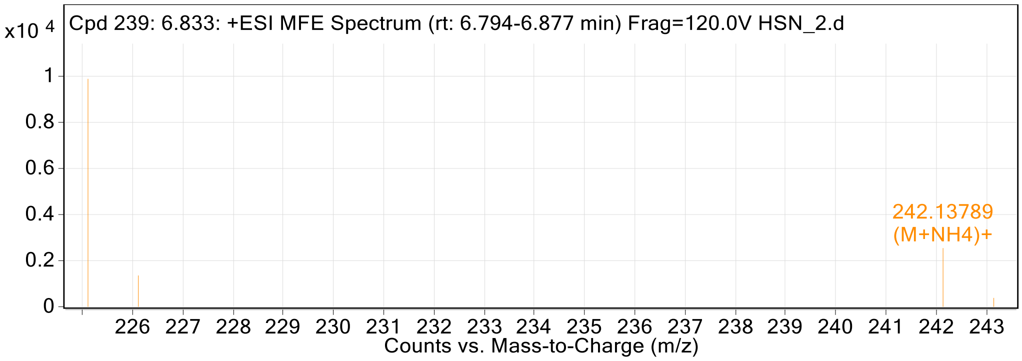

Peak List

m/z	z	Abund	Ion
225.10992	1	9862.88	(M+H)+
226.11293	1	1355.15	(M+H)+
242.13789	1	2539.22	(M+NH4)+
243.14794	1	383.01	(M+NH4)+

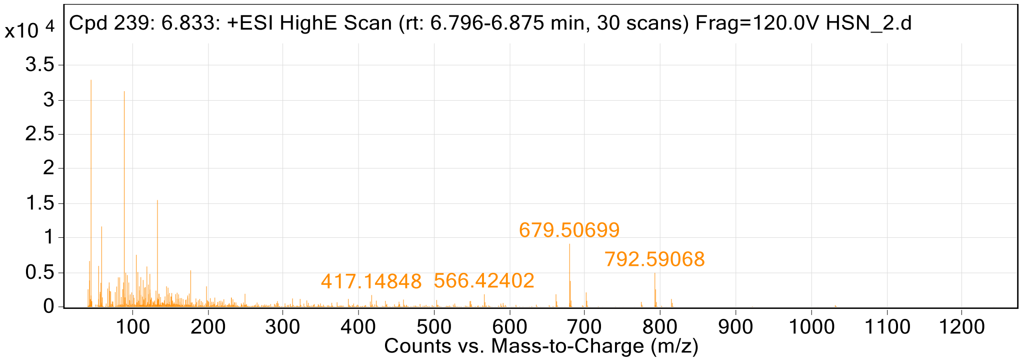

Peak List

m/z	z	Abund
43.01754		6641.38

45.03318		32898.43
55.05369		5929.41
59.04855		11647.29
89.05845	1	31252.83
105.06824		7540.94
119.08358		5848.12
133.0843		15475.37
177.11015		5294.05
679.50699	1	9146.67

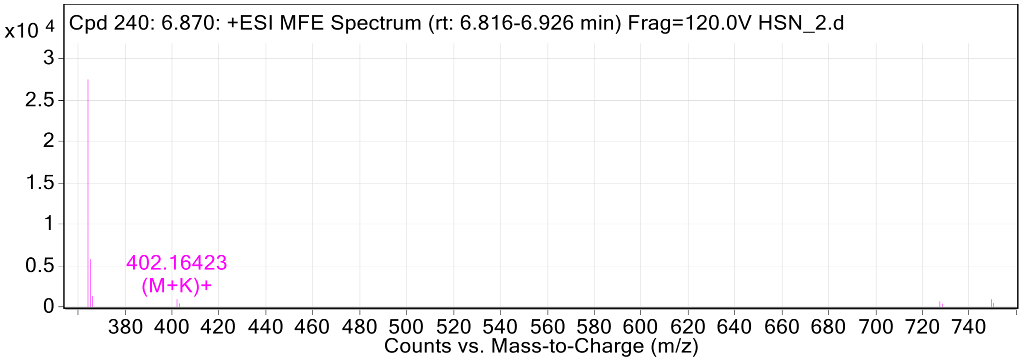

Peak List

m/z	z	Abund	Ion
364.20896	1	27506.24	(M+H)+
365.21162	1	5756.11	(M+H)+
366.21622	1	1299.21	(M+H)+
402.16423	1	925.2	(M+K)+
403.1703	1	406.76	(M+K)+
727.41257	1	658	(2M+H)+
728.41683	1	390.61	(2M+H)+
749.39479	1	908.03	(2M+Na)+
750.39667	1	474.77	(2M+Na)+

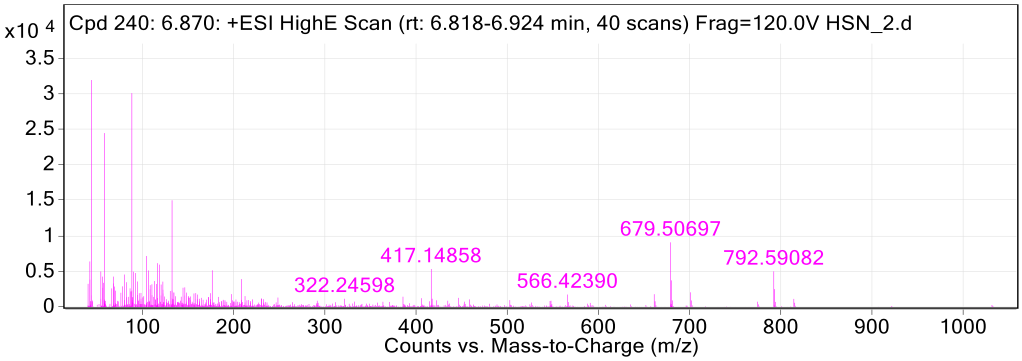

Peak List

m/z	z	Abund
43.01753		6377.37
45.03316		31929.95
59.04856		24458.71
89.05844	1	30084.38
105.0683		7144.94
117.08908		6137.51
119.08356		5929.84
133.08439		14991.35
417.14858	1	5311.87
679.50697	1	9069.09

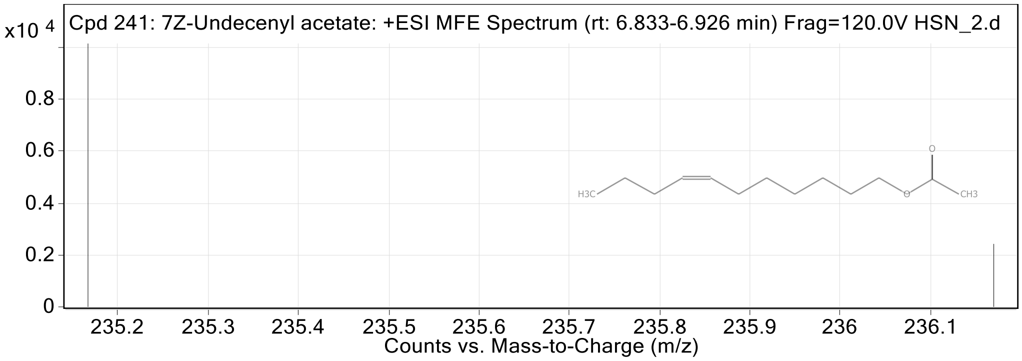

Peak List

m/z	z	Abund	Name	Formula	Ion	Score (DB)	Hits (DB)
235.16697	1	10162.95	7Z-Undecenyl acetate	C13 H24 O2	(M+Na)+	71.25	10
236.16994	1	2429.68		C13 H24 O2	(M+Na)+		

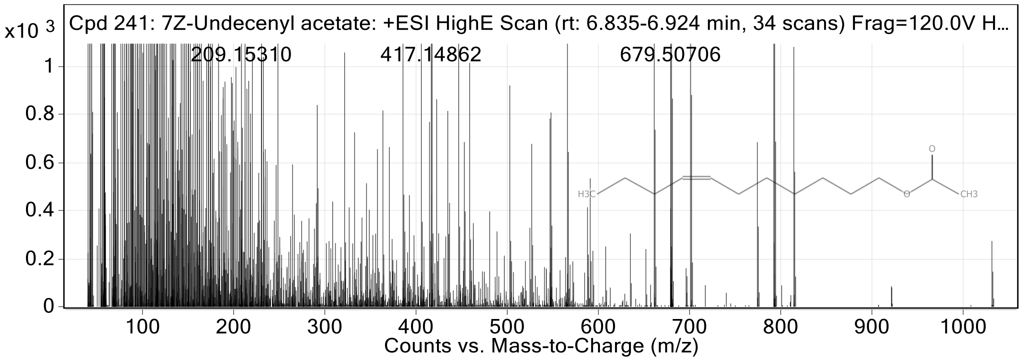

Peak List

m/z	z	Abund
43.01754		6381.57
45.03318		31473.58
59.04856		27370.73
89.05846	1	29516.06
105.06832		7164.89
117.08907		6943.82
119.08361		6087.21
133.08448		14766.84
417.14862	1	5960.1
679.50706	1	8744.83

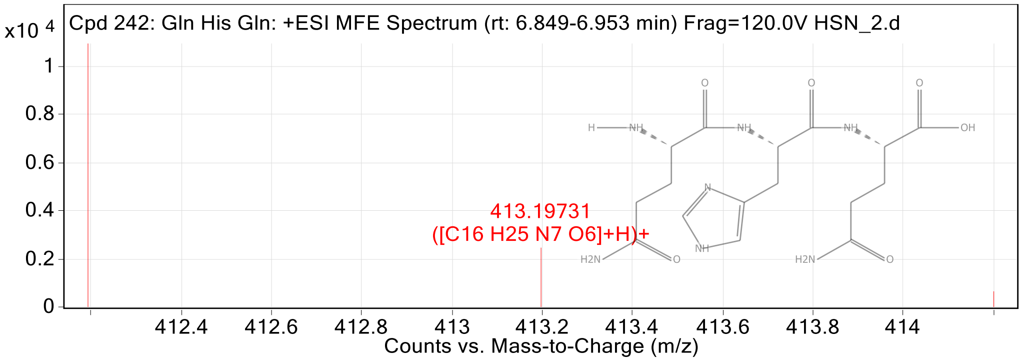

Peak List

m/z	z	Abund	Name	Formula	Ion	Score (DB)	Hits (DB)
412.19312	1	10942.76	Gln His Gln	C16 H25 N7 O6	(M+H)+	93.54	10
413.19731	1	2463.67		C16 H25 N7 O6	(M+H)+		
414.20074	1	650.09		C16 H25 N7 O6	(M+H)+		

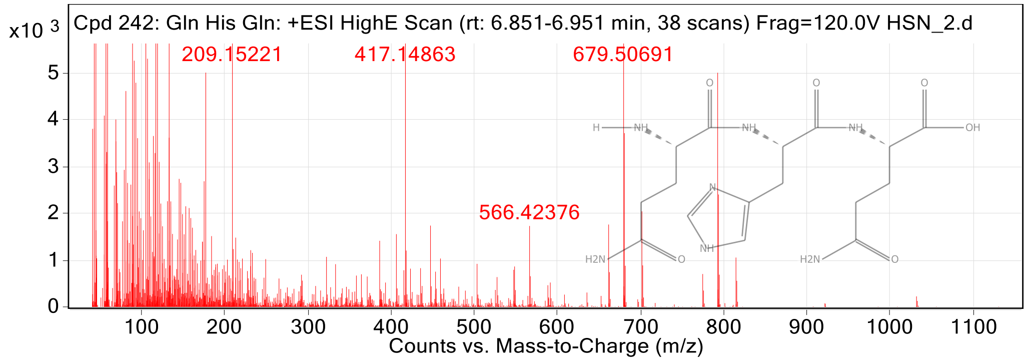

Peak List

m/z	z	Abund
43.01754		6442.9
45.03318		31763.98
57.06925		6425.17
59.04854		29677.29
89.05847	1	29480.2
105.0683		7634.89
117.08907		7477.83
133.0846		14618.88
209.15221	1	9165.65
679.50691	1	9069.45

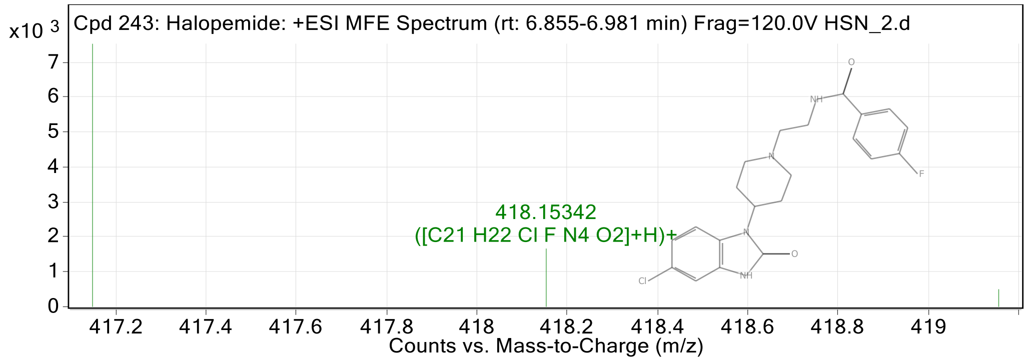

Peak List

m/z	z	Abund	Name	Formula	Ion	Score (DB)	Hits (DB)
417.14825	1	7529.01	Halopemide	C21 H22 Cl F N4 O2	(M+H)+	59.22	1
418.15342	1	1665.42		C21 H22 Cl F N4 O2	(M+H)+		
419.15603	1	501.96		C21 H22 Cl F N4 O2	(M+H)+		

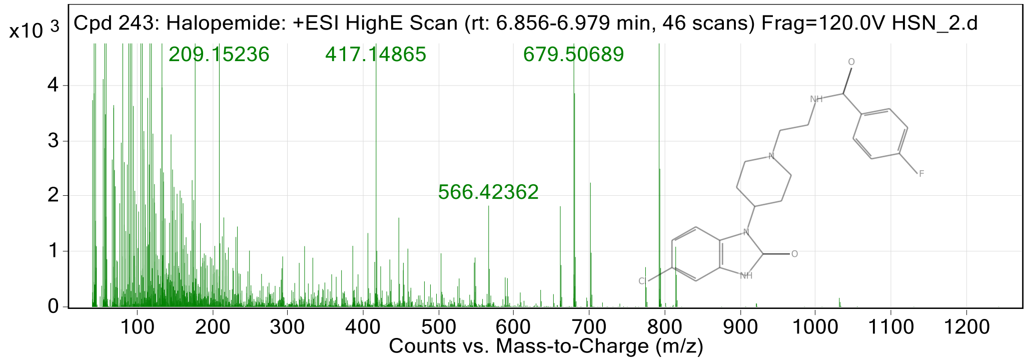

Peak List

m/z	z	Abund
43.01754		6597.83
45.03316		32135.25

57.06925		6098.18
59.04855		26865.14
89.05846	1	29877.17
105.06827		7748.42
117.08906		6706.35
133.08463		14803.2
209.15236	1	8356.9
679.50689	1	9399.7

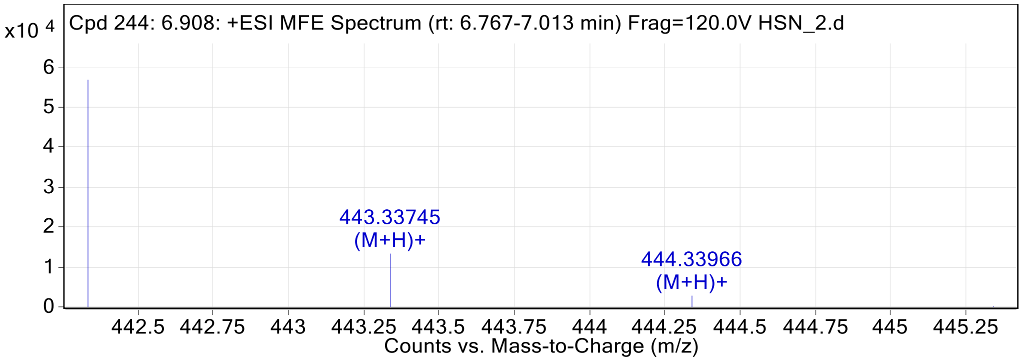

Peak List

m/z	z	Abund	Ion
442.33384	1	56906.98	(M+H)+
443.33745	1	13342.5	(M+H)+
444.33966	1	2804.74	(M+H)+
445.34183	1	173.85	(M+H)+

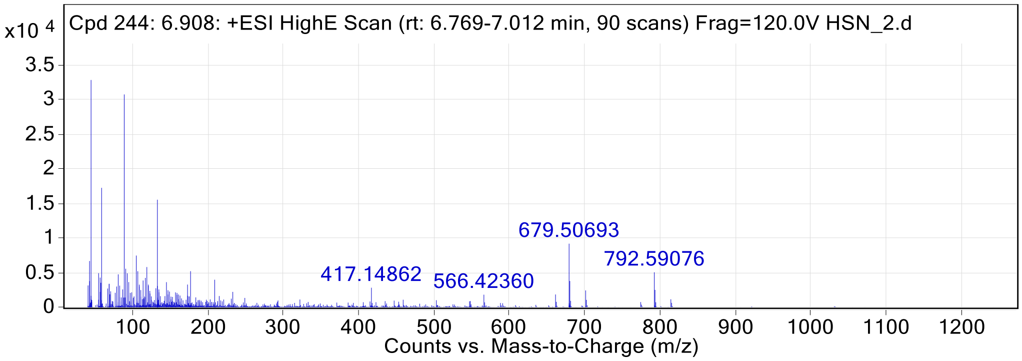

Peak List

m/z	z	Abund
43.01753		6662.61
45.03316		32857.02
59.04854		17249.4
89.05844	1	30766.14
91.05295		5545.05
105.06826		7448.24
119.08357		5787.01
133.08443		15530.76
177.11007		5190.36
679.50693	1	9168.34

Qualitative Analysis Report

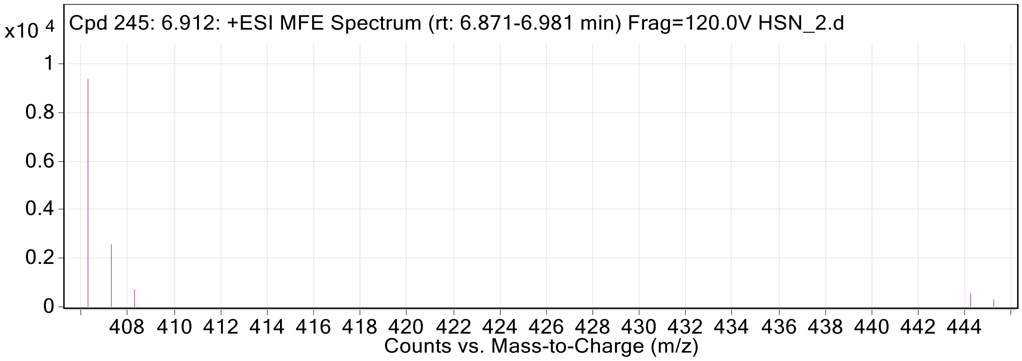

Peak List

m/z	z	Abund	Ion
406.2919	1	9393.2	(M+H)+
407.29583	1	2576.86	(M+H)+
408.29662	1	721.24	(M+H)+
444.25115	1	558.58	(M+K)+
445.24253	1	312.84	(M+K)+

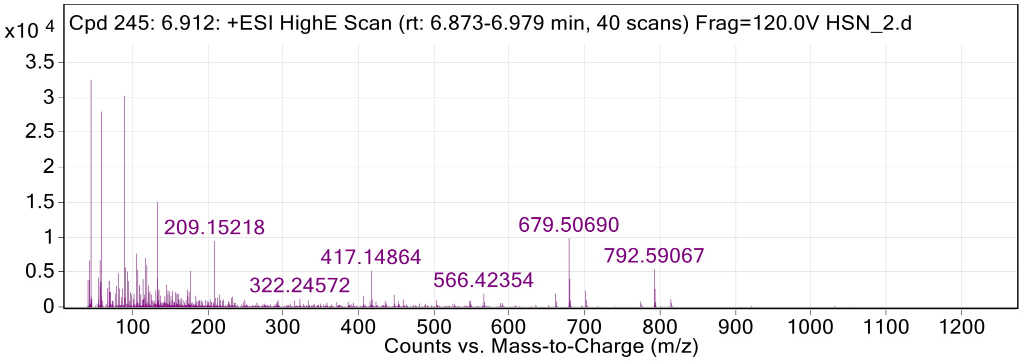

Peak List

m/z	z	Abund
43.01754		6638.63
45.03315		32481.75
57.06926		6681.54
59.04855		27957.96
89.05846	1	30183.81
105.06826		7616.04
117.08905		6985.66
133.08466		15020.16
209.15218	1	9430.54
679.5069	1	9803.54

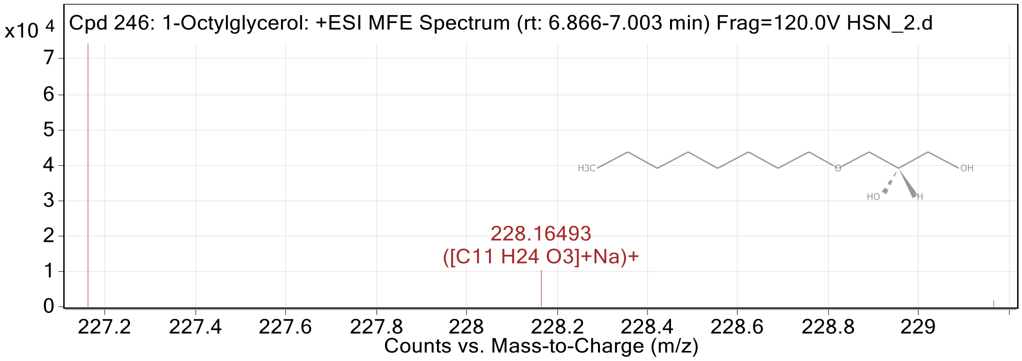

Peak List

m/z	z	Abund	Name	Formula	Ion	Score (DB)	Hits (DB)

227.16182	1	74400.91	1-Octylglycerol	C11 H24 O3	(M+Na)+	98.66	2
228.16493	1	10370.22		C11 H24 O3	(M+Na)+		
229.16635	1	1781.21		C11 H24 O3	(M+Na)+		

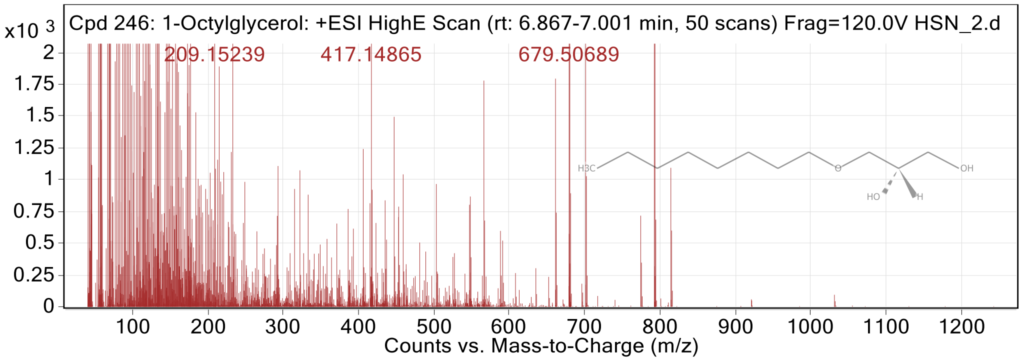

Peak List

m/z	z	Abund
43.01753		6464.54
45.03315		32363.61
59.04855		24450.48
89.05844	1	30062.49
105.06824		7427.78
117.08904		6092.13
119.08362		5903.4
133.0846		14864.98
209.15239	1	7881.67
679.50689	1	9154.43

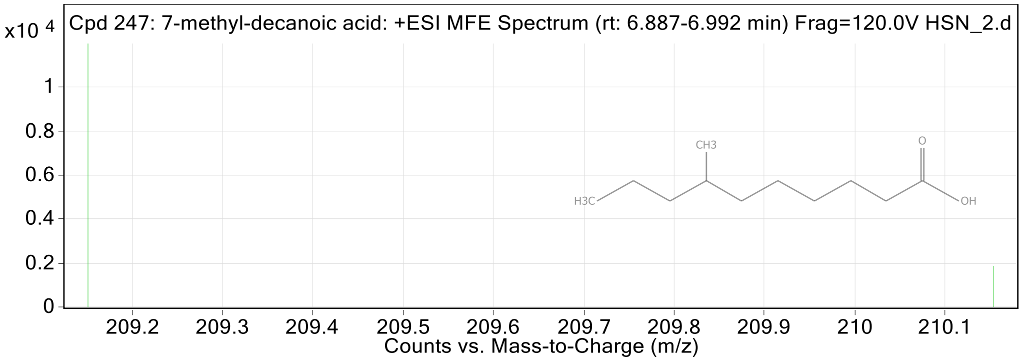

Peak List

m/z	z	Abund	Name	Formula	Ion	Score (DB)	Hits (DB)
209.15132	1	11997.01	7-methyl-decanoic acid	C11 H22 O2	(M+Na)+	82.01	10
210.15361	1	1871.75		C11 H22 O2	(M+Na)+		

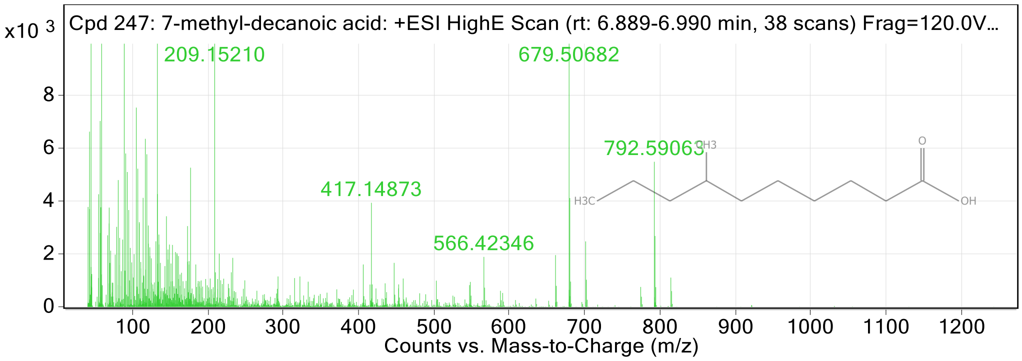

Peak List

m/z	z	Abund

43.01752		6613.13
45.03314		32876.44
57.06928		7015.8
59.04855		25624.59
89.05843	1	30490.4
105.06825		7520.82
117.08904		6342.33
133.08466		15120.39
209.1521	1	9933.79
679.50682	1	9952.68

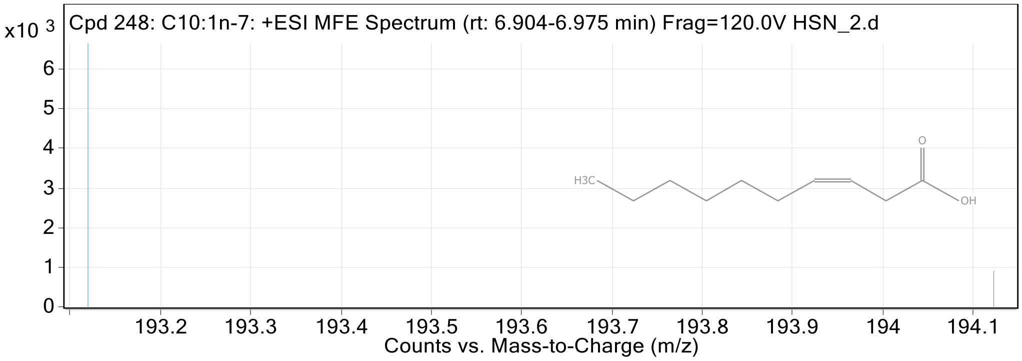

Peak List

m/z	z	Abund	Name	Formula	Ion	Score (DB)	Hits (DB)
193.11975	1	6654.33	C10:1n-7	C10 H18 O2	(M+Na)+	85.02	10
194.12274	1	906.88		C10 H18 O2	(M+Na)+		

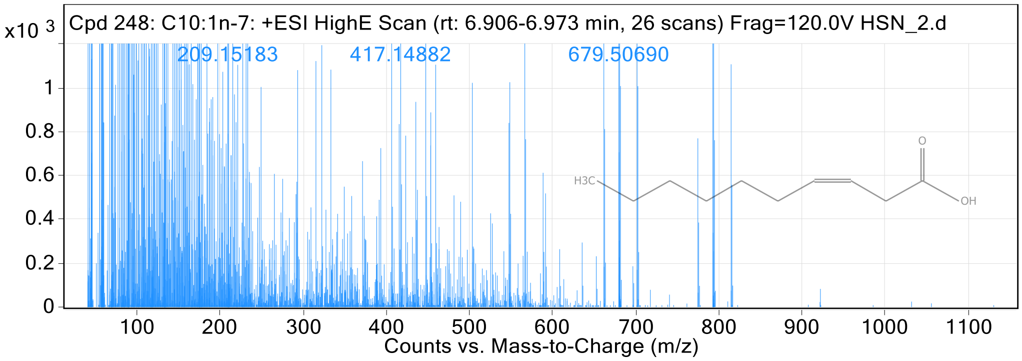

Peak List

m/z	z	Abund
43.01753		6775.57
45.03316		32731.95
57.06926		8692.37
59.04856		26140.94
89.05844	1	30478.79
105.06824		7959.57
117.08904		6512.4
133.08482		15275.55
209.15183	1	13345.71
679.5069	1	10386.72

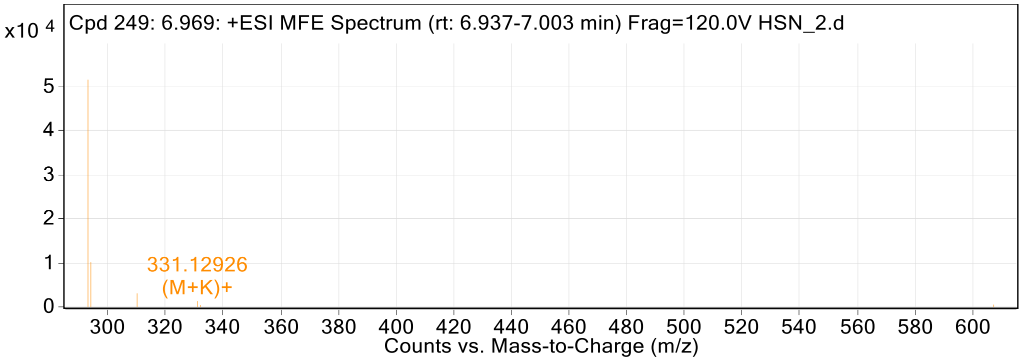

Peak List

m/z	z	Abund	Ion
293.17169	1	51555.09	(M+H)+
294.17488	1	10153.32	(M+H)+
310.1981	1	3056.7	(M+NH4)+
331.12926	1	1321.3	(M+K)+
332.1313	1	440.7	(M+K)+
607.31479	1	513.33	(2M+Na)+

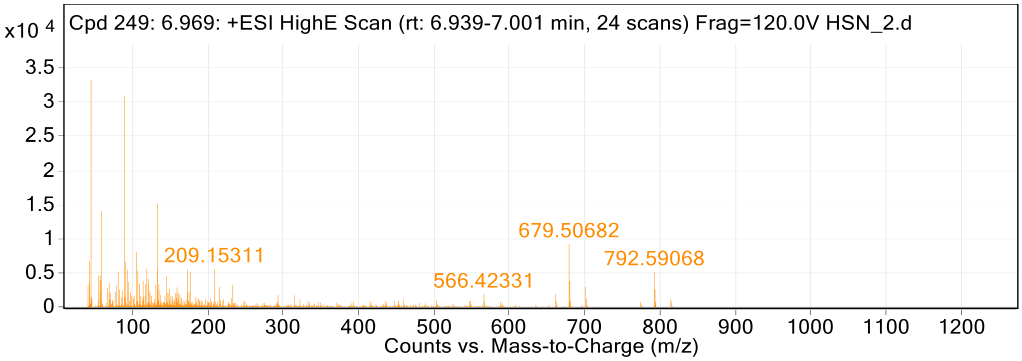

Peak List

m/z	z	Abund
43.01752		6754.07
45.03313		33268.94
59.04857		14047.23
89.05842	1	30879.96
91.0529		6568.73
105.06817		7996.74
119.08367		5584.79
133.08459		15156.38
209.15311		5553.89
679.50682	1	9202.75

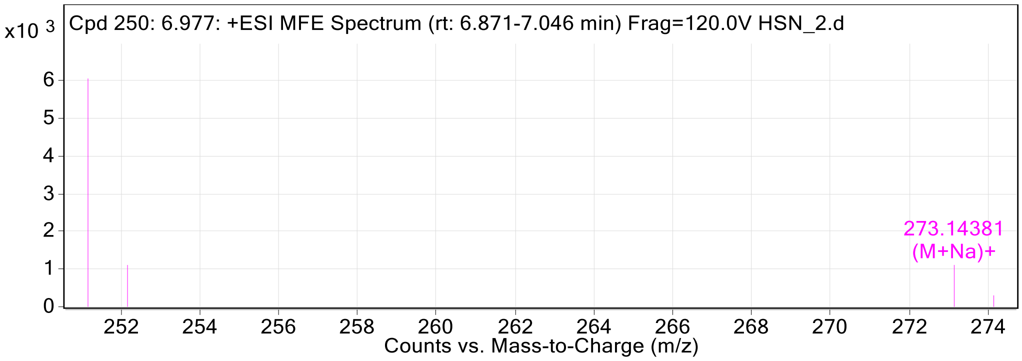

Peak List

m/z	z	Abund	Ion
251.16069	1	6064.95	(M+H)+
252.16393	1	1111.28	(M+H)+
273.14381	1	1111.72	(M+Na)+
274.14776	1	303.7	(M+Na)+

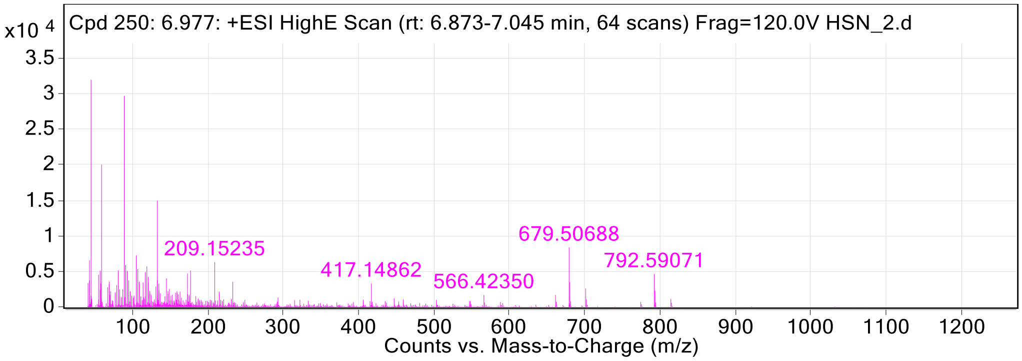

Peak List

m/z	z	Abund
43.01753		6553.13
45.03314		31967.85
59.04855		20026.06
89.05843	1	29709.1
91.0529		5905.18
105.06824		7240.96
119.08357		5694.18
133.08454		14945.51
209.15235		6296.03
679.50688	1	8364.34

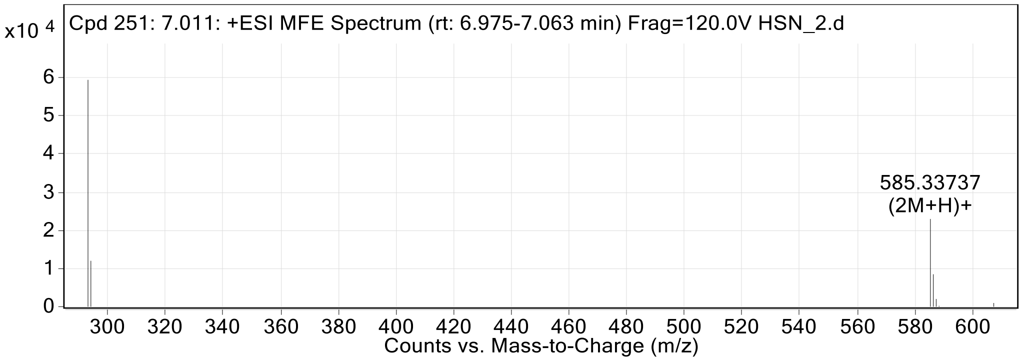

Peak List

m/z	z	Abund	Ion
293.17156	1	59463.89	(M+H)+
294.17534	1	12030.2	(M+H)+
585.33737	1	23001.71	(2M+H)+
586.34163	1	8505.57	(2M+H)+
587.34482	1	2027.01	(2M+H)+
588.34516	1	220.14	(2M+H)+
607.31715	1	964.96	(2M+Na)+

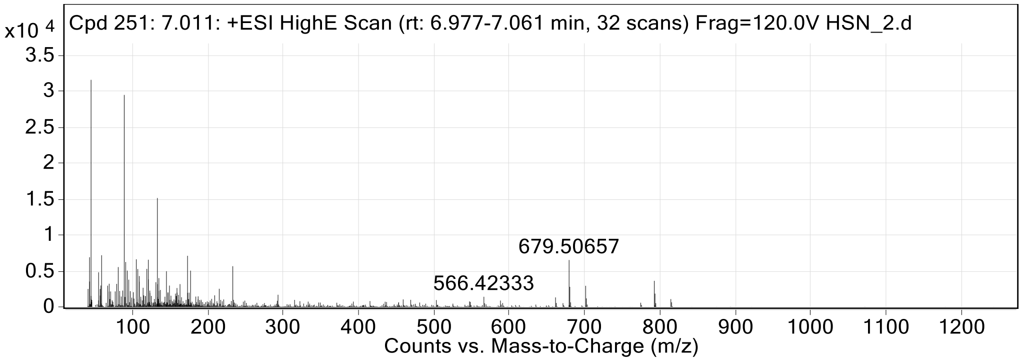

Peak List

m/z	z	Abund
43.0175		6913.43
45.03313		31609.22
59.04853		7190.78
89.05839	1	29509.43
91.05289		6253.24
105.06818		6624.8
121.06282		6565.34
133.08428	1	15156.65
173.09361	1	7110.43
679.50657	1	6525.37

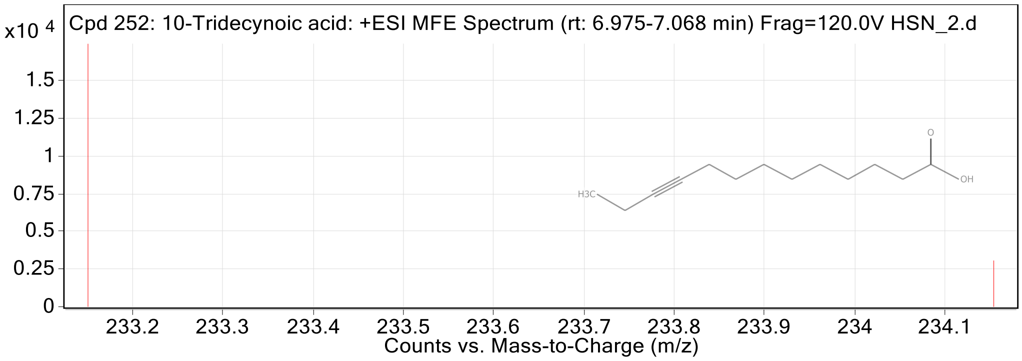

Peak List

m/z	z	Abund	Name	Formula	Ion	Score (DB)	Hits (DB)
233.15074	1	17463.92	10-Tridecynoic acid	C13 H22 O2	(M+Na)+	83.35	10
234.1541	1	3070.53		C13 H22 O2	(M+Na)+		

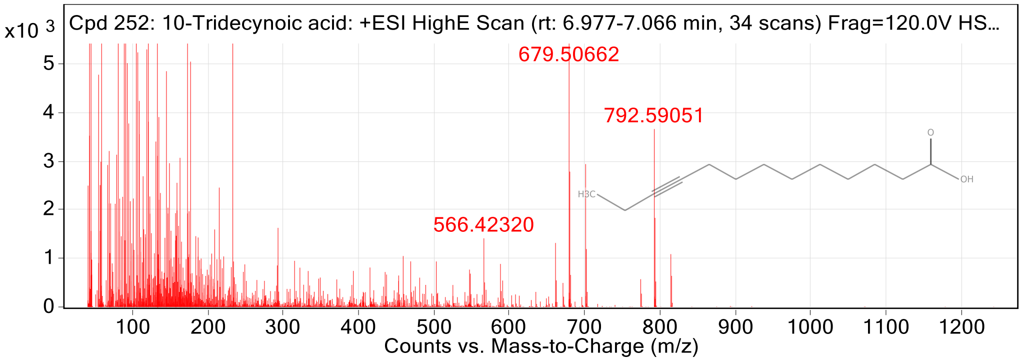

Peak List

m/z	z	Abund
43.01751		7099.11
45.03314		31694.68
59.04853		7323.8

89.05839	1	29581.74
91.05289		6176.82
105.06818		6557.44
121.06283		6272.68
133.0843	1	15163.17
173.09354	1	6817.31
679.50662	1	6570.05

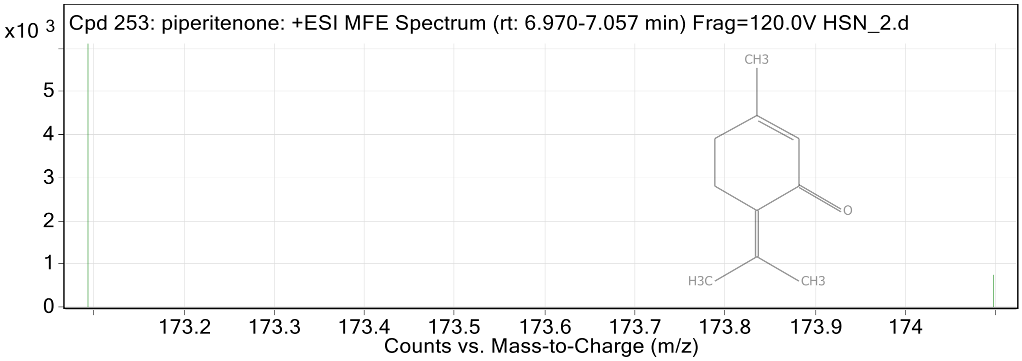

Peak List

m/z	z	Abund	Name	Formula	Ion	Score (DB)	Hits (DB)
173.09367	1	6118.03	piperitenone	C10 H14 O	(M+Na)+	86.01	10
174.09786	1	747.28		C10 H14 O	(M+Na)+		

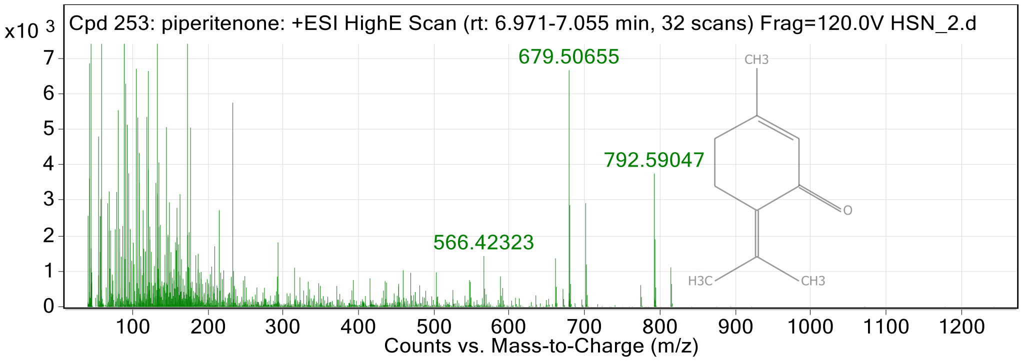

Qualitative Analysis Report

Peak List

m/z	z	Abund
43.01751		6851.22
45.03313		31421.24
59.04854		7400.35
89.05838	1	29538.85
91.05288		6277.96
105.06819		6695.86
121.06281		6634.82
133.0843	1	15092.48
173.09362	1	7404.44
679.50655	1	6657.76

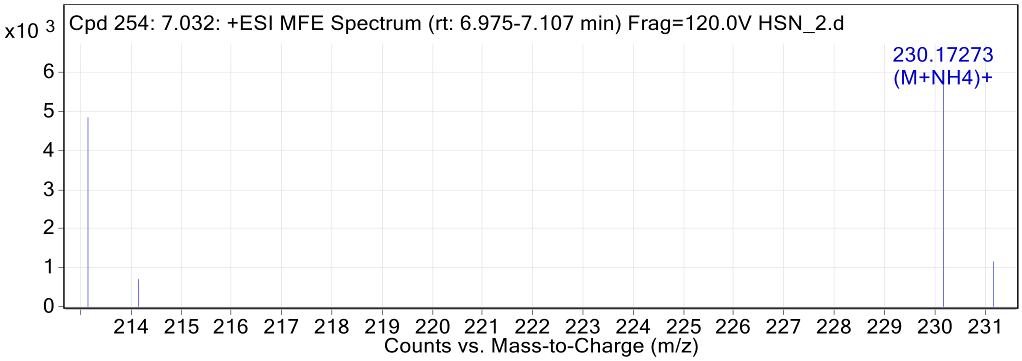

Peak List

m/z	z	Abund	Ion
213.14612	1	4852.59	(M+H)+
214.14896	1	706.03	(M+H)+
230.17273	1	5900.09	(M+NH4)+
231.17619	1	1160.53	(M+NH4)+

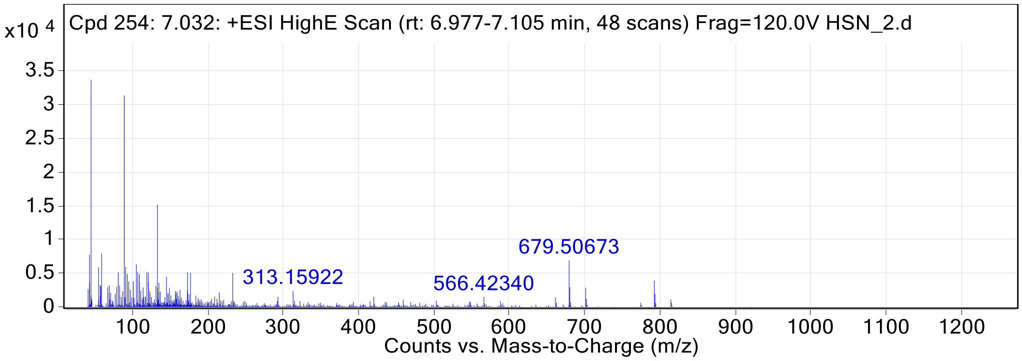

Peak List

m/z	z	Abund
43.01752		7740.74
45.03316		33708.05
55.05364		5863.07
59.04854		7931.46
89.05843	1	31384.01
91.05289		5907.1
105.06822		6295.15
133.08428		15173.13
173.09328		5163.03
679.50673	1	6911.97

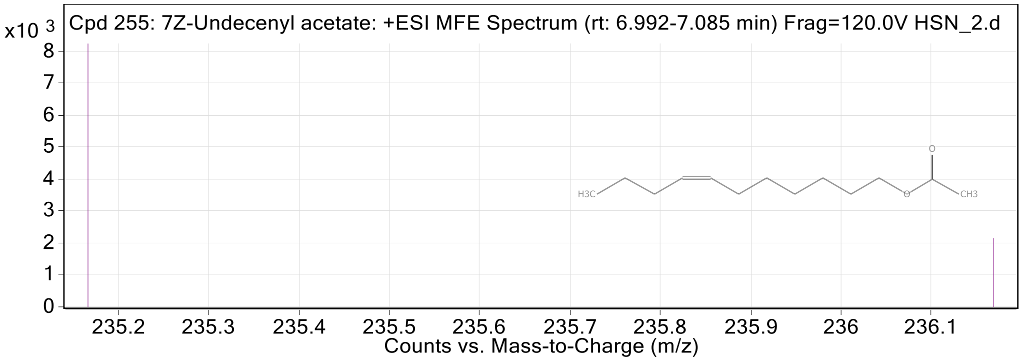

Peak List

m/z	z	Abund	Name	Formula	Ion	Score (DB)	Hits (DB)
235.16613	1	8248.85	7Z-Undecenyl acetate	C13 H24 O2	(M+Na)+	65.09	10
236.16933	1	2150.84		C13 H24 O2	(M+Na)+		

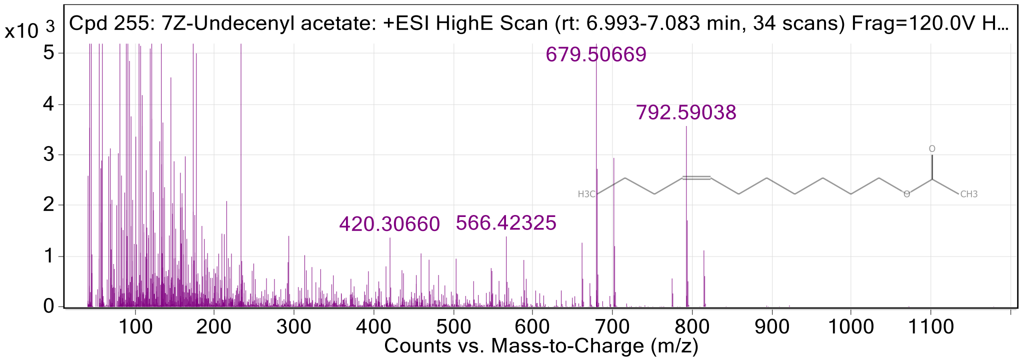

Peak List

m/z	z	Abund
43.01753		7821.68
45.03316		32212.09
59.04855		7251.25
89.05842	1	29927.78
91.0529		5908.53
105.06821		6381.41
121.06283		5842.69
133.08431	1	15127.06
173.0933		6045.01
679.50669	1	6353.13

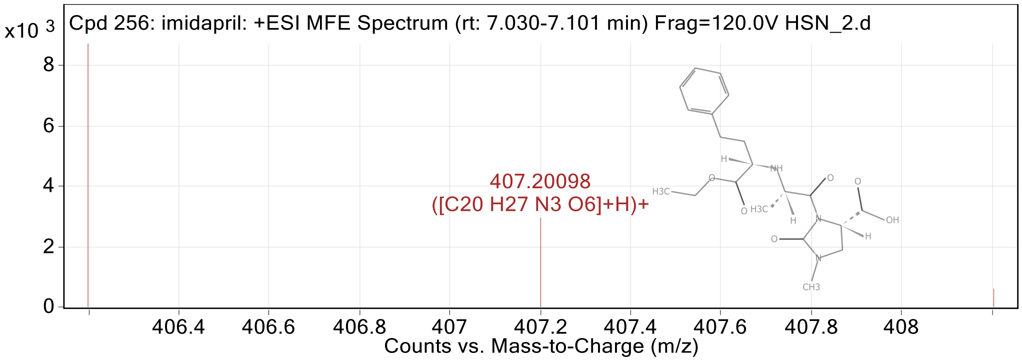

Peak List

m/z	z	Abund	Name	Formula	Ion	Score (DB)	Hits (DB)
406.19884	1	8727.71	imidapril	C20 H27 N3 O6	(M+H)+	80.72	1
407.20098	1	2953.12		C20 H27 N3 O6	(M+H)+		
408.2042	1	608.47		C20 H27 N3 O6	(M+H)+		

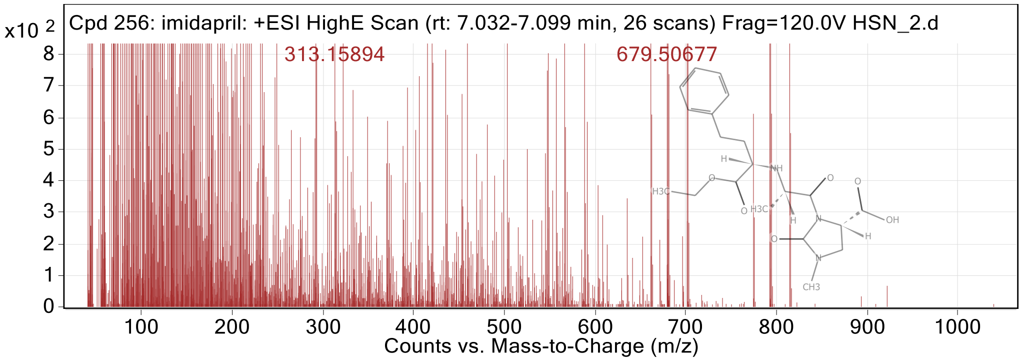

Peak List

m/z	z	Abund
43.01753		8885.01
45.03319		34861.11
55.05365		6261.7
59.04859		8339.49
89.05846	1	32563.21
91.05296		5431.23
101.05819		5512.38
105.06827		6031.67
133.08431		15219.87
679.50677	1	7445.01

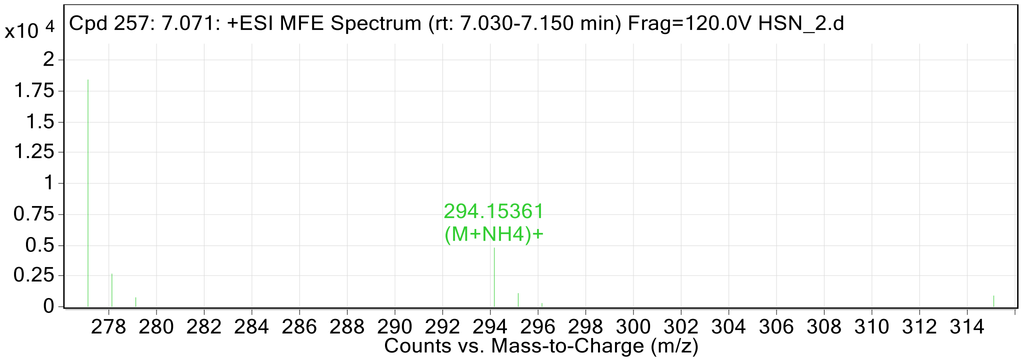

Peak List

m/z	z	Abund	Ion
277.12577	1	18444.02	(M+H)+
278.12879	1	2687.05	(M+H)+
279.12876	1	765.49	(M+H)+
294.15361	1	4801.44	(M+NH4)+
295.15329	1	1107.12	(M+NH4)+
296.1497	1	298.61	(M+NH4)+
315.08097	1	908.05	(M+K)+

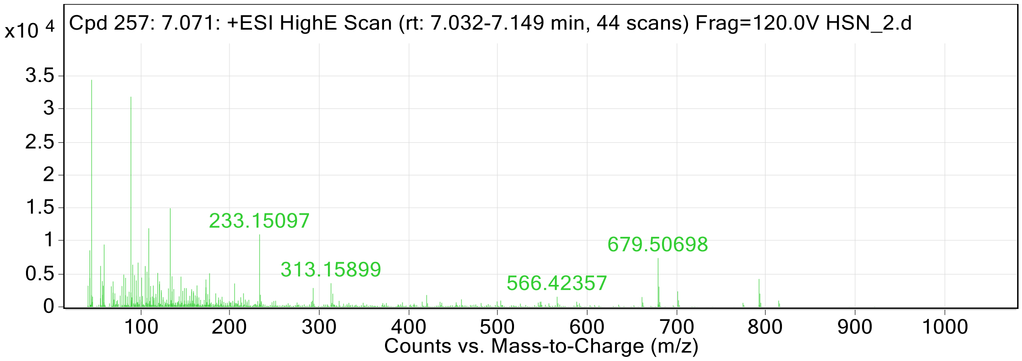

Peak List

m/z	z	Abund
43.01751		8582.52
45.03317		34437.03
59.04855		9455.95
89.05847	1	31878.5
91.05293		6400.54
97.06334		6714.2
109.06309		11921.64
133.08423		14958.98
233.15097	1	10994.61
679.50698	1	7409.7

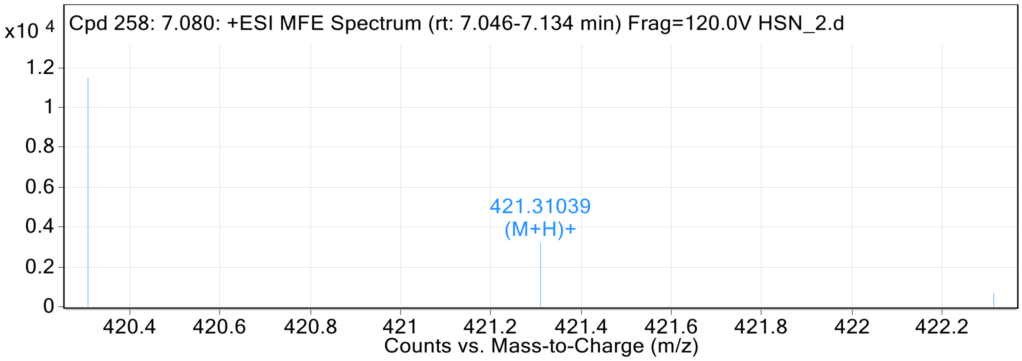

Peak List

m/z	z	Abund	Ion
420.30699	1	11467.45	(M+H)+
421.31039	1	3214.48	(M+H)+
422.31486	1	694.96	(M+H)+

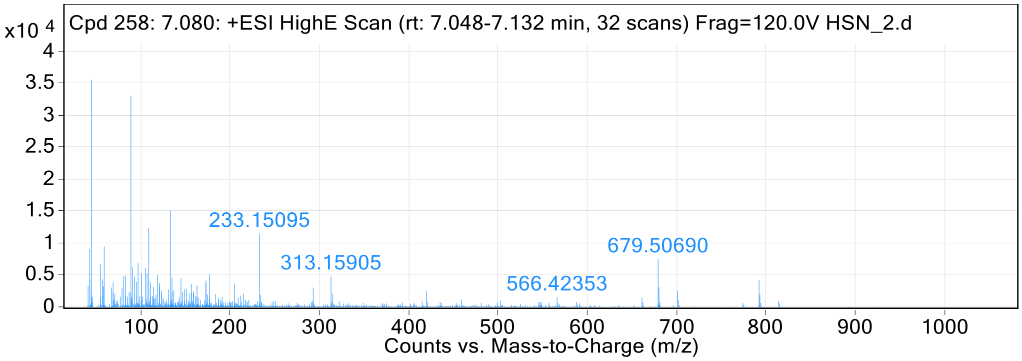

Peak List

m/z	z	Abund
43.01749		9030.93
45.03317		35480.16

55.0537		6680.48
59.04851		9449.85
89.05849	1	32975.04
97.06336		6855.91
109.0631		12317.29
133.08422		14957.83
233.15095	1	11443.95
679.5069	1	7468.77

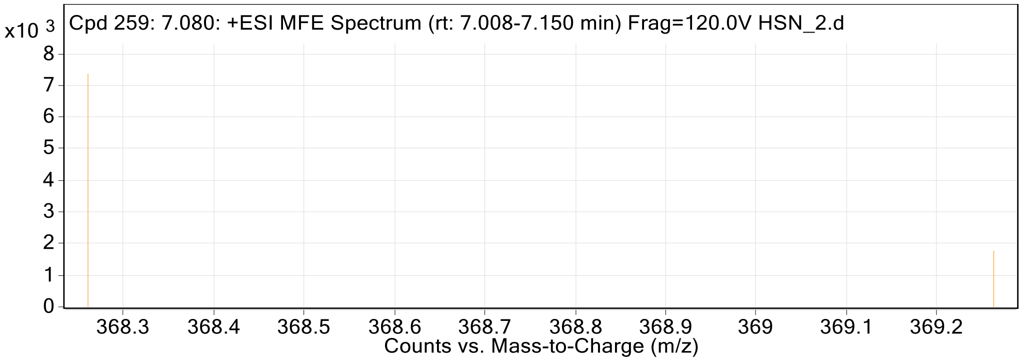

Peak List

m/z	z	Abund	Ion
368.26065	1	7367.05	(M+H)+
369.26315	1	1774.79	(M+H)+

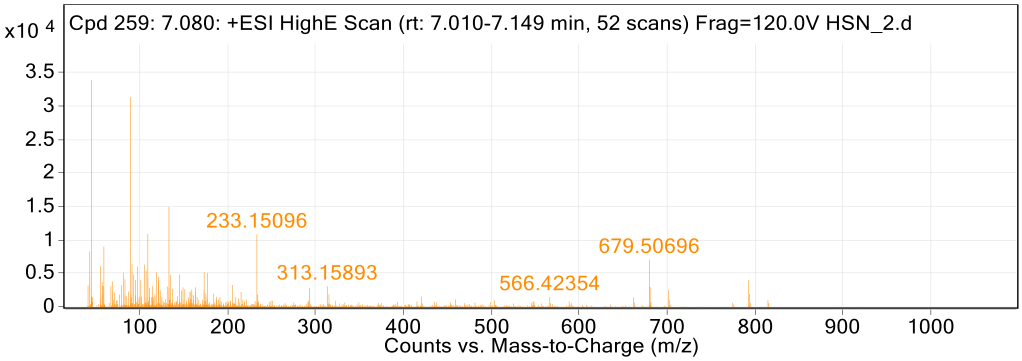

Peak List

m/z	z	Abund
43.01751		8246.05
45.03316		33855.47
59.04854		8993.48
89.05846	1	31323.17
91.0529		6404.11
105.06825		6289.55
109.06306		10896.38
133.08424		14887.93
233.15096	1	10815.54
679.50696	1	7027.84

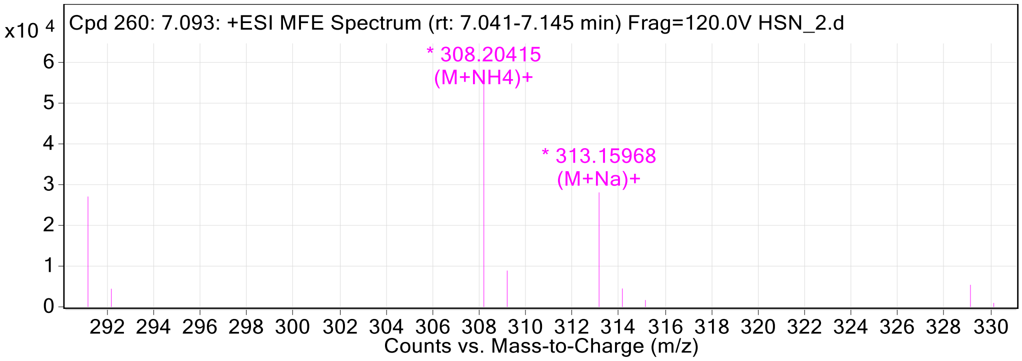

Peak List

m/z	z	Abund	Ion
291.17711	1	27050	(M+H)+
292.18126	1	4445.59	(M+H)+
308.20415	1	55776.5	(M+NH4)+
309.20702	1	8890.52	(M+NH4)+
313.15968	1	28054.29	(M+Na)+
314.16256	1	4508.69	(M+Na)+
315.15411	1	1674.36	(M+Na)+
329.13402	1	5407.52	(M+K)+
330.13705	1	955.34	(M+K)+

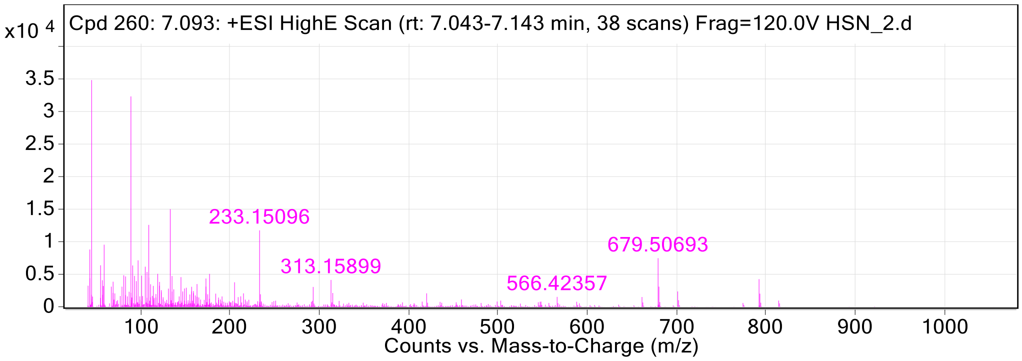

Peak List

m/z	z	Abund
43.0175		8810.84
45.03317		34855.93
55.05368		6382.03
59.04853		9546.21
89.05847	1	32348.88
97.06335		7140.91
109.06308		12592.4
133.0842		15011.88
233.15096	1	11753.11
679.50693	1	7473.97

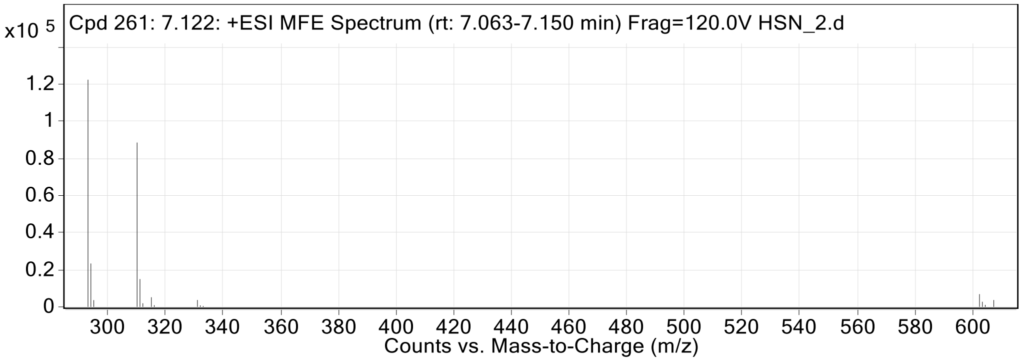
Cpd 261: 7.122: +ESI MFE Spectrum (rt: 7.063-7.150 min) Frag=120.0V HSN_2.d
x10 5
1.2
1
0.8
0.6
0.4
0.2
0
300 320 340 360 380 400 420 440 460 480 500 520 540 560 580 600
Counts vs. Mass-to-Charge (m/z)

Qualitative Analysis Report

Peak List

m/z	z	Abund	Ion
293.17163	1	122382.29	(M+H)+
294.17518	1	23371.49	(M+H)+
295.17884	1	3631.27	(M+H)+
310.1989	1	88542.92	(M+NH4)+
311.20168	1	14989.37	(M+NH4)+
315.15336	1	5189.24	(M+Na)+
331.13089	1	3718.04	(M+K)+
602.36421	1	6857.27	(2M+NH4)+
603.36803	1	2767.15	(2M+NH4)+
607.32022	1	3694.75	(2M+Na)+

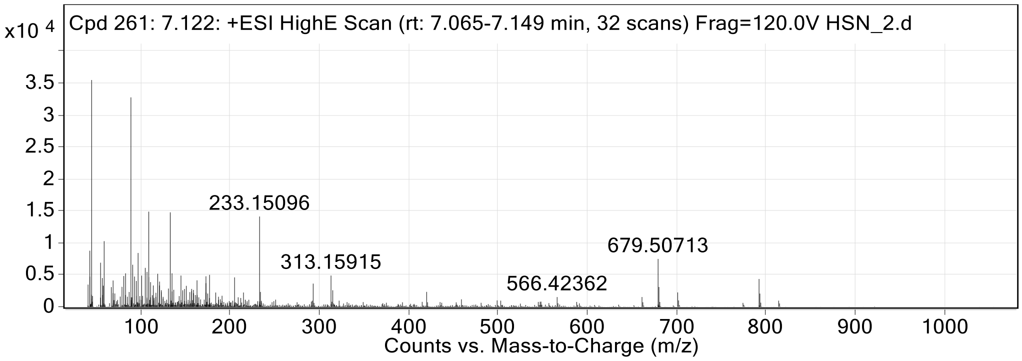

Peak List

m/z	z	Abund
43.01751		8764.03
45.03317		35456.18
55.05367		6887.22
59.04853		10260.29
89.05848	1	32743.3
97.06335		8408.87
109.0631		14870.35
133.0842		14759.53
233.15096	1	14112.37
679.50713	1	7474.71

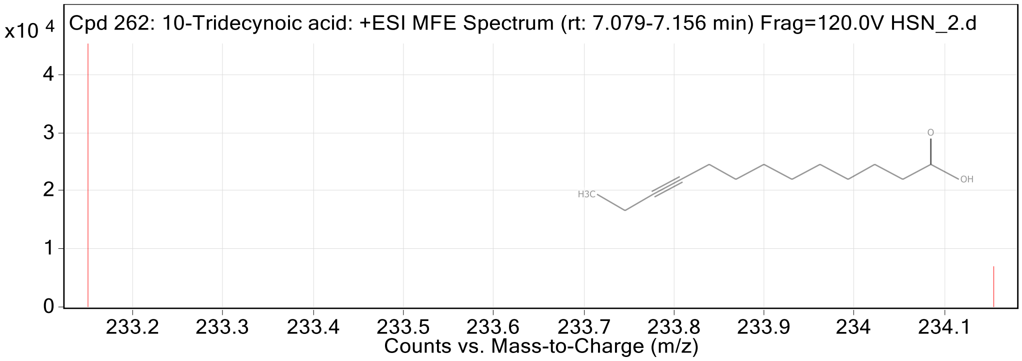

Peak List

m/z	z	Abund	Name	Formula	Ion	Score (DB)	Hits (DB)
233.15072	1	45339.91	10-Tridecynoic acid	C13 H22 O2	(M+Na)+	85.85	10
234.15445	1	7004.72		C13 H22 O2	(M+Na)+		

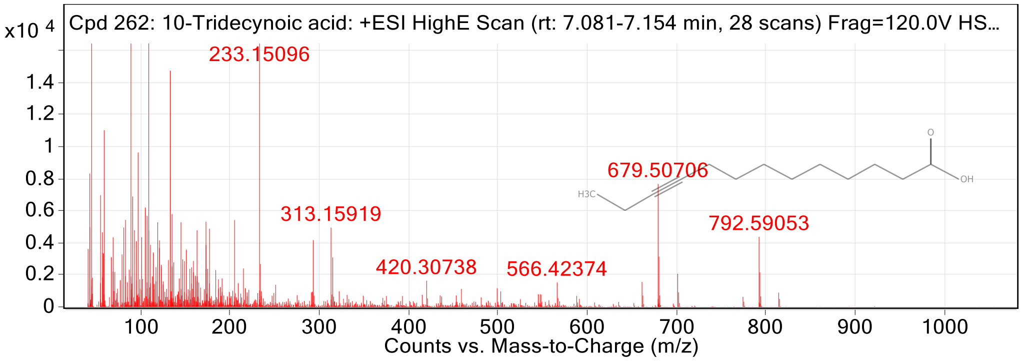

Peak List

m/z	z	Abund
43.01751		8317.62
45.03316		35333.84
55.05365		6965.9
59.04851		11018.09
89.05846	1	32692.24
97.06336		9629.65
109.06309		17177.27
133.08414		14736.07
233.15096	1	16432.37
679.50706	1	7671.8

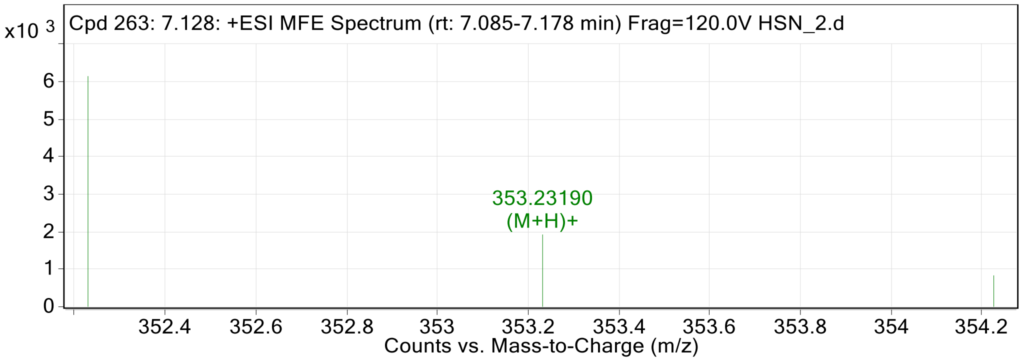

Peak List

m/z	z	Abund	Ion
352.23007	1	6149.4	(M+H)+
353.2319	1	1926.95	(M+H)+
354.22607	1	836.45	(M+H)+

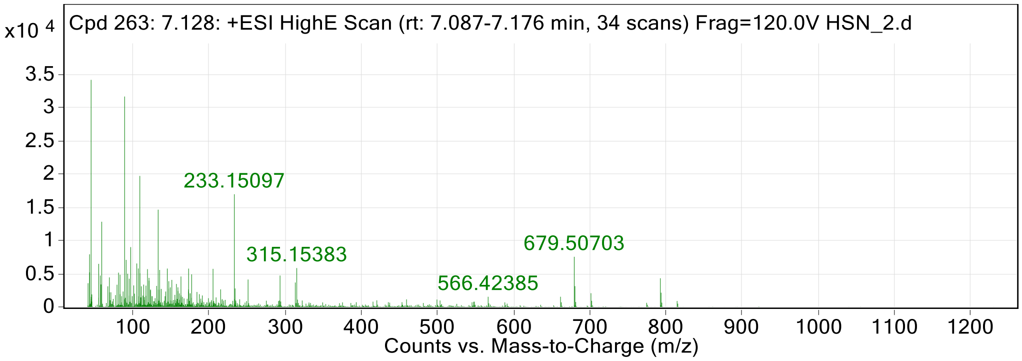

Peak List

m/z	z	Abund
43.0175		7922.75
45.03315		34195.82

59.04851		12826.11
89.05845	1	31686.33
91.05293		7081.55
97.0634		9007.28
109.0631	1	19727.97
133.08418		14643.76
233.15097	1	16967.36
679.50703	1	7548.22

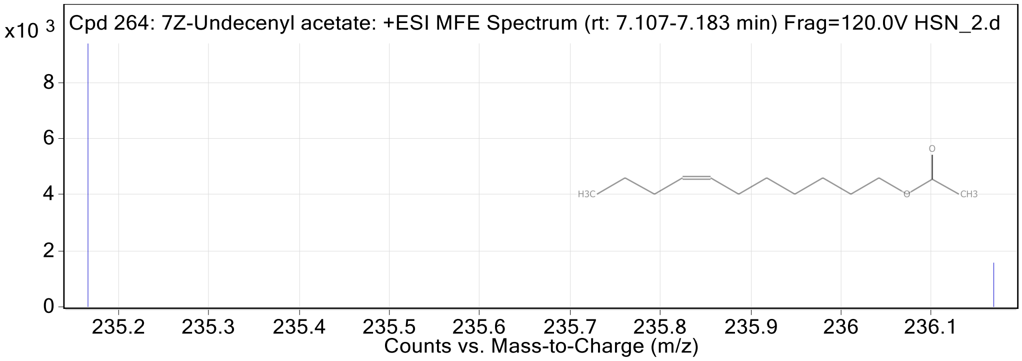

Peak List

m/z	z	Abund	Name	Formula	Ion	Score (DB)	Hits (DB)
235.16626	1	9387.91	7Z-Undecenyl acetate	C13 H24 O2	(M+Na)+	83.38	10
236.16928	1	1575.19		C13 H24 O2	(M+Na)+		

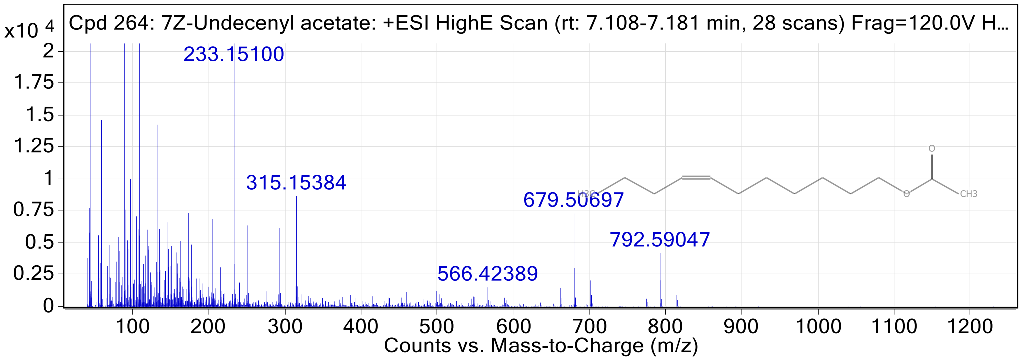

Peak List

m/z	z	Abund
43.01749		7734.06
45.03314		32126.08
59.04851		14595.4
89.05843		29766.07
91.05293		7587.34
97.06341		9975.02
109.06311	1	24213.63
133.0842		14244.78
233.151	1	20619.32
315.15384	1	8641.98

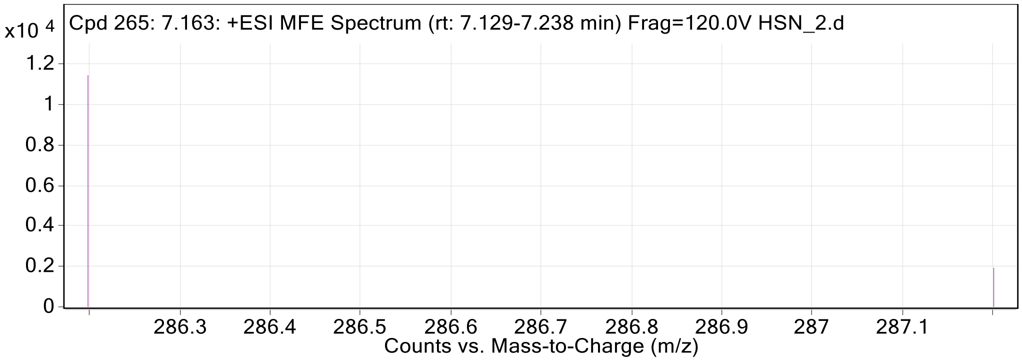

Peak List

m/z	z	Abund	Ion
286.19818	1	11452.38	(M+H)+
287.20127	1	1934.28	(M+H)+

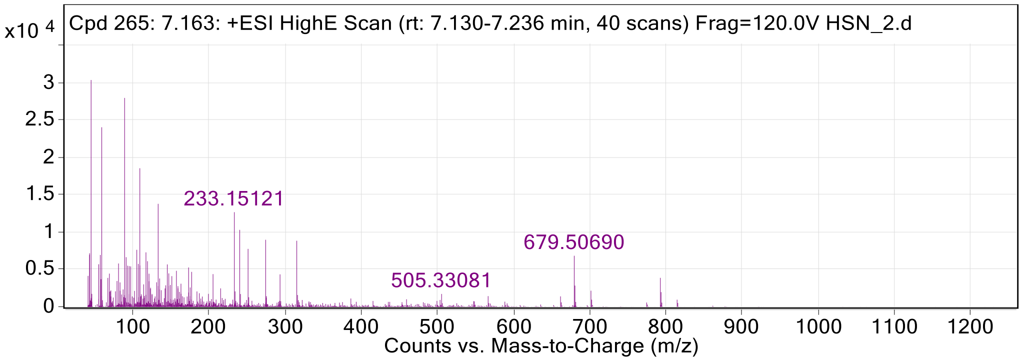

Peak List

m/z	z	Abund
45.03315		30337.41
59.04855		24011.51
89.05842		27938.04
109.06313	1	18529.65
133.08434		13767.5
233.15121	1	12650.73
240.22952	1	10283.72
251.16173		7738.42
274.27129	1	8957.01
315.15368	1	8826.59

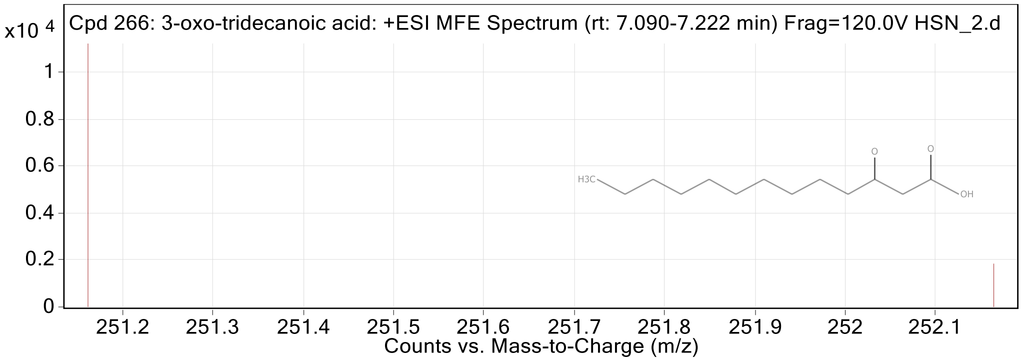

Peak List

m/z	z	Abund	Name	Formula	Ion	Score (DB)	Hits (DB)
251.16152	1	11226.82	3-oxo-tridecanoic acid	C13 H24 O3	(M+Na)+	85.11	10
252.16452	1	1845.7		C13 H24 O3	(M+Na)+		

Qualitative Analysis Report

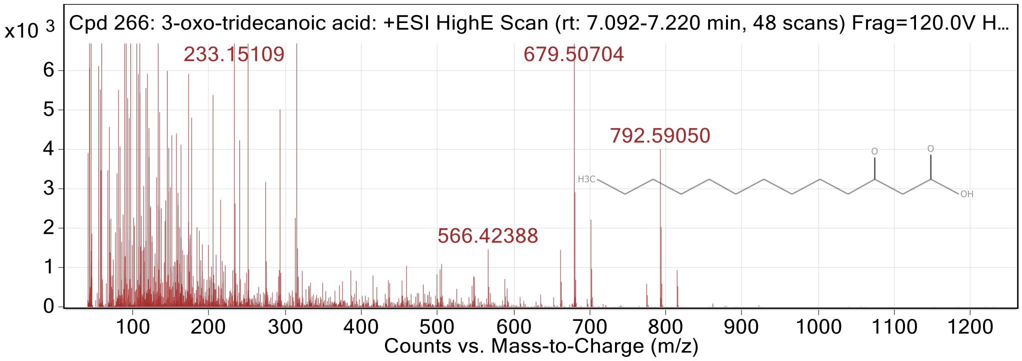

Peak List

m/z	z	Abund
43.01753		7561.85
45.03315		32555.7
59.04853		19169.53
89.05845		30026.48
97.0634		7595.67
109.06312	1	20823.95
133.0843		14159.84
233.15109	1	16163.97
315.15374	1	8256.8
679.50704	1	7095.04

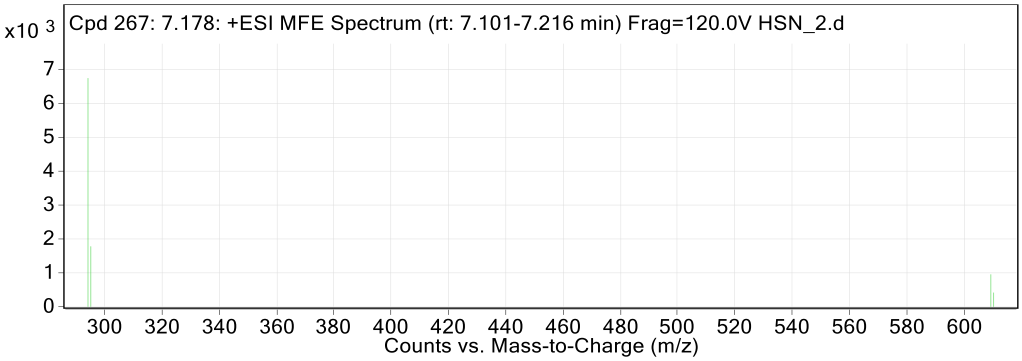

Peak List

m/z	z	Abund	Ion
294.20249	1	6738.21	(M+H)+
295.20838	1	1781.2	(M+H)+
609.37956	1	956.09	(2M+Na)+
610.3859	1	418.68	(2M+Na)+

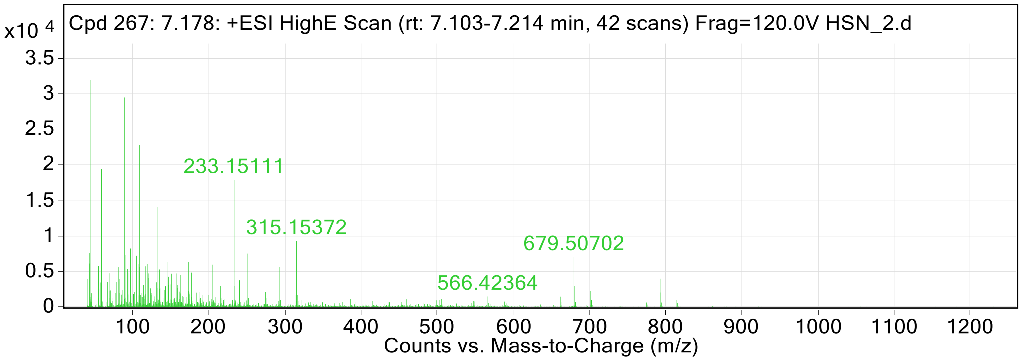

Peak List

m/z	z	Abund
43.01753		7565.9

45.03314		31968.32
59.04852		19397.15
89.05845		29498.1
97.06339		8201.8
109.06312	1	22796.73
133.0843		14037.18
233.15111	1	17889.91
251.16166		7486.15
315.15372	1	9278.67

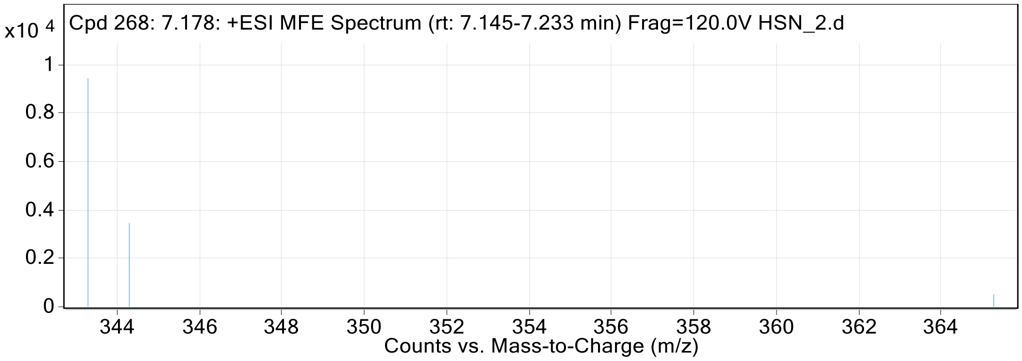

Peak List

m/z	z	Abund	Ion
343.29205	1	9453.42	(M+H)+
344.29282	1	3463.76	(M+H)+
365.27298	1	514.01	(M+Na)+

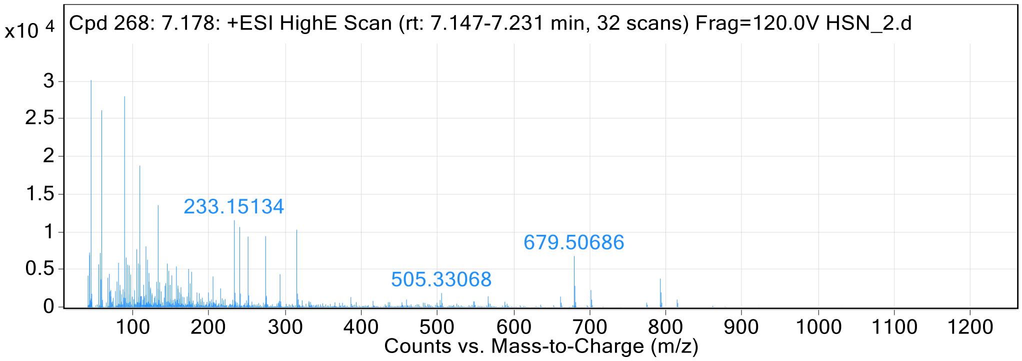

Peak List

m/z	z	Abund
45.03315		30129.58
59.04854		26114.78
89.05843		27956.72
109.06315	1	18761.04
133.08442		13523.1
233.15134	1	11524.07
240.22951	1	10614.79
251.16174	1	9341.58
274.27124	1	9407.9
315.15371	1	10247.81

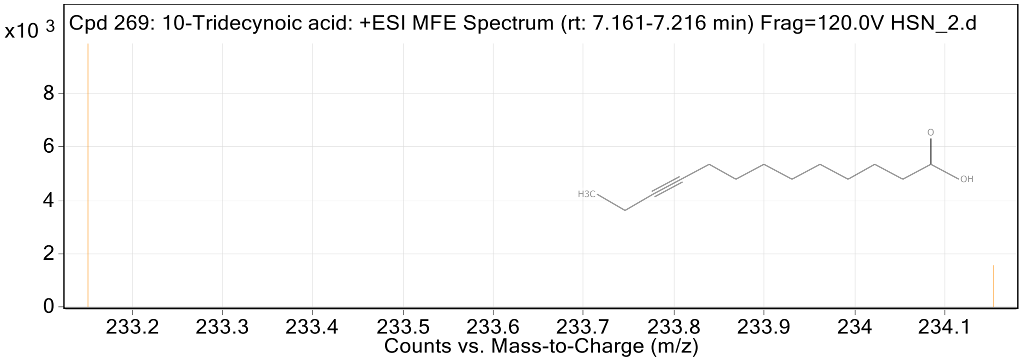

Peak List

m/z	z	Abund	Name	Formula	Ion	Score (DB)	Hits (DB)
233.15096	1	9896.45	10-Tridecynoic acid	C13 H22 O2	(M+Na)+	86.38	10
234.15434	1	1561.69		C13 H22 O2	(M+Na)+		

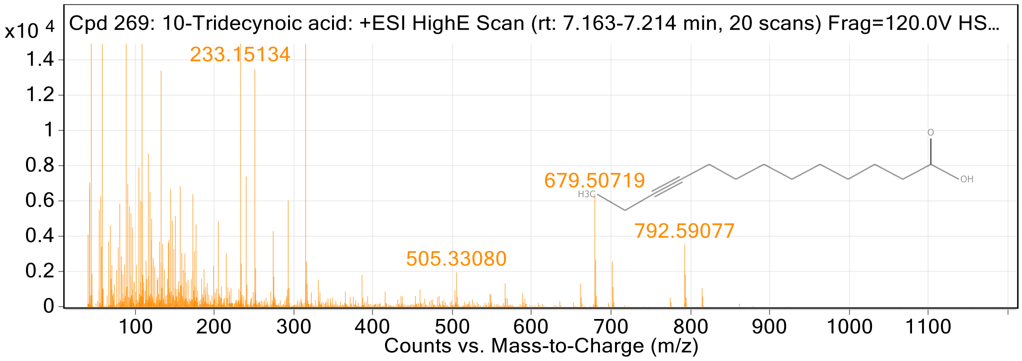

Peak List

m/z	z	Abund
45.03316		30212.2
59.04853		27630.04
89.05846		28135.14
105.0683		7878.79
109.06316	1	24606.85
117.08904		8664.91
133.0845		13365.72
233.15134	1	14893.6
251.16174	1	13434.8
315.15377	1	14871.58

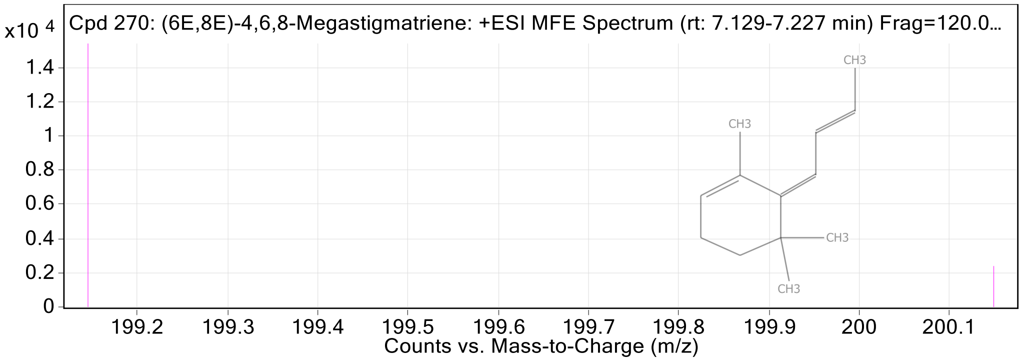

Peak List

m/z	z	Abund	Name	Formula	Ion	Score (DB)	Hits (DB)
199.14556	1	15402.33	(6E,8E)-4,6,8-Megastigmatriene	C13 H20	(M+Na)+	87.07	2
200.14905	1	2392.43		C13 H20	(M+Na)+		

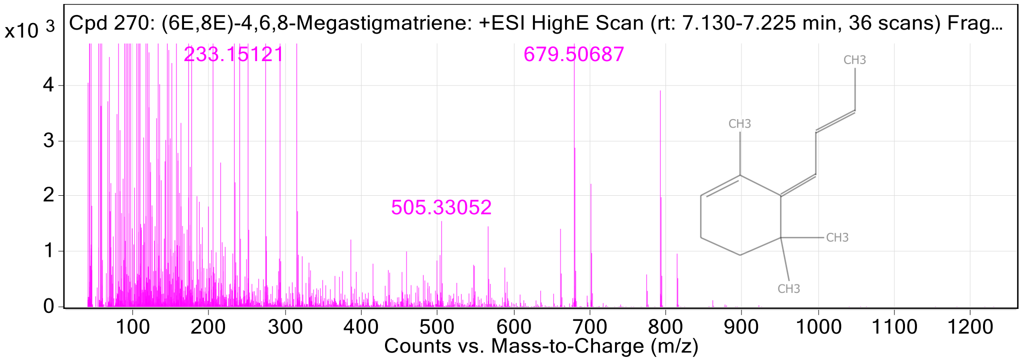

Peak List

m/z	z	Abund
45.03316		30743.54
59.04854		23269.97
89.05842		28410.49
105.06826		7542.09
109.06314	1	20161.66
133.08435		13810.67
233.15121	1	13839.13
240.2295		7433.71
251.16171	1	8501.61
315.15369	1	9719.81

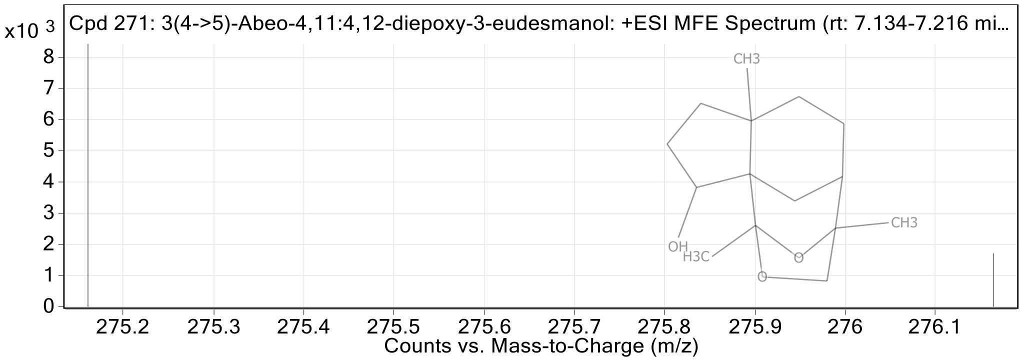

Peak List

m/z	z	Abund	Name	Formula	Ion	Score (DB)	Hits (DB)
275.16103	1	8448.35	3(4->5)-Abeo-4,11:4,12-diepoxy-3-eudesmanol	C15 H24 O3	(M+Na)+	80.74	10
276.16433	1	1717.34		C15 H24 O3	(M+Na)+		

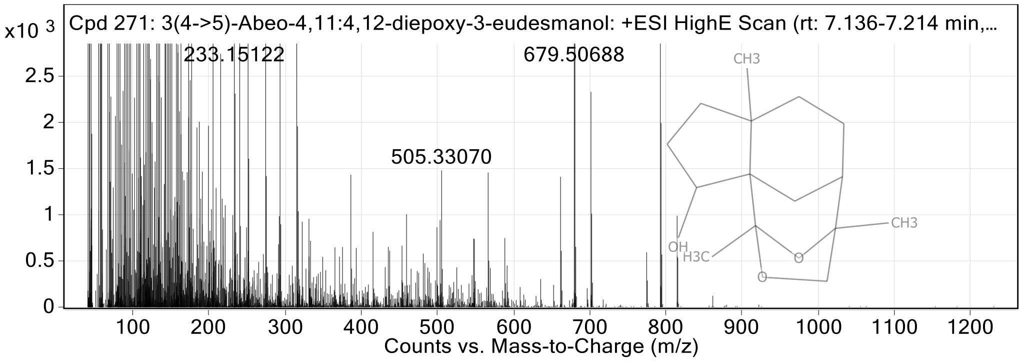

Peak List

m/z	z	Abund
43.01757		7160.62
45.03316		30905.49

59.04854		22925.06
89.05842		28632.96
105.06826		7525.66
109.06313	1	21625.62
133.08439		13812.66
233.15122	1	14361.28
251.16167	1	9881.99
315.15371	1	11179.96

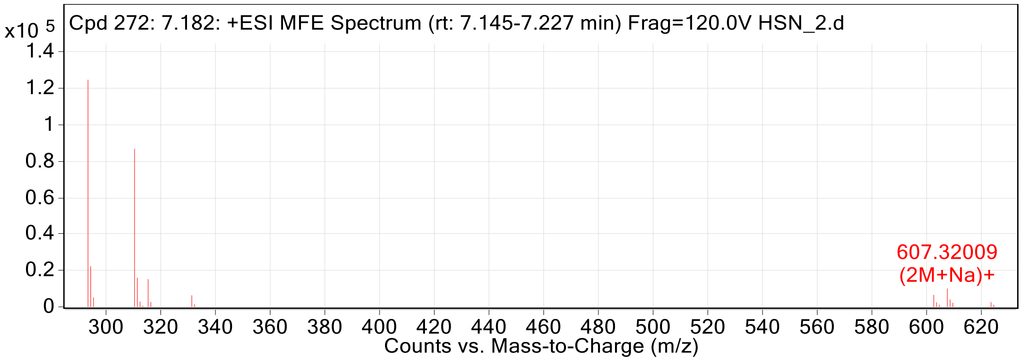

Peak List

m/z	z	Abund	Ion
293.17234	1	124801	(M+H)+
294.17551	1	22265.5	(M+H)+
295.18083	1	5150.22	(M+H)+
310.19859	1	86880.35	(M+NH4)+
311.20046	1	15984.35	(M+NH4)+
315.15361	1	15222.18	(M+Na)+
331.12788	1	6348.69	(M+K)+
602.36446	1	6591.2	(2M+NH4)+
607.32009	1	10081.87	(2M+Na)+
608.32398	1	4044.04	(2M+Na)+

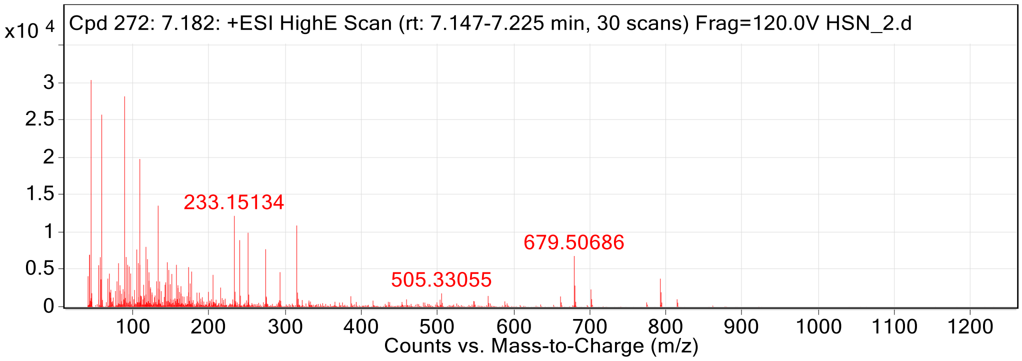

Peak List

m/z	z	Abund
45.03315		30350.06
59.04854		25709.35
89.05843		28163.63
109.06315	1	19772.73
117.08908		8012.39
133.08443		13529.96
233.15134	1	12162.17
240.2295	1	8913.33
251.16172	1	9901.95
315.15372	1	10873.95

Qualitative Analysis Report

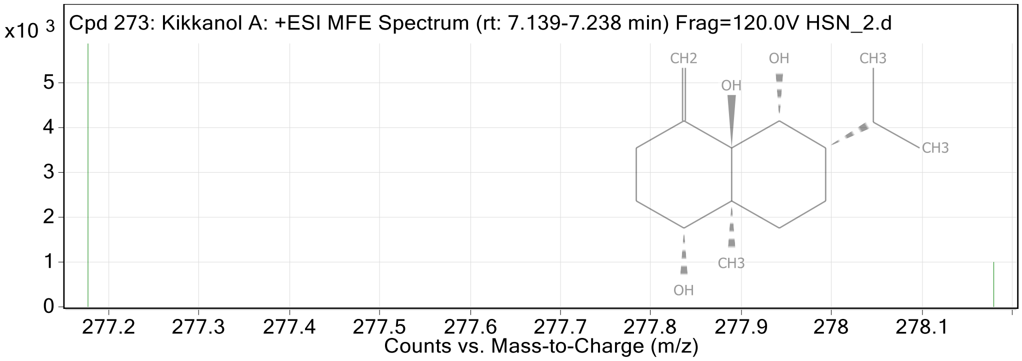

Peak List

m/z	z	Abund	Name	Formula	Ion	Score (DB)	Hits (DB)
277.17675	1	5886.65	Kikkanol A	C15 H26 O3	(M+Na)+	84.34	10
278.17977	1	1003.16		C15 H26 O3	(M+Na)+		

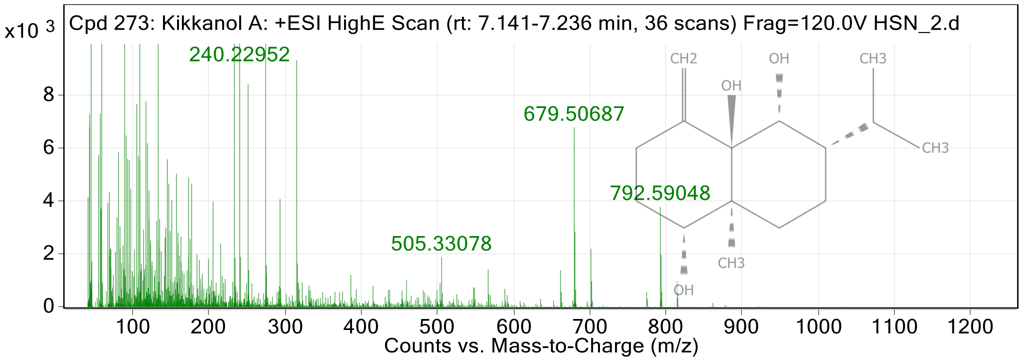

Peak List

m/z	z	Abund
45.03315		30102.21
59.04855		25500.13
89.05842		27760.14
109.06314	1	17823.56
133.08438		13646.06
233.15128	1	11210.77
240.22952	1	11426.36
251.16174	1	8415.27
274.27129	1	9952.23
315.15369	1	9318.64

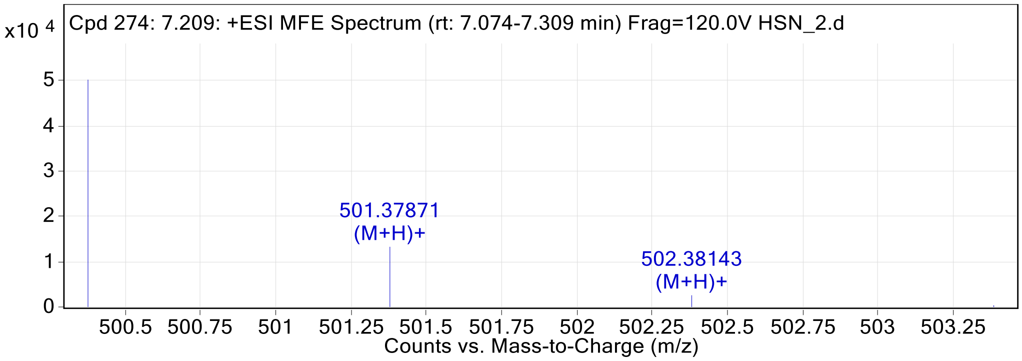

Peak List

m/z	z	Abund	Ion
500.37572	1	50239.67	(M+H)+
501.37871	1	13303.51	(M+H)+
502.38143	1	2579.36	(M+H)+

503.38477	1	366.16	(M+H)+

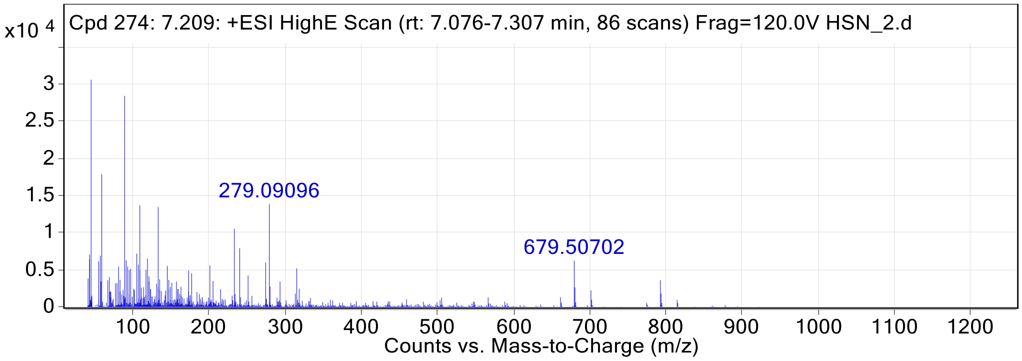

Peak List

m/z	z	Abund
43.01753		7023.86
45.03315		30584.13
59.04853		17872.21
89.05846		28401.11
105.06826		7163.01
109.06314		13657.16
133.08434		13430.4
233.15112	1	10504.17
240.22952		7889.65
279.09096	1	13824.59

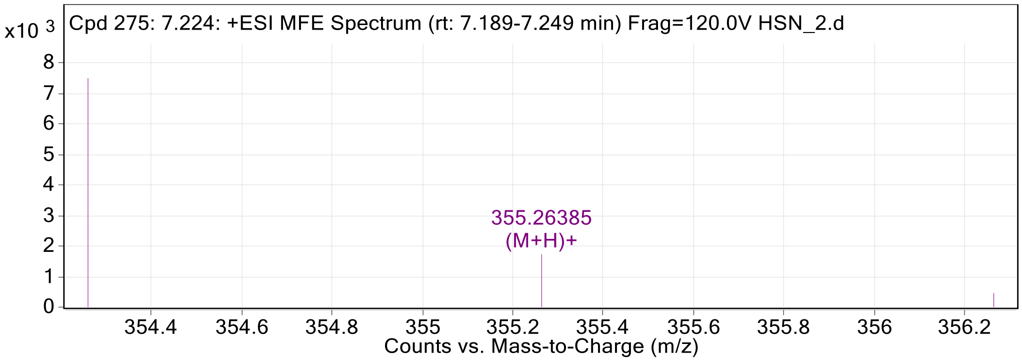

Peak List

m/z	z	Abund	Ion
354.26018	1	7500.5	(M+H)+
355.26385	1	1733.83	(M+H)+
356.26425	1	461.48	(M+H)+

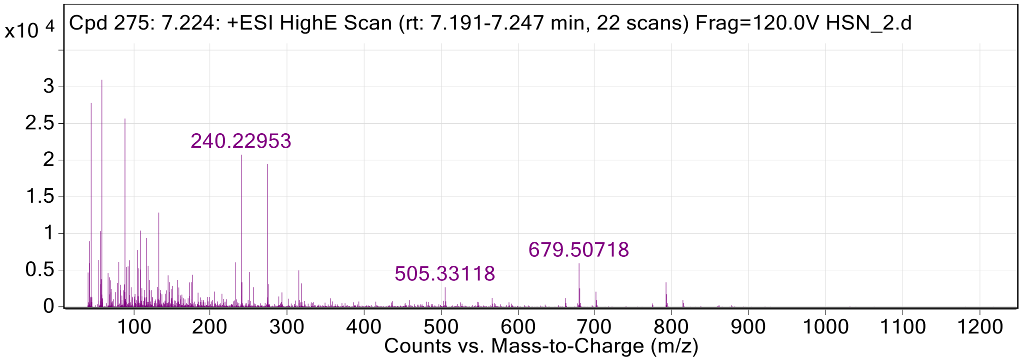

Peak List

m/z	z	Abund
43.05391		8948.67

45.03314		27825.39
57.0693		10331.65
59.04855		30996.27
89.05846		25706.58
109.06317		10402.1
117.08913		9415.8
133.08446		12859.98
240.22953	1	20770.98
274.2713	1	19490.38

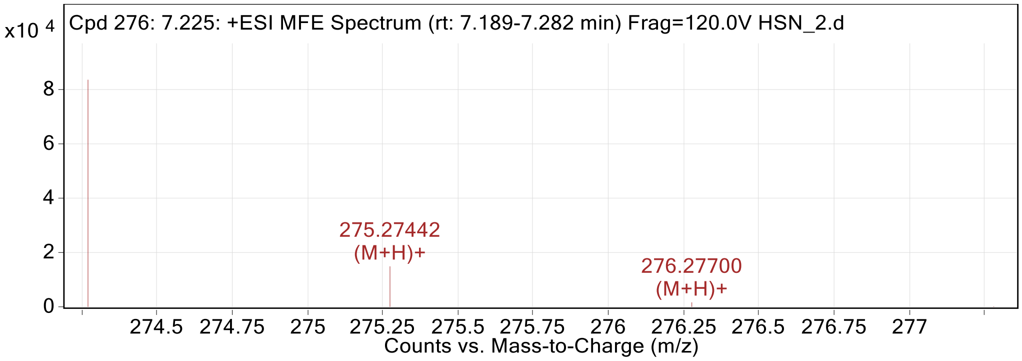

Peak List

m/z	z	Abund	Ion
274.27139	1	83843.33	(M+H)+
275.27442	1	14946.59	(M+H)+
276.277	1	1648.54	(M+H)+
277.27991	1	172.32	(M+H)+

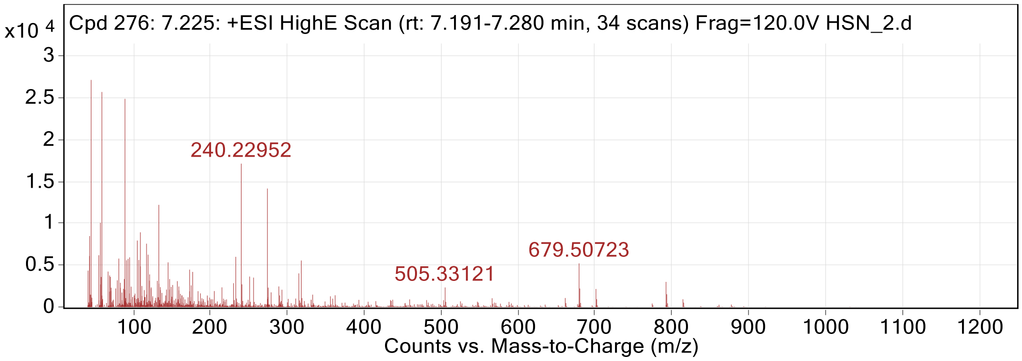

Peak List

m/z	z	Abund
43.05388		8476.37
45.03314		27121.62
57.0693		10059.68
59.04854		25680.88
89.05845		24860.07
105.06823		7933.3
109.06318		8916.16
133.0845		12198.07
240.22952	1	17121.06
274.2713	1	14147.02

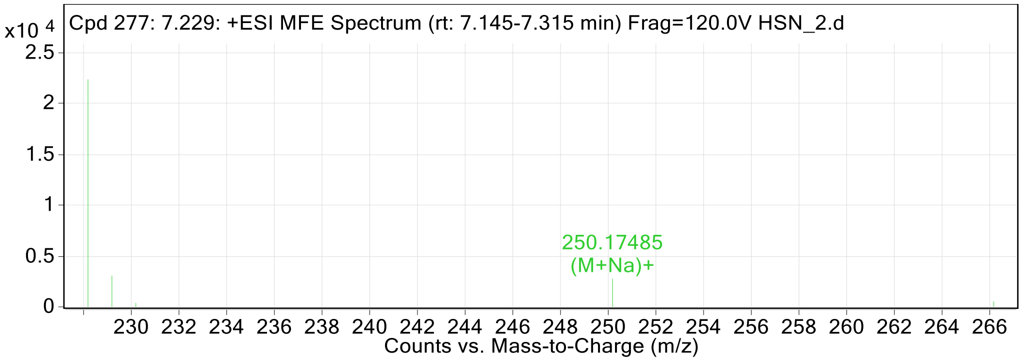

Peak List

m/z	z	Abund	Ion
228.19332	1	22363.06	(M+H)+
229.19619	1	3069.47	(M+H)+
230.1996	1	383.21	(M+H)+
250.17485	1	2754.47	(M+Na)+
266.14801	1	560.07	(M+K)+

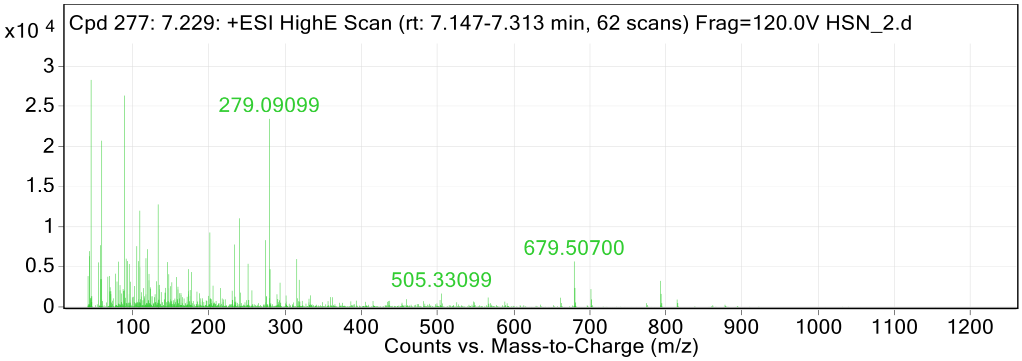

Peak List

m/z	z	Abund
45.03315		28267.69
59.04854		20716.38
89.05845		26328.07
109.06316		11993.67
133.08445		12760.28
201.04398		9267.52
233.15128		7771.69
240.22953	1	11024.42
274.27131	1	8307.14
279.09099	1	23438.2

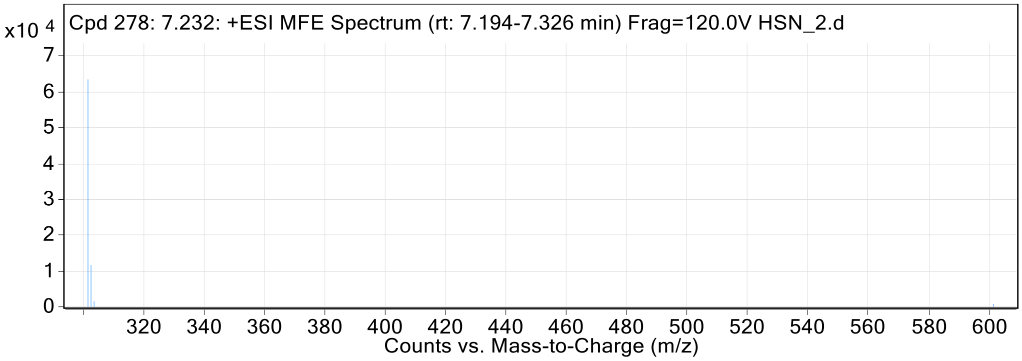

Peak List

m/z	z	Abund	Ion

301.28198	1	63496.37	(M+H)+
302.28503	1	11684.09	(M+H)+
303.28829	1	1549.46	(M+H)+
601.55637	1	796.64	(2M+H)+

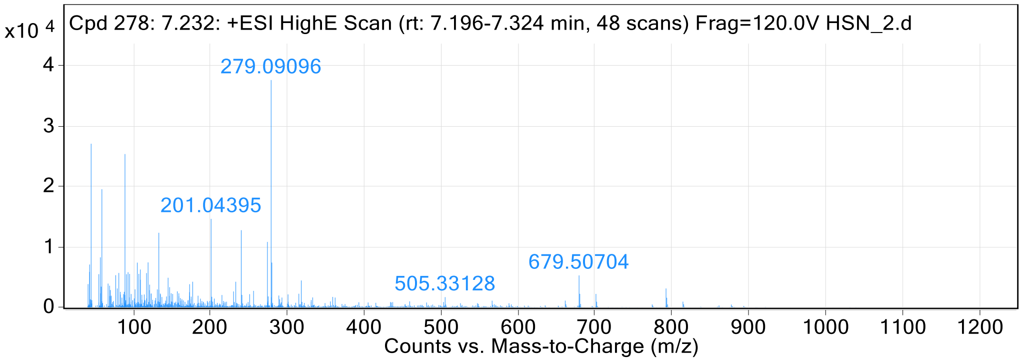

Peak List

m/z	z	Abund
45.03316		27085.85
57.0693		8248.51
59.04854		19545.69
89.05846		25375.49
119.0836		7410.28
133.0845		12322.57
201.04395	1	14624.33
240.22953	1	12751.26
274.27131	1	10801.65
279.09096	1	37646.39

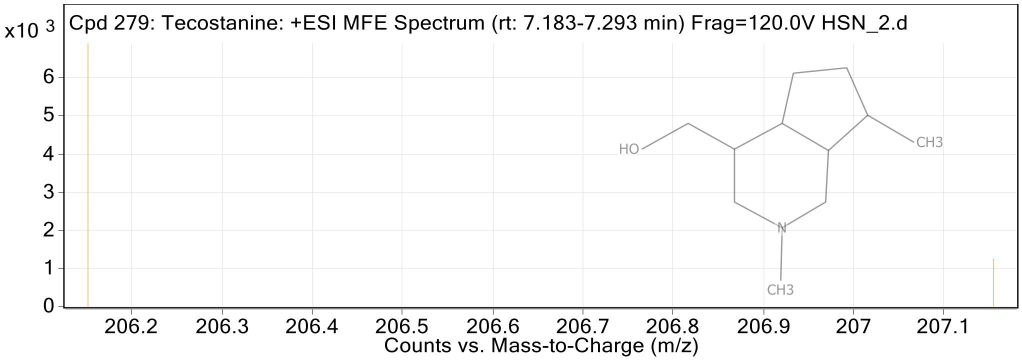

Peak List

m/z	z	Abund	Name	Formula	Ion	Score (DB)	Hits (DB)
206.15142	1	6901.86	Tecostanine	C11 H21 N O	(M+Na)+	78.48	1
207.15543	1	1259.83		C11 H21 N O	(M+Na)+		

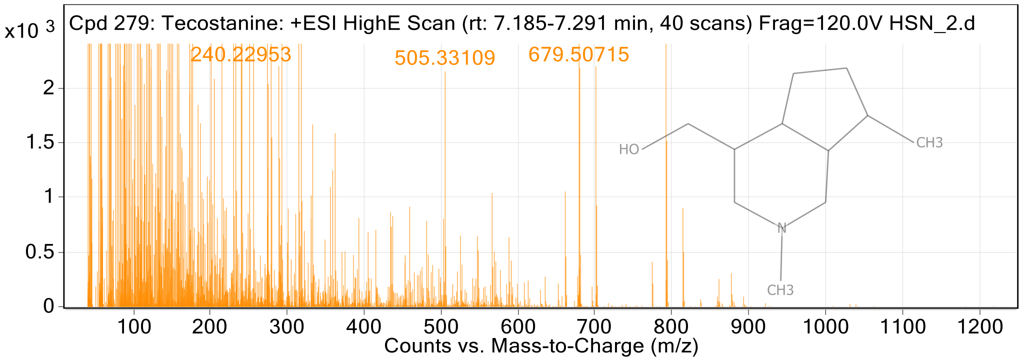

Peak List

m/z	z	Abund
43.05388		8047.36
45.03316		27332.13
57.06931		9424.48
59.04854		24174.76
89.05846		25206.86
109.06317		9822.33
133.08448		12261.68
240.22953	1	15242.21
274.2713	1	12370.04
279.09085	1	8596.03

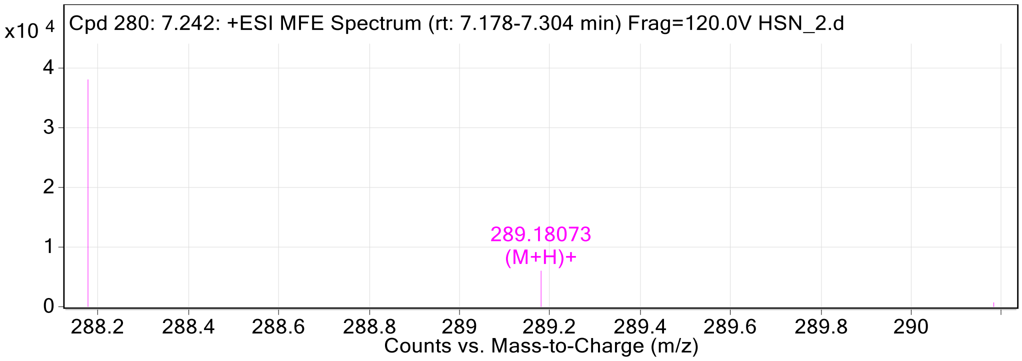

Peak List

m/z	z	Abund	Ion
288.17789	1	38187.59	(M+H)+
289.18073	1	6058.74	(M+H)+
290.1827	1	730.32	(M+H)+

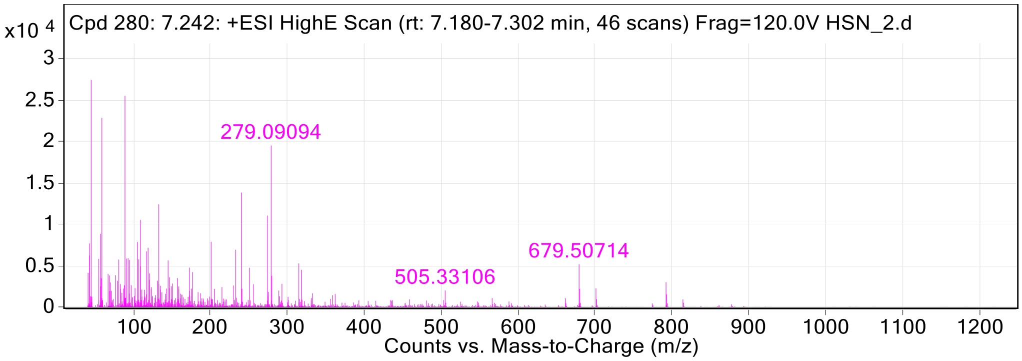

Peak List

m/z	z	Abund
45.03315		27446.22
57.06931		8826.57
59.04854		22854.59
89.05846		25508.15
109.06317		10523.53
133.08448		12390.27
201.0441		7868.64
240.22953	1	13819.71
274.27131	1	11033.19
279.09094	1	19515.05

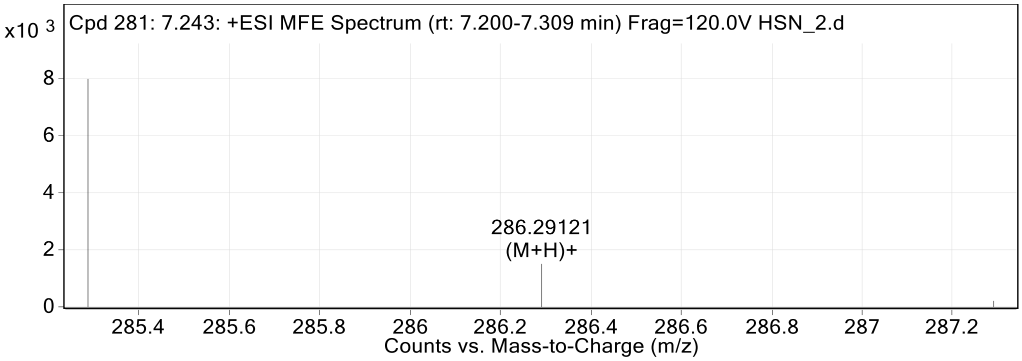

Peak List

m/z	z	Abund	Ion
285.28724	1	7989.84	(M+H)+
286.29121	1	1510.59	(M+H)+
287.2913	1	212.24	(M+H)+

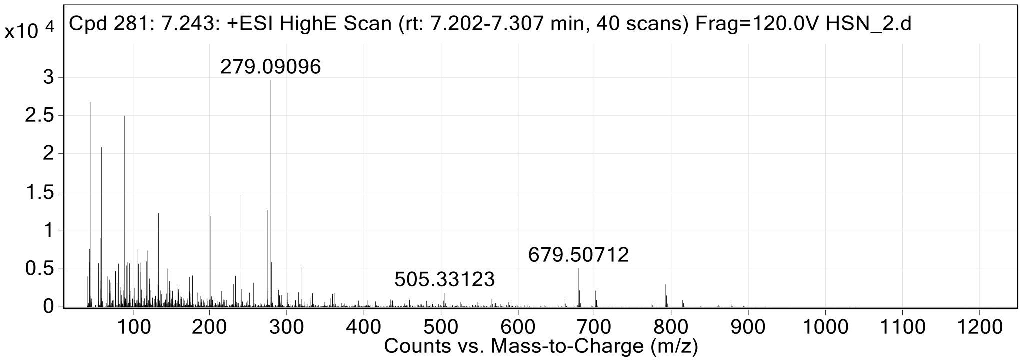

Peak List

m/z	z	Abund
43.05388		7623.28
45.03315		26841.77
57.06931		9066.36
59.04855		20922.22
89.05845		25028.06
133.08449		12275.56
201.04401	1	11936.13
240.22952	1	14663.54
274.27131	1	12725.62
279.09096	1	29710.35

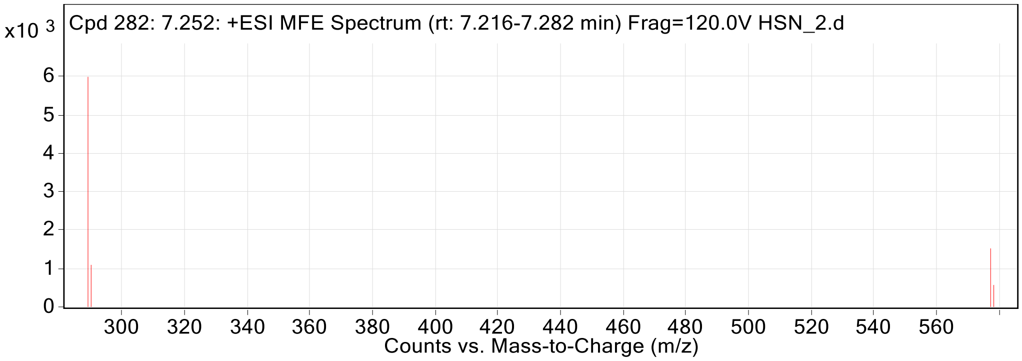

Peak List

m/z	z	Abund	Ion
289.13817	1	5987.51	(M+H)+
290.14223	1	1094.41	(M+H)+

577.2713	1	1522.22	(2M+H)+
578.27765	1	570.24	(2M+H)+

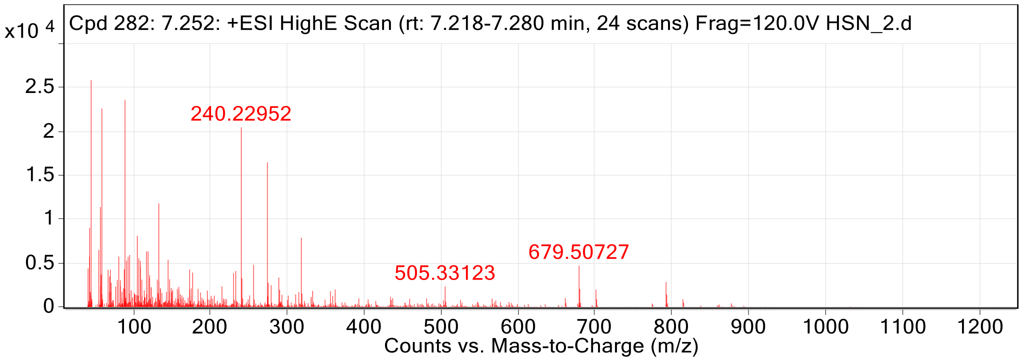

Peak List

m/z	z	Abund
43.05386		9018.51
45.03314		25870.84
57.06929		11391.15
59.04855		22631.92
89.05843		23608.55
105.06825		8097.84
133.08444		11812.18
240.22952	1	20473.03
274.27128	1	16473.81
318.29731	1	7872.02

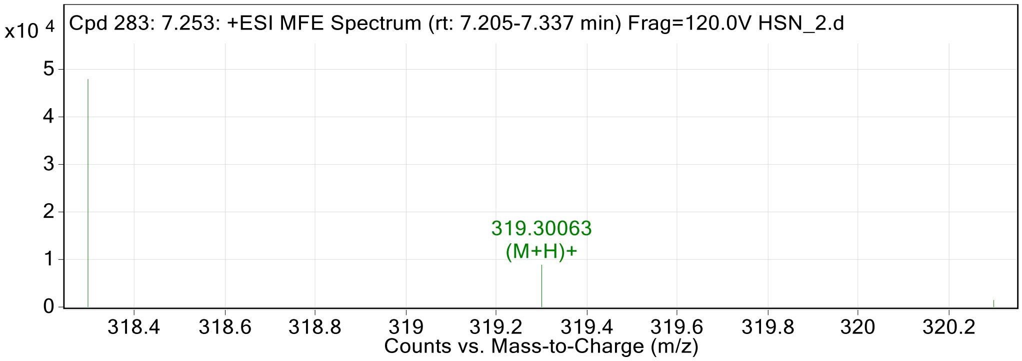

Peak List

m/z	z	Abund	Ion
318.29725	1	48020.41	(M+H)+
319.30063	1	8897.13	(M+H)+
320.30009	1	1467.26	(M+H)+

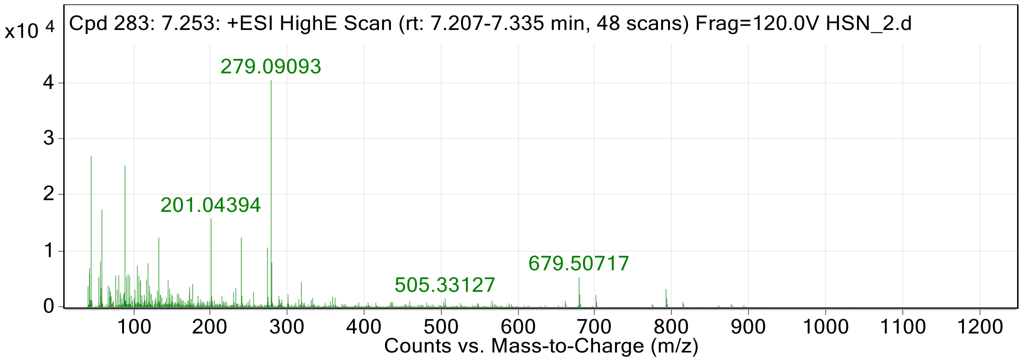

Peak List

m/z	z	Abund

45.03316		26970.37
57.06931		8049.8
59.04856		17346.65
89.05845		25274.15
133.08448		12344.95
201.04394	1	15751.35
240.22953	1	12362.44
274.2713	1	10590.26
279.09093	1	40499.36
280.09407	1	7879.2

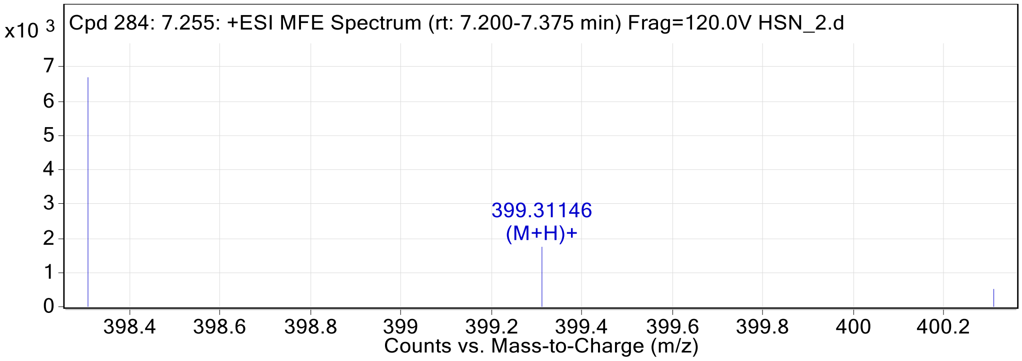

Peak List

m/z	z	Abund	Ion
398.30776	1	6699.1	(M+H)+
399.31146	1	1750.17	(M+H)+
400.31043	1	519.1	(M+H)+

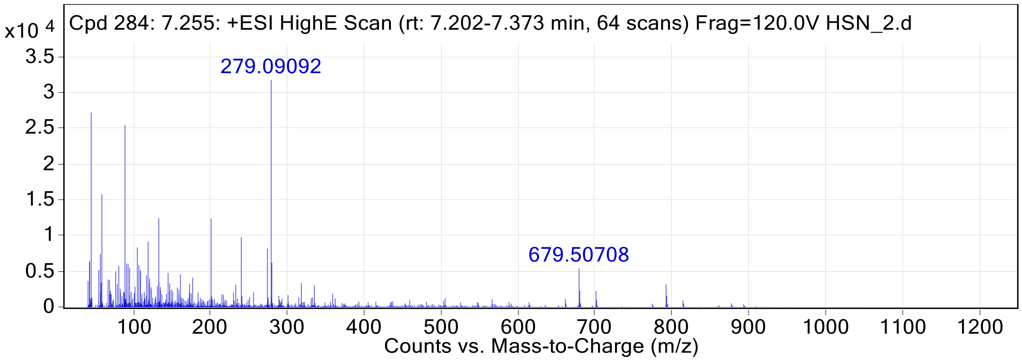

Peak List

m/z	z	Abund
45.03316		27192.56
59.04855		15750.29
89.05844		25453.53
105.06826		8330.38
119.0836		9131.88
133.08462		12410.71
201.04393	1	12329.68
240.22952	1	9748.46
274.2713	1	8145.21
279.09092	1	31744.32

Qualitative Analysis Report

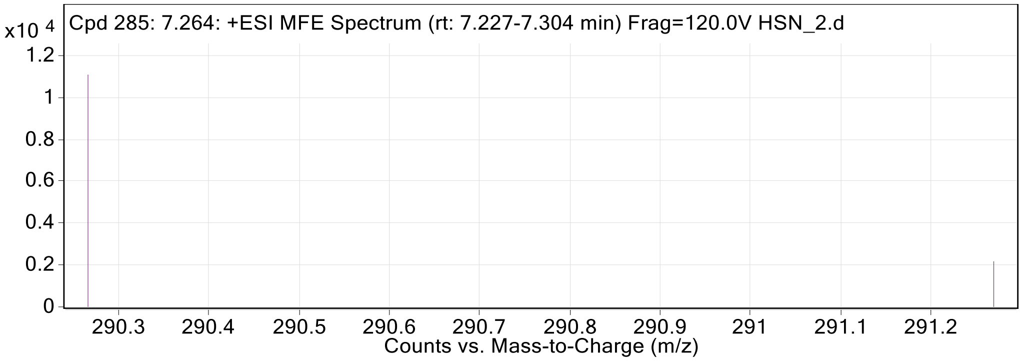

Peak List

m/z	z	Abund	Ion
290.26617	1	11084.49	(M+H)+
291.26958	1	2175.04	(M+H)+

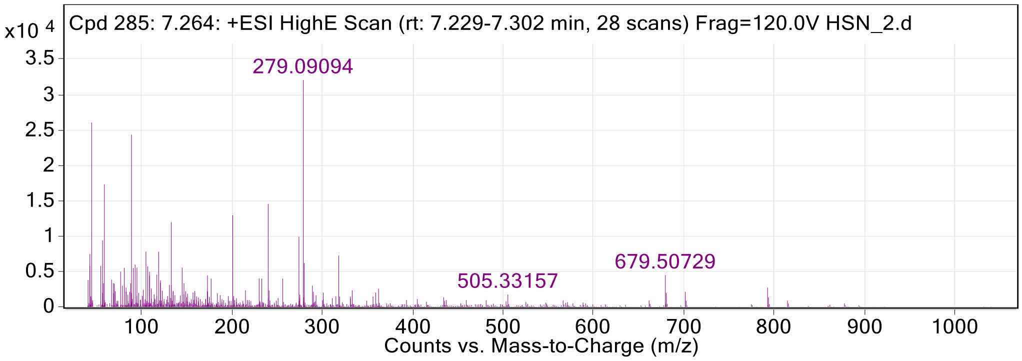

Peak List

m/z	z	Abund
45.03314		26040.29
57.0693		9369.82
59.04854		17304.46
89.05846		24315.36
105.06825		7782.42
133.08445		11940.25
201.0441	1	12927.05
240.22955	1	14535.3
274.27133	1	9878.09
279.09094	1	32052.27

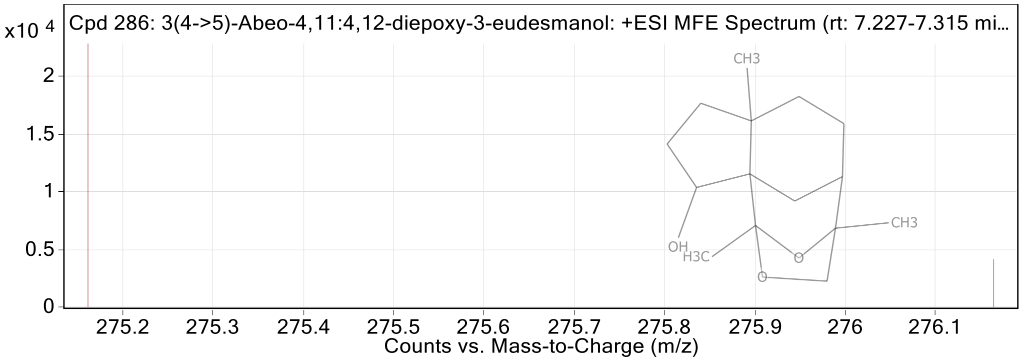

Peak List

m/z	z	Abund	Name	Formula	Ion	Score (DB)	Hits (DB)
275.16144	1	22854.13	3(4->5)-Abeo-4,11:4,12-diepoxy-3-eudesmanol	C15 H24 O3	(M+Na)+	85.37	10
276.16467	1	4141.55		C15 H24 O3	(M+Na)+		

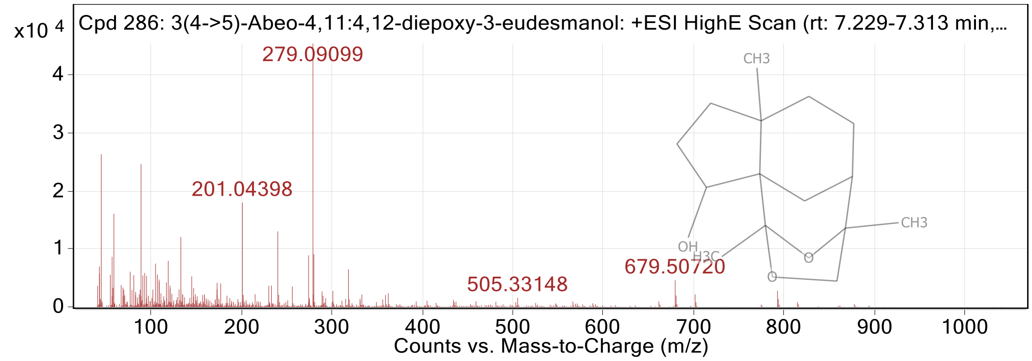

Peak List

m/z	z	Abund
45.03314		26315.47
57.0693		8643.8
59.04854		16035.48
89.05847		24607.22
133.08447		12038.7
201.04398	1	17955.82
240.22955	1	13003.57
274.27133	1	8878.21
279.09099	1	45404.36
280.09411	1	9078.82

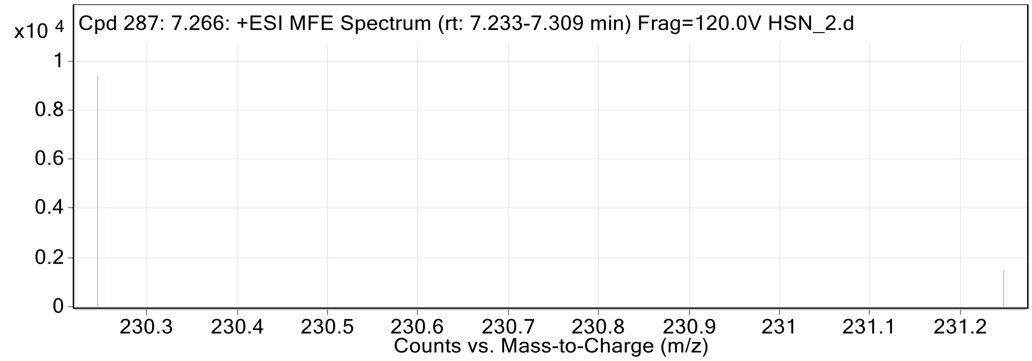

Peak List

m/z	z	Abund	Ion
230.24508	1	9393.19	(M+H)+
231.2487	1	1491.71	(M+H)+

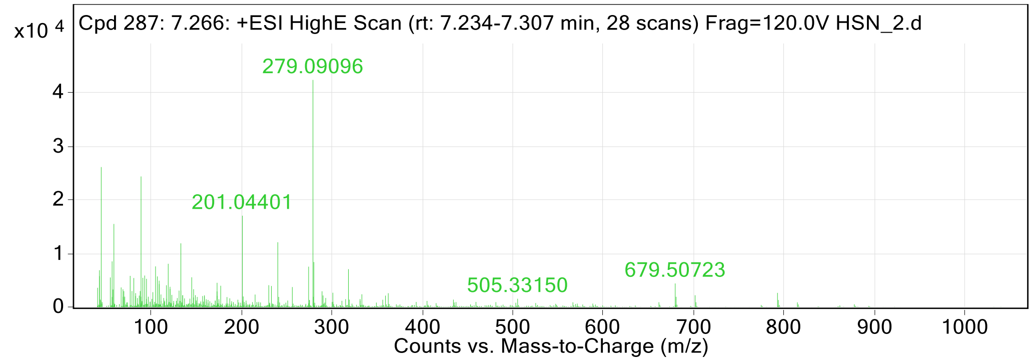

Peak List

m/z	z	Abund
45.03314		26140.45
57.06931		8522.88
59.04853		15510.9

89.05846		24387.94
119.08364		8067.55
133.08448		11888.79
201.04401	1	17051.62
240.22953	1	12093.69
279.09096	1	42424.89
280.09417	1	8393.97

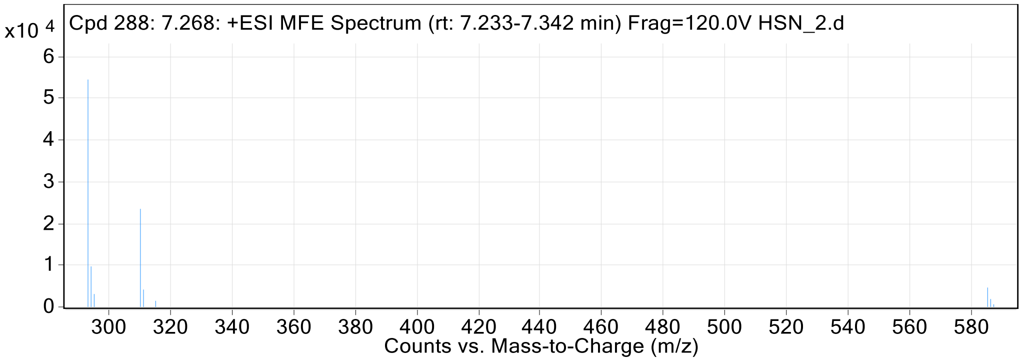

Peak List

m/z	z	Abund	Ion
293.17174	1	54537.31	(M+H)+
294.17531	1	9721.92	(M+H)+
295.18183	1	3099.28	(M+H)+
310.19821	1	23528.41	(M+NH4)+
311.20075	1	4168.82	(M+NH4)+
315.15431	1	1470.01	(M+Na)+
585.33845	1	4648.7	(2M+H)+
586.34204	1	1898.06	(2M+H)+
587.34016	1	630.49	(2M+H)+

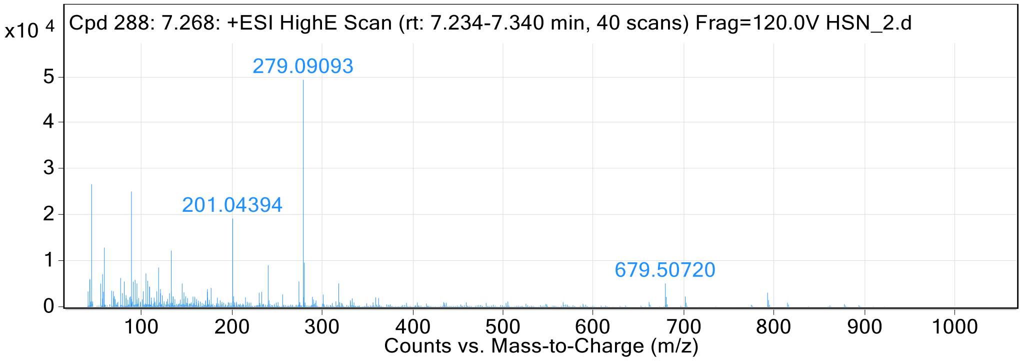

Peak List

m/z	z	Abund
45.03316		26650.08
59.04855		12859.68
89.05845		25074.26
105.06827		7248.77
119.08363		8543.91
133.0845		12204.62
201.04394	1	19179.99
240.22955	1	9028.4
279.09093	1	49348.65
280.09407	1	9596.38

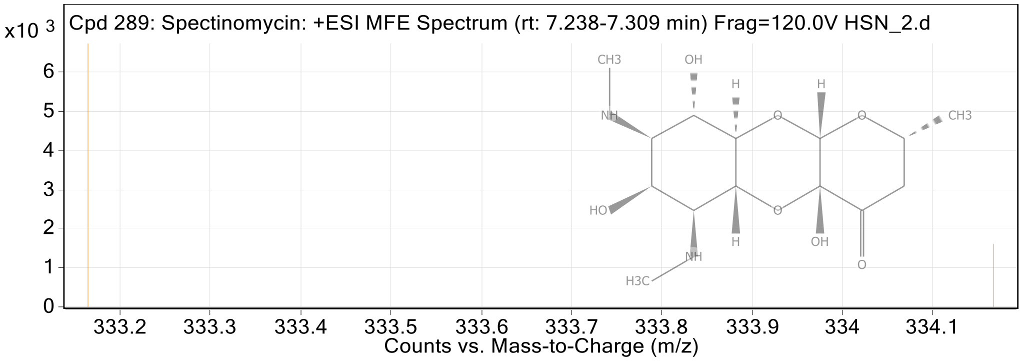

Peak List

m/z	z	Abund	Name	Formula	Ion	Score (DB)	Hits (DB)
333.16427	1	6730.56	Spectinomycin	C14 H24 N2 O7	(M+H)+	69.5	1
334.16774	1	1605.27		C14 H24 N2 O7	(M+H)+		

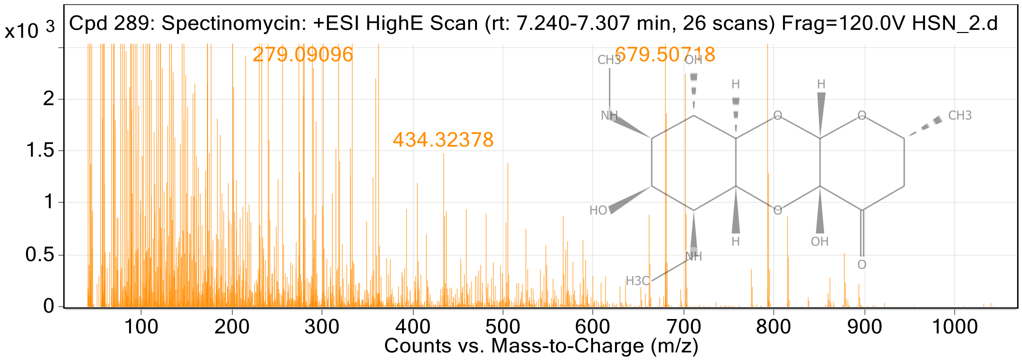

Peak List

m/z	z	Abund
45.03314		26109.55
57.06932		8178.72
59.04851		14459.85
89.05846		24531.69
119.08363		8293.4
133.0845		11777.34
201.04401	1	18363.28
240.22952	1	10275.43
279.09096	1	45688.35
280.09417	1	9039.66

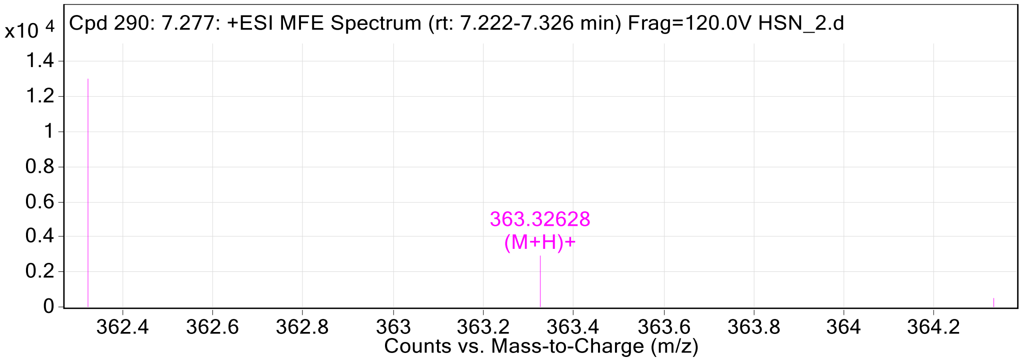

Peak List

m/z	z	Abund	Ion
362.32322	1	13021.12	(M+H)+
363.32628	1	2929.34	(M+H)+
364.33199	1	508.13	(M+H)+

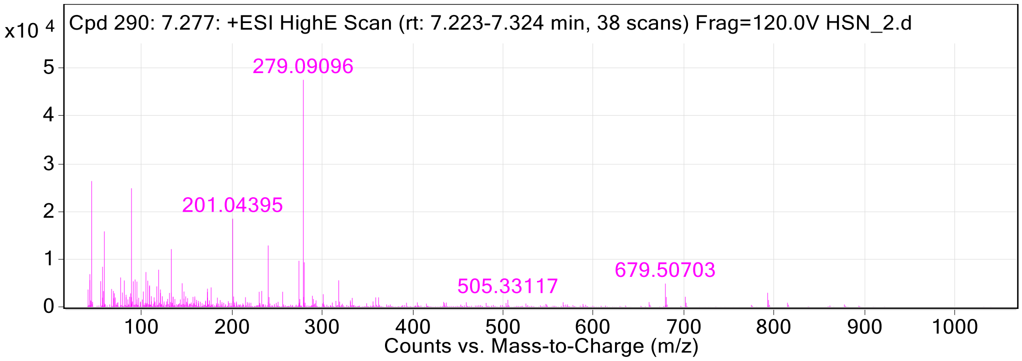

Peak List

m/z	z	Abund
45.03315		26350.8
57.06928		8397.72
59.04854		15807.65
89.05846		24841.48
133.08446		12097.33
201.04395	1	18472.84
240.22955	1	12855.62
274.27128	1	9646.82
279.09096	1	47547.32
280.09407	1	9328.36

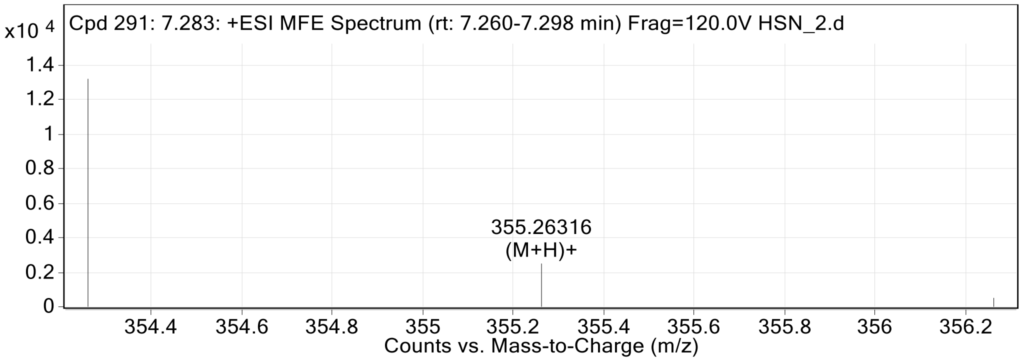

Peak List

m/z	z	Abund	Ion
354.26081	1	13225.34	(M+H)+
355.26316	1	2508.13	(M+H)+
356.26266	1	515.36	(M+H)+

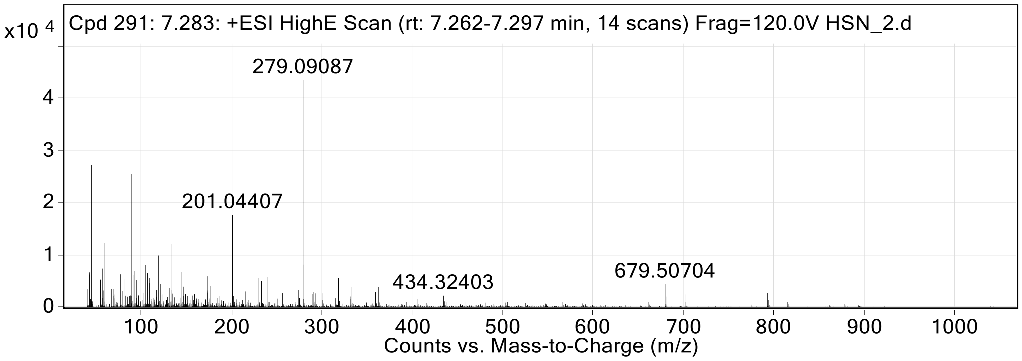

Peak List

m/z	z	Abund
45.03316		27216.09
57.06933		7328.77
59.04852		12204.09
89.05849		25482.79
105.06827		8047.65
119.08365		9835.76
133.08456		12004.03
201.04407	1	17644.89
279.09087	1	43548.66
280.09411	1	8083.54

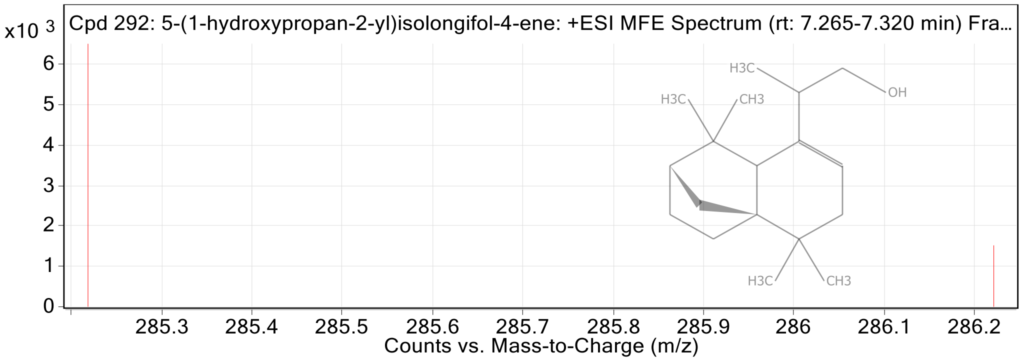

Peak List

m/z	z	Abund	Name	Formula	Ion	Score (DB)	Hits (DB)
285.21853	1	6517.51	5-(1-hydroxypropan-2-yl)isolongifol-4-ene	C18 H30 O	(M+Na)+	82.02	9
286.22132	1	1521.01		C18 H30 O	(M+Na)+		

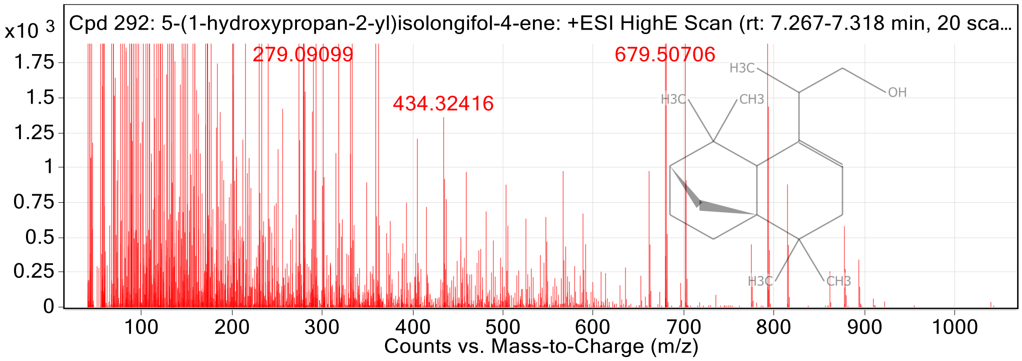

Peak List

m/z	z	Abund
45.03316		27429.99
59.04858		9813.7
77.0377		9375.9
89.05848		26211.98
105.06827		6926.02
119.08364		9737.66
133.08446		12484.62
201.04397	1	32356.2
279.09099	1	82972.92
280.09408	1	16469.4

Qualitative Analysis Report

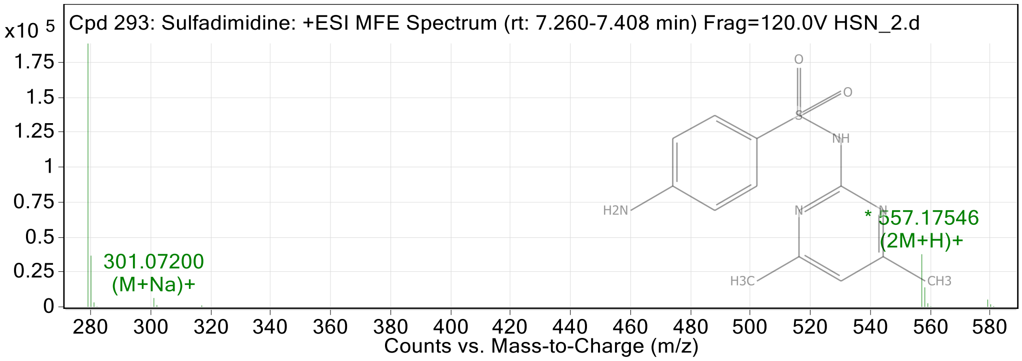

Peak List

m/z	z	Abund	Name	Formula	Ion	Score (DB)	Hits (DB)
279.09078	1	188605.16	Sulfadimidine	C12 H14 N4 O2 S	(M+H)+	87.46	2
280.09436	1	36679.95		C12 H14 N4 O2 S	(M+H)+		
281.09707	1	3229.74		C12 H14 N4 O2 S	(M+H)+		
301.072	1	6351.89			(M+Na)+		
302.07567	1	1296.92			(M+Na)+		
557.17546	1	37692.23			(2M+H)+		
558.17826	1	13986.32			(2M+H)+		
559.1822	1	2599.46			(2M+H)+		
579.15718	1	5239.22			(2M+Na)+		
580.15883	1	1821.75			(2M+Na)+		

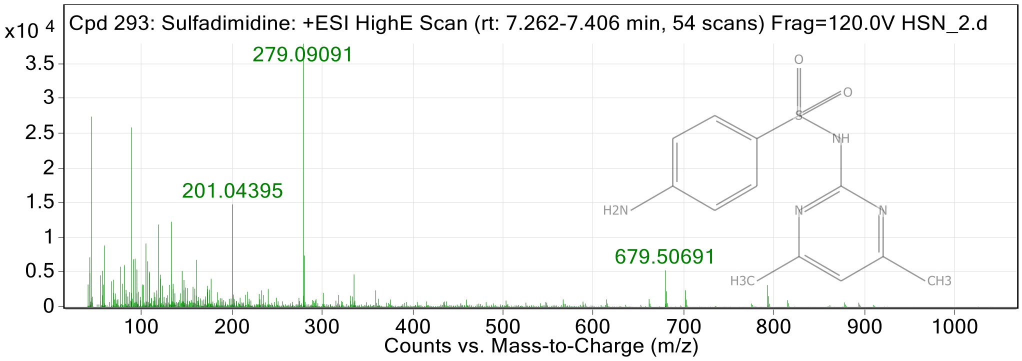

Peak List

m/z	z	Abund
43.01757		7119.24
45.03316		27390.09
59.04858		8817.87
89.05844		25836.28
105.06827		9111.9
119.08357	1	11846.71
133.08469		12241.11
201.04395	1	14746.29
279.09091	1	37910.93
280.09406	1	7388.32

Qualitative Analysis Report

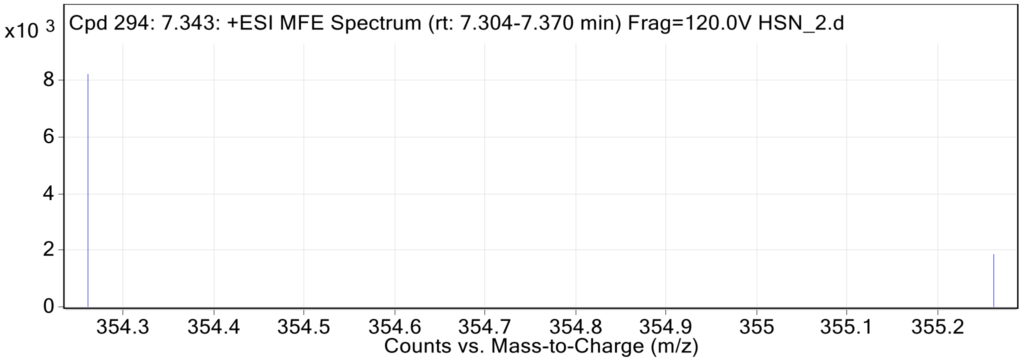

Peak List

m/z	z	Abund	Ion
354.26092	1	8223.86	(M+H)+
355.26258	1	1862.97	(M+H)+

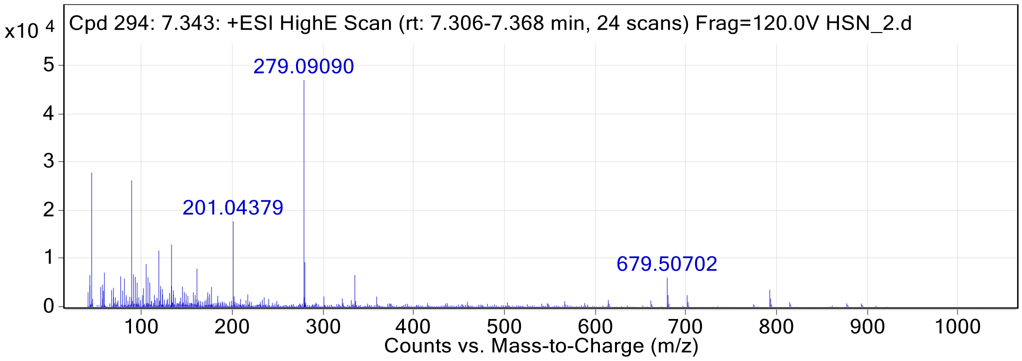

Peak List

m/z	z	Abund
45.03318		27834.61
59.04859		7055.5
89.05841		26233.15
105.06827		8827.92
119.0836	1	11633.36
133.08481		12915.53
161.09375		7890.58
201.04379	1	17728.97
279.0909	1	47053.06
280.094	1	9237.58

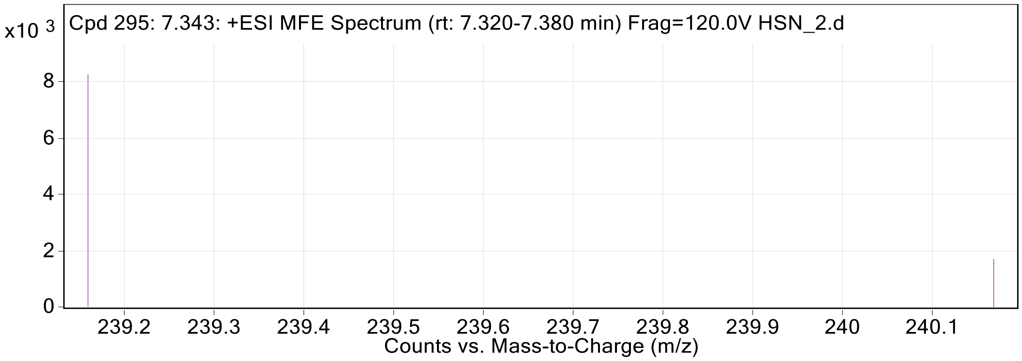

Peak List

m/z	z	Abund	Ion
239.1602	1	8271.22	(M+H)+
240.16907	1	1710.33	(M+H)+

Qualitative Analysis Report

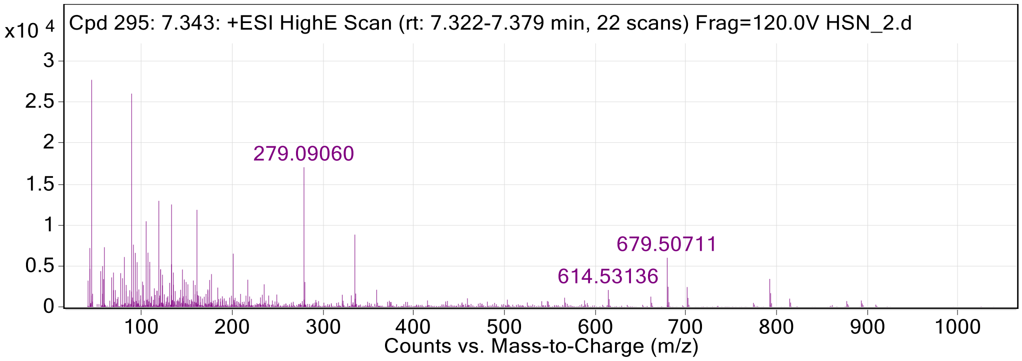

Peak List

m/z	z	Abund
45.03317		27720.59
59.04856		7285.97
89.05839		26038.73
91.05302		7594.74
105.06826		10452.89
119.08357	1	12946.51
133.08491		12502.68
161.0938	1	11849.07
279.0906	1	17047.56
335.17986	1	8821.84

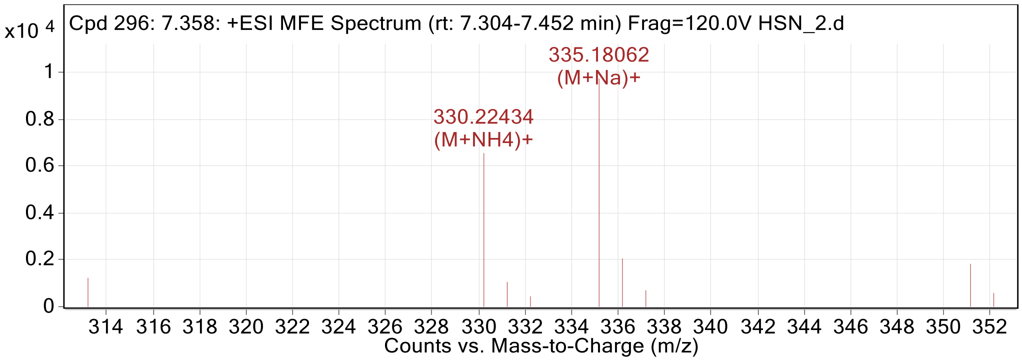

Peak List

m/z	z	Abund	Ion
313.19683	1	1238.31	(M+H)+
330.22434	1	6551.38	(M+NH4)+
331.22832	1	1055.94	(M+NH4)+
332.22809	1	447.27	(M+NH4)+
335.18062	1	9733.51	(M+Na)+
336.18425	1	2061.17	(M+Na)+
337.19177	1	706.24	(M+Na)+
351.15475	1	1830.02	(M+K)+
352.1554	1	591.46	(M+K)+

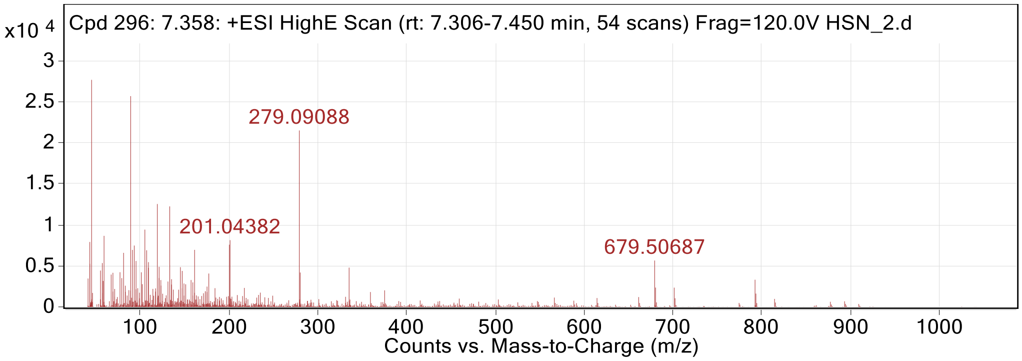

Peak List

m/z	z	Abund
43.01755		7915.97
45.03316		27691.04
59.04856		8663.23
89.05842		25705.41
105.06826		9423.67
119.08359	1	12548.06
133.08468		12256.29
200.23474		7582.51
201.04382		8138.12
279.09088	1	21517.85

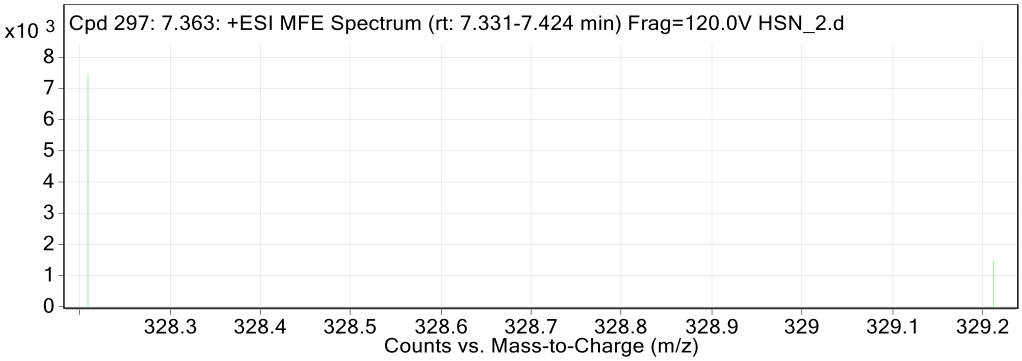

Peak List

m/z	z	Abund	Ion
328.20898	1	7423.64	(M+H)+
329.21244	1	1457.46	(M+H)+

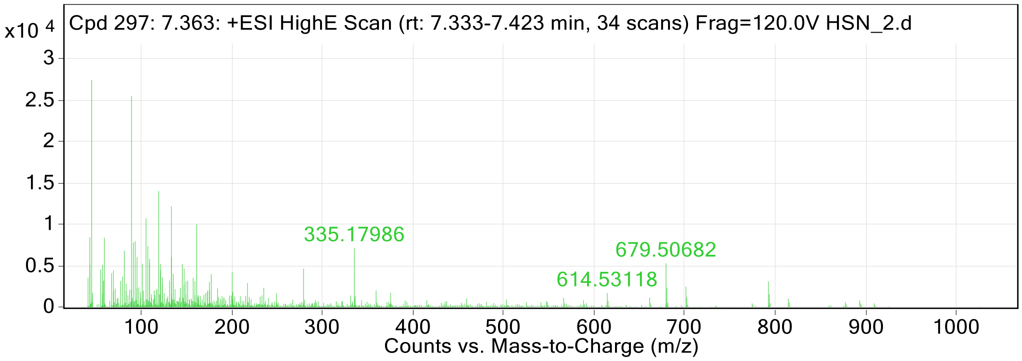

Peak List

m/z	z	Abund
43.01753		8427.96
45.03315		27473.88
59.04858		8380.2

89.0584		25536.9
91.05295		7782.06
93.0685		7961.38
105.06829		10714.77
119.08357	1	14026.63
133.08474		12162.88
161.09377		10008.24

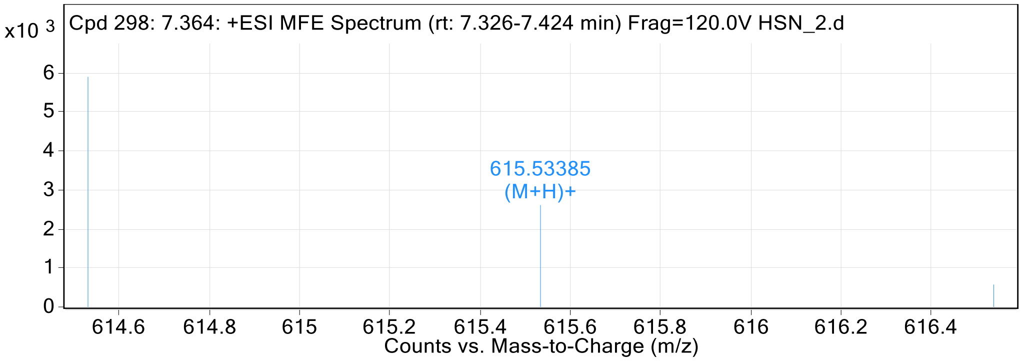

Peak List

m/z	z	Abund	Ion
614.53152	1	5894.39	(M+H)+
615.53385	1	2609.26	(M+H)+
616.53789	1	573.61	(M+H)+

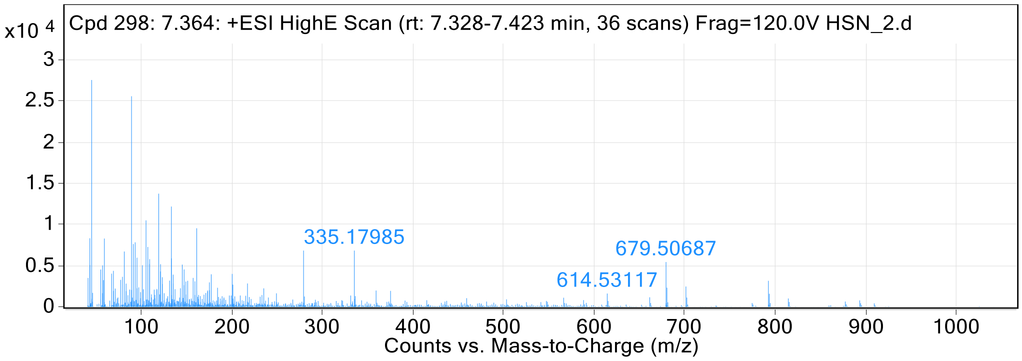

Peak List

m/z	z	Abund
43.01753		8326.98
45.03315		27578.54
59.04859		8291.96
89.05841		25601.4
91.05295		7621.91
93.06849		7832.97
105.06829		10503.02
119.08357	1	13750.71
133.08474		12185.05
161.09377		9540.83

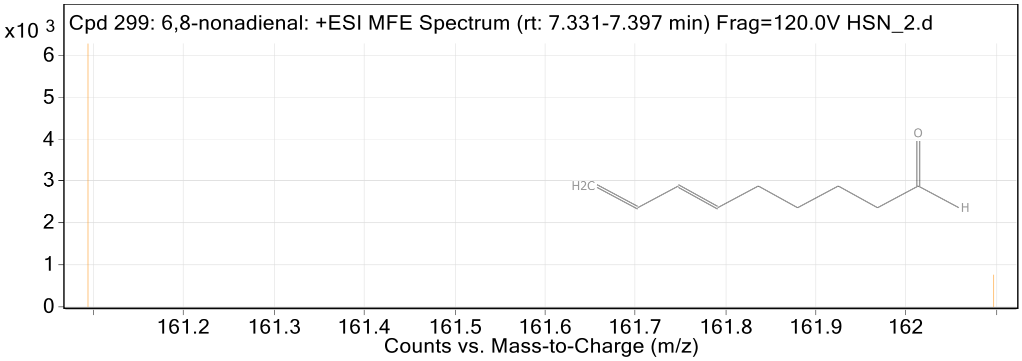

Cpd 299: 6,8-nonadienal: +ESI MFE Spectrum (rt: 7.331-7.397 min) Frag=120.0V HSN_2.d
x10 3
6
5
4
3
2
1
0
H2C
O
H
161.2
161.3
161.4
161.5
161.6
161.7
161.8
161.9
162
Counts vs. Mass-to-Charge (m/z)

Qualitative Analysis Report

Peak List

m/z	z	Abund	Name	Formula	Ion	Score (DB)	Hits (DB)
161.09381	1	6287.04	6,8-nonadienal	C9 H14 O	(M+Na)+	85.95	10
162.09698	1	768.38		C9 H14 O	(M+Na)+		

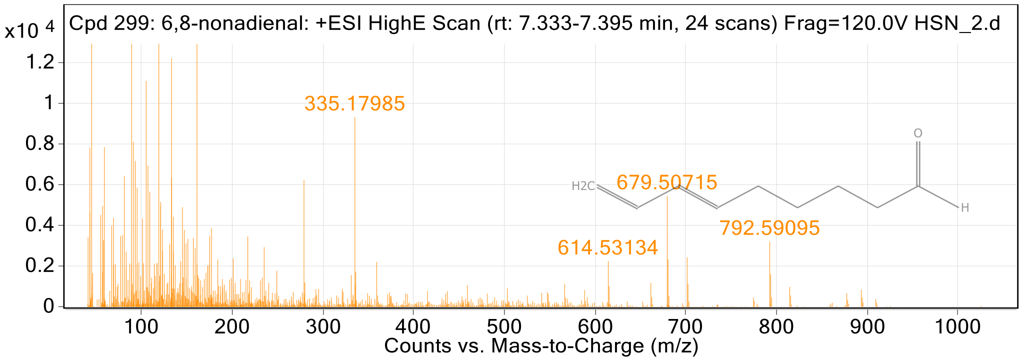

Peak List

m/z	z	Abund
43.01758		7808.15
45.03316		27352.57
59.04862		7841.49
89.05839		25686.68
91.05302		8102.02
105.06828		11092.04
119.08354	1	14015.12
133.08479		12210.58
161.09377	1	12920.4
335.17985	1	9309.1

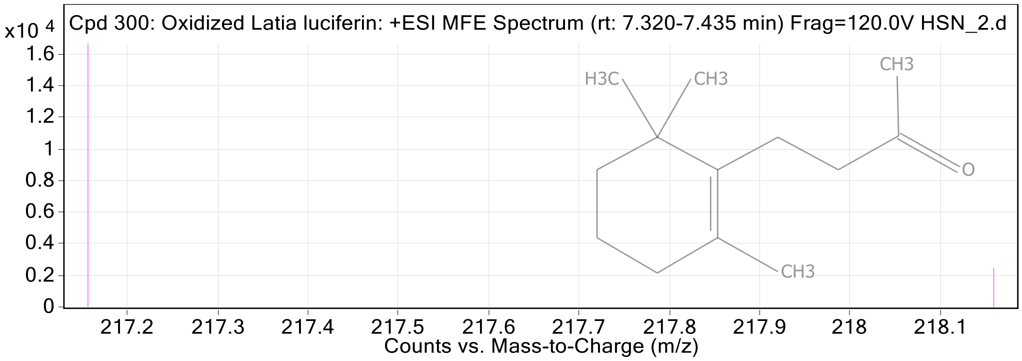

Peak List

m/z	z	Abund	Name	Formula	Ion	Score (DB)	Hits (DB)
217.15607	1	16673.74	Oxidized Latia luciferin	C13 H22 O	(M+Na)+	87.21	10
218.1595	1	2483.13		C13 H22 O	(M+Na)+		

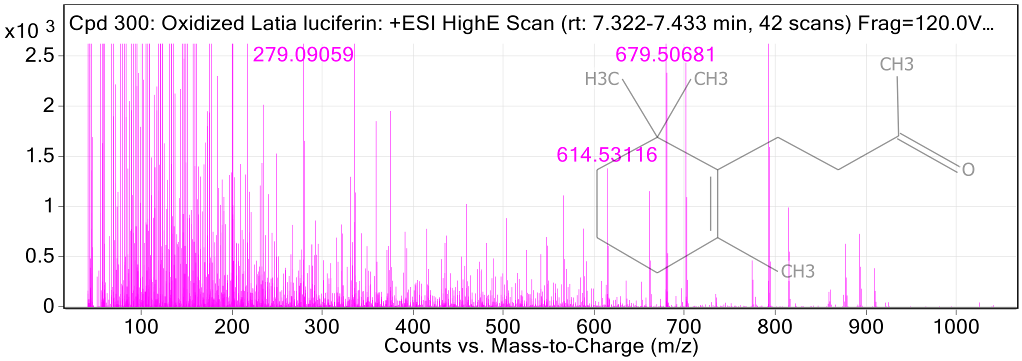

Peak List

m/z	z	Abund
43.01754		8210.28
45.03315		27591.62
59.04856		8419.47
89.05841		25581.77
93.06848		7657.53
105.06826		10043.36
119.08358	1	13187.85
133.08471		12139.57
161.09375		8428.62
279.09059	1	9403.52

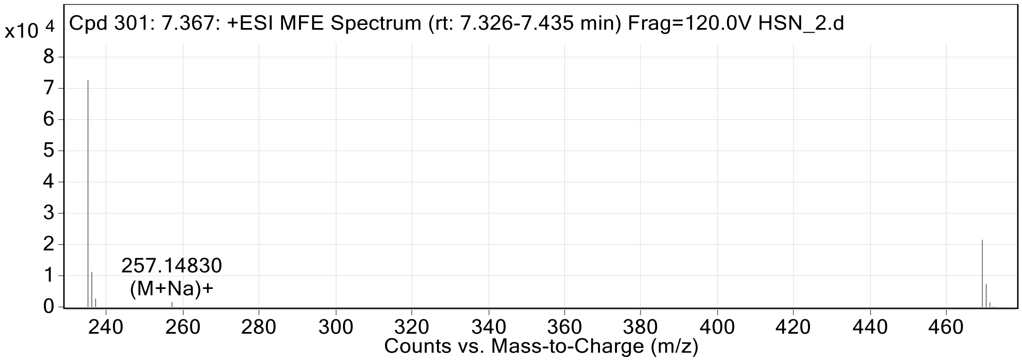

Peak List

m/z	z	Abund	Ion
235.16643	1	72827.4	(M+H)+
236.16998	1	11189.34	(M+H)+
237.17649	1	2699.55	(M+H)+
257.1483	1	1599.3	(M+Na)+
469.32747	1	21562.66	(2M+H)+
470.33077	1	7441.76	(2M+H)+
471.33292	1	1448.78	(2M+H)+
472.32373	1	185.85	(2M+H)+

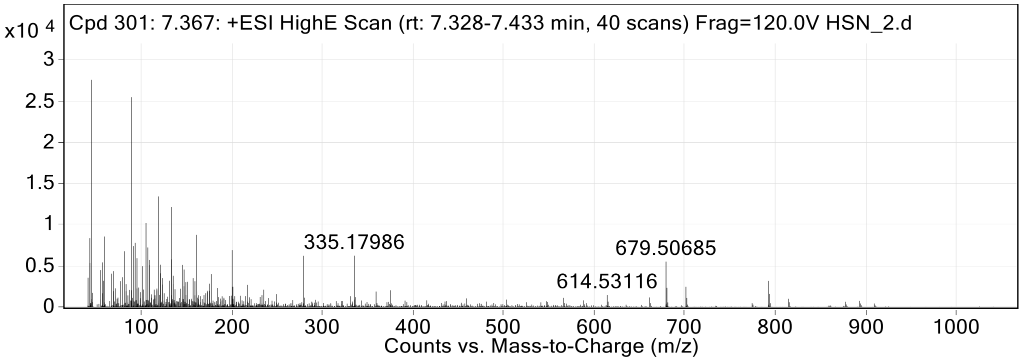

Peak List

m/z	z	Abund
43.01755		8360.11
45.03315		27628.27
59.04856		8558.39
89.05842		25512.08
91.05294		7404.02
93.06849		7802.99
105.06827		10225.78
119.08359	1	13435.79
133.08468		12171.19
161.09376		8777.5

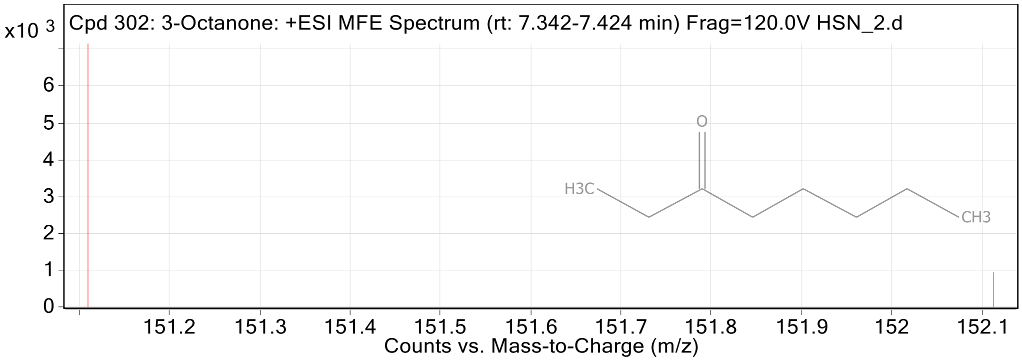

Peak List

m/z	z	Abund	Name	Formula	Ion	Score (DB)	Hits (DB)
151.10973	1	7160.76	3-Octanone	C8 H16 O	(M+Na)+	81.4	10
152.11294	1	940.53		C8 H16 O	(M+Na)+		

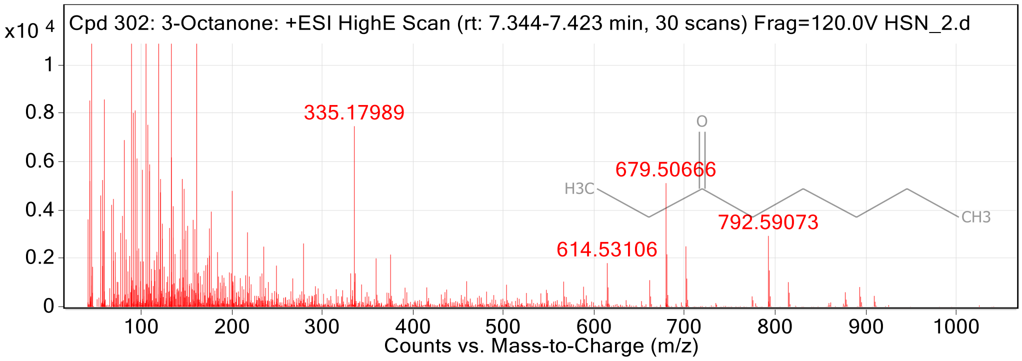

Peak List

m/z	z	Abund
43.01753		8524.87
45.03314		27398.04
59.04857		8568.17
89.05841		25402.38
91.05295		8011.33
93.06852		8114.92
105.06828		11137.87
119.08354	1	14253
133.08478		11960.01
161.09375	1	10876.24

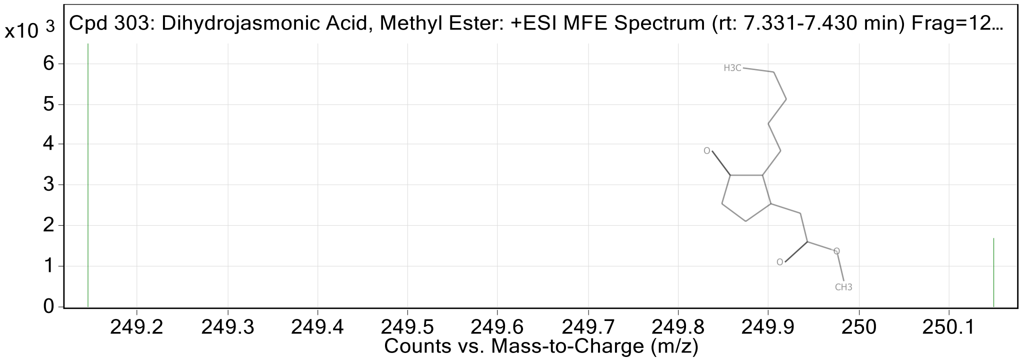

Peak List

m/z	z	Abund	Name	Formula	Ion	Score (DB)	Hits (DB)
249.14554	1	6493.14	Dihydrojasmonic Acid, Methyl Ester	C13 H22 O3	(M+Na)+	66.29	3
250.14935	1	1697.83		C13 H22 O3	(M+Na)+		

Qualitative Analysis Report

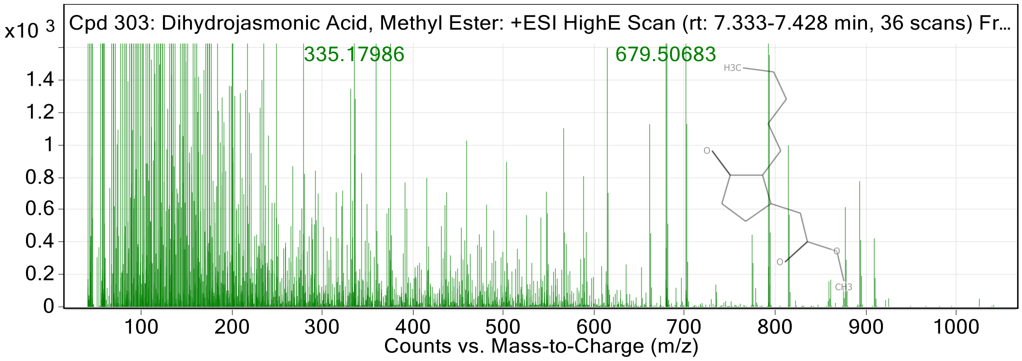

Peak List

m/z	z	Abund
43.01754		8444.79
45.03314		27561.86
59.04857		8492.31
89.05842		25510.83
91.05295		7665.83
93.06849		7962.81
105.06827		10567.12
119.08359	1	13845.06
133.08473		12111.45
161.09376		9585.06

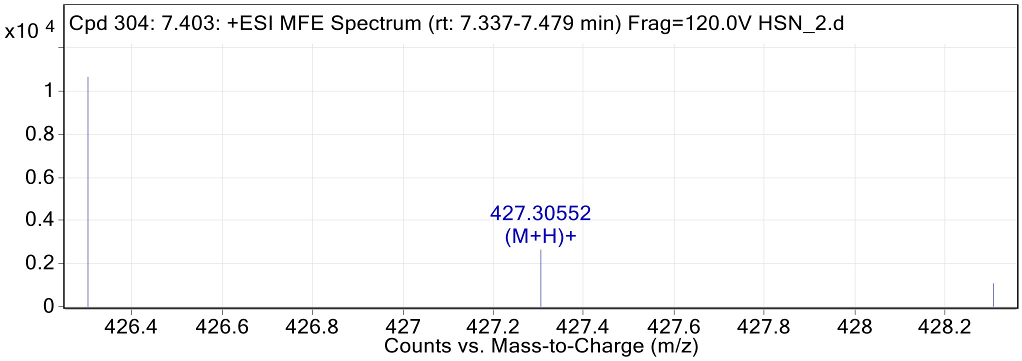

Peak List

m/z	z	Abund	Ion
426.3027	1	10673.4	(M+H)+
427.30552	1	2651.83	(M+H)+
428.30815	1	1088.94	(M+H)+

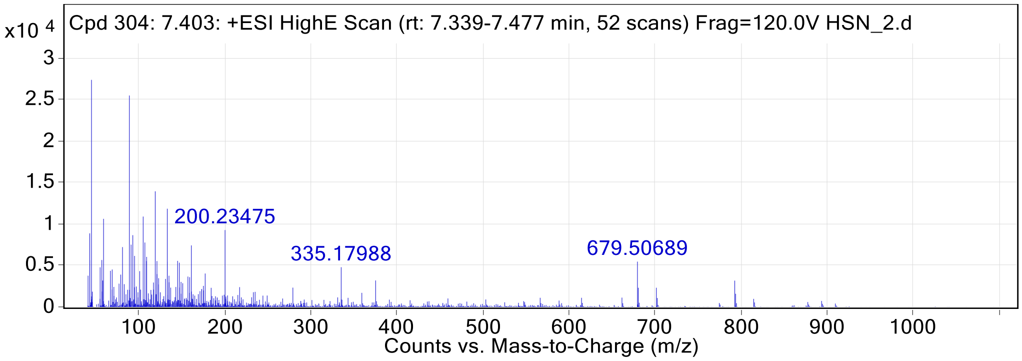

Peak List

m/z	z	Abund
43.01753		8852.31
45.03315		27332.9

59.04855		10603.68
89.05843		25451.57
93.06854		8637.06
105.06825		10876.96
107.08378		7753.58
119.08363	1	13914.88
133.08479		11822.22
200.23475		9251.68

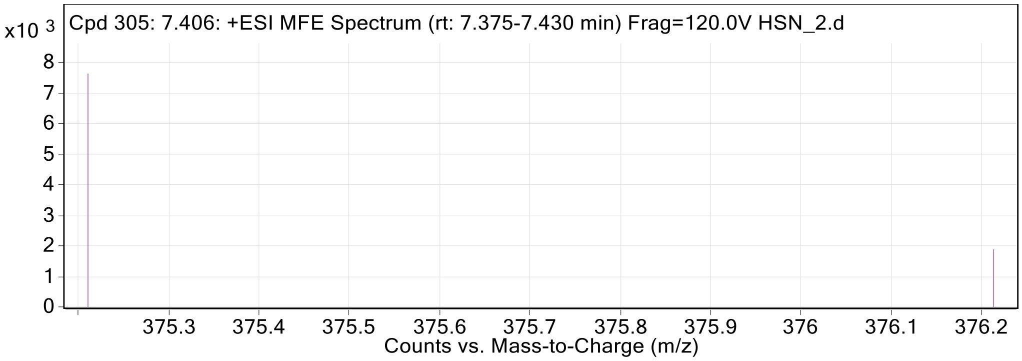

Peak List

m/z	z	Abund	Ion
375.21075	1	7656.18	(M+H)+
376.21339	1	1888.88	(M+H)+

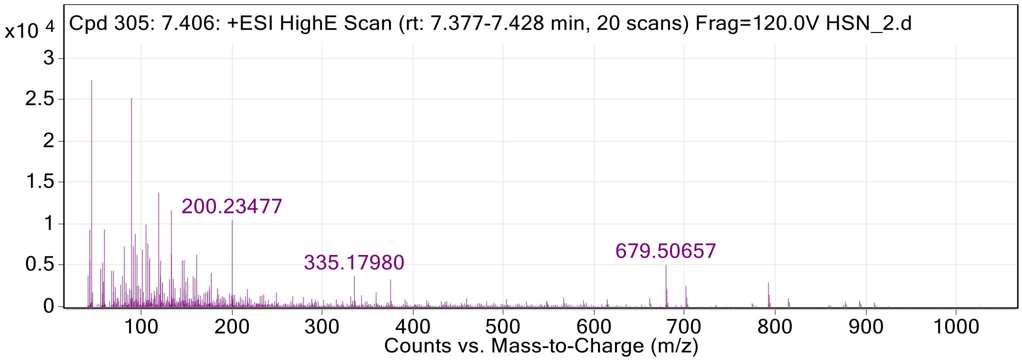

Peak List

m/z	z	Abund
43.01751		9273.05
45.03312		27373.66
59.04858		9339.69
89.05845		25220.54
93.06857		8788.22
105.06827		9934.63
107.08381		7669.56
119.08357	1	13809.68
133.08456		11665.51
200.23477	1	10474.11

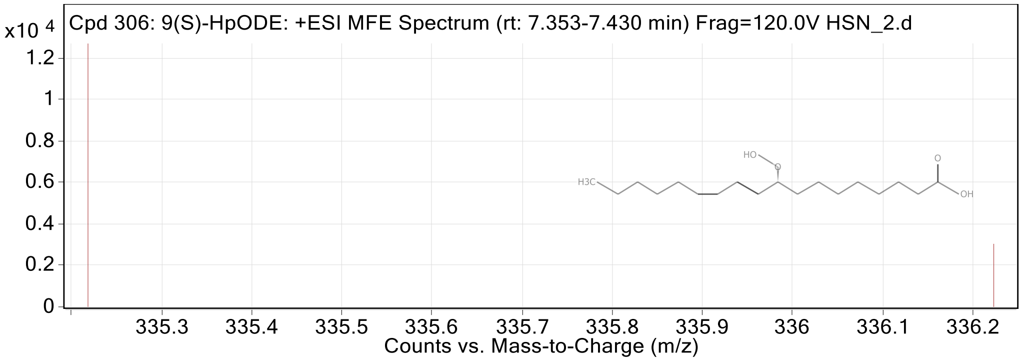

Peak List

m/z	z	Abund	Name	Formula	Ion	Score (DB)	Hits (DB)
335.21839	1	12706.8	9(S)-HpODE	C18 H32 O4	(M+Na)+	78.18	10
336.22319	1	3041.28		C18 H32 O4	(M+Na)+		

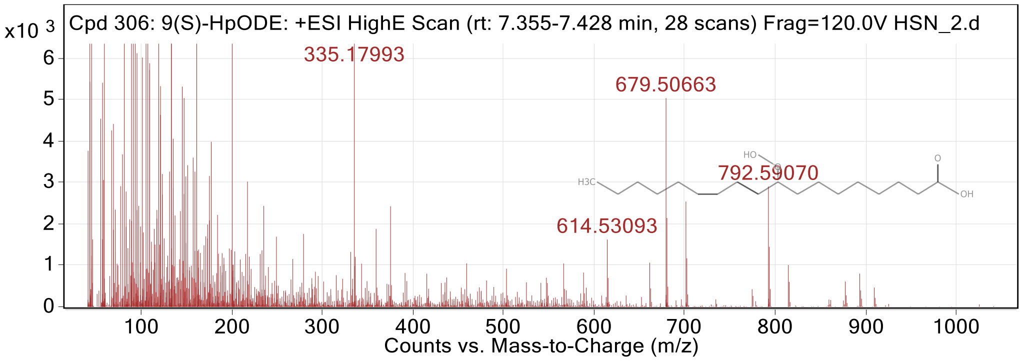

Peak List

m/z	z	Abund
43.01754		8699.41
45.03313		27466.2
59.04857		8930.44
89.05843		25280.49
91.05293		7927.26
93.06853		8237.69
105.06826		10909.22
119.08359	1	13942.24
133.08476		11535.63
161.09378	1	10443.98

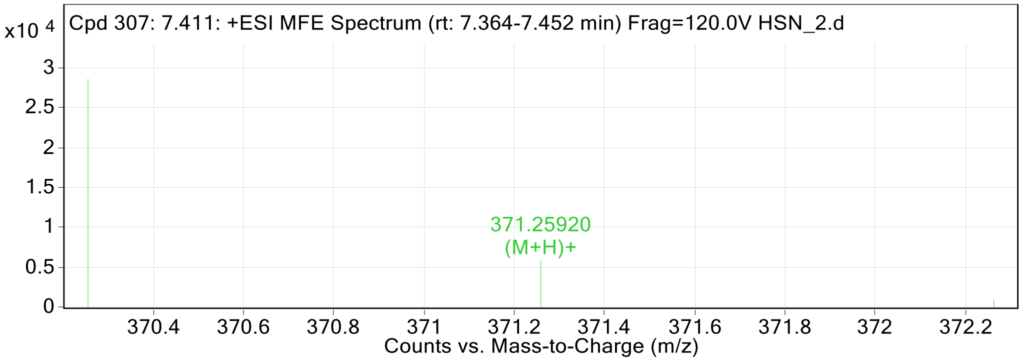

Peak List

m/z	z	Abund	Ion
370.25553	1	28587.69	(M+H)+
371.2592	1	5773.33	(M+H)+
372.26318	1	948.83	(M+H)+

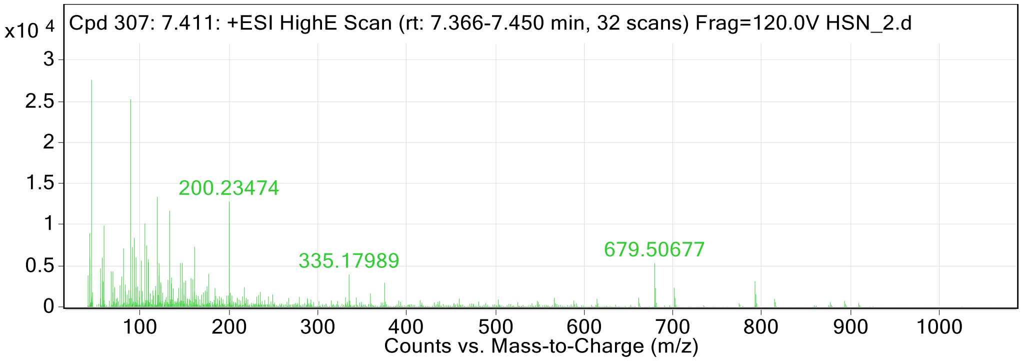

Peak List

m/z	z	Abund
43.01755		8928.28
45.03315		27598.8
59.04854		9898.91
89.05842		25238.84
93.06857		8393.98
105.06827		10139.95
107.08379		7477.42
119.08357	1	13347.87
133.08468		11678.34
200.23474	1	12795.49

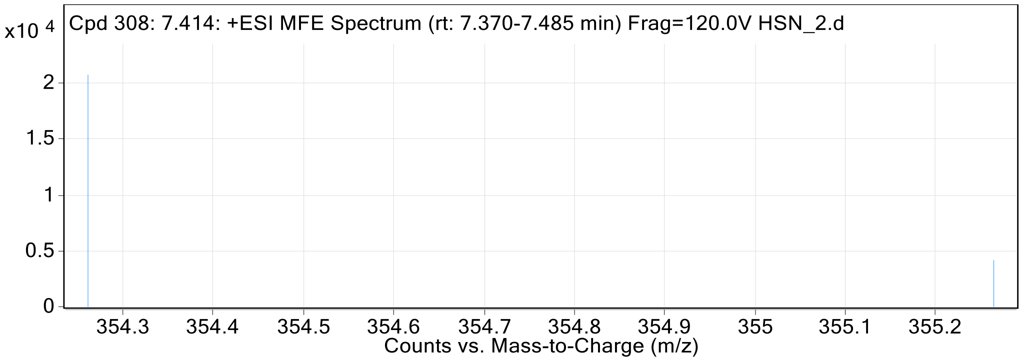

Peak List

m/z	z	Abund	Ion
354.2613	1	20758.76	(M+H)+
355.2644	1	4165.53	(M+H)+

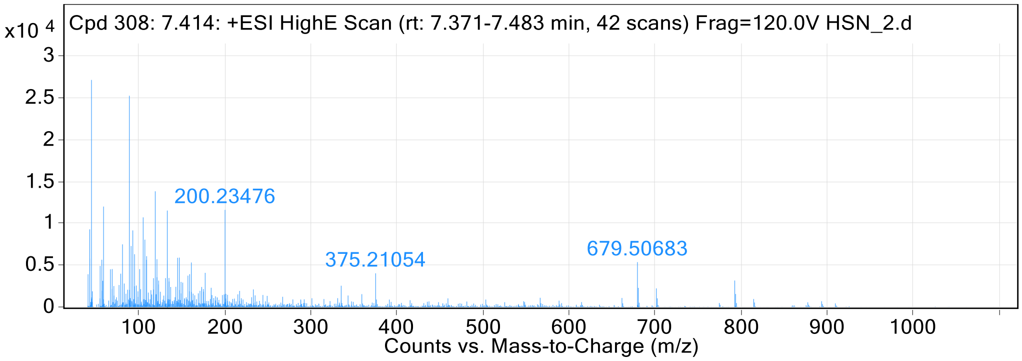

Peak List

m/z	z	Abund
43.01752		9279.99
45.03314		27149.57
59.04855		12003.74

89.05846		25259.45
93.06857		9153.29
105.06826		10713.93
107.08375		8051.76
119.08363	1	13830.31
133.08471		11526.69
200.23476	1	11610.01

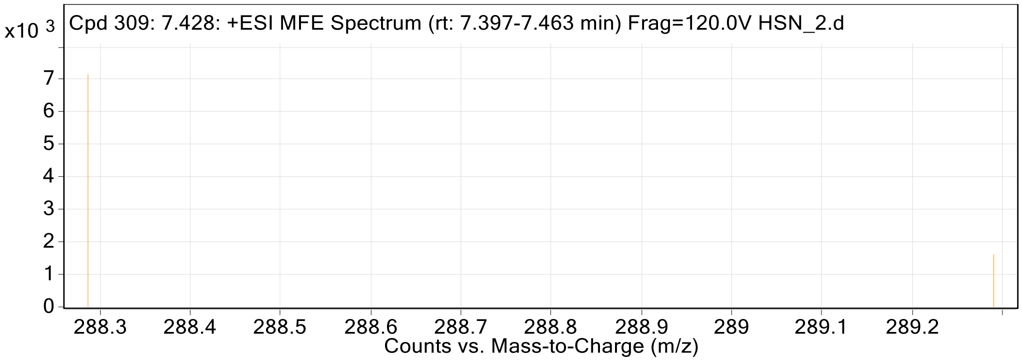

Peak List

m/z	z	Abund	Ion
288.28688	1	7164.49	(M+H)+
289.2905	1	1614.03	(M+H)+

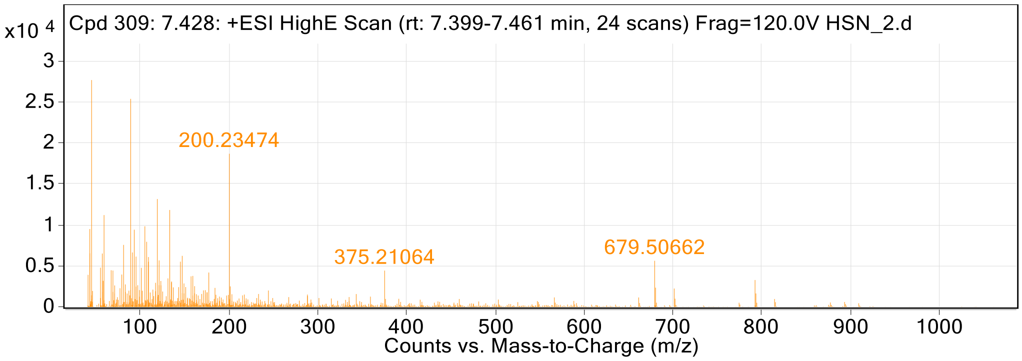

Peak List

m/z	z	Abund
43.01749		9484.61
45.03314		27660.97
59.0485		11187.38
89.05844		25363.57
93.06858		9402.48
105.06822		9820.77
107.08374		7928.56
119.08366	1	13143.56
133.0846		11807.47
200.23474	1	18671.17

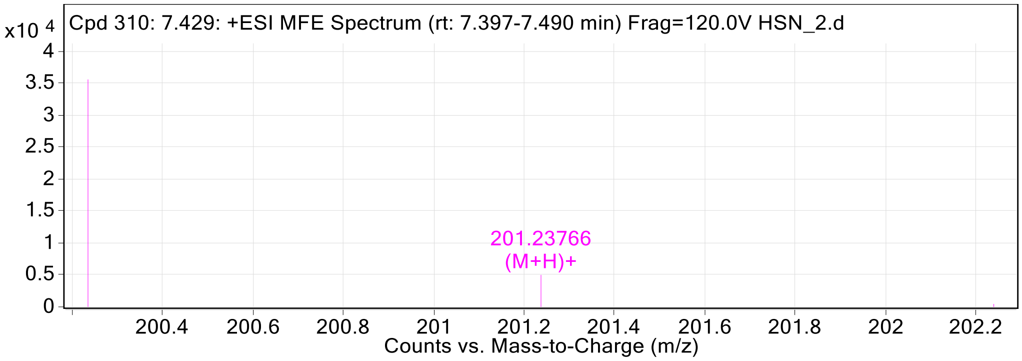

Peak List

m/z	z	Abund	Ion
200.23484	1	35550.08	(M+H)+
201.23766	1	4994.68	(M+H)+
202.24	1	457.08	(M+H)+

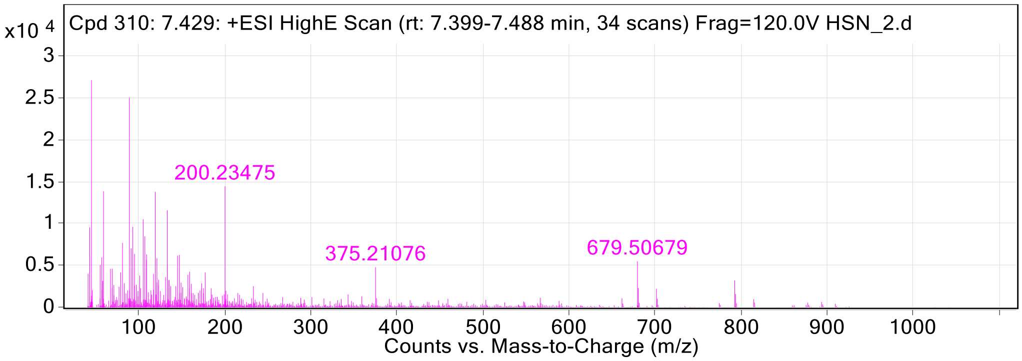

Peak List

m/z	z	Abund
43.0175		9548.02
45.03315		27180.8
59.04852		13867.59
89.05847		25108.67
93.06857		9608.3
105.06825		10500.53
107.08372		8479.15
119.08369	1	13800.64
133.08471		11590.7
200.23475	1	14462.17

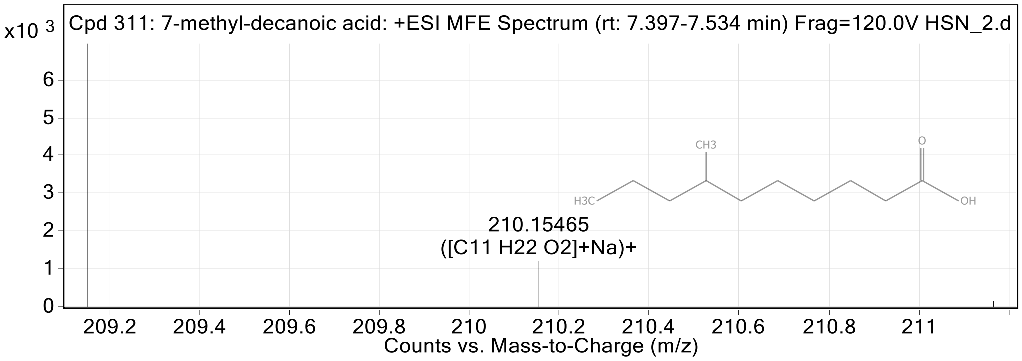

Peak List

m/z	z	Abund	Name	Formula	Ion	Score (DB)	Hits (DB)
209.15119	1	6957.66	7-methyl-decanoic acid	C11 H22 O2	(M+Na)+	90.52	10
210.15465	1	1210.8		C11 H22 O2	(M+Na)+		

211.16592	1	150.42		C11 H22 O2	(M+Na)+		

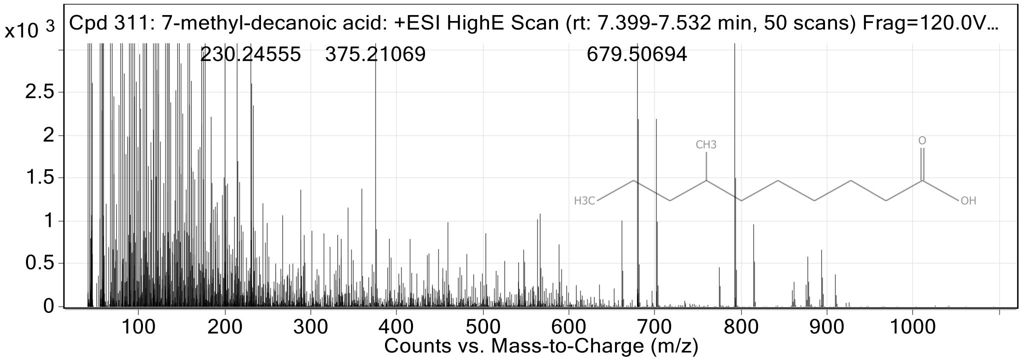

Peak List

m/z	z	Abund
43.01751		8919.71
45.03315		26549.76
58.0646		12190.11
59.04854		18094.43
89.05847		24496.72
105.06826		9822.64
119.08365	1	13597.11
133.08469		11676.55
200.23477	1	10395.04
230.24555	1	16462.24

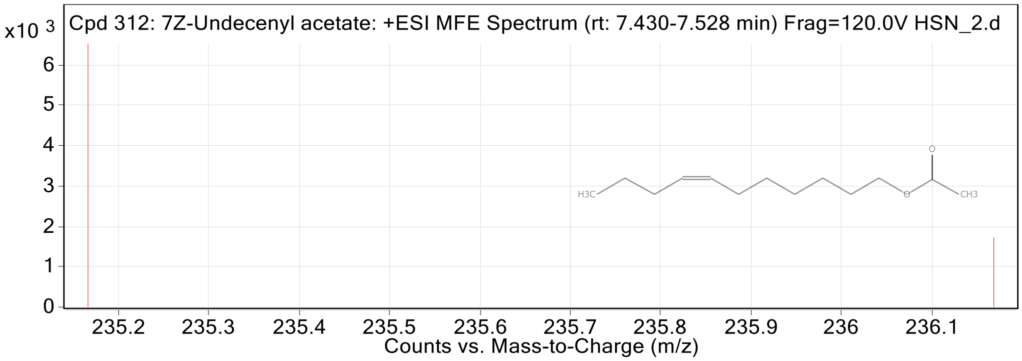

Peak List

m/z	z	Abund	Name	Formula	Ion	Score (DB)	Hits (DB)
235.16619	1	6529.97	7Z-Undecenyl acetate	C13 H24 O2	(M+Na)+	61.73	10
236.1685	1	1729.21		C13 H24 O2	(M+Na)+		

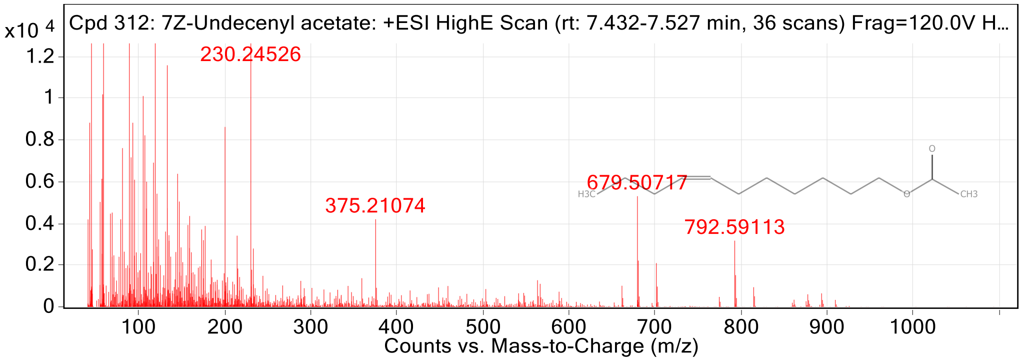

Peak List

m/z	z	Abund
43.01752		8831.85
45.03316		26276.86

58.06458		10189.96
59.04854		20520.44
89.05847		24370.79
93.06854		8827.85
105.06828		10110.77
119.08364	1	13905.3
133.08473		11584.47
230.24526	1	12635.28

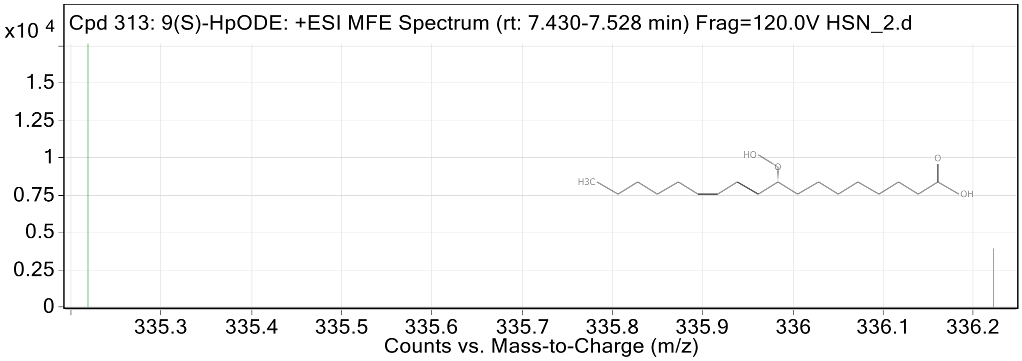

Peak List

m/z	z	Abund	Name	Formula	Ion	Score (DB)	Hits (DB)
335.2188	1	17665.5	9(S)-HpODE	C18 H32 O4	(M+Na)+	83.37	10
336.22251	1	3913.19		C18 H32 O4	(M+Na)+		

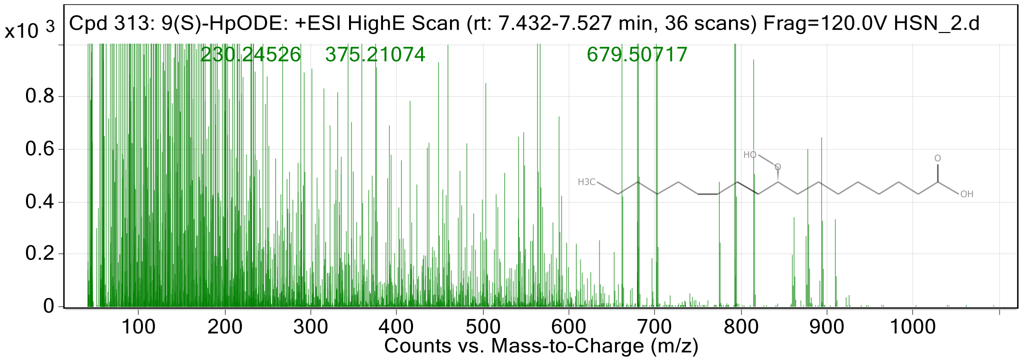

Peak List

m/z	z	Abund
43.01752		8831.85
45.03316		26276.86
58.06458		10189.96
59.04854		20520.44
89.05847		24370.79
93.06854		8827.85
105.06828		10110.77
119.08364	1	13905.3
133.08473		11584.47
230.24526	1	12635.28

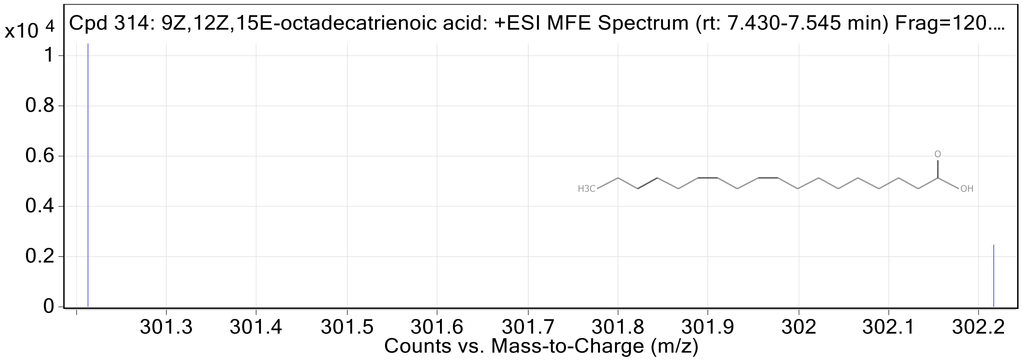

Peak List

m/z	z	Abund	Name	Formula	Ion	Score (DB)	Hits (DB)
301.21301	1	10469.46	9Z,12Z,15E-octadecatrienoic acid	C18 H30 O2	(M+Na)+	80.35	10
302.21634	1	2472.42		C18 H30 O2	(M+Na)+		

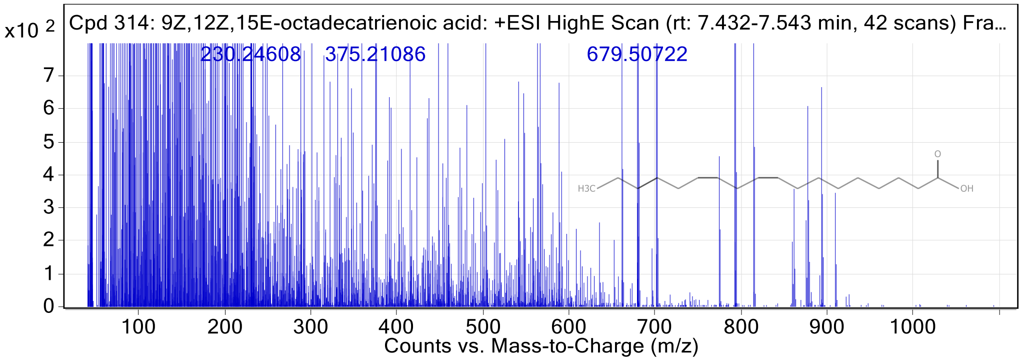

Peak List

m/z	z	Abund
43.01751		8413.69
45.03317		25595.82
58.06466	1	25972.52
59.04854		20649.75
89.05847		23992.03
93.06854		8232.19
105.06826		9517.86

m/z	z	Abund
119.08365	1	13174.72
133.0847		11534.3
230.24608	1	39271.74

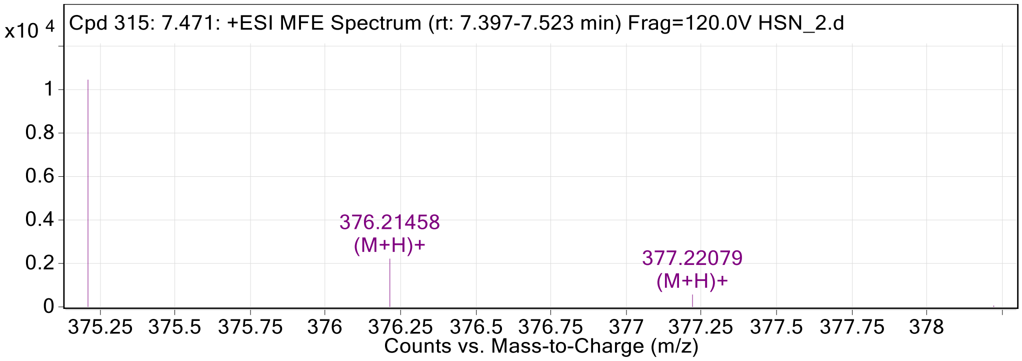

Peak List

m/z	z	Abund	Ion
375.21092	1	10458.53	(M+H)+
376.21458	1	2220.39	(M+H)+
377.22079	1	569.95	(M+H)+
378.22241	1	61.21	(M+H)+

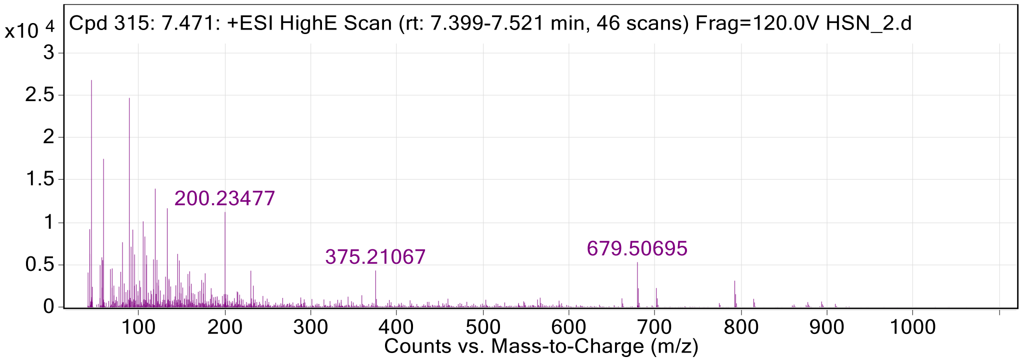

Peak List

m/z	z	Abund
43.01752		9191.83
45.03315		26802.14
59.04853		17494.71
89.05848		24690.43
93.06856		9135.5
105.06827		10114.04
107.08374		8324.03
119.08366	1	13975.4
133.08472		11659.58
200.23477	1	11222.79

Qualitative Analysis Report

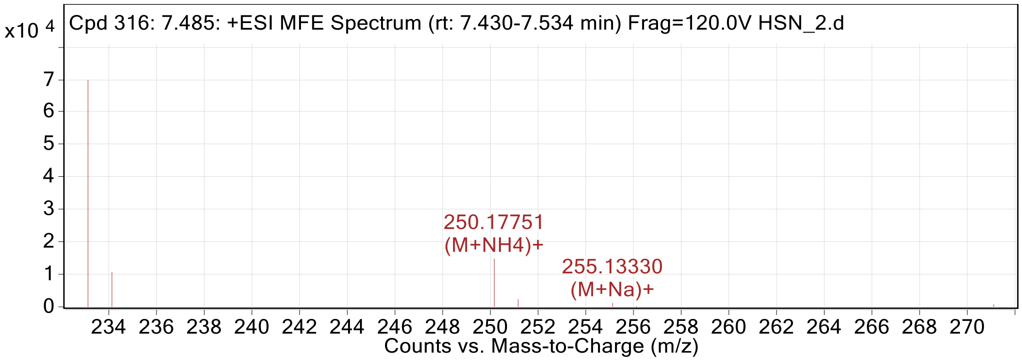

Peak List

m/z	z	Abund	Ion
233.1511	1	69917.12	(M+H)+
234.15461	1	10744.95	(M+H)+
250.17751	1	14866.5	(M+NH4)+
251.17588	1	2340.54	(M+NH4)+
255.1333	1	1203.51	(M+Na)+
256.13436	1	273.88	(M+Na)+
271.10871	1	746.88	(M+K)+

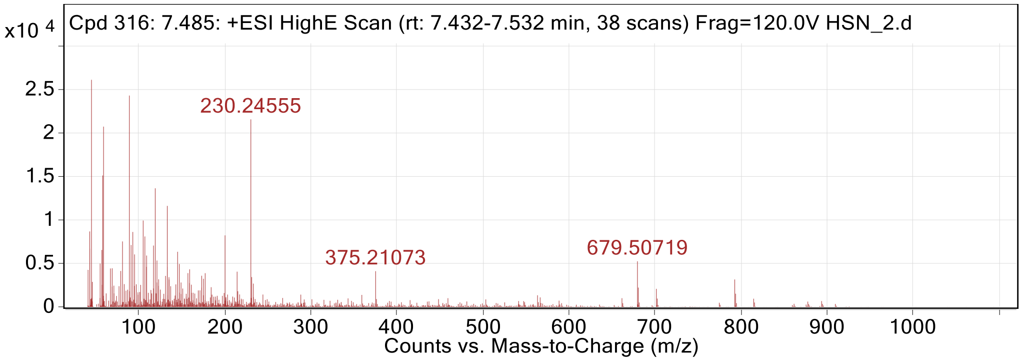

Peak List

m/z	z	Abund
43.01752		8667.59
45.03316		26097.96
58.06459		15100.37
59.04854		20715.63
89.05847		24287.55
93.06855		8605.95
105.06827		9919.07
119.08364	1	13626.22
133.08472		11601.82
230.24555	1	21552.81

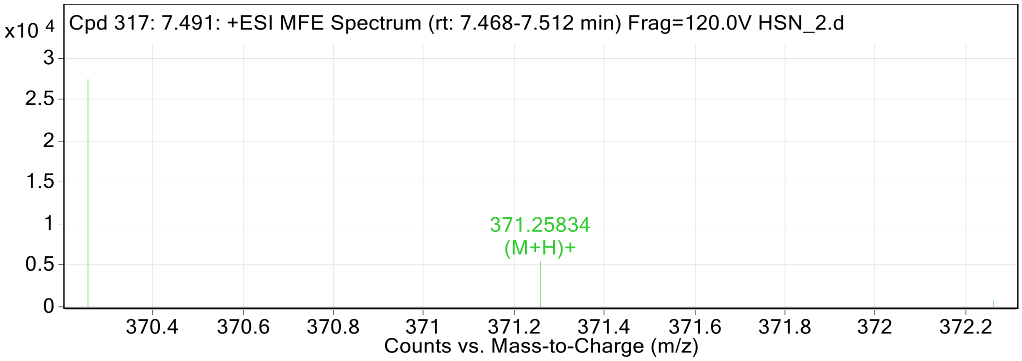

Peak List

m/z	z	Abund	Ion
370.25643	1	27471.41	(M+H)+
371.25834	1	5496.95	(M+H)+
372.26243	1	779.96	(M+H)+

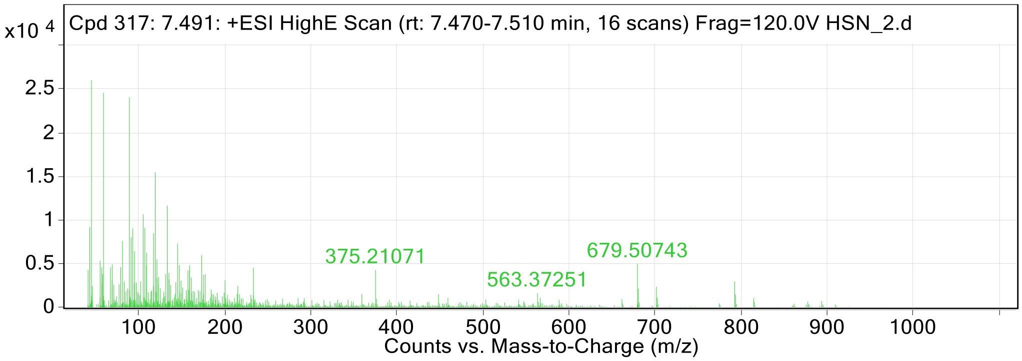

Peak List

m/z	z	Abund
43.01754		9171.42
45.03318		26031.43
59.04855		24574.24
89.05852		24053.3
93.06853		9045.22
105.06833		10633.62
107.08377		9100.48
117.08906		8466.98
119.08363	1	15466.08
133.08487		11629.93

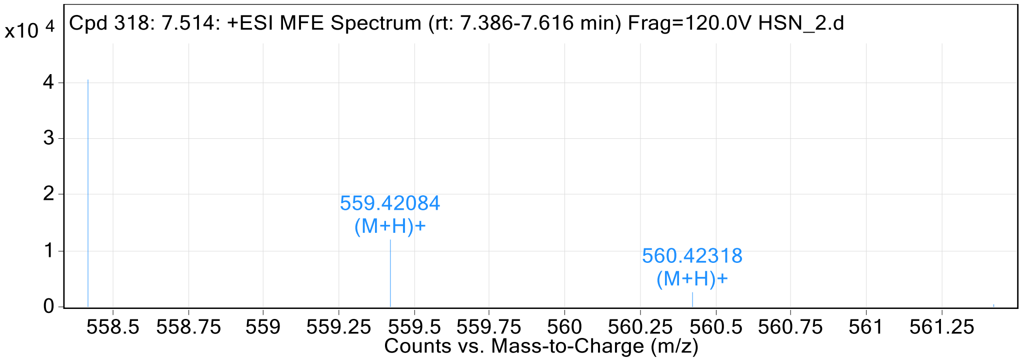

Peak List

m/z	z	Abund	Ion
558.41753	1	40575.3	(M+H)+
559.42084	1	12012.61	(M+H)+

560.42318	1	2620.22	(M+H)+
561.42261	1	463.36	(M+H)+

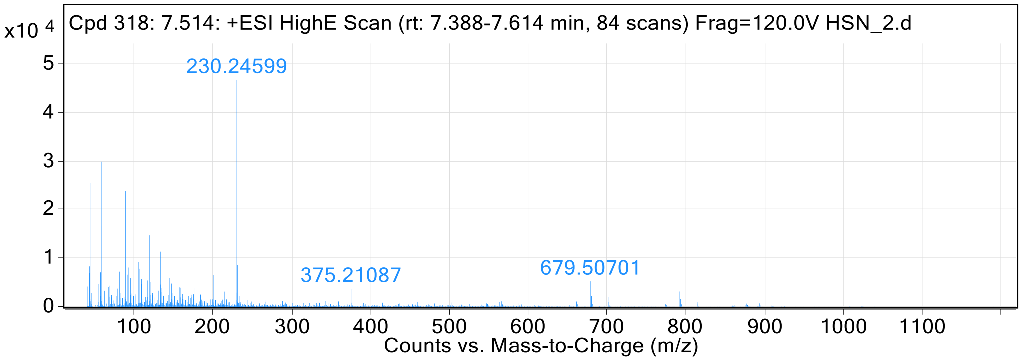

Peak List

m/z	z	Abund
43.01752		8272.94
45.03316		25455.44
58.06466	1	29841.63
59.04855		16617.14
89.05849		23829.43
105.06826		9132.2
119.08363	1	14670.78
133.0847		11309.51
230.24599	1	46706.44
231.24862	1	8575.73

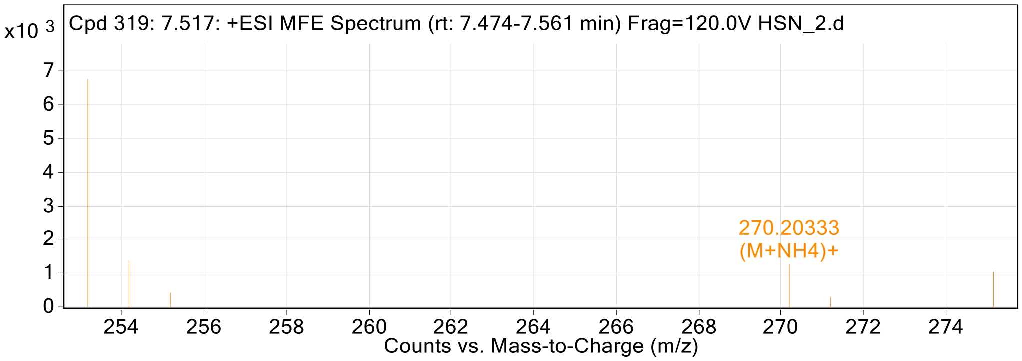

Peak List

m/z	z	Abund	Ion
253.17727	1	6773.84	(M+H)+
254.17764	1	1349.61	(M+H)+
255.18249	1	414.21	(M+H)+
270.20333	1	1266.98	(M+NH4)+
271.20548	1	280.98	(M+NH4)+
275.16019	1	1045.11	(M+Na)+

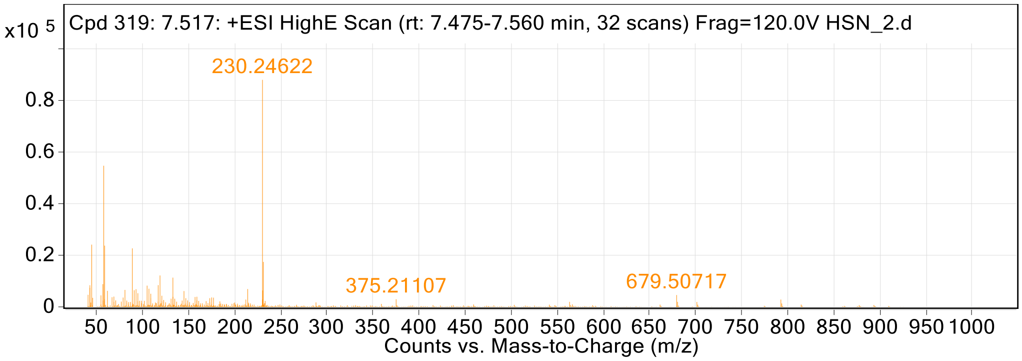

Peak List

m/z	z	Abund
45.03316		24177.2
57.06927		8843.55
58.06469	1	54836.64
59.04855		23790.4
89.05849		22729.94
117.08911		8447.27
119.08363	1	12204.87
133.08464		11360.97
230.24622	1	88192.73
231.24865	1	17459.41

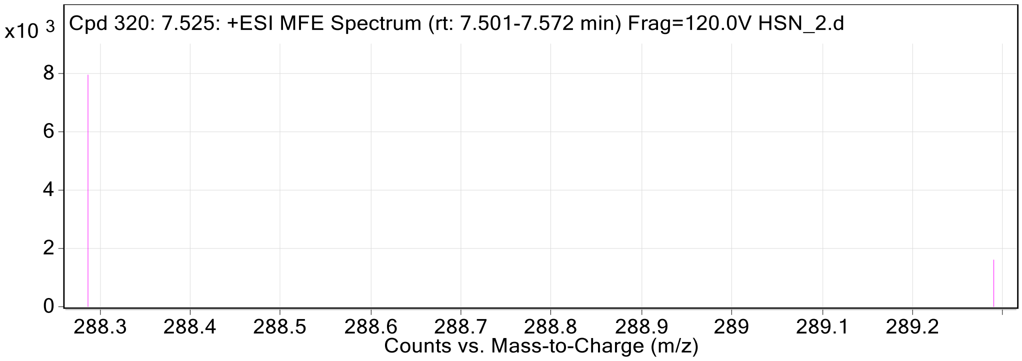

Peak List

m/z	z	Abund	Ion
288.28677	1	7942.96	(M+H)+
289.29009	1	1610.63	(M+H)+

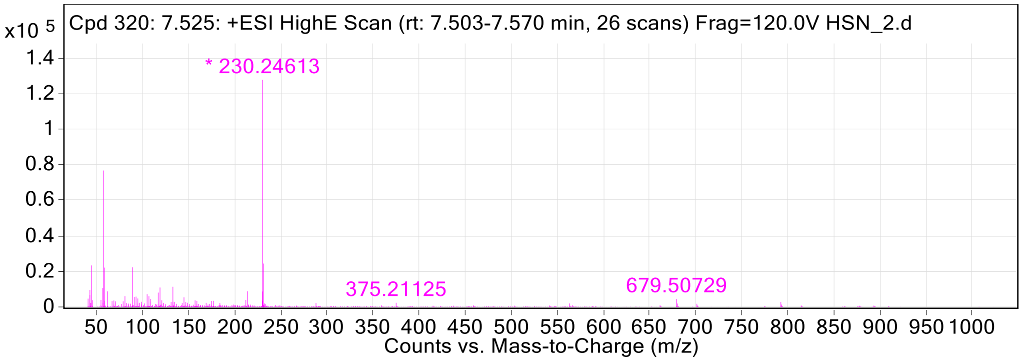

Peak List

m/z	z	Abund
43.05392		9584.99
45.03316		23350.24
57.06929		10640.1

58.06467	1	76761.13
59.04856		22149.82
89.05849		22274.17
119.08366	1	10933.96
133.08459		11362.47
230.24613	1	127721.66
231.24866	1	24411.15

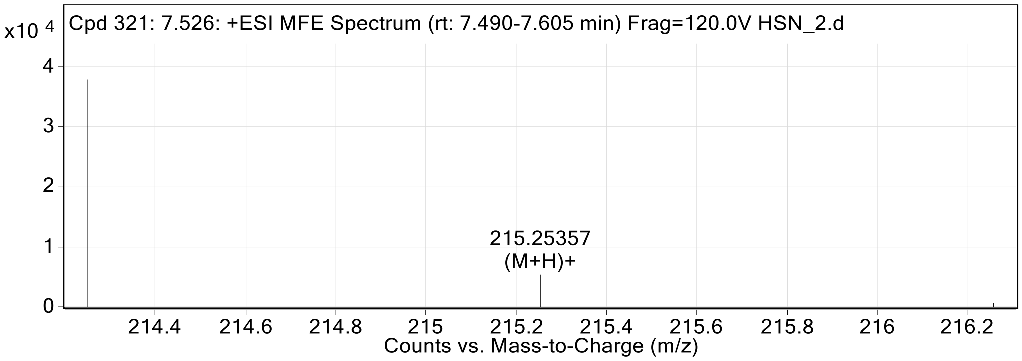

Peak List

m/z	z	Abund	Ion
214.25047	1	37803.35	(M+H)+
215.25357	1	5346.58	(M+H)+
216.25776	1	630.84	(M+H)+

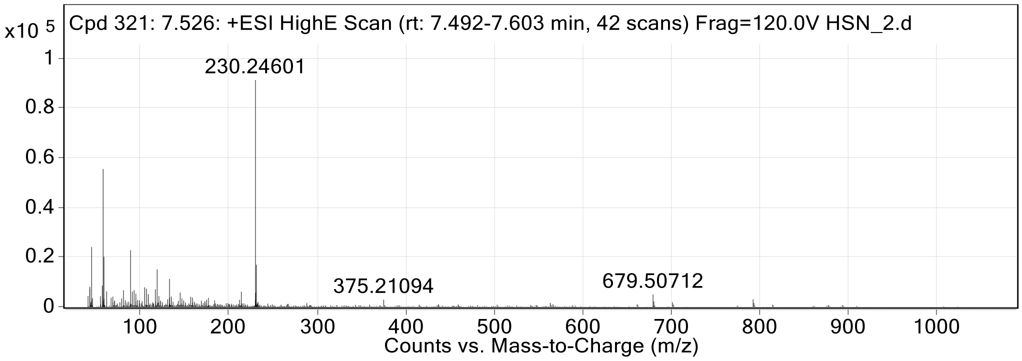

Peak List

m/z	z	Abund
43.05395		8028.26
45.03317		23990.94
57.0693		8458.67
58.06466	1	55404.48
59.04855		20113.51
89.0585		22710.69
119.08361	1	15017.15
133.08469		11169.58
230.24601	1	91179.11
231.24862	1	16874.76

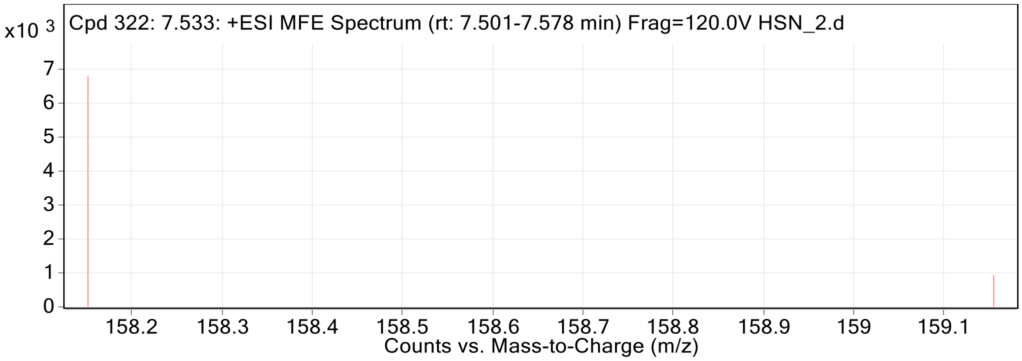

Peak List

m/z	z	Abund	Ion
158.15157	1	6822.21	(M+H)+
159.15545	1	942.42	(M+H)+

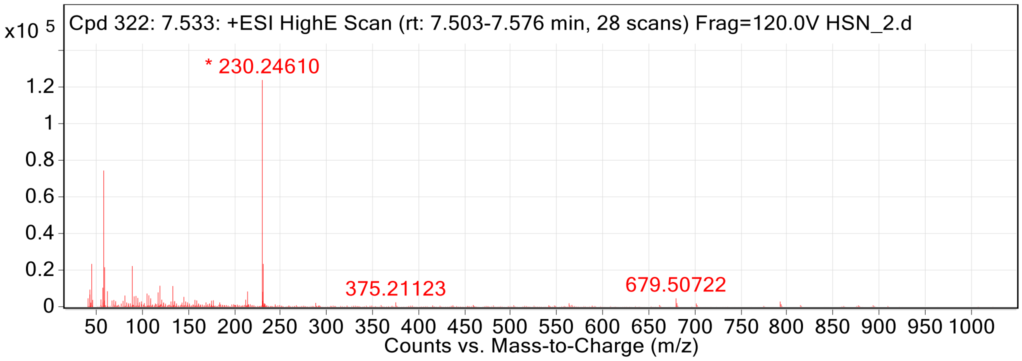

Peak List

m/z	z	Abund
43.05392		9354.94
45.03316		23404.97
57.06929		10400.41
58.06467	1	74458.66
59.04856		21577.57
89.05848		22250.51
119.08365	1	11470.3
133.08465		11318.58
230.2461	1	123976.43
231.24864	1	23401.68

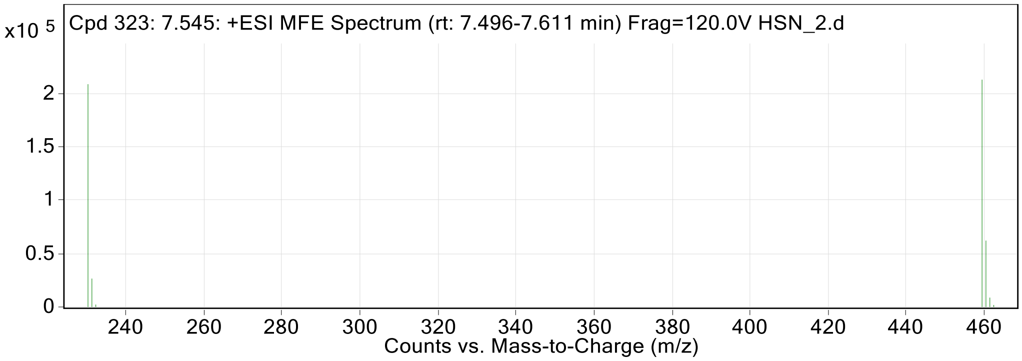

Peak List

m/z	z	Abund	Ion
230.24526	1	208782.86	(M+H)+
231.24863	1	26595.7	(M+H)+
232.25095	1	2229.17	(M+H)+

459.48458	1	213018.48	(2M+H)+
460.48796	1	62205.3	(2M+H)+
461.49198	1	8763.78	(2M+H)+
462.4946	1	1865.13	(2M+H)+

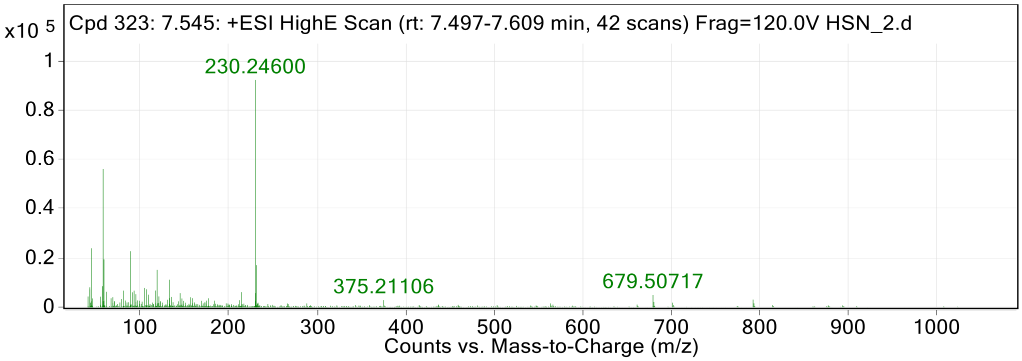

Peak List

m/z	z	Abund
43.05394		7998.19
45.03317		23827.05
57.0693		8466.41
58.06466	1	55998.9
59.04855		19334.49
89.05849		22644.09
119.08362	1	15133.57
133.08469		11113.86
230.246	1	92153.21
231.24862	1	17013.45

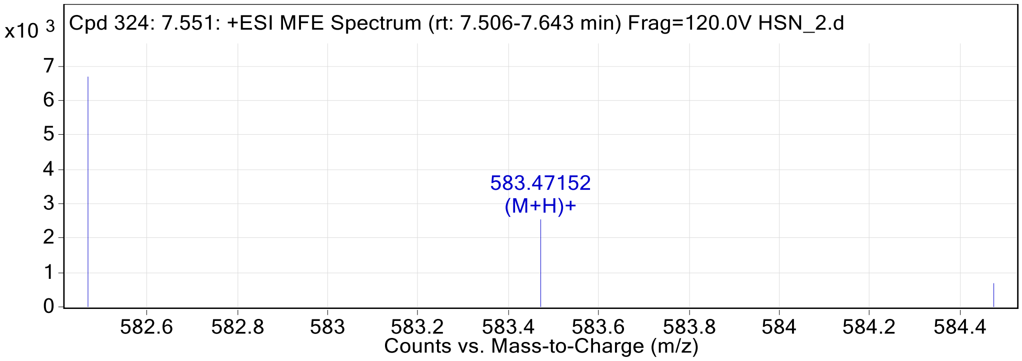

Peak List

m/z	z	Abund	Ion
582.46891	1	6688.33	(M+H)+
583.47152	1	2541.15	(M+H)+
584.4748	1	688.53	(M+H)+

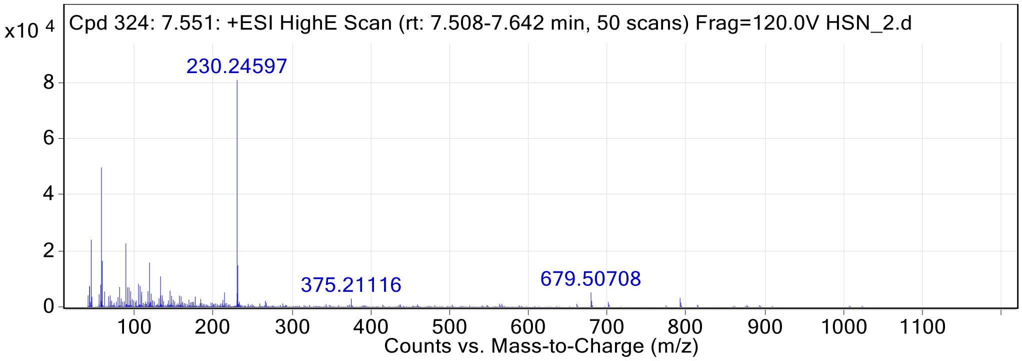

Peak List

m/z	z	Abund
45.03317		23911.76
57.0693		7919.27
58.06465	1	49647.39
59.04856		16391.87
89.05849		22577.93
105.06821		8197.43
119.08363	1	15778.38
133.08478		10857.6
230.24597	1	80831.81
231.24863	1	14812.75

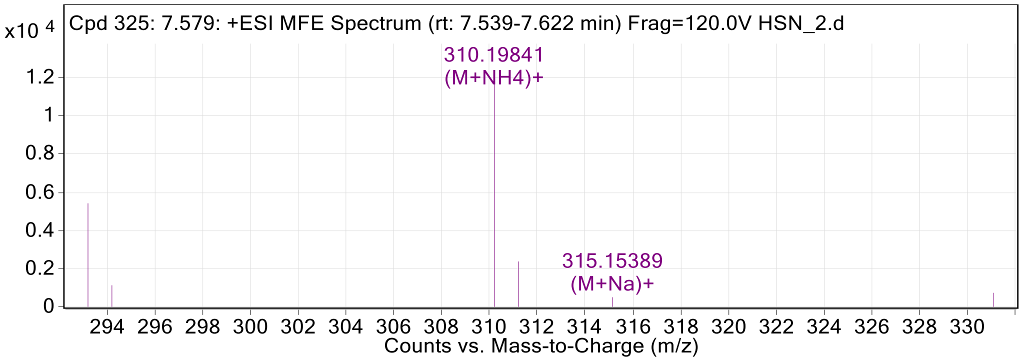

Peak List

m/z	z	Abund	Ion
293.17218	1	5424.66	(M+H)+
294.17507	1	1126.91	(M+H)+
310.19841	1	11954.83	(M+NH4)+
311.20232	1	2376.66	(M+NH4)+
315.15389	1	499.33	(M+Na)+
331.12603	1	731.32	(M+K)+

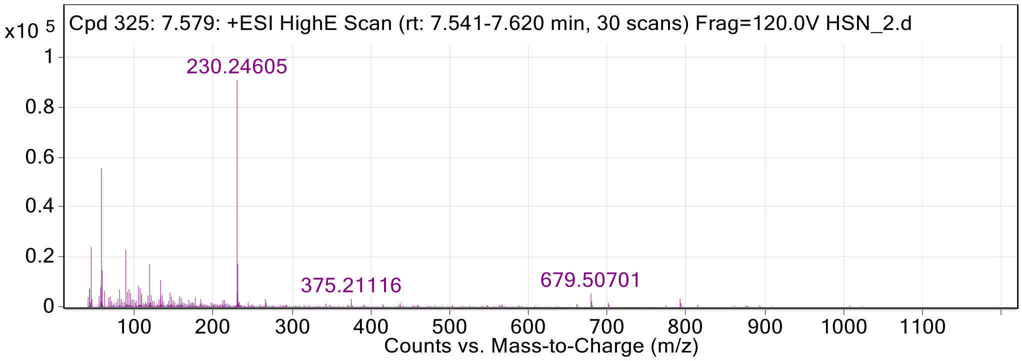

Peak List

m/z	z	Abund
45.03318		23848.68
57.0693		7856.01
58.06467	1	55408.73
59.04855		14568.06
89.0585		22732.75
105.06824		8133.92
119.08364	1	17016.63
133.08484		10627.62
230.24605	1	90784.7
231.24859	1	17102.26

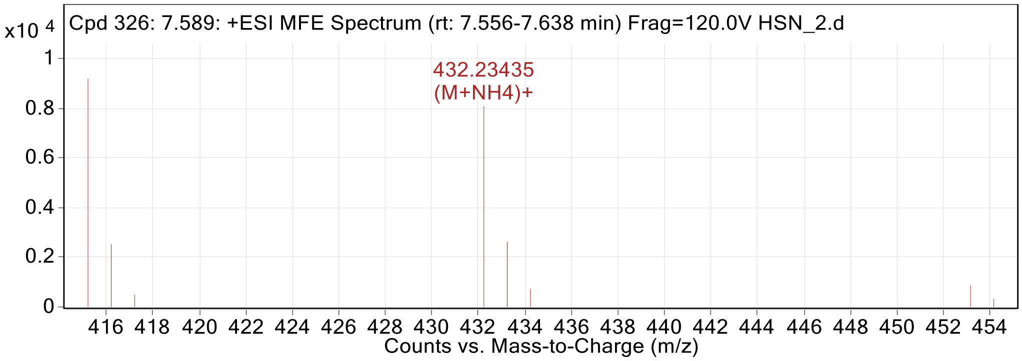

Peak List

m/z	z	Abund	Ion
415.2082	1	9197.54	(M+H)+
416.21146	1	2529.16	(M+H)+
417.2163	1	484.45	(M+H)+
432.23435	1	8095.28	(M+NH4)+
433.23982	1	2635.29	(M+NH4)+
434.24033	1	733.44	(M+NH4)+
453.16537	1	876.88	(M+K)+
454.16482	1	328.94	(M+K)+

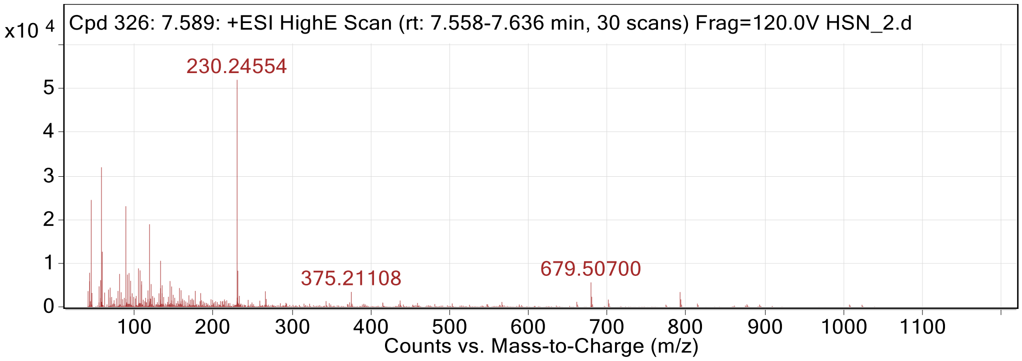

Peak List

m/z	z	Abund
45.03317		24491.98
58.0646	1	31974.28
59.04856		12628.91
89.05849		23057.63
105.06817		8813.2
107.08383		8350.28
119.08362	1	18921.52
133.08498		10557.11
230.24554	1	52000.5
231.24862	1	8277.56

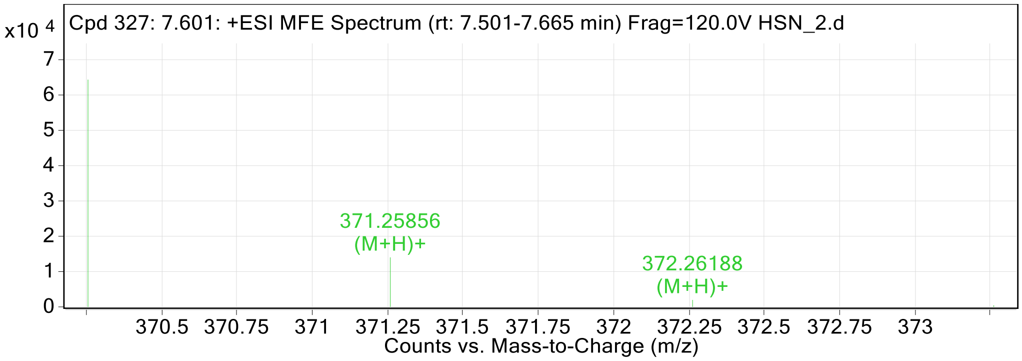

Peak List

m/z	z	Abund	Ion
370.25544	1	64221.12	(M+H)+
371.25856	1	13985.74	(M+H)+
372.26188	1	1965.49	(M+H)+
373.26147	1	447.81	(M+H)+

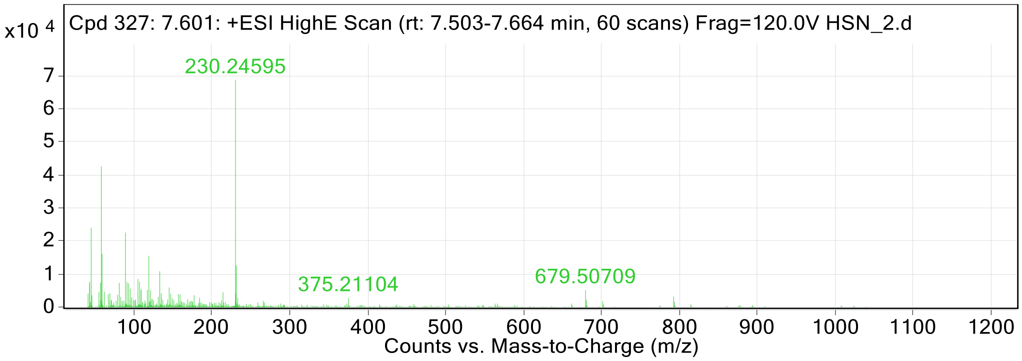

Peak List

m/z	z	Abund
45.03317		24001.14
58.06465	1	42547.36
59.04855		16127.09
89.05848		22523.85
105.06819		8476.36
107.08387		7704.48
119.08362	1	15512.84
133.08477		10812.13
230.24595	1	68727.15
231.24863	1	12560.23

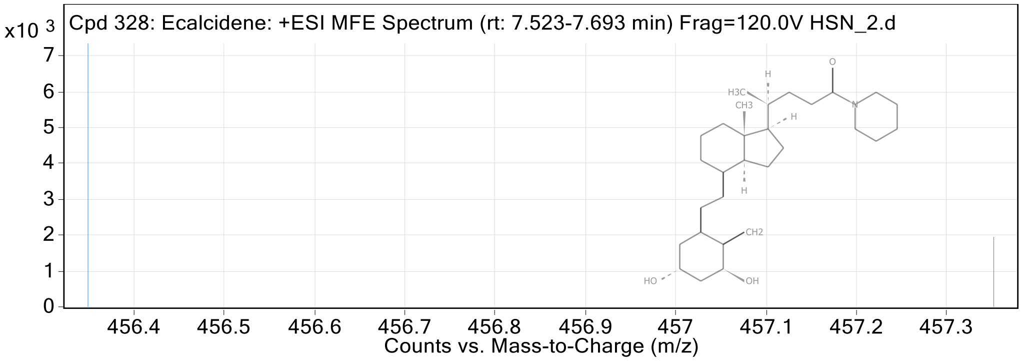

Peak List

m/z	z	Abund	Name	Formula	Ion	Score (DB)	Hits (DB)
456.34917	1	7351.63	Ecalcidene	C29 H45 N O3	(M+H)+	71.31	1

457.35182	1	1951.87		C29 H45 N O3	(M+H)+		

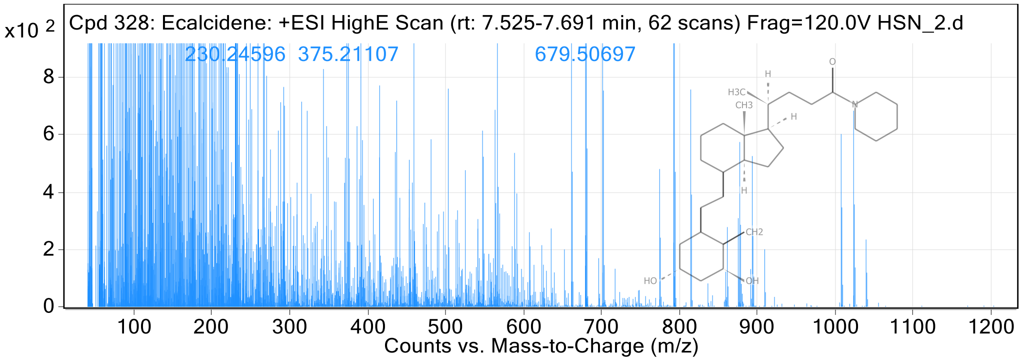

Peak List

m/z	z	Abund
43.01752		7982.52
45.03317		23902.95
58.06465	1	40099.32
59.04856		14281.7
89.05847		22390.3
105.06819		8682.91
119.0836	1	15559.94
133.08466		10654.9
230.24596	1	64730.09
231.24863	1	11942.76

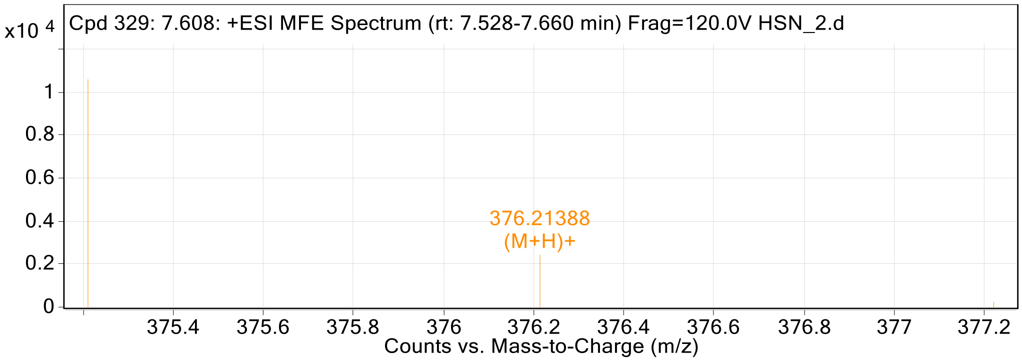

Peak List

m/z	z	Abund	Ion
375.21076	1	10596.28	(M+H)+
376.21388	1	2434.87	(M+H)+
377.22099	1	252.46	(M+H)+

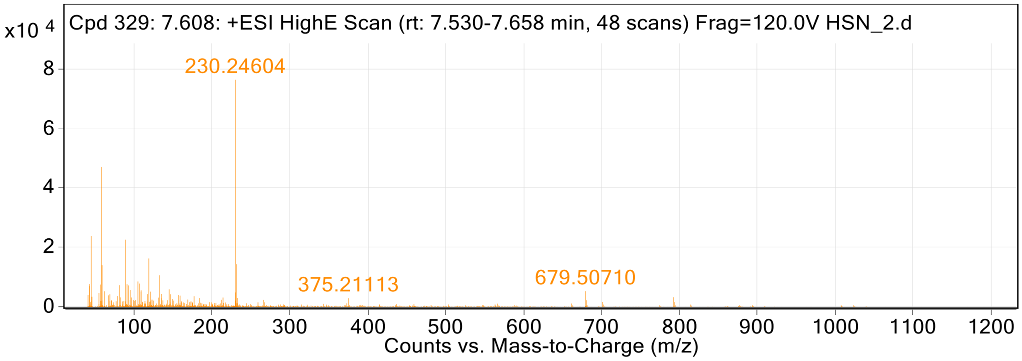

Peak List

m/z	z	Abund
45.03317		23860.86

58.06467	1	46998.03
59.04856		13969.1
89.05849		22554.54
105.06816		8495.26
107.08389		7793.22
119.08362	1	16225.05
133.08483		10582.39
230.24604	1	76307.24

Qualitative Analysis Report

m/z	z	Abund
231.24866	1	14317.79

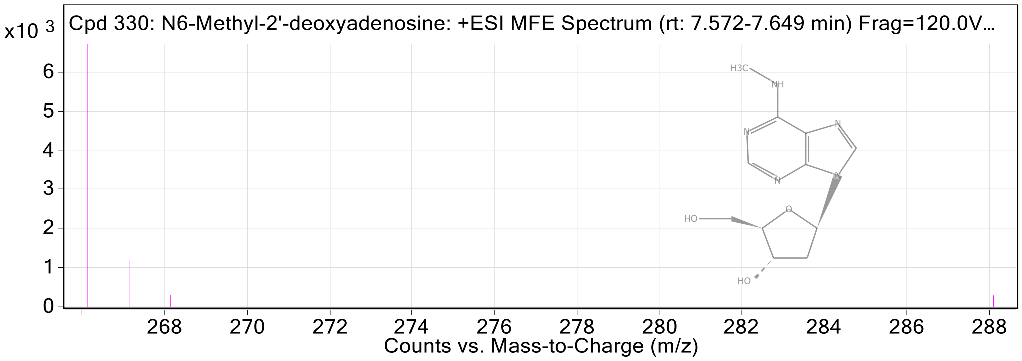

Peak List

m/z	z	Abund	Name	Formula	Ion	Score (DB)	Hits (DB)
266.12559	1	6723.42	N6-Methyl-2'-deoxyadenosine	C11 H15 N5 O3	(M+H)+	89.39	1
267.12931	1	1183.4		C11 H15 N5 O3	(M+H)+		
268.13056	1	302.14		C11 H15 N5 O3	(M+H)+		
288.1079	1	293.44			(M+Na)+		

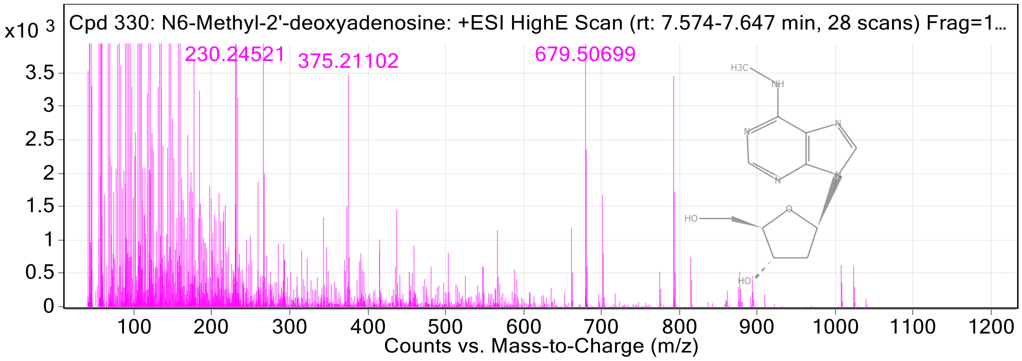

Peak List

m/z	z	Abund
45.03318		24738.72
58.06458		18257.69
59.04857		11549.94
89.05848		22971.54
91.05305		8594.57
105.0682		9364.35
107.08387		8874.48
119.08362	1	20205.14
133.08493		10518.98
230.24521	1	26667.93

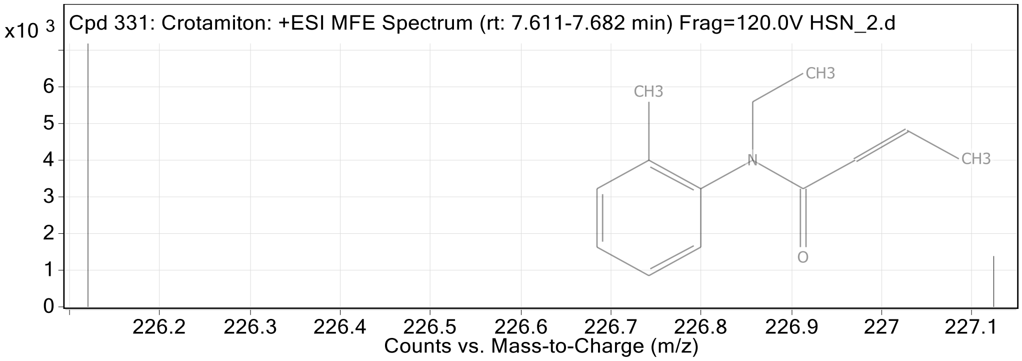

Peak List

m/z	z	Abund	Name	Formula	Ion	Score (DB)	Hits (DB)
226.12042	1	7166.67	Crotamiton	C13 H17 N O	(M+Na)+	81.17	2
227.12418	1	1382.03		C13 H17 N O	(M+Na)+		

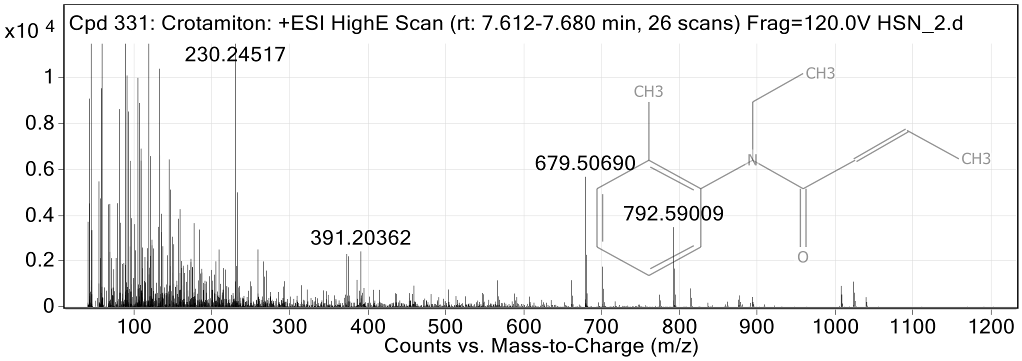

Peak List

m/z	z	Abund
43.01751		9093.31
45.03316		24555.55
58.0646		9544.46
59.04853		11527.46
89.05843		22374.44
91.05308		10105.97
105.06813		10000.99
119.08359	1	15909.04
133.0847		10399.91
230.24517	1	11496.45

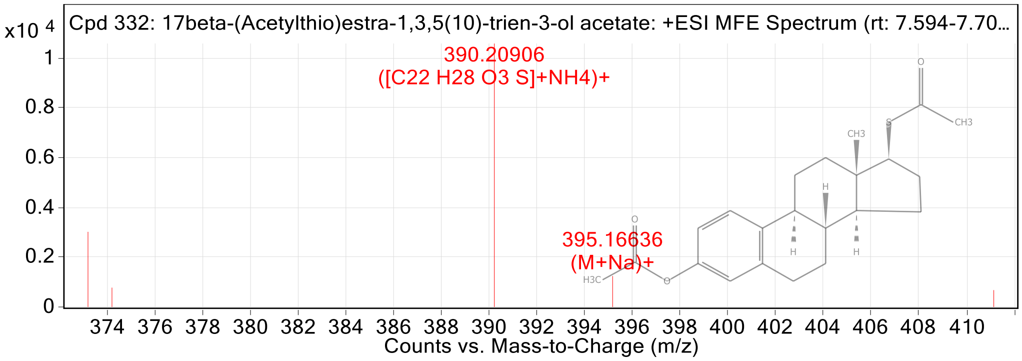

Peak List

m/z	z	Abund	Name	Formula	Ion	Score (DB)	Hits (DB)
373.18272	1	3018.28			(M+H)+		
374.18757	1	776.09			(M+H)+		

390.20906	1	10584.83	17beta-(Acetylthio)estra-1,3,5(10)-trien-3-ol acetate	C22 H28 O3 S	(M+NH4)+	46.32	1
395.16636	1	1242.66			(M+Na)+		
411.13776	1	677.03			(M+K)+		

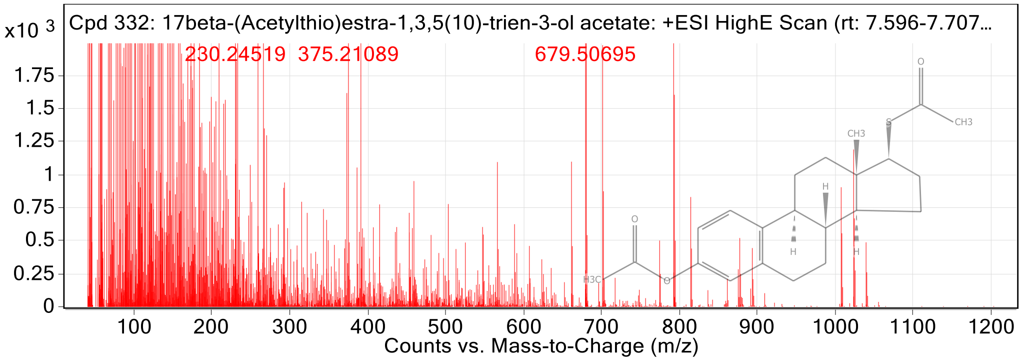

Peak List

m/z	z	Abund
43.01751		8827.95
45.03317		24392.42
58.06457		9784.75
59.04854		11540.41
89.05845		22790.6
91.05303		8597.11
105.06818		9484.08
119.08359	1	15998.35
133.08471		10451.59
230.24519	1	11956.73

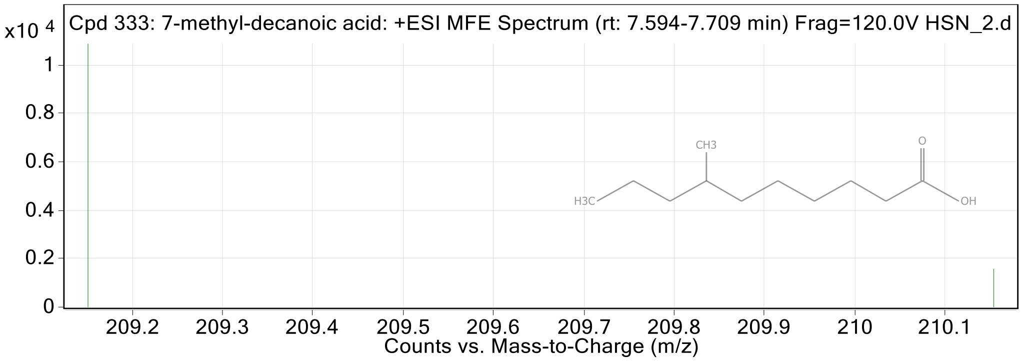

Peak List

m/z	z	Abund	Name	Formula	Ion	Score (DB)	Hits (DB)
209.1512	1	10888.95	7-methyl-decanoic acid	C11 H22 O2	(M+Na)+	85.09	10
210.15401	1	1584.96		C11 H22 O2	(M+Na)+		

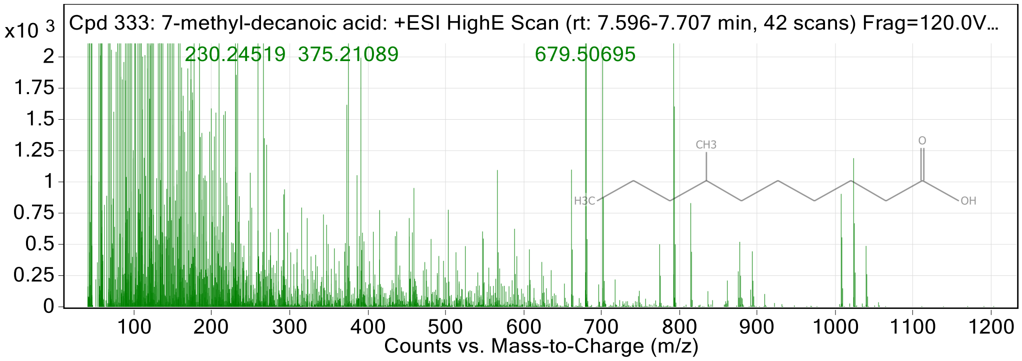

Peak List

m/z	z	Abund
43.01751		8827.95
45.03317		24392.42
58.06457		9784.75
59.04854		11540.41
89.05845		22790.6
91.05303		8597.11
105.06818		9484.08
119.08359	1	15998.35
133.08471		10451.59
230.24519	1	11956.73

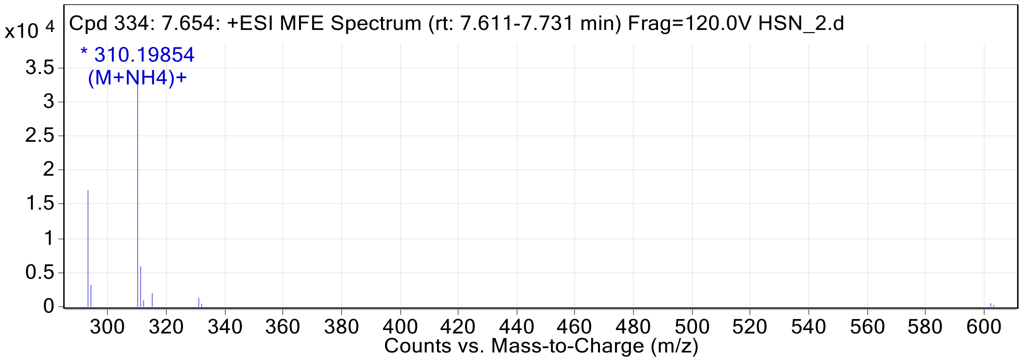

Peak List

m/z	z	Abund	Ion
293.17171	1	17064.84	(M+H)+
294.1756	1	3219.94	(M+H)+
310.19854	1	33217.96	(M+NH4)+
311.20129	1	5907.83	(M+NH4)+
312.20748	1	943.89	(M+NH4)+
315.15466	1	2004.96	(M+Na)+
331.12761	1	1301.49	(M+K)+
332.13251	1	447.22	(M+K)+
602.3581	1	517.02	(2M+NH4)+
603.36563	1	265.21	(2M+NH4)+

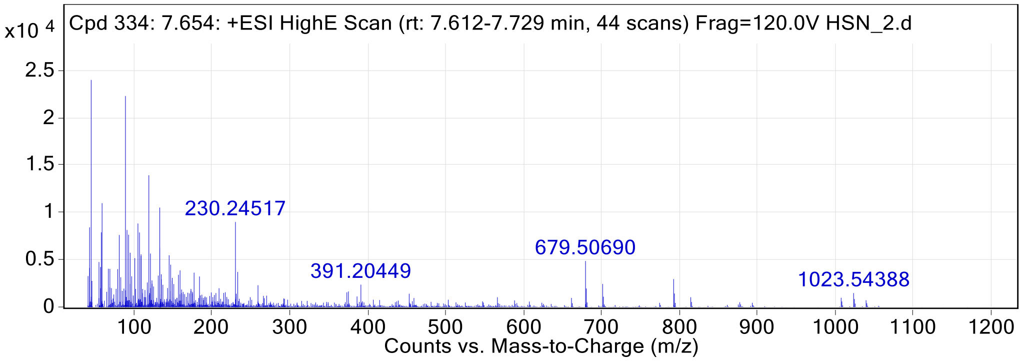

Peak List

m/z	z	Abund
43.01749		8408.5
45.03316		23975.69
58.06456		7879.28
59.04853		10963.91
89.05843		22279.21
91.05302		8124.61
105.06815		8820.35
119.0836	1	13905.23

133.08455		10497.04
230.24517	1	8978.09

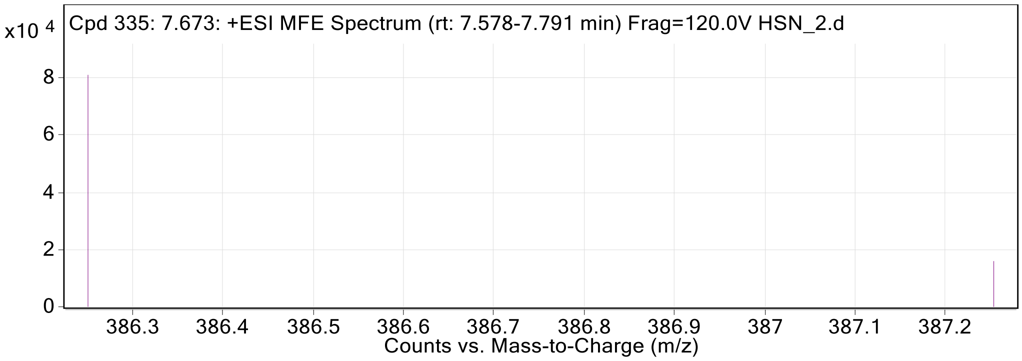

Peak List

m/z	z	Abund	Ion
386.25037	1	81009.35	(M+H)+
387.25377	1	15963.73	(M+H)+

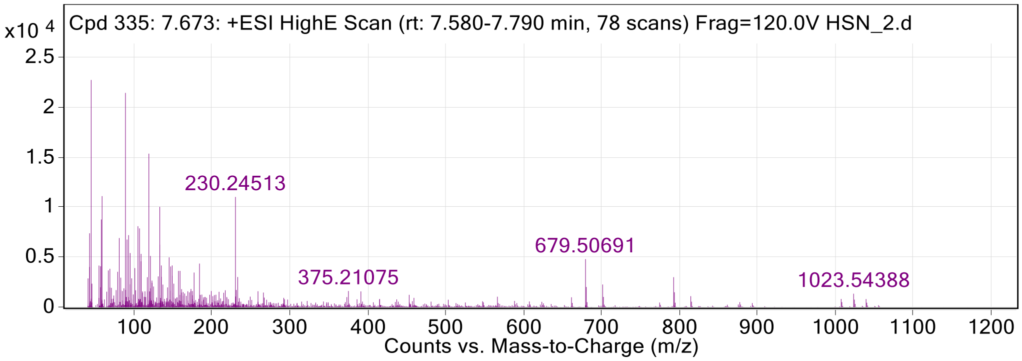

Peak List

m/z	z	Abund
43.01747		7376.69
45.03315		22720.38
58.06456		8754.94
59.04854		11095.81
89.05844		21426.52
105.0682		8083.9
107.08381		7864.99
119.08358	1	15347.36
133.08457		10023.64
230.24513	1	11014.66

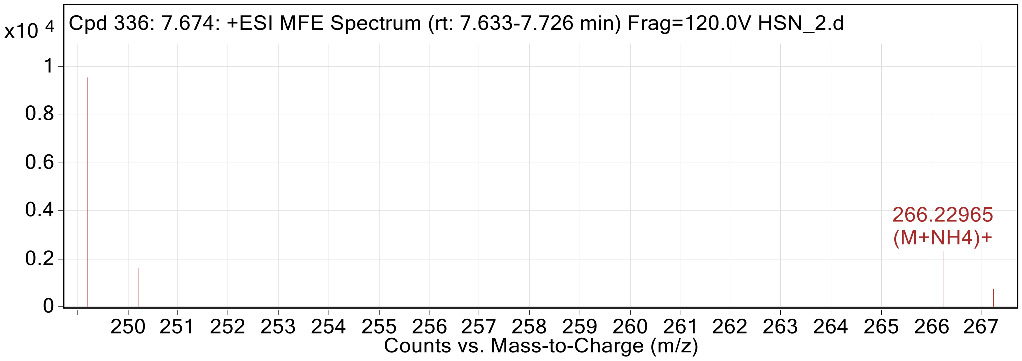

Peak List

m/z	z	Abund	Ion

249.2034	1	9545.37	(M+H)+
250.20697	1	1619.35	(M+H)+
266.22965	1	2313.41	(M+NH4)+
267.23435	1	748.59	(M+NH4)+

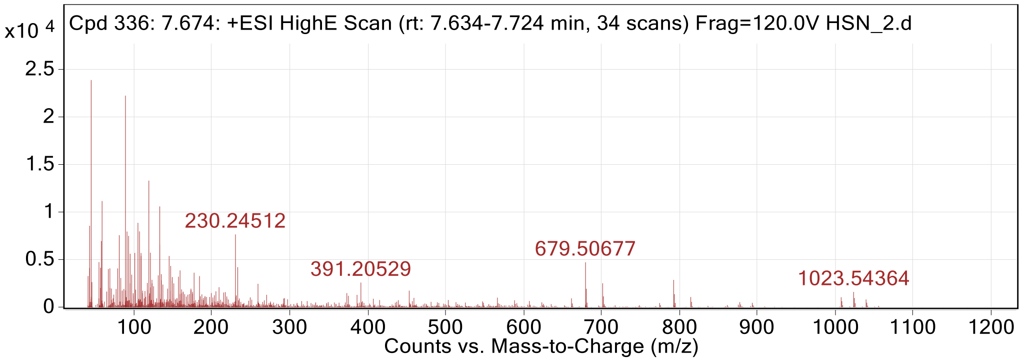

Peak List

m/z	z	Abund
43.01748		8545.52
45.03317		23854.33
59.04855		11141.34
89.05842		22215.82
91.05299		7935.09
105.06817		8847.38
107.08387		7955.61
119.08357	1	13286.38
133.08443		10577.66
230.24512		7636.7

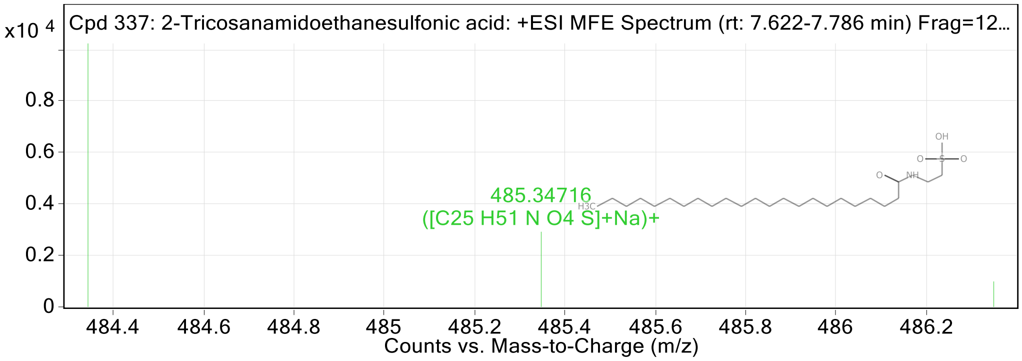

Peak List

m/z	z	Abund	Name	Formula	Ion	Score (DB)	Hits (DB)
484.34399	1	10241.73	Tricosanamidoethanesulfonic acid	C25 H51 N O4 S	(M+Na)+	92.26	1
485.34716	1	2921.47		C25 H51 N O4 S	(M+Na)+		
486.34888	1	989.49		C25 H51 N O4 S	(M+Na)+		

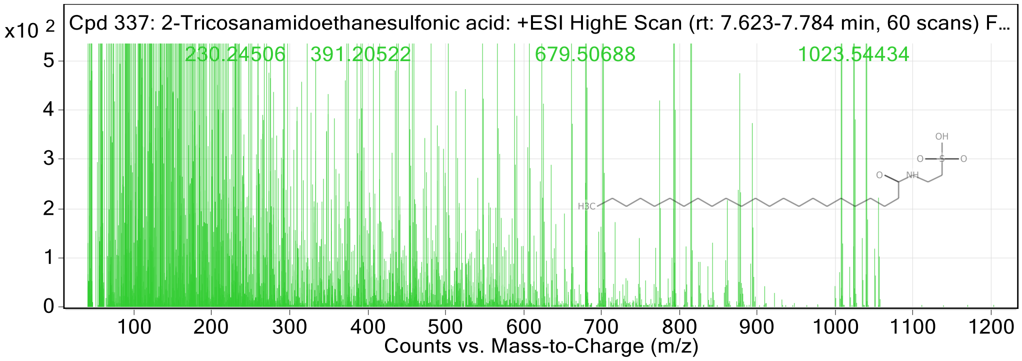

Peak List

m/z	z	Abund
43.01746		7253.52
45.03315		22346.46
59.04854		10664.66
89.05842		21126.9
91.05299		6810.55
93.06844		6898.9
105.06817		7813.9
107.08381		7383.34
119.08358	1	12155.73
133.08447		9982.77

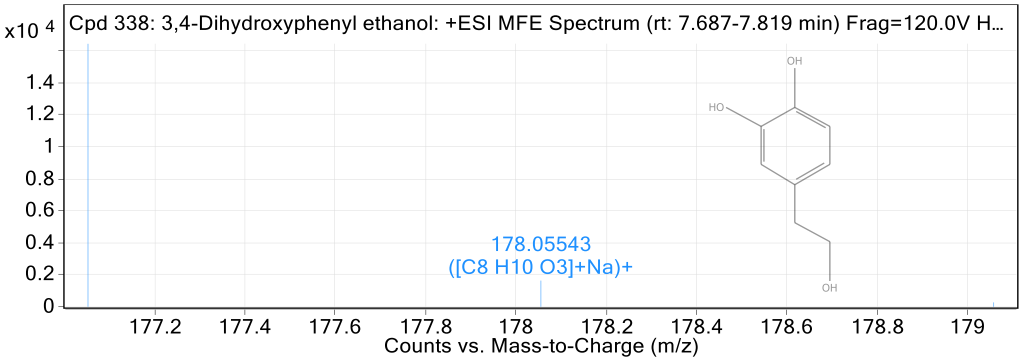

Peak List

m/z	z	Abund	Name	Formula	Ion	Score (DB)	Hits (DB)
177.0522	1	16425.96	3,4-Dihydroxyphenyl ethanol	C8 H10 O3	(M+Na)+	99.35	8
178.05543	1	1635.99		C8 H10 O3	(M+Na)+		
179.05852	1	273.46		C8 H10 O3	(M+Na)+		

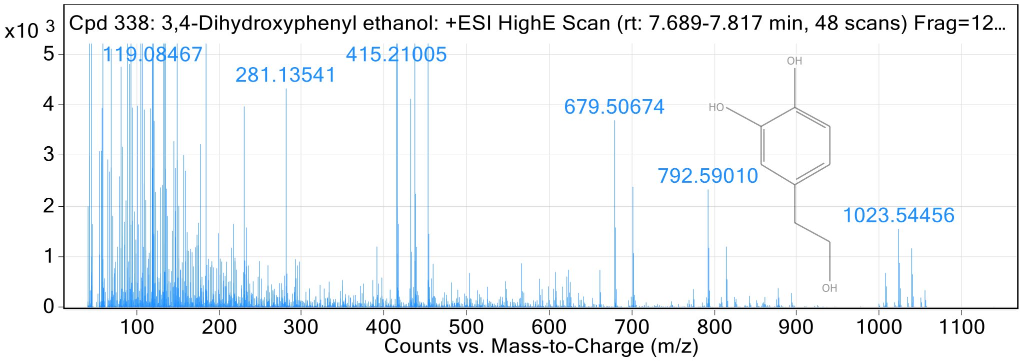

Peak List

m/z	z	Abund
45.03313		20507.15

59.04852		12144.58
89.05843		19436.62
107.08378		13352.82
119.08467		53243.95
133.08435		9655.41
135.07843	1	24296.87
415.21005	1	24298.14
416.21173	1	9671.95
437.18989	1	9081

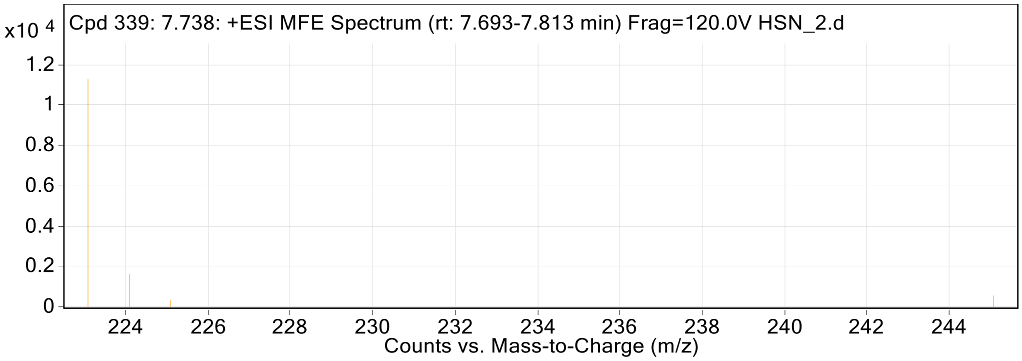

Peak List

m/z	z	Abund	Ion
223.09364	1	11289.78	(M+H)+
224.09722	1	1616.98	(M+H)+
225.09948	1	330.03	(M+H)+
245.07645	1	555.26	(M+Na)+

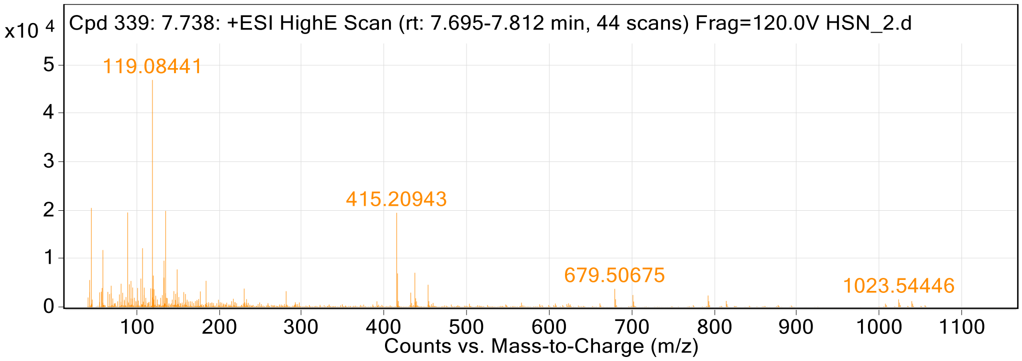

Peak List

m/z	z	Abund
45.03312		20462.02
59.04853		11766.04
89.05842		19524.32
107.08376		12143.93
119.08441	1	46924.28
133.08438		9560.49
135.07839	1	19854.17
149.02108		7764.52
415.20943	1	19464.67
437.18994	1	7069.19

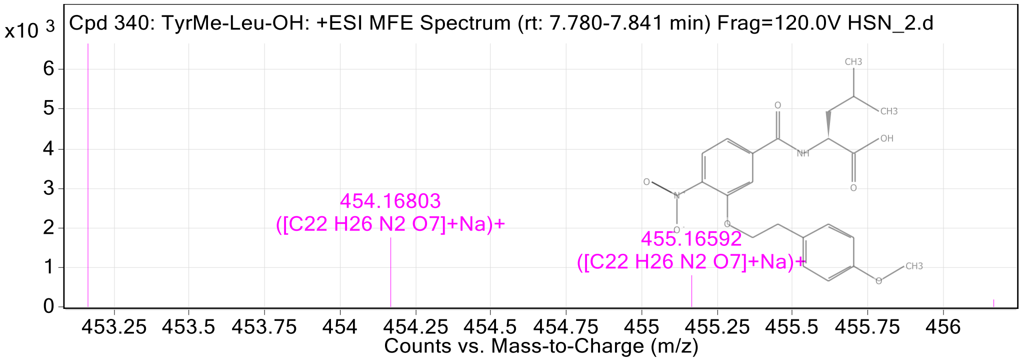

Peak List

m/z	z	Abund	Name	Formula	Ion	Score (DB)	Hits (DB)
453.16422	1	6664.13	TyrMe-Leu-OH	C22 H26 N2 O7	(M+Na)+	82.37	2
454.16803	1	1755.28		C22 H26 N2 O7	(M+Na)+		
455.16592	1	800.39		C22 H26 N2 O7	(M+Na)+		
456.16714	1	187.59		C22 H26 N2 O7	(M+Na)+		

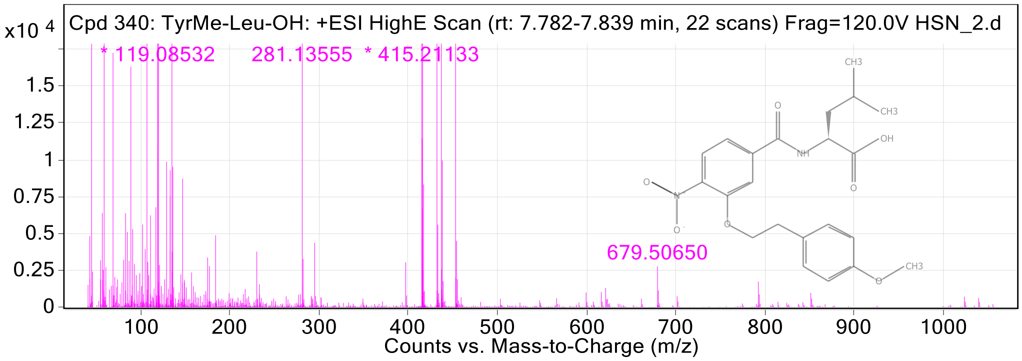

Peak List

m/z	z	Abund
107.08384	1	36328
119.08532		186127.61
119.17974		22751.37
120.08698	1	34250.95
135.07849	1	100325.5
281.13555	1	21075.25
415.21133	1	109780.13
416.21181	1	52253.48
432.23458	1	22726.05
437.18999	1	41170.96

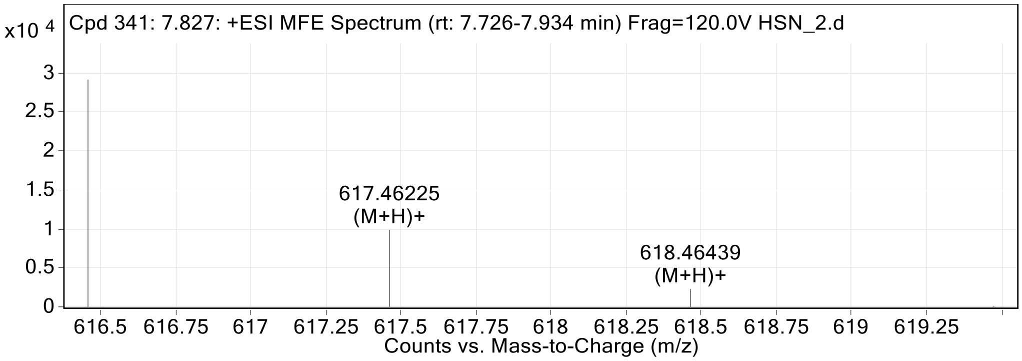

Peak List

m/z	z	Abund	Ion
616.45871	1	29128.19	(M+H)+

617.46225	1	9869.93	(M+H)+
618.46439	1	2286.41	(M+H)+
619.47352	1	58.6	(M+H)+

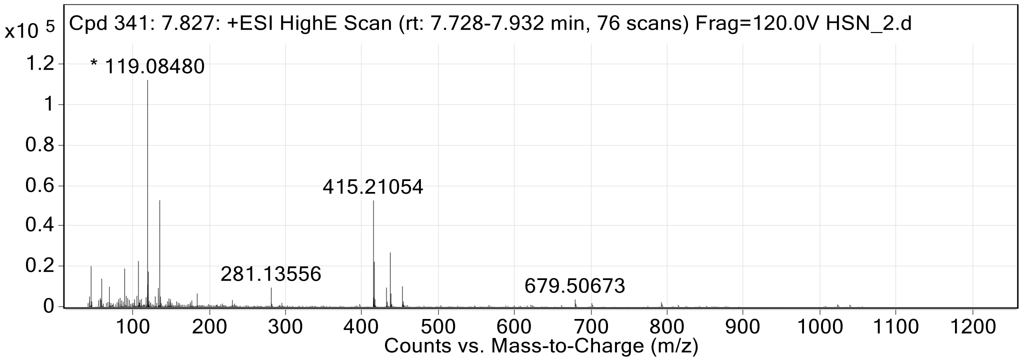

Peak List

m/z	z	Abund
45.03314		20079.25
59.04856		13864.5
89.05844		18891.95
107.08384	1	22606.78
119.0848		112249.92
120.08694		17391.51
135.07845	1	52738.55
415.21054	1	52613.64
416.21174	1	22220.77
437.18993	1	26851.19

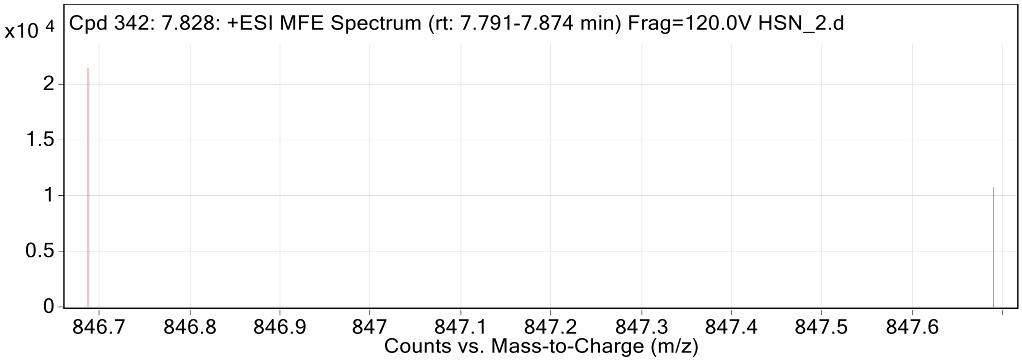

Peak List

m/z	z	Abund	Ion
846.68749	1	21436.9	(M+H)+
847.69023	1	10727.74	(M+H)+

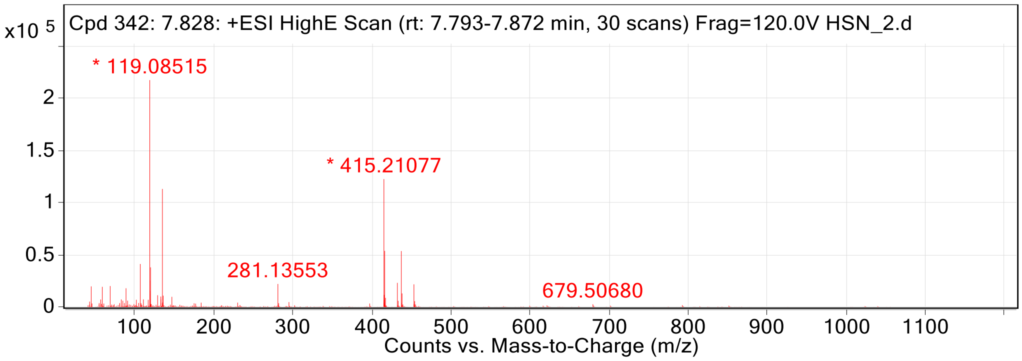

Peak List

m/z	z	Abund

107.08388	1	41061.65
119.08515		217421.72
119.17977	1	25241.22
120.08696	1	37827.27
135.07848	1	113031.38
281.13553	1	21904.68
415.21077	1	122485.3
416.21175	1	53621.99
432.23449	1	22967.86
437.18995	1	53447.2

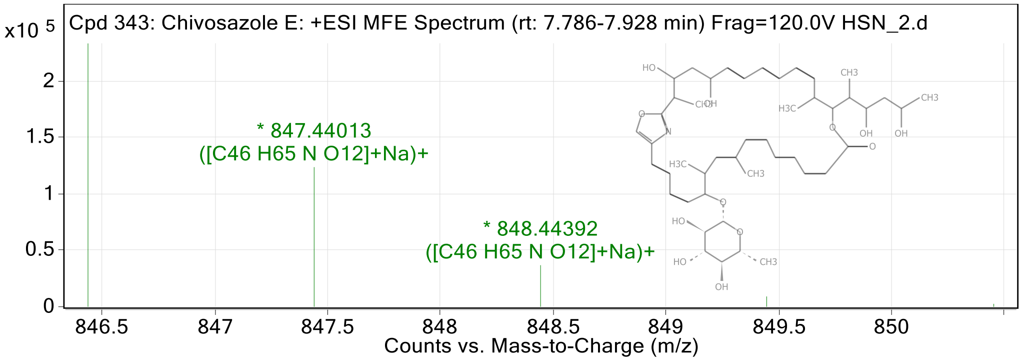

Peak List

m/z	z	Abund	Name	Formula	Ion	Score (DB)	Hits (DB)
846.43754	1	233135.48	Chivosazole E	C46 H65 N O12	(M+Na)+	92.88	1
847.44013	1	123557.9		C46 H65 N O12	(M+Na)+		
848.44392	1	36789.88		C46 H65 N O12	(M+Na)+		
849.44632	1	9124.18		C46 H65 N O12	(M+Na)+		
850.45315	1	2580.3		C46 H65 N O12	(M+Na)+		

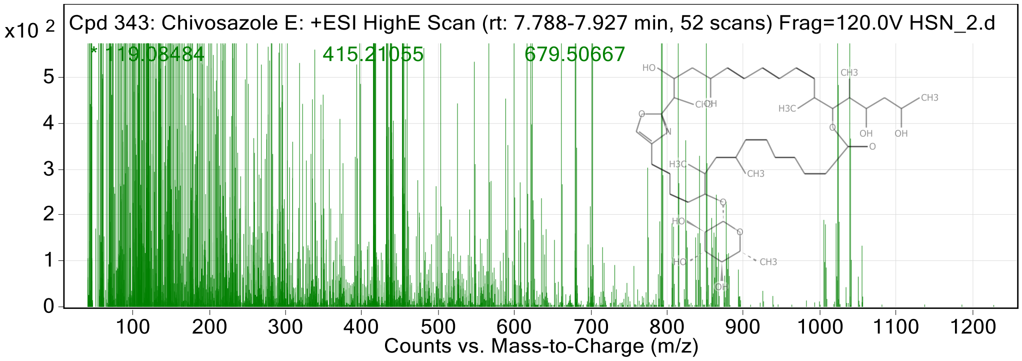

Peak List

m/z	z	Abund
45.03315		20219.25
89.05846		18716.77
107.08386	1	29938.4
119.08484		158517.42
119.17976		15725.69
120.08694	1	24876.99
135.07845	1	75440.04
415.21055	1	76513.47
416.21174	1	32357.54
437.18993	1	38731.34

Qualitative Analysis Report

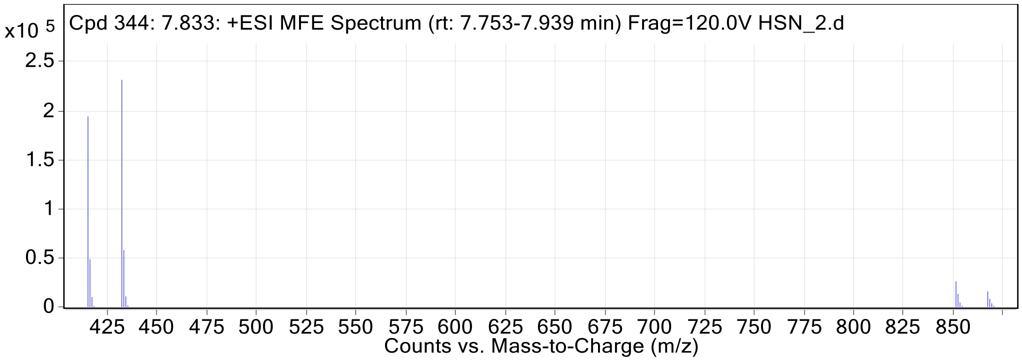

Peak List

m/z	z	Abund	Ion
415.20824	1	194157.95	(M+H)+
416.2114	1	48670.47	(M+H)+
417.21406	1	10175.85	(M+H)+
432.23347	1	231383.3	(M+NH4)+
433.23755	1	57901.07	(M+NH4)+
434.24057	1	10745.95	(M+NH4)+
851.39295	1	26032.23	(2M+Na)+
852.39597	1	13237.63	(2M+Na)+
867.36722	1	15670.19	(2M+K)+
868.37054	1	8190.05	(2M+K)+

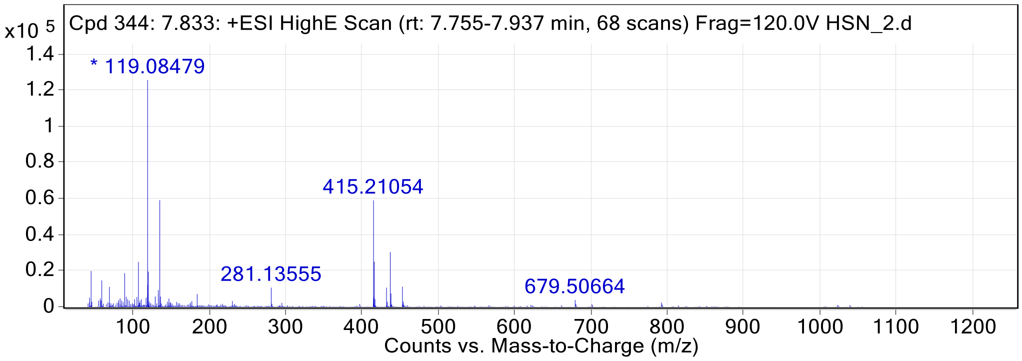

Peak List

m/z	z	Abund
45.03314		19800.89
59.04856		14576.66
89.05842		18633.81
107.08385	1	24811.26
119.08479		125516.81
120.08694	1	19451.02
135.07845	1	59005.55
415.21054	1	58918.86
416.21175	1	24875.96

m/z	z	Abund
437.18994	1	30218.25

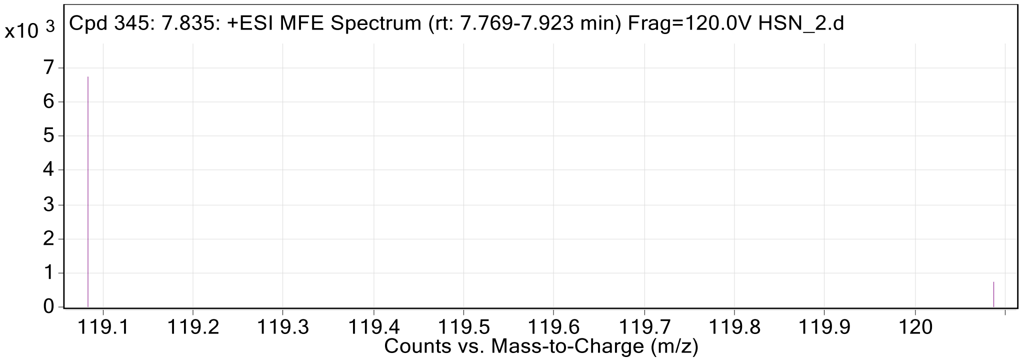

Peak List

m/z	z	Abund	Ion
119.08375	1	6750.53	(M+H)+
120.08733	1	738.96	(M+H)+

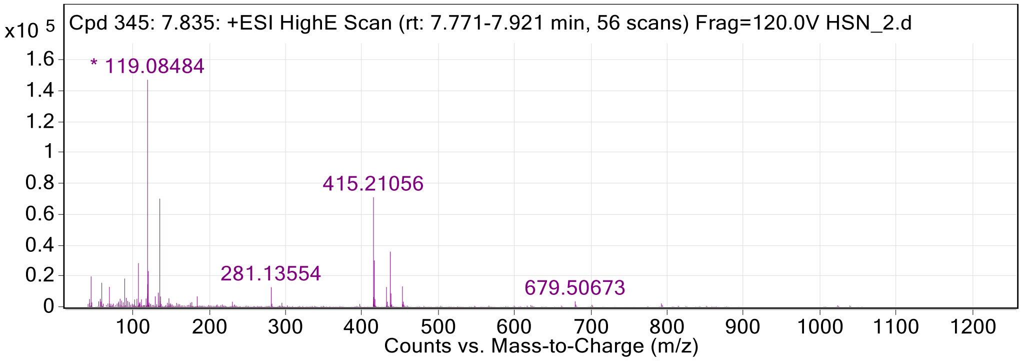

Peak List

m/z	z	Abund
45.03314		19682.83
59.04855		15554.27
89.05845		18307.24
107.08385	1	28305.89
119.08484		147204.8
120.08694	1	23133.89
135.07845	1	70120.2
415.21056	1	71005.33
416.21174	1	30030.98
437.18993	1	35771.93

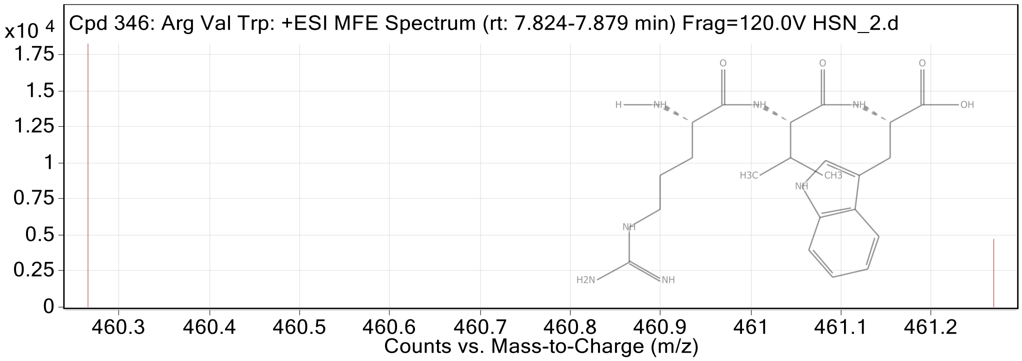

Peak List

m/z	z	Abund	Name	Formula	Ion	Score (DB)	Hits (DB)
460.26553	1	18282.27	Arg Val Trp	C22 H33 N7 O4	(M+H)+	80.38	6

461.2691	1	4728.41		C22 H33 N7 O4	(M+H)+		

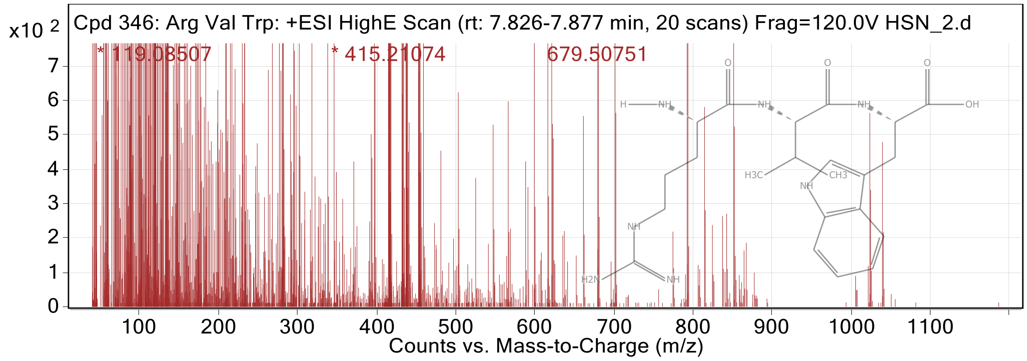

Peak List

m/z	z	Abund
107.08391	1	40056.36
119.08507		213921.91
119.17975		24109.43
120.08692	1	36247.56
135.07847	1	108213.54
415.21074	1	114706.71
416.21173	1	49635.41
432.23443	1	21245.32
437.1899	1	56754.22
453.16379	1	21230.55

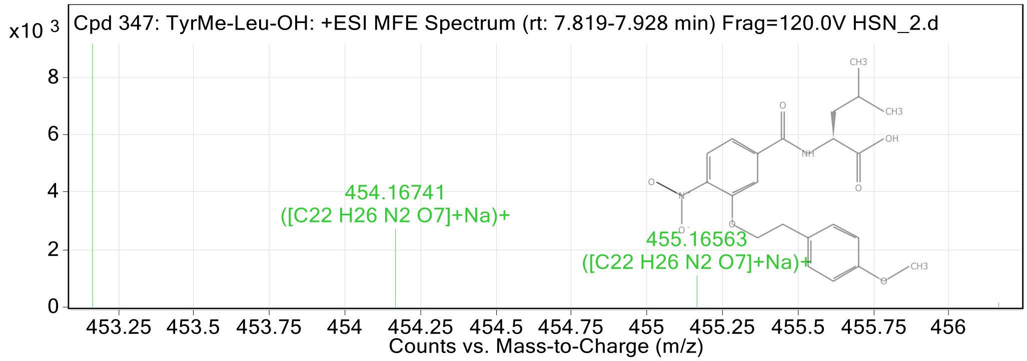

Peak List

m/z	z	Abund	Name	Formula	Ion	Score (DB)	Hits (DB)
453.16385	1	9156.38	TyrMe-Leu-OH	C22 H26 N2 O7	(M+Na)+	84.2	2
454.16741	1	2715.34		C22 H26 N2 O7	(M+Na)+		
455.16563	1	1092		C22 H26 N2 O7	(M+Na)+		
456.16498	1	157.26		C22 H26 N2 O7	(M+Na)+		

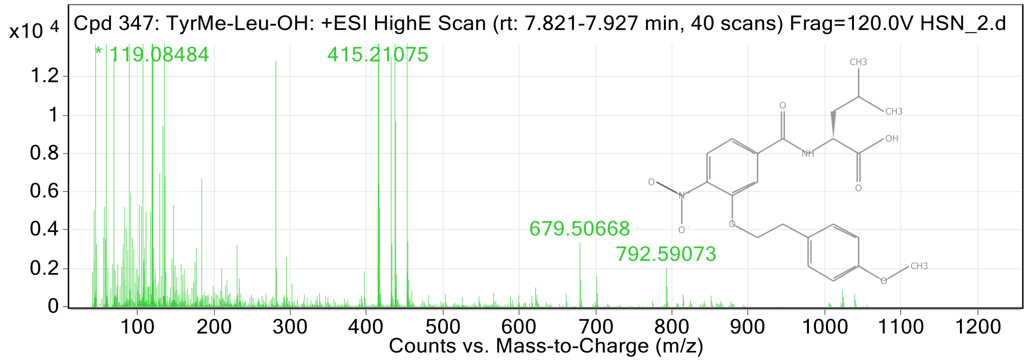

Peak List

m/z	z	Abund

45.03315		20604.13
89.05844		19330.9
107.08389	1	28576.17
119.08484		151112.23
119.17976		14770.43
120.08693	1	23582.4
135.07846	1	71325.96
415.21075	1	70603.39
416.21175	1	30542.35
437.18995	1	39722.77

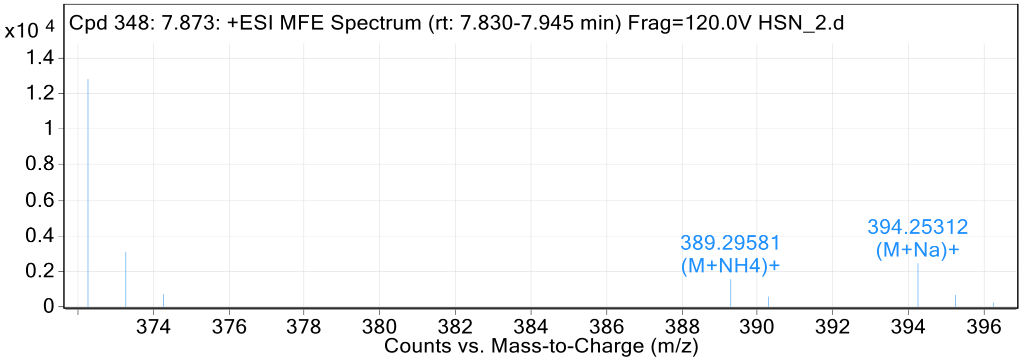

Peak List

m/z	z	Abund	Ion
372.27117	1	12826.04	(M+H)+
373.27384	1	3100.72	(M+H)+
374.27709	1	713.9	(M+H)+
389.29581	1	1551.65	(M+NH4)+
390.29929	1	570.97	(M+NH4)+
394.25312	1	2447.18	(M+Na)+
395.25123	1	661.13	(M+Na)+
396.26024	1	238.28	(M+Na)+

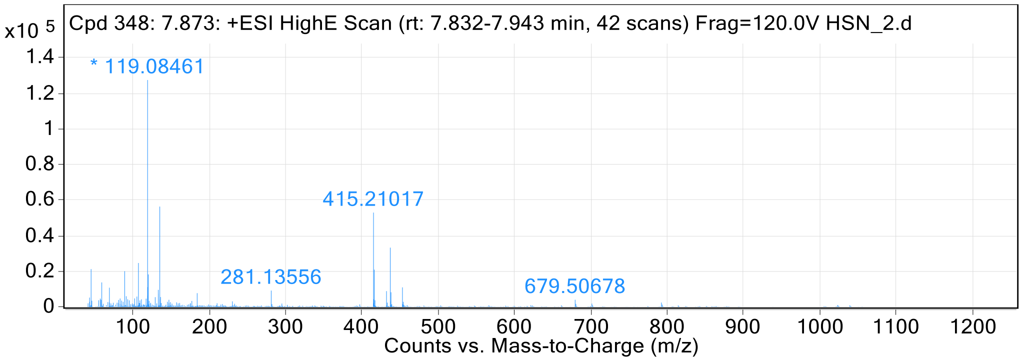

Peak List

m/z	z	Abund
45.03315		21168.5
59.04858		13676.33
89.0584		19991.43
107.08388	1	24599.67
119.08461	1	127508.36
120.08692	1	18173.48
135.07844	1	56326.64
415.21017	1	52950.89
416.21171	1	20852.91
437.18992	1	33230.15

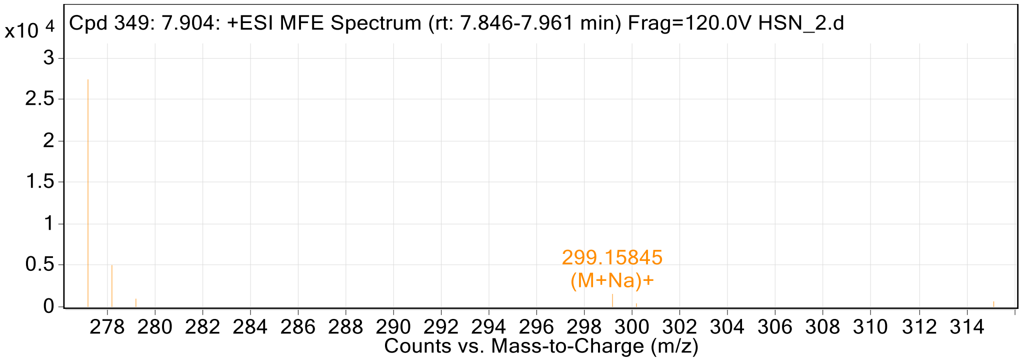

Peak List

m/z	z	Abund	Ion
277.17681	1	27434.28	(M+H)+
278.18064	1	5032.01	(M+H)+
279.18638	1	988.58	(M+H)+
299.15845	1	1561.87	(M+Na)+
300.16136	1	425.62	(M+Na)+
315.13591	1	664.78	(M+K)+

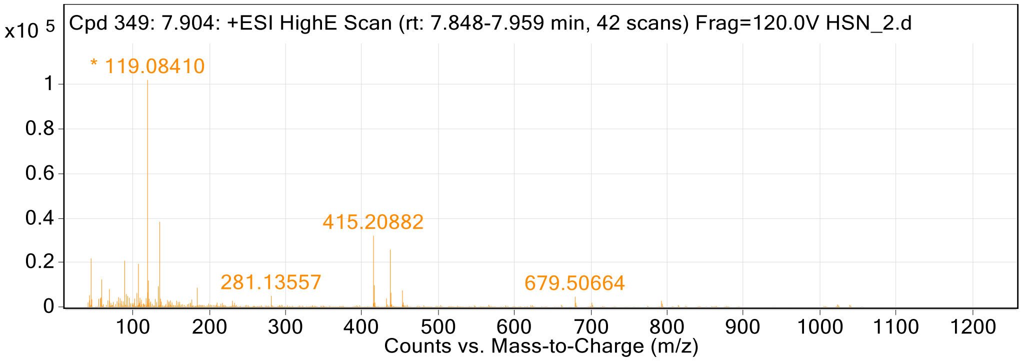

Peak List

m/z	z	Abund
45.03316		21853.83
59.04856		12382.93
89.0584		20728.58
107.08385	1	19391.75
119.0841	1	102135.09
120.08688	1	11849.42
135.07839	1	38283.43
415.20882	1	32050.43
416.21154	1	9756.58
437.18987	1	25766.55

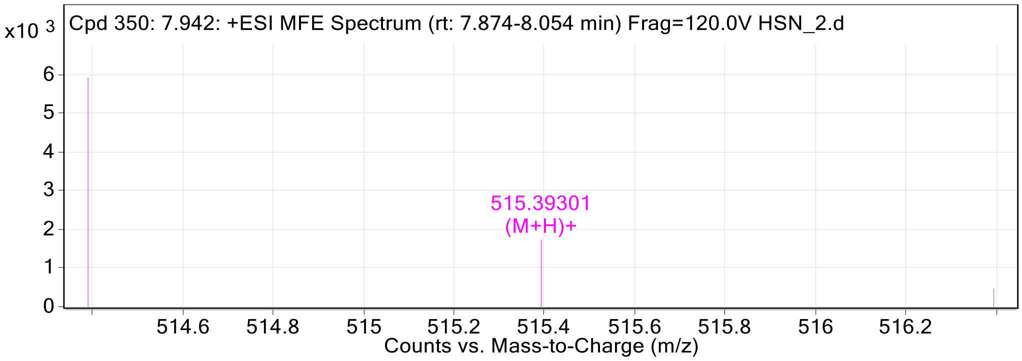

Peak List

m/z	z	Abund	Ion
514.3905	1	5920.09	(M+H)+
515.39301	1	1736.29	(M+H)+
516.39444	1	468.37	(M+H)+

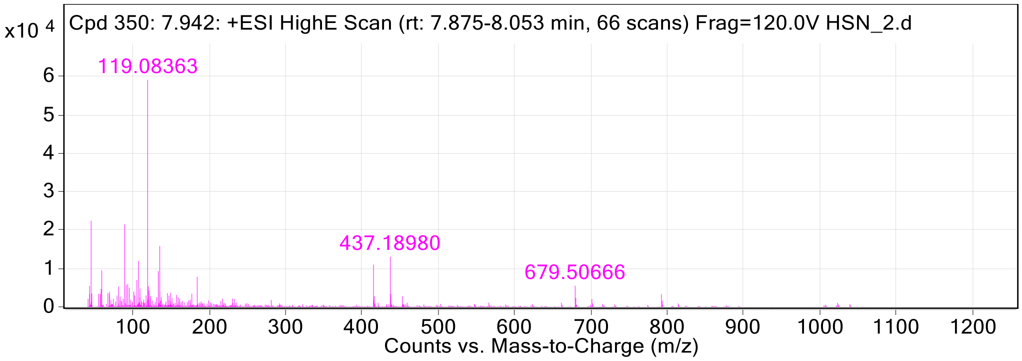

Peak List

m/z	z	Abund
45.03312		22421.63
59.04856		9524.45
89.05837		21460.85
107.08375		11941.44
119.08363	1	59082.37
133.08478		9303.38
133.09786		9152.69
135.07831	1	15827.29
415.20795	1	10968.34
437.1898	1	13063.86

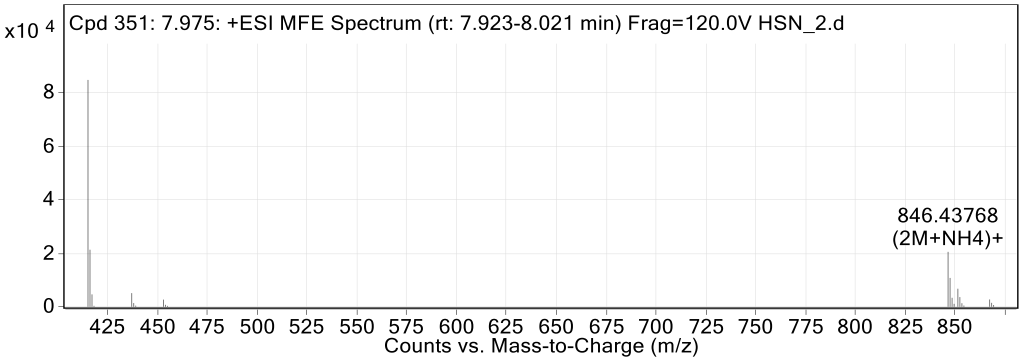

Peak List

m/z	z	Abund	Ion
415.20762	1	84797.31	(M+H)+
416.21164	1	21323.37	(M+H)+
417.21486	1	4629.99	(M+H)+
437.19019	1	5096.83	(M+Na)+
846.43768	1	20526.03	(2M+NH4)+
847.44092	1	10779.03	(2M+NH4)+
848.44453	1	3355.15	(2M+NH4)+
851.39154	1	6747.76	(2M+Na)+
852.39607	1	3613.76	(2M+Na)+
867.36664	1	2724.2	(2M+K)+

Qualitative Analysis Report

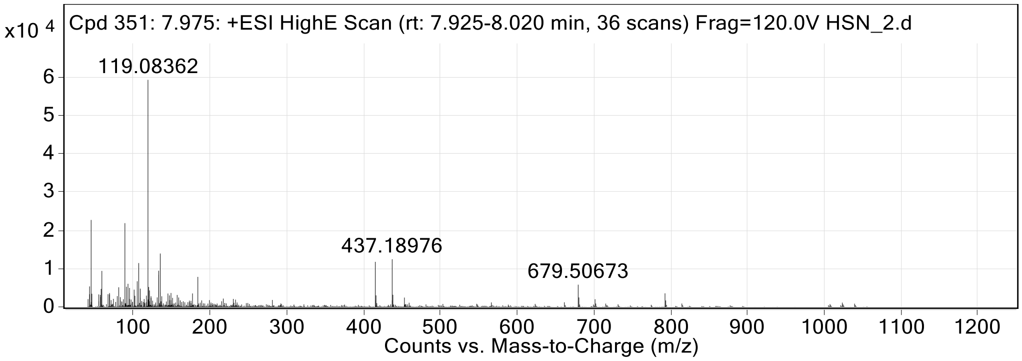

Peak List

m/z	z	Abund
45.03312		22703.49
59.04859		9389.81
89.05837		21870.14
107.08373		11439.18
119.08362	1	59298.3
133.08482		9440.56
133.09793		8379.17
135.07833	1	13930.39
415.20798	1	11764.17
437.18976	1	12444.06

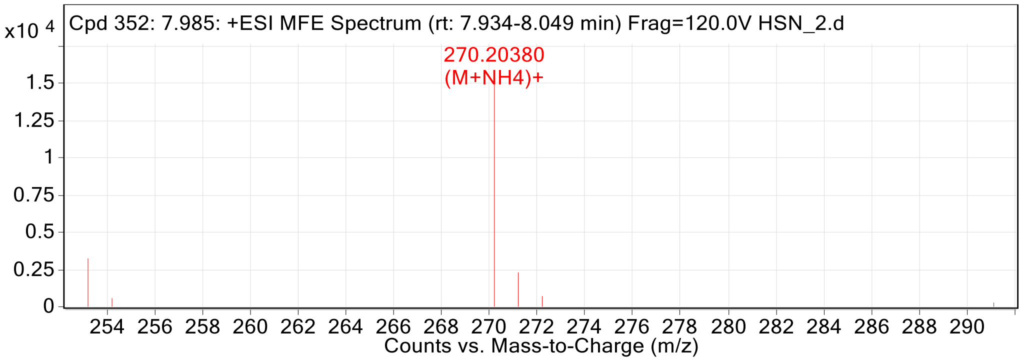

Peak List

m/z	z	Abund	Ion
253.17698	1	3254.62	(M+H)+
254.18392	1	579.91	(M+H)+
270.2038	1	15263.22	(M+NH4)+
271.20599	1	2311.35	(M+NH4)+
272.21197	1	715.89	(M+NH4)+

m/z	z	Abund	Ion
291.12877	1	280.56	(M+K)+

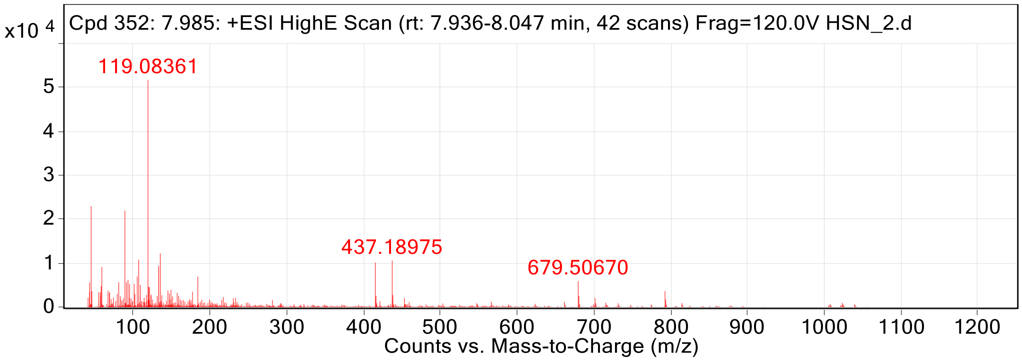

Peak List

m/z	z	Abund
45.03311		22952.35
59.04858		9113.83
89.05836		21982.38
107.08371		10764.93
119.08361	1	51721.97
133.08477		9384.99
133.09792		8869.38
135.07834	1	12195.57
415.20798	1	10133.85
437.18975	1	10561.7

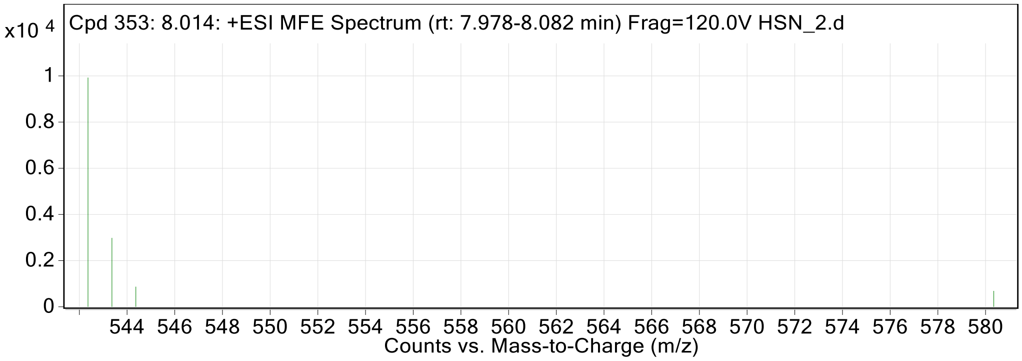

Peak List

m/z	z	Abund	Ion
542.38553	1	9930.48	(M+H)+
543.38861	1	2984.71	(M+H)+
544.39096	1	871.33	(M+H)+
580.34177	1	688.75	(M+K)+

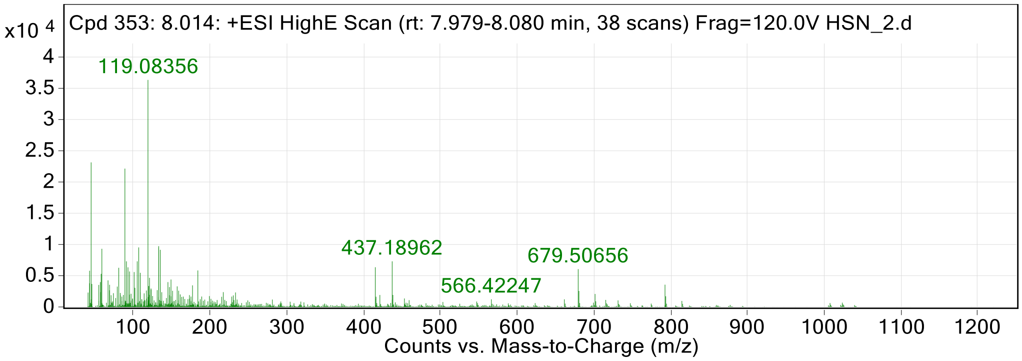

Peak List

m/z	z	Abund
45.03312		23093.76
59.04855		9293.57
89.0584		22102.72
91.0528		7287.52
105.06812		7289.9
107.08368		9506.41
119.08356	1	36270.14
133.0848		9432.86
133.09792		9712.42
135.07832		9126.96

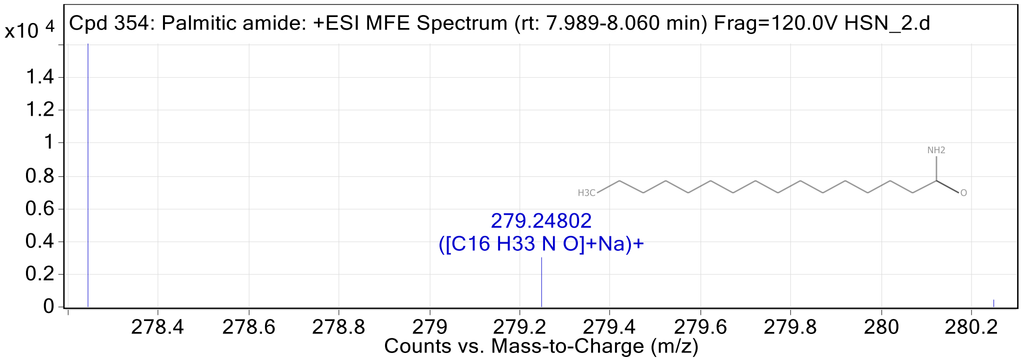

Peak List

m/z	z	Abund	Name	Formula	Ion	Score (DB)	Hits (DB)
278.24468	1	16092.36	Palmitic amide	C16 H33 N O	(M+Na)+	96.57	1
279.24802	1	3034.58		C16 H33 N O	(M+Na)+		
280.2482	1	448.71		C16 H33 N O	(M+Na)+		

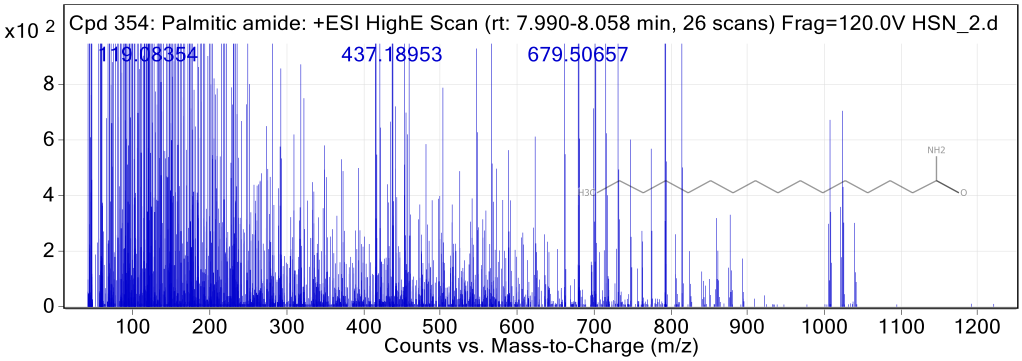

Peak List

m/z	z	Abund
45.03312		23087.55
59.04856		8562.72
89.0584		22277.56
101.05816		6692.28
105.06815		7631.32
107.08368		9031.53
119.08354	1	30603.26
133.0847		9455.74
133.09791		9828.22
135.07836		7804.63

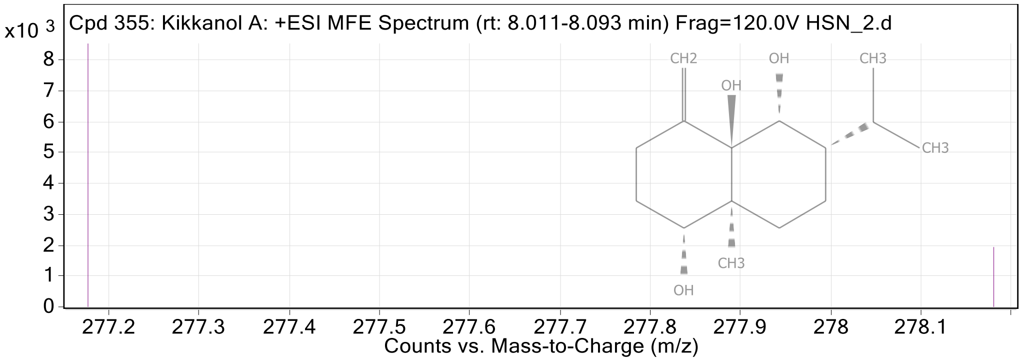

Peak List

m/z	z	Abund	Name	Formula	Ion	Score (DB)	Hits (DB)
277.17679	1	8530.57	Kikkanol A	C15 H26 O3	(M+Na)+	77.03	10
278.1807	1	1937.62		C15 H26 O3	(M+Na)+		

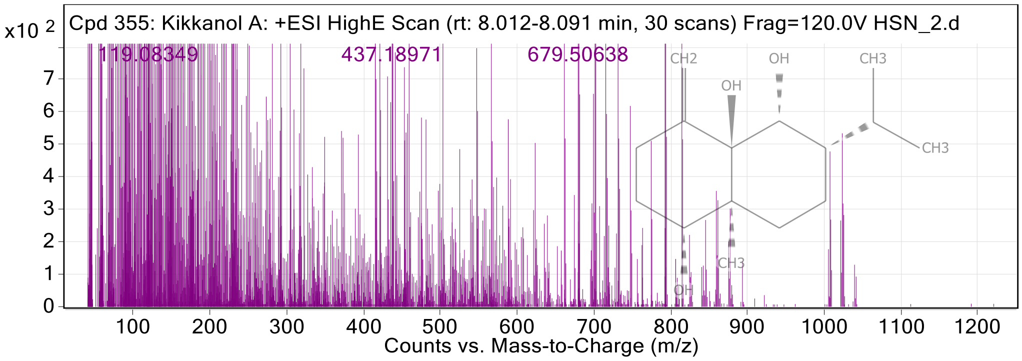

Peak List

m/z	z	Abund
45.03313		23279.68
59.04851		10017.69
81.06872		6929.36
89.05836		21869.21
91.05283		8415.93
105.06812		7455.85
107.08368		8754.88
119.08349	1	23491.6
133.08479		9535.6
133.09798	1	10383.78

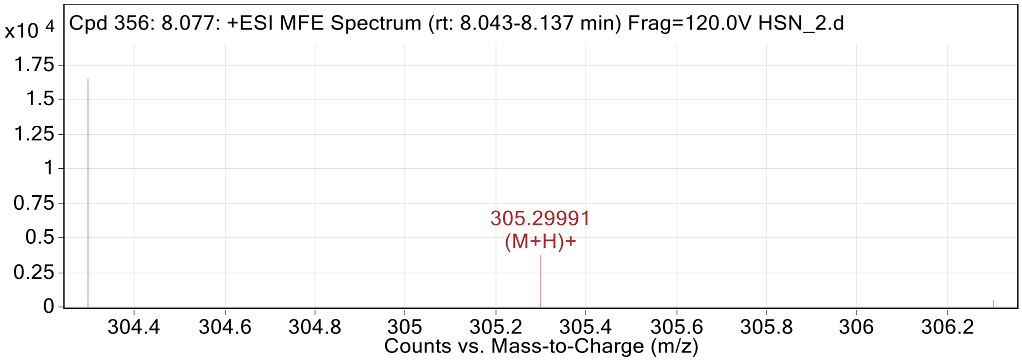

Peak List

m/z	z	Abund	Ion
304.29668	1	16478.64	(M+H)+
305.29991	1	3769.29	(M+H)+
306.30335	1	495.71	(M+H)+

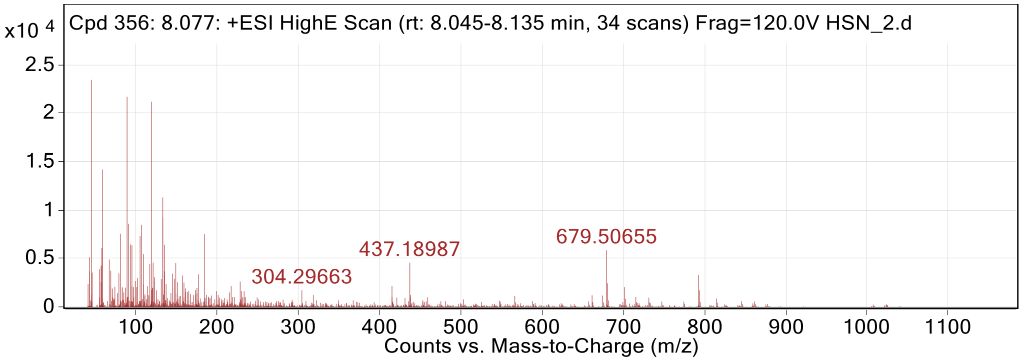

Peak List

m/z	z	Abund
45.03313		23417.78
59.04857		14169.02
81.06871		7545.53
89.05839		21677.73
91.05284		8578.42
107.0837		8478.03
119.08353	1	21173.39
133.08477		9266.32
133.09791	1	11284.17
184.07092		7520.6

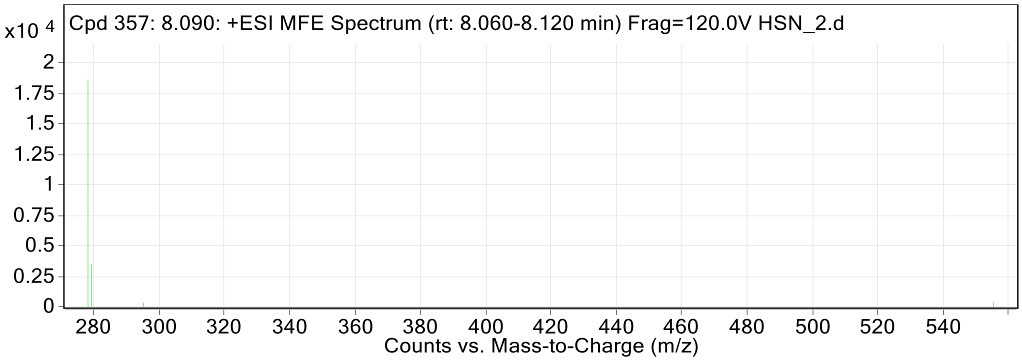

Peak List

m/z	z	Abund	Ion
278.24491	1	18642.64	(M+H)+
279.24819	1	3465.19	(M+H)+
295.27328	1	327.28	(M+NH4)+
555.48352	1	382.29	(2M+H)+

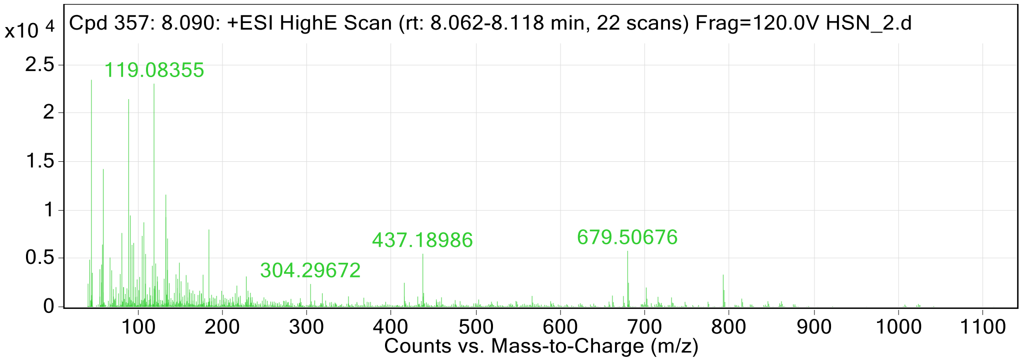

Peak List

m/z	z	Abund
45.03314		23459.94

59.04859		14240.03
81.06874		7634.32
89.0584		21464.89
91.05289		9441.73
107.08368		8732.48
119.08355	1	23063.77
133.08473		9276.57
133.09791	1	11594.22
184.07099		7990.06

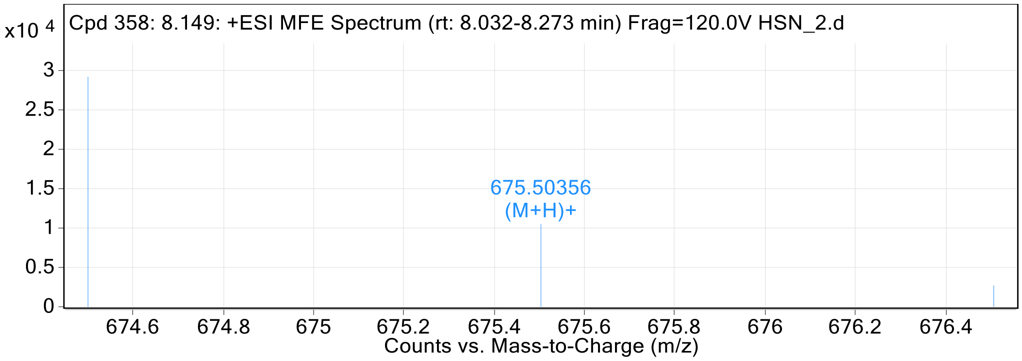

Peak List

m/z	z	Abund	Ion
674.50051	1	29147.49	(M+H)+
675.50356	1	10497.64	(M+H)+
676.50631	1	2701.75	(M+H)+

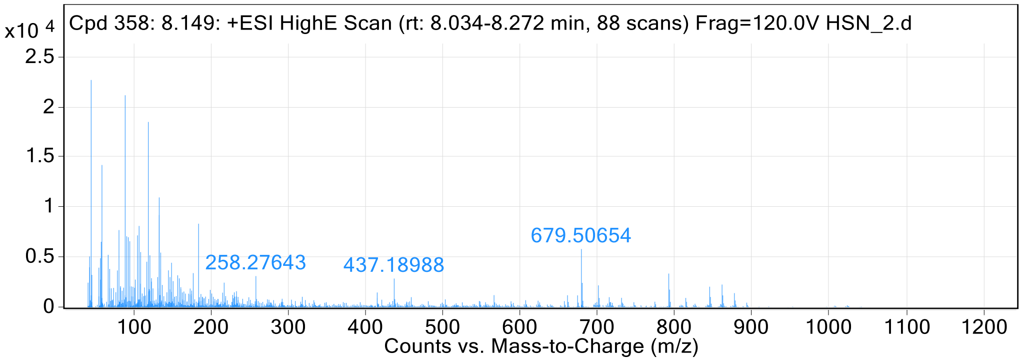

Peak List

m/z	z	Abund
45.03314		22677.7
59.04856		14186.18
81.06874		7686.22
89.05842		21152.67
105.06817		7156.44
107.08378		8098.25
119.08355	1	18485.84
133.08477		9184.58
133.09796	1	10946.17
184.07091		8321.4

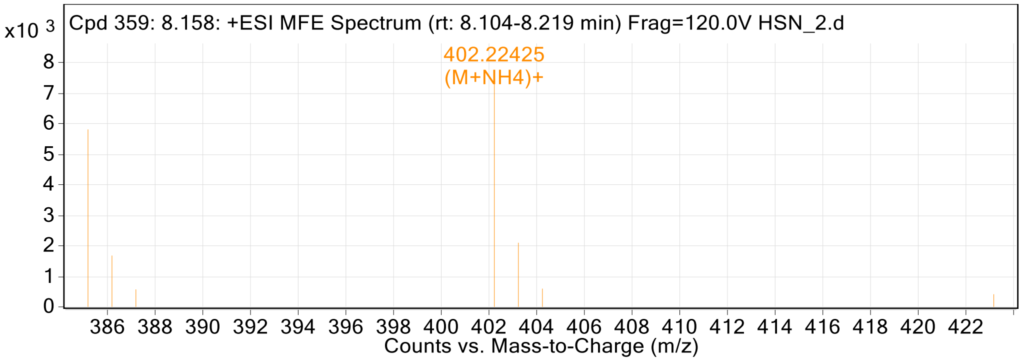

Peak List

m/z	z	Abund	Ion
385.19785	1	5811.13	(M+H)+
386.20265	1	1680.22	(M+H)+
387.20539	1	572.16	(M+H)+
402.22425	1	7490.58	(M+NH4)+
403.23222	1	2100.4	(M+NH4)+
404.23935	1	597.21	(M+NH4)+
423.15174	1	413.92	(M+K)+

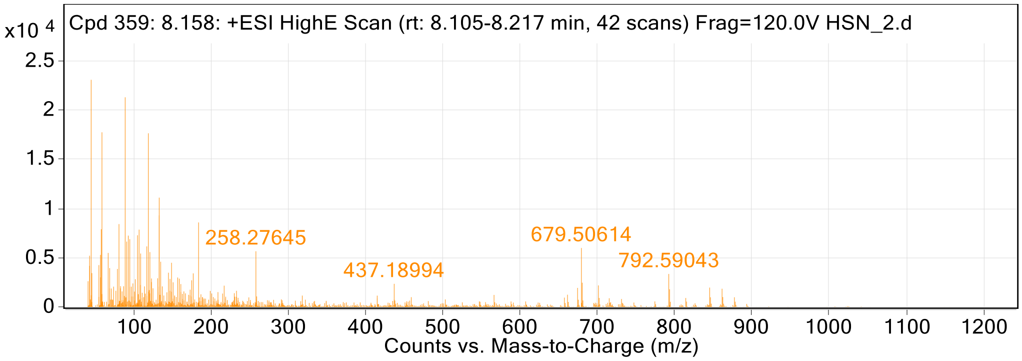

Peak List

m/z	z	Abund
45.03312		23093.48
58.06453		7892.79
59.04857		17754.32
81.06873		8417.42
89.0584		21315.87
107.08381		7854.2
119.08356	1	17651.97
133.08479		9298.78
133.09781	1	11108.3
184.07091		8586.08

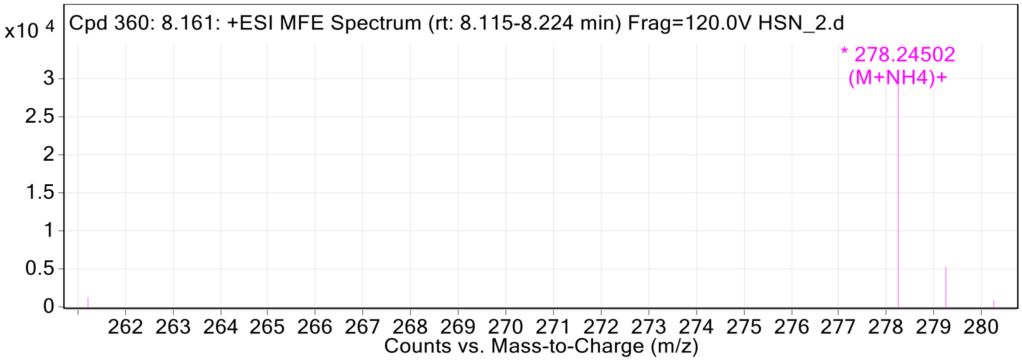

Peak List

m/z	z	Abund	Ion
261.21763	1	1200.69	(M+H)+
278.24502	1	29919.4	(M+NH4)+
279.24804	1	5282.44	(M+NH4)+
280.25434	1	863.72	(M+NH4)+

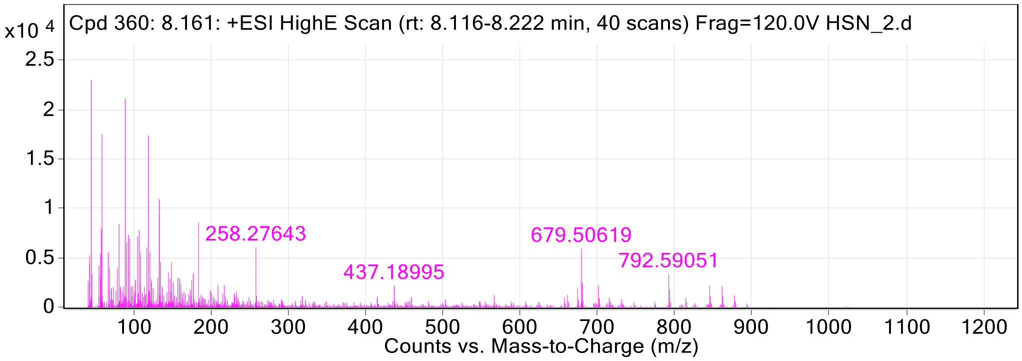

Peak List

m/z	z	Abund
45.03313		23007.57
58.06453		7931.45
59.04856		17514.49
81.06873		8396.42
89.0584		21087.99
107.08381		7804.05
119.08356	1	17410.68
133.08481		9343.27
133.09787	1	10938.28
184.07089		8585.57

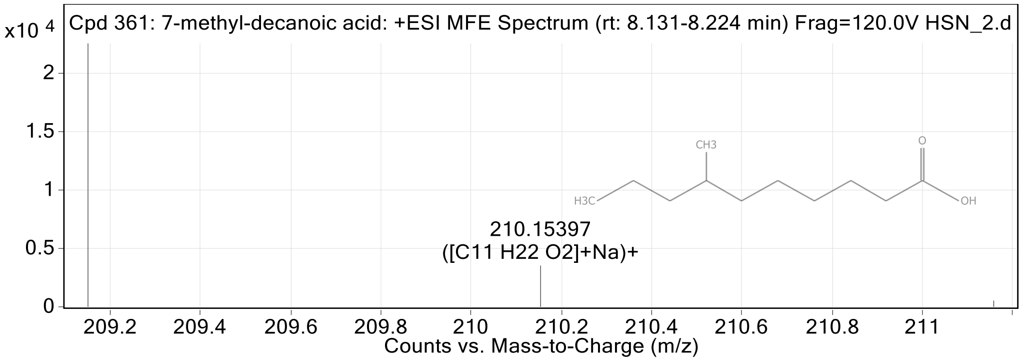

Peak List

m/z	z	Abund	Name	Formula	Ion	Score (DB)	Hits (DB)
209.15101	1	22494.44	7-methyl-decanoic acid	C11 H22 O2	(M+Na)+	95.77	10

210.15397	1	3521.96		C11 H22 O2	(M+Na)+		
211.15838	1	535.48		C11 H22 O2	(M+Na)+		

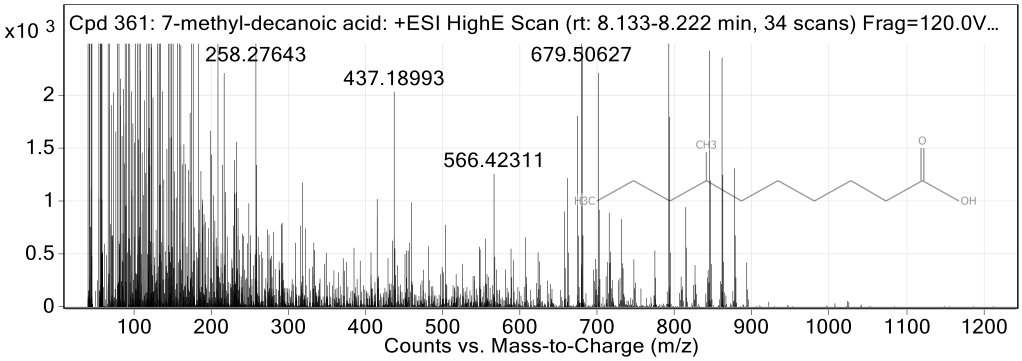

Peak List

m/z	z	Abund
45.03312		22862.93
58.06453		8344.15
59.04857		17324.04
81.06874		8404.32
89.05841		20883.41
107.08382		7895.01
119.08357	1	17702.88
133.08478		9357.14
133.09784		10989.76
184.0709		8674.54

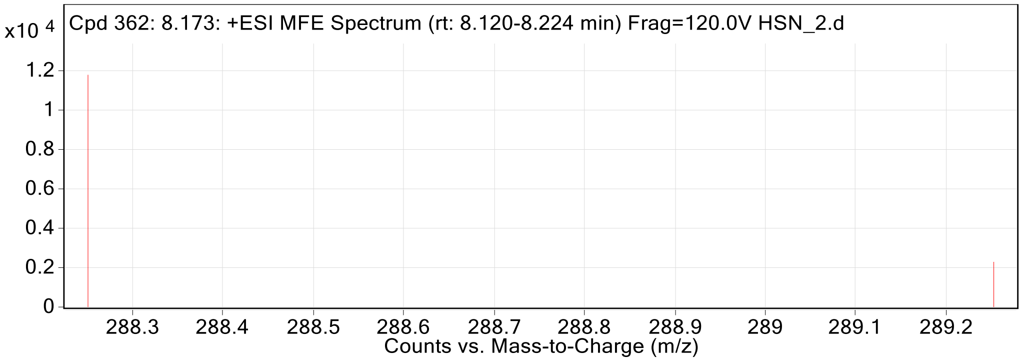

Peak List

m/z	z	Abund	Ion
288.2505	1	11791.63	(M+H)+
289.25272	1	2292.83	(M+H)+

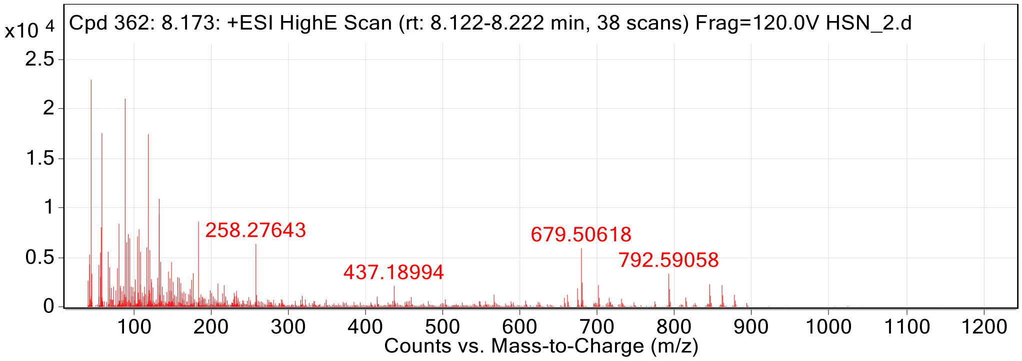

Peak List

m/z	z	Abund
45.03313		22961.17

58.06453		8024.82
59.04855		17558.19
81.06873		8406
89.0584		21069.06
107.08383		7837.22
119.08356	1	17469.88
133.08479		9373.35
133.09785		10920.76
184.07089		8611.35

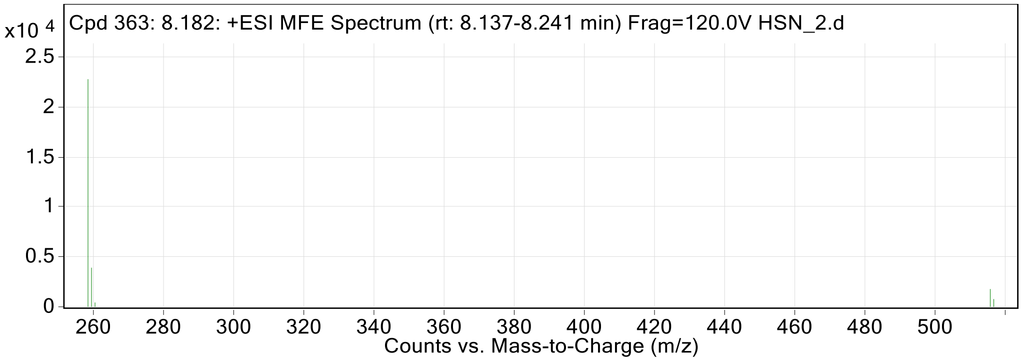

Peak List

m/z	z	Abund	Ion
258.27622	1	22783.51	(M+H)+
259.27962	1	3916.36	(M+H)+
260.28183	1	433.51	(M+H)+
515.54801	1	1779.49	(2M+H)+
516.55046	1	776.48	(2M+H)+

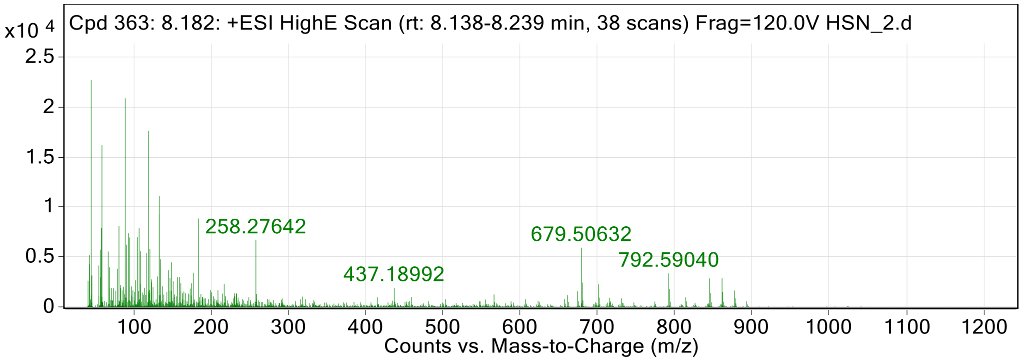

Peak List

m/z	z	Abund
45.03313		22709.71
58.06451		7912.95
59.04856		16167.95
81.06875		8079.73
89.05842		20870.38
107.08385		7871.08
119.08357	1	17598.04
133.08479		9251.62
133.09791		11069.93
184.0709		8840.05

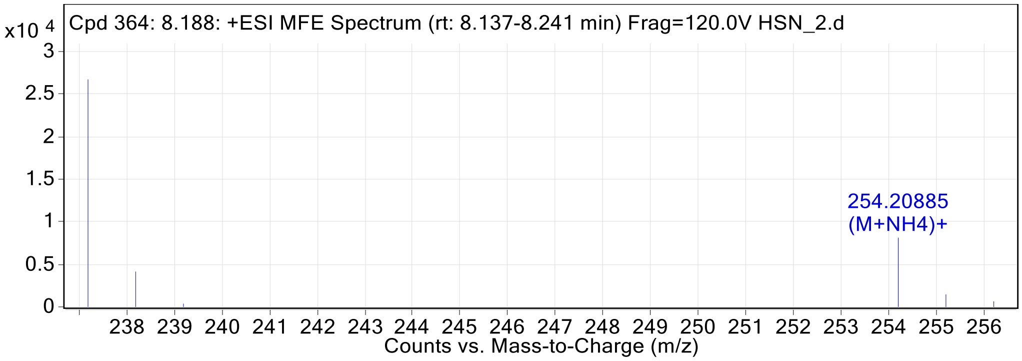

Peak List

m/z	z	Abund	Ion
237.18223	1	26741.46	(M+H)+
238.18519	1	4151.33	(M+H)+
239.18874	1	376.95	(M+H)+
254.20885	1	8135.47	(M+NH4)+
255.21101	1	1468.28	(M+NH4)+
256.21659	1	644.14	(M+NH4)+

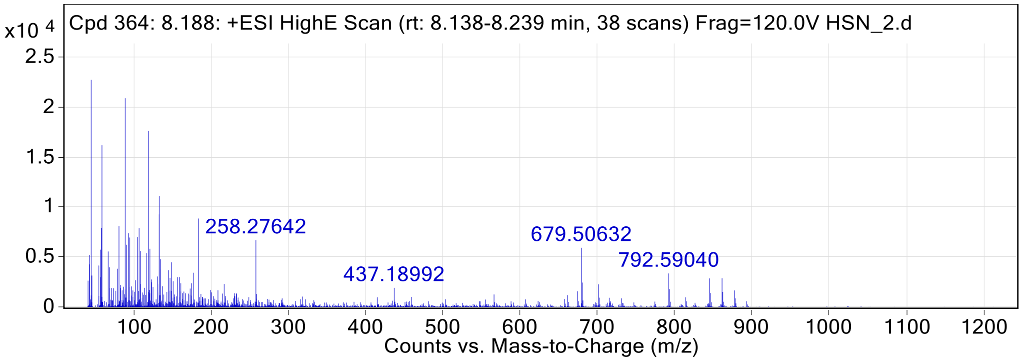

Peak List

m/z	z	Abund
45.03313		22709.71
58.06451		7912.95
59.04856		16167.95
81.06875		8079.73
89.05842		20870.38
107.08385		7871.08
119.08357	1	17598.04
133.08479		9251.62
133.09791		11069.93
184.0709		8840.05

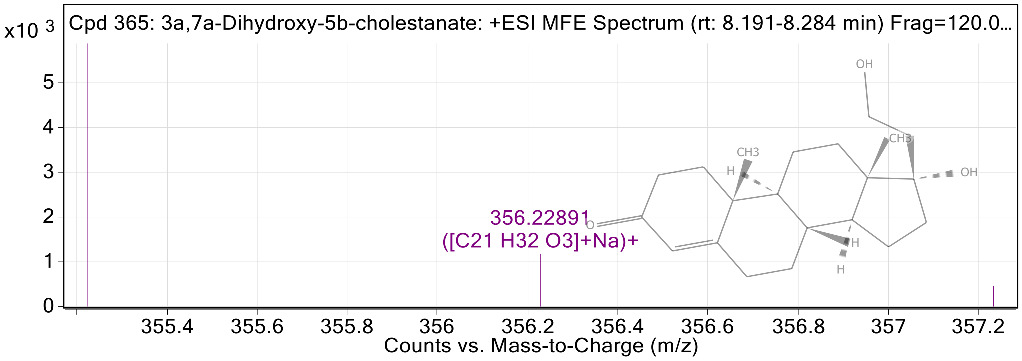

Peak List

Qualitative Analysis Report

m/z	z	Abund	Name	Formula	Ion	Score (DB)	Hits (DB)
355.22435	1	5885.12	3a,7a-Dihydroxy-5b-cholestanate	C21 H32 O3	(M+Na)+	86.75	10
356.22891	1	1171.14		C21 H32 O3	(M+Na)+		
357.2336	1	464.27		C21 H32 O3	(M+Na)+		

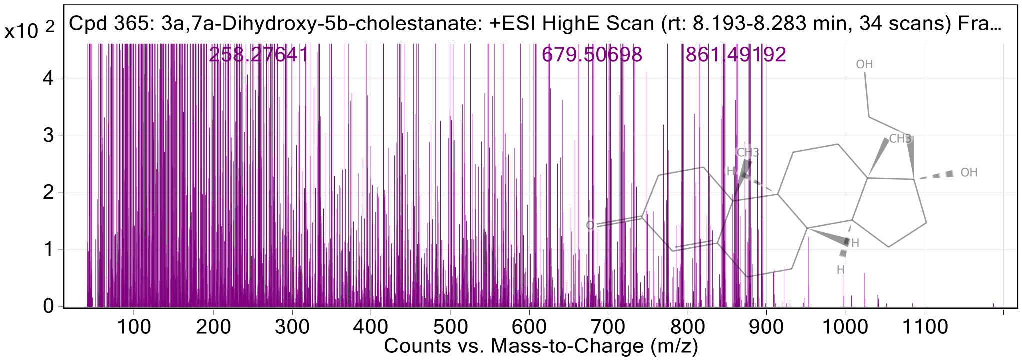

Peak List

m/z	z	Abund
45.03318		21394.1
59.04857		11169.78
89.05847		20348.35
93.06855		7471.79
105.06824		7002.77
107.08381		7547.53
119.08357	1	13921.02
133.0849		8851.63
133.09807		10394.18
184.0709		9579.22

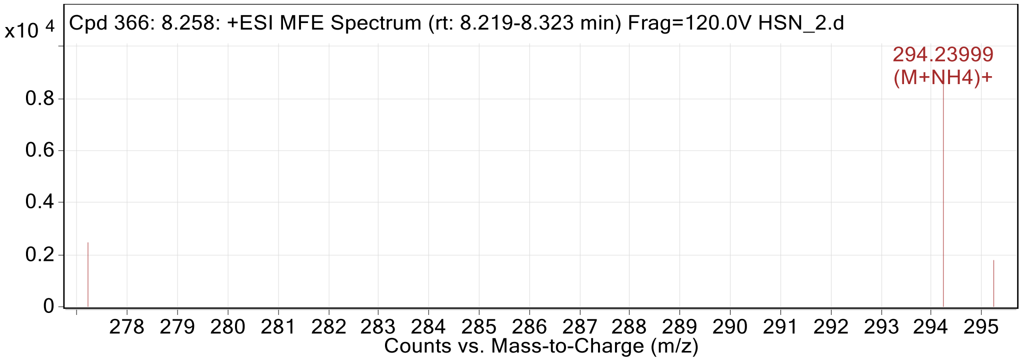

Peak List

m/z	z	Abund	Ion
277.21448	1	2475.81	(M+H)+
294.23999	1	8931.92	(M+NH4)+
295.24256	1	1795.02	(M+NH4)+

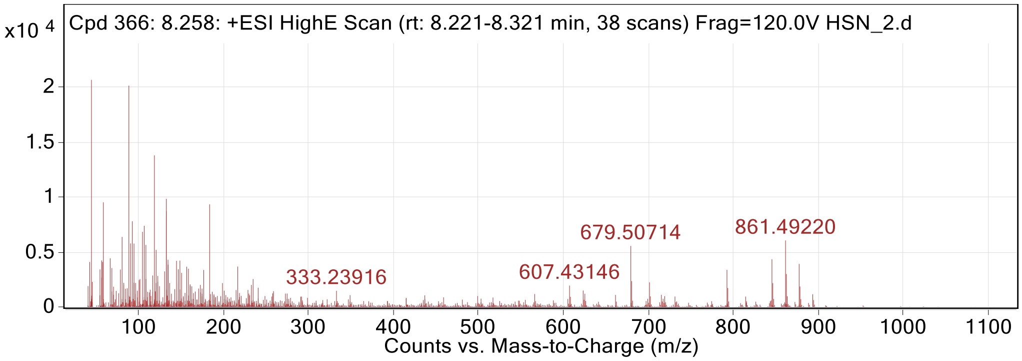

Peak List

m/z	z	Abund
45.03317		20696.49
59.04861		9529.59
89.05847		20168.85
93.06849		7805.43
105.06821		6839.45
107.08381		7390.63
119.08359	1	13814.09
133.08489		8731.22
133.09822		9850.66
184.07089		9347.3

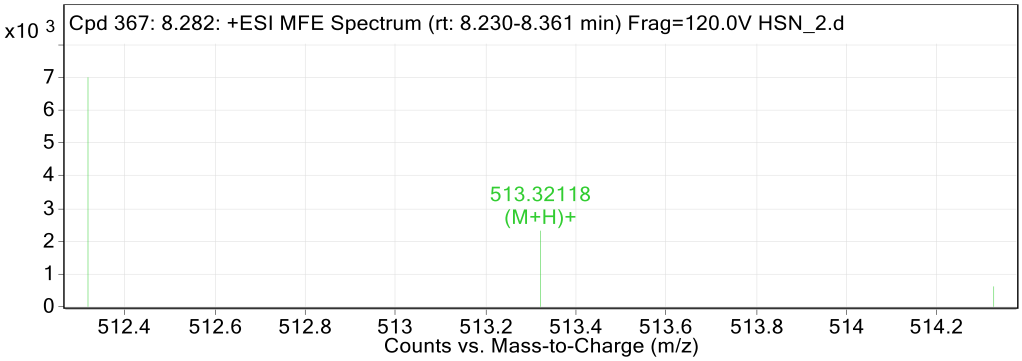

Peak List

m/z	z	Abund	Ion
512.31822	1	7009.42	(M+H)+
513.32118	1	2324.22	(M+H)+
514.32571	1	621.99	(M+H)+

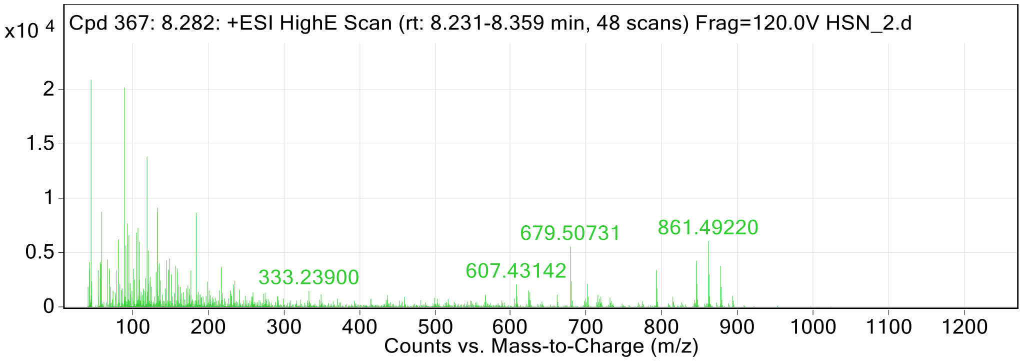

Peak List

m/z	z	Abund
45.03317		20852.62
59.04859		8731.9

89.05849		20159.98
93.06847		7675.55
105.06819		6810.55
107.08387		7250.11
119.0836	1	13789.08
133.08502		8653.88
133.09818		9100.79
184.07088		8628.45

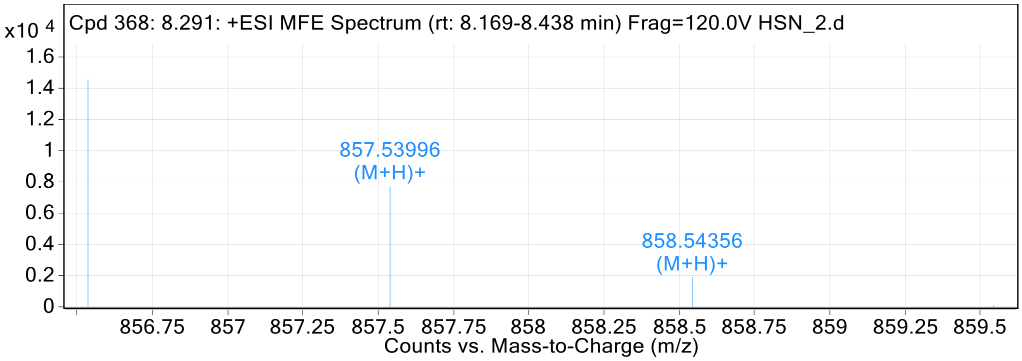

Peak List

m/z	z	Abund	Ion
856.53687	1	14578.64	(M+H)+
857.53996	1	7745.41	(M+H)+
858.54356	1	1901.09	(M+H)+
859.5445	1	113.09	(M+H)+

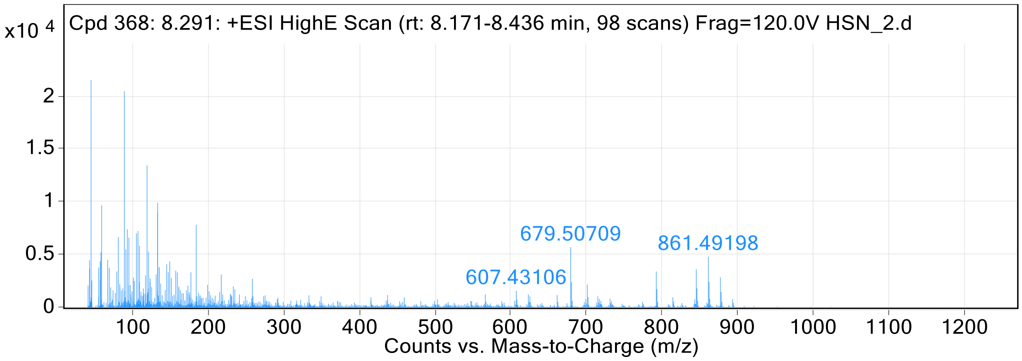

Peak List

m/z	z	Abund
45.03316		21510.56
59.04858		9627.1
89.05847		20443.35
93.06852		7355.81
105.06822		6977.99
107.08385		7204.21
119.08358	1	13414.17
133.08492		8914.11
133.09802		9891.46
184.07092		7786.68

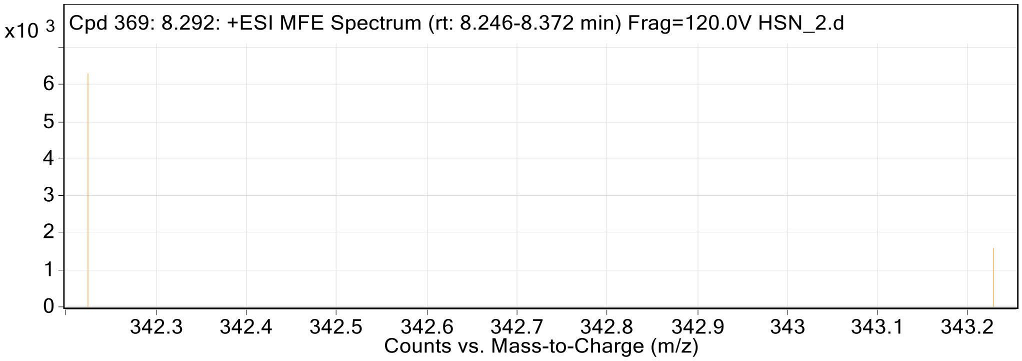

Peak List

m/z	z	Abund	Ion
342.22451	1	6299.53	(M+H)+
343.22864	1	1590.32	(M+H)+

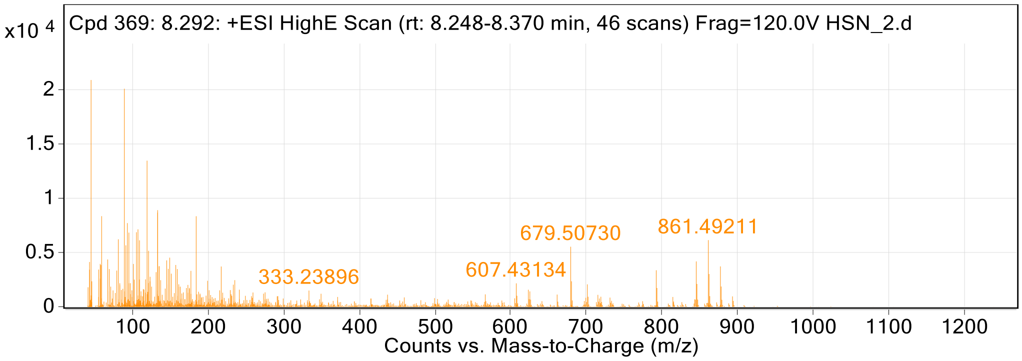

Peak List

m/z	z	Abund
45.03317		20878.28
59.04859		8340.09
89.05847		20071.93
93.06847		7700
105.0682		6859.79
107.08384		7126.59
119.08358	1	13449.69
133.08498		8665.79
133.09816		8891.78
184.07091		8334.25

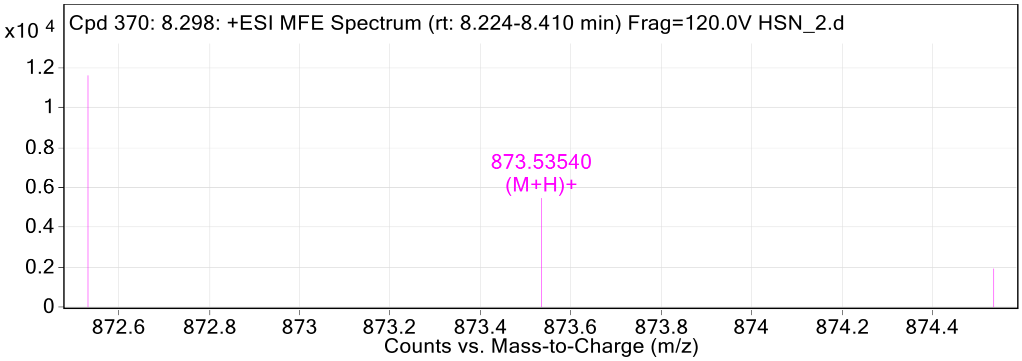

Peak List

m/z	z	Abund	Ion
872.53175	1	11618.58	(M+H)+
873.5354	1	5454.14	(M+H)+
874.53632	1	1924.96	(M+H)+

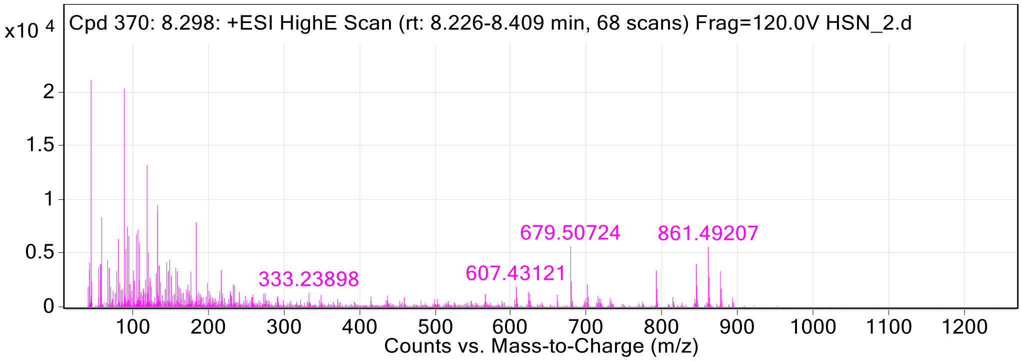

Peak List

m/z	z	Abund
45.03316		21121.07
59.04861		8360.71
89.05848		20305.36
93.0685		7434.25
105.06822		6786.95
107.08385		7181.26
119.08357	1	13188.52
133.08495		8775.06
133.09815		9491.6
184.07093		7840.26

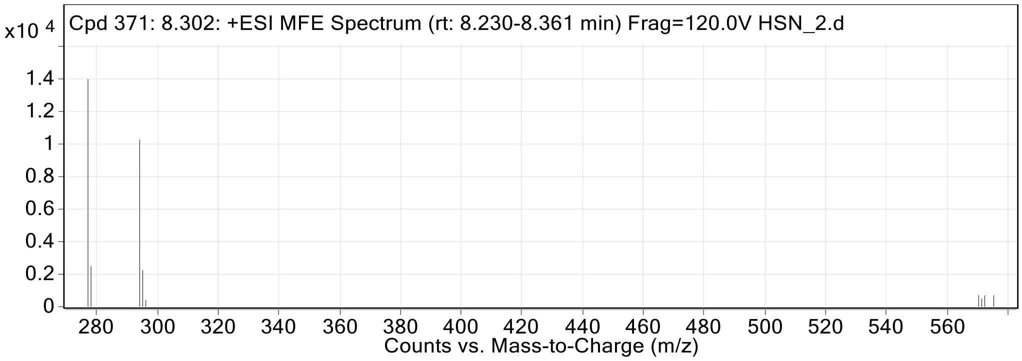

Peak List

m/z	z	Abund	Ion
277.17745	1	13959.13	(M+H)+
278.18057	1	2487.85	(M+H)+
294.20367	1	10254.57	(M+NH4)+
295.20725	1	2237.08	(M+NH4)+
296.2155	1	412.38	(M+NH4)+
570.37276	1	704.99	(2M+NH4)+
571.38363	1	506.52	(2M+NH4)+
572.36201	1	688.25	(2M+NH4)+
575.33481	1	693.27	(2M+Na)+

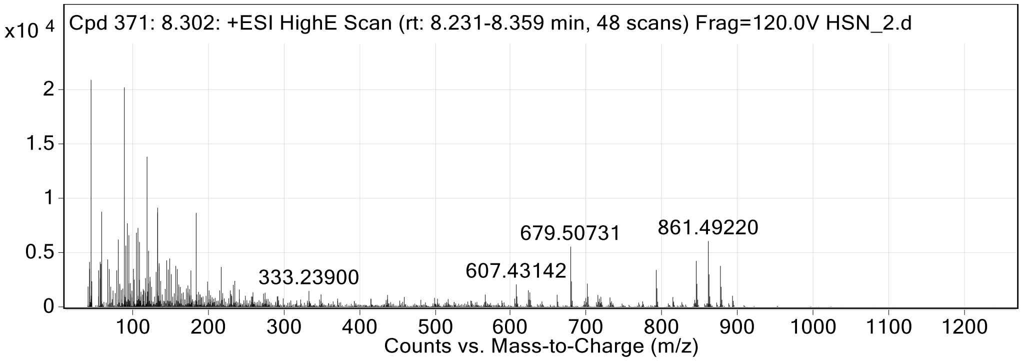

Peak List

m/z	z	Abund
45.03317		20852.62
59.04859		8731.9
89.05849		20159.98
93.06847		7675.55
105.06819		6810.55
107.08387		7250.11
119.0836	1	13789.08
133.08502		8653.88
133.09818		9100.79
184.07088		8628.45

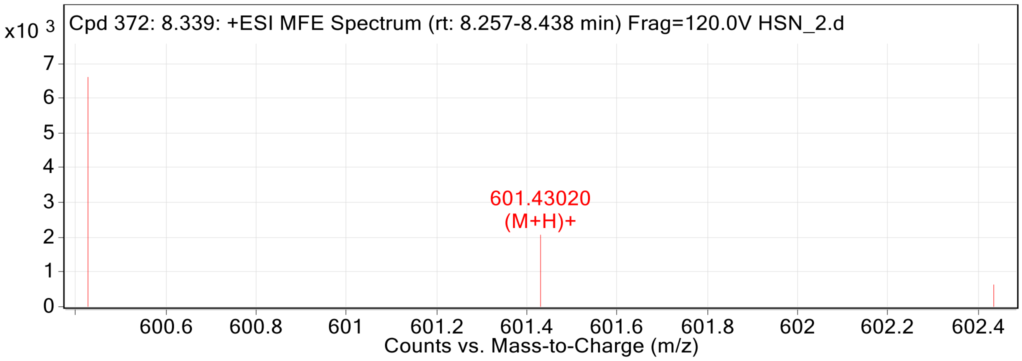

Peak List

m/z	z	Abund	Ion
600.42741	1	6609.77	(M+H)+
601.4302	1	2071.56	(M+H)+
602.4344	1	638.25	(M+H)+

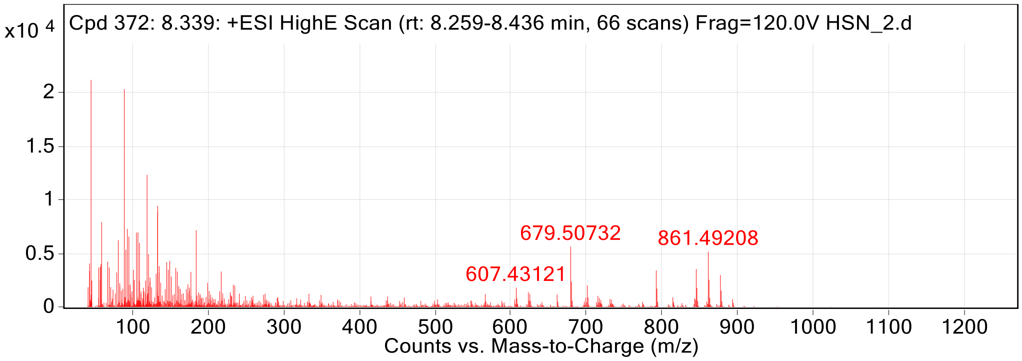

Peak List

m/z	z	Abund
45.03316		21168.22
59.04858		7898.74

89.05848		20303.16
93.0685		7276.44
105.06823		6921.6
107.08385		6948.9
119.08358	1	12310.94
133.08492		8834.26
133.09807		9422.87
184.07096		7145.81

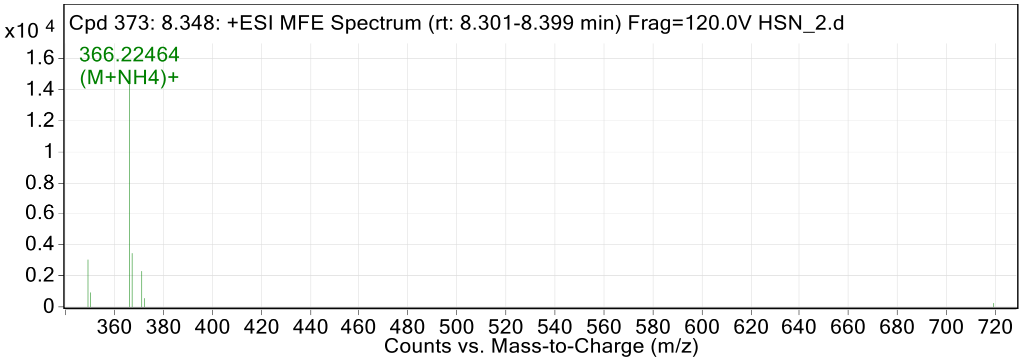

Peak List

m/z	z	Abund	Ion
349.19602	1	3045.47	(M+H)+
350.20112	1	925.95	(M+H)+
366.22464	1	14662.7	(M+NH4)+
367.22841	1	3452.25	(M+NH4)+
371.18026	1	2305.02	(M+Na)+
372.1845	1	556.99	(M+Na)+
719.36721	1	244.07	(2M+Na)+

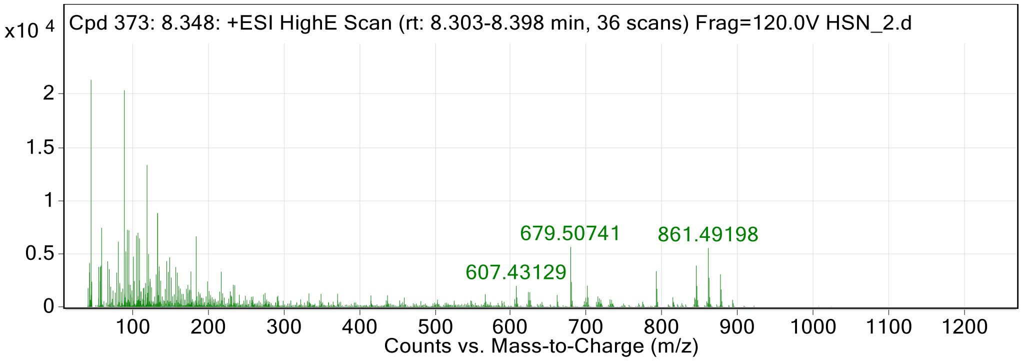

Peak List

m/z	z	Abund
45.03316		21343.27
59.04862		7426.43
89.05847		20360.34
93.06849		7243.25
95.08412		7196.01
105.06823		6706.88
107.08388		6954.63
119.08355	1	13328.44
133.08505		8753.53
133.09814		8825.38

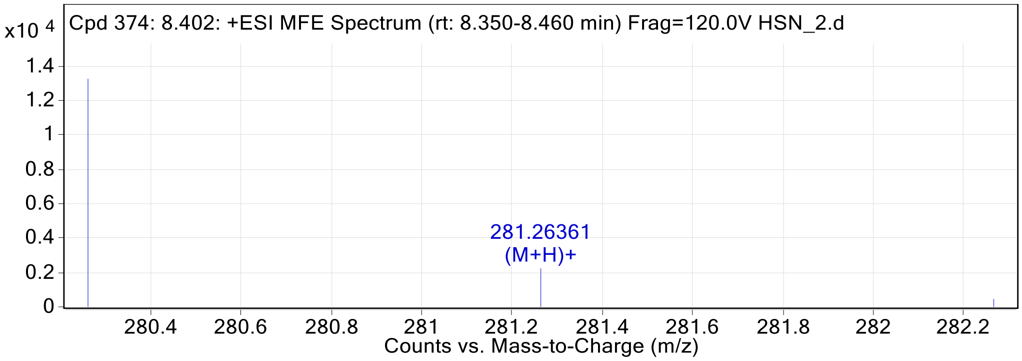

Peak List

m/z	z	Abund	Ion
280.26046	1	13259.62	(M+H)+
281.26361	1	2238.06	(M+H)+
282.26697	1	459.31	(M+H)+

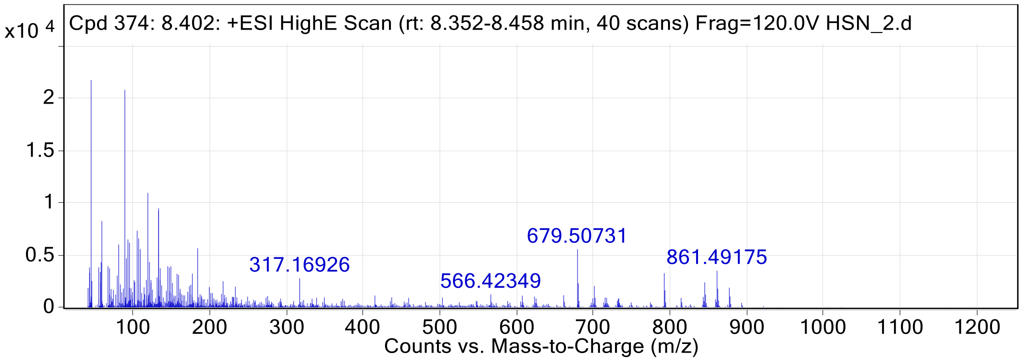

Peak List

m/z	z	Abund
45.03314		21737.93
59.04857		8239.01
89.05847		20799.84
93.06856		6471.13
95.08408		6164.17
105.06826		7310.22
107.08386		6575.18
119.08361	1	10934.8
133.08491		9223.88
133.0979		9458.07

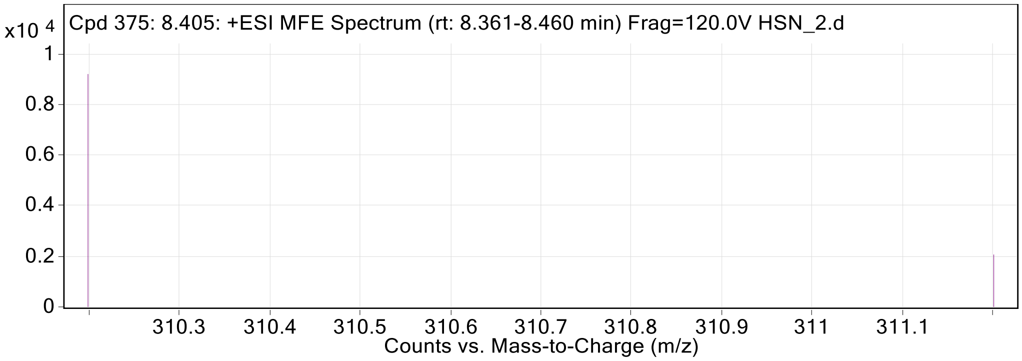

Peak List

m/z	z	Abund	Ion
310.19856	1	9199.63	(M+H)+
311.2012	1	2068.12	(M+H)+

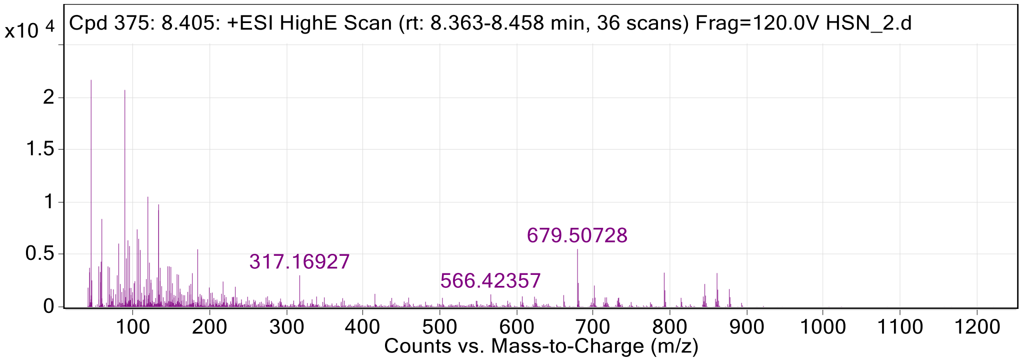

Peak List

m/z	z	Abund
45.03314		21707.13
59.04857		8403.75
81.06885		6051.57
89.05847		20741.78
93.06857		6362.09
105.06827		7410.85
107.08386		6510.69
119.08362	1	10534.39
133.08485		9245.58
133.09788	1	9801.69

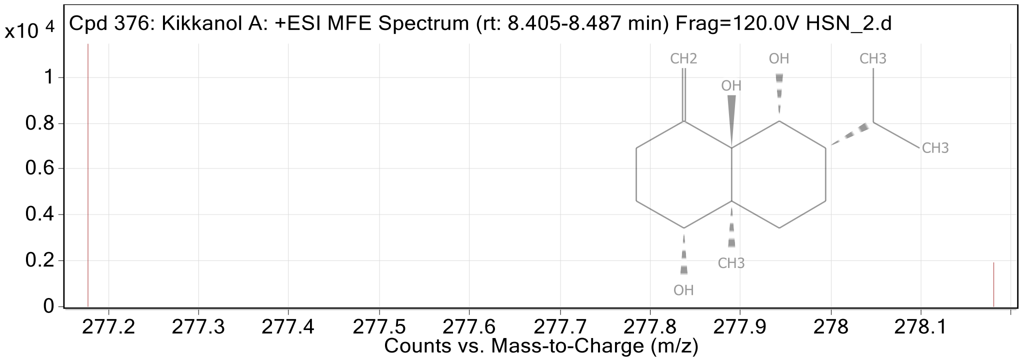

Peak List

m/z	z	Abund	Name	Formula	Ion	Score (DB)	Hits (DB)
277.17742	1	11466.65	Kikkanol A	C15 H26 O3	(M+Na)+	86.44	10
278.18042	1	1934.97		C15 H26 O3	(M+Na)+		

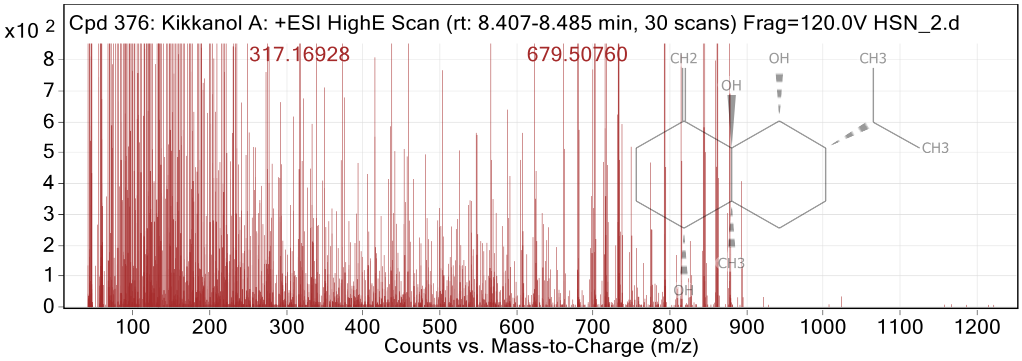

Peak List

m/z	z	Abund
45.03315		21944.94
59.04852		12163.88
89.05846		21002.85

93.06851		5814.3
105.06821		8133.23
107.08392		6109.47
119.08372		9141.82
133.08483		9394.24
133.09774	1	9302.55
317.16928		5543.86

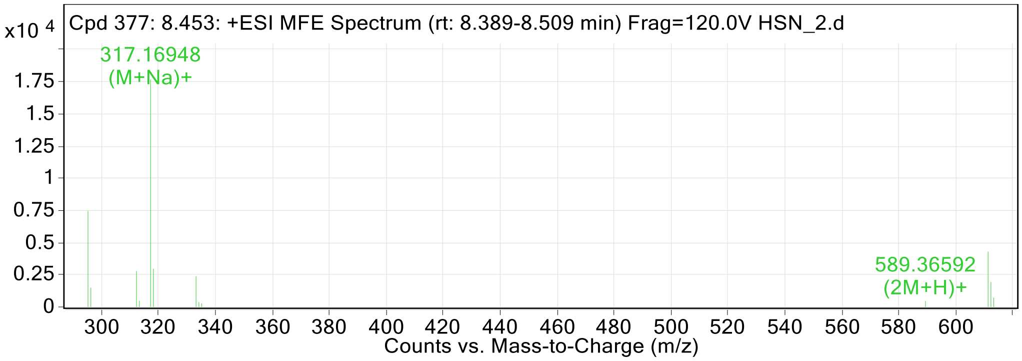

Peak List

m/z	z	Abund	Ion
295.18781	1	7451.77	(M+H)+
296.1924	1	1498.26	(M+H)+
312.21443	1	2791.23	(M+NH4)+
313.22241	1	469.36	(M+NH4)+
317.16948	1	17675.97	(M+Na)+
318.17322	1	2963.84	(M+Na)+
333.14368	1	2393.18	(M+K)+
611.35168	1	4298.98	(2M+Na)+
612.3536	1	1935.28	(2M+Na)+
613.35301	1	719.78	(2M+Na)+

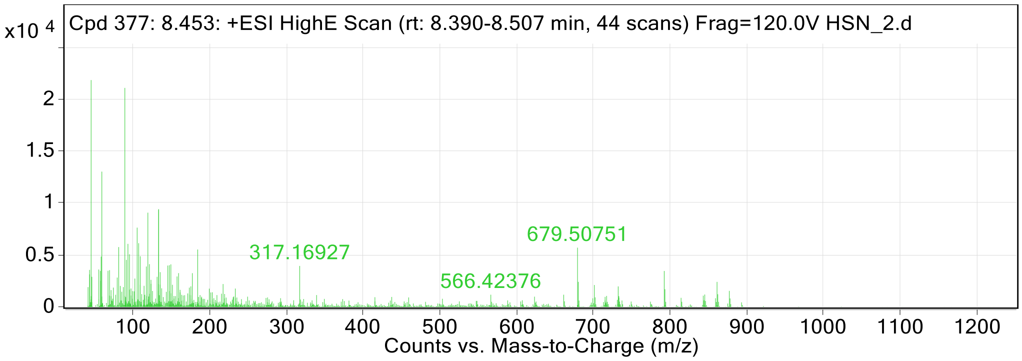

Peak List

m/z	z	Abund
45.03316		21829.47
59.04855		13014.5
81.06885		5749.99
89.05846		21073.43
93.0685		6058.98
105.06823		7621.68
107.08386		6124.63
119.0837		9061.22
133.0849		9325.44
133.09781	1	9404.21

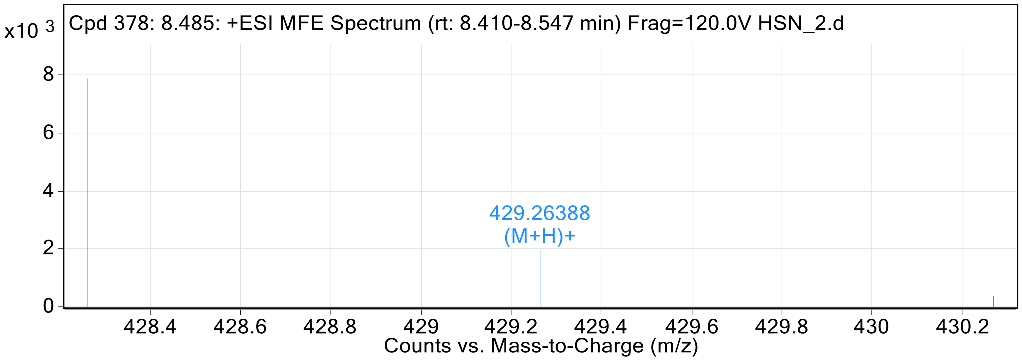

Peak List

m/z	z	Abund	Ion
428.26134	1	7876.57	(M+H)+
429.26388	1	1974.47	(M+H)+
430.26879	1	361.94	(M+H)+

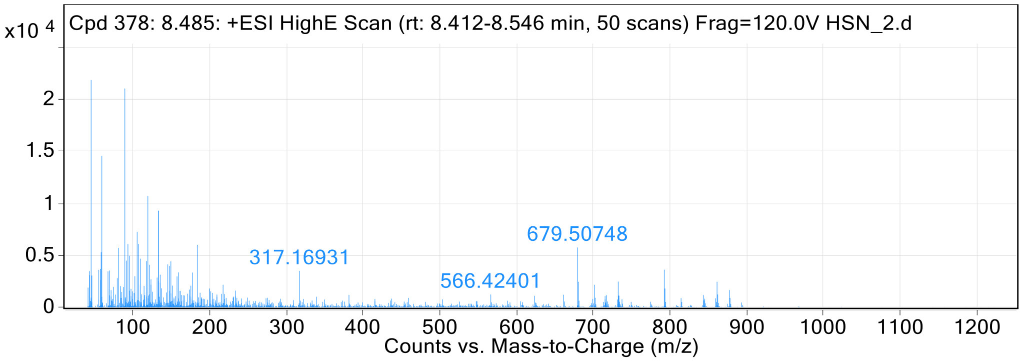

Peak List

m/z	z	Abund
45.03317		21888.88
59.04854		14566.45
89.05846		21070.13
93.06852		6071.15
105.06824		7242.79
107.0839		6090.77
119.08369	1	10691.19
133.08482		9289.74
133.09787		9291.84
184.07089		5998.92

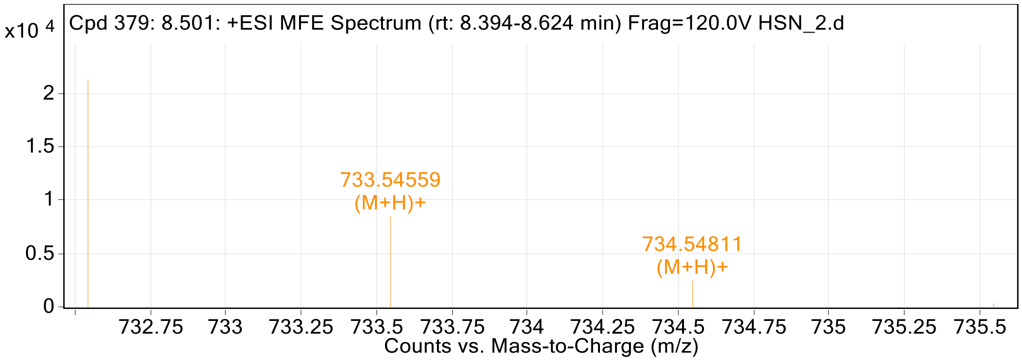

Peak List

m/z	z	Abund	Ion
732.54242	1	21303.42	(M+H)+
733.54559	1	8489.57	(M+H)+

734.54811	1	2467.22	(M+H)+
735.54722	1	330.73	(M+H)+

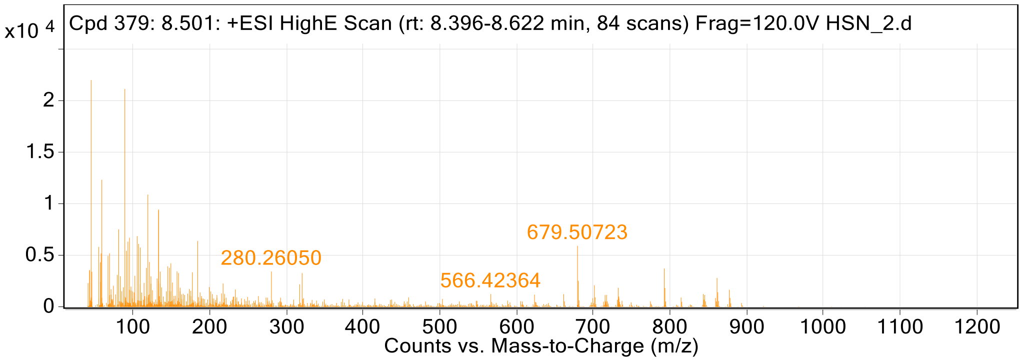

Peak List

m/z	z	Abund
45.03316		21985.14
59.04855		12321.05
81.06885		7508.49
89.05847		21121.79
95.08407		6699.67
105.06827		6848.96
119.08366	1	10883.24
133.08489		9344.98
133.09789		9434.14
184.07093		6380.41

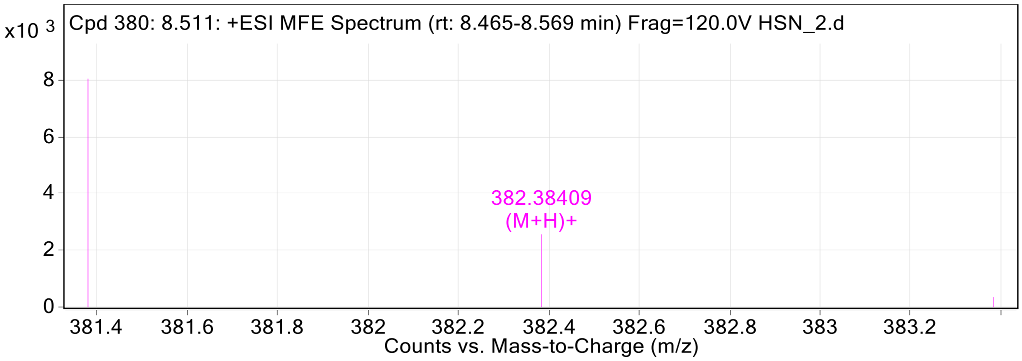

Peak List

m/z	z	Abund	Ion
381.38096	1	8047.54	(M+H)+
382.38409	1	2558.54	(M+H)+
383.38419	1	352.02	(M+H)+

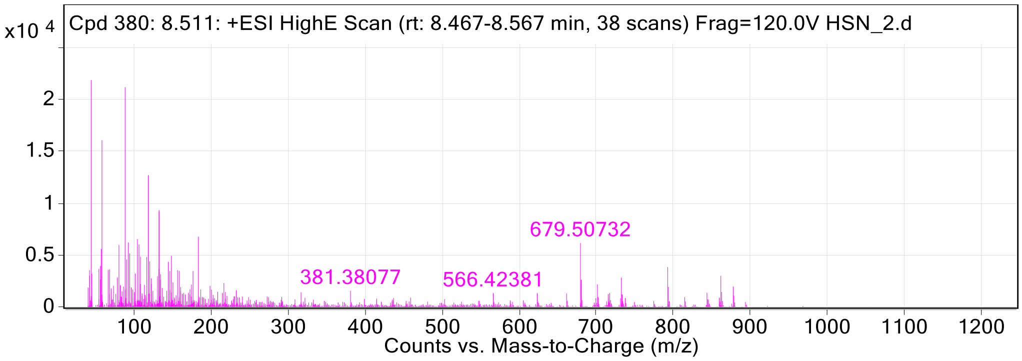

Peak List

m/z	z	Abund

45.03319		21880.97
59.04854		16082.25
89.05845		21192.19
93.06854		6215.99
105.06829		6533.35
119.08367	1	12699.16
133.08488		9330.47
133.09803		9196.46
184.07087		6760.98
679.50732	1	6158.7

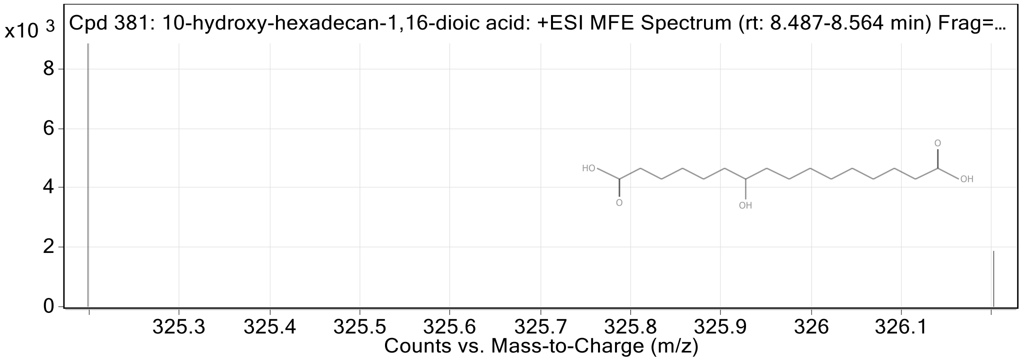

Peak List

m/z	z	Abund	Name	Formula	Ion	Score (DB)	Hits (DB)
325.1985	1	8852.66	10-hydroxy-hexadecan-1,16-dioic acid	C16 H30 O5	(M+Na)+	82.55	3
326.20225	1	1877.9		C16 H30 O5	(M+Na)+		

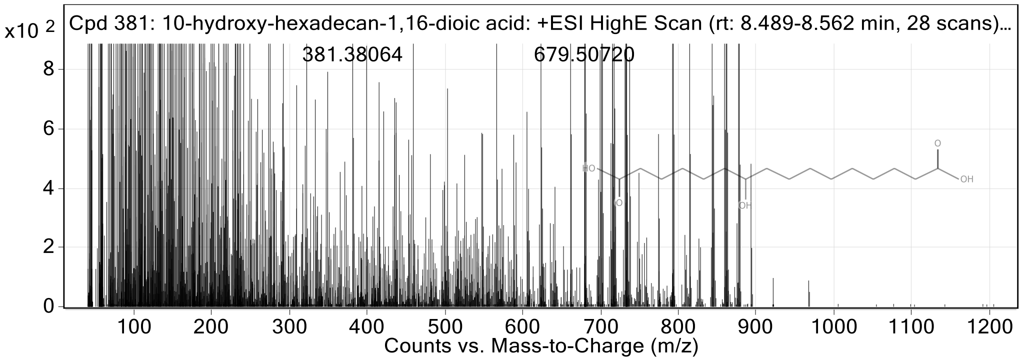

Peak List

m/z	z	Abund
45.03319		21852.87
59.04856		15815.53
81.06884		6211.99
89.05845		21146.78
93.06858		6411.27
119.08365	1	13958.69
133.08496		9268.85
133.09802		9243.72
184.07087		7041.2
679.5072	1	6272.18

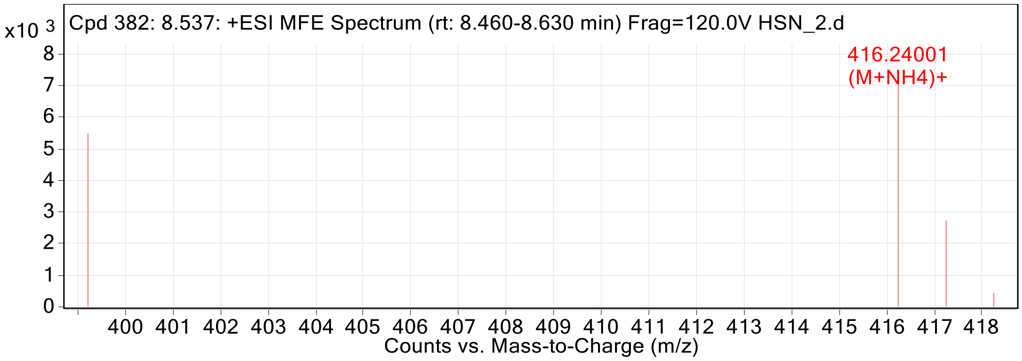

Peak List

m/z	z	Abund	Ion
399.21328	1	5482.26	(M+H)+
416.24001	1	7240.08	(M+NH4)+
417.24678	1	2731.48	(M+NH4)+
418.24817	1	434.97	(M+NH4)+

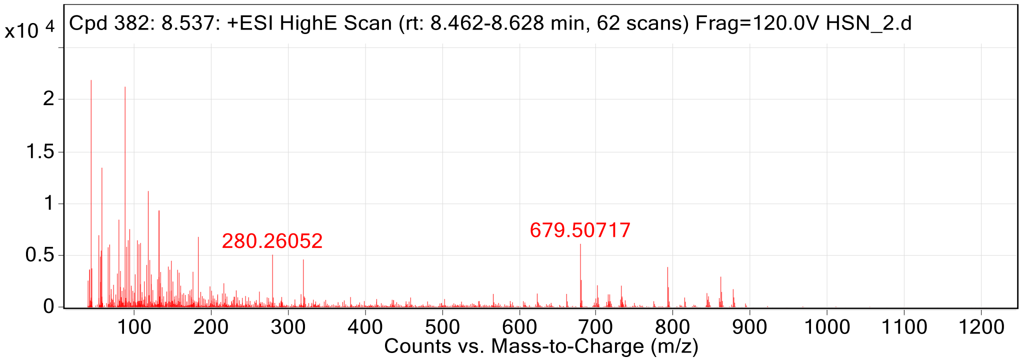

Peak List

m/z	Abund
45.03317	21902.22
55.0537	6928.13
59.04855	13441.44
81.06884	8428.09
89.05845	21254.72
95.08407	7512.54
119.08365	11187.75
133.08487	9271.92
133.09795	9335.06
184.07091	6764.73

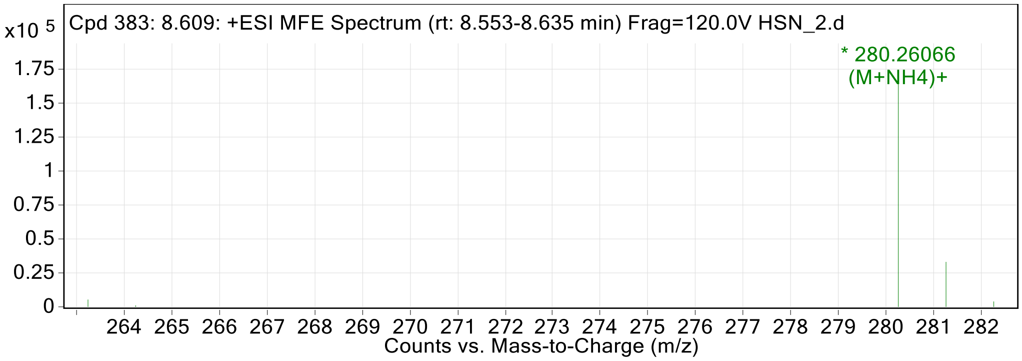

Peak List

m/z	z	Abund	Ion
263.23419	1	5396.4	(M+H)+

264.23653	1	1071.81	(M+H)+
280.26066	1	167766.63	(M+NH4)+
281.26372	1	33180.33	(M+NH4)+
282.26722	1	3977.46	(M+NH4)+

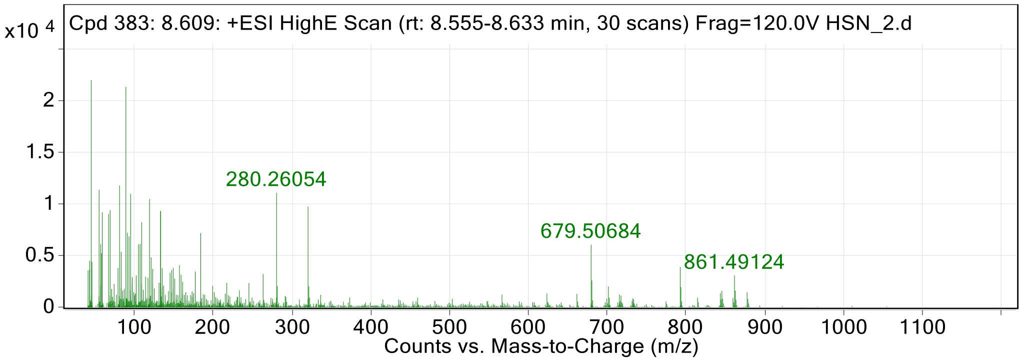

Peak List

m/z	z	Abund
45.03315		21972.86
55.05369		11359.96
69.06902		9388.94
81.06885		11773.34
89.05847		21315.37
95.08405		10964.93
119.08364		10463.52
133.09797		9296.39
280.26054	1	11061.72
320.25306	1	9721.92

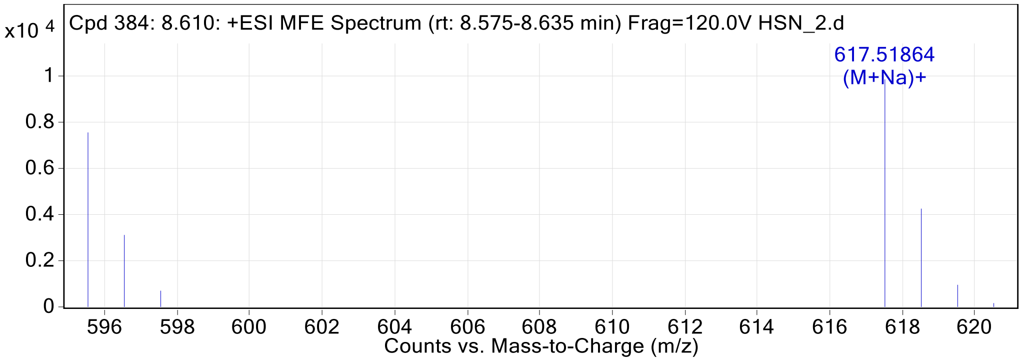

Peak List

m/z	z	Abund	Ion
595.53731	1	7538.18	(M+H)+
596.53969	1	3108.79	(M+H)+
597.54398	1	700.13	(M+H)+
617.51864	1	9831.97	(M+Na)+
618.52114	1	4246	(M+Na)+
619.52541	1	955.12	(M+Na)+
620.52106	1	162.76	(M+Na)+

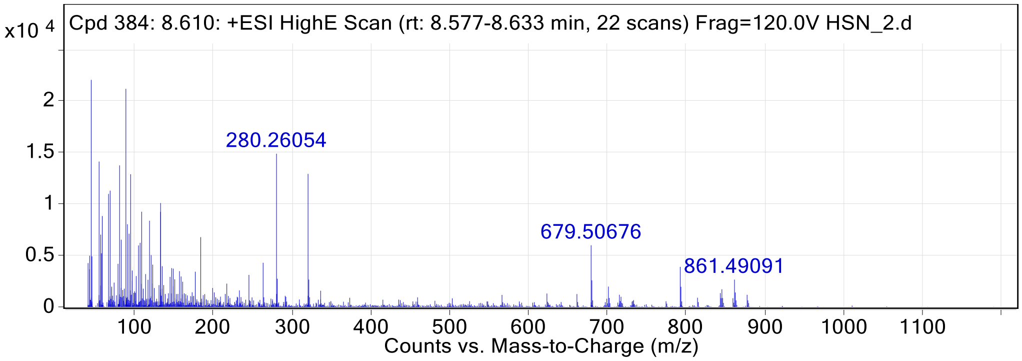

Peak List

m/z	z	Abund
45.03315		22057.66
55.05369		14118.42
67.05347		10973.87
69.06903		11294.81
81.06885		13746.66
89.05847		21182.73
95.08401	1	12888.26
133.09785		10083.92
280.26054	1	14877.4
320.25305	1	12920.39

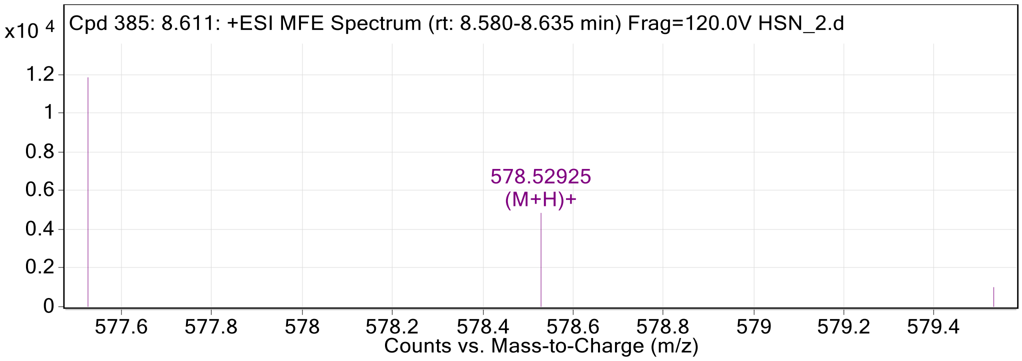

Peak List

m/z	z	Abund	Ion
577.52581	1	11840.64	(M+H)+
578.52925	1	4843.83	(M+H)+
579.53202	1	1012.04	(M+H)+

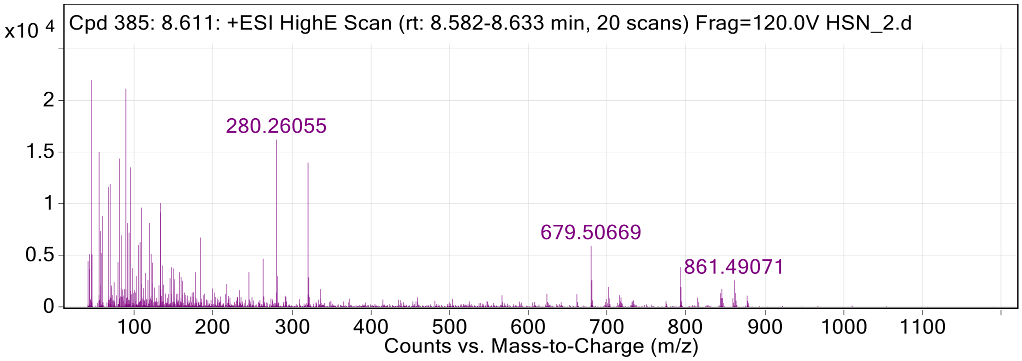

Peak List

m/z	z	Abund
45.03316		21963.91
55.05369		14968.49

67.05347		11582.74
69.06903		11905.48
81.06885		14349.72
89.05845		21116.65
95.08402	1	13491.59
133.09783		10071.87
280.26055	1	16191.23
320.25305	1	13962.67

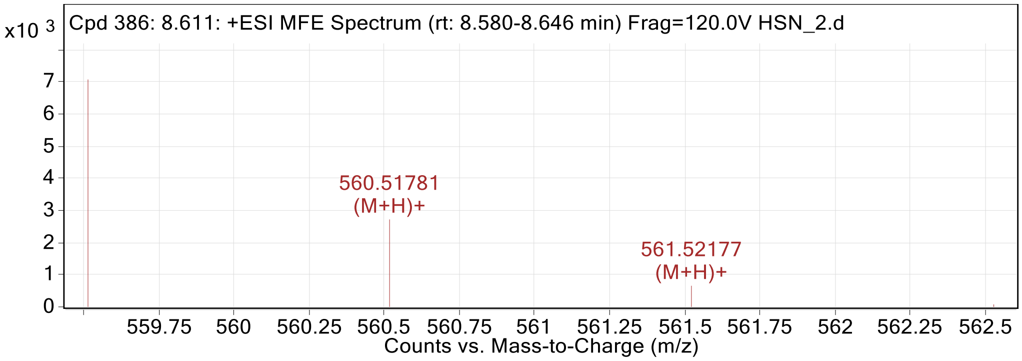

Peak List

m/z	z	Abund	Ion
559.51474	1	7069.45	(M+H)+
560.51781	1	2718.66	(M+H)+
561.52177	1	656.61	(M+H)+
562.52737	1	77.02	(M+H)+

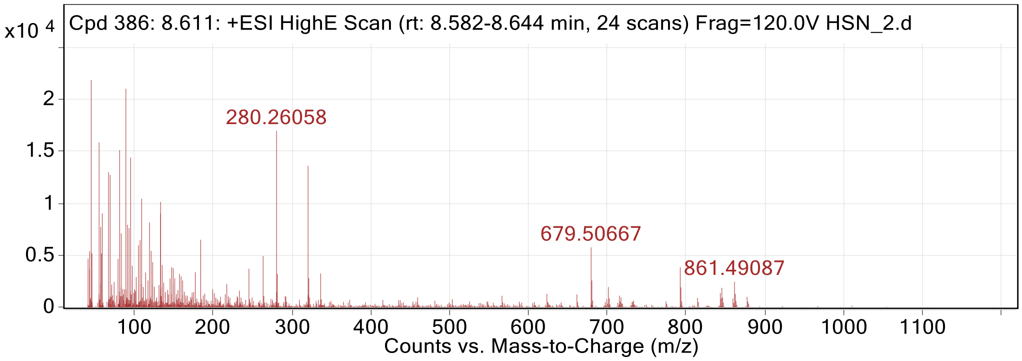

Peak List

m/z	z	Abund
45.03317		21897.93
55.05369		15876.13
67.05341		12987.96
69.06903		12737.84
81.06883		15133.43
89.05843		21045.63
95.08402	1	14420.01
109.09936		10446.05
280.26058	1	16990.7
320.25301	1	13620.61

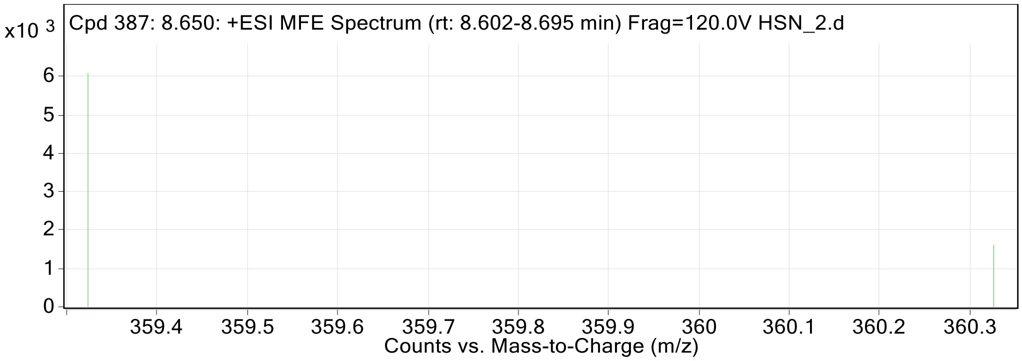

Peak List

m/z	z	Abund	Ion
359.32335	1	6084.21	(M+H)+
360.32656	1	1617.66	(M+H)+

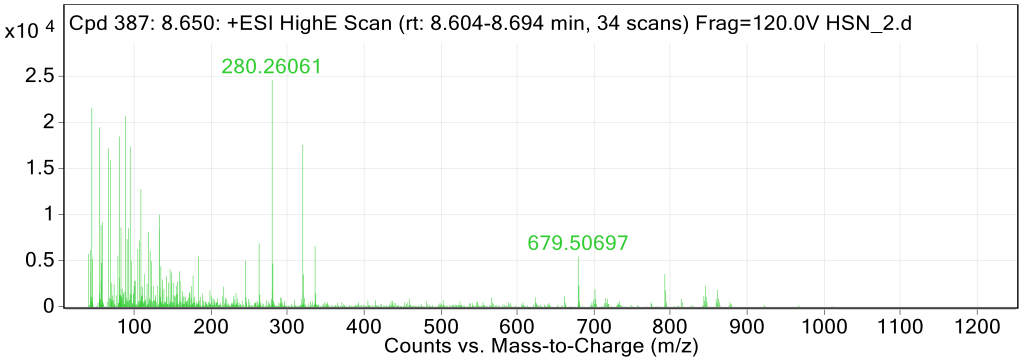

Peak List

m/z	z	Abund
45.03317		21523.04
55.0537		19402.33
67.05342		17140.45
69.06902		15875.98
81.06884		18402.92
89.05844		20620.46
95.0841	1	17321.87
109.09943		12725.49
280.26061	1	24514.04
320.25294	1	17496.58

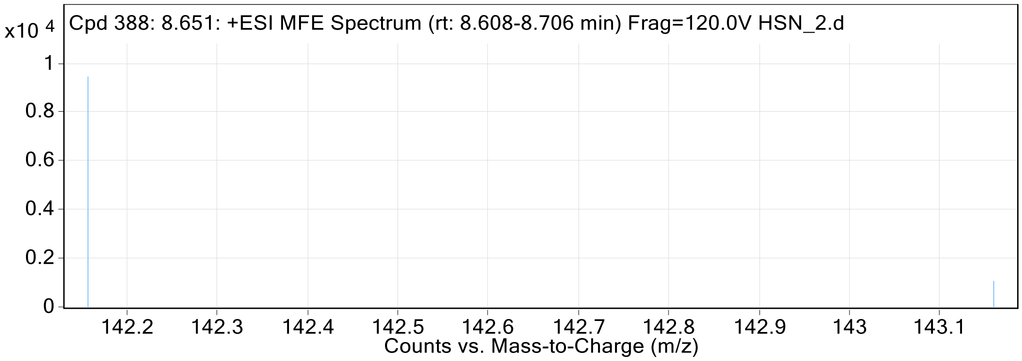

Peak List

m/z	z	Abund	Ion
142.1567	1	9457.6	(M+H)+
143.15996	1	1068.42	(M+H)+

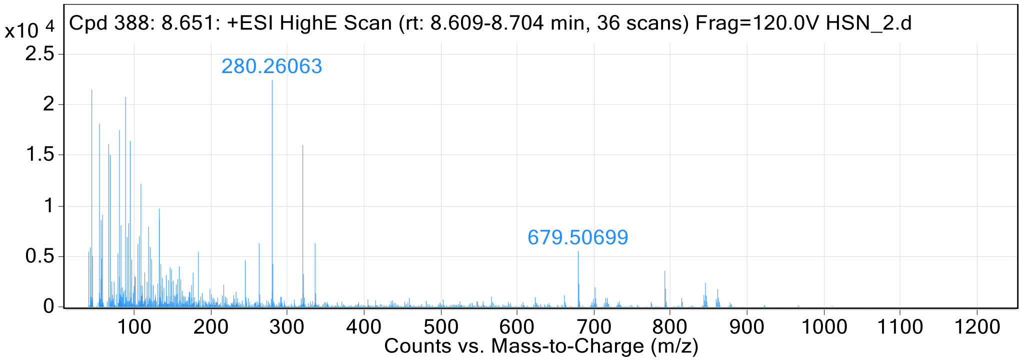

Peak List

m/z	z	Abund
45.03317		21509.39
55.05371		18146.13
67.05343		16116
69.06902		15021.01
81.06883		17510.13
89.05844		20785.08
95.08411		16419.91
109.09945		12179.67
280.26063	1	22465.43
320.25295	1	16017.71

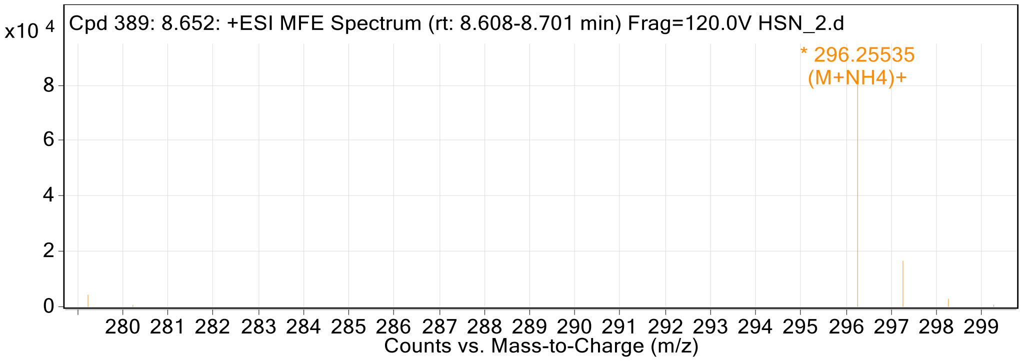

Peak List

m/z	z	Abund	Ion
279.23012	1	4372.56	(M+H)+
280.2233	1	699.45	(M+H)+
296.25535	1	81939.04	(M+NH4)+
297.25798	1	16600.88	(M+NH4)+
298.26311	1	2923.95	(M+NH4)+
299.26661	1	745.92	(M+NH4)+

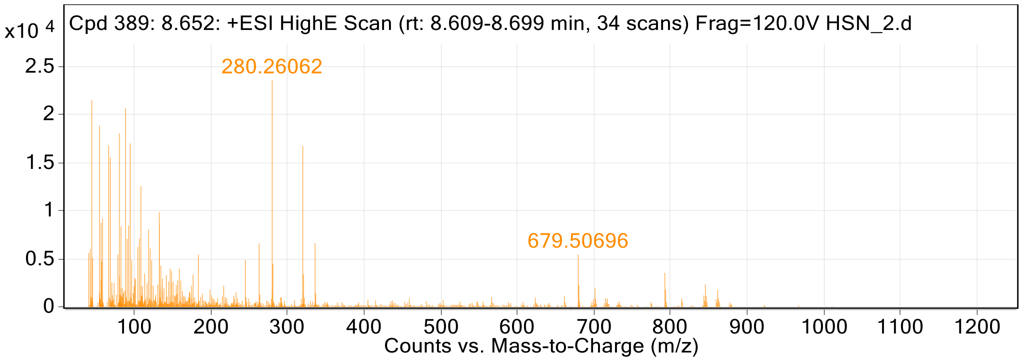

Peak List

m/z	z	Abund
45.03317		21489.15
55.05372		18850.23
67.05343		16780.08
69.06902		15552.96
81.06883		18055.02
89.05844		20651.12
95.08411		17005.28
109.09945		12557.88
280.26062	1	23585.54
320.25294	1	16729.95

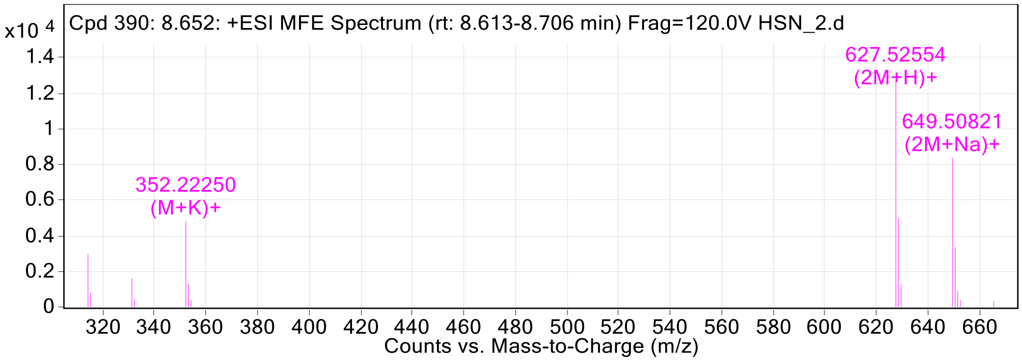

Peak List

m/z	z	Abund	Ion
314.26596	1	2969.43	(M+H)+
331.29338	1	1599.79	(M+NH4)+
352.2225	1	4812.13	(M+K)+
353.2257	1	1283.6	(M+K)+
627.52554	1	12788.21	(2M+H)+
628.52929	1	4992.43	(2M+H)+
629.53154	1	1253.92	(2M+H)+
649.50821	1	8370.23	(2M+Na)+
650.5107	1	3358.98	(2M+Na)+
651.51307	1	916.1	(2M+Na)+

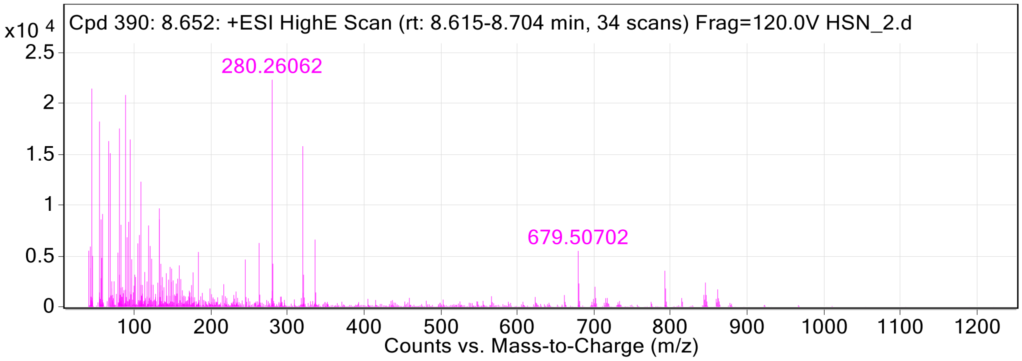

Peak List

m/z	z	Abund
45.03317		21430.19
55.05371		18194.07
67.05342		16271.94
69.06901		15079.04
81.06882		17509.47
89.05844		20802.05
95.08411		16433.45
109.09945		12286.07

280.26062	1	22299.91
320.25292	1	15770.31

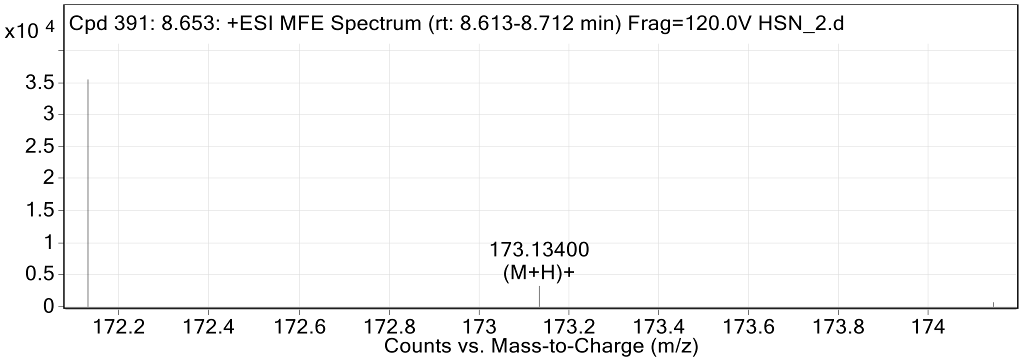

Peak List

m/z	z	Abund	Ion
172.13088	1	35464.46	(M+H)+
173.134	1	3261.18	(M+H)+
174.14465	1	712.3	(M+H)+

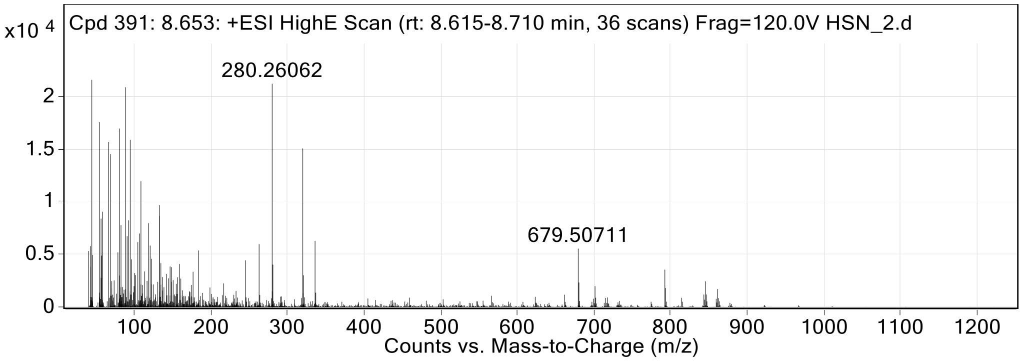

Peak List

m/z	z	Abund
45.03317		21531.95
55.05371		17519.82
67.05342		15626.92
69.06901		14490.36
81.06882		16918.36
89.05844		20833.97
95.0841		15833.83
109.09945		11912.32
280.26062	1	21162.01
320.25291	1	15029.71

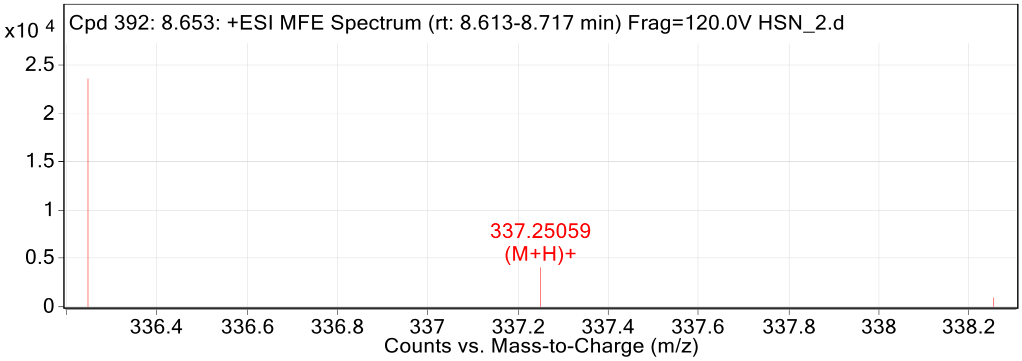

Peak List

m/z	z	Abund	Ion
336.24745	1	23651.77	(M+H)+
337.25059	1	4075.25	(M+H)+
338.25458	1	950.08	(M+H)+

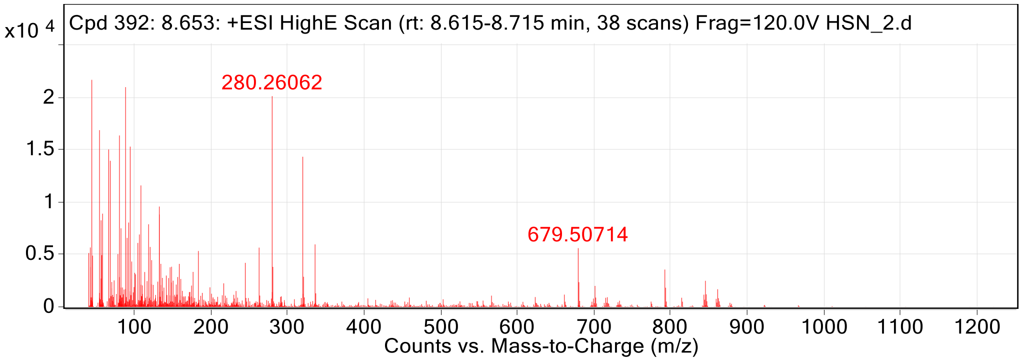

Peak List

m/z	z	Abund
45.03317		21657.2
55.05371		16856.95
67.05342		15015.11
69.06901		13936.89
81.06882		16351.47
89.05844		20960.42
95.0841		15286.24
109.09946		11584.57
280.26062	1	20104.33
320.2529	1	14321.67

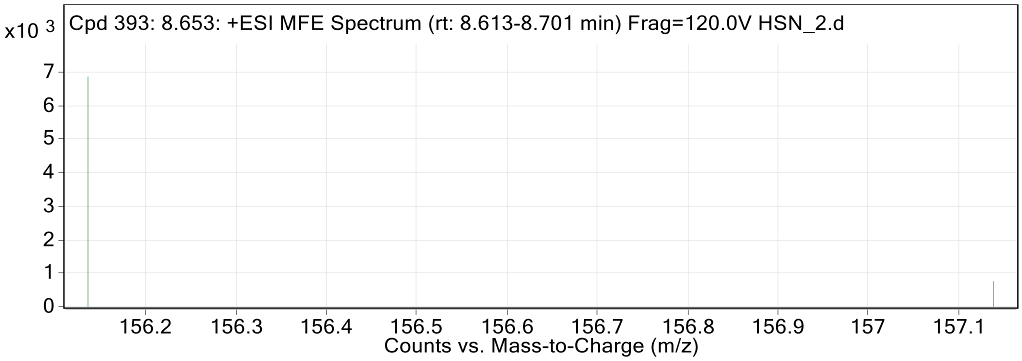

Peak List

m/z	z	Abund	Ion
156.13624	1	6868.52	(M+H)+
157.13888	1	762.52	(M+H)+

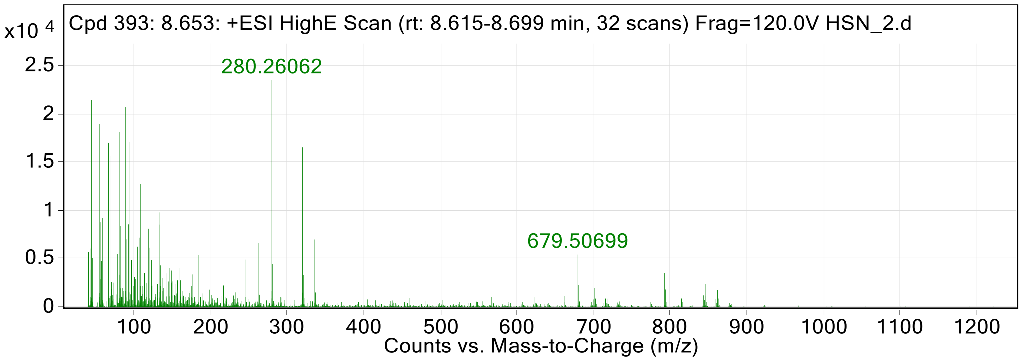

Peak List

m/z	z	Abund
45.03317		21403.74
55.05371		18945.17
67.05342		16987.27
69.06901		15647.87
81.06882		18088.36
89.05844		20660.78
95.08412	1	17056.24
109.09944		12694.56
280.26062	1	23479.68
320.25292	1	16511.6

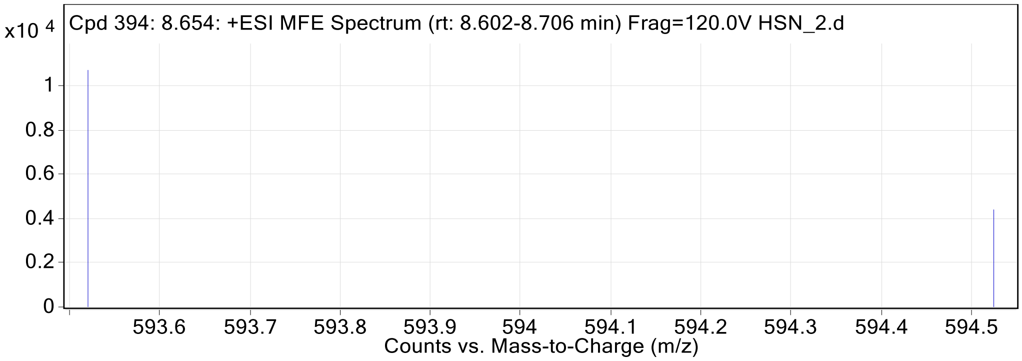

Peak List

m/z	z	Abund	Ion
593.52062	1	10707.18	(M+H)+
594.52462	1	4403.03	(M+H)+

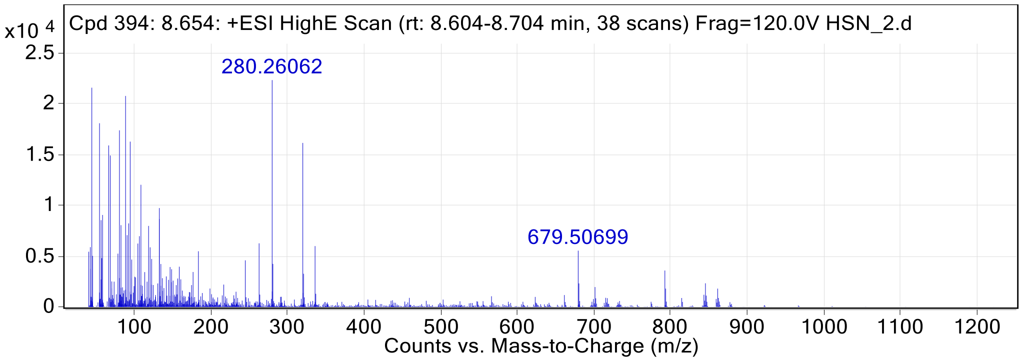

Peak List

m/z	z	Abund
45.03317		21592.9
55.0537		18089.1
67.05343		15903.76
69.06902		14908.66
81.06883		17392.8
89.05844		20777.51
95.0841	1	16276.86
109.09944		12024.62
280.26062	1	22340.93
320.25295	1	16148.26

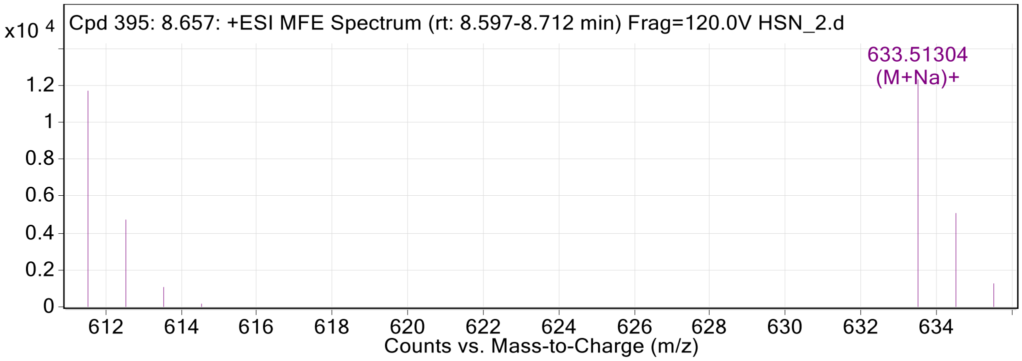

Peak List

m/z	z	Abund	Ion
611.53135	1	11716.02	(M+H)+
612.53452	1	4730.94	(M+H)+
613.53592	1	1075.25	(M+H)+
614.53924	1	171.8	(M+H)+
633.51304	1	12328.53	(M+Na)+
634.51667	1	5083.76	(M+Na)+
635.51898	1	1271.32	(M+Na)+

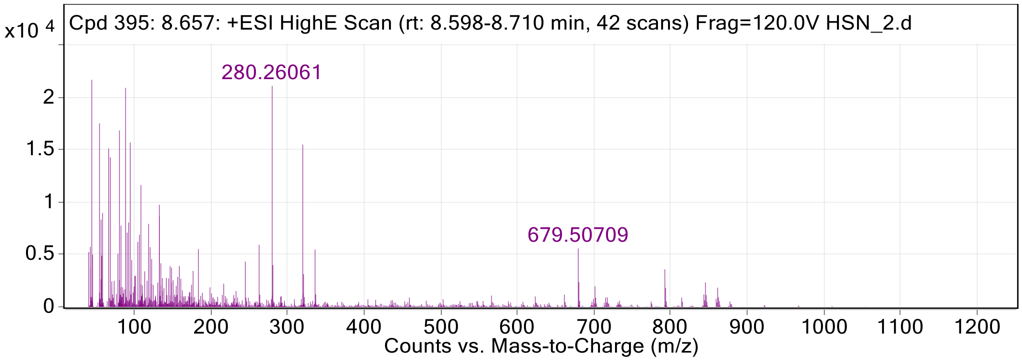

Peak List

m/z	z	Abund
45.03318		21644.49
55.0537		17487.26
67.05343		15100.76
69.06903		14264.58
81.06884		16818.36
89.05844		20875.18
95.08409	1	15669.92
109.09944		11620.86
280.26061	1	21063.89
320.25295	1	15463.49

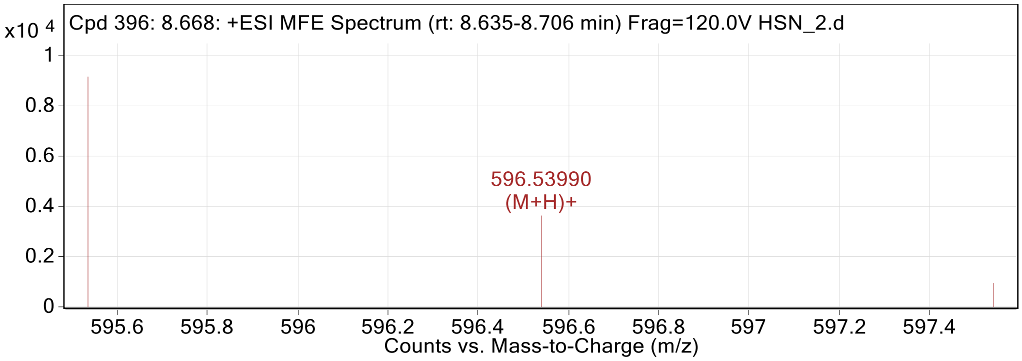

Peak List

m/z	z	Abund	Ion
595.53519	1	9180.97	(M+H)+
596.5399	1	3640.49	(M+H)+
597.54265	1	954.19	(M+H)+

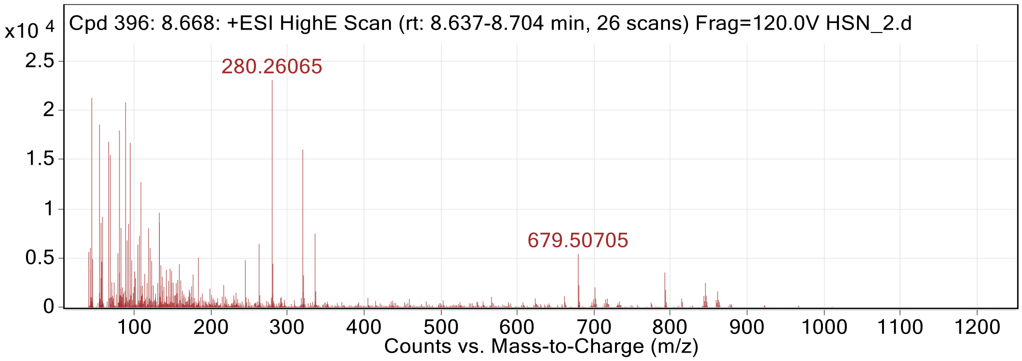

Peak List

m/z	z	Abund
45.03318		21254.13
55.0537		18554.9
67.05342		16814.13
69.06903		15456.77
81.06882		17950.64
89.05845		20789.73
95.08414		16703.13
109.09947		12710.41
280.26065	1	23074.11
320.25293	1	16013.19

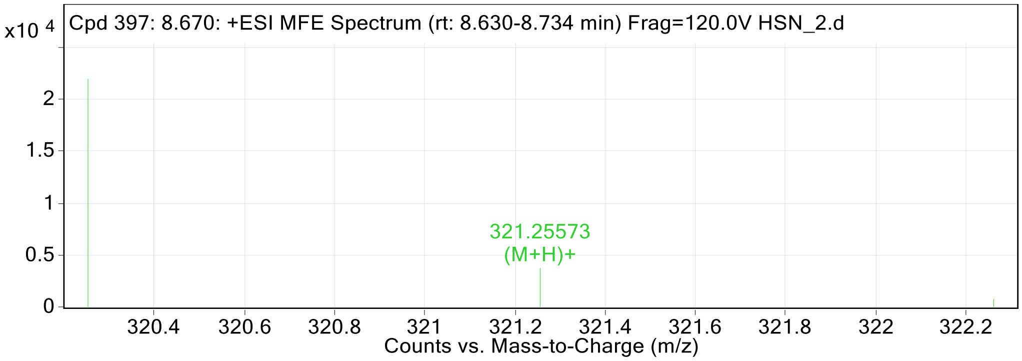

Peak List

m/z	z	Abund	Ion
320.25318	1	22000.69	(M+H)+
321.25573	1	3754.53	(M+H)+

322.26155	1	741.92	(M+H)+

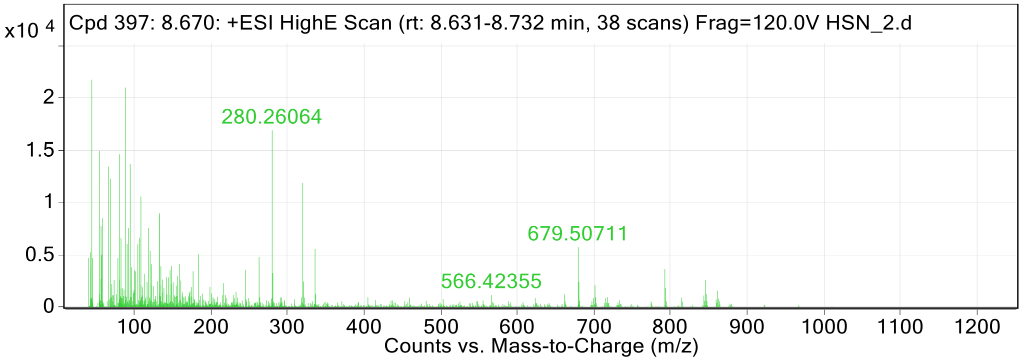

Peak List

m/z	z	Abund
45.03317		21742.14
55.05371		14905.21
67.05343		13430.42
69.06902		12270.09
81.06881		14604.66
89.05846		20995.77
95.08412		13668.96
109.09949		10562.69
280.26064	1	16901.19
320.2529	1	11873.55

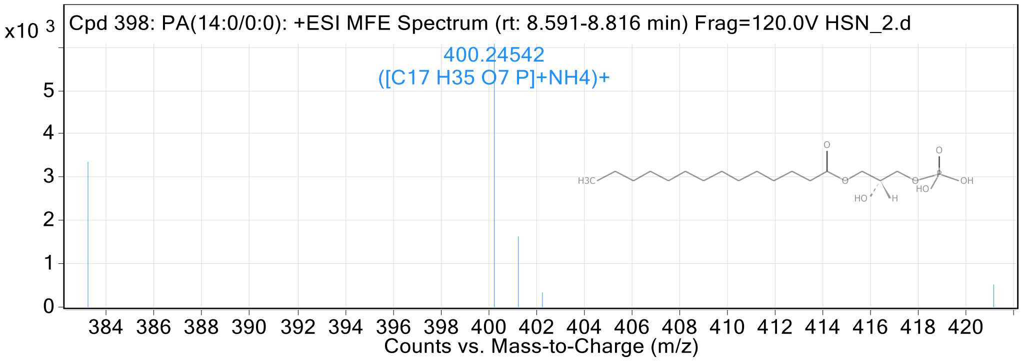

Peak List

m/z	z	Abund	Name	Formula	Ion	Score (DB)	Hits (DB)
383.21918	1	3352.34			(M+H)+		
400.24542	1	6082.07	PA(14:0/0:0)	C17 H35 O7 P	(M+NH4)+	73.16	2
401.2527	1	1631.09		C17 H35 O7 P	(M+NH4)+		
402.26137	1	341		C17 H35 O7 P	(M+NH4)+		
421.17665	1	518.64			(M+K)+		

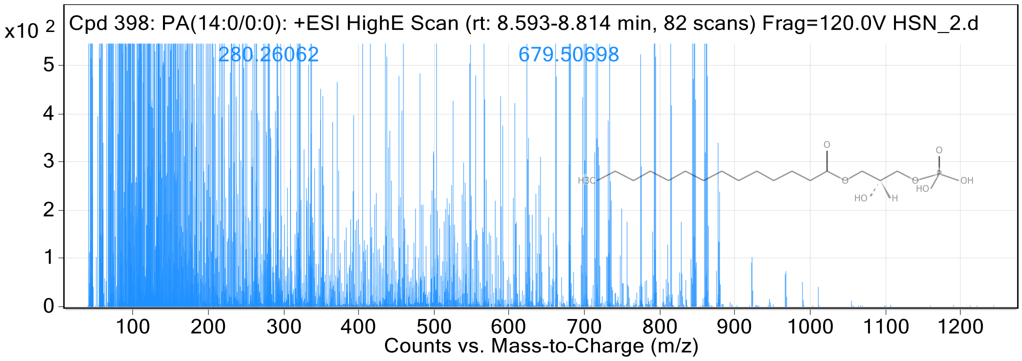

Peak List

m/z	z	Abund
45.03316		20596.21
55.0537		13880.15
67.05344		13595.05
69.06903		10977.84
81.06882		13538.9
89.05845		19652.92
95.08407		13506.31
109.09944		10076.82
133.09766		9108.85
280.26062	1	14626.9

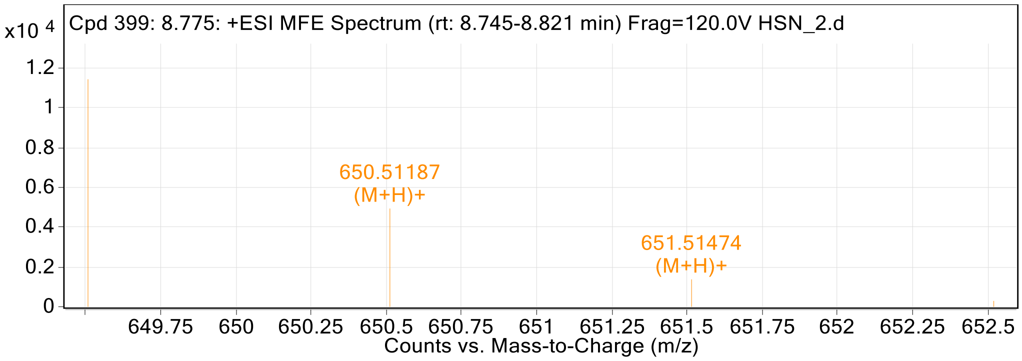

Peak List

m/z	z	Abund	Ion
649.50816	1	11425.39	(M+H)+
650.51187	1	4933.6	(M+H)+
651.51474	1	1376.76	(M+H)+
652.51941	1	294.21	(M+H)+

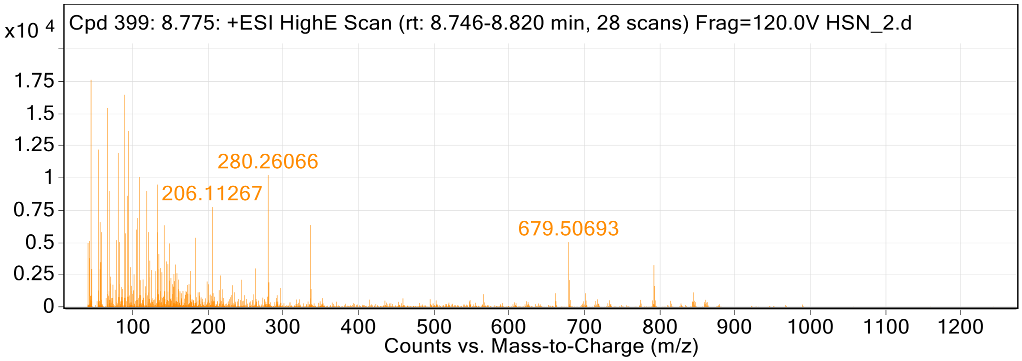

Peak List

m/z	z	Abund
45.03314		17599.68
55.05369		12193.73
67.05345		15406.35
69.06897		8971.17
81.06878		11933.09
89.05848		16442.02
95.08404		13625.15
109.09944		10065.8
133.09726		9478.91
280.26066	1	10210.72

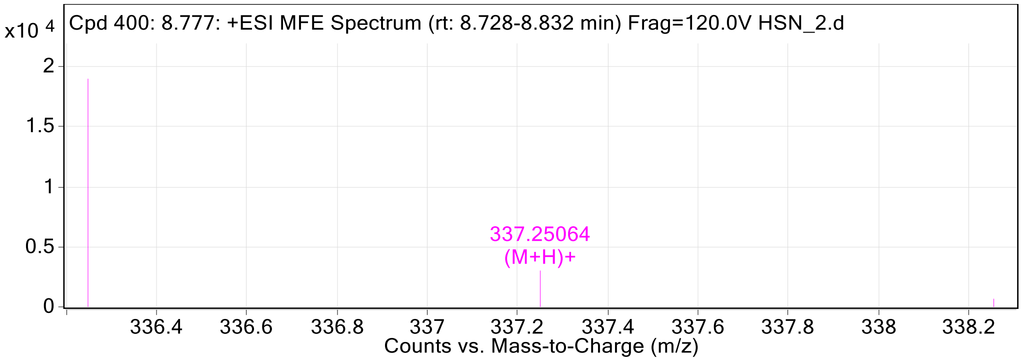

Peak List

m/z	z	Abund	Ion
336.24797	1	19000.74	(M+H)+
337.25064	1	3025.36	(M+H)+
338.256	1	678.61	(M+H)+

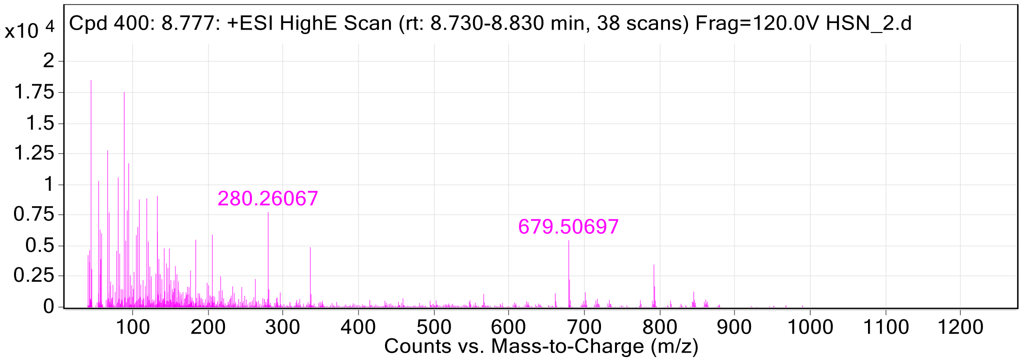

Peak List

m/z	Abund
45.03314	18511.15
55.05369	10278.84
67.05346	12773.17
81.06877	10562.14
89.05845	17524.23
93.06851	7898.65
95.08401	11726.46
109.09943	8775.74
119.0836	8851.1
133.09717	9067.98

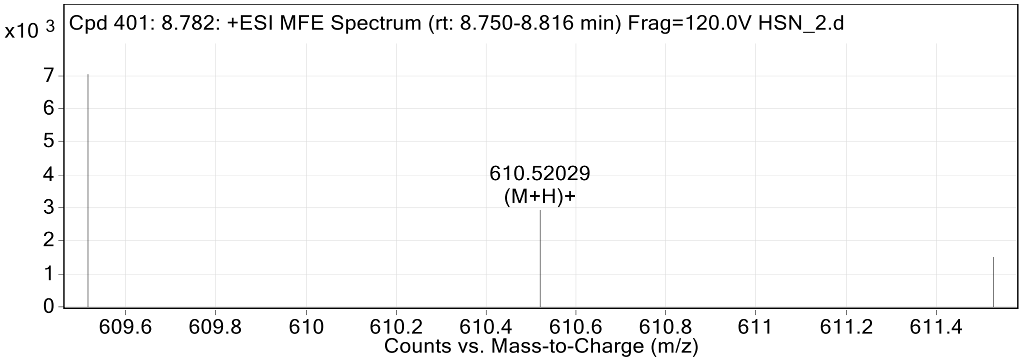

Peak List

m/z	z	Abund	Ion
609.51575	1	7038.4	(M+H)+
610.52029	1	2937.28	(M+H)+

611.52813	1	1514.19	(M+H)+

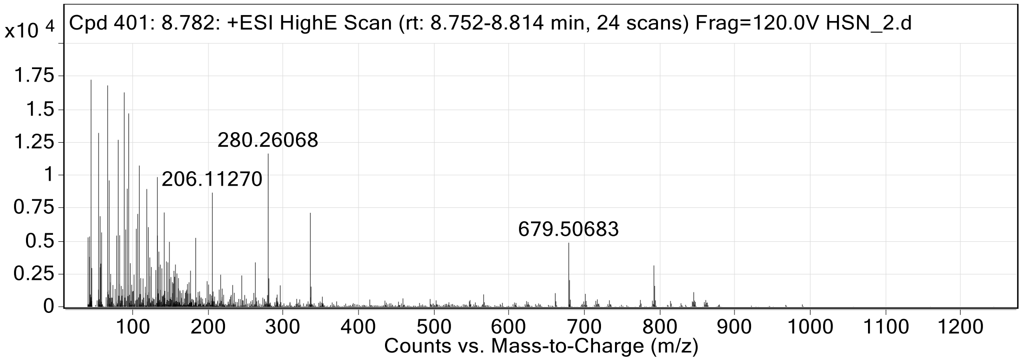

Peak List

m/z	z	Abund
45.03315		17255.27
55.0537		13210.65
67.05346		16822.93
69.069		9600.96
81.06879		12686.11
89.05849		16290.83
95.08404		14692.22
109.09944		10732.04
133.09719		9855.41
280.26068	1	11643.22

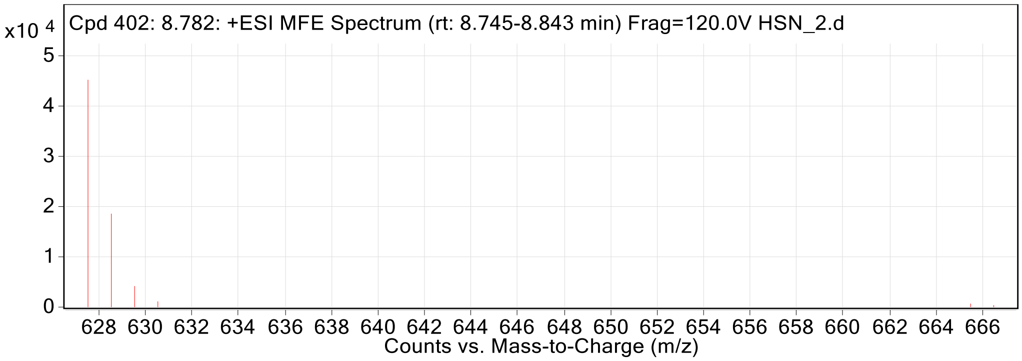

Peak List

m/z	z	Abund	Ion
627.52544	1	45146.75	(M+H)+
628.52949	1	18548.28	(M+H)+
629.5306	1	4167.47	(M+H)+
630.53069	1	1128.2	(M+H)+
665.48304	1	688.97	(M+K)+
666.47967	1	381.22	(M+K)+

Qualitative Analysis Report

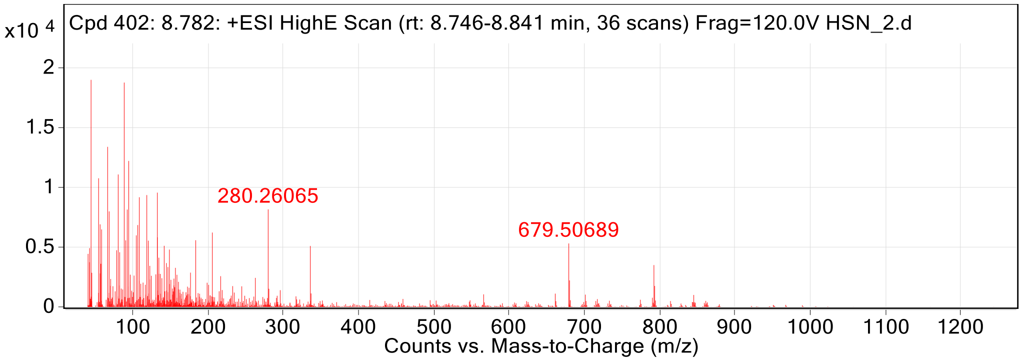

Peak List

m/z	z	Abund
45.03314		19014.04
55.05369		10768.42
67.05345		13402.64
81.06877		11089.88
89.05847		18788.24
95.08403		12223.19
109.0994		9179.52
119.08362		9364.18
133.09714		9569.23
280.26065	1	8163.81

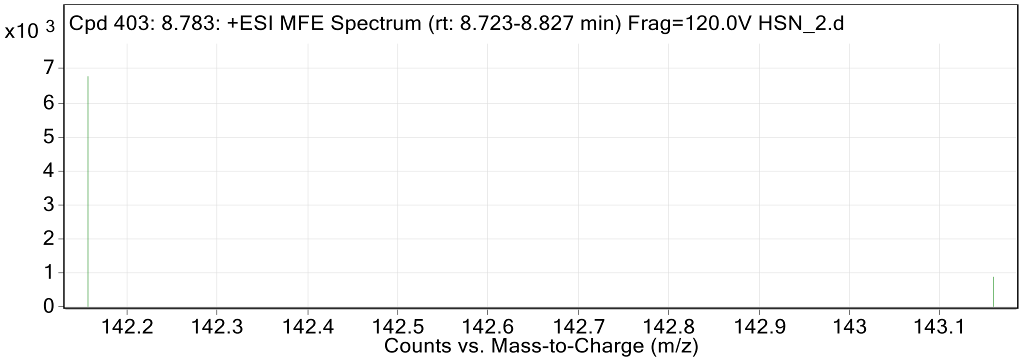

Peak List

m/z	z	Abund	Ion
142.15667	1	6777.99	(M+H)+
143.16004	1	881.66	(M+H)+

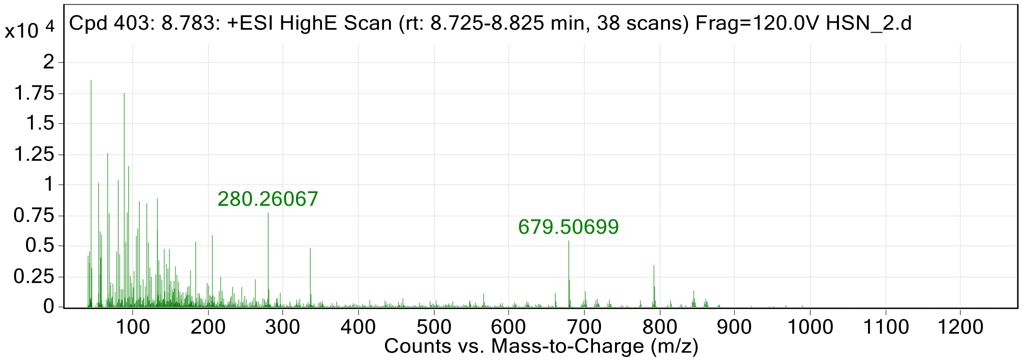

Peak List

m/z	Abund
45.03314	18577.44
55.05369	10170.49
67.05346	12596.01

81.06878	10386.5
89.05846	17504.92
93.0685	7745.37
95.08403	11526.27
109.09943	8657.84
119.08361	8482.51
133.09711	8871.57

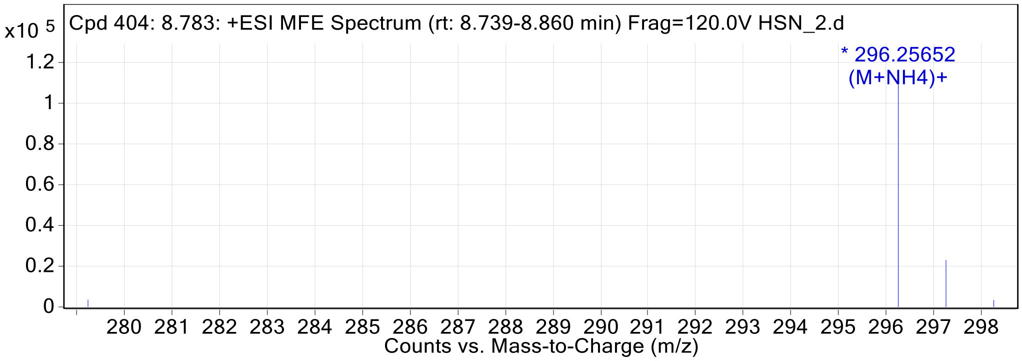

Peak List

m/z	z	Abund	Ion
279.23202	1	3537.59	(M+H)+
296.25652	1	111997.97	(M+NH4)+
297.25875	1	23091.43	(M+NH4)+
298.2626	1	3412.38	(M+NH4)+

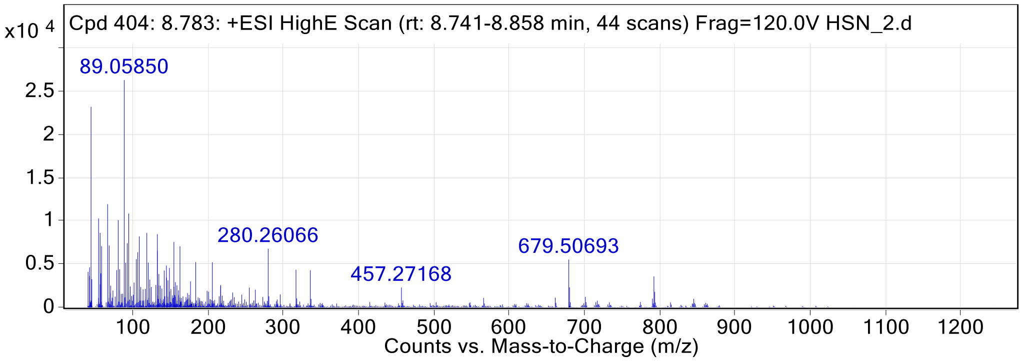

Peak List

m/z	Abund
45.03317	23217.99
55.0537	10270.74
57.06923	8599.66
67.05346	11925.76
81.06877	10074.25
89.0585	26307.9
95.08405	10842.15
109.0994	8187.9
119.08362	8591.02
133.09708	8456.88

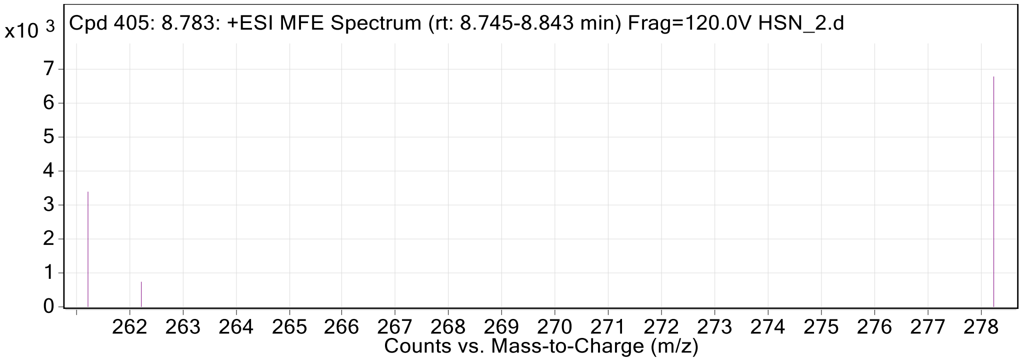

Peak List

m/z	z	Abund	Ion
261.21867	1	3400.44	(M+H)+
262.22204	1	741.35	(M+H)+
278.24491	1	6800.15	(M+NH4)+

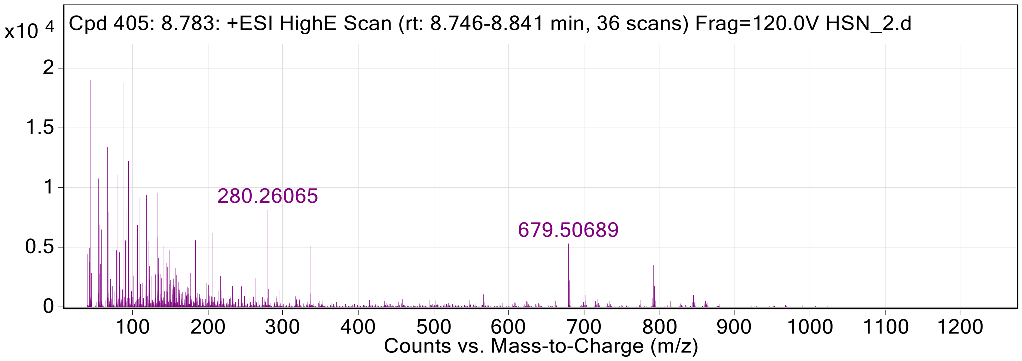

Peak List

m/z	z	Abund
45.03314		19014.04
55.05369		10768.42
67.05345		13402.64
81.06877		11089.88
89.05847		18788.24
95.08403		12223.19
109.0994		9179.52
119.08362		9364.18
133.09714		9569.23
280.26065	1	8163.81

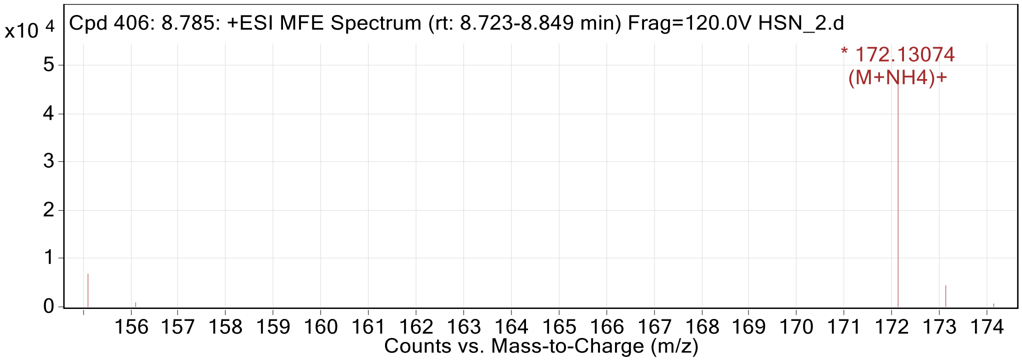

Peak List

m/z	z	Abund	Ion
155.10445	1	6866.63	(M+H)+
156.10637	1	929.33	(M+H)+

172.13074	1	47022.73	(M+NH4)+
173.13409	1	4490.7	(M+NH4)+
174.14211	1	696.71	(M+NH4)+

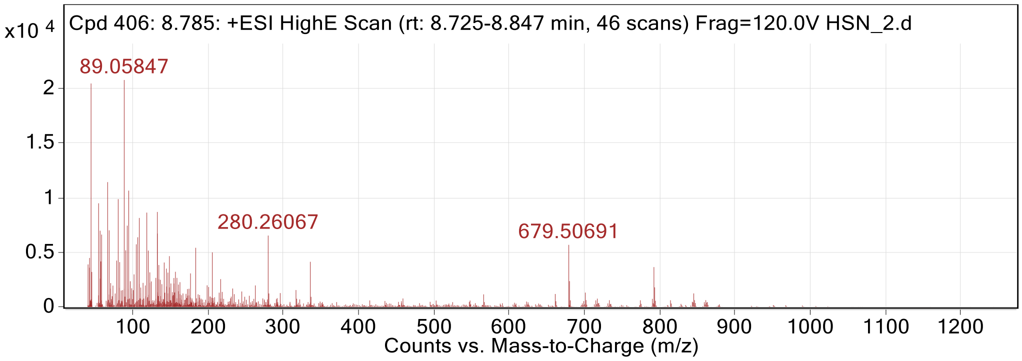

Peak List

m/z	Abund
45.03314	20459.4
55.0537	9478.7
67.05345	11418.84
81.06877	9858.32
89.05847	20778.42
93.0685	7430.86
95.08404	10639.68
109.09941	8134.03
119.08362	8624.95
133.09718	8684.83

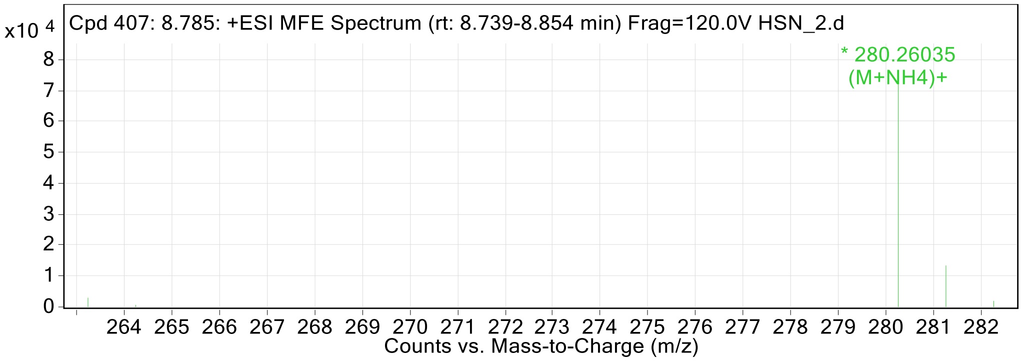

Peak List

m/z	z	Abund	Ion
263.23361	1	2983.45	(M+H)+
264.23805	1	636.45	(M+H)+
280.26035	1	73511.18	(M+NH4)+
281.26373	1	13418.89	(M+NH4)+
282.26848	1	1957.17	(M+NH4)+

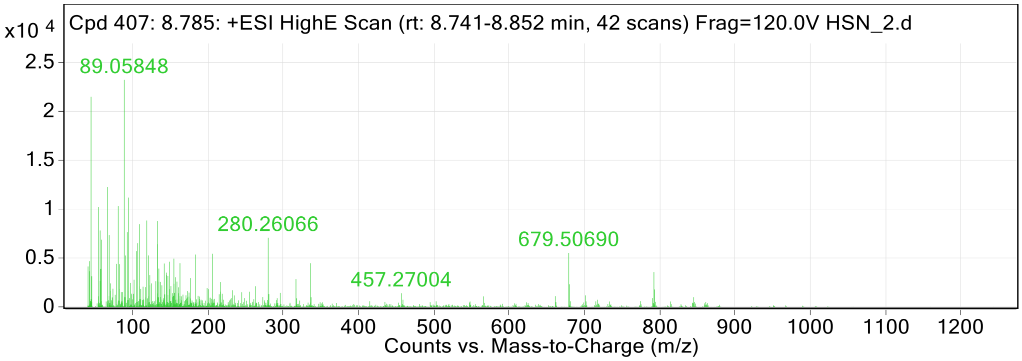

Peak List

m/z	Abund
45.03316	21479.52
55.0537	10217.87
57.06925	7805.42
67.05346	12245.3
81.06877	10302.48
89.05848	23205.44
95.08405	11172.56
109.0994	8442.89
119.08362	8825.35
133.09713	8779.84

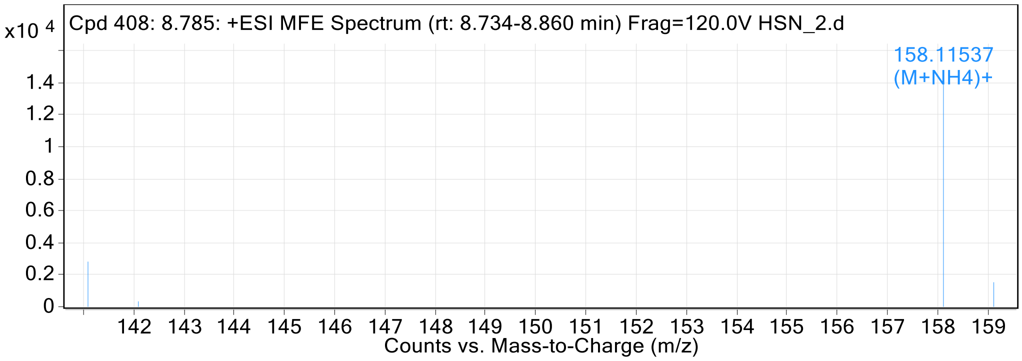

Peak List

m/z	z	Abund	Ion
141.08946	1	2821.09	(M+H)+
142.09191	1	337.72	(M+H)+
158.11537	1	14213.55	(M+NH4)+
159.11708	1	1533.18	(M+NH4)+

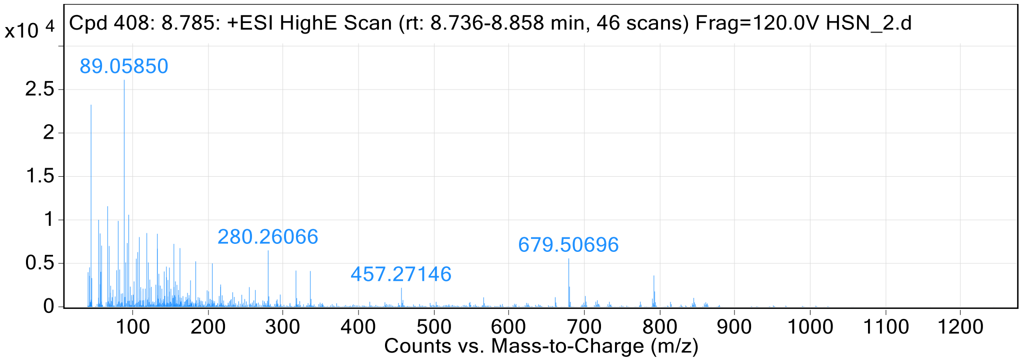

Peak List

m/z	Abund
45.03317	23211.7

55.0537	9992.29
57.06923	8428.25
67.05346	11554.81
81.06877	9868.8
89.0585	26068.93
95.08405	10576.14
109.0994	8009.9
119.08362	8472.93
133.09708	8389.82

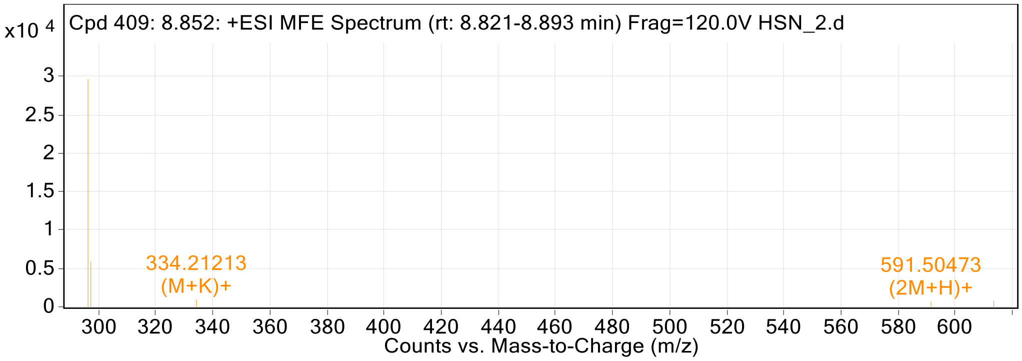

Peak List

m/z	z	Abund	Ion
296.25584	1	29653.94	(M+H)+
297.25877	1	5890.88	(M+H)+
334.21213	1	945.39	(M+K)+
591.50473	1	708.02	(2M+H)+
613.48426	1	760.09	(2M+Na)+

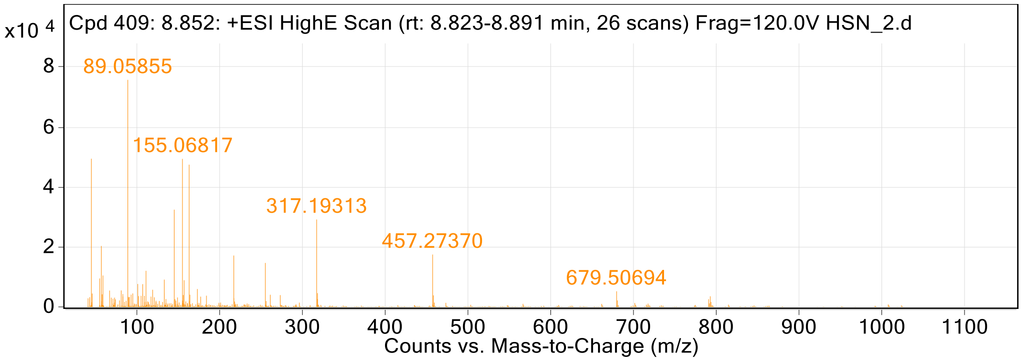

Peak List

m/z	z	Abund
45.03323		49443.31
57.06932		20329.11
89.05855	1	75738.87
145.12025	1	32436.69
155.06817	1	49386.25
163.13078	1	47454
217.1047	1	17135.39
255.1565	1	14667.69
317.19313	1	29156.52
457.2737	1	17470.8

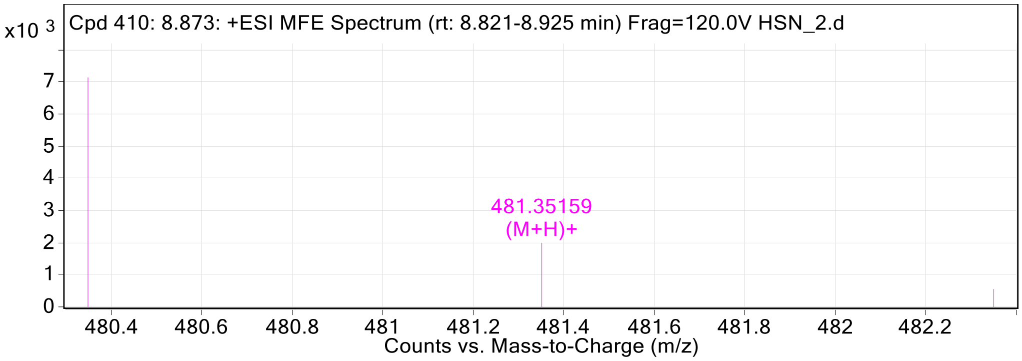

Peak List

m/z	z	Abund	Ion
480.34866	1	7145.47	(M+H)+
481.35159	1	1994.67	(M+H)+
482.35043	1	554.84	(M+H)+

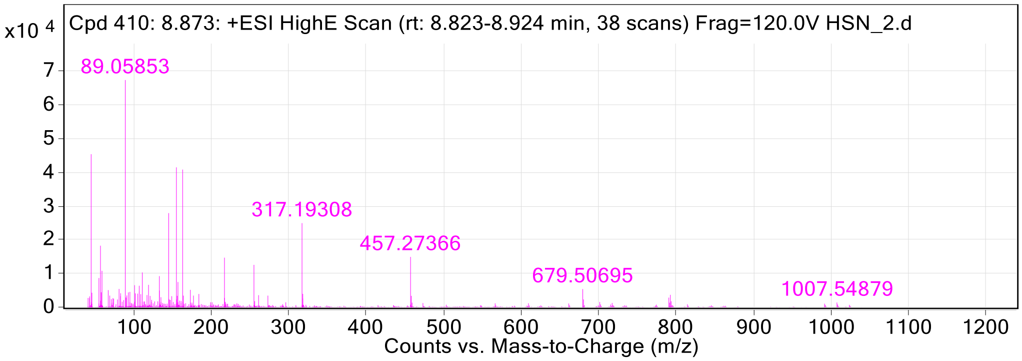

Peak List

m/z	z	Abund
45.03322		45396.3
57.06931		18225.88
89.05853	1	67406.71
145.12024	1	27900.83
155.06813	1	41486.45
163.13078	1	40828.51
217.10468	1	14674.85
255.15646	1	12536.04
317.19308	1	24925.88
457.27366	1	14896.25

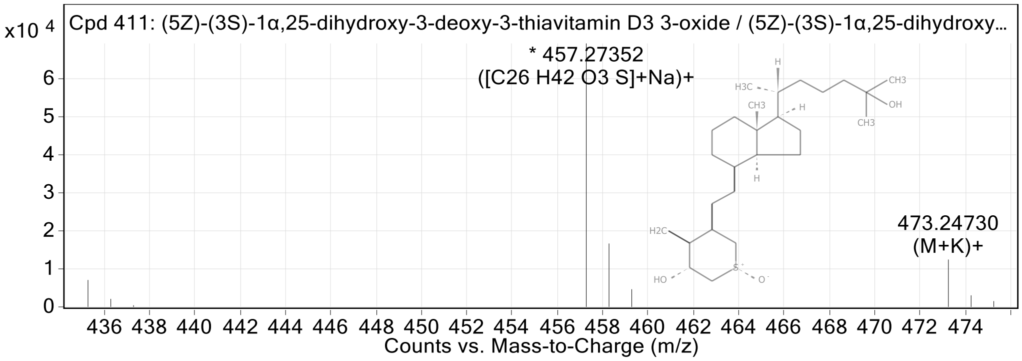

Peak List

m/z	z	Abund	Name	Formula	Ion	Score (DB)	Hits (DB)
435.2919	1	7068.88			(M+H)+		
436.29775	1	2111.04			(M+H)+		

437.30104	1	433.21			(M+H)+		
457.27352	1	69189.35	(5Z)-(3S)-1α,25-dihydroxy-3-deoxy-3-thiavitamin D3 3-oxide / (5Z)-(3S)-1α,25-dihydroxy-3-deoxy-3-thiacholecalciferol 3-oxide	C26 H42 O3 S	(M+Na)+	89.93	8
458.27717	1	16643.13		C26 H42 O3 S	(M+Na)+		
459.27951	1	4644.23		C26 H42 O3 S	(M+Na)+		
473.2473	1	12433.12			(M+K)+		
474.25015	1	3039.21			(M+K)+		
475.24999	1	1522.52			(M+K)+		

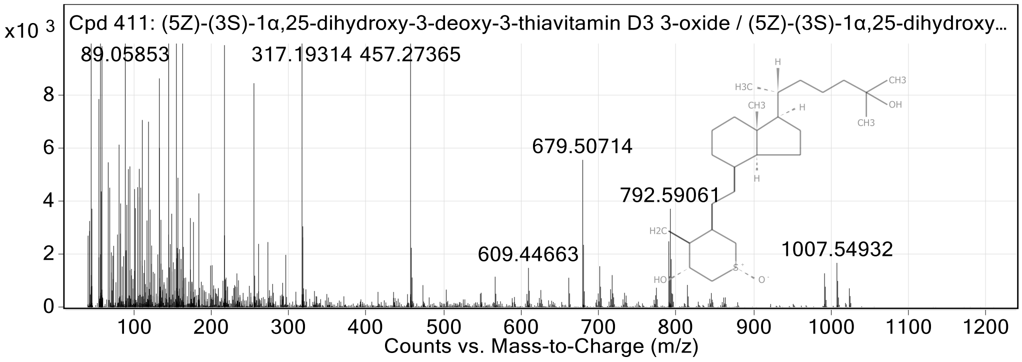

Peak List

m/z	z	Abund
45.03322		36741.46
57.0693		13727.63
59.04863		9860.13
89.05853	1	50966.41
145.12024	1	18722.02
155.06814	1	27787.86
163.13078	1	27292.69
217.10479		9900.54
317.19314	1	16750.17
457.27365	1	9960.59

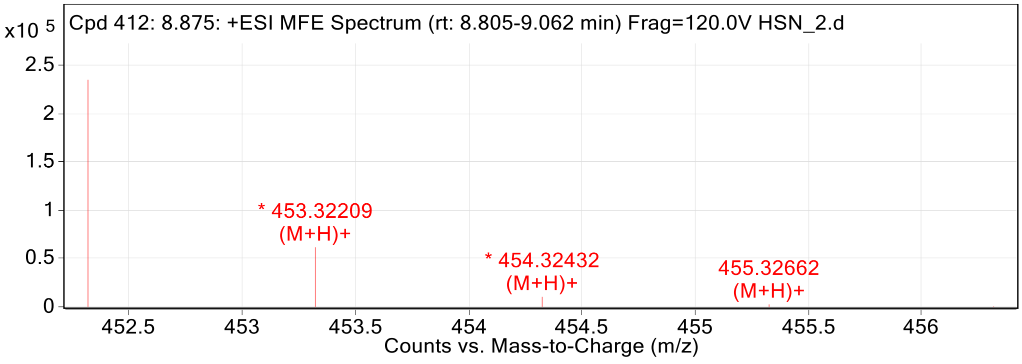

Peak List

m/z	z	Abund	Ion
452.31837	1	234795.16	(M+H)+
453.32209	1	61481.46	(M+H)+
454.32432	1	10391.51	(M+H)+
455.32662	1	2481.79	(M+H)+

m/z	z	Abund	Ion
456.31918	1	440.01	(M+H)+

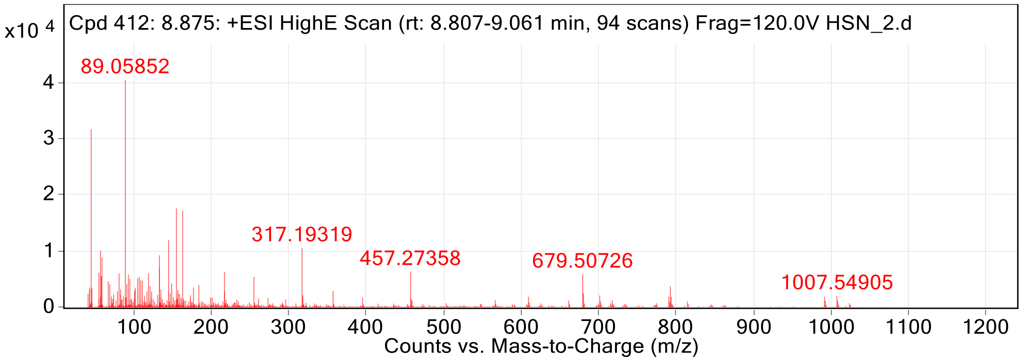

Peak List

m/z	z	Abund
45.0332		31674.53
57.06931		10019.84
59.04863		8780.61
89.05852	1	40423.79
133.08468		9200.16
133.09741		7077.56
145.12023		11875.5
155.06816	1	17561.61
163.13079	1	17165.41
317.19319	1	10502.11

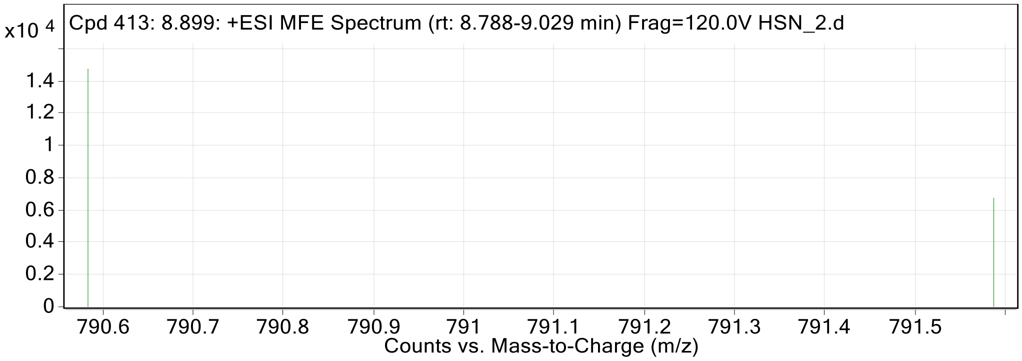

Peak List

m/z	z	Abund	Ion
790.58389	1	14754.44	(M+H)+
791.58717	1	6758.31	(M+H)+

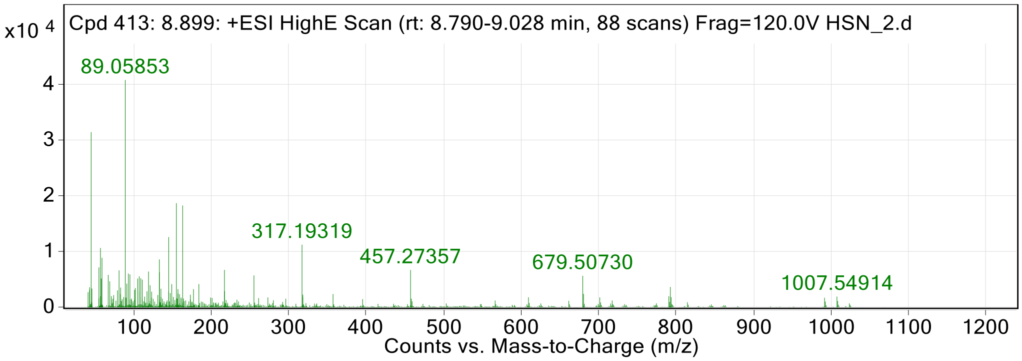

Peak List

m/z	z	Abund
45.03321		31414.84

55.05368		7127.08
57.06929		10610.07
59.04863		8851.82
89.05853	1	40774.08
133.0847		8557.54
145.12023		12570.79
155.06815	1	18651.29
163.13079	1	18245.4
317.19319	1	11197.19

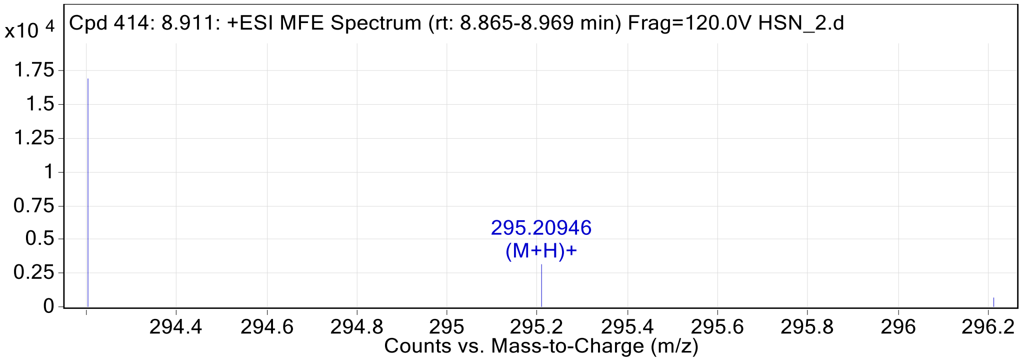

Peak List

m/z	z	Abund	Ion
294.20353	1	16934.52	(M+H)+
295.20946	1	3158.73	(M+H)+
296.21233	1	686.3	(M+H)+

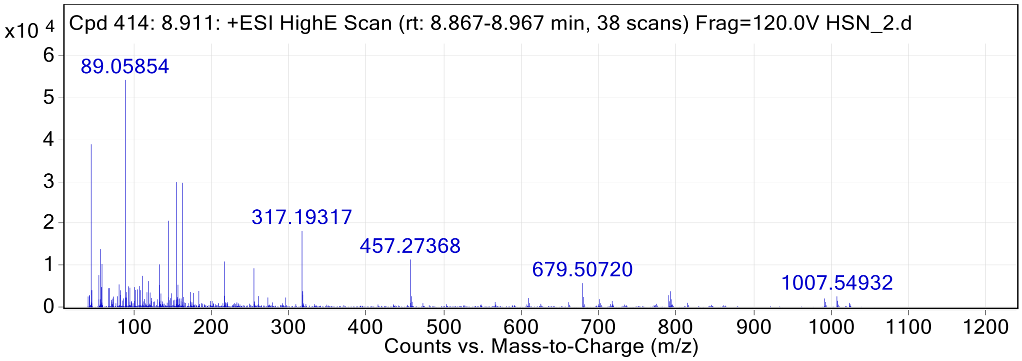

Peak List

m/z	z	Abund
45.03322		38898.66
57.06932		13867.69
59.04861		10315.59
89.05854	1	54276.64
145.12024	1	20639.8
155.06814	1	29825.84
163.13077	1	29721.45
217.10468		10876.2
317.19317	1	18225.77
457.27368	1	11351.51

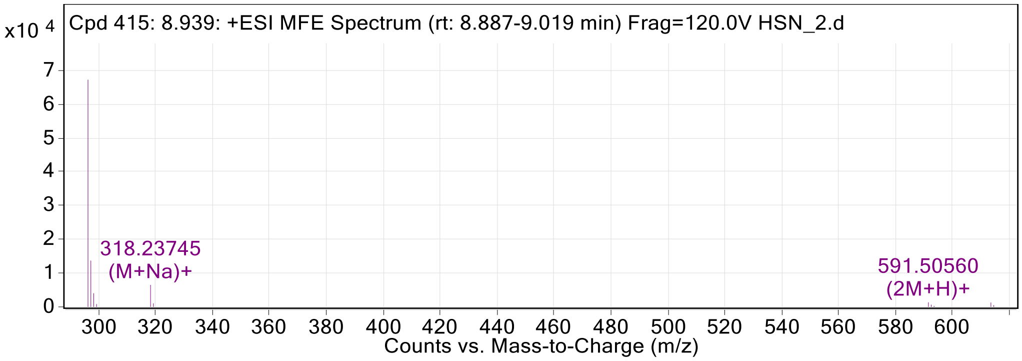

Peak List

m/z	z	Abund	Ion
296.25512	1	67189.88	(M+H)+
297.25908	1	13692.17	(M+H)+
298.26774	1	4046.3	(M+H)+
299.27186	1	823.82	(M+H)+
318.23745	1	6498.52	(M+Na)+
319.23995	1	1040.34	(M+Na)+
591.5056	1	1344.03	(2M+H)+
592.50888	1	652.47	(2M+H)+
613.48497	1	1292.78	(2M+Na)+
614.48979	1	523.47	(2M+Na)+

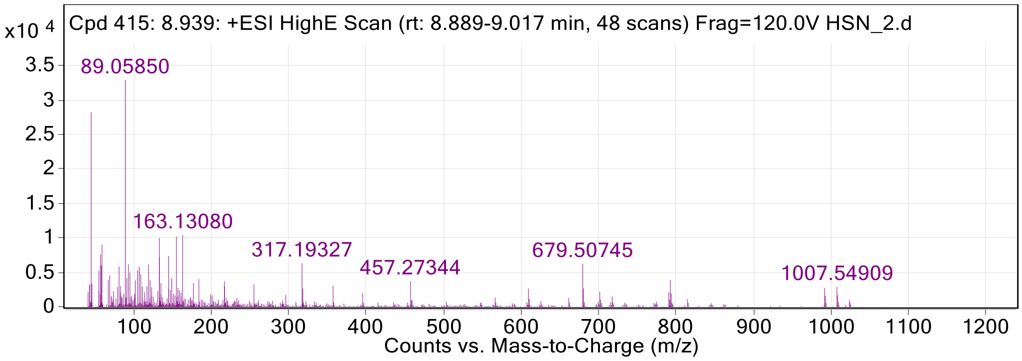

Peak List

m/z	z	Abund
45.0332		28214.88
57.06931		7590.58
59.04862		9015.23
89.0585	1	32894.73
133.08476		9969.29
133.09786		7163.08
145.12022		7353.29
155.06806		10163.42
163.1308		10409.23
317.19327		6309.55

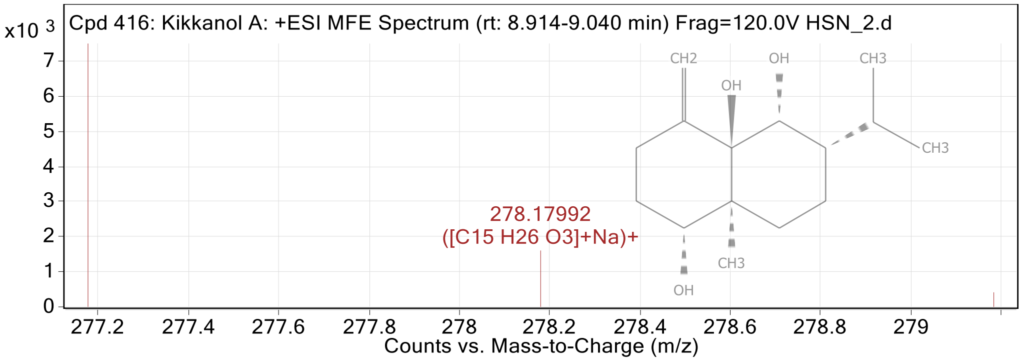

Peak List

m/z	z	Abund	Name	Formula	Ion	Score (DB)	Hits (DB)
277.17718	1	7504.39	Kikkanol A	C15 H26 O3	(M+Na)+	91.9	10
278.17992	1	1605.94		C15 H26 O3	(M+Na)+		
279.1837	1	414.74		C15 H26 O3	(M+Na)+		

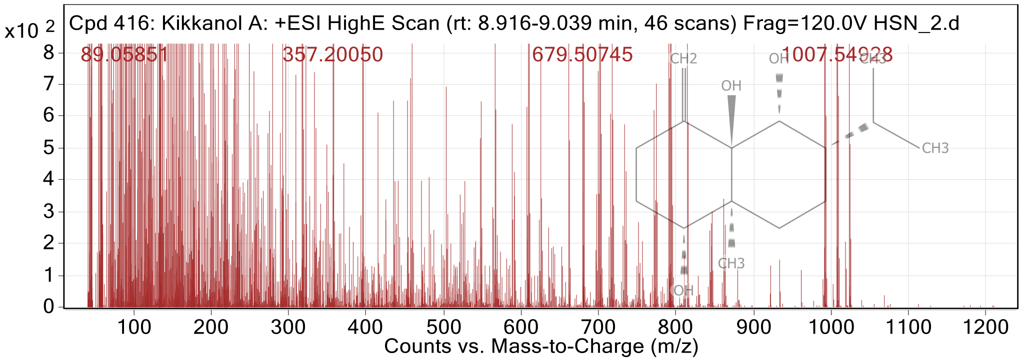

Peak List

m/z	z	Abund
45.03319		23482.5
58.06462		6717.08
59.04863		8054.24
81.06878		6168.9
89.05851		23954.99
93.06854		6697.75
107.08378		5888.59
133.08481		9955.49
133.09772		8211.18
679.50745	1	6390.48

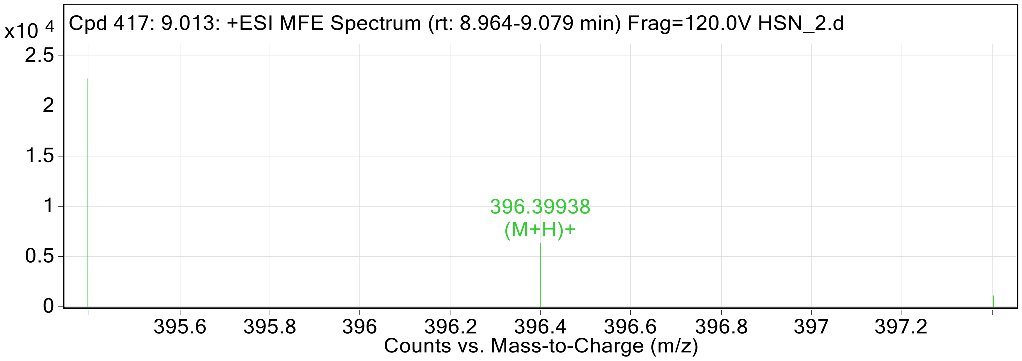

Peak List

m/z	z	Abund	Ion
395.39632	1	22743.52	(M+H)+
396.39938	1	6344.02	(M+H)+

397.40345	1	1119.29	(M+H)+

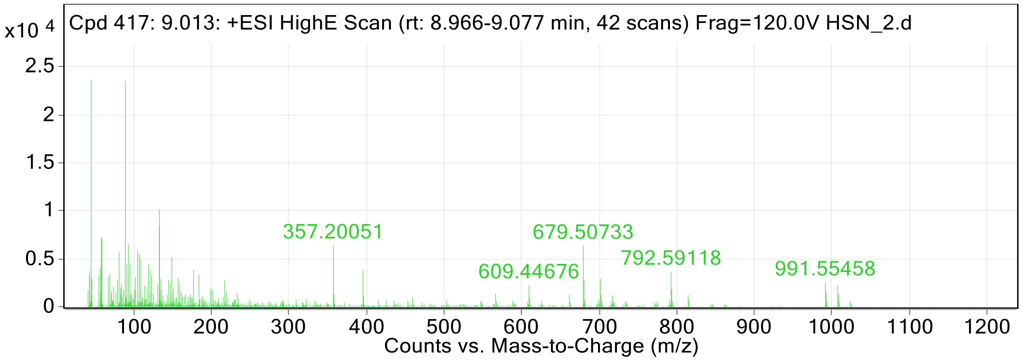

Peak List

m/z	z	Abund
45.03315		23676.89
58.06462		7204.99
59.04859		7041.89
89.05848		23535.47
93.06853		6523.4
105.06825		5877.95
133.08476		10159.6
133.09765		8469.83
357.20051	1	6416.75
679.50733	1	6375.12

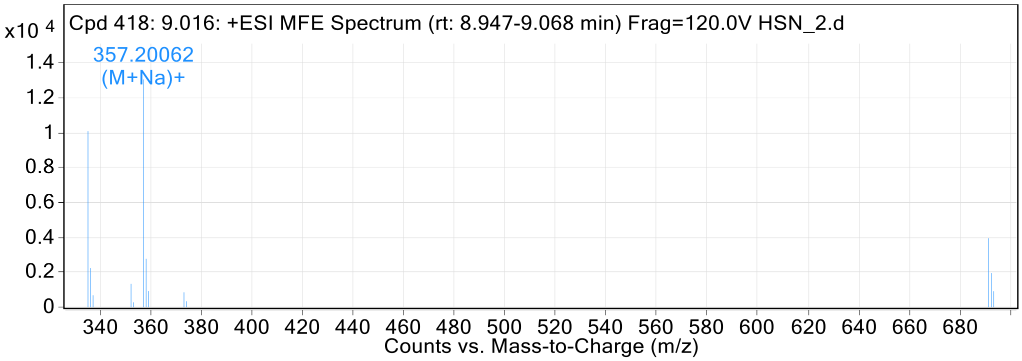

Peak List

m/z	z	Abund	Ion
335.21874	1	10095.34	(M+H)+
336.22359	1	2243.97	(M+H)+
352.24631	1	1336.87	(M+NH4)+
357.20062	1	13072.63	(M+Na)+
358.20368	1	2775.56	(M+Na)+
359.1896	1	924.5	(M+Na)+
373.17615	1	843.91	(M+K)+
691.41416	1	3945.27	(2M+Na)+
692.41684	1	1956.94	(2M+Na)+
693.4158	1	910.14	(2M+Na)+

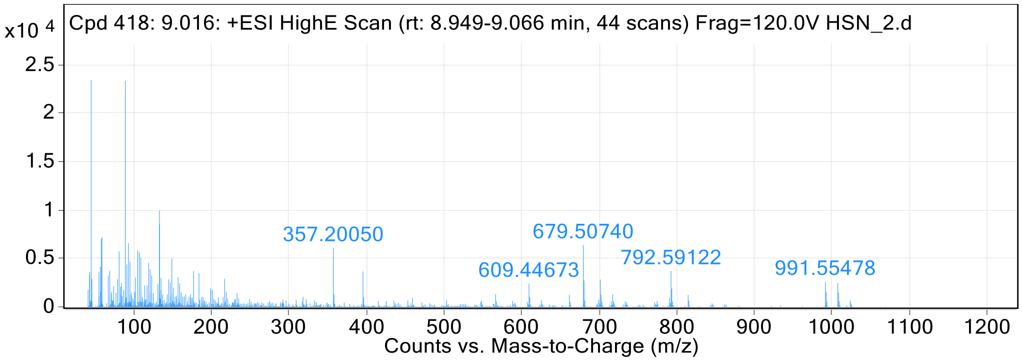

Peak List

m/z	z	Abund
45.03317		23446.8
58.06462		7028.5
59.04863		7199.15
89.05849		23333.45
93.06854		6599.73
105.06826		5839.54
133.08477		9950.78
133.09767		8503.64
357.2005	1	6089.56
679.5074	1	6388.25

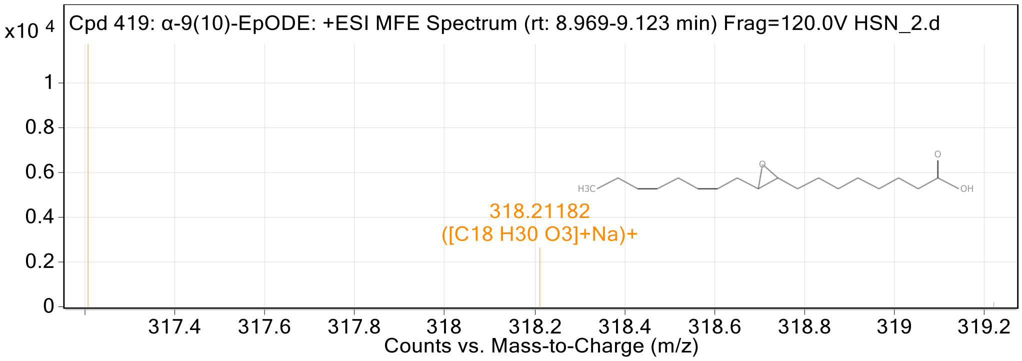

Peak List

m/z	z	Abund	Name	Formula	Ion	Score (DB)	Hits (DB)
317.20805	1	11725.95	α-9(10)-EpODE	C18 H30 O3	(M+Na)+	96.35	10
318.21182	1	2638.48		C18 H30 O3	(M+Na)+		
319.21989	1	195.57		C18 H30 O3	(M+Na)+		

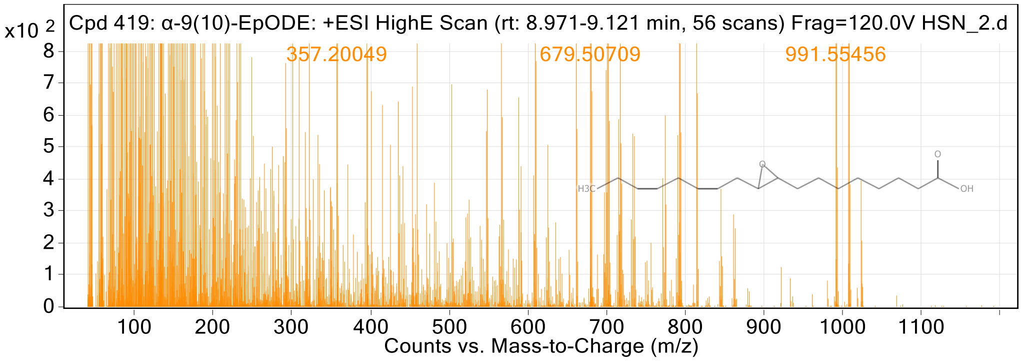

Peak List

m/z	z	Abund
45.03314		24066.01
58.06458		7330.57

59.04856		6895.93
89.05846	1	23597.23
93.06848		6401.85
105.0682		5666.03

m/z	z	Abund
133.08483		10133.87
133.09744		7249.78
149.09385		6571.81
679.50709	1	6803.37

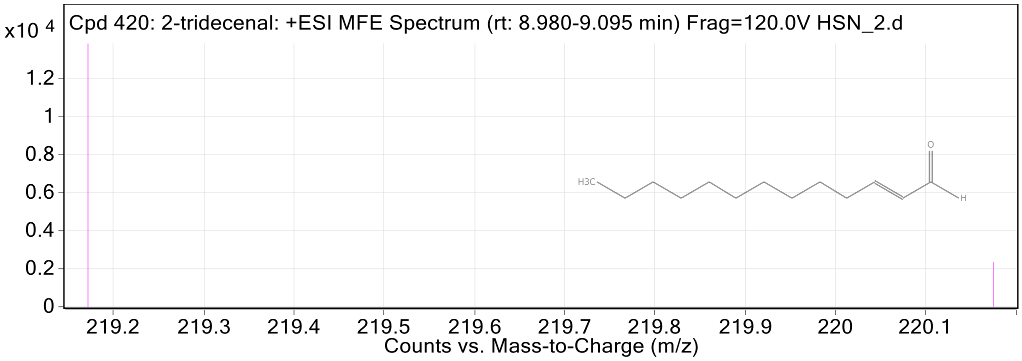

Peak List

m/z	z	Abund	Name	Formula	Ion	Score (DB)	Hits (DB)
219.17174	1	13879.6	2-tridecenal	C13 H24 O	(M+Na)+	85.36	10
220.17525	1	2352.63		C13 H24 O	(M+Na)+		

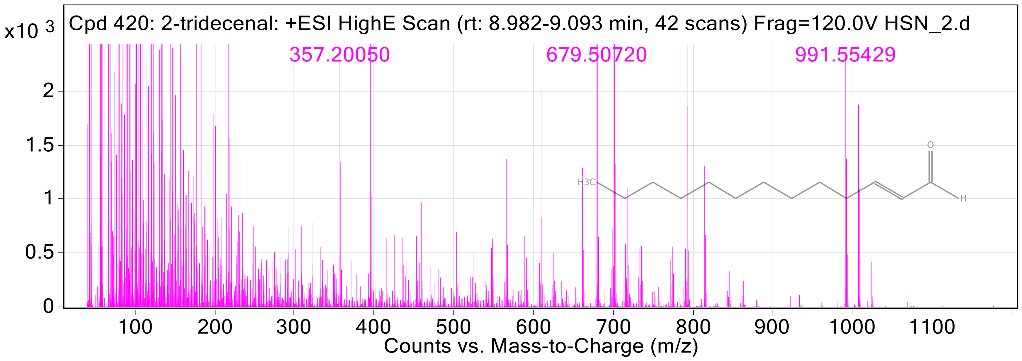

Peak List

m/z	z	Abund
45.03314		23885.7
58.06459		7413.84
59.04857		6871.9
89.05847	1	23544.78
93.06851		6359.56
105.06822		5791.02
133.08487		9999.22
133.09752	1	7987.36
357.2005	1	6364.68
679.5072	1	6516.93

Qualitative Analysis Report

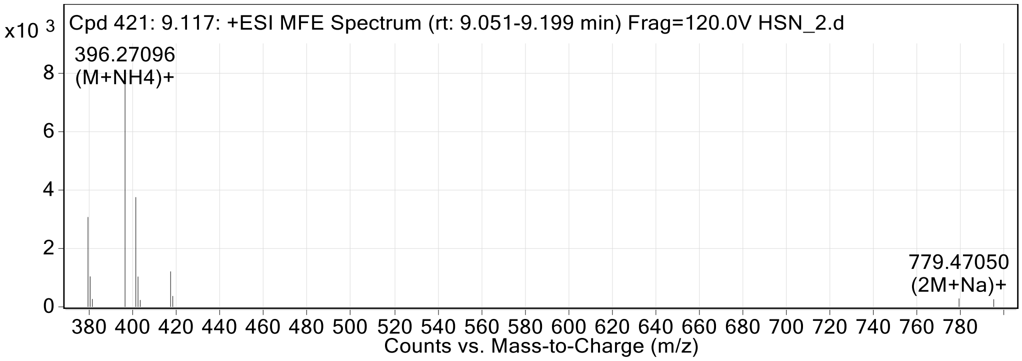

Peak List

m/z	z	Abund	Ion
379.2446	1	3084.28	(M+H)+
380.2485	1	1044.98	(M+H)+
381.2384	1	270.53	(M+H)+
396.27096	1	7826.81	(M+NH4)+
401.2265	1	3766.83	(M+Na)+
402.22971	1	1039.37	(M+Na)+
417.20312	1	1216.8	(M+K)+
418.21086	1	373.43	(M+K)+
779.4705	1	288.16	(2M+Na)+
795.43457	1	262.36	(2M+K)+

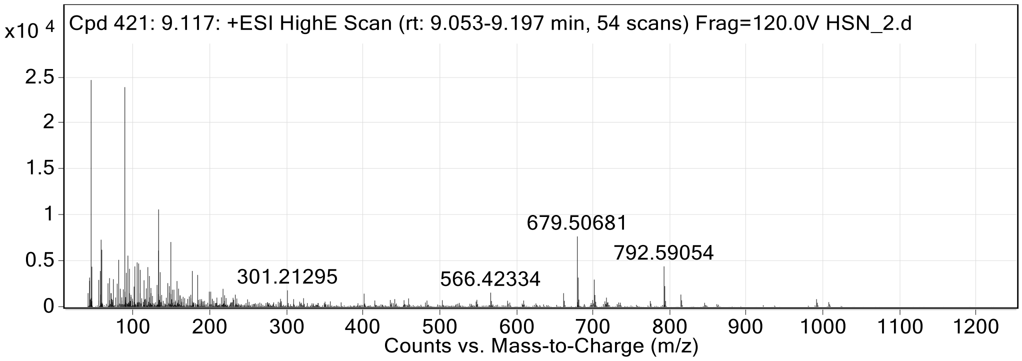

Peak List

m/z	z	Abund
45.03313		24663.35
58.06457		7288.27
59.04855		6197.3
81.06872		5100.2
89.05843	1	23887.68
93.06846		5559.1
133.08468		10585.67
133.09731		6111.34
149.09373		7040.84
679.50681	1	7650.21

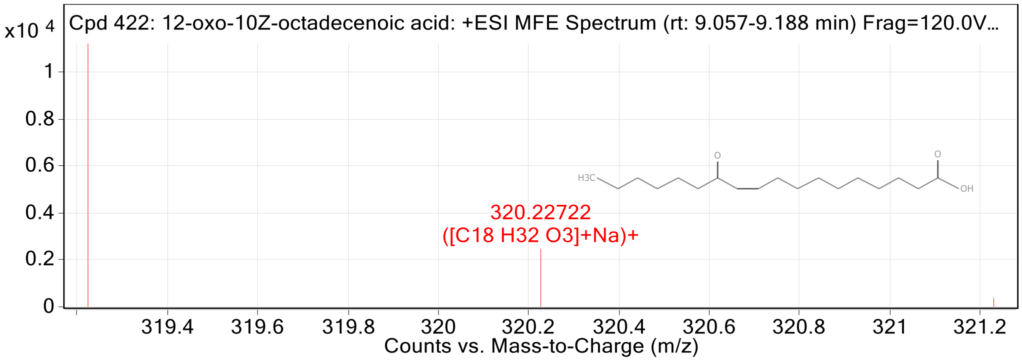

Peak List

m/z	z	Abund	Name	Formula	Ion	Score (DB)	Hits (DB)
319.22384	1	11223.63	12-oxo-10Z-octadecenoic acid	C18 H32 O3	(M+Na)+	97.95	10
320.22722	1	2472.86		C18 H32 O3	(M+Na)+		
321.23096	1	379.6		C18 H32 O3	(M+Na)+		

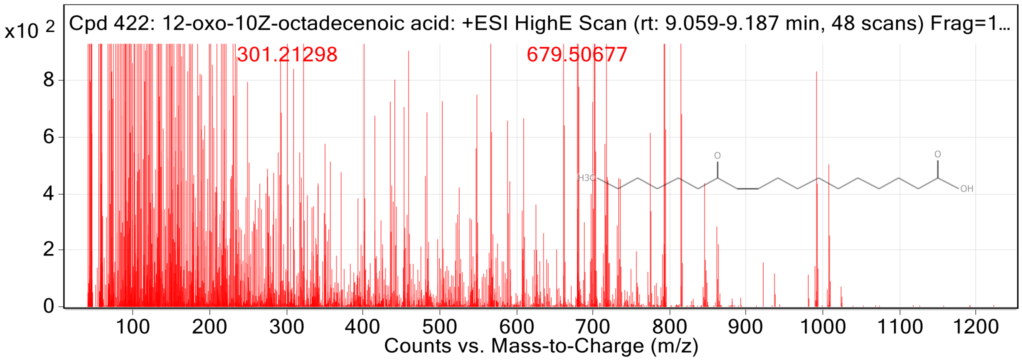

Peak List

m/z	z	Abund
45.03314		24694.18
58.06458		7268.87
59.04854		6310.08
81.06871		5181.51
89.05844	1	23867.01
93.06847		5577.67
133.08469		10649.19
133.0972		5618.82
149.09371		7393.84
679.50677	1	7651.37

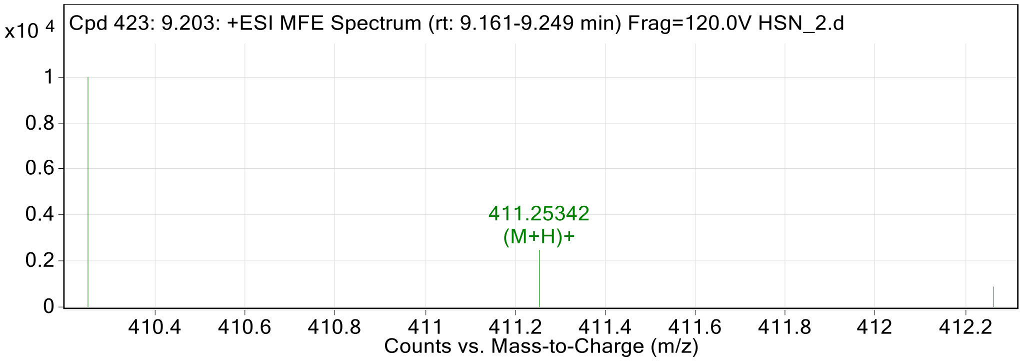

Peak List

m/z	z	Abund	Ion
410.25052	1	9995.05	(M+H)+

411.25342	1	2480.6	(M+H)+
412.26344	1	889.42	(M+H)+

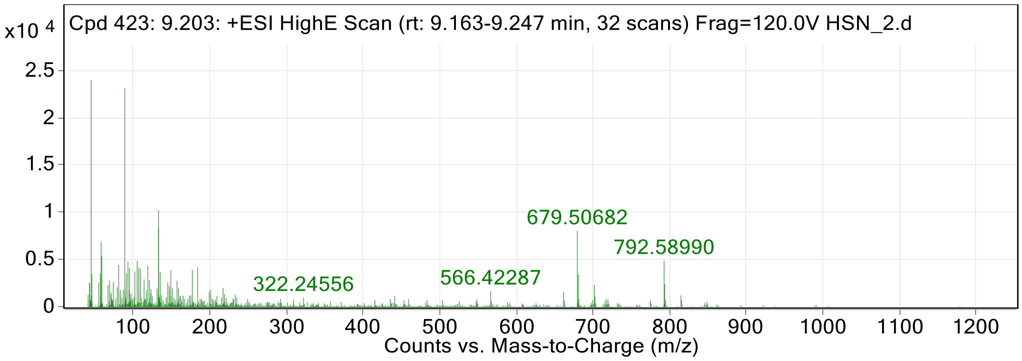

Peak List

m/z	z	Abund
45.03313		23978.68
58.06453		6879.2
59.04853		5348.65
89.05838	1	23110.65
93.06842		4767.84
105.06817		4865.53
133.08471		10191.12
133.09755	1	8326.28
679.50682	1	8019.4
792.5899	1	4869.29

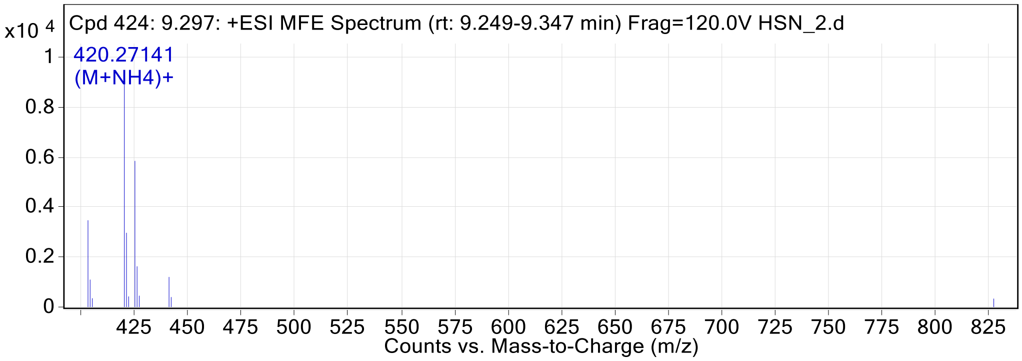

Peak List

m/z	z	Abund	Ion
403.2445	1	3468.63	(M+H)+
404.24693	1	1096.62	(M+H)+
420.27141	1	9125.4	(M+NH4)+
421.27529	1	2968.63	(M+NH4)+
422.27931	1	422.68	(M+NH4)+
425.22694	1	5842.78	(M+Na)+
426.23137	1	1626.52	(M+Na)+
427.23795	1	453.45	(M+Na)+
441.20028	1	1201.28	(M+K)+
442.20607	1	398.16	(M+K)+

Qualitative Analysis Report

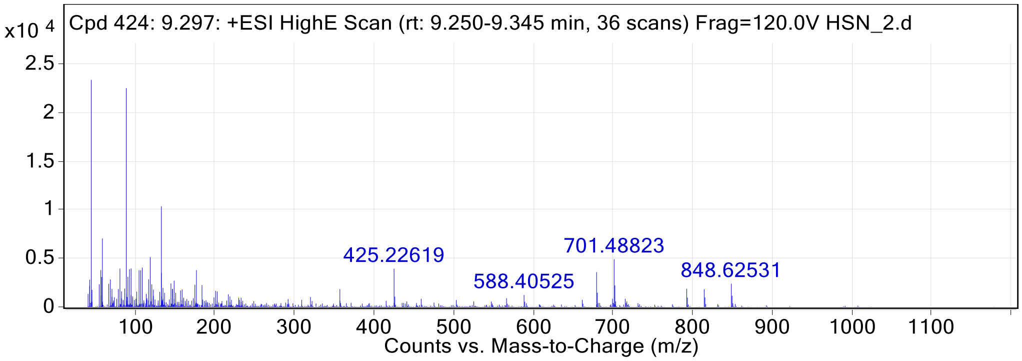

Peak List

m/z	z	Abund
45.03319		23378.47
59.04851		7046.87
81.06884		3943.22
89.05846	1	22528.5
95.08401		3954.25
109.09938		4048.35
119.08352		5124.78
133.08454		10352.21
425.22619	1	3932.67
701.48823	1	4903.25

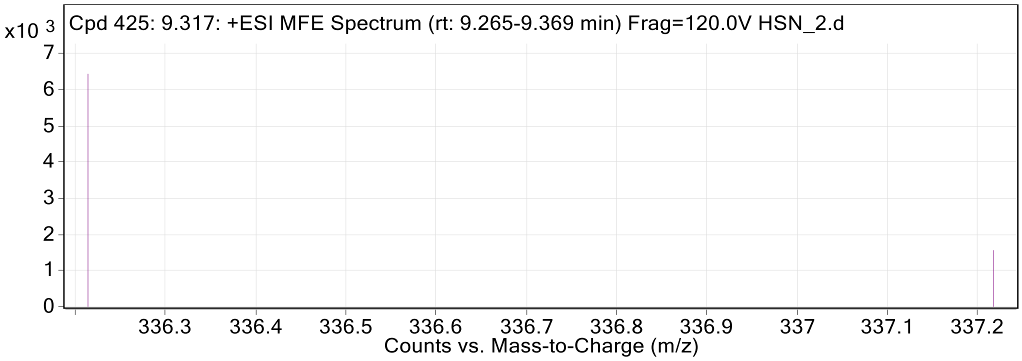

Peak List

m/z	z	Abund	Ion
336.21404	1	6436.82	(M+H)+
337.2181	1	1563.4	(M+H)+

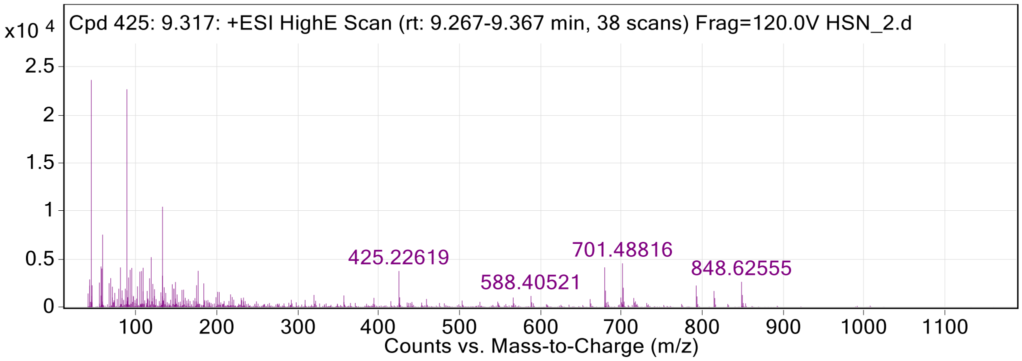

Peak List

m/z	z	Abund
45.03318		23663.48
57.06923		4219.43
59.04852		7537.08

81.06881		4119.56
89.05845	1	22690.3
109.09943		4082.74
119.08354		5189.06
133.0845		10447.61
679.5067	1	4126.57
701.48816	1	4546.11

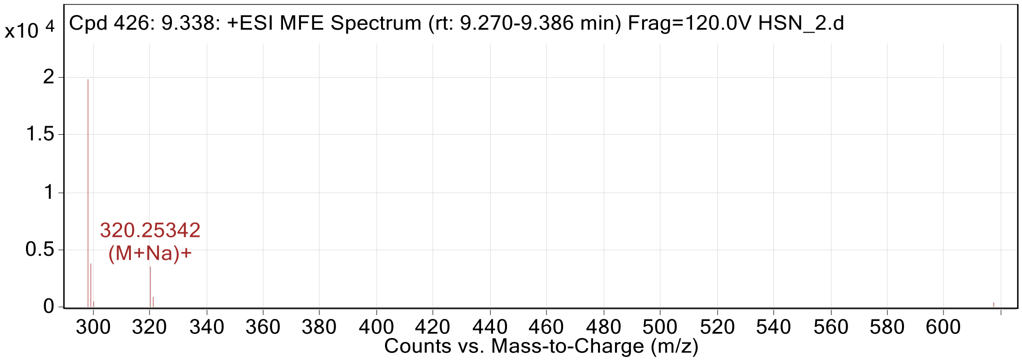

Peak List

m/z	z	Abund	Ion
298.27103	1	19839.03	(M+H)+
299.2745	1	3789.94	(M+H)+
300.27279	1	479.37	(M+H)+
320.25342	1	3506.27	(M+Na)+
321.25561	1	874.87	(M+Na)+
617.52005	1	399.54	(2M+Na)+

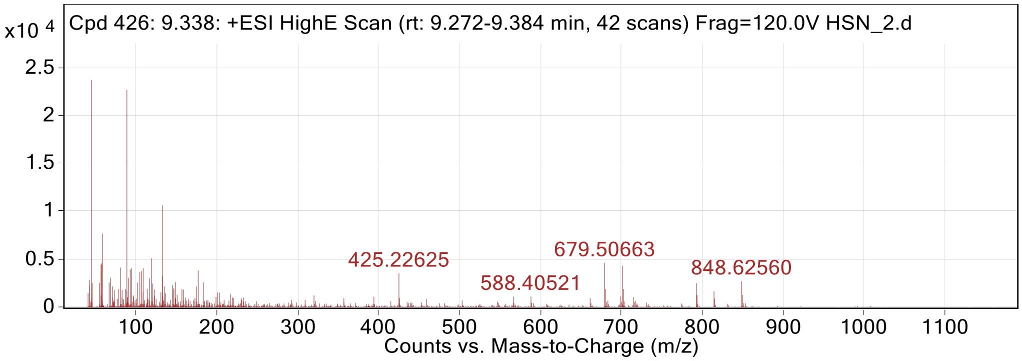

Peak List

m/z	z	Abund
45.03318		23703.72
57.0692		4418.96
58.0646		4571.52
59.04853		7637.38
81.0688		4116.13
89.05845	1	22657.95
119.08352		5081.17
133.08452		10606.46
679.50663	1	4589.82
701.4881	1	4296.42

Qualitative Analysis Report

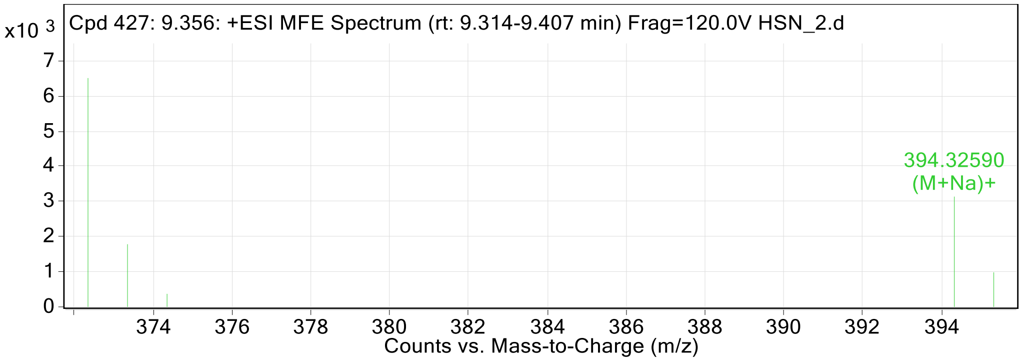

Qualitative Analysis Report

Peak List

m/z	z	Abund	Ion
372.34416	1	6507.72	(M+H)+
373.34685	1	1782.2	(M+H)+
374.35028	1	370.68	(M+H)+
394.3259	1	3136.2	(M+Na)+
395.32716	1	982.46	(M+Na)+

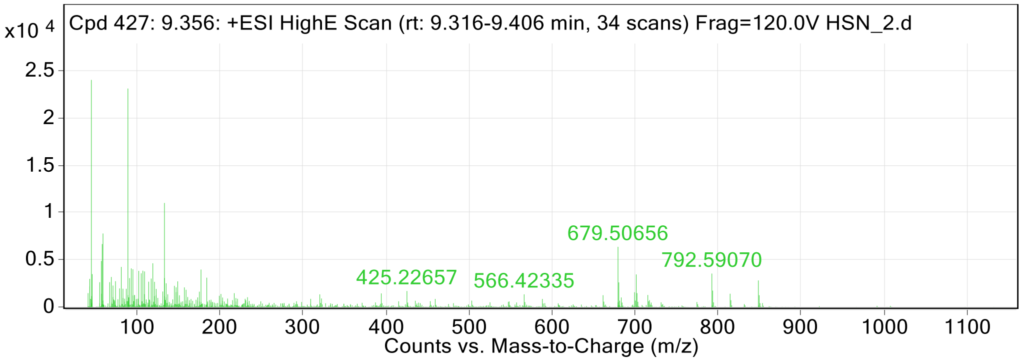

Peak List

m/z	z	Abund
45.03319		24005.08
57.06926		4860.69
58.06462		6648.45
59.04853		7765.54
81.06881		4232.9
89.05843	1	23094.08
93.06853		4075.63
119.08361		4617.38
133.08463		10994.75
679.50656	1	6359.13

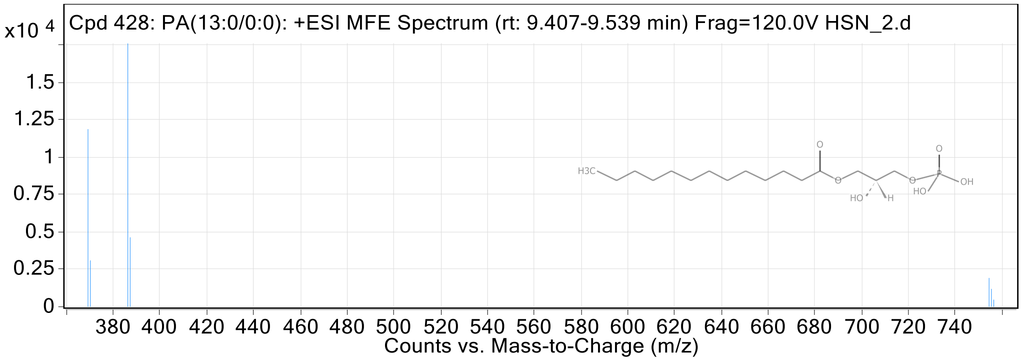

Peak List

m/z	z	Abund	Name	Formula	Ion	Score (DB)	Hits (DB)
369.20311	1	11888.27			(M+H)+		
370.20684	1	3103.28			(M+H)+		
386.22932	1	17633.75	PA(13:0/0:0)	C16 H33 O7 P	(M+NH4)+	71.36	1
387.23351	1	4648.45		C16 H33 O7 P	(M+NH4)+		
754.42674	1	1929.36			(2M+NH4)+		
755.42886	1	1198.02			(2M+NH4)+		
756.43341	1	476.98			(2M+NH4)+		

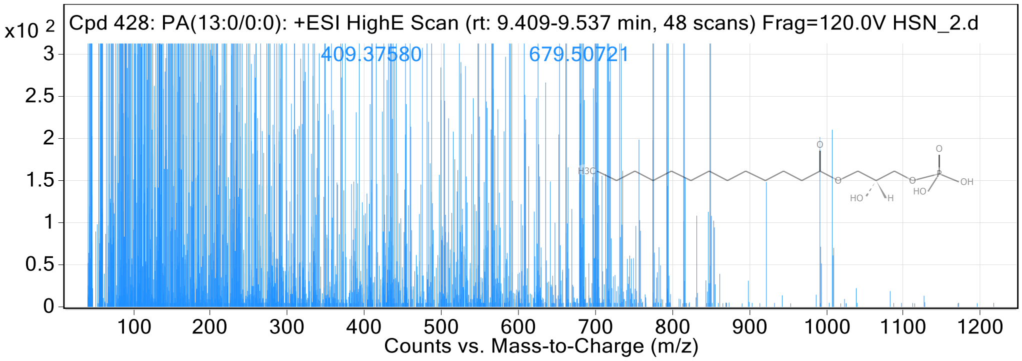

Peak List

m/z	z	Abund
45.0332		25896.48
57.06929		4844.92
58.06457		7423.64
59.04858		5305.92
89.05851	1	24417.61
102.12624		6289.66
107.08384		4737.27
119.08365	1	12402.14
133.08486		11590.12
679.50721	1	7222.55

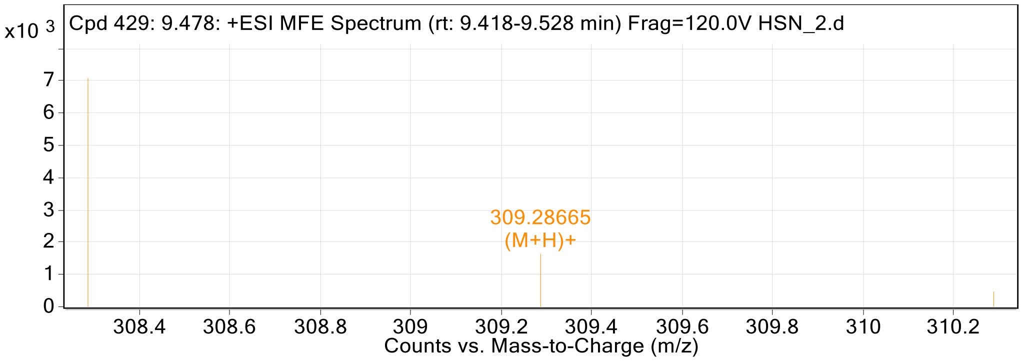

Peak List

m/z	z	Abund	Ion
308.2856	1	7091.09	(M+H)+
309.28665	1	1643.03	(M+H)+
310.28859	1	470.21	(M+H)+

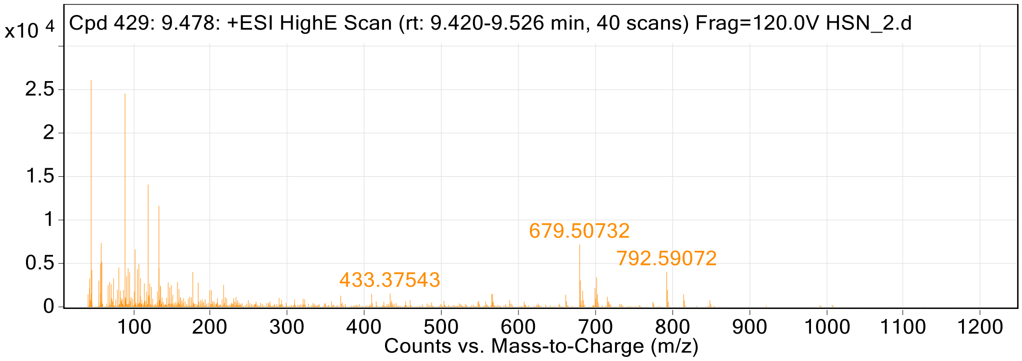

Peak List

m/z	z	Abund
45.03319		26149.86
57.06929		4978.56

58.06458		7368.7
59.04856		5194.86
89.0585	1	24577.3
102.12624		6605.48
107.08382		4947.15
119.08364	1	14085.72
133.08485		11664.97
679.50732	1	7190.4

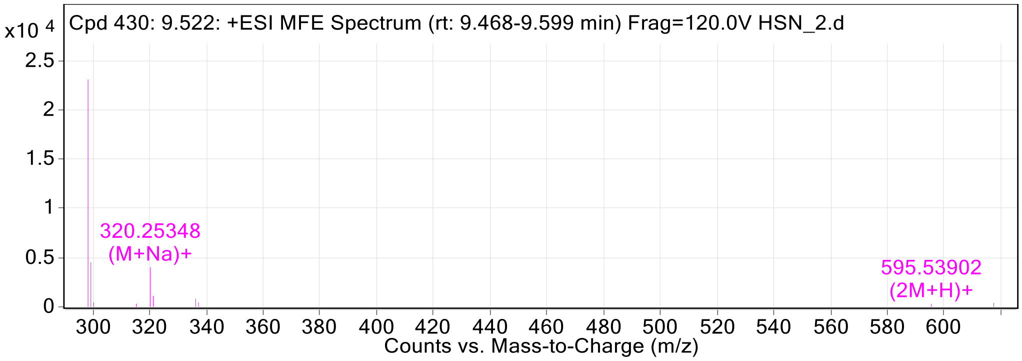

Peak List

m/z	z	Abund	Ion
298.27112	1	23084.82	(M+H)+
299.27472	1	4533.66	(M+H)+
300.27768	1	465.85	(M+H)+
315.30159	1	314.6	(M+NH4)+
320.25348	1	4009.08	(M+Na)+
321.25592	1	1114.11	(M+Na)+
336.22681	1	834.97	(M+K)+
337.23114	1	458.52	(M+K)+
595.53902	1	301.46	(2M+H)+
617.51747	1	405.12	(2M+Na)+

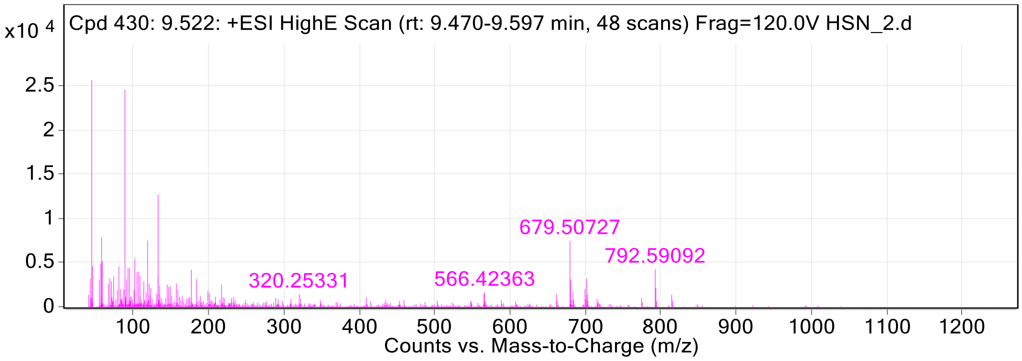

Peak List

m/z	z	Abund
45.03318		25629.92
57.06931		4888.4
58.06456		7866.27
59.04863		5152.42
81.06882		4554.69
89.05853	1	24550.58
102.12627		5416.53
119.08373		7445.38
133.08465		12670.53
679.50727	1	7451.03

Qualitative Analysis Report

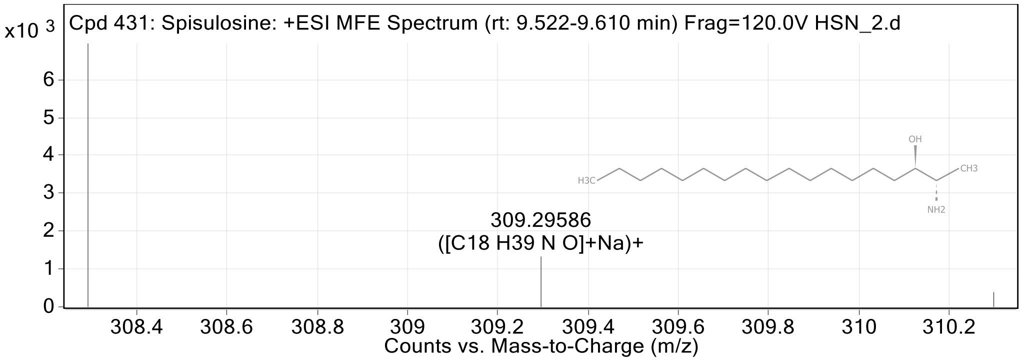

Peak List

m/z	z	Abund	Name	Formula	Ion	Score (DB)	Hits (DB)
308.29193	1	6953.47	Spisulosine	C18 H39 N O	(M+Na)+	94.78	1
309.29586	1	1330.65		C18 H39 N O	(M+Na)+		
310.29901	1	389.74		C18 H39 N O	(M+Na)+		

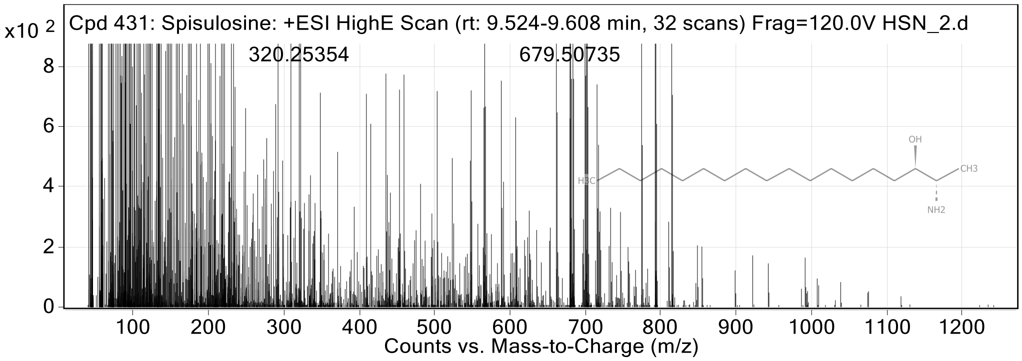

Peak List

m/z	z	Abund
45.03318		25142.05
57.06929		4425.9
58.0646		8197.63
59.04867		5263.92
89.05852	1	24610.78
102.1262		4683.97
133.08457	1	12864.54
177.11044		4337.6
679.50735	1	7617.14
792.59111	1	4382.93

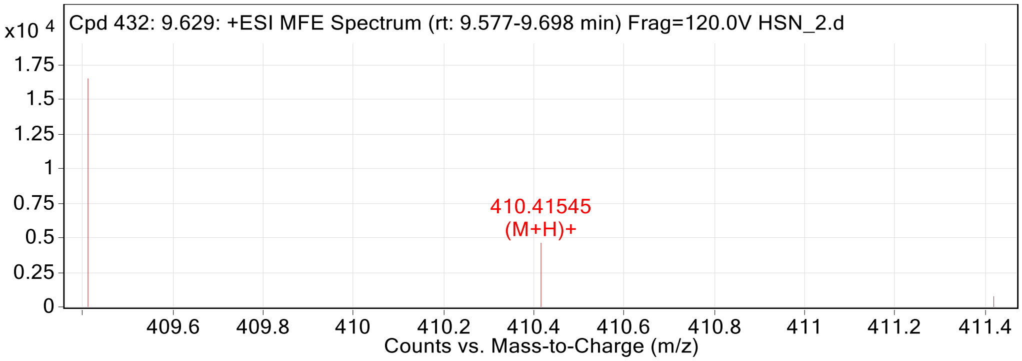

Peak List

m/z	z	Abund	Ion
409.41154	1	16514.63	(M+H)+
410.41545	1	4626.04	(M+H)+

411.41886	1	770.29	(M+H)+

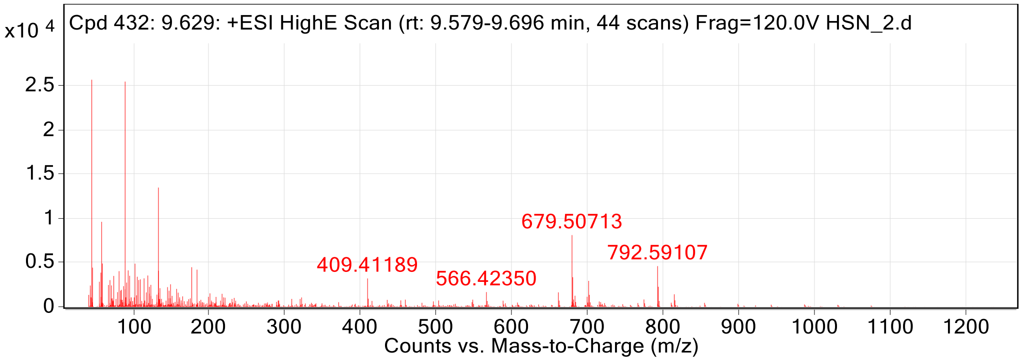

Peak List

m/z	z	Abund
45.03315		25630.74
46.0648		4416.68
58.06458		9589.93
59.04862		4879.62
89.0585	1	25423.32
102.12622		4862.49
133.08475	1	13456.08
177.11016		4465.89
679.50713	1	8090.63
792.59107	1	4589.06

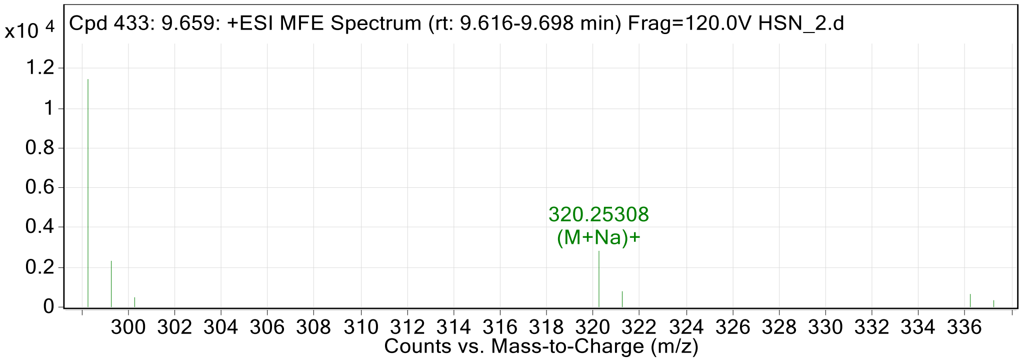

Peak List

m/z	z	Abund	Ion
298.2713	1	11468.15	(M+H)+
299.27435	1	2326.17	(M+H)+
300.27984	1	493.08	(M+H)+
320.25308	1	2828.6	(M+Na)+
321.25329	1	795.66	(M+Na)+
336.22601	1	657.41	(M+K)+
337.2362	1	344.69	(M+K)+

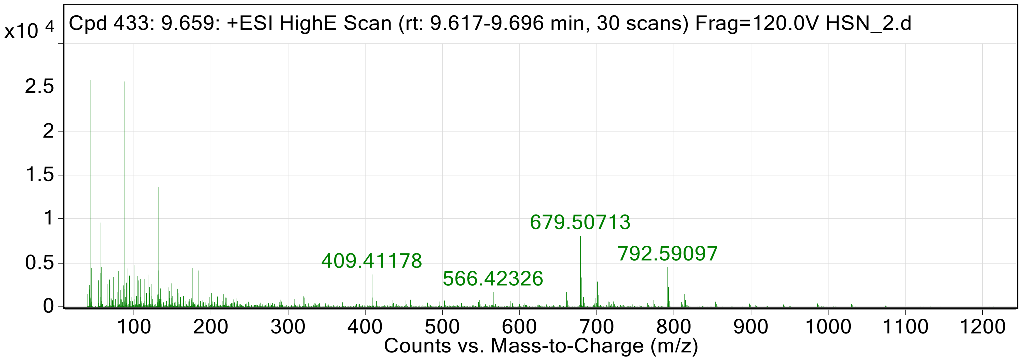

Peak List

m/z	z	Abund
45.03314		25860.03
46.06483		4413.04
58.06454		9593.31
59.04863		4543.98
89.0585	1	25690.76
102.12631		4722.97
133.08505	1	13678.92
177.11002		4412.23
679.50713	1	8086.06
792.59097	1	4509.24

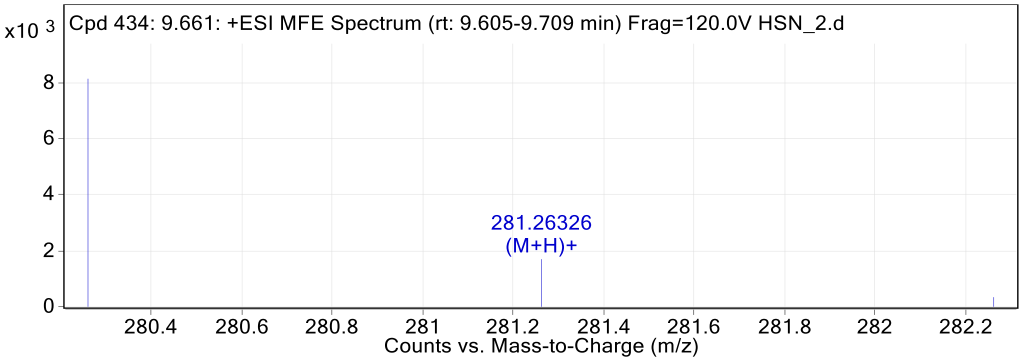

Peak List

m/z	z	Abund	Ion
280.26076	1	8133.76	(M+H)+
281.26326	1	1698.02	(M+H)+
282.26241	1	342.51	(M+H)+

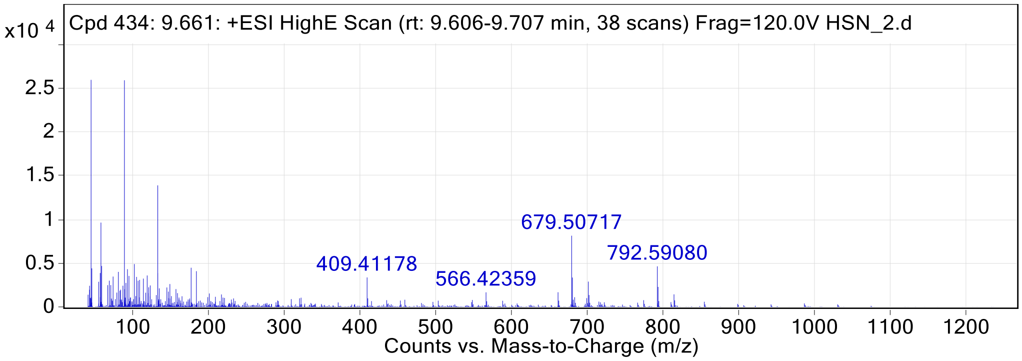

Peak List

m/z	z	Abund
45.03315		25963.24
46.06482		4409.04
58.06457		9641.83
59.04861		4679.66
89.0585	1	25917.1
102.12624		4900.26
133.08496	1	13896.73
177.11008		4479.91
679.50717	1	8143
792.5908	1	4636.99

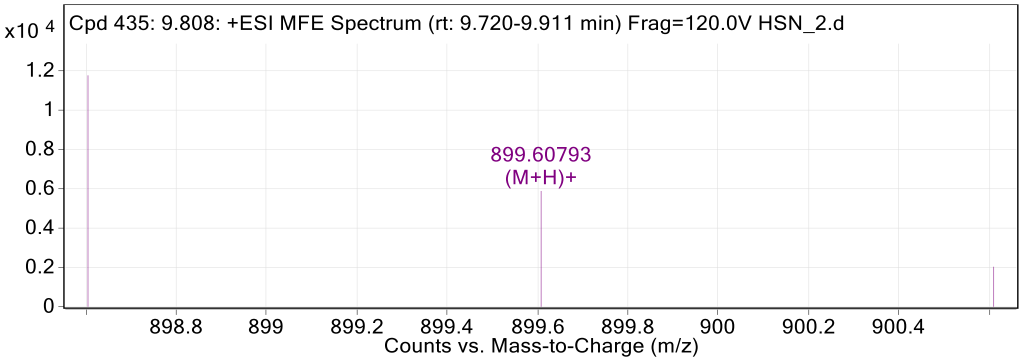

Peak List

m/z	z	Abund	Ion
898.60467	1	11754.9	(M+H)+
899.60793	1	5881.5	(M+H)+
900.61032	1	2033.22	(M+H)+

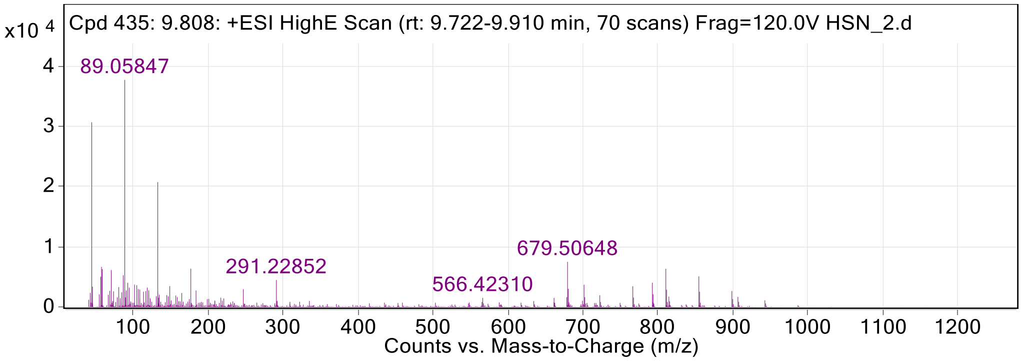

Peak List

m/z	z	Abund
45.03317		30721.19
58.06462		6662.59
59.04857		6282.48
71.08465		6134.06
87.04282		5264.33
89.05847	1	37776.98
133.08469	1	20767.95
177.10998		6351.67
679.50648	1	7503.59
810.55249	1	6336.72

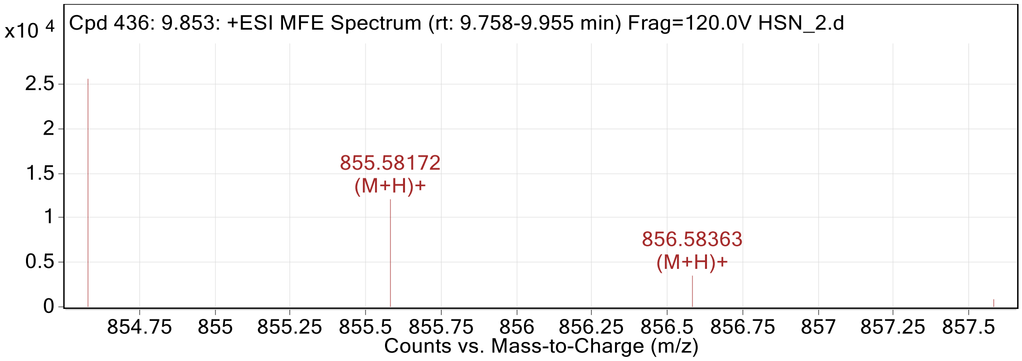

Peak List

m/z	z	Abund	Ion
854.57834	1	25611.03	(M+H)+
855.58172	1	12082.39	(M+H)+
856.58363	1	3508.06	(M+H)+
857.58338	1	858.83	(M+H)+

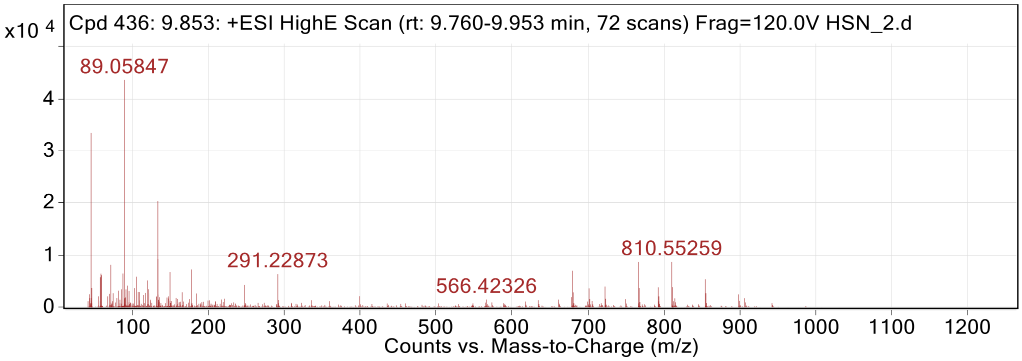

Peak List

m/z	z	Abund
45.03317		33436.73
71.08466		8116.44
89.05847	1	43619.18
133.08527	1	20315.34
133.09723	1	9253.01
149.09395		6735.19
177.1099		7208.69
679.50629	1	6976.55
766.52645	1	8680.15
810.55259	1	8680.92

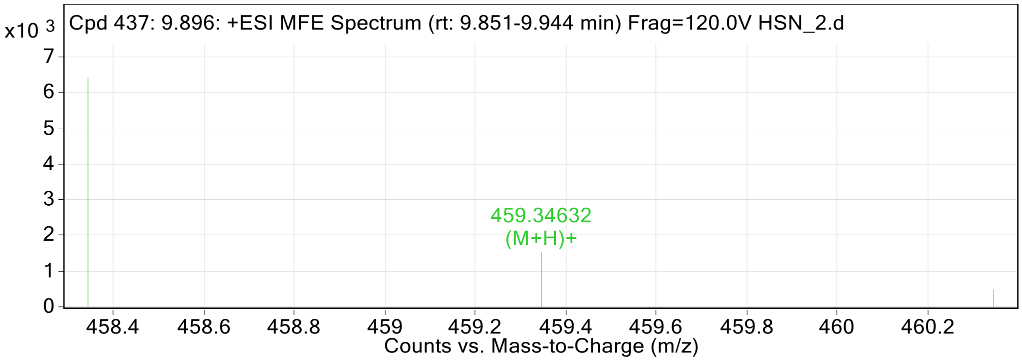

Peak List

m/z	z	Abund	Ion
458.34395	1	6434.14	(M+H)+

459.34632	1	1541.64	(M+H)+
460.34625	1	483.45	(M+H)+

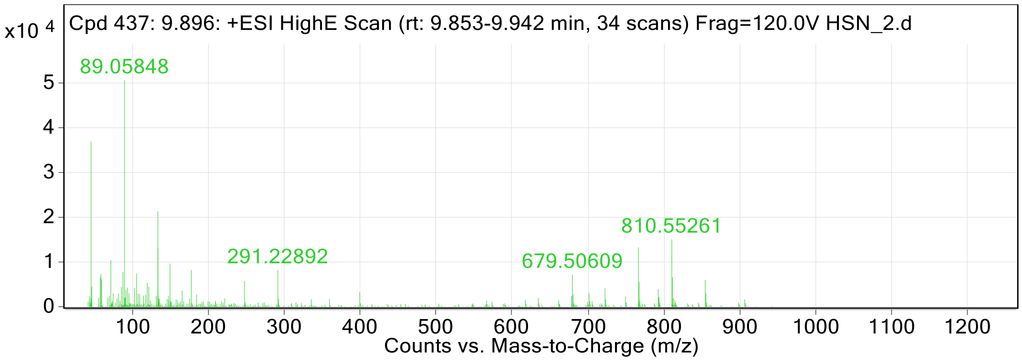

Peak List

m/z	z	Abund
45.03316		36847.11
71.08466		10388.65
89.05848	1	50547.85
133.08568	1	21242.84
133.09692	1	13164.38
149.09395		9602.95
177.10983		8215.28
291.22892	1	8197.37
766.52653	1	13255.53
810.55261	1	15028.02

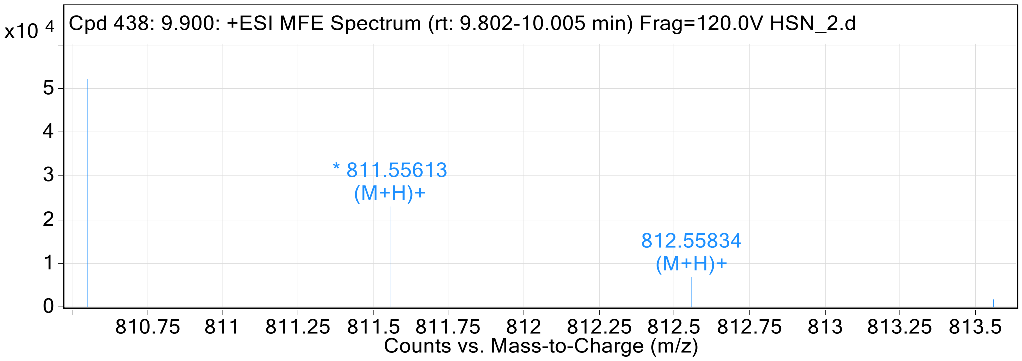

Peak List

m/z	z	Abund	Ion
810.55201	1	52176.31	(M+H)+
811.55613	1	22985.82	(M+H)+
812.55834	1	6814.55	(M+H)+
813.56082	1	1708.72	(M+H)+

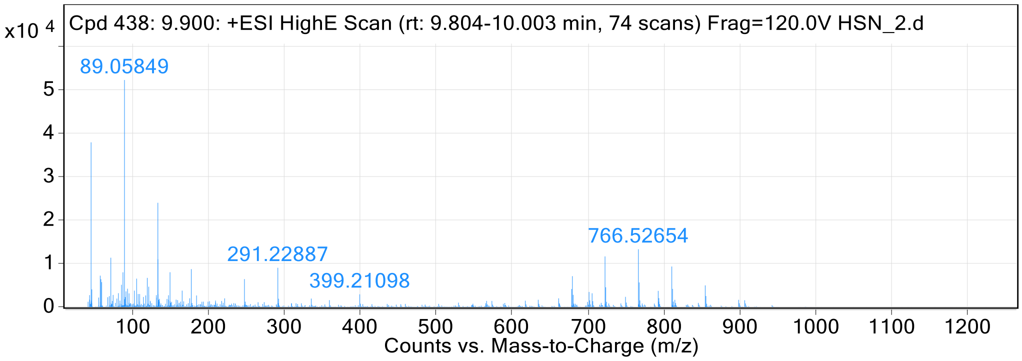

Peak List

m/z	z	Abund
45.03318		37836.14
71.08468		11280.74
89.05849	1	52174.03
133.08519	1	23922.63
133.09698	1	10935.77
177.1099		8653.22
291.22887	1	8964.22
722.50036	1	11581.37
766.52654	1	13223.33
810.55256	1	9256.75

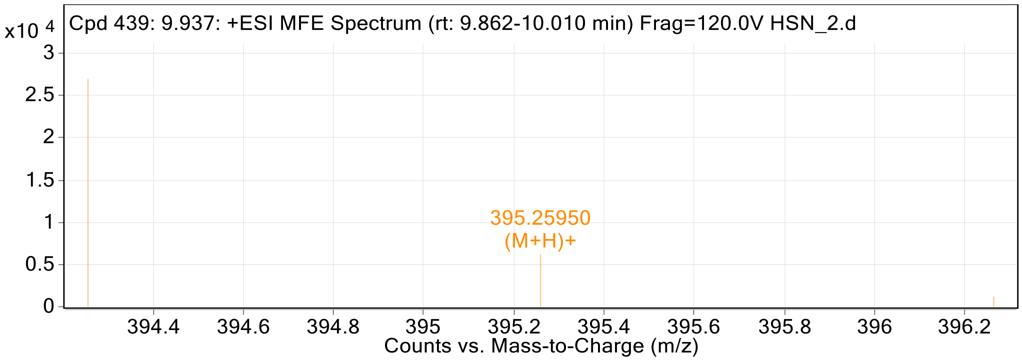

Peak List

m/z	z	Abund	Ion
394.25555	1	27000.58	(M+H)+
395.2595	1	6213.39	(M+H)+
396.26468	1	1179.31	(M+H)+

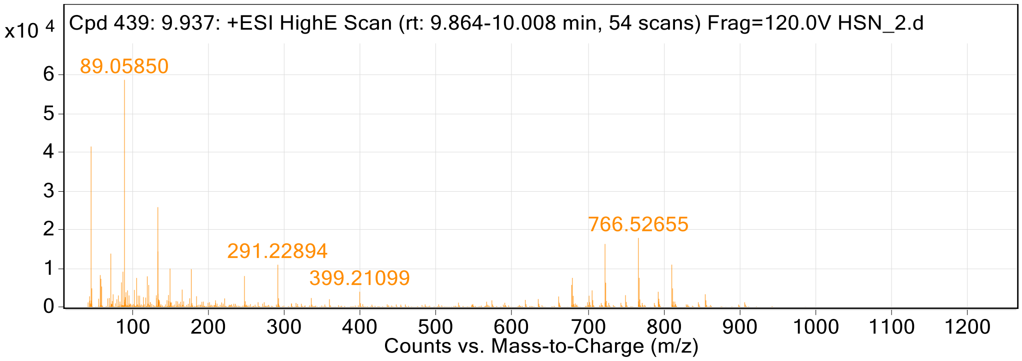

Peak List

m/z	z	Abund
45.03318		41526.35
71.08468		13794.72
89.0585	1	58786.54
133.08531	1	25823.93
133.09691	1	14345.81
149.09397		9906.67
291.22894	1	10919.8
722.50039	1	16295.39
766.52655	1	17857.04
810.55255	1	10935.93

Qualitative Analysis Report

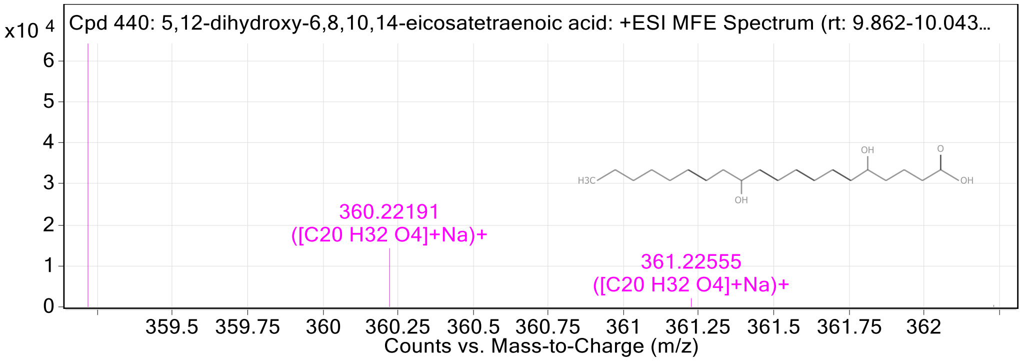

Peak List

m/z	z	Abund	Name	Formula	Ion	Score (DB)	Hits (DB)
359.21867	1	64280.04	5,12-dihydroxy-6,8,10,14-eicosatetraenoic acid	C20 H32 O4	(M+Na)+	98.77	10
360.22191	1	14309.45		C20 H32 O4	(M+Na)+		
361.22555	1	2128.7		C20 H32 O4	(M+Na)+		
362.23166	1	475.06		C20 H32 O4	(M+Na)+		

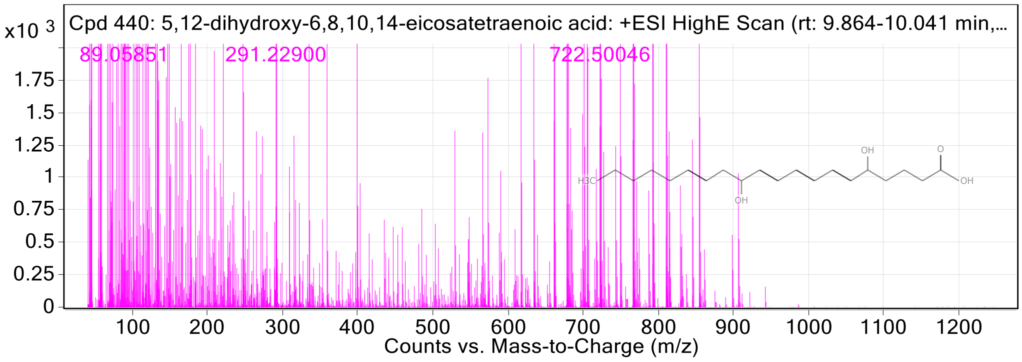

Peak List

m/z	z	Abund
45.0332		44246.96
71.08469		15979.29
89.05851	1	63235.12
133.08496	1	29981.35
133.09699	1	11962.79
177.10992		10612.27
291.229	1	12194.39
678.47421		10958.1
722.50046	1	18871.49
766.5265	1	15978.15

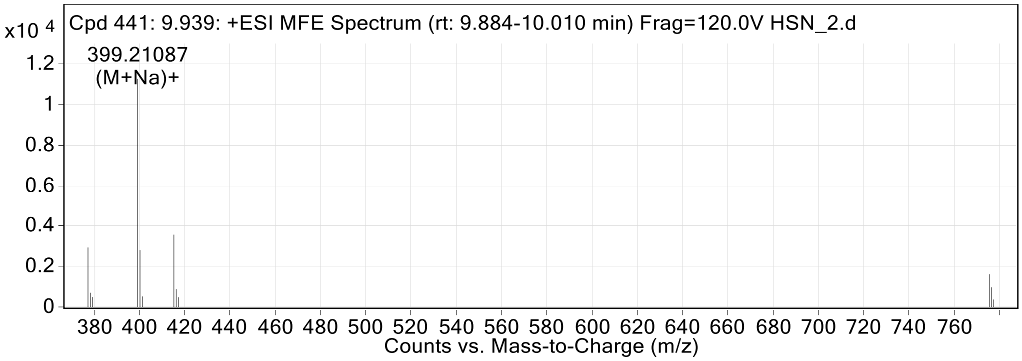

Peak List

m/z	z	Abund	Ion

377.22906	1	2944.29	(M+H)+
378.23356	1	704.31	(M+H)+
379.23973	1	490.73	(M+H)+
399.21087	1	11288.71	(M+Na)+
400.21547	1	2817.85	(M+Na)+
401.218	1	520.47	(M+Na)+
415.18473	1	3580.2	(M+K)+
416.18876	1	883.36	(M+K)+
775.43424	1	1620.66	(2M+Na)+
776.4366	1	973.88	(2M+Na)+

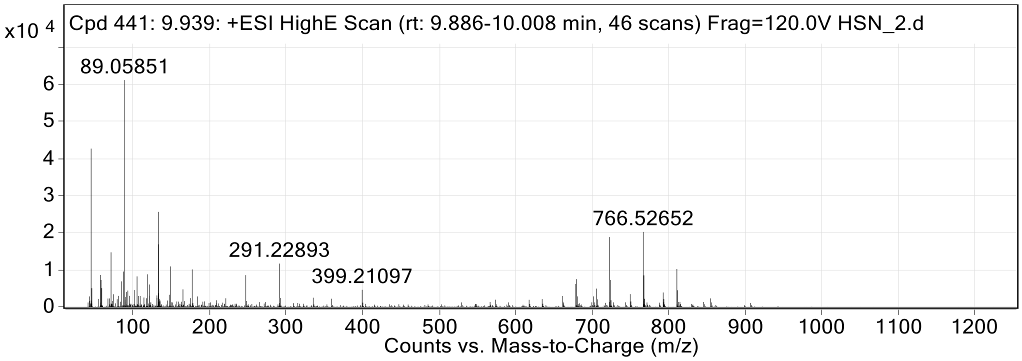

Peak List

m/z	z	Abund
45.03319		42656.24
71.08468		14697.69
89.05851	1	61143.73
133.08547	1	25597.89
133.09691	1	16840.74
149.09396		10890.77
291.22893	1	11651.71
722.5004	1	18799.97
766.52652	1	20194.01
810.55242	1	10189.95

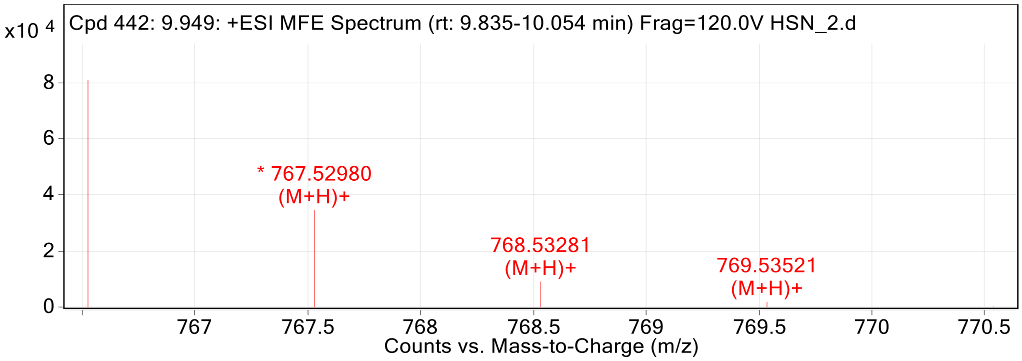

Peak List

m/z	z	Abund	Ion
766.52637	1	80926.92	(M+H)+
767.5298	1	34541.15	(M+H)+
768.53281	1	9065.45	(M+H)+
769.53521	1	1840.78	(M+H)+
770.54016	1	163.51	(M+H)+

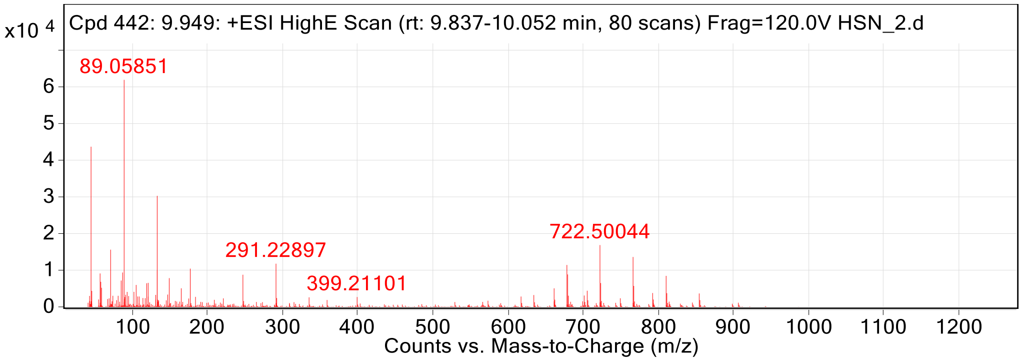

Peak List

m/z	z	Abund
45.0332		43621.68
71.08469		15575.75
89.05851	1	61815.92
133.08485	1	30231.92
133.09702	1	9930.14
177.10991		10411.74
291.22897	1	11759.18
678.47428	1	11410.27
722.50044	1	16833.32
766.52651	1	13585.68

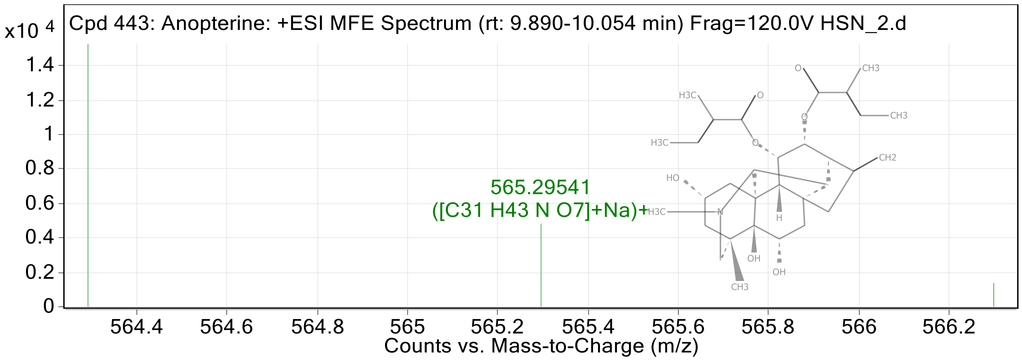

Peak List

m/z	z	Abund	Name	Formula	Ion	Score (DB)	Hits (DB)
564.29165	1	15287.34	Anopterine	C31 H43 N O7	(M+Na)+	93.59	1
565.29541	1	4829.28		C31 H43 N O7	(M+Na)+		
566.2985	1	1382.73		C31 H43 N O7	(M+Na)+		

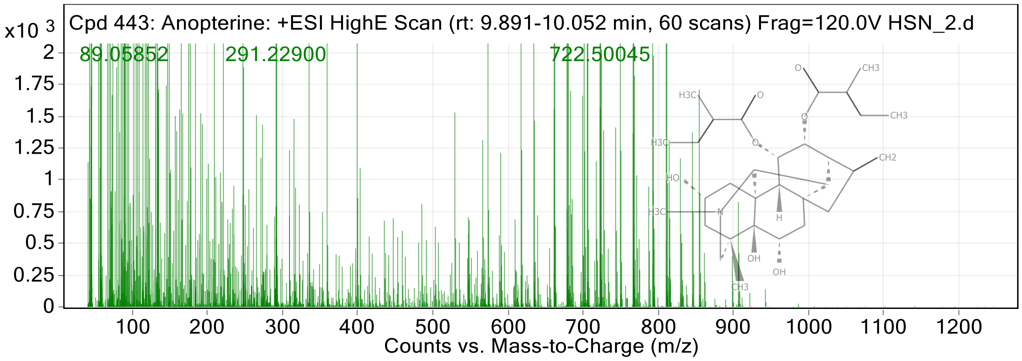

Peak List

m/z	z	Abund
45.0332		47011.71
71.08469		18188.47

89.05852	1	67816.87
133.08493	1	32025.29
133.09702	1	13240.18
177.10993		11434.66
291.229	1	13602.21
678.47426	1	14320.12
722.50045	1	21660.63
766.52649	1	16964.02

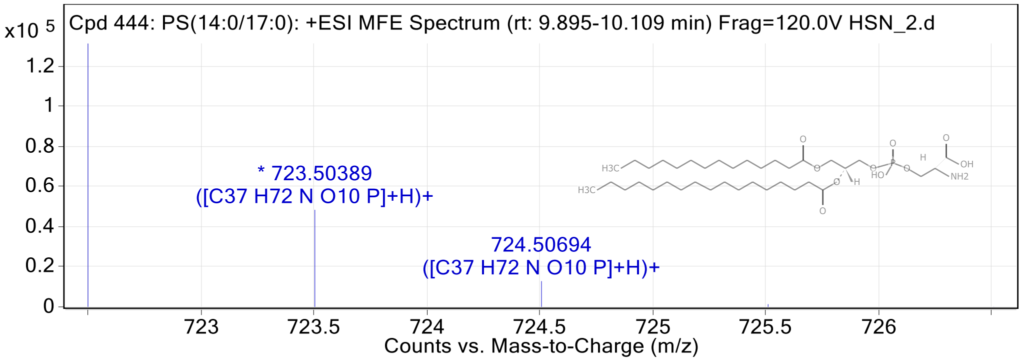

Peak List

m/z	z	Abund	Name	Formula	Ion	Score (DB)	Hits (DB)
722.49969	1	131339.36	PS(14:0/17:0)	C37 H72 N O10 P	(M+H)+	84.89	8
723.50389	1	48379.22		C37 H72 N O10 P	(M+H)+		
724.50694	1	12827.59		C37 H72 N O10 P	(M+H)+		
725.5099	1	1336.74		C37 H72 N O10 P	(M+H)+		
726.50879	1	57.14		C37 H72 N O10 P	(M+H)+		

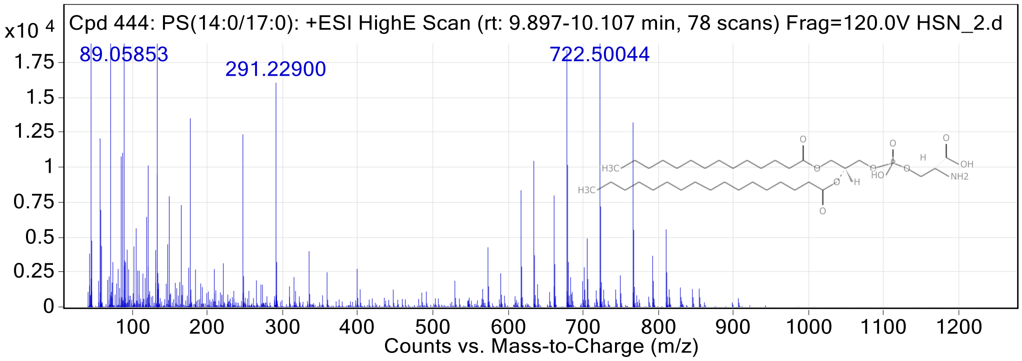

Peak List

m/z	z	Abund
45.03322		52249.7
71.08471		21969.55
89.05853	1	75235.95
133.08476	1	39462.72
177.10997		13502.99
247.203	1	12345.24
291.229	1	16047.65
678.47434	1	18381.12
722.50044	1	18851.14
766.52649	1	13200.86

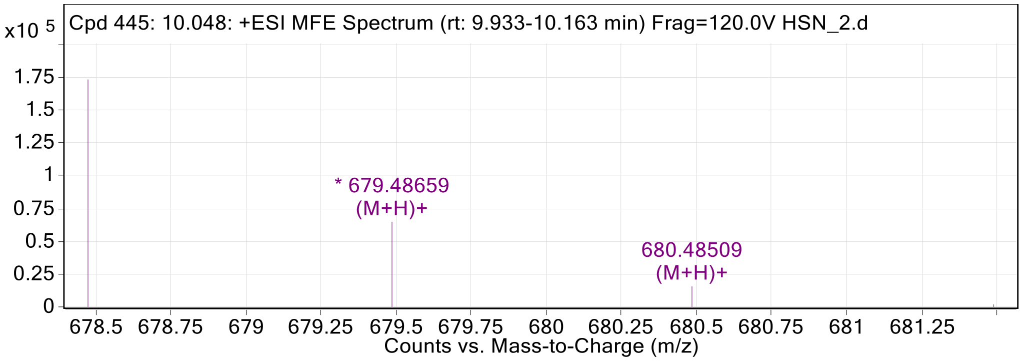

Peak List

m/z	z	Abund	Ion
678.47474	1	173381.14	(M+H)+
679.48659	1	64812.28	(M+H)+
680.48509	1	15677.39	(M+H)+
681.4896	1	1894.65	(M+H)+

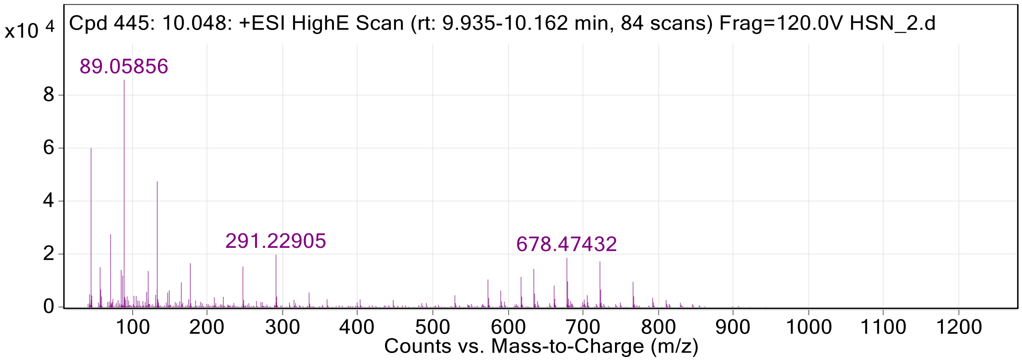

Peak List

m/z	z	Abund
45.03324		59910.5
57.06932		14984.28
71.0847		27416.58
89.05856	1	85539.38
133.08421	1	47324.38
177.11001		16465.75
247.20305	1	15234.91
291.22905	1	19754.52
678.47432	1	18547.44
722.50047	1	17147.6

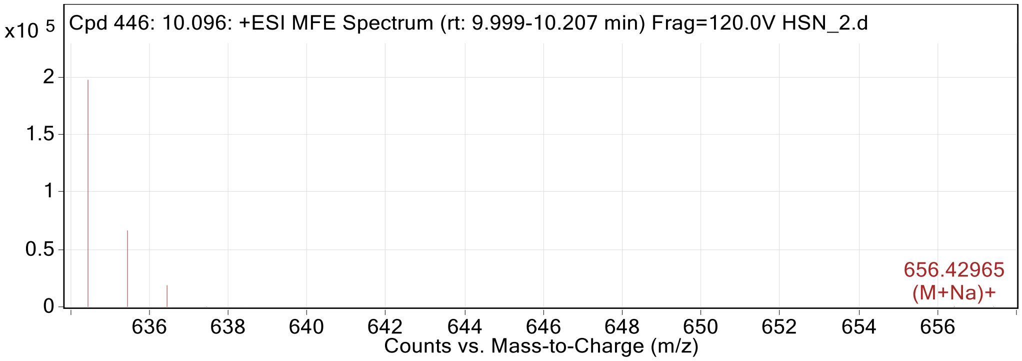

Peak List

m/z	z	Abund	Ion
634.44904	1	197351.14	(M+H)+

635.45198	1	66388.07	(M+H)+
636.45444	1	18788.8	(M+H)+
637.45762	1	286.07	(M+H)+
638.45904	1	49.12	(M+H)+
656.42965	1	549.19	(M+Na)+
657.42867	1	347.57	(M+Na)+

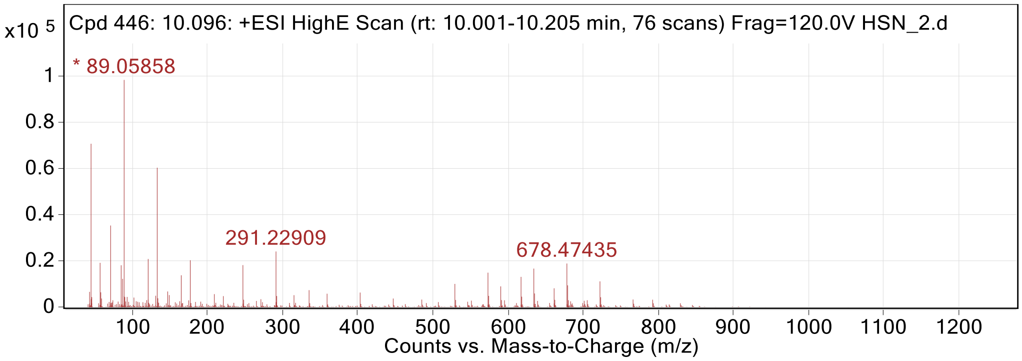

Peak List

m/z	z	Abund
45.03326		70753.5
57.06934		19126.18
71.08472	1	35299.51
89.05858	1	98407.75
121.06294	1	20793.51
133.08414	1	60361.88
177.11007	1	20245.35
247.20304	1	18141.17
291.22909	1	24050.65
678.47435	1	18834.22

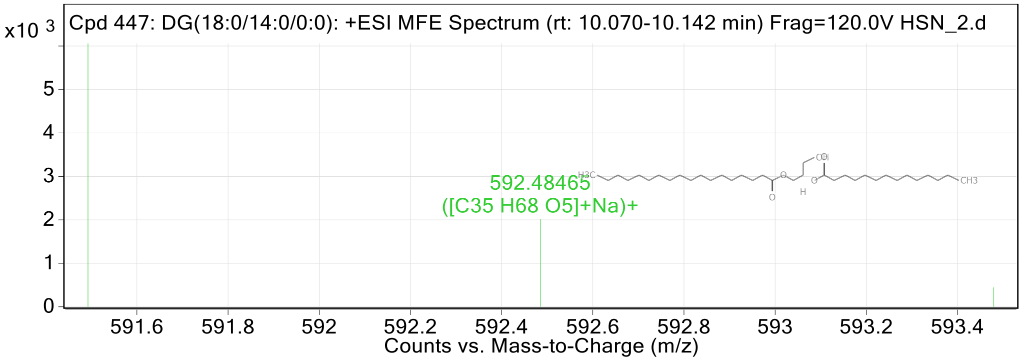

Peak List

m/z	z	Abund	Name	Formula	Ion	Score (DB)	Hits (DB)
591.49373	1	6064.64	DG(18:0/14:0/0:0)	C35 H68 O5	(M+Na)+	22.21	7
592.48465	1	2015.06		C35 H68 O5	(M+Na)+		
593.47808	1	446.01		C35 H68 O5	(M+Na)+		

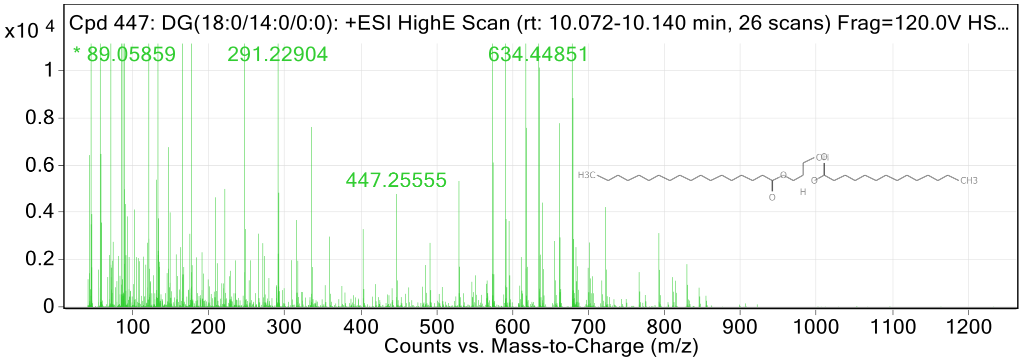

Peak List

m/z	z	Abund
45.03326		70991.07
71.0847		34759.32
89.05859	1	99787.36
133.08411	1	60957.6
177.11002	1	20507.37
247.20318	1	19280.66
291.22904	1	24559.95
573.39549	1	19206.07
617.42192	1	22200.58
634.44851	1	29214.28

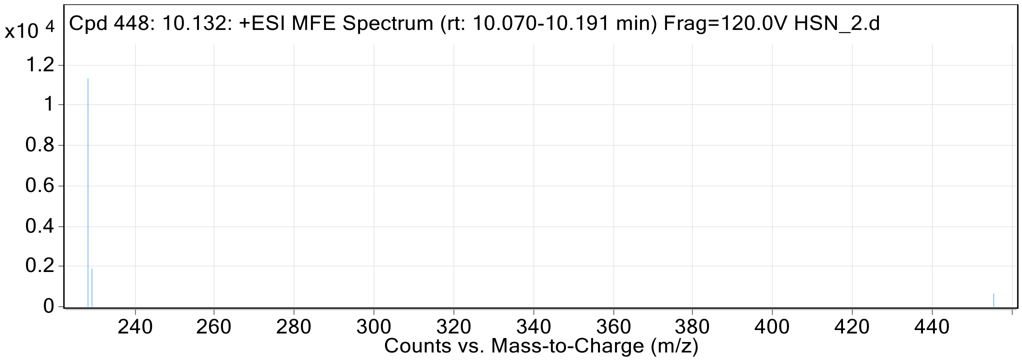

Peak List

m/z	z	Abund	Ion
228.22973	1	11333.22	(M+H)+
229.23298	1	1894.78	(M+H)+
455.45181	1	656.28	(2M+H)+

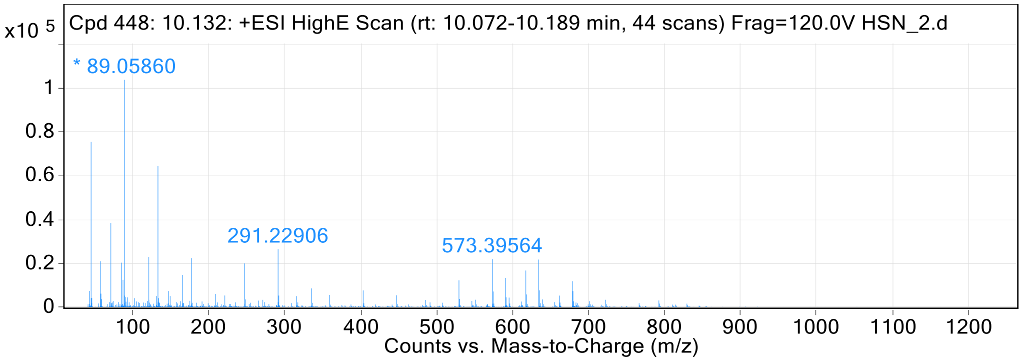

Peak List

m/z	z	Abund
45.03327	1	75581.53
57.06935		20885.96

71.08472	1	38439.65
89.0586	1	103917.51
121.06295	1	22884.02
133.08414	1	64535.81
177.11008	1	22346.06
291.22906	1	26356.99
573.39564	1	21866.63
634.44847	1	21666.62

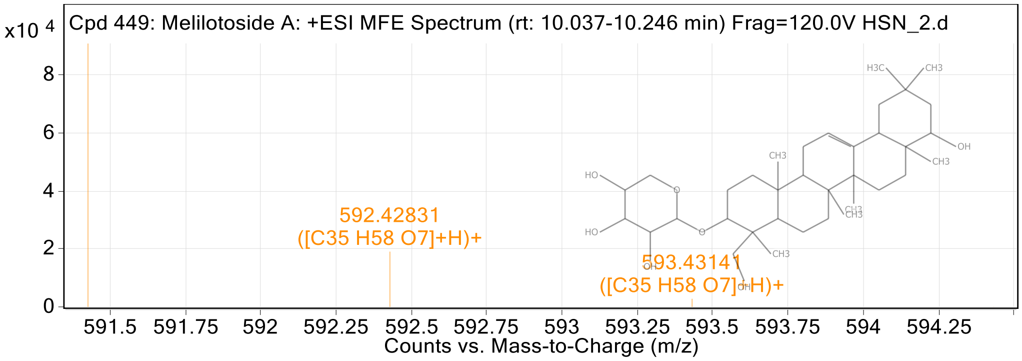

Peak List

m/z	z	Abund	Name	Formula	Ion	Score (DB)	Hits (DB)
591.42567	1	90833.95	Melilotoside A	C35 H58 O7	(M+H)+	76.28	5
592.42831	1	19053.46		C35 H58 O7	(M+H)+		
593.43141	1	2794.26		C35 H58 O7	(M+H)+		
594.43325	1	34.77		C35 H58 O7	(M+H)+		

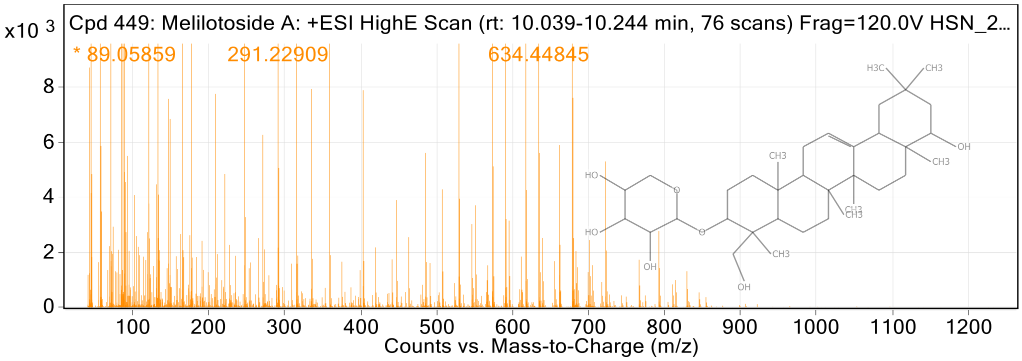

Peak List

m/z	z	Abund
45.03329	1	79058.1
57.06936		22683.58
71.08472	1	41703.34
85.10004		21503.72
89.05859	1	106293.13
121.06294	1	29227.96
133.08415	1	65590.47
177.11006	1	21951.01
247.20307	1	19078.1
291.22909	1	26154.18

Qualitative Analysis Report

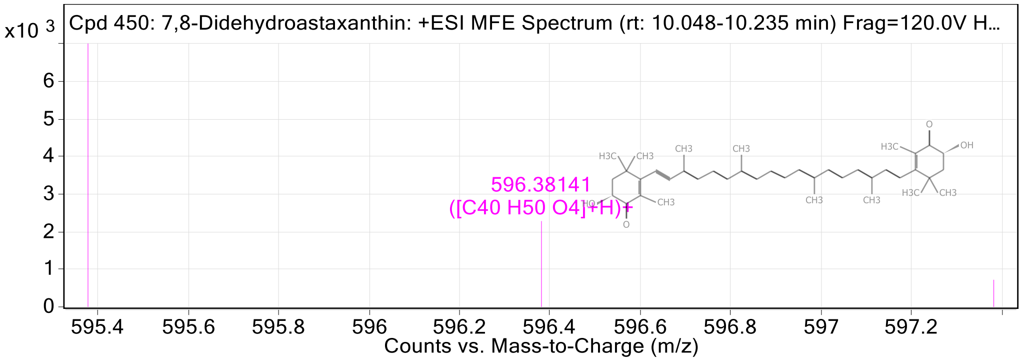

Peak List

m/z	z	Abund	Name	Formula	Ion	Score (DB)	Hits (DB)
595.37786	1	7009.38	7,8-Didehydroastaxanthin	C40 H50 O4	(M+H)+	86.5	3
596.38141	1	2282.59		C40 H50 O4	(M+H)+		
597.38239	1	721.2		C40 H50 O4	(M+H)+		

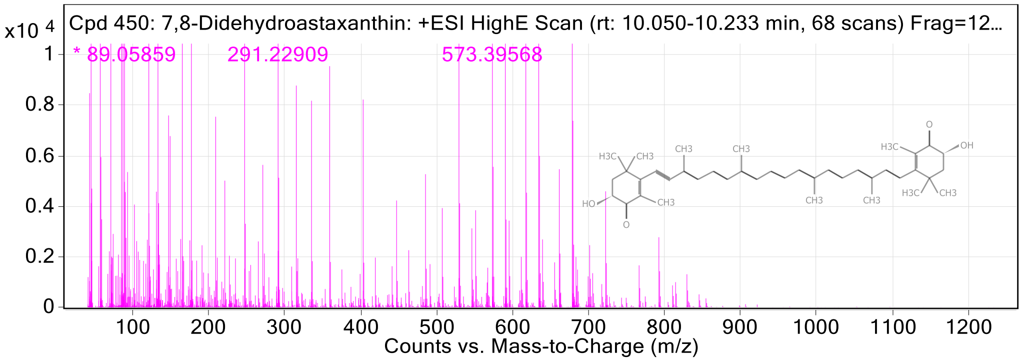

Peak List

m/z	z	Abund
45.03329	1	78869.81
57.06936		22438.38
71.08471	1	41332.41
85.10005		21411.96
89.05859	1	106506.38
121.06293	1	28490.79
133.08414	1	65817.28
177.11007	1	22363.21
247.20308	1	19378.13
291.22909	1	26472.38

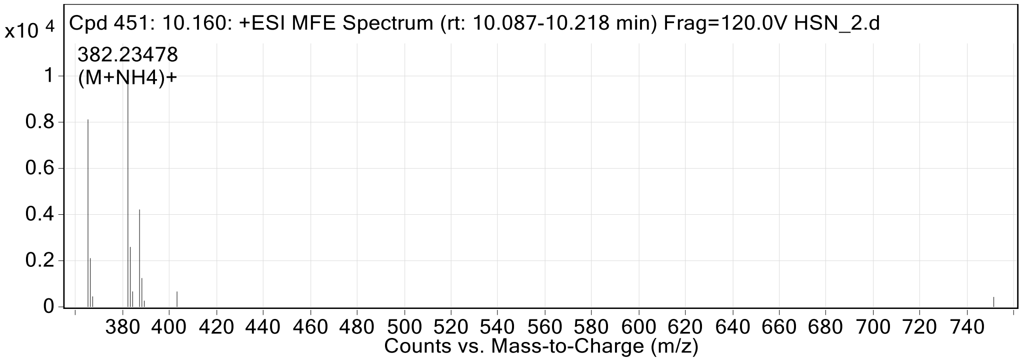

Peak List

m/z	z	Abund	Ion
365.20827	1	8112.77	(M+H)+
366.21062	1	2108.85	(M+H)+

367.2152	1	453.51	(M+H)+
382.23478	1	9861.87	(M+NH4)+
383.23833	1	2594.55	(M+NH4)+
384.24491	1	668.38	(M+NH4)+
387.18992	1	4215.72	(M+Na)+
388.19319	1	1247.48	(M+Na)+
403.16471	1	666.08	(M+K)+
751.38761	1	430.51	(2M+Na)+

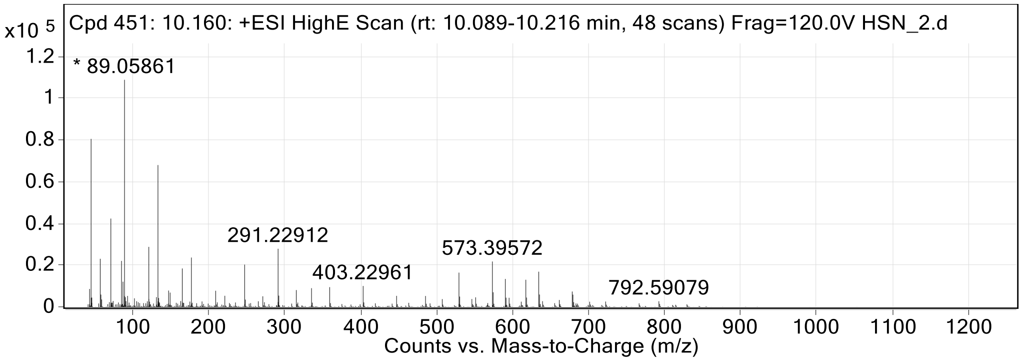

Peak List

m/z	z	Abund
45.03329	1	80511.73
57.06937		23041.74
71.08472	1	42322.68
85.10004		22026.9
89.05861	1	108837.99
121.06295	1	28741.88
133.08412	1	67971.44
177.1101	1	23634.95
291.22912	1	27849.95
573.39572	1	21737.7

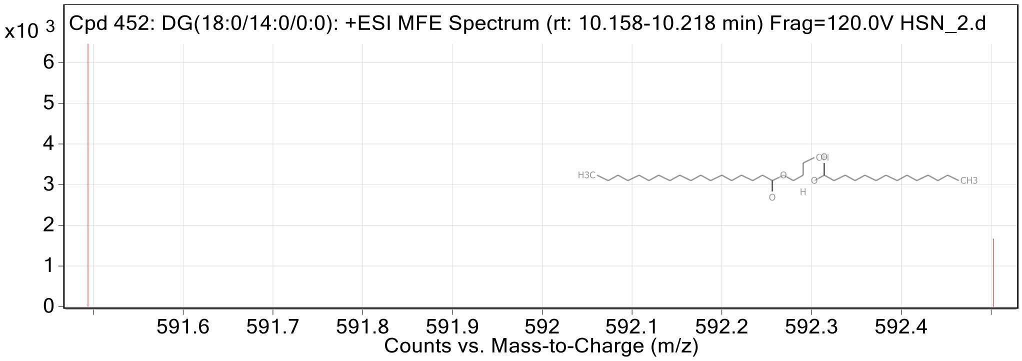

Peak List

m/z	z	Abund	Name	Formula	Ion	Score (DB)	Hits (DB)
591.49344	1	6463.56	DG(18:0/14:0/0:0)	C35 H68 O5	(M+Na)+	57.6	7
592.50328	1	1675.52		C35 H68 O5	(M+Na)+		

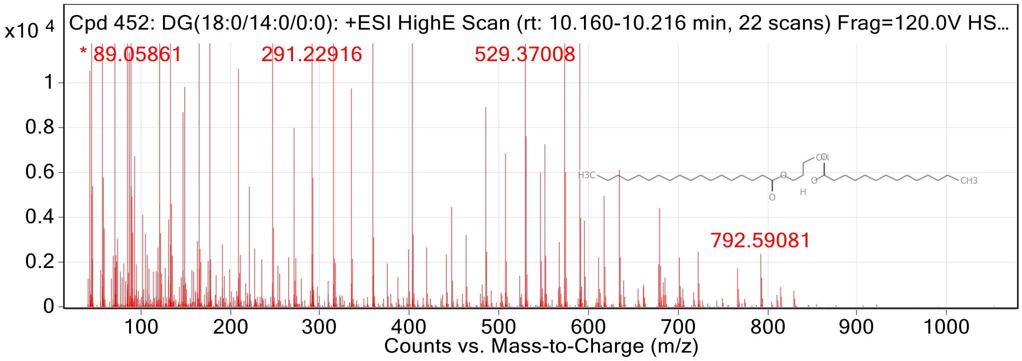

Peak List

m/z	z	Abund
45.03332	1	88592.55
57.06938		26449.02
71.08473	1	49110.05
85.10005		24754.16
89.05861	1	116556.5
121.06293	1	38778.43
133.08416	1	73279.33
177.11008	1	25584.3
291.22916	1	30062.58
529.37008	1	25553.27

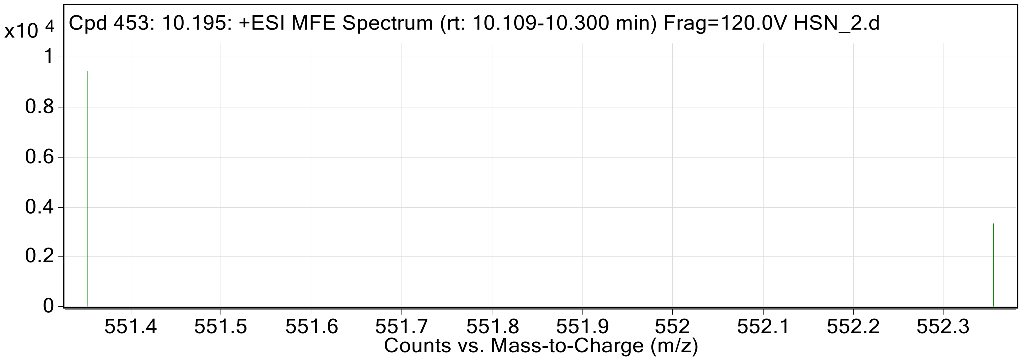

Peak List

m/z	z	Abund	Ion
551.35192	1	9455.36	(M+H)+
552.35496	1	3338.45	(M+H)+

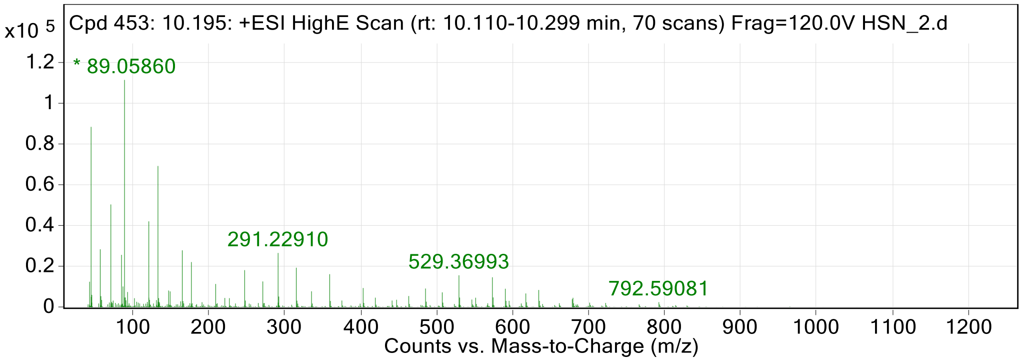

Peak List

m/z	z	Abund
45.03331	1	88555.88
57.06937		28368.84
71.08472	1	50405.74

85.10005		25612.04
89.0586	1	111631.34
121.06295	1	42095.16
133.08413	1	69311.65
165.08876	1	27842.82
177.11003	1	22074.34
291.2291	1	26543.75

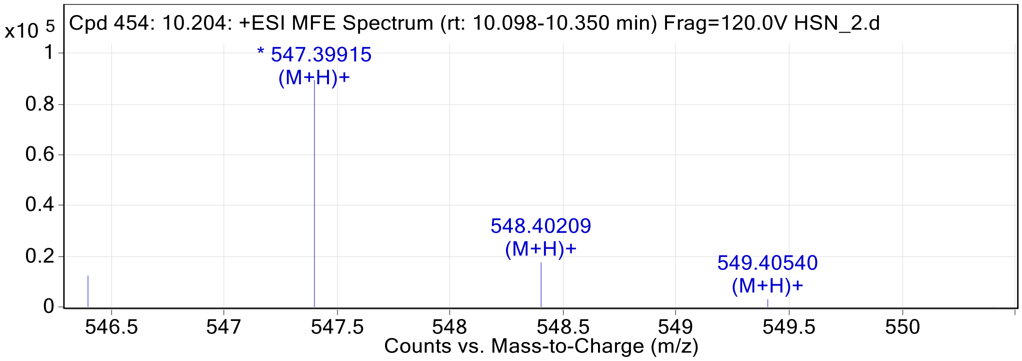

Peak List

m/z	z	Abund	Ion
546.39715	1	12392.28	(M+H)+
547.39915	1	89633.09	(M+H)+
548.40209	1	17574.39	(M+H)+
549.4054	1	3021.94	(M+H)+
550.40537	1	75.4	(M+H)+

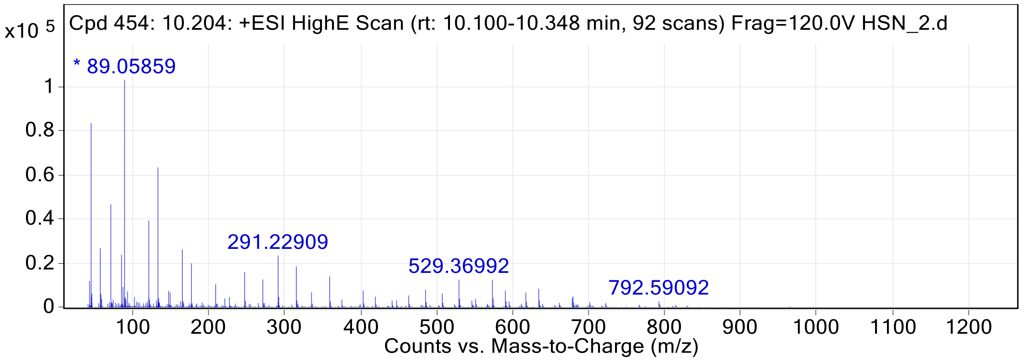

Peak List

m/z	z	Abund
45.03329	1	83644.87
57.06936		26693.1
71.08471	1	46675.99
85.10004		23750.92
89.05859	1	103184.35
121.06294	1	39326.47
133.08412	1	63463.31
165.08877	1	26174.77
177.11001	1	19880.75
291.22909	1	23361.07

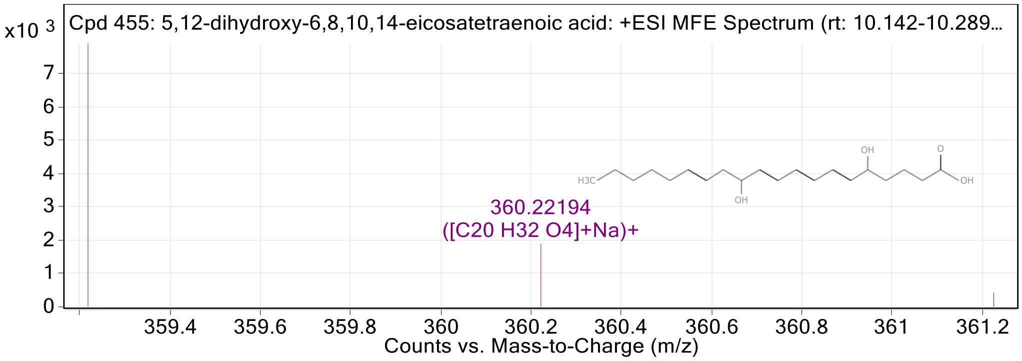

Peak List

m/z	z	Abund	Name	Formula	Ion	Score (DB)	Hits (DB)
359.21862	1	7909.84	5,12-dihydroxy-6,8,10,14-eicosatetraenoic acid	C20 H32 O4	(M+Na)+	96.84	10
360.22194	1	1895.46		C20 H32 O4	(M+Na)+		
361.22528	1	425.69		C20 H32 O4	(M+Na)+		

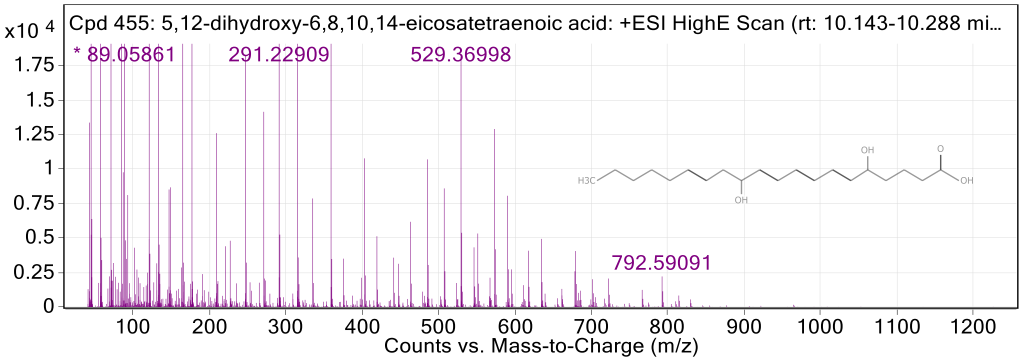

Peak List

m/z	z	Abund
45.03332	1	92165.03
57.06937		30088.72
71.08472	1	53440.52
85.10005		26977.4
89.05861	1	115136.14
121.06295	1	46704.28
133.08414	1	71678.36
165.08877	1	30793.43
177.10999	1	22542.85
291.22909	1	27363.25

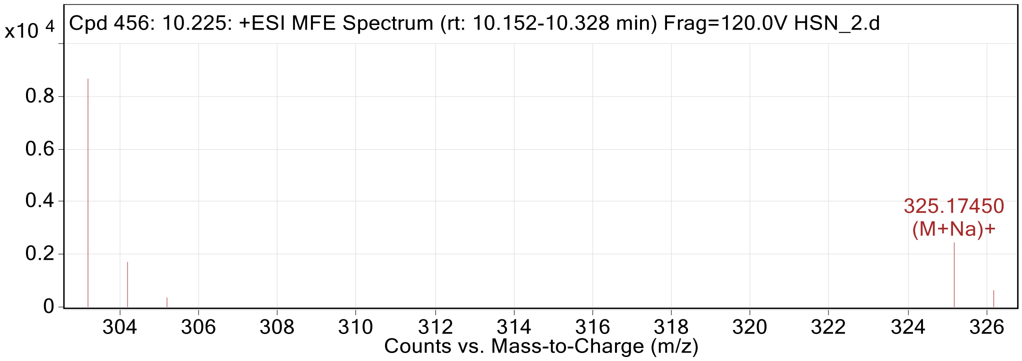

Peak List

m/z	z	Abund	Ion
303.19258	1	8675.03	(M+H)+

304.19625	1	1711.77	(M+H)+
305.1978	1	356.93	(M+H)+
325.1745	1	2452.84	(M+Na)+
326.17826	1	641.7	(M+Na)+

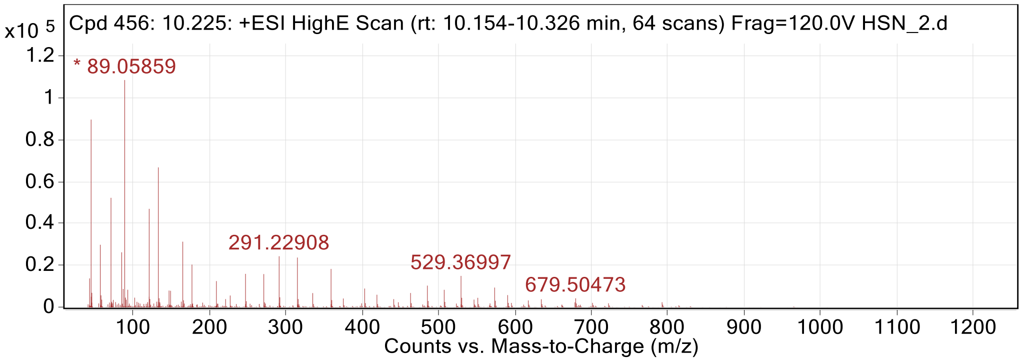

Peak List

m/z	z	Abund
45.03331	1	89736.68
57.06935		29826.59
71.08472	1	52294.48
85.10004		26211.13
89.05859	1	108637.66
121.06293	1	47017.44
133.08412	1	66839.58
165.08876	1	31298.16
291.22908	1	24338.85
315.17747	1	23704.29

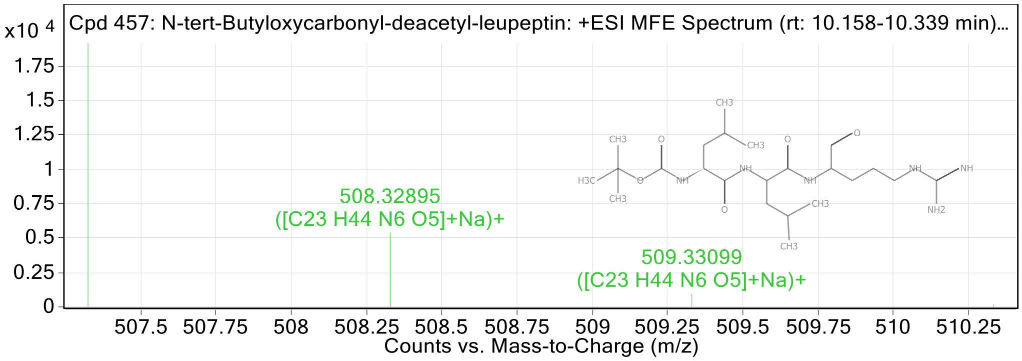

Peak List

m/z	z	Abund	Name	Formula	Ion	Score (DB)	Hits (DB)
507.32549	1	19157.04	N-tert-Butyloxycarbonyl-deacetyl-leupeptin	C23 H44 N6 O5	(M+Na)+	97.88	2
508.32895	1	5401.66		C23 H44 N6 O5	(M+Na)+		
509.33099	1	969.12		C23 H44 N6 O5	(M+Na)+		
510.33359	1	176.95		C23 H44 N6 O5	(M+Na)+		

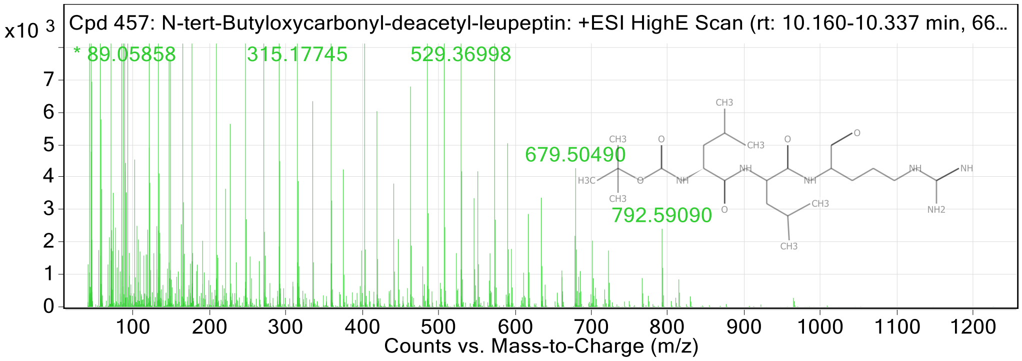

Peak List

m/z	z	Abund
45.0333	1	88561.84
57.06936		29464.45
71.08472	1	51335.83
85.10004		25725.47
89.05858	1	106224.86
121.06293	1	46651.76
133.08412	1	65202.2
165.08877	1	31159.38
291.22908	1	23351.36
315.17745	1	23795.97

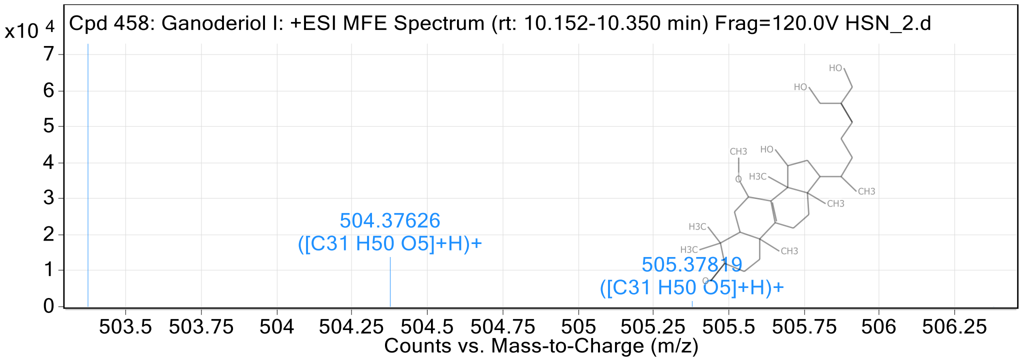

Peak List

m/z	z	Abund	Name	Formula	Ion	Score (DB)	Hits (DB)
503.37342	1	73177.88	Ganoderiol I	C31 H50 O5	(M+H)+	77.71	1
504.37626	1	13837.34		C31 H50 O5	(M+H)+		
505.37819	1	1605.7		C31 H50 O5	(M+H)+		
506.37878	1	57.92		C31 H50 O5	(M+H)+		

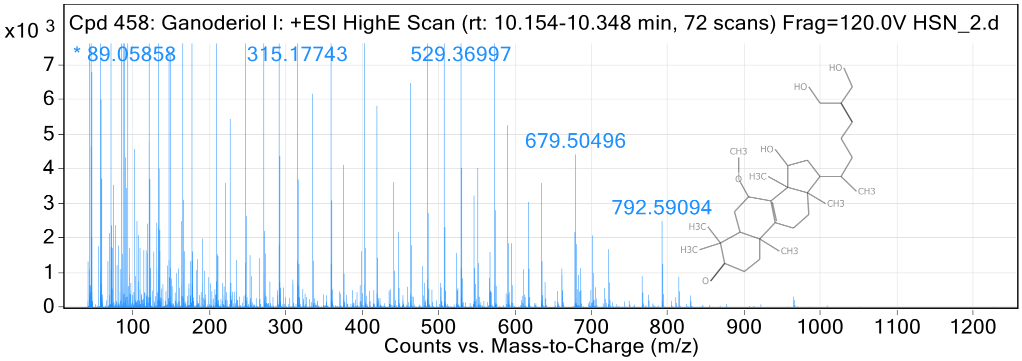

Peak List

m/z	z	Abund
45.0333	1	86415.36

57.06936		28510.76
71.08472	1	49514.84
85.10004		24852.99
89.05858	1	103459.07
121.06293	1	44719.57
133.08412	1	63384.27
165.08877	1	29846.44
291.22907	1	22604.94
315.17743	1	22610.32

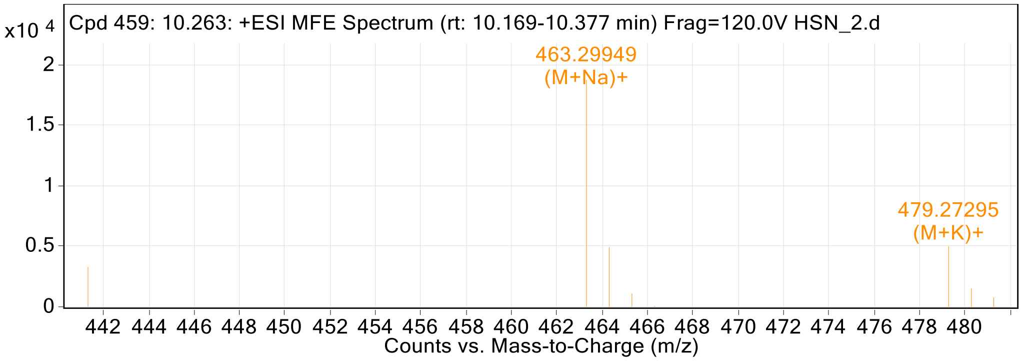

Peak List

m/z	z	Abund	Ion
441.31726	1	3300.97	(M+H)+
463.29949	1	18789.9	(M+Na)+
464.30307	1	4949.04	(M+Na)+
465.30627	1	1114.58	(M+Na)+
466.30685	1	78.29	(M+Na)+
479.27295	1	5008.31	(M+K)+
480.27743	1	1540.13	(M+K)+
481.26381	1	772.34	(M+K)+

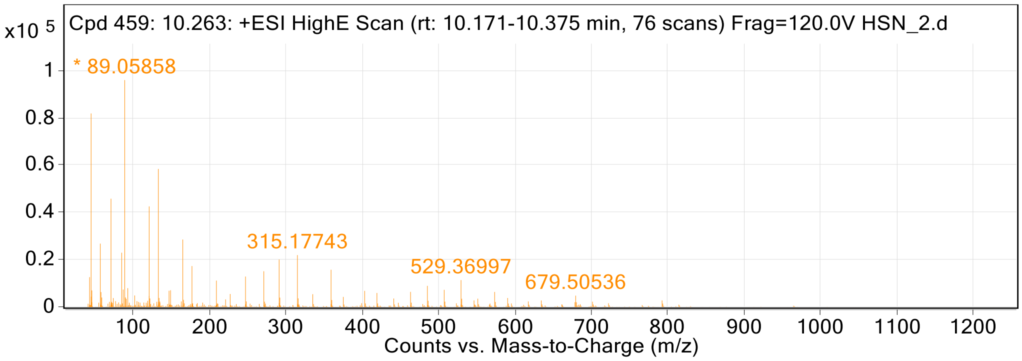

Peak List

m/z	z	Abund
45.0333	1	81782.58
57.06936		26721.49
71.08472	1	45720.8
85.10003		22875.23
89.05858	1	95896.23
121.06293	1	42471.61
133.08413	1	58313.82
165.08877	1	28457.46
291.22909	1	19988.53
315.17743	1	21871.2

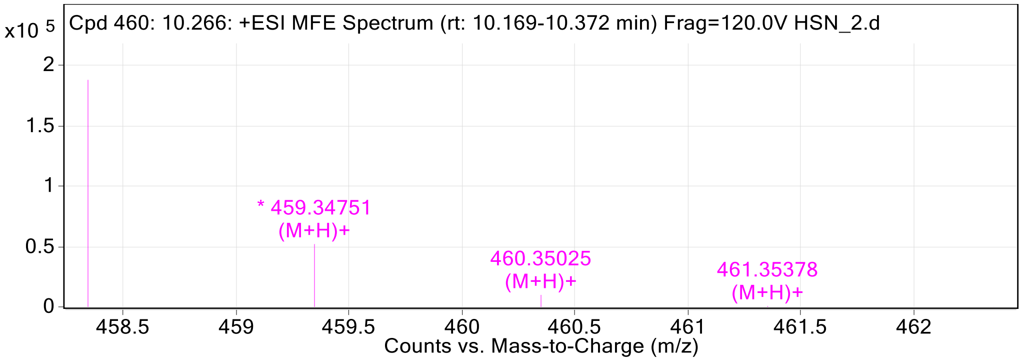

Peak List

m/z	z	Abund	Ion
458.34478	1	188093.75	(M+H)+
459.34751	1	52040.53	(M+H)+
460.35025	1	9935.36	(M+H)+
461.35378	1	699.66	(M+H)+
462.35463	1	39.67	(M+H)+

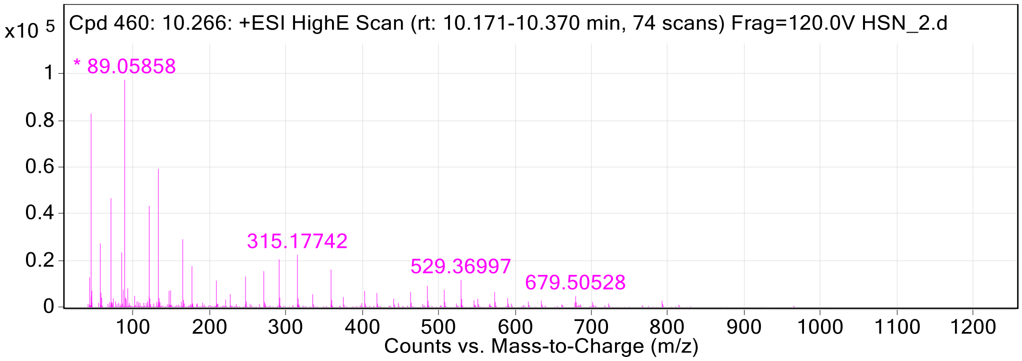

Peak List

m/z	z	Abund
45.0333	1	82886.11
57.06936		27186.6
71.08472	1	46620.36
85.10004		23320.96
89.05858	1	97274.33
121.06293	1	43288.03
133.08413	1	59212.45
165.08877	1	29003.53
291.22909	1	20381.71
315.17742	1	22336.65

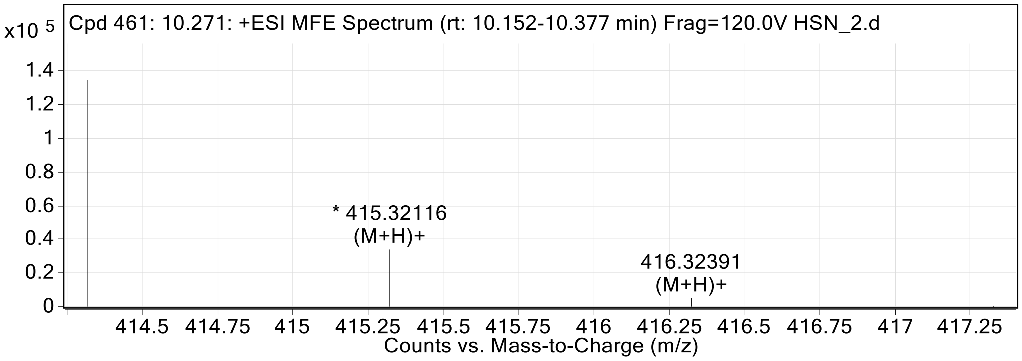

Peak List

m/z	z	Abund	Ion

414.31812	1	134834.64	(M+H)+
415.32116	1	33982.7	(M+H)+
416.32391	1	5013.77	(M+H)+
417.32745	1	334.98	(M+H)+

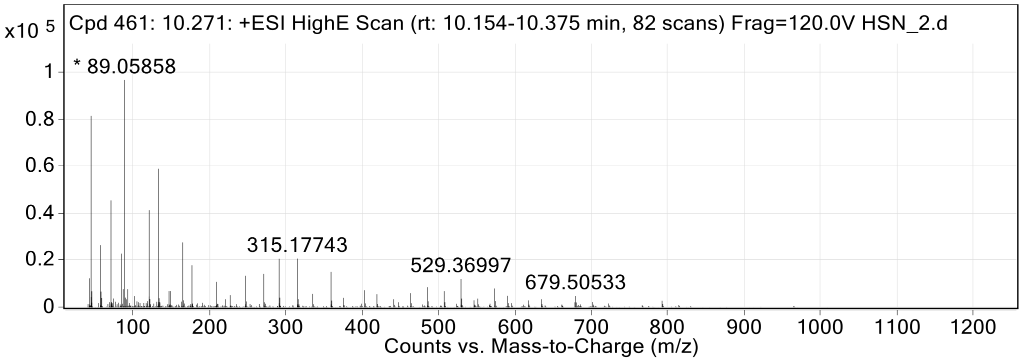

Peak List

m/z	z	Abund
45.0333	1	81565.38
57.06936		26412.16
71.08472	1	45480.5
85.10003		22796.6
89.05858	1	96830.93
121.06293	1	41269.52
133.08413	1	59052.38
165.08877	1	27556.3
291.22907	1	20629.65
315.17743	1	20674.05

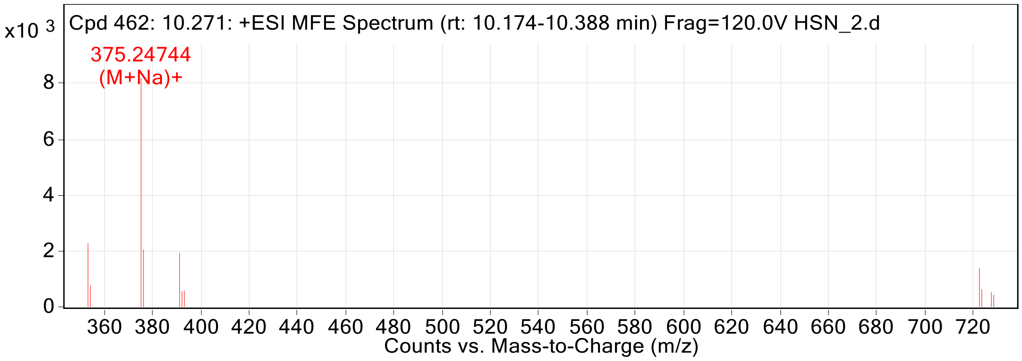

Peak List

m/z	z	Abund	Ion
353.26537	1	2283.85	(M+H)+
354.26894	1	777.92	(M+H)+
375.24744	1	8189.43	(M+Na)+
376.25087	1	2053.32	(M+Na)+
391.22128	1	1935.03	(M+K)+
392.22258	1	563.15	(M+K)+
393.20829	1	578.95	(M+K)+
722.55446	1	1394.15	(2M+NH4)+
723.55283	1	632.87	(2M+NH4)+
727.5069	1	525.21	(2M+Na)+

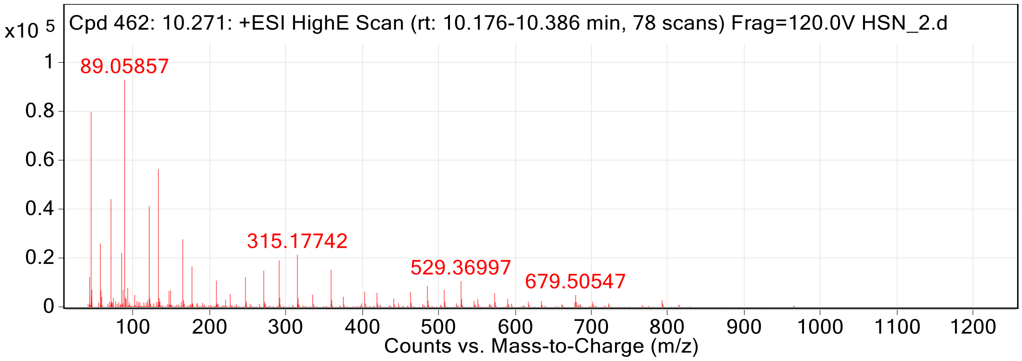

Peak List

m/z	z	Abund
45.0333	1	79649.93
57.06936		25879.78
71.08472	1	44016.54
85.10004		22013.25
89.05857	1	92785.5
121.06293	1	41229.01
133.08413	1	56259.81
165.08877	1	27638.15
291.22908	1	18991.01
315.17742	1	21308.65

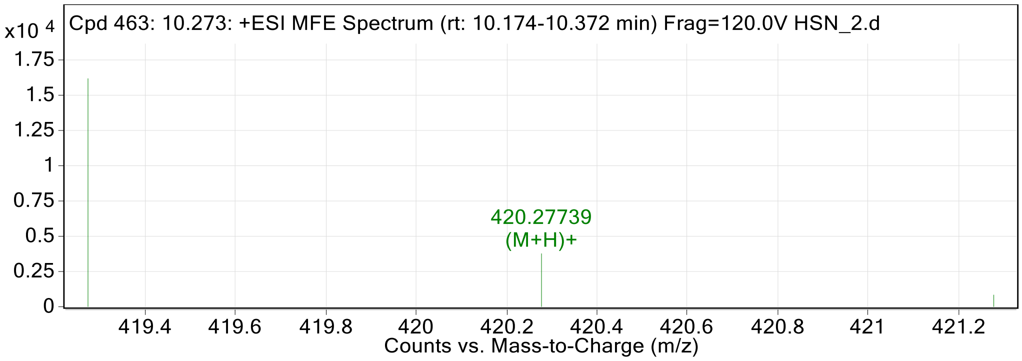

Peak List

m/z	z	Abund	Ion
419.27346	1	16160.05	(M+H)+
420.27739	1	3772.54	(M+H)+
421.27859	1	848.64	(M+H)+

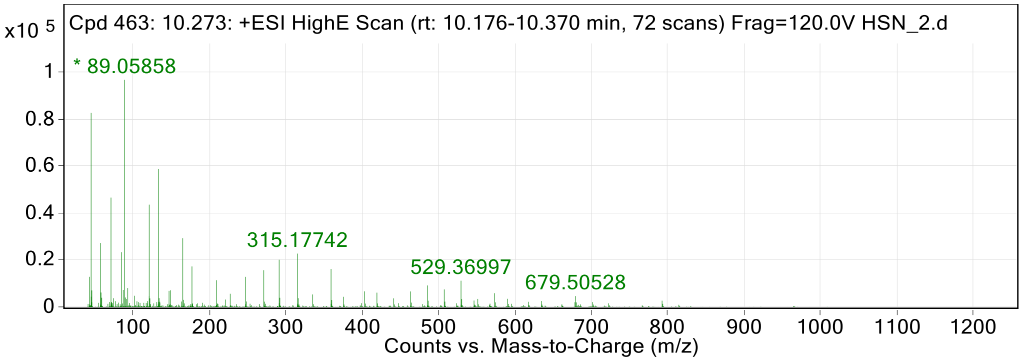

Peak List

m/z	z	Abund
45.0333	1	82801.68
57.06936		27301.32

71.08472	1	46662.98
85.10004		23343.24
89.05858	1	96898.06
121.06293	1	43656.76
133.08412	1	58888.71
165.08877	1	29276.04
291.22909	1	20139.37
315.17742	1	22749.55

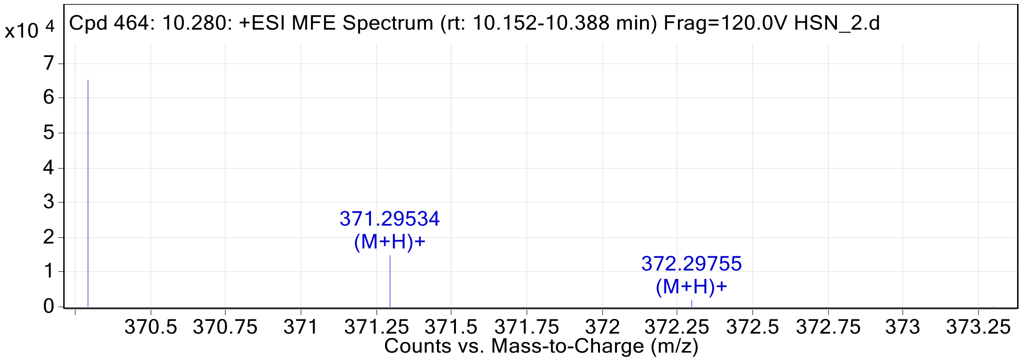

Peak List

m/z	z	Abund	Ion
370.29224	1	65359.52	(M+H)+
371.29534	1	14873.24	(M+H)+
372.29755	1	1948.92	(M+H)+

m/z	z	Abund	Ion
373.30057	1	163.15	(M+H)+

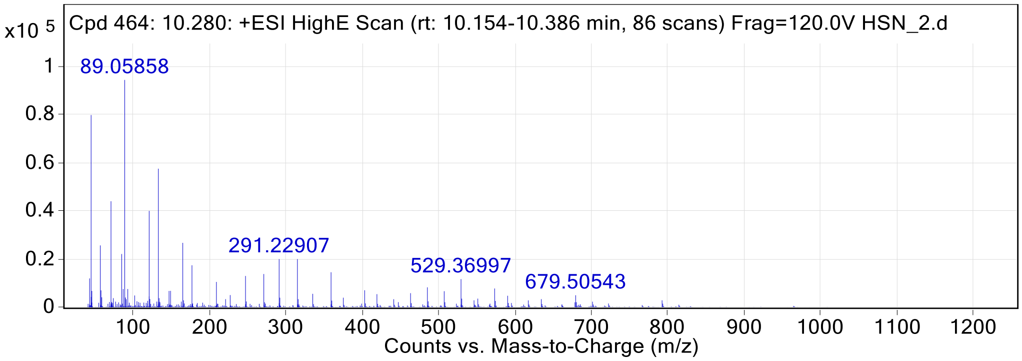

Peak List

m/z	z	Abund
45.0333	1	79737.56
57.06936		25577.91
71.08472	1	43931.19
85.10004		22010.17
89.05858	1	94313.15
121.06293	1	39908.7
133.08414	1	57447.02
165.08877	1	26639.68
291.22907	1	19907.13
315.17742	1	19884.65

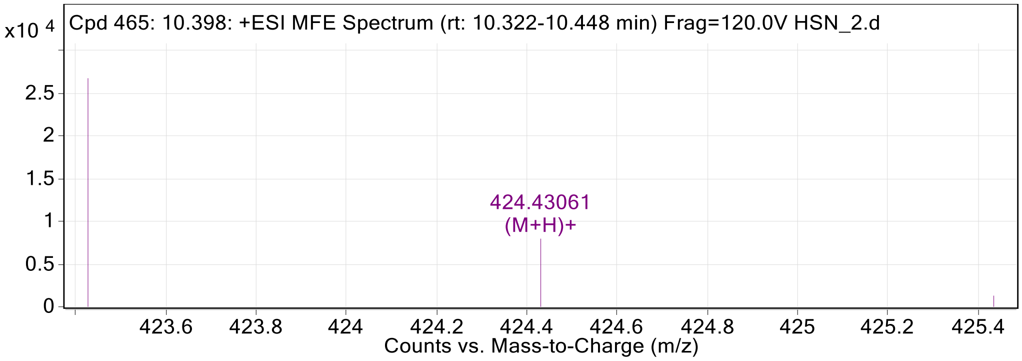

Peak List

m/z	z	Abund	Ion
423.42723	1	26729.1	(M+H)+
424.43061	1	7966.97	(M+H)+
425.43446	1	1302.08	(M+H)+

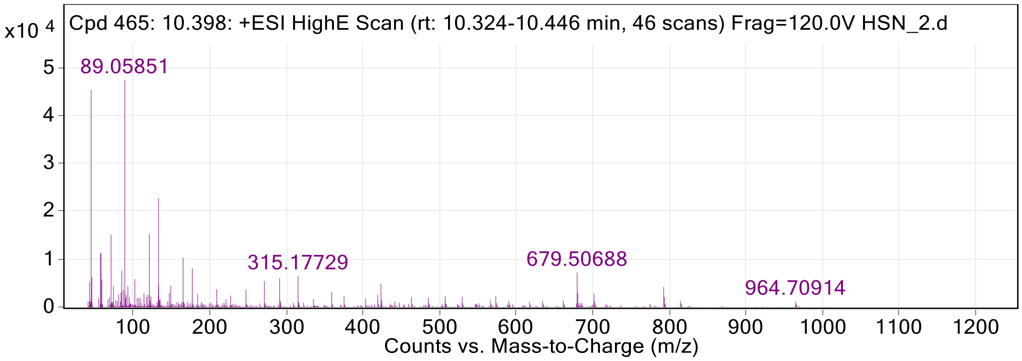

Peak List

m/z	z	Abund

45.03322	1	45297.32
57.06936		11137.54
58.06468		11388.45
71.08469	1	15072.73
85.10004		7601.91
89.05851	1	47375
121.06285	1	15261.03
133.08458	1	22735.01
165.08878	1	10344.09
177.11		8062.35

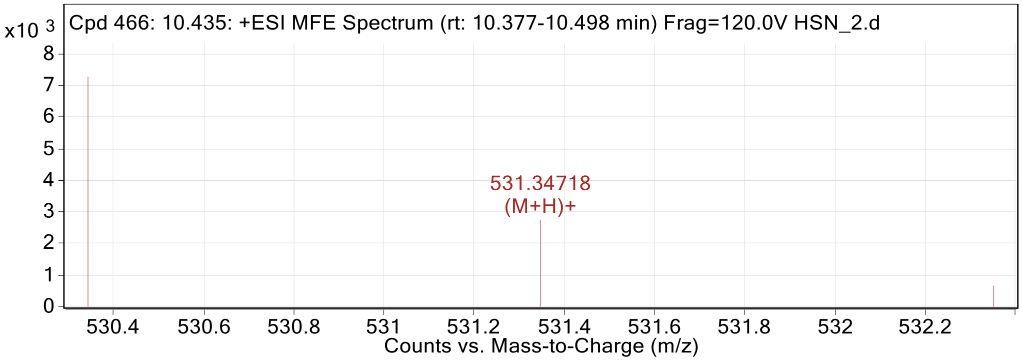

Peak List

m/z	z	Abund	Ion
530.34318	1	7278.45	(M+H)+
531.34718	1	2756.76	(M+H)+
532.35261	1	668.54	(M+H)+

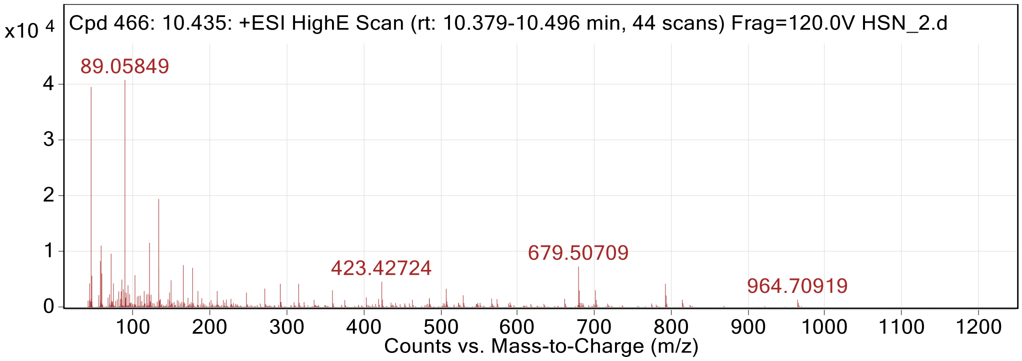

Peak List

m/z	z	Abund
45.03318		39459.73
57.06933		8228.73
58.06462		11011.92
71.08463		9551.55
89.05849	1	40711.68
121.0628	1	11508.82
133.0855	1	19345.02
133.09696		7139.05
165.08867		7495.81
679.50709	1	7256.72

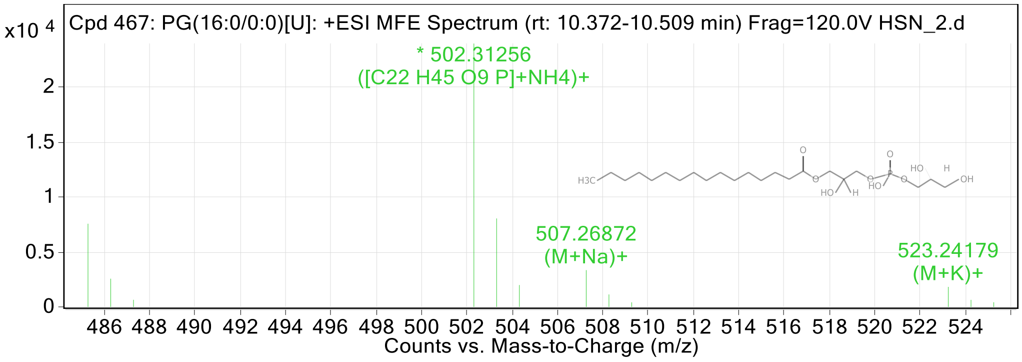

Peak List

m/z	z	Abund	Name	Formula	Ion	Score (DB)	Hits (DB)
485.28643	1	7566.55			(M+H)+		
486.28987	1	2564.7			(M+H)+		
487.29411	1	644.28			(M+H)+		
502.31256	1	23971.43	PG(16:0/0:0)[U]	C22 H45 O9 P	(M+NH4)+	85.75	2
503.31576	1	8044.66		C22 H45 O9 P	(M+NH4)+		
504.31907	1	1988.45		C22 H45 O9 P	(M+NH4)+		
507.26872	1	3345.44			(M+Na)+		
508.27111	1	1132.26			(M+Na)+		
523.24179	1	1819.33			(M+K)+		
524.24561	1	639.03			(M+K)+		

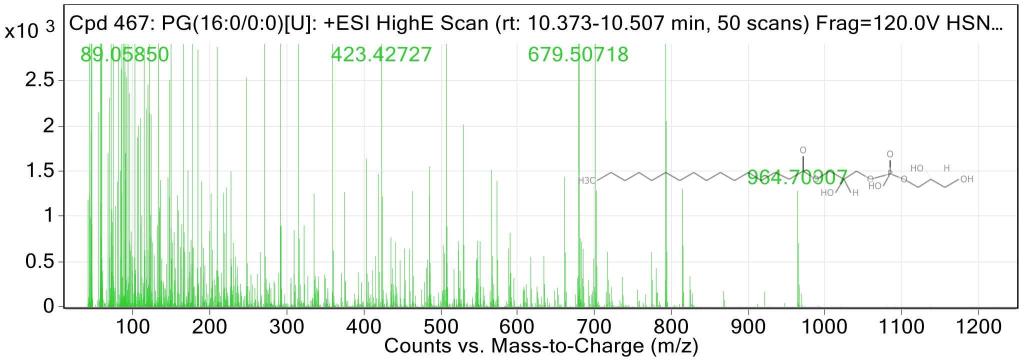

Peak List

m/z	z	Abund
45.03319	1	39497.93
57.06933		8276.87
58.06463		10865.92
71.08465		9570.33
89.0585	1	40676.18
121.06279	1	11521.21
133.08533	1	19692.47
165.08866		7517.21
177.10987		7007.61
679.50718	1	7257.14

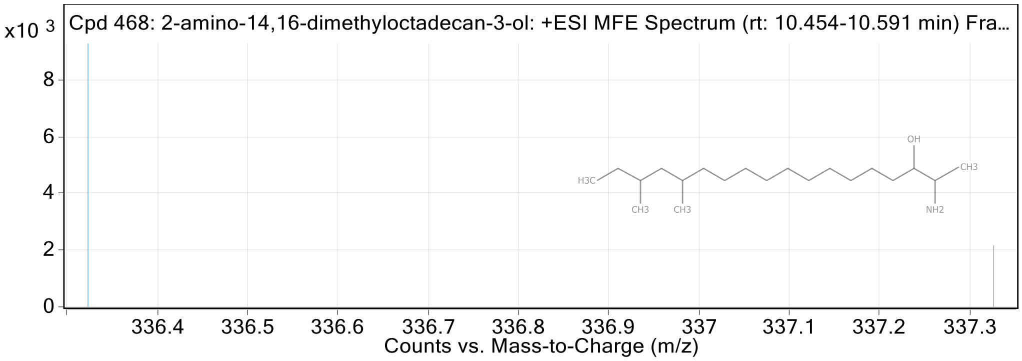

Peak List

m/z	z	Abund	Name	Formula	Ion	Score (DB)	Hits (DB)
336.32286	1	9278.3	2-amino-14,16-dimethyloctadecan-3-ol	C20 H43 N O	(M+Na)+	83.38	1
337.32672	1	2165.95		C20 H43 N O	(M+Na)+		

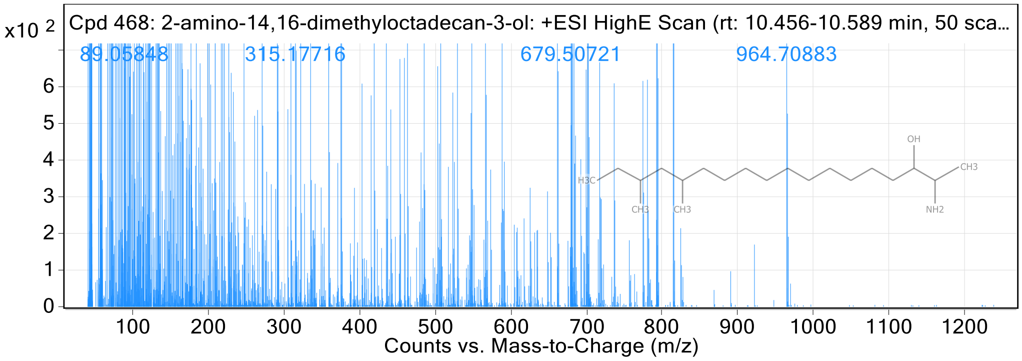

Peak List

m/z	z	Abund
45.03318		35153.33
57.0693		6183.71
58.06465		9356.36
71.08462		6933.39
89.05848	1	35159.49
121.06284	1	9390.19
133.08447	1	18443.13
165.08866		6055.4
177.10989		5579.15
679.50721	1	7675.09

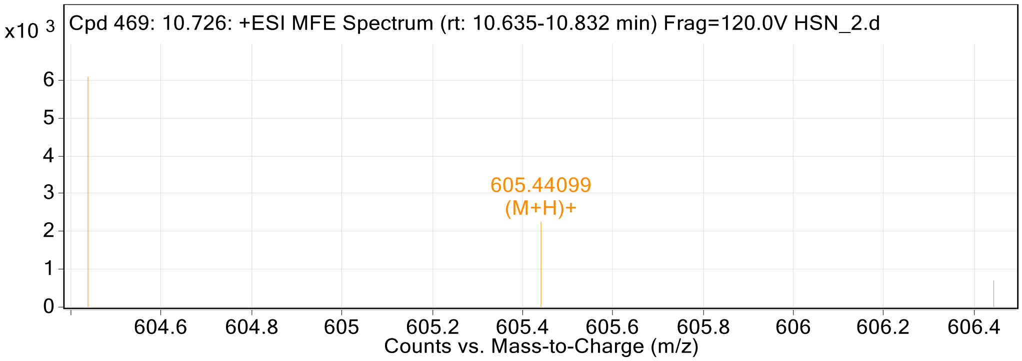

Peak List

m/z	z	Abund	Ion
604.43766	1	6094.84	(M+H)+
605.44099	1	2261.78	(M+H)+

606.44354	1	704.15	(M+H)+

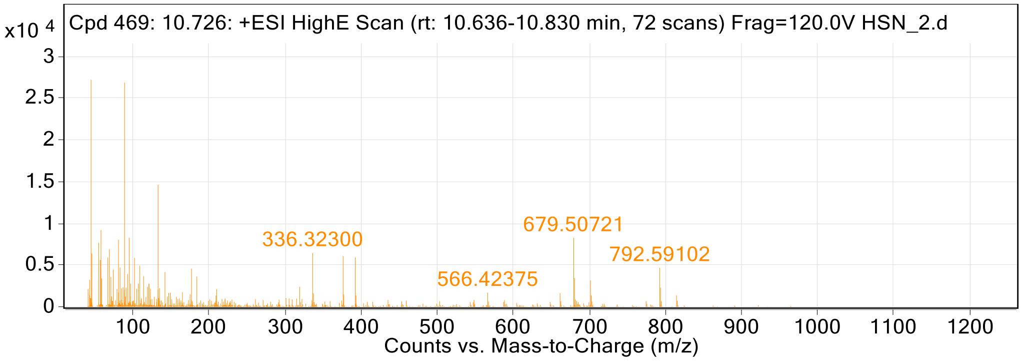

Peak List

m/z	z	Abund
45.03319		27216.05
55.05374		7663.7
58.06461		9190.18
69.06905		6882.78
81.06878		8043.71
89.05848	1	26876.83
95.08417		8283.31
133.08436	1	14617.45
336.323	1	6456.11
679.50721	1	8249.27

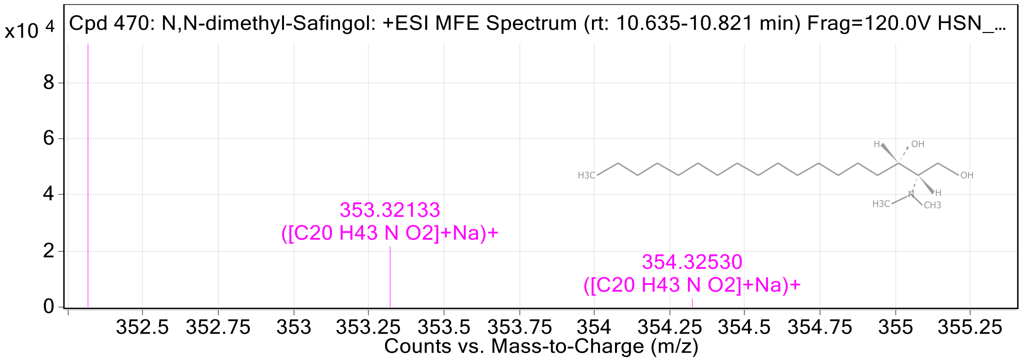

Peak List

m/z	z	Abund	Name	Formula	Ion	Score (DB)	Hits (DB)
352.31764	1	93804.05	N,N-dimethyl-Safingol	C20 H43 N O2	(M+Na)+	97.31	1
353.32133	1	21587.98		C20 H43 N O2	(M+Na)+		
354.3253	1	3077.57		C20 H43 N O2	(M+Na)+		
355.32651	1	133.27		C20 H43 N O2	(M+Na)+		

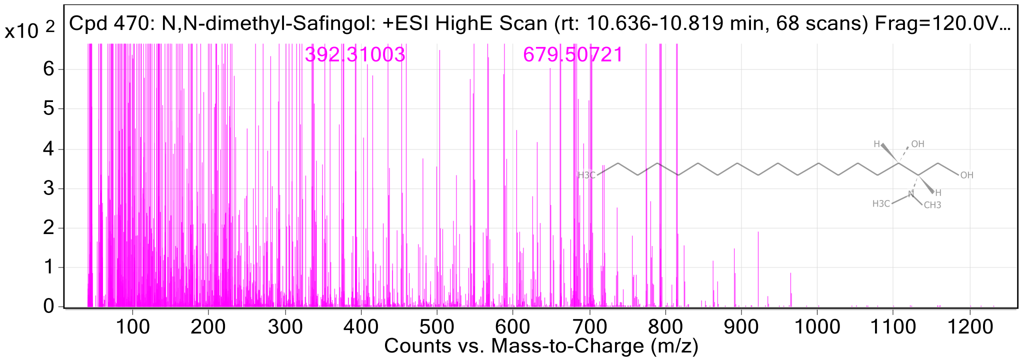

Peak List

m/z	z	Abund

45.03319		27295.24
46.06479		6358.28
55.05375		7279.24
58.06462		9272.97
69.06904		6444.18
81.06879		7713.01
89.05849	1	26857.28
95.08418		8017.37
133.08435	1	14645.06
679.50721	1	8306.32

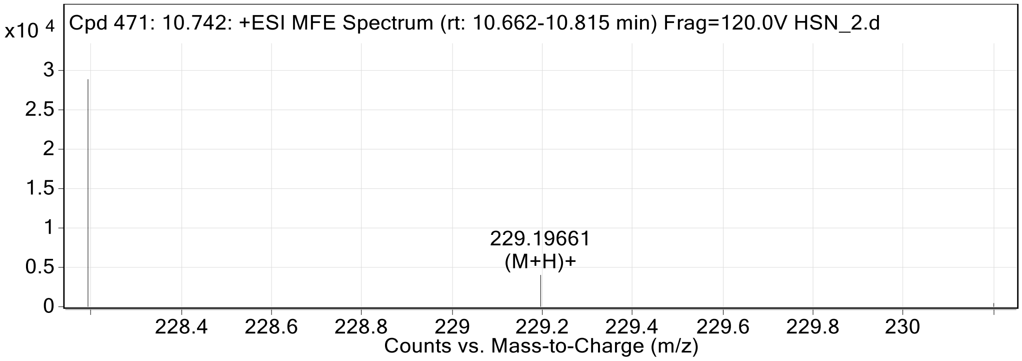

Peak List

m/z	z	Abund	Ion
228.19316	1	28886.14	(M+H)+
229.19661	1	4039.66	(M+H)+
230.20094	1	461.86	(M+H)+

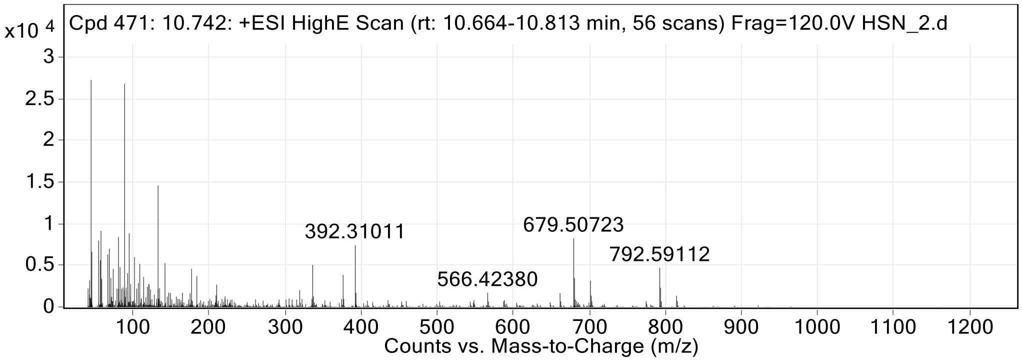

Peak List

m/z	z	Abund
45.03319		27228.23
55.05376		7948.75
58.06463		9132.27
69.06905		7006.12
81.06878		8390.59
89.05849	1	26778.86
95.08419		8844.41
133.08441	1	14532.93
392.31011	1	7427.79
679.50723	1	8232.05

Qualitative Analysis Report

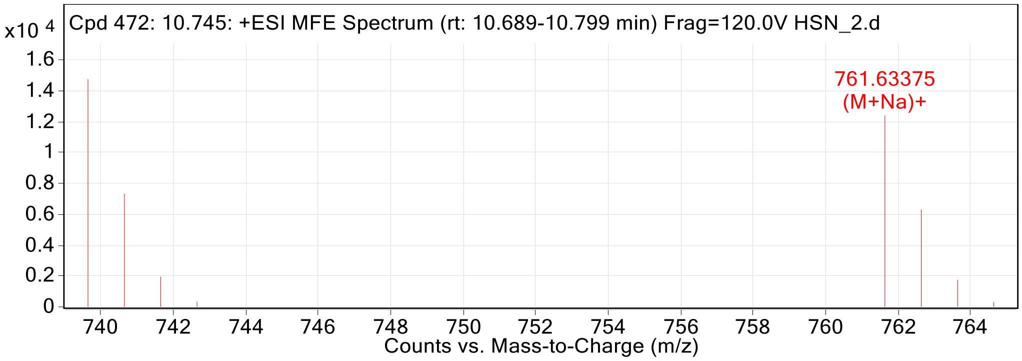

Peak List

m/z	z	Abund	Ion
739.65165	1	14767.14	(M+H)+
740.65542	1	7346.15	(M+H)+
741.65882	1	1954.06	(M+H)+
742.65971	1	331.63	(M+H)+
761.63375	1	12423.6	(M+Na)+
762.63695	1	6312.9	(M+Na)+
763.64052	1	1747.52	(M+Na)+
764.63697	1	317.02	(M+Na)+

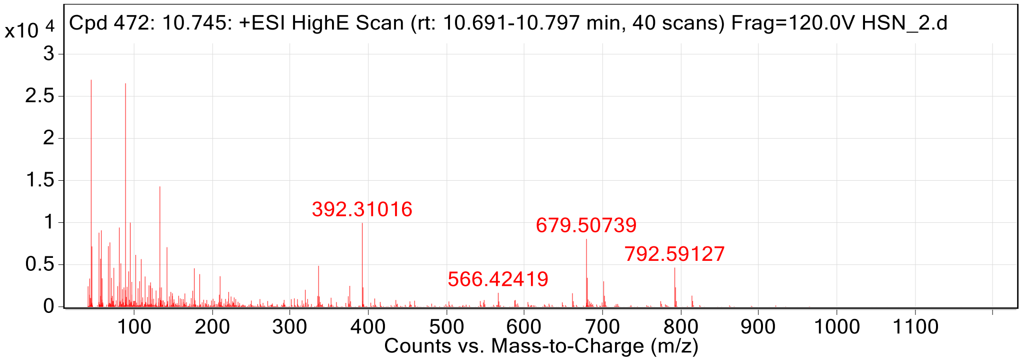

Peak List

m/z	z	Abund
45.03319		26963.38
55.05375		8800.22
58.06461		9081.32
69.06906		7645.24
81.06877		9412.96
89.05849	1	26542.71
95.08419		9995.94
133.08441		14294.12
392.31016	1	9956.68
679.50739	1	8063.29

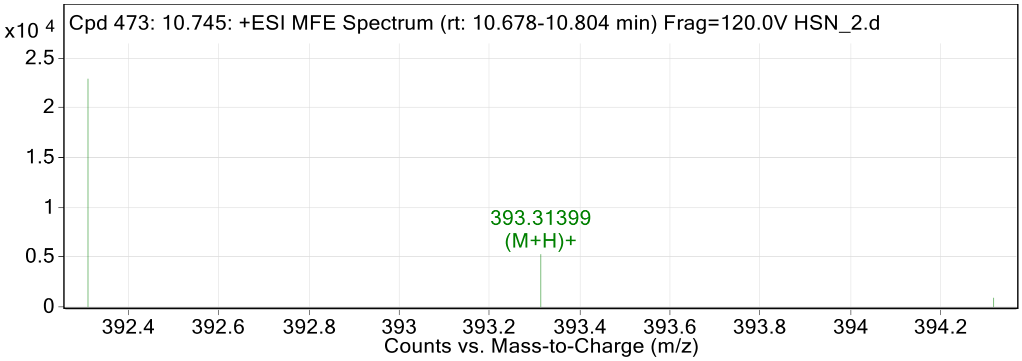

Peak List

m/z	z	Abund	Ion
392.31045	1	22914.76	(M+H)+
393.31399	1	5274.28	(M+H)+
394.31764	1	918.01	(M+H)+

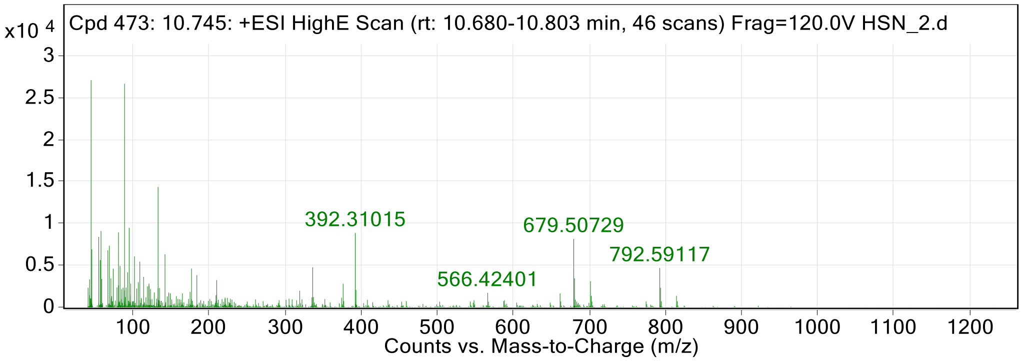

Peak List

m/z	z	Abund
45.03319		27082.42
55.05375		8382.13
58.06463		9082.83
69.06905		7326.2
81.06878		8918.56
89.05849	1	26646.77
95.08419		9453.2
133.08441		14339.3
392.31015	1	8848.57
679.50729	1	8138.97

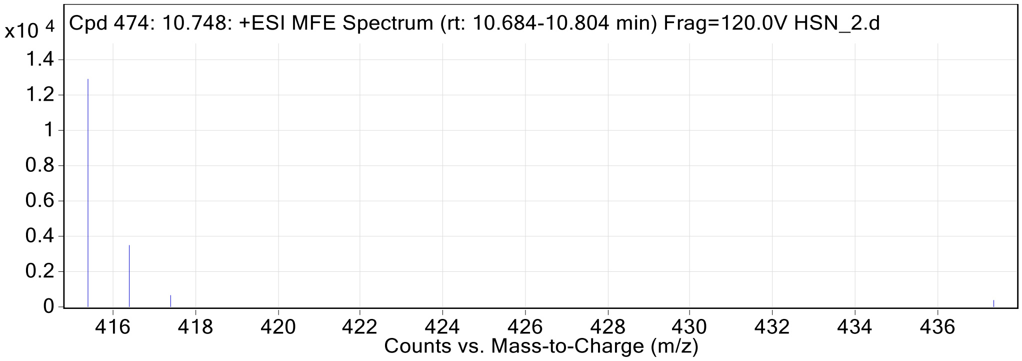

Peak List

m/z	z	Abund	Ion
415.38611	1	12906.91	(M+H)+
416.38924	1	3497.81	(M+H)+

417.39189	1	664.56	(M+H)+
437.37238	1	384.2	(M+Na)+

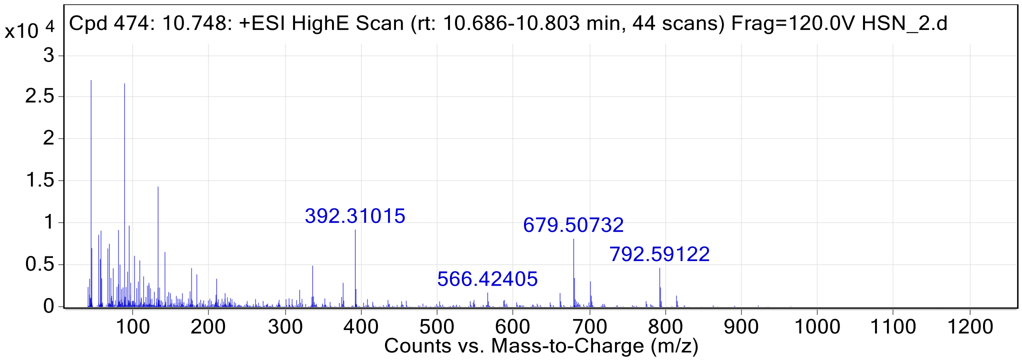

Peak List

m/z	z	Abund
45.03319		27046.46
55.05375		8587.63
58.06462		9084.94
69.06906		7510.99
81.06878		9152.95
89.0585	1	26648.23
95.08419		9683.86
133.08441		14336.99
392.31015	1	9206.47
679.50732	1	8127.16

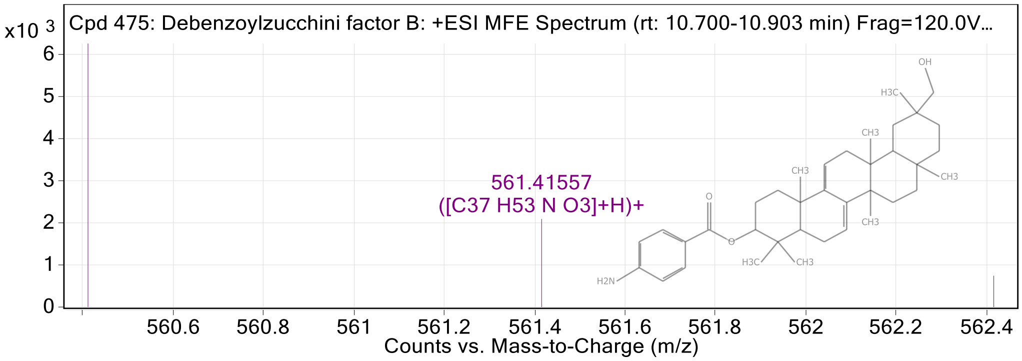

Peak List

m/z	z	Abund	Name	Formula	Ion	Score (DB)	Hits (DB)
560.41166	1	6251.57	Debenzoylzucchini factor B	C37 H53 N O3	(M+H)+	83.3	1
561.41557	1	2089.62		C37 H53 N O3	(M+H)+		
562.41614	1	741.7		C37 H53 N O3	(M+H)+		

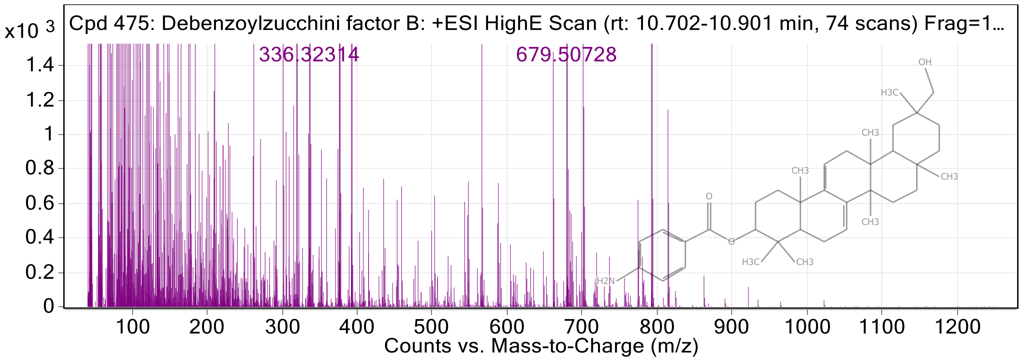

Peak List

m/z	z	Abund

45.03319		26347.91
55.05373		12134.76
67.05347		8682.61
69.06909		11202.15
81.06881		12488.02
89.05851	1	26162.39
95.08418		12648.99
133.08449		13963.96
336.32314	1	22712.8
376.31542	1	22414.63

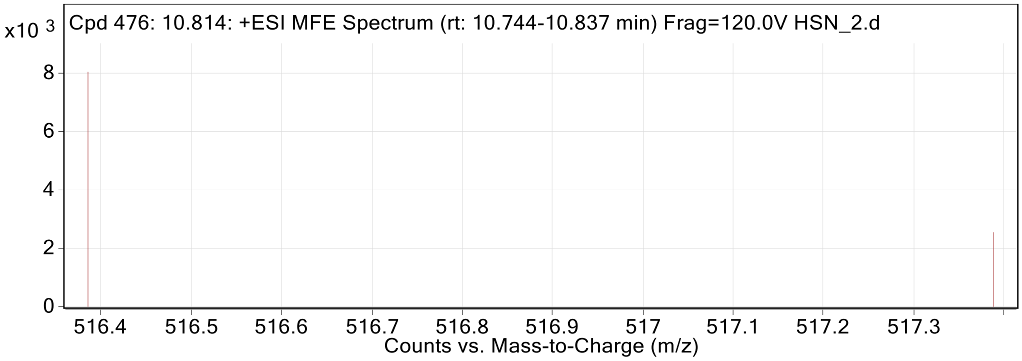

Peak List

m/z	z	Abund	Ion
516.3856	1	8059.12	(M+H)+
517.38863	1	2552.69	(M+H)+

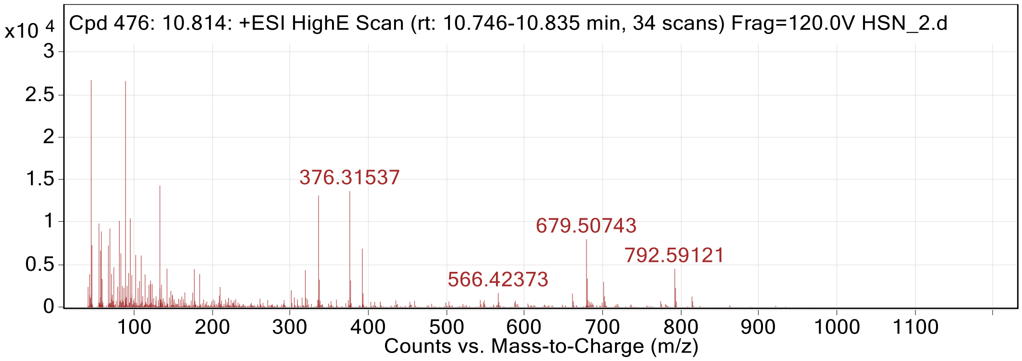

Peak List

m/z	z	Abund
45.03318		26740.85
55.05375		9827.89
58.06459		8904.53
69.06906		9241.56
81.06878		10151.31
89.05852	1	26609.92
95.08413	1	10439.06
133.0844		14303.83
336.32298	1	13131.74
376.31537	1	13663.29

Qualitative Analysis Report

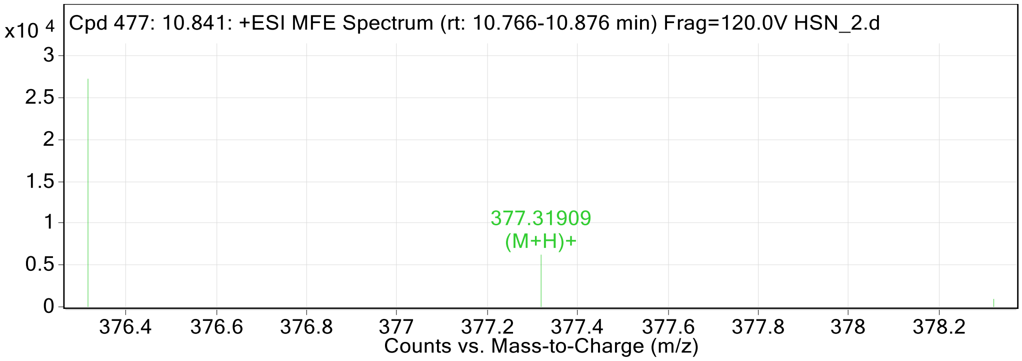

Peak List

m/z	z	Abund	Ion
376.31542	1	27274.09	(M+H)+
377.31909	1	6243.12	(M+H)+
378.32182	1	953.37	(M+H)+

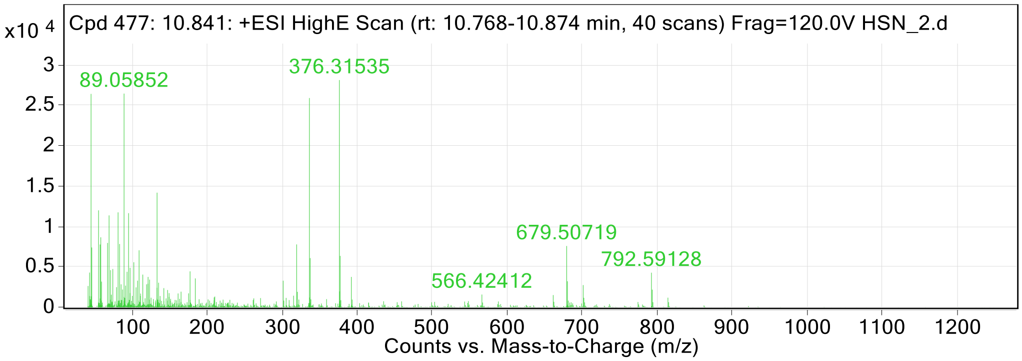

Peak List

m/z	z	Abund
45.0332		26414.59
55.05371		11985.29
58.06457		8644.73
69.0691		11360.81
81.0688		11736.02
89.05852	1	26452.88
95.08415		11635.88
133.08445	1	14175.64
336.32313	1	25901.58
376.31535	1	28129.88

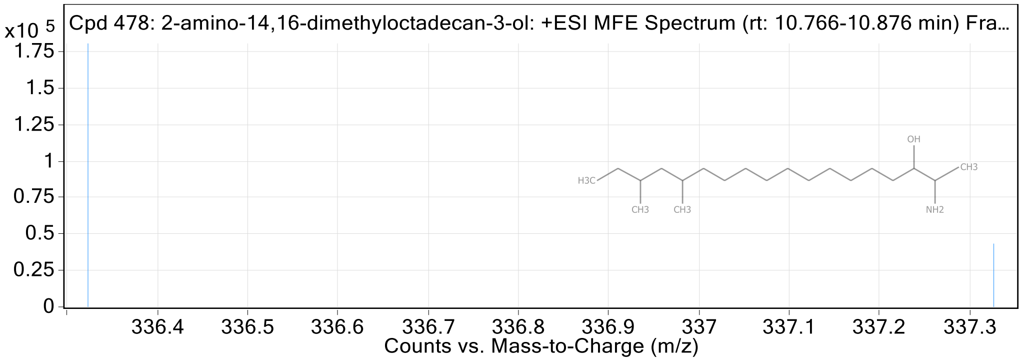

Peak List

m/z	z	Abund	Name	Formula	Ion	Score (DB)	Hits (DB)
336.32281	1	180472.16	2-amino-14,16-dimethyloctadecan-3-ol	C20 H43 N O	(M+Na)+	82.73	1

337.3266	1	43351.63		C20 H43 N O	(M+Na)+		

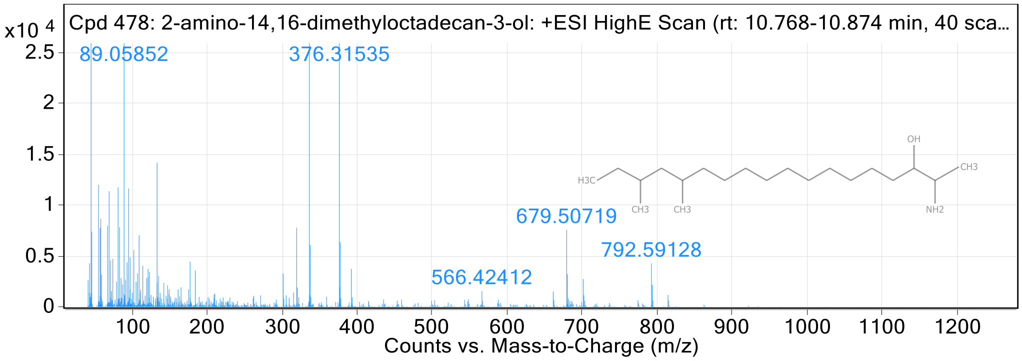

Peak List

m/z	z	Abund
45.0332		26414.59
55.05371		11985.29
58.06457		8644.73
69.0691		11360.81
81.0688		11736.02
89.05852	1	26452.88
95.08415		11635.88
133.08445	1	14175.64
336.32313	1	25901.58
376.31535	1	28129.88

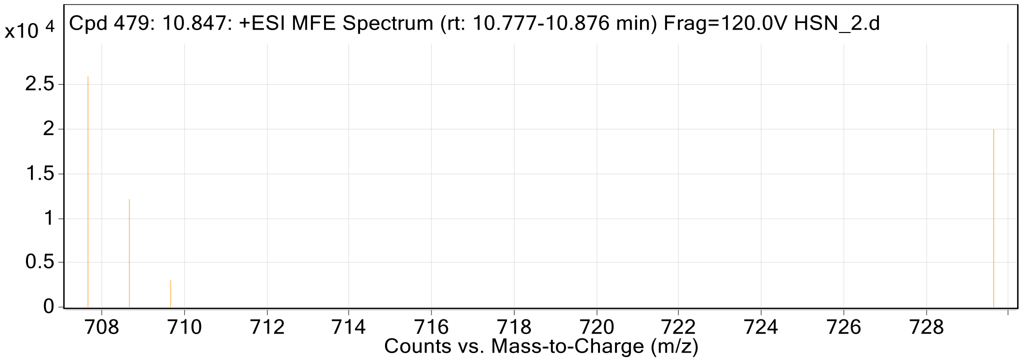

Peak List

m/z	z	Abund	Ion
707.66146	1	25928.21	(M+H)+
708.66484	1	12137.03	(M+H)+
709.66839	1	3020.22	(M+H)+
729.64324	1	20007.03	(M+Na)+

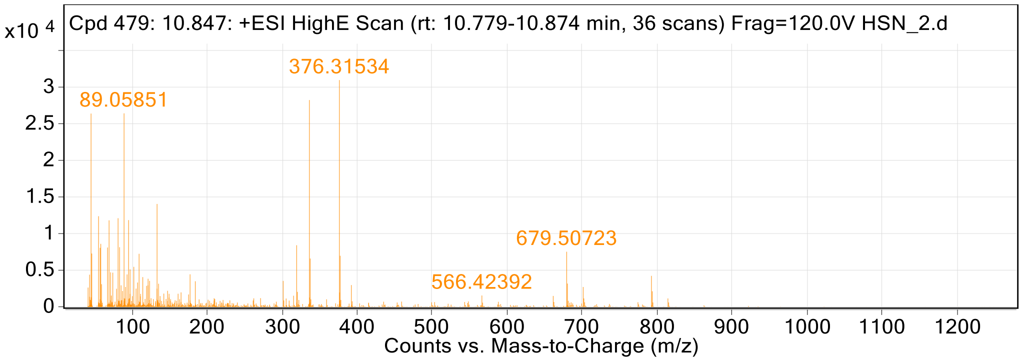

Peak List

m/z	z	Abund

45.0332		26439.67
55.05371		12395.61
58.06457		8613.25
69.0691		11829.31
81.0688		12120.39
89.05851	1	26467.77
95.08414	1	11861.53
133.08446		14069.78
336.32314	1	28286.76
376.31534	1	31018.17

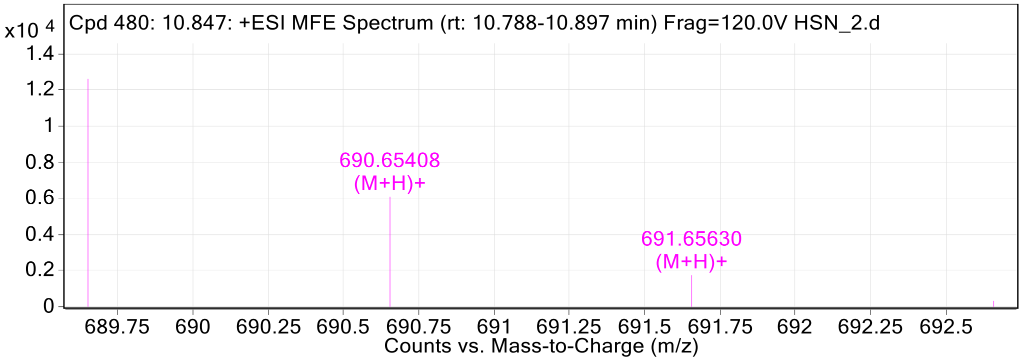

Peak List

m/z	z	Abund	Ion
689.65107	1	12620.92	(M+H)+
690.65408	1	6097.46	(M+H)+
691.6563	1	1748.75	(M+H)+
692.65934	1	328.24	(M+H)+

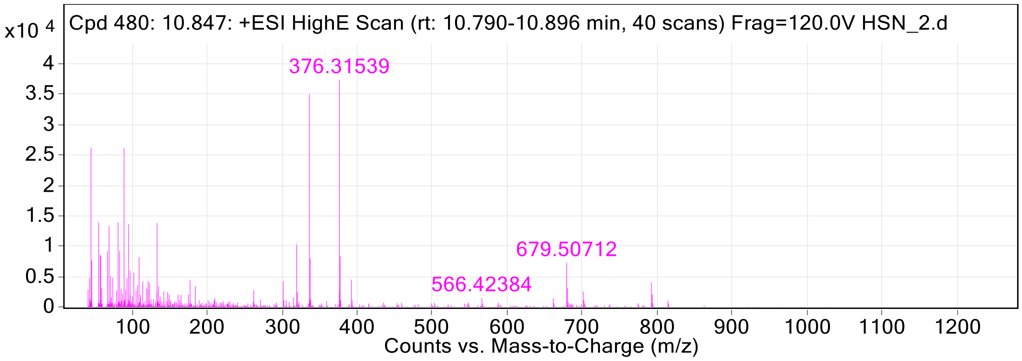

Peak List

m/z	z	Abund
45.03319		26206.27
55.05373		13966.76
69.06911		13298.19
81.0688		13947.66
89.05851	1	26160.68
95.08416	1	13635.17
133.08448		13811.57
319.29674	1	10278.34
336.32317	1	35026.99
376.31539	1	37379.37

Qualitative Analysis Report

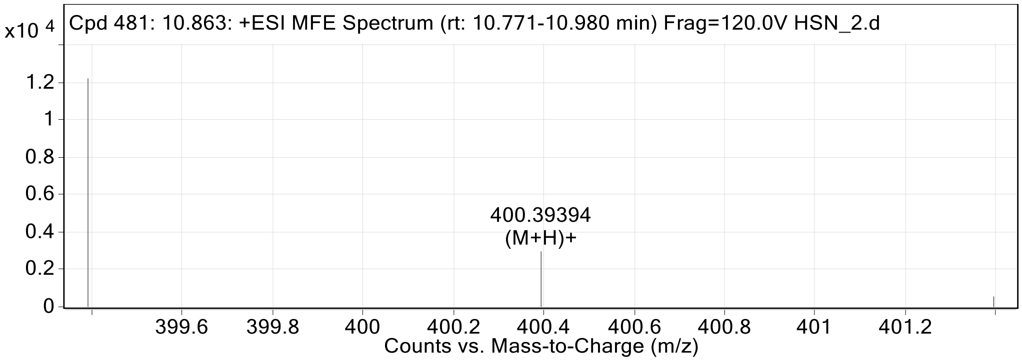

Peak List

m/z	z	Abund	Ion
399.39108	1	12205.88	(M+H)+
400.39394	1	2965.28	(M+H)+
401.39588	1	555.38	(M+H)+

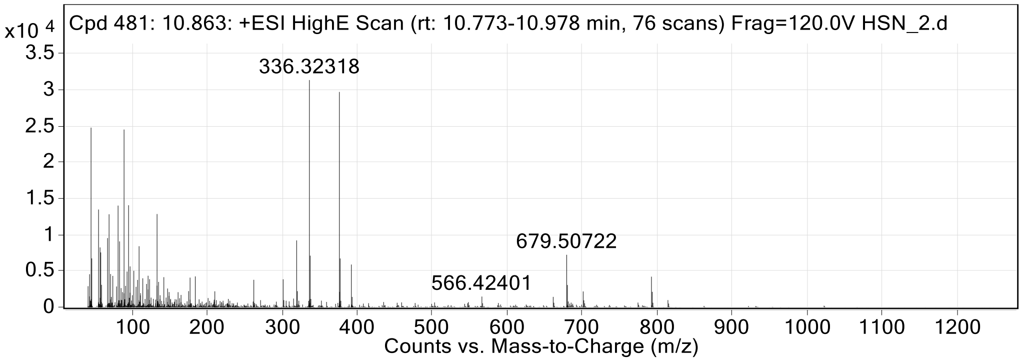

Peak List

m/z	z	Abund
45.03317		24787.66
55.05373		13476.11
67.05344		9532.87
69.06911		12816.96
81.06882		14017.51
89.0585	1	24523.51
95.08416	1	14064.35
133.08449		12851.58
336.32318	1	31358.45
376.31546	1	29714.22

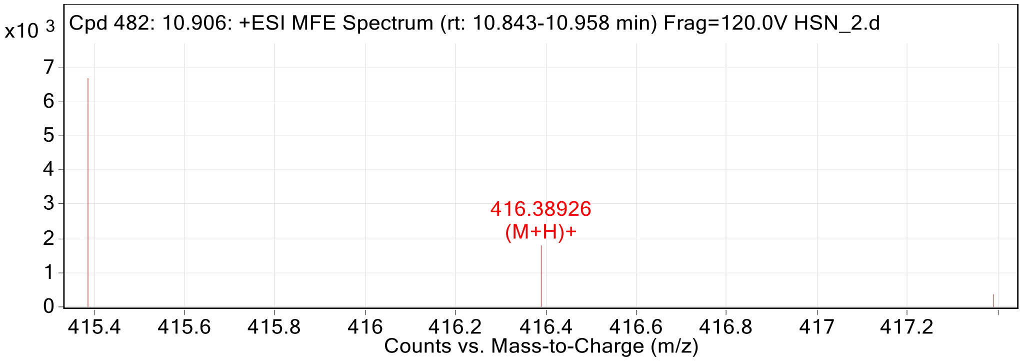

Peak List

m/z	z	Abund	Ion
415.38608	1	6685.83	(M+H)+
416.38926	1	1798.97	(M+H)+

417.39117	1	370.15	(M+H)+

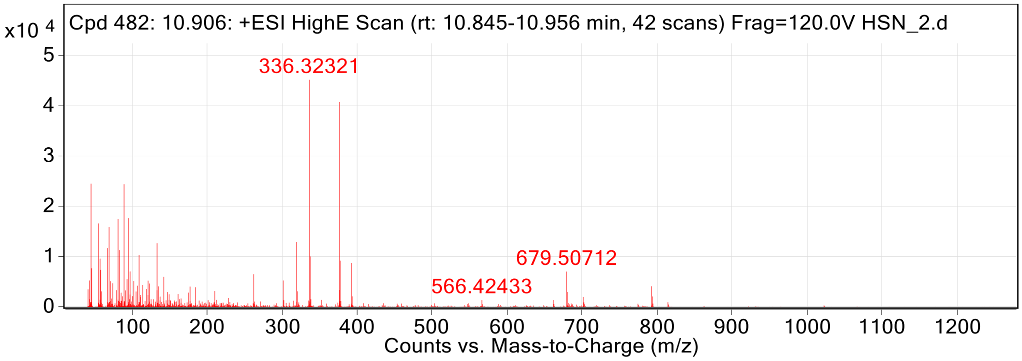

Peak List

m/z	z	Abund
45.03318		24461.26
55.05373		16542.73
69.06913		15857.43
81.06883	1	17460.14
89.05851	1	24321.63
95.08418	1	17585.17
133.08465		12603.26
319.29688	1	12901.96
336.32321	1	45099.77
376.3155	1	40647.83

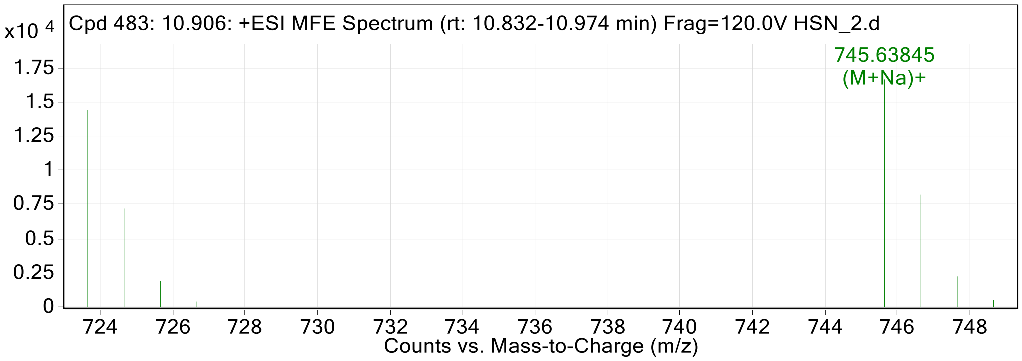

Peak List

m/z	z	Abund	Ion
723.65639	1	14429.61	(M+H)+
724.65947	1	7209.19	(M+H)+
725.66237	1	1914.53	(M+H)+
726.66656	1	393.05	(M+H)+
745.63845	1	16658.01	(M+Na)+
746.64205	1	8225.65	(M+Na)+
747.6444	1	2234.94	(M+Na)+
748.64603	1	506.91	(M+Na)+

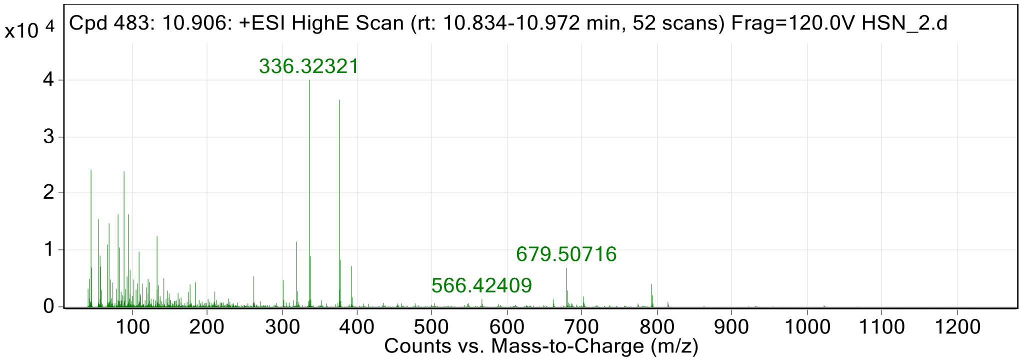

Peak List

m/z	z	Abund
45.03317		24156.71
55.05372		15445.1
69.06912		14714.66
81.06882	1	16288.83
89.05851		23853.84
95.08417	1	16304.69
133.08462		12444.9
319.29688	1	11491.9
336.32321	1	39964.77
376.3155	1	36459.46

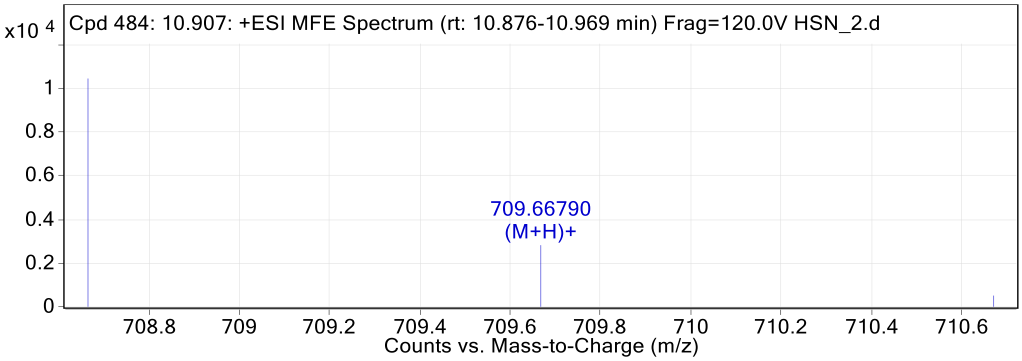

Peak List

m/z	z	Abund	Ion
708.66494	1	10447.36	(M+H)+
709.6679	1	2819.68	(M+H)+
710.67106	1	512.42	(M+H)+

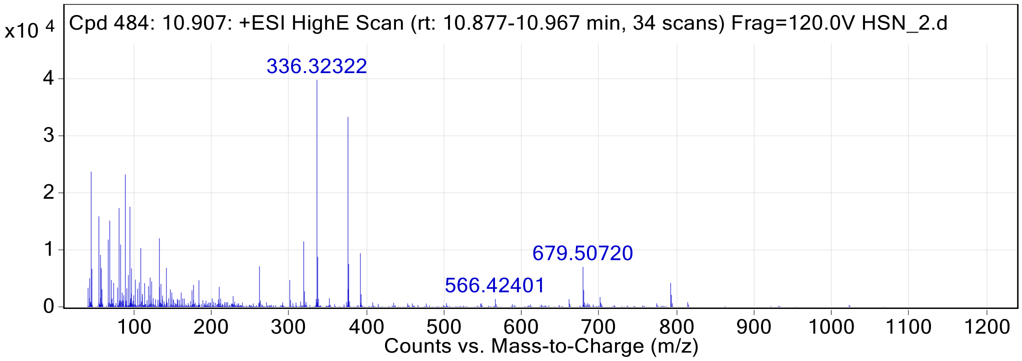

Peak List

m/z	z	Abund
45.03315		23718.71
55.05375		15881.36

67.05346		11792.32
69.06913		15122.26
81.06884	1	17332.33
89.05848		23206.71
95.08416	1	17551.94
133.08457		12027.37
336.32322	1	39801.31
376.31557	1	33322.84

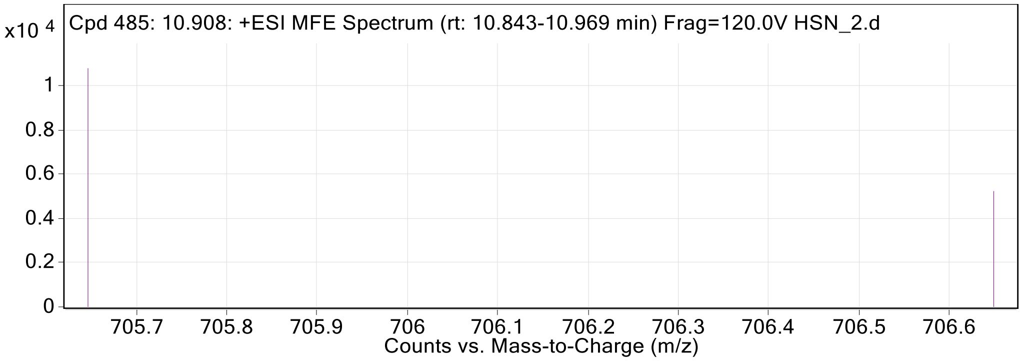

Peak List

m/z	z	Abund	Ion
705.64627	1	10794.49	(M+H)+
706.64931	1	5237.54	(M+H)+

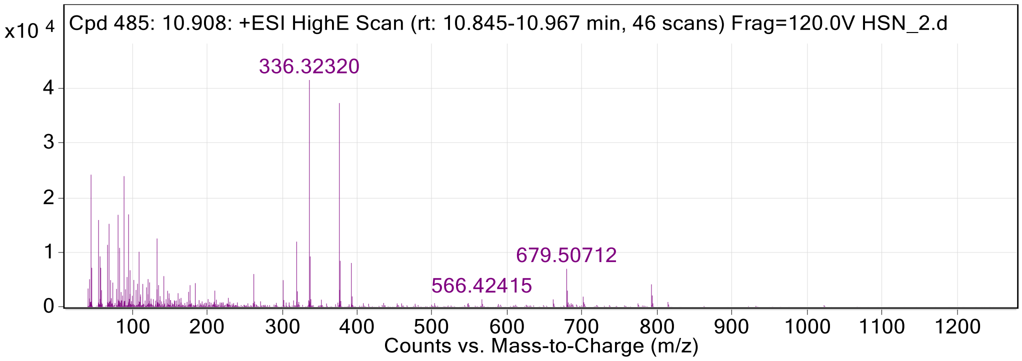

Peak List

m/z	z	Abund
45.03317		24211.65
55.05373		15900.21
69.06912		15196.36
81.06882	1	16848.88
89.0585	1	23940.66
95.08417	1	16929.25
133.08463		12507.89
319.29689	1	11931.57
336.3232	1	41573.36
376.31549	1	37343.87

Qualitative Analysis Report

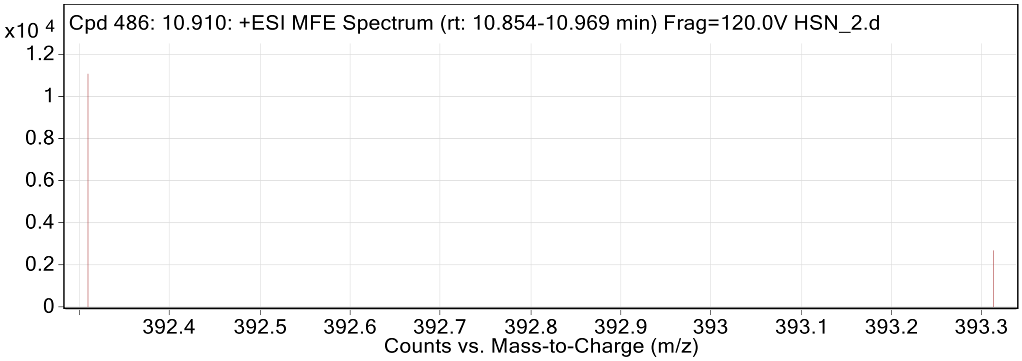

Peak List

m/z	z	Abund	Ion
392.30933	1	11051.32	(M+H)+
393.31327	1	2672.64	(M+H)+

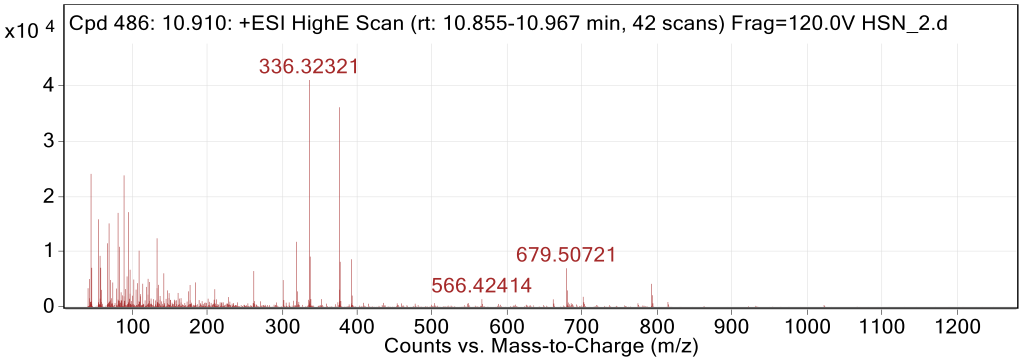

Peak List

m/z	z	Abund
45.03317		24053.54
55.05373		15827.16
69.06912		15086.9
81.06883	1	16990.09
89.05851		23783.84
95.08418	1	17124.12
133.08457		12391.3
319.29692	1	11754.57
336.32321	1	41054.43
376.31553	1	36117.84

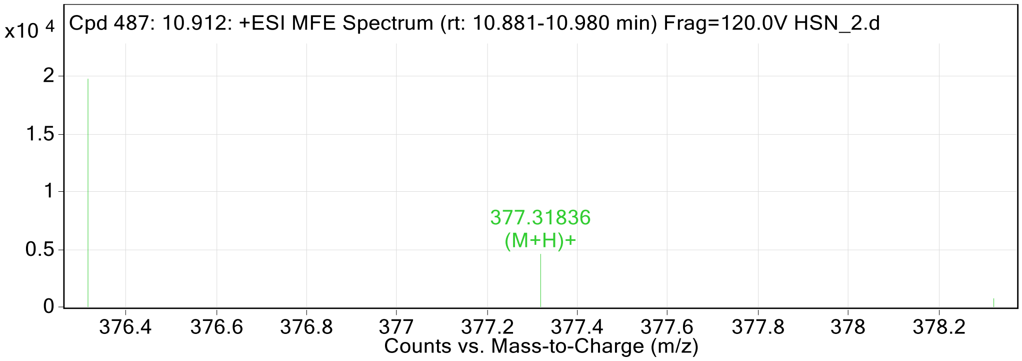

Peak List

m/z	z	Abund	Ion
376.31547	1	19812.61	(M+H)+
377.31836	1	4602.31	(M+H)+
378.32232	1	749.54	(M+H)+

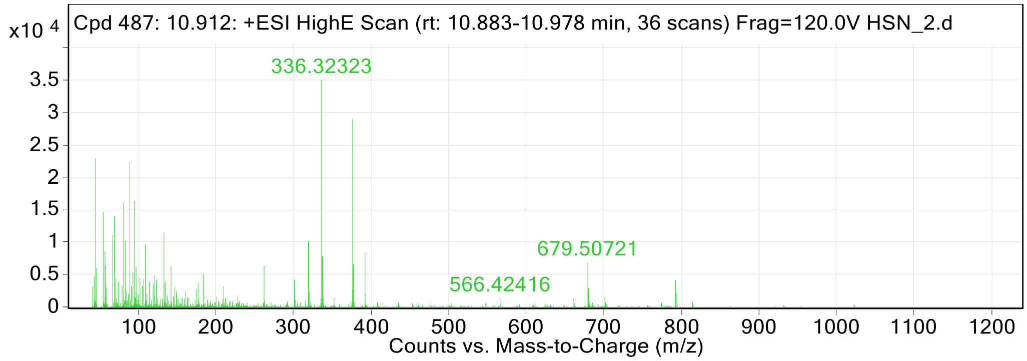

Peak List

m/z	z	Abund
45.03314		22955.81
55.05372		14712.01
67.05344		11049.85
69.06911		14002.84
81.06884	1	16153.51
89.0585		22441.95
95.08415	1	16396.71
133.08455		11446.56
336.32323	1	35068.85
376.31558	1	28999.67

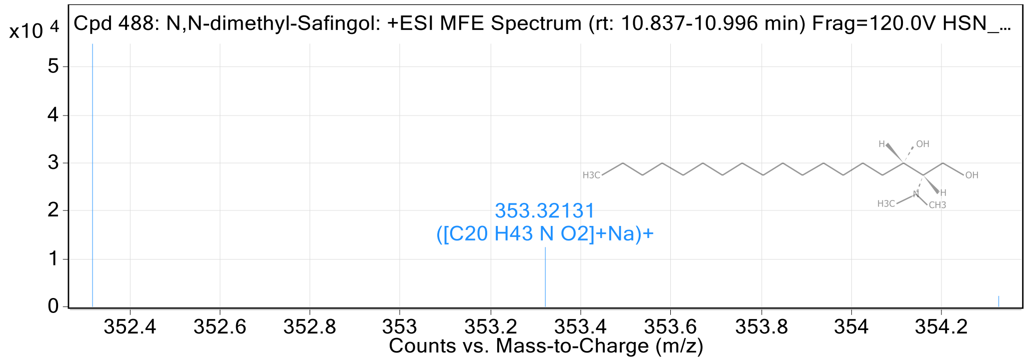

Peak List

m/z	z	Abund	Name	Formula	Ion	Score (DB)	Hits (DB)
352.31781	1	54917.07	N,N-dimethyl-Safingol	C20 H43 N O2	(M+Na)+	97.53	1
353.32131	1	12450.98		C20 H43 N O2	(M+Na)+		
354.32617	1	2264.98		C20 H43 N O2	(M+Na)+		

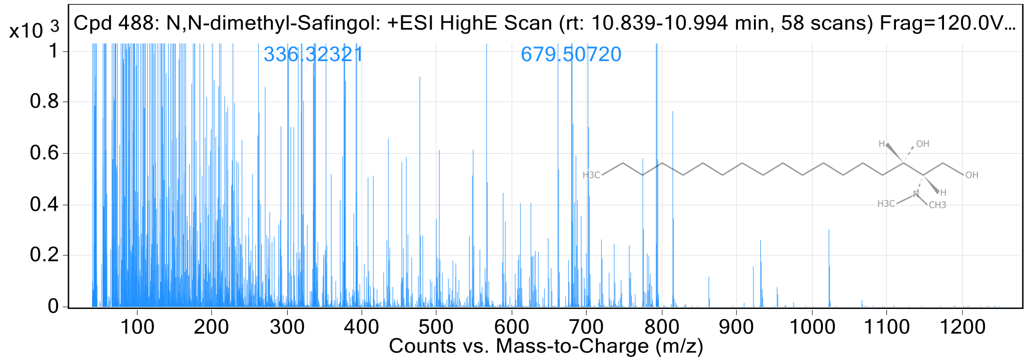

Peak List

m/z	z	Abund
45.03316		23028.38
55.05372		13919.98

67.05344		10037.33
69.06911		13248.35
81.06881		14817.56
89.0585	1	22756.49
95.08417	1	14815.3
133.08458		11847.71
336.32321	1	34507.47
376.31549	1	31267.4

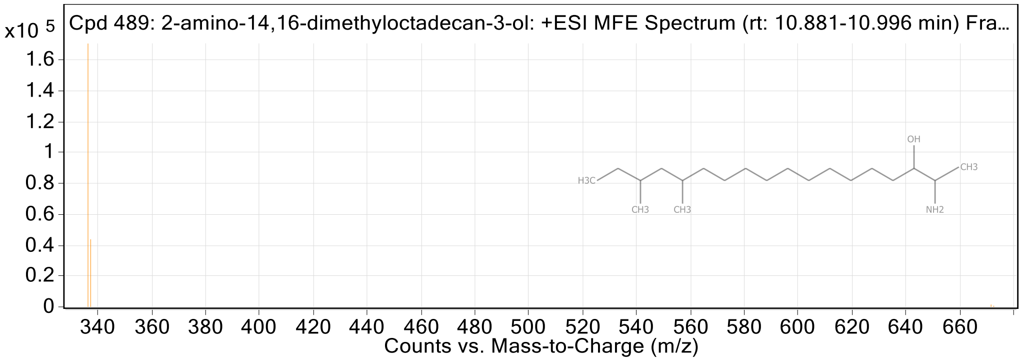

Peak List

m/z	z	Abund	Name	Formula	Ion	Score (DB)	Hits (DB)
336.32288	1	170665.5	2-amino-14,16-dimethyloctadecan-3-ol	C20 H43 N O	(M+Na)+	80.6	1
337.32586	1	43769.48		C20 H43 N O	(M+Na)+		
671.64056	1	1514.02			(2M+H)+		
672.64459	1	744.22			(2M+H)+		

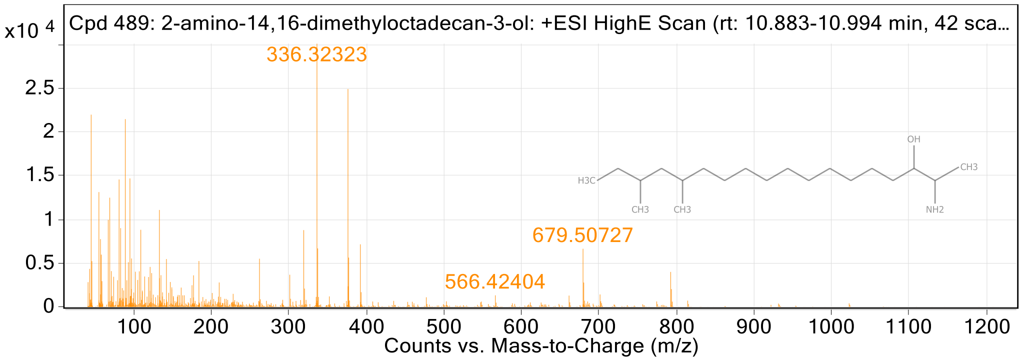

Peak List

m/z	z	Abund
45.03314		21998.6
55.05373		13130.19
67.05344		9952.7
69.06911		12488.94
81.06882		14574.35
89.0585		21484
95.08416	1	14698.1
133.08452		11082.34
336.32323	1	30135.93
376.31558	1	24934.76

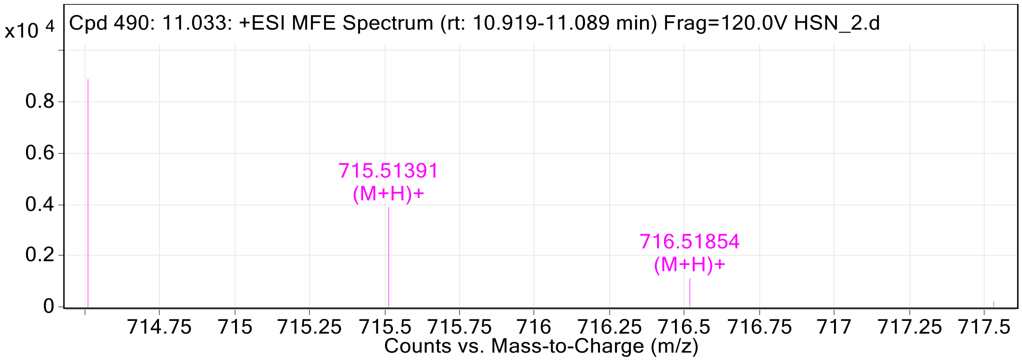

Peak List

m/z	z	Abund	Ion
714.51045	1	8894.44	(M+H)+
715.51391	1	3895.53	(M+H)+
716.51854	1	1128.5	(M+H)+
717.53232	1	203.22	(M+H)+

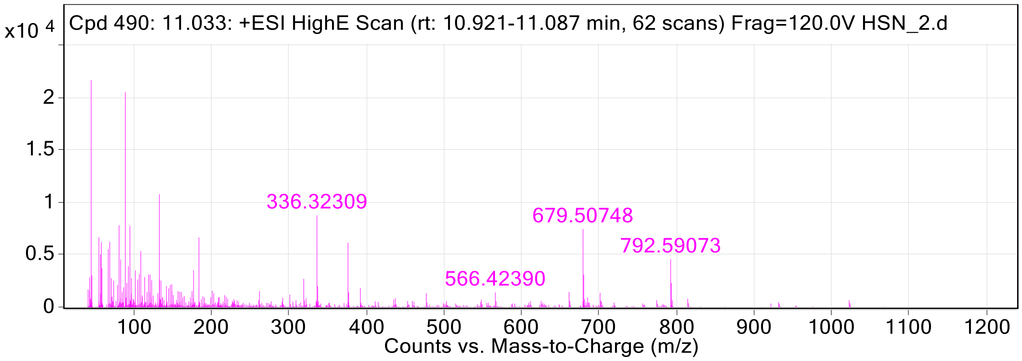

Peak List

m/z	z	Abund
45.03317		21678.13
55.0537		6670.67
69.06911		6216.92
81.0688		7810.73
89.0585	1	20504.91
95.08411		7743.94
133.08443		10780.46
184.071		6640.69
336.32309	1	8764.18
679.50748	1	7444.8

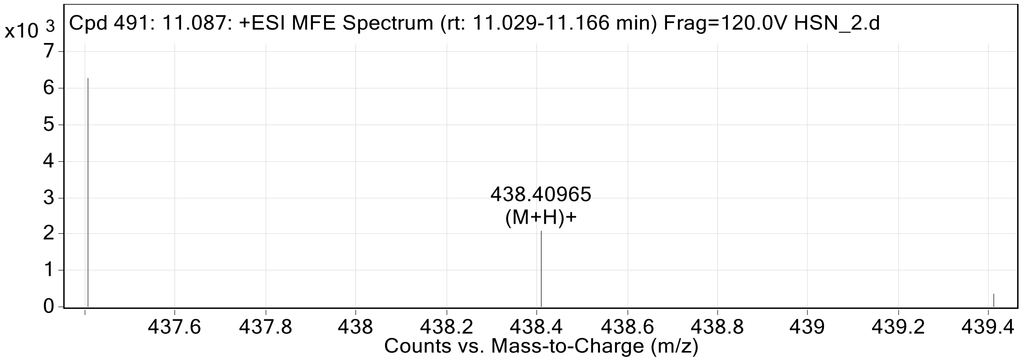

Peak List

m/z	z	Abund	Ion
437.40688	1	6285.61	(M+H)+

438.40965	1	2091.29	(M+H)+
439.41079	1	359.46	(M+H)+

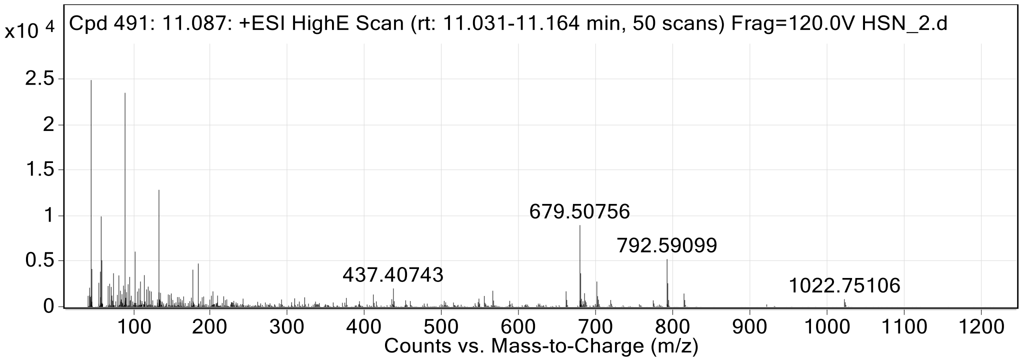

Peak List

m/z	z	Abund
45.03321		24882.15
46.06473		4148.42
58.06466		9914.81
59.0486		5099.14
89.05854	1	23480.84
102.12625		6052.45
133.08439	1	12834.39
184.07103		4744.68
679.50756	1	8960.69
792.59099	1	5240.09

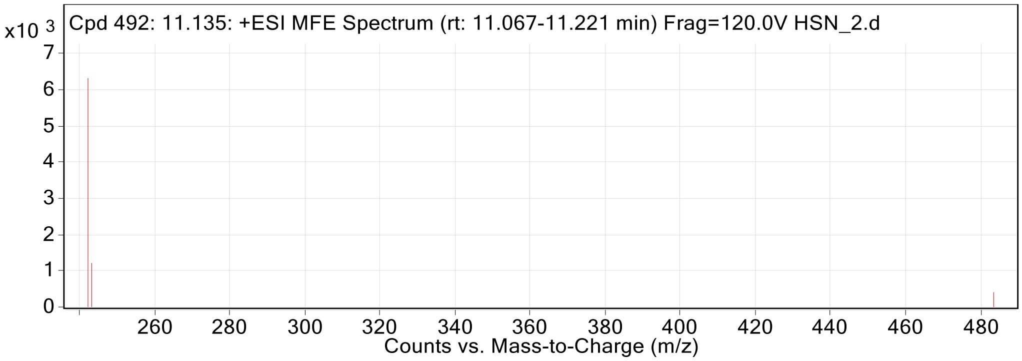

Peak List

m/z	z	Abund	Ion
242.2454	1	6330.39	(M+H)+
243.24863	1	1215.22	(M+H)+
483.48436	1	410.49	(2M+H)+

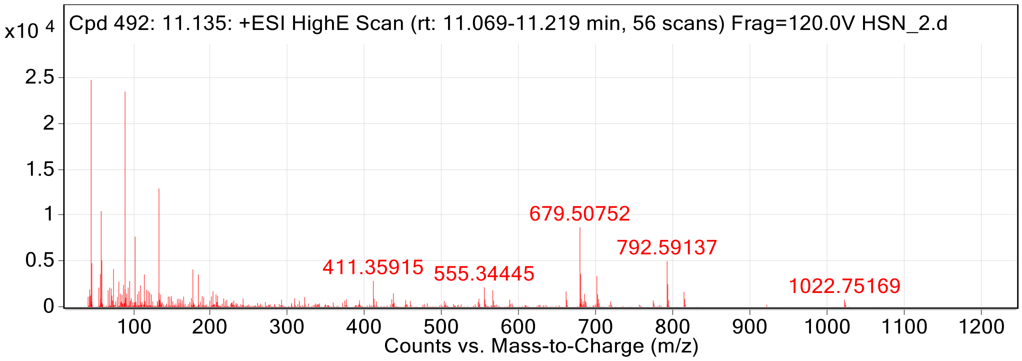

Peak List

m/z	z	Abund

45.03322		24741.53
46.06472		4755.28
58.06463		10448.23
59.04862		5050.61
74.09558		4121.16
89.05855	1	23487.01
102.12629		7659.12
133.08435	1	12918.84
679.50752	1	8676.93
792.59137	1	4987.68

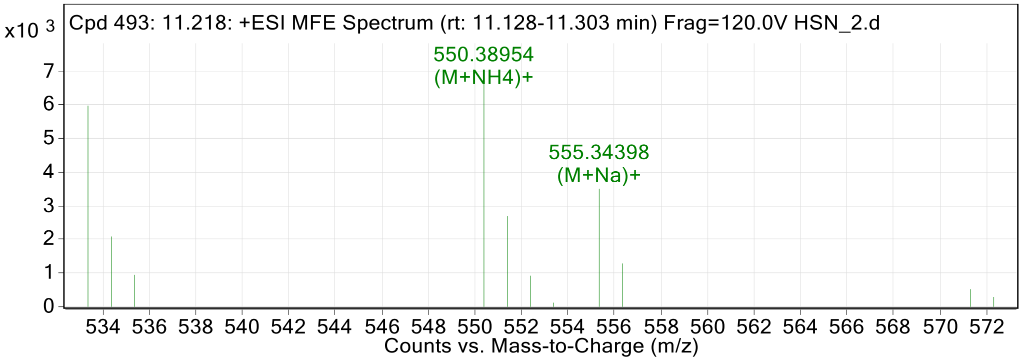

Peak List

m/z	z	Abund	Ion
533.36253	1	5984.13	(M+H)+
534.36595	1	2089.42	(M+H)+
535.36346	1	950.73	(M+H)+
550.38954	1	6770.22	(M+NH4)+
551.39176	1	2698.5	(M+NH4)+
552.39105	1	924.18	(M+NH4)+
555.34398	1	3512.52	(M+Na)+
556.34825	1	1287.19	(M+Na)+
571.31868	1	523.16	(M+K)+
572.3122	1	292.6	(M+K)+

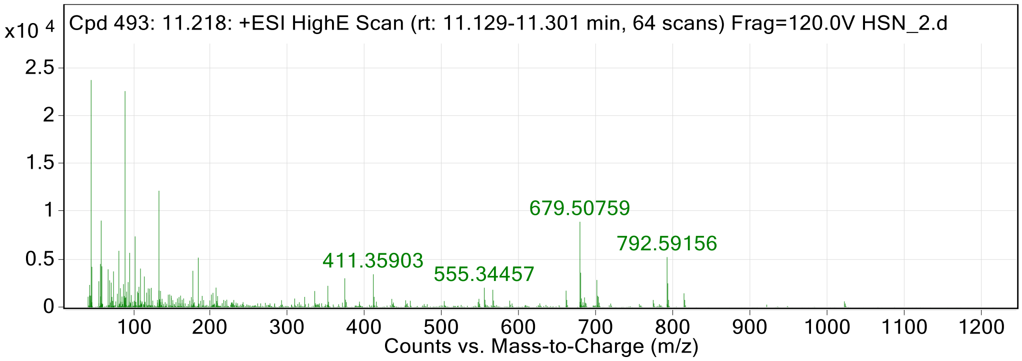

Peak List

m/z	z	Abund
45.03322		23678.97
58.06462		9015.27
81.06891		5864.01
89.05857	1	22523.72
95.0842		5652.86
102.12629		7366.23
133.08435	1	12129.27
184.07116		5151.48
679.50759	1	8892.07

792.59156	1	5209.95

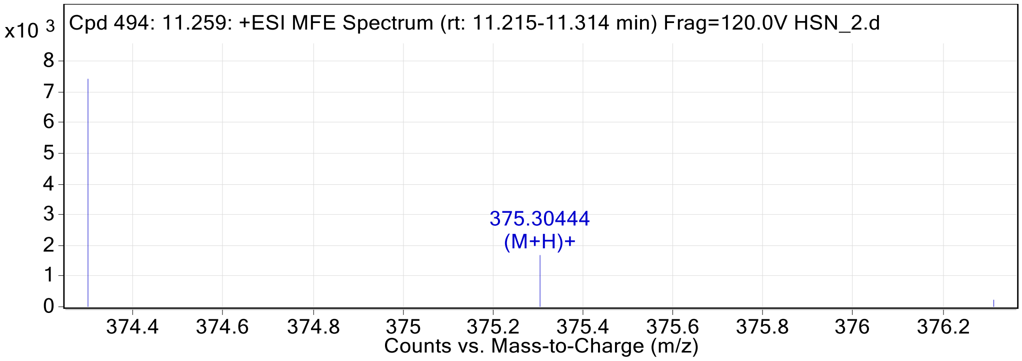

Peak List

m/z	z	Abund	Ion
374.29969	1	7428.88	(M+H)+
375.30444	1	1685.04	(M+H)+
376.31292	1	229.12	(M+H)+

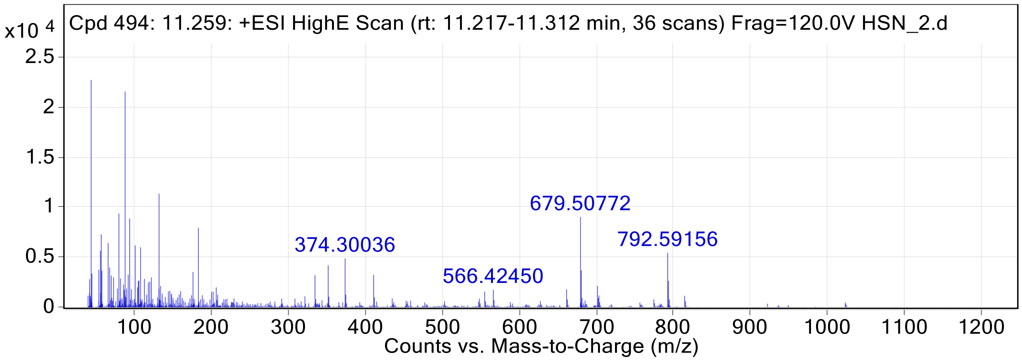

Peak List

m/z	z	Abund
45.03322		22737.26
58.06467		7279.6
67.0535		6409.34
81.06888		9355.48
89.05855	1	21590.81
95.08423		8866.22
102.12629		6169.7
133.08439		11351.59
184.07107		7944.67
679.50772	1	9018.18

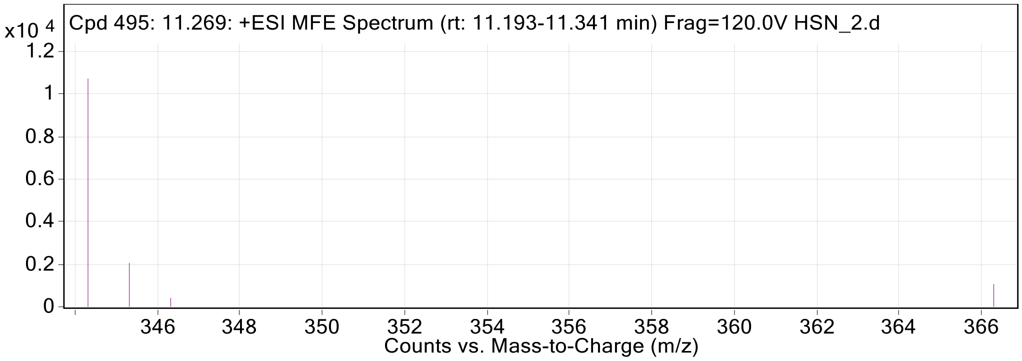

Peak List

m/z	z	Abund	Ion

344.31271	1	10715.94	(M+H)+
345.31576	1	2062.02	(M+H)+
346.32113	1	417.74	(M+H)+
366.29581	1	1070.14	(M+Na)+

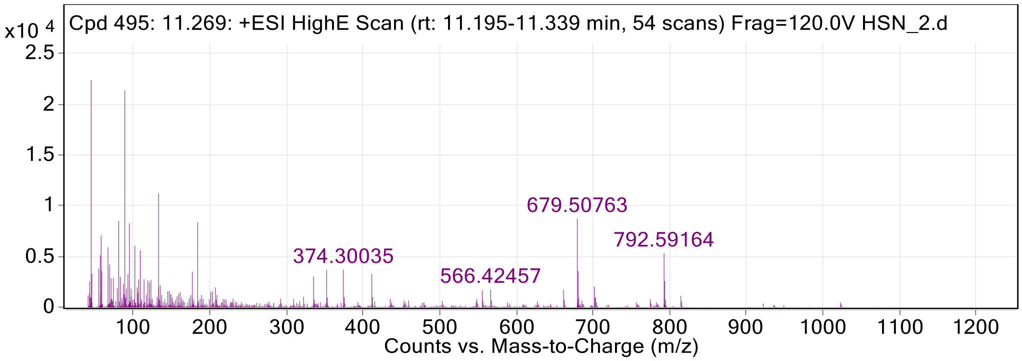

Peak List

m/z	z	Abund
45.03321		22405.33
58.06467		7055.26
67.05347		5913.61
81.06887		8506.22
89.05855	1	21388.6
95.0842		8275.05
102.12628		6067.27
133.08438		11194.64
184.07107		8391.25
679.50763	1	8721.56

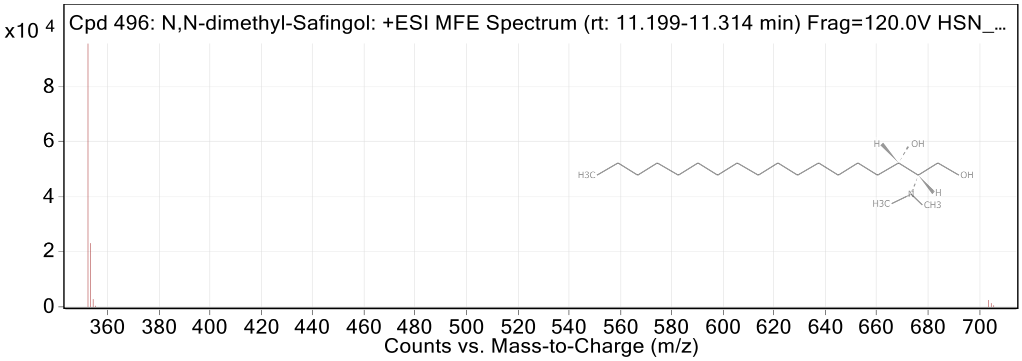

Peak List

m/z	z	Abund	Name	Formula	Ion	Score (DB)	Hits (DB)
352.31814	1	95642.29	N,N-dimethyl-Safingol	C20 H43 N O2	(M+Na)+	98.91	1
353.32165	1	23116.98		C20 H43 N O2	(M+Na)+		
354.32494	1	2844.94		C20 H43 N O2	(M+Na)+		
355.32776	1	418.07		C20 H43 N O2	(M+Na)+		
703.63066	1	2518.43			(2M+H)+		
704.63261	1	1380.63			(2M+H)+		
705.6369	1	566.28			(2M+H)+		

Qualitative Analysis Report

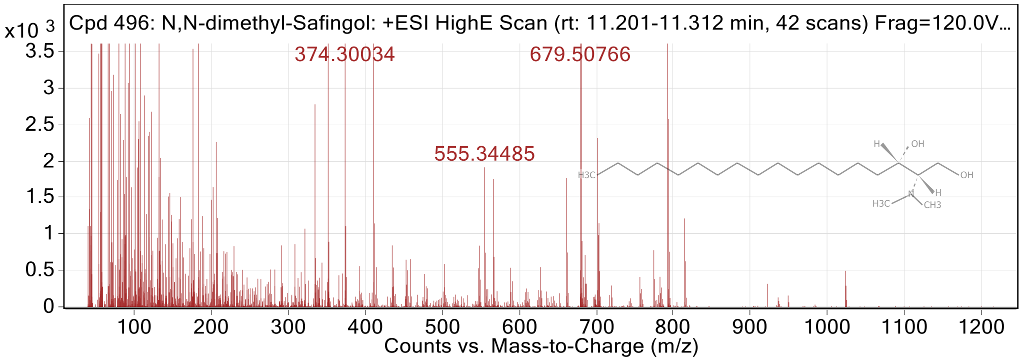

Peak List

m/z	z	Abund
45.03322		22850.14
58.06466		7600.13
67.0535		5726.58
81.06888		8395.05
89.05855	1	21696.88
95.08424		8033.19
102.1263		6786.4
133.08439	1	11520.73
184.07109		7296.71
679.50766	1	8952.35

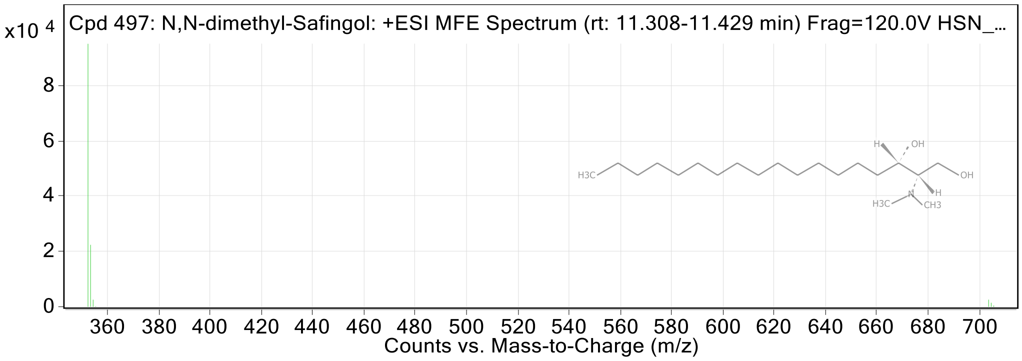

Peak List

m/z	z	Abund	Name	Formula	Ion	Score (DB)	Hits (DB)
352.31839	1	95197.26	N,N-dimethyl-Safingol	C20 H43 N O2	(M+Na)+	99.31	1
353.32131	1	22414.47		C20 H43 N O2	(M+Na)+		
354.32485	1	2565.39		C20 H43 N O2	(M+Na)+		
355.32603	1	178.56		C20 H43 N O2	(M+Na)+		
703.63019	1	2568.36			(2M+H)+		
704.63388	1	1448.95			(2M+H)+		
705.63846	1	449.93			(2M+H)+		

Qualitative Analysis Report

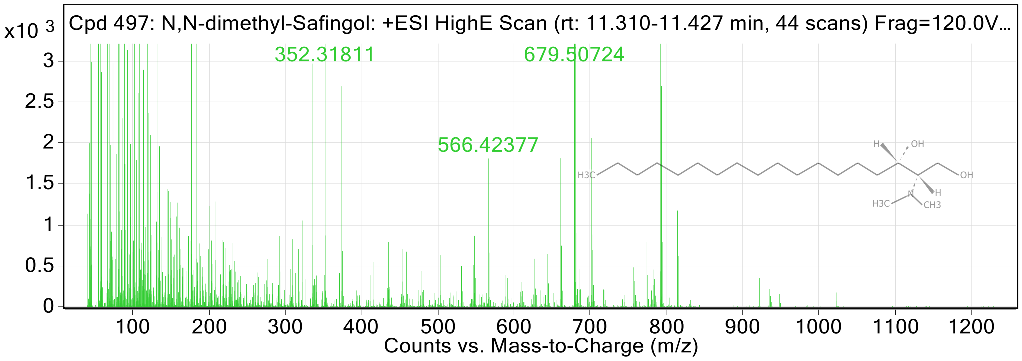

Peak List

m/z	z	Abund
45.03319		23211.79
58.06471		7595.43
81.06882		6610.6
89.05854	1	21819
95.08413		6642.5
119.08372		6138.66
133.08433		11539.69
184.07098		8313.75
679.50724	1	9094.56
792.59116	1	5516.36

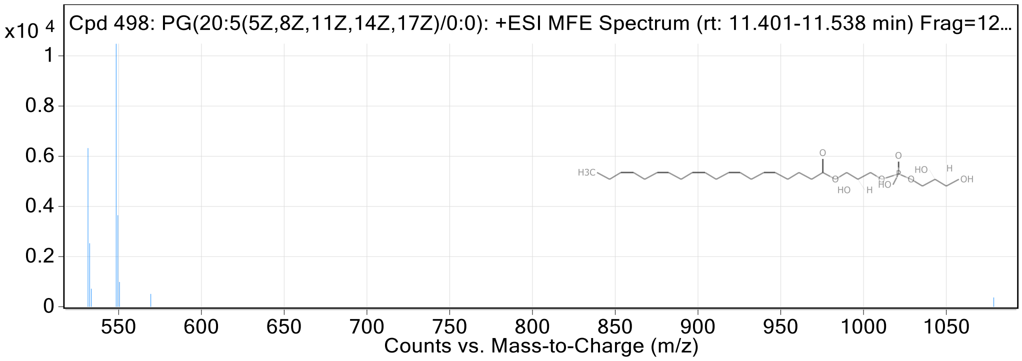

Peak List

m/z	z	Abund	Name	Formula	Ion	Score (DB)	Hits (DB)
531.2708	1	6326.5			(M+H)+		
532.27484	1	2533.1			(M+H)+		
533.27974	1	719.66			(M+H)+		
548.29661	1	10489.94	PG(20:5(5Z,8Z,11Z,14Z,17Z)/0:0)	C26 H43 O9 P	(M+NH4)+	88.71	1
549.30064	1	3651.08		C26 H43 O9 P	(M+NH4)+		
550.30549	1	988.1		C26 H43 O9 P	(M+NH4)+		
569.22549	1	511.98			(M+K)+		
1078.56098	1	371.01			(2M+NH4)+		

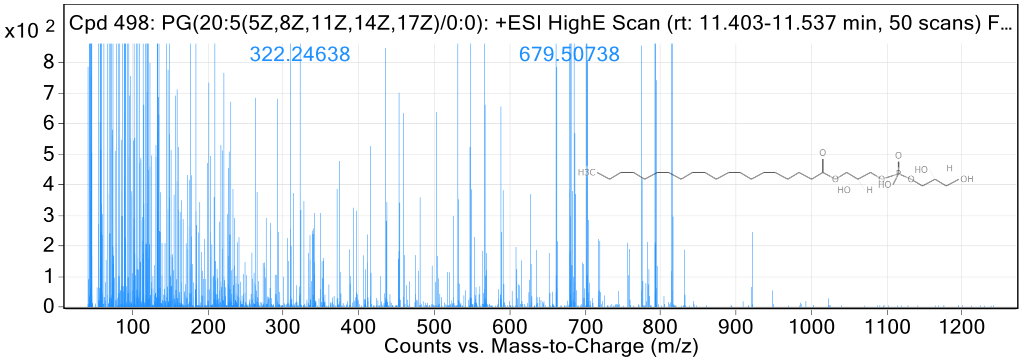

Peak List

m/z	z	Abund
45.03322		23104.08
46.06481		4180.29
58.0647		10638.24
74.09557		4517.81
89.05857	1	21709.94
102.12625	1	7136.02
119.0837	1	8950.89
133.08422	1	11912.47
679.50738	1	9241.23
792.59134	1	5165.83

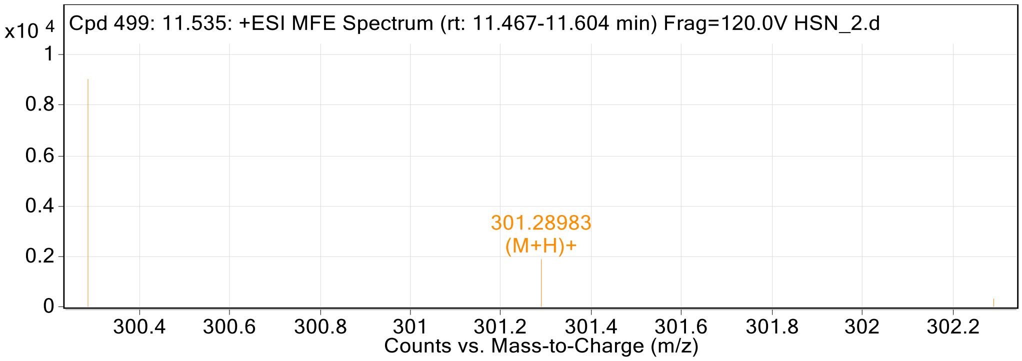

Peak List

m/z	z	Abund	Ion
300.28664	1	9040.74	(M+H)+
301.28983	1	1887.68	(M+H)+
302.29101	1	323.17	(M+H)+

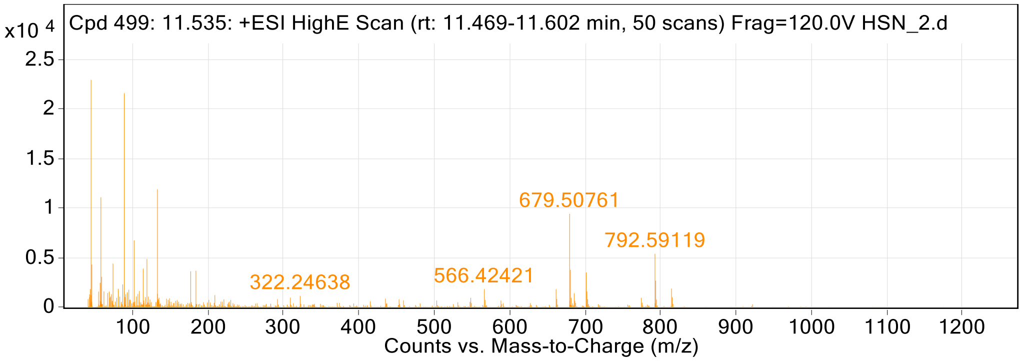

Peak List

m/z	z	Abund
45.03323		22955.48
46.06483		4285.47

58.06463		11078.76
74.09555		4383.52
89.05856	1	21597.25
102.12624	1	6719.78
119.08371		4833.72
133.08416	1	11887.62
679.50761	1	9416.15
792.59119	1	5370.43

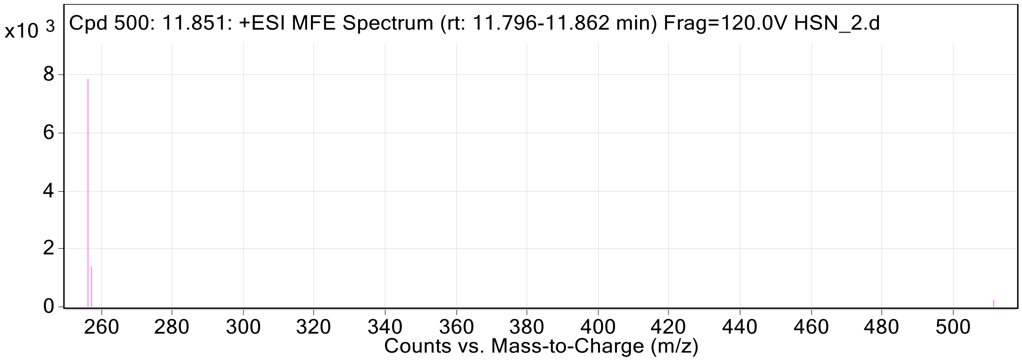

Peak List

m/z	z	Abund	Ion
256.26108	1	7860.68	(M+H)+
257.26438	1	1393.28	(M+H)+
511.51498	1	243.14	(2M+H)+

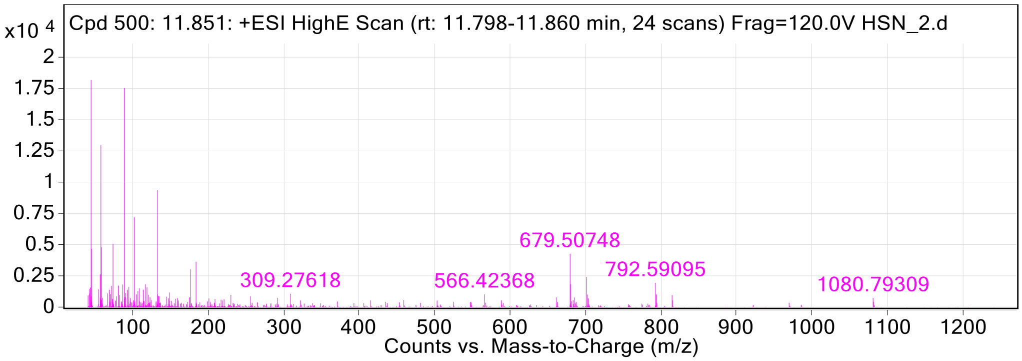

Peak List

m/z	z	Abund
45.03318		18137.88
46.06479		4644.71
58.06457	1	12941.4
59.0486		4790.71
74.09556		5037
89.05854	1	17496.38
102.12609		7180.15
133.08419	1	9334.32
184.07105		3625.52
679.50748	1	4255.19

Qualitative Analysis Report

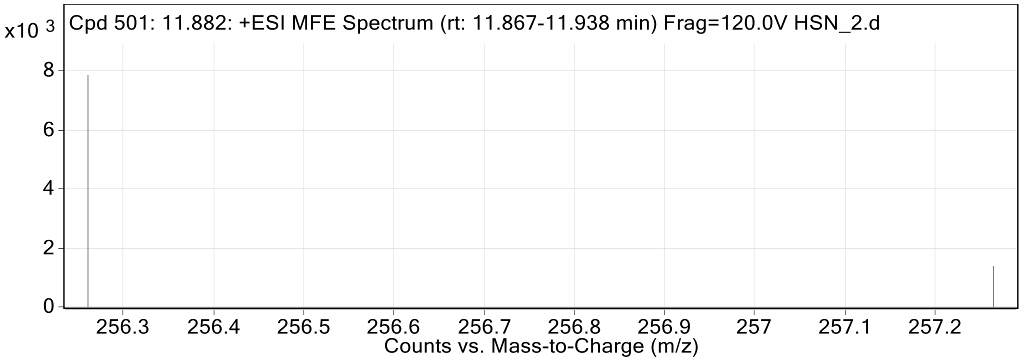

Peak List

m/z	z	Abund	Ion
256.26083	1	7865.96	(M+H)+
257.26491	1	1392.66	(M+H)+

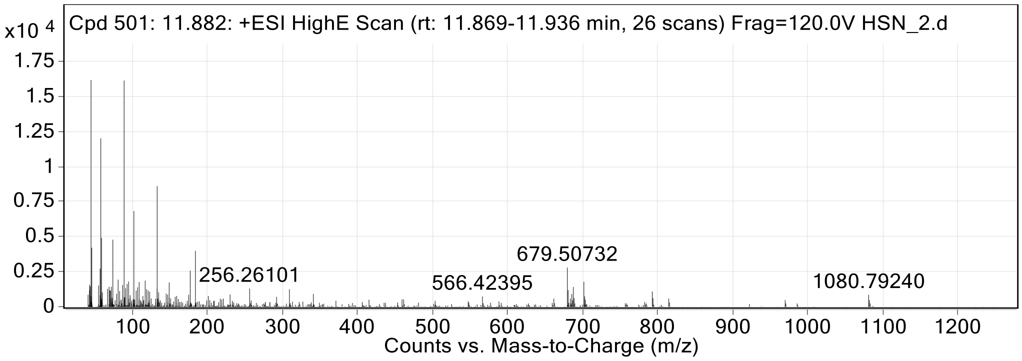

Peak List

m/z	z	Abund
45.03323		16168.7
46.06488		4204.8
58.06465	1	12010.87
59.04865		4909.36
74.09558		4780.22
89.0585	1	16132.52
102.12634	1	6821.73

m/z	z	Abund
133.08429	1	8594.62
184.07118		3983.81
679.50732	1	2791.62

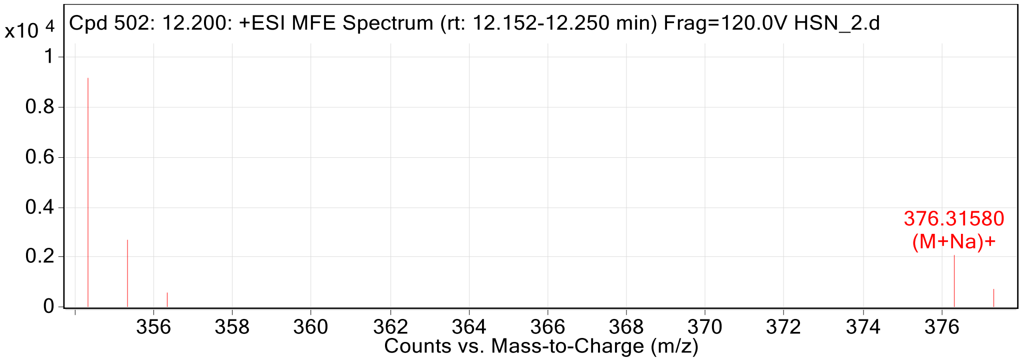

Peak List

m/z	z	Abund	Ion
354.3341	1	9177.43	(M+H)+
355.33696	1	2689.7	(M+H)+
356.34465	1	573.7	(M+H)+
376.3158	1	2078.51	(M+Na)+
377.31803	1	718.59	(M+Na)+

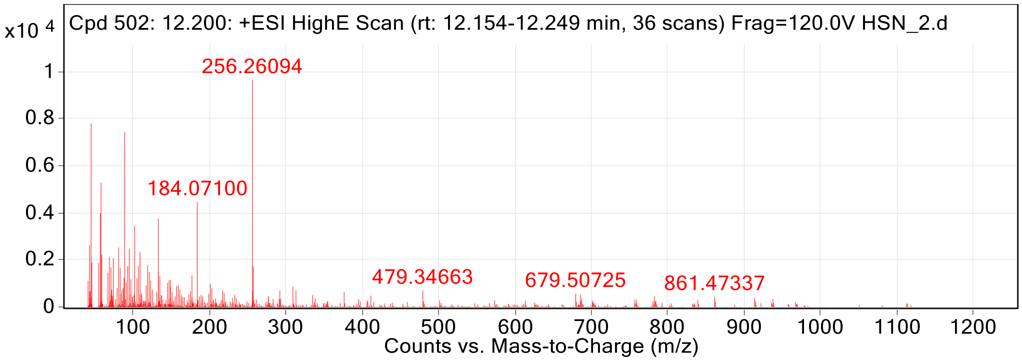

Peak List

m/z	z	Abund
43.05398		2625.24
45.03317		7797.72
57.06934		3976.79
58.06456		5283.69
81.06888		2535.15
89.05857		7434.14
102.12609		3449.11
133.08464		3758.24
184.071		4462.29
256.26094	1	9645.29

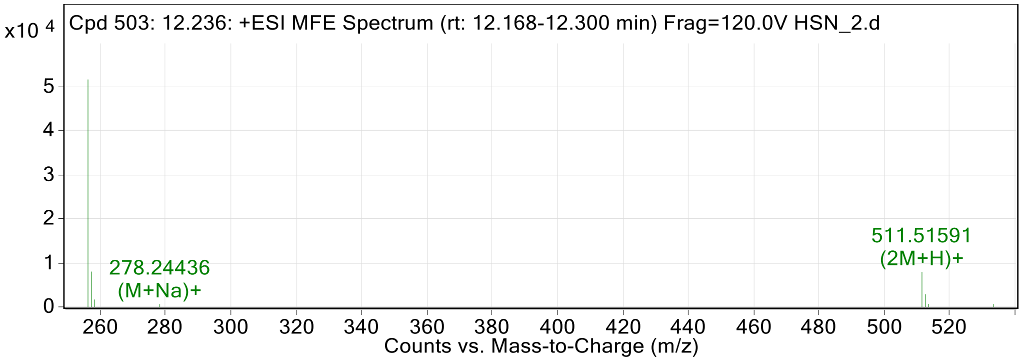

Peak List

m/z	z	Abund	Ion
256.2612	1	51530.11	(M+H)+
257.26428	1	7991.63	(M+H)+
258.27146	1	1666.61	(M+H)+
278.24436	1	634.12	(M+Na)+
511.51591	1	7923.59	(2M+H)+
512.51918	1	2875.21	(2M+H)+
513.52085	1	671.31	(2M+H)+
533.49465	1	641.68	(2M+Na)+

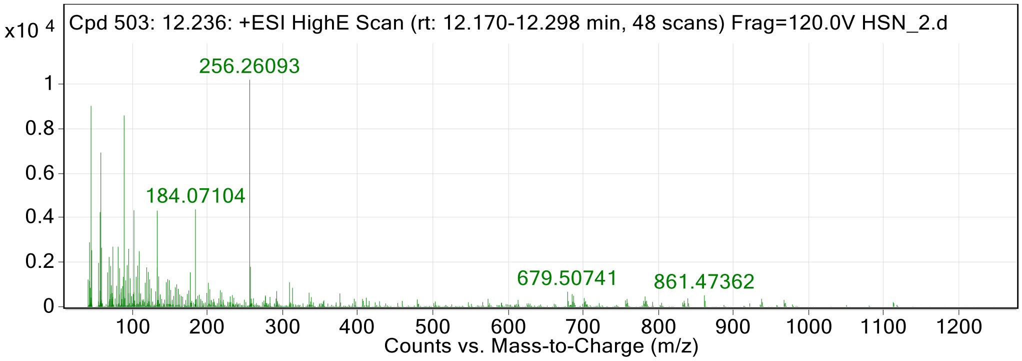

Peak List

m/z	z	Abund
43.05392		2900.26
45.0332		9026.83
57.0694		4255.06
58.06462		6930.97
74.09549		2697.42
89.05855		8594.12
102.12617		4333.83
133.08447		4319.79
184.07104		4374.68
256.26093	1	10200.52

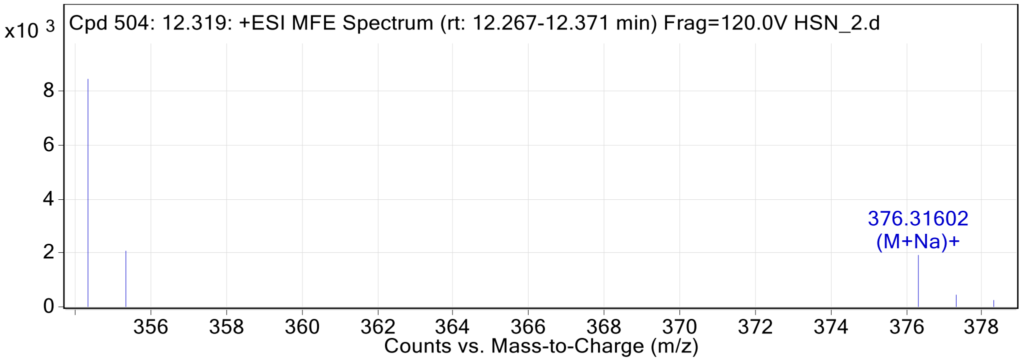

Peak List

m/z	z	Abund	Ion
354.33379	1	8457.4	(M+H)+
355.33708	1	2081.82	(M+H)+
376.31602	1	1924.04	(M+Na)+
377.3186	1	451.35	(M+Na)+
378.31269	1	252.24	(M+Na)+

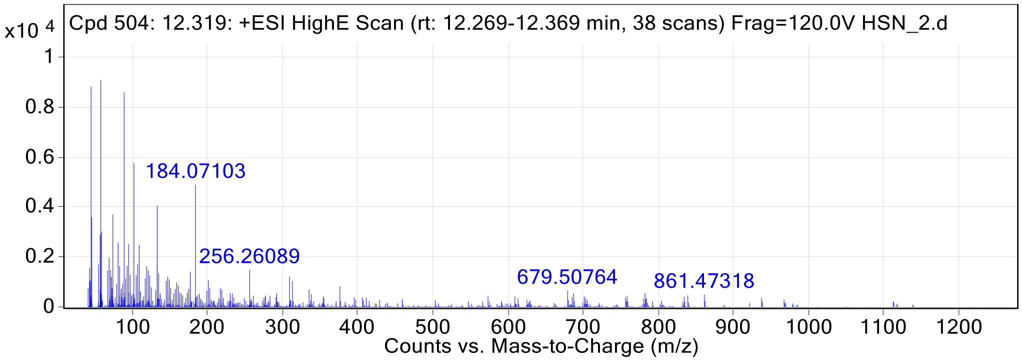

Peak List

m/z	z	Abund
45.03321		8818.82
46.06479		3584.16
57.06941		2898.42
58.06465	1	9084.08
59.04862		3010.4
74.09541		3696.54
89.05852		8586.39
102.12628		5762.89
133.08445		4051.03
184.07103		4894.09

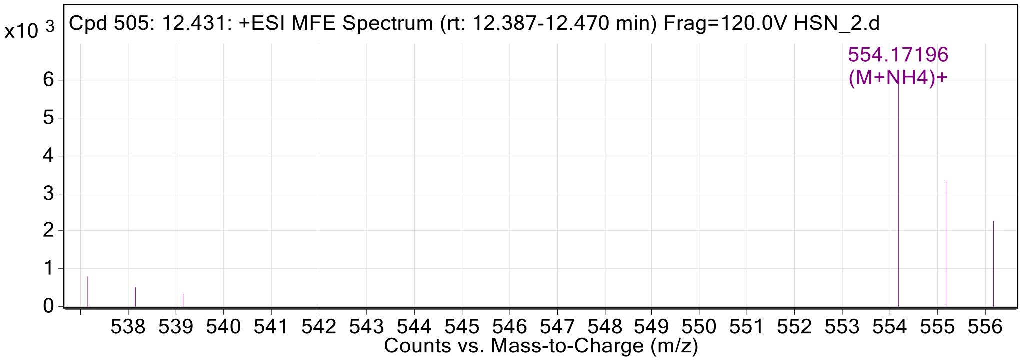

Peak List

m/z	z	Abund	Ion

537.14716	1	797.14	(M+H)+
538.14861	1	515.6	(M+H)+
539.14981	1	343.48	(M+H)+
554.17196	1	6065.84	(M+NH4)+
555.17304	1	3341.36	(M+NH4)+
556.16995	1	2275.4	(M+NH4)+

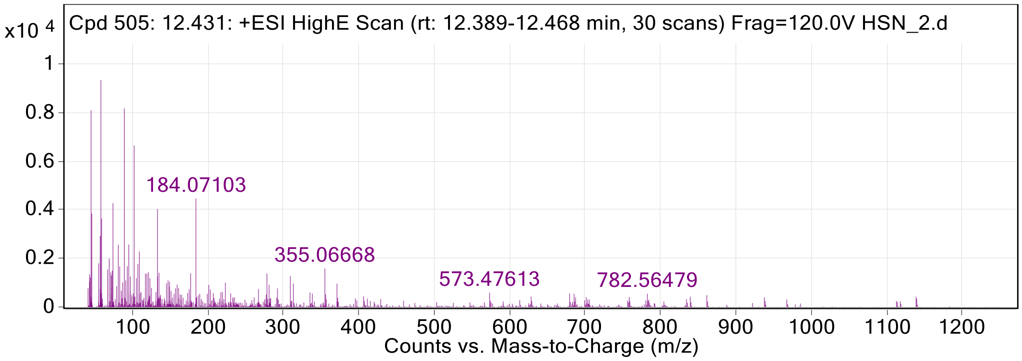

Peak List

m/z	z	Abund
45.0332		8109.3
46.0649		3847.84
57.06928		2924.86
58.0646	1	9352.15
59.04859		3644.45
74.09571		4276.72
89.05854		8177.08
102.1263		6646.62
133.08442		4033.62
184.07103		4465.58

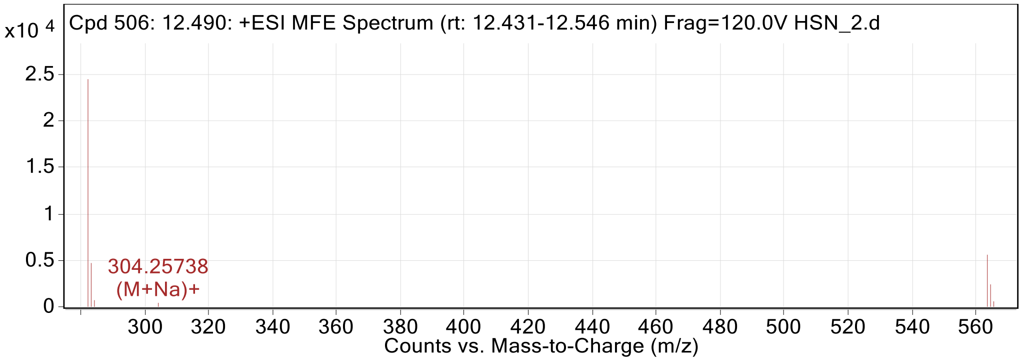

Peak List

m/z	z	Abund	Ion
282.27646	1	24518.54	(M+H)+
283.27986	1	4714.42	(M+H)+
284.28569	1	712.02	(M+H)+
304.25738	1	423.38	(M+Na)+
563.54708	1	5608.52	(2M+H)+
564.54919	1	2413.91	(2M+H)+
565.55362	1	588.07	(2M+H)+

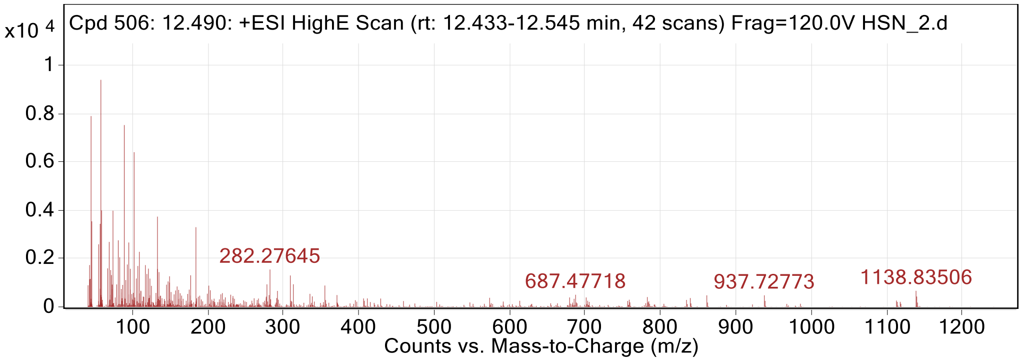

Peak List

m/z	Abund
45.03314	7891.21
46.06481	3539.28
57.06936	3435.11
58.06462	9388.96
59.04855	3989.1
74.09556	3976.6
89.05847	7518.56
102.12621	6397.04
133.08454	3732.08
184.07116	3295.48

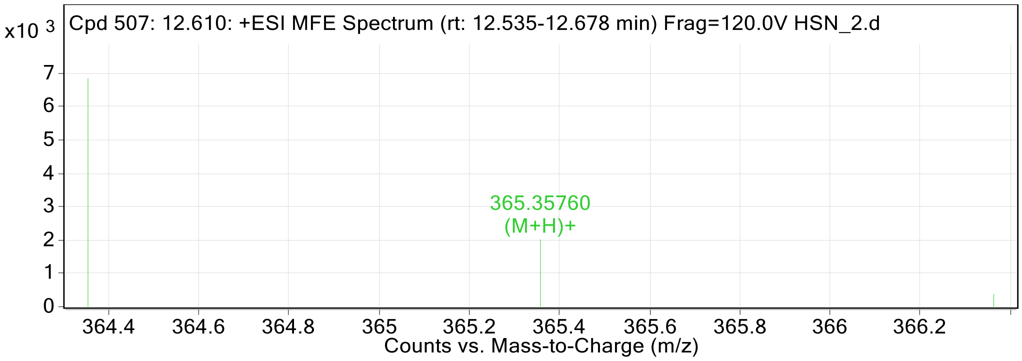

Peak List

m/z	z	Abund	Ion
364.35443	1	6841.99	(M+H)+
365.3576	1	2017.33	(M+H)+
366.36288	1	381.49	(M+H)+

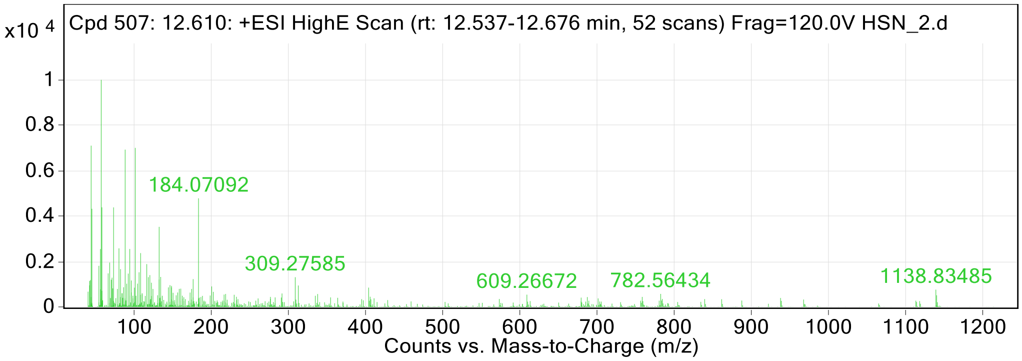

Peak List

m/z	z	Abund
45.03314		7099.56
46.06481		4321.19

58.06462	1	9994.82
59.04863		4382.99
74.09549		4380.14
81.06884		2577.02
89.05855		6923.73
102.12627	1	7000.71
133.08459		3524.13
184.07092		4778.63

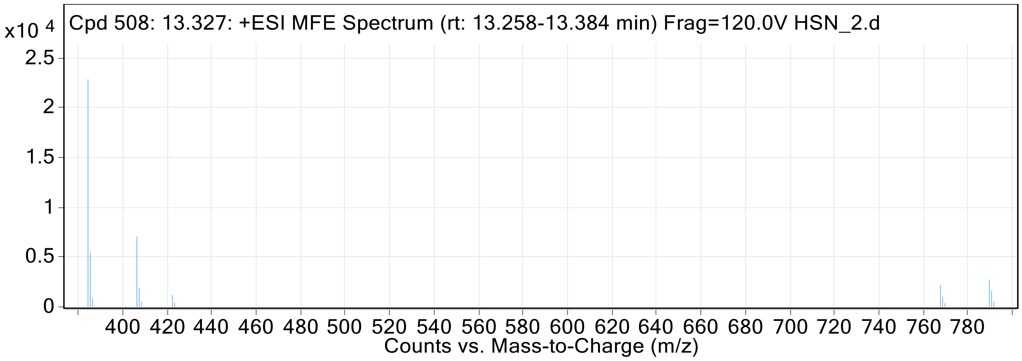

Peak List

m/z	z	Abund	Ion
384.3443	1	22862.55	(M+H)+
385.34766	1	5444.86	(M+H)+
386.34894	1	842.74	(M+H)+
406.32565	1	7063.02	(M+Na)+
407.3287	1	1846.71	(M+Na)+
422.30021	1	1187.81	(M+K)+
767.68302	1	2206.22	(2M+H)+
768.68569	1	1033.71	(2M+H)+
789.66407	1	2742.09	(2M+Na)+
790.66616	1	1602.16	(2M+Na)+

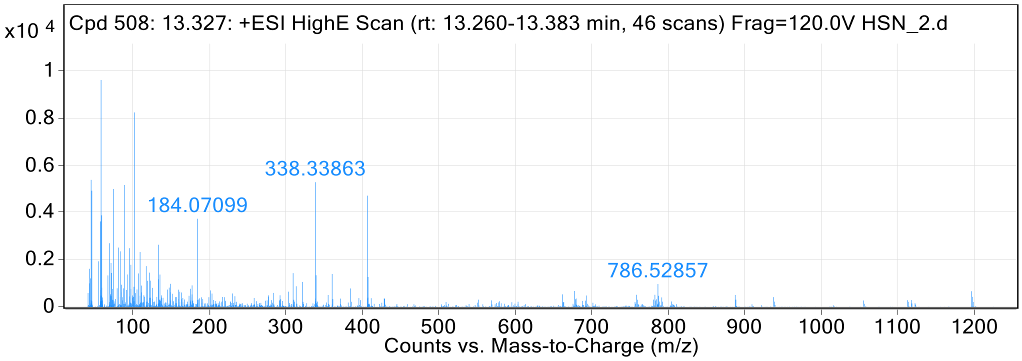

Peak List

m/z	z	Abund
45.03315		5379.48
46.06476		4920.05
58.06463		9613.09
59.04867		3875.57
74.0955		4993.33
89.05846		5162.65
102.12622	1	8238.61
184.07099		3730.28
338.33863	1	5280.03
406.32581	1	4709.57

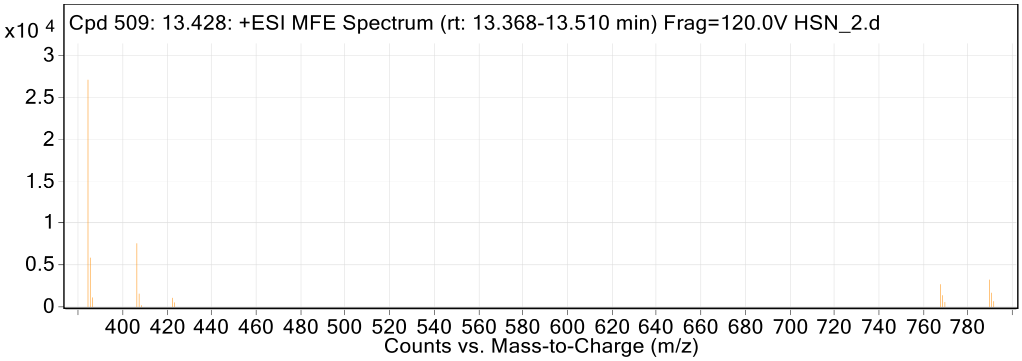

Peak List

m/z	z	Abund	Ion
384.34393	1	27155.88	(M+H)+
385.34724	1	5883.8	(M+H)+
386.35058	1	1120.49	(M+H)+
406.32604	1	7583.99	(M+Na)+
407.32924	1	1580.36	(M+Na)+
422.29881	1	1085.79	(M+K)+
767.68175	1	2697.99	(2M+H)+
768.68483	1	1383.17	(2M+H)+
789.66351	1	3254.86	(2M+Na)+
790.66686	1	1690.99	(2M+Na)+

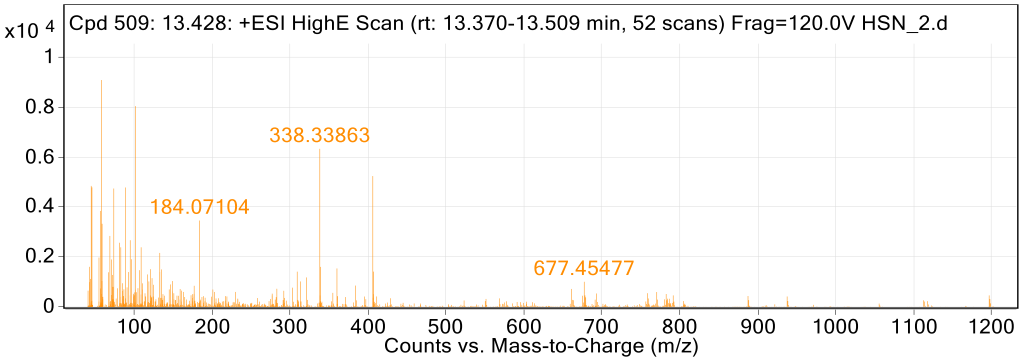

Peak List

m/z	z	Abund
45.03315		4845.17
46.06482		4786.92
57.06933		3840.18
58.06462		9090
74.09558		4740.13
89.05852		4781
102.12622	1	8043.94
184.07104		3456.71
338.33863	1	6331.67
406.32606	1	5239.72

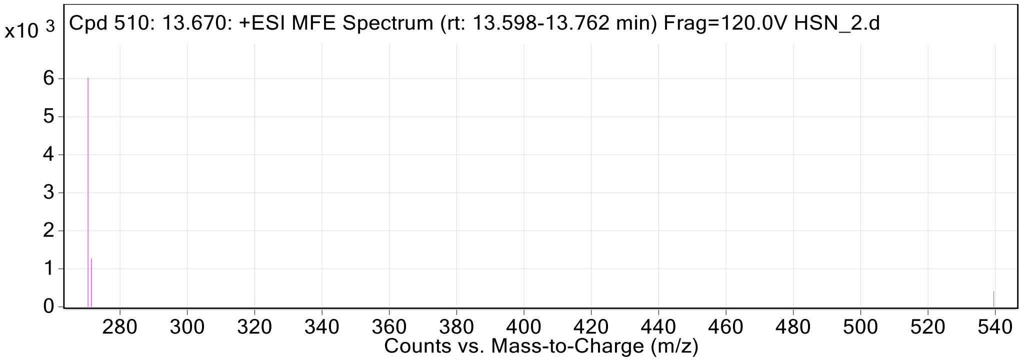

Peak List

m/z	z	Abund	Ion
270.27624	1	6036.38	(M+H)+
271.27984	1	1284.49	(M+H)+
539.54358	1	413.4	(2M+H)+

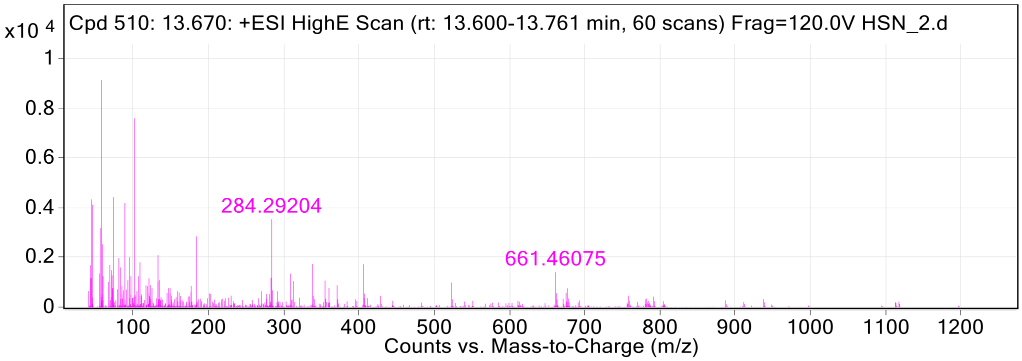

Peak List

m/z	z	Abund
45.03315		4338.96
46.06475		4129.02
57.06933		3187.69
58.06457	1	9152.97
59.04872		2526.29
74.09556		4437.67
89.05847		4197.16
102.12619	1	7610.33
184.07107		2848.51
284.29204	1	3536.81

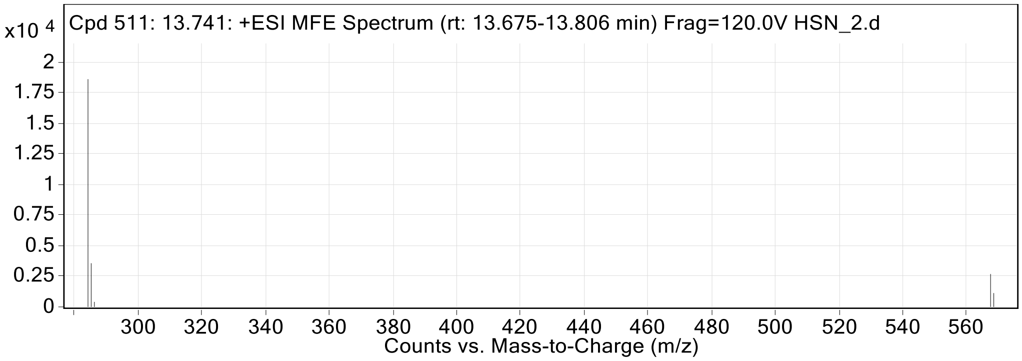

Peak List

m/z	z	Abund	Ion
284.29237	1	18616.79	(M+H)+
285.29551	1	3570.64	(M+H)+

286.29879	1	410.76	(M+H)+
567.57827	1	2688.29	(2M+H)+
568.58168	1	1130.87	(2M+H)+

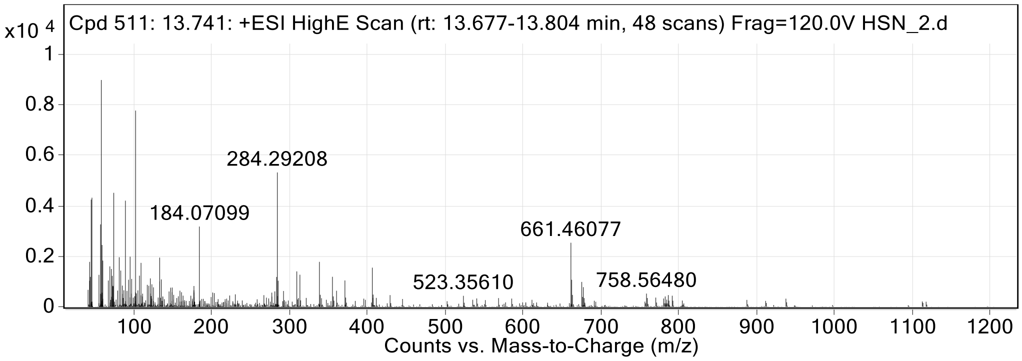

Peak List

m/z	z	Abund
45.03316		4244.54
46.06476		4319.35
57.06937		3262.07
58.06459	1	8983.66
74.09554		4512.34
89.05854		4202.21
102.12624	1	7771.13
184.07099		3176.74
284.29208	1	5319.85
661.46077	1	2534.94

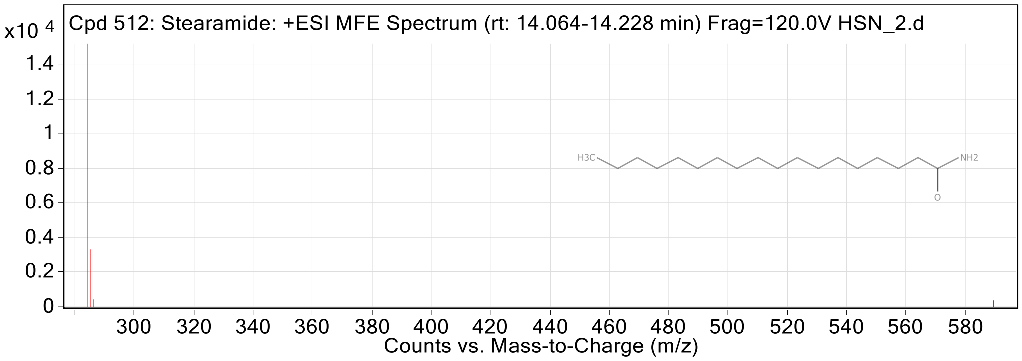

Peak List

m/z	z	Abund	Name	Formula	Ion	Score (DB)	Hits (DB)
284.29348	1	15188.57	Stearamide	C18 H37 N O	(M+H)+	93.92	1
285.29705	1	3315.14		C18 H37 N O	(M+H)+		
286.30143	1	428.68		C18 H37 N O	(M+H)+		
589.55981	1	371.72			(2M+Na)+		

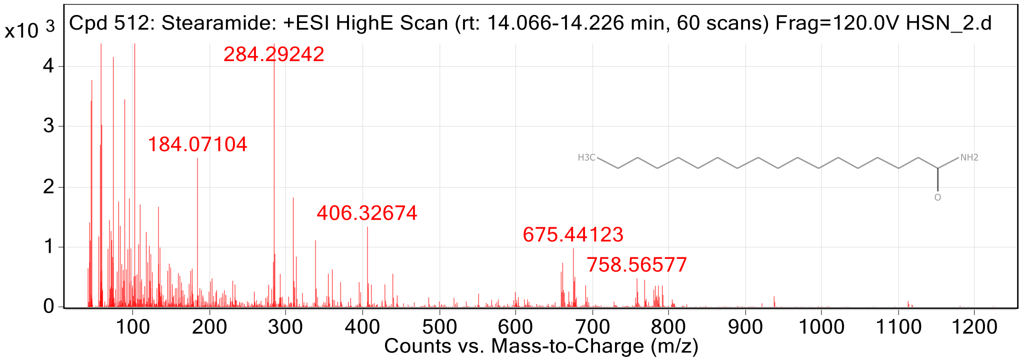

Peak List

m/z	z	Abund
45.03319		3430.51
46.06489		3776
57.0694		2696.09
58.06469	1	8499.24
59.04874		3030.93
74.0956		4160.49
89.05861		3455.88
102.12633	1	7557.4
184.07104		2479.91
284.29242	1	4385.98

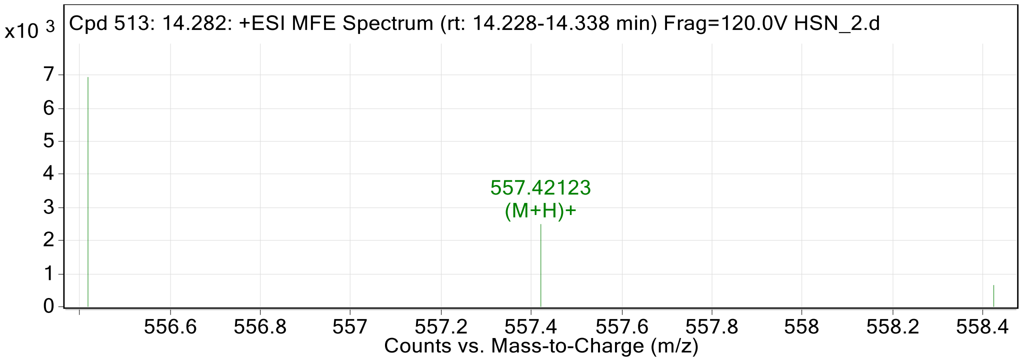

Peak List

m/z	z	Abund	Ion
556.41818	1	6941.85	(M+H)+
557.42123	1	2498.4	(M+H)+
558.4244	1	655.58	(M+H)+

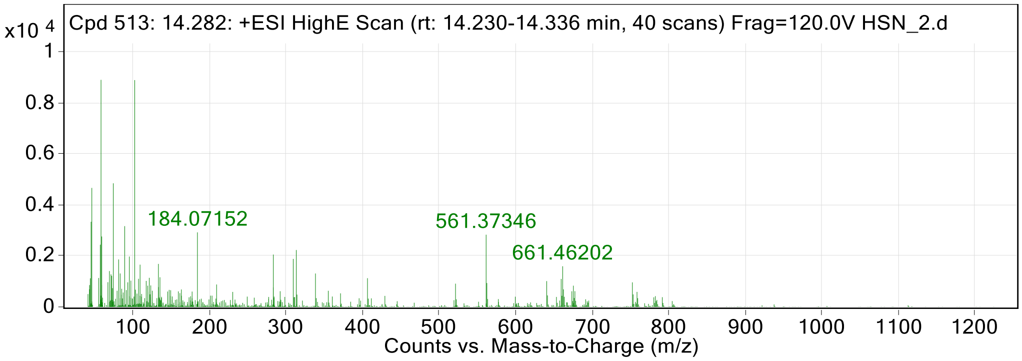

Peak List

m/z	z	Abund
45.03326		3339.15
46.06489		4664.64

57.06951		2439.63
58.06472		8901.8
59.04884		2761.05
74.09576		4846.04
89.05867		3166.03
102.12654	1	8892.21
184.07152		2921.82
561.37346	1	2831.08

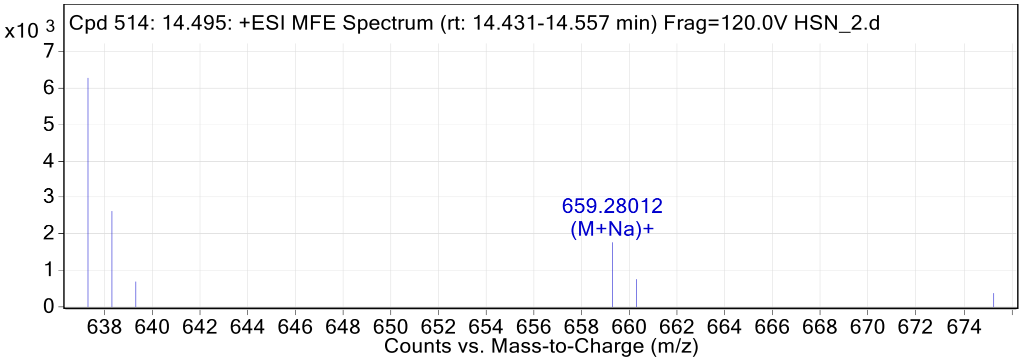

Peak List

m/z	z	Abund	Ion
637.29823	1	6279.39	(M+H)+
638.30185	1	2625.21	(M+H)+
639.30387	1	693.24	(M+H)+
659.28012	1	1767.96	(M+Na)+
660.28362	1	758.55	(M+Na)+
675.25493	1	378.08	(M+K)+

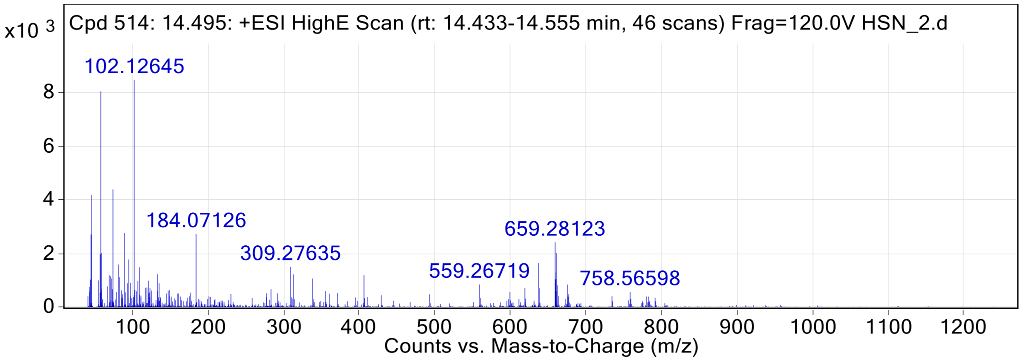

Peak List

m/z	z	Abund
45.0332		2703.67
46.06487		4173.06
58.06471		8053.19
59.04877		2020.38
74.09566		4389.02
89.05864		2759.3
102.12645	1	8483.89
184.07126		2724.55
659.28123	1	2411.37
661.46185	1	2011.77

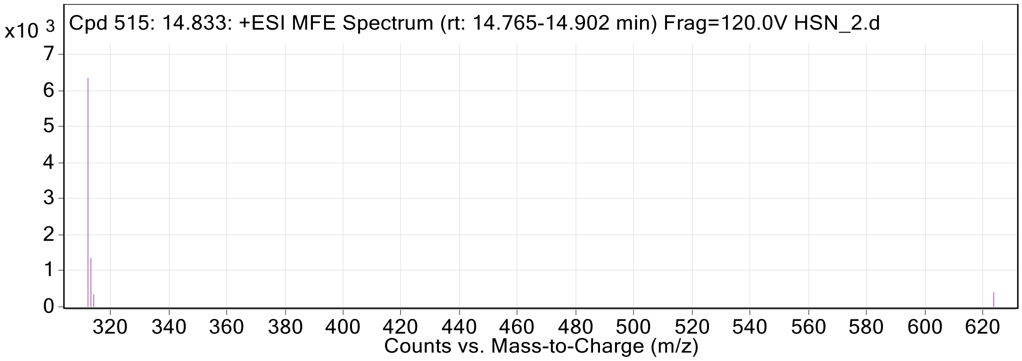

Peak List

m/z	z	Abund	Ion
312.3233	1	6352.67	(M+H)+
313.32766	1	1362.43	(M+H)+
314.32899	1	347.91	(M+H)+
623.63957	1	407	(2M+H)+

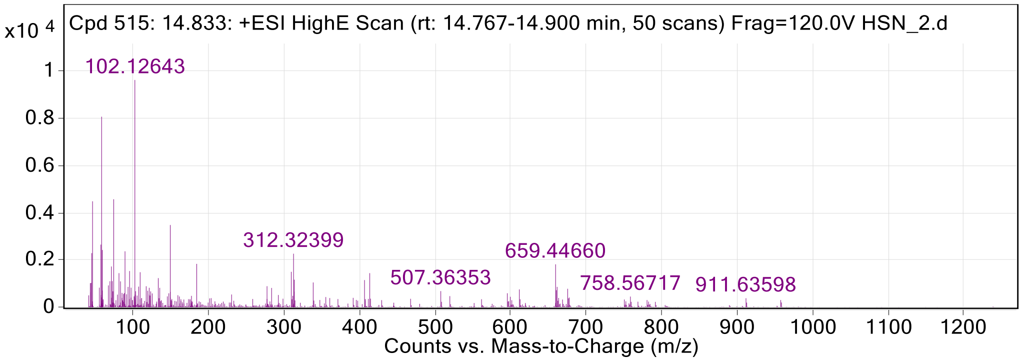

Peak List

m/z	z	Abund
45.03323		2283.5
46.06488		4480.27
57.06937		2645.06
58.06464		8067.96
59.04878		2417.36
74.09563		4567.02
89.05868		2361.07
102.12643	1	9622.91
149.02139		3476.16
312.32399	1	2260.01

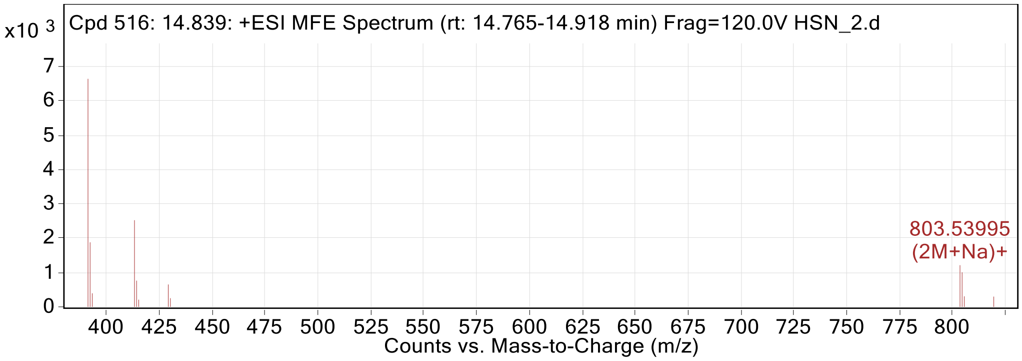

Peak List

m/z	z	Abund	Ion
391.28208	1	6640.29	(M+H)+

392.28423	1	1879.09	(M+H)+
393.28692	1	394.92	(M+H)+
413.26353	1	2523.27	(M+Na)+
414.26803	1	763.03	(M+Na)+
429.23809	1	651.59	(M+K)+
803.53995	1	1213.4	(2M+Na)+

m/z	z	Abund	Ion
804.54185	1	1006.65	(2M+Na)+
805.55337	1	307.47	(2M+Na)+
819.51499	1	297.75	(2M+K)+

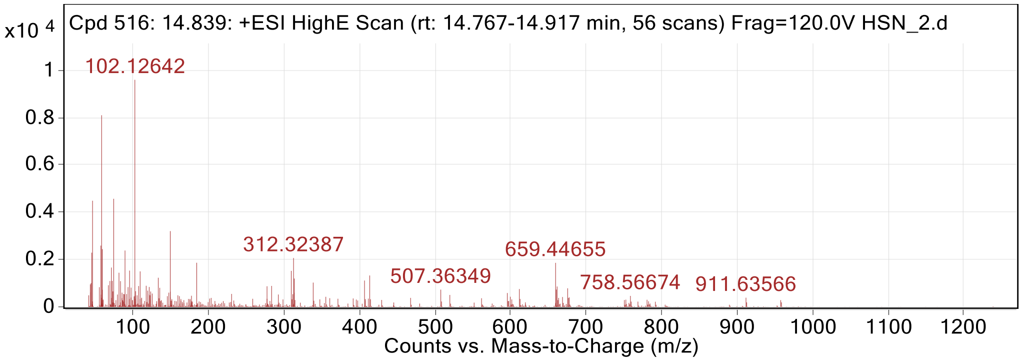

Peak List

m/z	z	Abund
45.03324		2290.9
46.06487		4487.6
57.06934		2589.33
58.06463		8101.35
59.04877		2439.85
74.09561		4570.08
89.05864		2382.67
102.12642	1	9598.62
149.02134		3203.09
312.32387	1	2065.5

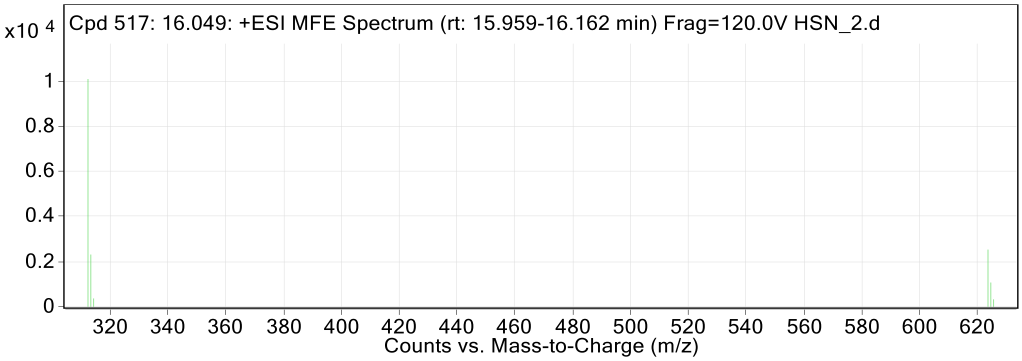

Peak List

m/z	z	Abund	Ion
312.32425	1	10092.8	(M+H)+
313.32796	1	2315.08	(M+H)+
314.33109	1	368.35	(M+H)+
623.64319	1	2534.42	(2M+H)+
624.64519	1	1074.8	(2M+H)+
625.64478	1	328.4	(2M+H)+

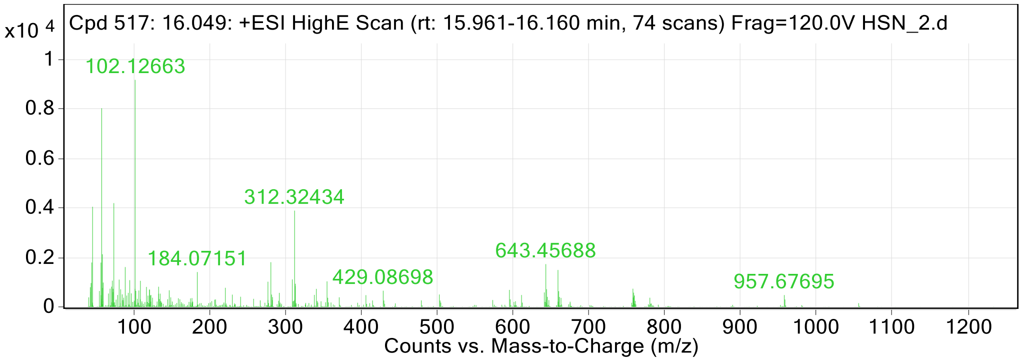

Peak List

m/z	z	Abund
45.0333		1793.33
46.06494		4046.94
57.06954		1790.94
58.06478		8029.85
59.04889		2126.14
74.09577		4194.19
102.12663	1	9176.6
281.04949	1	1805.87
312.32434	1	3891.26
643.45688	1	1728.51

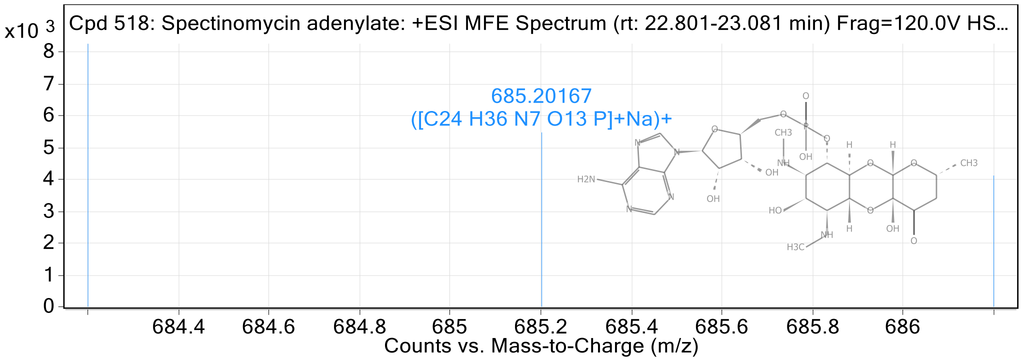

Peak List

m/z	z	Abund	Name	Formula	Ion	Score (DB)	Hits (DB)
684.20006	1	8257.43	Spectinomycin adenylate	C24 H36 N7 O13 P	(M+Na)+	60.56	1
685.20167	1	5472.82		C24 H36 N7 O13 P	(M+Na)+		
686.19936	1	4119.31		C24 H36 N7 O13 P	(M+Na)+		

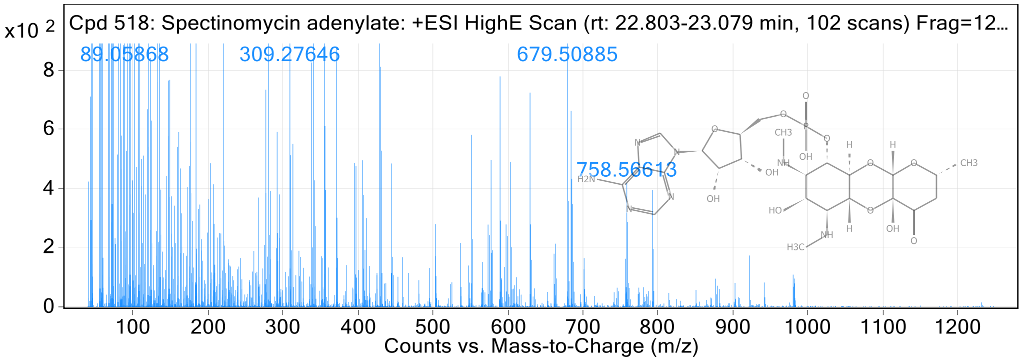

Peak List

m/z	z	Abund
45.03326		6305.14
46.06482		2239.8

57.06946		2044.21
58.06476		2162.29
89.05868		6324.79
102.12653		2171.04
109.09977		1941.26
133.08449		3499.04
184.07154		2024.99
309.27646	1	2398.89

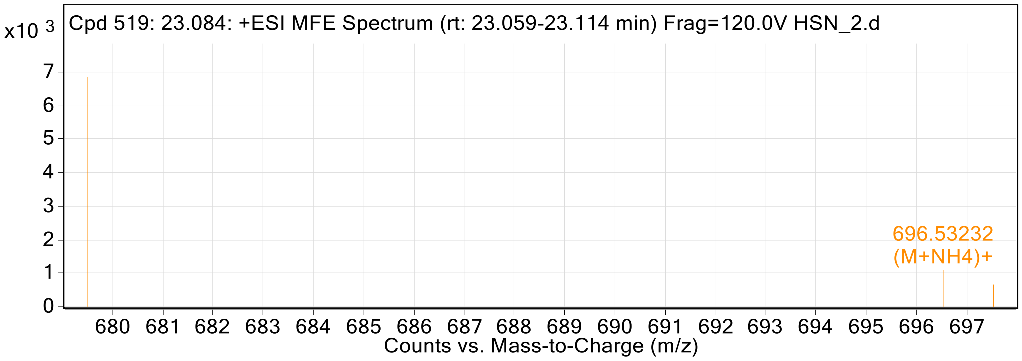

Peak List

m/z	z	Abund	Ion
679.50745	1	6858.45	(M+H)+
696.53232	1	1091.93	(M+NH4)+
697.53365	1	661.42	(M+NH4)+

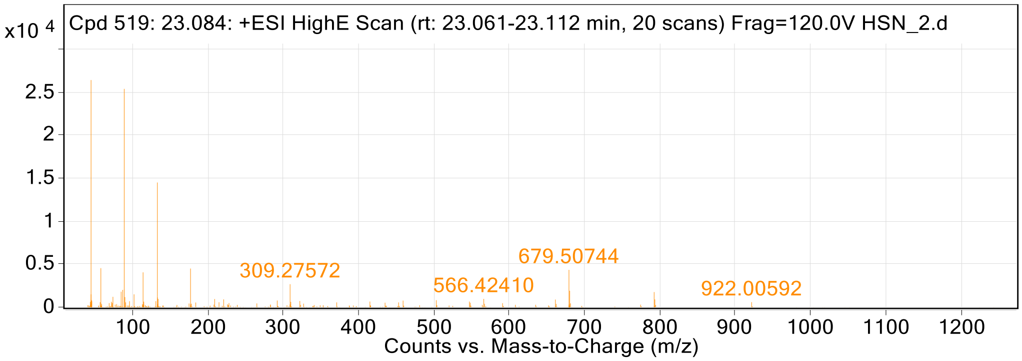

Peak List

m/z	z	Abund
45.03317	1	26423.13
58.06454		4529.96
87.04268		1998.83
89.05848	1	25392.35
89.07917		2063.24
114.08962	1	4043.75
133.08394	1	14498.19
177.10989	1	4484.47
309.27572	1	2664.64
679.50744	1	4332.15

--- End Of Report ---
